# Deoxyradiofluorination of Phenols via a Difluoromethoxy Nucleofuge Enabled by Organic Photoredox Catalysis

**DOI:** 10.1021/acscentsci.6c00127

**Published:** 2026-05-07

**Authors:** Maulik N. Mungalpara, Xuedan Wu, Xinrui Ma, Zhengbo Zhu, Meijuan Jiang, Victor W. Pike, Shuiyu Lu, He Zhang, Zhanhong Wu, Peyton O. Kinon, Yiyun Huang, David A. Nicewicz, Zibo Li

**Affiliations:** † Biomedical Research Imaging Center, Department of Radiology, and UNC Lineberger Comprehensive Cancer Center, University of North Carolina at Chapel Hill, Chapel Hill, North Carolina 27514, United States; ‡ Molecular Imaging Branch, National Institute of Mental Health, National Institutes of Health, Bethesda, Maryland 20892−1003, United States; § Department of Chemistry, University of North Carolina at Chapel Hill, Chapel Hill, North Carolina 27599, United States; ∥ Department of Radiology and Biomedical Imaging, Yale University, New Haven, Connecticut 06520, United States

## Abstract

The aryl difluoromethoxy (−OCF_2_H) group is identified as an efficient nucleofuge for organic photoredox-catalyzed deoxyradiofluorination of a broad range of phenols. This nucleofuge expands upon the Nicewicz group’s polarity reversed cation radical-accelerated nucleophilic aromatic substitution (CRA-S_N_Ar) strategy. Its discovery emerged from a systematic modification of the aryl methoxy (−OMe) group, previously established as being a potent nucleofuge for CRA-S_N_Ar amination and cyanation, but ineffective for C­(sp^2^)–O (radio)­fluorination. The present protocol enables the labeling of a wide range of (hetero)­arenes with fluorine-18 (*β* ^+^, 97%; *t*_1/2_ = 109.8 min) not only at the *ortho* and *para* positions to electron-donating groups but also at electronically disfavored *meta* positions. Key features of this protocol include step-economical precursor synthesis, efficient site-selective radiofluorination, and easy radiotracer isolation. The utility of this protocol is showcased through the late-stage radiofluorination of substrates containing a −OCF_2_H group including bioactive phenols and therapeutics. This protocol also has the capacity to access ^18^F-labeled synthons and potential positron emission tomography (PET) tracers.

## Graphical abstract

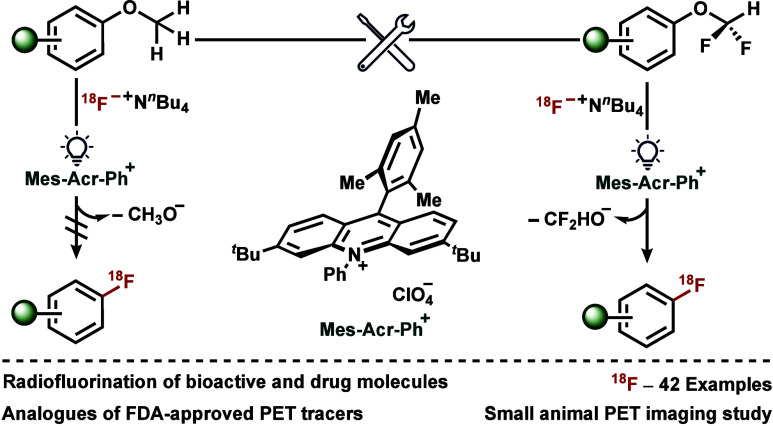

Fluorine-18 is the quintessential radionuclide for positron emission tomography (PET)—a noninvasive imaging modality commonly utilized for biomedical research, early stage disease diagnosis, and drug discovery.^1,2^ With its attractive physical properties, such as a practically useful physical half-life (*t*_1/2_ = 109.8 min), high efficiency for decay by positron emission (97%), and low positron energy (*β* ^+^, 635 keV), fluorine-18 has become widely used for the development of tracers for PET imaging.^3,4^ Fluorine-18 can be generated from cyclotrons as nucleophilic [^18^F]­fluoride ion or as electrophilic [^18^F]­fluorine (^18^F–F) but only the former can be easily produced in high molar activity.^5^ [^18^F]­Fluoride is obtainable in high amounts (multi curies) through ^18^O­(p,n)^18^F nuclear reaction by irradiating ^18^O-enriched water with a proton beam.^6^ The high hydration energy of [^18^F]­fluoride and consequent sluggish nucleophilicity when partially hydrated hinder its fast and efficient incorporation into (hetero)­arene scaffolds.^7^ Therefore, facile radiosynthetic strategies for forming the kinetically disfavored but strong aryl C­(sp^2^)–^18^F bond (bond dissociation energy of C­(sp^2^)–F ∼115 kcal mol^–1^) are highly sought after to deliver rapid and efficient syntheses of ^18^F-labeled PET tracers.^8–10^ As such, both electrophilic aromatic substitution (S_E_Ar) and nucleophilic aromatic substitution (S_N_Ar) methods have traditionally been employed to construct C­(sp^2^)–^18^F bonds using tailored precursors (Figure 1a,b).^11,12^ Nevertheless, the [^18^F]­S_E_Ar strategy has well-known limitations of low yields and low molar activity for synthesized radiotracers, because of carrier dilution with fluorine-19. [^18^F]­S_N_Ar addition–elimination has remained the flagship method for introducing fluorine-18 into electron-deficient (hetero)­arenes.^11,12^ This approach is restricted to substrates with electron-withdrawing groups *ortho* or *para* to a potential nucleofuge in order to stabilize Meisenheimer intermediates. Moreover, both conventional radiofluorination methods entail the prefunctionalization of substrates at the desired labeling site and harsh reaction conditions for the formation of high energy Wheland and Meisenheimer intermediates, which can limit the scope of bioactive molecules for late-stage radiofluorination to produce clinically useful PET tracers. Scope for (hetero)­aryl radiofluorination expanded with the introduction of hypervalent electrophilic precursors, such as diaryliodonium salts,^13–16^ aryliodonium ylides,^17^ and triarylsulfonium salts^18,19^ for transition-metal-free C­(sp^2^)–H radiofluorination (Figure 1c). These reactions are mechanistically distinct from classical S_N_Ar reactions in not proceeding through Meisenheimer complexes, but by ligand exchange and then reductive coupling.^13,18^

**Figure 1:**
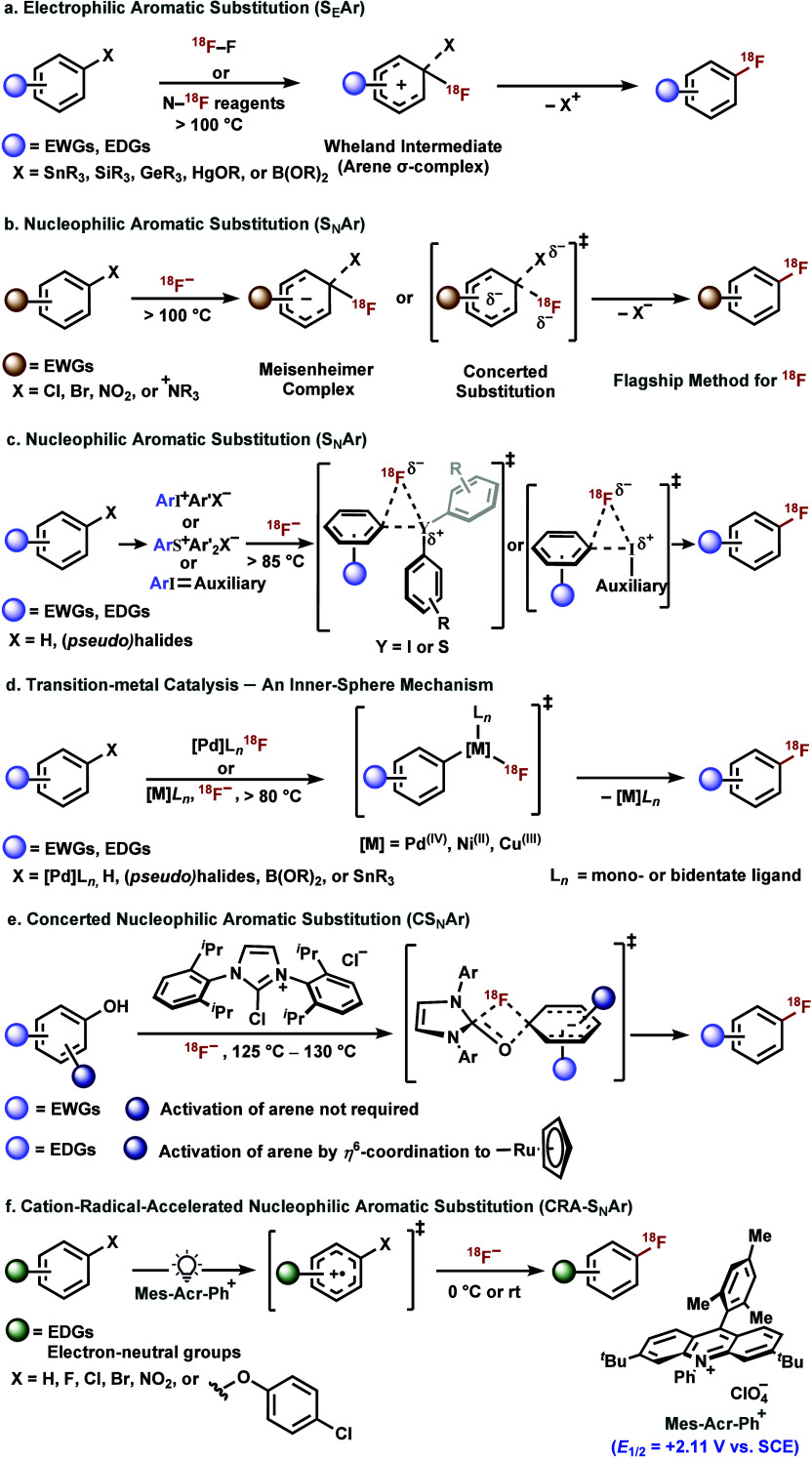
Mechanisms investigated for the aryl C­(sp^2^)–^18^F bond formation. a. Electrophilic aromatic substitution (S_E_Ar). b. Nucleophilic aromatic substitution (S_N_Ar). c. S_N_Ar (not through the Meisenheimer complexes). d. Transition-metal catalysis, an inner-sphere mechanism. e. Concerted S_N_Ar. f. Cation radical-accelerated S_N_Ar (CRA-S_N_Ar).

Furthermore, transition-metal-mediated strategies have been introduced to facilitate the radiofluorination of (hetero)­arenes at lower energy barriers (Figure 1d). Notable methods include rapid C­(sp^2^)–^18^F bond formation by reductive elimination from high-valent aryl–palladium­(IV)^20^ and aryl–nickel­(II) [^18^F]­fluoride complexes,^21^ and copper-mediated nucleophilic radiofluorination of (hetero)­aryl boronic acids and esters,^22–24^ aryl halides,^25^ or (hetero)­aryl stannanes.^26,27^ Although transition-metal-mediated radiofluorination methods have demonstrated enhanced reactivity and selectivity, the preparation of stoichiometric complex organometallic species, the use of bespoke oxidants, and reagent air-sensitivity can hinder their application in certain situations. Direct targeting of C­(sp^2^)–H bonds for nucleophilic radiofluorination has some feasibility as exemplified by Cu-mediated aminoquinoline directed^28^ *ortho*-C­(sp^2^)–H-, and Ir/Cu-mediated sequential *meta*-C­(sp^2^)–H radiofluorination of (hetero)­arenes.^29^ However, regioselectivity is still an unsolved problem for this approach. Selective displacement of commonly found arene functionalities by [^18^F]­fluoride may address the issue of site-selectivity. For example, a deoxyradiofluorination method involving a concerted S_N_Ar reaction wherein a uronium intermediate formed between a readily accessible phenol and chloroimidazolium chloride can directly elute [^18^F]­fluoride from an anion exchange cartridge to yield a radiofluorinated (hetero)­arene (Figure 1e).^30,31^ This method is mainly applicable to electron-deficient phenols whereas electron-rich phenols require η^6^-coordination to ruthenium for effective radiofluorination.^32,33^

We have unveiled a polarity reversed photoredox-catalyzed strategy (CRA-S_N_Ar) to radiofluorinate electron-rich and electron-neutral (hetero)­arenes.^34–36^ This strategy exploits the excited-state reactivity of the photoactivated acridinium compound, Mes-Acr-Ph^+^ to oxidize (hetero)­arenes to corresponding cation radicals for direct C­(sp^2^)–H radiofluorination,^34^ deoxy­(radio)­fluorination,^35^ and (*pseudo*)­halide interconversion radiofluorination (Figure 1f).^36^ An aryloxy nucleofuge, the 4-chlorophenoxy motif (4–Cl–C_6_H_4_O−), proved to be a promising group for phenol deoxy­(radio)­fluorination.^35^ Notably, the aryl methoxy (−OCH_3_) group failed as a nucleofuge. Whereas an aryl–OCH_3_ group is a potent nucleofuge for CRA-S_N_Ar amination^37^ and cyanation,^38^ deprotonation of acidic C–H bonds in the arene cation radical intermediates is likely responsible for the unsuccessful C­(sp^2^)–O fluorination.^39^ Additionally, the strong basicity of unquenched methoxide (p*K*_a_ = 15.2 for methanol) may also contribute to its poor nucleofugality because functional groups with weak basicity generally constitute the better leaving groups in traditional S_N_Ar methods (Figure 2a).^40^ In CRA-S_N_Ar amination^37^ and cyanation,^38^ the methoxide nucleofuge could potentially be neutralized by proton and trimethylsilyl species of nucleophiles, respectively.^41^ Given the above considerations, the current study attempted to modify the aryl–OCH_3_, so that the resulting functionality would be weakly basic while improving its nucleofugality under photoredox conditions.

**Figure 2:**
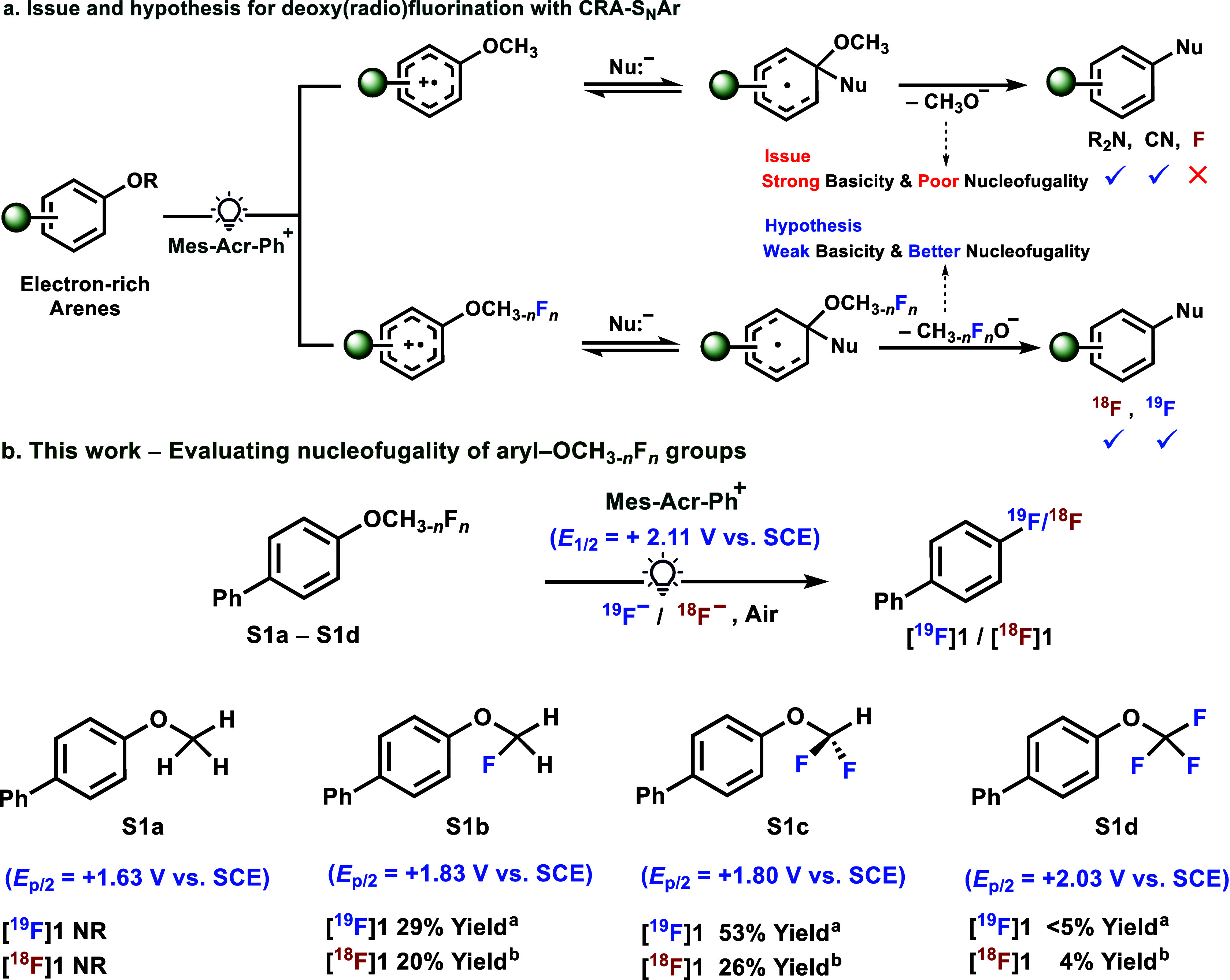
a. Issue and hypothesis for phenol deoxy­(radio)­fluorination with CRA-S_N_Ar. b. This Work: Evaluating the nucleofugality of aryl–OCH_3–*n*_F_*n*_ groups for photoredox-catalyzed deoxyradiofluorination of phenols, **S1a**–**S1d**. **S** means methyl/fluoromethyl aryl ether substrate. ^a^Isolated yield. ^b^All yields are decay-corrected and were calculated by HPLC isolation of labeled product from azeotropically dried [^18^F]­TBAF, and they are from one experiment. Reaction conditions: for ^19^F-chemistry, substrate (0.1–0.3 mmol, 1.0 equiv), Mes-Acr-Ph^+^BF_4_^–^ (0.05 equiv), CsF (5.0 equiv), TBAHSO_4_ (0.75 equiv), CH_2_Cl_2_:H_2_O (0.1 M, 25:1 v/v), 450–455 nm LED, 48 h, 33 °C, air; for ^18^F-chemistry, substrate (0.05 mmol), Mes-Acr-Ph^+^ClO_4_^–^ (1.5 mg), [^18^F]­TBAF (5–20 mCi), TBAHCO_3_ (25 μL), 450 nm laser (2.35 W), 30 min, rt, air. NR means no reaction.

Herein, we disclose the realization of our hypothesis for the photoredox-catalyzed deoxyradiofluorination of phenols with a bespoke alkoxy nucleofuge that enables radiofluorination of nonactivated electron-rich (hetero)­arenes. This modified aryl–OCH_3–*n*_F_*n*_ group can be embedded in phenols from its carbene precursor in a one-step reaction (see Supporting Information), which permits arene radiofluorination under mild photoredox conditions and which eases radiotracer isolation. Deoxy­(radio)­fluorination proceeds in a site-selective manner, as other alkoxy substituents in the same molecule remain intact. The main application of our protocol is showcased by the late-stage radiofluorination of analogues of PET tracers approved by the US Food and Drug Administration (FDA).

## Results and Discussion

### Evaluation of Nucleofugality for −OCH_3–*n*_F_*n*_ Groups in Phenol Deoxy­(radio)­fluorination

To identify an alkoxy nucleofuge with weak basicity for CRA-S_N_Ar phenol deoxy­(radio)­fluorination, we explored −OCH_3–*n*_F_*n*_ as a potential nucleofuge. *Ab initio* molecular orbital calculations at the G2 level have shown a progressive increase in acidity upon successive fluorine substitutions in methanol.^42^ We also hypothesized that an anomeric n_O_–σ*_C–F_ stabilization effect may enhance the electrophilicity of C­(sp^2^) bearing −OCH_3–*n*_F_*n*_ groups to facilitate attack of fluoride ion.^43^ At the outset, we decided to introduce fluorine atoms successively into the aryl–OCH_3_ group. We envisioned monofluoromethoxy (−OCFH_2_), difluoromethoxy (−OCF_2_H), and trifluoromethoxy (−OCF_3_) groups as potential nucleofuges and added them to a 4,4′-biphenyl framework (Figure 2b). Subsequently, we evaluated their nucleofugalities under previously reported photoredox-deoxy­(radio)­fluorination conditions with 450–455 nm blue light irradiation.^35^ The most significant distinction arises from the stoichiometry of the fluoride reagent. In ^19^F-chemistry, fluoride is employed in excess, whereas in ^18^F-chemistry, [^18^F]­fluoride is the limiting reagent (pmol to nmol). Both protocols employ the acridinium photocatalyst, Mes-Acr-Ph^+^ (*E*_1/2_ = +2.11 V vs. saturated calomel electrode, SCE), but with different counterions, BF_4_^–^ for ^19^F-chemistry and ClO_4_^–^ for ^18^F-chemistry—to avoid the isotopic dilution of [^18^F]­fluoride. In ^19^F-chemistry, the inclusion of a phase transfer reagent, tetra-*n*-butylammonium bisulfate (TBAHSO_4_), in the biphasic solvent system CH_2_Cl_2_:H_2_O (25:1 v/v) is found to significantly enhance product yields. This effect is attributed to improving the solubility of cesium fluoride (CsF) and the in situ formation of tetra-*n*-butylammonium fluoride (TBAF) in the presence of trace water. Notably, TBAF is unstable at room temperature, undergoes Hofmann elimination and decomposes to tributylamine and bifluoride.^44^ In contrast, ^18^F-chemistry utilizes tetra-*n*-butylammonium bicarbonate (TBAHCO_3_) as a mild base to prevent Hofmann elimination in the DCE:CH_3_CN:^*t*^BuOH monophasic-solvent system.

Under these conditions, −OCFH_2_ and −OCF_3_ groups displayed modest to poor nucleofugality for aryl deoxy­(radio)­fluorination, whereas the −OCF_2_H-containing substrate **S1c** (*E*_p/2_ = +1.80 V vs. SCE) furnished the desired product [^19^F]**1** in 53% yield and [^18^F]**1** in 26% yield. Despite the robust stability and high electronegativity of the −OCF_3_ group (*χ* = +3.7 for −OCF_3_ vs. *χ* = +2.7 for −OCH_3_ based on Pauling’s electronegativity scale),^45,46^ inefficient deoxy­(radio)­fluorination of **S1d** (*E*_p/2_ = +2.03 V vs. SCE) is likely due to a thermodynamic mismatch with Mes-Acr-Ph^+^.^47^ Even photocatalysts with higher oxidation potential failed to deliver reasonable deoxy­(radio)­fluorination when tested with commercially available 1-methoxy-4-(trifluoromethoxy)­benzene (*E*_p/2_ = +1.91 V vs. SCE) (Table S2 in the Supporting Information). This suggests that the combined effects of the high oxidation potential and strong electron-withdrawing nature of the −OCF_3_ substituent significantly hinder arene cation radical formation and subsequent C­(sp^2^)–O bond activation. We surmised that the unique characteristics of the −OCF_2_H group should render the −OCF_2_H group to adjust easily to the reverse polarity change of the arene cation radical, enabling efficient polarity matching with Mes-Acr-Ph^+^ under photoredox conditions. These characteristics include axially nonisotropic shape (dihedral angle (Φ) = 0–50°), electrostatic potential (by contrast to its isotropic CH_3_ and CF_3_ counterparts), and a tendency to interconvert between two characteristic conformations (i.e., *endo-endo* conformation (highly lipophilic) and *endo-exo* conformation (highly polar)).^48,49^ As a result, developing a negative charge on the departing OCF_2_H moiety is better stabilized during C­(sp^2^)–O bond cleavage, leading to more efficient nucleofugation. After a detailed screening of photocatalysts with higher oxidation potential and modulating reaction parameters, the −OCF_2_H installed model substrate **S1c** furnished the desired product [^19^F]**1** in an impressive 76% isolated yield (Table S3 in the Supporting Information).

### Substrate Scope for CRA-S_N_Ar with ^18^F

Despite the modest nucleofugality of the −OCF_2_H group in CRA-S_N_Ar arene deoxyfluorination (Figure S2 in the Supporting Information), we were intrigued to explore its reactivity for the analogous radiofluorination method. If successful, this protocol for the site-selective C­(sp^2^)–^18^F bond formation would be appealing to access ^18^F-labeled bioactive and drug molecules as potential PET agents at late-synthesis stage under mild photoredox conditions, as indicated from our prior study.^50^ Radiochemical yield is based on the decay-corrected activity of the labeled compound and even 5% yields may be sufficient to obtain high-contrast PET imaging data in animals and humans^51^ because [^18^F]­fluoride can be produced in high activities.

We delineated the scope of phenol deoxyradiofluorination with the −OCF_2_H nucleofuge (Figure 3). Maintenance of an adequate loading of photocatalyst rather than substrate concentration was essential for efficient deoxyradiofluorination. Also, switching air to oxygen sparging improved yields in most cases, likely because oxygen aids Mes-Acr-Ph^+^ photocatalyst turnover and accelerates the extrusion of −OCF_2_H nucleofuge from cyclohexadienyl radical to furnish the desired radiofluorination (Figure S3 in the Supporting Information).^52–54^ Simple ^18^F-labeled arenes, such as [^18^F]­4-fluoro-1,1′-biphenyl ([^18^F]**1**), [^18^F]­2-fluoro-1,1′-biphenyl ([^18^F]**2**), [^18^F]­4-fluoro cyclohexylbenzene ([^18^F]**4**), and [^18^F]­4-(4-(fluoro)­phenyl)­butan-2-one ([^18^F]**6**), were readily obtained in excellent yields. While naphthalene substrate **S3** resulted in the desired deoxyradiofluorination product [^18^F]**3** along with arene C–H radiofluorination at various positions under oxygen. A switch to nitrogen sparging gave solely the desired product [^18^F]**3** in good yield. However, its partially hydrogenated analogue, tetrahydronaphthalene substrate **S5** (*E*_p/2_ = +2.01 V vs. SCE) could only be radiofluorinated in 5% yield, presumably due to its insufficient aryl ring electron density.

**Figure 3:**
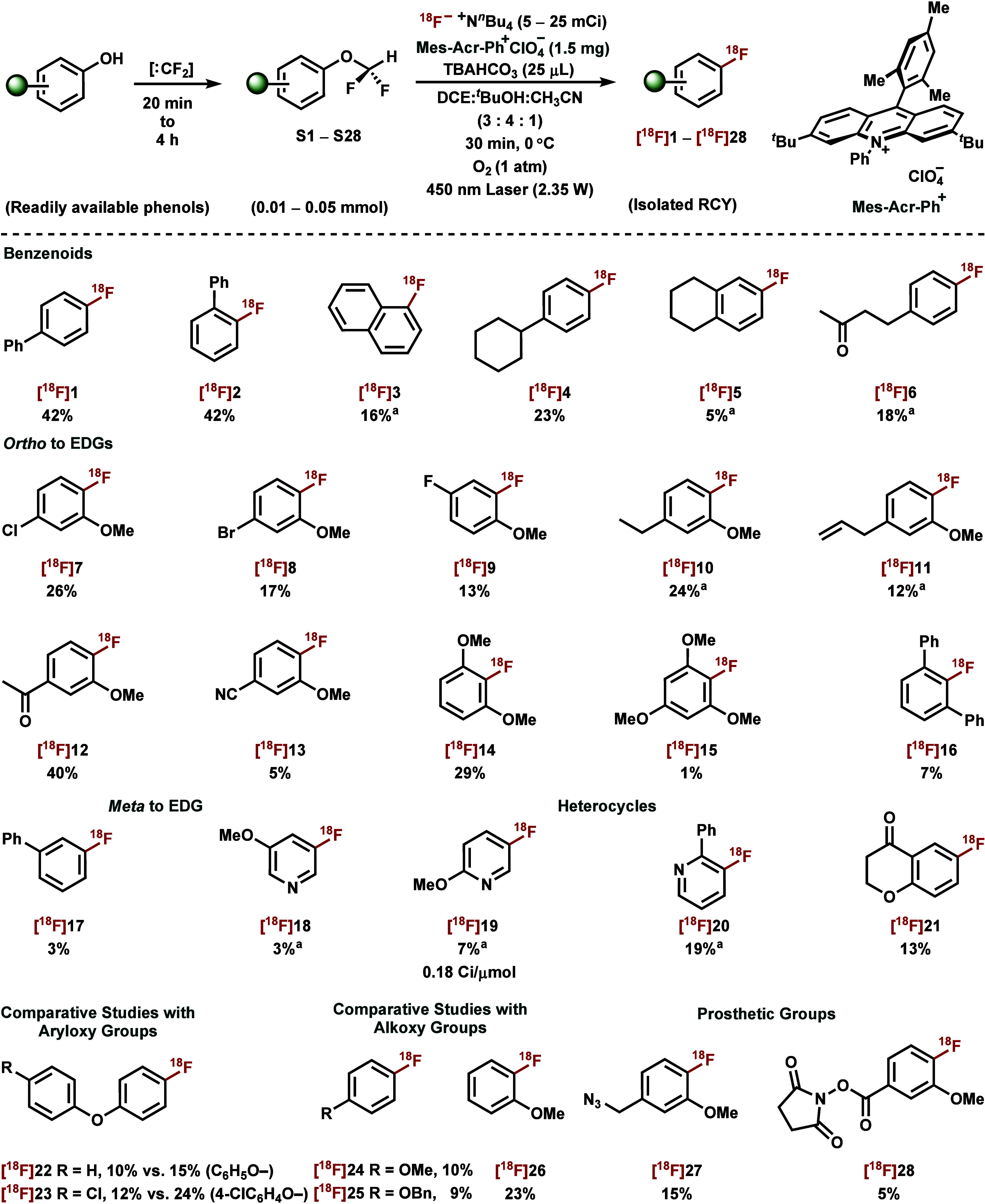
Exploration of substrate scope for CRA-S_N_Ar arene deoxyradiofluorination. **S1**–**S28**, **S** means difluormethoxy­(hetero)­arene substrate. All yields are decay-corrected and were calculated by HPLC isolation of labeled product from azeotropically dried [^18^F]­TBAF; and they are from an average of two experiments (*n* = 2). ^a^N_2_ atmosphere was used instead of O_2_.

Next, we set out to determine the functional group compatibility. Substrates with *ortho* electron-donating groups fared better. Halogenated anisoles **S7**–**S9** were well-tolerated under our protocol to produce the corresponding ^18^F-labeled products [^18^F]**7**–[^18^F]**9** in moderate yields (13–26%), along with minor byproducts largely due to ^18^F/halide interconversions. Ethylguaiacol and eugenol derivatives yielded solely deoxyradiofluorination products [^18^F]**10** and [^18^F]**11**, respectively, in moderate yields regardless of competitive benzylic oxidation. Moreover, substrates containing both electron-withdrawing (such as keto and nitrile groups) and -donating groups can also be accommodated well under our reaction conditions, as shown by giving the corresponding radiofluorinated products [^18^F]**12** and [^18^F]**13** in good yields.

Increasing the electron density of the arene proved to be beneficial for substrate **S14**, but not for **S15** as the yield was comparable to substrate **S26** bearing an *ortho* methoxy group. A pronounced difference in reactivity is observed between [^18^F]**14** and [^18^F]**15** (29% vs. 1%). This discrepancy likely arises from the mismatch between low *E*_p/2_ value for the super electron-rich substrate (*E*_p/2_ < +1.5 V vs. SCE) and the higher oxidation potential of Mes-Acr-Ph^+^ (*E*_1/2_ = +2.11 V vs. SCE).^55^ As a consequence, cation radical of super electron-rich arene tends to rapidly accept an electron back from the excited state of photocatalyst (Mes-Acr-Ph^+^*), leading to fast back-electron transfer and quenching of the Mes-Acr-Ph^+^* via charge transfer excited state.^56,57^ On the contrary, deoxyfluorination was less efficient with the substrate **S16** (1,3-diphenyl groups) vs. substrate **S2** (monophenyl group), presumably due to steric hindrance from the larger phenyl rings.

Notably, this method was also compatible with substrates (**S17**, **S18**, (±)-**S40**, and (±)-**S41** *vide infra*) containing electron-rich group at *meta* position. In each case, deoxyradiofluorination proceeded, albeit in a low yield. Normally difficult-to-oxidize heterocyclic substrates,^58^ such as pyridines **S18**–**S20** and chromane **S21**, can also be deoxyradiofluorinated in moderate yields. The low molar activity (*A*_m_) observed for [^18^F]**19** and [^18^F]**39** (*vide infra*) is probably due to the tendency of fluorinated ethers to show a *generalized anomeric effect*,^59,60^ which means C–F bond can transition between covalent and electrostatic character. This effect may promote isotopic dilution through exchange with [^19^F]­fluoride ions during the radiosynthesis. Additionally, the relatively small quantity of [^18^F]­fluoride (5–25 mCi) used further contributes to the reduction in *A*_m_. While the effect of isotopic dilution is not controllable, we expect acceptable molar activities (0.5–1.0 Ci/μmol) of ^18^F-radiotracers using our protocol can be achieved by performing radiosynthesis with higher amounts of starting radioactivity (^18^F_c_ > 1.0 Ci).^61^

Furthermore, we evaluated the propensity of aryloxy groups to function as nucleofuges in the same molecules bearing −OCF_2_H substituent. In these comparative studies, the nucleofugality of C_6_H_5_O– was observed to be 1.5-fold higher in substrate **S22** and for 4–Cl–C_6_H_4_O– twice as high in substrate **S23** relative to the −OCF_2_H group for CRA-S_N_Ar deoxyfluorination. Although aryloxy groups, C_6_H_5_O– and 4–Cl–C_6_H_4_O–, exhibit superior nucleofugality, the −OCF_2_H group offers practical advantages including faster and more convenient installation into phenolic substrates and no issue of site-selectivity. We were also intrigued to investigate the nucleofugality of −OCF_2_H in the presence of other alkoxy groups in the same molecule. Results from the products [^18^F]**24**–[^18^F]**26** indicate that −OMe and −OBn groups were largely unaffected under our photoredox conditions, allowing deoxyradiofluorination to proceed in a site-selective manner. Nevertheless, the corresponding crude radio-HPLC tracers do display minor additional signals, which are most plausibly attributed to side reactions such as C­(sp^2^)–H- and benzylic radiofluorination.

^18^F-Labeled arenes with highly reactive functionality can serve as prosthetic groups that can be used to label structurally sensitive bioactive ligands for the construction of novel PET agents.^62^ Under our conditions, the radiofluorination of precursor **S27** proceeded smoothly to furnish ^18^F-labeled benzyl azide, ([^18^F]**27**), a useful synthon for ^18^F-labeling by azide–alkyne cycloaddition.^63^ Similarly, without detailed optimization, methoxy [^18^F]*N*-succinimidyl fluorobenzoate, ([^18^F]**28**) was obtained in 5% yield, which can be employed to access ^18^F-radiolabeled peptides and proteins through amide coupling.^64^

### Radiofluorination of Bioactive and Drug Molecules

After gaining these encouraging results, we explored the site-selective late-stage radiofluorination of drug candidates and bioactive molecules to synthesize potential ^18^F-labeled PET tracers (Figure 4a).

**Figure 4:**
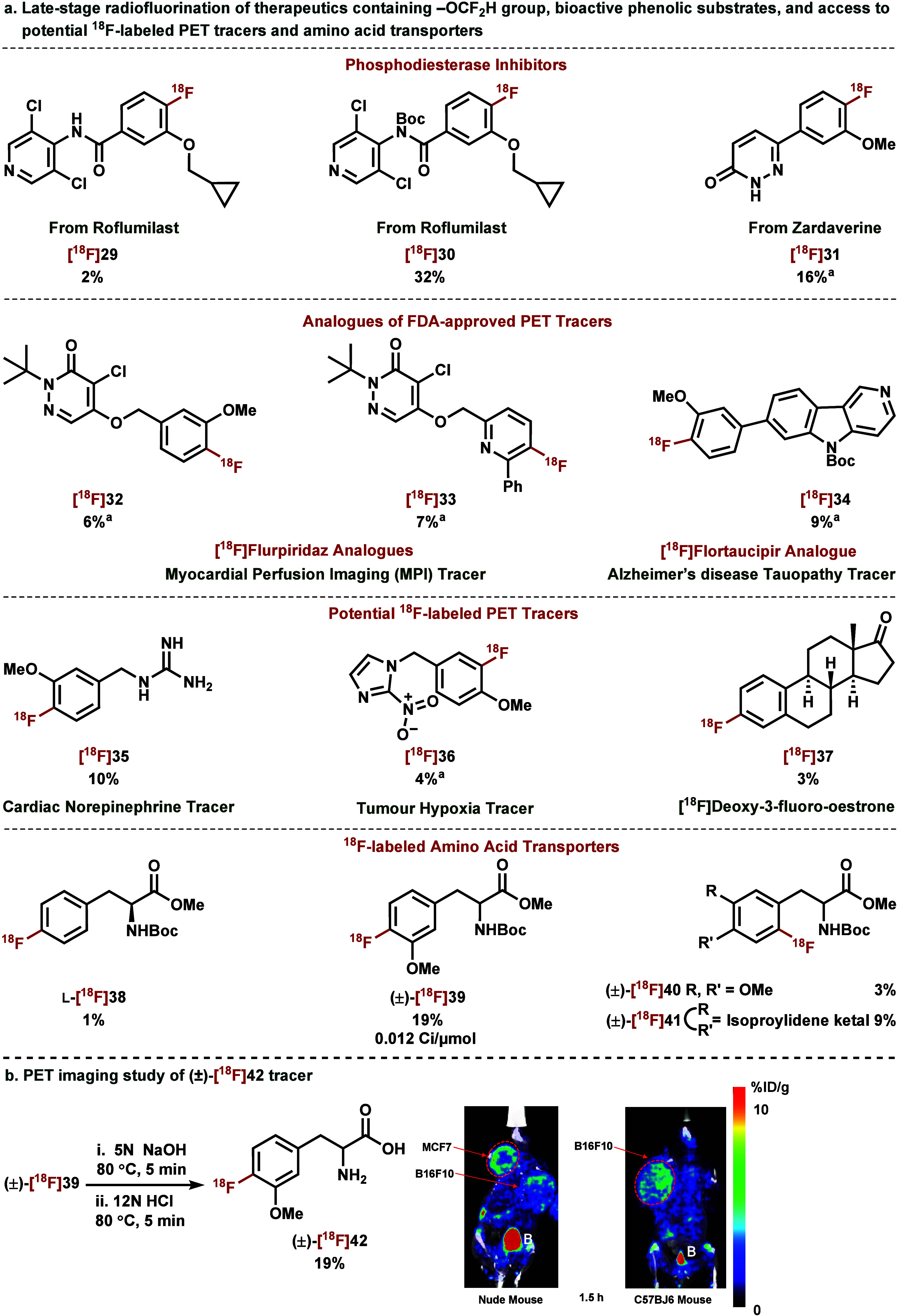
a. Late-stage CRA-S_N_Ar deoxyradiofluorination of therapeutics containing −OCF_2_H group, bioactive phenolic substrates, and access to potential PET tracers. b. PET imaging study of (±)-[^18^F]**42**. All yields are decay-corrected and were calculated by HPLC isolation of labeled product from azeotropically dried [^18^F]­TBAF, and they are from an average of two experiments (*n* = 2). ^a^N_2_ atmosphere was used instead of O_2_.

Our protocol offers an efficient approach to derivatize commercially available drugs containing a −OCF_2_H moiety to PET agents by converting the −OCF_2_H group into ^18^F. For example, Roflumilast **S29** is an approved phosphodiesterase-4 (PDE4) inhibitor and an anti-inflammatory drug that has been used for treating severe chronic obstructive pulmonary disease. Roflumilast **S29** has not yet been labeled for PET imaging studies but has often been applied in conjunction with the first reported PDE4 PET radioligand, (*R*)-[^11^C]­rolipram, to study brain PDE4 occupancy for a better understanding of cognitive function.^65^ Using our method, radiofluorination of **S29** was initially low yielding (2%) due to the presence of the oxidizable benzamide group—a known limitation of our strategy.^66^ However, we found that the carbamate–protected version **S30** rendered the [^18^F]­Roflumilast analogue, [^18^F]**30**, with complete site-selectivity in a good yield (32%). Similarly, a pyridazinone, Zardaverine **S31**—a dual PDE4 ^67^ and PDE3A inhibitor—was also functionalized with [^18^F]­fluoride in useful yield (16%). Although the binding affinity of these derivatives requires further validation due to the introduced structural modifications, our method does provide a simple approach to synthesize potential PET agents from these readily available drugs.

Another pyridazinone, [^18^F]­Flurpiridaz, is a recently FDA-approved myocardial perfusion imaging (MPI) radiotracer.^68^ Whereas [^18^F]­Flurpiridaz has shown excellent diagnostic performance for MPI, there remain limitations to be addressed for maximizing its clinical utility.^69^ Under our radiolabeling conditions, we were able to introduce a C­(sp^2^)–^18^F bond to furnish analogues [^18^F]**32** and [^18^F]**33** in useful yields. In a similar vein, the development of novel Alzheimer’s disease (AD) and non-AD tauopathy PET tracers is highly desirable to minimize the off-target binding of reported PET tracers, including Tauvid.^70^ With our method, we produced a new potential tau-PET tracer [^18^F]**34** based on a pyrido-indole scaffold. These results underscore the operational simplicity of our deoxyradiofluorination protocol, offering a generalized approach for the site-selective introduction of [^18^F]­fluoride into pharmaceuticals and its ability to access analogues of FDA-approved radiotracers.

There has been a growing interest for the development of ^18^F-labeled benzylguanidine PET tracers.^71^ By implementing our protocol, the radiolabeling of benzylguanidine derivative **S35** was streamlined to a 100 min one-pot synthesis, whereas previous methods for each radiosynthesis of the benzylguanidines, [^18^F]­MFBG and [^18^F]­PFBG, required three steps.^72^ The metabolic stability of aliphatic ^18^F-labeled probes for PET imaging of hypoxia remain a major concern because the corresponding radiometabolites compete with the original tracer for hypoxia specific uptake, and hence results in poor imaging contrast.^73^ Under our standard conditions, the nitroimidazole derivative [^18^F]**36** can be obtained as a potential hypoxia PET agent containing the C­(sp^2^)–F bond. It would be interesting to study its stability in the future. To further demonstrate the broad applicability of our protocol, we successfully synthesized [^18^F]­deoxy-3-fluoro-oestrone ([^18^F]**37**), [^18^F]­(*S*)-*N*-Boc-*O*-methyl-4-fluoro-tyrosine ((*S*)-[^18^F]**38**), its derivative ((±)-[^18^F]**39**), dimethoxy-((±)-[^18^F]**40**), and isopropylidene ketal-protected [^18^F]*N*-Boc-2-fluoro-phenylalanine, ((±)-[^18^F]**41**). Based on prior studies, no racemization is expected under Mes-Acr-Ph^+^ photoredox conditions when acidic deprotection is used for enantiopure amino acid substrates.^74,75^ Overall, when applying this methodology to sterically complex molecules, we typically obtain tracers in modest yields with byproducts present in low abundance. These byproducts likely arise from a combination of factors including the presence of potential radical sites, competing C–H functionalization pathways, oxidation at the susceptible positions such as benzylic sites, or enhanced isotopic exchange. Despite these challenges, the rapid radiofluorination of these compounds as precursors to bioactive tracers and their easy chromatographic purification make this methodology especially attractive.

### Imaging Study

For the preclinical *in vivo* evaluation of (±)-[^18^F]**42** in a small animal, about 80 μCi of the tracer was intravenously injected into B16F10 and MCF7 tumor-bearing mouse and the animal was scanned at 1.5 h post injection (p.i.) for 15 min in a PET/CT scanner. MCF7 tumor uptake was 4.9% ID/g while the B16F10 tumor was 2.9% ID/g (Figure 4b). Liver, kidney, and muscle uptake were 1.8%, 4.6%, and 1.1% ID/g, respectively. Our tracer demonstrated high uptake in MCF7 and B16F10 tumors. A comparison of this uptake with that of [^18^F]­FET ([^18^F]*O*-2-fluoroethyl-l-tyrosine) is therefore of future interest.^76^

## Conclusions

This work set out to identify an alternate nucleofuge based on the alkoxy functionality for the organic photoredox-catalyzed deoxy­(radio)­fluorination of phenols. The problematic aryl–OCH_3_ functionality was strategically modified to the −OCF_2_H group to effect C­(sp^2^)–O (radio)­fluorination via a CRA-S_N_Ar mechanism. This method readily permits radiofluorination of a range of electron-rich (hetero)­arenes with complete site-selectivity under operationally mild conditions. Late-stage radiofluorination of bioactive molecules and drug candidates underscore the radiolabeling potential of this protocol, particularly for challenging substrates having an electron-rich group at *meta* position. Simple modulation of FDA-approved PET tracers by our method can open new avenues to access ^18^F-labeled PET tracers with potential applications in cardiac, neurology, and oncology disease studies.

## Experimental Section

All experimental procedures are listed in the Supporting Information.

## Supplementary Material



## Data Availability

The data underlying this study are available in the published article and its Supporting Information.

## Notes

General methods, materials and procedures, synthesis data, compound characterization, NMR data, HPLC data, and examples of radiochromatograms (PDF)
