# Supplementary material for: Deoxyradiofluorination of Phenols via a Difluoromethoxy Nucleofuge Enabled by Organic Photoredox Catalysis
Source: ACS Cent Sci. 2026 May 7;12(5):638–48. doi: 10.1021/acscentsci.6c00127 (PMC13220213; doi:10.1021/acscentsci.6c00127)

Deoxyradiofluorination of Phenols via a Difluoromethoxy Nucleofuge Enabled by Organic Photoredox Catalysis

Maulik N. Mungalpara[1], Xuedan Wu[1], Xinrui Ma[1], Zhengbo Zhu[1], Meijuan Jiang[2], Victor W. Pike[2], Shuiyu Lu[2], He Zhang[1], Zhanhong Wu[1], Peyton O. Kinon[3], Yiyun Huang[4], David A. Nicewicz[3*], Zibo Li[1*]

Affiliation of authors

[1] *Biomedical Research Imaging Center, Department of Radiology, and UNC Lineberger Comprehensive Cancer Center, University of North Carolina at Chapel Hill, Chapel Hill, NC 27514, United States.*

[2] *Molecular Imaging Branch, National Institute of Mental Health, National Institutes of Health, Bethesda, MD 20892–1003, United States.*

[3] *Department of Chemistry, University of North Carolina at Chapel Hill, Chapel Hill, NC 27599, United States.*

[4] *Department of Radiology and Biomedical Imaging, Yale University, New Haven, CT 06520, United States.*

[*] Zibo Li zibo_li@med.unc.edu (Z.L.)

[*] David A. Nicewicz nicewicz@unc.edu (D.A.N.)

Table of Contents

1. Supplemental Methods 3

1.1 Materials and Methods 3

1.1.1 General Reagents 3

1.1.2 Analytical Instruments 4

1.1.3 General Information for Radiochemistry 4

1.2 Electrochemical Measurements 5

1.3 Optimisation Studies and Miscellaneous Information for CRA-S_NAr Arene Deoxy(radio)fluorination 9

1.3.1 Optimisation studies for CRA-S_NAr arene deoxy(radio)fluorination via a trifluoromethoxy nucleofuge 9

1.3.2 Optimisation studies for CRA-S_NAr arene deoxyfluorination via a difluoromethoxy nucleofuge 12

1.3.3 Substrate scope for CRA-S_NAr arene deoxyfluorination via a difluoromethoxy nucleofuge 14

1.3.4 Postulated mechanism for CRA-S_NAr arene deoxyfluorination via a difluoromethoxy nucleofuge 16

1.4 Experimental Procedures 17

1.4.1 General conditions for difluoromethylation of phenols (Synthesis of precursors) 17

1.4.2 General conditions for CRA-S_NAr arene deoxyfluorination via a difluoromethoxy nucleofuge – ^{19}F chemistry 18

1.4.3 Procedure for anhydrous [^{18}F]TBAF solution in CH_3CN 20

1.4.4 General conditions for CRA-S_NAr arene radiodeoxyfluorination via a difluoromethoxy Nucleofuge – ^{18}F chemistry 20

1.4.5 General HPLC conditions for characterisation of ^{18}F-tracers 21

1.4.6 Radiochemical Yield (%Yield) calculation (Isolated %Yield) 22

1.4.7 Experimental Data 23

1.4.8 Molar activity calculation for [^{18}F]-5-(fluoro)-2-methoxypyridine (**[^{18}F]19**) 247

1.4.9 Molar activity calculation for [^{18}F]-methyl 2-((*tert*-butoxycarbonyl)amino)-3-(4-fluoro-3-methoxyphenyl)propanoate (**[^{18}F]39**) 249
1.4.10 Small animal PET imaging study 251
2. Supplementary References 252
3. NMR spectra of new compounds (^{1}H NMR, ^{13}C NMR, and ^{19}F NMR) 254

1. Supplemental Methods

1.1 Materials and Methods

1.1.1 General Reagents

Reagents were purchased from Ambeed, AA Blocks, Sigma-Aldrich, Acros, Alfa Aesar, TCI, Matrix Scientific, Combi-Blocks, Oakwood Chemical, Chem Impex International, Chemscene, and Fisher Scientific and were used as received unless otherwise noted. Solvents for reactions, filtration, transfers, and chromatography were certified ACS grade. Evaporation of solvents was carried out under reduced pressure on the rotary evaporator below 42 °C. Acetonitrile (CH_3CN), dichloromethane (CH_2Cl_2), tetrahydrofuran (THF), dimethyl sulfoxide (DMSO), and dimethylformamide (DMF) were dried by passing through activated alumina under nitrogen prior to use. Anhydrous 1,2-dichloroethane (1,2-DCE) and anhydrous ^{t}BuOH were purchased from Sigma-Aldrich and Fisher Scientific and were used as received.

Purification of the reaction mixture was performed by Biotage® Isolera flash chromatography, column chromatography, or preparative TLC. The stationary phase for chromatography was silica gel RediSep® columns. Solvents for purification were purchased from Thermo Fisher Scientific Inc. TLC visualisation was carried out using ultraviolet light (254 nm) and different staining reagents such as potassium permanganate, 2,4-dinitropyridine, ethanolic phosphomolybdic acid, or ninhydrin followed by heating when applicable.

1.1.2 Analytical Instruments

NMR spectroscopy was performed on Bruker 500 MHz or Bruker 400 MHz (^{1}H NMR at 500 MHz or 400 MHz, ^{13}C NMR at 126 MHz or 101 MHz, ^{19}F NMR at 470 MHz or 376 MHz) spectrometer with solvent resonance as the internal standard (^{1}H NMR: $CDCl_3$ at 7.26 ppm; ^{13}C NMR: $CDCl_3$ at 77.16 ppm and ^{1}H NMR: MeOD-d^4 at 3.31 ppm; ^{13}C NMR: MeOD-d^4 at 49.00 ppm). Chemical shifts are reported in parts per million (ppm). All spectra were run in $CDCl_3$ unless otherwise stated. Spin multiplicities are described as s (singlet), bs (broad singlet), d (doublet), dd (doublet of doublets), ddd (doublet of doublet of doublets), dddd (doublet of doublet of doublet of doublets), t (triplet), td (triplet of doublets), q (quartet), quint (quintet), m (multiplet), dt (doublet of triplet), ddt (doublet of doublet of triplets), dtd (doublet of triplet of doublets), dq (doublet of quartets). Coupling constants (*J* values) are reported in Hertz (Hz).

High Resolution Mass Spectrometry data (HRMS) were obtained using a ThermoScientific Q Exactive HF-X mass spectrometer with electrospray ionization (ESI) in positive or negative mode and atmospheric pressure chemical ionization (APCI).

Electrochemical potentials were obtained with a standard set of conditions to main internal consistency.

1.1.3 General Information for Radiochemistry

All chemicals are ACS reagent grade purity or above and used without further purification. Ultrapure water was obtained from a Millipore MilliQ Gradient A10 system. Pre-conditioned Sep-PAK® light QMA cartridges were purchased from Waters and were flushed with 6 ml of water before use. Aqueous tetra-*n*-butylammonium bicarbonate ($TBAHCO_3$) solution (20% w/w) was prepared by bubbling CO_2 to the aqueous tetra-*n*-butylammonium hydroxide solution (20% w/w) diluted from a concentrated aqueous tetrabutylammonium hydroxide solution (54~56% w/w), which was purchased from Sigma Aldrich. The pH of the $TBAHCO_3$ solution was in the range of 9.0 to 10.0. Anhydrous ethanol was the Koptec's Pure Ethanol 200-Proof purchased from Fisher Scientific. Anhydrous *tert*-butyl alcohol (Purity: >99.0%, GC grade) was purchased from Fisher Scientific. ^{18}F activity was counted using a CRC-25 PET dose calibrator from Capintec. The reaction was timed with a lab timer. Ultrahigh-performance liquid chromatography (UHPLC) was acquired on a SHIMADZU chromatography system (Model CBM-20A) and

analysed using LabSolutions software. The λ absorbance detector and the model 2200 scaler ratemeter radiation detector was added to the UHPLC system. All radiochemical yields (yields) were calculated with respect to the starting ^{18}F activity of the eluted fluoride. The identities of the ^{18}F-labeled compounds were confirmed by comparison to authentic ^{19}F standards.

The PET/CT imaging was acquired by Sedecal Super Argus 4R PET/CT instrument in Small Animal Imaging Facility of the Biomedical Research Imaging Centre (BRIC) at the University of North Carolina at Chapel Hill.

1.2 Electrochemical Measurements

Electrochemical half peak redox potential ($E_{p/2}$) were obtained from cyclic voltammograms following the reported procedure.[1] All measurements were performed in CH_3CN with tetrabutylammonium hexafluorophosphate [0.1 M] as the electrolyte. Cyclic voltammograms were collected using a glassy carbon working electrode, a platinum wire counter electrode, and an Ag/AgCl reference electrode in saturated NaCl. The observed half peak potential was referenced to a saturated calomel electrode (SCE) by subtraction of 30 mV to the value obtained vs Ag/AgCl. For a typical measurement, the potential was increased from an initial potential of 0 V to a vertex potential of 2.7 V, then returning to a final potential of 0 V. With these parameters, all compounds listed in **Table S1** exhibited irreversible oxidation waves.

**Structure**	**Half peak redox potential ($E_{p/2}$) vs. SCE (V)**
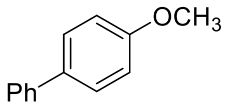	1.63
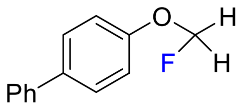	1.83

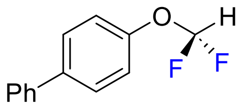	1.80
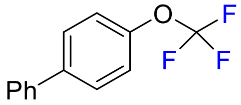	2.03
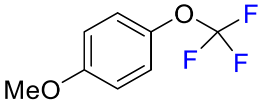	1.91
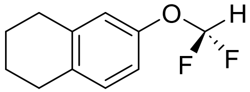	2.01
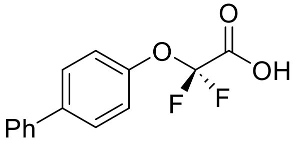	1.95
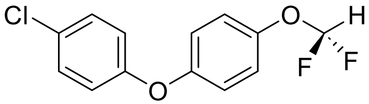	2.19

Table S1: Electrochemical half peak redox potential ($E_{p/2}$) for arenes examined.

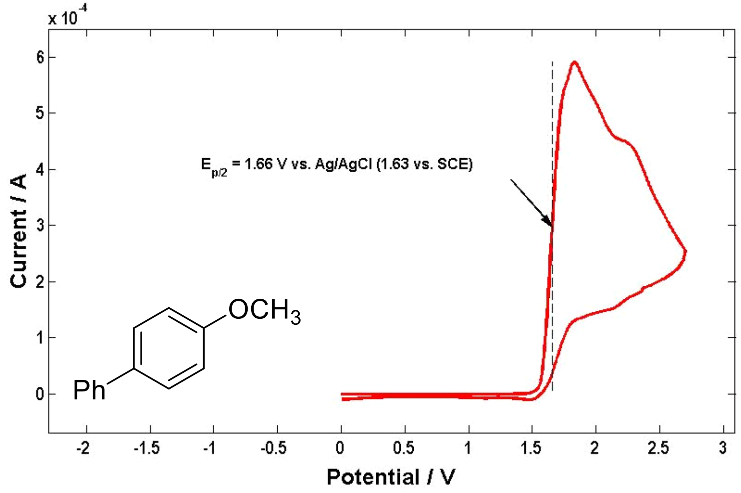

x 10⁻⁴
Current / A
Potential / V
E p/2 = 1.66 V vs. Ag/AgCl (1.63 vs. SCE)
OCH3
Ph

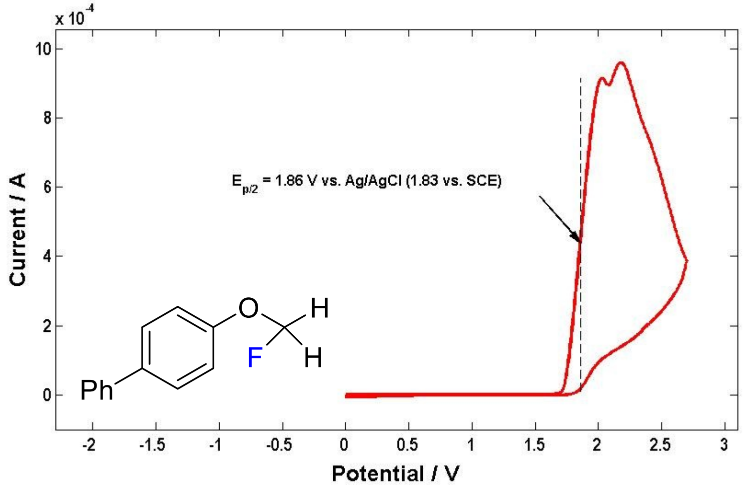

x 10⁻⁴
Current / A
Potential / V
E p/2 = 1.86 V vs. Ag/AgCl (1.83 vs. SCE)
O
H
F
H
Ph

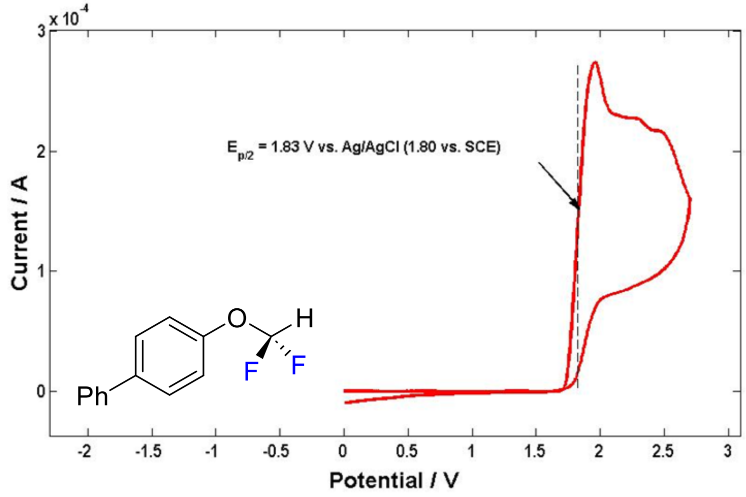

x 10⁻⁴
Current / A
Potential / V
E p/2 = 1.83 V vs. Ag/AgCl (1.80 vs. SCE)
O
H
F
F
Ph

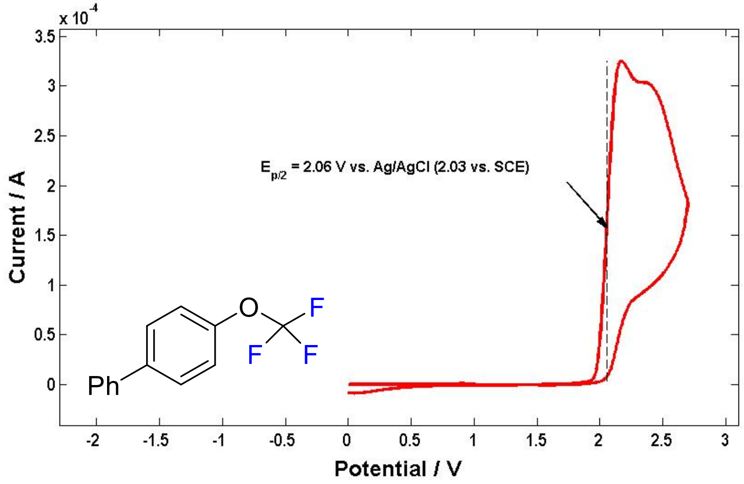

x 10⁻⁴
Current / A
Potential / V
E p/2 = 2.06 V vs. Ag/AgCl (2.03 vs. SCE)
O
F
F
F
Ph

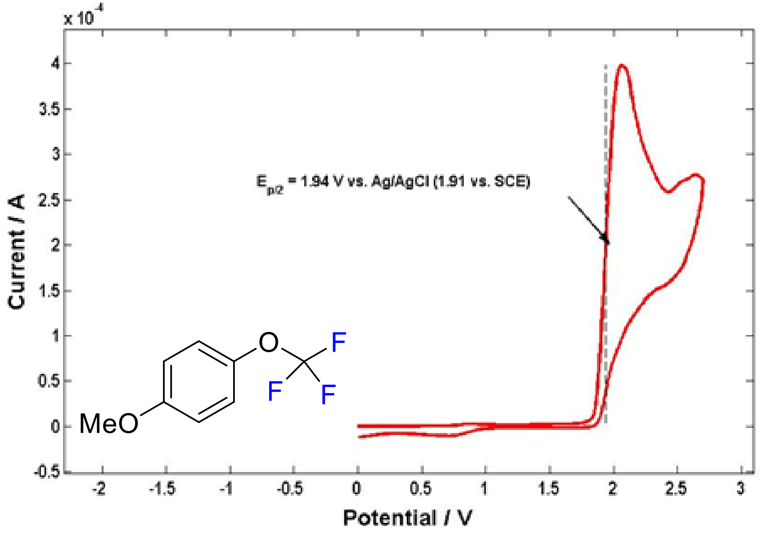

x 10^-4
Current / A
Potential / V
E_p/2 = 1.94 V vs. Ag/AgCl (1.91 vs. SCE)
MeO
O
F
F
F

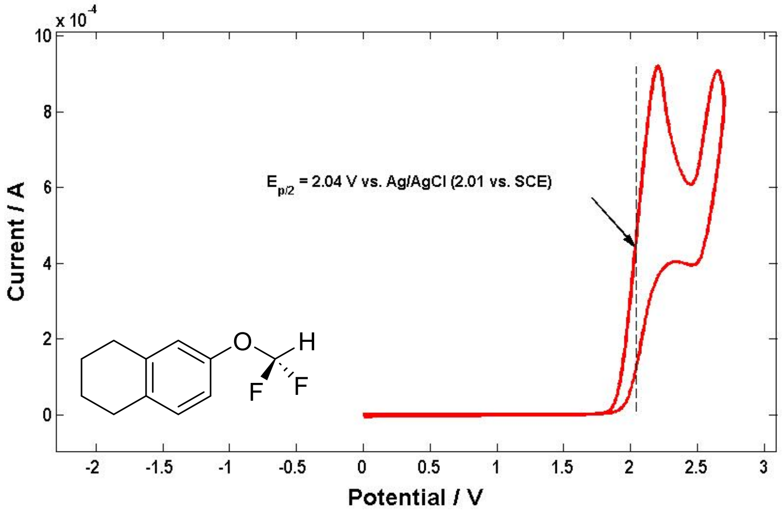

x 10^-4
Current / A
Potential / V
E_p/2 = 2.04 V vs. Ag/AgCl (2.01 vs. SCE)
O
H
F
F

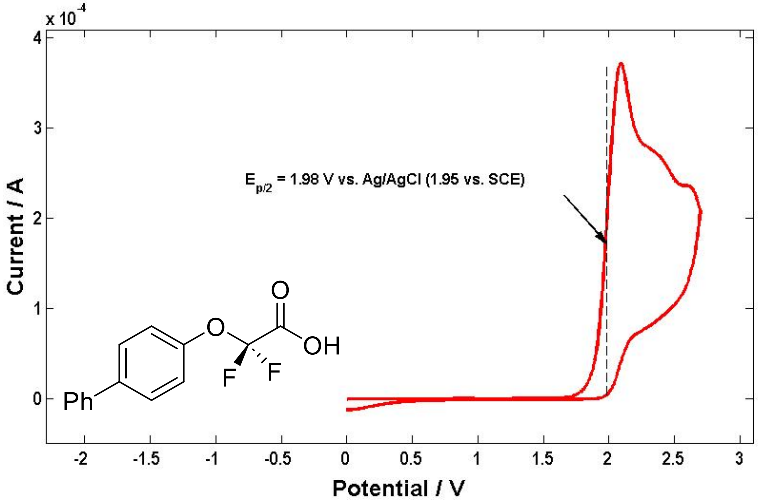

x 10^-4
Current / A
Potential / V
E_p/2 = 1.98 V vs. Ag/AgCl (1.95 vs. SCE)
Ph
O
O
OH
F
F

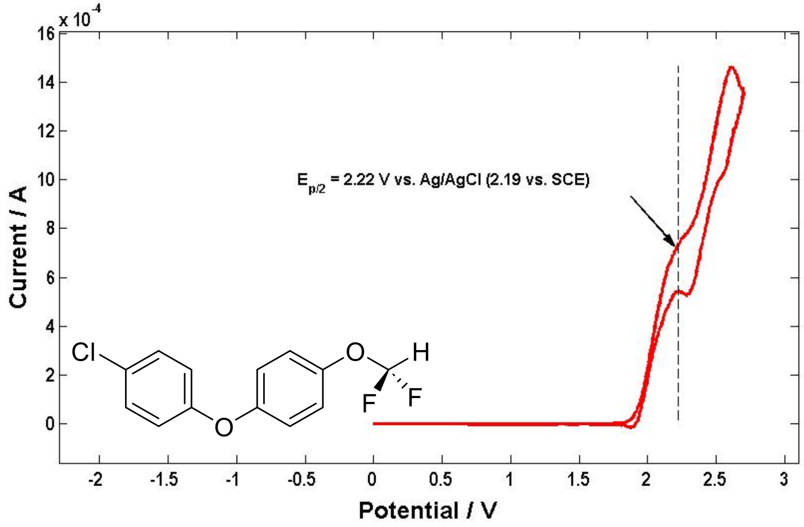

x 10^-4
Current / A
Potential / V
E_p/2 = 2.22 V vs. Ag/AgCl (2.19 vs. SCE)
Cl
O
O
H
F
F

1.3 Optimisation Studies and Miscellaneous Information for CRA-S_NAr Arene Deoxy(radio)fluorination

1.3.1 Optimisation studies for CRA-S$_N$Ar arene deoxy(radio)fluorination via a trifluoromethoxy nucleofuge

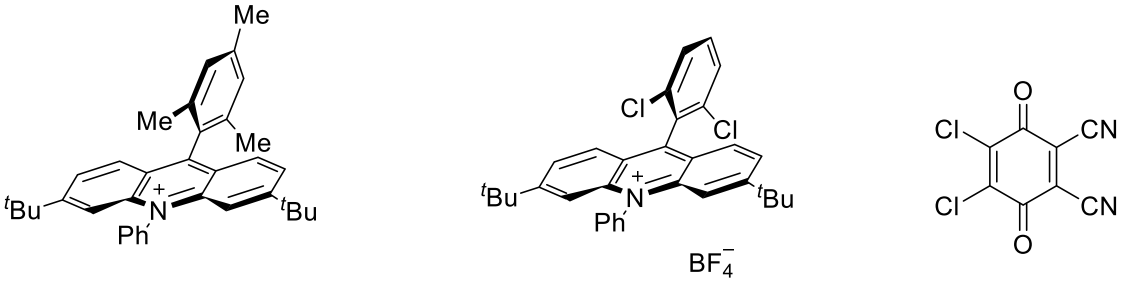

P1 ClO_4^- **Cat.BF$_4$** BF_4^- ($E_{1/2}$ = +2.11 V versus SCE)

P2 ($E_{1/2}$ = +2.21 V versus SCE)

P3 ($E_{1/2}$ = +3.18 V versus SCE)

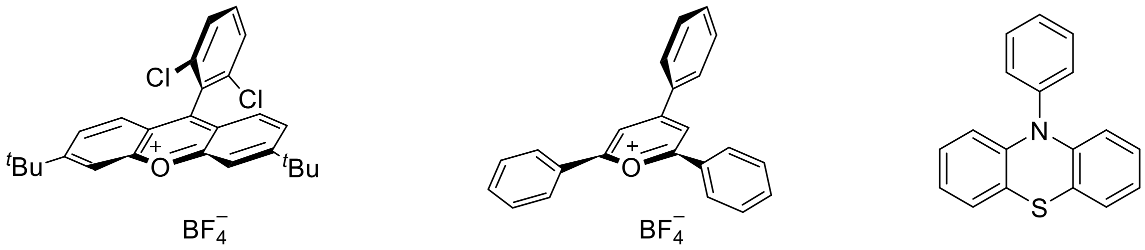

P4 ($E_{1/2}$ = +2.21 V versus SCE)

P5 ($E_{1/2}$ = +2.55 V versus SCE)

P6 ($E_{1/2}$ = +2.02 V versus SCE)

Figure S1: List of photocatalysts studied for CRA-S$_N$Ar arene deoxy(radio)fluorination via a trifluoromethoxy nucleofuge.

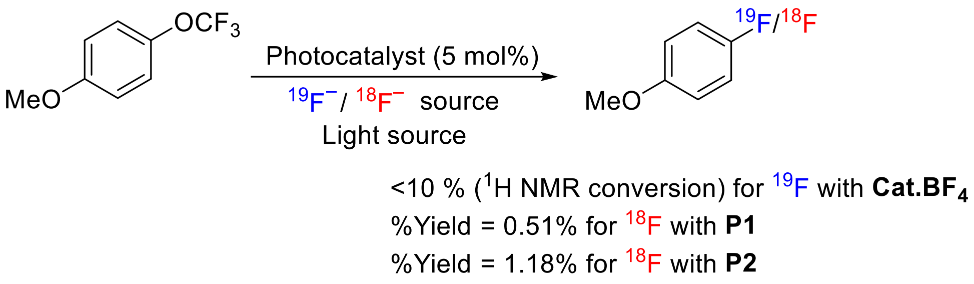

Reaction conditions:

For Cold-chemistry: Substrate (0.1 mmol, 1.0 equiv), Cat.BF_4 (0.05 equiv), CsF (5.0 equiv), $TBAHSO_4$ (0.75 equiv), CH_2Cl_2 : H_2O (1.2 mL : 50 μL), 450-455 nm LED, 48 h, 33 °C, air

For Hot-chemistry: Substrate (0.05 mmol, 1.0 equiv), P1 or P2 (0.05 equiv), [^{18}F]TBAF (5–25 mCi), $TBAHCO_3$ (25 μL), 450 nm laser, 30 min, rt, N_2 atm

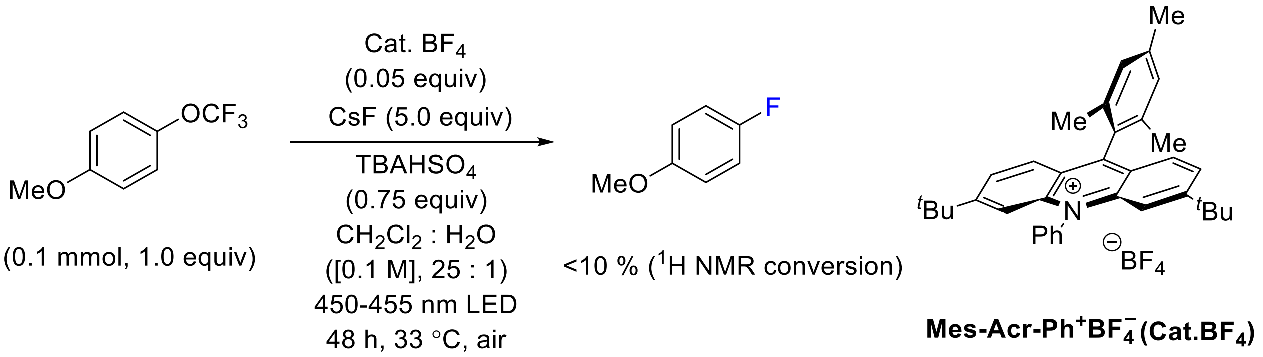

Entry	Deviation from above conditions	Yield based on ^{1}H NMR conversion (%)
**1**	after 72 h	<10
**2**	concentration doubled	4
**3**	1,2-dichloroethane: H_2O (1.2 mL : 50 µl), 70 °C	5
**4**	P4	2
**5**	P5	NR
**6**	P3	NR

NR means no reaction

Table S2: Attempted optimisation for $^{19}F^{-}$ photoredox-deoxyfluorination via a trifluoromethoxy nucleofuge.

1.3.2 Optimisation studies for CRA-S$_N$Ar arene deoxyfluorination via a difluoromethoxy nucleofuge

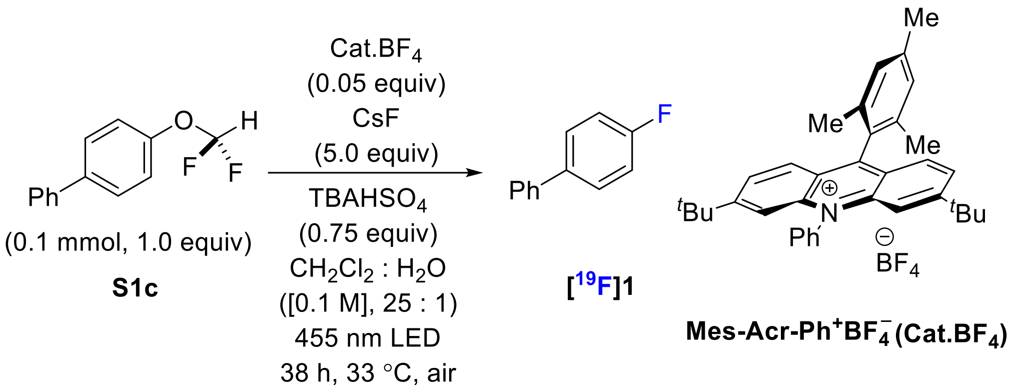

Entry	Deviation from standard conditions	Yield (%)
1	None	53[a]
2	P2	32[b]
3	P3	NR
4	P4	NR
5	P5	NR
6	P6*[2]	NR
7	Cat.BF_4*	>5 [b]
8	2.5 %v/v PEG	25[b]
9	38 h	73[b]
10	CH_2Cl_2 : H_2O [0.2 M], 38 h	59[b]
11	**O_2, 38 h**	**89[b], 76[a]**

12	O_2, 38 h, 18-Crown-6 (5.0 equiv)	4[b]
13	N_2 + TBHP (2.0 equiv)	3[b]
14	N_2 + H_2O_2 (2.0 equiv)	3[b]
15	N_2 + $K_2S_2O_8$ (2.0 equiv)	16[b]

[a]Isolated Yield, [b]based on ^{1}H NMR Conversion, NR means no reaction

Cat.BF_4^* = Cgr(Anode) Pt(Cathod), P1 (0.05 equiv), CsF (5.0 equiv), $TBAHSO_4$ (0.75 equiv), CH_2Cl_2 : H_2O ([0.1 M] 25 : 1, 455 nm LED, 24 h, rt, 1.5 V.

$P6^{*2}$ = P6 (0.05 equiv), O_2, $LiClO_4$ (0.2 equiv), CsF (5.0 equiv), TFE: HFIP (9: 1) 390 nm LED,18 h, rt

Table S3: Evaluation of model substrate **S1c** for $^{19}F^-$ photoredox-deoxyfluorination.

1.3.3 Substrate scope for CRA-S_NAr arene deoxyfluorination via a difluoromethoxy nucleofuge

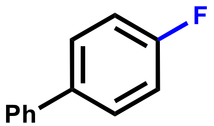

$[^{19}F]$1
76%[a]

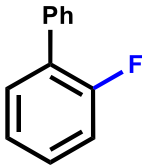

$[^{19}F]$2
47%[a]

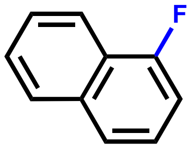

$[^{19}F]$3
48%[a]

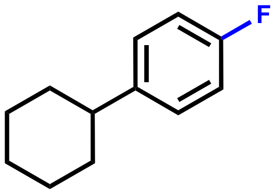

$[^{19}F]$4
12%[b]

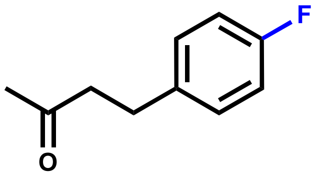

$[^{19}F]$6
13%[b]

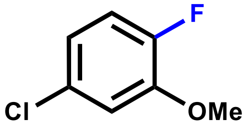

$[^{19}F]$7
40%[b]

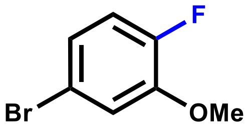

$[^{19}F]$8
35%[a]

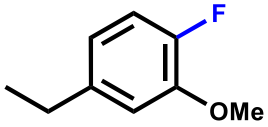

$[^{19}F]$10
13%[b]

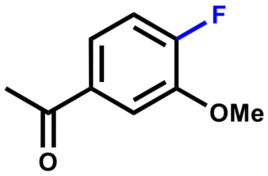

$[^{19}F]$12
18%[a]

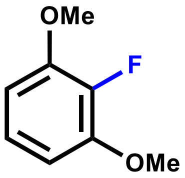

$[^{19}F]$14
10%[a]

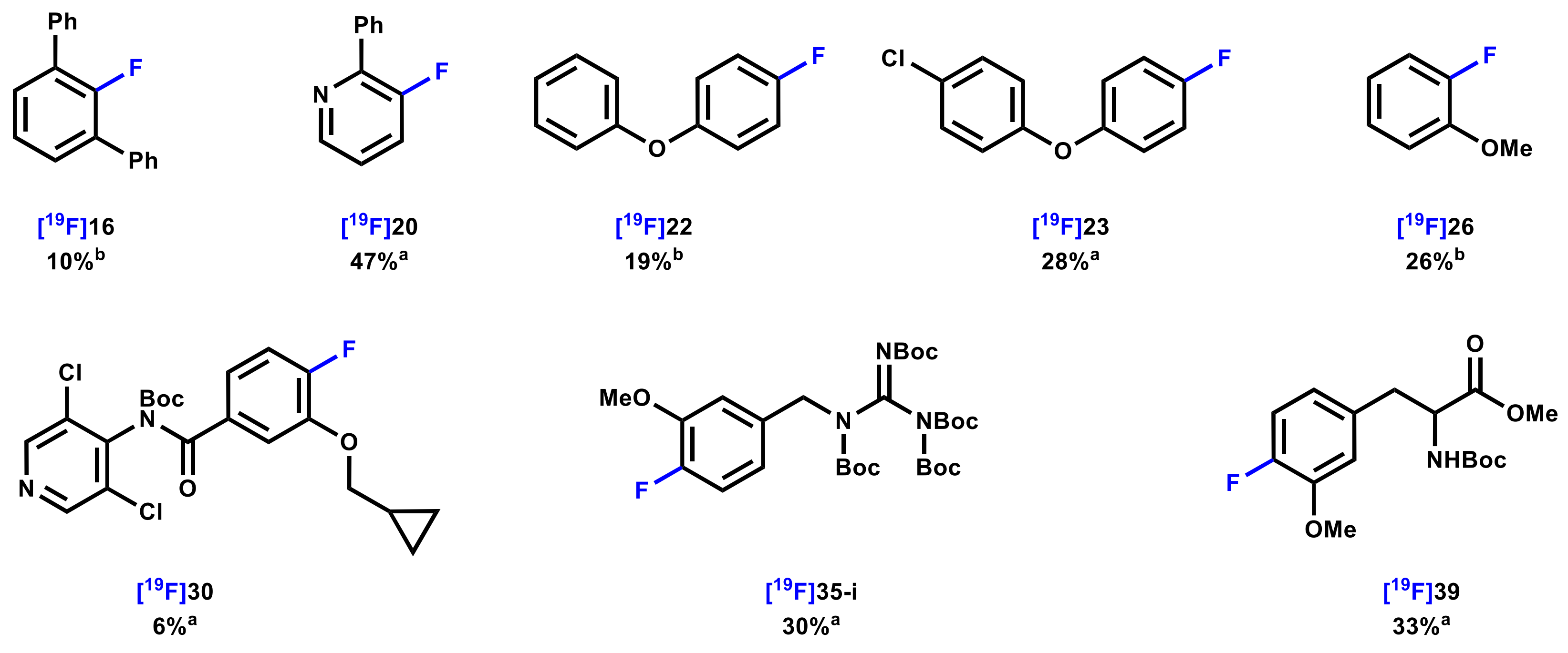

Figure S2: Exploration of substrate scope for CRA-S_NAr arene deoxyfluorination. [a]Isolated Yield, [b]based on ^1H NMR conversion.

1.3.4 Postulated mechanism for CRA-SNAr arene deoxyfluorination via a difluoromethoxy nucleofuge

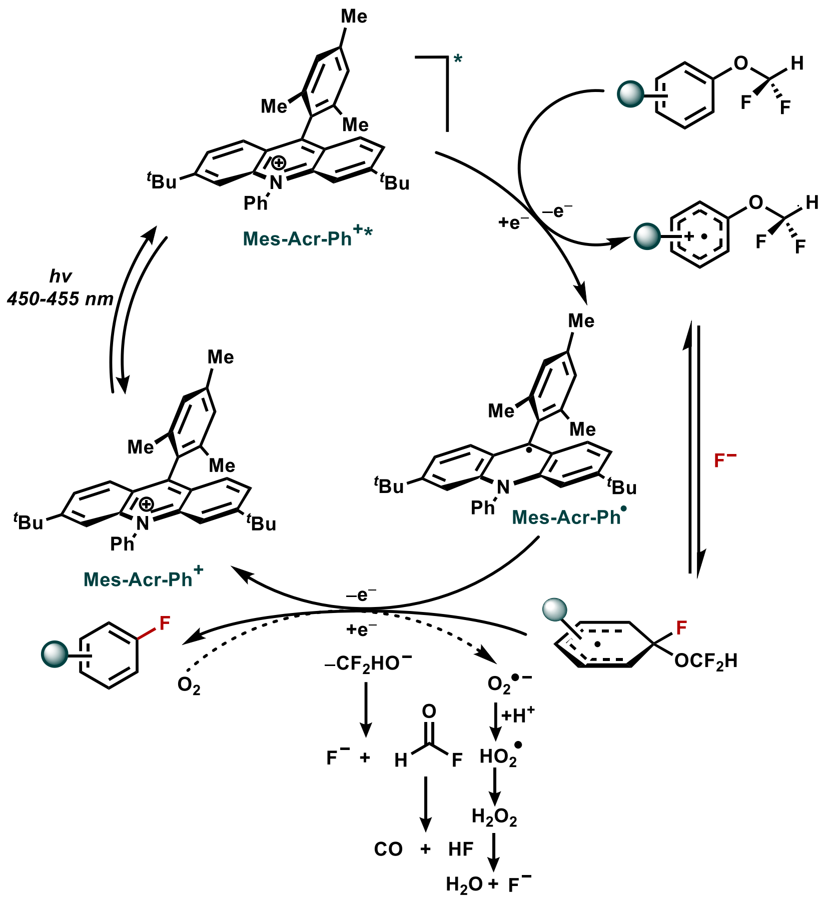

Figure S3: Postulated mechanism for CRA-SNAr arene deoxyfluorination via a difluoromethoxy nucleofuge.

1.4 Experimental Procedures

1.4.1 General conditions for difluoromethylation of phenols (Synthesis of precursors)

Conditions A

Diethyl (bromodifluoromethyl)phosphonate (2 equiv) was added to a solution of appropriate phenol (1 equiv) and KOH (20 equiv) in CH_3CN : H_2O (1:1) at 0 °C. The reaction mixture was allowed to stir at 0 °C. After 20 minutes to 4 h (stated for individual precursor synthesis), the reaction mixture was diluted with EtOAc (10 mL), and the organic phase was separated. The aqueous layer was extracted with EtOAc (2 ×5 mL). The combined organics dried (Na_2SO_4) and concentrated under reduced pressure, which was subsequently purified by column chromatography as described.

Conditions B

A solution of phenol (1.0 equiv) in THF was added dropwise to a slurry of NaH (90% or 60%, 10 equiv) in THF at 0 °C under N_2 atmosphere. After stirring the slurry for 30 min at 0 °C, H_2O (50 equiv) was slowly added at 0 °C (exothermic reaction) and the reaction mixture allowed to stir for 10 minutes. Diethyl (bromodifluoromethyl)phosphonate (2 equiv) was slowly added at 0 °C (exothermic reaction). When effervescence of gases ceased, the reaction mixture was brought to room temperature and stirred for another 2 h. The reaction mixture was added with EtOAc and brine, and the aqueous phase was extracted with EtOAc (3 ×10 mL), the combined organics dried (Na_2SO_4) and concentrated under reduced pressure. The resulting crude was purified using column chromatography to furnish the pure compound.

Conditions C

A 100 mL Schlenk tube was charged with Phenol (0.1 mmol), [fac-Ir(ppy)$_3$] (1 mol%), and Cs_2CO_3 (10 equiv). The Schlenk tube was purged and degassed with argon three times. Difluorobromoacetic acid (1.0 equiv) in degassed anhydrous DMF was added to the Schlenk tube and the reaction mixture was irradiated with 23W CFL under argon atmosphere overnight. Next morning, ice-cold water was added, and the aqueous

phase was extracted with EtOAc (2 ×10 mL). The combined organics was dried (Na_2SO_4), concentrated under reduced pressure, and purified by column chromatography.

1.4.2 General conditions for CRA-S_NAr arene deoxyfluorination via a difluoromethoxy nucleofuge – ^{19}F chemistry

Conditions I

To an oven dry cylindrical quartz glass tube equipped with a Teflon-coated magnetic stirrer was added CsF (5 equiv) under an inert atmosphere. After removing from the inert atmosphere, the tube was charged with **Cat.BF$_4$** (0.05 equiv), tetrabutylammonium hydrogen sulphate, $TBAHSO_4$ (0.75 equiv), and the arene substrate (1.0 equiv). The reaction mixture was dissolved in CH_2Cl_2 [0.083 M] and Milli Q water [0.5 M] was subsequently added. The reaction tube was then sealed with a Telfon-lined septum screw cap and an electric tape before placing air / N_2 / O_2 balloon (as stated for the individual compound). The tube was positioned on a stir plate and irradiated with blue LED lamps (450–455 nm) (**Figure S4)** for the time specified. The crude reaction mixture was concentrated under reduced pressure and purified by column chromatography as described.

1.4.2.1 Photoreactor configuration for ^{19}F–chemistry

Small-scale reactions (0.1–0.3 mmol) in cylindrical quartz tubes were irradiated using Blue LED lamps (450–455 nm). For the large-scale reaction (1.37 mmol), round bottom flask (RBF) was used (**Figure S4**). A cooling fan was installed on the top to the reactor to aid in heat dissipation – generated from nonradiative decay pathways of the excited state catalyst and from high power LEDs. An equilibrium temperature of 33 °C was measured with a standard alcohol thermometer. The cylindrical quartz glass tubes and blue LED lamps (450–455 nm) were provided by LED Radiofluidics Corp.

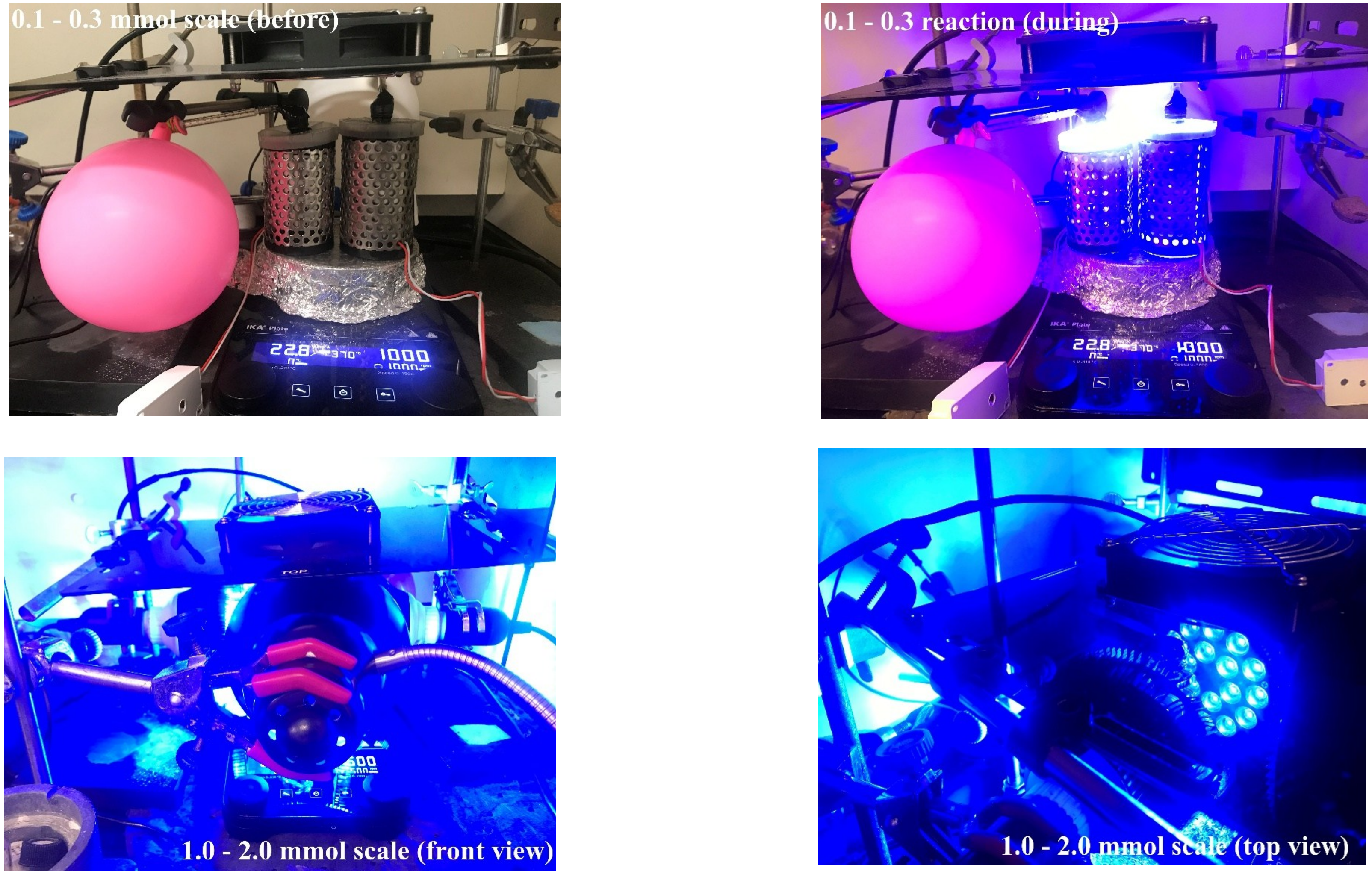

Figure S4: Photocatalysed fluorination set-ups using blue LEDs in fume hood.

1.4.3 Procedure for anhydrous [^{18}F]TBAF solution in CH_3CN

[^{18}F]Fluoride was produced via the $^{18}O(p,n)^{18}F$ reaction by proton irradiation (40 µA, 1 h) of an [^{18}O]H_2O containing target in a GE PETTrace cyclotron. The aqueous solution of [^{18}F]fluoride was delivered into a hot cell and passed through a QMA cartridge (water preconditioned) to trap the [^{18}F]fluoride. The [^{18}F]fluoride was eluted into a 5 mL V-vial (sealed with a Teflon-lined septum screw cap) with 70 µL tetrabutylammonium bicarbonate ($TBAHCO_3$) solution (20%, w/w), 100 µL H_2O and 500 µL CH_3CN. This solution was azeotropically dried with CH_3CN (1 mL x 4) under a stream of nitrogen gas at 95 °C. The resulting residue was diluted with 1 mL anhydrous CH_3CN to obtain the [^{18}F]TBAF solution (typically 1.5–1.9 Ci), which was further used for the labeling reactions.

1.4.4 General conditions for CRA-S$_N$Ar arene radiodeoxyfluorination via a difluoromethoxy Nucleofuge – ^{18}F chemistry

The precursor (0.01 mmol to 0.05 mmol), photocatalyst **P1** (1.5 mg) were weighed into a 4 mL scintillation vial, dissolved in anhydrous 1,2-DCE (300 µL), anhydrous ^{t}BuOH (400 µL), anhydrous CH_3CN (45–65 µL, total volume of CH_3CN 100 µL), and transferred into a 5 mL V-vial. A solution of $TBAHCO_3$ in CH_3CN (25 µL, ~ 60 mg/mL) was added before a 10–30 µL aliquot of [^{18}F]TBAF in anhydrous CH_3CN (typically 5–25 mCi) was added to the reaction vial via pipette. The reaction V-vial was then affixed to a block (LED Radiofluidics Corp) and cooled using an ice-bath. A needle connecting to an air / O_2 / N_2 filled balloon was inserted to the bottom of the V-vial and the reaction medium was continuously sparged. The reaction was then irradiated top-down with an optic fiber of the 450 nm blue diode laser (MDL-D-450, 450nm, the power rating was set to 2.35 W after fiber coupling) for 30 minutes (**Figure S5**). ^{18}F activity was recorded at the end of the reaction. The resulting solution was filtered through an alumina cartridge (when described), and the resulting solution was diluted with H_2O and evenly mixed with CH_3CN (200 µL). An aliquot of the reaction mixture (typically 200 – 1000 µCi) was taken for radio-HPLC analysis.

1.4.4.1 Photoreactor configuration for ^{18}F–chemistry

The 450 nm blue diode laser (MDL-D-450, 450 nm, the power rating set to 2.35 W after fiber coupling) used for the labeling reaction was purchased from Changchun New Industries Optoelectronics Tech. Co. Ltd.

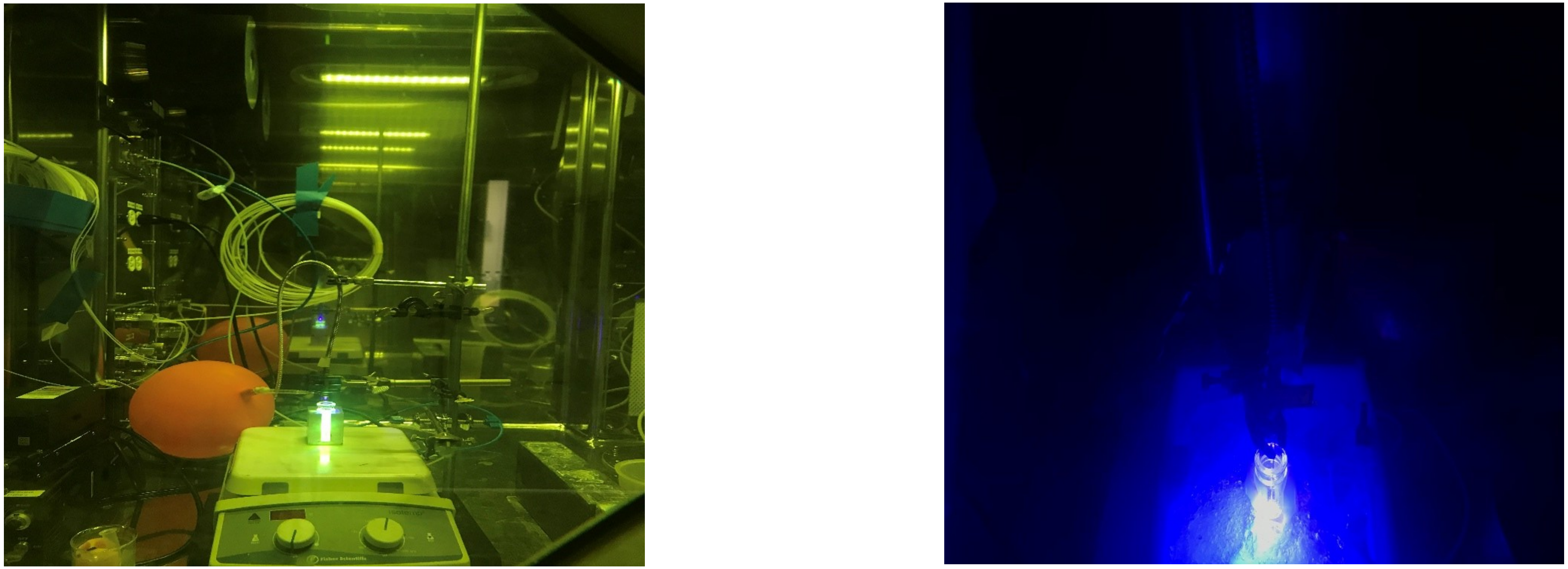

Figure S5: Photocatalysed radiofluorination set-ups using blue diode laser in hot cell.

1.4.5 General HPLC conditions for characterisation of ^{18}F-tracers

Column: Phenomenex, Kinetex® 5μm F5 100 Å, 250 × 4.6 mm LC Column (unless otherwise specified)

Solvent A: 0.1% TFA water, Solvent B: 0.1% TFA acetonitrile (unless otherwise specified)

Example 5–95 method: Isocratic / Gradient elution: 5% Solvent B for 0 to 2 min, **5% – 95% Solvent B for 2 to 22.5 min**, 95% Solvent B for 22.5 to 28 min, 95% – 5% Solvent B for 28 to 30 min, and 5% Solvent B for 30 to 35 min. Flow rate: 1 mL/min.

All the radiochemical reactions were subjected to radio-HPLC using general HPLC conditions, where the gradient (highlighted above) for Solvent B for 2 to 22.5 min was tuned for each tracer for the better separation as mentioned in the respective section. The HPLC conditions for quality control (QC) of each labeled arene is also listed in the respective section.

1.4.6 Radiochemical Yield (%Yield) calculation (Isolated %Yield)

At the end of the reaction, ^{18}F activity was recorded (this activity denoted **a**). The reaction solution was filtered through an alumina cartridge (when described), V-vial was rinsed with CH_3CN (200 µL), passed through the same alumina cartridge, and ^{18}F activity was again recorded (this activity denoted **b**). An aliquot of the reaction mixture (typically 200 – 1000 µCi) was taken, diluted with MilliQ H_2O (400 – 600 µL) before injecting for radio-HPLC analysis. The activity injected into HPLC was measured, and the time was recorded, and the decay-corrected corresponding to the time **(t)** of the isolation of the desired radiotracer (this activity denoted **c)**. The fraction corresponding to radiotracer was collected and its activity was measured (this activity denoted **d**) and the time **(t)** was recorded. The isolated decay-corrected radiochemical yield (%Yield) was calculated by (**b**/**a** ×100) × (**d**/**c** ×100) / 100.

$$\text{Alumina Filtration Yield } (\mathbf{X}) = \frac{\text{18F activity after alumina flitration } (\mathbf{b})}{\text{18F activity at the end of the reaction } (\mathbf{a})} \times 100$$

$$\text{HPLC Purity of Filtrate } (\mathbf{Y}) = \frac{\text{18F activity of the desired radiotracer } (\mathbf{d})}{\text{18F activity injected into HPLC (decay−corrected)} (\mathbf{c})} \times 100$$

$$\%\text{Yield} = \frac{\mathbf{X} \times \mathbf{Y}}{100}$$

QC was run separately to ensure the purity of the isolated radiolabeled compound.

Commercial or synthesised (as described) ^{19}F–standards were used to confirm the identity of the radiolabeled compound.

1.4.7 Experimental Data

For each substrate, the experimental data is given in the following order.

i. Synthesis and characterisation of arene substrate

ii. Synthesis and characterisation of authentic fluoroarene standards / CAS number of commercially available fluoroarenes

iii. Radio-HPLC analysis and characterisation for ^{18}F–radiolabled arenes

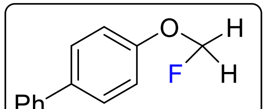

S1b

Arene substrate

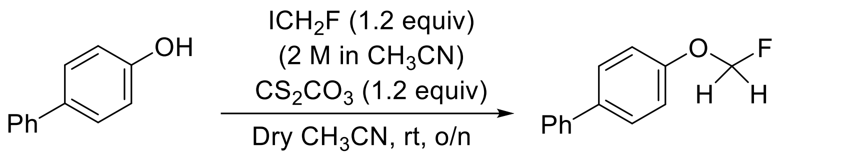

(170 mg, 1.0 mmol
1.0 equiv)

(202 mg, >99% crude yield)

4-(Fluoromethoxy)-1,1'-biphenyl (S1b)

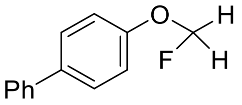

To a mixture of [1,1'-biphenyl]-4-ol (170 mg, 1.0 mmol, 1.0 equiv), Cs_2CO_3 (392 mg, 1.2 mmol, 1.2 equiv) in dry CH_3CN (2 mL, 0.5 M), fluoroiodomethane (0.6 mL, 1.2 mmol, 1.2 equiv, 2 M in CH_3CN) was added slowly at rt. The reaction mixture was stirred overnight at rt then quenched with water next morning. CH_3CN was evaporated under reduced pressure and aqueous layer was extracted with EtOAc (3 ×5 mL).

The combined organics washed with 1 N NaOH and brine, dried (Na_2SO_4) and concentrated under reduced pressure to give **S1b** as a white solid (202 mg, > 99% crude yield).

Note: The compound **S1b** was not stable on silica gel column and directly utilised for the next reaction.

^{1}H NMR ($CDCl_3$, 400 MHz): δ 7.58–7.55 (m, 4H), 7.43 (tt, J = 7.3, 1.8 Hz, 2H), 7.33 (tt, J = 7.3, 1.2 Hz, 1H), 7.16 (dt, J = 8.4, 2.8 Hz, 2H), 5.82 (d, J= 54.8 Hz, 2H).

^{13}C NMR ($CDCl_3$, 101 MHz): δ 156.43 (d, J = 3.6 Hz), 140.56, 136.82, 128.93, 128.54, 127.25, 126.06, 117.08 (d, J = 1.4 Hz), 100.92 (d, J= 218.4 Hz).

^{19}F NMR ($CDCl_3$, 376 MHz): δ –148.46 (t, J = 54.1 Hz).

<u>Deoxyfluorination and Authentic fluoroarene standard</u>

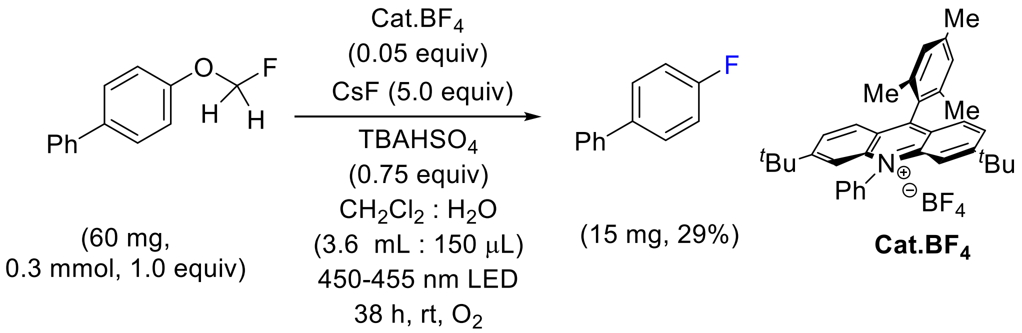

4-Fluoro-1,1'-biphenyl ([^{19}F]1)

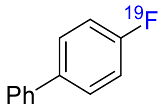

General conditions **I** were followed using 4-(fluoromethoxy)-1,1'-biphenyl (**S1b**) (60 mg, 0.3 mmol, 1.0 equiv), Mes-Acr-$Ph^+BF_4^-$ (0.05 equiv), CsF (5.0 equiv), $TBAHSO_4$ (0.75 equiv), CH_2Cl_2 : H_2O (0.1 M, 25:1), 450-455 nm LED, 38 h, 33 °C, O_2.

Purification: Isocratic column chromatography [SiO_2, EtOAc:Hexanes (02:98)] to obtain white solid of **[^{19}F]1** (15 mg, 29%).

R_f : 0.6 (EtOAc : Hexanes 0.2:9.8)

^{1}H NMR ($CDCl_3$, 400 MHz): δ 7.57–7.52 (m, 4H), 7.43 (tt, J = 7.3, 1.8 Hz, 2H), 7.34 (tt, J = 7.3, 1.2 Hz, 1H), 7.12 (dt, J = 8.7, 2.1 Hz, 2H).

^{13}C NMR ($CDCl_3$, 101 MHz): δ 163.59, 161.64, 140.40, 137.50 (d, J = 2.9 Hz), 128.95, 128.82 (d, J = 7.9 Hz), 127.39, 127.16, 115.85, 115.64.

^{19}F NMR ($CDCl_3$, 376 MHz): δ –115.88 (ddd, J = 14.4, 8.3, 4.1 Hz).

HRMS (ESI-TOF) m/z: $[M + H]^+$ Calcd. for $C_{12}H_{10}F$ 173.0767; found 173.0766.

Data are comparable to that commercially available compound. CAS number: 324-74-3

Radio-HPLC analysis and characterization

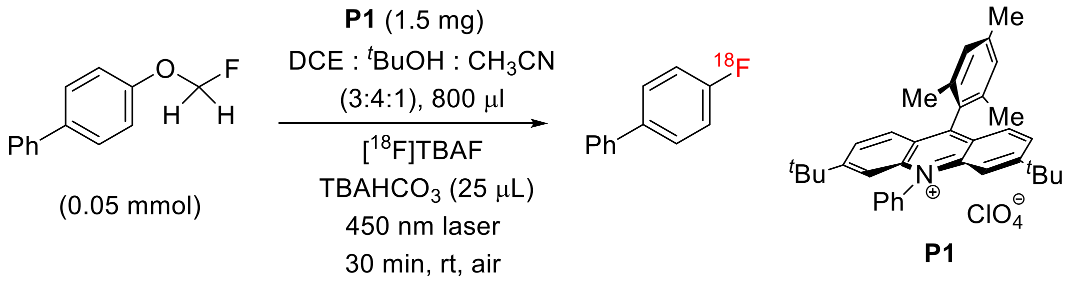

Entry	Activity at starting	Activity at EOS	Alumina Filtration	Injected Dose	Collected Dose	Isolation Time	Decay Corrected	Alumina Filtration Yield	HPLC Purity of Filtrate	%Yield
1*	15.13 mCi	10.56 mCi	3.98 mCi	289 µCi	82 µCi	17.0 min	259.58 µCi	37.68%	31.58%	11.89%
2**	14.76 mCi	10.93 mCi	4.09 mCi	356 µCi	261 µCi	5.0 min	344.93 µCi	37.41%	75.66%	28.30%
										**Average RCY: 20.1% (n=2)**

Table **S4**: HPLC isolated RCYs for **[^{18}F]1**.

*HPLC Conditions – method 1: Column: Phenomenex, Kinetex® 5µm F5 100 Å, 250 × 4.6 mm LC Column

Solvent A: 0.1% TFA water, Solvent B: 0.1% TFA acetonitrile; Isocratic / Gradient elution: 20% Solvent B for 0 to 2 min, 20% – 95% Solvent B for 2 to 22.5 min, Flow rate: 1 mL/min.

** HPLC Conditions – method 2: Column: CHIRALPAK® 5µm AD-H 250 × 4.6 mm LC Column

Solvent A: Hexane; Solvent B: ^{i}PrOH; Isocratic 0 to 22 min: 0.1% solvent B. Flow rate: 1 mL/min.

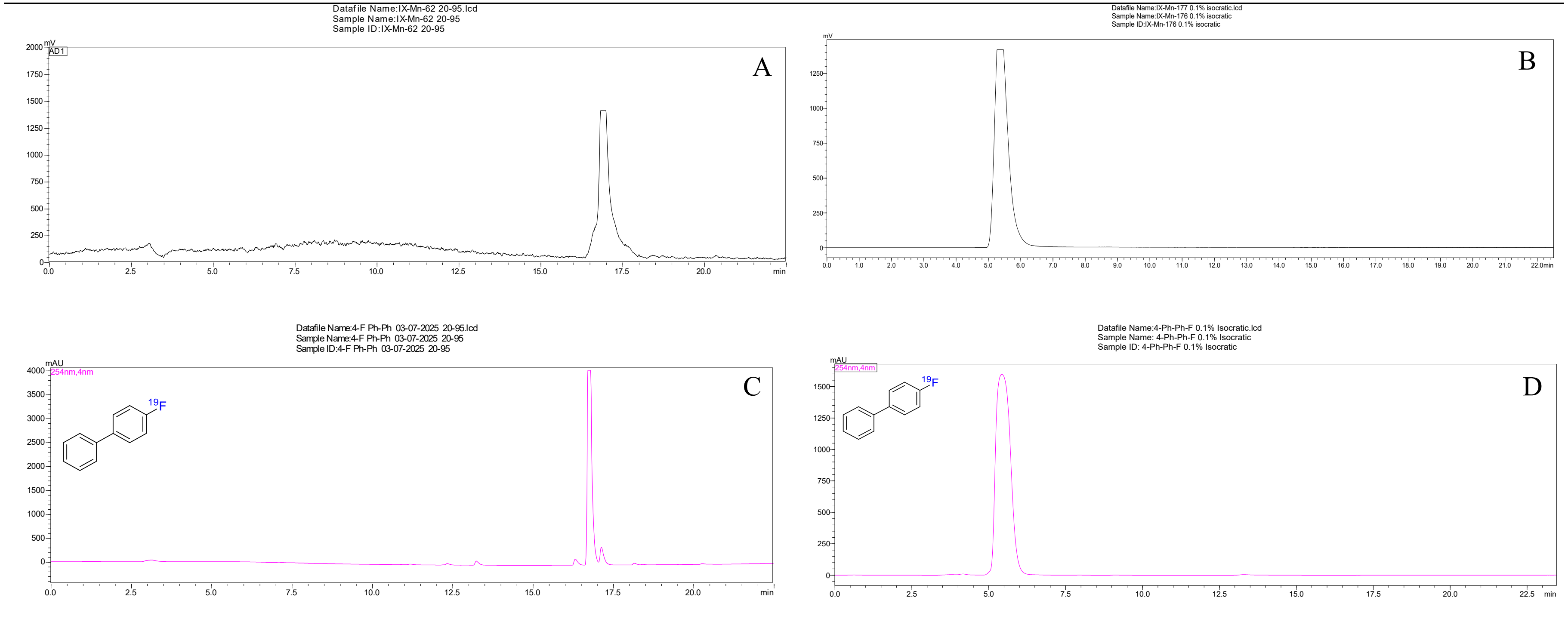

Figure S6: Radio-HPLC analysis of reaction mixture from **S1b**. Reaction mixture with HPLC method 1 (A), authentic **[^{19}F]1** with HPLC method 1 (C), reaction mixture with HPLC method 2 (B), and authentic **[^{19}F]1** with HPLC method 2 (D).

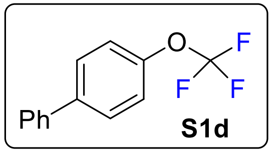

Arene substrate

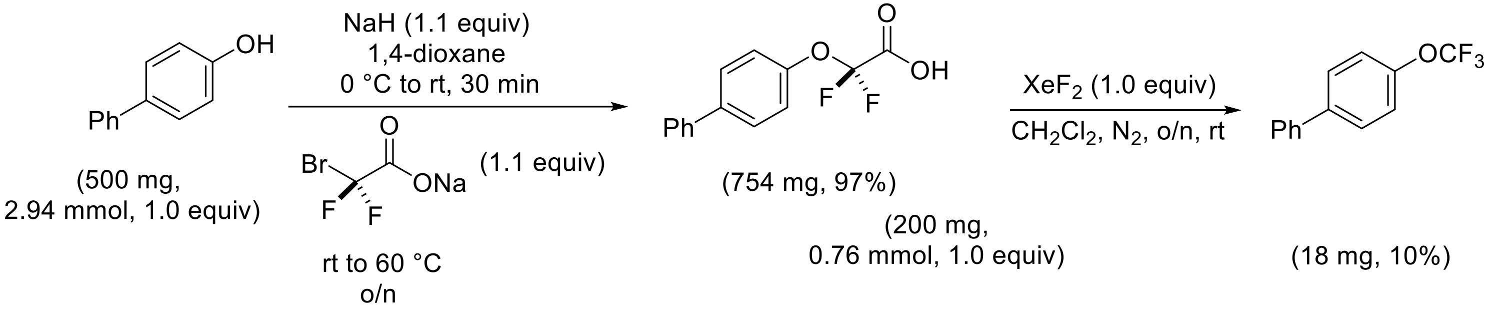

2-([1,1'-Biphenyl]-4-yloxy)-2,2-difluoroacetic acid (S1d-I1)

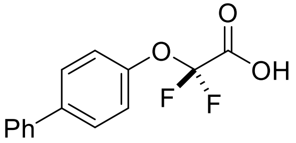

A solution of [1,1'-biphenyl]-4-ol (500 mg, 2.94 mmol, 1.0 equiv) in 3 mL 1,4-dioxane was added dropwise to a slurry of NaH (86 mg, 3.23 mmol, 1.1 equiv) in 3 mL 1,4-dioxane at 0 °C under N_2 atmosphere. The slurry was stirred at room temperature for 30 min, then $BrCF_2COONa$ (633 mg, 3.23 mmol, 1.1 equiv) and 4 mL 1,4-dioxane were added at 0 °C. The mixture was heated at 60 °C overnight. Next morning, the reaction mixture was cooled down to room temperature and acidified with 3 M HCl to pH = 1. The aqueous phase was extracted with EtOAc (3 × 5 mL), the combined organics dried (Na_2SO_4) and concentrated under reduced pressure, which was subsequently washed three times with n-heptane and dried under vacuum to obtain the title compound **S1d-I1** as a white solid (754 mg, 97% crude yield).

^{1}H NMR (MeOD-d_4, 400 MHz): δ 7.65 (dt, J = 8.7, 2.9 Hz, 2H), 7.62–7.59 (m, 2H), 7.44 (tt, J = 7.2, 1.8 Hz, 2H), 7.35 (tt, J = 7.4, 1.5 Hz, 1H), 7.30 (d, J = 8.8 Hz, 2H).

^{13}C NMR (MeOD-d_4, 101 MHz): δ 150.55 (t, J = 2.2 Hz), 140.98 (d, J = 58.3 Hz), 129.59 (d, J = 68.3 Hz), 128.57, 127.98, 122.80, 117.18 (q, J = 272.5 Hz).

^{19}F NMR (MeOD-d_4, 376 MHz): δ –78.42.

HRMS (ESI-TOF) m/z: $[M – H]^+$ Calcd. for $C_{14}H_9F_2O_3$ 263.0520; found 263.0526.

4-(Trifluoromethoxy)-1,1'-biphenyl (S1d)

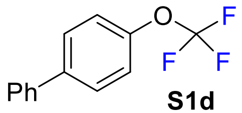

S1d

In a glove box, to a solution of 2-([1,1'-biphenyl]-4-yloxy)-2,2-difluoroacetic acid **S1d-I1** (200 mg, 0.76 mmol, 1.0 equiv) in dry CH_2Cl_2 (10 mL) in 20 mL scintillation vial was added XeF_2 (128 mg, 0.76 mmol, 1.0 equiv). The reaction mixture was stirred at room temperature overnight. Next morning, reaction vial was transferred to fume hood, water (20 mL) was carefully added, and organic phase was separated. The aqueous layer was extracted with CH_2Cl_2 (2 × 5 mL). The combined organics dried (Na_2SO_4), concentrated under reduced pressure, and the crude mixture was purified using column chromatography.

Purification: Gradient column chromatography [SiO_2, EtOAc:Hexanes 00:100 to 0.5:99.5)] to obtain white solid of **S1d** (18 mg, 10%).

R_f : 0.8 (EtOAc : Hexanes 0.2:9.8)

^{1}H NMR ($CDCl_3$, 400 MHz): δ 7.60 (dt, J = 8.7, 2.0 Hz, 2H), 7.57–7.55 (m, 2H), 7.45 (tt, J = 7.2, 1.8 Hz, 2H), 7.37 (tt, J = 7.2, 2.1 Hz, 1H), 7.28 (d, J = 8.1 Hz, 2H).

^{13}C NMR ($CDCl_3$, 101 MHz): δ 148.79 (d, $J = 2.5$ Hz), 140.14, 140.00, 129.03, 128.60, 127.80, 127.26, 121.36, 120.67 (q, $J = 257.1$ Hz).

^{19}F NMR ($CDCl_3$, 376 MHz): δ –57.80.

Data are comparable to that reported in literature.[3]

Deoxyfluorination and Authentic fluoroarene standard

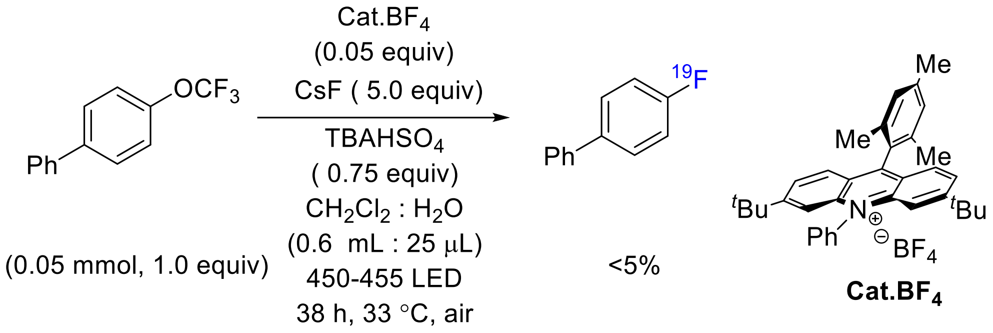

General conditions **I** were followed using 4-(trifluoromethoxy)-1,1'-biphenyl **S1d** (12 mg, 0.05 mmol, 1.0 equiv), Mes-Acr-$Ph^+BF_4^-$ (0.05 equiv), CsF (5.0 equiv), $TBAHSO_4$ (0.75 equiv), CH_2Cl_2 : H_2O (0.1 M, 25:1), 450-455 nm LED, 38 h, 33 °C, O_2.

Data are comparable to that commercially available compound. CAS number: 324-74-3

Radio-HPLC analysis and characterization

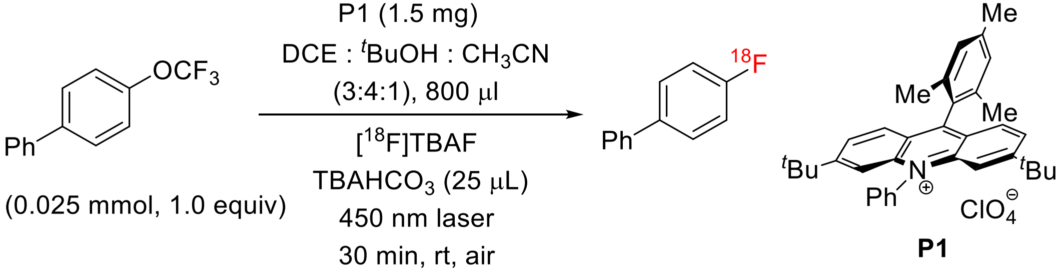

Entry	Activity at starting	Activity at EOS	Alumina Filtration	Injected Dose	Collected Dose	Isolation Time	Decay Corrected	Filtration Yield	HPLC Purity of Filtrate	%Yield
1*	16.91 mCi	13.19 mCi	–	942 µCi	34 µCi	19.2 min	833.74 µCi	–	–	4.0%

Table S5: HPLC isolated RCYs for **[^{18}F]1**.

HPLC conditions – method 1: Column: Phenomenex, Gemini® 5µm F5 100 Å, 250 × 4.6 mm LC Column

Solvent A: 0.1% TFA water, Solvent B: 0.1% TFA acetonitrile; Isocratic / Gradient elution: 40% Solvent B for 0 to 2 min, 40% – 95% Solvent B for 2 to 22.5 min, Flow rate: 1 mL/min.

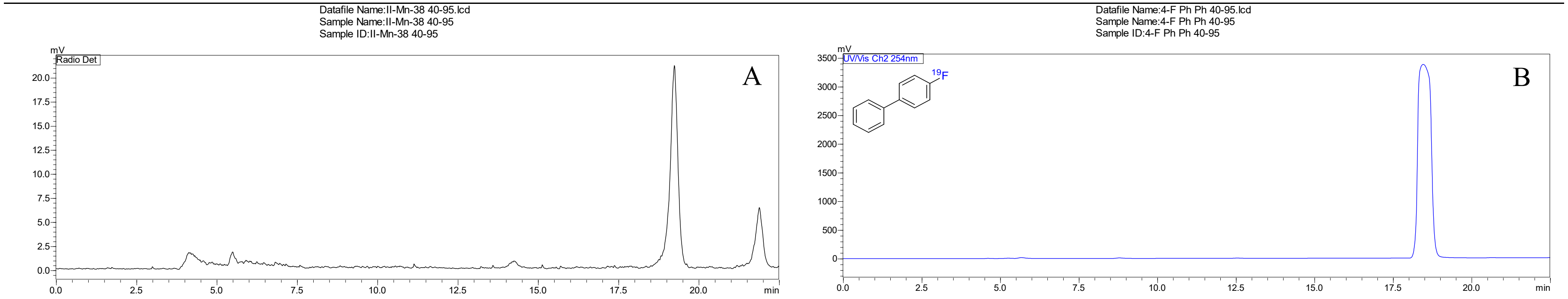

Figure S7: Radio-HPLC analysis of reaction mixture from **S1d**. Reaction mixture with HPLC method-1 (A) and authentic **[^{19}F]1** with HPLC method-1 (B).

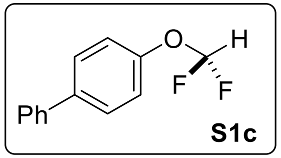

<u>Arene substrate:</u>

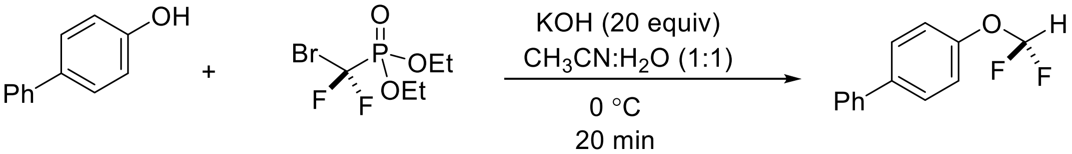

(340 mg, 2.0 mmol, 1.0 equiv) (2.0 equiv) (226 mg, 51%)

4-(Difluoromethoxy)-1,1'-biphenyl (S1c)

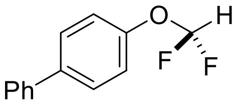

General conditions **A** were followed using [1,1'-biphenyl]-4-ol (340 mg, 2.0 mmol, 1.0 equiv), $BrCF_2P(O)(OC_2H_5)_2$ (1.068 g, 4.0 mmol, 2.0 equiv), KOH (2.24 g, 40 mmol, 20 equiv), CH_3CN: H_2O (5 mL : 5 mL) for 20 min.

Purification: Gradient column chromatography [SiO_2, EtOAc:Hexanes 00:100 to 2:98)] to obtain white solid of **S1c** (226 mg, 51%).

R_f : 0.8 (EtOAc : Hexanes 0.2:9.8)

^{1}H NMR ($CDCl_3$, 400 MHz): δ 7.57 (dt, J = 8.8, 2.8 Hz, 4H), 7.44 (tt, J = 7.3, 1.8 Hz, 2H), 7.36 (tt, J = 7.1, 1.2 Hz, 1H), 7.19 (d, J = 8.6 Hz, 2H), 6.55 (t, J= 73.9 Hz, 1H).

^{13}C NMR ($CDCl_3$, 101 MHz): δ 150.74 (t, J = 2.1 Hz), 140.22, 138.76, 129.00, 128.65, 127.59, 127.17, 119.98, 116.09 (t, J = 260.9 Hz).

^{19}F NMR ($CDCl_3$, 376 MHz): δ –80.67 (d, J = 73.5 Hz).

HRMS (ESI-TOF) m/z: $[M + H]^+$ Calcd. for $C_{13}H_{11}F_2O$ 221.0778; found 221.0772 and $[M + Na]^+$ Calcd. for $C_{13}H_{11}F_2O$ 243.0597; found 243.0594.

Deoxyfluorination and Authentic fluoroarene standard

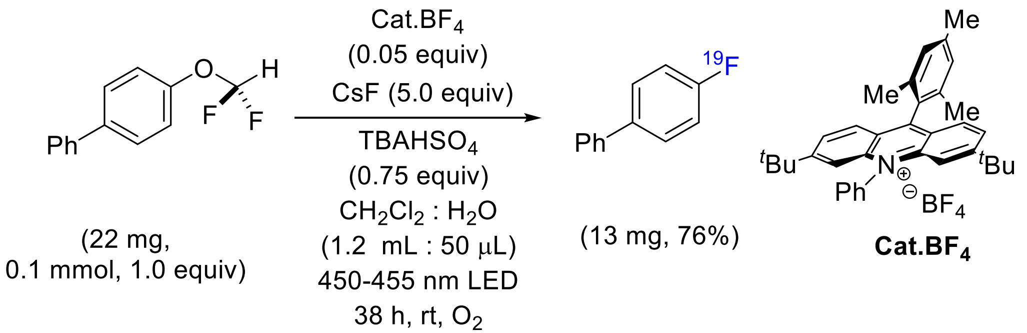

General conditions **I** were followed using 4-(difluoromethoxy)-1,1'-biphenyl **S1c** (22 mg, 0.1 mmol, 1.0 equiv), Mes-Acr-$Ph^+BF_4^-$ (0.05 equiv), CsF (5.0 equiv), $TBAHSO_4$ (0.75 equiv), CH_2Cl_2 : H_2O (0.1 M, 25:1), 450-455 nm LED, 48 h, 33 °C, O_2.

Purification: Isocratic column chromatography [SiO_2, EtOAc:Hexanes (0.2:9.8)] to obtain white solid of **[^{19}F]1** (13 mg, 76%).

Data are comparable to that commercially available compound. CAS number: 324-74-3

Radio-HPLC analysis and characterization

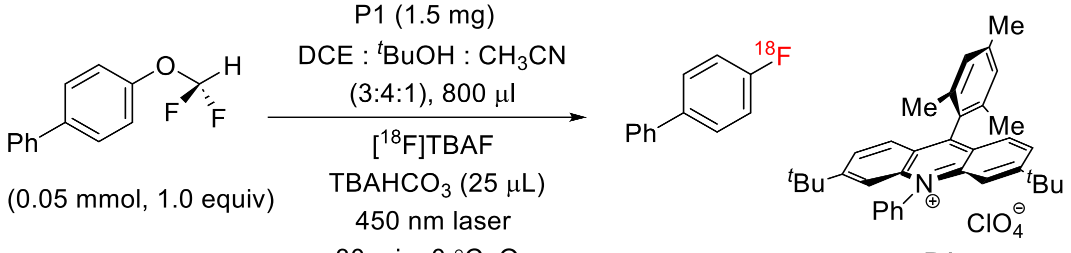

P1

Entry	Activity at starting	Activity at EOS	Alumina Filtration	Injected Dose	Collected Dose	Isolation Time	Decay Corrected	Filtration Yield	HPLC Purity of Filtrate	%Yield
1*	10.41 mCi	8.60 mCi	–	744 µCi	313 µCi	14.5 min	677.47 µCi	–	–	46.20%
2**	14.74 mCi	11.15 mCi	5.42 mCi	640 µCi	461 µCi	13.6 min	581.79 µCi	48.60%	79.24%	38.51%
										**Average %Yield: 42.4% (n=2)**

Table S6: HPLC isolated RCYs for **[^{18}F]1**

* HPLC conditions – method 1: Column: Phenomenex, Gemini® 5µm F5 100 Å, 250 × 4.6 mm LC Column

Solvent A: 0.1% TFA water, Solvent B: 0.1% TFA acetonitrile; Isocratic / Gradient elution: 55% Solvent B for 0 to 2 min, 55% – 95% Solvent B for 2 to 22.5 min. Flow rate: 1 mL/min.

** HPLC Conditions – method 2: Column: Phenomenex, Kinetex® 5µm F5 100 Å, 250 × 4.6 mm LC Column

Solvent A: 0.1% TFA water, Solvent B: 0.1% TFA acetonitrile; Isocratic / Gradient elution: 20% Solvent B for 0 to 2 min, 20% – 95% Solvent B for 2 to 22.5 min. Flow rate: 1 mL/min.

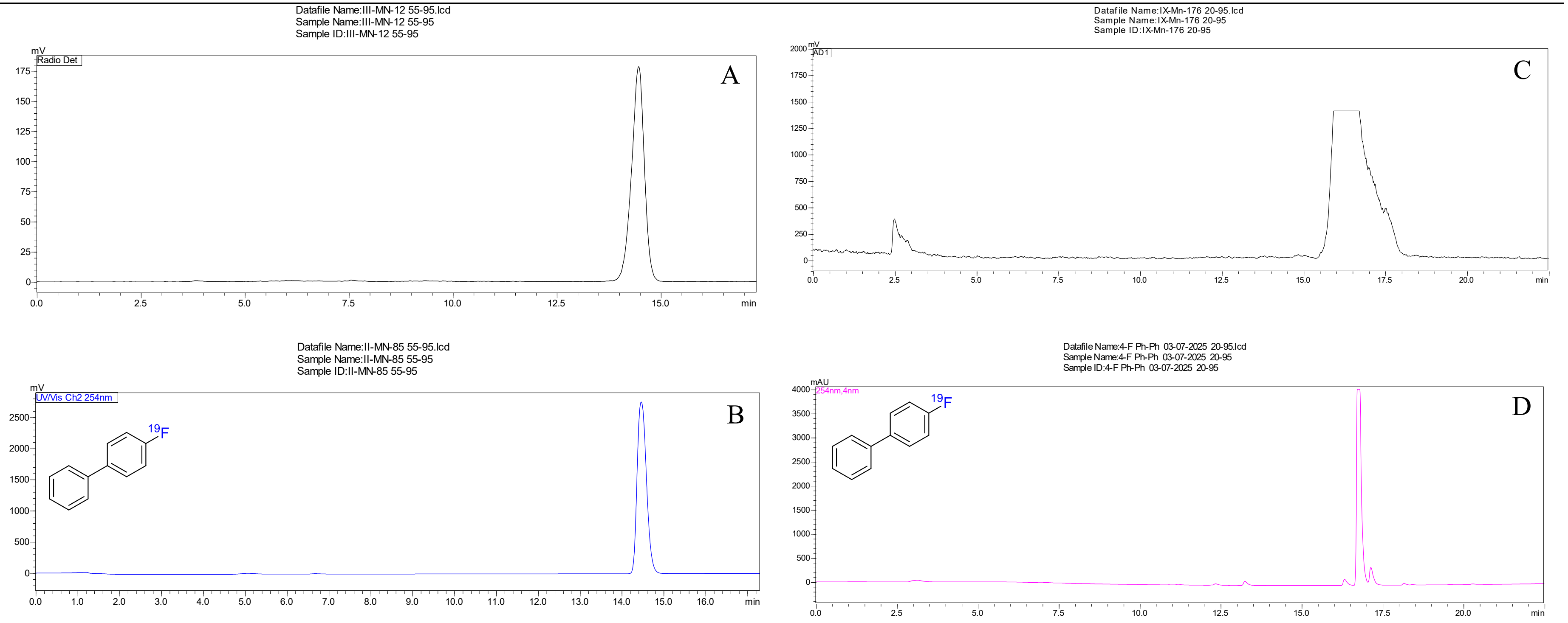

Figure S8: Radio-HPLC analysis of reaction mixture from **S1c**. Reaction mixture with HPLC method 1 (A), authentic **[^{19}F]1** with HPLC method 1 (B), reaction mixture with HPLC method 2 (C), and authentic **[^{19}F]1** with HPLC method 2 (D).

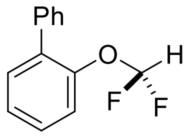

S2

Arene substrate:

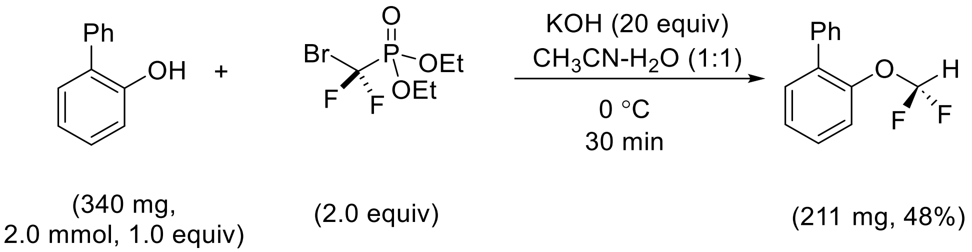

2-(Difluoromethoxy)-1,1'-biphenyl (S2)

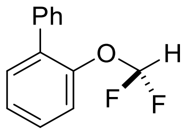

General conditions **A** were followed using [1,1'-biphenyl]-2-ol (340 mg, 2.0 mmol, 1.0 equiv), $BrCF_2P(O)(OC_2H_5)_2$ (1.068 g, 4.0 mmol, 2.0 equiv), KOH (2.24 g, 40 mmol, 20 equiv), CH_3CN: H_2O (5 mL : 5 mL) for 20 min.

Purification: Gradient column chromatography [SiO_2, EtOAc:Hexanes 00:100 to 2:98)] to obtain colourless liquid of **S2** (211 mg, 48%).

R_f : 0.8 (EtOAc : Hexanes 0.2:9.8)

^{1}H NMR ($CDCl_3$, 400 MHz): δ 7.51–7.48 (m, 2H), 7.45–7.41 (m, 3H), 7.39–7.34 (m, 2H), 7.29 (dt, J = 7.4, 1.4 Hz, 1H), 7.25–7.23 (m, 1H), 6.32 (t, J = 74.3 Hz, 1H).

^{13}C NMR ($CDCl_3$, 101 MHz): δ 148.29 (t, J = 2.9 Hz), 137.13, 134.26, 131.60, 129.54, 128.86, 128.41, 127.73, 126.02, 120.47, 116.28 (t, J = 261.1 Hz).

^{19}F NMR ($CDCl_3$, 376 MHz): δ –80.63 (d, J = 74.9 Hz).

HRMS (ESI-TOF) m/z: $[M + Na]^+$ Calcd. for $C_{13}H_{11}F_2O$ 243.0597; found 243.0593.

<u>Deoxyfluorination and Authentic fluoroarene standard</u>

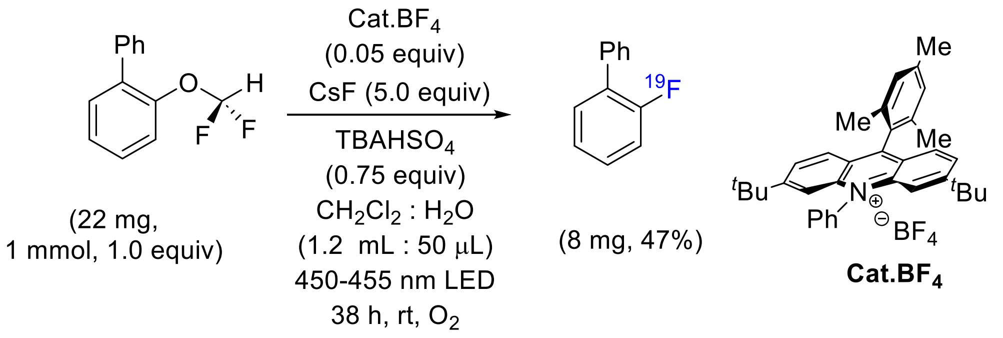

2-Fluoro-1,1'-biphenyl ([^{19}F]2)

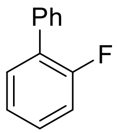

General conditions **I** were followed using 2-(difluoromethoxy)-1,1'-biphenyl **S2** (22 mg, 0.1mmol, 1.0 equiv), Mes-Acr-$Ph^+BF_4^-$ (0.05 equiv), CsF (5.0 equiv), $TBAHSO_4$ (0.75 equiv), CH_2Cl_2 : H_2O (0.1 M, 25:1), 450-455 nm LED, 38 h, 33 °C, O_2.

R_f: 0.8 (EtOAc : Hexanes 02:98)

^1H NMR ($CDCl_3$, 400 MHz): δ 7.57–7.54 (m, 2H), 7.47–7.42 (m, 3H), 7.39–7.29 (m, 2H) 7.21 (td, J = 7.5, 1.3 Hz, 1H), 7.34 (tt, J = 7.3, 1.2 Hz, 1H), 7.15 (ddd, J = 10.7, 8.7, 1.2 Hz, 1H).

^{13}C NMR ($CDCl_3$, 101 MHz): δ 161.14, 158.68, 135.96, 130.92 (d, J = 3.6 Hz), 129.18 (d, J = 2.9 Hz), 129.09 (d, J = 7.9 Hz), 128.57, 127.79, 124.47 (d, J = 3.57 Hz), 116.23 (d, J = 23.2 Hz).

^{19}F NMR ($CDCl_3$, 376 MHz): δ –118.07 (ddd, J = 11.0, 8.3, 5.5 Hz).

HRMS (ESI-TOF) m/z: $[M + H]^+$ Calcd. for $C_{12}H_{10}F$ 173.0767; found 173.0766.

Radio-HPLC analysis and characterization

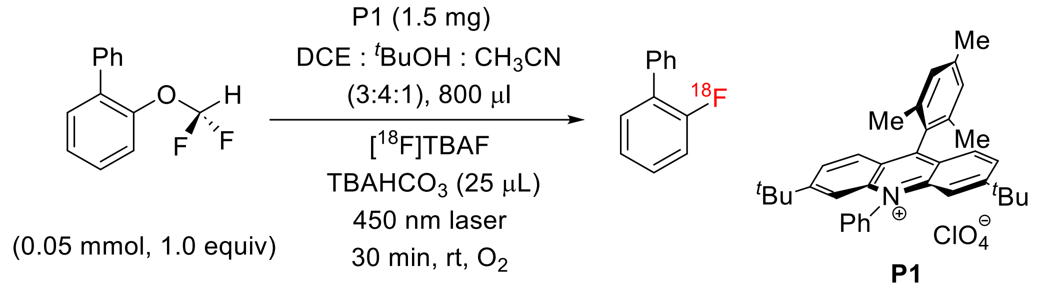

Entry	Activity at starting	Activity at EOS	Alumina Filtration	Injected Dose	Collected Dose	Isolation Time	Decay Corrected	Filtration Yield	HPLC Purity of Filtrate	%Yield
1*	11.70 mCi	8.71 mCi	5.14 mCi	175 μCi	121 μCi	16.5 min	158.09 μCi	59.01%	76.53%	45.16%
2**	9.90 mCi	8.31 mCi	–	490 μCi	166 μCi	18.4 min	435.51 μCi	–	–	38.11%
										**Average %Yield: 41.6% (n=2)**

Table S7: HPLC isolated RCYs for **[^{18}F]2**

* HPLC Conditions – method 1: Column: Phenomenex, Kinetex® 5μm F5 100 Å, 250 × 4.6 mm LC Column

Solvent A: 0.1% TFA water, Solvent B: 0.1% TFA acetonitrile; Isocratic / Gradient elution: 20% Solvent B for 0 to 2 min, 20% – 95% Solvent B for 2 to 22.5 min. Flow rate: 1 mL/min.

** HPLC conditions – method 2: Column: Phenomenex, Gemini® 5μm F5 100 Å, 250 × 4.6 mm LC Column

Solvent A: 0.1% TFA water, Solvent B: 0.1% TFA acetonitrile; Isocratic / Gradient elution: 40% Solvent B for 0 to 2 min, 40% – 95% Solvent B for 2 to 22.5 min. Flow rate: 1 mL/min.

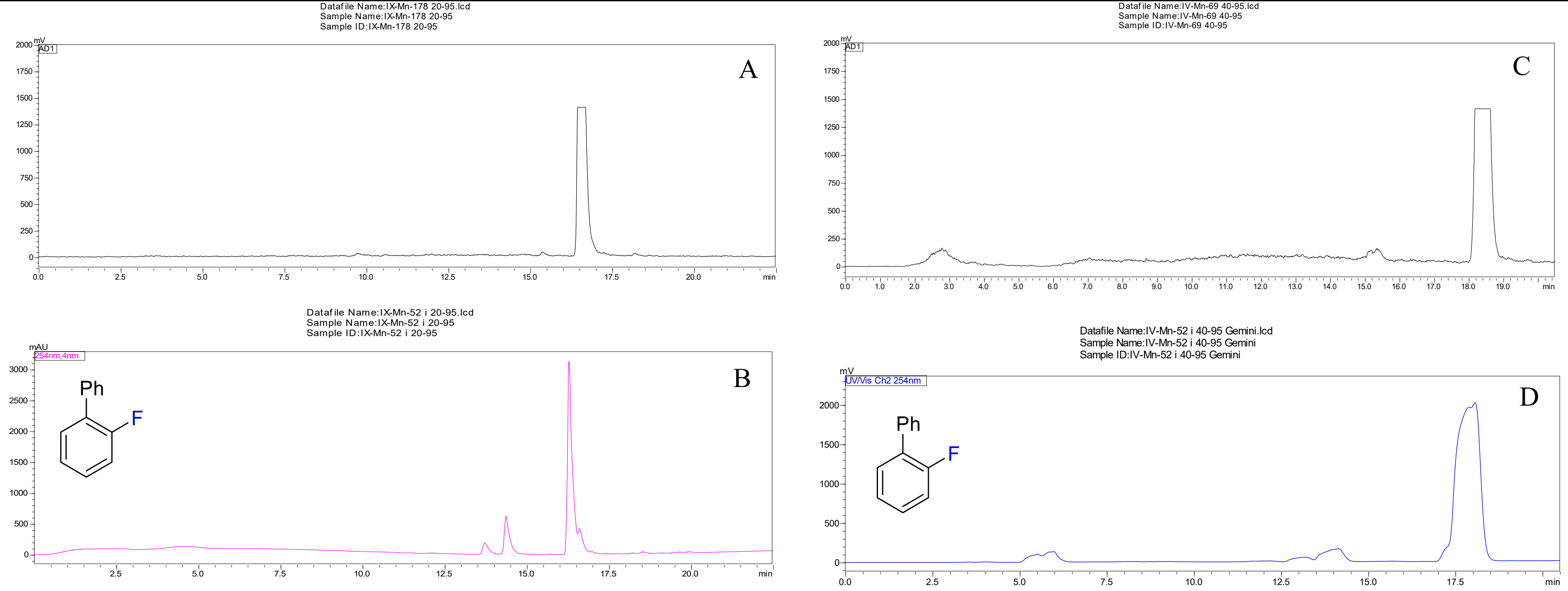

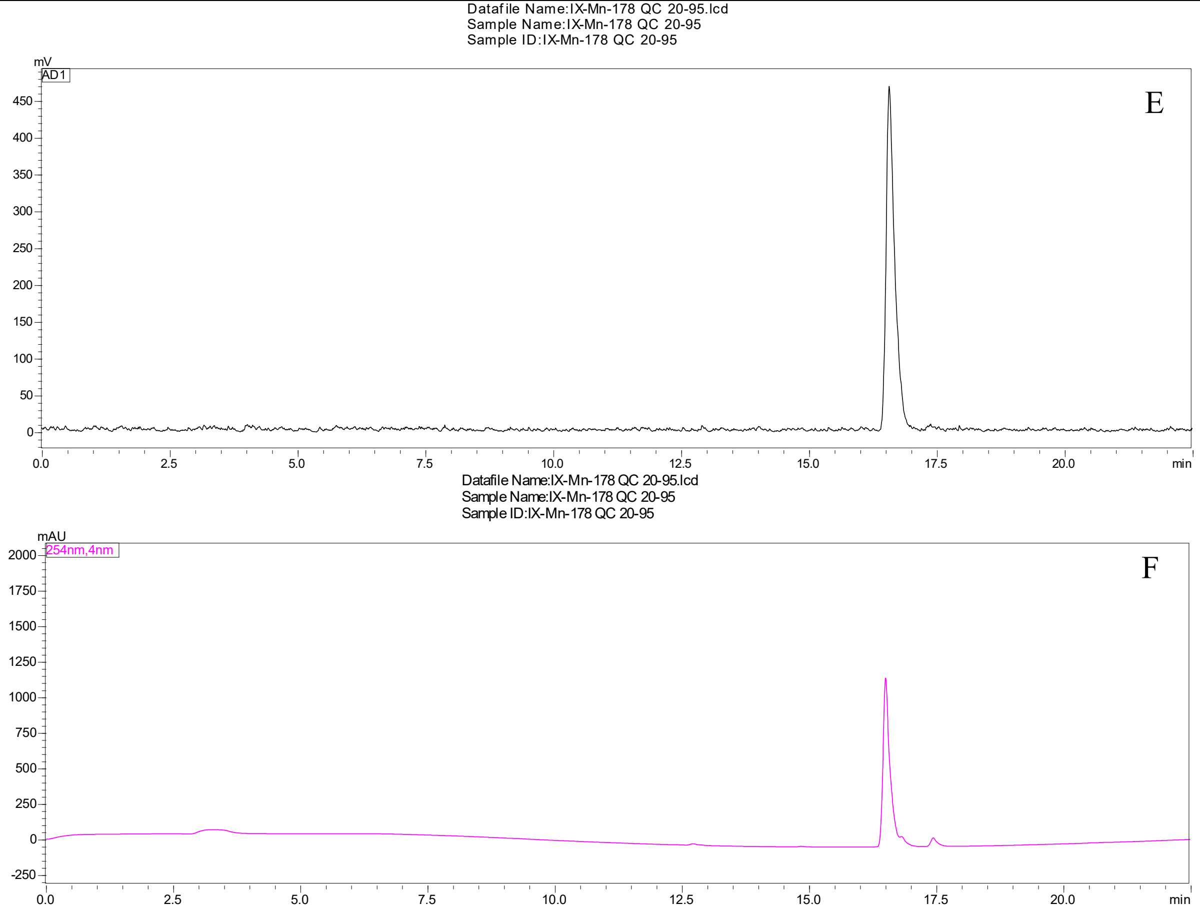

Figure S9: Radio-HPLC analysis of reaction mixture from **S2**. Reaction mixture with HPLC method 1 (A), authentic **[^{19}F]2** with HPLC method 1 (B), reaction mixture with HPLC method 2 (C), authentic **[^{19}F]2** with HPLC method 2 (D), and QC for **[^{18}F]2** (E and F).

HPLC Conditions for QC: Column: Phenomenex, Kinetex® 5µm F5 100 Å, 250 × 4.6 mm LC Column

Solvent A: 0.1% TFA water, Solvent B: 0.1% TFA acetonitrile; Isocratic / Gradient elution: 20% Solvent B for 0 to 2 min, 20% – 95% Solvent B for 2 to 22.5 min. Flow rate: 1 mL/min.

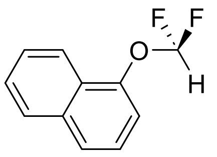

S3

Arene substrate:

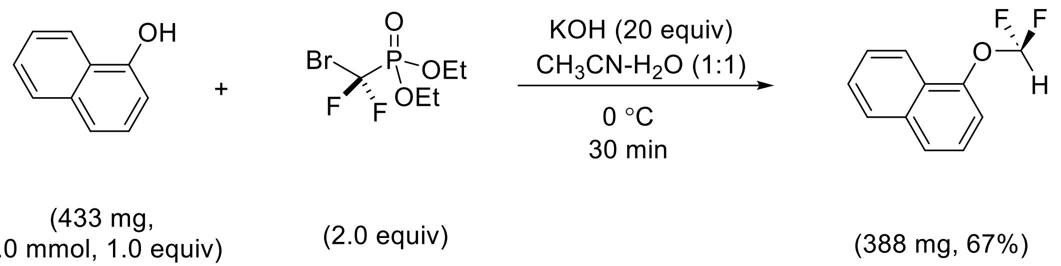

1-(Difluoromethoxy)naphthalene (S3)

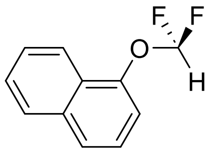

General conditions **A** were followed using naphthalen-1-ol (433 mg, 3.0 mmol, 1.0 equiv), $BrCF_2P(O)(OC_2H_5)_2$ (1.068 g, 4.0 mmol, 2.0 equiv), KOH (2.24 g, 40 mmol, 20 equiv), CH_3CN: H_2O (5 mL : 5 mL) for 30 min.

Purification: Gradient column chromatography [SiO_2, EtOAc:Hexanes 00:100 to 2:98)] to obtain colourless liquid of **S3** (388 mg, 67%).

R_f: 0.8 (EtOAc : Hexanes 0.2:9.8)

^{1}H NMR ($CDCl_3$, 400 MHz): δ 8.22–8.16 (m, 1H), 7.90–7.84 (m, 1H), 7.71 (d, J = 8.3 Hz, 1H), 7.56 (dddd, J = 12.6, 8.7, 6.8, 2.1 Hz, 2H), 7.42 (t, J = 7.8 Hz, 1H), 7.20 (d, J = 7.6 Hz, 1H), 6.67 (t, J= 74.2 Hz, 1H).

^{13}C NMR ($CDCl_3$, 101 MHz): δ 147.58 (t, J = 2.9 Hz), 134.84, 127.88, 127.08, 126.74, 126.60, 125.51, 125.46, 121.75, 116.71 (t, J = 258.6 Hz), 113.83.

^{19}F NMR ($CDCl_3$, 376 MHz): δ –79.86 (d, J = 73.5 Hz).

HRMS (ESI-TOF) m/z: $[M + H]^+$ Calcd. for $C_{11}H_9F_2O$ 195.0621; found 195.0618.

Deoxyfluorination and Authentic fluoroarene standard

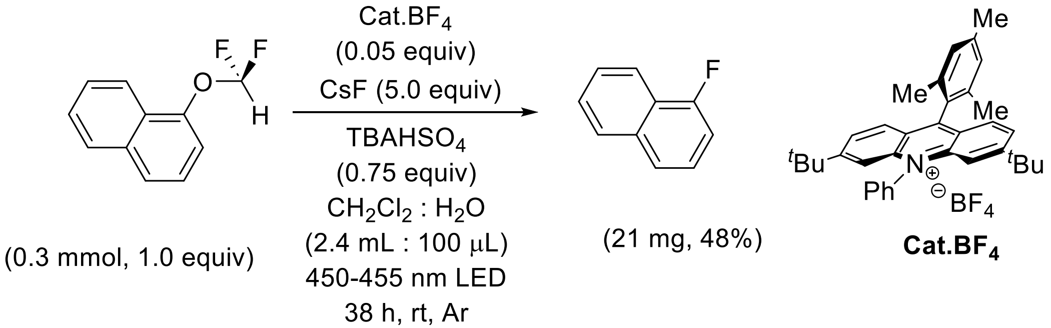

General conditions **I** were followed using 1-(difluoromethoxy)naphthalene **S3** (60 mg, 0.3 mmol, 1.0 equiv), Mes-Acr-$Ph^+BF_4^-$ (0.05 equiv), CsF (5.0 equiv), $TBAHSO_4$ (0.75 equiv), CH_2Cl_2 : H_2O (0.1 M, 25:1), 450-455 nm LED, 38 h, 33 °C, Ar balloon.

Purification: Gradient column chromatography [SiO_2, EtOAc:Hexanes 00:100 to 2:98)] to obtain colourless liquid of **[^{19}F]3** (21 mg, 48%). Data are comparable to that commercially available compound. CAS Number: 321-38-0

Radio-HPLC analysis and characterization

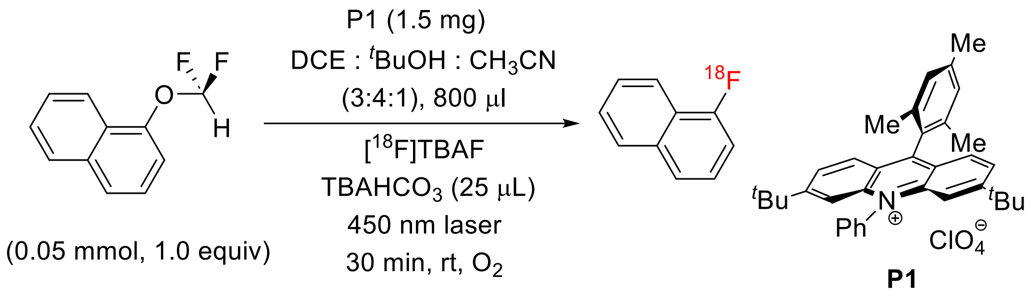

Entry	Activity at starting	Activity at EOS	Alumina Filtration	Injected Dose	Collected Dose	Isolation Time	Decay Corrected	Filtration Yield	HPLC Purity of Filtrate	%Yield
1[*]	9.76 mCi	7.16 mCi	4.74 mCi	439.7 µCi	99.2 µCi	16.0 min	397.44 µCi	66.20%	24.95%	16.51%
2[**]	9.47 mCi	7.44 mCi	4.19 mCi	223.0 µCi	56.0 µCi	12.6 min	206.59 µCi	56.31%	27.10%	15.26%
										**Average %Yield: 15.9% (n=2)**

Table S8: HPLC isolated RCYs for **[^{18}F]3**

[*] HPLC Conditions – method 1: Column: Phenomenex, Kinetex® 5µm F5 100 Å, 250 × 4.6 mm LC Column

Solvent A: 0.1% TFA water, Solvent B: 0.1% TFA acetonitrile; Isocratic / Gradient elution: 20% Solvent B for 0 to 2 min, 20% – 95% Solvent B for 2 to 22.5 min. Flow rate: 1 mL/min.

[*] HPLC Conditions – method 2: Column: Phenomenex, Kinetex® 5µm F5 100 Å, 250 × 4.6 mm LC Column

Solvent A: 0.1% TFA water, Solvent B: 0.1% TFA acetonitrile; Isocratic / Gradient elution: 40% Solvent B for 0 to 2 min, 40% – 70% Solvent B for 2 to 22.5 min. Flow rate: 1 mL/min.

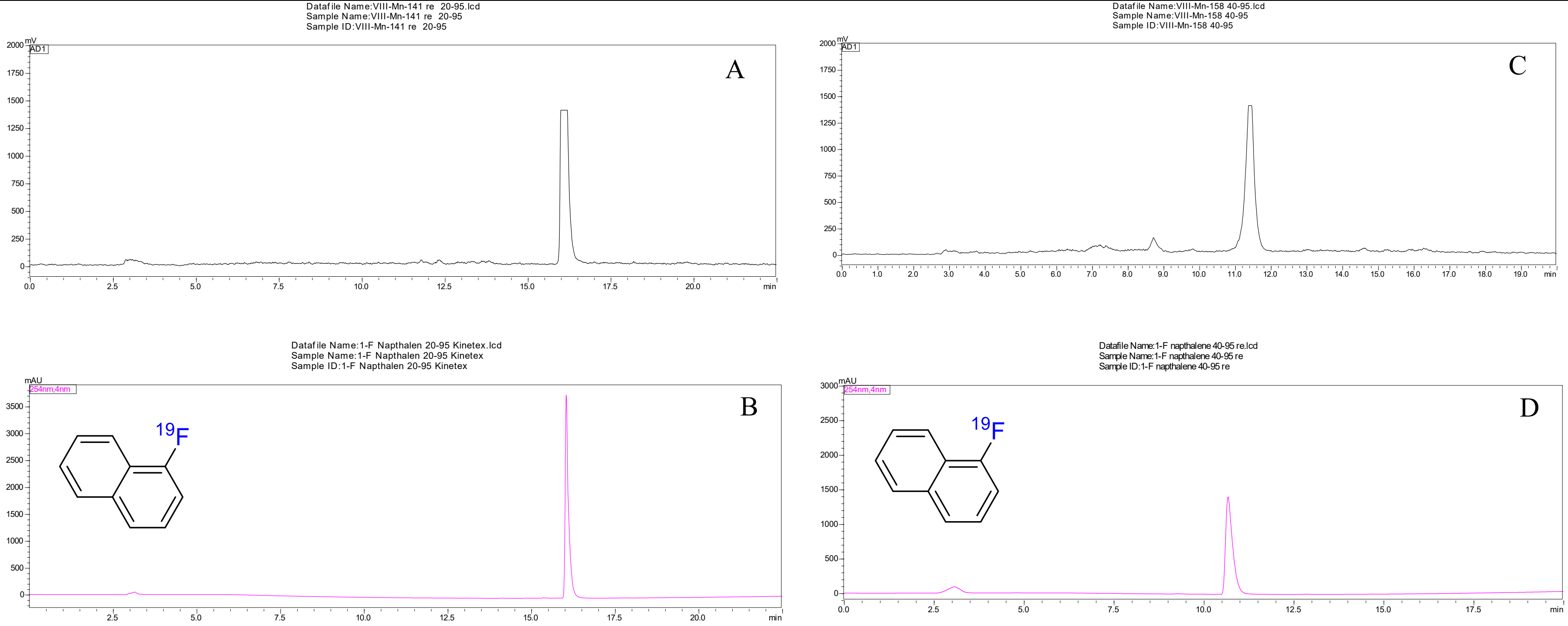

Figure S10: Radio-HPLC analysis of reaction mixture from **S3**. Reaction mixture with HPLC method 1 (A), authentic **[^{19}F]3** with HPLC method 1 (B), reaction mixture with HPLC method 2 (C), and authentic **[^{19}F]3** with HPLC method 2 (D).

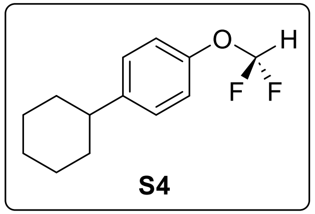

S4

Arene substrate:

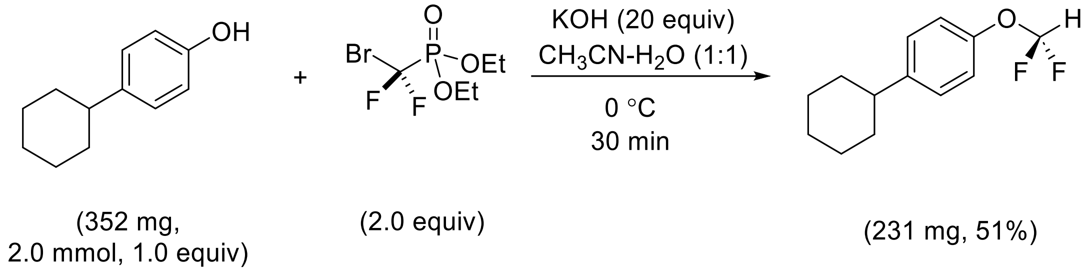

1-Cyclohexyl-4-(difluoromethoxy)benzene (S4)

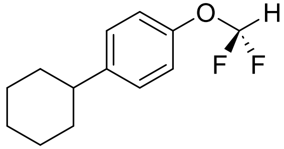

General conditions **A** were followed using 4-cyclohexylphenol (353 mg, 2.0 mmol, 1.0 equiv), $BrCF_2P(O)(OC_2H_5)_2$ (1.068 g, 4.0 mmol, 2.0 equiv), KOH (2.24 g, 40 mmol, 20 equiv), CH_3CN: H_2O (5 mL : 5 mL) for 30 min.

Purification: Gradient column chromatography [SiO_2, EtOAc:Hexanes 00:100 to 2:98)] to obtain colourless liquid of **S4** (231 mg, 51%).

R_f : 0.8 (EtOAc : Hexanes 0.2:9.8)

^{1}H NMR ($CDCl_3$, 400 MHz): δ 7.19 (dt, J = 8.5, 2.8 Hz, 2H), 7.03 (dt, J = 8.7, 2.6 Hz, 2H), 6.67 (t, J = 74.3 Hz, 1H), 2.53–2.45 (m, 1H), 1.86–1.83 (m, 4H), 1.77–1.73 (m, 1H), 4.15–1.31 (m, 4H), 1.29–1.88 (m, 1H).

^{13}C NMR ($CDCl_3$, 101 MHz): δ 149.34 (t, J = 2.9 Hz), 149.49, 128.15, 119.55, 116.28 (t, J = 260.4 Hz), 44.02, 34.64, 26.95, 26.19.

^{19}F NMR ($CDCl_3$, 376 MHz): δ –80.34 (d, J = 74.9 Hz).

HRMS (ESI-TOF) m/z: $[M + H]^+$ Calcd. for $C_{13}H_{17}F_2O$ 227.1247; found 227.1251 and $[M + Na]^+$ Calcd. for $C_{13}H_{16}F_2NaO$ 249.1067; found 249.1062.

Deoxyfluorination and Authentic fluoroarene standard

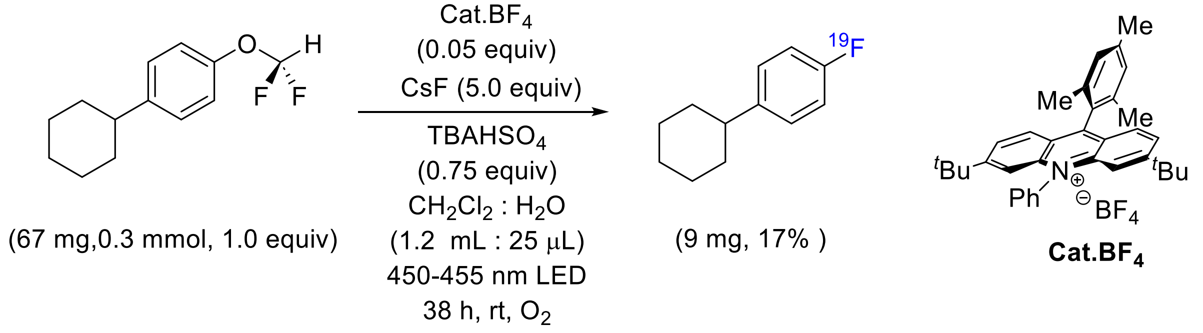

General conditions **I** were followed using 1-cyclohexyl-4-(difluoromethoxy)benzene (67 mg, 0.3 mmol, 1.0 equiv), Mes-Acr-$Ph^+BF_4^-$ (0.05 equiv), CsF (5.0 equiv), $TBAHSO_4$ (0.75 equiv), CH_2Cl_2 : H_2O (0.1 M, 25:1), 450-455 nm LED, 48 h, 33 °C, O_2 balloon.

Purification: Isocratic column chromatography [SiO_2, EtOAc:Hexanes 00:100)] to obtain colourless liquid of **[^{19}F]4** (9 mg, 17%).

Note: The compound **[^{19}F]4** is volatile under reduced pressure.

^{19}F NMR ($CDCl_3$, 376 MHz): δ –118.07 (ddd, J = 15.3, 9.7, 5.5 Hz).

Data are comparable to that reported in literature.[4,5]

Radio-HPLC analysis and characterization

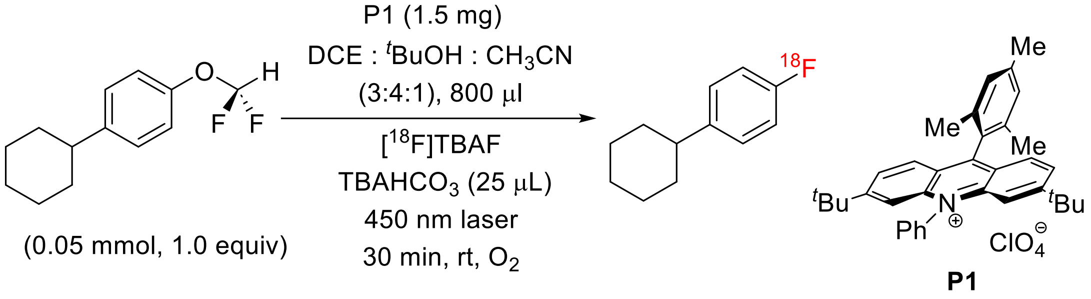

Entry	Activity at starting	Activity at EOS	Alumina Filtration	Injected Dose	Collected Dose	Isolation Time	Decay Corrected	Filtration Yield	HPLC Purity of Filtrate	%Yield
1*	9.27 mCi	7.44 mCi	-	631 µCi	141 µCi	14.0 min	577.61 µCi	-	-	24.41%
2**	10.54 mCi	8.44 mCi	3.76 mCi	492 µCi	205 µCi	18.5 min	438.90 µCi	44.54%	46.70%	20.80%
										**Average %Yield: 22.6% (n=2)**

Table S9: HPLC isolated RCYs for **[^{18}F]4**

* HPLC conditions – method 1: Column: Phenomenex, Gemini® 5µm F5 100 Å, 250 × 4.6 mm LC Column

Solvent A: 0.1% TFA water, Solvent B: 0.1% TFA acetonitrile; Isocratic / Gradient elution: 55% Solvent B for 0 to 2 min, 55% – 95% Solvent B for 2 to 22.5 min. Flow rate: 1 mL/min.

** HPLC Conditions – method 2: Column: Phenomenex, Kinetex® 5µm F5 100 Å, 250 × 4.6 mm LC Column

Solvent A: 0.1% TFA water, Solvent B: 0.1% TFA acetonitrile; Isocratic / Gradient elution: 20% Solvent B for 0 to 2 min, 20% – 95% Solvent B for 2 to 22.5 min. Flow rate: 1 mL/min.

Datafile Name:V-Mn-01 55-95.lcd
Sample Name:V-Mn-01 55-95
Sample ID:V-Mn-01 55-95

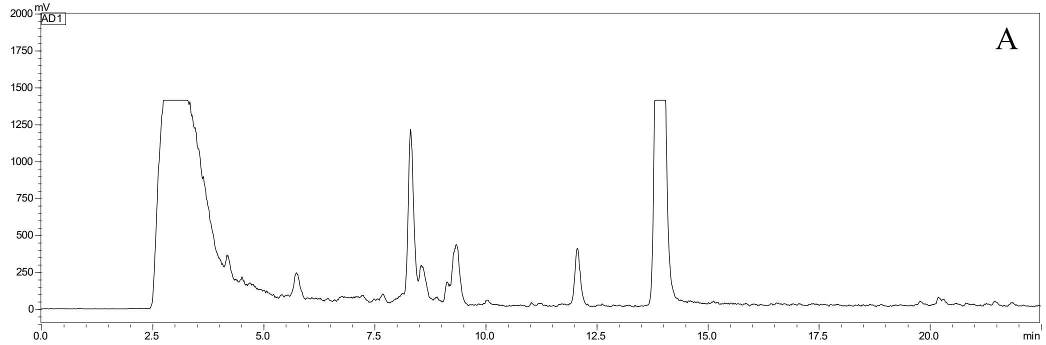

Datafile Name:IX-Mn-72 20-95.lcd
Sample Name:IX-Mn-72 20-95
Sample ID:IX-Mn-72 20-95

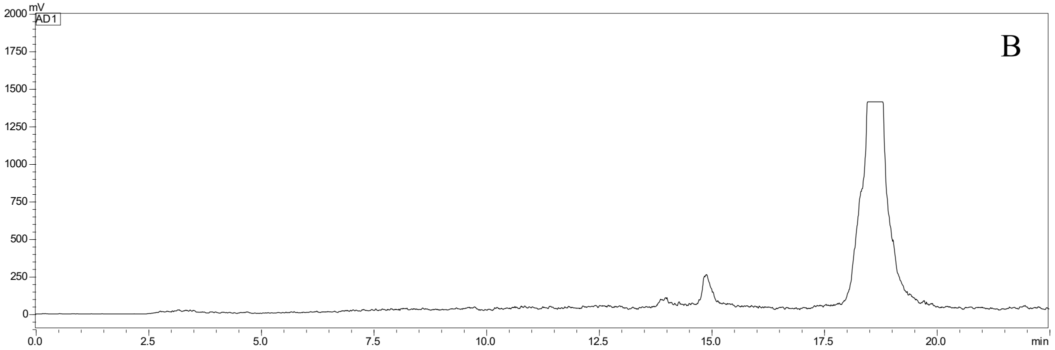

Datafile Name:IX-Mn-66 i ref 20-95.lcd
Sample Name:IX-Mn-66 i ref 20-95
Sample ID:IX-Mn-66 i ref 20-95

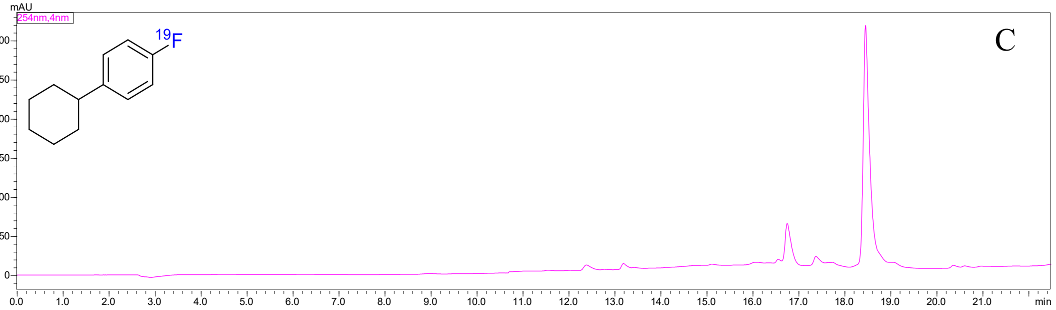

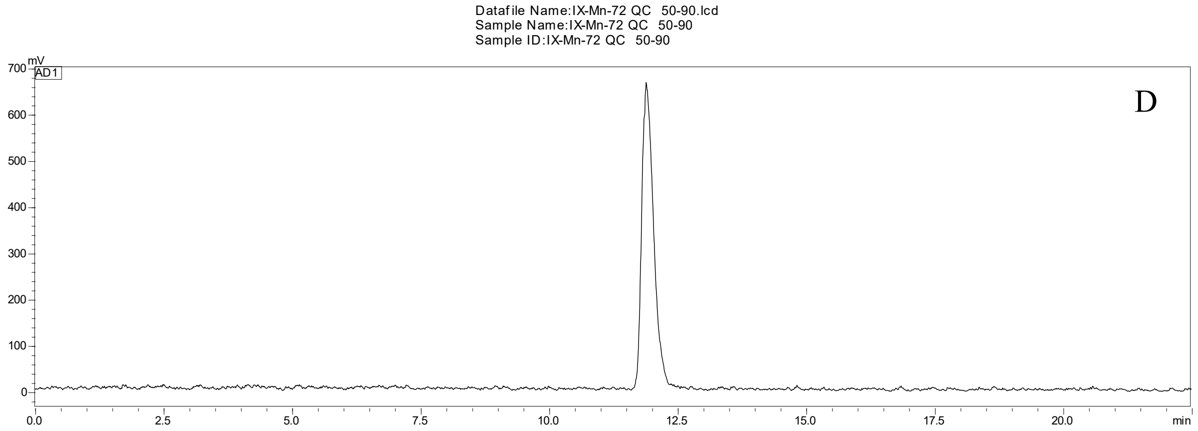

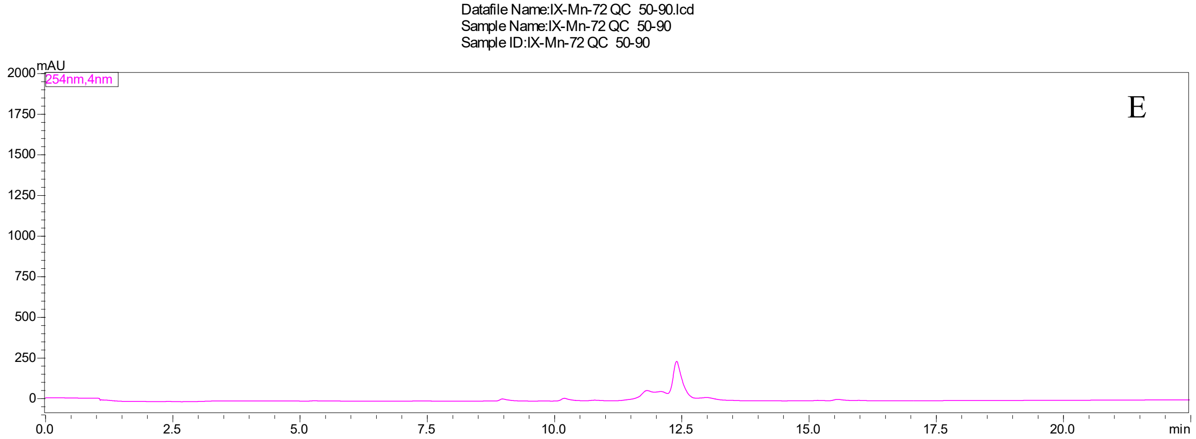

Figure S11: Radio-HPLC analysis of reaction mixture from **S4**. Reaction mixture with HPLC method 1 (A), reaction mixture with HPLC method 2 (B), and authentic **[^{19}F]4** with HPLC method 2 (C), and QC for **[^{18}F]4** (D and E).

HPLC Conditions for QC: Column: Phenomenex, Kinetex® 5µm F5 100 Å, 250 × 4.6 mm LC Column

Solvent A: 0.1% TFA water, Solvent B: 0.1% TFA acetonitrile; Isocratic / Gradient elution: 50% Solvent B for 0 to 2 min, 50% – 90% Solvent B for 2 to 22.5 min. Flow rate: 1 mL/min.

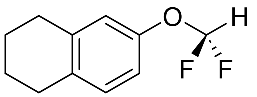

S5

Arene substrate:

6-(Difluoromethoxy)-1,2,3,4-tetrahydronaphthalene (S5)

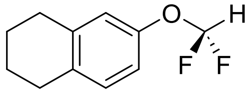

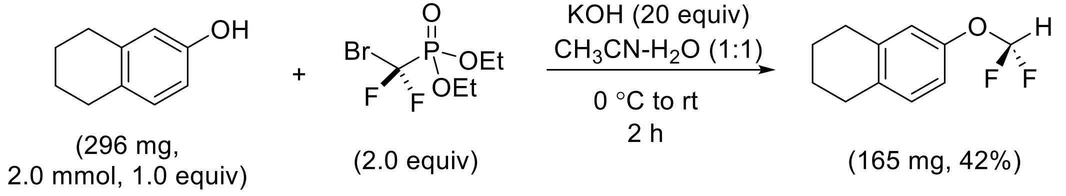

General conditions **A** were followed using 5,6,7,8-tetrahydronaphthalen-2-ol (296 mg, 2.0 mmol, 1.0 equiv), $BrCF_2P(O)(OC_2H_5)_2$ (1.068 g, 4.0 mmol, 2.0 equiv), KOH (2.24 g, 40 mmol, 20 equiv), CH_3CN: H_2O (5 mL : 5 mL) for 2 h.

Purification: Gradient column chromatography [SiO_2, EtOAc:Hexanes 00:100 to 2:98)] to obtain colourless liquid of **S5** (165 mg, 42%).

R_f : 0.8 (EtOAc : Hexanes 0.2:9.8)

^{1}H NMR ($CDCl_3$, 400 MHz): δ 7.04 (d, J = 8.2 Hz, 1H), 6.85 (dd, J = 10.8, 8.2 Hz, 2H), 6.55 (t, J= 74.5 Hz, 1H), 2.75–2.72 (m, 4H), 1.80–1.78 (m, 4H).

^{13}C NMR ($CDCl_3$, 101 MHz): δ 149.06 (t, J = 2.9 Hz), 139.00, 134.51, 130.40, 119.97, 117.01, 116.37 (t, J = 258.6 Hz), 29.62, 28.89, 23.10 (d, J = 23.6 Hz).

^{19}F NMR ($CDCl_3$, 376 MHz): δ –80.17 (d, J = 74.9 Hz).

HRMS (ESI-TOF) m/z: $[M + Na]^+$ Calcd. for $C_{11}H_{12}F_2NaO$ 221.0754; found 221.0750.

Authentic fluoroarene standard

Data are comparable to that reported in literature.[6]

Radio-HPLC analysis and characterization

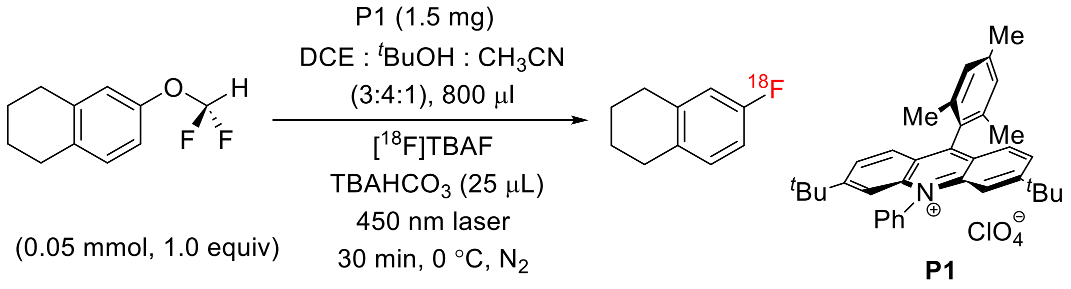

Entry	Activity at starting	Activity at EOS	Alumina Filtration	Injected Dose	Collected Dose	Isolation Time	Decay Corrected	Filtration Yield	HPLC Purity of Filtrate	%Yield
1	17.00 mCi	12.07 mCi	6.52 mCi	598 µCi	55 µCi	16.8 min	540.07 µCi	54.10%	10.18%	5.50%
2	16.16 mCi	11.20 mCi	2.98 mCi	588 µCi	69 µCi	16.8 min	531.04 µCi	26.60%	12.99%	3.45%
								**Average %Yield: 4.5% (n=2)**		

Table S10: HPLC isolated RCYs for **[^{18}F]5**

HPLC Conditions – method 1: Column: Phenomenex, Kinetex® 5µm F5 100 Å, 250 × 4.6 mm LC Column

Solvent A: 0.1% TFA water, Solvent B: 0.1% TFA acetonitrile; Isocratic / Gradient elution: 20% Solvent B for 0 to 2 min, 20% – 95% Solvent B for 2 to 22.5 min. Flow rate: 1 mL/min.

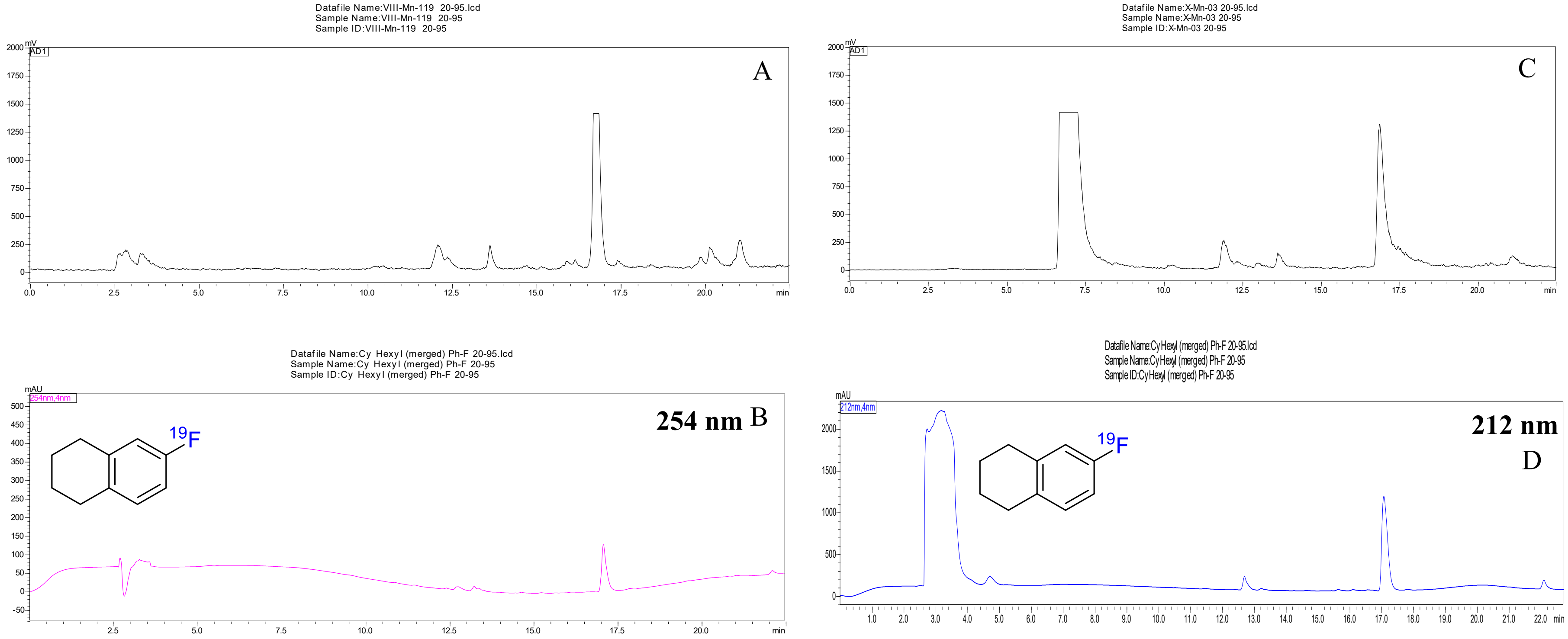

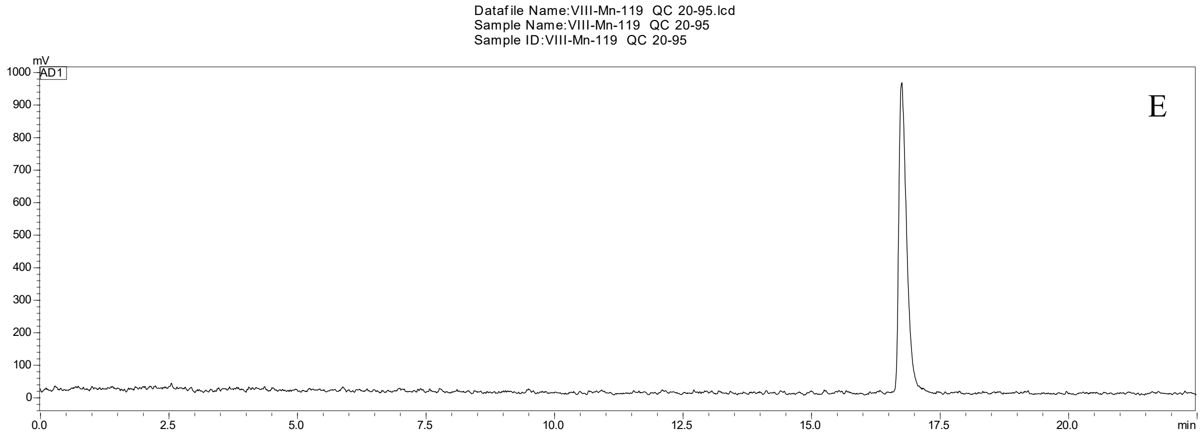

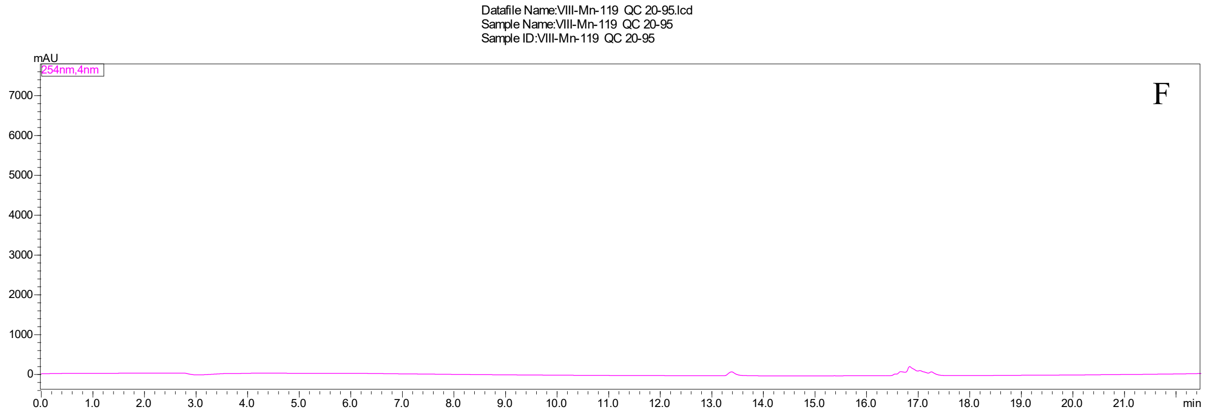

Figure S12: Radio-HPLC analysis of reaction mixture from **S5**. Reaction mixture with HPLC method 1 (A), authentic **[^{19}F]5** with HPLC method 1 (B), reaction mixture with HPLC method 1 (C), authentic **[^{19}F]5** with HPLC method 1 (D), and QC for **[^{18}F]2** (E and F).

HPLC Conditions for QC: Column: Phenomenex, Kinetex® 5µm F5 100 Å, 250 × 4.6 mm LC Column

Solvent A: 0.1% TFA water, Solvent B: 0.1% TFA acetonitrile; Isocratic / Gradient elution: 20% Solvent B for 0 to 2 min, 20% – 90% Solvent B for 2 to 22.5 min. Flow rate: 1 mL/min.

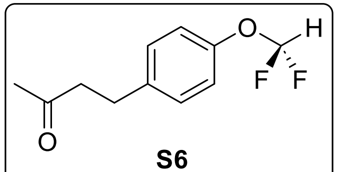

S6

Arene substrate:

4-(4-(Difluoromethoxy)phenyl)butan-2-one (**S6)**

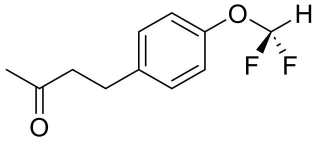

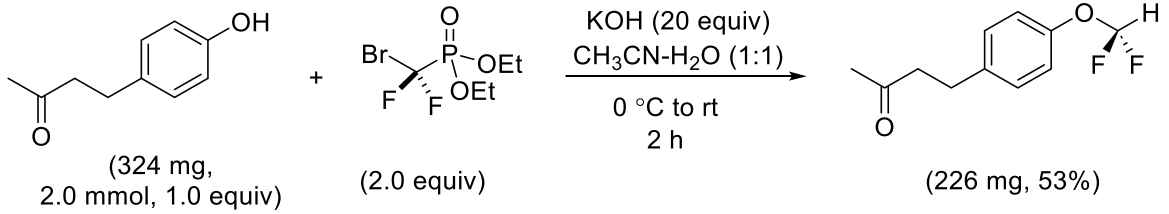

General conditions **A** were followed using 4-(4-hydroxyphenyl)butan-2-one (324 mg, 2.0 mmol, 1.0 equiv), $BrCF_2P(O)(OC_2H_5)_2$ (1.068 g, 4.0 mmol, 2.0 equiv), KOH (2.24 g, 40 mmol, 20 equiv), CH_3CN: H_2O (5 mL : 5 mL) for 2 h.

Purification: Gradient column chromatography [SiO_2, EtOAc:Hexanes 00:100 to 30:70)] to obtain colourless liquid of **S6** (226 mg, 53%).

R_f : 0.4 (EtOAc : Hexanes 3:7)

^{1}H NMR ($CDCl_3$, 400 MHz): δ 7.17 (dt, J = 8.7, 3.1 Hz, 2H), 7.03 (d, J = 8.6, 3.5 Hz, 2H), 6.47 (t, J= 74.2 Hz, 1H), 2.89-2.86 (m, 2H), 2.77-2.73 (m, 2H), 2.14 (s, 3H).

^{13}C NMR (CDCl$_3$, 101 MHz): δ 207.57, 149.49 (t, J = 2.7 Hz), 138.31, 129.65, 119.72, 116.01 (t, J = 259.4 Hz), 45.02, 30.09, 28.89.

^{19}F NMR (CDCl$_3$, 376 MHz): δ –80.58 (d, J = 74.9 Hz).

HRMS (ESI-TOF) m/z: [M + Na]$^+$ Calcd. for $C_{11}H_{12}F_2NaO_2$ 237.0703; found 237.0701.

Authentic fluoroarene standard

^{19}F-Reference compound – **[^{19}F]6** was purchased from the commercial supplier. CAS Number 63416-61-5

Radio-HPLC analysis and characterization

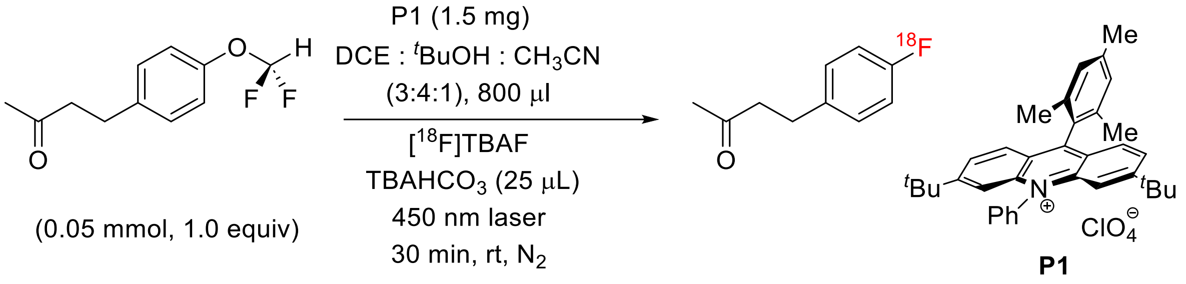

Entry	Activity at starting	Activity at EOS	Alumina Filtration	Injected Dose	Collected Dose	Isolation Time	Decay Corrected	Filtration Yield	HPLC Purity of Filtrate	%Yield
1	16.69 mCi	12.42 mCi	4.44 mCi	681 μCi	333 μCi	12.6 min	630.90 μCi	35.75%	52.78%	18.86%
2	17.53 mCi	13.87 mCi	4.76 mCi	367 μCi	164 μCi	12.6 min	340.00 μCi	34.32%	48.23%	16.55%
										**Average %Yield: 17.7% (n=2)**

Table S11: HPLC isolated RCYs for **[^{18}F]6**

HPLC Conditions – method 1 : Column: Phenomenex, Kinetex® 5μm F5 100 Å, 250 × 4.6 mm LC Column

Solvent A: 0.1% TFA water, Solvent B: 0.1% TFA acetonitrile; Isocratic / Gradient elution: 20% Solvent B for 0 to 2 min, 20% – 95% Solvent B for 2 to 22.5 min. Flow rate: 1 mL/min.

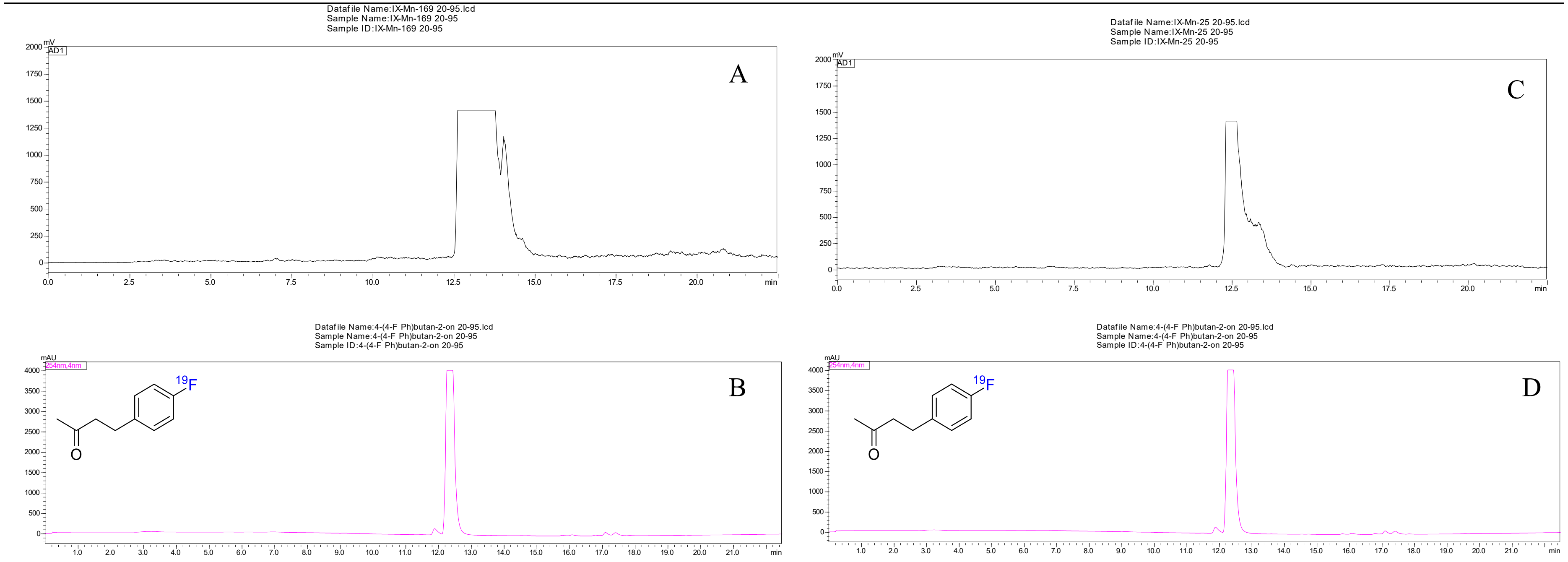

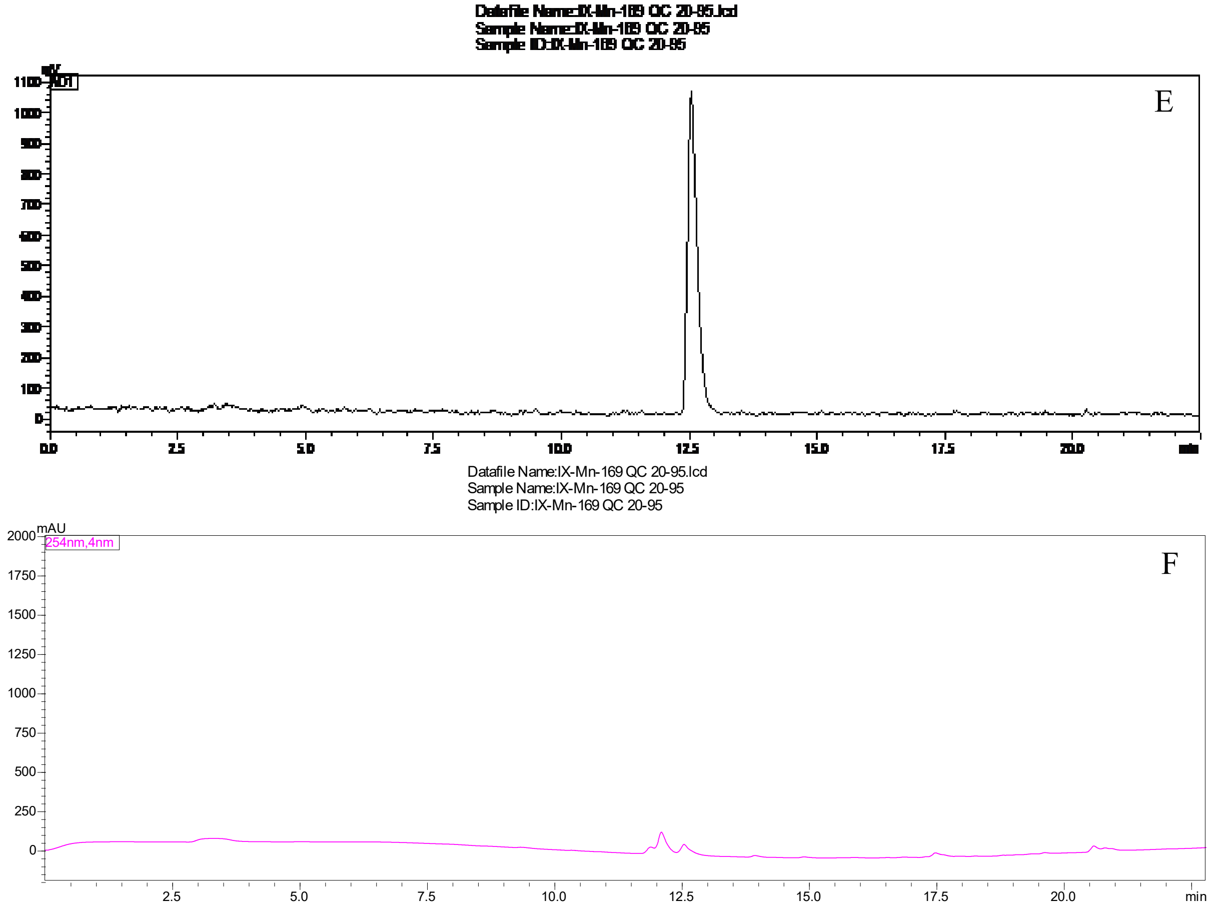

Figure S13: Radio-HPLC analysis of reaction mixture from **S6**. Reaction mixture with HPLC method 1 (A), authentic **[^{19}F]6** with HPLC method 1 (B), reaction mixture with HPLC method 1 (C), authentic **[^{19}F]6** with HPLC method 1 (D), and QC for **[^{18}F]6** (E and F).

HPLC Conditions for QC: Column: Phenomenex, Kinetex® 5µm F5 100 Å, 250 × 4.6 mm LC Column

Solvent A: 0.1% TFA water, Solvent B: 0.1% TFA acetonitrile; Isocratic / Gradient elution: 20% Solvent B for 0 to 2 min, 20% – 90% Solvent B for 2 to 22.5 min. Flow rate: 1 mL/min.

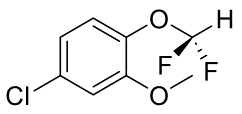

S7

Arene substrate:

4-Chloro-1-(difluoromethoxy)-2-methoxybenzene (S7)

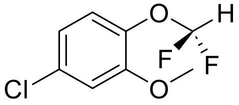

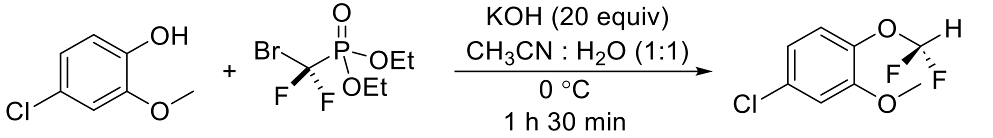

(317 mg, 2.0 mmol, 1.0 equiv) (2.0 equiv) (293 mg, 58%)

General conditions **A** were followed using 4-chloro-2-methoxyphenol (317 mg, 2.0 mmol, 1.0 equiv), $BrCF_2P(O)(OC_2H_5)_2$ (1.068 g, 4.0 mmol, 2.0 equiv), KOH (2.24 g, 40 mmol, 20 equiv), CH_3CN: H_2O (5 mL : 5 mL) for 1 h 30 min.

Purification: Gradient column chromatography [SiO_2, EtOAc:Hexanes 00:100 to 10:90)] to obtain colourless liquid of **S7** (293 mg, 58%).

R_f : 0.7 (EtOAc : Hexanes 1:9)

^{1}H NMR ($CDCl_3$, 400 MHz): δ 7.09 (d, J = 8.5 Hz, 1H), 6.95 (d, J = 2.3 Hz, 1H), 6.91 (dd, J = 8.5, 2.4 Hz, 1H), 6.50 (t, J = 74.5 Hz, 1H), 3.87 (s, 3H).

^{13}C NMR ($CDCl_3$, 101 MHz): δ 151.78, 139.01 (t, J = 3.6 Hz), 131.99, 123.49, 120.94, 118.66 (t, J = 260.4 Hz), 113.46, 56.34.

^{19}F NMR ($CDCl_3$, 376 MHz): δ –81.73 (d, J = 75.8 Hz).

HRMS (APCI-TOF) m/z: $[M]^{+\cdot}$ Calcd. for $C_8H_7{}^{35}ClF_2O_2$ 208.0097; found 208.0081 and $[M]^{+\cdot}$ Calcd. for $C_8H_7{}^{37}ClF_2O_2$ 210.0068; found 210.0051.

<u>Deoxyfluorination and Authentic fluoroarene standard</u>

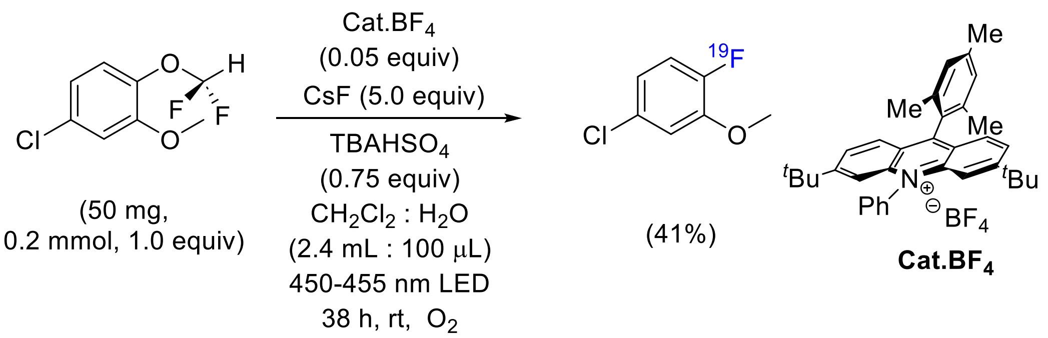

General conditions **I** were followed using 4-chloro-1-(difluoromethoxy)-2-methoxybenzene **S7** (50 mg, 0.2 mmol, 1.0 equiv), Mes-Acr-$Ph^+BF_4^-$ (0.05 equiv), CsF (5.0 equiv), $TBAHSO_4$ (0.75 equiv), CH_2Cl_2 : H_2O (0.1 M, 25:1), 450-455 nm LED, 38 h, 33 °C, O_2 balloon. The compound **[^{19}F]7** in 41% (relative to staring material) as determined by ^{19}F NMR spectroscopic analysis of the crude reaction mixture

^{19}F NMR ($CDCl_3$, 376 MHz): δ –137.58 (ddd, J = 9.7, 6.9, 4.2 Hz).

Data are comparable to that commercially available compound. CAS Number 1092349-89-7

Radio-HPLC analysis and characterization

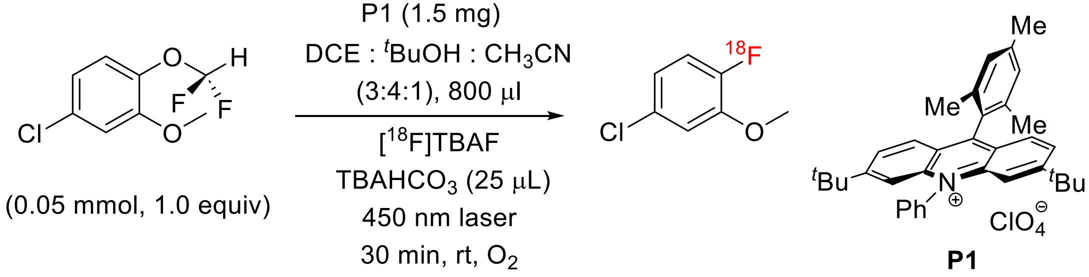

Entry	Activity at starting	Activity at EOS	Alumina Filtration	Injected Dose	Collected Dose	Isolation Time	Decay Corrected	Filtration Yield	HPLC Purity of Filtrate	%Yield
1	10.76 mCi	8.36 mCi	3.68 mCi	399 μCi	217 μCi	14.6 min	362.94 μCi	43.06%	59.78%	25.74%
2	10.64 mCi	4.23 mCi	1.63 mCi	193 μCi	123 μCi	14.6 min	176.57 μCi	38.43%	69.66%	26.77%
										**Average %Yield: 26.2% (n=2)**

Table S12: HPLC isolated RCYs for **[^{18}F]7**

HPLC Conditions – method 1: Column: Phenomenex, Kinetex® 5µm F5 100 Å, 250 ✕ 4.6 mm LC Column

Solvent A: 0.1% TFA water, Solvent B: 0.1% TFA acetonitrile; Isocratic / Gradient elution: 20% Solvent B for 0 to 2 min, 20% – 95% Solvent B for 2 to 22.5 min. Flow rate: 1 mL/min.

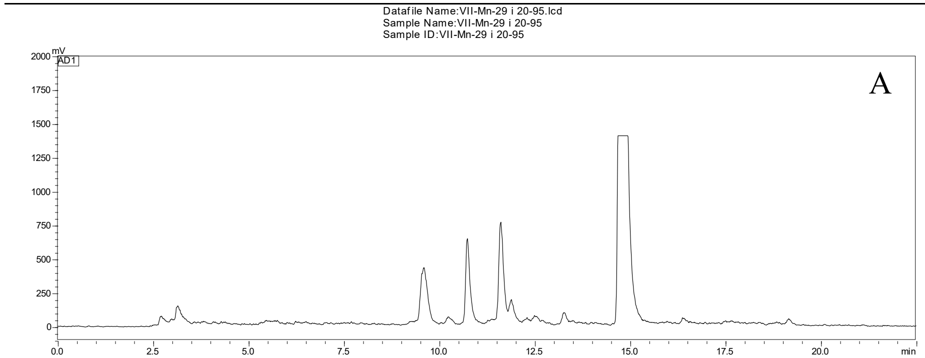
Datafile Name:VII-Mn-29 i 20-95.lcd
Sample Name:VII-Mn-29 i 20-95
Sample ID:VII-Mn-29 i 20-95
mV
AD1
A
min

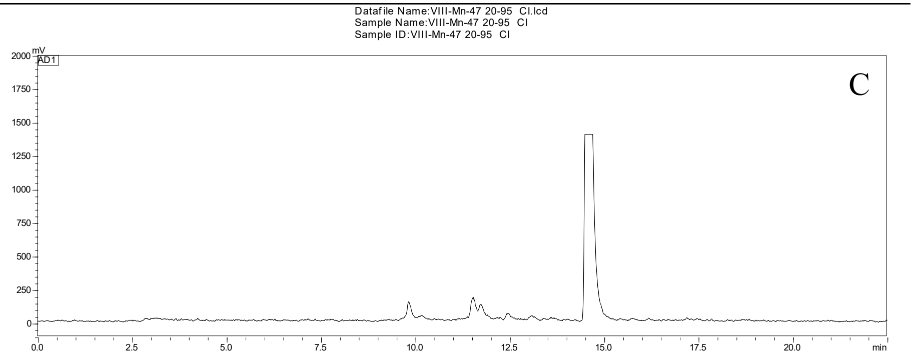
Datafile Name:VIII-Mn-47 20-95 Cl.lcd
Sample Name:VIII-Mn-47 20-95 Cl
Sample ID:VIII-Mn-47 20-95 Cl
mV
AD1
C
min

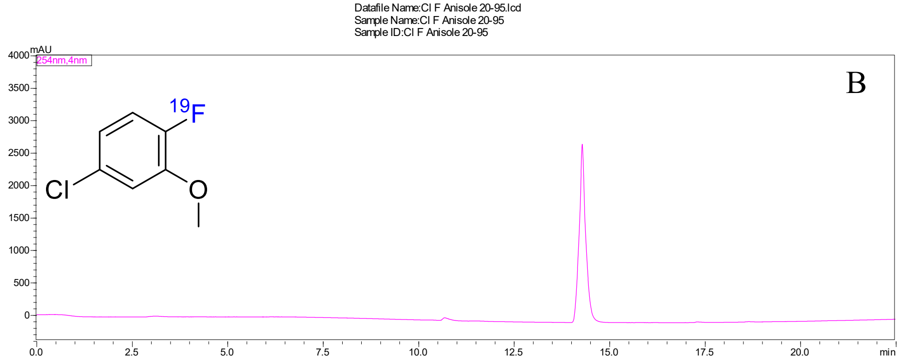
Datafile Name:Cl F Anisole 20-95.lcd
Sample Name:Cl F Anisole 20-95
Sample ID:Cl F Anisole 20-95
mAU
254nm,4nm
B
19F
Cl
O
min

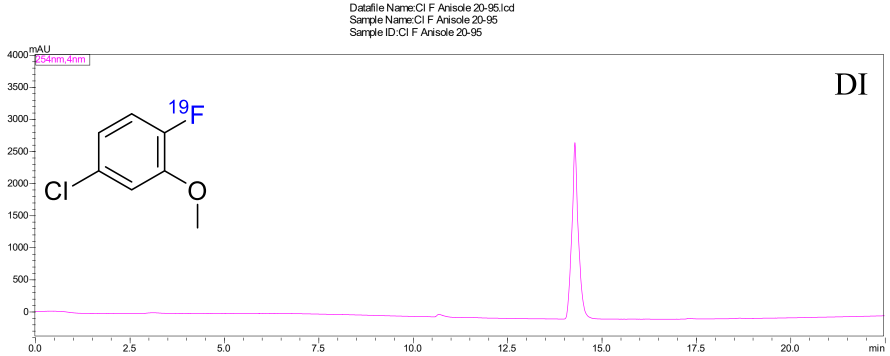
Datafile Name:Cl F Anisole 20-95.lcd
Sample Name:Cl F Anisole 20-95
Sample ID:Cl F Anisole 20-95
mAU
254nm,4nm
DI
19F
Cl
O
min

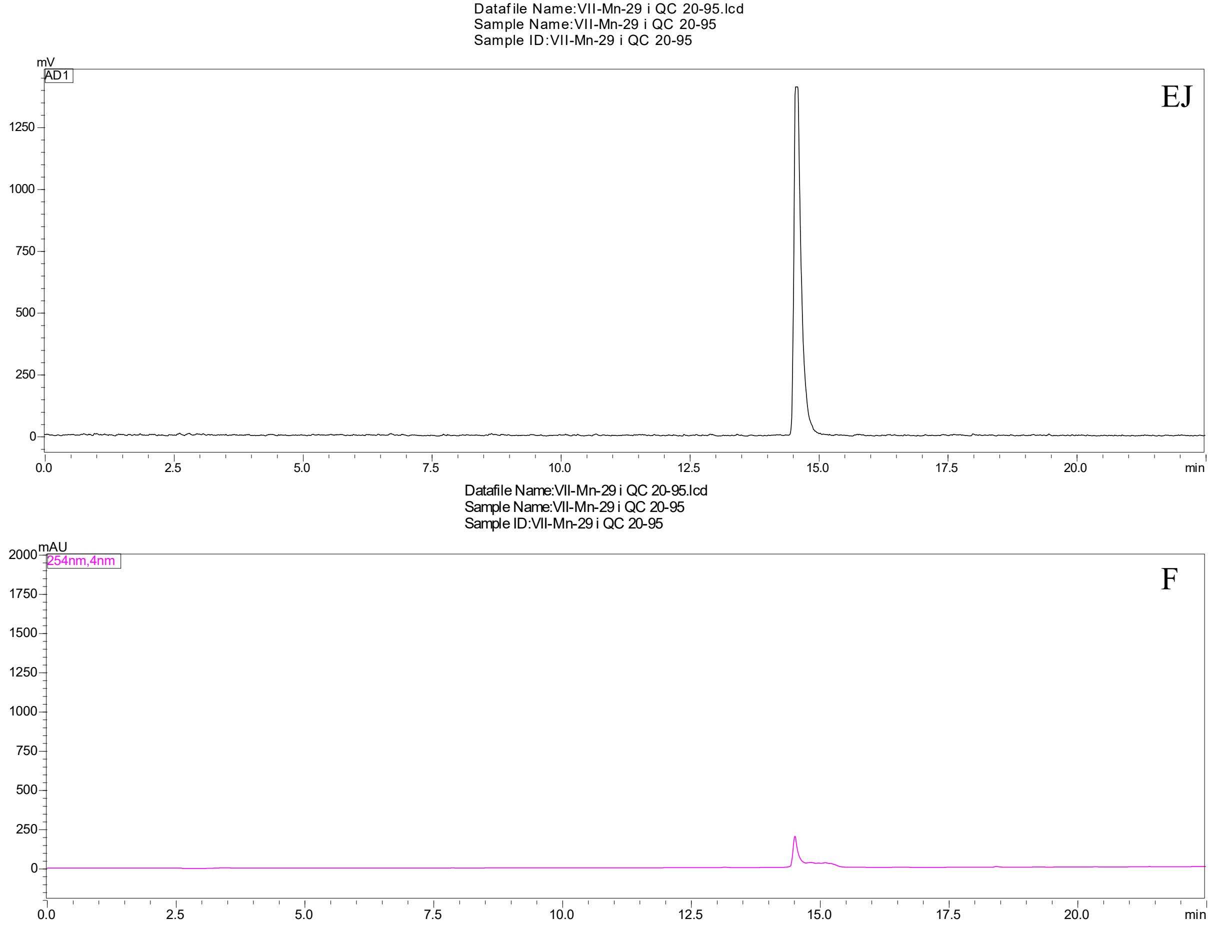

Figure S14: Radio-HPLC analysis of reaction mixture from **S7**. Reaction mixture with HPLC method 1 (A), authentic **[^{19}F]7** with HPLC method 1 (B), reaction mixture with HPLC method 1 (C), authentic **[^{19}F]7**with HPLC method 1 (D), and QC for **[^{18}F]7** (E and F).

HPLC Conditions for QC: Column: Phenomenex, Kinetex® 5µm F5 100 Å, 250 × 4.6 mm LC Column

Solvent A: 0.1% TFA water, Solvent B: 0.1% TFA acetonitrile; Isocratic / Gradient elution: 20% Solvent B for 0 to 2 min, 20% – 90% Solvent B for 2 to 22.5 min. Flow rate: 1 mL/min

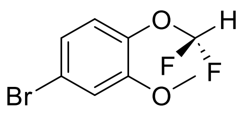

S8

Arene substrate:

4-Bromo-1-(difluoromethoxy)-2-methoxybenzene (S8)

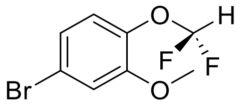

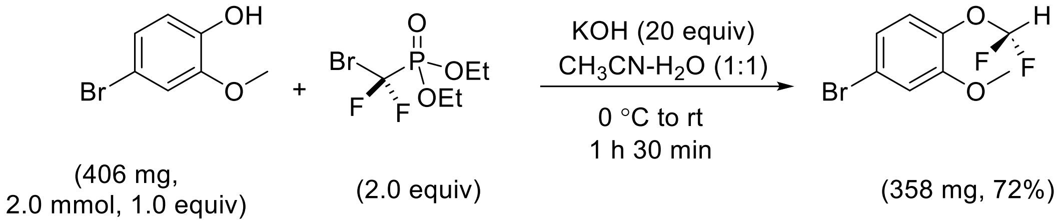

General conditions **A** were followed using 4-bromo-2-methoxyphenol (406 mg, 2.0 mmol, 1.0 equiv), $BrCF_2P(O)(OC_2H_5)_2$ (1.068 g, 4.0 mmol, 2.0 equiv), KOH (2.24 g, 40 mmol, 20 equiv), CH_3CN : H_2O (5 mL : 5 mL) for 1 h 30 min.

Purification: Gradient column chromatography [SiO_2, EtOAc:Hexanes 00:100 to 10:90)] to obtain pale yellowish liquid of **S8** (358 mg, 72%).

R_f : 0.8 (EtOAc : Hexanes 1:9)

^{1}H NMR ($CDCl_3$, 400 MHz): δ 7.07 (dt, J = 8.5, 2.0 Hz, 2H), 7.03 (d, J = 8.5 Hz, 1H), 6.60 (t, J = 74.8 Hz, 1H), 3.87 (s, 3H).

^{13}C NMR ($CDCl_3$, 101 MHz): δ 151.91, 139.01 (t, J = 3.6 Hz), 124.00, 123.85, 119.28, 116.20, 115.80 (t, J = 261.8 Hz), 56.23.

^{19}F NMR ($CDCl_3$, 376 MHz): δ –81.72 (d, J = 75.3 Hz).

HRMS (ESI-TOF) m/z: $[M + Na]^+$ Calcd. for $C_8H_7{}^{79}BrF_2NaO_2$ 274.9495; found 274.9496; and $[M + Na]^+$ Calcd. for $C_8H_7{}^{81}BrF_2NaO_2$ 276.9475; found 276.9476.

Deoxyfluorination and Authentic fluoroarene standard

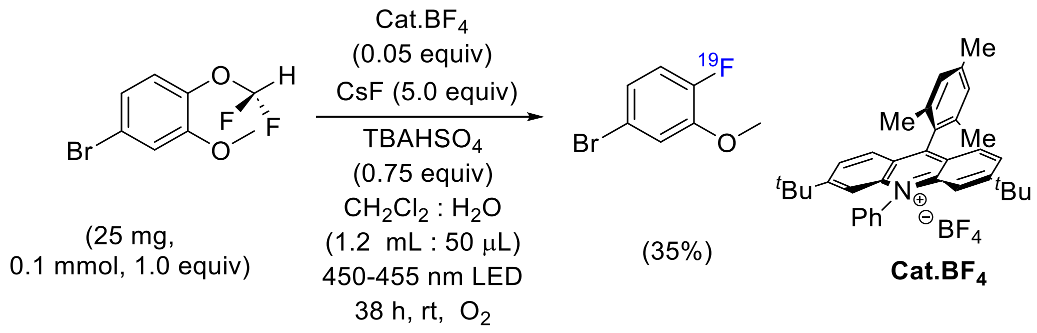

General conditions **I** were followed using 4-bromo-1-(difluoromethoxy)-2-methoxybenzene **S8** (25 mg, 0.1 mmol, 1.0 equiv), Mes-Acr-$Ph^+BF_4^-$ (0.05 equiv), CsF (5.0 equiv), $TBAHSO_4$ (0.75 equiv), CH_2Cl_2 : H_2O (0.1 M, 25:1), 450-455 nm LED, 38 h, 33 °C, O_2 balloon.

Purification: Gradient column chromatography [SiO_2, EtOAc:Hexanes 00:100 to 2:98)] to obtain colourless liquid of **[^{19}F]8** (7 mg, 35%).

^{19}F NMR ($CDCl_3$, 376 MHz): δ –136.88 (ddd, J = 12.1, 6.9, 3.5 Hz).

Data are comparable to that commercially available compound. CAS Number 103291-07-2

Radio-HPLC analysis and characterization

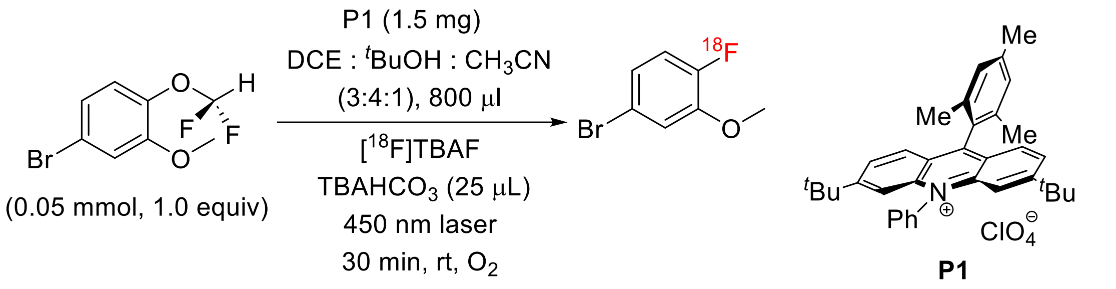

Entry	Activity at starting	Activity at EOS	Alumina Filtration	Injected Dose	Collected Dose	Isolatio n Time	Decay Corrected	Filtratio n Yield	HPLC Purity of Filtrate	%Yield
1	14.16 mCi	11.80 mCi	6.09 mCi	382 μCi	132 μCi	15.0 min	347.47 μCi	51.61%	37.98%	19.60%
2	15.90 mCi	13.48 mCi	6.84 μCi	573 μCi	148 μCi	18.5 min	521.21 μCi	50.74 %	28.39%	14.40%
										**Average %Yield: 17% (n=2)**

Table S13: HPLC isolated RCYs for **[^{18}F]8**

HPLC Conditions – method 1: Column: Phenomenex, Kinetex® 5μm F5 100 Å, 250 ✕ 4.6 mm LC Column

Solvent A: 0.1% TFA water, Solvent B: 0.1% TFA acetonitrile; Isocratic / Gradient elution: 20% Solvent B for 0 to 2 min, 20% – 95% Solvent B for 2 to 22.5 min. Flow rate: 1 mL/min.

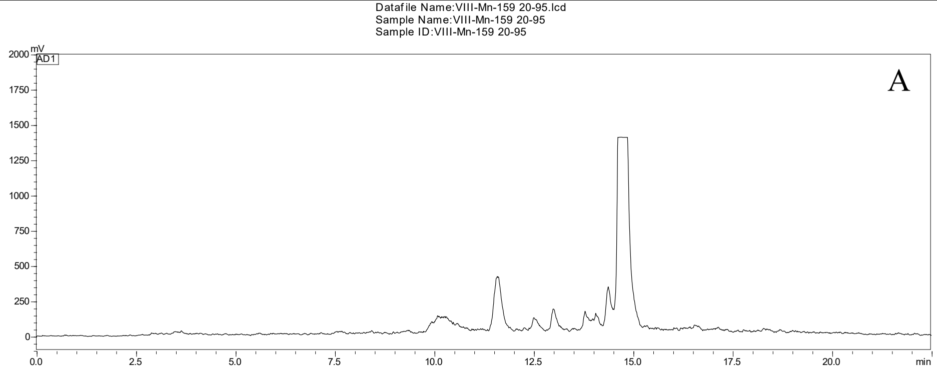
Datafile Name:VIII-Mn-159 20-95.lcd
Sample Name:VIII-Mn-159 20-95
Sample ID:VIII-Mn-159 20-95
mV
AD1
A
min

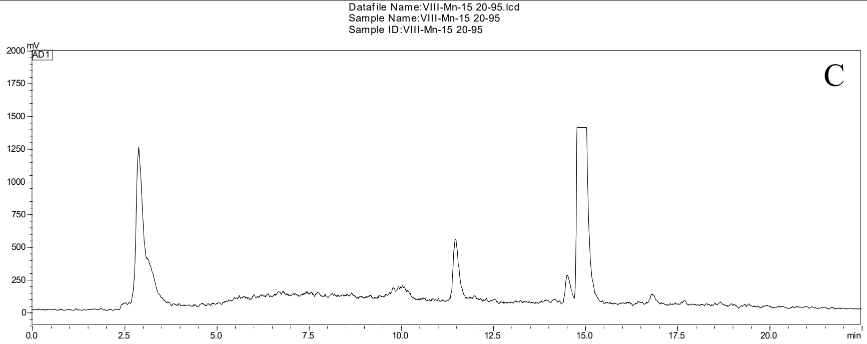
Datafile Name:VIII-Mn-15 20-95.lcd
Sample Name:VIII-Mn-15 20-95
Sample ID:VIII-Mn-15 20-95
mV
AD1
C
min

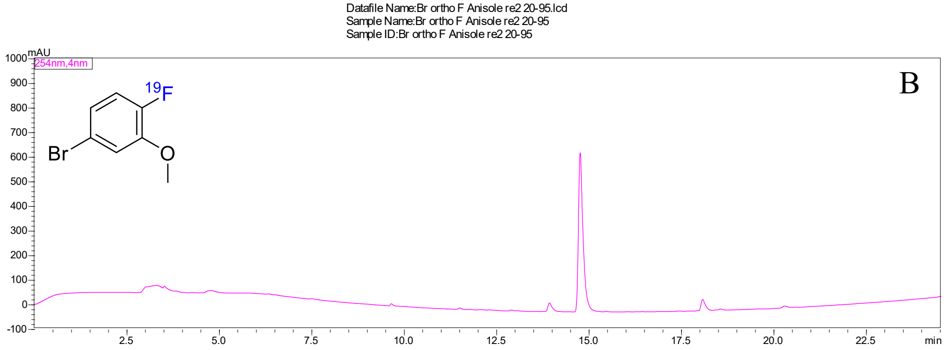
Datafile Name:Br ortho F Anisole re2 20-95.lcd
Sample Name:Br ortho F Anisole re2 20-95
Sample ID:Br ortho F Anisole re2 20-95
mAU
254nm,4nm
B
19F
Br
O
min

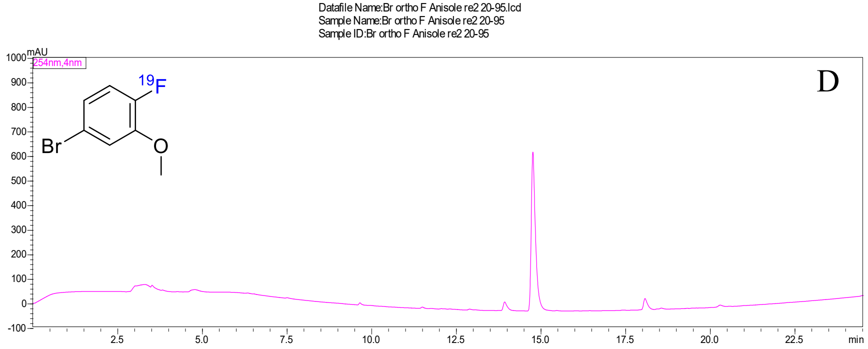
Datafile Name:Br ortho F Anisole re2 20-95.lcd
Sample Name:Br ortho F Anisole re2 20-95
Sample ID:Br ortho F Anisole re2 20-95
mAU
254nm,4nm
D
19F
Br
O
min

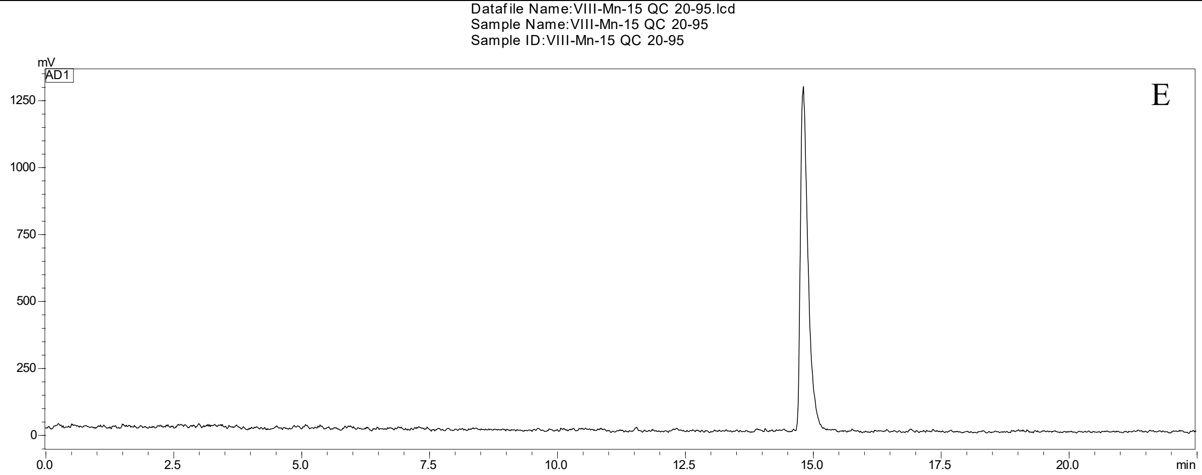

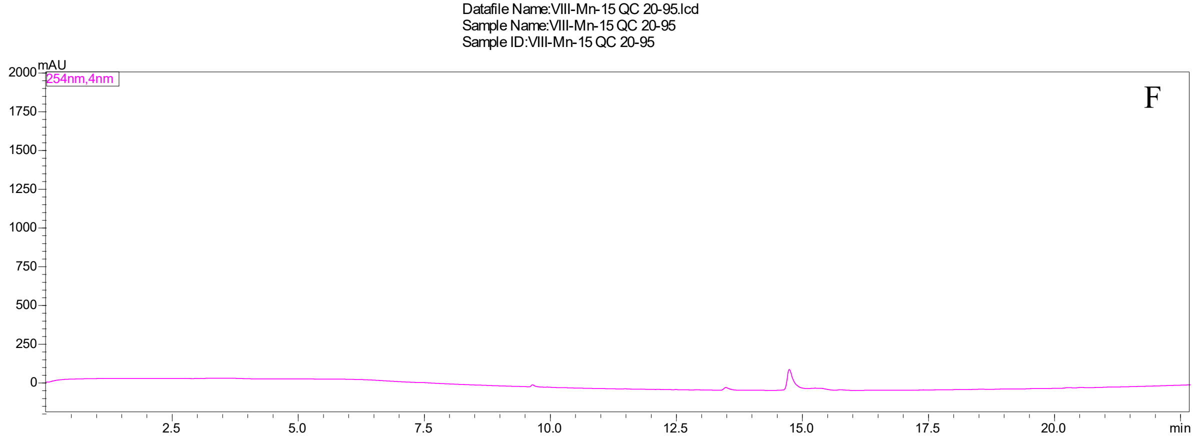

Figure S15: Radio-HPLC analysis of reaction mixture from **S8**. Reaction mixture with HPLC method 1 (A), authentic **[^{19}F]8** with HPLC method 1 (B), reaction mixture with HPLC method 1 (C), authentic **[^{19}F]8** with HPLC method 1 (D), and QC for **[^{18}F]8** (E and F).

HPLC Conditions for QC: Column: Phenomenex, Kinetex® 5µm F5 100 Å, 250 × 4.6 mm LC Column

Solvent A: 0.1% TFA water, Solvent B: 0.1% TFA acetonitrile; Isocratic / Gradient elution: 20% Solvent B for 0 to 2 min, 20% – 90% Solvent B for 2 to 22.5 min. Flow rate: 1 mL/min.

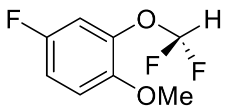

S9

Arene substrate:

2-(Difluoromethoxy)-4-fluoro-1-methoxybenzene (S9)

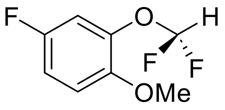

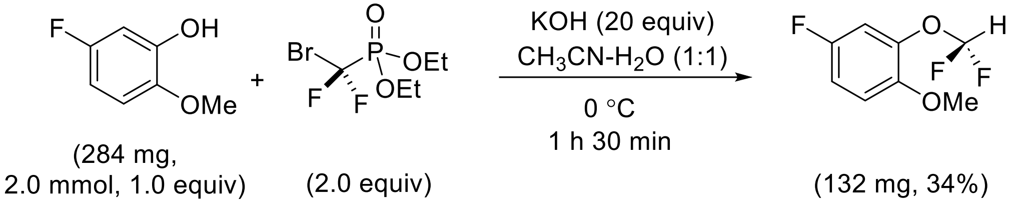

General conditions **A** were followed using 5-fluoro-2-methoxyphenol (284 mg, 2.0 mmol, 1.0 equiv), $BrCF_2P(O)(OC_2H_5)_2$ (1.068 g, 4.0 mmol, 2.0 equiv), KOH (2.24 g, 40 mmol, 20 equiv), CH_3CN : H_2O (5 mL : 5 mL) for 1 h 30 min.

Purification: Gradient column chromatography [SiO_2, EtOAc:Hexanes 05:95 to 10:90)] to obtain colourless liquid of **S9** (132 mg, 34%).

R_f : 0.4 (EtOAc : Hexanes 1:9)

^{1}H NMR ($CDCl_3$, 400 MHz): δ 6.95–6.92 (m, 1H), 6.91 (d, J = 1.8 Hz, 1H), 6.89 (d, J = 2.0 Hz, 1H), 6.56 (t, J= 74.7 Hz, 1H), 3.85 (s, 3H).

^{13}C NMR ($CDCl_3$, 101 MHz): δ 156.51(d, J = 241.0 Hz), 147.74 (d, J = 3.5 Hz), 140.21 (dt, J = 10.2, 3.6 Hz), 116.03 (t, J = 260.9 Hz), 113.3 (d, J = 8.7 Hz), 112.65 (d, J = 22.5 Hz), 110.56 (d, J = 26.1 Hz), 56.67.

^{19}F NMR ($CDCl_3$, 376 MHz): δ –81.98 (d, J = 74.7 Hz), –121.12 (ddd, J = 13.7, 8.3, 5.5 Hz).

HRMS (ESI-TOF) m/z: $[M + Na]^+$ Calcd. for $C_8H_7F_3NaO_2$ 215.0296; found 215.0296.

Authentic fluoroarene standard

^{19}F-Reference compound – **[^{19}F]9** was purchased from the commercial supplier. CAS Number 452-10-8

Radio-HPLC analysis and characterization

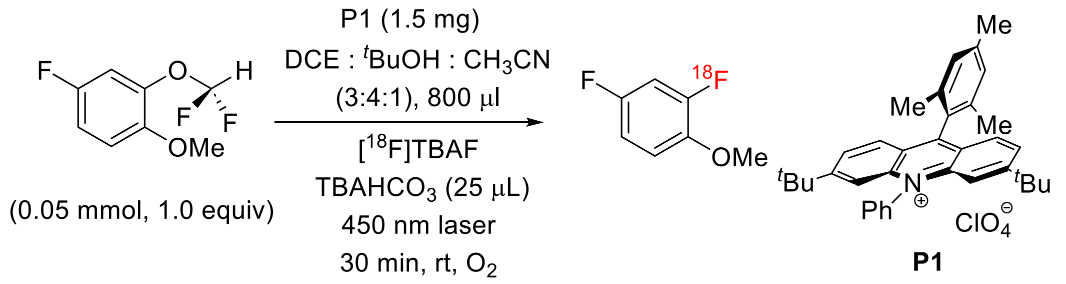

Entry	Activity at starting	Activity at EOS	Alumina Filtration	Injected Dose	Collected Dose	Isolation Time	Decay Corrected	Filtration Yield	HPLC Purity of Filtrate	%Yield
1	7.46 mCi	6.06 mCi	3.43 mCi	388 µCi	93 µCi	15.1 min	352.89 µCi	56.60%	26.35%	14.91%
2	10.72 mCi	8.48 mCi	5.95 mCi	454 µCi	63 µCi	15.1 min	415.28 µCi	70.16 %	15.17%	10.64%
										**Average %Yield: 12.8% (n=2)**

Table S14: HPLC isolated RCYs for **[^{18}F]9**

HPLC Conditions – method 1: Column: Phenomenex, Kinetex® 5µm F5 100 Å, 250 ✕ 4.6 mm LC Column

Solvent A: 0.1% TFA water, Solvent B: 0.1% TFA acetonitrile; Isocratic / Gradient elution: 20% Solvent B for 0 to 2 min, 20% – 95% Solvent B for 2 to 22.5 min. Flow rate: 1 mL/min

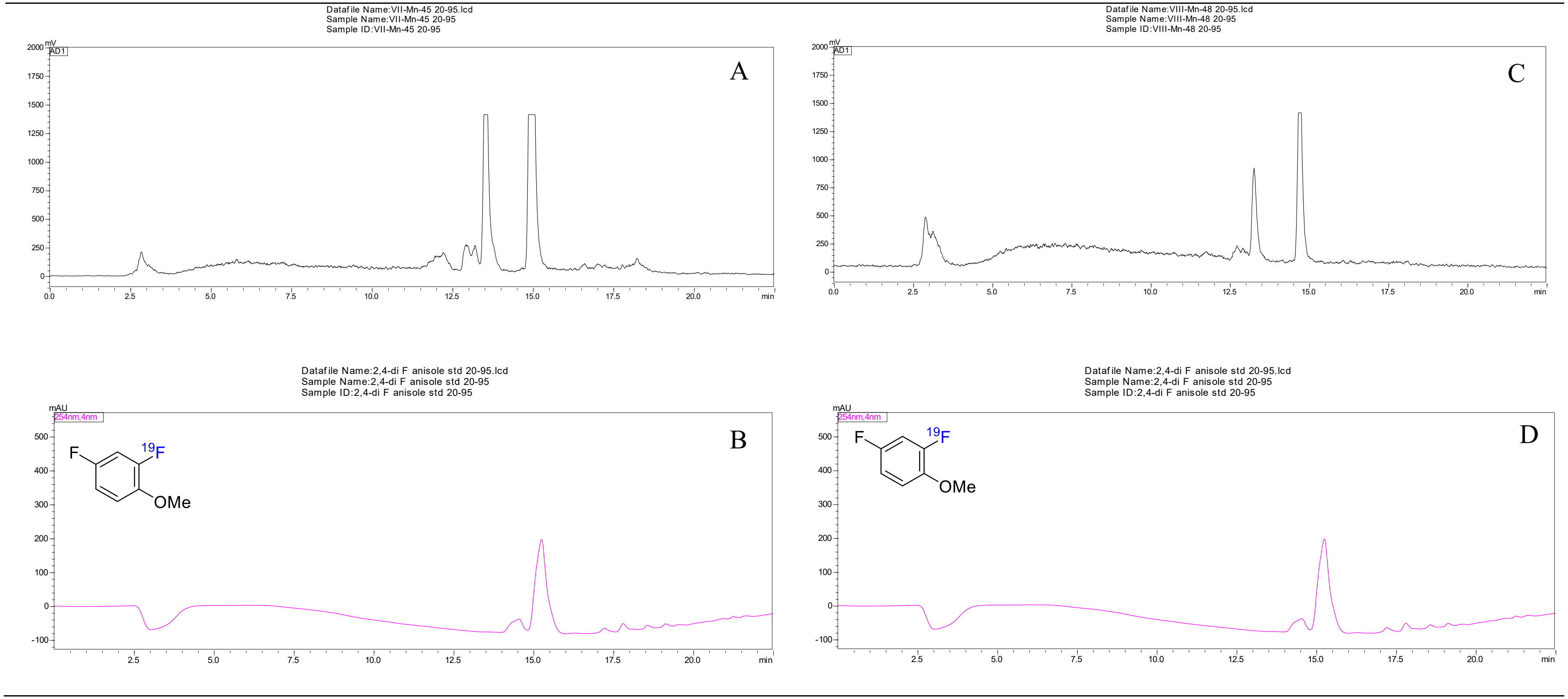
Datafile Name:VII-Mn-45 20-95.lcd
Sample Name:VII-Mn-45 20-95
Sample ID:VII-Mn-45 20-95
A
Datafile Name:VIII-Mn-48 20-95.lcd
Sample Name:VIII-Mn-48 20-95
Sample ID:VIII-Mn-48 20-95
C
Datafile Name:2,4-di F anisole std 20-95.lcd
Sample Name:2,4-di F anisole std 20-95
Sample ID:2,4-di F anisole std 20-95
B
F
19F
OMe
Datafile Name:2,4-di F anisole std 20-95.lcd
Sample Name:2,4-di F anisole std 20-95
Sample ID:2,4-di F anisole std 20-95
D
F
19F
OMe

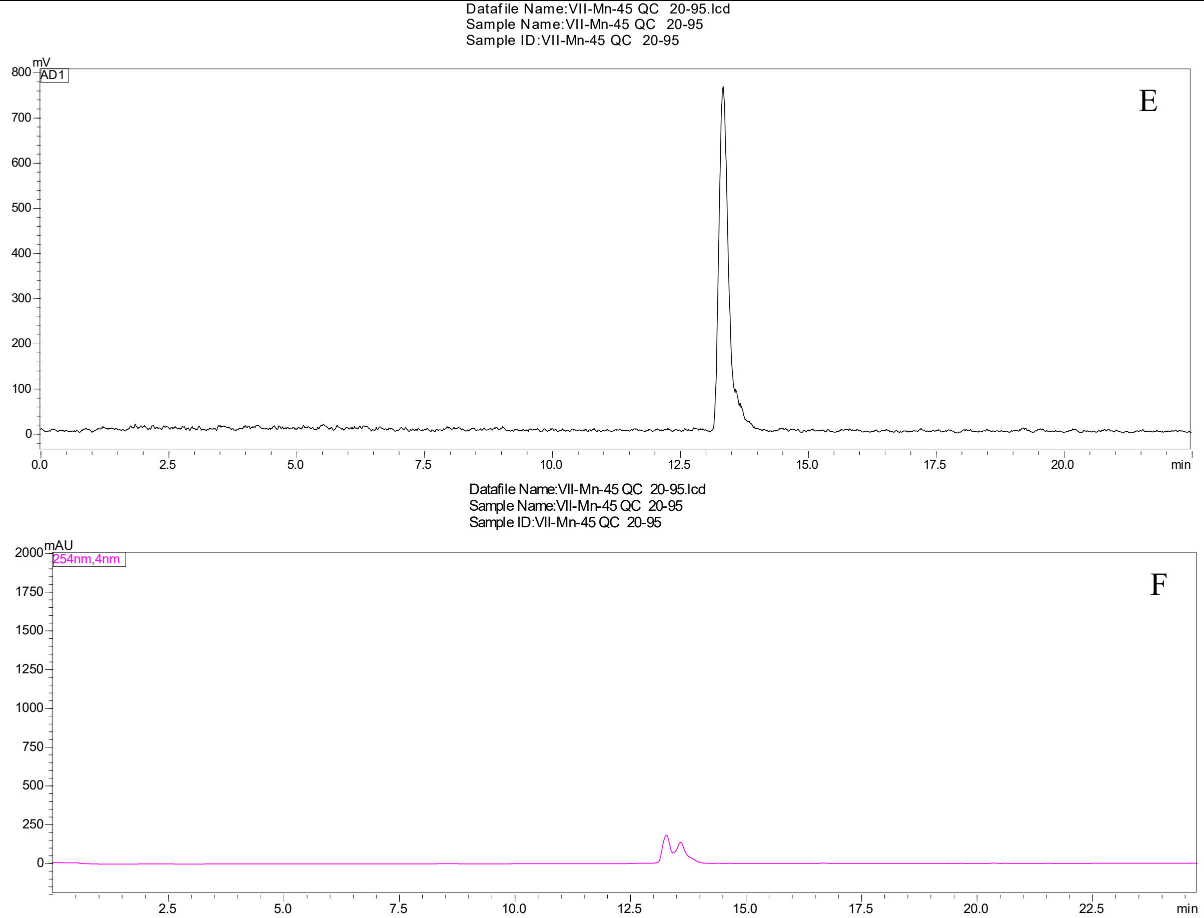

Figure S16: Radio-HPLC analysis of reaction mixture from **S9**. Reaction mixture with HPLC method 1 (A), authentic **[^{19}F]9** with HPLC method 1 (B), reaction mixture with HPLC method 1 (C), authentic **[^{19}F]9** with HPLC method 1 (D), and QC for **[^{18}F]9** (E and F).

HPLC Conditions for QC: Column: Phenomenex, Kinetex® 5µm F5 100 Å, 250 × 4.6 mm LC Column

Solvent A: 0.1% TFA water, Solvent B: 0.1% TFA acetonitrile; Isocratic / Gradient elution: 20% Solvent B for 0 to 2 min, 20% – 90% Solvent B for 2 to 22.5 min. Flow rate: 1 mL/min.

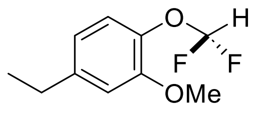

S10

Arene substrate:

1-(Difluoromethoxy)-4-ethyl-2-methoxybenzene (S10)

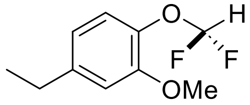

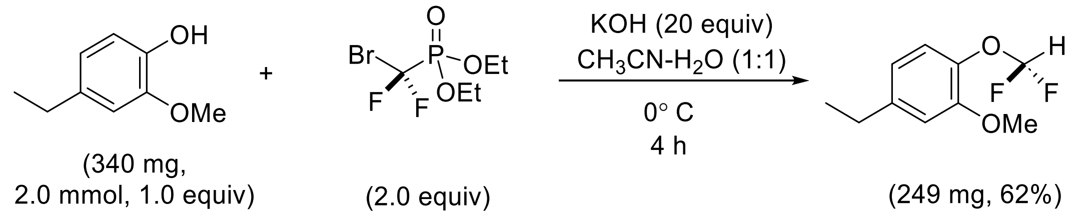

General conditions **A** were followed using 4-ethyl-2-methoxyphenol (340 mg, 2.0 mmol, 1.0 equiv), $BrCF_2P(O)(OC_2H_5)_2$ (1.068 g, 4.0 mmol, 2.0 equiv), KOH (2.24 g, 40 mmol, 20 equiv), CH_3CN: H_2O (5 mL : 5 mL) for 4 h.

Purification: Isocratic column chromatography [SiO_2, EtOAc:Hexanes 05:95] to obtain colourless liquid of **S10** (249 mg, 62%).

R_f : 0.8 (EtOAc : Hexanes 1:9)

^{1}H NMR ($CDCl_3$, 400 MHz): δ 7.06 (d, J = 8.1 Hz, 1H), 6.79 (d, J = 1.9 Hz, 1H), 6.75 (dd, J = 8.1, 1.9 Hz, 1H), 6.51 (t, J = 75.6 Hz, 1H), 3.87 (s, 3H), 2.63 (q, J = 7.6 Hz, 2H), 1.24 (t, J = 7.6 Hz, 3H).

^{13}C NMR ($CDCl_3$, 101 MHz): δ 150.88, 143.16, 137.90 (t, J = 3.7 Hz), 122.26, 120.04, 116.39 (t, J = 259.7 Hz), 112.36, 55.90, 28.74, 15.56.

^{19}F NMR ($CDCl_3$, 376 MHz): δ –81.31 (d, J = 76.3 Hz).

HRMS (ESI-TOF) m/z: $[M + Na]^+$ Calcd. for $C_{10}H_{12}F_2NaO_2$ 225.0703; found 225.0701.

Authentic fluoroarene standard

4-Ethyl-1-fluoro-2-methoxybenzene ([^{19}F]10)

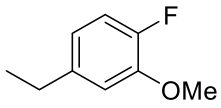

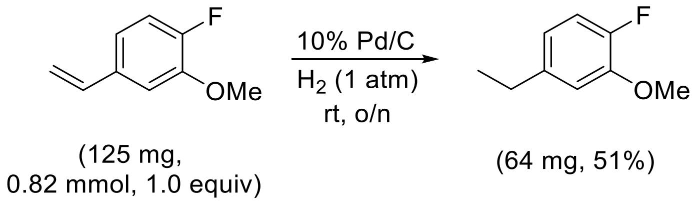

A 25 mL RBF was charged with 1-fluoro-2-methoxy-4-vinylbenzene (125 mg, 0.82 mmol, 1.0 equiv) in 2 mL MeOH. The RBF was purged and degassed with argon for three times. Pd/C 10% wt. (10 mg) was transferred to RBF. Argon was replaced with hydrogen, and the RBF was purged and degassed with hydrogen for three times. The reaction mixture was stirred under hydrogen atmosphere overnight. Next morning, the reaction mixture was diluted with MeOH and passed through celite and MeOH was evaporated under reduced pressure, and the crude was subsequently purified by column chromatography.

Purification: Gradient column chromatography [SiO_2, EtOAc:Hexanes 0:0 to 5:95)] to obtain the titled compound **[^{19}F]10** as colourless semisolid (64 mg, 51%).

R_f : 0.8 (EtOAc : Hexanes 1:9)

^{1}H NMR ($CDCl_3$, 500 MHz): δ 6.98 (dd, J = 11.4, 8.2 Hz, 1H), 6.79 (dd, J = 8.2, 1.9 Hz, 1H), 6.70 (dddd, J = 8.1, 6.4, 4.3, 2.1 Hz, 1H), 3.89 (s, 3H), 2.61 (q, J = 7.6 Hz, 2H), 1.23 (t, J = 7.6 Hz, 1H)

^{13}C NMR ($CDCl_3$, 126 MHz): δ 150.88 (d, J = 242.5 Hz), 147.37 (d, J = 10.2 Hz), 140.63 (d, J = 3.6 Hz), 119.91 (d, J = 6.5 Hz), 115.74 (d, J = 18.4 Hz), 113.27 (d, J = 1.5 Hz), 56.31, 28.71, 15.85.

^{19}F NMR ($CDCl_3$, 470 MHz): δ –140.15 (ddd, J =12.5, 8.3, 4.2 Hz).

HRMS (ESI-TOF) m/z: [M + Na]$^+$ Calcd. for $C_{10}H_{11}FNaO$ 177.0692; found 177.0690.

Radio-HPLC analysis and characterization

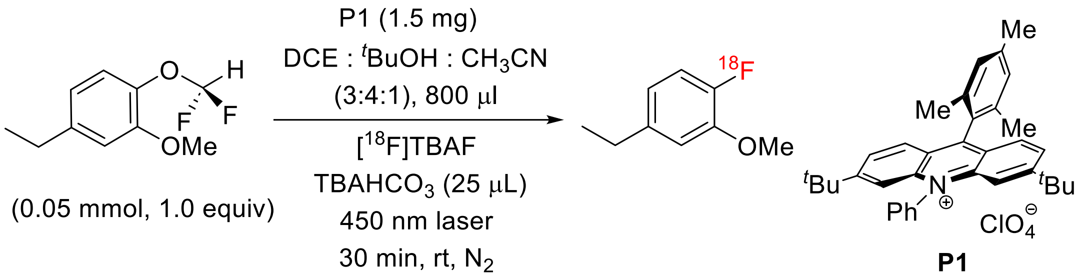

Entry	Activity at starting	Activity at EOS	Alumina Filtration	Injected Dose	Collected Dose	Isolation Time	Decay Corrected	Filtration Yield	HPLC Purity of Filtrate	%Yield
1*	8.45 mCi	6.38 mCi	3.18 mCi	277.3 µCi	123.8 µCi	8.0 min	263.63 µCi	49.84%	46.95%	23.39%
2	8.45 mCi	6.38 mCi	3.18 mCi	197.5 µCi	71.5 µCi	15.9 min	178.52 µCi	49.84%	40.05%	19.96%
3	20.10 mCi	16.03 mCi	8.38 mCi	406 µCi	203 µCi	17.6 min	364.44 µCi	52.27%	55.70%	29.11%
										**Average %Yield: 24.2% (n=2)**

Table S15: HPLC isolated RCYs for **[^{18}F]10**

HPLC Conditions – method 1: Column: Phenomenex, Kinetex® 5µm F5 100 Å, 250 ✕ 4.6 mm LC Column

Solvent A: 0.1% TFA water, Solvent B: 0.1% TFA acetonitrile; Isocratic / Gradient elution: 5% Solvent B for 0 to 2 min, 5% – 95% Solvent B for 2 to 22.5 min. Flow rate: 1 mL/min

* Entry 1 (not shown) and entry 2 are from the same experiment.

*HPLC Conditions – method 2: Column: Phenomenex, Kinetex® 5µm F5 100 Å, 250 ✕ 4.6 mm LC Column

Solvent A: 0.1% TFA water, Solvent B: 0.1% TFA acetonitrile; Isocratic / Gradient elution: 40% Solvent B for 0 to 2 min, 40% – 95% Solvent B for 2 to 22.5 min. Flow rate: 1 mL/min

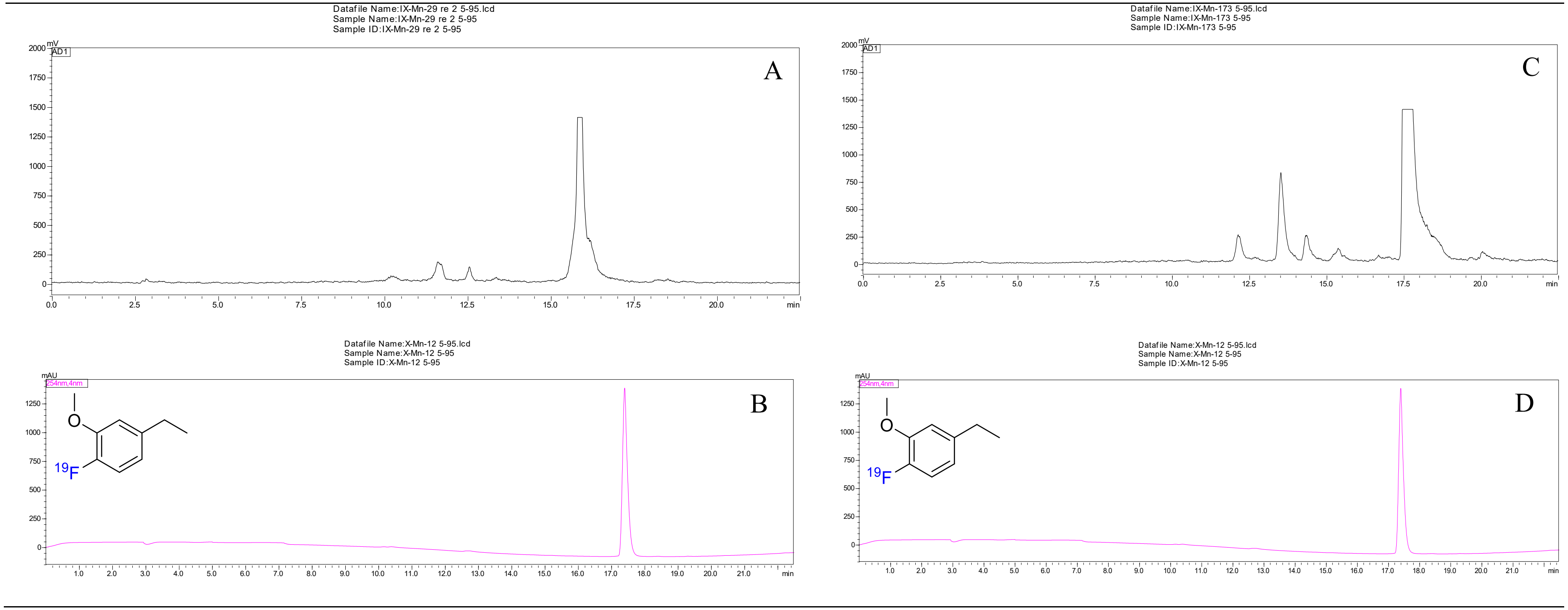

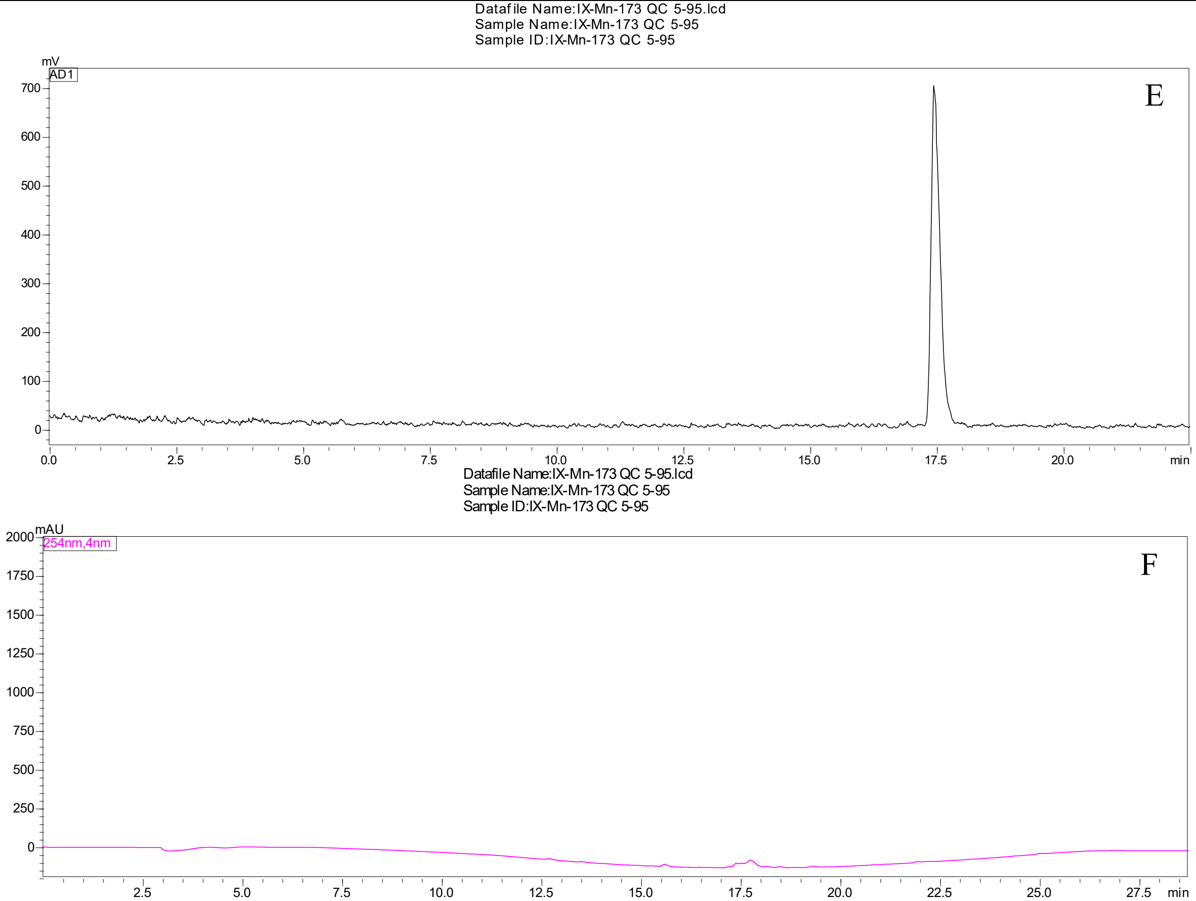

Figure S17: Radio-HPLC analysis of reaction mixture from **S10**. Reaction mixture with HPLC method 1 (A), authentic **[^{19}F]10** with HPLC method 1 (B), reaction mixture with HPLC method 1 (C), authentic **[^{19}F]10** with HPLC method 1 (D), and QC for **[^{18}F]10** (E and F).

HPLC Conditions for QC: Column: Phenomenex, Kinetex® 5µm F5 100 Å, 250 × 4.6 mm LC Column

Solvent A: 0.1% TFA water, Solvent B: 0.1% TFA acetonitrile; Isocratic / Gradient elution: 5% Solvent B for 0 to 2 min, 5% – 95% Solvent B for 2 to 22.5 min. Flow rate: 1 mL/min.

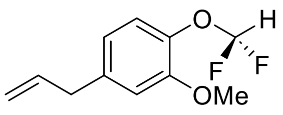

S11

Arene substrate:

4-Allyl-1-(difluoromethoxy)-2-methoxybenzene (**S11**)

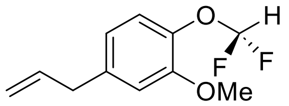

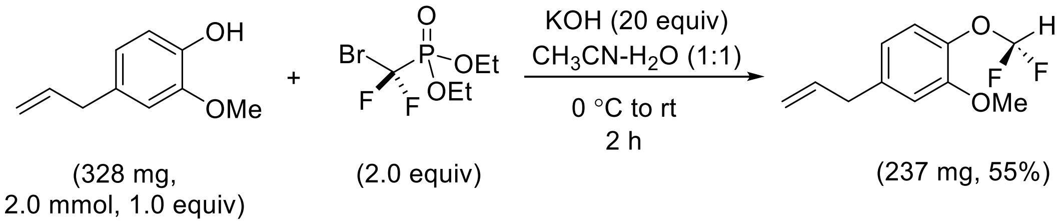

General conditions **A** were followed using 4-allyl-2-methoxyphenol (328 mg, 2.0 mmol, 1.0 equiv), $BrCF_2P(O)(OC_2H_5)_2$ (1.068 g, 4.0 mmol, 2.0 equiv), KOH (2.24 g, 40 mmol, 20 equiv), CH_3CN: H_2O (5 mL : 5 mL) for 2 h.

Purification: Isocratic column chromatography [SiO_2, EtOAc:Hexanes 05:95] to obtain pale greenish liquid of **S11** (237 mg, 55%).

R_f : 0.7 (EtOAc : Hexanes 1:9)

^{1}H NMR ($CDCl_3$, 400 MHz): δ 7.07 (d, J = 8.4 Hz, 1H), 6.78 (d, J = 1.8 Hz, 1H), 6.75 (dd, J = 8.1, 1.9 Hz, 1H), 6.51 (t, J = 75.9 Hz, 1H), 5.93 (dtd, J = 16.2, 9.6, 6.7 Hz, 1H), 5.13–5.07 (m, 2H), 3.87 (s, 3H), 3.36 (d, J = 6.7 Hz, 2H).

^{13}C NMR ($CDCl_3$, 101 MHz): δ 151.10, 139.09, 138.37 (d, J= 3.6 Hz), 137.01, 122.45, 120.09, 116.44 (t, J= 260.0 Hz), 116.43, 113.07, 56.05, 40.10.

^{19}F NMR ($CDCl_3$, 376 MHz): δ –81.36 (d, J = 74.9 Hz).

HRMS (ESI-TOF) m/z: $[M + Na]^+$ Calcd. for $C_{11}H_{12}F_2NaO_2$ 237.0703; found 237.0701.

Authentic fluoroarene standard

Data are comparable to that commercially available compound. CAS Number 1256479-13-6

Radio-HPLC analysis and characterization

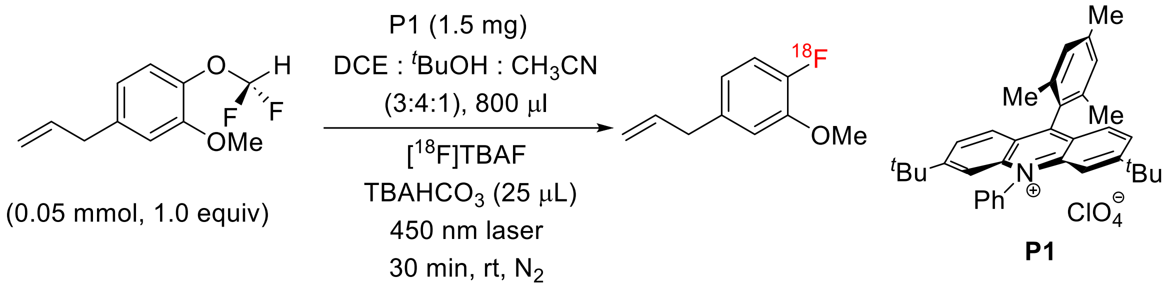

Entry	Activity at starting	Activity at EOS	Alumina Filtration	Injected Dose	Collected Dose	Isolation Time	Decay Corrected	Filtration Yield	HPLC Purity of Filtrate	%Yield
1	15.96 mCi	12.46 mCi	5.80 mCi	666 µCi	152 µCi	15.6 min	605.43 µCi	46.54%	25.10%	11.68%
2	17.35 mCi	13.91 mCi	5.85 mCi	317 µCi	77 µCi	15.6 min	288.07 µCi	42.05%	26.72%	11.23%
										**Average %Yield: 11.5% (n=2)**

Supplementary **Table S16**: HPLC isolated RCYs for **[^{18}F]11**

HPLC Conditions – method 1: Column: Phenomenex, Kinetex® 5µm F5 100 Å, 250 × 4.6 mm LC Column

Solvent A: 0.1% TFA water, Solvent B: 0.1% TFA acetonitrile; Isocratic / Gradient elution: 20% Solvent B for 0 to 2 min, 20% – 95% Solvent B for 2 to 22.5 min. Flow rate: 1 mL/min

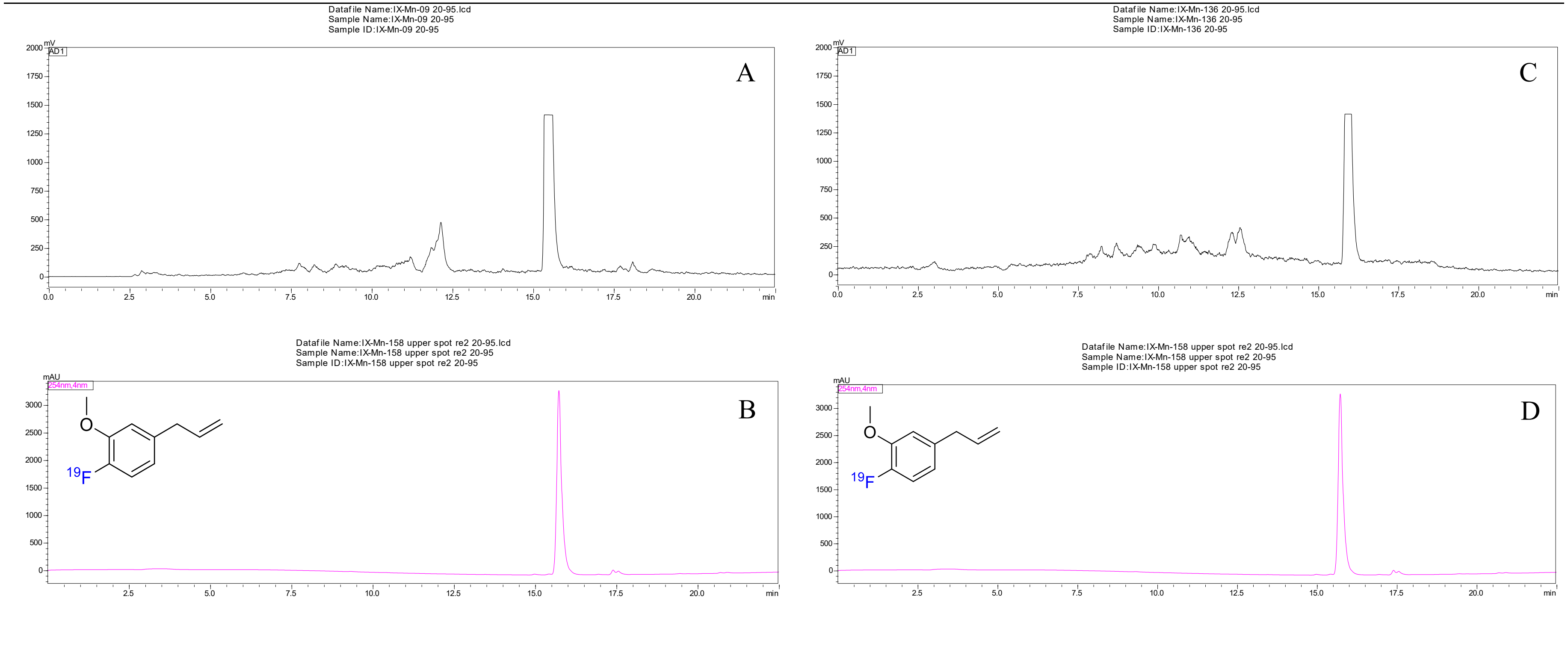
Datafile Name:IX-Mn-09 20-95.lcd
Sample Name:IX-Mn-09 20-95
Sample ID:IX-Mn-09 20-95
A
Datafile Name:IX-Mn-136 20-95.lcd
Sample Name:IX-Mn-136 20-95
Sample ID:IX-Mn-136 20-95
C
Datafile Name:IX-Mn-158 upper spot re2 20-95.lcd
Sample Name:IX-Mn-158 upper spot re2 20-95
Sample ID:IX-Mn-158 upper spot re2 20-95
B
Datafile Name:IX-Mn-158 upper spot re2 20-95.lcd
Sample Name:IX-Mn-158 upper spot re2 20-95
Sample ID:IX-Mn-158 upper spot re2 20-95
D
mV
AD1
mAU
254nm,4nm
min
O
19F

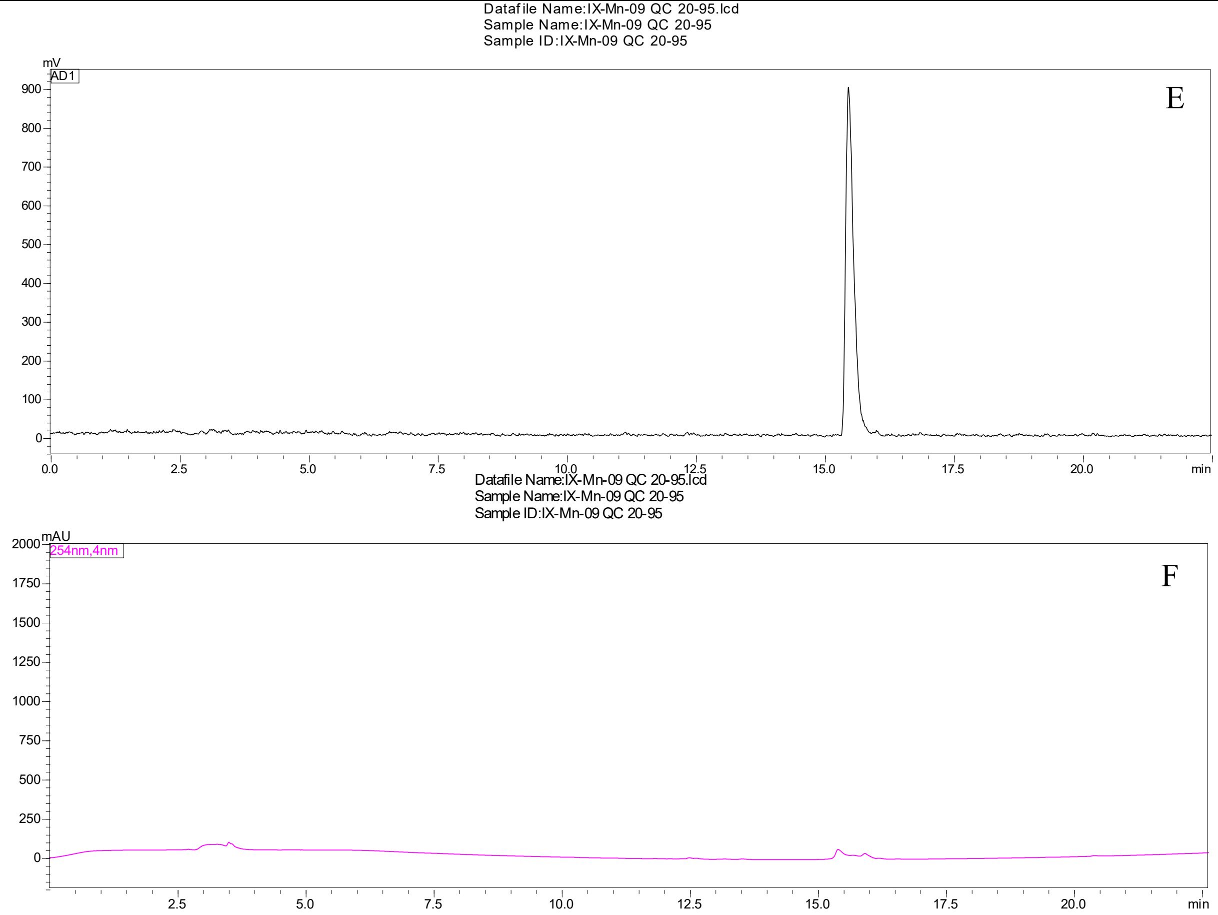

Figure S18: Radio-HPLC analysis of reaction mixture from **S11**. Reaction mixture with HPLC method 1 (A), authentic **[^{19}F]11** with HPLC method 1 (B), reaction mixture with HPLC method 1 (C), authentic **[^{19}F]11** with HPLC method 1 (D), and QC for **[^{18}F]11** (E and F).

HPLC Conditions for QC: Column: Phenomenex, Kinetex® 5µm F5 100 Å, 250 × 4.6 mm LC Column

Solvent A: 0.1% TFA water, Solvent B: 0.1% TFA acetonitrile; Isocratic / Gradient elution: 20% Solvent B for 0 to 2 min, 20% – 95% Solvent B for 2 to 22.5 min. Flow rate: 1 mL/min.

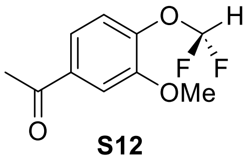

S12

<u>Arene substrate:</u>

1-(4-(Difluoromethoxy)-3-methoxyphenyl)ethan-1-one (S12)

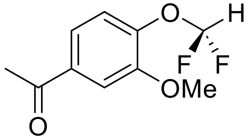

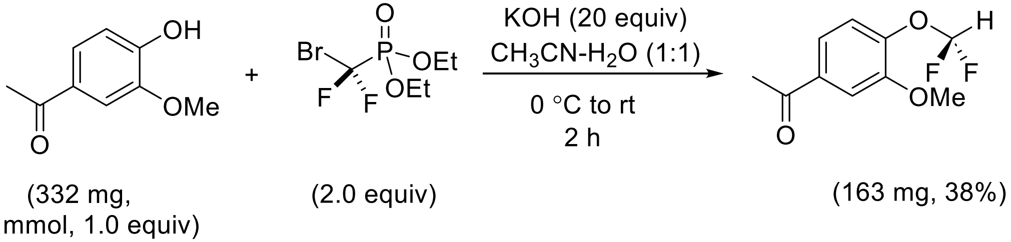

General conditions **A** were followed using 1-(4-hydroxy-3-methoxyphenyl)ethan-1-one (332 mg, 2.0 mmol, 1.0 equiv), $BrCF_2P(O)(OC_2H_5)_2$ (1.068 g, 4.0 mmol, 2.0 equiv), KOH (2.24 g, 40 mmol, 20 equiv), CH_3CN: H_2O (5 mL : 5 mL) for 2 h.

Purification: Gradient column chromatography [SiO_2, EtOAc:Hexanes 05:95 to 30:70)] to obtain colourless liquid of **S12** (168 mg, 38%).

R_f : 0.3 (EtOAc : Hexanes 1:9)

^{1}H NMR ($CDCl_3$, 400 MHz): δ 7.59 (d, J = 1.9 Hz, 1H), 7.52 (dd, J = 8.3, 1.9 Hz, 1H), 7.20 (dd, J = 8.3, 3.3 Hz, 1H), 6.72 (t, J = 75.8 Hz, 1H), 3.92 (s, 3H), 2.58 (s, 3H).

^{13}C NMR ($CDCl_3$, 101 MHz): δ 196.82, 151.13, 143.97 (t, J= 2.9 Hz), 135.45, 122.30, 121.28, 115.79 (t, J= 260.0 Hz), 111.78, 56.25, 26.59.

^{19}F NMR ($CDCl_3$, 376 MHz): δ –81.90 (d, J = 73.9 Hz).

HRMS (ESI-TOF) m/z: $[M + Na]^+$ Calcd. for $C_{10}H_{10}F_2NaO_3$ 239.0496; found 239.0494.

Deoxyfluorination and Authentic fluoroarene standard

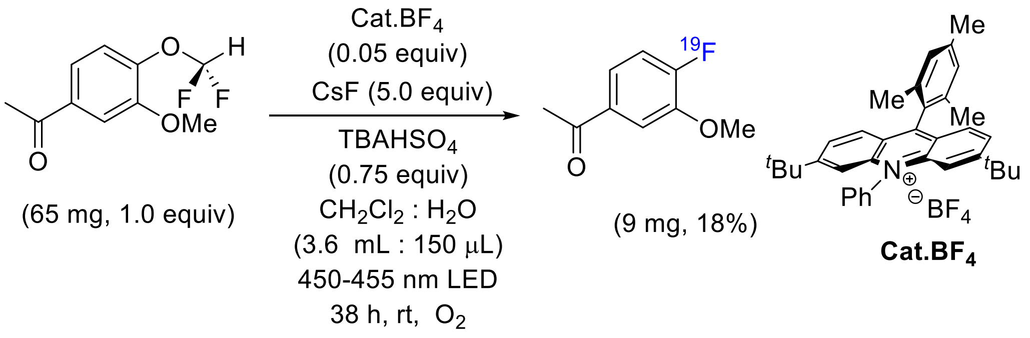

General conditions **I** were followed using 1-(4-(difluoromethoxy)-3-methoxyphenyl)ethan-1-one **S12** (65 mg, 0.3 mmol, 1.0 equiv), Mes-Acr-$Ph^+BF_4^-$ (0.05 equiv), CsF (5.0 equiv), $TBAHSO_4$ (0.75 equiv), CH_2Cl_2 : H_2O (0.1 M, 25:1), 450-455 nm LED, 38 h, 33 °C, O_2 balloon.

Purification: Gradient column chromatography [SiO_2, EtOAc:Hexanes 00:100 to 10:90)] to obtain yellow semisolid of **[^{18}F]12** (9 mg, 18%).

R_f : 0.4 (EtOAc : Hexanes 1:9)

^{1}H NMR ($CDCl_3$, 400 MHz): δ 7.61 (dd, J = 8.3, 2.0 Hz, 1H), 7.52 (ddd, J = 6.4, 4.3, 2.1 Hz, 1H), 7.14 (dd, J = 10.6, 8.4 Hz, 1H), 3.94 (s, 3H), 2.59 (s, 3H).

^{13}C NMR ($CDCl_3$, 101 MHz): δ 196.72, 155.80 (d, J= 255.1 Hz), 148.13 (d, J= 10.9 Hz), 134.03 (d, J= 3.6 Hz), 122.59 (d, J= 7.9 Hz), 115.96 (d, J= 19.7 Hz), 112.72 (d, J= 3.6 Hz), 56.04, 26.57.

^{19}F NMR ($CDCl_3$, 376 MHz): δ –127.02 (ddd, J = 11.1, 8.3, 4.2 Hz).

Data are comparable to that commercially available compound. CAS Number 64287-19-0

Radio-HPLC analysis and characterization

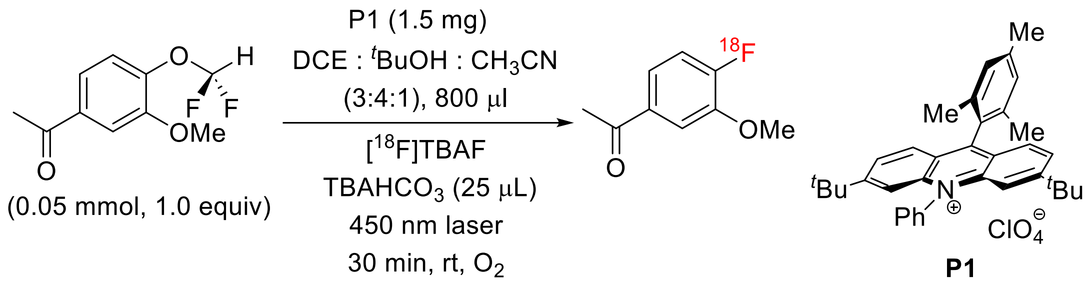

Entry	Activity at starting	Activity at EOS	Alumina Filtration	Injected Dose	Collected Dose	Isolation Time	Decay Corrected	Filtration Yield	HPLC Purity of Filtrate	%Yield
1	13.71 mCi	10.99 mCi	6.54 mCi	626 µCi	358 µCi	11.2 min	582.76 µCi	59.50%	61.43%	36.55%
2	12.63 mCi	9.75 mCi	5.17 mCi	210 µCi	162 µCi	11.2 min	195.86 µCi	53.02%	82.17%	43.03%
										**Average %Yield: 39.8% (n=2)**

Table S17: HPLC isolated RCYs for **[^{18}F]12**

HPLC Conditions: Column: Phenomenex, Kinetex® 5µm F5 100 Å, 250 × 4.6 mm LC Column

Solvent A: 0.1% TFA water, Solvent B: 0.1% TFA acetonitrile; Isocratic / Gradient elution: 20% Solvent B for 0 to 2 min, 20% – 95% Solvent B for 2 to 22.5 min. Flow rate: 1 mL/min

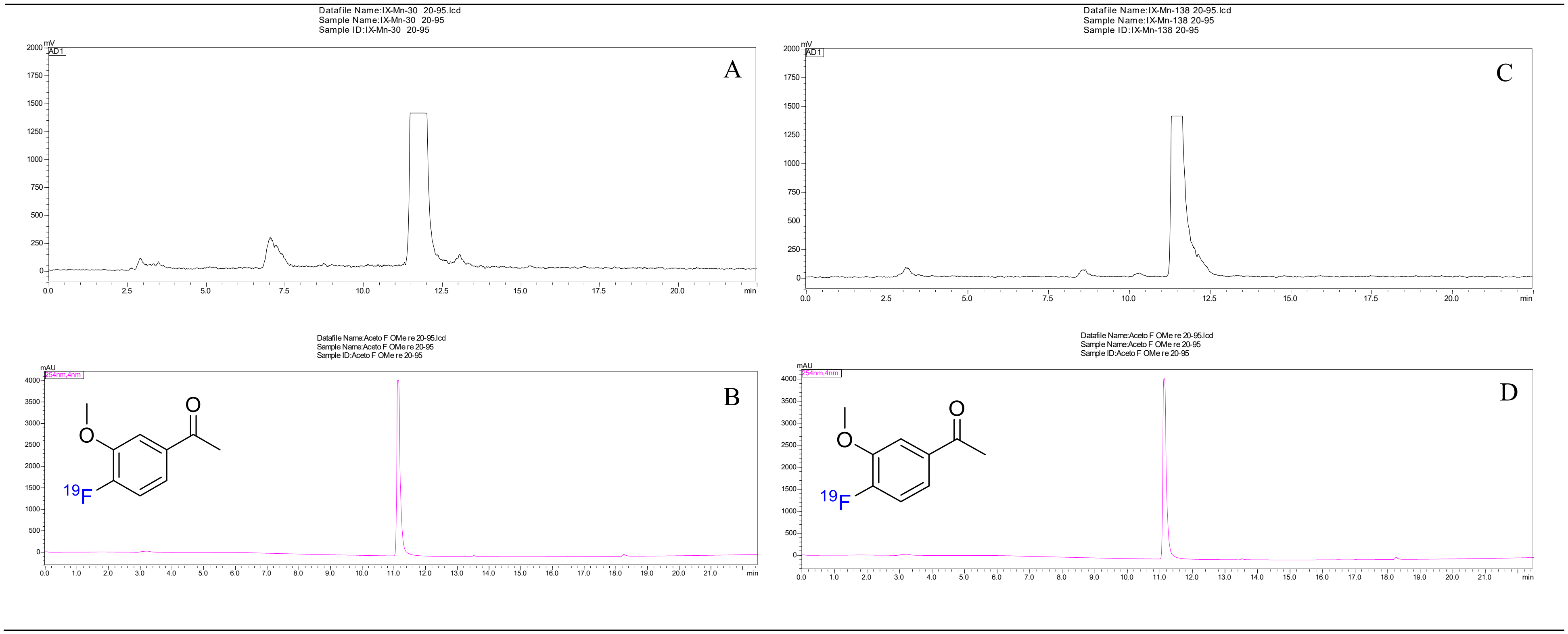
Datafile Name:IX-Mn-30 20-95.lcd
Sample Name:IX-Mn-30 20-95
Sample ID:IX-Mn-30 20-95
A
Datafile Name:IX-Mn-138 20-95.lcd
Sample Name:IX-Mn-138 20-95
Sample ID:IX-Mn-138 20-95
C
Datafile Name:Aceto F OMe re 20-95.lcd
Sample Name:Aceto F OMe re 20-95
Sample ID:Aceto F OMe re 20-95
B
D
19F

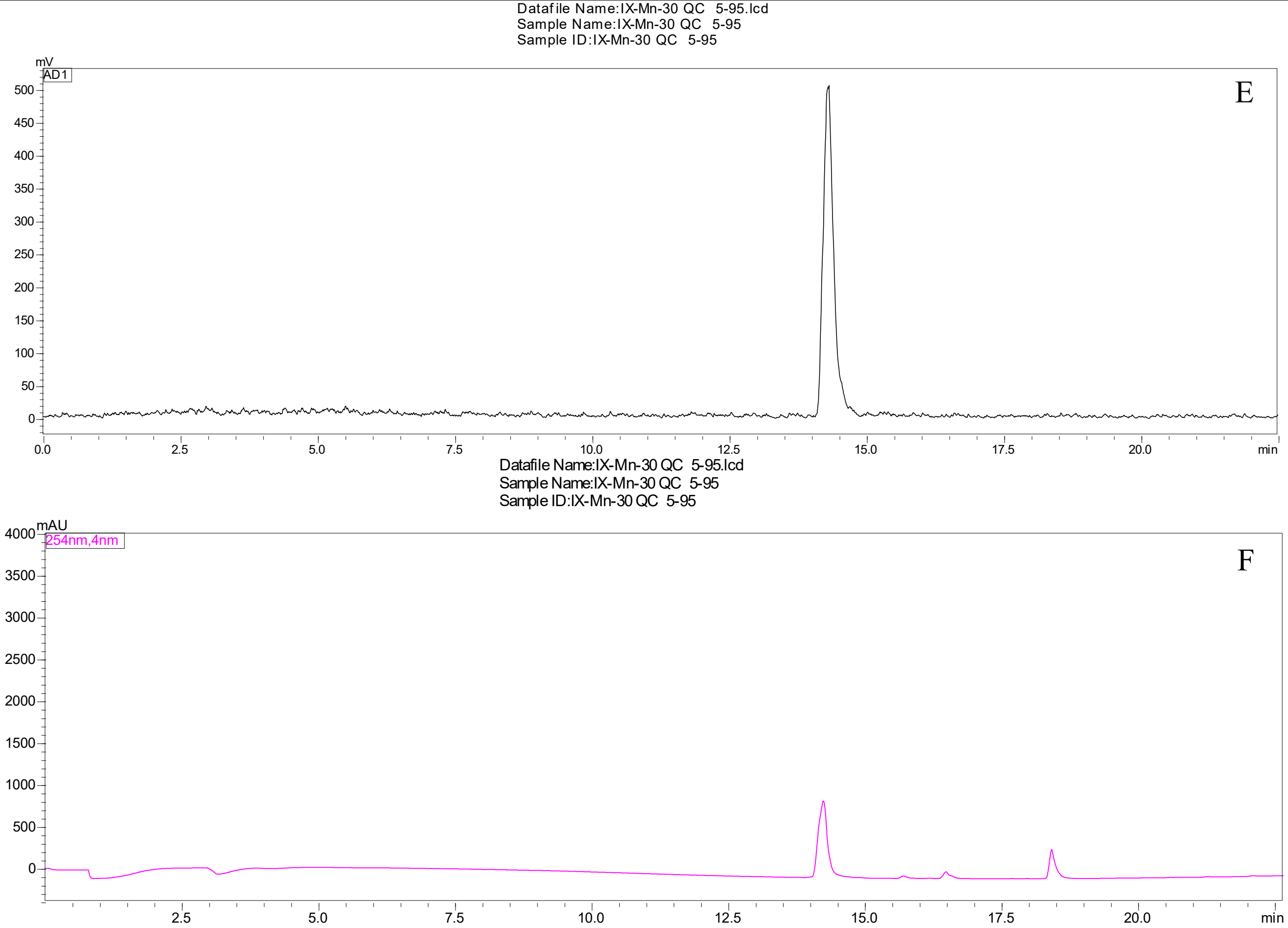

Figure S19: Radio-HPLC analysis of reaction mixture from **S12**. Reaction mixture with HPLC method 1 (A), authentic **[^{19}F]12** with HPLC method 1 (B), reaction mixture with HPLC method 1 (C), authentic **[^{19}F]12** with HPLC method 1 (D), and QC for **[^{18}F]12** (E and F).

HPLC Conditions for QC: Column: Phenomenex, Kinetex® 5µm F5 100 Å, 250 × 4.6 mm LC Column
Solvent A: 0.1% TFA water, Solvent B: 0.1% TFA acetonitrile; Isocratic / Gradient elution: 5% Solvent B for 0 to 2 min, 5% – 95% Solvent B for 2 to 22.5 min. Flow rate: 1 mL/min.

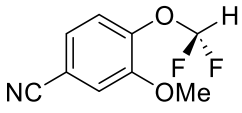

S13

Arene substrate:

4-(Difluoromethoxy)-3-methoxybenzonitrile (S13)

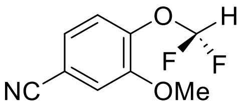

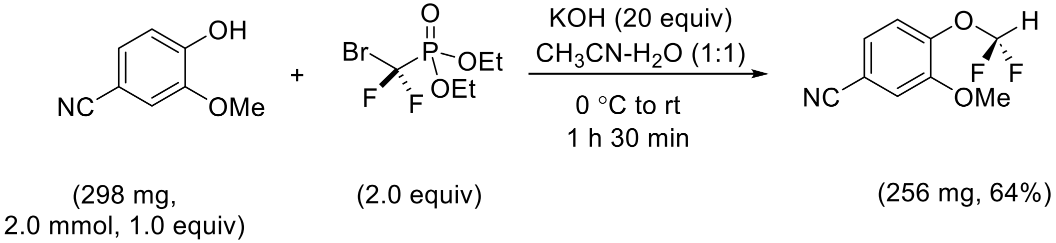

General conditions **A** were followed using 4-hydroxy-3-methoxybenzonitrile (298 mg, 2.0 mmol, 1.0 equiv), $BrCF_2P(O)(OC_2H_5)_2$ (1.068 g, 4.0 mmol, 2.0 equiv), KOH (2.24 g, 40 mmol, 20 equiv), CH_3CN : H_2O (5 mL : 5 mL) for 1 h 30 min.

Purification: Gradient column chromatography [SiO_2, EtOAc:Hexanes 05:95 to 30:70)] to obtain white solid of **S13** (256 mg, 64%).

R_f : 0.4 (EtOAc : Hexanes 3:7)

^{1}H NMR ($CDCl_3$, 400 MHz): δ 7.28 (d, J = 8.2, 1.7 Hz, 1H), 7.26–7.25 (m, 1H), 7.23–7.21 (m, 1H), 6.71 (t, J = 71.9 Hz, 1H), 3.92 (s, 3H).

^{13}C NMR ($CDCl_3$, 101 MHz): δ 151.49, 143.67 (t, J = 2.9 Hz), 125.76, 122.51, 118.18 (d, J = 7.2 Hz), 115.98, 114.23 (t, J = 262.5 Hz), 110.2, 56.50.

^{19}F NMR ($CDCl_3$, 376 MHz): δ –82.08 (d, J = 73.7 Hz).

HRMS (ESI-TOF) m/z: $[M + Na]^+$ Calcd. for $C_9H_7F_2NNaO_2$ 222.0343; found 222.0341.

Authentic fluoroarene standard

^{19}F-Reference compound – **[^{19}F]13** was purchased from the commercial supplier. CAS Number 243128-37-2

Radio-HPLC analysis and characterization

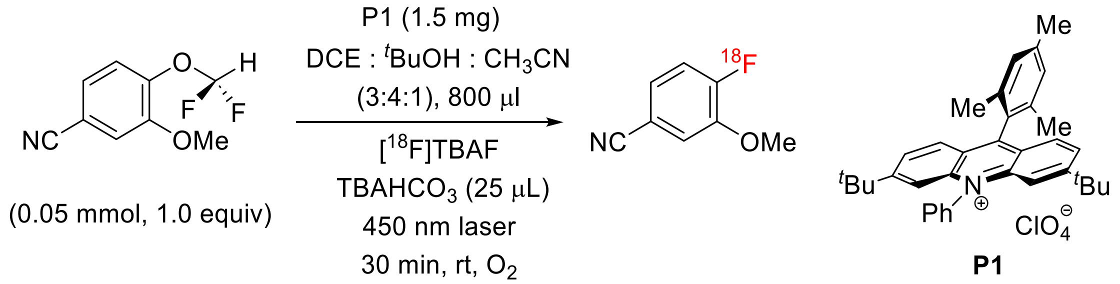

Entry	Activity at starting	Activity at EOS	Alumina Filtration	Injected Dose	Collected Dose	Isolation Time	Decay Corrected	Filtration Yield	HPLC Purity of Filtrate	%Yield
1	5.34 mCi	4.20 mCi	924 µCi	489 µCi	175 µCi	10.14 min	458.40 µCi	22.00%	38.17 %	8.39%
2	8.20 mCi	6.71 mCi	964 µCi	315 µCi	45 µCi	10.14 min	295.69 µCi	14.36%	15.21%	2.18%
									**Average %Yield: 5.3% (n=2)**	

Table S18: HPLC isolated RCYs for **[^{18}F]13**

HPLC Conditions – method 1: Column: Phenomenex, Kinetex® 5µm F5 100 Å, 250 × 4.6 mm LC Column

Solvent A: 0.1% TFA water, Solvent B: 0.1% TFA acetonitrile; Isocratic / Gradient elution: 30% Solvent B for 0 to 2 min, 30% – 95% Solvent B for 2 to 22.5 min. Flow rate: 1 mL/min

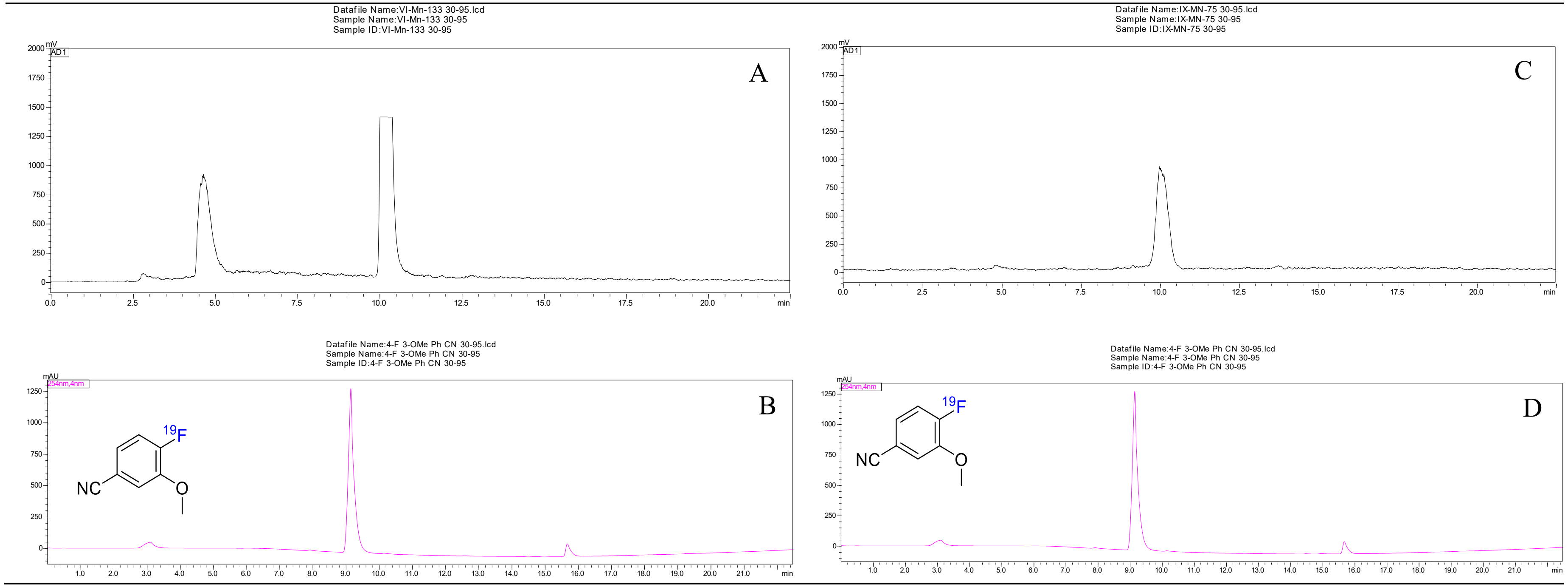
Datafile Name:VI-Mn-133 30-95.lcd
Sample Name:VI-Mn-133 30-95
Sample ID:VI-Mn-133 30-95
mV
AD1
A
min
Datafile Name:IX-MN-75 30-95.lcd
Sample Name:IX-MN-75 30-95
Sample ID:IX-MN-75 30-95
mV
AD1
C
min
Datafile Name:4-F 3-OMe Ph CN 30-95.lcd
Sample Name:4-F 3-OMe Ph CN 30-95
Sample ID:4-F 3-OMe Ph CN 30-95
mAU
254nm,4nm
B
19F
NC
O
min
Datafile Name:4-F 3-OMe Ph CN 30-95.lcd
Sample Name:4-F 3-OMe Ph CN 30-95
Sample ID:4-F 3-OMe Ph CN 30-95
mAU
254nm,4nm
D
19F
NC
O
min

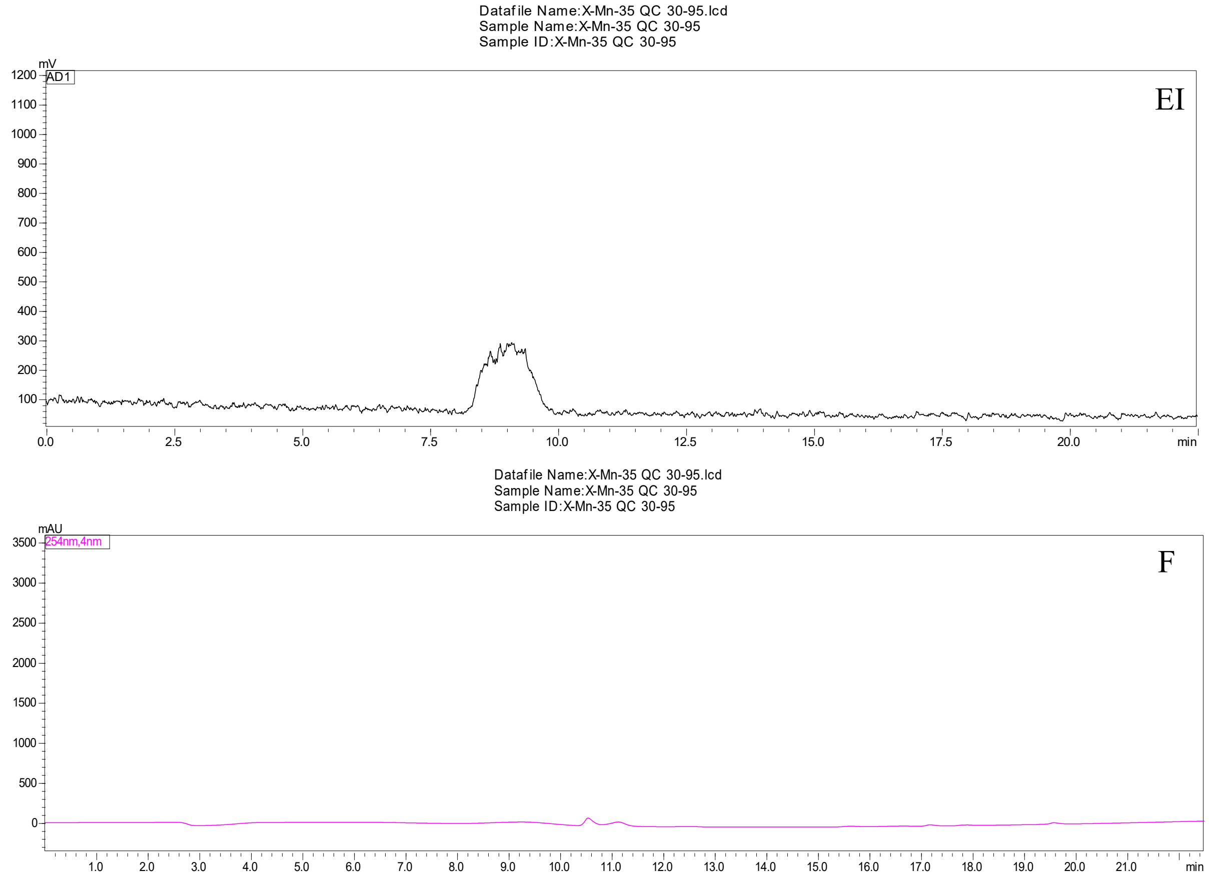

Figure S20: Radio-HPLC analysis of reaction mixture from **S13**. Reaction mixture with HPLC method 1 (A), authentic **[^{19}F]13** with HPLC method 1 (B), reaction mixture with HPLC method 1 (C), authentic **[^{19}F]13** with HPLC method 1 (D), and QC for **[^{18}F]13** (E and F).

HPLC Conditions for QC: Column: Phenomenex, Kinetex® 5µm F5 100 Å, 250 × 4.6 mm LC Column

Solvent A: 0.1% TFA water, Solvent B: 0.1% TFA acetonitrile; Isocratic / Gradient elution: 30% Solvent B for 0 to 2 min, 30% – 95% Solvent B for 2 to 22.5 min. Flow rate: 1 mL/min.

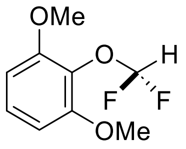

S14

Arene substrate:

2-(Difluoromethoxy)-1,3-dimethoxybenzene (S14)

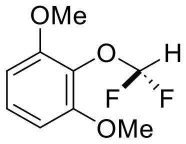

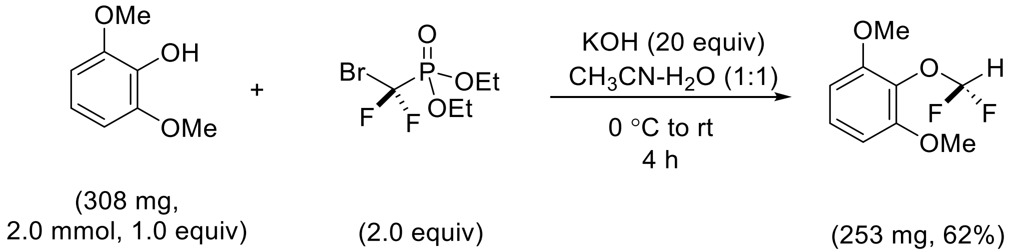

General conditions **A** were followed using 2,6-dimethoxyphenol (308 mg, 2.0 mmol, 1.0 equiv), $BrCF_2P(O)(OC_2H_5)_2$ (1.068 g, 4.0 mmol, 2.0 equiv), KOH (2.24 g, 40 mmol, 20 equiv), CH_3CN: H_2O (5 mL : 5 mL) for 4 h.

Purification: Gradient column chromatography [SiO_2, EtOAc:Hexanes 05:95 to 10:90)] to obtain white solid of **S14** (253 mg, 62%).

R_f : 0.5 (EtOAc : Hexanes 1:9)

^{1}H NMR ($CDCl_3$, 400 MHz): δ 7.12 (d, J = 8.4 Hz, 1H), 6.61 (d, J = 8.4 Hz, 2H), 6.54 (t, J = 76.4 Hz, 1H), 3.86 (s, 6H).

^{13}C NMR ($CDCl_3$, 101 MHz): δ 153.27, 129.46 (t, J = 3.6 Hz), 126.49, 117.01 (t, J = 259.5 Hz), 105.31, 56.37.

^{19}F NMR ($CDCl_3$, 376 MHz): δ –81.62 (d, J = 76.3 Hz).

HRMS (ESI-TOF) m/z: $[M + Na]^+$ Calcd. for $C_9H_{11}F_2O_3$ 227.0496; found 227.0493.

Authentic fluoroarene standard

^{19}F-Reference compound – **[^{19}F]13** was purchased from the commercial supplier. CAS Number 195136-68-6

Radio-HPLC analysis and characterization

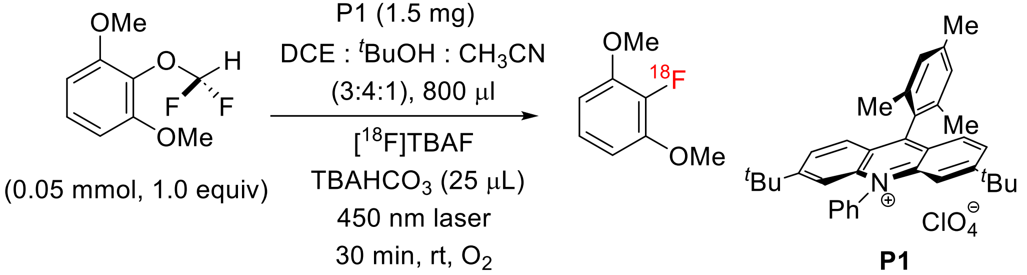

Entry	Activity at starting	Activity at EOS	Alumina Filtration	Injected Dose	Collected Dose	Isolation Time	Decay Corrected	Filtration Yield	HPLC Purity of Filtrate	%Yield
1*	14.57 mCi	11.06 mCi	8.18 mCi	371 µCi	167 µCi	17.8 min	332.95 µCi	73.96%	50.15%	37.09%
2*	10.24 mCi	8.40 mCi	5.47 mCi	443 µCi	124 µCi	16.2 min	400.34 µCi	65.11 %	30.97%	20.16%
								**Average %Yield: 28.6% (n=2)**		

Table S19: HPLC isolated RCYs for **[^{18}F]14**

*HPLC Conditions – method 1: Column: Phenomenex, Kinetex® 5µm F5 100 Å, 250 ✕ 4.6 mm LC Column

Solvent A: 0.1% TFA water, Solvent B: 0.1% TFA acetonitrile; Isocratic / Gradient elution: 5% Solvent B for 0 to 2 min, 5% – 60% Solvent B for 2 to 22.5 min. Flow rate: 1 mL/min

**HPLC Conditions – method 2: Column: Phenomenex, Kinetex® 5µm F5 100 Å, 250 ✕ 4.6 mm LC Column

Solvent A: 0.1% TFA water, Solvent B: 0.1% TFA acetonitrile; Isocratic / Gradient elution: 5% Solvent B for 0 to 2 min, 5% – 75% Solvent B for 2 to 22.5 min. Flow rate: 1 mL/min

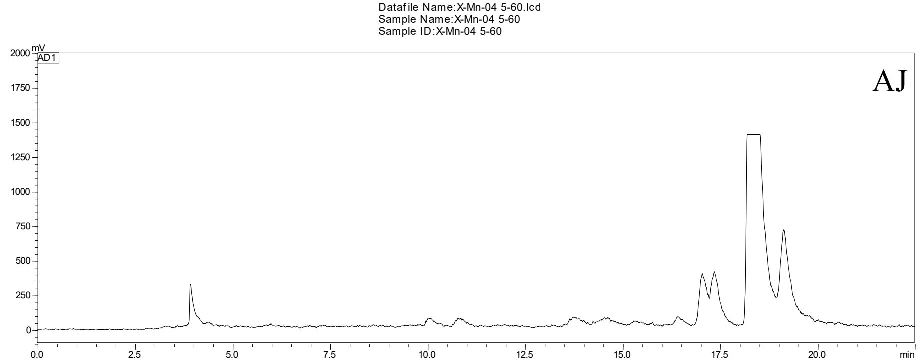

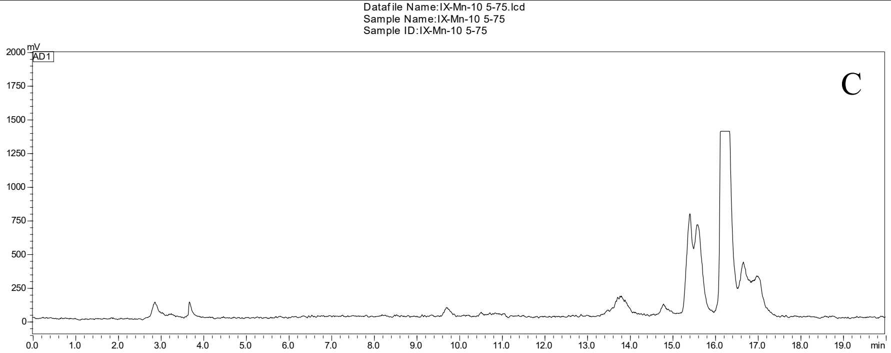

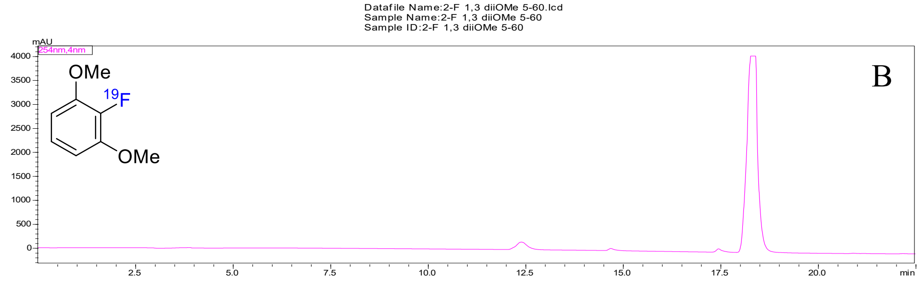

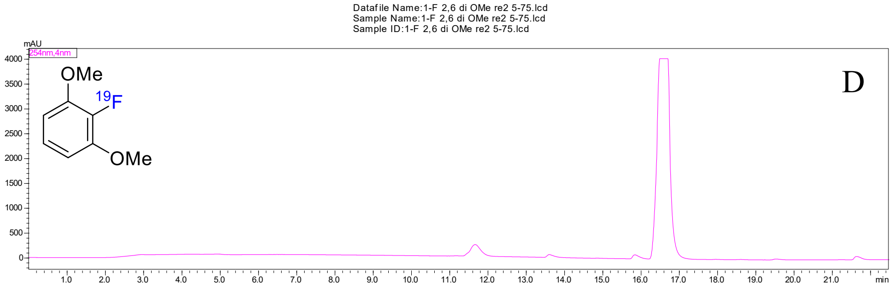

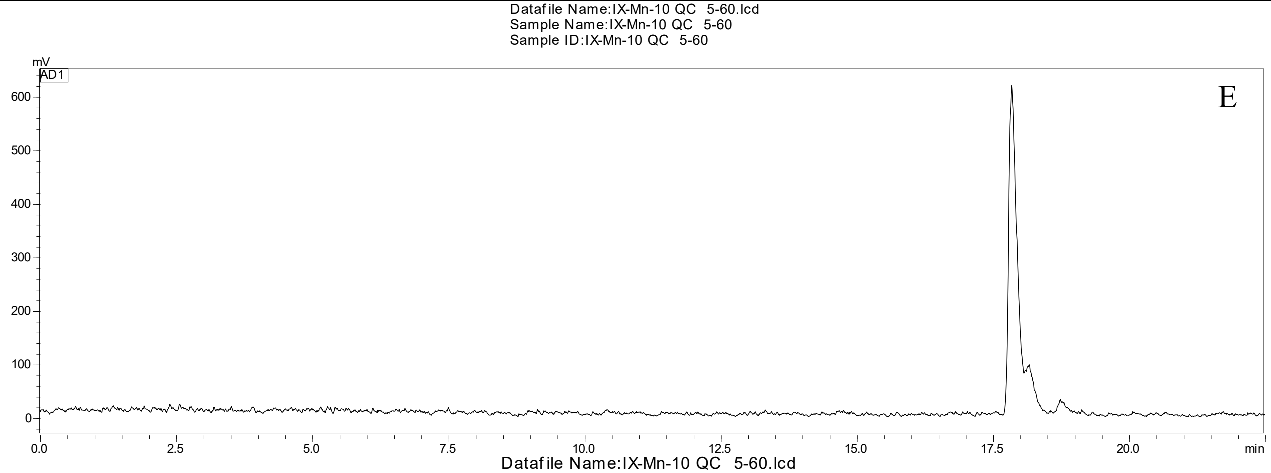

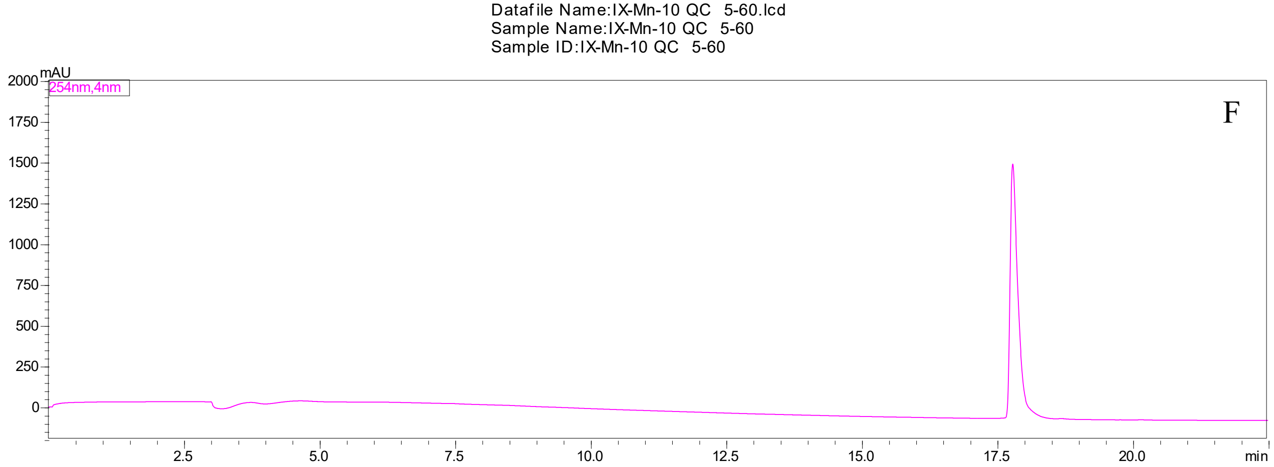

Figure S21: Radio-HPLC analysis of reaction mixture from **S14**. Reaction mixture with HPLC method 1 (A), authentic **[^{19}F]14** with HPLC method 1 (B), reaction mixture with HPLC method 2 (C), authentic **[^{19}F]14** with HPLC method 2 (D), and QC for **[^{18}F]14** (E and F).

HPLC Conditions for QC: Column: Phenomenex, Kinetex® 5µm F5 100 Å, 250 × 4.6 mm LC Column

Solvent A: 0.1% TFA water, Solvent B: 0.1% TFA acetonitrile; Isocratic / Gradient elution: 5% Solvent B for 0 to 2 min, 5% – 60% Solvent B for 2 to 22.5 min. Flow rate: 1 mL/min.

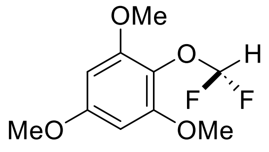

S15

Arene substrate:

2-(Difluoromethoxy)-1,3,5-trimethoxybenzene (S15)

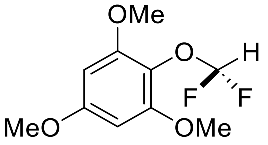

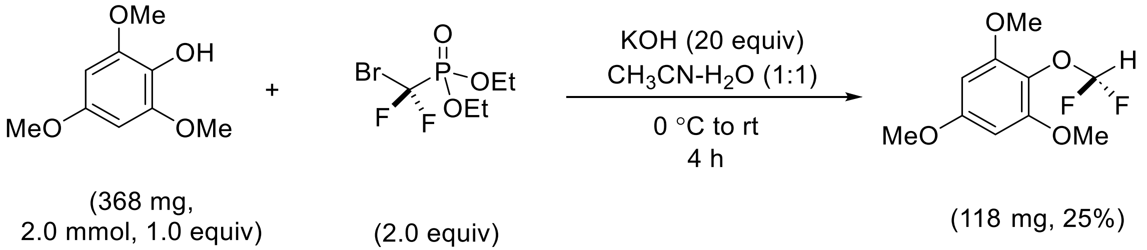

General conditions **A** were followed using 2,4,6-trimethoxyphenol (368 mg, 2.0 mmol, 1.0 equiv), $BrCF_2P(O)(OC_2H_5)_2$ (1.068 g, 4.0 mmol, 2.0 equiv), KOH (2.24 g, 40 mmol, 20 equiv), CH_3CN: H_2O (7.5 mL : 7.5 mL) for 4 h.

Purification: Gradient column chromatography [SiO_2, EtOAc:Hexanes 05:95 to 15:85)] to obtain white solid of **S15** (118 mg, 25%).

R_f : 0.7 (EtOAc : Hexanes 3:7)

^{1}H NMR ($CDCl_3$, 400 MHz): δ 6.44 (t, J = 76.8 Hz, 1H), 6.16 (s, 2H), 3.85 (s, 6H), 3.79 (s, 3H).

^{13}C NMR ($CDCl_3$, 101 MHz): δ 158.27, 153.62, 123.43 (t, J= 2.9 Hz), 117.15 (t, J= 259.7 Hz), 91.56, 56.26, 55.58.

^{19}F NMR ($CDCl_3$, 376 MHz): δ –81.70 (d, J = 76.3 Hz).

HRMS (ESI-TOF) m/z: $[M + H]^+$ Calcd. for $C_{10}H_{13}F_2O_4$ 235.0782; found 235.0779.

Authentic fluoroarene standard

Data are comparable to that reported in literature.[7]

Radio-HPLC analysis and characterization

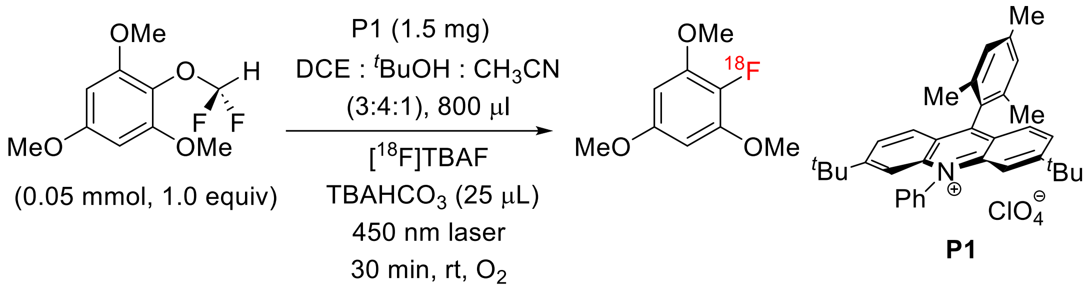

Entry	Activity at starting	Activity at EOS	Alumina Filtration	Injected Dose	Collected Dose	Isolation Time	Decay Corrected	Filtration Yield	HPLC Purity of Filtrate	%Yield
1	9.51 mCi	7.95 mCi	2.45 mCi	284 µCi	17 µCi	13.1 min	261.34 µCi	30.81%	6.50%	2.00%
2*	7.07 mCi	5.95 mCi	1.49 mCi	480 µCi	11 µCi	13.1 min	441.70 µCi	25.05%	2.37%	0.59%
										**Average %Yield: 1.3% (n=2)**

Table S20: HPLC isolated RCYs for **[^{18}F]15**

HPLC Conditions – method 1: Column: Phenomenex, Kinetex® 5µm F5 100 Å, 250 ✕ 4.6 mm LC Column

Solvent A: 0.1% TFA water, Solvent B: 0.1% TFA acetonitrile; Isocratic / Gradient elution: 20% Solvent B for 0 to 2 min, 20% – 95% Solvent B for 2 to 22.5 min. Flow rate: 1 mL/min

*N_2 sparging instead of O_2

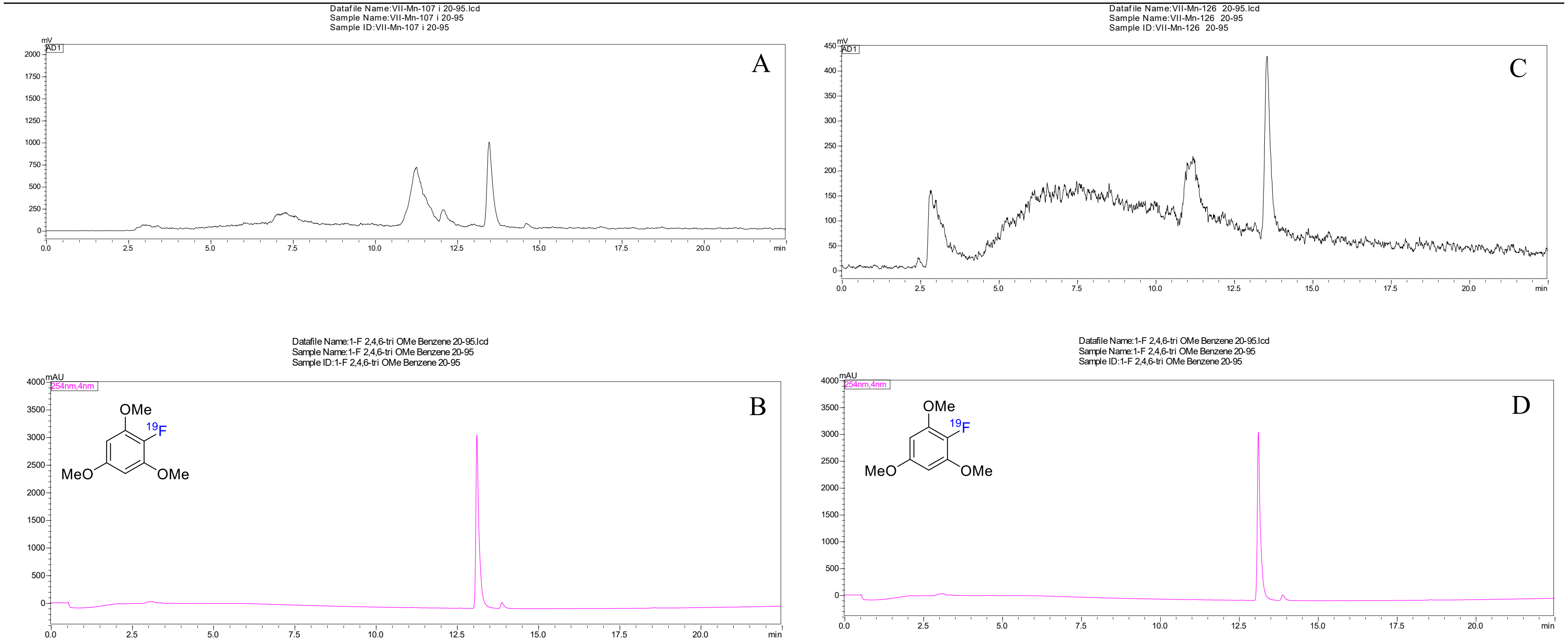

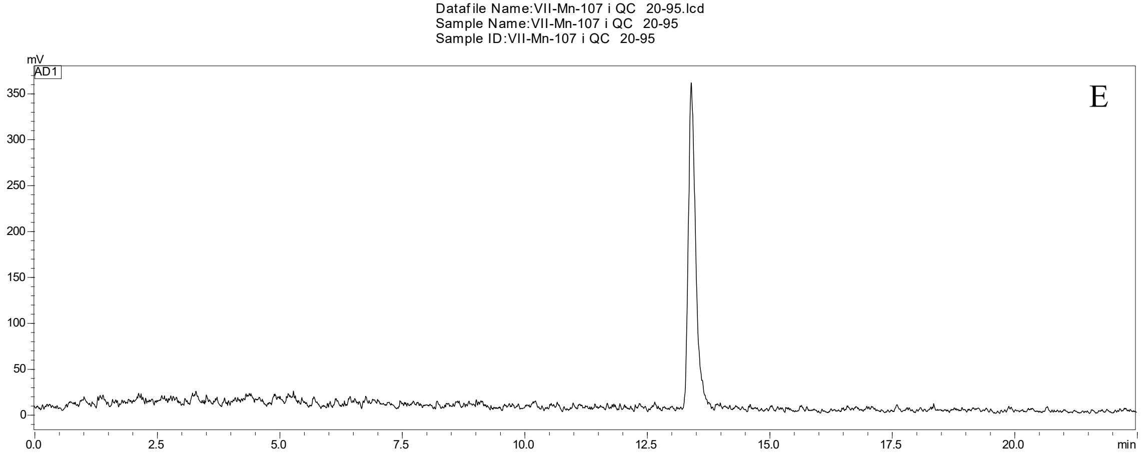

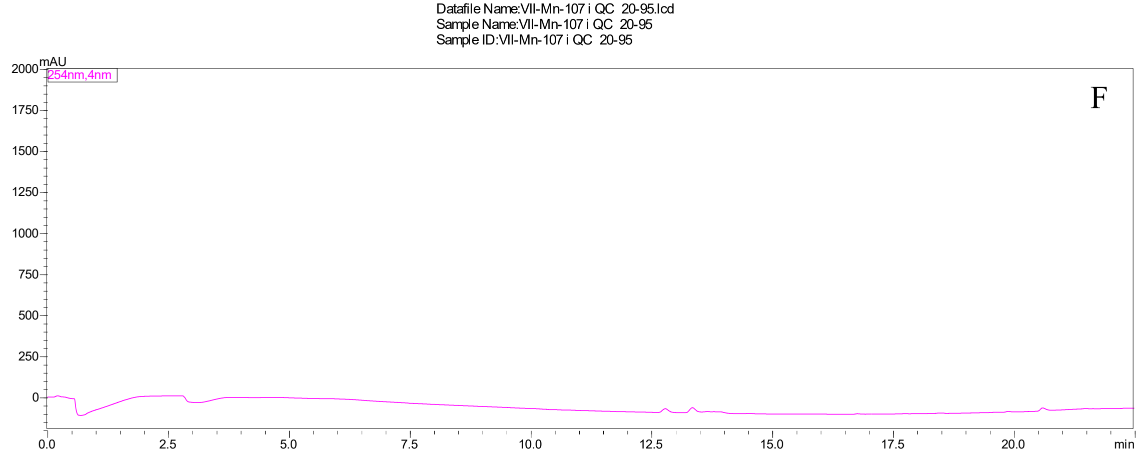

Figure S22: Radio-HPLC analysis of reaction mixture from **S15**. Reaction mixture with HPLC method 1 (A), authentic **[^{19}F]15** with HPLC method 1 (B), reaction mixture with HPLC method 1 (C), authentic **[^{19}F]15** with HPLC method 1 (D), and QC for **[^{18}F]15** (E and F).

HPLC Conditions for QC: Column: Phenomenex, Kinetex® 5µm F5 100 Å, 250 × 4.6 mm LC Column

Solvent A: 0.1% TFA water, Solvent B: 0.1% TFA acetonitrile; Isocratic / Gradient elution: 20% Solvent B for 0 to 2 min, 20% – 95% Solvent B for 2 to 22.5 min. Flow rate: 1 mL/min.

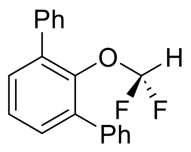

S16

Arene substrate:

2'-(Difluoromethoxy)-1,1':3',1''-terphenyl (S16)

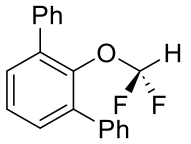

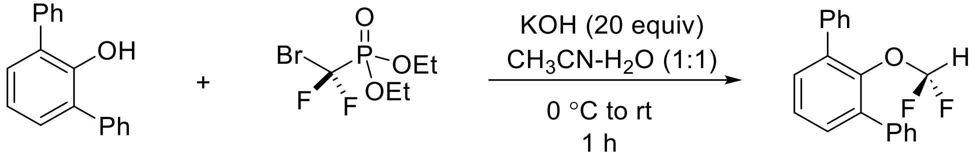

General conditions **A** were followed using [1,1':3',1''-terphenyl]-2'-ol (492 mg, 2.0 mmol, 1.0 equiv), $BrCF_2P(O)(OC_2H_5)_2$ (1.068 g, 4.0 mmol, 2.0 equiv), KOH (2.24 g, 40 mmol, 20 equiv), CH_3CN : H_2O (5 mL : 5 mL) for 1 h.

Purification: Gradient column chromatography [SiO_2, EtOAc:Hexanes 00:100 to 10:90)] to obtain white solid of **S16** (248 mg, 42%).

R_f : 0.8 (EtOAc : Hexanes 0.5:9.5)

^{1}H NMR ($CDCl_3$, 400 MHz): δ 7.57 (dd, J = 8.3, 1.2 Hz, 4H), 7.47–7.43 (m, 4H), 7.41–7.37 (m, 5H), 5.78 (t, J = 75.3 Hz, 1H).

^{13}C NMR ($CDCl_3$, 101 MHz): δ 145.33 (t, J = 2.9 Hz), 137.54, 136.77, 130.77, 129.70, 128.49, 127.85, 126.88, 116.59 (t, J = 260.6 Hz).

^{19}F NMR (CDCl$_3$, 376 MHz): δ –82.23 (d, J = 76.7 Hz).

HRMS (ESI-TOF) m/z: $[M + Na]^+$ Calcd. for $C_{19}H_{14}F_2NaO$ 319.0910; found 319.0910.

<u>Deoxyfluorination and Authentic fluoroarene standard</u>

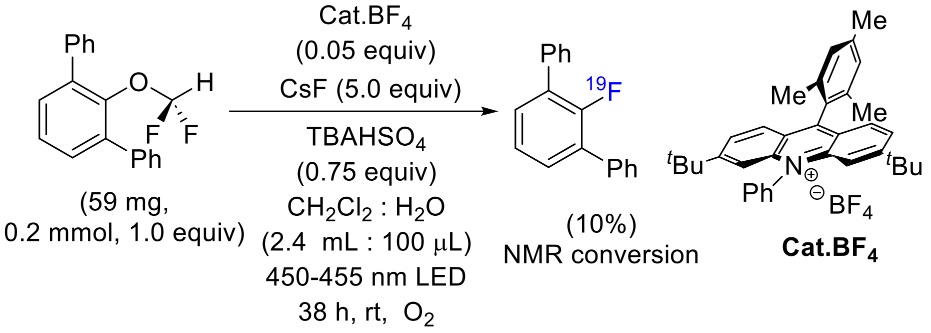

General conditions **I** were followed using 2'-(difluoromethoxy)-1,1':3',1"-terphenyl **S16** (59 mg, 0.2 mmol, 1.0 equiv), Mes-Acr-Ph$^+$BF$_4^-$ (0.05 equiv), CsF (5.0 equiv), TBAHSO$_4$ (0.75 equiv), CH_2Cl_2 : H_2O (0.1 M, 25:1), 450-455 nm LED, 38 h, 33 °C, O_2 balloon.

^{19}F NMR (CDCl$_3$, 376 MHz): δ –123.35 (ddd, J = 13.7, 8.3, 4.2 Hz).

HRMS (ESI-TOF) m/z: $[M + Na]^+$ Calcd. for $C_{18}H_{13}FNa$ 271.0899; found 271.0896.

Data are comparable to that commercially available compound. CAS Number 1678501-58-0

Radio-HPLC analysis and characterization

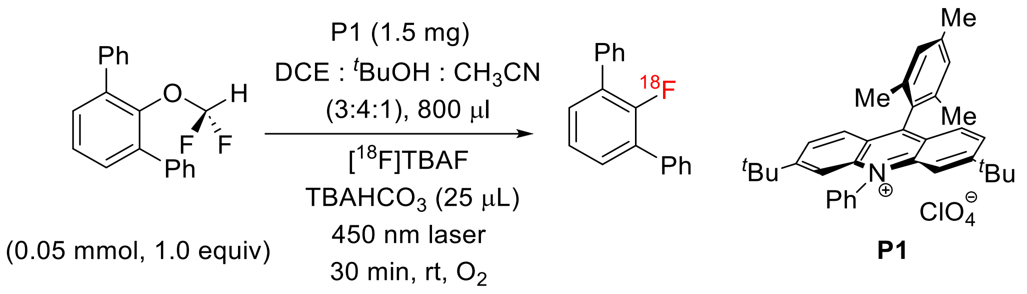

Entry	Activity at starting	Activity at EOS	Alumina Filtration	Injected Dose	Collected Dose	Isolation Time	Decay Corrected	Filtration Yield	HPLC Purity of Filtrate	%Yield
1	10.02 mCi	8.12 mCi	2.42 mCi	388 µCi	106 µCi	18.3 min	346.20 µCi	29.80%	30.61%	9.12%
2	12.52 mCi	9.11 mCi	2.23 mCi	215 µCi	45 µCi	18.3 min	191.85 µCi	24.47%	20.93%	5.12%
								**Average %Yield: 7.1% (n=2)**		

Table S21: HPLC isolated RCYs for **[^{18}F]16**

HPLC Conditions – method 1: Column: Phenomenex, Kinetex® 5µm F5 100 Å, 250 ✕ 4.6 mm LC Column

Solvent A: 0.1% TFA water, Solvent B: 0.1% TFA acetonitrile; Isocratic / Gradient elution: 30% Solvent B for 0 to 2 min, 30% – 95% Solvent B for 2 to 22.5 min. Flow rate: 1 mL/min

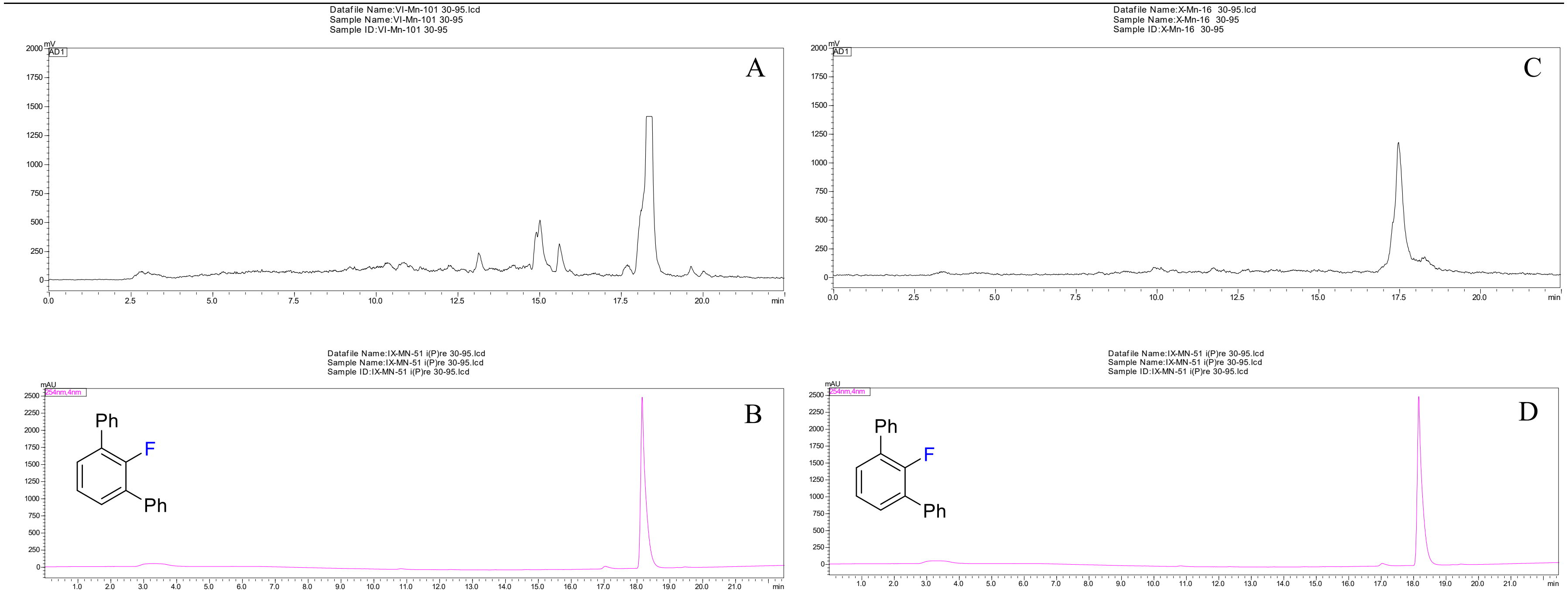
Datafile Name:VI-Mn-101 30-95.lcd
Sample Name:VI-Mn-101 30-95
Sample ID:VI-Mn-101 30-95
A
Datafile Name:X-Mn-16 30-95.lcd
Sample Name:X-Mn-16 30-95
Sample ID:X-Mn-16 30-95
C
Datafile Name:IX-MN-51 i(P)re 30-95.lcd
Sample Name:IX-MN-51 i(P)re 30-95.lcd
Sample ID:IX-MN-51 i(P)re 30-95.lcd
B
Ph
F
Ph
Datafile Name:IX-MN-51 i(P)re 30-95.lcd
Sample Name:IX-MN-51 i(P)re 30-95.lcd
Sample ID:IX-MN-51 i(P)re 30-95.lcd
D
Ph
F
Ph

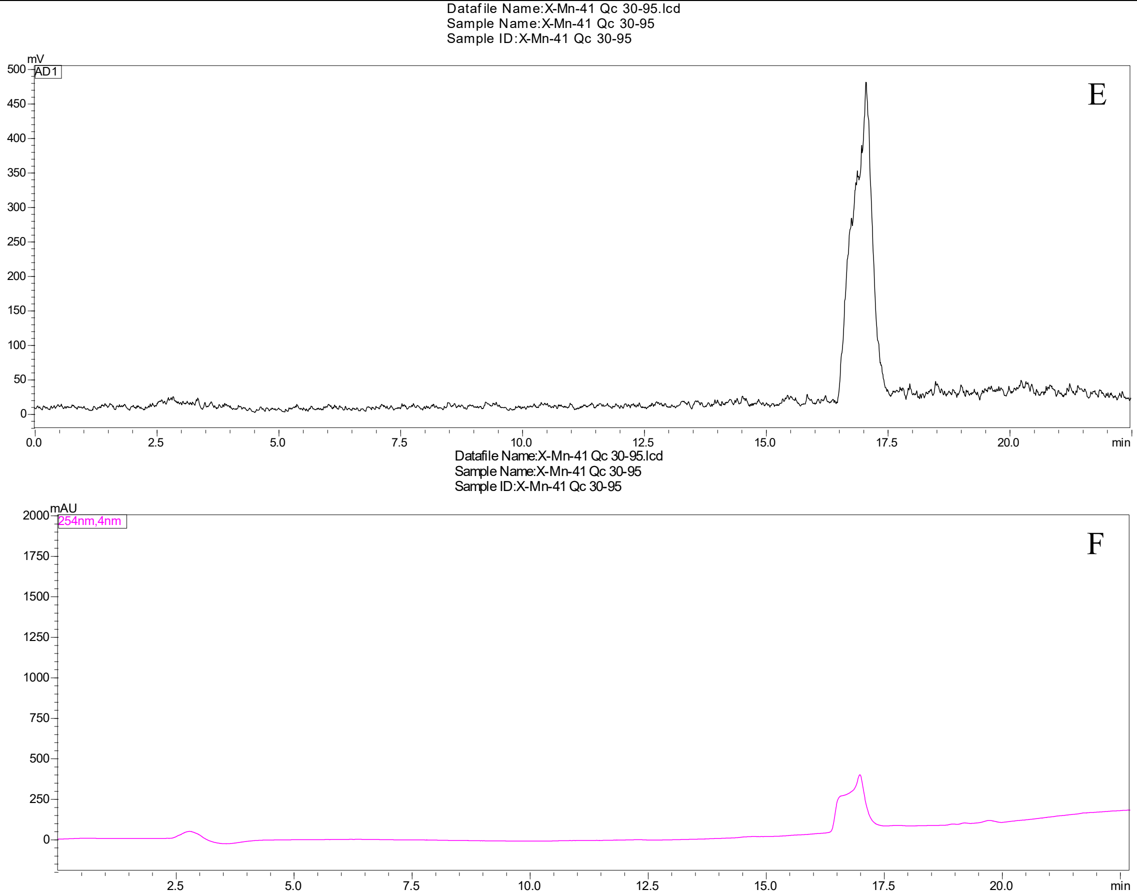

Figure S23: Radio-HPLC analysis of reaction mixture from **S16**. Reaction mixture with HPLC method 1 (A), authentic **[^{19}F]16** with HPLC method 1 (B), reaction mixture with HPLC method 1 (C), authentic **[^{19}F]16** with HPLC method 1 (D), and QC for **[^{18}F]16** (E and F).

HPLC Conditions for QC: Column: Phenomenex, Kinetex® 5µm F5 100 Å, 250 × 4.6 mm LC Column

Solvent A: 0.1% TFA water, Solvent B: 0.1% TFA acetonitrile; Isocratic / Gradient elution: 30% Solvent B for 0 to 2 min, 30% – 95% Solvent B for 2 to 22.5 min. Flow rate: 1 mL/min.

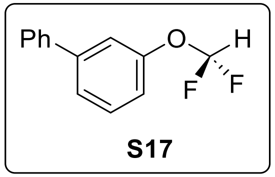

Arene substrate:

3-(Difluoromethoxy)-1,1'-biphenyl (**S17)**

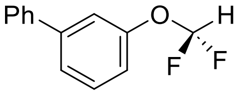

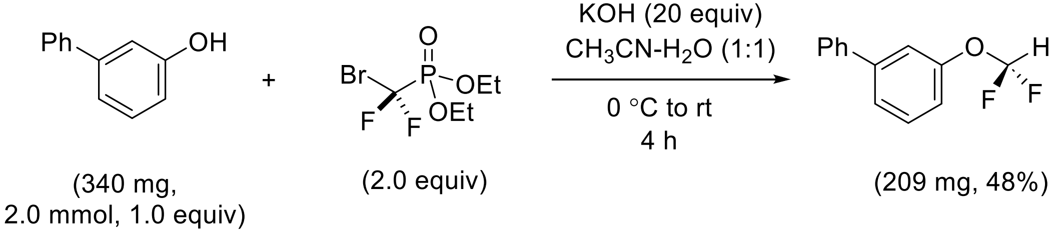

General conditions **A** were followed using [1,1'-biphenyl]-3-ol (340 mg, 2.0 mmol, 1.0 equiv), $BrCF_2P(O)(OC_2H_5)_2$ (1.068 g, 4.0 mmol, 2.0 equiv), KOH (2.24 g, 40 mmol, 20 equiv), CH_3CN: H_2O (5 mL : 5 mL) for 4 h.

Purification: Gradient column chromatography [SiO_2, EtOAc:Hexanes 00:100 to 10:90)] to obtain colourless liquid of **S17** (211 mg, 48%).

R_f : 0.8 (EtOAc : Hexanes 0.5:9.5)

^{1}H NMR ($CDCl_3$, 400 MHz): δ 7.59–7.57 (m, 2H), 7.47-7.42 (m, 4H), 7.38 (td, J = 7.4, 1.3 Hz, 1H), 7.35-7.34 (m, 1H), 7.11 (dt, J = 7.4, 1.4 Hz, 1H), 6.57 (t, J= 73.9 Hz, 1H).

^{13}C NMR ($CDCl_3$, 101 MHz): δ 151.81 (t, J = 2.8 Hz), 143.48, 140.12, 130.25, 129.05, 128.03, 127.29, 124.34, 118.53, 118.29, 116.16 (t, J = 259.5 Hz).

^{19}F NMR (CDCl$_3$, 376 MHz): δ –80.48 (d, J = 74.6 Hz).

HRMS (ESI-TOF) m/z: [M + Na]$^+$ Calcd. for $C_{13}H_{10}F_2NaO$ 243.0597; found 243.0594.

Authentic fluoroarene standard

^{19}F-Reference compound – **[^{19}F]17** was purchased from the commercial supplier. CAS Number 367-22-8

Radio-HPLC analysis and characterization

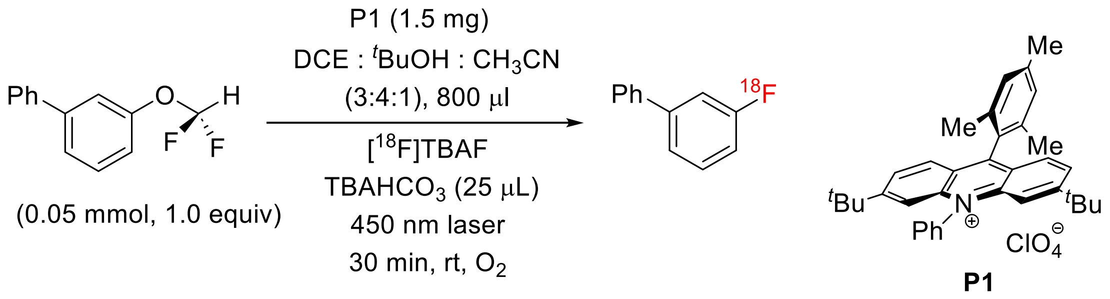

Entry	Activity at starting	Activity at EOS	Alumina Filtration	Injected Dose	Collected Dose	Isolation Time	Decay Corrected	Filtration Yield	HPLC Purity of Filtrate	%Yield
1*	10.64 mCi	8.31 mCi	2.52 mCi	279 μCi	30 μCi	16.5 min	250.60 μCi	30.32%	11.97%	3.62%
2**	12.54 mCi	9.42 mCi	–	718 μCi	19 μCi	14.5 min	653.80 μCi	–	–	2.90%
										**Average %Yield: 3.3% (n=2)**

Table S22: HPLC isolated RCYs for **[^{18}F]17**

*HPLC Conditions – method 1: Column: Phenomenex, Kinetex® 5μm F5 100 Å, 250 ✕ 4.6 mm LC Column

Solvent A: 0.1% TFA water, Solvent B: 0.1% TFA acetonitrile; Isocratic / Gradient elution: 20% Solvent B for 0 to 2 min, 20% – 95% Solvent B for 2 to 22.5 min. Flow rate: 1 mL/min

** HPLC Conditions – method 2: Column: Phenomenex, Gemini® 5µm F5 100 Å, 250 ✕ 4.6 mm LC Column

Solvent A: 0.1% TFA water, Solvent B: 0.1% TFA acetonitrile; Isocratic / Gradient elution: 55% Solvent B for 0 to 2 min, 55% – 95% Solvent B for 2 to 22.5 min. Flow rate: 1 mL/min

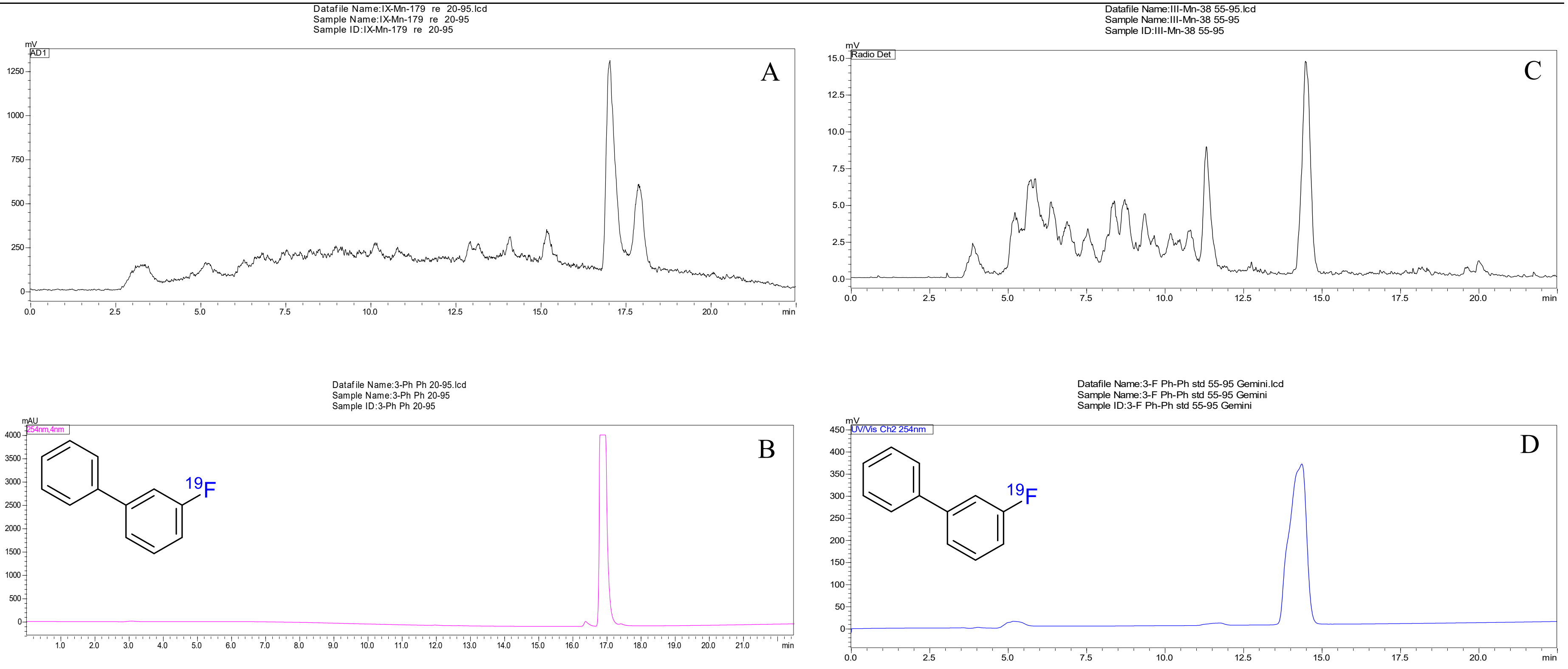

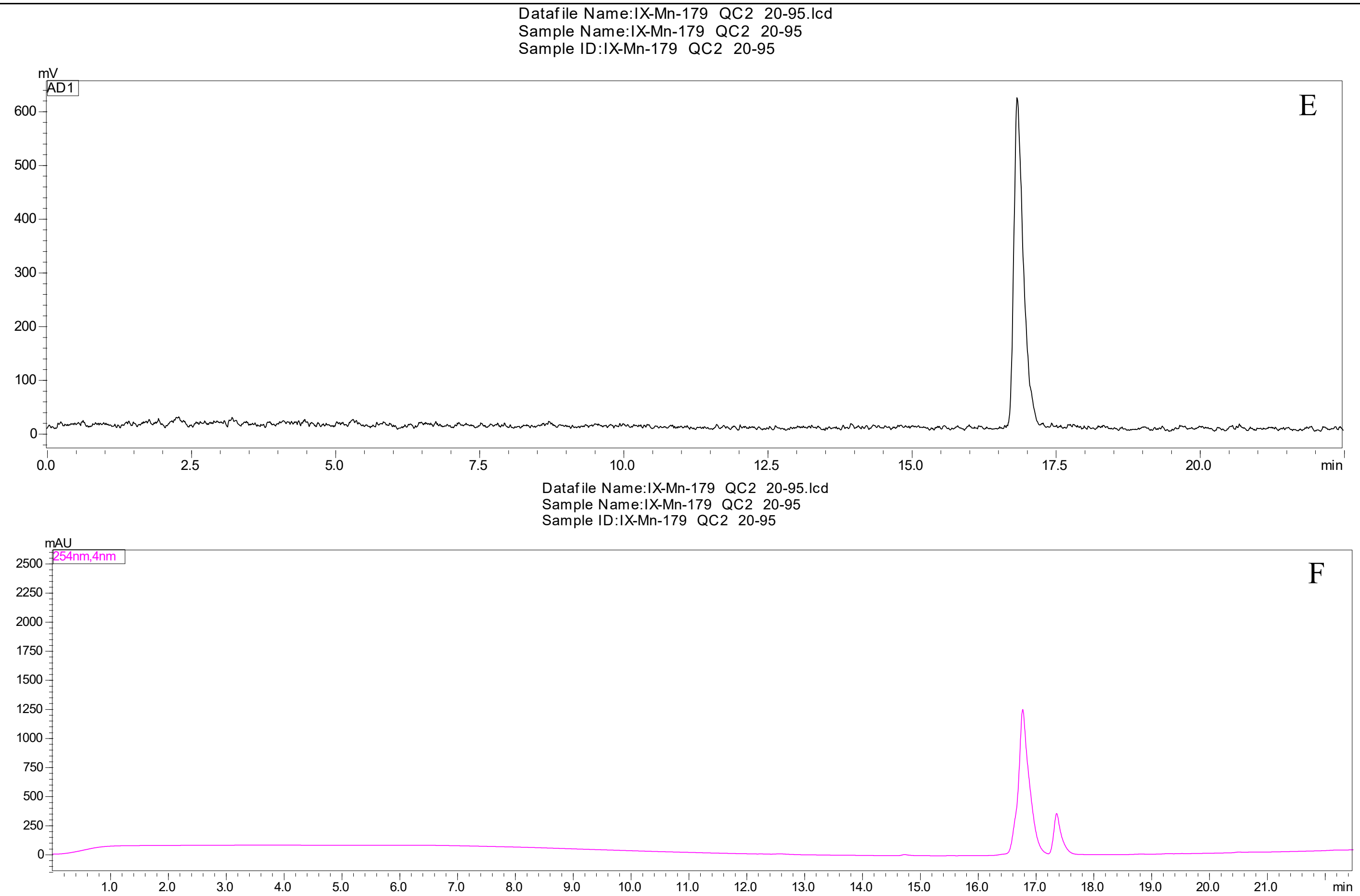

Figure S24: Radio-HPLC analysis of reaction mixture from **S17**. Reaction mixture with HPLC method 1 (A), authentic **[^{19}F]17** with HPLC method 1 (B), reaction mixture with HPLC method 2 (C), authentic **[^{19}F]17** with HPLC method 2 (D), and QC for **[^{18}F]17** (E and F).

HPLC Conditions for QC: Column: Phenomenex, Kinetex® 5µm F5 100 Å, 250 × 4.6 mm LC Column

Solvent A: 0.1% TFA water, Solvent B: 0.1% TFA acetonitrile; Isocratic / Gradient elution: 20% Solvent B for 0 to 2 min, 20% – 95% Solvent B for 2 to 22.5 min. Flow rate: 1 mL/min.

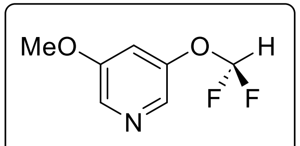

S18

Arene substrate:

3-(Difluoromethoxy)-5-methoxypyridine (S18)

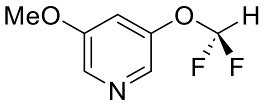

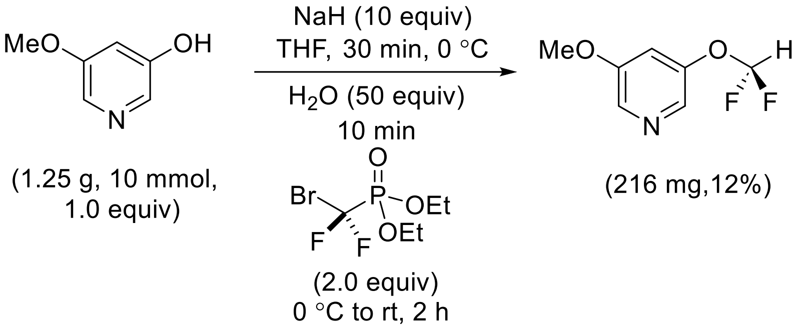

General conditions **B** were followed using 5-methoxypyridin-3-ol (1.25 g, 10.0 mmol, 1.0 equiv), $BrCF_2P(O)(OC_2H_5)_2$ (5.3 g, 20.0 mmol, 2.0 equiv), NaH(90%) (2.6 g, 100 mmol, 10 equiv), H_2O (9.0 mL 500 mmol), THF (20 mL) for 2 h.

Purification: Isocratic column chromatography [SiO_2, EtOAc:Hexanes 30:70)] to obtain colourless liquid of **S18** (216 mg, 12%).

R_f : 0.3 (EtOAc : Hexanes 3:7)

^{1}H NMR ($CDCl_3$, 400 MHz): δ 8.19 (d, J = 2.5 Hz, 1H), 8.09 (d, J = 2.1 Hz, 1H), 7.00 (t, J = 2.4 Hz, 1H), 6.53 (t, J = 72.8 Hz, 1H), 3.86 (s, 3H).

^{13}C NMR ($CDCl_3$, 101 MHz): δ 156.51, 148.08 (t, J = 2.9 Hz), 134.62, 133.55, 115.49 (t, J = 263.3 Hz), 56.04.

^{19}F NMR ($CDCl_3$, 376 MHz): δ –81.36 (d, J = 72.6 Hz).

HRMS (ESI-TOF) m/z: $[M + Na]^+$ Calcd. for $C_7H_8F_2NO_2$ 176.0523; found 176.0525.

Authentic fluoroarene standard

^{19}F-Reference compound – **[^{19}F]18** was purchased from the commercial supplier. CAS Number 1060801-62-8

Radio-HPLC analysis and characterization

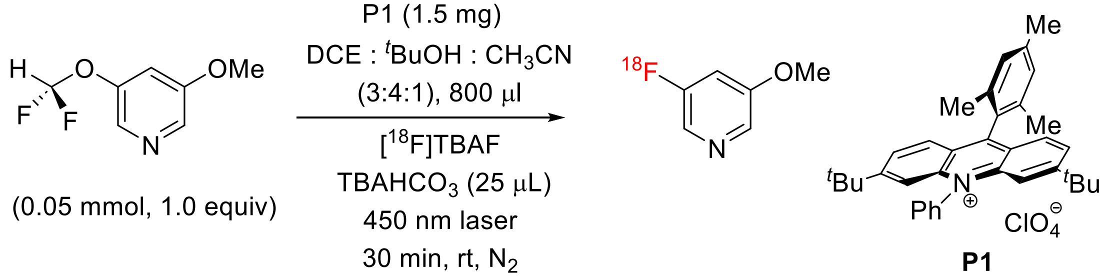

Entry	Activity at starting	Activity at EOS	Alumina Filtration	Injected Dose	Collected Dose	Isolation Time	Decay Corrected	Filtration Yield	HPLC Purity of Filtrate	%Yield
1	11.35 mCi	8.04 mCi	2.54 mCi	362 µCi	34 µCi	10.5 min	339.66 µCi	31.59%	10.01%	3.16%

Supplementary **Table S23**: HPLC isolated RCYs for **[^{18}F]18**

HPLC Conditions - method 1: Column: Phenomenex, Kinetex® 5µm F5 100 Å, 250 × 4.6 mm LC Column

Solvent A: 0.1% TFA water, Solvent B: 0.1% TFA acetonitrile; Isocratic / Gradient elution: 5% Solvent B for 0 to 2 min, 5% – 95% Solvent B for 2 to 22.5 min. Flow rate: 1 mL/min

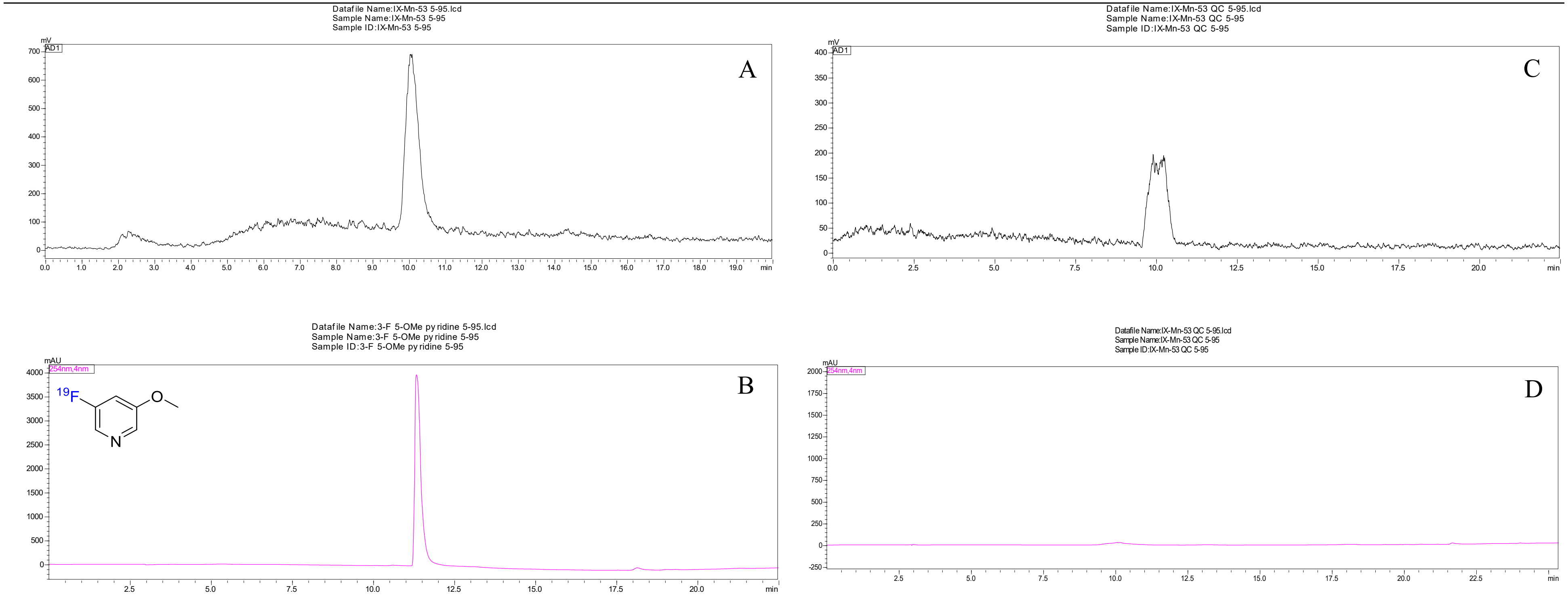

Figure S25: Radio-HPLC analysis of reaction mixture from **S18**. Reaction mixture with HPLC method 1 (A), authentic **[^{19}F]18** with HPLC method 1 (B), and QC for **[^{18}F]18** (C and D).

HPLC Conditions for QC: Column: Phenomenex, Kinetex® 5µm F5 100 Å, 250 × 4.6 mm LC Column

Solvent A: 0.1% TFA water, Solvent B: 0.1% TFA acetonitrile; Isocratic / Gradient elution: 5% Solvent B for 0 to 2 min, 5% – 95% Solvent B for 2 to 22.5 min. Flow rate: 1 mL/min.

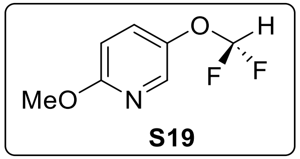

Arene substrate:

5-(Difluoromethoxy)-2-methoxypyridine (S19)

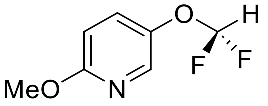

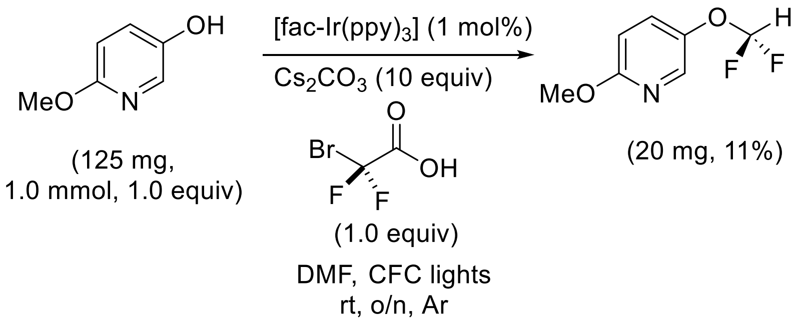

General conditions **C** were followed using 6-methoxypyridin-3-ol Phenol (125 mg, 0.1 mmol, 1.0 equiv), [fac-Ir(ppy)$_3$] (7 mg, 0.01 mmol, 1 mol%), and Cs_2CO_3 (978 mg, 3.0 mmol, 10 equiv). The Schlenk tube was purged and degassed with argon three times. Difluorobromoacetic acid (175 mg, 1.0 mmol, 1.0 equiv) in degassed anhydrous DMF.

Purification: Gradient column chromatography [SiO_2, EtOAc:Hexanes 00:100 to 5:95)] to obtain colourless liquid of **S19** (20 mg, 11%).

R_f : 0.7 (EtOAc : Hexanes 1:9)

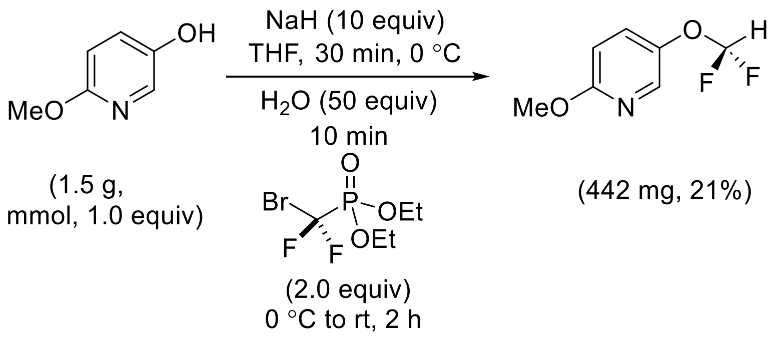

General conditions **B** were followed using 6-methoxypyridin-3-ol (1.5 g, 12.0 mmol, 1.0 equiv), $BrCF_2P(O)(OC_2H_5)_2$ (6.4 g, 24.0 mmol, 2.0 equiv), NaH(90%) (3.2 g, 120 mmol, 10 equiv), H_2O (10.8 mL, 600 mmol), and THF (20 mL) for 2 h.

Purification: Gradient column chromatography [SiO_2, EtOAc:Hexanes 00:100 to 5:95)] to obtain colourless liquid of **S19** (442 mg, 21%).

R_f : 0.7 (EtOAc : Hexanes 1:9)

^{1}H NMR ($CDCl_3$, 400 MHz): δ 8.01(d, J = 2.5 Hz, 1H), 7.39 (d, J = 8.6 Hz, 1H), 6.72 (d, J = 8.9 Hz, 1H), 6.43 (t, J= 73.4 Hz, 1H), 3.91 (s, 3H).

^{13}C NMR ($CDCl_3$, 101 MHz): δ 161.88, 141.96 (t, J= 2.9 Hz), 139.07, 132.26, 115.76 (t, J= 262.6 Hz), 111.54, 53.88.

^{19}F NMR ($CDCl_3$, 376 MHz): δ –81.16 (dd, J = 72.1, 4.1 Hz).

HRMS (ESI-TOF) m/z: $[M + Na]^+$ Calcd. for $C_7H_8F_2NO_2$ 176.0523; found 176.0520.

<u>Authentic fluoroarene standard</u>

^{19}F-Reference compound – **[^{19}F]19** was purchased from the commercial supplier. CAS Number 51173-04-7

Radio-HPLC analysis and characterization

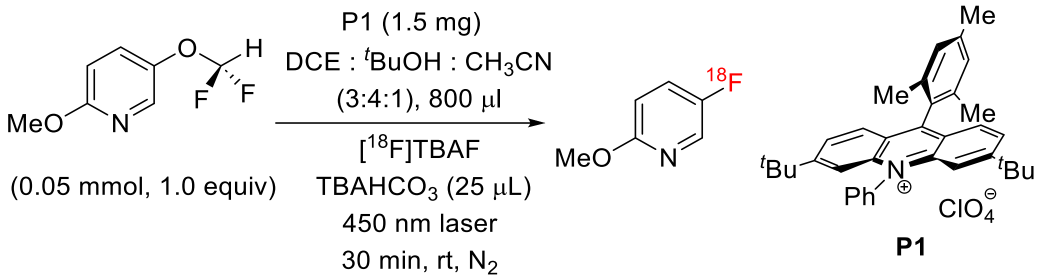

Entry	Activity at starting	Activity at EOS	Alumina Filtration	Injected Dose	Collected Dose	Isolation Time	Decay Corrected	Filtration Yield	HPLC Purity of Filtrate	%Yield
1	13.83 mCi	10.20 mCi	3.96 mCi	671.2 µCi	139 µCi	11.2 min	626.02 µCi	38.82%	22.20%	8.61%
2	19.20 mCi	15.42 mCi	4.87 mCi	671.0 µCi	72 µCi	11.2 min	625.84 µCi	31.58 %	11.50 %	3.63%
3	10.65 mCi	8.13 mCi	3.32 mCi	625 µCi	132 µCi	11.2 min	576.34 µCi	40.88%	22.90%	9.36%
								**Average %Yield: 7.2% (n=3)**		

Table S24: HPLC isolated RCYs for **[^{18}F]19**

HPLC Conditions – method 1: Column: Phenomenex, Kinetex® 5µm F5 100 Å, 250 ✕ 4.6 mm LC Column

Solvent A: 0.1% TFA water, Solvent B: 0.1% TFA acetonitrile; Isocratic / Gradient elution: 5% Solvent B for 0 to 2 min, 5% – 95% Solvent B for 2 to 22.5 min. Flow rate: 1 mL/min

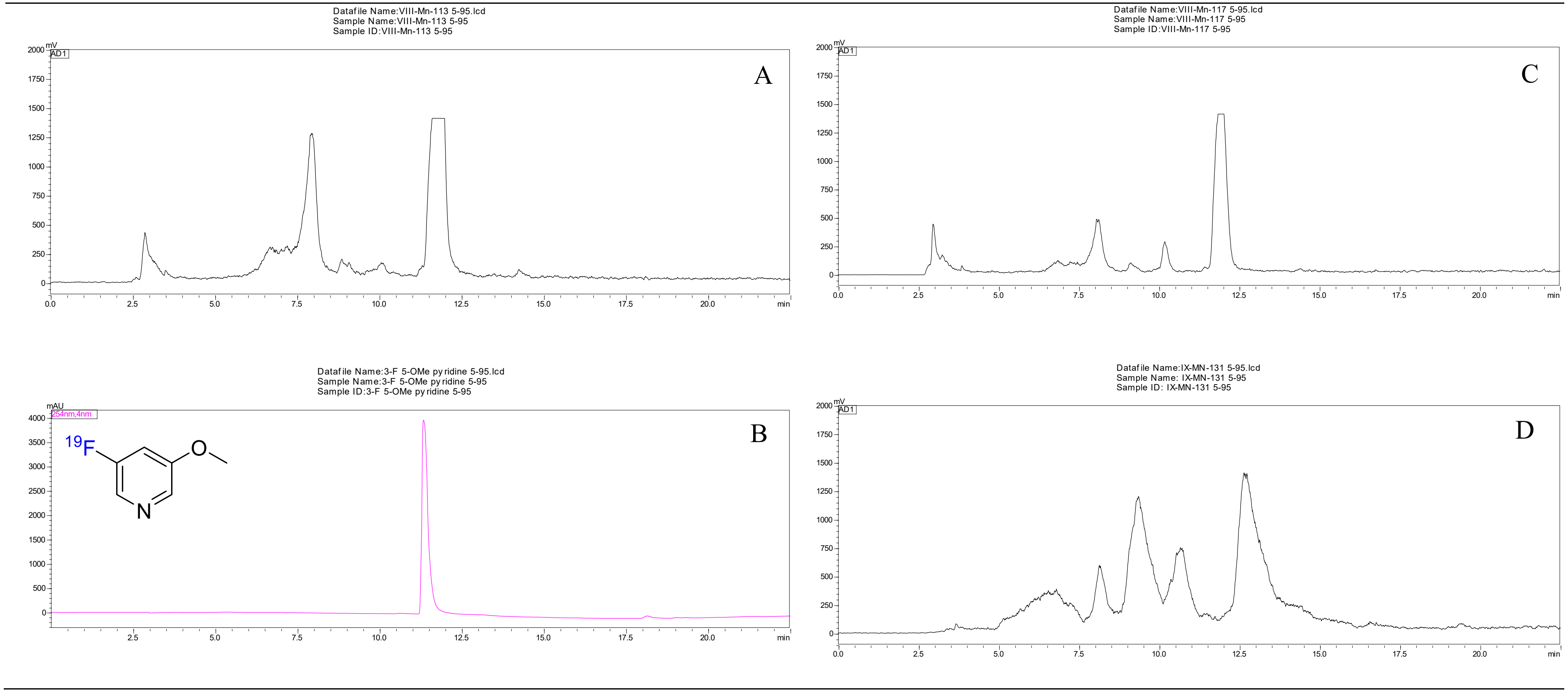
Datafile Name:VIII-Mn-113 5-95.lcd
Sample Name:VIII-Mn-113 5-95
Sample ID:VIII-Mn-113 5-95
A
Datafile Name:VIII-MN-117 5-95.lcd
Sample Name:VIII-MN-117 5-95
Sample ID:VIII-MN-117 5-95
C
Datafile Name:3-F 5-OMe pyridine 5-95.lcd
Sample Name:3-F 5-OMe pyridine 5-95
Sample ID:3-F 5-OMe pyridine 5-95
B
19F
N
O
Datafile Name:IX-MN-131 5-95.lcd
Sample Name: IX-MN-131 5-95
Sample ID: IX-MN-131 5-95
D
min

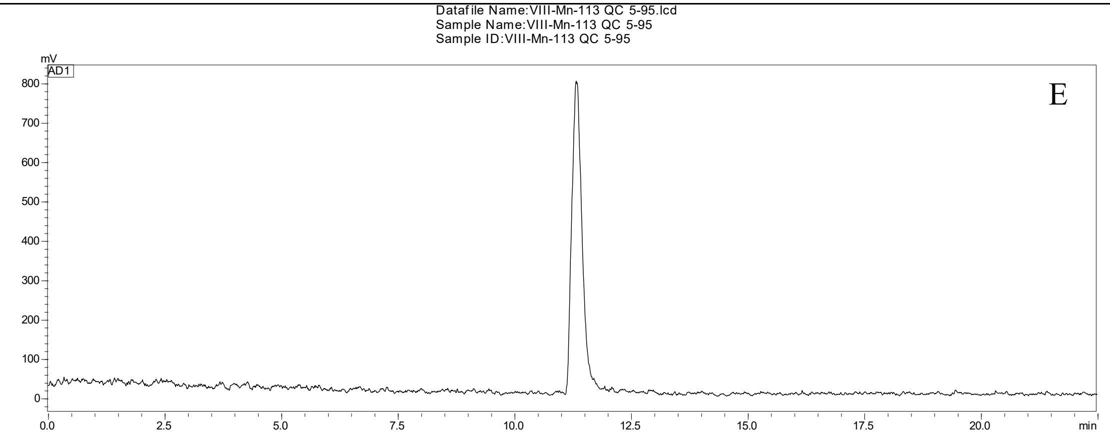

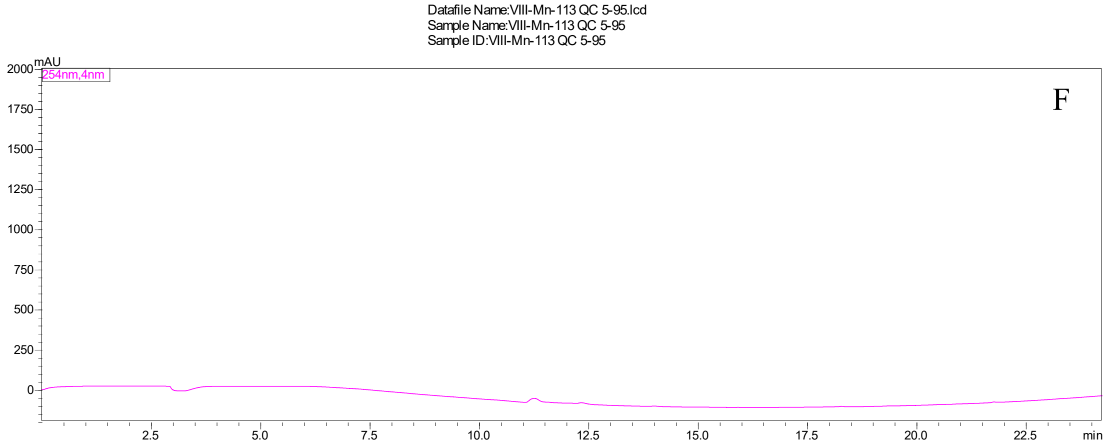

Figure S26: Radio-HPLC analysis of reaction mixture from **S19**. Reaction mixture with HPLC method 1 (A), authentic **[^{19}F]19** with HPLC method 1 (B), reaction mixture with HPLC method 1 (C), reaction mixture with HPLC method 1 (D), and QC for **[^{18}F]19** (E and F).

HPLC Conditions for QC: Column: Phenomenex, Kinetex® 5µm F5 100 Å, 250 × 4.6 mm LC Column

Solvent A: 0.1% TFA water, Solvent B: 0.1% TFA acetonitrile; Isocratic / Gradient elution: 5% Solvent B for 0 to 2 min, 5% – 95% Solvent B for 2 to 22.5 min. Flow rate: 1 mL/min.

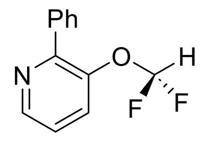

S20

Arene substrate:

3-(Difluoromethoxy)-2-phenylpyridine (**S20)**

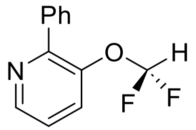

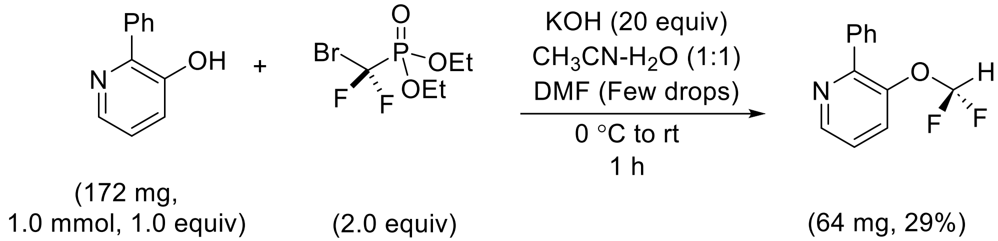

General conditions **A** were followed using 2-phenylpyridin-3-ol (172 mg, 1.00 mmol, 1.0 equiv), $BrCF_2P(O)(OC_2H_5)_2$ (534 mg, 2.0 mmol, 2.0 equiv), KOH (1.12 g, 20 mmol, 20 equiv), CH_3CN: H_2O: DMF (3 mL : 3 mL : Few drops) for 1 h.

Purification: Gradient column chromatography [SiO_2, EtOAc:Hexanes 05:95 to 30:70)] to obtain off-yellow semi-solid of **S20** (64 mg, 29%).

R_f : 0.6 (EtOAc : Hexanes 3:7)

^{1}H NMR ($CDCl_3$, 400 MHz): δ 8.58 (dd, J = 4.6, 1.4 Hz, 1H), 7.86–7.83 (m, 2H), 7.60 (dq, J = 8.3, 1.2 Hz, 1H), 7.50–7.41 (m, 3H), 7.29 (dd, J = 8.3, 4.6 Hz, 1H), 6.39 (t, J = 73.6 Hz, 1H).

^{13}C NMR ($CDCl_3$, 101 MHz): δ 151.37, 146.81, 145.22 (t, J = 2.9 Hz), 136.55, 129.42, 129.14, 128.66, 128.42, 123.10, 115.87 (t, J = 262.9 Hz).

^{19}F NMR ($CDCl_3$, 376 MHz): δ –81.15 (d, J = 73.5 Hz).

HRMS (ESI-TOF) m/z: $[M + Na]^+$ Calcd. for $C_{12}H_9F_2NNaO$ 244.0550; found 244.0548.

<u>Deoxyfluorination and Authentic fluoroarene standard</u>

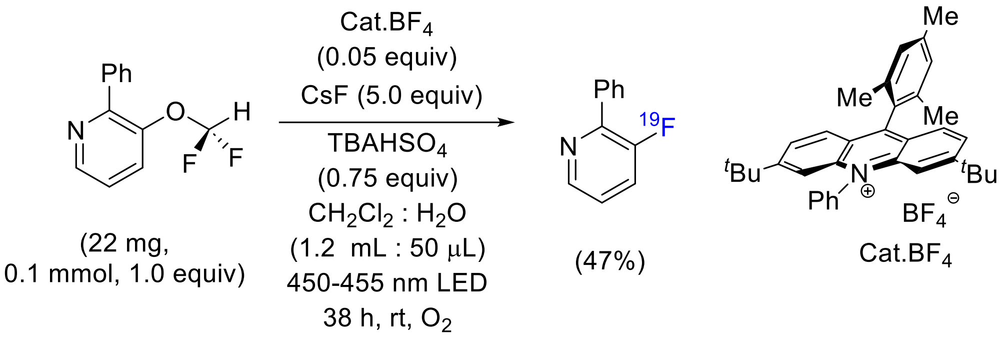

General conditions **I** were followed using 3-(difluoromethoxy)-2-phenylpyridine **S20** (22 mg, 0.05 mmol, 1.0 equiv), Mes-Acr-$Ph^+BF_4^-$ (0.05 equiv), CsF (5.0 equiv), $TBAHSO_4$ (0.75 equiv), CH_2Cl_2 : H_2O (0.1 M, 25:1), 450-455 nm LED, 38 h, 33 °C, O_2.

Purification: Gradient column chromatography [SiO_2, EtOAc:Hexanes 5:95 to 30:70)] to obtain **[^{19}F]20** (8 mg, 47%).

R_f : 0.4 (EtOAc : Hexanes 3:7)

^{1}H NMR ($CDCl_3$, 400 MHz): δ 8.53 (dt, J = 4.6, 1.5 Hz, 1H), 7.98–7.95 (m, 2H), 7.52–7.47 (m, 2H), 7.47–7.42 (m, 2H), 7.29–7.25 (m, 1H).

^{13}C NMR ($CDCl_3$, 101 MHz): δ 158.98, 156.38, 146.42 (d, J = 11.6 Hz), 145.54 (d, J = 5.1 Hz), 135.48 (d, J = 6.1 Hz), 129.35, 128.93 (d, J = 5.8 Hz), 128.59, 124.22 (d, J = 20.2 Hz), 123.57 (d, J = 3.6 Hz).

^{19}F NMR ($CDCl_3$, 376 MHz): δ –122.98 (ddd, J = 12.5, 6.9, 4.2 Hz).

Data are comparable to that commercially available compound. CAS Number 1214342-78-5

Radio-HPLC analysis and characterization

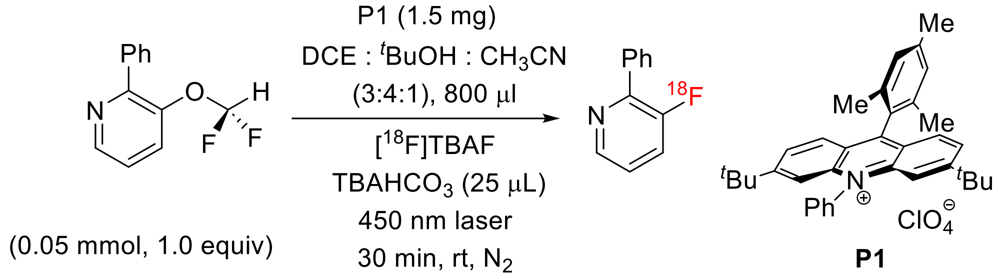

Entry	Activity at starting	Activity at EOS	Alumina Filtration	Injected Dose	Collected Dose	Isolation Time	Decay Corrected	Filtration Yield	HPLC Purity of Filtrate	%Yield
1*	7.27 mCi	5.63 mCi	2.52 mCi	546 µCi	241 µCi	11.5 min	506.68 µCi	44.76%	47.56%	21.28%
2**	5.98 mCi	4.64 mCi	1.80 mCi	285 µCi	116 µCi	13.6 min	260.88 µCi	38.83 %	44.57%	17.30%
										**Average %Yield: 19.3% (n=2)**

Table S25: HPLC isolated RCYs for **[^{18}F]20**

*HPLC Conditions – method 1: Column: Phenomenex, Kinetex® 5µm F5 100 Å, 250 ✕ 4.6 mm LC Column

Solvent A: 0.1% TFA water, Solvent B: 0.1% TFA acetonitrile; Isocratic / Gradient elution: 20% Solvent B for 0 to 2 min, 20% – 95% Solvent B for 2 to 22.5 min. Flow rate: 1 mL/min

*HPLC Conditions – method 2: Column: Phenomenex, Kinetex® 5µm F5 100 Å, 250 ✕ 4.6 mm LC Column

Solvent A: 0.1% TFA water, Solvent B: 0.1% TFA acetonitrile; Isocratic / Gradient elution: 5% Solvent B for 0 to 2 min, 5% – 95% Solvent B for 2 to 22.5 min. Flow rate: 1 mL/min

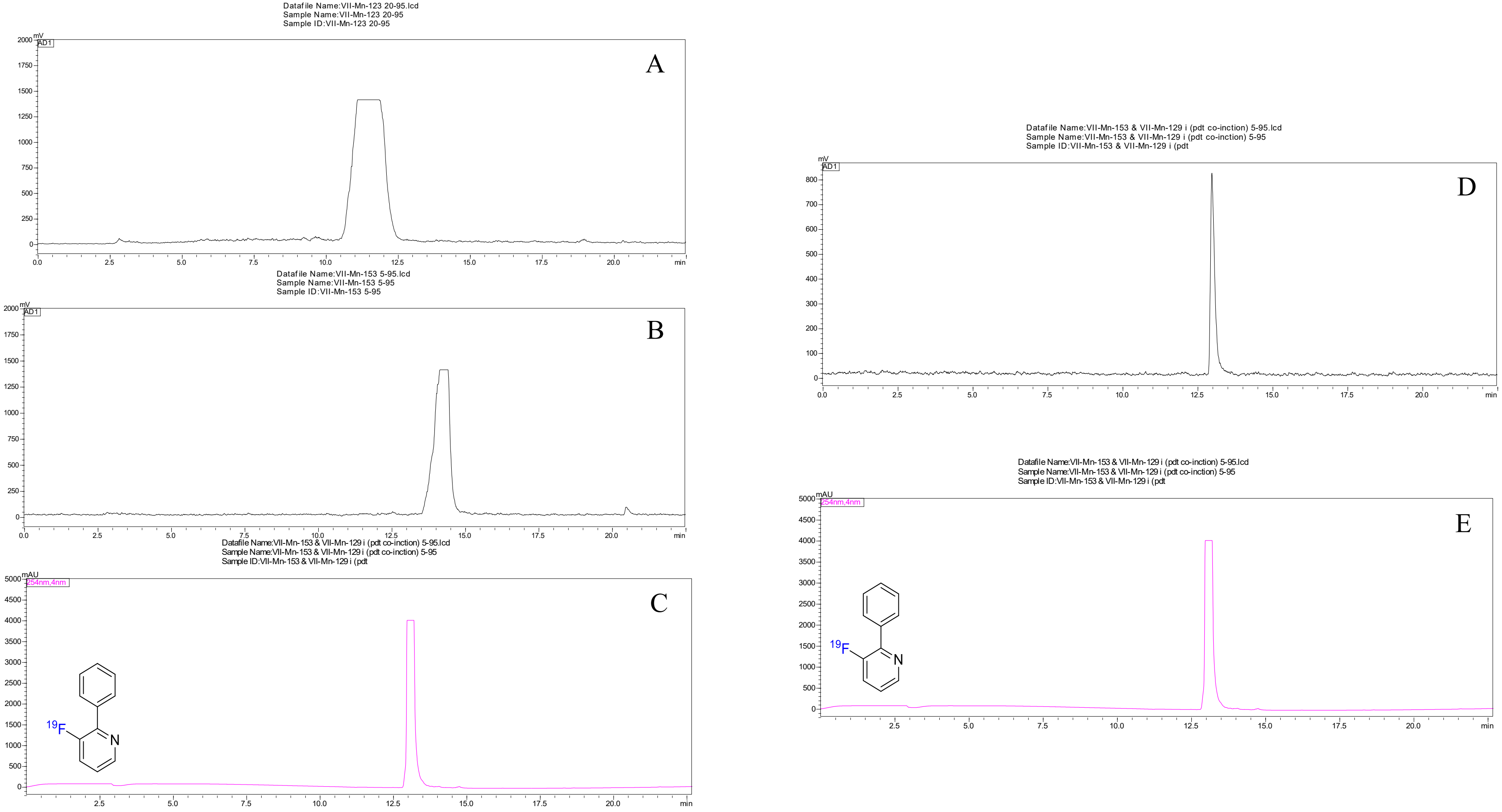

Figure S27: Radio-HPLC analysis of reaction mixture from **S20**. Reaction mixture with HPLC method 1 (A), reaction mixture with HPLC method 2 (B), authentic **[^{19}F]20** with HPLC method 1 (C), and HPLC trace of **[^{18}F]20** and **[^{19}F]20** co-injection (D and E).

HPLC Conditions for co-injection: Column: Phenomenex, Kinetex® 5µm F5 100 Å, 250 × 4.6 mm LC Column
Solvent A: 0.1% TFA water, Solvent B: 0.1% TFA acetonitrile; Isocratic / Gradient elution: 5% Solvent B for 0 to 2 min, 5% – 95% Solvent B for 2 to 22.5 min. Flow rate: 1 mL/min.

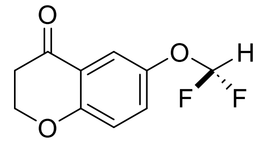

S21

Arene substrate:

6-(Difluoromethoxy)chroman-4-one (**S21)**

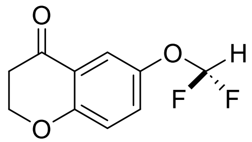

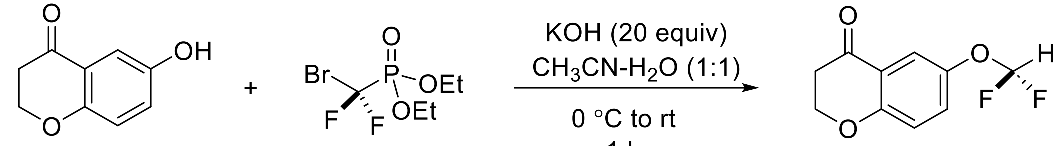

(250 mg, 1.52 mmol, 1.0 equiv) (2.0 equiv) (210 mg, 64%)

General conditions **A** were followed using 6-hydroxychroman-4-one (250 mg, 1.52 mmol, 1.0 equiv), $BrCF_2P(O)(OC_2H_5)_2$ (814 mg, 3.04 mmol, 2.0 equiv), KOH (1.70 g, 30.4 mmol, 20 equiv), CH_3CN: H_2O (5 mL : 5 mL) for 1 h.

Purification: Gradient column chromatography [SiO_2, EtOAc:Hexanes 05:95 to 20:80)] to obtain off-yellow solid of **S21** (210 mg, 64%).

R_f : 0.4 (EtOAc : Hexanes 2:8)

^{1}H NMR ($CDCl_3$, 400 MHz): δ 7.63 (d, J = 3.2 Hz, 1H), 7.27 (dd, J = 8.9, 3.4 Hz, 1H), 6.98 (d, J = 8.9 Hz, 1H), 6.49 (t, J = 73.8 Hz, 1H), 4.53 (t, J = 6.4 Hz, 2H), 2.82 (t, J = 6.7 Hz, 2H).

^{13}C NMR ($CDCl_3$, 101 MHz): δ 191.06, 159.53, 144.94 (t, J = 2.9 Hz), 128.86, 121.67, 119.72, 117.48, 115.87 (t, J = 261.3 Hz), 67.31, 37.60.

^{19}F NMR ($CDCl_3$, 376 MHz): δ –81.29 (d, J = 73.5 Hz).

HRMS (ESI-TOF) m/z: $[M + H]^+$ Calcd. for $C_{10}H_9F_2O_3$ 215.0520; found 215.0519.

Authentic fluoroarene standard

^{19}F-Reference compound – **[^{19}F]21** was purchased from the commercial supplier. CAS Number 66892-34-0

Radio-HPLC analysis and characterization

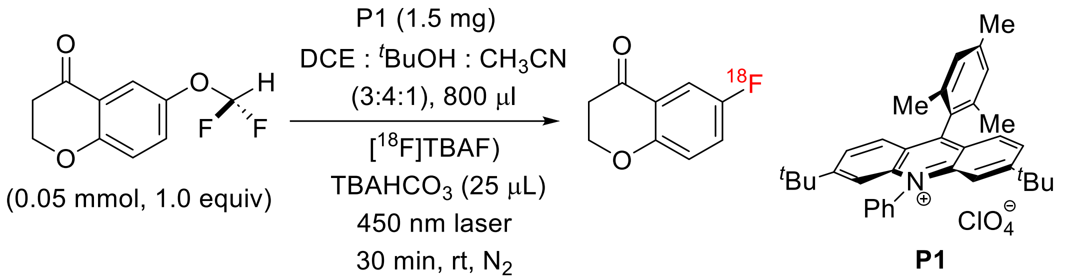

Entry	Activity at starting	Activity at EOS	Alumina Filtration	Injected Dose	Collected Dose	Isolation Time	Decay Corrected	Filtration Yield	HPLC Purity of Filtrate	%Yield
1	12.48 mCi	8.84 mCi	3.20 mCi	510 µCi	265 µCi	16.2 min	460.89 µCi	36.19%	57.40%	20.77%
2	10.19 mCi	8.15 mCi	2.63 mCi	566 µCi	88 µCi	16.2 min	511.50 µCi	32.26%	17.20%	5.72%
									**Average %Yield: 13.2% (n=2)**	

Table S26: HPLC isolated RCYs for **[^{18}F]21**

HPLC Conditions – method 1: Column: Phenomenex, Kinetex® 5µm F5 100 Å, 250 ✕ 4.6 mm LC Column

Solvent A: 0.1% TFA water, Solvent B: 0.1% TFA acetonitrile; Isocratic / Gradient elution: 5% Solvent B for 0 to 2 min, 5% – 65% Solvent B for 2 to 22.5 min. Flow rate: 1 mL/min

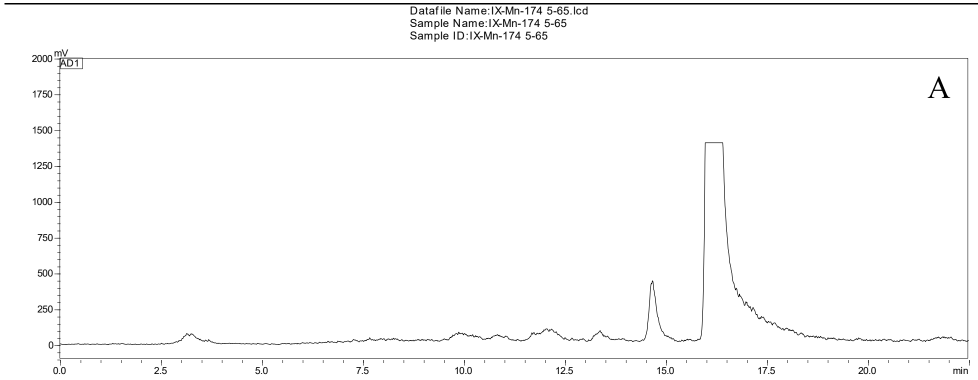

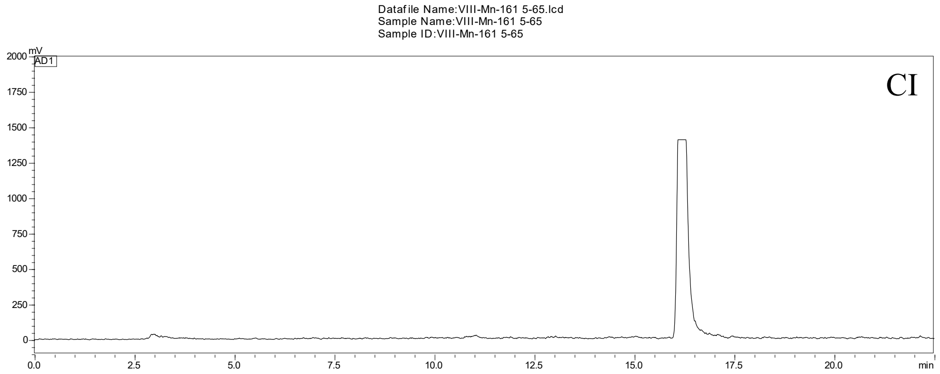

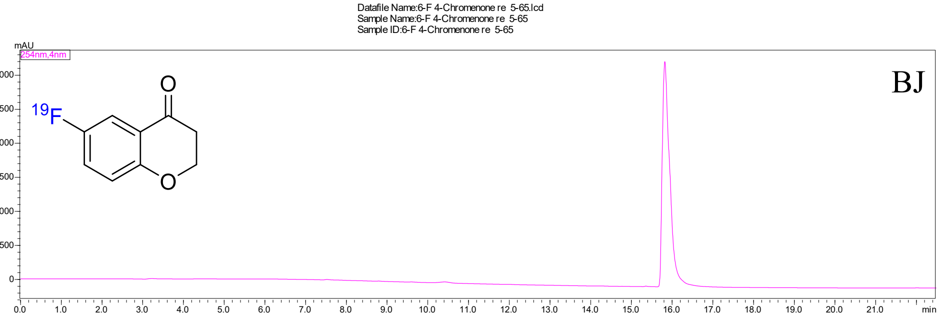

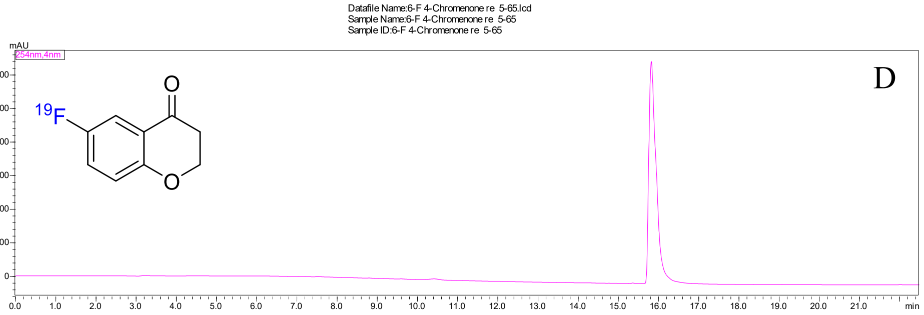

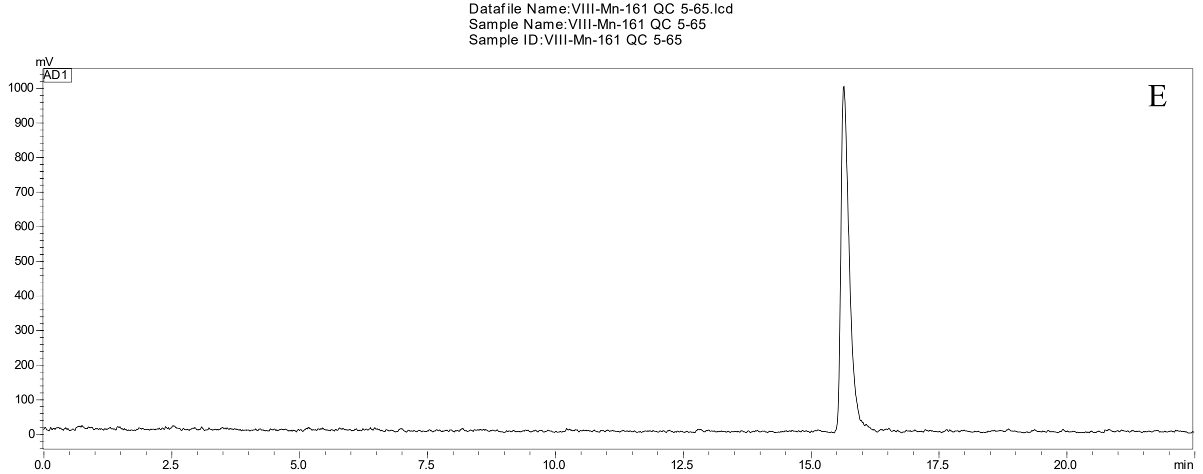

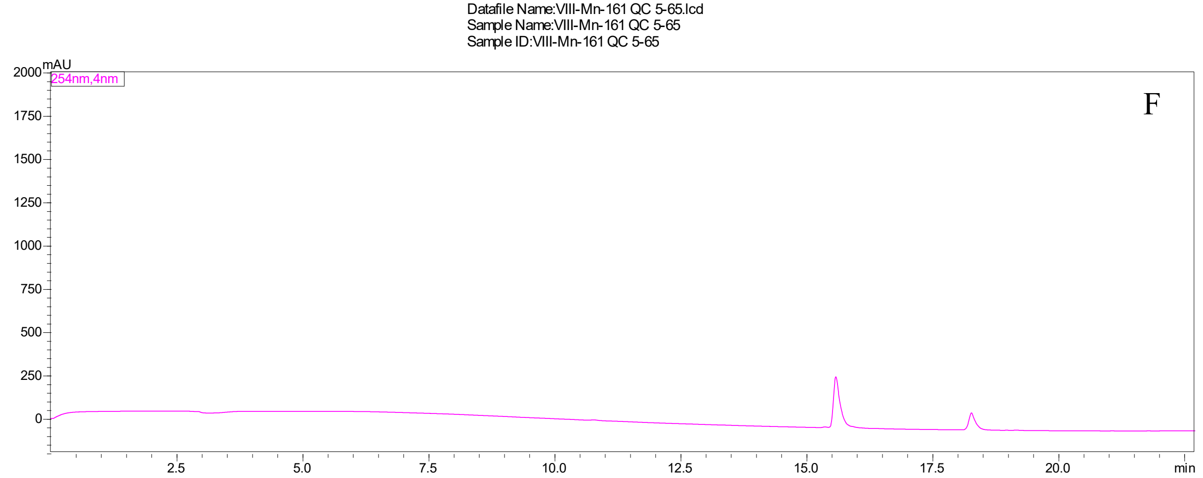

Figure S28: Radio-HPLC analysis of reaction mixture from **S21**. Reaction mixture with HPLC method 1 (A), authentic **[^{19}F]21** with HPLC method 1 (B), reaction mixture with HPLC method 1 (C), authentic **[^{19}F]21** with HPLC method 1 (D), and QC for **[^{18}F]21** (E and F).

HPLC Conditions for QC: Column: Phenomenex, Kinetex® 5µm F5 100 Å, 250 × 4.6 mm LC Column
Solvent A: 0.1% TFA water, Solvent B: 0.1% TFA acetonitrile; Isocratic / Gradient elution: 5% Solvent B for 0 to 2 min, 5% – 65% Solvent B for 2 to 22.5 min. Flow rate: 1 mL/min.

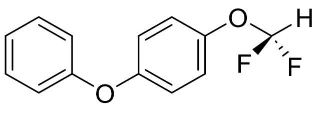

S22

Arene substrate:

1-(Difluoromethoxy)-4-phenoxybenzene (S22)

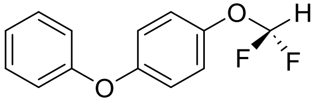

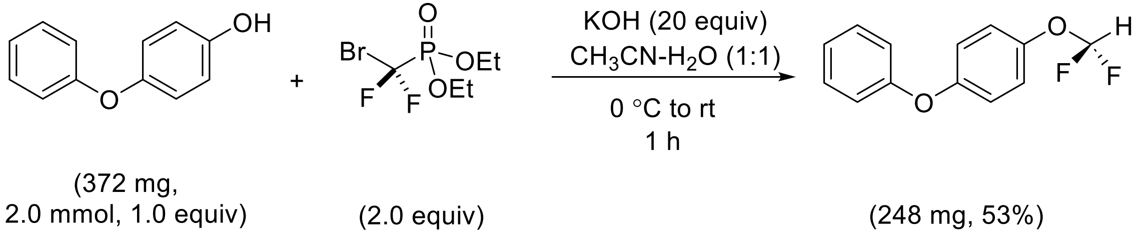

General conditions **A** were followed using 4-phenoxyphenol (372 mg, 2.0 mmol, 1.0 equiv), $BrCF_2P(O)(OC_2H_5)_2$ (1.6 g, 6.0 mmol, 2.0 equiv), KOH (3.36 g, 60 mmol, 20 equiv), CH_3CN: H_2O (5 mL : 5 mL) for 1 h.

Purification: Gradient column chromatography [SiO_2, EtOAc:Hexanes 00:100 to 2:98)] to obtain colourless liquid of **S22** (342 mg, 48%).

R_f : 0.8 (EtOAc : Hexanes 1:9)

^{1}H NMR ($CDCl_3$, 400 MHz): δ 7.35 (dq, J = 7.5, 2.2 Hz, 2H), 7.14–7.09 (m, 3H), 7.02–6.98 (m, 4H), 6.47 (t, J = 74.0 Hz, 1H).

^{13}C NMR ($CDCl_3$, 101 MHz): δ 157.29, 154.83, 146.58 (t, J = 2.9 Hz), 129.98, 123.62, 121.54, 120.09, 118.88, 116.16 (t, J = 260.1 Hz).

^{19}F NMR ($CDCl_3$, 376 MHz): δ –80.67 (d, J = 72.8 Hz).

HRMS (ESI-TOF) m/z: $[M + Na]^+$ Calcd. for $C_{13}H_{10}F_2NaO_2$ 259.0547; found 259.0551.

Deoxyfluorination and Authentic fluoroarene standard

^{19}F-Reference compound – **[^{19}F]22** was purchased from the commercial supplier. CAS Number 330-84-7

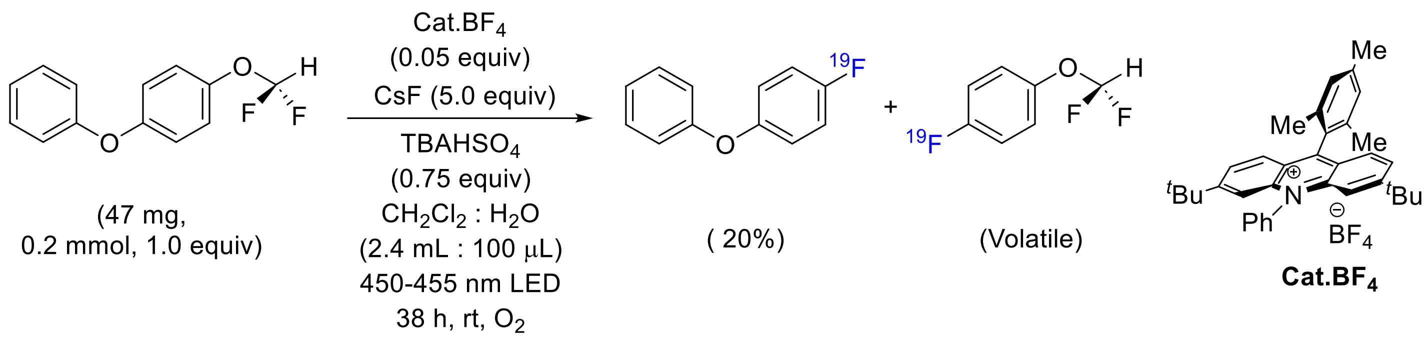

Radio-HPLC analysis and characterization

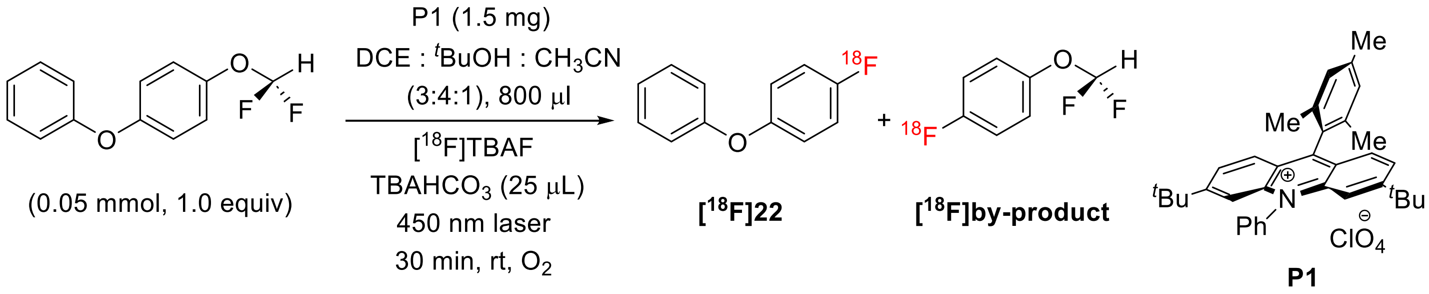

Entry	Activity at starting	Activity at EOS	Alumina Filtration	Injected Dose	Collected Dose	Isolation Time	Decay Corrected	Filtration Yield	HPLC Purity of Filtrate	%Yield
1*	9.23 mCi	5.04 mCi	2.71 mCi	460 µCi	93 µCi	15.7 min	418.12 µCi	53.76%	22.24%	11.95%
2**	7.50 mCi	5.89 mCi	2.98 mCi	215 µCi	31 µCi	17.5 min	193.01 µCi	50.59%	16.06%	8.12%
								**Average %Yield: 10.0% (n=2)**		
3*	9.23 mCi	5.04 mCi	2.71 mCi	460 µCi	93 µCi	15.7 min	418.12 µCi	53.76%	32.22%	17.32%
4**	7.50 mCi	5.89 mCi	2.98 mCi	215 µCi	134 µCi	14.7 min	193.01 µCi	50.59%	26.44%	13.37%
								**Average %Yield: 15.3% (n=2)**		

Table S27: HPLC isolated RCYs for **[^{18}F]22** and **[^{18}F]**by-product (Entry 3 and 4).

*HPLC Conditions – method 1: Column: Phenomenex, Kinetex® 5µm F5 100 Å, 250 ✕ 4.6 mm LC Column

Solvent A: 0.1% TFA water, Solvent B: 0.1% TFA acetonitrile; Isocratic / Gradient elution: 30% Solvent B for 0 to 2 min, 30% – 95% Solvent B for 2 to 22.5 min. Flow rate: 1 mL/min

**HPLC Conditions – method 2: Column: Phenomenex, Kinetex® 5µm F5 100 Å, 250 ✕ 4.6 mm LC Column

Solvent A: 0.1% TFA water, Solvent B: 0.1% TFA acetonitrile; Isocratic / Gradient elution: 20% Solvent B for 0 to 2 min, 20% – 95% Solvent B for 2 to 22.5 min. Flow rate: 1 mL/min

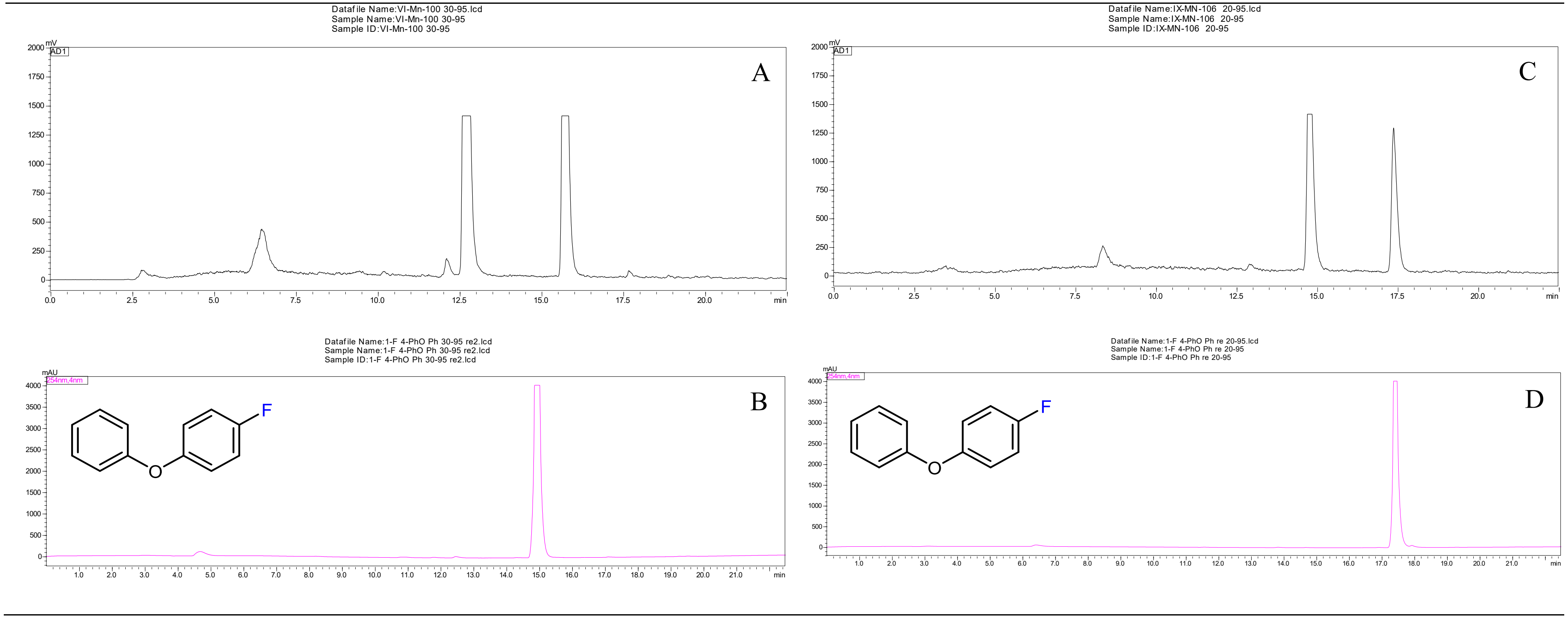
Datafile Name:VI-Mn-100 30-95.lcd
Sample Name:VI-Mn-100 30-95
Sample ID:VI-Mn-100 30-95
mV
AD1
A
min
Datafile Name:IX-MN-106 20-95.lcd
Sample Name:IX-MN-106 20-95
Sample ID:IX-MN-106 20-95
mV
AD1
C
min
Datafile Name:1-F 4-PhO Ph 30-95 re2.lcd
Sample Name:1-F 4-PhO Ph 30-95 re2.lcd
Sample ID:1-F 4-PhO Ph 30-95 re2.lcd
mAU
254nm,4nm
B
O
F
min
Datafile Name:1-F 4-PhO Ph re 20-95.lcd
Sample Name:1-F 4-PhO Ph re 20-95
Sample ID:1-F 4-PhO Ph re 20-95
mAU
254nm,4nm
D
O
F
min

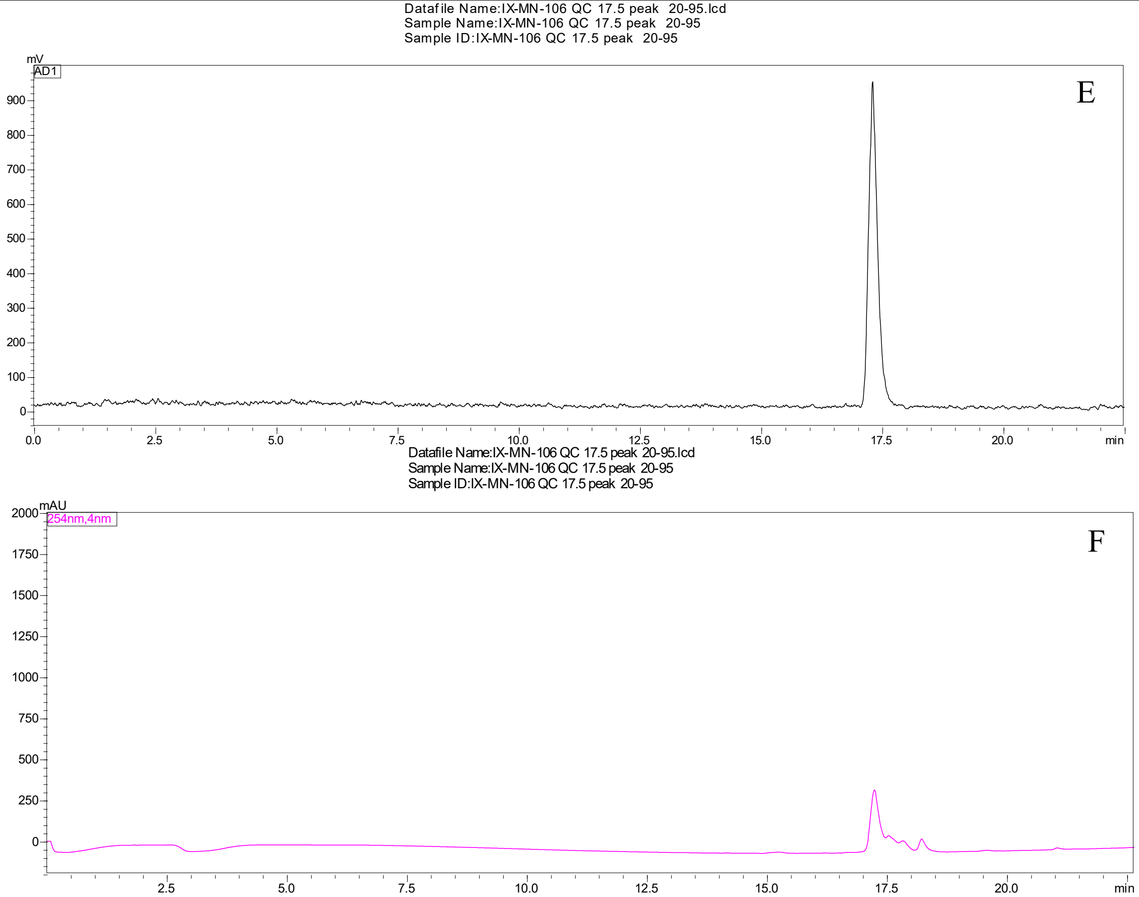

Figure S29: Radio-HPLC analysis of reaction mixture from **S22**. Reaction mixture with HPLC method 1 (A), authentic **[^{19}F]22** with HPLC method 1 (B), reaction mixture with HPLC method 2 (C), authentic **[^{19}F]22** with HPLC method 2 (D), and QC for **[^{18}F]22** (E and F).

HPLC Conditions for QC: Column: Phenomenex, Kinetex® 5µm F5 100 Å, 250 × 4.6 mm LC Column

Solvent A: 0.1% TFA water, Solvent B: 0.1% TFA acetonitrile; Isocratic / Gradient elution: 20% Solvent B for 0 to 2 min, 20% – 95% Solvent B for 2 to 22.5 min. Flow rate: 1 mL/min.

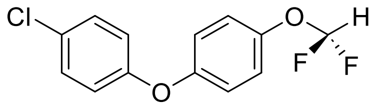

S23

Arene substrate:

1-Chloro-4-(4-(difluoromethoxy)phenoxy)benzene (S23)

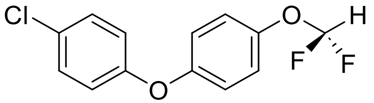

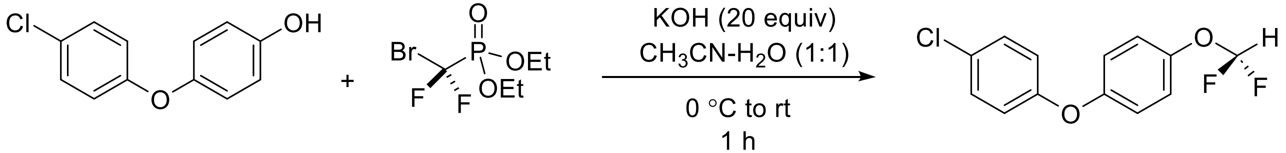

General conditions **A** were followed using 4-(4-chlorophenoxy)phenol (662 mg, 3.0 mmol, 1.0 equiv), $BrCF_2P(O)(OC_2H_5)_2$ (1.6 g, 6.0 mmol, 2.0 equiv), KOH (3.36 g, 60 mmol, 20 equiv), CH_3CN: H_2O (7.5 mL : 7.5 mL) for 1 h.

Purification: Gradient column chromatography [SiO_2, EtOAc:Hexanes 00:100 to 2:98)] to obtain colourless liquid of **S23** (342 mg, 48%).

R_f : 0.6 (EtOAc : Hexanes 0.2:0.8)

^{1}H NMR ($CDCl_3$, 400 MHz): δ 7.30 (dt, J = 12.6, 3.4 Hz, 2H), 7.12 (dt, J = 9.1, 3.5 Hz, 2H), 6.99 (dt, J = 9.1, 3.5 Hz, 2H), 6.93 (dt, J = 8.9, 3.3 Hz, 2H), 6.48 (t, J = 73.6 Hz, 1H). ^{13}C NMR ($CDCl_3$, 101 MHz): δ 155.99, 154.41, 146.83 (t, J = 2.9 Hz), 129.97, 128.64, 121.68, 120.11 (d, J = 13.2 Hz), 116.06 (t, J = 260.5 Hz). ^{19}F NMR ($CDCl_3$, 376 MHz): δ –80.80 (d, J = 73.5 Hz).

HRMS (ESI-TOF) m/z: $[M + H]^+$ Calcd. for $C_{13}H_{10}{}^{35}ClF_2O_2$ 271.0337; found 271.0333.

Deoxyfluorination and Authentic fluoroarene standard

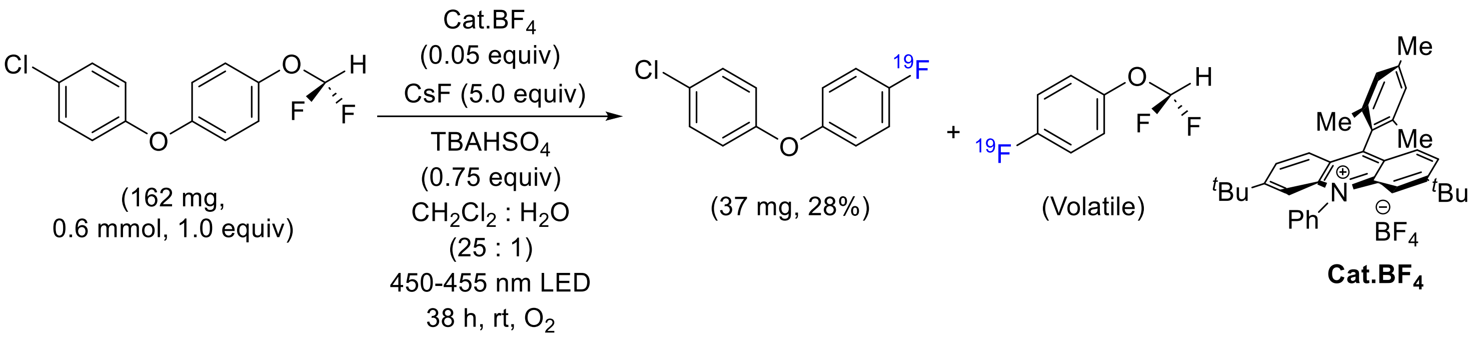

1-Chloro-4-(4-(fluoro)phenoxy)benzene ([^{19}F]23)

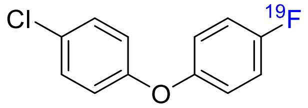

General conditions **I** were followed using 1-chloro-4-(4-(difluoromethoxy)phenoxy)benzene **S23** (162 mg, 0.6 mmol, 1.0 equiv), Mes-Acr-$Ph^+BF_4^-$ (0.05 equiv), CsF (5.0 equiv), $TBAHSO_4$ (0.75 equiv), CH_2Cl_2 : H_2O (0.1 M, 25:1), 450-455 nm LED, 38 h, 33 °C, O_2.

Purification: Isocratic column chromatography [SiO_2, EtOAc:Hexanes 2:98)] to obtain the titled compound **[^{19}F]23** as colourless liquid (37 mg, 28%). R_f : 0.7 (EtOAc : Hexanes 0.2:0.8)

^{1}H NMR ($CDCl_3$, 400 MHz): δ 7.24 (dt, J = 9.0, 2.2 Hz, 2H), 7.00 (tt, J = 9.2, 2.5 Hz, 2H), 6.96–6.91 (m, 2H), 6.86 (dt, J = 8.9, 3.3 Hz, 2H).

^{13}C NMR ($CDCl_3$, 101 MHz): δ 160.36, 157.95, 156.51, 152.67 (d, J = 2.9 Hz), 129.89, 128.28, 120.75 (d, J = 7.9 Hz), 119.57, 116.59 (d, J = 23.5 Hz).

^{19}F NMR ($CDCl_3$, 376 MHz): δ –119.46 (ddd, J = 12.5, 8.3, 5.4 Hz).

<u>Radio-HPLC analysis and characterization</u>

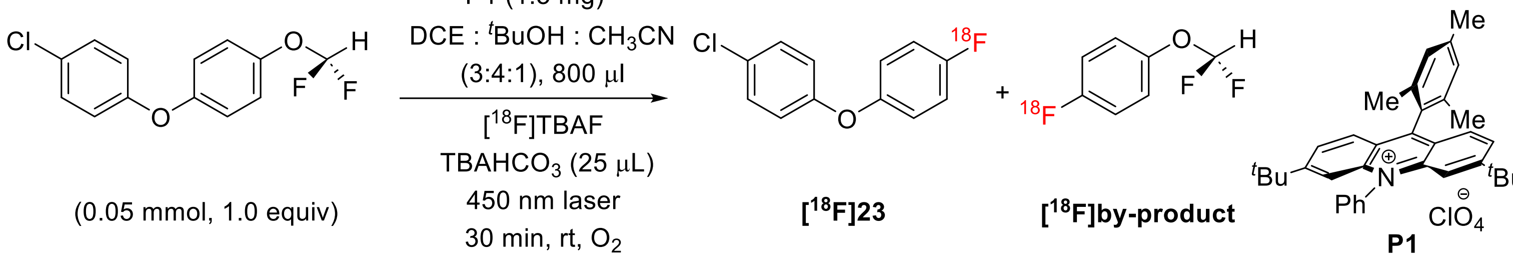

Entry	Activity at starting	Activity at EOS	Alumina Filtration	Injected Dose	Collected Dose	Isolation Time	Decay Corrected	Filtration Yield	HPLC Purity of Filtrate	%Yield
1	13.47 mCi	10.17 mCi	6.94 mCi	366 µCi	75 µCi	18.0 min	326.67 µCi	68.32%	22.95%	15.67%
2	10.89 mCi	7.99 mCi	3.51 mCi	272 µCi	41 µCi	18.0 min	242.77 µCi	43.92%	16.88%	7.41%
										**Average %Yield: 11.5% (n=2)**
3	13.47 mCi	10.17 mCi	6.94 mCi	366 µCi	145 µCi	14.3 min	334.96 µCi	68.32%	43.28%	32.98%
4	10.89 mCi	7.99 mCi	3.51 mCi	272 µCi	82 µCi	14.3 min	248.93 µCi	43.92%	32.94%	14.46%
										**Average %Yield: 23.7% (n=2)**

Table S28: HPLC isolated RCYs for **[^{18}F]23** (Entry 1 and 2) and **[^{18}F]**by-product (Entry 3 and 4).

HPLC Conditions – method 1: Column: Phenomenex, Kinetex® 5µm F5 100 Å, 250 ✕ 4.6 mm LC Column

Solvent A: 0.1% TFA water, Solvent B: 0.1% TFA acetonitrile; Isocratic / Gradient elution: 20% Solvent B for 0 to 2 min, 20% – 95% Solvent B for 2 to 22.5 min. Flow rate: 1 mL/min

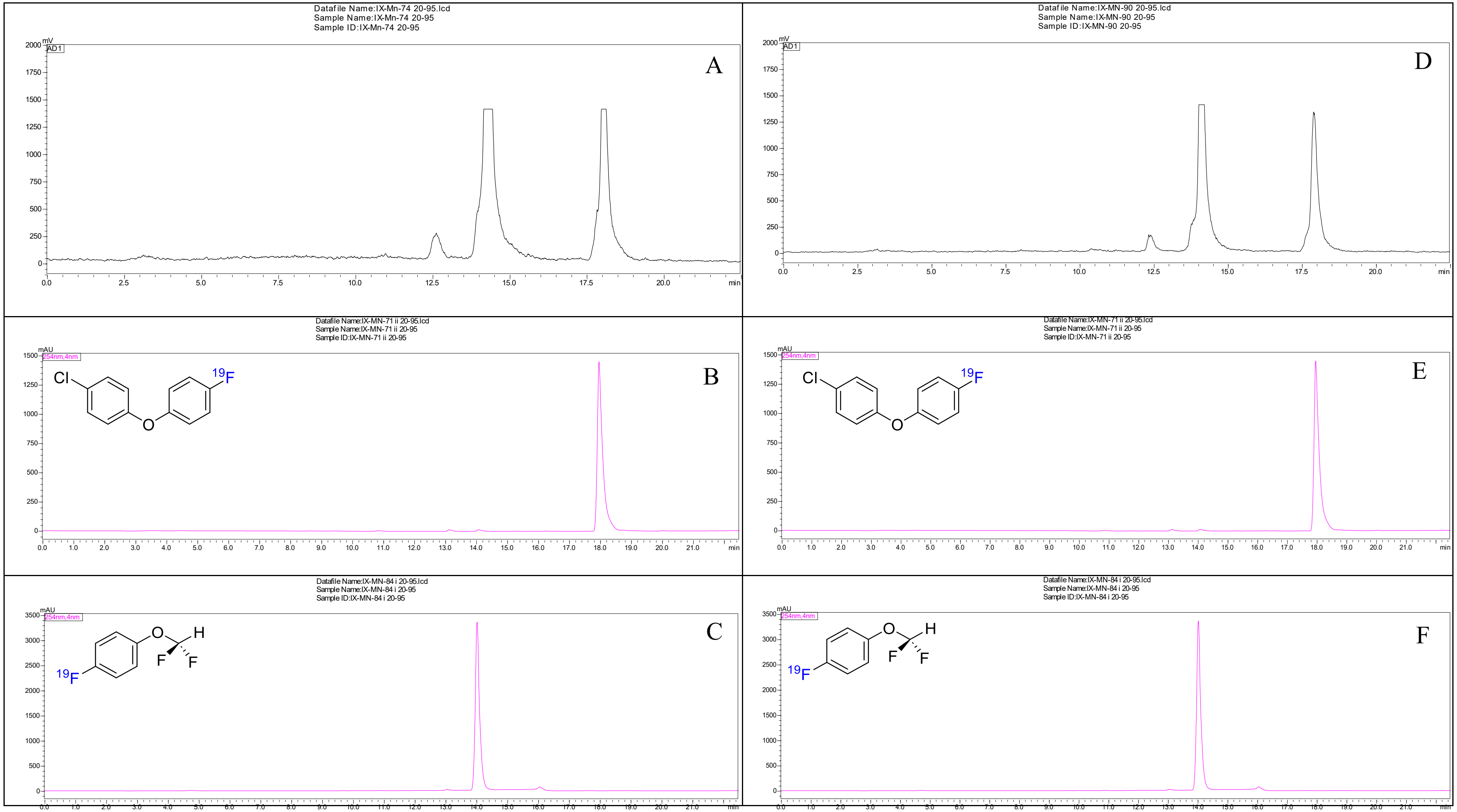

Datafile Name:IX-MN-90 QC Peak 18.0 20-95.lcd
Sample Name:IX-MN-90 QC Peak 18.0 20-95
Sample ID:IX-MN-90 QC Peak 18.0 20-95

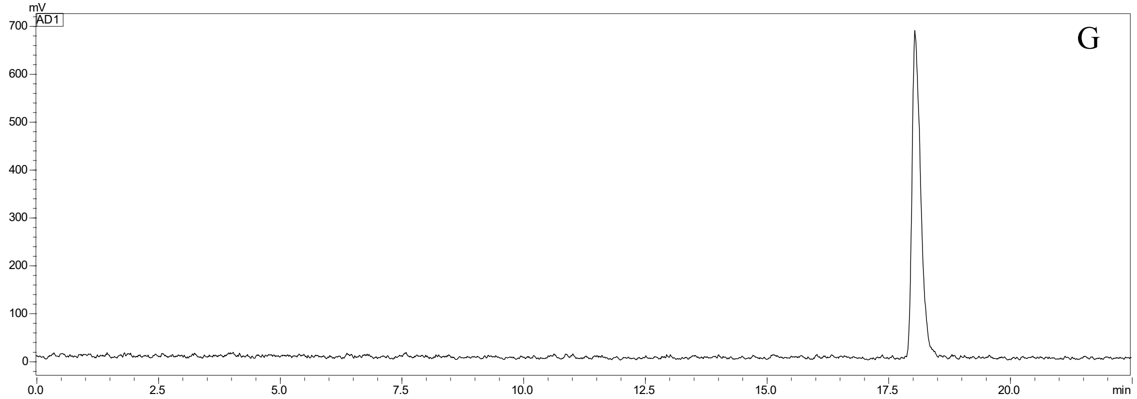

Datafile Name:IX-MN-90 QC Peak 18.0 20-95.lcd
Sample Name:IX-MN-90 QC Peak 18.0 20-95
Sample ID:IX-MN-90 QC Peak 18.0 20-95

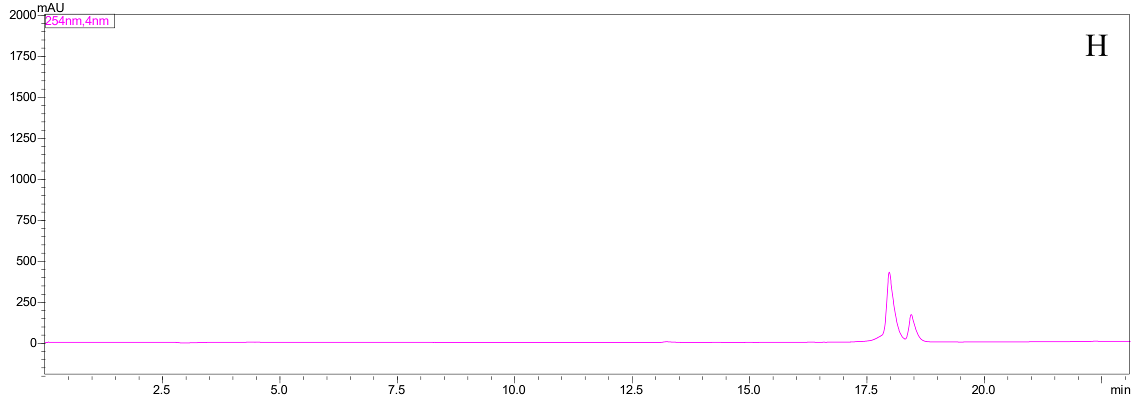

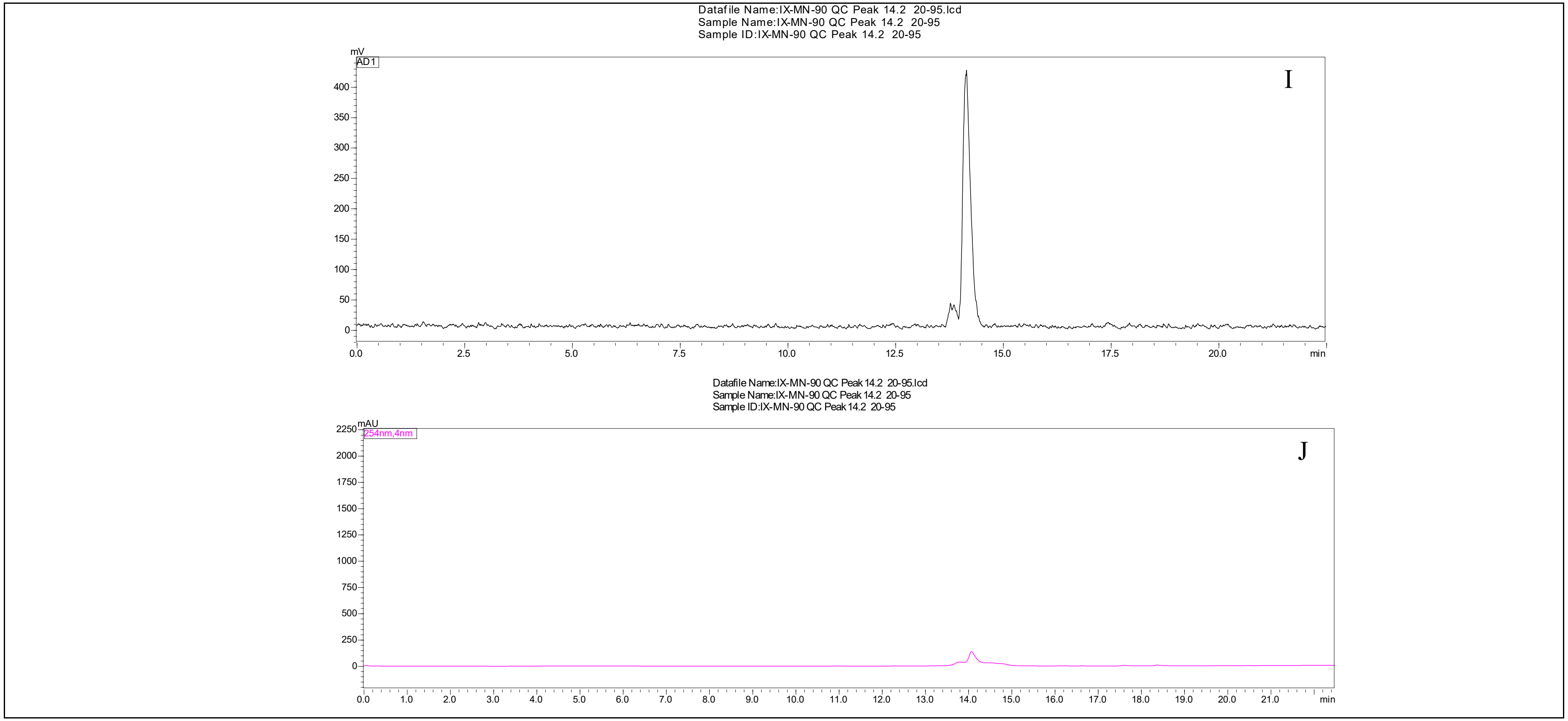

Figure S30: Radio-HPLC analysis of reaction mixture from **S23**. Reaction mixture with HPLC method 1 (A), authentic **[^{19}F]23** with HPLC method 1 (B), authentic **[^{19}F]by-product** with HPLC method 1 (C), reaction mixture with HPLC method 1 (D), authentic **[^{19}F]23** with HPLC method 1 (E), authentic **[^{19}F]by-product** with HPLC method 1 (F), QC for **[^{18}F]23** (G and H), and QC for **[^{18}F]by-product** (I and J).

HPLC Conditions for QC: Column: Phenomenex, Kinetex® 5µm F5 100 Å, 250 × 4.6 mm LC Column

Solvent A: 0.1% TFA water, Solvent B: 0.1% TFA acetonitrile; Isocratic / Gradient elution: 20% Solvent B for 0 to 2 min, 20% – 95% Solvent B for 2 to 22.5 min. Flow rate: 1 mL/min.

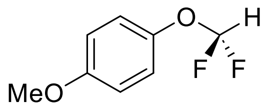

S24

Arene substrate:

1-(Difluoromethoxy)-4-methoxybenzene (S24)

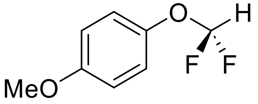

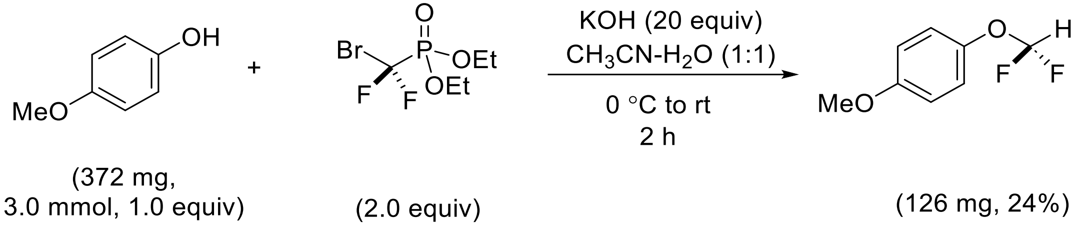

General conditions **A** were followed using 4-(methoxy)phenol (372 mg, 2.0 mmol, 1.0 equiv), $BrCF_2P(O)(OC_2H_5)_2$ (1.6 g, 6.0 mmol, 2.0 equiv), KOH (3.36 g, 60 mmol, 20 equiv), CH_3CN: H_2O (5 mL : 5 mL) for 2 h.

Purification: Gradient column chromatography [SiO_2, EtOAc:Hexanes 00:100 to 5:95)] to obtain colourless liquid of **S24** (126 mg, 24%).

R_f: 0.7 (EtOAc : Hexanes 1:9)

^{1}H NMR ($CDCl_3$, 400 MHz): δ 7.06 (dt, J = 9.1, 3.7 Hz, 2H), 6.87 (dt, J = 9.1, 3.7 Hz, 2H), 6.42 (t, J = 74.7 Hz, 1H), 3.79 (s, 3H).

^{13}C NMR ($CDCl_3$, 101 MHz): δ 157.37, 144.70 (t, J = 2.9 Hz), 121.47, 116.38 (t, J = 259.5 Hz), 114.84, 55.72.

^{19}F NMR ($CDCl_3$, 376 MHz): δ –80.42 (d, J = 74.9 Hz).

HRMS (ESI-TOF) m/z: $[M + H]^+$ Calcd. for $C_8H_8F_2NaO_2$ 197.0390; found 197.0394.

Authentic fluoroarene standard

^{19}F-Reference compound – **[^{19}F]24** was purchased from the commercial supplier. CAS Number 459-60-9

Radio-HPLC analysis and characterization

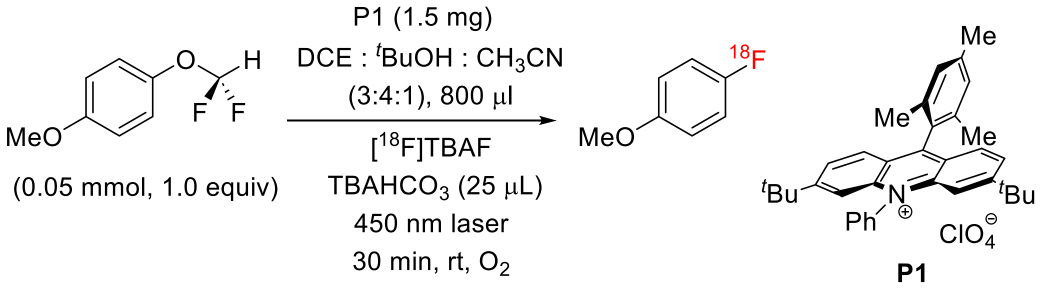

Entry	Activity at starting	Activity at EOS	Alumina Filtration	Injected Dose	Collected Dose	Isolation Time	Decay Corrected	Filtration Yield	HPLC Purity of Filtrate	%Yield
1	12.20 mCi	9.48 mCi	2.93 mCi	391μCi	163 μCi	12.8 min	362.16 μCi	30.90%	45.00%	13.91%
2	8.20 mCi	6.62 mCi	1.64 mCi	245 μCi	54 μCi	12.8 min	225.69 μCi	24.77%	23.92%	5.92%
								**Average %Yield: 9.9% (n=2)**		

Table S29: HPLC isolated RCYs for **[^{18}F]24**

HPLC Conditions – method 1: Column: Phenomenex, Kinetex® 5μm F5 100 Å, 250 ✕ 4.6 mm LC Column

Solvent A: 0.1% TFA water, Solvent B: 0.1% TFA acetonitrile; Isocratic / Gradient elution: 20% Solvent B for 0 to 2 min, 20% – 95% Solvent B for 2 to 22.5 min. Flow rate: 1 mL/min

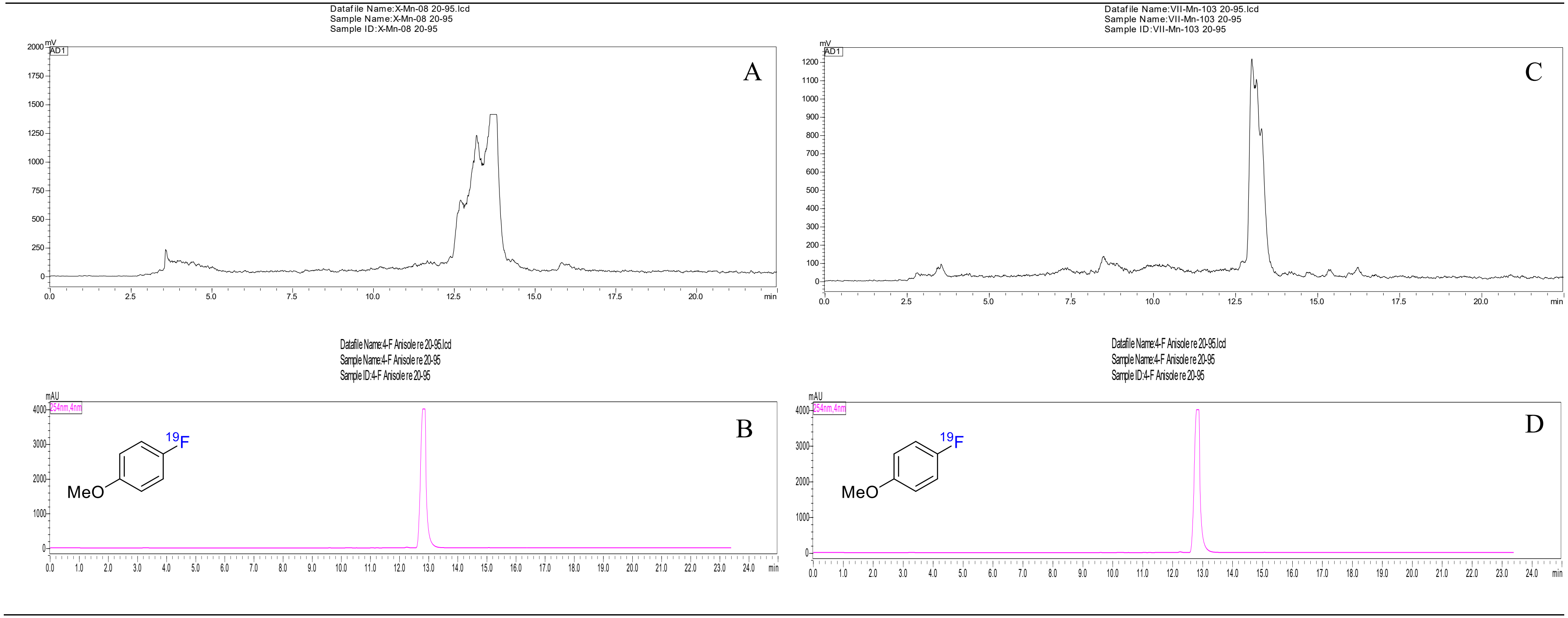
Datafile Name:X-Mn-08 20-95.lcd
Sample Name:X-Mn-08 20-95
Sample ID:X-Mn-08 20-95
A
Datafile Name:VII-Mn-103 20-95.lcd
Sample Name:VII-Mn-103 20-95
Sample ID:VII-Mn-103 20-95
C
Datafile Name:4-F Anisole re 20-95.lcd
Sample Name:4-F Anisole re 20-95
Sample ID:4-F Anisole re 20-95
B
MeO
19F
D
mV
mAU
min

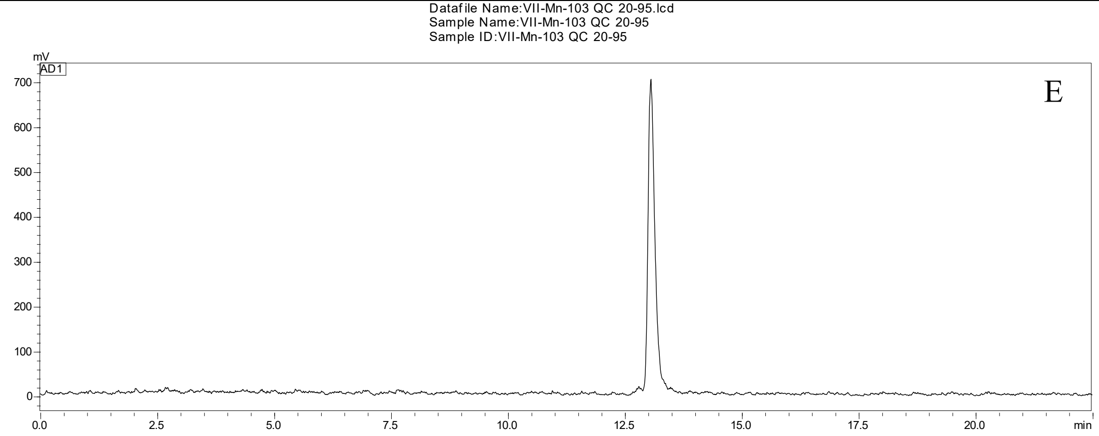

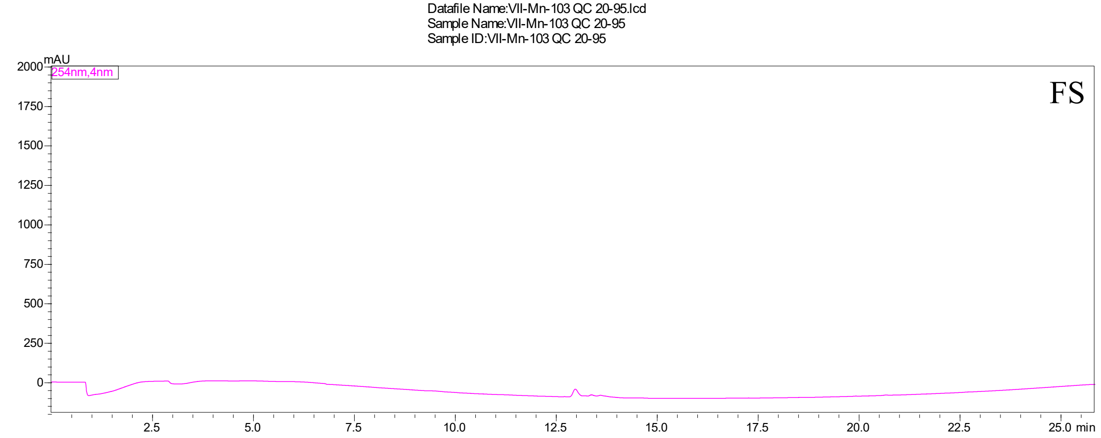

Figure S31: Radio-HPLC analysis of reaction mixture from **S24**. Reaction mixture with HPLC method 1 (A), authentic **[^{19}F]24** with HPLC method 1 (B), reaction mixture with HPLC method 1 (C), authentic **[^{19}F]24** with HPLC method 1 (D), and QC for **[^{18}F]24** (E and F).

HPLC Conditions for QC: Column: Phenomenex, Kinetex® 5µm F5 100 Å, 250 × 4.6 mm LC Column

Solvent A: 0.1% TFA water, Solvent B: 0.1% TFA acetonitrile; Isocratic / Gradient elution: 20% Solvent B for 0 to 2 min, 20% – 95% Solvent B for 2 to 22.5 min. Flow rate: 1 mL/min.

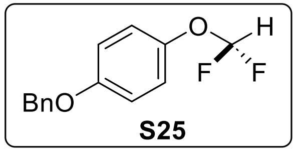

Arene substrate:

1-(Benzyloxy)-4-(difluoromethoxy)benzene (S25)

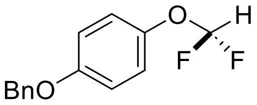

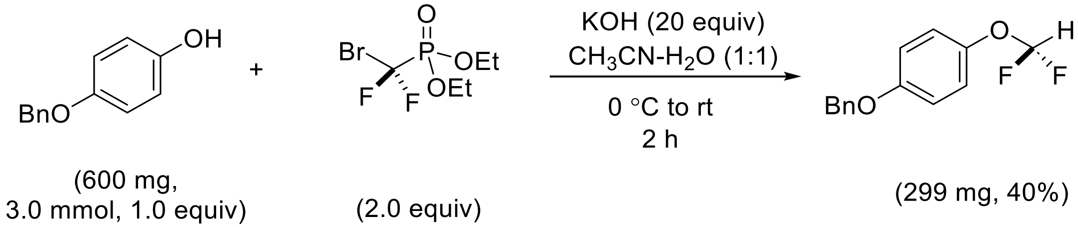

General conditions **A** were followed using 4-(benzyloxy)phenol (600 mg, 2.0 mmol, 1.0 equiv), $BrCF_2P(O)(OC_2H_5)_2$ (1.6 g, 6.0 mmol, 2.0 equiv), KOH (3.36 g, 60 mmol, 20 equiv), CH_3CN: H_2O (7.5 mL : 7.55 mL) for 2 h.

Purification: Gradient column chromatography [SiO_2, EtOAc:Hexanes 00:100 to 10:90)] to obtain white solid of **S25** (299 mg, 40%).

R_f : 0.7 (EtOAc : Hexanes 1:9)

^{1}H NMR ($CDCl_3$, 400 MHz): δ 7.44–7.37 (m, 4H), 7.36–7.32 (m, 1H), 7.07 (d, J = 7.8 Hz, 2H), 6.95 (dd, J = 8.7, 1.6 Hz, 2H), 6.43 (t, J = 74.3 Hz, 1H), 5.05 (s, 2H).

^{13}C NMR ($CDCl_3$, 101 MHz): δ 156.53, 144.90 (t, J = 2.8 Hz), 136.83, 128.78, 128.23, 127.59, 121.47, 117.64 (t, J = 259.6 Hz), 115.89, 70.58.

^{19}F NMR ($CDCl_3$, 376 MHz): δ –80.39 (d, J = 72.9 Hz).

HRMS (ESI-TOF) m/z: $[M + H]^+$ Calcd. for $C_{14}H_{12}F_2NaO_2$ 273.0703; found 273.0702.

Authentic fluoroarene standard

^{19}F-Reference compound – **[^{19}F]25** was purchased from the commercial supplier. CAS Number 370-78-5

Radio-HPLC analysis and characterization

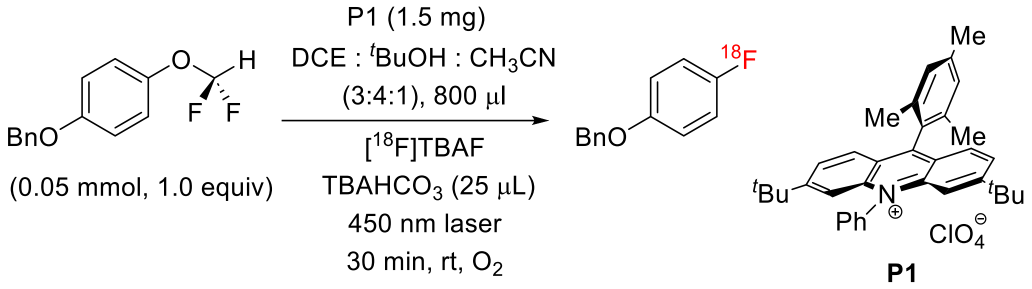

Entry	Activity at starting	Activity at EOS	Alumina Filtration	Injected Dose	Collected Dose	Isolation Time	Decay Corrected	Filtration Yield	HPLC Purity of Filtrate	%Yield
1	7.85 mCi	6.02 mCi	2.12 mCi	263 µCi	79 µCi	17.2 min	236.23 µCi	35.21%	33.34%	11.73%
2	14.38 mCi	11.58 mCi	5.08 mCi	444 µCi	45 µCi	14.9 min	312.14 µCi	43.86%	14.41%	6.32%
										**Average %Yield: 9.0% (n=2)**

Table S30: HPLC isolated RCYs for **[^{18}F]25**

HPLC Conditions – method 1 : Column: Phenomenex, Kinetex® 5µm F5 100 Å, 250 ✕ 4.6 mm LC Column

Solvent A: 0.1% TFA water, Solvent B: 0.1% TFA acetonitrile; Isocratic / Gradient elution: 20% Solvent B for 0 to 2 min, 20% – 95% Solvent B for 2 to 22.5 min. Flow rate: 1 mL/min

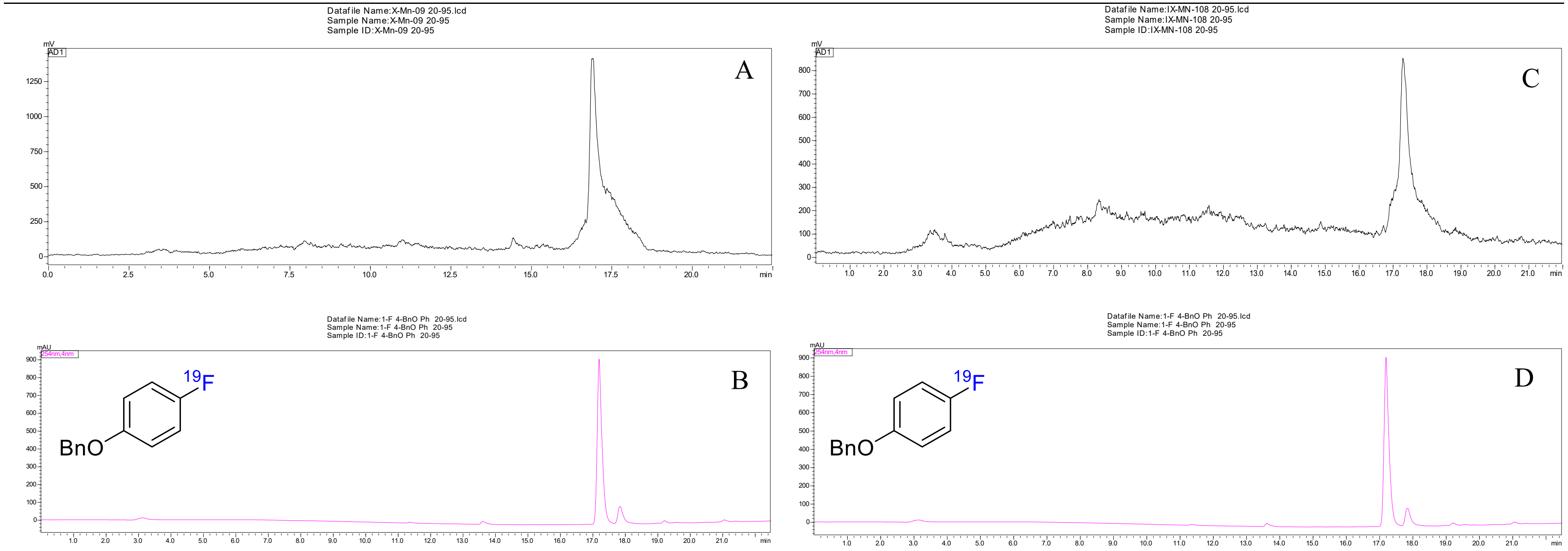
Datafile Name:X-Mn-09 20-95.lcd
Sample Name:X-Mn-09 20-95
Sample ID:X-Mn-09 20-95
A
Datafile Name:IX-MN-108 20-95.lcd
Sample Name:IX-MN-108 20-95
Sample ID:IX-MN-108 20-95
C
Datafile Name:1-F 4-BnO Ph 20-95.lcd
Sample Name:1-F 4-BnO Ph 20-95
Sample ID:1-F 4-BnO Ph 20-95
B
BnO
19F
Datafile Name:1-F 4-BnO Ph 20-95.lcd
Sample Name:1-F 4-BnO Ph 20-95
Sample ID:1-F 4-BnO Ph 20-95
D
BnO
19F
min

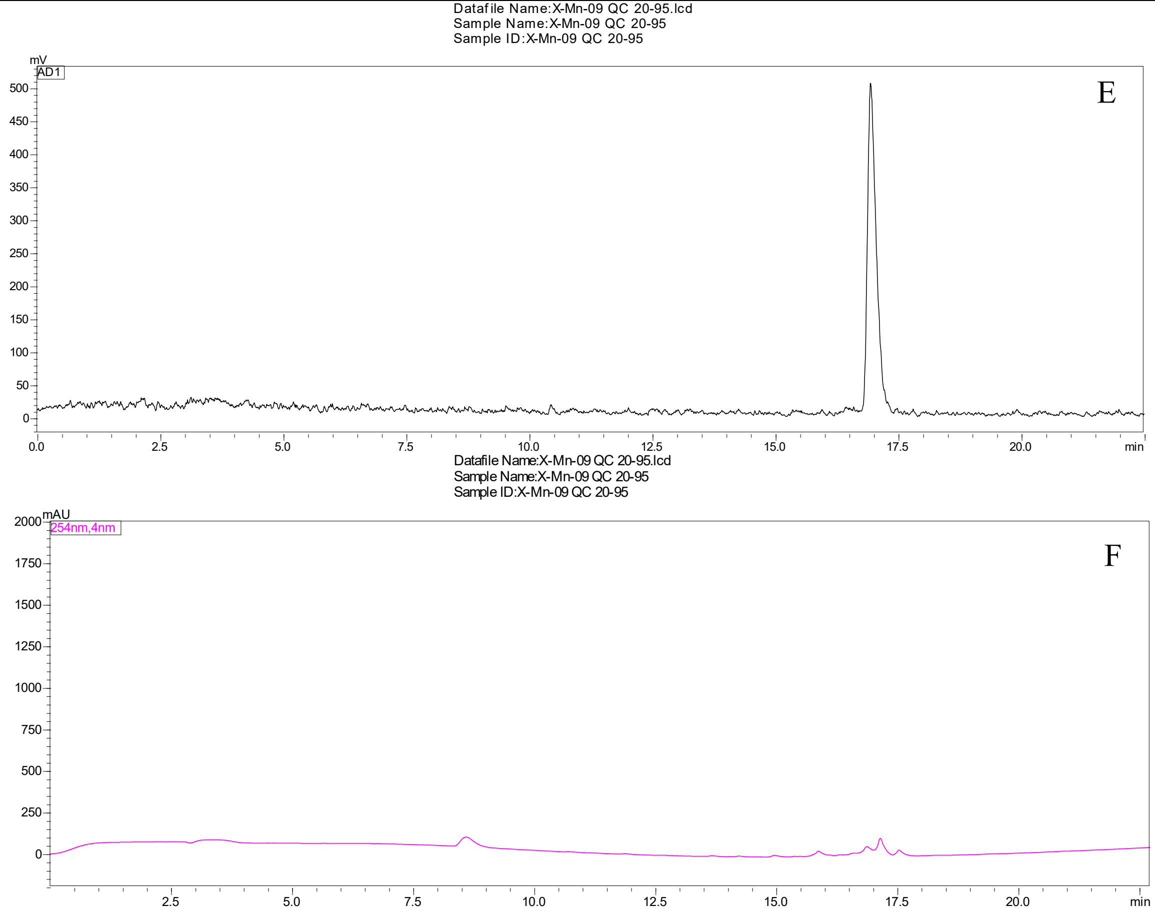

Figure S32: Radio-HPLC analysis of reaction mixture from **S25**. Reaction mixture with HPLC method 1 (A), authentic **[^{19}F]25** with HPLC method 1 (B), reaction mixture with HPLC method 1 (C), authentic **[^{19}F]25** with HPLC method 1 (D), and QC for **[^{18}F]25** (E and F).

HPLC Conditions for QC: Column: Phenomenex, Kinetex® 5µm F5 100 Å, 250 × 4.6 mm LC Column

Solvent A: 0.1% TFA water, Solvent B: 0.1% TFA acetonitrile; Isocratic / Gradient elution: 20% Solvent B for 0 to 2 min, 20% – 95% Solvent B for 2 to 22.5 min. Flow rate: 1 mL/min.

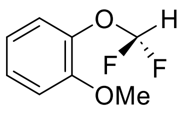

S26

<u>Arene substrate:</u>

1-(Difluoromethoxy)-2-methoxybenzene (S26)

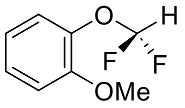

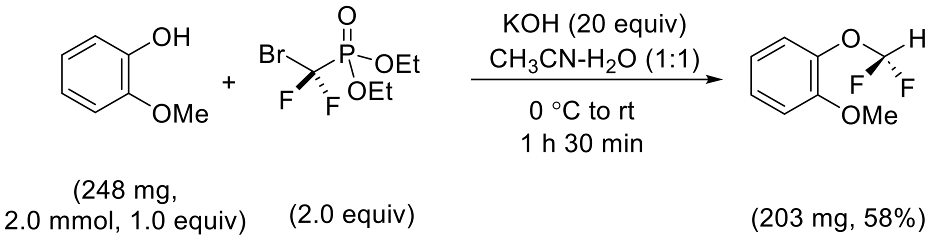

General conditions **A** were followed using 2-methoxyphenol (248 mg, 2.0 mmol, 1.0 equiv), $BrCF_2P(O)(OC_2H_5)_2$ (1.6 g, 6.0 mmol, 2.0 equiv), KOH (3.36 g, 60 mmol, 20 equiv), CH_3CN : H_2O (5 mL : 5 mL) for 1 h 30 min.

Purification: Gradient column chromatography [SiO_2, EtOAc:Hexanes 05:95 to 20:80)] to obtain colourless liquid of **S26** (203 mg, 58%).

R_f: 0.6 (EtOAc : Hexanes 2:8)

^{1}H NMR ($CDCl_3$, 400 MHz): δ 7.21–7.16 (m, 2H), 6.98 (d, J= 8.2, 1.3 Hz, 1H), 6.94 (td, J= 7.6, 1.4 Hz, 1H), 6.55 (t, J= 75.4 Hz, 1H), 3.88 (s, 3H).

^{13}C NMR ($CDCl_3$, 101 MHz): δ 151.27, 140.16 (t, J= 2.9 Hz), 126.79 122.53, 121.12, 116.39 (t, J= 259.3 Hz), 112.82, 56.06.

^{19}F NMR ($CDCl_3$, 376 MHz): δ –81.39 (d, J = 75.1 Hz).

HRMS (ESI-TOF) m/z: $[M + H]^+$ Calcd. for $C_8H_8F_2NaO_2$ 197.0390; found 197.0389.

Authentic fluoroarene standard

[19]F-Reference compound – **[^{19}F]26** was purchased from the commercial supplier. CAS Number 459-60-9

Radio-HPLC analysis and characterization

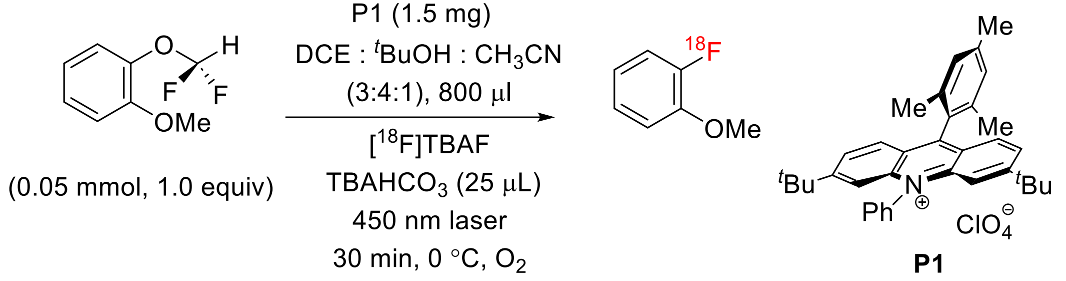

Entry	Activity at starting	Activity at EOS	Alumina Filtration	Injected Dose	Collected Dose	Isolation Time	Decay Corrected	Filtration Yield	HPLC Purity of Filtrate	%Yield
1	9.22 mCi	7.31mCi	3.99 mCi	310 µCi	121 µCi	15.0 min	281.98 µCi	54.58%	42.91%	23.42%
2	10.30 mCi	8.02 mCi	4.49 mCi	384 µCi	141 µCi	15.0 min	349.29 µCi	55.98%	40.36%	22.59%
								**Average %Yield: 23.0% (n=2)**		

Table S31: HPLC isolated RCYs for **[^{18}F]26**

HPLC Conditions – method 1: Column: Phenomenex, Kinetex® 5µm F5 100 Å, 250 ✕ 4.6 mm LC Column

Solvent A: 0.1% TFA water, Solvent B: 0.1% TFA acetonitrile; Isocratic / Gradient elution: 5% Solvent B for 0 to 2 min, 5% – 95% Solvent B for 2 to 22.5 min. Flow rate: 1 mL/min

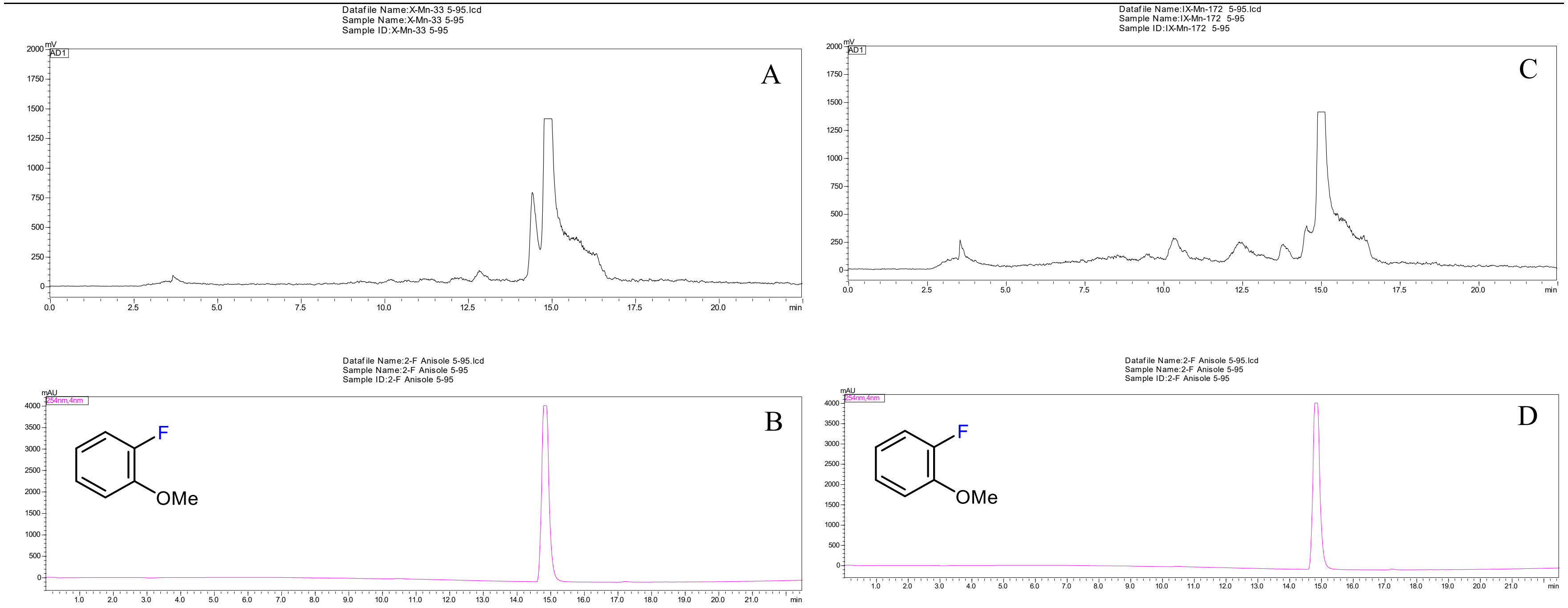
Datafile Name:X-Mn-33 5-95.lcd
Sample Name:X-Mn-33 5-95
Sample ID:X-Mn-33 5-95
A
Datafile Name:IX-Mn-172 5-95.lcd
Sample Name:IX-Mn-172 5-95
Sample ID:IX-Mn-172 5-95
C
Datafile Name:2-F Anisole 5-95.lcd
Sample Name:2-F Anisole 5-95
Sample ID:2-F Anisole 5-95
B
F
OMe
Datafile Name:2-F Anisole 5-95.lcd
Sample Name:2-F Anisole 5-95
Sample ID:2-F Anisole 5-95
D
F
OMe

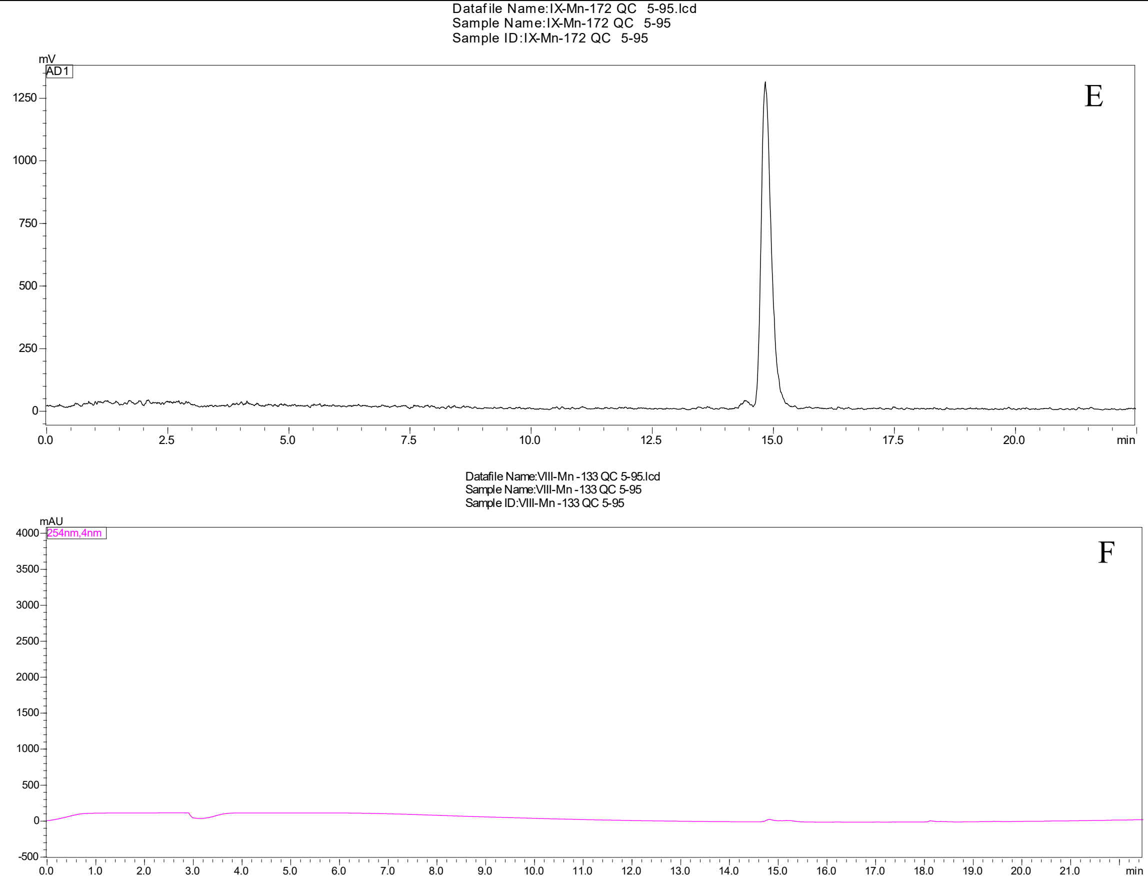

Figure S33: Radio-HPLC analysis of reaction mixture from **S26**. Reaction mixture with HPLC method 1 (A), authentic **[^{19}F]26** with HPLC method 1 (B), reaction mixture with HPLC method 1 (C), authentic **[^{19}F]26** with HPLC method 1 (D), and QC for **[^{18}F]26** (E and F).

HPLC Conditions for QC: Column: Phenomenex, Kinetex® 5µm F5 100 Å, 250 × 4.6 mm LC Column

Solvent A: 0.1% TFA water, Solvent B: 0.1% TFA acetonitrile; Isocratic / Gradient elution: 5% Solvent B for 0 to 2 min, 5% – 95% Solvent B for 2 to 22.5 min. Flow rate: 1 mL/min.

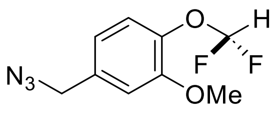

S27

Arene substrate:

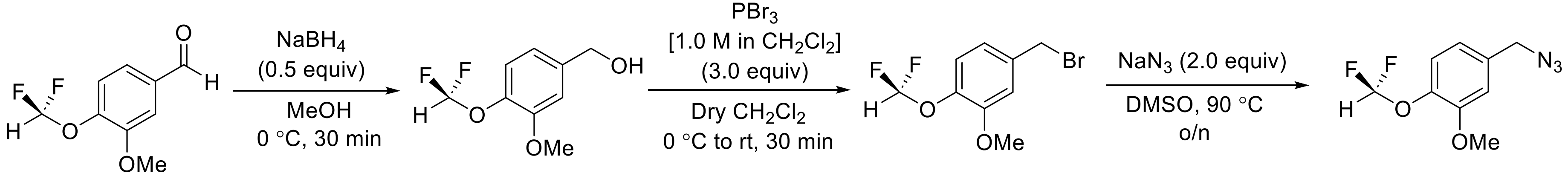

4-(Difluoromethoxy)-3-methoxybenzaldehyde (S27–I1)

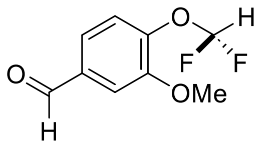

General conditions **A** were followed using 4-hydroxy-3-methoxybenzaldehyde (304 mg, 2.0 mmol, 1.0 equiv), $BrCF_2P(O)(OC_2H_5)_2$ (1.068 g, 4.0 mmol, 2.0 equiv), KOH (2.24 g, 40 mmol, 20 equiv), CH_3CN: H_2O (5 mL : 5 mL) for 2 h.

Purification: Gradient column chromatography [SiO_2, EtOAc:Hexanes 05:95 to 10:90)] to obtain white solid of **S27–I1** (253 mg, 63%).

R_f : 0.7 (EtOAc : Hexanes 3:7)

^{1}H NMR ($CDCl_3$, 400 MHz): δ 9.94 (s, 1H), 7.50 (d, J = 1.7 Hz, 1H), 7.46 (dd, J = 8.1, 1.8 Hz, 1H), 7.46 (d, J = 8.1 Hz, 1H), 6.76 (t, J = 76.4 Hz, 1H), 3.9 (S, 3H).

^{13}C NMR ($CDCl_3$, 101 MHz): δ 190.91, 151.68, 145.06 (t, J= 2.9 Hz), 134.68, 125.17, 121.64, 116.99 (t, J= 261.9 Hz), 111.08, 56.34.

^{19}F NMR ($CDCl_3$, 376 MHz): δ –81.98 (d, J = 74.4 Hz).

HRMS (ESI-TOF) m/z: $[M + H]^+$ Calcd. for $C_9H_9F_2O_3$ 203.0520; found 203.0518.

(4-(Difluoromethoxy)-3-methoxyphenyl)methanol (S27–I2)

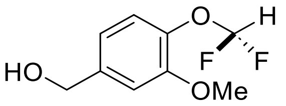

To a solution of 4-(difluoromethoxy)-3-methoxybenzaldehyde **S27–I1** (150 mg, 0.74 mmol, 1.0 equiv) in MeOH was added $NaBH_4$ (14 mg, 0.37 mmol, 0.5 equiv) at 0 °C and the resulting reaction mixture allowed to stir until the staring material consumed (30 min, TLC monitored). Upon completion of the reaction, saturated solution of NH_4Cl was added and MeOH was evaporated under reduced pressure. The aqueous layer was extracted with EtOAc (2 × 10 mL). The combined organics dried (Na_2SO_4) and concentrated under pressure, which was subsequently used for the next step without purification. The purity of the product >95% based on NMR characterisation.

White solid (132 mg, 87%)

R_f : 0.3 (EtOAc : Hexanes 3:7)

^{1}H NMR ($CDCl_3$, 400 MHz): δ 7.09 (d, J = 8.1 Hz, 1H), 6.98 (d, J = 1.9 Hz, 1H), 6.86 (dd, J = 8.1, 1.8 Hz, 1H), 6.62 (t, J = 75.3 Hz, 1H), 4.62 (s, 2H), 3.85 (s, 3H).

^{13}C NMR ($CDCl_3$, 101 MHz): δ 151.22, 139.79, 139.25 (t, J = 2.9 Hz), 122.29, 119.21, 116.29 (t, J = 259.6 Hz), 111.29, 64.69, 55.99.

^{19}F NMR ($CDCl_3$, 376 MHz): δ –81.48 (d, J = 76.8 Hz).

4-(Bromomethyl)-1-(difluoromethoxy)-2-methoxybenzene (S27–I3)

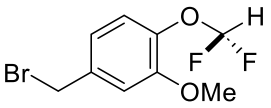

To a solution of (4-(difluoromethoxy)-3-methoxyphenyl)methanol **S27–I2** (130 mg, 0.6 mmol, 1.0 equiv) in dry CH_2Cl_2 (6 mL) was added dropwise a solution of PBr_3 (1.8 mL, 0.93 mmol, 3.0 equiv, (1.0 M in CH_2Cl_2) at 0 °C under Ar atmosphere. The resulting reaction was stirred

at 0 °C until the staring material consumed (1 h, TLC monitored). The reaction mixture cooled to room temperature and washed with the saturated solution of $Na_2S_2O_3$. The aqueous layer was extracted with CH_2Cl_2 (3 ×5 mL). The combined organics dried (Na_2SO_4) and concentrated under reduced pressure, which was subsequently used for the next step without purification. The purity of the product >95% based on NMR characterisation.

Pale yellowish semisolid (113 mg, 71%)

R_f : 0.7 (EtOAc : Hexanes 1:9)

^{1}H NMR ($CDCl_3$, 400 MHz): δ 7.11 (d, J = 8.1 Hz, 1H), 6.99 (d, J = 1.9 Hz, 1H), 6.96 (dd, J = 8.1, 2.0 Hz, 1H), 6.64 (t, J = 74.9 Hz, 1H), 4.46 (s, 2H), 3.89 (s, 3H).

^{13}C NMR ($CDCl_3$, 101 MHz): δ 151.31, 140.07 (t, J = 2.9 Hz), 136.50, 122.49, 121.63, 116.15 (t, J = 260.2 Hz), 113.49, 56.17, 33.03.

^{19}F NMR ($CDCl_3$, 376 MHz): δ –81.54 (d, J = 74.9 Hz).

4-(Azidomethyl)-1-(difluoromethoxy)-2-methoxybenzene (S27)

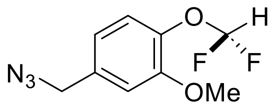

A 4 mL scintillation vial was charged with 4-(bromomethyl)-1-(difluoromethoxy)-2-methoxybenzene **S27–I3** (100 mg, 0.38 mmol, 1.0 equiv) and NaN_3 solution in DMSO (0.2 mL, 2 M in DMSO) (49 mg, 0.76, 2.0 equiv). The resulting reaction mixture was heated at 90 °C overnight. After cooling down to room temperature in the next morning, the reaction mixture diluted with ice-cold H_2O and EtOAc. The aqueous layer was extracted with EtOAc (3 ×5 mL). The combined organics dried (Na_2SO_4) and concentrated under reduced pressure, which was subsequently purified by column chromatography.

Purification: Gradient column chromatography [SiO_2, EtOAc:Hexanes 00:100 to 10:90)] to obtain the titled compound **S27** as colourless liquid (76 mg, 87%).

R_f : 0.5 (EtOAc : Hexanes 0.5:9.5)

^{1}H NMR ($CDCl_3$, 400 MHz): δ 7.16 (d, J = 8.1 Hz, 1H), 6.92 (d, J = 1.9 Hz, 1H), 6.87 (dd, J = 8.1, 1.9 Hz, 1H), 6.64 (t, J = 75.0 Hz, 1H), 4.43 (s, 2H), 3.90 (s, 3H).

^{13}C NMR ($CDCl_3$, 101 MHz): δ 151.50, 139.96 (t, J = 3.2 Hz), 134.27, 122.63, 120.68, 117.48 (t, J = 260.1 Hz), 112.47, 56.18, 54.55.

^{19}F NMR ($CDCl_3$, 376 MHz): δ –81.54 (d, J = 74.9 Hz).

HRMS (ESI-TOF) m/z: $[M + H]^+$ Calcd. for $C_9H_{10}F_2N_3O_2$ 230.0741; found 230.0741; and $[M + Na]^+$ Calcd. for $C_9H_9F_2N_3NaO_2$ 230.0561; found 230.0559.

Authentic fluoroarene standard

^{19}F-Reference compound – **[^{19}F]27**, data are comparable to that reported in the literature.[8]

Radio-HPLC analysis and characterization

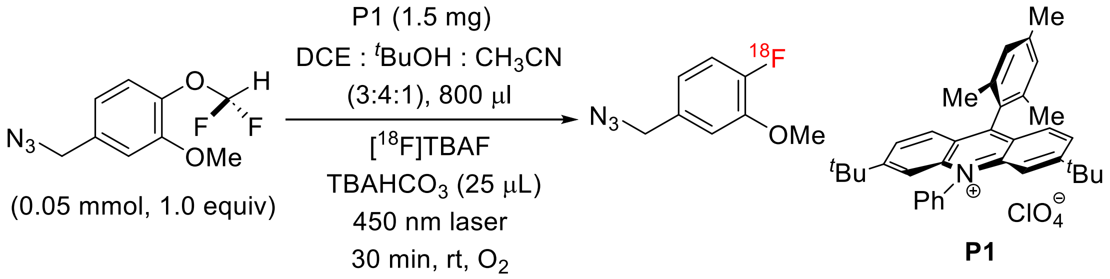

Entry	Activity at starting	Activity at EOS	Alumina Filtration	Injected Dose	Collected Dose	Isolation Time	Decay Corrected	Filtration Yield	HPLC Purity of Filtrate	%Yield
1	14.62 mCi	11.42 mCi	4.43 mCi	544 μCi	218 μCi	14.3 min	497.44 μCi	38.79%	43.82%	16.99%
2	10.05 mCi	7.93 mCi	2.90 mCi	354 μCi	112 μCi	14.3 min	323.02 μCi	36.56%	34.67 %	12.67%
								**Average %Yield: 14.8% (n=2)**		

Table S32: HPLC isolated RCYs for **[^{18}F]27**

HPLC Conditions – method 1: Column: Phenomenex, Kinetex® 5μm F5 100 Å, 250 ✕ 4.6 mm LC Column

Solvent A: 0.1% TFA water, Solvent B: 0.1% TFA acetonitrile; Isocratic / Gradient elution: 20% Solvent B for 0 to 2 min, 20% – 95% Solvent B for 2 to 22.5 min. Flow rate: 1 mL/min

Datafile Name:VIII-Mn-139 20-95.lcd
Sample Name:VIII-Mn-139 20-95
Sample ID:VIII-Mn-139 20-95

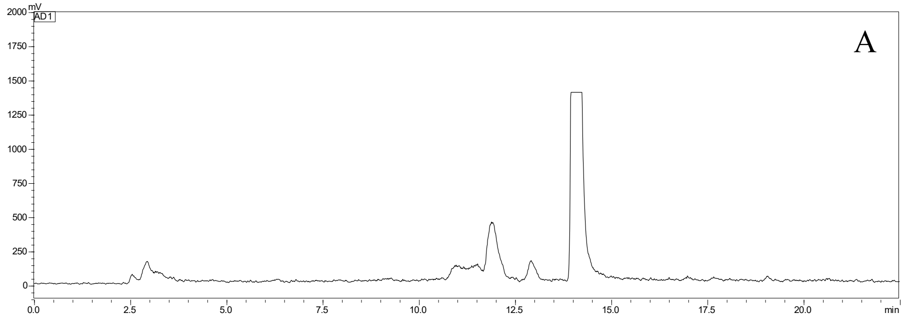

Datafile Name:IX-MN-98 20-95.lcd
Sample Name:IX-MN-98 20-95
Sample ID:IX-MN-98 20-95

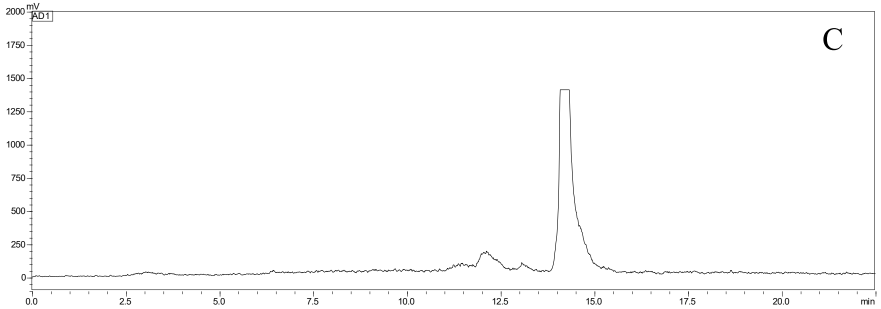

Datafile Name:OMe Bn N3 ref 20-95.lcd
Sample Name:OMe Bn N3 ref 20-95
Sample ID:OMe Bn N3 ref 20-95

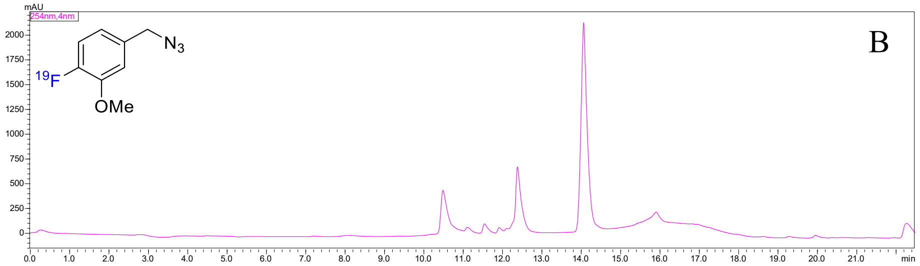

Datafile Name:OMe Bn N3 ref 20-95.lcd
Sample Name:OMe Bn N3 ref 20-95
Sample ID:OMe Bn N3 ref 20-95

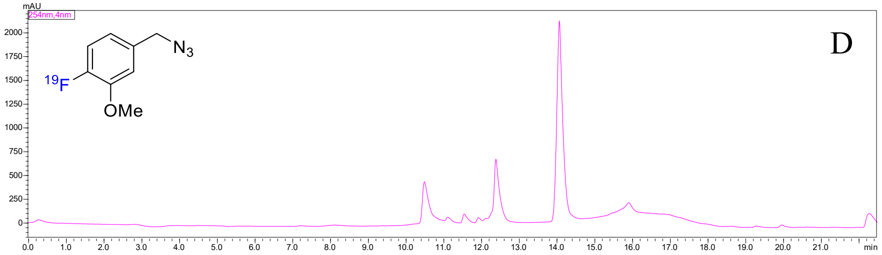

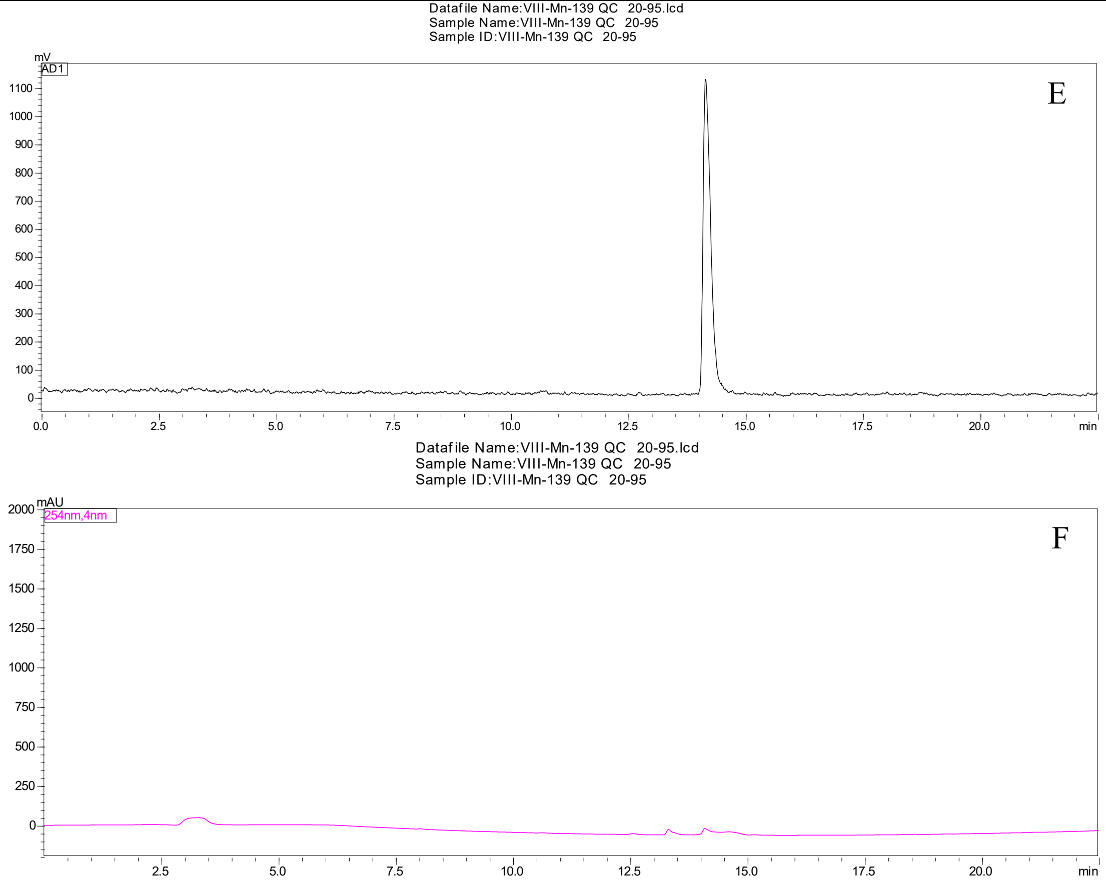

Figure S34: Radio-HPLC analysis of reaction mixture from **S27**. Reaction mixture with HPLC method 1 (A), authentic **[^{19}F]27** with HPLC method 1 (B), reaction mixture with HPLC method 1 (C), authentic **[^{19}F]27** with HPLC method 1 (D), and QC for **[^{18}F]27** (E and F).

HPLC Conditions for QC: Column: Phenomenex, Kinetex® 5µm F5 100 Å, 250 × 4.6 mm LC Column

Solvent A: 0.1% TFA water, Solvent B: 0.1% TFA acetonitrile; Isocratic / Gradient elution: 20% Solvent B for 0 to 2 min, 20% – 95% Solvent B for 2 to 22.5 min. Flow rate: 1 mL/min.

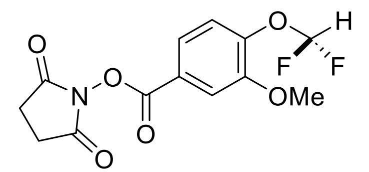

S28

Arene substrate:

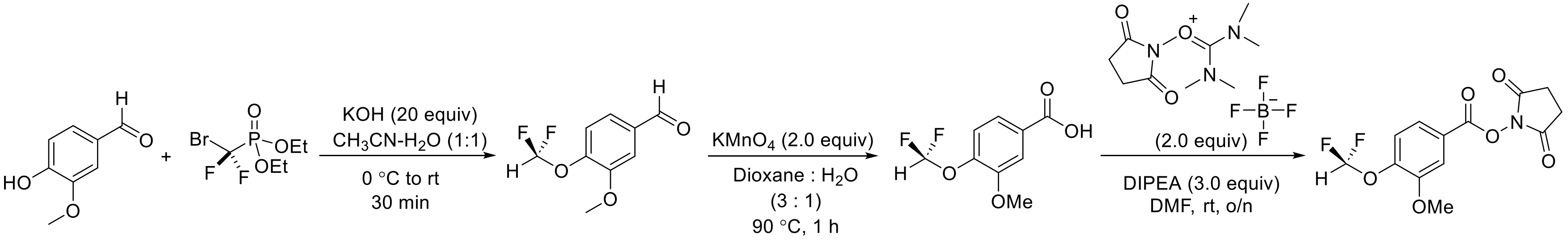

4-(Difluoromethoxy)-3-methoxybenzoic acid (S28-I1)

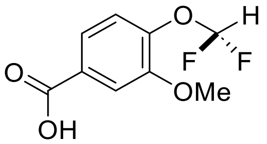

To a solution of 4-(difluoromethoxy)-3-methoxybenzaldehyde **S27–I1** (600 mg, 2.74 mmol, 1.0 equiv) in 1,4-dioxane (6 mL) was added dropwise a solution of $KMnO_4$ (910 mg, 5.76 mmol, 2.1 equiv) in H_2O (3 mL). The resulting reaction heated at 90 °C until the staring material consumed (1 h, TLC monitored). The reaction mixture cooled to room temperature and washed with the saturated solution of $Na_2S_2O_3$ and 1 N HCl. The aqueous layer was extracted with EtOAc (3 ×10 mL). The combined organics dried (Na_2SO_4) and concentrated under reduced pressure, which was subsequently purified by column chromatography.

Purification: Gradient column chromatography [SiO_2, EtOAc:Hexanes 50:50 to 100:0)] to obtain the titled compound **S28-I1** as pale orange solid (99 mg, 17%).

R_f : 0.1 (EtOAc : Hexanes 10:0)

^{1}H NMR (MeOD-d^4, 400 MHz): δ 7.70 (s, 1H), 7.65 (d, J = 8.1 Hz, 1H), 7.21 (d, J = 8.1 Hz, 1H), 6.84 (t, J = 74.6 Hz, 1H), 3.92 (s, 3H).

^{13}C NMR (MeOD-d^4, 101 MHz): δ 168.80, 152.06, 145.30 (t, J = 2.8 Hz), 123.97, 121.90, 118.73 (t, J = 258.3 Hz), 114.89, 56.55.

^{19}F NMR (MeOD-d^4, 376 MHz): δ –83.76 (d, J = 74.1 Hz).

HRMS (ESI-TOF) m/z: $[M + H]^+$ Calcd. for $C_9H_9F_2O_4$ 219.0469; found 219.0468.

2,5-Dioxopyrrolidin-1-yl 4-(difluoromethoxy)-3-methoxybenzoate (S28)

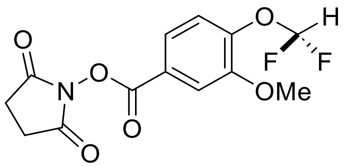

A 4 mL scintillation vial was charged with 4-(difluoromethoxy)-3-methoxybenzoic acid **S28-I1** (90 mg, 0.41 mmol, 1.0 equiv), 2-(2,5-dioxopyrrolidin-1-yl)-1,1,3,3-tetramethyluronium tetrafluoroborate – TSTU (250 mg, 0.83 mmol, 2.0 equiv). The screw-capped vial was purged and degassed with argon for three times. Anhydrous DMF (1.6 mL, [0.25 M]) followed by DIPEA (0.21 mL, 1.23 mmol, 3.0 equiv) were transferred by syringe and the screw-capped tube was stirred at room temperature overnight. Next morning, the reaction mixture diluted with ice-cold H_2O and EtOAc. The aqueous layer was extracted with EtOAc (3 × 5 mL). The combined organics dried (Na_2SO_4) and concentrated under reduced pressure, which was subsequently purified by column chromatography.

Purification: Gradient column chromatography [SiO_2, EtOAc:Hexanes 10:90 to 30:70)] to obtain the titled compound **S28** as pale-yellow solid (98 mg, 39%).

R_f : 0.4 (EtOAc : Hexanes 3:7)

^{1}H NMR ($CDCl_3$, 400 MHz): δ 7.77 (dd, J = 8.4, 1.8 Hz, 1H), 7.68 (d, J = 1.8 Hz, 1H), 7.25 (d, J = 8.4 Hz, 1H), 6.75 (t, J = 74.0 Hz, 1H), 3.94 (s, 3H), 2.92 (bs, 4H).

^{13}C NMR ($CDCl_3$, 101 MHz): δ 169.28, 161.19, 151.07, 145.39 (t, J = 3.2 Hz), 124.30, 123.00, 121.44, 115.60 (t, J = 262.2 Hz), 56.43, 25.78.

^{19}F NMR ($CDCl_3$, 376 MHz): δ –82.04 (d, J = 78.3 Hz).

HRMS (ESI-TOF) m/z: $[M + Na]^+$ Calcd. for $C_{13}H_{11}F_2NNaO_6$ 338.0452; found 338.0449.

Authentic fluoroarene standard

2,5-Dioxopyrrolidin-1-yl 4-fluoro-3-methoxybenzoate ([^{19}F]28)

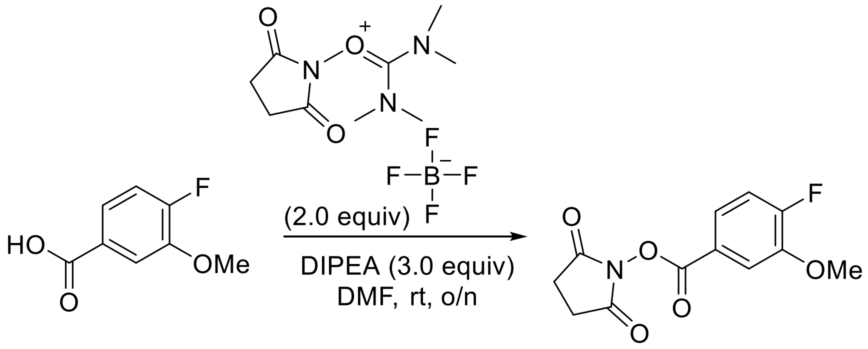

A 4 mL scintillation vial was charged with 4-fluoro-3-methoxybenzoic acid (50 mg, 0.29 mmol, 1.0 equiv), 2-(2,5-dioxopyrrolidin-1-yl)-1,1,3,3-tetramethyluronium tetrafluoroborate – TSTU (174 mg, 0.58 mmol, 2.0 equiv). The screw-capped vial was purged and degassed with argon for three times. Anhydrous DMF (0.97 mL, [0.3 M]) followed by DIPEA (0.152 mL, 1.23 mmol, 3.0 equiv) were transferred by syringe and the screw-capped tube was stirred at room temperature overnight. Next morning, the reaction mixture diluted with ice-cold H_2O and EtOAc. The aqueous layer was extracted with EtOAc (3 × 5 mL). The combined organics dried (Na_2SO_4) and concentrated under reduced pressure, which was subsequently purified by column chromatography.

Purification: Gradient column chromatography [SiO_2, EtOAc:Hexanes 10:90 to 30:70: 50:50)] to obtain the titled compound **[^{19}F]28** as white solid (59 mg, 77%).

R_f : 0.4 (EtOAc : Hexanes 3:7)

^{1}H NMR ($CDCl_3$, 400 MHz): δ 7.76 (ddd, J = 6.4, 4.3, 2.1 Hz, 1H), 7.68 (dd, J = 8.0, 2.0 Hz, 1H), 7.19 (dd, J = 10.6, 8.5 Hz, 1H), 3.93 (s, 3H), 2.90 (bs, 4H).

^{13}C NMR ($CDCl_3$, 101 MHz): δ 169.37, 161.16, 158.10, 155.54, 148.26 (d, J= 11.0 Hz), 124.67 (d, J= 8.5 Hz), 121.55 (d, J= 3.6 Hz), 116.69 (d, J= 19.6 Hz), 115.25 (d, J= 3.6 Hz), 56.50, 25.78.

^{19}F NMR ($CDCl_3$, 376 MHz): δ –123.14 (ddd, J = 10.2, 6.3, 3.6 Hz).

HRMS (ESI-TOF) m/z: $[M + Na]^+$ Calcd. for $C_{12}H_{10}FNNaO_5$ 290.0441; found 290.0439.

Radio-HPLC analysis and characterization

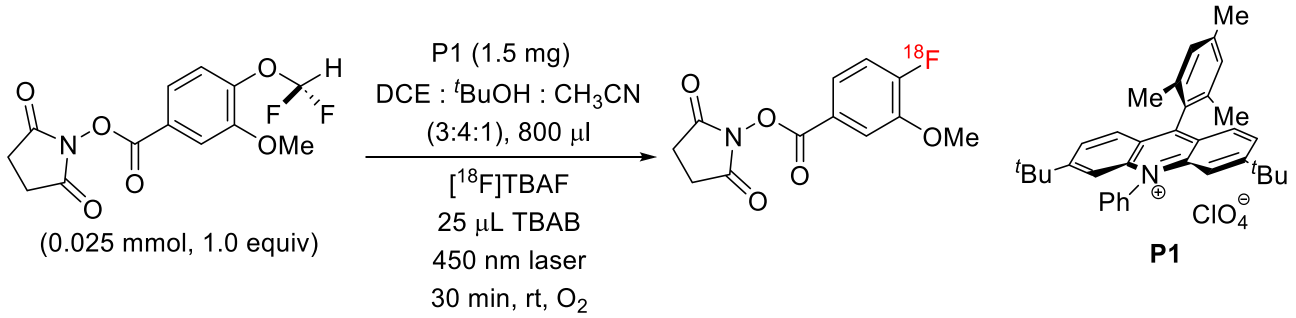

Note: (Substrate **S28** is base sensitive.TBAB must be added just before the reaction)

Entry	Activity at starting	Activity at EOS	Alumina Filtration	Injected Dose	Collected Dose	Isolation Time	Decay Corrected	Filtration Yield	HPLC Purity of Filtrate	%Yield
1	19.09 mCi	15.26 mCi	3.90 mCi	427 µCi	91 µCi	11.9 min	395.83 µCi	25.56%	22.98%	5.87%
2	11.81 mCi	9.45 mCi	1.60 mCi	863 µCi	147 µCi	11.9 min	804.32 µCi	16.94%	18.27%	3.09%
										**Average %Yield: 4.5% (n=2)**

Table S33: HPLC isolated RCYs for **[^{18}F]28**

HPLC Conditions – method 1: Column: Phenomenex, Kinetex® 5µm F5 100 Å, 250 ✕ 4.6 mm LC Column

Solvent A: 0.1% TFA water, Solvent B: 0.1% TFA acetonitrile; Isocratic / Gradient elution: 20% Solvent B for 0 to 2 min, 20% – 95% Solvent B for 2 to 22.5 min. Flow rate: 1 mL/min

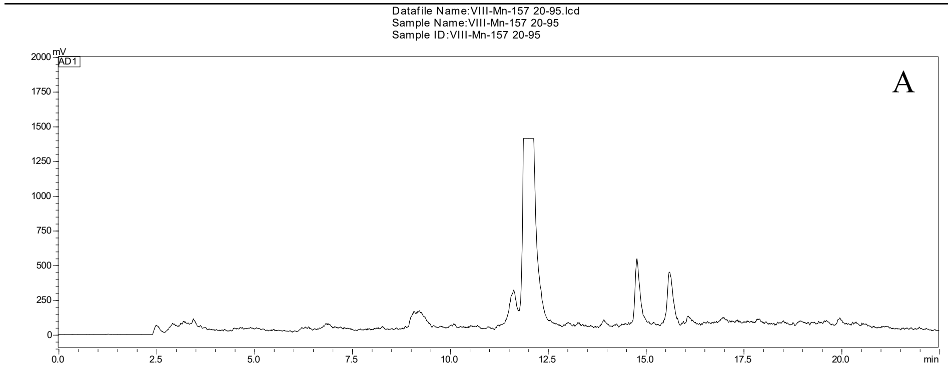
Datafile Name:VIII-Mn-157 20-95.lcd
Sample Name:VIII-Mn-157 20-95
Sample ID:VIII-Mn-157 20-95
A

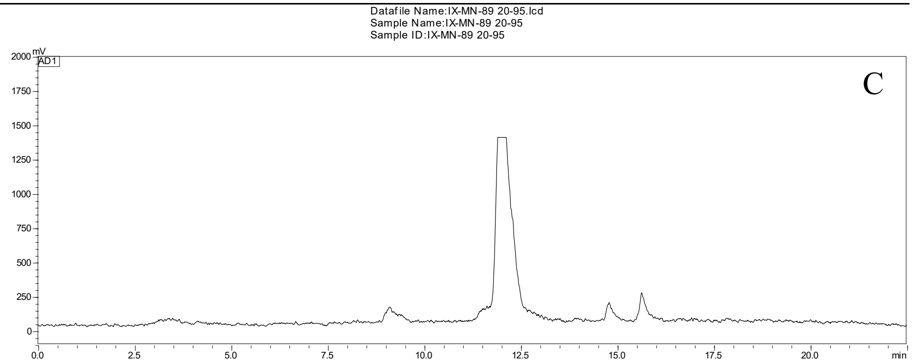
Datafile Name:IX-MN-89 20-95.lcd
Sample Name:IX-MN-89 20-95
Sample ID:IX-MN-89 20-95
C

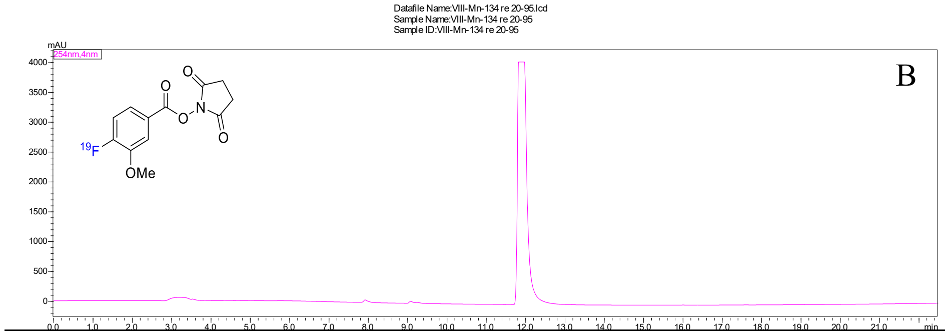
Datafile Name:VIII-Mn-134 re 20-95.lcd
Sample Name:VIII-Mn-134 re 20-95
Sample ID:VIII-Mn-134 re 20-95
B

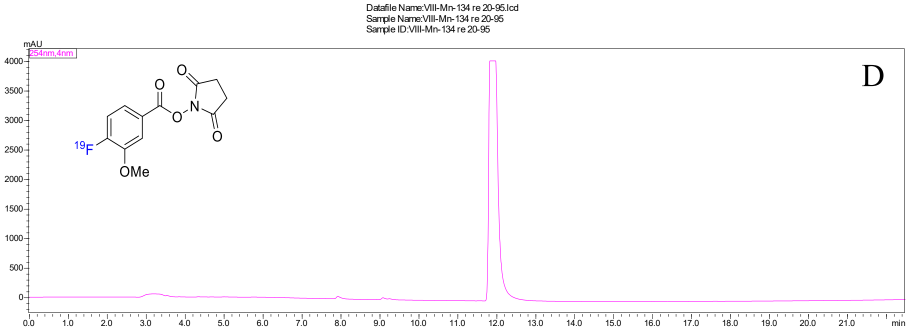
Datafile Name:VIII-Mn-134 re 20-95.lcd
Sample Name:VIII-Mn-134 re 20-95
Sample ID:VIII-Mn-134 re 20-95
D

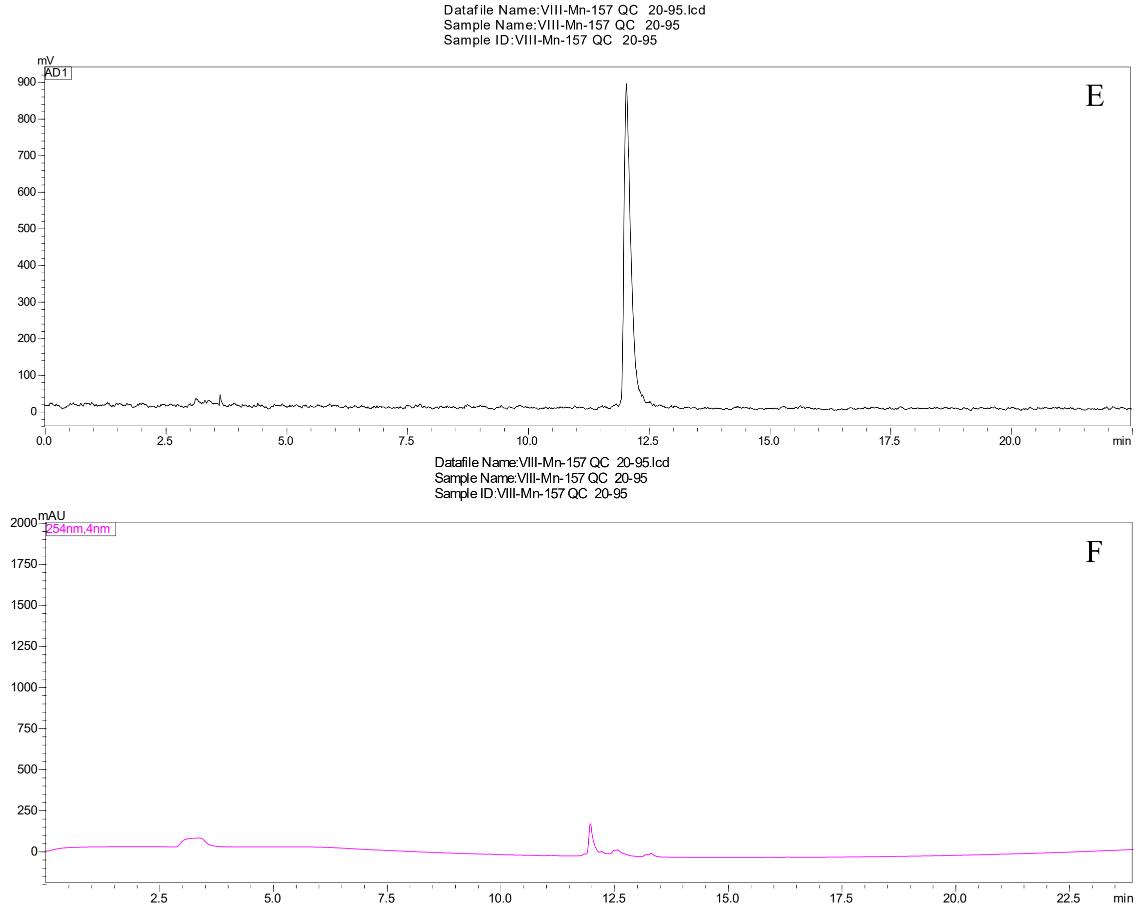

Figure S35: Radio-HPLC analysis of reaction mixture from **S28**. Reaction mixture with HPLC method 1 (A), authentic **[^{19}F]28** with HPLC method 1 (B), reaction mixture with HPLC method 1 (C), authentic **[^{19}F]28** with HPLC method 1 (D), and QC for **[^{18}F]28** (E and F).

HPLC Conditions for QC: Column: Phenomenex, Kinetex® 5µm F5 100 Å, 250 × 4.6 mm LC Column

Solvent A: 0.1% TFA water, Solvent B: 0.1% TFA acetonitrile; Isocratic / Gradient elution: 20% Solvent B for 0 to 2 min, 20% – 95% Solvent B for 2 to 22.5 min. Flow rate: 1 mL/min.

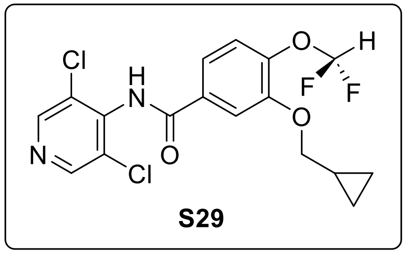

S29

Radio-HPLC analysis

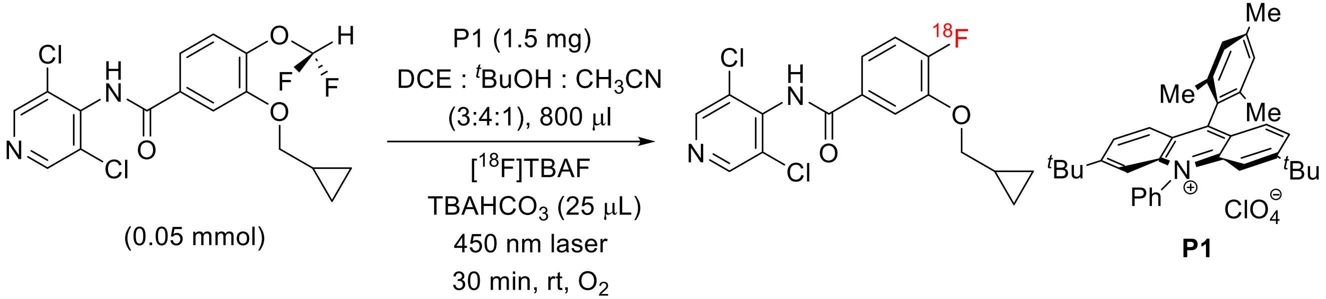

Entry	Activity at starting	Activity at EOS	Alumina Filtration	Injected Dose	Collected Dose	Isolation Time	Decay Corrected	Filtration Yield	HPLC Purity of Filtrate	%Yield
1	14.49 mCi	8.15 mCi	1.35 mCi	263 µCi	33 µCi	14.8 min	239.23 µCi	16.62%	13.79%	2.29%

Table S34: HPLC isolated RCYs for **[^{18}F]29**

HPLC Conditions method – 1: Column: Phenomenex, Kinetex® 5µm F5 100 Å, 250 ✕ 4.6 mm LC Column

Solvent A: 0.1% TFA water, Solvent B: 0.1% TFA acetonitrile; Isocratic / Gradient elution: 20% Solvent B for 0 to 2 min, 20% – 95% Solvent B for 2 to 22.5 min. Flow rate: 1 mL/min

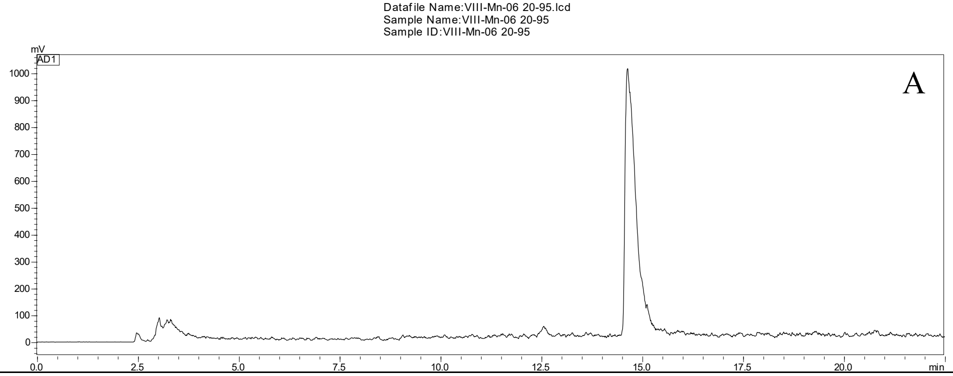
Datafile Name:VIII-Mn-06 20-95.lcd
Sample Name:VIII-Mn-06 20-95
Sample ID:VIII-Mn-06 20-95
mV
AD1
A
min

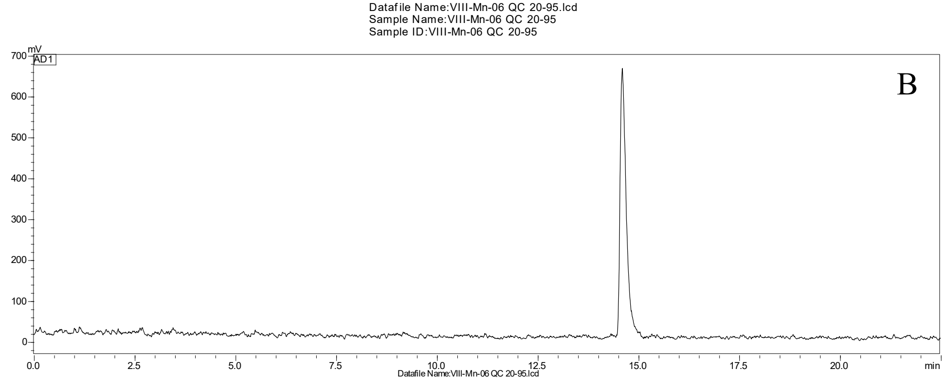
Datafile Name:VIII-Mn-06 QC 20-95.lcd
Sample Name:VIII-Mn-06 QC 20-95
Sample ID:VIII-Mn-06 QC 20-95
mV
AD1
B
min

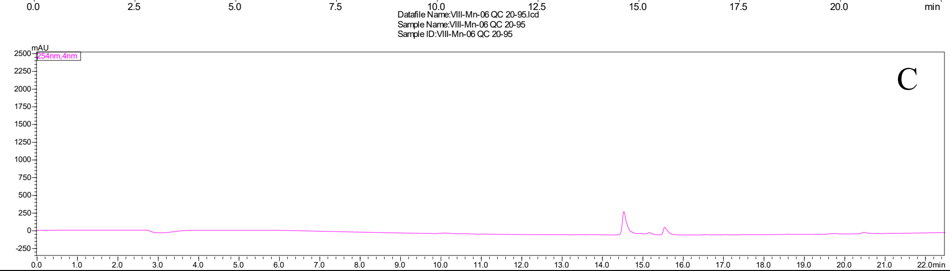
Datafile Name:VIII-Mn-06 QC 20-95.lcd
Sample Name:VIII-Mn-06 QC 20-95
Sample ID:VIII-Mn-06 QC 20-95
mAU
254nm,4nm
C
min

Figure S36: Radio-HPLC analysis of reaction mixture from **S29**. Reaction mixture with HPLC method 1 (A), and QC for **[^{18}F]29** (B and C)

HPLC Conditions for QC: Column: Phenomenex, Kinetex® 5µm F5 100 Å, 250 × 4.6 mm LC Column

Solvent A: 0.1% TFA water, Solvent B: 0.1% TFA acetonitrile; Isocratic / Gradient elution: 20% Solvent B for 0 to 2 min, 20% – 95% Solvent B for 2 to 22.5 min. Flow rate: 1 mL/min.

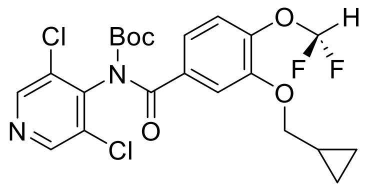

S30

Arene substrate:

***tert*-Butyl (3-(cyclopropylmethoxy)-4-(difluoromethoxy)benzoyl)(3,5-dichloropyridin-4-yl)carbamate (S30)**

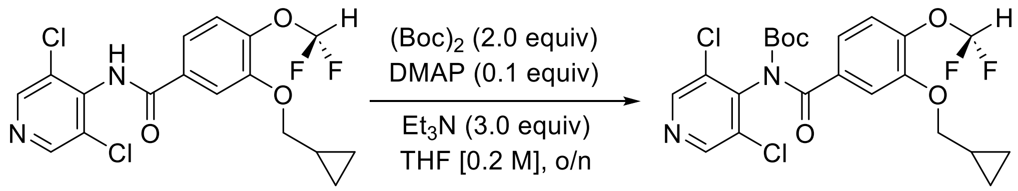

A 20 mL scintillation vial was charged with Roflumilast **S29** (403 mg, 1.0 mmol, 1.0 equiv), DMAP (12 mg, 0.1 mmol, 0.1 equiv). Anhydrous THF (5 mL, [0.2 M]) followed by Et_3N (0.42 mL, 3.0 mmol, 3.0 equiv) and $(Boc)_2O$ (0.46, 2.0 mmol, 2.0 equiv) were transferred by syringe and the reaction mixture was stirred at room temperature overnight. Next morning, the reaction mixture diluted with EtOAc and H_2O. The

aqueous layer was extracted with EtOAc (3 × 5 mL). The combined organics dried (Na_2SO_4) and concentrated under reduced pressure, which was subsequently purified by column chromatography.

Purification: Gradient column chromatography [SiO_2, EtOAc:Hexanes 5:95 to 30:70)] to obtain the titled compound **S30** as white solid (362 mg, 72%).

R_f : 0.6 (EtOAc : Hexanes 1:9)

^{1}H NMR ($CDCl_3$, 400 MHz): δ 8.62 (s, 2H), 7.39 (dd, J = 8.2, 19 Hz, 1H), 7.37 (d, J = 1.9 Hz, 1H), 7.24 (d, J = 8.2 Hz, 1H), 6.82 (t, J = 74.9 Hz, 1H), 3.92 (d, J = 6.9 Hz, 2H), 1.35–1.23 (m, 1H), 1.30 (s, 9H), 0.67 (dt, J = 8.0, 1.8 Hz, 2H), 0.37 (dt, J = 5.8, 4.8 Hz, 2H).

^{13}C NMR ($CDCl_3$, 101 MHz): δ 169.29, 150.48, 150.34, 148.74, 143.48 (t, J= 2.9 Hz), 142.33, 133.29, 131.92, 121.82 (d, J= 16.6 Hz), 115.88 (t, J= 260.1 Hz), 114.61, 85.41, 74.30, 27.53, 10.14, 3.41.

^{19}F NMR ($CDCl_3$, 376 MHz): δ –81.94 (d, J = 74.8 Hz).

HRMS (ESI-TOF) m/z: $[M + H]^+$ Calcd. for $C_{22}H_{23}Cl_2F_2N_2O_5$ 503.0952; found 503.0953 and $[M + Na]^+$ Calcd. for $C_{22}H_{22}Cl_2F_2N_2NaO_5$ 525.0772; found 525.0773.

<u>Deoxyfluorination and Authentic fluoroarene standard</u>

***tert*-Butyl (3-(cyclopropylmethoxy)-4-fluorobenzoyl)(3,5-dichloropyridin-4-yl)carbamate ([^{19}F]30)**

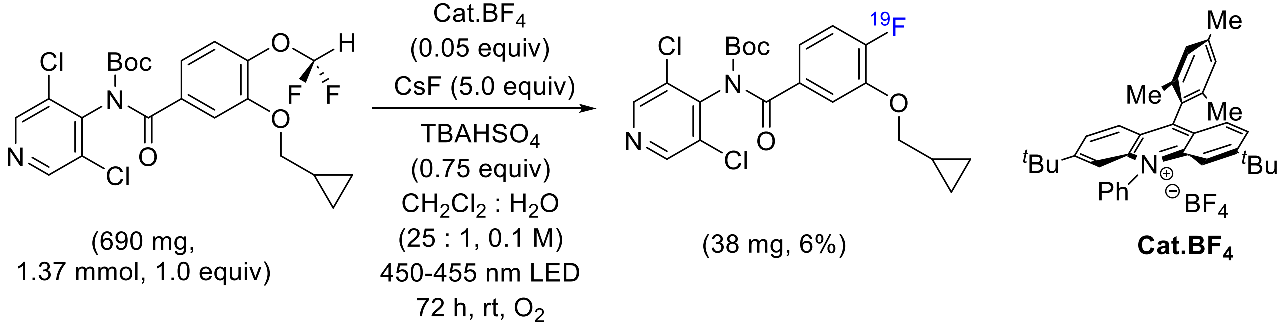

General conditions **I** were followed using **S30** (690 mg, 1.37 mmol, 1.0 equiv), Mes-Acr-Ph$^+$BF$_4^-$ (0.05 equiv), CsF (5.0 equiv), $TBAHSO_4$ (0.75 equiv), CH_2Cl_2 : H_2O (0.1 M, 25:1), 450-455 nm LED, 72 h, 33 °C, O_2.

Purification: Isocratic column chromatography [SiO_2, EtOAc:Hexanes 10:90] to obtain the titled compound **[^{19}F]30** as colourless semisolid (38 mg, 6%).

R$_f$: 0.7 (EtOAc : Hexanes 1:9)

^{1}H NMR ($CDCl_3$, 400 MHz): δ 8.62 (s, 2H), 7.40–7.36 (m, 2H), 7.15 (dd, J = 10.6, 8.9 Hz, 1H), 3.92 (d, J = 6.9 Hz, 2H), 1.35–1.28 (m, 1H) 1.31 (s, 9H), 0.69–0.64 (m, 2H), 0.39–0.35 (m, 2H).

^{13}C NMR ($CDCl_3$, 101 MHz): δ 169.26, 156.66, 154.13, 150.39, 148.69, 147.28 (d, J = 11.5 Hz), 142.48, 131.97, 131.43 (d, J = 3.6 Hz), 122.08 (d, J = 7.9 Hz), 116.12, 115.92, 115.70 (d, J = 2.9 Hz), 85.32, 74.63, 27.58, 10.23, 3.49.

^{19}F NMR ($CDCl_3$, 376 MHz): δ –126.46 (ddd, J = 11.1, 6.9, 4.2 Hz).

HRMS (ESI-TOF) m/z: [M + H]$^+$ Calcd. for $C_{21}H_{22}Cl_2FN_2O_4$ 455.0941; found 455.0943.

Note: The compound **[^{19}F]30** has minute inseparable starting material **S30** impurity.

Radio-HPLC analysis and characterization

0.05 mmol Scale

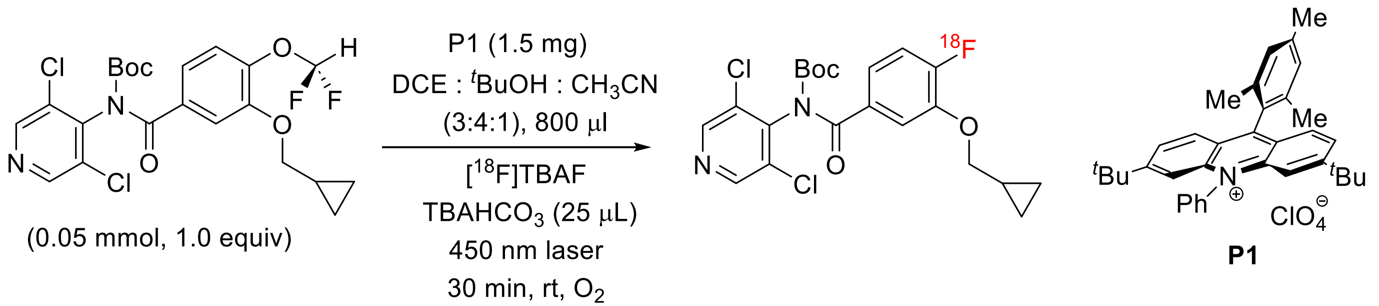

Entry	Activity at starting	Activity at EOS	Alumina Filtration	Injected Dose	Collected Dose	Isolation Time	Decay Corrected	Filtration Yield	HPLC Purity of Filtrate	%Yield
1	4.17 mCi	3.15 mCi	-	474 μCi	141 μCi	17.1 min	425.75 μCi	-	-	33.12%
2	20.07 mCi	16.54 mCi	7.96 mCi	2504 μCi	1413 μCi	17.1 min	2262.18 μCi	48.12%	62.46%	30.05%
										**Average %Yield: 31.6% (n=2)**

Table S35: HPLC isolated RCYs for **[^{18}F]30**

HPLC Conditions – method 1: Column: Phenomenex, Kinetex® 5μm F5 100 Å, 250 ✕ 4.6 mm LC Column

Solvent A: 0.1% TFA water, Solvent B: 0.1% TFA acetonitrile; Isocratic / Gradient elution: 40% Solvent B for 0 to 2 min, 40% – 95% Solvent B for 2 to 22.5 min. Flow rate: 1 mL/min

Datafile Name:V-Mn-47 40-95.lcd
Sample Name:V-Mn-47 40-95
Sample ID:V-Mn-47 40-95

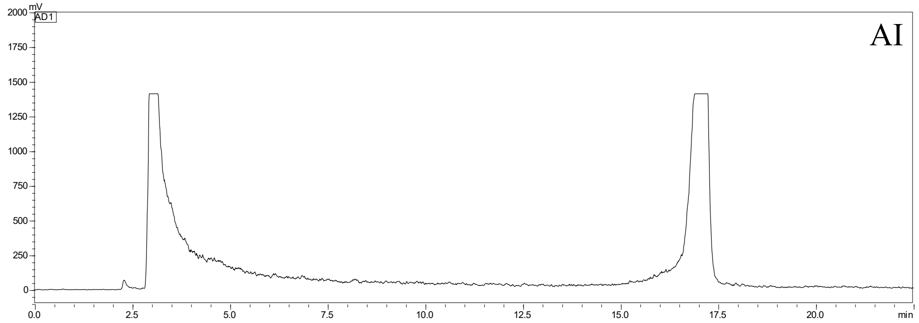

Datafile Name:VI-Mn-57 40-95.lcd
Sample Name:VI-Mn-57 40-95
Sample ID:VI-Mn-57 40-95

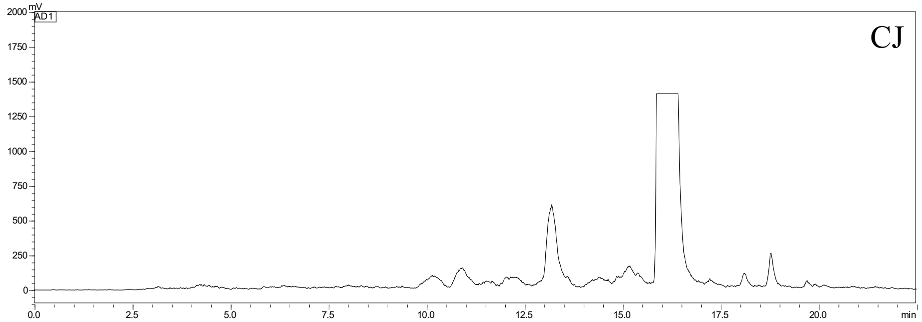

Datafile Name:V-Mn-53 ii 40-95 07-06-2024.lcd
Sample Name:V-Mn-53 ii 40-95 07-06-2024
Sample ID:V-Mn-53 ii 40-95 07-06-2024

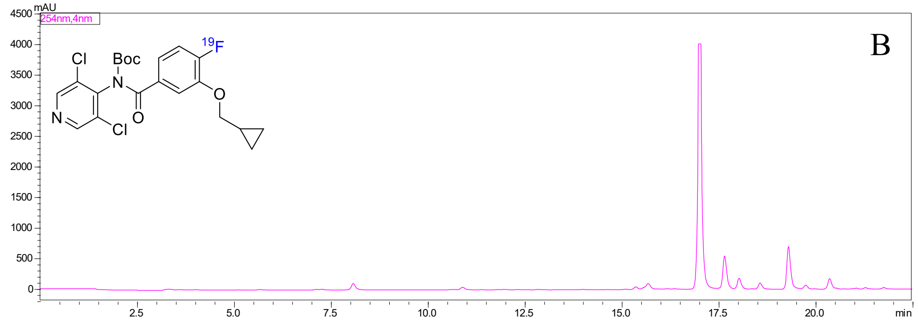

Datafile Name:V-Mn-53 ii 40-95 07-06-2024.lcd
Sample Name:V-Mn-53 ii 40-95 07-06-2024
Sample ID:V-Mn-53 ii 40-95 07-06-2024

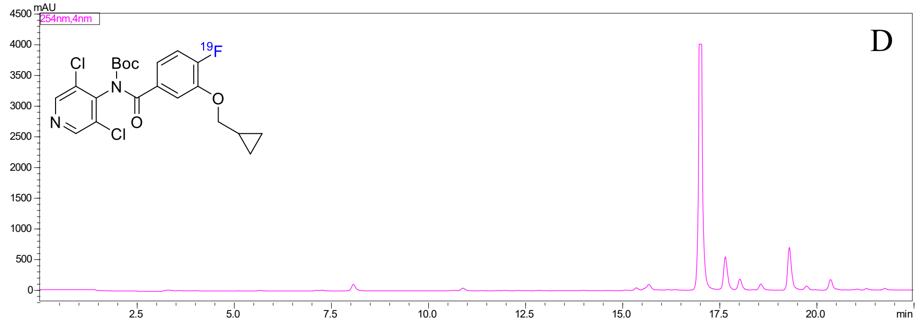

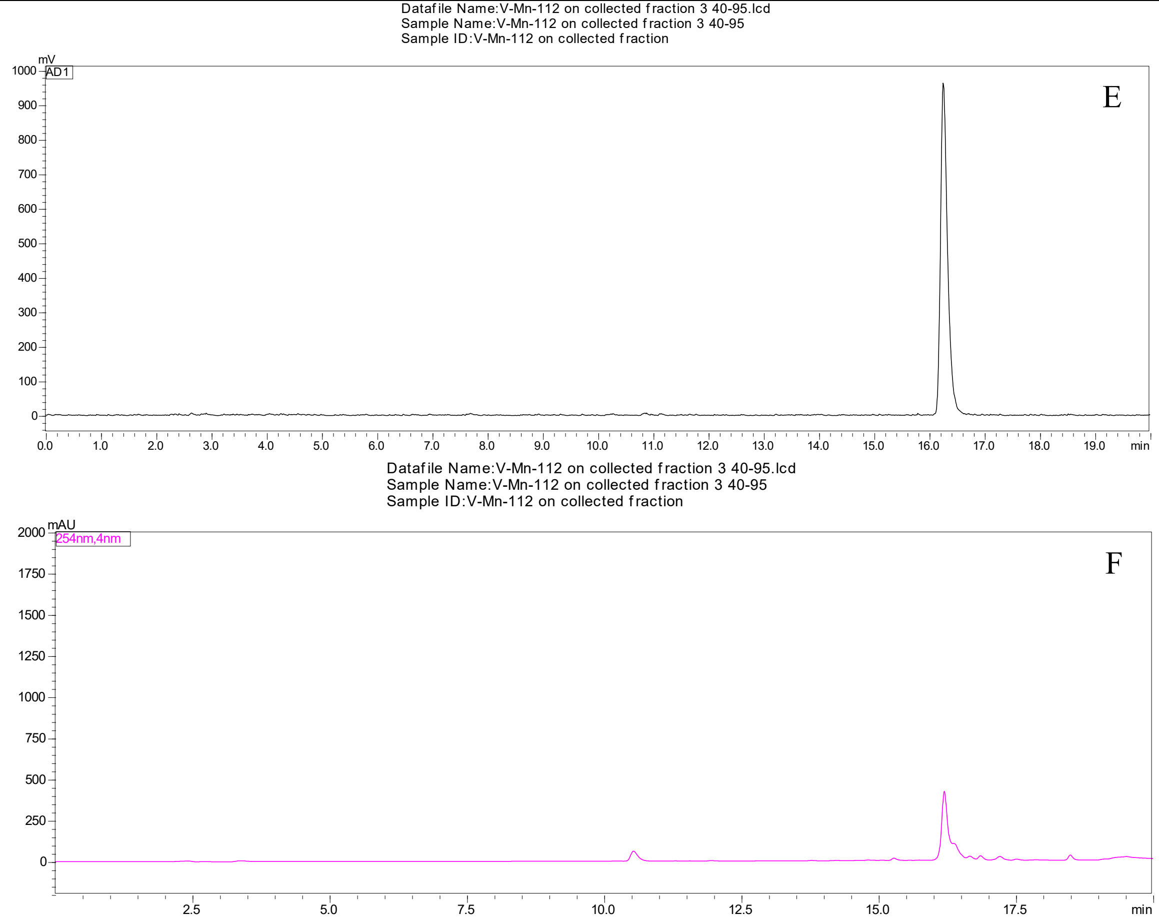

Figure S37: Radio-HPLC analysis of reaction mixture from **S30**. Reaction mixture with HPLC method 1 (A), authentic **[^{19}F]30** with HPLC method 1 (B), reaction mixture with HPLC method 1 (C), authentic **[^{19}F]30** with HPLC method 1 (D), and QC for **[^{18}F]30** (E and F).

HPLC Conditions for QC: Column: Phenomenex, Kinetex® 5µm F5 100 Å, 250 × 4.6 mm LC Column

Solvent A: 0.1% TFA water, Solvent B: 0.1% TFA acetonitrile; Isocratic / Gradient elution: 40% Solvent B for 0 to 2 min, 40% – 95% Solvent B for 2 to 22.5 min. Flow rate: 1 mL/min.

0.01mmol Scale

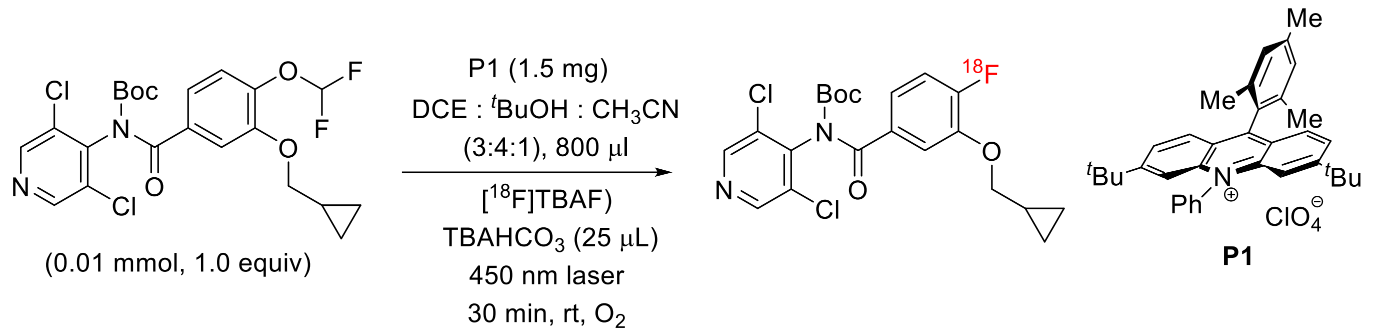

Entry	Activity at starting	Activity at EOS	Alumina Filtration	Injected Dose	Collected Dose	Isolation Time	Decay Corrected	Filtration Yield	HPLC Purity of Filtrate	%Yield
1	8.10 mCi	6.16 mCi	1.032 mCi	814 µCi	582 µCi	17.1 min	735.77 µCi	16.75%	79.10%	13.24%
2	4.38 mCi	3.63 mCi	595 µCi	423 µCi	301 µCi	17.1 min	382.35 µCi	16.39%	78.72%	12.90%
										**Average %Yield: 13.0% (n=2)**

Table S36: HPLC isolated RCYs for **[^{18}F]30**

HPLC Conditions: Column: Phenomenex, Kinetex® 5µm F5 100 Å, 250 × 4.6 mm LC Column

Solvent A: 0.1% TFA water, Solvent B: 0.1% TFA acetonitrile; Isocratic / Gradient elution: 40% Solvent B for 0 to 2 min, 40% – 95% Solvent B for 2 to 22.5 min. Flow rate: 1 mL/min

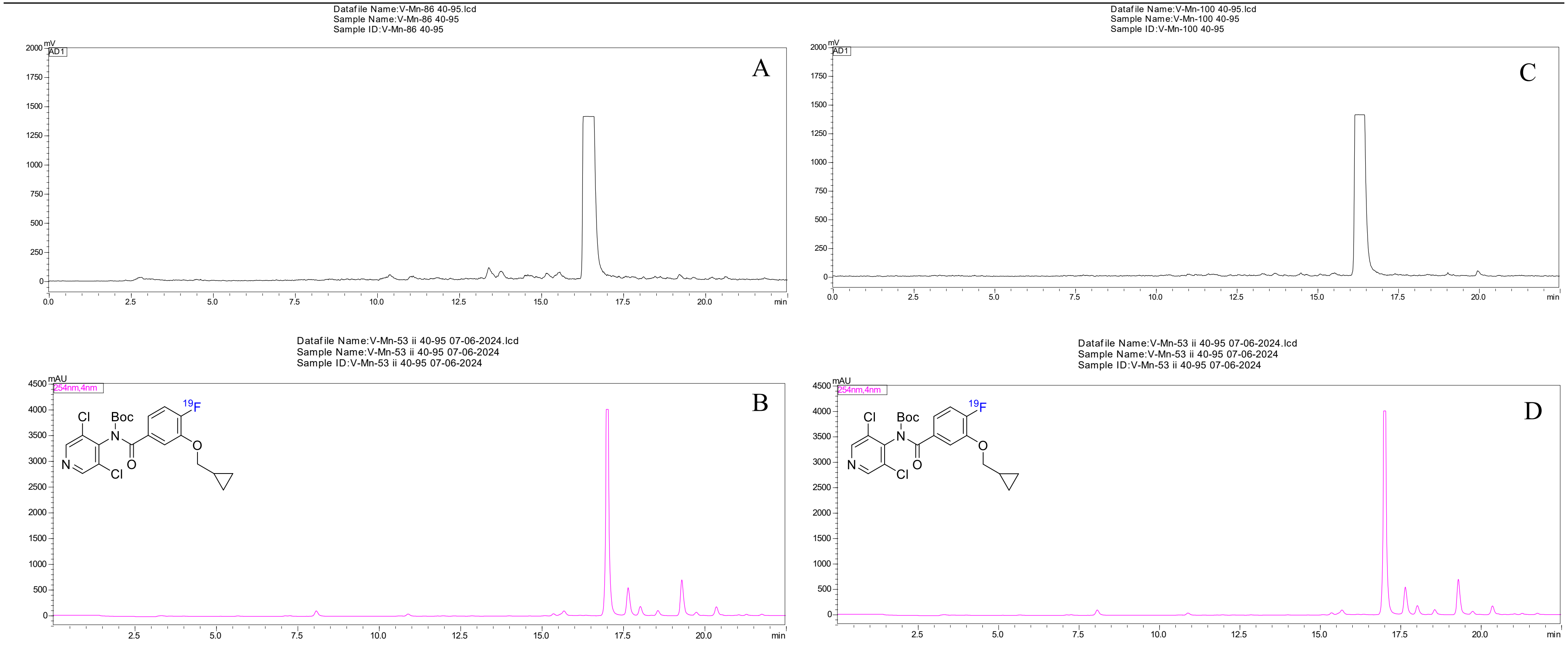

Figure S38: Radio-HPLC analysis of reaction mixture from **S30**. Reaction mixture with HPLC method 1 (A), authentic **[^{19}F]30** with HPLC method 1 (B), reaction mixture with HPLC method 1 (C), and authentic **[^{19}F]30** with HPLC method 1 (D).

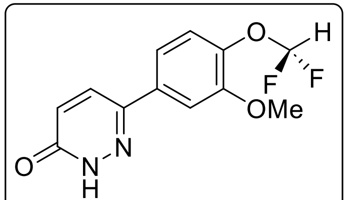

S31

Authentic fluoroarene standard

6-(4-Fluoro-3-methoxyphenyl)pyridazin-3(*2H*)-one ([^{19}F]31)

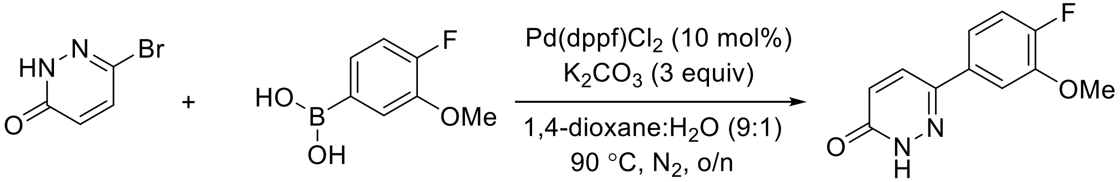

A 50 mL pressure tube was charged with 6-bromopyridazin-3(*2H*)-one (875 mg, 5.0 mmol, 1.0 equiv), (4-fluoro-3-methoxyphenyl)boronic acid (935 mg, 5.5 mmol, 1.0 equiv), K_2CO_3 (2.0 g, 12 mmol, 3.0 equiv), and Pd(dppf)Cl_2 (366 mg, 0.5 mmol, 1.0 equiv). The tube was purged and degassed with nitrogen for three times. Degassed 1,4-dioxane : H_2O (9:1) [0.5 M] was transferred by syringe and the screw-capped tube was heated at 90 °C overnight. Next morning, the reaction mixture cooled down to room temperature and diluted with EtOAc and H_2O. The aqueous layer was extracted with EtOAc (2 × 10 mL). The combined organics dried (Na_2SO_4) and concentrated under reduced pressure, which was subsequently purified by column chromatography.

Purification: Gradient column chromatography [SiO_2, EtOAc:Hexanes 70:30 to 100:00)] to obtain the titled compound **[^{19}F]31** as white powder (803 mg, 73%).

R_f : 0.4 (EtOAc : Hexanes 100:00)

^{1}H NMR (MeOD-d^4, 400 MHz): δ 8.03 (d, J = 9.9 Hz, 1H), 7.61 (dd, J = 8.2 Hz, 1H), 7.42 (ddd, J = 6.4, 4.2, 2.2 Hz, 1H), 7.20 (dd, J = 11.0, 8.5 Hz, 1H), 7.06 (d, J = 9.9 Hz, 1H), 3.94 (s, 3H).

^{13}C NMR (CDCl$_3$, 101 MHz): δ 163.27, 155.97, 149.54 (d, J= 10.9 Hz), 146.49, 133.49, 132.69 (d, J= 3.6 Hz), 130.80, 119.97 (d, J= 7.3 Hz), 117.17 (d, J= 18.9 Hz), 112.37 (d, J= 2.4 Hz), 56.76.

^{19}F NMR (CDCl$_3$, 376 MHz): δ –135.59 (ddd, J = 11.1, 6.9, 4.2 Hz).

HRMS (ESI-TOF) m/z: [M + H]$^+$ Calcd. for $C_{11}H_{10}FN_2O_2$ 221.0726; found 221.0727.

Radio-HPLC analysis and characterization

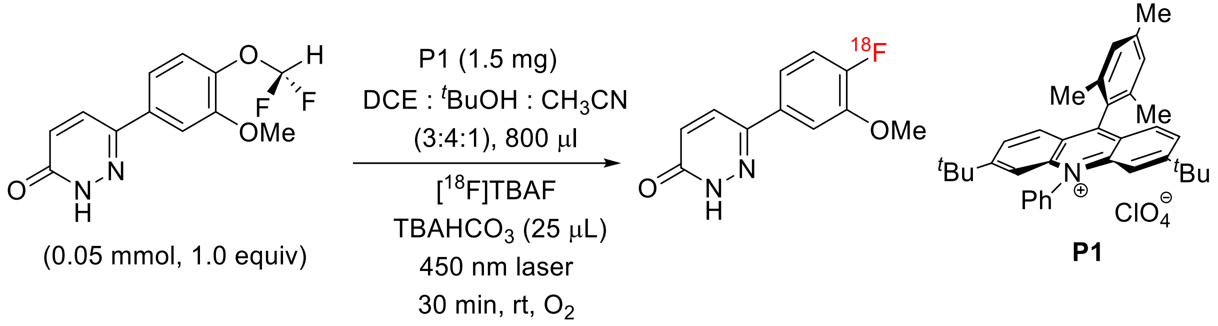

Entry	Activity at starting	Activity at EOS	Alumina Filtration	Injected Dose	Collected Dose	Isolation Time	Decay Corrected	Filtration Yield	HPLC Purity of Filtrate	%Yield
1	8.84 mCi	6.82 mCi	3.26 mCi	624 µCi	197 µCi	12.2 min	578.34 µCi	47.80 %	34.06 %	16.28%
2	8.53 mCi	6.71 mCi	3.31 µCi	471 µCi	135 µCi	12.2 min	436.53 µCi	49.32%	30.83%	15.20%
										**Average %Yield: 15.7% (n=2)**

Table S37: HPLC isolated RCYs for **[^{18}F]31**

HPLC Conditions – method 1: Column: Phenomenex, Kinetex® 5µm F5 100 Å, 250 ✕ 4.6 mm LC Column

Solvent A: 0.1% TFA water, Solvent B: 0.1% TFA acetonitrile; Isocratic / Gradient elution: 5% Solvent B for 0 to 2 min, 5% – 95% Solvent B for 2 to 22.5 min. Flow rate: 1 mL/min

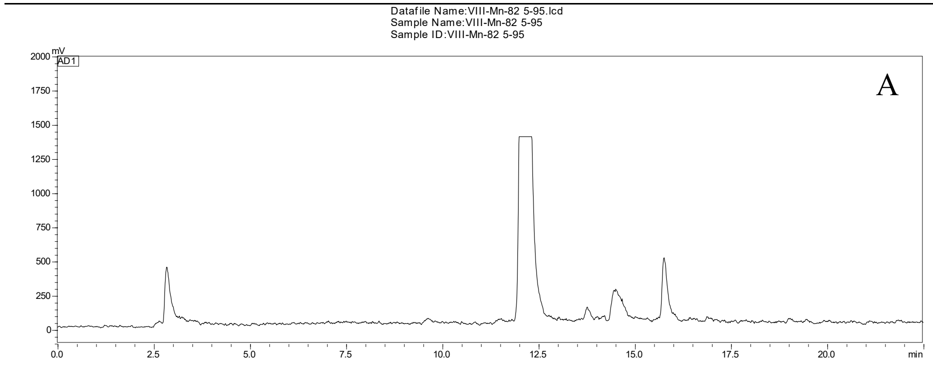

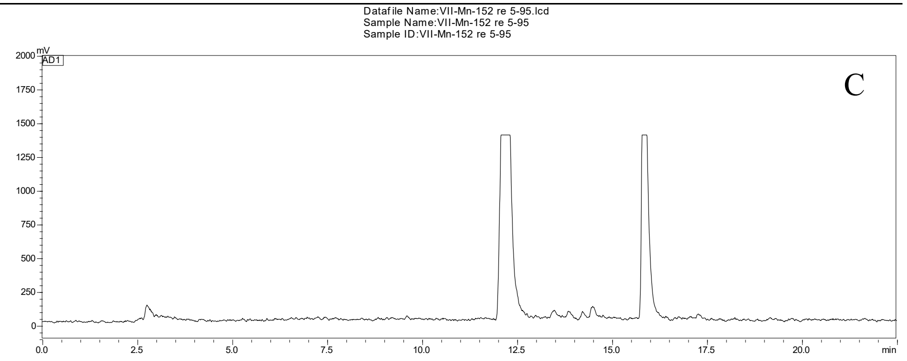

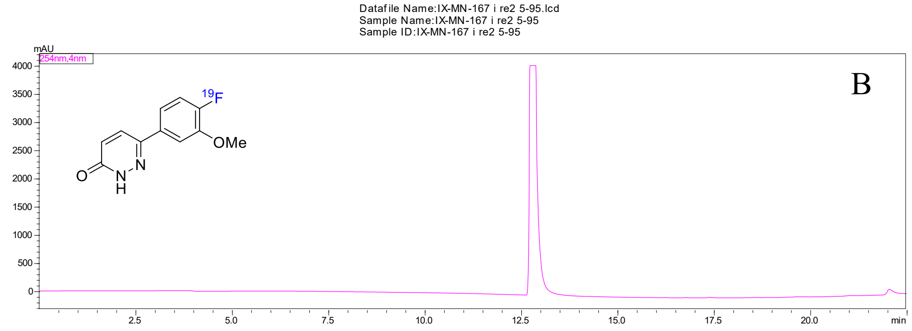

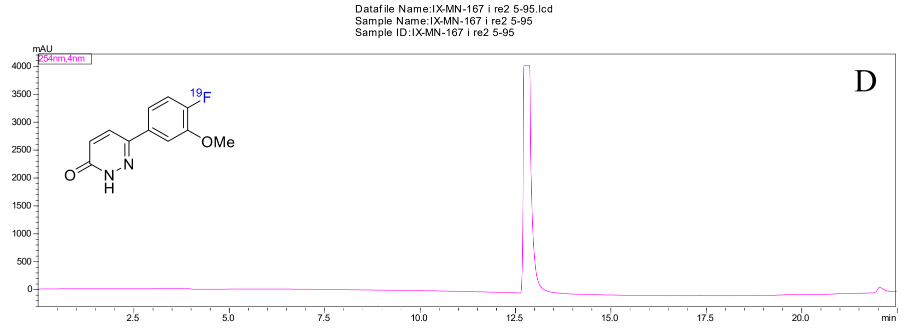

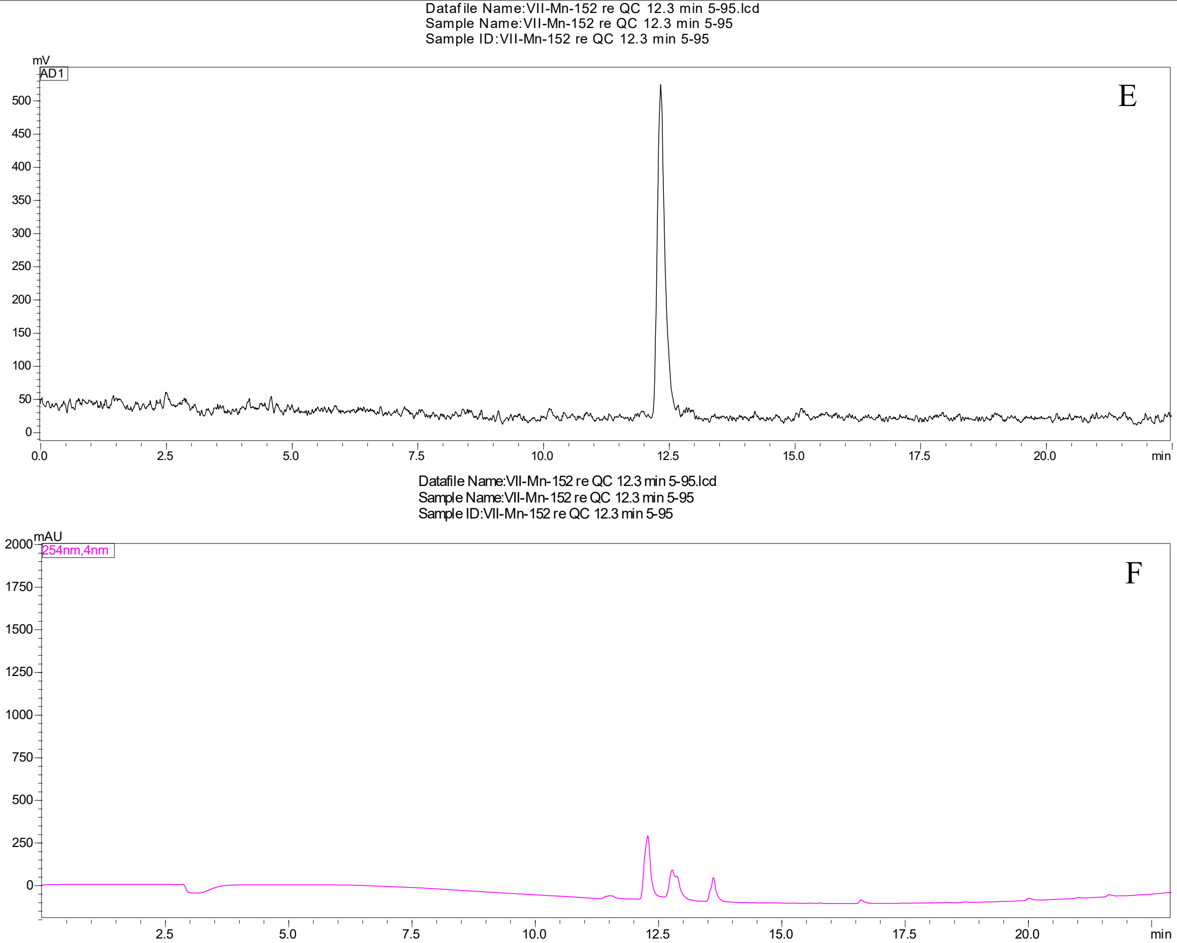

Figure S39: Radio-HPLC analysis of reaction mixture from **S31**. Reaction mixture with HPLC method 1 (A), authentic **[^{19}F]31** with HPLC method 1 (B), reaction mixture with HPLC method 1 (C), authentic **[^{19}F]31** with HPLC method 1 (D), and QC for **[^{18}F]31** (E and F).

HPLC Conditions for QC: Column: Phenomenex, Kinetex® 5μm F5 100 Å, 250 × 4.6 mm LC Column
Solvent A: 0.1% TFA water, Solvent B: 0.1% TFA acetonitrile; Isocratic / Gradient elution: 5% Solvent B for 0 to 2 min, 5% – 95% Solvent B for 2 to 22.5 min. Flow rate: 1 mL/min.

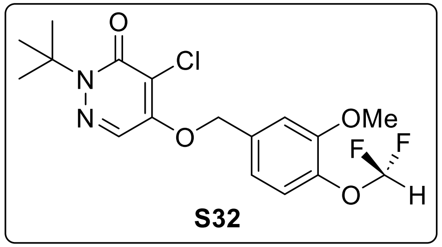

Arene substrate:

2-(*tert*-Butyl)-4-chloro-5-((4-(difluoromethoxy)-3-methoxybenzyl)oxy)pyridazin-3(2*H*)-one (S32)

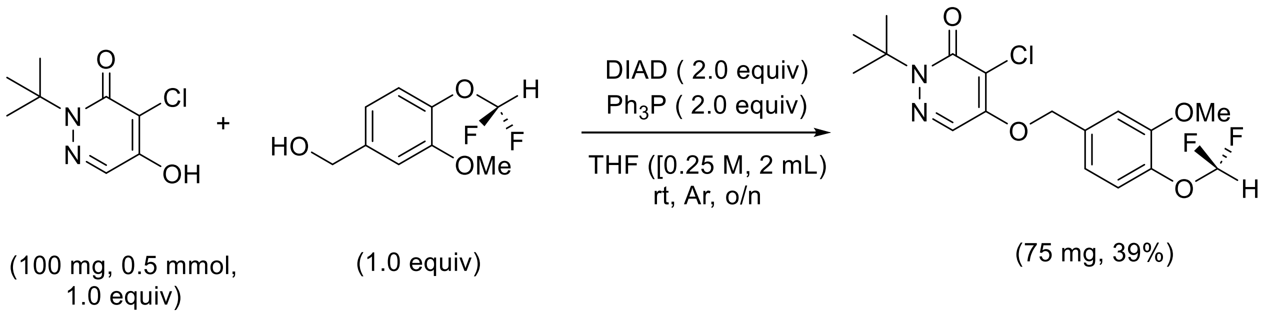

A 4 mL scintillation vial was charged with 2-(*tert*-butyl)-4-chloro-5-hydroxypyridazin-3(2*H*)-one (100 mg, 0.5 mmol, 1.0 equiv), (4-(difluoromethoxy)-3-methoxyphenyl)methanol **S27–I2** (102 mg, 0.5 mmol, 1.0 equiv), Ph_3P (288 mg, 1.1 mmol, 2.2 equiv). The screw-capped vial was purged and degassed with argon for three times. Anhydrous THF (2 mL, [0.5 M]) followed by DIAD (0.19 mL, 1.0 mmol, 2.0 equiv) were transferred by syringe and the screw-capped tube was stirred at room temperature overnight. Next morning, the reaction mixture diluted with EtOAc and H_2O. The aqueous layer was extracted with EtOAc (3 × 5 mL). The combined organics dried (Na_2SO_4) and concentrated under reduced pressure, which was subsequently purified by column chromatography.

Purification: Gradient column chromatography [SiO_2, EtOAc:Hexanes 10:90 to 30:70)] to obtain the titled compound **S32** as white powder (75 mg, 39%).

R_f : 0.6 (EtOAc : Hexanes 3:7)

^{1}H NMR ($CDCl_3$, 400 MHz): δ 7.72 (s, 1H), 7.19 (d, J = 8.1 Hz, 1H), 7.06 (d, J = 2.3 Hz, 1H), 6.95 (dd, J = 8.1, 1.8 Hz, 1H), 6.56 (t, J = 74.9 Hz, 1H), 5.27 (s, 2H), 3.89 (s, 3H), 1.64 (s, 9H).

^{13}C NMR ($CDCl_3$, 101 MHz): δ 159.09, 153.65, 151.75, 140.25 (t, J= 3.2 Hz), 133.67, 125.09, 122.75, 119.58, 116.09 (t, J= 259.7 Hz), 111.36, 71.46, 66.67, 56.23, 27.98.

^{19}F NMR ($CDCl_3$, 376 MHz): δ –81.64 (d, J = 75.0 Hz).

HRMS (ESI-TOF) m/z: $[M + H]^+$ Calcd. for $C_{17}H_{20}{}^{35}ClF_2N_2O_4$ 389.1080; found 389.1076 and $[M + Na]^+$ Calcd. for $C_{17}H_{19}{}^{35}ClF_2N_2NaO_4$ 411.0899; found 411.0897.

***<u>Authentic fluoroarene standard</u>* ([^{19}F]32)**

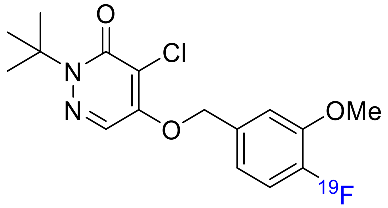

^{19}F-Reference compound – **[^{19}F]32**: data are comparable to that reported in the literature.[9]

Radio-HPLC analysis and characterization

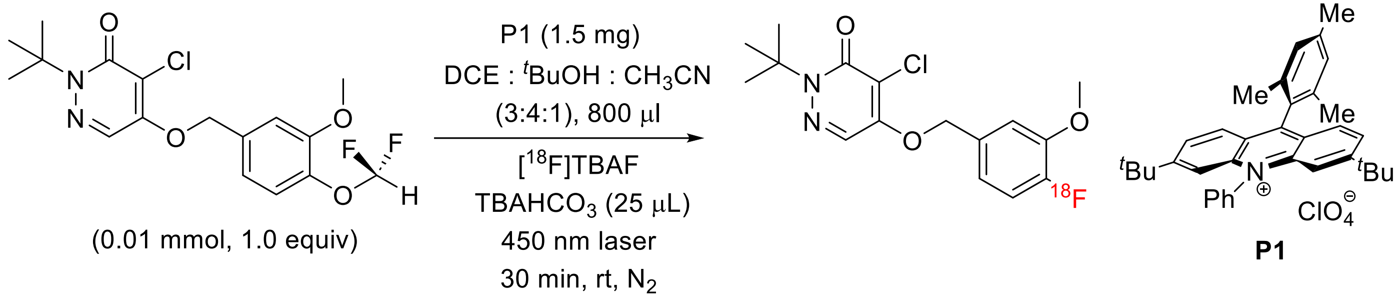

Entry	Activity at starting	Activity at EOS	Alumina Filtration	Injected Dose	Collected Dose	Isolation Time	Decay Corrected	Filtration Yield	HPLC Purity of Filtrate	%Yield
1*	9.08 mCi	7.01 mCi	1.26 mCi	1097 μCi	234 μCi	14.6 min	998.59 μCi	18.93%	23.43%	4.43%
2**	5.09 mCi	3.02 mCi	3.31 mCi	1034 μCi	180 μCi	12.5 min	947.21 μCi	41.58%	19.00%	7.90%
3**	19.7 mCi	14.94 mCi	5.28 mCi	426 μCi	65 μCi	12.5 min	390.36 μCi	35.34%	16.65%	5.88%
								**Average %Yield: 6.1% (n=3)**		

Table S38: HPLC isolated RCYs for **[^{18}F]32**

*HPLC Conditions – method 1: Column: Phenomenex, Kinetex® 5μm F5 100 Å, 250 ✕ 4.6 mm LC Column

Solvent A: 0.1% TFA water, Solvent B: 0.1% TFA acetonitrile; Isocratic / Gradient elution: 30% Solvent B for 0 to 2 min, 30% – 95% Solvent B for 2 to 22.5 min. Flow rate: 1 mL/min

**HPLC Conditions – method 2: Column: Phenomenex, Kinetex® 5μm F5 100 Å, 250 ✕ 4.6 mm LC Column

Solvent A: 0.1% TFA water, Solvent B: 0.1% TFA acetonitrile; Isocratic / Gradient elution: 40% Solvent B for 0 to 2 min, 40% – 75% Solvent B for 2 to 22.5 min. Flow rate: 1 mL/min

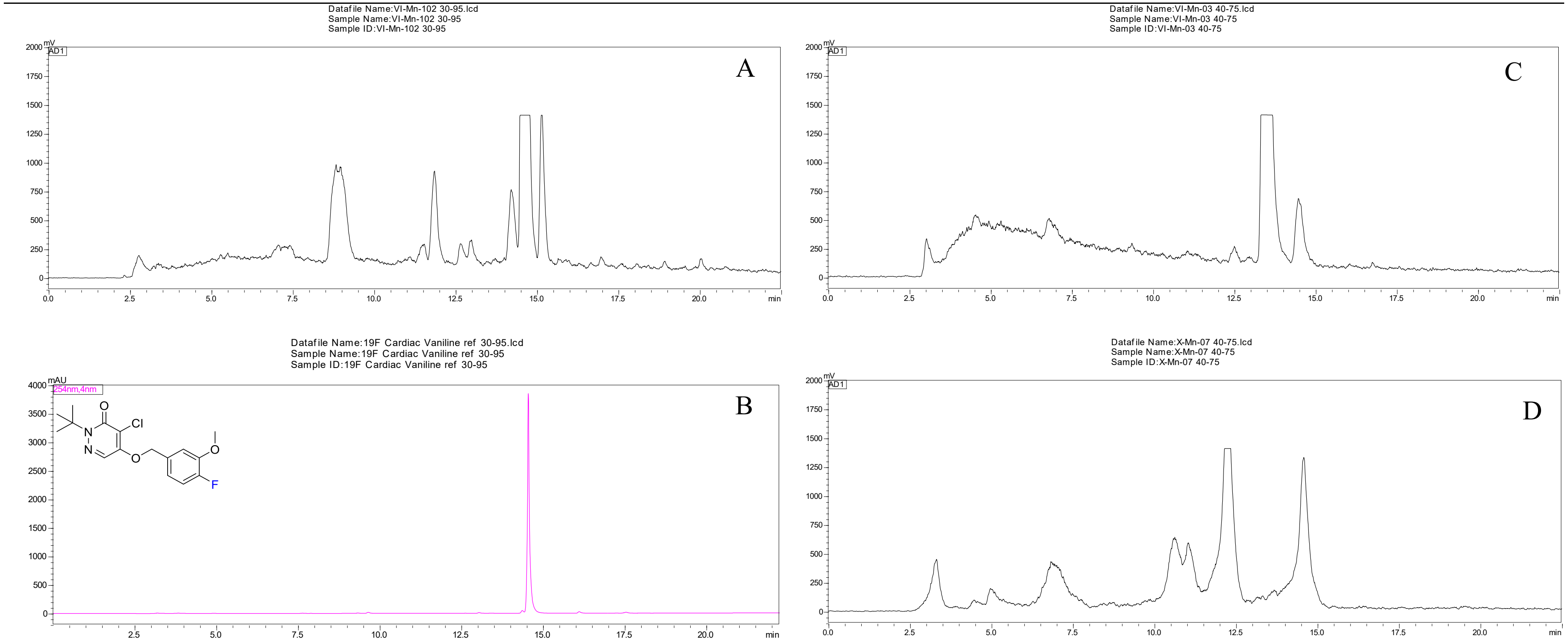

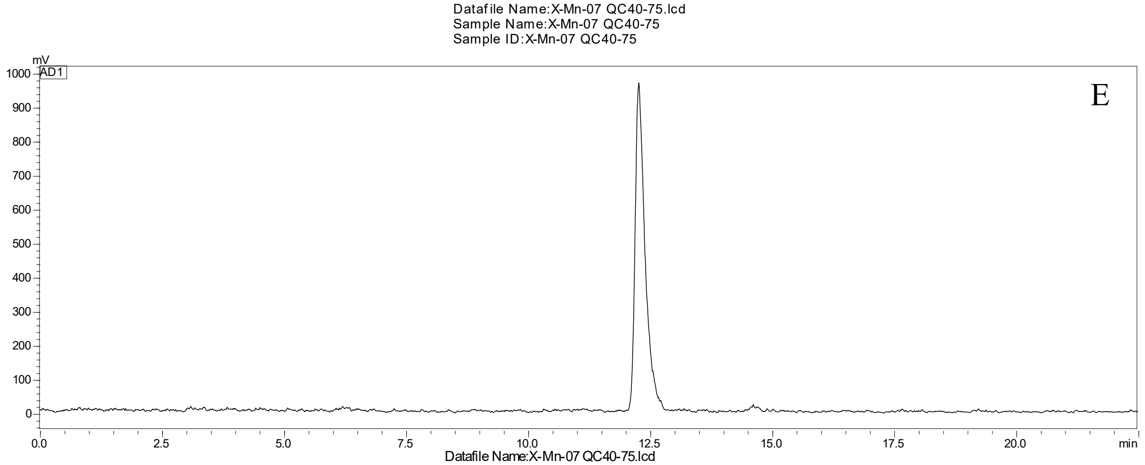

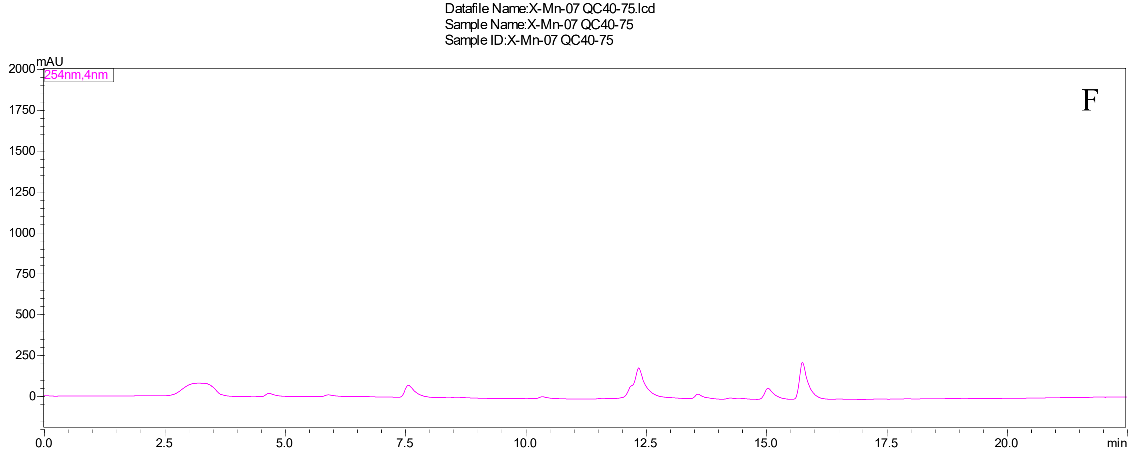

Figure S40: Radio-HPLC analysis of reaction mixture from **S32**. Reaction mixture with HPLC method 1 (A), authentic **[^{19}F]32** with HPLC method 1 (B), reaction mixture with HPLC method 2 (C), reaction mixture with HPLC method 2 (D), and QC for **[^{18}F]32** (E and F).

HPLC Conditions for QC: Column: Phenomenex, Kinetex® 5μm F5 100 Å, 250 × 4.6 mm LC Column

Solvent A: 0.1% TFA water, Solvent B: 0.1% TFA acetonitrile; Isocratic / Gradient elution: 40% Solvent B for 0 to 2 min, 40% – 75% Solvent B for 2 to 22.5 min. Flow rate: 1 mL/min.

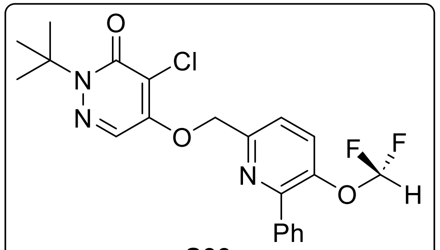

S33

Arene substrate:

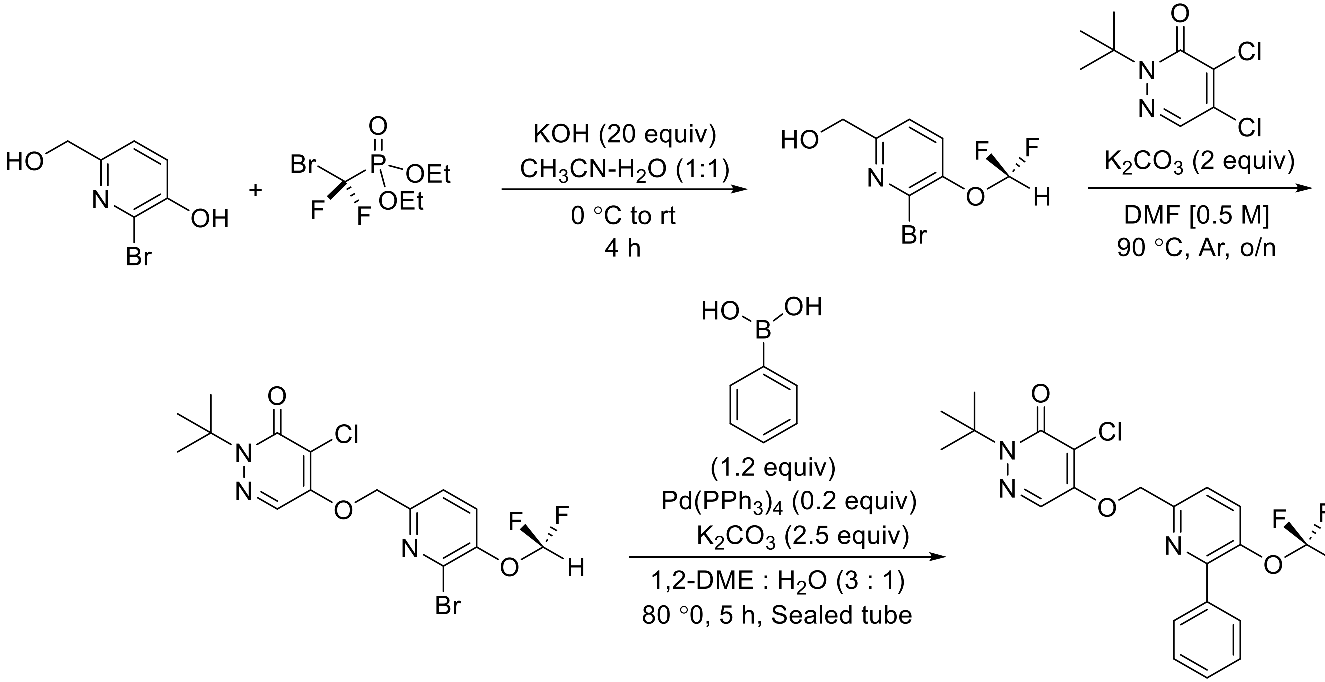

(6-Bromo-5-(difluoromethoxy)pyridin-2-yl)methanol (S33–I1)

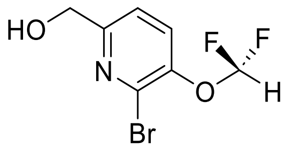

General conditions **A** were followed using 2-bromo-6-(hydroxymethyl)pyridin-3-ol (1.8 g, 8.82 mmol, 1.0 equiv), $BrCF_2P(O)(OC_2H_5)_2$ (1.068 g, 4.0 mmol, 2.0 equiv), KOH (2.24 g, 40 mmol, 20 equiv), 1,4-dioxane : CH_3CN : H_2O (7.5 mL : 7.5 mL : 7.5 mL) for 4 h.

Purification: Gradient column chromatography [SiO_2, EtOAc:Hexanes 10:90 to 30:70)] to obtain white solid of **S33–I1** (538 mg, 24%).

R_f : 0.6 (EtOAc : Hexanes 3:7)

^{1}H NMR ($CDCl_3$, 400 MHz): δ 7.55 (d, J = 8.2 Hz, 1H), 7.33 (d, J = 8.2 Hz, 1H), 6.66 (t, J = 72.4 Hz, 1H), 4.74 (s, 2H).

^{13}C NMR ($CDCl_3$, 101 MHz): δ 158.06, 143.91 (t, J = 2.6 Hz), 135.17, 130.46, 120.47, 116.81 (t, J = 266.1 Hz), 64.05.

^{19}F NMR ($CDCl_3$, 376 MHz): δ –81.96 (d, J = 72.4 Hz).

HRMS (ESI-TOF) m/z: $[M + Na]^+$ Calcd. for $C_7H_6{}^{79}BrF_2NNaO_2$ 275.9448; found 275.9445

5-((6-Bromo-5-(difluoromethoxy)pyridin-2-yl)methoxy)-2-(*tert*-butyl)-4-chloropyridazin-3(*2H*)-one (S33–I2)

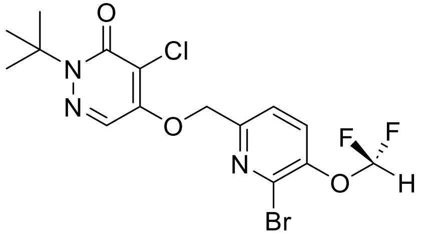

A 4 mL scintillation vial was charged with 2-(*tert*-butyl)-4,5-dichloropyridazin-3(*2H*)-one (110 mg, 0.5 mmol, 1.0 equiv), (6-bromo-5-(difluoromethoxy)pyridin-2-yl)methanol **S33–I1** (126 mg, 0.5 mmol, 1.0 equiv), and K_2CO_3 (138 g, 1.0 mmol, 2.0 equiv). The vial was purged and degassed with nitrogen for three times. Anhydrous DMF (1.0 mL) [0.5 M] was transferred by syringe and the screw-capped tube was heated at 90 °C overnight. Next morning, the reaction mixture cooled to room temperature and diluted with ice-cold H_2O. The aqueous layer was extracted with EtOAc (3 × 5 mL). The combined organics dried (Na_2SO_4) and concentrated under reduced pressure, which was subsequently purified by column chromatography.

Purification: Gradient column chromatography [SiO_2, EtOAc:Hexanes 10:90 to 40:60)] to obtain pale orange solid of **S33–I2** (68 mg, 31%).
R_f : 0.6 (EtOAc : Hexanes 3:7)
^{1}H NMR ($CDCl_3$, 400 MHz): δ 7.76 (s, 1H), 7.64 (d, J = 8.3 Hz, 1H), 7.58 (d, J = 8.3 Hz, 1H), 6.61 (t, J = 72.0 Hz, 1H), 5.36 (s, 2H), 1.65 (s, 9H).
^{13}C NMR ($CDCl_3$, 101 MHz): δ 159.00, 153.24, 152.77, 144.77 (t, J = 2.9 Hz), 135.49, 130.35, 124.67, 121.28, 118.64, 115.36 (t, J = 266.7 Hz), 71.00, 66.87, 27.98.
^{19}F NMR ($CDCl_3$, 376 MHz): δ –82.11 (d, J = 72.8 Hz).
HRMS (ESI-TOF) m/z: $[M + H]^+$ Calcd. for $C_{15}H_{16}{}^{79}BrClF_2N_3O_3$ 438.0032; found 438.0032 and Calcd. for $C_{15}H_{16}{}^{81}BrClF_2N_3O_3$ 440.0011 found 440.0010
m/z: $[M + Na]^+$ Calcd. for $C_{15}H_{15}{}^{79}BrClF_2N_3NaO_3$ 459.9851; found 459.9851 and Calcd. for $C_{15}H_{16}{}^{81}BrClF_2N_3NaO_3$ 461.9831; found 461.9828.

2-(*tert*-Butyl)-4-chloro-5-((5-(difluoromethoxy)-6-phenylpyridin-2-yl)methoxy)pyridazin-3(*2H*)-one (S33)

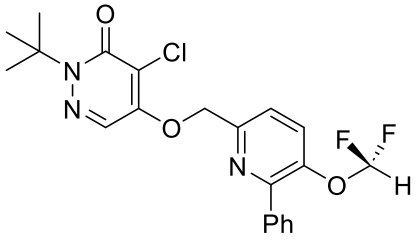

A 15 mL pressure tube was charged with 5-((6-bromo-5-(difluoromethoxy)pyridin-2-yl)methoxy)-2-(*tert*-butyl)-4-chloropyridazin-3(*2H*)-one **S33–I2** (35 mg, 0.08 mmol, 1.0 equiv), phenylboronic acid (12 mg, 0.096 mmol, 1.2 equiv), K_2CO_3 (25 mg, 0.18 mmol, 2.3 equiv), and $Pd(PPh_3)_4$ (5 mg, 0.004 mmol, 0.05 equiv). The tube was purged and degassed with nitrogen for three times. Degassed 1,2-DME : H_2O (3:1) [0.04 M] was transferred by syringe and the screw-capped tube was heated at 80 °C for 5h. The reaction mixture cooled down to room temperature and diluted with EtOAc and H_2O. The aqueous layer was extracted with EtOAc (2 ×10 mL). The combined organics dried (Na_2SO_4) and concentrated under reduced pressure, which was subsequently purified by column chromatography.

Purification: Gradient column chromatography [SiO_2, EtOAc:Hexanes 10:90 to 30:70)] to obtain beige colour solid of **S33** (19 mg, 31%).
R_f : 0.5 (EtOAc : Hexanes 3:7)
^{1}H NMR ($CDCl_3$, 400 MHz): δ 7.84 (d, J = 1.9 Hz, 1H), 7.83 (s, 1H), 7.82 (d, J = 1.2 Hz, 1H), 7.68 (d, J = 8.4 Hz, 1H), 7.55 (d, J = 8.5 Hz, 1H), 7.50–7.44 (m, 3H), 6.42 (t, J = 72.9 Hz, 1H), 5.45 (s, 2H), 1.65 (s, 9H).
^{13}C NMR ($CDCl_3$, 101 MHz): δ 159.07, 153.57, 151.89, 150.91, 144.75 (t, J= 2.7 Hz), 135.92, 129.69, 129.47, 129.40, 128.52, 125.00, 120.87, 118.36, 115.70 (t, J= 263.8 Hz), 71.91, 66.69, 27.99.
^{19}F NMR ($CDCl_3$, 376 MHz): δ –81.35 (d, J = 74.2 Hz).
HRMS (ESI-TOF) m/z: $[M + H]^+$ Calcd. for $C_{21}H_{21}{}^{35}ClF_2N_3O_3$ 436.1240; found 436.1241 and $[M + Na]^+$ Calcd. for $C_{21}H_{20}{}^{35}ClF_2N_3NaO_3$ 458.1059; found 458.1059.

Authentic fluoroarene standard

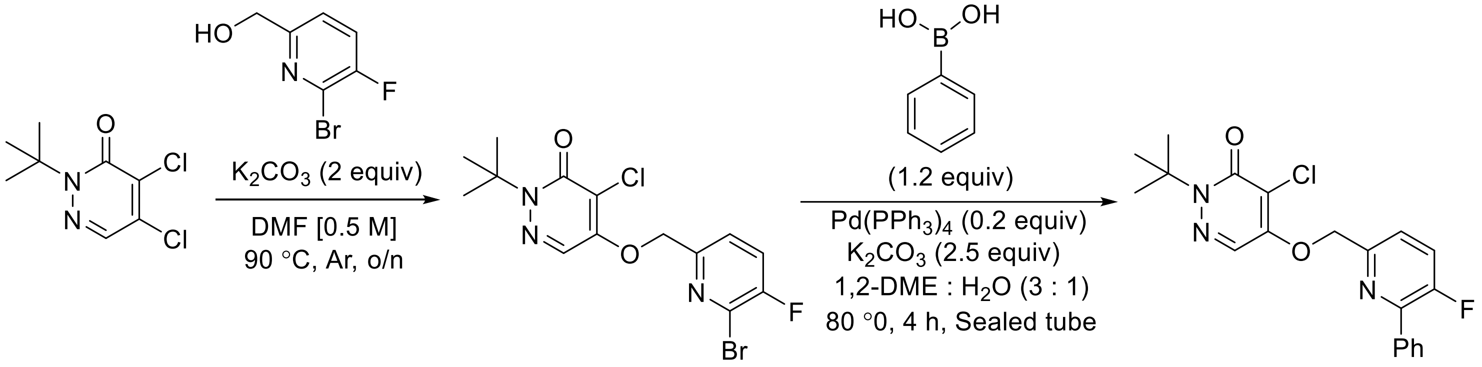

5-((6-Bromo-5-fluoropyridin-2-yl)methoxy)-2-(*tert*-butyl)-4-chloropyridazin-3(*2H*)-one ([^{19}F]33–I1)

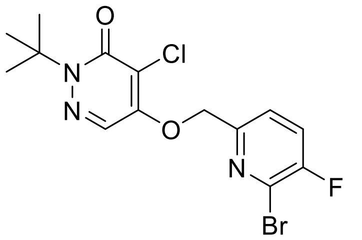

A 4 mL scintillation vial was charged with 2-(*tert*-butyl)-4,5-dichloropyridazin-3(*2H*)-one (108 mg, 0.49 mmol, 1.0 equiv), (6-bromo-5-fluoropyridin-2-yl)methanol (100 mg, 0.49 mmol, 1.0 equiv), and K_2CO_3 (135 g, 0.98 mmol, 2.0 equiv). The vial was purged and degassed with nitrogen for three times. Anhydrous DMF (0.98 mL) [0.5 M] was transferred by syringe and the screw-capped tube was heated at 90 °C overnight. Next morning, the reaction mixture cooled down to room temperature and diluted with ice-cold H_2O. The aqueous layer was extracted with EtOAc (3 × 5 mL). The combined organics dried (Na_2SO_4) and concentrated under reduced pressure, which was subsequently purified by column chromatography.

Purification: Gradient column chromatography [SiO_2, EtOAc:Hexanes 10:90 to 30:70)] to obtain pale yellow solid of **[^{19}F]33–I1** (70 mg, 37%).

R$_f$: 0.6 (EtOAc : Hexanes 3:7)

^{1}H NMR ($CDCl_3$, 400 MHz): δ 7.76 (s, 1H), 7.58 (dd, J = 8.4, 3.6 Hz, 1H), 7.54–7.49 (m, 1H), 5.36 (s, 2H), 1.65 (s, 9H).

^{13}C NMR ($CDCl_3$, 101 MHz): δ 158.98, 157.28, 154.69, 153.23, 151.53 (d, J = 5.1 Hz), 129.64 (d, J = 24.9 Hz), 124.69, 121.98 (d, J = 3.6 Hz), 118.64, 71.04, 68.65, 27.98.

^{19}F NMR ($CDCl_3$, 376 MHz): δ –112.39 (ddd, J = 11.1, 7.6, 4.1 Hz).

HRMS (ESI-TOF) m/z: $[M + H]^+$ Calcd. for $C_{14}H_{15}{}^{79}Br^{35}ClFN_3O_2$ 390.0020; found 390.0019 and
$[M + Na]^+$ Calcd. for $C_{14}H_{14}{}^{79}Br^{35}ClFN_3NaO_2$ 411.9840; found 411.9839.

2-(*tert*-Butyl)-4-chloro-5-((5-fluoro-6-phenylpyridin-2-yl)methoxy)pyridazin-3(*2H*)-one ([^{19}F]33)

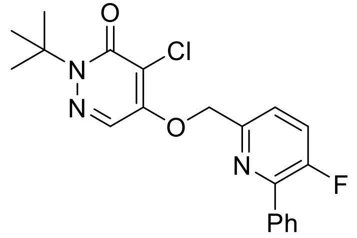

A 15 mL pressure tube was charged with 5-((6-bromo-5-fluoropyridin-2-yl)methoxy)-2-(*tert*-butyl)-4-chloropyridazin-3(2*H*)-one **[^{19}F]33–I1** (39 mg, 0.1 mmol, 1.0 equiv), phenylboronic acid (15 mg, 0.12 mmol, 1.2 equiv), K_2CO_3 (32 mg, 0.23 mmol, 2.3 equiv), and $Pd(PPh_3)_4$ (5 mg, 0.005 mmol, 0.05 equiv). The tube was purged and degassed with nitrogen for three times. Degassed 1,2-DME : H_2O (3:1) [0.5 M] was transferred by syringe and the screw-capped tube was heated at 80 °C for 4 h. The reaction mixture cooled down to room temperature and diluted with EtOAc and H_2O. The aqueous layer was extracted with EtOAc (2 ×10 mL). The combined organics dried (Na_2SO_4) and concentrated under reduced pressure, which was subsequently purified by column chromatography.

Purification: Gradient column chromatography [SiO_2, EtOAc:Hexanes 10:90 to 20:80)] to obtain beige colour solid of **[^{19}F]33** (36 mg, 31%).

R_f : 0.6 (EtOAc : Hexanes 2:8)

^{1}H NMR ($CDCl_3$, 400 MHz): δ 7.98–7.95 (m, 2H), 7.84 (s, 1H), 7.59–7.54 (m, 1H), 7.52–7.43 (m, 4H), 5.45 (s, 2H), 1.64 (s, 9H).

^{13}C NMR ($CDCl_3$, 101 MHz): δ 159.07, 158.47, 155.87, 153.59, 150.56 (d, *J* = 4.7 Hz), 145.86 (d, *J* = 11.6 Hz), 134.72 (t, *J* = 2.9 Hz), 129.73, 128.91 (d, *J* = 5.8 Hz), 128.69, 125.56 (d, *J* = 21.3 Hz), 125.01, 121.54 (d, *J* = 4.5 Hz), 118.32, 71.92, 66.65, 27.98.

^{19}F NMR ($CDCl_3$, 376 MHz): δ –123.72 (ddd, *J* = 13.1, 3.8, 1.4 Hz).

HRMS (ESI-TOF) m/z: $[M + H]^+$ Calcd. for $C_{20}H_{20}{}^{35}ClFN_3O_2$ 388.1228; found 388.1236 and
$[M + Na]^+$ Calcd. for $C_{20}H_{19}{}^{35}ClFN_3NaO_2$ 410.1048; found 410.1050.

Radio-HPLC analysis and characterization

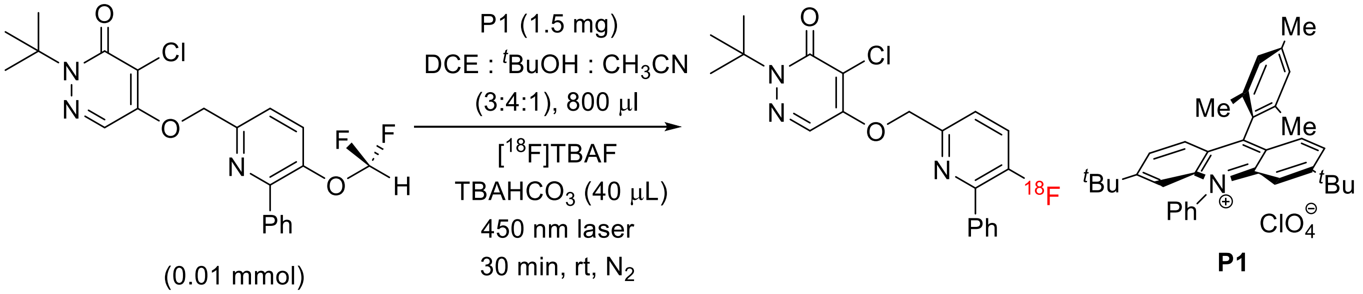

Entry	Activity at starting	Activity at EOS	Alumina Filtration	Injected Dose	Collected Dose	Isolation Time	Decay Corrected	Filtration Yield	HPLC Purity of Filtrate	%Yield
1*	18.23 mCi	14.73 mCi	4.33 mCi	816 μCi	201 μCi	17.7 min	732.40 μCi	29.39%	27.44%	8.06%
2**	16.90 mCi	12.64 mCi	3.84 mCi	1014 μCi	228 μCi	16.0 min	910.78 μCi	30.37%	25.03%	7.60%
3**	47.2 mci	36.5 mCi	9.79 mCi	2207 μCi	324 μCi	16.0 min	1994.91	26.82%	16.24%	4.35%
								**Average %Yield: 6.7% (n=2)**		

Supplementary **Table S39**: HPLC isolated RCYs for **[^{18}F]33**

*HPLC Conditions – method 1: Column: Phenomenex, Kinetex® 5μm F5 100 Å, 250 ✕ 4.6 mm LC Column

Solvent A: 0.1% TFA water, Solvent B: 0.1% TFA acetonitrile; Isocratic / Gradient elution: 20% Solvent B for 0 to 2 min, 20% – 95% Solvent B for 2 to 22.5 min. Flow rate: 1 mL/min

**HPLC Conditions – method 2: Column: Phenomenex, Kinetex® 5μm F5 100 Å, 250 ✕ 4.6 mm LC Column

Solvent A: 0.1% TFA water, Solvent B: 0.1% TFA acetonitrile; Isocratic / Gradient elution: 42% Solvent B for 0 to 2 min, 42% – 60% Solvent B for 2 to 22.5 min. Flow rate: 1 mL/min

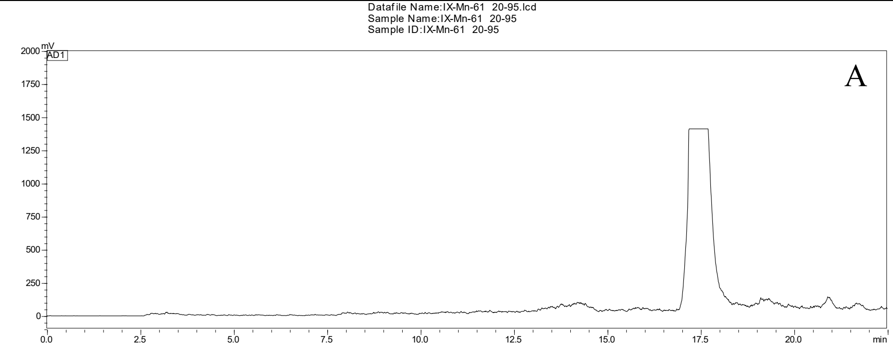

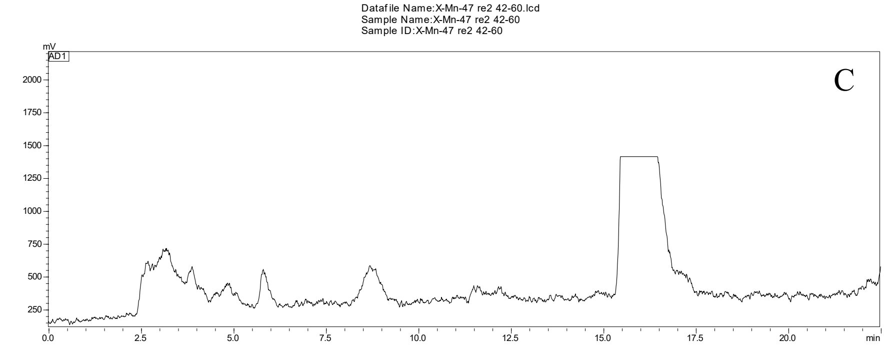

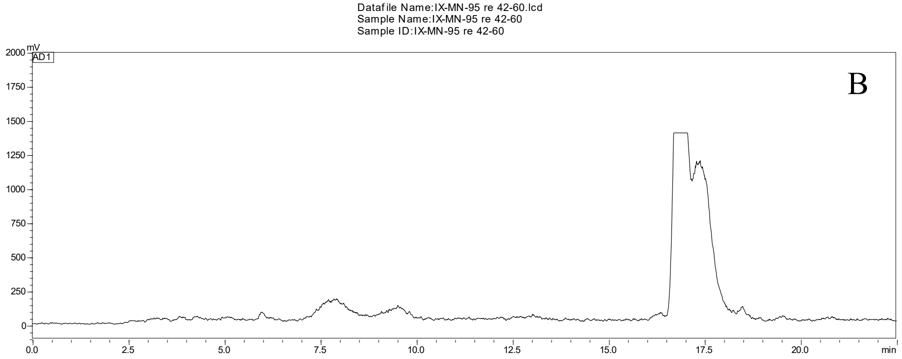

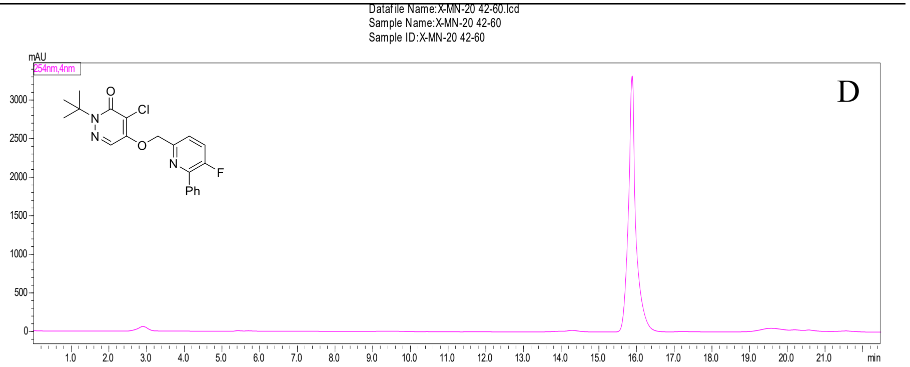

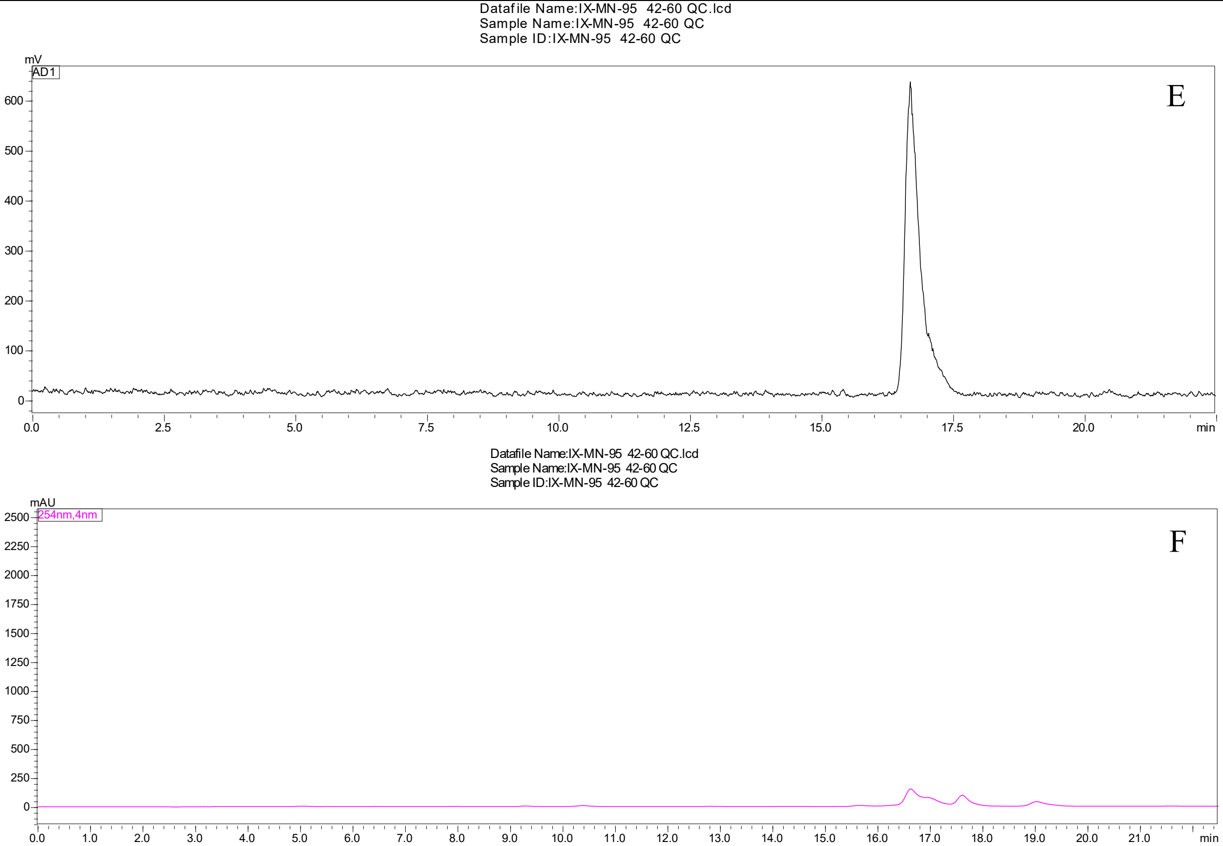

Figure S41: Radio-HPLC analysis of reaction mixture from **S33**. Reaction mixture with HPLC method 1 (A), reaction mixture with HPLC method 2 (B), reaction mixture with HPLC method 2 (C), authentic **[^{19}F]33** with HPLC method 2 (D) and QC for **[^{18}F]33** (E and F).

HPLC Conditions for QC: Column: Phenomenex, Kinetex® 5µm F5 100 Å, 250 × 4.6 mm LC Column

Solvent A: 0.1% TFA water, Solvent B: 0.1% TFA acetonitrile; Isocratic / Gradient elution: 42% Solvent B for 0 to 2 min, 42% – 60% Solvent B for 2 to 22.5 min. Flow rate: 1 mL/min.

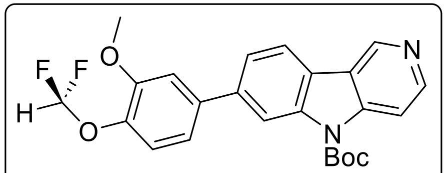

Arene substrate:

2-(4-(Difluoromethoxy)-3-methoxyphenyl)-4,4,5,5-tetramethyl-1,3,2-dioxaborolane (S34–I1)

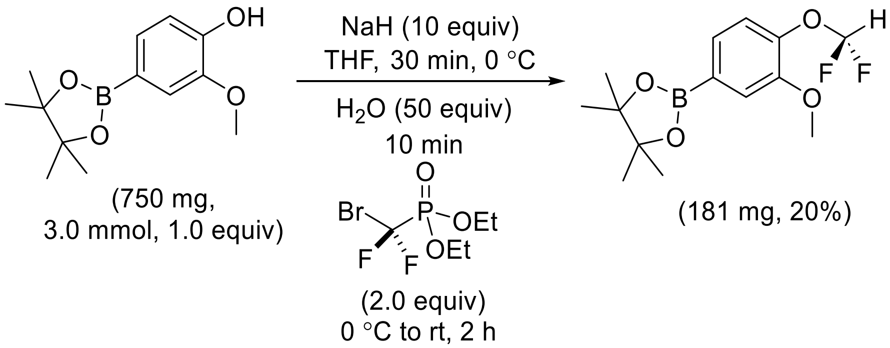

General conditions **B** were followed using 2-methoxy-4-(4,4,5,5-tetramethyl-1,3,2-dioxaborolan-2-yl)phenol (750 mg, 3.0 mmol, 1.0 equiv), NaH (60%) (800 mg, 30 equiv, 10 mmol), H_2O (2.7 mL, 150 mmol, 50 equiv), and $BrCF_2P(O)(OC_2H_5)_2$ (1.6 g, 6.0 mmol, 2.0 equiv), and THF (12 mL, 0.25M).

Purification: Gradient column chromatography [SiO_2, EtOAc:Hexanes 00:100 to 20:80)] to obtain white solid of **S34–I1** (181 mg, 20%).

R_f: 0.5 (EtOAc : Hexanes 3:7)

^{1}H NMR ($CDCl_3$, 400 MHz): δ 7.40 (dd, J = 9.0, 1.3 Hz, 2H), 7.14 (d, J = 7.8 Hz, 1H), 6.58 (t, J = 74.9 Hz, 1H), 3.92 (s, 3H), 1.35 (s, 12H).

^{13}C NMR ($CDCl_3$, 101 MHz): δ 150.43, 142.71 (t, J = 2.9 Hz), 128.14, 121.34, 118.45, 116.21 (t, J = 260.1 Hz), 84.19, 56.15, 24.98.

^{19}F NMR ($CDCl_3$, 376 MHz): δ –81.44 (d, J = 74.3 Hz).

HRMS (ESI-TOF) m/z: $[M + Na]^+$ Calcd. for $C_{14}H_{19}BF_2NaO_4$ 323.1242; found 323.1247.

***tert*-Butyl-7-(4-(difluoromethoxy)-3-methoxyphenyl)-5*H*-pyrido[4,3-b]indole-5-carboxylate (S34)**

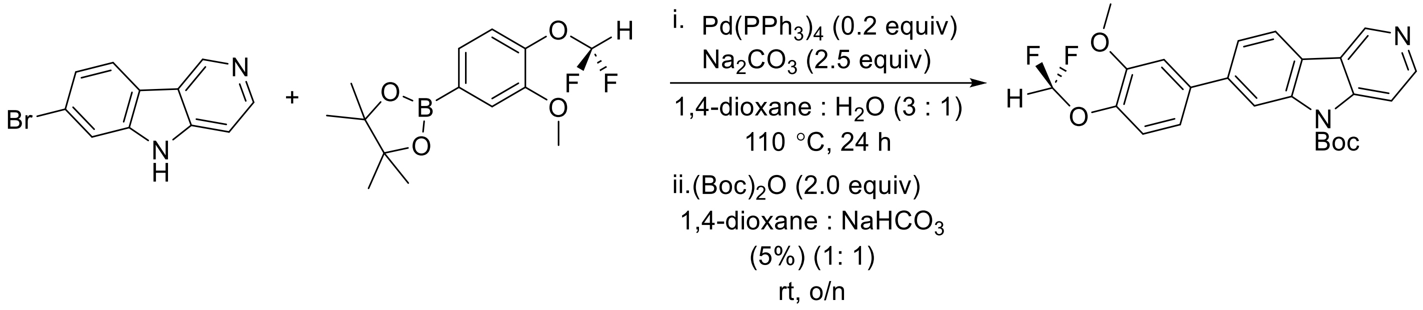

A 15 mL pressure tube was charged with 7-bromo-5H-pyrido[4,3-b]indole (82 mg, 0.33 mmol, 1.0 equiv), 2-(4-(difluoromethoxy)-3-methoxyphenyl)-4,4,5,5-tetramethyl-1,3,2-dioxaborolane **S31–I1** (100 mg, 0.33 mmol, 1.0 equiv), Na_2CO_3 (87 mg, 0.83 mmol, 2.5 equiv), and $Pd(PPh_3)_4$ (76 mg, 0.066 mmol, 0.2 equiv). The tube was purged and degassed with nitrogen for three times. Degassed 1,4-dioxane : H_2O (3:1) [6 mL, 0.05 M] was transferred by syringe and the screw-capped tube was heated at 110 °C for overnight. The reaction mixture cooled down to room temperature and diluted with EtOAc and H_2O. The aqueous layer was extracted with EtOAc (2 × 10 mL). The combined organics dried (Na_2SO_4) and concentrated under reduced pressure, which was subsequently dissolved in 1,4-dioxane: 5% $NaHCO_3$ in H_2O (3 mL : 3mL). $(Boc)_2O$ (76 µL, 0.66 mmol, 2.0 equiv) was added and a reaction mixture was stirred overnight at room temperature. The reaction mixture was diluted with EtOAc and H_2O and the aqueous layer was extracted with EtOAc (2 × 5 mL). The combined organics dried (Na_2SO_4) and concentrated under reduced pressure, which was subsequently purified by column chromatography.

Purification: Gradient column chromatography [SiO_2, EtOAc:Hexanes 20:80 to 40:60)] to obtain pale yellow solid of **S34** (68 mg, 47%).

R_f : 0.3 (EtOAc : Hexanes 3:7)

^{1}H NMR (MeOD-d^4, 400 MHz): δ 9.22 (s, 1H), 8.54 (d, J = 5.9 Hz, 1H), 8.51 (s, 1H), 8.22 (d, J = 5.6 Hz, 1H), 8.18 (d, J = 8.1 Hz, 1H), 7.70–7.63 (m, 2H), 7.38 (s, 1H), 7.28–7.24 (m, 1H), 6.89 (t, J = 75.3 Hz, 1H), 3.99 (s, 3H), 1.8 (s, 9H).

^{13}C NMR (MeOD-d^4, 126 MHz): δ 152.84, 151.61, 147.21, 145.47, 142.94, 142.16, 141.34 (t, J= 2.8 Hz), 141.24, 140.28, 133.79 (d, J= 3.9 Hz), 133.08 (d, J= 10.1 Hz), 129.98 (d, J= 12.1 Hz), 124.47, 123.56 (d, J= 64.2 Hz), 121.77, 120.87, 118.10 (t, J= 257.9 Hz), 115.76, 112.73 (d, J= 63.1 Hz), 86.93, 56.66, 28.48.

^{19}F NMR (MeOD-d^4, 376 MHz): δ –83.28 (d, J = 75.7 Hz).

HRMS (ESI-TOF) m/z: $[M + H]^+$ Calcd. for $C_{24}H_{23}F_2N_2O_4$ 441.1626; found 441.1624.

<u>Authentic fluoroarene standard</u>

***tert*-Butyl 7-(4-fluoro-3-methoxyphenyl)-5*H*-pyrido[4,3-b]indole-5-carboxylate ([^{19}F]34)**

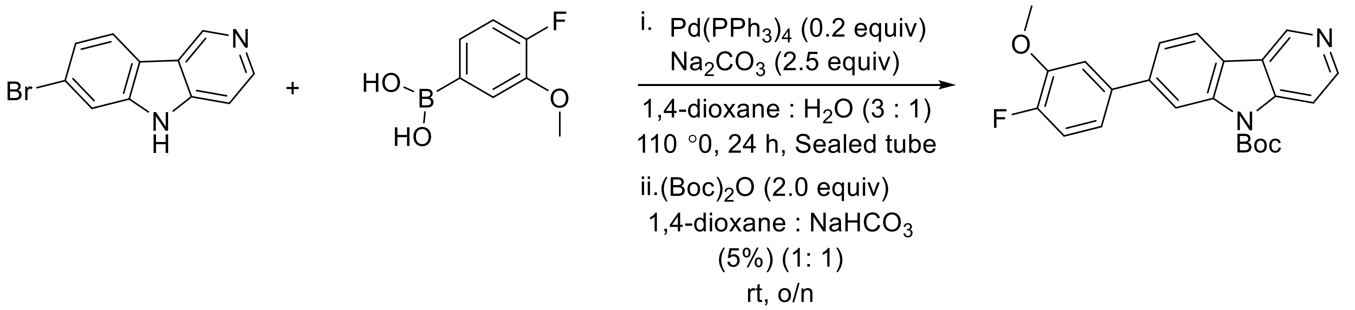

A 15 mL pressure tube was charged with 7-bromo-5H-pyrido[4,3-b]indole (124 mg, 0.5 mmol, 1.0 equiv), (4-fluoro-3-methoxyphenyl)boronic acid (93 mg, 0.55 mmol, 1.0 equiv), Na_2CO_3 (132 mg, 1.25 mmol, 2.5 equiv), and $Pd(PPh_3)_4$ (116 mg, 0.1 mmol, 0.2 equiv). The tube was purged and degassed with nitrogen for three times. Degassed 1,4-dioxane : H_2O (3:1) [6 mL, 0.05 M] was transferred by syringe and the screw-capped tube was heated at 110 °C for overnight. The reaction mixture cooled down to room temperature and diluted with EtOAc and H_2O. The aqueous layer was extracted with EtOAc (2 × 10 mL). The combined organics dried (Na_2SO_4) and concentrated under reduced pressure, which

was subsequently dissolved in 1,4-dioxane: 5% $NaHCO_3$ in H_2O (3 mL : 3 mL). $(Boc)_2O$ (230 μL, 1.0 mmol, 2.0 equiv) was added and a reaction mixture was stirred overnight at room temperature. The reaction mixture was diluted with EtOAc and H_2O and the aqueous layer was extracted with EtOAc (2 ×5 mL). The combined organics dried (Na_2SO_4) and concentrated under reduced pressure, which was subsequently purified by column chromatography.

Purification: Gradient column chromatography [SiO_2, EtOAc:Hexanes 20:80 to 40:60 to 80:20)] to obtain pale yellow semi-solid of **[^{19}F]34** (139 mg, 71%).

R_f : 0.4 (EtOAc : Hexanes 3:7)

^{1}H NMR ($CDCl_3$, 400 MHz): *δ* 9.29 (s, 1H), 8.64 (d, *J* = 5.8 Hz, 1H), 8.54 (s, 1H), 8.19 (d, *J* = 5.8 Hz, 1H), 8.10 (d, *J* = 8.6 Hz, 1H), 8.08 (dd, *J* = 7.6, 1.5 Hz, 1H), 7.26 (dd, *J* = 7.7, 1.6 Hz, 1H), 7.21–7.17 (m, 2H), 3.98 (s, 3H), 1.79 (s, 9H).

^{13}C NMR ($CDCl_3$, 101 MHz): *δ* 153.76, 151.30, 150.43, 147.99 (d, *J* = 10.9 Hz), 146.25, 144.25, 141.75, 141.13, 139.22, 138.06 (d, *J* = 3.8 Hz), 123.47, 122.32 (d, *J* = 67.6 Hz), 120.50, 120.12 (d, *J* = 7.3 Hz), 116.54 (d, *J* = 18.4 Hz), 115.18, 112.96 (d, *J* = 2.4 Hz), 111.39, 85.61, 56.53, 28.43.

^{19}F NMR ($CDCl_3$, 376 MHz): *δ* –136.87 (ddd, *J* = 10.5, 8.6, 5.5 Hz).

HRMS (ESI-TOF) m/z: $[M + H]^+$ Calcd. for $C_{23}H_{22}FN_2O_3$ 393.1614; found 393.1612.

Radio-HPLC analysis and characterization

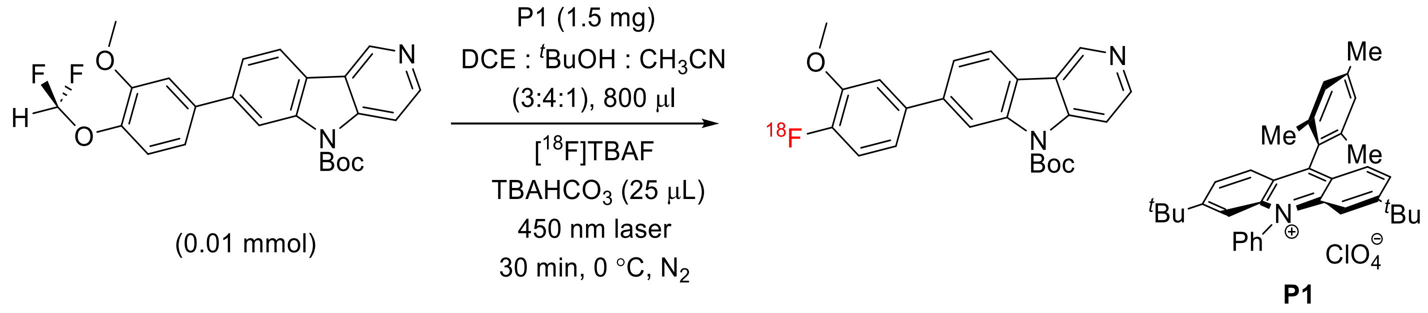

Entry	Activity at starting	Activity at EOS	Alumina Filtration	Injected Dose	Collected Dose	Isolation Time	Decay Corrected	Filtration Yield	HPLC Purity of Filtrate	%Yield
1	18.42 mCi	12.55 mCi	5.74 mCi	714 μCi	120 μCi	15.0 min	649.47 μCi	45.73%	18.47%	8.44%
2	15.74 mCi	13.93 mCi	4.82 mCi	332 μCi	87 μCi	15.0 min	301.36 μCi	34.60%	28.86%	9.98%
								**Average %Yield: 9.2% (n=2)**		

Table S40: HPLC isolated RCYs for **[^{18}F]34**

HPLC Conditions method – 1: Column: Phenomenex, Kinetex® 5μm F5 100 Å, 250 × 4.6 mm LC Column

Solvent A: 0.1% TFA water, Solvent B: 0.1% TFA acetonitrile; Isocratic / Gradient elution: 20% Solvent B for 0 to 2 min, 20% – 95% Solvent B for 2 to 22.5 min. Flow rate: 1 mL/min

HPLC Conditions method – 2: Column: Phenomenex, Luna® 5μm F5 100 Å, 250 × 4.6 mm LC Column

Solvent A: 0.1% TFA water, Solvent B: 0.1% TFA acetonitrile; Isocratic / Gradient elution: 30% Solvent B for 0 to 2 min, 30% – 95% Solvent B for 2 to 22.5 min. Flow rate: 1 mL/min

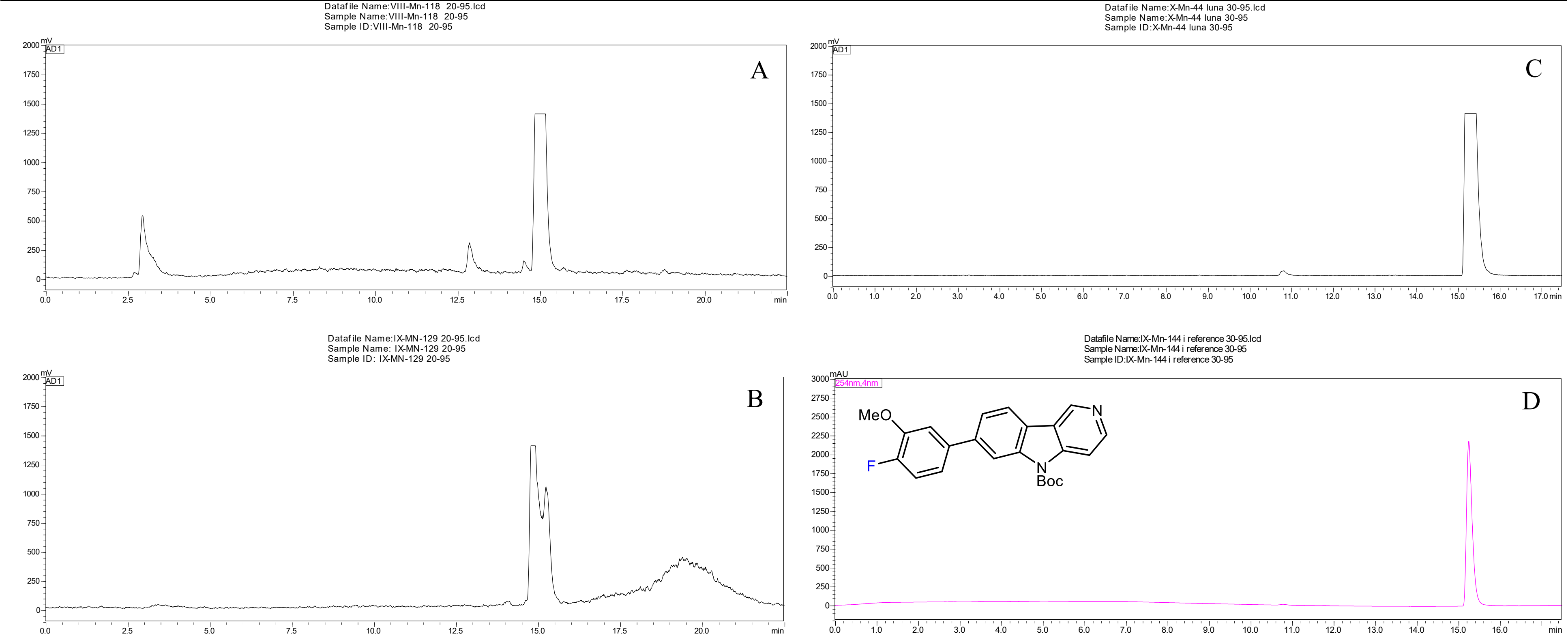

Figure S42: Radio-HPLC analysis of reaction mixture from **S34**. Reaction mixture with HPLC method 1 (A), reaction mixture with HPLC method 1 (B), reaction mixture with HPLC method 2 (C), and authentic **[^{19}F]34** with HPLC method 2 (D).

.

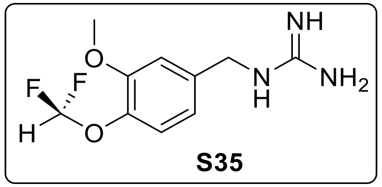

Arene substrate:

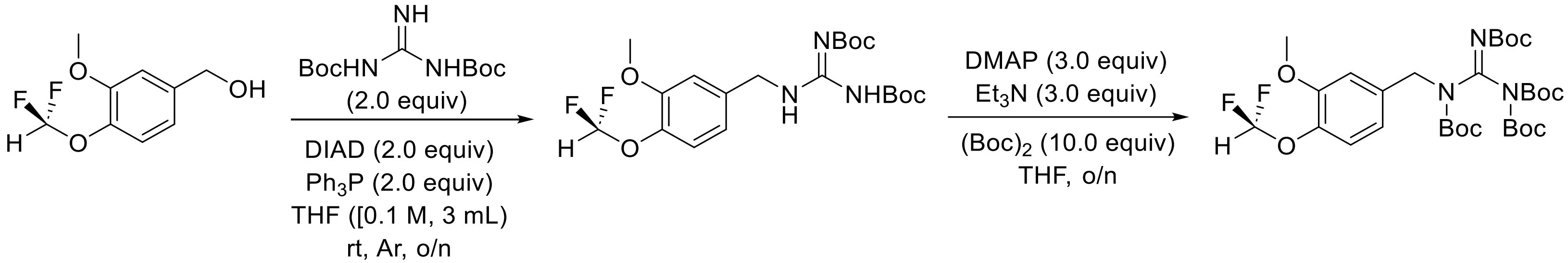

1-(Difluoromethoxy)-2-methoxy-*N*,*N′*-di-Boc-benzyl guanidine (S35-I1)

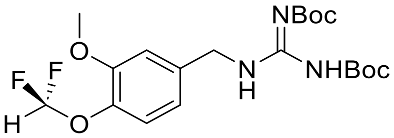

A 20 mL scintillation vial was charged with (4-(difluoromethoxy)-3-methoxyphenyl)methanol **S27–I2** (70 mg, 0.34 mmol, 1.0 equiv), *N*,*N′*-di-Boc-guanidine (176 mg, 0.68 mmol, 1.0 equiv), Ph_3P (196 mg, 0.75 mmol, 2.2 equiv). The screw-capped vial was purged and degassed with argon for three times. Anhydrous THF (3 mL, [0.1 M]) followed by DIAD (0.13 mL, 0.68 mmol, 2.0 equiv) were transferred by syringe and the screw-capped tube was stirred at room temperature overnight. Next morning, the reaction mixture diluted with EtOAc and H_2O. The aqueous layer was extracted with EtOAc (3 × 5 mL). The combined organics dried (Na_2SO_4) and concentrated under reduced pressure, which was subsequently purified by column chromatography.

Purification: Gradient column chromatography [SiO_2, EtOAc:Hexanes 5:95 to 15:85)] to obtain the titled compound **S35-I1** as white solid (65 mg, 43%).

R*f* : 0.7 (EtOAc : Hexanes 1:9)

^{1}H NMR ($CDCl_3$, 400 MHz): δ 9.63–9.10 (m, 2H), 7.15 (d, *J* = 1.8 Hz, 1H), 7.07 (d, *J* = 8.2 Hz, 1H), 6.86 (dd, *J* = 1.9 Hz, 1H), 6.53 (t, *J* = 75.5 Hz, 1H), 5.11 (s, 2H), 3.86 (s, 3H), 1.48 (s, 9H), 1.42 (s, 9H).

^{13}C NMR ($CDCl_3$, 101 MHz): δ 163.76, 160.83, 154.96, 150.87, 138.99 (t, *J* = 2.9 Hz), 137.61, 121.96, 120.18, 116.34 (t, *J* = 259.7 Hz), 112.89, 84.50, 79.02, 56.01, 47.32, 28.44, 28.08.

^{19}F NMR ($CDCl_3$, 376 MHz): δ –81.46 (d, *J* = 75.6 Hz).

HRMS (ESI-TOF) m/z: $[M + H]^+$ Calcd. for $C_{20}H_{30}F_2N_3O_6$ 446.2103; found 446.2100 and $[M + Na]^+$ Calcd. for $C_{20}H_{29}F_2N_3NaO_6$ 468.1922; found 468.1921.

1-(Difluoromethoxy)-2-methoxy-*N*,*N'*,*N'''*,*N''''*-tetra-Boc-benzyl guanidine (S35)

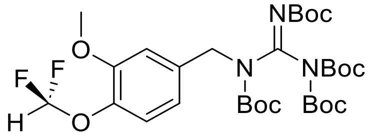

A 4 mL scintillation vial was charged with 1-(difluoromethoxy)-2-methoxy-*N*,*N'*-di-Boc-benzyl guanidine **S35-I1** (65 mg, 0.315 mmol, 1.0 equiv), DMAP (4 mg, 0.03 mmol, 0.2 equiv). Anhydrous THF (1 mL, [0.15 M]) followed by Et_3N (62.46 µL, 0.45 mmol, 3.0 equiv) and $(Boc)_2O$ (345 µL, 1.5 mmol, 10 equiv) were transferred by syringe and the reaction mixture was stirred at room temperature overnight. Next morning, the reaction mixture diluted with EtOAc and H_2O. The aqueous layer was extracted with EtOAc (3 × 5 mL). The combined organics dried (Na_2SO_4) and concentrated under reduced pressure, which was subsequently purified by column chromatography.

Purification: Gradient column chromatography [SiO_2, EtOAc:Hexanes 5:95 to 30:70)] to obtain the titled compound **S35** as white solid (68 mg, 70%).

R_f : 0.6 (EtOAc : Hexanes 1:9)

^{1}H NMR ($CDCl_3$, 400 MHz): δ 7.22 (d, J = 1.8 Hz, 1H), 7.05 (d, J = 8.2 Hz, 1H), 6.98 (d, J = 8.2, 1.9 Hz, 1H), 6.59 (t, J = 75.5 Hz, 1H), 4.99 (s, 2H), 3.86 (s, 3H), 1.47 (s, 9H), 1.42–1.41 (m, 27H).

^{13}C NMR ($CDCl_3$, 101 MHz): δ 157.45, 151.34, 150.91, 147.58, 144.44, 139.16 (t, J = 2.9 Hz), 136.21, 122.04, 120.69, 116.29 (t, J = 259.7 Hz), 113.42, 84.15, 83.87, 82.13, 56.09, 49.59, 28.06 (d, J = 7.3 Hz), 27.91.

^{19}F NMR ($CDCl_3$, 376 MHz): δ –81.52 (d, J = 74.9 Hz).

HRMS (ESI-TOF) m/z: $[M + H]^+$ Calcd. for $C_{30}H_{46}F_2N_3O_{10}$ 646.3151; found 646.3158 and $[M + Na]^+$ Calcd. for $C_{30}H_{45}F_2N_3NaO_{10}$ 668.2971; found 668.2977.

Deoxyfluorination and Authentic fluoroarene standard

1-Fluoro-2-methoxy- *N,N′,N′′′,N′′′′*-tetra-Boc-benzyl guanidine ([^{19}F]35-i)

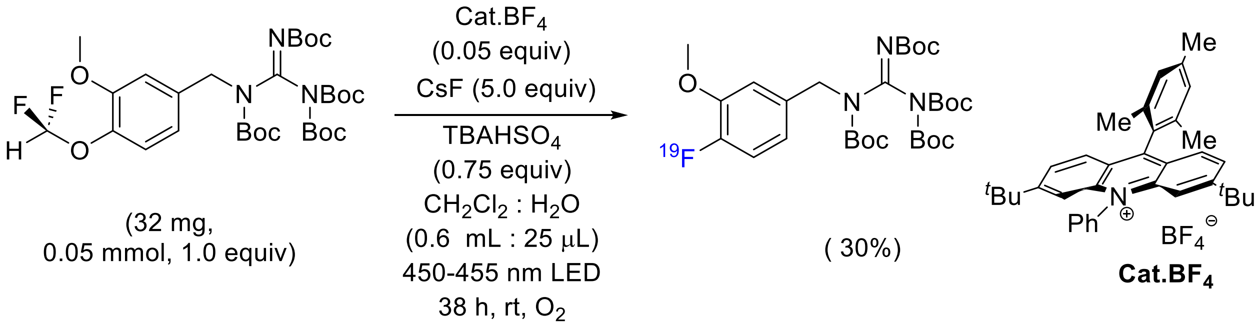

General conditions **I** were followed using **S35** (32 mg, 0.05 mmol, 1.0 equiv), Mes-Acr-$Ph^+BF_4^-$ (0.05 equiv), CsF (5.0 equiv), $TBAHSO_4$ (0.75 equiv), CH_2Cl_2 : H_2O (0.1 M, 25:1), 450-455 nm LED, 38 h, 33 °C, O_2.

Purification: Gradient column chromatography [SiO_2, EtOAc:Hexanes 5:95 to 15:85)] to obtain the titled compound **[^{19}F]35-i** as white solid (9 mg, 30%).

R$_f$: 0.5 (EtOAc : Hexanes 1:9)

^{1}H NMR ($CDCl_3$, 400 MHz): δ 7.21 (dd, J = 8.7, 1.5 Hz, 1H), 6.99–6.94 (m, 2H), 4.97 (s, 2H), 3.88 (s, 3H), 1.48 (s, 9H), 1.43 (s, 27H).

^{13}C NMR ($CDCl_3$, 101 MHz): δ 157.62, 152.94, 151.47, 150.99, 147.70, 147.47 (d, J = 11.0 Hz), 144.63, 133.88 (d, J = 4.6 Hz), 120.83 (d, J = 9.5 Hz), 115.67 (d, J = 18.4 Hz), 114.36 (d, J = 2.0 Hz), 84.17, 83.99, 82.25, 56.47, 49.73, 28.20 (d, J = 9.2 Hz), 28.07.

^{19}F NMR ($CDCl_3$, 376 MHz): δ –137.60 (ddd, J = 15.0, 8.8, 5.5 Hz).

HRMS (ESI-TOF) m/z: $[M + H]^+$ Calcd. for $C_{29}H_{45}FN_3O_9$ 598.3140; found 598.3139 and $[M + Na]^+$ Calcd. for $C_{29}H_{44}FN_3NaO_9$ 620.2959; found 620.2958.

1-Fluoro-2-methoxy-benzyl guanidine-2,2,2-trilfluoracetate ([^{19}F]35)

A 4 mL scintillation vial was charged with 1-fluoro-2-methoxy-tetra-Boc-benzyl guanidine **[^{19}F]35-i** and TFA (200 µL) and the reaction mixture was heated at 80 °C for 4 h. Upon completion of the reaction, TFA was evaporated under a stream of N_2 to yield **[^{19}F]35** as dark brown solid.

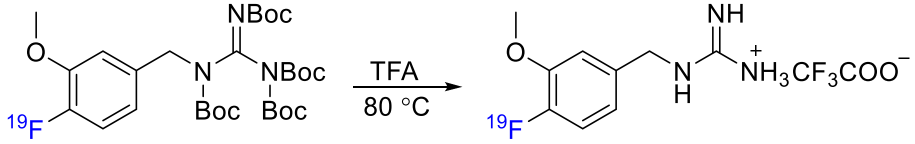

^{1}H NMR (MeOD-d^4, 400 MHz): δ 7.11–7.07 (m, 2H), 6.89 (ddd, J = 6.4, 4.2, 2.2 Hz, 1H), 4.38 (s, 2H), 3.89 (s, 3H).

^{13}C NMR (MeOD-d^4, 101 MHz): δ 167.97 (q, J = 35.9Hz), 158.76 (d, J = 5.6 Hz), 154.56, 152.12, 149.33 (d, J = 10.9 Hz), 134.18 (d, J = 3.9 Hz), 120.67 (d, J = 7.1 Hz), 116.99 (d, J = 18.8 Hz), 113.98 (d, J = 1.9 Hz), 56.69, 45.53.

^{19}F NMR (MeOD-d^4, 376 MHz): δ –138.29 (ddd, J = 11.9, 8.5, 4.3 Hz).

HRMS (ESI-TOF) m/z: $[M + H]^+$ Calcd. for $C_9H_{13}FN_3O$ 198.1043; found 198.1041.

Radio-HPLC analysis and characterization

One-pot experiment

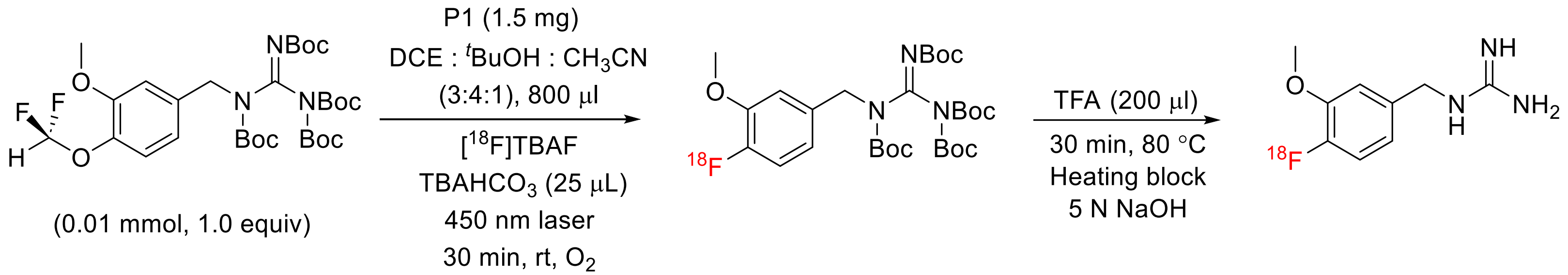

Entry	Activity at starting	Activity at EOS	Alumina Filtration	Injected Dose	Collected Dose	Isolation Time	Decay Corrected	Filtration Yield	HPLC Purity of Filtrate	%Yield
1*	34.1 mCi	26.9 mCi	11.78 mCi	2.70 mCi	710 µCi	14.9 min	2.46 mCi	43.79%	28.86 %	12.63%
2	16.95 mCi	13.11 mCi	6.81 mCi	1276 µCi	178 µCi	14.9 min	1167.30 µCi	51.94 %	15.24 %	7.92%
										**Average %Yield: 10.3% (n=2)**

Table S41: HPLC isolated RCYs for **[18F]35**

HPLC Conditions method – 1 : Column: Phenomenex, Kinetex® 5µm F5 100 Å, 250 ✕ 4.6 mm LC Column

Solvent A: 0.1% TFA water, Solvent B: 0.1% TFA acetonitrile; Isocratic / Gradient elution: 2% Solvent B for 0 to 2 min, 2% – 45% Solvent B for 2 to 22.5 min. Flow rate: 1 mL/min

*Set up on 34.1 mCi to get enough ^{18}F-product for imaging study

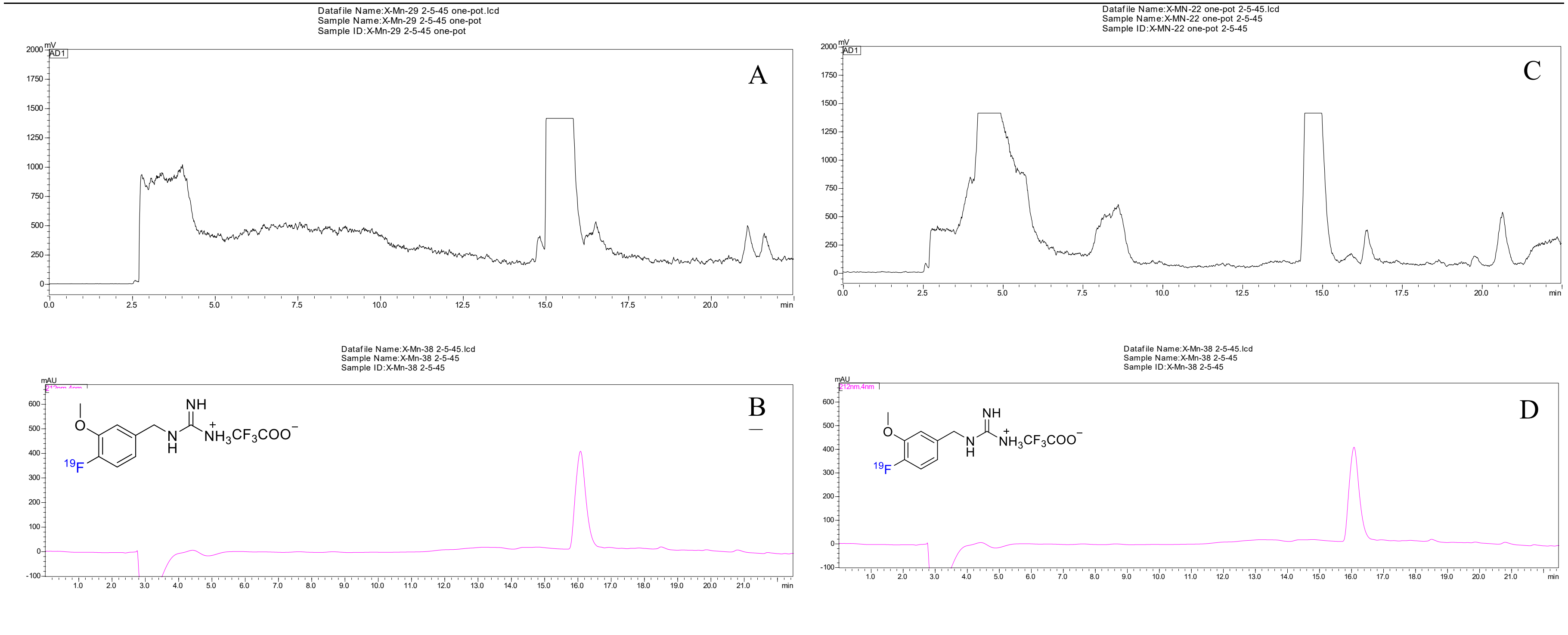

Figure S43: Radio-HPLC analysis of reaction mixture from **S35**. Reaction mixture with HPLC method 1 (A), authentic **[^{19}F]35** with HPLC method 1 (B), reaction mixture with HPLC method 1 (C), and authentic **[^{19}F]35** with HPLC method 1 (D).

Two-steps experiment

Step 1: Deoxyradiofluorination

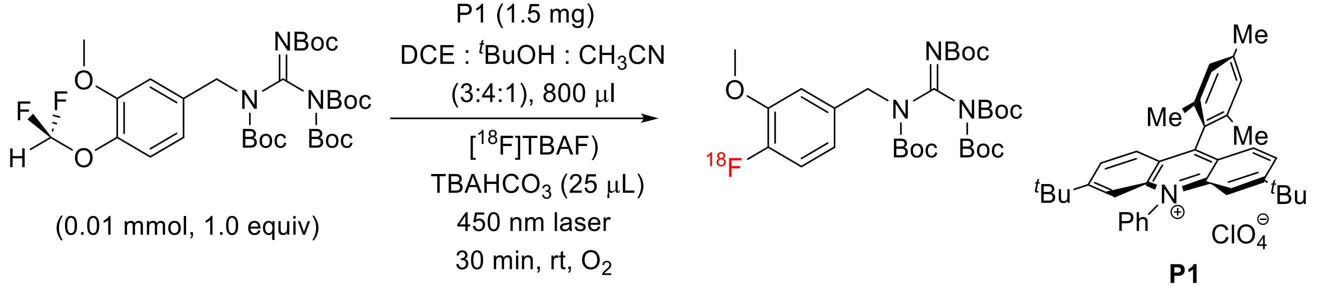

Entry	Activity at starting	Activity at EOS	Alumina Filtration	Injected Dose	Collected Dose	Isolation Time	Decay Corrected	Filtration Yield	HPLC Purity of Filtrate	%Yield
1	7.43 mCi	6.35 mCi	963 µCi	318 µCi	139 µCi	9.7 min	300.21 µCi	15.16%	46.30%	7.01%

Table S42: HPLC isolated RCYs for **[^{18}F]35-i**

HPLC Conditions method – 1: Column: Phenomenex, Kinetex® 5µm F5 100 Å, 250 ✕ 4.6 mm LC Column

Solvent A: 0.1% TFA water, Solvent B: 0.1% TFA acetonitrile; Isocratic / Gradient elution: 70% Solvent B for 0 to 2 min, 70% – 100% Solvent B for 2 to 22.5 min. Flow rate: 1 mL/min

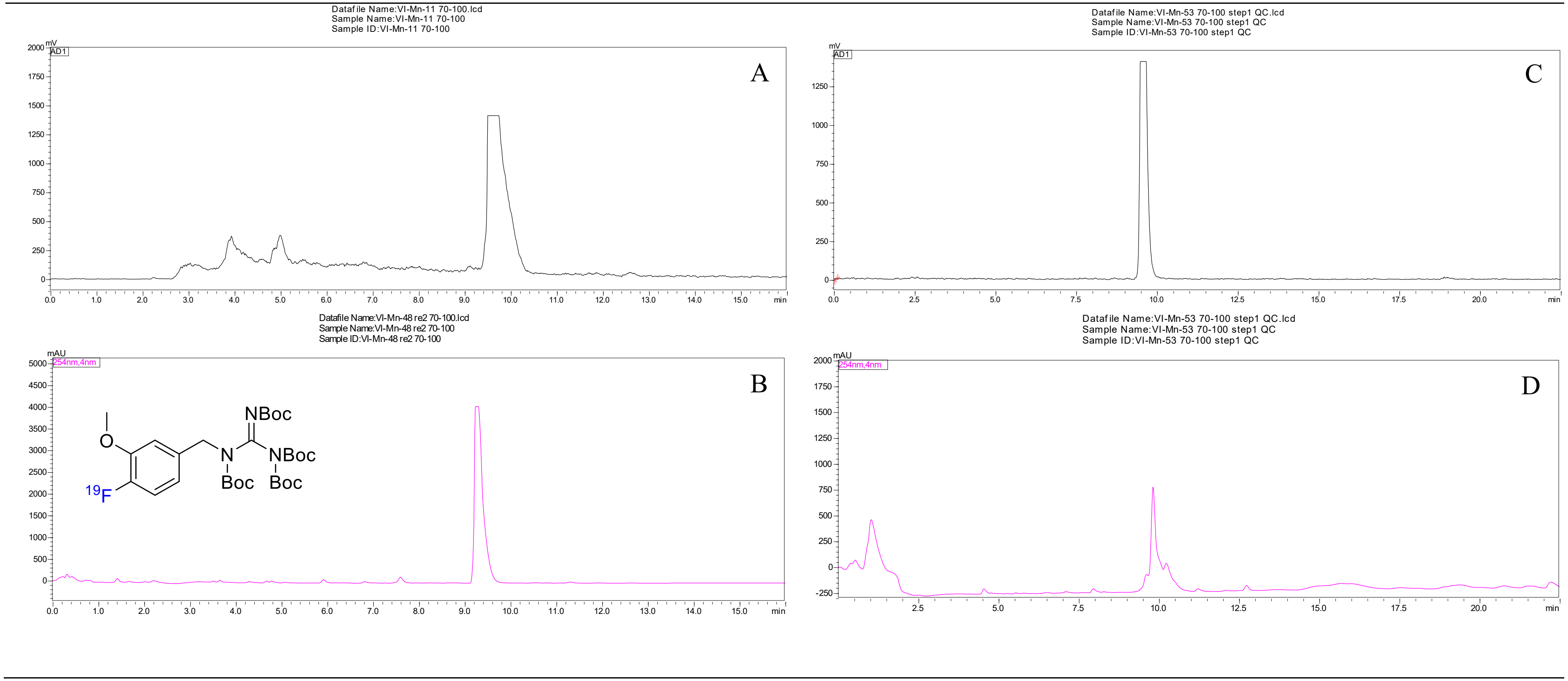
Datafile Name:VI-Mn-11 70-100.lcd
Sample Name:VI-Mn-11 70-100
Sample ID:VI-Mn-11 70-100
A
Datafile Name:VI-Mn-53 70-100 step1 QC.lcd
Sample Name:VI-Mn-53 70-100 step1 QC
Sample ID:VI-Mn-53 70-100 step1 QC
C
Datafile Name:VI-Mn-48 re2 70-100.lcd
Sample Name:VI-Mn-48 re2 70-100
Sample ID:VI-Mn-48 re2 70-100
B
NBoc
NBoc
Boc
Boc
19F
Datafile Name:VI-Mn-53 70-100 step1 QC.lcd
Sample Name:VI-Mn-53 70-100 step1 QC
Sample ID:VI-Mn-53 70-100 step1 QC
D

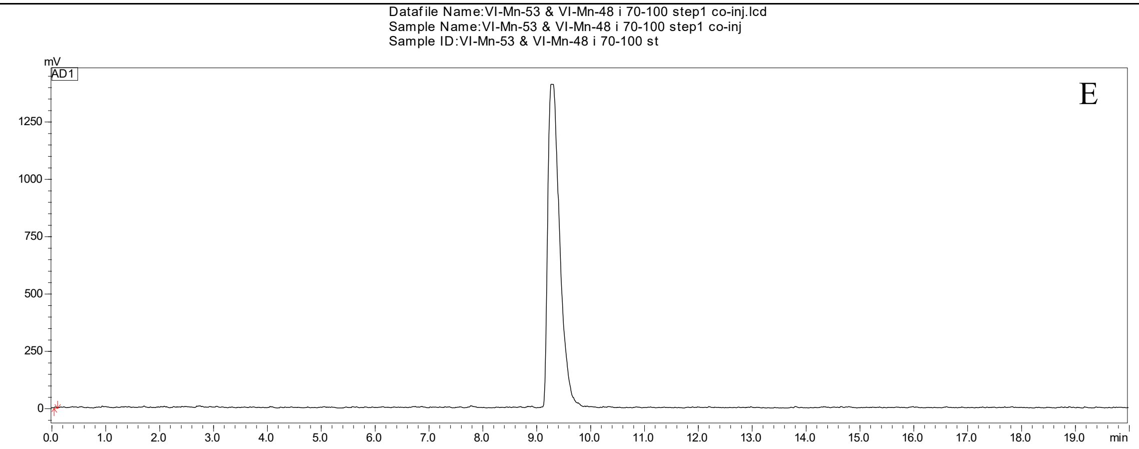

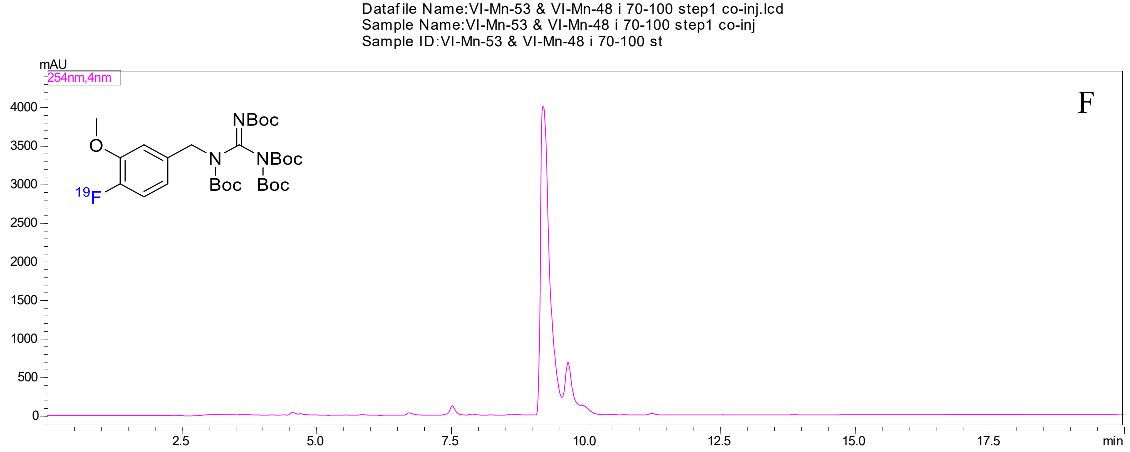

Figure S44: Radio-HPLC analysis of reaction mixture from **S35-i**. Reaction mixture with HPLC method 1 (A), authentic **[^{19}F]35-i** with HPLC method 1 (B), QC for **[^{18}F]35-i** (C and D), and Co-injection of **[^{18}F]35-i** and **[^{19}F]35-i** (E and F).

HPLC Conditions for QC and Co-injection: Column: Phenomenex, Kinetex® 5μm F5 100 Å, 250 × 4.6 mm LC Column
Solvent A: 0.1% TFA water, Solvent B: 0.1% TFA acetonitrile; Isocratic / Gradient elution: 70% Solvent B for 0 to 2 min, 70% – 100% Solvent B for 2 to 22.5 min. Flow rate: 1 mL/min

Step 2: Boc deprotection on isolated [^{18}F]35-i tracer

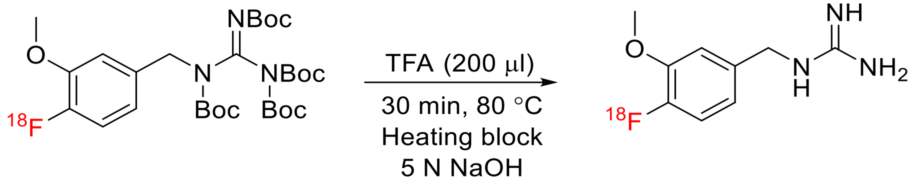

Entry	Activity at starting	Activity at EOS	Alumina Filtration	Injected Dose	Collected Dose	Isolation Time	Decay Corrected	Filtration Yield	HPLC Purity of Filtrate	%Yield
2	16.95 mCi	13.11 mCi	6.81 mCi	49 µCi	42 µCi	14.9 min	44.82µCi	51.94 %	93.70%	48.66%

Table S43: HPLC isolated RCYs for **[^{18}F]35**

HPLC conditions – method 1: Column: Phenomenex, Kinetex® 5µm F5 100 Å, 250 × 4.6 mm LC Column

Solvent A: 0.1% TFA water, Solvent B: 0.1% TFA acetonitrile; Isocratic / Gradient elution: 2% Solvent B for 0 to 2 min, 2% – 45% Solvent B for 2 to 22.5 min. Flow rate: 1 mL/min

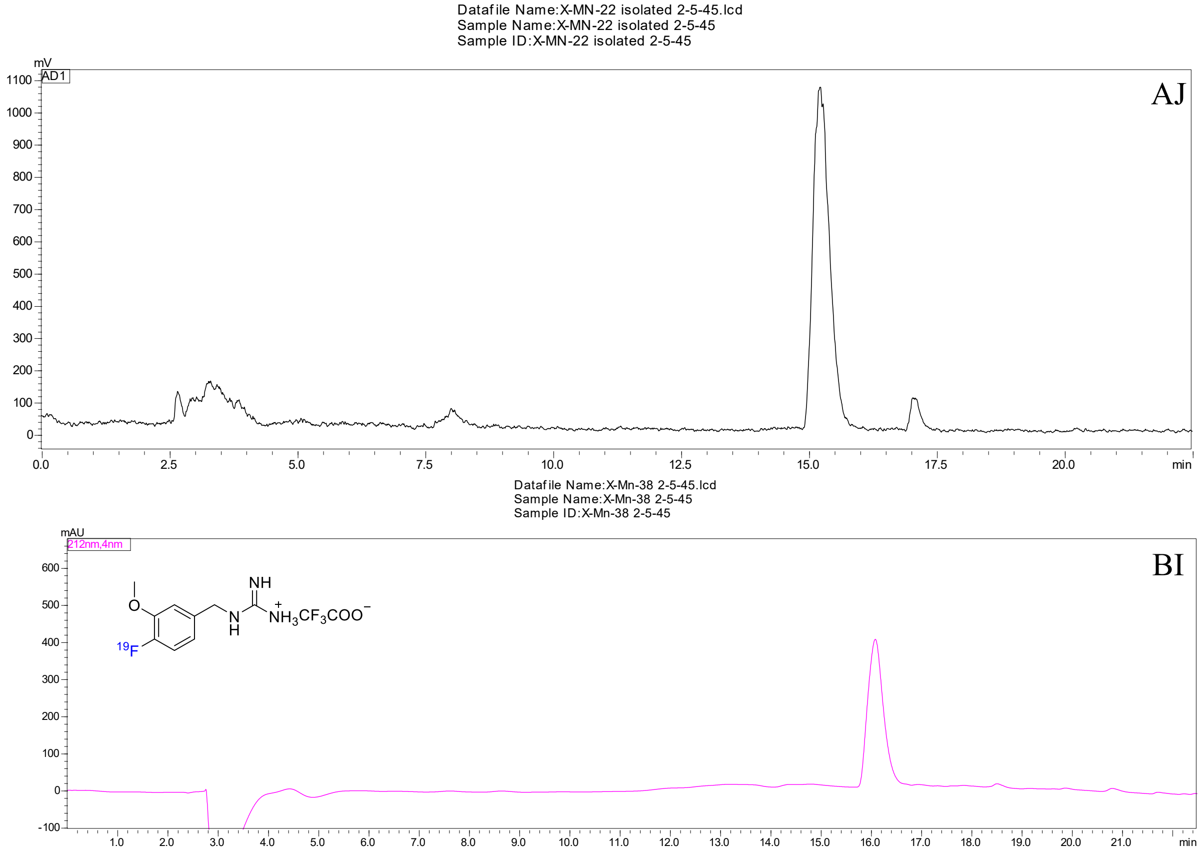

Figure S45: Radio-HPLC analysis of reaction mixture from **S35**. Reaction mixture with HPLC method 1 (A), and authentic **[^{19}F]35** with HPLC method 1 (B)

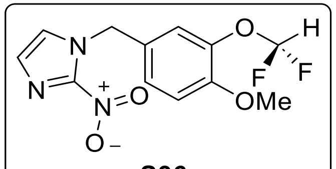

Arene substrate:

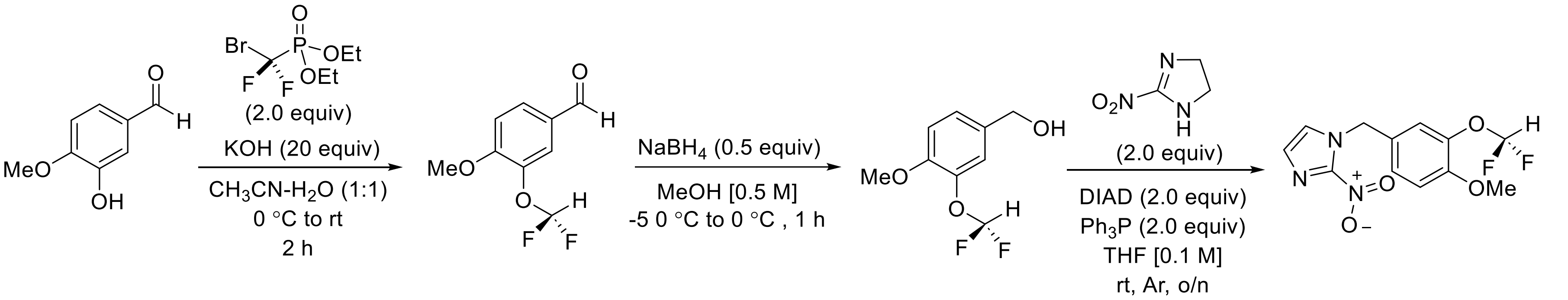

3-(Difluoromethoxy)-4-methoxybenzaldehyde (S36-I1)

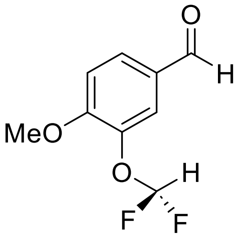

General conditions **A** were followed using 3-hydroxy-4-methoxybenzaldehyde (608 mg, 4.0 mmol, 1.0 equiv), $BrCF_2P(O)(OC_2H_5)_2$ (3.2 g, 8.0 mmol, 2.0 equiv), KOH (g, 40 mmol, 20 equiv), CH_3CN: H_2O (7.5 mL : 7.5 mL) for 2 h.

Purification: Gradient column chromatography [SiO_2, EtOAc:Hexanes 05:95 to 20:80)] to obtain white solid of **S36-I1** (411 mg, 51%).

R_f : 0.5 (EtOAc : Hexanes 7:3)

^{1}H NMR ($CDCl_3$, 400 MHz): δ 9.86 (s, 1H), 7.74 (dd, J = 8.1, 1.5 Hz, 1H), 7.68 (s, 1H), 7.09 (d, J = 8.6 Hz, 1H), 6.67 (t, J = 74.3 Hz, 1H), 3.97 (s, 3H).

^{13}C NMR ($CDCl_3$, 101 MHz): δ 190.06, 156.36, 140.45 (t, J = 2.9 Hz), 130.13, 129.91, 122.17, 115.88 (t, J = 262.8 Hz), 112.38, 56.35.

^{19}F NMR ($CDCl_3$, 376 MHz): δ –81.84 (d, J = 73.4 Hz).

HRMS (ESI-TOF) m/z: $[M + H]^+$ Calcd. for $C_9H_9F_2O_3$ 203.0520; found 203.0516.

(3-(Difluoromethoxy)-4-methoxyphenyl)methanol (**S36-I2**)

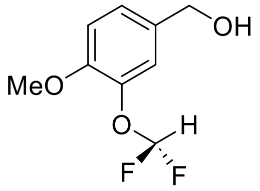

To a solution of 3-(difluoromethoxy)-4-methoxybenzaldehyde **S36-I1** (300 mg, 1.49 mmol, 1.0 equiv) in MeOH was added $NaBH_4$ (28 mg, 0.74 mmol, 0.5 equiv) at –5 °C and the resulting reaction mixture allowed to stir until the staring material consumed (1 h, TLC monitored). Upon completion of the reaction, saturated solution of NH_4Cl was added and MeOH was evaporated under reduced pressure. The aqueous layer was extracted with EtOAc (2 × 10 mL). The combined organics dried (Na_2SO_4) and concentrated under pressure, which was subsequently used for the next step without purification. The purity of the product >95% based on NMR characterisation.

White solid (237 mg, 78%)

R_f : 0.3 (EtOAc : Hexanes 7:3)

^{1}H NMR ($CDCl_3$, 400 MHz): δ 7.21–7.15 (m, 2H), 6.95 (d, J = 8.9 Hz, 1H), 6.56 (t, J = 75.3 Hz, 1H), 4.62 (s, 2H), 3.88 (s, 3H).

^{13}C NMR ($CDCl_3$, 101 MHz): δ 150.68, 140.08 (t, J = 2.9 Hz), 134.03, 125.34, 121.39, 116.30 (t, J = 259.6 Hz), 113.72, 112.78, 64.60, 56.24.

^{19}F NMR ($CDCl_3$, 376 MHz): δ –81.39 (d, J = 75.0 Hz).

HRMS (ESI-TOF) m/z: $[M + Na]^+$ Calcd. for $C_9H_{10}F_2NaO_3$ 227.0496; found 227.0494.

1-(3-(Difluoromethoxy)-4-methoxybenzyl)-2-nitro-1*H*-imidazole (S36)

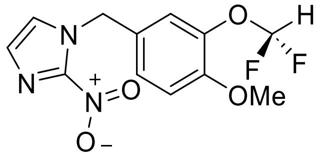

A 4 mL scintillation vial was charged with (3-(difluoromethoxy)-4-methoxyphenyl)methanol **S36-I2** (65 mg, 0.32 mmol, 1.0 equiv), 2-nitro-4,5-dihydro-1*H*-imidazole (72 mg, 0.64 mmol, 2.0 equiv), Ph_3P (184 mg, 0.70 mmol, 2.2 equiv). The screw-capped vial was purged and degassed with argon for three times. Anhydrous THF (1.6 mL, [0.2 M]) followed by DIAD (0.13 mL, 0.64 mmol, 2.0 equiv) were transferred by syringe and the screw-capped vial was stirred at room temperature overnight. Next morning, the reaction mixture diluted with EtOAc and H_2O. The aqueous layer was extracted with EtOAc (3 × 5 mL). The combined organics dried (Na_2SO_4) and concentrated under reduced pressure, which was subsequently purified by column chromatography.

Purification: Gradient column chromatography [SiO_2, EtOAc:Hexanes 30:70 to 50:50)] to obtain the titled compound **S36** as pale-yellow solid (52 mg, 54%).

R_f : 0.5 (EtOAc : Hexanes 5:5)

^{1}H NMR ($CDCl_3$, 400 MHz): δ 7.15 (d, J = 1.0 Hz, 1H), 7.08–7.06 (m, 3H), 6.96–6.94 (m, 1H), 6.55 (t, J = 75.6 Hz, 1H), 5.52 (s, 2H), 3.88 (s, 3H).

^{13}C NMR ($CDCl_3$, 101 MHz): δ 151.69, 140.16 (t, J= 2.8 Hz), 128.71, 127.00, 126.49, 125.78, 122.44, 115.96 (t, J= 260.9 Hz), 113.19, 56.23, 52.68.

^{19}F NMR ($CDCl_3$, 376 MHz): δ –81.76 (d, J = 77.1 Hz).

HRMS (ESI-TOF) m/z: $[M + Na]^+$ Calcd. for $C_{12}H_{12}F_2N_3O_4$ 300.0796; found 300.0791.

Authentic fluoroarene standard

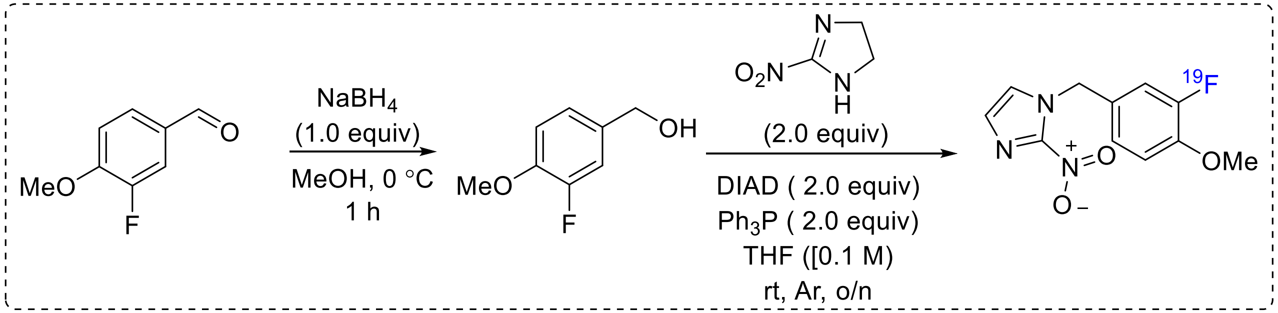

1-(3-Fluoro-4-methoxybenzyl)-2-nitro-1*H*-imidazole ([^{19}F]36)

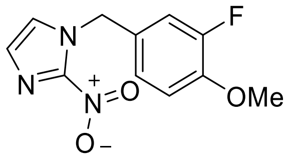

A 20 mL scintillation vial was charged with (3-fluoro-4-methoxyphenyl)methanol (100 mg, 0.64 mmol, 1.0 equiv), 2-nitro-4,5-dihydro-1*H*-imidazole (514 mg, 1.28 mmol, 2.0 equiv), Ph_3P (367 mg, 1.40 mmol, 2.2 equiv). The screw-capped vial was purged and degassed with argon for three times. Anhydrous THF (3.2 mL, [0.2 M]) followed by DIAD (0.25 mL, 1.28 mmol, 2.0 equiv) were transferred by syringe and the screw-capped vial was stirred at room temperature overnight. Next morning, the reaction mixture diluted with EtOAc and H_2O. The aqueous layer was extracted with EtOAc (3 ×5 mL). The combined organics dried (Na_2SO_4) and concentrated under reduced pressure, which was subsequently purified by column chromatography.

Purification: Gradient column chromatography [SiO_2, EtOAc:Hexanes 30:70 to 50:50)] to obtain the titled compound **[^{19}F]36** as pale-yellow solid (59 mg, 37%).

R_f : 0.5 (EtOAc : Hexanes 5:5)

^{1}H NMR ($CDCl_3$, 400 MHz): δ 7.15 (d, J = 1.0 Hz, 1H), 7.06 (d, J = 0.7 Hz, 1H), 6.98–6.92 (m, 3H), 5.51 (s, 2H), 3.88 (s, 3H).

^{13}C NMR ($CDCl_3$, 101 MHz): δ 153.82, 151.35, 148.30 (t, J = 11.1 Hz), 128.70, 126.96 (d, J = 5.8 Hz), 125.72, 124.17 (d, J = 3.6 Hz), 115.92 (t, J = 20.1 Hz), 113.92 (t, J = 2.2 Hz), 56.43, 52.72.

^{19}F NMR ($CDCl_3$, 376 MHz): δ –133.20 (ddd, J = 8.3, 6.9, 1.6 Hz).

HRMS (ESI-TOF) m/z: $[M + H]^+$ Calcd. for $C_{11}H_{11}FN_3O$ 252.0784; found 252.0779.

<u>Radio-HPLC analysis and characterization</u>

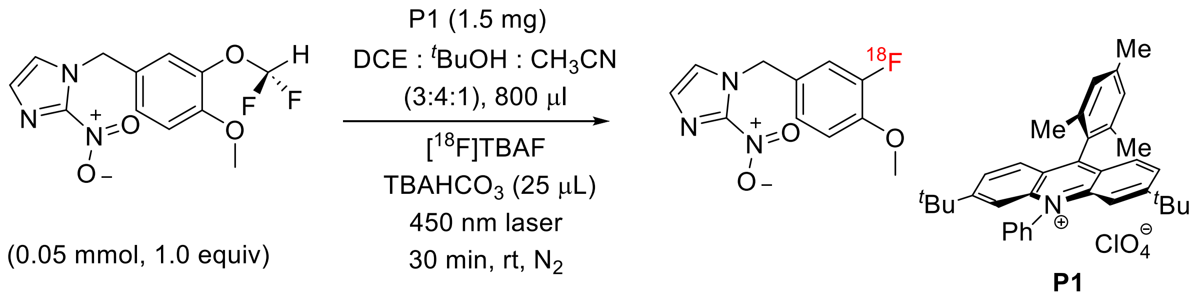

Entry	Activity at starting	Activity at EOS	Alumina Filtration	Injected Dose	Collected Dose	Isolation Time	Decay Corrected	Filtration Yield	HPLC Purity of Filtrate	%Yield
1*	9.70 mCi	7.71 mCi	1.27 mCi	174 µCi	50 µCi	12.3 min	160.96 µCi	16.47%	31.06%	5.12%
2**	31.7 mCi	23.4 mCi	6.0 mCi	1506 µCi	198 µCi	12.3 min	1395.65 µCi	25.64%	14.18%	3.63%
										**Average %Yield: 4.4% (n=2)**

Table S44: HPLC isolated RCYs for **[^{18}F]36.**

HPLC Conditions – method 1: Column: Phenomenex, Kinetex® 5µm F5 100 Å, 250 ✕ 4.6 mm LC Column

Solvent A: 0.1% TFA water, Solvent B: 0.1% TFA acetonitrile; Isocratic / Gradient elution: 20% Solvent B for 0 to 2 min, 20% – 95% Solvent B for 2 to 22.5 min. Flow rate: 1 mL/min

*O_2 sparging instead of N_2

**Set up on 31.7 mCi to get enough ^{18}F-tracer for imaging study

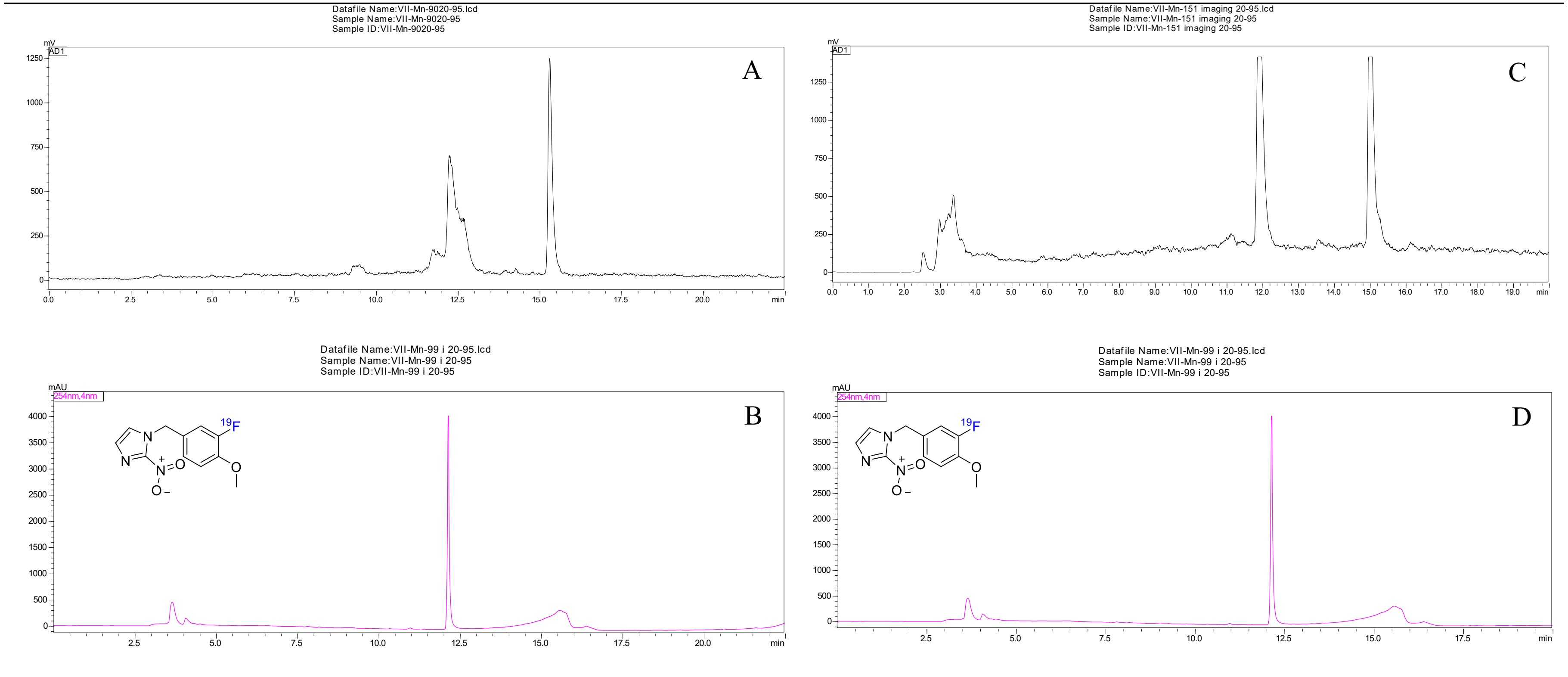

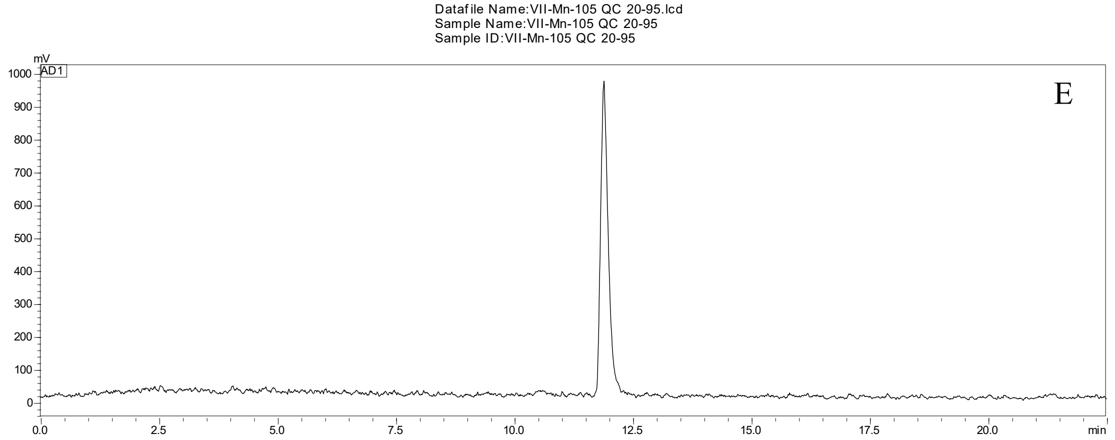

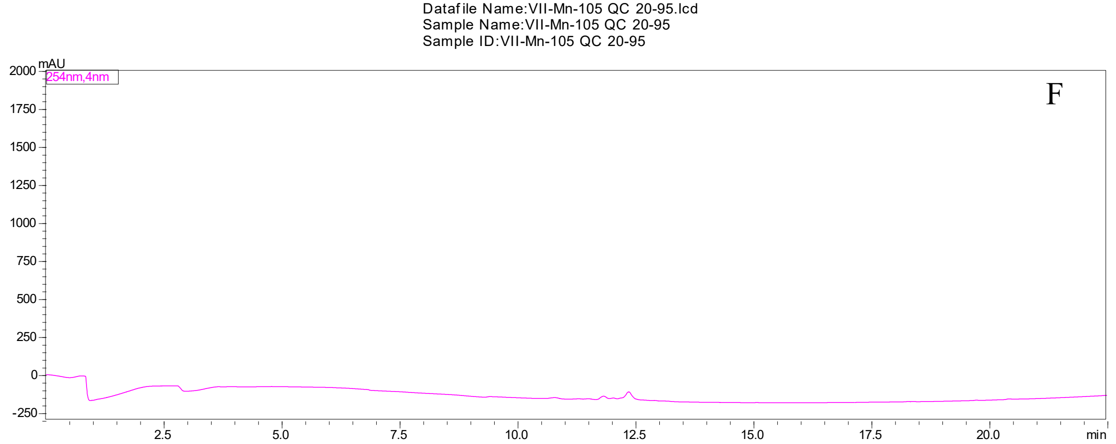

Figure S46: Radio-HPLC analysis of reaction mixture from **S36**. Reaction mixture with HPLC method 1 (A), authentic **[^{19}F]36** with HPLC method 1 (B), reaction mixture with HPLC method 1 (C), authentic **[^{19}F]36** with HPLC method 1 (D), and QC for **[^{18}F]36** (E and F).

HPLC Conditions for QC: Column: Phenomenex, Kinetex® 5µm F5 100 Å, 250 × 4.6 mm LC Column

Solvent A: 0.1% TFA water, Solvent B: 0.1% TFA acetonitrile; Isocratic / Gradient elution: 2% Solvent B for 0 to 2 min, 20% – 95% Solvent B for 2 to 22.5 min. Flow rate: 1 mL/min.

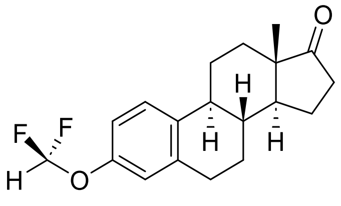

S37

Arene substrate:

(8*R*,9*S*,13*S*,14*S*)-3-(Difluoromethoxy)-13-methyl-6,7,8,9,11,12,13,14,15,16-decahydro-17*H*-cyclopenta[*a*]phenanthren-17-one (S37)

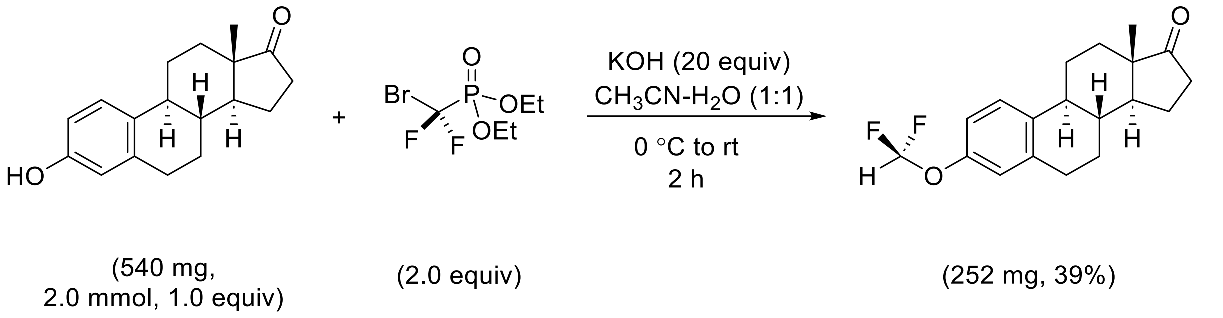

General conditions **A** were followed using estrone (540 mg, 2.0 mmol, 1.0 equiv), $BrCF_2P(O)(OC_2H_5)_2$ (1.068 g, 4.0 mmol, 2.0 equiv), KOH (2.24 g, 40 mmol, 20 equiv), CH_3CN: H_2O (5 mL : 5 mL) for 2 h.

Purification: Gradient column chromatography [SiO_2, EtOAc:Hexanes 05:95 to 10:90)] to obtain white solid of **S37** (252 mg, 39%).

R_f : 0.6 (EtOAc : Hexanes 1:9)

^{1}H NMR ($CDCl_3$, 400 MHz): δ 7.26 (d, J = 8.9 Hz, 1H), 6.90 (dd, J = 8.5, 2.5 Hz, 1H), 6.86 (d, J = 2.3 Hz, 1H), 6.42 (t, J = 74.3 Hz, 1H), 2.92 (dd, J = 8.8, 4.2 Hz, 1H), 2.52 (dd, J = 19.0, 8.8 Hz, 2H), 2.43–2.35 (m, 1H), 2.29 (td, J = 10.5, 4.3 Hz, 1H), 2.19–2.10 (m, 1H), 2.09–1.99 (m, 2H), 1.99–1.92 (m, 1H), 1.68–1.58 (m, 2H), 1.55–1.39 (m, 4H), 0.92 (s, 3H).

^{13}C NMR ($CDCl_3$, 126 MHz): δ 220.76, 149.29 (t, J = 2.9 Hz), 138.56, 137.12, 126.85, 119.77, 116.94, 116.19 (t, J = 258.9 Hz), 50.49, 48.03, 44.15, 38.15, 35.94, 31.62, 29.55, 26.40, 25.90, 21.68, 13.92.

^{19}F NMR ($CDCl_3$, 376 MHz): δ –80.36 (d, J = 73.5 Hz).

HRMS (ESI-TOF) m/z: $[M + H]^+$ Calcd. for $C_{19}H_{23}F_2O_2$ 321.1666; found 321.1671.

Authentic fluoroarene standard

^{19}F-Reference compound – **[^{19}F]37**, data are comparable to that reported in the literature.[10]

Radio-HPLC analysis and characterization

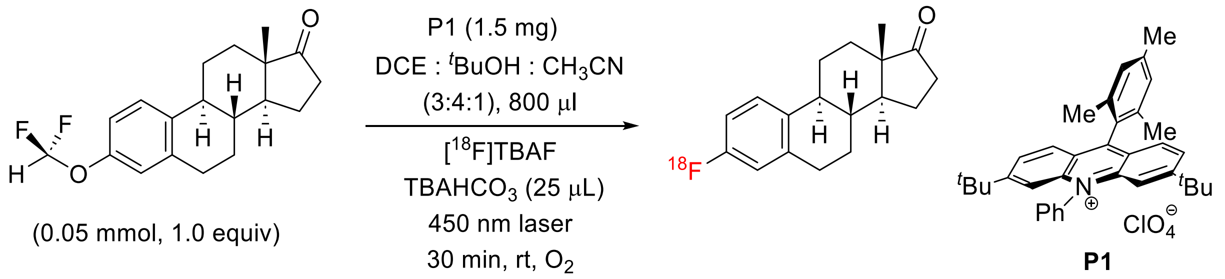

Entry	Activity at starting	Activity at EOS	Alumina Filtration	Injected Dose	Collected Dose	Isolation Time	Decay Corrected	Filtration Yield	HPLC Purity of Filtrate	%Yield
1	8.04 mCi	6.22 mCi	1.96 mCi	354 μCi	46 μCi	13.2 min	325.13 μCi	31.55%	14.14%	4.46%
2	16.02 mCi	12.98 mCi	4.02 mCi	484 μCi	47 μCi	13.2 min	444.91 μCi	30.97%	10.56%	3.27%
3	11.06 mCi	8.67 mCi	2.06 mCi	512 μCi	31 μCi	13.2 min	471.08 μCi	23.76%	6.49%	1.54%

Average %Yield: 3.1% (n=3)

Table S45: HPLC isolated RCYs for **[^{18}F]37**

HPLC Conditions – method 1: Column: Phenomenex, Kinetex® 5µm F5 100 Å, 250 ✕ 4.6 mm LC Column

Solvent A: 0.1% TFA water, Solvent B: 0.1% TFA acetonitrile; Isocratic / Gradient elution: 40% Solvent B for 0 to 2 min, 40% – 95% Solvent B for 2 to 22.5 min. Flow rate: 1 mL/min

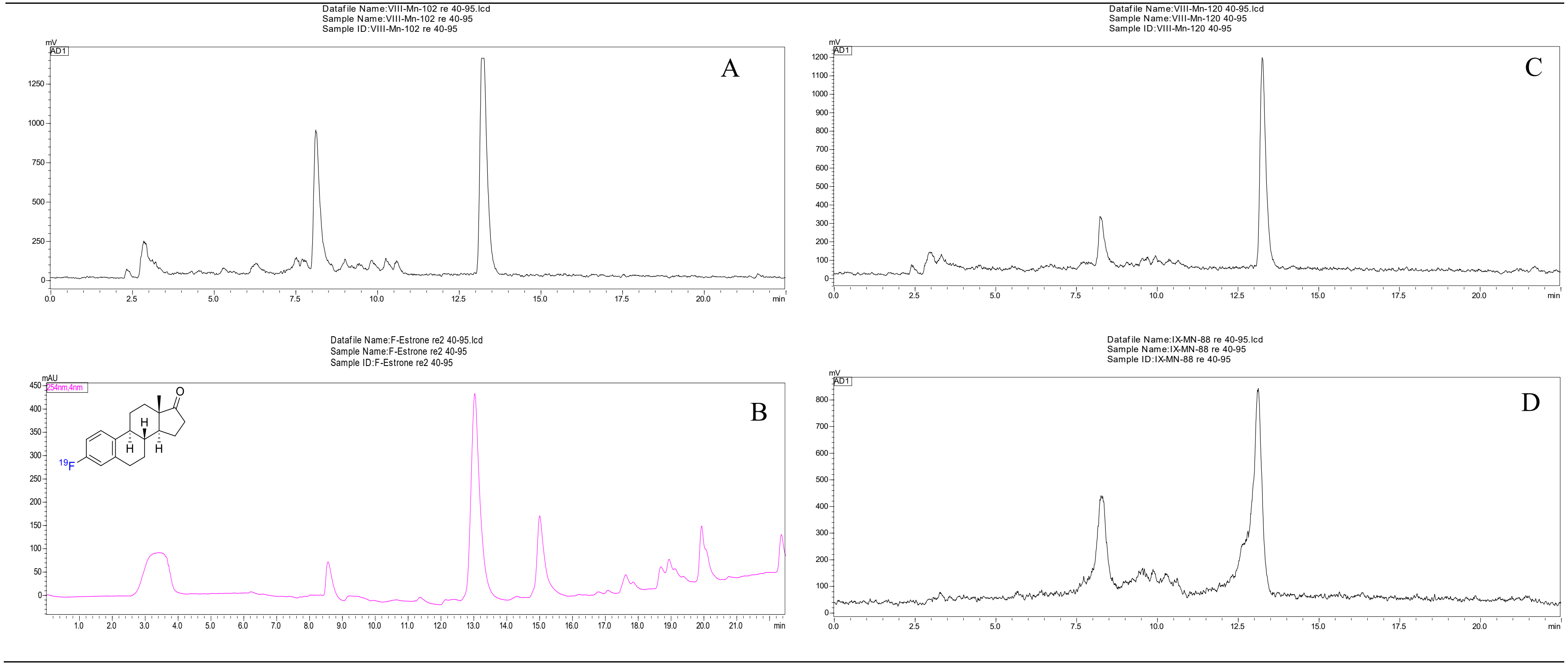

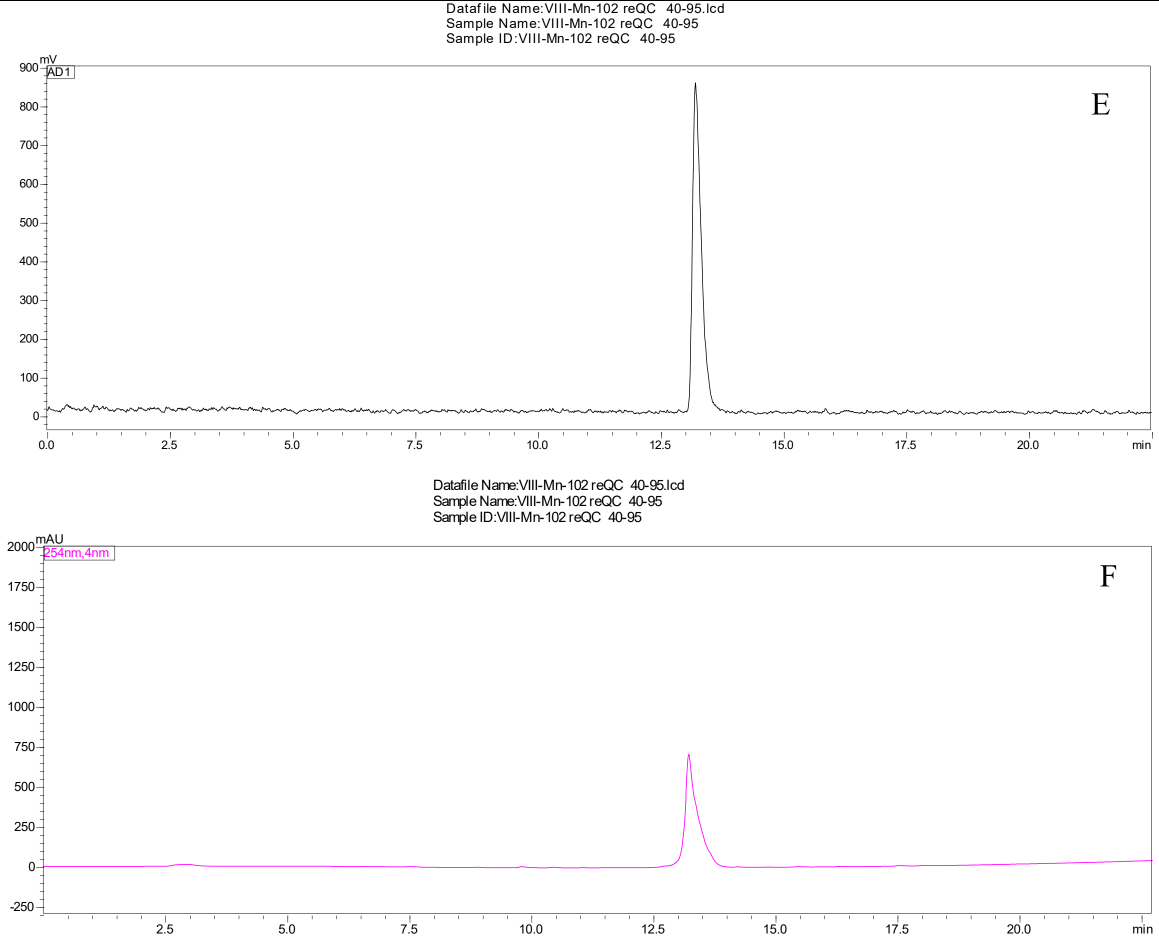

Figure S47: Radio-HPLC analysis of reaction mixture from **S37**. Reaction mixture with HPLC method 1 (A), authentic **[^{19}F]37** with HPLC method 1 (B), reaction mixture with HPLC method 1 (C), authentic **[^{19}F]37** with HPLC method 1 (D), and QC for **[^{18}F]37** (E and F).

HPLC Conditions for QC: Column: Phenomenex, Kinetex® 5µm F5 100 Å, 250 × 4.6 mm LC Column

Solvent A: 0.1% TFA water, Solvent B: 0.1% TFA acetonitrile; Isocratic / Gradient elution: 40% Solvent B for 0 to 2 min, 40% – 95% Solvent B for 2 to 22.5 min. Flow rate: 1 mL/min.

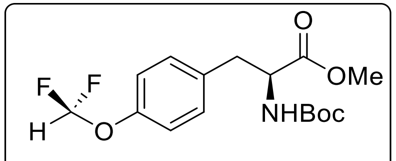

L–S38

Arene substrate:

Methyl (*S*)-2-((*tert*-butoxycarbonyl)amino)-3-(4-(difluoromethoxy)phenyl)propanoate (L–S38)

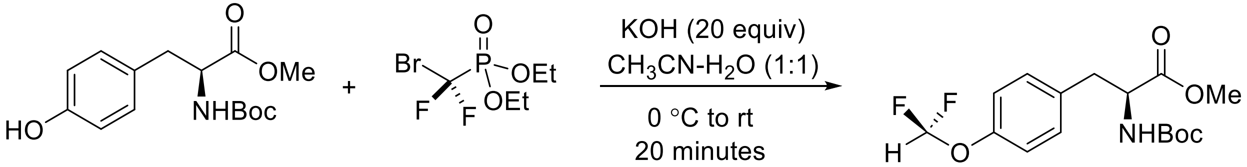

General conditions **A** were followed using methyl (*tert*-butoxycarbonyl)-L-tyrosinate (260 mg, 0.87 mmol, 1.0 equiv), $BrCF_2P(O)(OC_2H_5)_2$ (469 mg, 1.75 mmol, 2.0 equiv), KOH (974 mg, 17.4 mmol, 20 equiv), CH_3CN: H_2O (7.5 mL : 7.5 mL) for 20 minutes.

Purification: Gradient column chromatography [SiO_2, EtOAc:Hexanes 05:95 to 20:80)] to obtain white solid of **L–S38** (77 mg, 26%).

R_f : 0.86 (EtOAc : Hexanes 7:3)

^{1}H NMR ($CDCl_3$, 400 MHz): δ 7.12 (d, J= 8.5 Hz, 2H), 7.04 (d, J= 8.5 Hz, 2H), 6.48 (t, J= 73.9 Hz, 1H), 4.98 (d, J= 7.4 Hz, 1H), 4.60 (dd, J= 13.1, 6.1 Hz, 1H), 3.72 (s, 3H), 3.12 (dd, J= 13.8, 5.6 Hz, 1H), 3.02 (dd, J= 13.8, 5.9 Hz, 1H), 1.41 (s, 9H).

^{13}C NMR ($CDCl_3$, 101 MHz): δ 172.26, 155.14, 150.39 (t, J = 2.9 Hz), 133.44, 130.82, 119.73, 116.03 (t, J = 259.6 Hz), 80.19, 54.48, 52.43, 37.82, 28.39.

^{19}F NMR ($CDCl_3$, 376 MHz): δ –80.69 (d, J =74.4 Hz).

HRMS (ESI-TOF) m/z: $[M + H]^+$ Calcd. for $C_{16}H_{22}F_2NO_5$ 346.1466; found 346.1461, and $[M + Na]^+$ Calcd. for $C_{16}H_{21}F_2NNaO_5$ 368.1285; found 368.1282.

Authentic fluoroarene standard

[19]F-Reference compound – **[^{19}F]38** was purchased from the commercial supplier. CAS Number 86129-35-3

Radio-HPLC analysis and characterization

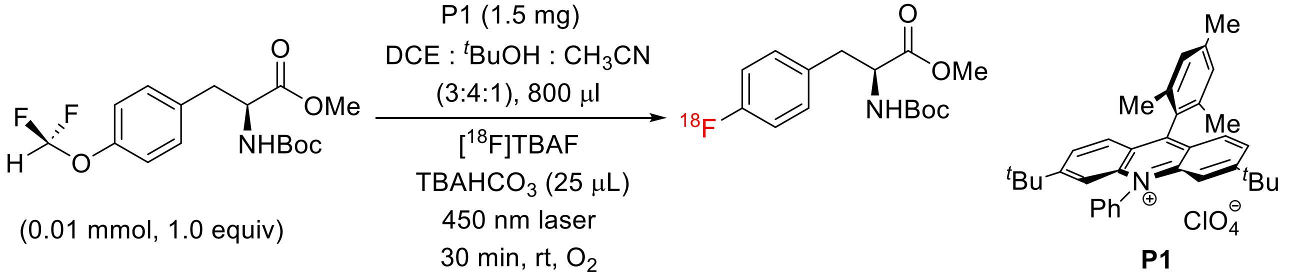

Entry	Activity at starting	Activity at EOS	Alumina Filtration	Injected Dose	Collected Dose	Isolation Time	Decay Corrected	Filtration Yield	HPLC Purity of Filtrate	%Yield
1	9.53 mCi	8.14 mCi	280 µCi	83 µCi	38 µCi	10.8 min	77.43 µCi	3.43%	49.07%	1.68%
2	19.8 mCi	14.01 mCi	2.22 mCi	453 µCi	22 µCi	10.0 min	422.60 µCi	15.87%	5.17 %	0.82%
3*	6.96 mCi	6.22 mCi	157 µCi	132 µCi	46 µCi	10.8 min	123.14 µCi	2.52%	37.35%	0.94%
										**Average %Yield: 1.1% (n=3)**

Supplementary **Table S45**: HPLC isolated RCYs for **[^{19}F]38**

HPLC Conditions – method 1: Column: Phenomenex, Kinetex® 5µm F5 100 Å, 250 ✕ 4.6 mm LC Column

Solvent A: 0.1% TFA water, Solvent B: 0.1% TFA acetonitrile; Isocratic / Gradient elution: 40% Solvent B for 0 to 2 min, 40% – 95% Solvent B for 2 to 22.5 min. Flow rate: 1 mL/min

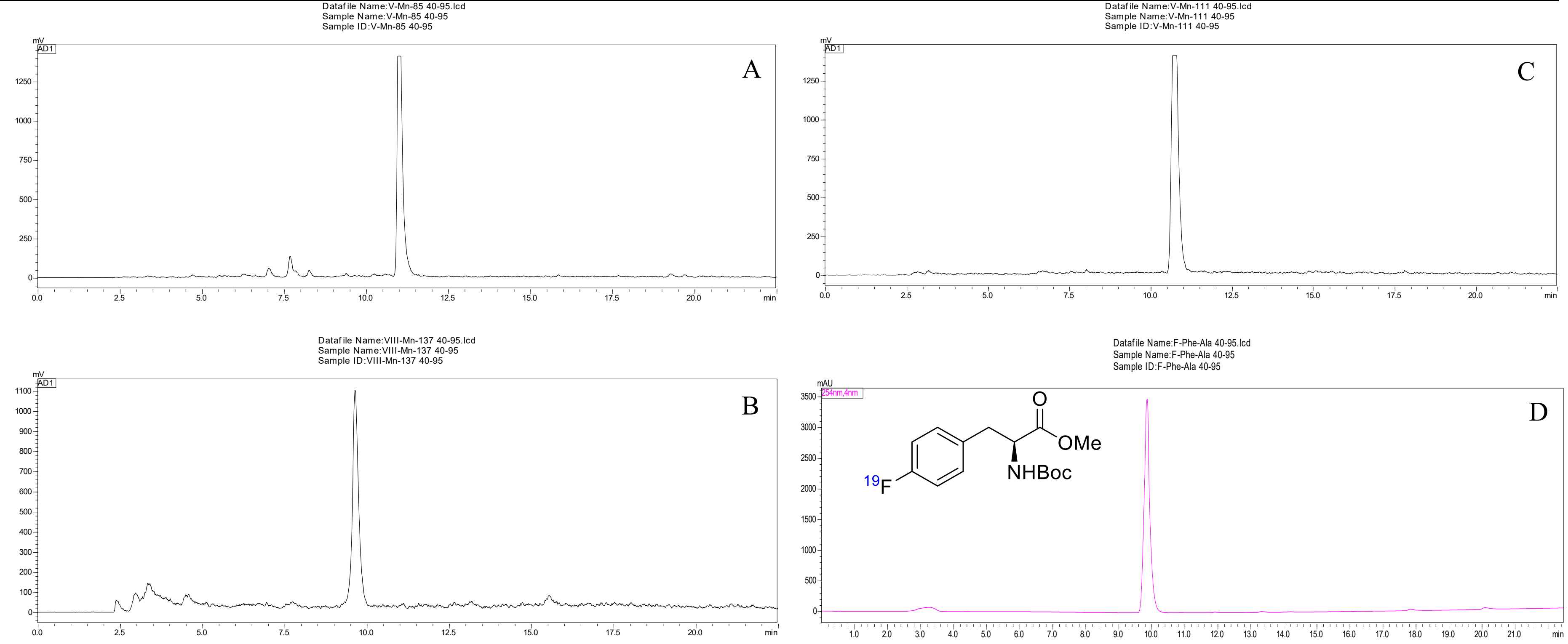

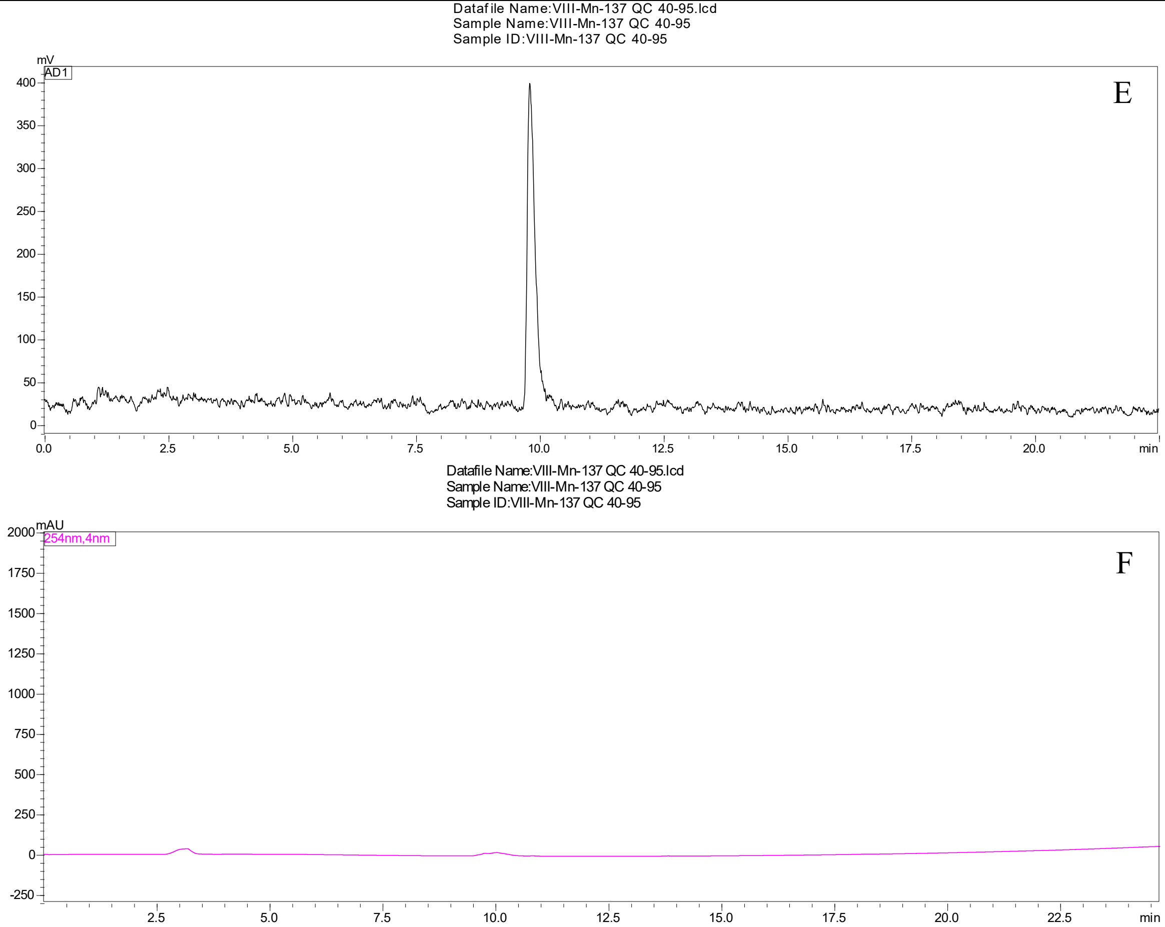

Figure S48: Radio-HPLC analysis of reaction mixture from **S38**. Reaction mixture with HPLC method 1 (A), reaction mixture with HPLC method 1 (B), reaction mixture with HPLC method 1 (C), authentic **[^{19}F]38** with HPLC method 1 (D), and QC for **[^{18}F]38** (E and F).

HPLC Conditions for QC: Column: Phenomenex, Kinetex® 5µm F5 100 Å, 250 × 4.6 mm LC Column

Solvent A: 0.1% TFA water, Solvent B: 0.1% TFA acetonitrile; Isocratic / Gradient elution: 40% Solvent B for 0 to 2 min, 40% – 95% Solvent B for 2 to 22.5 min. Flow rate: 1 mL/min.

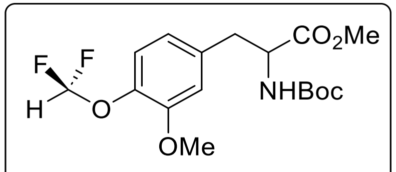

S39

<u>Arene substrate:</u>

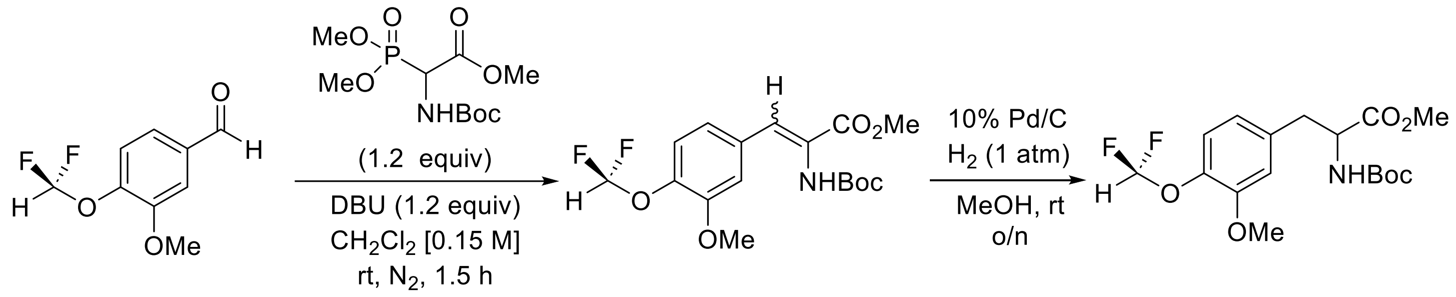

Methyl 2-((*tert*-butoxycarbonyl)amino)-3-(4-(difluoromethoxy)-3-methoxyphenyl)acrylate (S39-I1)

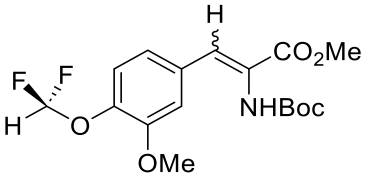

A 50 mL RBF was charged with 4-(difluoromethoxy)-3-methoxybenzaldehyde (655 mg, 3.0 mmol, 1.0 equiv), methyl 2-((*tert*-butoxycarbonyl)amino)-2-(dimethoxyphosphoryl)acetate (1070 mg, 3.6 mmol, 1.0 equiv). The screw-capped vial was purged and degassed with argon for three times. Anhydrous CH_2Cl_2 (15 mL, [0.2 M]) followed by DBU (0.54 mL, 3.6 mmol, 1.2 equiv) were transferred by syringe and the reaction mixture was stirred at room temperature for 1.5 h. The reaction mixture diluted with CH_2Cl_2 and H_2O. The aqueous layer was

extracted with CH_2Cl_2 (3 × 10 mL). The combined organics dried (Na_2SO_4) and concentrated under reduced pressure, which was subsequently purified by column chromatography.

Purification: Gradient column chromatography [SiO_2, EtOAc:Hexanes 5:95 to 30:70)] to obtain the titled compound **S39-I1** as white solid (930 mg, 83%).

R_f : 0.4 (EtOAc : Hexanes 3:7)

^{1}H NMR ($CDCl_3$, 400 MHz): δ 7.21 (d, J = 13.6 Hz, 2H), 7.14–7.08 (m, 2H), 6.57 (t, J = 75.4 Hz, 1H), 6.23 (bs, 1H), 3.87 (s, 3H), 3.86 (s, 3H), 1.39 (s, 9H).

^{13}C NMR ($CDCl_3$, 101 MHz): δ 165.99, 152.72, 140.48 (t, J = 2.9 Hz), 132.89, 129.25, 122.97, 121.94, 116.07 (t, J = 260.3 Hz), 113.64, 81.31, 56.01, 52.84, 28.22.

^{19}F NMR ($CDCl_3$, 376 MHz): δ –81.53 (d, J = 75.0 Hz).

HRMS (ESI-TOF) m/z: $[M + Na]^+$ Calcd. for $C_{17}H_{21}F_2NNaO_6$ 396.1235; found 396.1230.

Methyl 2-((*tert*-butoxycarbonyl)amino)-3-(4-(difluoromethoxy)-3-methoxyphenyl)propanoate (S39)

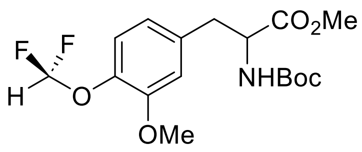

A 100 mL RBF was charged with methyl 2-((*tert*-butoxycarbonyl)amino)-3-(4-(difluoromethoxy)-3-methoxyphenyl)acrylate **S39-I1** (900 mg, 2.41 mmol, 1.0 equiv) in 10 mL MeOH. The RBF was purged and degassed with argon for three times. Pd/C 10% wt. (20 mg) was transferred to RBF. Argon was replaced with hydrogen, and the RBF was purged and degassed with hydrogen for three times. The reaction mixture was stirred under hydrogen atmosphere overnight. Next morning, the reaction mixture was diluted with MeOH and passed through celite and MeOH was evaporated under reduced pressure, and the crude was subsequently purified by column chromatography.

Purification: Isocratic column chromatography [SiO_2, EtOAc:Hexanes 20:80) to obtain the titled compound **S39** as white solid (458 mg, 51%).

R_f : 0.5 (EtOAc : Hexanes 1:9)

^{1}H NMR ($CDCl_3$, 400 MHz): δ 7.06 (d, J = 8.1 Hz, 1H), 6.7 (d, J = 1.9 Hz, 1H), 6.68 (d, J = 8.1 Hz, 1H), 6.60 (t, J = 75.5 Hz, 1H), 4.99 (d, J = 7.7 Hz, 1H), 4.60 (q, J = 6.2 Hz, 1H), 3.85 (s, 3H), 3.72 (s, 3H), 3.11 (dd, J = 13.9, 5.8 Hz, 1H), 3.01 (dd, J = 13.8, 6.1 Hz, 1H), 1.41 (s, 9H).

^{13}C NMR ($CDCl_3$, 101 MHz): δ 172.28, 155.14, 151.08, 139.12, 135.03, 122.39, 121.83, 116.31 (t, J = 259.6 Hz), 113.77, 80.21, 56.06, 54.43, 52.46, 38.31, 28.42.

^{19}F NMR ($CDCl_3$, 376 MHz): δ –81.40 (d, J =74.9 Hz).

HRMS (ESI-TOF) m/z: $[M + Na]^+$ Calcd. for $C_{17}H_{23}F_2NNaO_6$ 398.1391; found 398.1388.

Deoxyfluorination and Authentic fluoroarene standard

Methyl 2-((*tert*-butoxycarbonyl)amino)-3-(4-fluoro-3-methoxyphenyl)propanoate ([^{19}F]39)

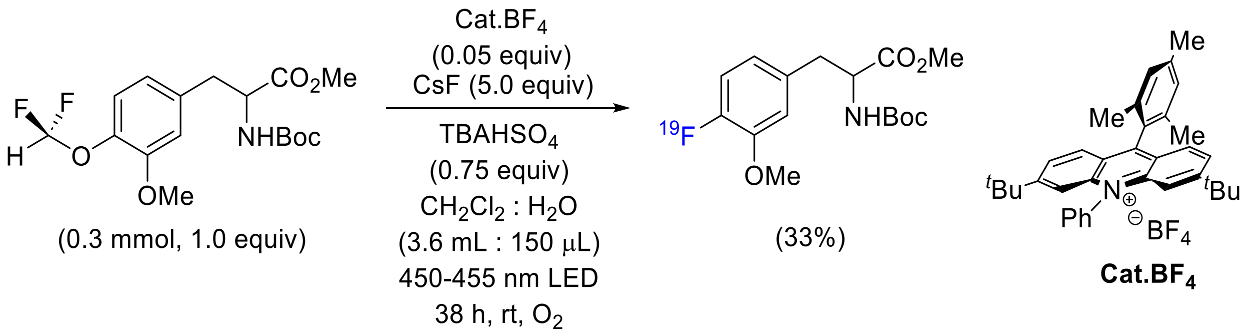

General conditions **I** were followed using methyl 2-((*tert*-butoxycarbonyl)amino)-3-(4-(difluoromethoxy)-3-methoxyphenyl)propanoate **S39** (75 mg, 3 mmol, 1.0 equiv), Mes-Acr-$Ph^+BF_4^-$ (0.05 equiv), CsF (5.0 equiv), $TBAHSO_4$ (0.75 equiv), CH_2Cl_2 : H_2O (0.1 M, 25:1), 450-455 nm LED, 38 h, 33 °C, O_2.

Purification: Gradient column chromatography [SiO_2, EtOAc:Hexanes 5:95 to 20:80)] to obtain the titled compound **[^{19}F]39** as off-white solid (32 mg, 33%).

R_f : 0.6 (EtOAc : Hexanes 3:7)

^{1}H NMR ($CDCl_3$, 400 MHz): δ 6.97 (dd, J = 11.2, 8.2 Hz, 1H), 6.72 (dd, J = 8.2, 1.9 Hz, 1H), 6.64-6.61 (m, 1H), 4.98 (d, J = 7.4 Hz, 1H), 4.55 (d, J = 6.4 Hz, 1H), 3.86 (s, 3H), 3.71 (s, 3H), 3.08 (dd, J = 13.8, 5.8 Hz, 1H), 2.99 (dd, J = 13.9, 5.8 Hz, 1H), 1.41 (s, 9H).

^{13}C NMR ($CDCl_3$, 101 MHz): δ 172.36, 155.17, 153.01, 150.58, 147.63 (d, J = 10.9 Hz), 132.49 (d, J = 4.3 Hz), 121.66 (d, J = 6.3 Hz), 116.09 (d, J = 18.2 Hz), 114.56 (d, J = 2.2 Hz), 80.21, 56.34, 54.46, 52.45, 38.21, 28.46.

^{19}F NMR ($CDCl_3$, 376 MHz): δ –137.82 (ddd, J = 12.5, 8.3, 4.2 Hz).

HRMS (ESI-TOF) m/z: $[M + H]^+$ Calcd. for $C_{10}H_{13}FNO_3$ 328.1560; found 328.1555, and $[M + Na]^+$ Calcd. for $C_{16}H_{22}FNNaO_5$ 350.1380; found 350.1374.

2-Amino-3-(4-(fluoro)-3-methoxyphenyl)propanoic acid ([^{19}F]42)

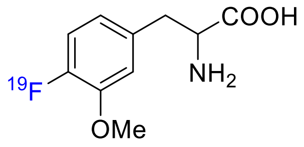

HRMS (ESI-TOF) m/z: $[M + H]^+$ Calcd. for $C_{10}H_{13}FNO_3$ 214.0879; found 214.0875.

^{19}F-Reference compound – **[^{19}F]42**, data are comparable to that reported in the literature.[10]

Radio-HPLC analysis and characterization

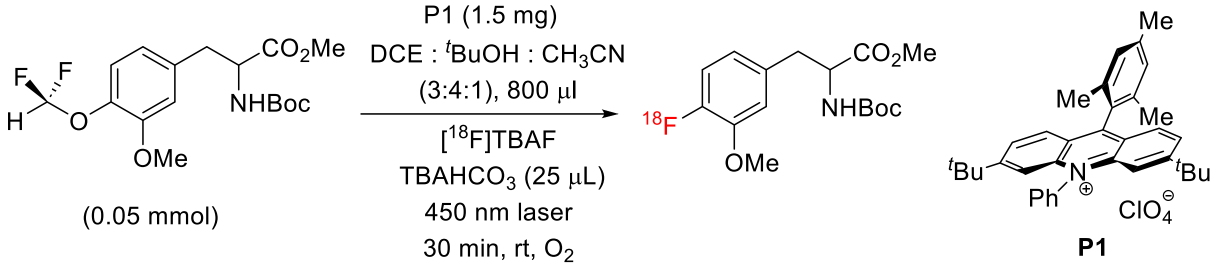

Entry	Activity at starting	Activity at EOS	Alumina Filtration	Injected Dose	Collected Dose	Isolation Time	Decay Corrected	Filtration Yield	HPLC Purity of Filtrate	%Yield
1	16.17 mCi	12.81 mCi	6.68 mCi	464 μCi	198 μCi	15.0 min	424.33 μCi	52.14%	46.66%	24.32%
2	11.4 mCi	8.92 mCi	4.57 mCi	1140 μCi	292 μCi	15.0 min	1036.97 μCi	51.23%	28.15%	14.42%
										**Average %Yield: 19.4% (n=2)**

Table S47: HPLC isolated RCYs for **[^{18}F]39**

HPLC Conditions – method 1: Column: Phenomenex, Kinetex® 5μm F5 100 Å, 250 ✕ 4.6 mm LC Column

Solvent A: 0.1% TFA water, Solvent B: 0.1% TFA acetonitrile; Isocratic / Gradient elution: 20% Solvent B for 0 to 2 min, 20% – 95% Solvent B for 2 to 22.5 min. Flow rate: 1 mL/min

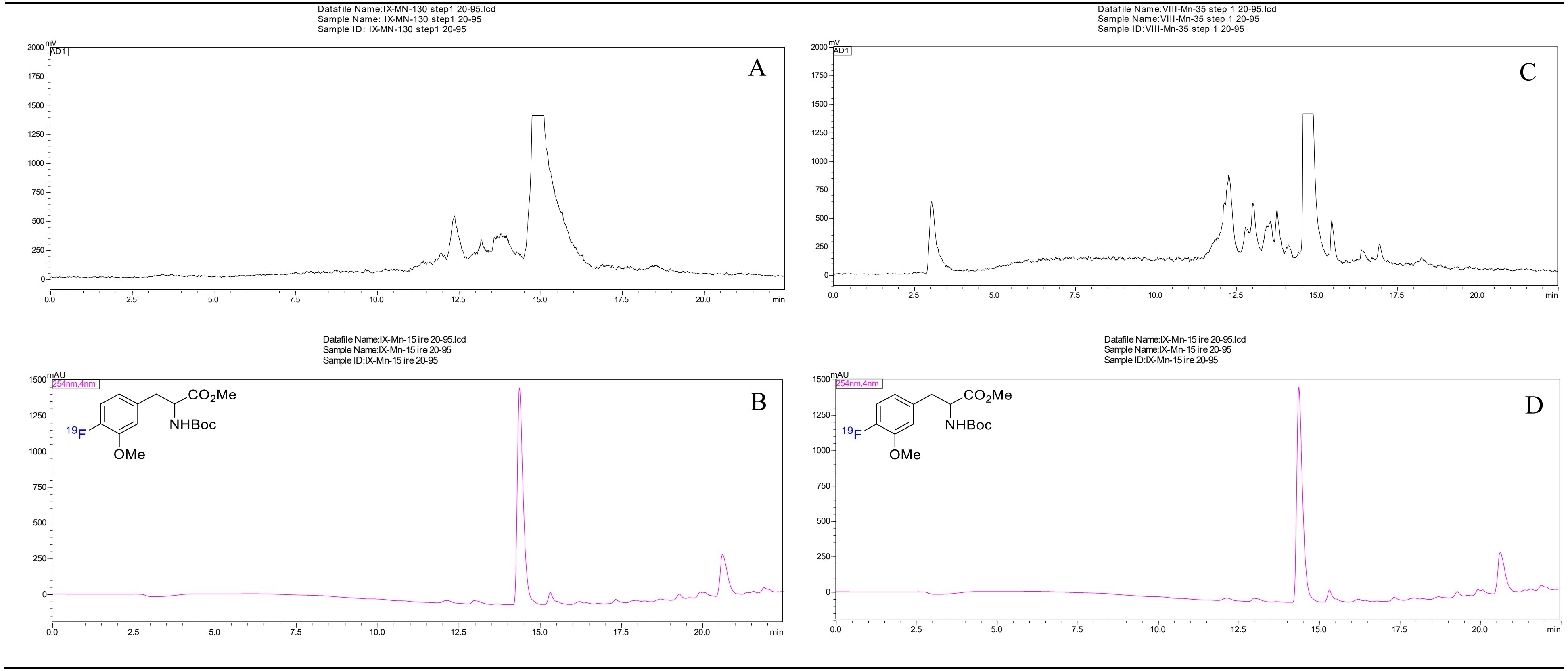

Datafile Name:IX-MN-130 step1 20-95.lcd
Sample Name: IX-MN-130 step1 20-95
Sample ID: IX-MN-130 step1 20-95
A
Datafile Name:VIII-Mn-35 step 1 20-95.lcd
Sample Name:VIII-Mn-35 step 1 20-95
Sample ID:VIII-Mn-35 step 1 20-95
C
Datafile Name:IX-Mn-15 ire 20-95.lcd
Sample Name:IX-Mn-15 ire 20-95
Sample ID:IX-Mn-15 ire 20-95
B
Datafile Name:IX-Mn-15 ire 20-95.lcd
Sample Name:IX-Mn-15 ire 20-95
Sample ID:IX-Mn-15 ire 20-95
D

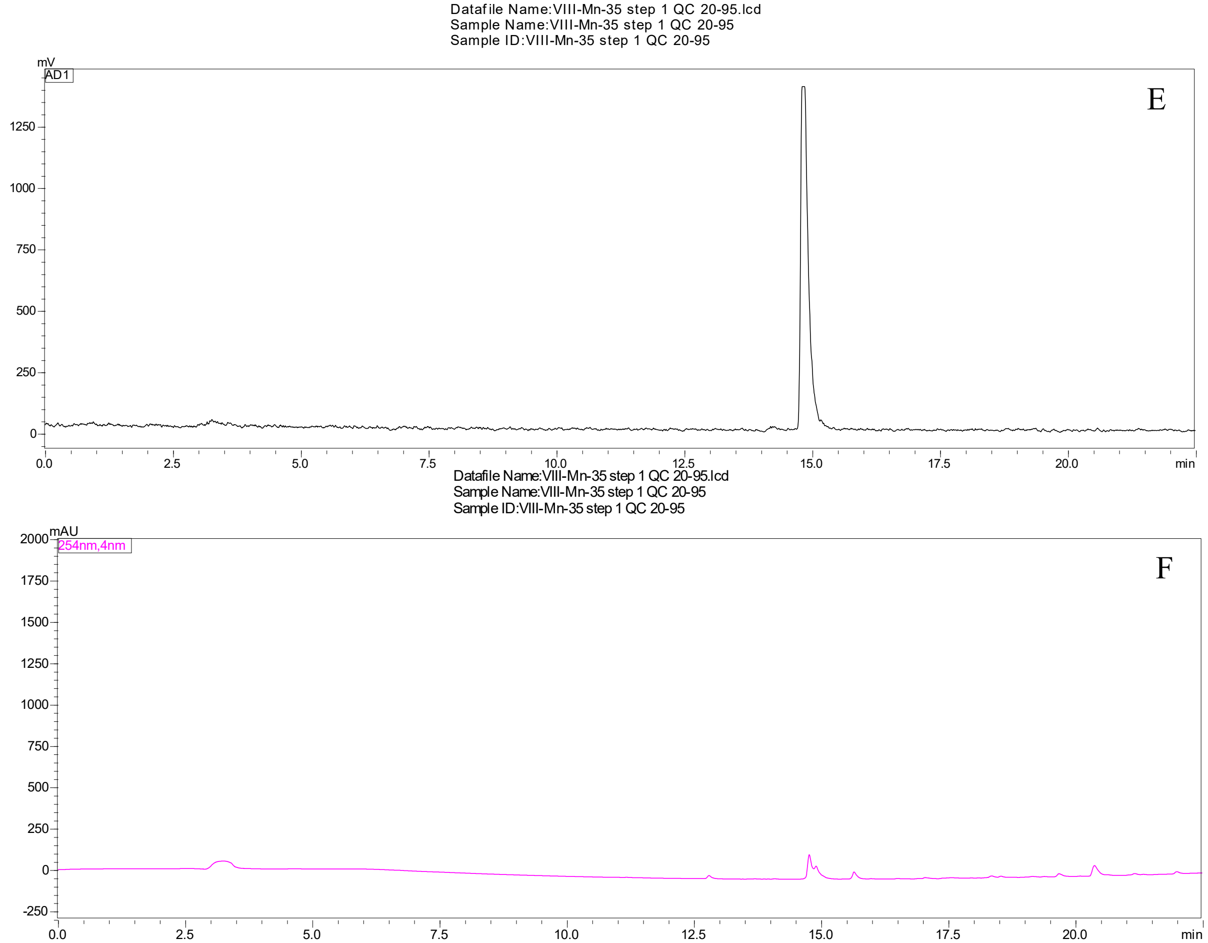

Figure S49: Radio-HPLC analysis of reaction mixture from **S39**. Reaction mixture with HPLC method 1 (A), authentic **[^{19}F]39** with HPLC method 1, reaction mixture with HPLC method 1 (C), authentic **[^{19}F]39** with HPLC method 1 (D), and QC for **[^{18}F]39** (E and F).

HPLC Conditions for QC: Column: Phenomenex, Kinetex® 5µm F5 100 Å, 250 × 4.6 mm LC Column

Solvent A: 0.1% TFA water, Solvent B: 0.1% TFA acetonitrile; Isocratic / Gradient elution: 20% Solvent B for 0 to 2 min, 20% – 95% Solvent B for 2 to 22.5 min. Flow rate: 1 mL/min.

One-pot experiment

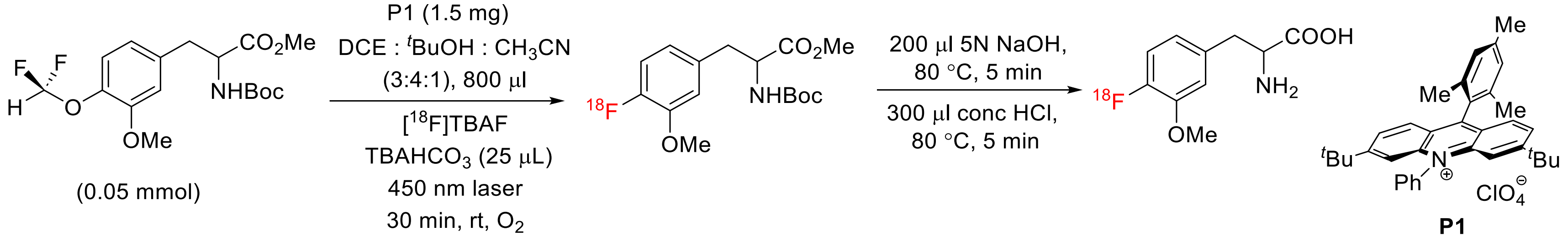

Entry	Activity at starting	Activity at EOS	Alumina Filtration	Injected Dose	Collected Dose	Isolation time	Decay Corrected	Filtration yield	HPLC Purity of Filtrate	%Yield
1*	17.16 mCi	13.08 mCi	6.23 mCi	513 µCi	252 µCi	10.8-11.3 min	478.43 µCi	47.62%	52.67%	25.08%
2**	11.4 mCi	8.92 mCi	4.57 mCi	398 µCi	89 µCi	13.2 min	361.57 µCi	51.23%	24.53%	12.56%
										**Average %Yield: 18.8% (n=2)**

Supplementary **Table S48**: HPLC isolated RCYs for **[^{18}F]42**

*HPLC Conditions – method 1: Column: Phenomenex, Kinetex® 5µm F5 100 Å, 250 × 4.6 mm LC Column

Solvent A: 0.1% TFA water, Solvent B: 0.1% TFA acetonitrile; Gradient elution: 0% - 5% solvent B for 5 min then 5% - 95% solvent B for 5 min - 22.5 min, Flow rate: 1 ml/min

**HPLC Conditions – method 2: Column: Phenomenex, Kinetex® 5µm F5 100 Å, 250 × 4.6 mm LC Column

Solvent A: 0.1% TFA water, Solvent B: 0.1% TFA acetonitrile; Gradient elution: 0% - 5% solvent B for 11 min then 5% - 95% solvent B for 11 min - 22.5 min, Flow rate: 1 ml/min

Datafile Name:VIII-Mn-39 step2 0-5-95 (0-5 5 min, 5-95 22 min).lcd
Sample Name:VIII-Mn-39 step2 0-5-95 (0-5 5 min, 5-95 22 min)
Sample ID:VIII-Mn-39 step2 0-5-95 (0-5 5

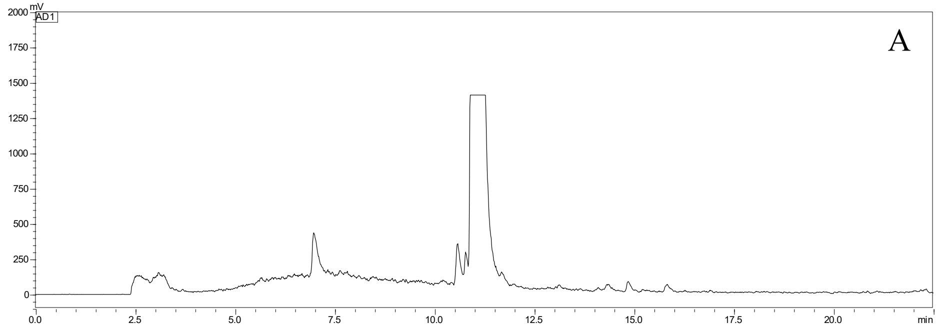

Datafile Name:OMe Tyrosine ref 0-5-95 (0-5 5 min, 5-95 22 min).lcd
Sample Name:OMe Tyrosine ref 0-5-95 (0-5 5 min, 5-95 22 min)
Sample ID:OMe Tyrosine ref 0-5-95 (0-5 5

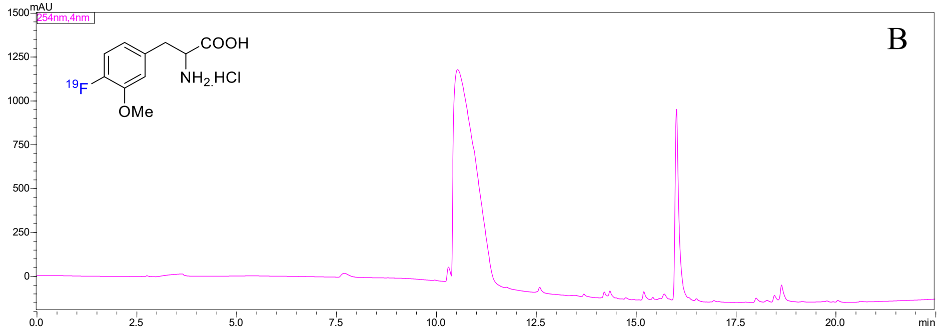

Datafile Name:VIII-Mn-35 step 2 0-5-95.lcd
Sample Name:VIII-Mn-35 step 2 0-5-95
Sample ID:VIII-Mn-35 step 2 0-5-95

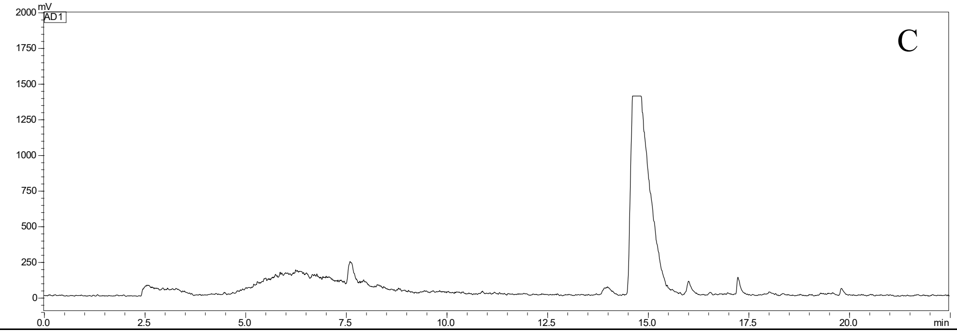

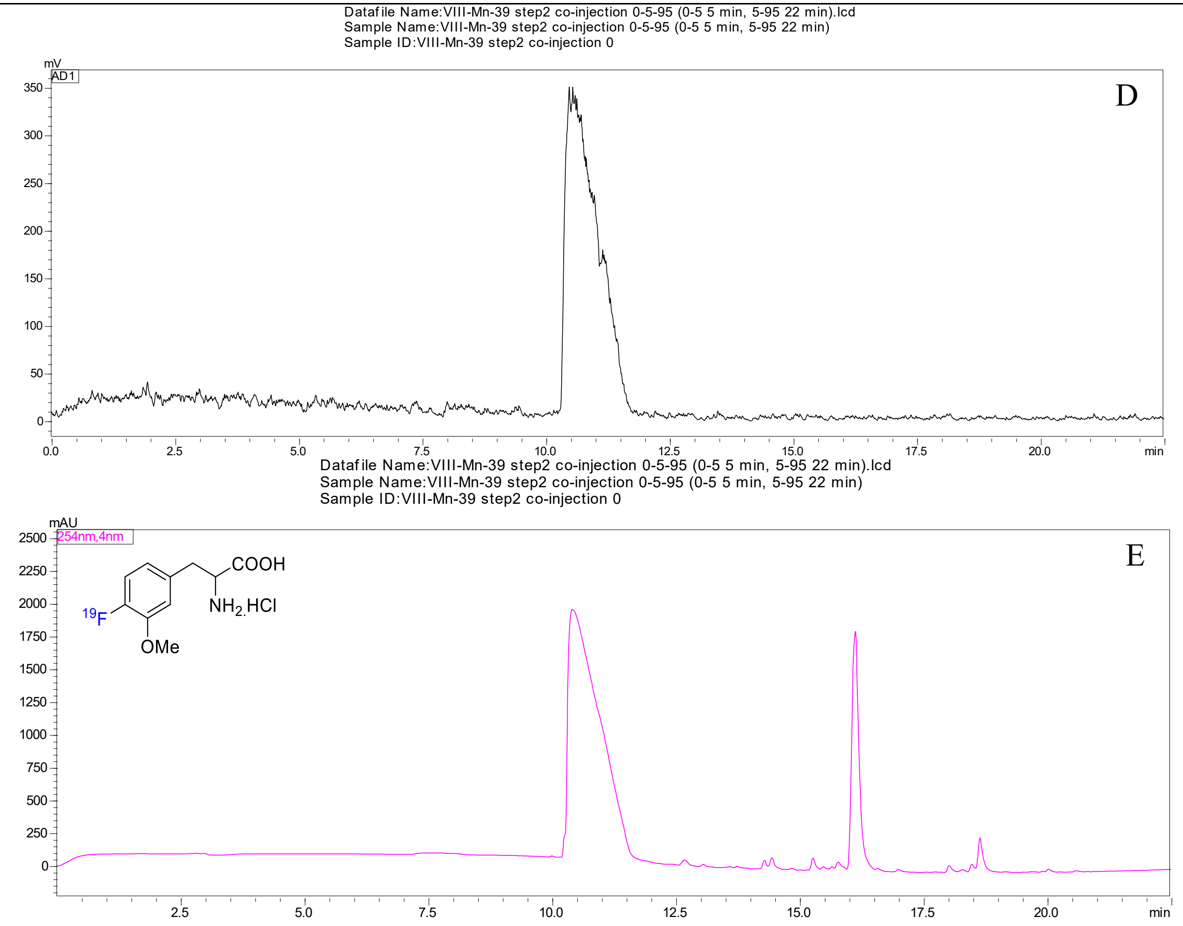

Figure S50: Radio-HPLC analysis of reaction mixture from **S42**. Reaction mixture with HPLC method 1 (A), authentic **[^{19}F]39** with HPLC method 1, reaction mixture with HPLC method 2 (C), HPLC trace of the co-injection for **[^{18}F]42 and [^{19}F]42** with HPLC method 1 (D and E).

HPLC Conditions: Column: Phenomenex, Kinetex® 5µm F5 100 Å, 250 ✕ 4.6 mm LC Column

Solvent A: 0.1% TFA water, Solvent B: 0.1% TFA acetonitrile; Gradient elution: 0% - 5% solvent B for 5 min then 5% - 95% solvent B for 5 min - 22.5 min, Flow rate: 1 ml/min

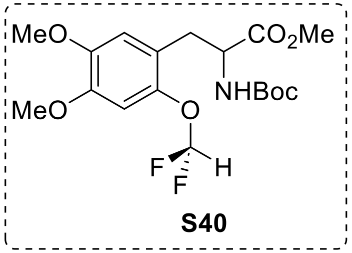

Arene substrate:

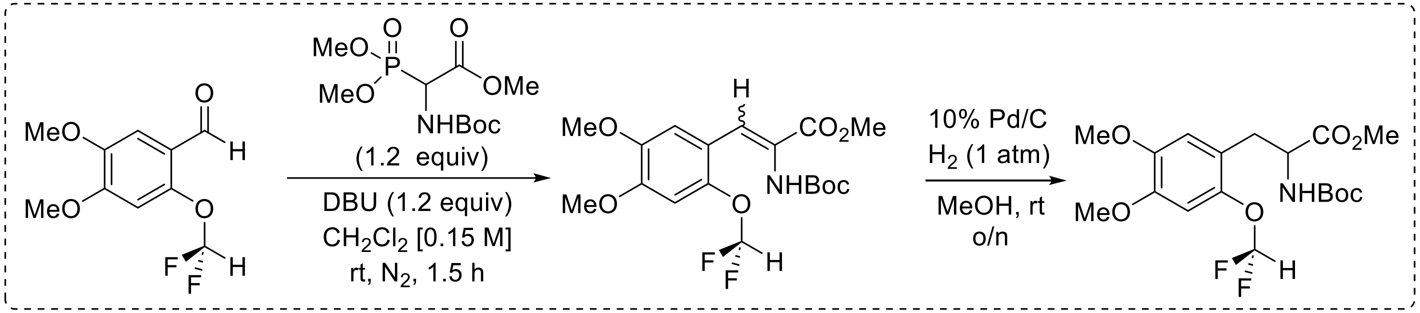

Methyl 2-((*tert*-butoxycarbonyl)amino)-3-(2-(difluoromethoxy)-4,5-dimethoxyphenyl)acrylate (S40-I1)

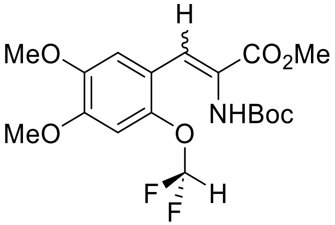

A 4 mL scintillation vial was charged with 2-(difluoromethoxy)-4,5-dimethoxybenzaldehyde (120 mg, 0.52 mmol, 1.0 equiv), methyl 2-((*tert*-butoxycarbonyl)amino)-2-(dimethoxyphosphoryl)acetate (185 mg, 0.6226 mmol, 1.0 equiv). The screw-capped vial was purged and degassed with argon for three times. Anhydrous CH_2Cl_2 (3.5 mL, [0.15 M]) followed by DBU (92.53 µL, 0.62 mmol, 1.2 equiv) were transferred by syringe and the reaction mixture was stirred at room temperature for 1.5 h. The reaction mixture was diluted with CH_2Cl_2 and H_2O. The

aqueous layer was extracted with CH_2Cl_2 (3 × 5 mL). The combined organics dried (Na_2SO_4) and concentrated under reduced pressure, which was subsequently purified by column chromatography.

Purification: Gradient column chromatography [SiO_2, EtOAc:Hexanes 5:95 to 30:70)] to obtain the titled compound **S40-I1** as white solid (78 mg, 37%).

R_f : 0.5 (EtOAc : Hexanes 3:7)

^{1}H NMR ($CDCl_3$, 400 MHz): δ 7.38 (s, 1H), 7.21 (s, 1H), 6.71 (s, 1H), 6.46 (t, J = 74.5 Hz, 1H), 3.89 (s, 3H), 3.85 (d, J = 8.7 Hz, 6H), 1.37 (s, 9H).

^{13}C NMR ($CDCl_3$, 101 MHz): δ 165.99, 150.46, 146.73, 143.46, 123.12, 119.52 (t, J = 280.7 Hz), 110.86, 105.18, 81.10, 56.21 (d, J = 10.9 Hz), 52.86, 28.24.

^{19}F NMR ($CDCl_3$, 376 MHz): δ –80.29 (d, J = 74.2 Hz).

HRMS (ESI-TOF) m/z: $[M + H]^+$ Calcd. for $C_{18}H_{24}F_2NO_7$ 404.1521; found 404.1516, and $[M + Na]^+$ Calcd. for $C_{18}H_{23}F_2NNaO_7$ 426.1340; found 426.1334.

Methyl 2-((*tert*-butoxycarbonyl)amino)-3-(2-(difluoromethoxy)-4,5-dimethoxyphenyl)propanoate (S40)

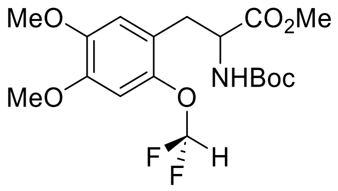

A 25 mL RBF was charged with methyl 2-((*tert*-butoxycarbonyl)amino)-3-(2-(difluoromethoxy)-4,5-dimethoxyphenyl)acrylate **S40-I1** (70 mg, 0.17 mmol, 1.0 equiv) in 2 mL MeOH. The RBF was purged and degassed with argon for three times. Pd/C 10% wt. (10 mg) was transferred to RBF. Argon was replaced with hydrogen, and the RBF was purged and degassed with hydrogen for three times. The reaction mixture was

stirred under hydrogen atmosphere overnight. Next morning, the reaction mixture was diluted with MeOH and passed through celite and MeOH was evaporated under reduced pressure, and the crude was subsequently purified by column chromatography.

Purification: Gradient column chromatography [SiO_2, EtOAc:Hexanes 5:95 to 40:60)] to obtain the titled compound **S40** as off-grey solid (20 mg, 29%).

R_f : 0.4 (EtOAc : Hexanes 3:7)

^{1}H NMR ($CDCl_3$, 400 MHz): δ 6.66 (s, 2H), 6.43 (t, J = 74.4 Hz, 1H), 5.04 (d, J = 8.0 Hz, 1H), 4.53 (q, J = 7.0 Hz, 1H), 3.85 (s, 6H), 3.73 (s, 3H), 3.12 (dd, J = 13.9, 5.7 Hz, 1H), 2.97 (dd, J = 13.9, 7.2 Hz, 1H), 1.37 (s, 9H).

^{13}C NMR ($CDCl_3$, 101 MHz): δ 172.49, 155.13, 148.69, 146.75, 143.19, 119.94, 116.76 (t, J = 259.6 Hz), 113.69, 104.65, 80.00, 56.28 (d, J = 6.4 Hz), 53.79, 52.49, 32.68, 28.38.

^{19}F NMR ($CDCl_3$, 376 MHz): δ –79.48 (dd J =150.5, 75.9 Hz).

HRMS (ESI-TOF) m/z: $[M + H]^+$ Calcd. for $C_{18}H_{26}F_2NO_7$ 406.1667; found 404.1673, and $[M + Na]^+$ Calcd. for $C_{18}H_{25}F_2NNaO_7$ 428.1497; found 428.1494.

Authentic fluoroarene standard

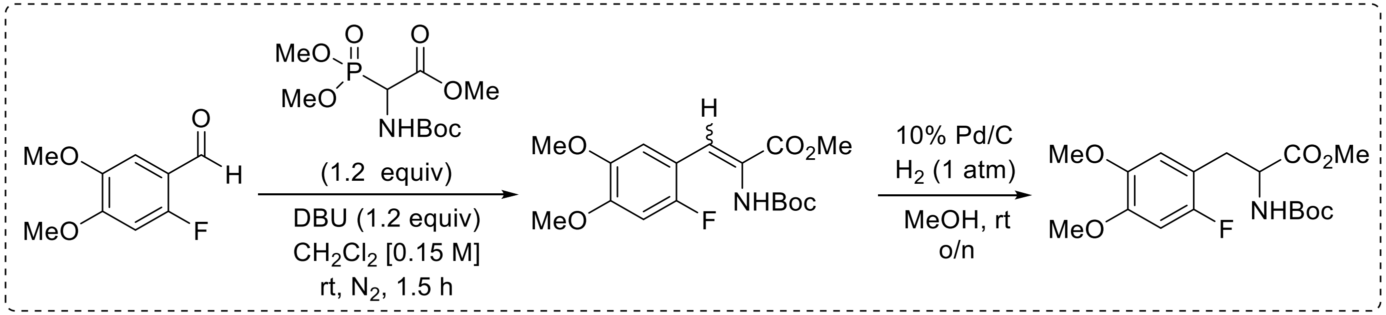

Methyl 2-((*tert*-butoxycarbonyl)amino)-3-(2-fluoro-4,5-dimethoxyphenyl)acrylate ([^{19}F]40-i)

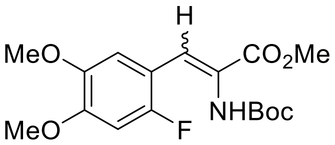

A 4 mL scintillation vial was charged with 2-fluoro-4,5-dimethoxybenzaldehyde (184 mg, 1.0 mmol, 1.0 equiv), methyl 2-((tert-butoxycarbonyl)amino)-2-(dimethoxyphosphoryl)acetate (357 mg, 1.2 mmol, 1.0 equiv). The screw-capped vial was purged and degassed with argon for three times. Anhydrous CH_2Cl_2 (10 mL, [0.1 M]) followed by DBU (180 µL, 0.62 mmol, 1.2 equiv) were transferred by syringe and the reaction mixture was stirred at room for 1.5 h. The reaction mixture diluted with CH_2Cl_2 and H_2O. The aqueous layer was extracted with CH_2Cl_2 (3 × 5 mL). The combined organics dried (Na_2SO_4) and concentrated under reduced pressure, which was subsequently purified by column chromatography.

Purification: Gradient column chromatography [SiO_2, EtOAc:Hexanes 5:95 to 40:60)] to obtain the titled compound **[^{19}F]40-i** as white solid (263 mg, 74%).

R_f : 0.4 (EtOAc : Hexanes 3:7)

^{1}H NMR ($CDCl_3$, 400 MHz): δ 7.39 (s, 1H), 7.20 (d, J = 6.8 Hz, 1H), 6.62 (d, J = 11.5 Hz, 1H), 3.88 (s, 3H), 3.84 (d, J = 8.9 Hz, 6H), 1.39 (s, 9H).

^{13}C NMR ($CDCl_3$, 101 MHz): δ 166.06, 157.15, 154.69, 152.91, 151.16 (d, J = 10.2 Hz), 145.15, 122.27 (d, J = 4.2 Hz), 113.31 (d, J = 14.2 Hz), 110.88 (d, J = 3.8 Hz), 99.77 (d, J = 28.8 Hz), 81.07, 56.30 (d, J = 4.3 Hz), 52.77, 28.25.

^{19}F NMR ($CDCl_3$, 376 MHz): δ –118.83.

HRMS (ESI-TOF) m/z: $[M + H]^+$ Calcd. for $C_{17}H_{23}FNO_6$ 356.1509; found 356.1505, and $[M + Na]^+$ Calcd. for $C_{17}H_{22}FNNaO_6$ 378.1329; found 378.1325.

Methyl 2-((*tert*-butoxycarbonyl)amino)-3-(2-fluoro-4,5-dimethoxyphenyl)propanoate ([^{19}F]41)

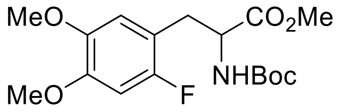

A 25 mL RBF was charged with methyl 2-((*tert*-butoxycarbonyl)amino)-3-(2-fluoro-4,5-dimethoxyphenyl)acrylate **[^{19}F]40-i** (184 mg, 1.0 mmol, 1.0 equiv) in 3 mL MeOH. The RBF was purged and degassed with argon for three times. Pd/C 10% wt. (10 mg) was transferred to RBF. Argon was replaced with hydrogen, and the RBF was purged and degassed with hydrogen for three times. The reaction mixture was stirred under hydrogen atmosphere overnight. Next morning, the reaction mixture was diluted with MeOH and passed through celite and MeOH was evaporated under reduced pressure, and the crude was subsequently purified by column chromatography.

Purification: Isocratic column chromatography [SiO_2, EtOAc:Hexanes 30:70) to obtain the titled compound **[^{19}F]40** as white solid (63 mg, 37%).

R_f : 0.5 (EtOAc : Hexanes 3:7)

^{1}H NMR ($CDCl_3$, 400 MHz): δ 6.61 (d, J = 8.0 Hz, 1H), 6.58 (d, J = 2.0 Hz, 1H), 5.05 (d, J = 7.9 Hz, 1H), 4.56 (q, J = 7.9Hz, 1H), 3.83 (d, J = 3.6 Hz, 6H), 3.72 (d, J = 1.9 Hz, 3H), 3.10 (dd, J = 14.0, 5.6 Hz, 1H), 3.02 (dd, J = 13.7, 5.9 Hz, 1H), 1.41 (s, 9H).

^{13}C NMR ($CDCl_3$, 101 MHz): δ 172.42, 156.77, 155.16, 154.39, 149.05 (d, J = 9.9 Hz), 145.25, 113.62 (d, J = 7.3 Hz), 113.43 (d, J = 17.5 Hz), 100.10 (d, J = 28.3 Hz), 80.06, 56.38 (d, J = 29.1 Hz), 53.89, 52.51, 31.62, 28.47.

^{19}F NMR ($CDCl_3$, 376 MHz): δ –124.99 (ddd, J =11.1, 6.9, 1.6 Hz).

HRMS (ESI-TOF) m/z: $[M + H]^+$ Calcd. for $C_{17}H_{25}FNO_6$ 358.1666; found 358.1661, and $[M + Na]^+$ Calcd. for $C_{17}H_{24}FNNaO_6$ 380.1485; found 380.1481.

Radio-HPLC analysis and characterization

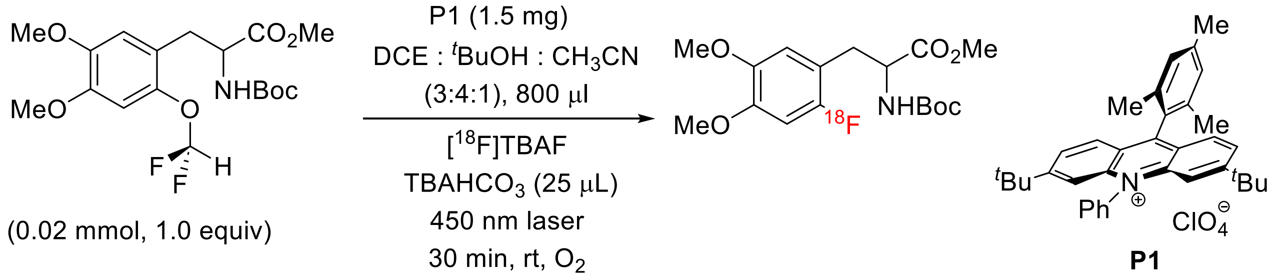

Entry	Activity at starting	Activity at EOS	Alumina Filtration	Injected Dose	Collected Dose	Isolation time	Decay Corrected	Filtratio n yield	HPLC Purity of Filtrate	%Yield
1	6.03 mCi	4.22 mCi	781 µCi	239 µCi	41 µCi	13.8 min	219.97 µCi	18.50%	18.63%	3.45%
2	21.5 mCi	16.50 mCi	4.55 mCi	644 µCi	34 µCi	13.8 min	592.99 µCi	27.57%	5.73%	1.58%
										**Average %Yield: 2.5% (n=2)**

Table S49: HPLC isolated RCYs for **[^{18}F]40**

HPLC Conditions – method 1: Column: Phenomenex, Kinetex® 5µm F5 100 Å, 250 ✕ 4.6 mm LC Column

Solvent A: 0.1% TFA water, Solvent B: 0.1% TFA acetonitrile; Isocratic / Gradient elution: 20% Solvent B for 0 to 2 min, 20% – 95% Solvent B for 2 to 22.5 min. Flow rate: 1 mL/min

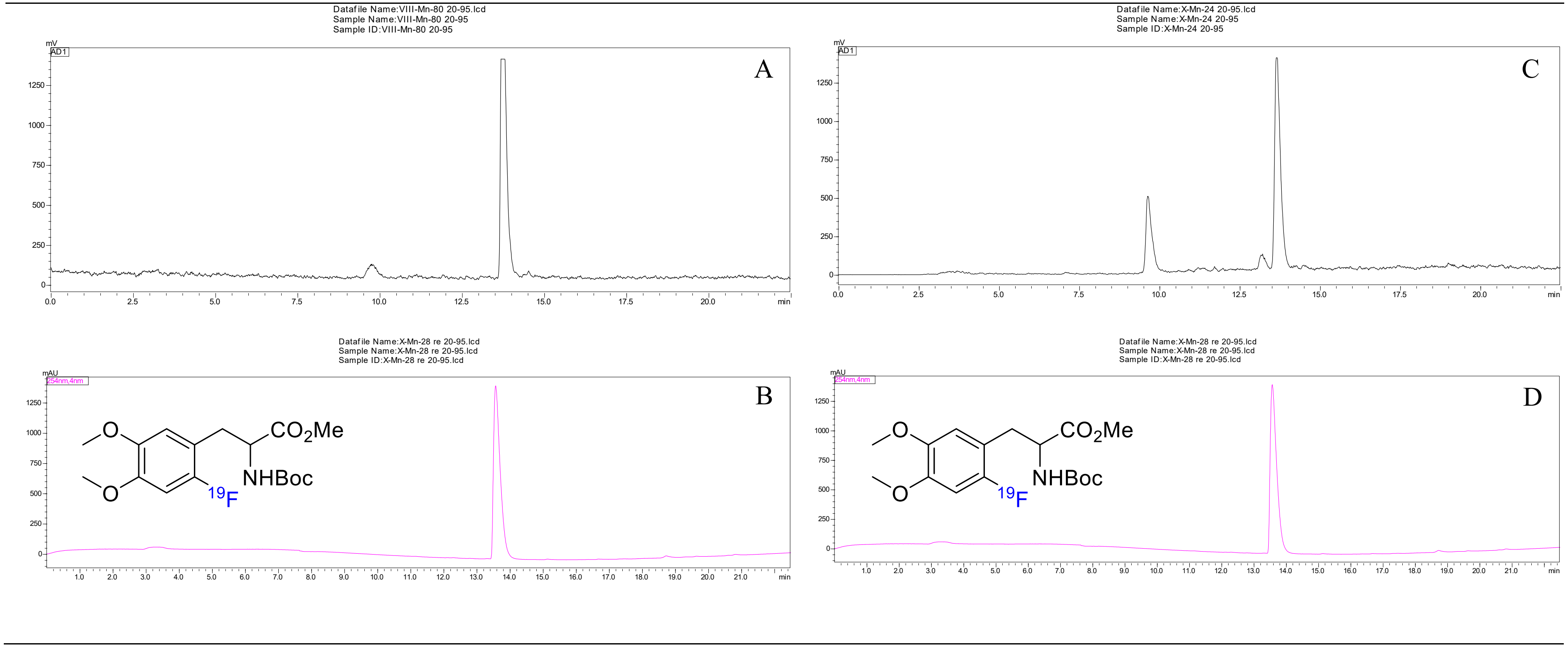
Datafile Name:VIII-Mn-80 20-95.lcd
Sample Name:VIII-Mn-80 20-95
Sample ID:VIII-Mn-80 20-95
A
Datafile Name:X-Mn-24 20-95.lcd
Sample Name:X-Mn-24 20-95
Sample ID:X-Mn-24 20-95
C
Datafile Name:X-Mn-28 re 20-95.lcd
Sample Name:X-Mn-28 re 20-95.lcd
Sample ID:X-Mn-28 re 20-95.lcd
B
CO2Me
NHBoc
19F
Datafile Name:X-Mn-28 re 20-95.lcd
Sample Name:X-Mn-28 re 20-95.lcd
Sample ID:X-Mn-28 re 20-95.lcd
D
CO2Me
NHBoc
19F

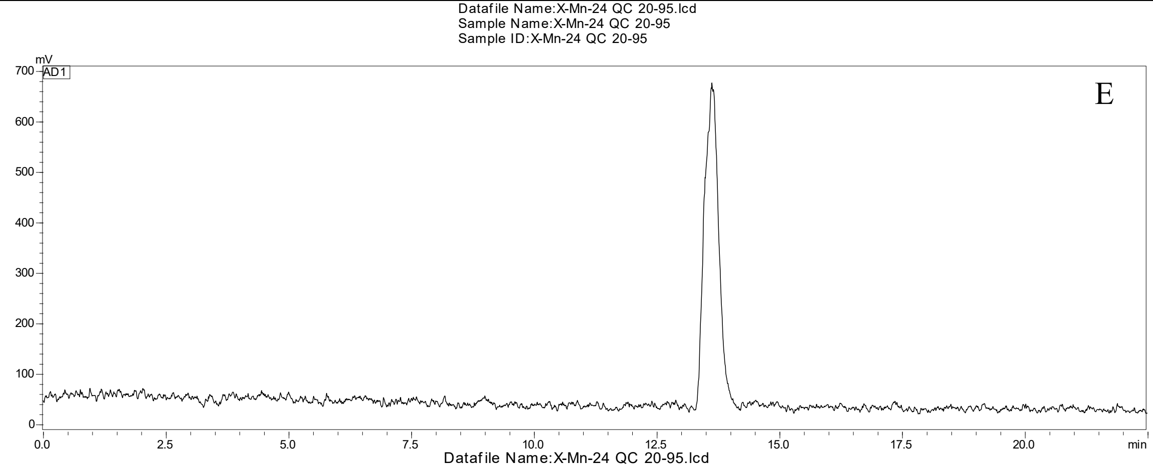

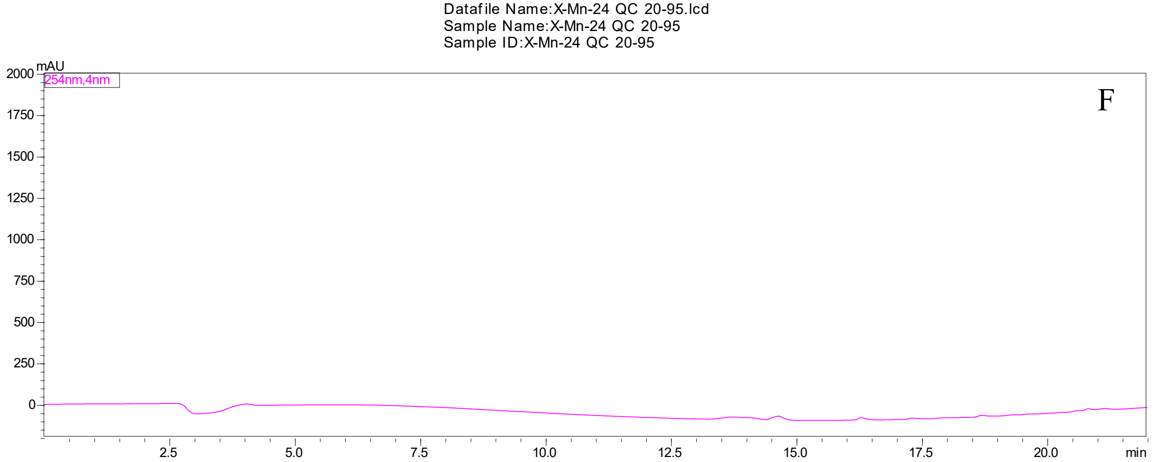

Figure S51: Radio-HPLC analysis of reaction mixture from **S40**. Reaction mixture with HPLC method 1 (A), authentic **[^{19}F]40** with HPLC method 1 (B), reaction mixture with HPLC method 1 (C), authentic **[^{19}F]40** with HPLC method 1 (D), and QC for **[^{18}F]40** (E and F).

HPLC Conditions for QC: Column: Phenomenex, Kinetex® 5µm F5 100 Å, 250 × 4.6 mm LC Column

Solvent A: 0.1% TFA water, Solvent B: 0.1% TFA acetonitrile; Isocratic / Gradient elution: 20% Solvent B for 0 to 2 min, 20% – 95% Solvent B for 2 to 22.5 min. Flow rate: 1 mL/min.

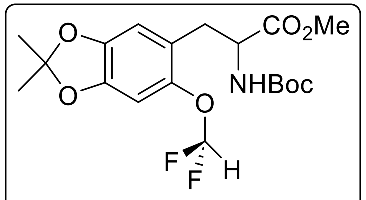

S41

Arene substrate:

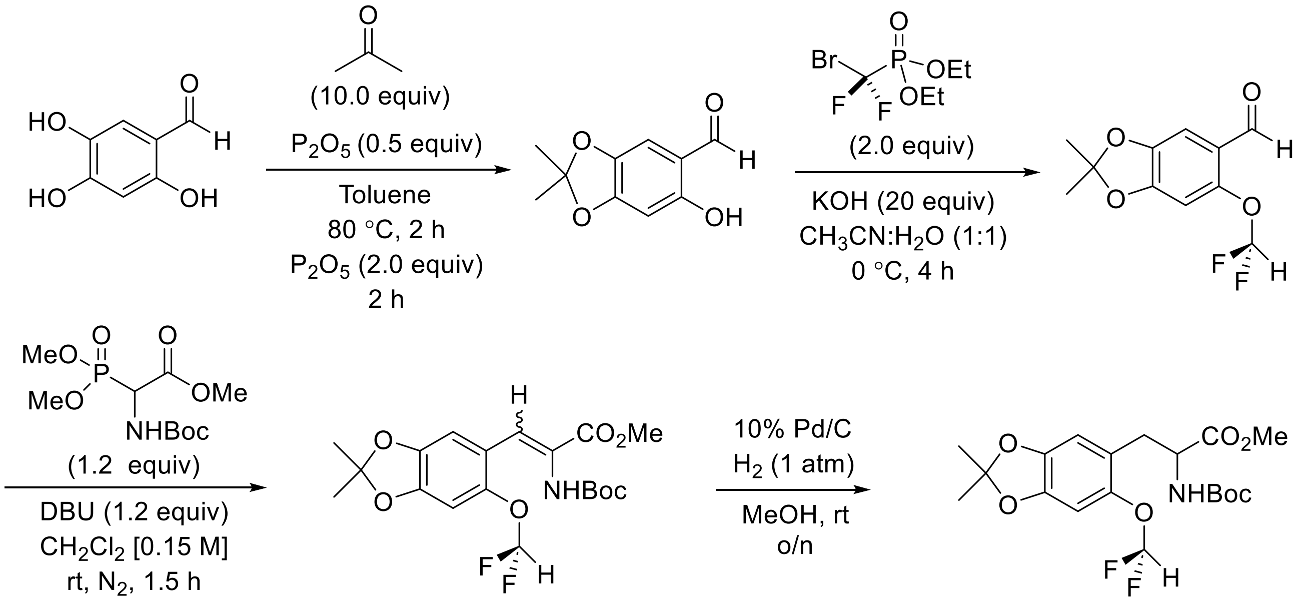

6-Hydroxy-2,2-dimethylbenzo[*d*][1,3]dioxole-5-carbaldehyde (S41-I1)

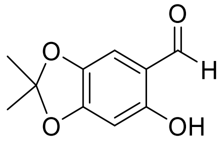

A 100 mL two-neck RBF was charged with 2,4,5-trihydroxybenzaldehyde (616 mg, 4.0 mmol, 1.0 equiv) and P_2O_5 (284 mg, 2.0 mmol, 0.5 equiv). The RBF was purged and degassed with argon for three times. Anhydrous toluene (10 mL, [0.2 M]) followed by acetone (5 mL, [0.4 M]) were transferred by syringe and the reaction mixture was stirred at 80 °C for 2 h. After 2 h, four portions of P_2O_5 (200 mg) were transferred to the RBF every 30 minutes during heating. After cooling, the reaction mixture was quenched with 25% NaOH and diluted with EtOAc and H_2O. The aqueous layer was extracted with EtOAc (3 ×10 mL). The combined organics dried (Na_2SO_4) and concentrated under reduced pressure, which was subsequently purified by column chromatography.

Purification: Gradient column chromatography [SiO_2, EtOAc:Hexanes 10:90 isocratic elution) to obtain the titled compound **S41-I1** as off-green solid (150 mg, 19%).

R_f : 0.5 (EtOAc : Hexanes 1:9)

^{1}H NMR ($CDCl_3$, 400 MHz): δ 11.71 (s, 1H), 9.59 (s, 1H), 6.75 (s, 1H), 6.37 (s, 1H), 1.69 (s, 6H).

^{13}C NMR ($CDCl_3$, 101 MHz): δ 193.69, 161.53, 155.31, 141.48, 120.13, 113.49, 109.24, 98.18, 25.98.

HRMS (ESI-TOF) m/z: $[M + H]^+$ Calcd. for $C_{10}H_{11}O_4$ 195.0657; found 195.0653.

6-(Difluoromethoxy)-2,2-dimethylbenzo[*d*][1,3]dioxole-5-carbaldehyde (S41-I2)

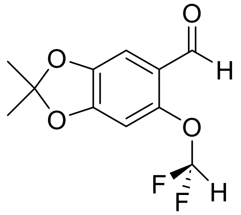

General conditions **A** were followed using 6-hydroxy-2,2-dimethylbenzo[d][1,3]dioxole-5-carbaldehyde **S41-I1** (150 mg, 0.77 mmol, 1.0 equiv), $BrCF_2P(O)(OC_2H_5)_2$ (409 mg, 1.53 mmol, 2.0 equiv), KOH (862 mg, 15.4 mmol, 20 equiv), CH_3CN: H_2O (4 mL : 4 mL) for 4 h. Purification: Isocratic column chromatography [SiO_2, EtOAc:Hexanes 2:98 isocratic elution) to obtain the titled compound **S41-I2** as off-green solid (52 mg, 28%).

R_f : 0.5 (EtOAc : Hexanes 0.5:9.5)

^{1}H NMR ($CDCl_3$, 400 MHz): δ 10.17 (s, 1H), 7.22 (s, 1H), 6.63 (s, 1H), 6.55 (t, J = 72.9 Hz, 1H), 1.71 (s, 6H).

^{13}C NMR ($CDCl_3$, 101 MHz): δ 187.14, 153.55, 149.58 (t, J = 2.7 Hz), 146.07, 122.49, 121.13, 115.92 (t, J = 263.3 Hz), 105.97, 102.14, 26.04.

^{19}F NMR ($CDCl_3$, 376 MHz): δ –81.39 (d, J = 72.8 Hz).

HRMS (ESI-TOF) m/z: $[M + H]^+$ Calcd. for $C_{11}H_{11}F_2O_4$ 245.0625; found 245.0621.

Methyl 2-((*tert*-butoxycarbonyl)amino)-3-(6-(difluoromethoxy)-2,2-dimethylbenzo[*d*][1,3]dioxol-5-yl)acrylate (S41-I3)

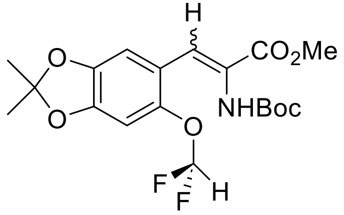

A 4 mL scintillation vial was charged with 6-(difluoromethoxy)-2,2-dimethylbenzo[d][1,3]dioxole-5-carbaldehyde **S41-I2** (52 mg, 0.21 mmol, 1.0 equiv), methyl 2-((*tert*-butoxycarbonyl)amino)-2-(dimethoxyphosphoryl)acetate (76 mg, 0.26 mmol, 1.0 equiv). The screw-capped vial was purged and degassed with argon for three times. Anhydrous CH_2Cl_2 (2.1 mL, [0.1 M]) followed by DBU (38.80 µL, 0.26 mmol, 1.2 equiv) were transferred by syringe and the reaction mixture was stirred at room for 1.5 h. The reaction mixture diluted with CH_2Cl_2 and H_2O. The aqueous layer was extracted with CH_2Cl_2 (3 ×5 mL). The combined organics dried (Na_2SO_4) and concentrated under reduced pressure, which was subsequently purified by column chromatography.

Purification: Gradient column chromatography [SiO_2, EtOAc:Hexanes 5:95 to 20:80)] to obtain the titled compound **S41-I3** as white solid (84 mg, 97%).

R_f : 0.3 (EtOAc : Hexanes 1:9)

^{1}H NMR ($CDCl_3$, 500 MHz): δ 7.34 (s, 1H), 7.05 (s, 1H), 6.61 (s, 1H), 6.43 (t, J= 73.9 Hz), 3.85 (s, 3H), 1.67 (s, 6H), 1.39 (s, 9H).

^{13}C NMR ($CDCl_3$, 126 MHz): δ 166.01, 148.80, 145.39, 144.08 (t, J= 4.6 Hz), 123.22, 120.09, 119.89, 116.73 (t, J= 260.8 Hz), 109.39, 102.76, 81.02, 52.79, 28.18, 25.96.

^{19}F NMR ($CDCl_3$, 470 MHz): δ –79.65 (dd, J = 273.6, 77.9 Hz).

HRMS (ESI-TOF) m/z: $[M + H]^+$ Calcd. for $C_{19}H_{24}F_2NO_7$ 416.1521; found 416.1516, and $[M + Na]^+$ Calcd. for $C_{19}H_{23}F_2NNaO_7$ 438.1340; found 438.1336.

Methyl 2-((*tert*-butoxycarbonyl)amino)-3-(6-(difluoromethoxy)-2,2-dimethylbenzo[*d*][1,3]dioxol-5-yl)propanoate (S41)

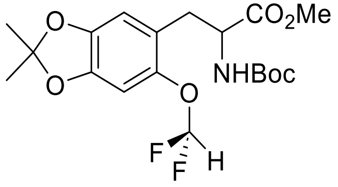

A 25 mL RBF was charged with methyl 2-((*tert*-butoxycarbonyl)amino)-3-(6-(difluoromethoxy)-2,2-dimethylbenzo[*d*][1,3]dioxol-5-yl)acrylate **S41-I3** (70 mg, 0.17 mmol, 1.0 equiv) in 2 mL MeOH. The RBF was purged and degassed with argon for three times. Pd/C 10% wt. (10 mg) was transferred to RBF. Argon was replaced with hydrogen, and the RBF was purged and degassed with hydrogen for three times. The reaction mixture was stirred under hydrogen atmosphere overnight. Next morning, the reaction mixture was diluted with MeOH and passed through celite and MeOH was evaporated under reduced pressure, and the crude was subsequently purified by column chromatography.

Purification: Gradient column chromatography [SiO_2, EtOAc:Hexanes 5:95 to 40:60)] to obtain the titled compound **S41** as colourless liquid (67 mg, 94%).

R_f : 0.4 (EtOAc : Hexanes 3:7)

^{1}H NMR ($CDCl_3$, 500 MHz): δ 6.56 (s, 1H), 6.53 (s, 1H), 6.39 (t, J = 74.2 Hz, 1H), 5.04 (d, J = 8.4 Hz, 1H), 4.48 (q, J = 7.6 Hz, 1H), 3.74 (s, 3H), 3.09 (dd, J = 13.9, 5.4 Hz, 1H), 2.89 (dd, J = 13.9, 7.5 Hz, 1H), 1.65 (d, J = 2.9 Hz, 6H), 1.39 (s, 9H).

^{13}C NMR ($CDCl_3$, 126 MHz): δ 172.54, 155.79, 147.15, 145.11, 143.54, 120.33, 119.35, 116.80 (t, J = 260.4 Hz), 110.17, 101.80, 79.96, 53.88, 52.49, 33.01, 28.38, 25.95.

^{19}F NMR ($CDCl_3$, 470 MHz): δ –80.64 (dd J =165.7, 74.7 Hz).

HRMS (ESI-TOF) m/z: $[M + Na]^+$ Calcd. for $C_{19}H_{25}F_2NNaO_7$ 440.1497; found 440.1496.

Radio-HPLC analysis and characterization

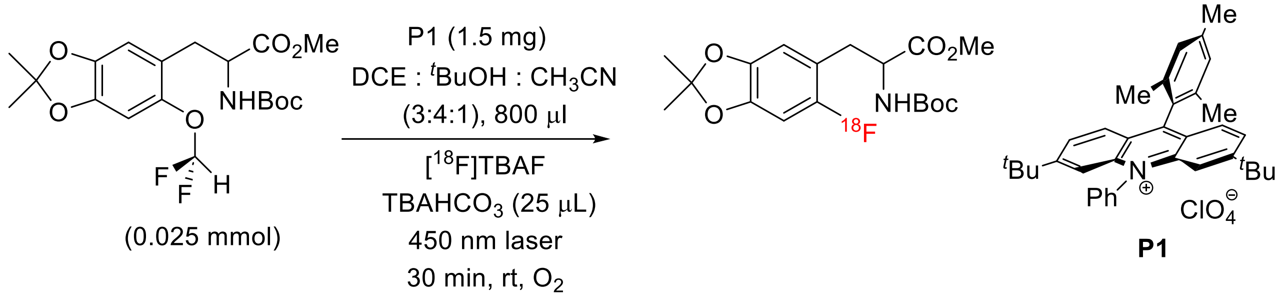

Entry	Activity at starting	Activity at EOS	Alumina Filtration	Injected Dose	Collected Dose	Isolation time	Decay Corrected	Filtration yield	HPLC Purity of Filtrate	%Yield
1	25.0 mCi	18.59 mCi	3.98 mCi	501 µCi	173 µCi	16.4 min	452.66 µCi	21.40%	38.21%	8.17%
2	18.98 mCi	14.05 mCi	4.30 mCi	544 µCi	144 µCi	16.4 min	491.52 µCi	30.60%	29.29%	8.96%
								**Average %Yield: 8.6% (n=2)**		

Supplementary **Table S50**: HPLC isolated RCYs for **[^{18}F]41**

HPLC Conditions – method 1: Column: Phenomenex, Kinetex® 5µm F5 100 Å, 250 ✕ 4.6 mm LC Column

Solvent A: 0.1% TFA water, Solvent B: 0.1% TFA acetonitrile; Isocratic / Gradient elution: 20% Solvent B for 0 to 2 min, 20% – 95% Solvent B for 2 to 22.5 min. Flow rate: 1 mL/min

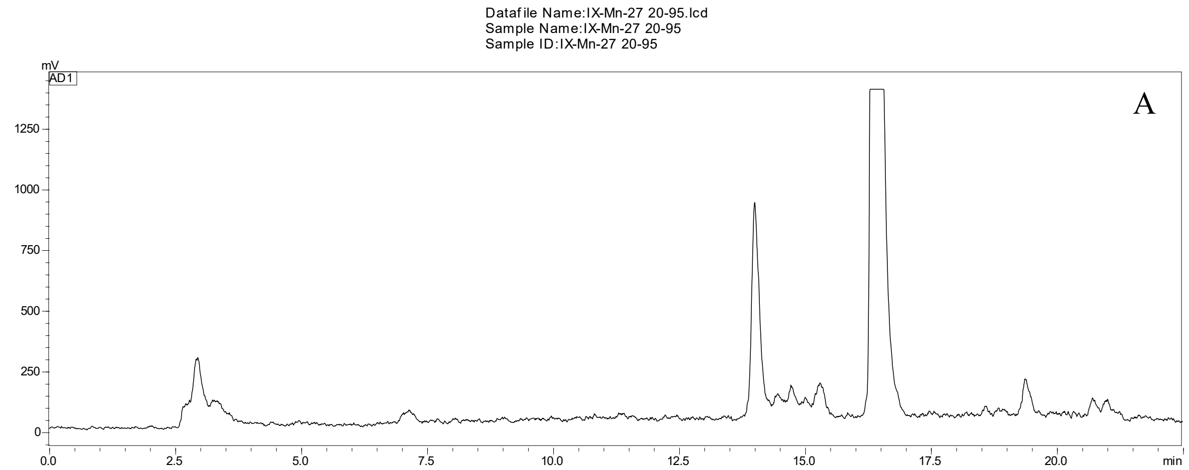
Datafile Name:IX-Mn-27 20-95.lcd
Sample Name:IX-Mn-27 20-95
Sample ID:IX-Mn-27 20-95
mV
AD1
A
1250
1000
750
500
250
0
0.0
2.5
5.0
7.5
10.0
12.5
15.0
17.5
20.0
min

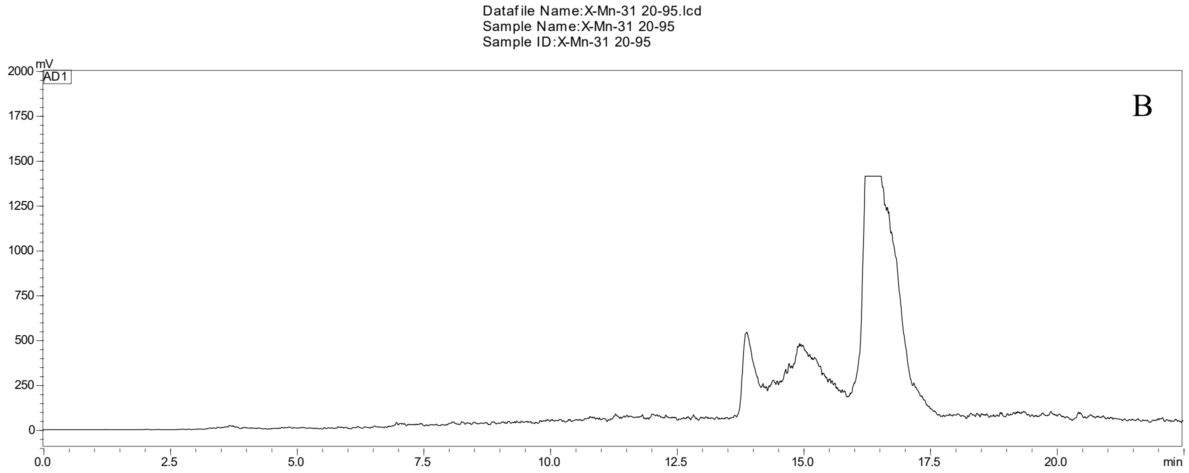
Datafile Name:X-Mn-31 20-95.lcd
Sample Name:X-Mn-31 20-95
Sample ID:X-Mn-31 20-95
mV
AD1
B
2000
1750
1500
1250
1000
750
500
250
0
0.0
2.5
5.0
7.5
10.0
12.5
15.0
17.5
20.0
min

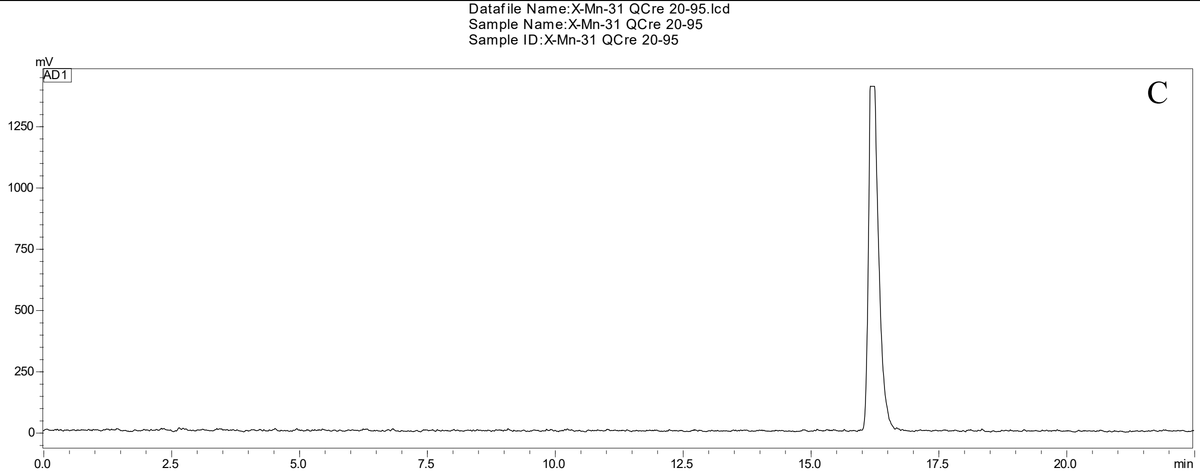

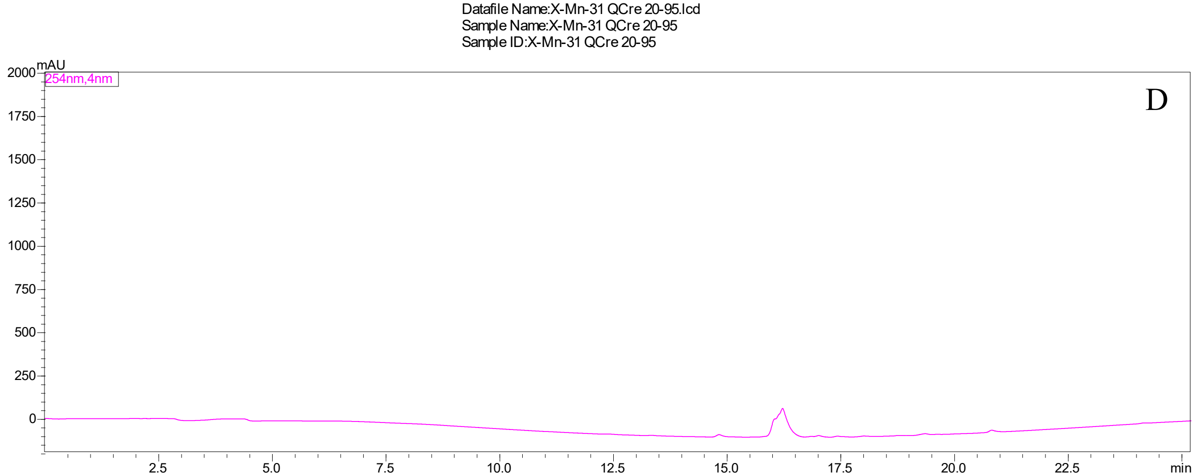

Figure S52: Radio-HPLC analysis of reaction mixture from **S41**. Reaction mixture with HPLC method 1 (A), reaction mixture with HPLC method 1 (B), and QC for **[^{18}F]41** (C and D).

HPLC Conditions for QC: Column: Phenomenex, Kinetex® 5µm F5 100 Å, 250 × 4.6 mm LC Column

Solvent A: 0.1% TFA water, Solvent B: 0.1% TFA acetonitrile; Isocratic / Gradient elution: 20% Solvent B for 0 to 2 min, 20% – 95% Solvent B for 2 to 22.5 min. Flow rate: 1 mL/min.

1.4.8 Molar activity calculation for [^{18}F]-5-(fluoro)-2-methoxypyridine [^{18}F]19

Molar activity was calculated using a calibration curve of the corresponding fluorinated arene standard. A calibration curve [Y axis = UV area, X axis = mass (µg)] was created from the HPLC trace of a standard solution of 5-fluoro-2-methoxypyridine **[^{19}F]19**. The radiolabeled product from the labeling reaction was collected and purified by HPLC; the UV area corresponding to the radio peak was then recorded. Dividing the product decay-corrected radioactivity by the mole number gives the molar activity (Ci/µmol).

The Isolated tracer [^{18}F]-5-(fluoro)-2-methoxypyridine **[^{18}F]19** has a molar activity of 0.18 Ci/µmol, which is decay-corrected to the end of bombardment (EOB).

A

5-Fluoro-2-methoxypyridine [^{19}F]19 (µg)	UV area (212 nm)
0	0
0.05	119586
0.1	245709
0.25	719977
0.5	1096917
1	2716315
1.5	3841740
2	5175537

B

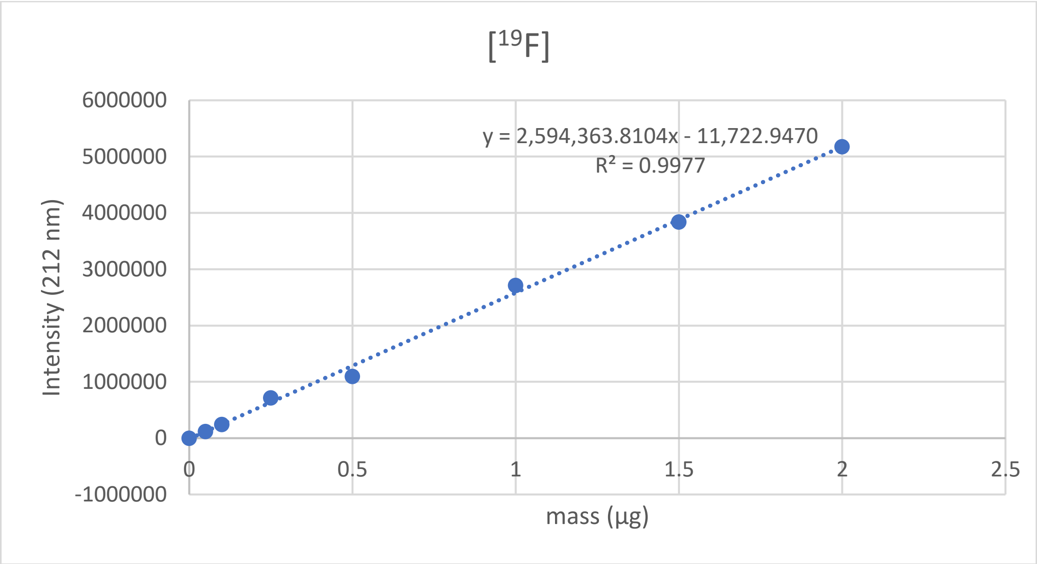

C

Time from EOB	Decay Corrected (EOB) activity	UV (212nm)	Mass	n	Molar activity
7 h 34 min	1250 µCi	2295170	0.88 µg	0.00699 µmol	0.18 Ci/µmol

Figure S53: A and **B** Standard curve data for 5-Fluoro-2-methoxypyridine **[^{19}F]19**; **C** the molar activity for **[^{18}F]19** calculated from parts **A** and **B**.

1.4.9 Molar activity calculation for [^{18}F]-methyl 2-((*tert*-butoxycarbonyl)amino)-3-(4-fluoro-3-methoxyphenyl)propanoate ([^{18}F]39)

Molar activity was calculated using a calibration curve of the corresponding fluorinated arene standard. The same procedure was followed as described in 1.4.8. The Isolated tracer [^{18}F]-methyl 2-((*tert*-butoxycarbonyl)amino)-3-(4-fluoro-3-methoxyphenyl)propanoate **([^{18}F]39)** has a molar activity of 0.0125 Ci/µmol, which is decay-corrected to the end of bombardment (EOB).

A

**[^{19}F]39 (µg)**	**Intensity (212 nm)**
0	0
0.05	76771
0.1	118156
0.5	617354
1	1165915
1.5	1981853
2	2317959

B

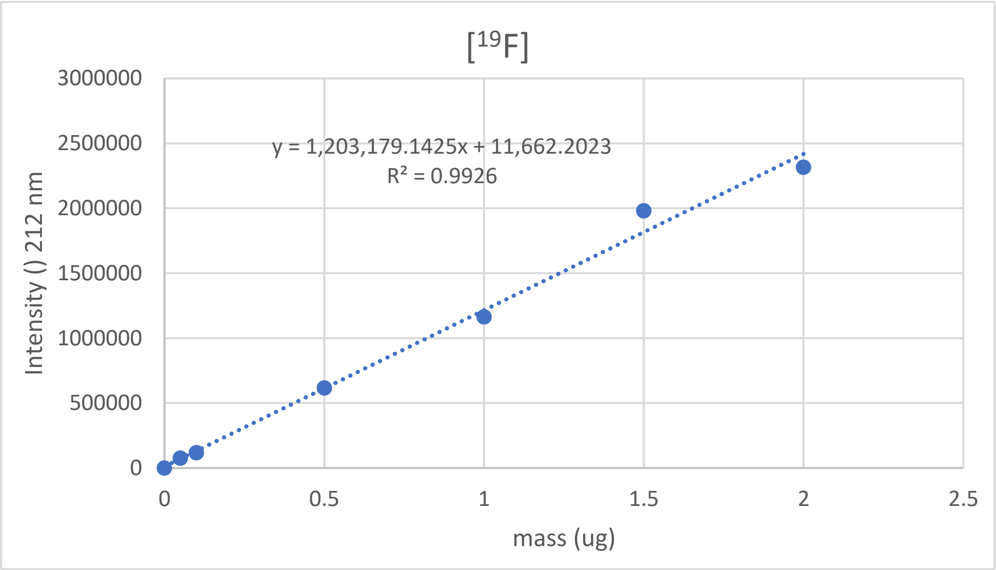

C

Time From EOB	Decay Corrected (EOB) Activity	UV (212 nm)	Mass	n	Molar activity
5 h 04 min	553uCi	17541923	14.57 ug	0.0445	0.0125 Ci/µmol

Figure S54: **A** and **B** Standard curve data for **[^{19}F]39**; **C** the molar activity for **[^{18}F]39** calculated from parts **A** and **B**.

1.4.10 Small animal PET imaging study

MCF-7 human breast adenocarcinoma cells (ATCC, Manassas, VA, USA) and B16F10 murine melanoma cells were cultured in Modified Eagle's Medium (MEM, Gibco, Waltham, MA, USA) and Roswell Park Memorial Institute (RPMI) 1640 (Gibco, Waltham, MA, USA) respectively, both supplemented with 10% fetal bovine serum (FBS, Gibco). Cells were maintained in a humidified incubator at 37°C with 5% CO_2. The cells were passaged upon reaching 70-80% confluence using 0.25% trypsin-EDTA (Gibco) for detachment. Animal experiments were performed in compliance with the protocol approved by the University of North Carolina Institutional Animal Care and Use Committee (IACUC). The approved protocol number is 23-096.0. Female athymic BALB/c nude mice, aged 6–8 weeks (weight: 18–22 g), were obtained from animal facility and housed in a controlled environment (temperature 22 ± 2 °C, 12–hour light/dark cycle) with free access to food and water. For tumor establishment, MCF-7 and B16F10 cells were harvested at 70–80% confluence, trypsinized, and resuspended in sterile phosphate-buffered saline (PBS) at a concentration of 4×10^6 cells and $0.1–0.5 \times 10^6$ cells, respectively, in a total volume of 100 µL. The mice were anesthetized using 2.5% isoflurane (AbbVie, Lake County, IL, USA) and tumor cells were subcutaneously injected into the left and right shoulder of the mice. To support the growth of ER^+ MCF-7 xenografts, mice were implanted subcutaneously with slow-release 17*β*-estradiol pellets (0.72 mg, 60-day release) five to seven days before tumor inoculation. B16F10 tumors were also inoculated in C57BL/6 mouse. The animals are sent to PET/CT images when the tumor volume reaches 150 mm^3.

PET/CT imaging was performed using a small animal PET/CT scanner (SuperARgus-4R, Sedecal, Madrid, Spain) to evaluate the biodistribution of ^{18}F-labeled tracers. Animals were injected intravenously with ((±)-[^{18}F]**42**) at a dose of approximately 80 µCi per mouse. Animals were anesthetized using 2.5% isoflurane in oxygen for induction and maintained at 2% isoflurane during the imaging procedure. Mice were positioned prone on the scanner bed, and body temperature was maintained using a heated lamp. CT imaging was performed first for anatomical localization using a tube voltage of 70 kV and a current of 300 µA with a 360-projections. PET acquisition was conducted immediately after CT, with an acquisition time of 15 minutes per bed at 1.5 h post injection (p.i.).

PET data were reconstructed using the 3D ordered subsets expectation maximization (OSEM) algorithm. Regions of interest (ROIs) were drawn manually over the areas of tracer uptake using AMIDE software to quantify activity concentration as percentage of injected dose per gram (%ID/g).

2. Supplementary References

(1) Roth, H.; Romero, N.; Nicewicz, D. Experimental and Calculated Electrochemical Potentials of Common Organic Molecules for Applications to Single-Electron Redox Chemistry. *Synlett* **2015**, *27* (05), 714–723. https://doi.org/10.1055/s-0035-1561297.

(2) Li, P.; Deetz, A. M.; Hu, J.; Meyer, G. J.; Hu, K. Chloride Oxidation by One- or Two-Photon Excitation of *N*-Phenylphenothiazine. *J. Am. Chem. Soc.* **2022**, *144* (38), 17604–17610. https://doi.org/10.1021/jacs.2c07107.

(3) Zhou, M.; Ni, C.; Zeng, Y.; Hu, J. Trifluoromethyl Benzoate: A Versatile Trifluoromethoxylation Reagent. *J. Am. Chem. Soc.* **2018**, *140* (22), 6801–6805. https://doi.org/10.1021/jacs.8b04000.

(4) Mills, L. R. Iron-Catalyzed Kumada Arylation of Aliphatic Alcohol-Derived Electrophiles via Sulfonate-to-Halide Substitution. *Organometallics* **2025**, *44* (7), 858–865. https://doi.org/10.1021/acs.organomet.5c00001.

(5) Yasuda, S.; Yorimitsu, H.; Oshima, K. Vanadium-Catalyzed Cross-Coupling Reactions of Alkyl Halides with Aryl Grignard Reagents. *Bull. Chem. Soc. Jpn.* **2008**, *81* (2), 287–290. https://doi.org/10.1246/bcsj.81.287.

(6) Wartmann, C.; Nandi, S.; Neudörfl, J.; Berkessel, A. Titanium Salalen Catalyzed Enantioselective Benzylic Hydroxylation. *Angew. Chem. Int. Ed.* **2023**, *62* (35), e202306584. https://doi.org/10.1002/anie.202306584.

(7) Nicolaou, K. C.; Nilewski, C.; Hale, C. R. H.; Ioannidou, H. A.; ElMarrouni, A.; Koch, L. G. Total Synthesis and Structural Revision of Viridicatumtoxin B. *Angew. Chem. Int. Ed.* **2013**, *52* (33), 8736–8741. https://doi.org/10.1002/anie.201304691.

(8) Wang, Y.; Pan, L.; Lu, K.; Hu, M.; Zheng, C.; Li, M.; Xie, Y.; Yang, C.; Sun, H.; Wu, X.; Wu, H.; Chen, W. Photocatalyzed ^{18}F-Fluorination: A Streamlined Radiolabeling Approach for Rapid Adoption and Automation. *Org. Lett.* **2025**, *27* (26), 7224–7229. https://doi.org/10.1021/acs.orglett.5c02193.

(9) Zhu, Z.; Huang, C.-Y.; Russell, R. W.; Kinon, P.; Ma, X.; Mao, Y.; Wu, X.; Wu, Z.; Nicewicz, D. A.; Li, Z. Integrated Transient Chromophores for Efficient Photo-Induced Radiofluorination. *Chem* **2026**, 102908. https://doi.org/10.1016/j.chempr.2025.102908.

(10) Tay, N. E. S.; Chen, W.; Levens, A.; Pistritto, V. A.; Huang, Z.; Wu, Z.; Li, Z.; Nicewicz, D. A. ^{19}F- and ^{18}F-Arene Deoxyfluorination via Organic Photoredox-Catalysed Polarity-Reversed Nucleophilic Aromatic Substitution. *Nat. Catal.* **2020**, *3* (9), 734–742. https://doi.org/10.1038/s41929-020-0495-0.

3. NMR spectra of new compounds (^{1}H NMR, ^{13}C NMR, and ^{19}F NMR

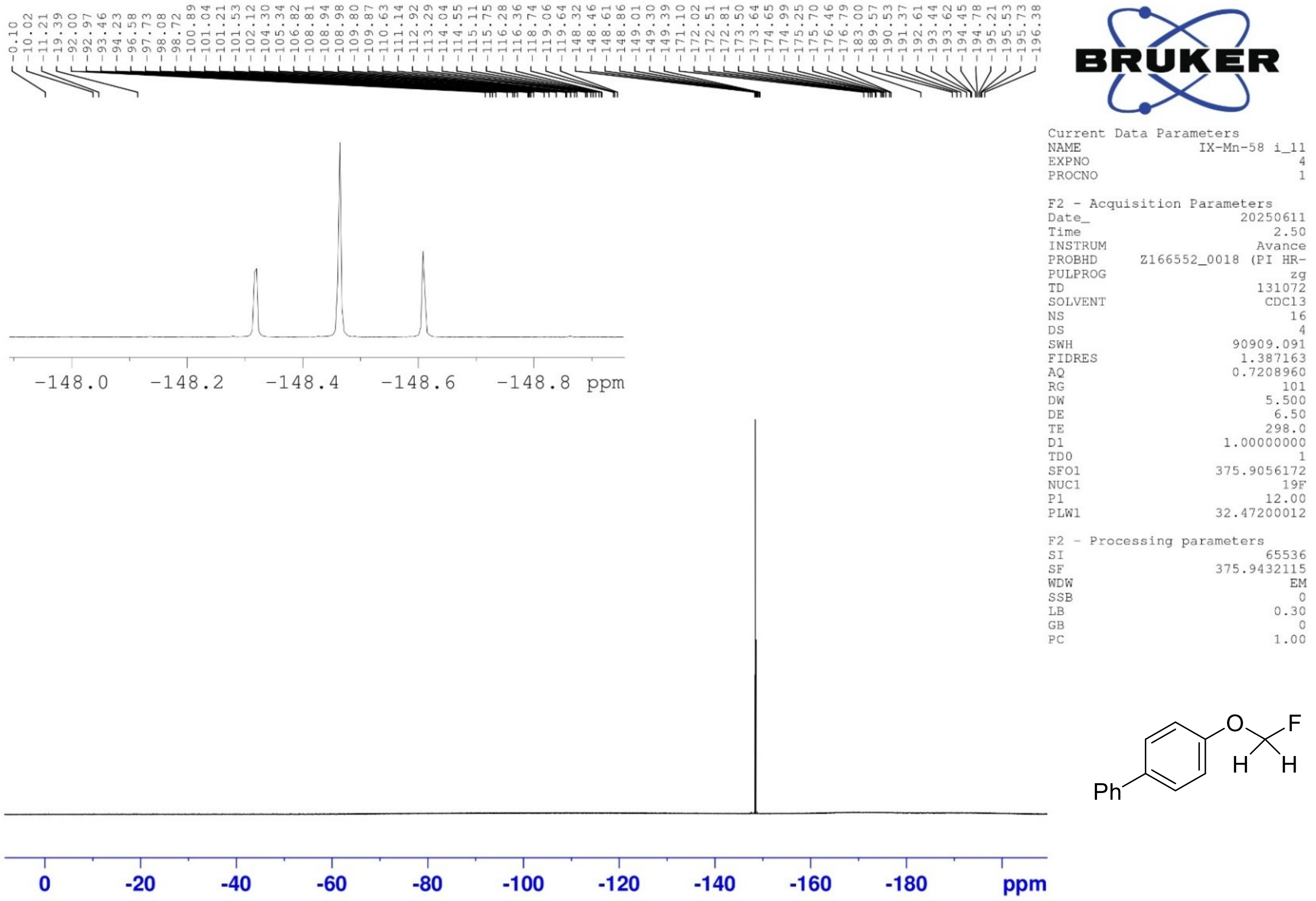

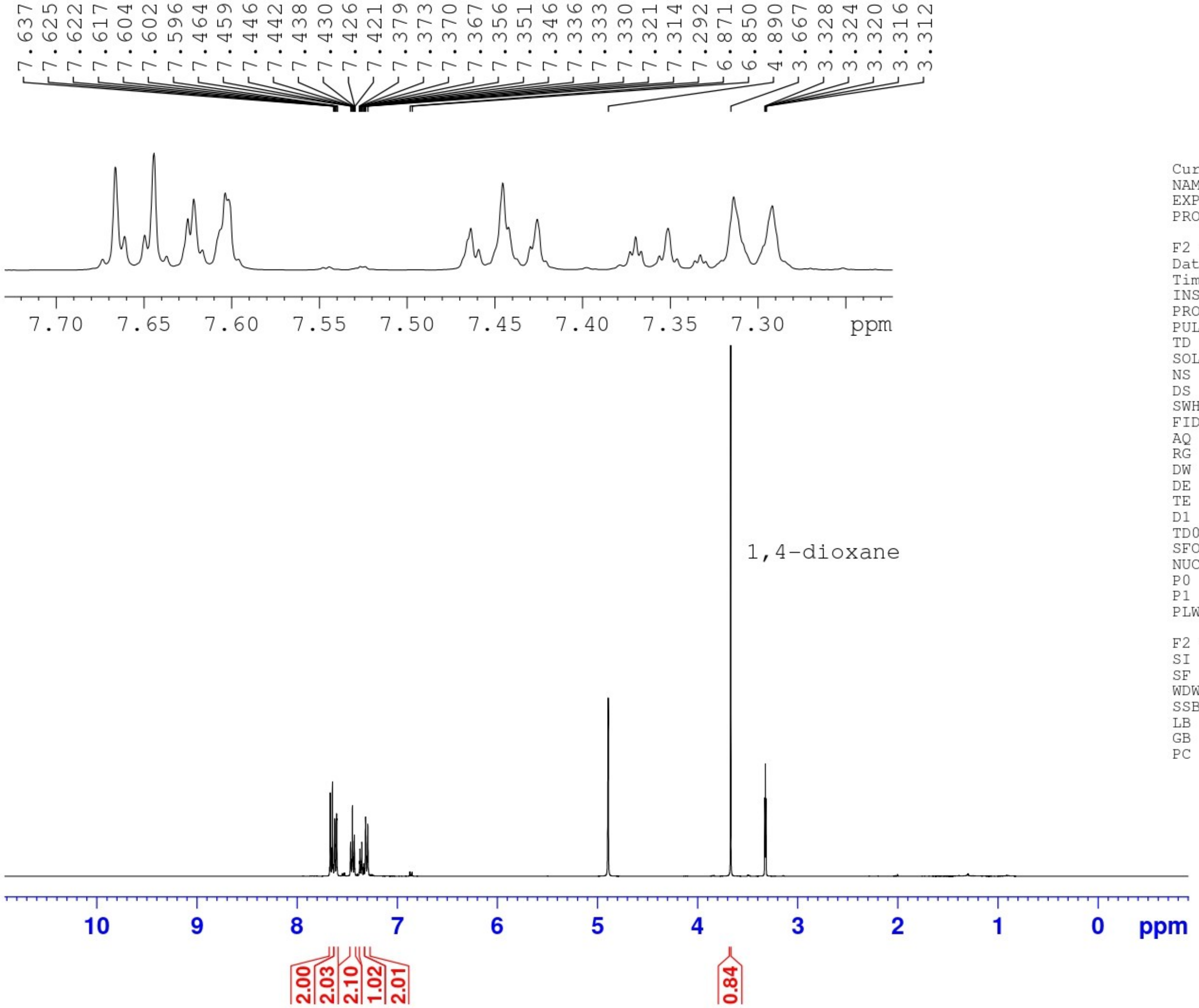

Current Data Parameters
NAME IX-Mn-82 Re_10
EXPNO 1
PROCNO 1

F2 - Acquisition Parameters
Date_ 20250915
Time 17.16
INSTRUM Avance
PROBHD Z166552_0018 (PI HR-
PULPROG zg30
TD 65536
SOLVENT MeOD
NS 16
DS 2
SWH 7812.500
FIDRES 0.238419
AQ 4.1943040
RG 101
DW 64.000
DE 6.67
TE 298.0
D1 1.00000000
TD0 1
SFO1 399.5424672
NUC1 1H
P0 2.60
P1 7.80
PLW1 21.19799995

F2 - Processing parameters
SI 65536
SF 399.5400035
WDW EM
SSB 0
LB 0.30
GB 0
PC 1.00

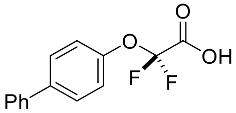

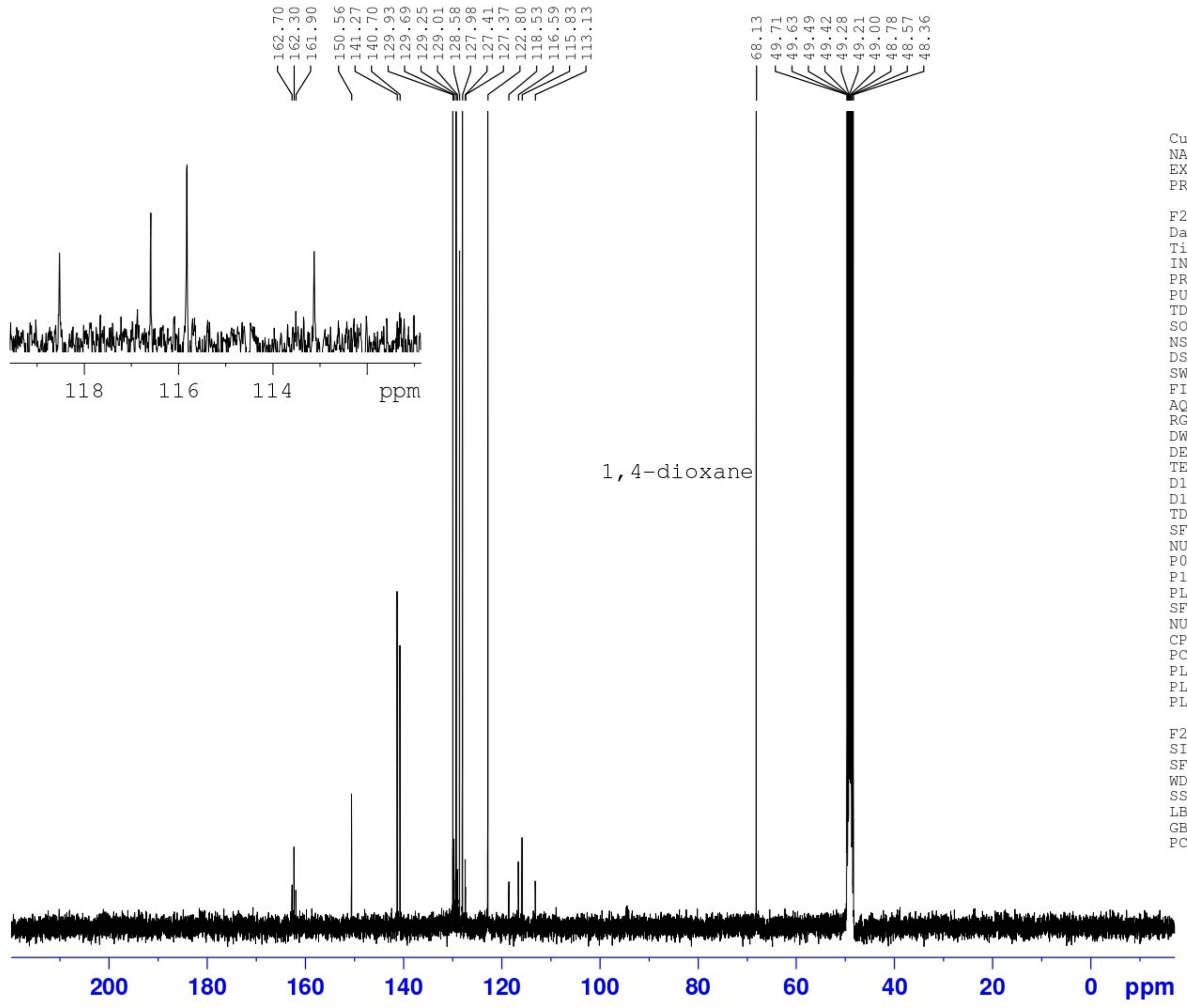

Current Data Parameters
NAME IX-Mn-82 Re_12
EXPNO 1
PROCNO 1

F2 - Acquisition Parameters

Date_	20250915
Time	20.02
INSTRUM	Avance
PROBHD	Z166552_0018 (PI HR-
PULPROG	zgpg30
TD	65536
SOLVENT	MeOD
NS	2048
DS	4
SWH	23809.524
FIDRES	0.726609
AQ	1.3762560
RG	101
DW	21.000
DE	6.50
TE	298.0
D1	2.00000000
D11	0.03000000
TD0	1
SFO1	100.4744593
NUC1	13C
P0	2.67
P1	8.00
PLW1	88.22599792
SFO2	399.5415982
NUC2	1H
CPDPRG[2]	waltz65
PCPD2	90.00
PLW2	21.19799995
PLW12	0.15922000
PLW13	0.08008700

F2 - Processing parameters

SI	32768
SF	100.4642725
WDW	EM
SSB	0
LB	1.00
GB	0
PC	1.40

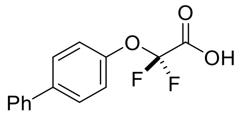

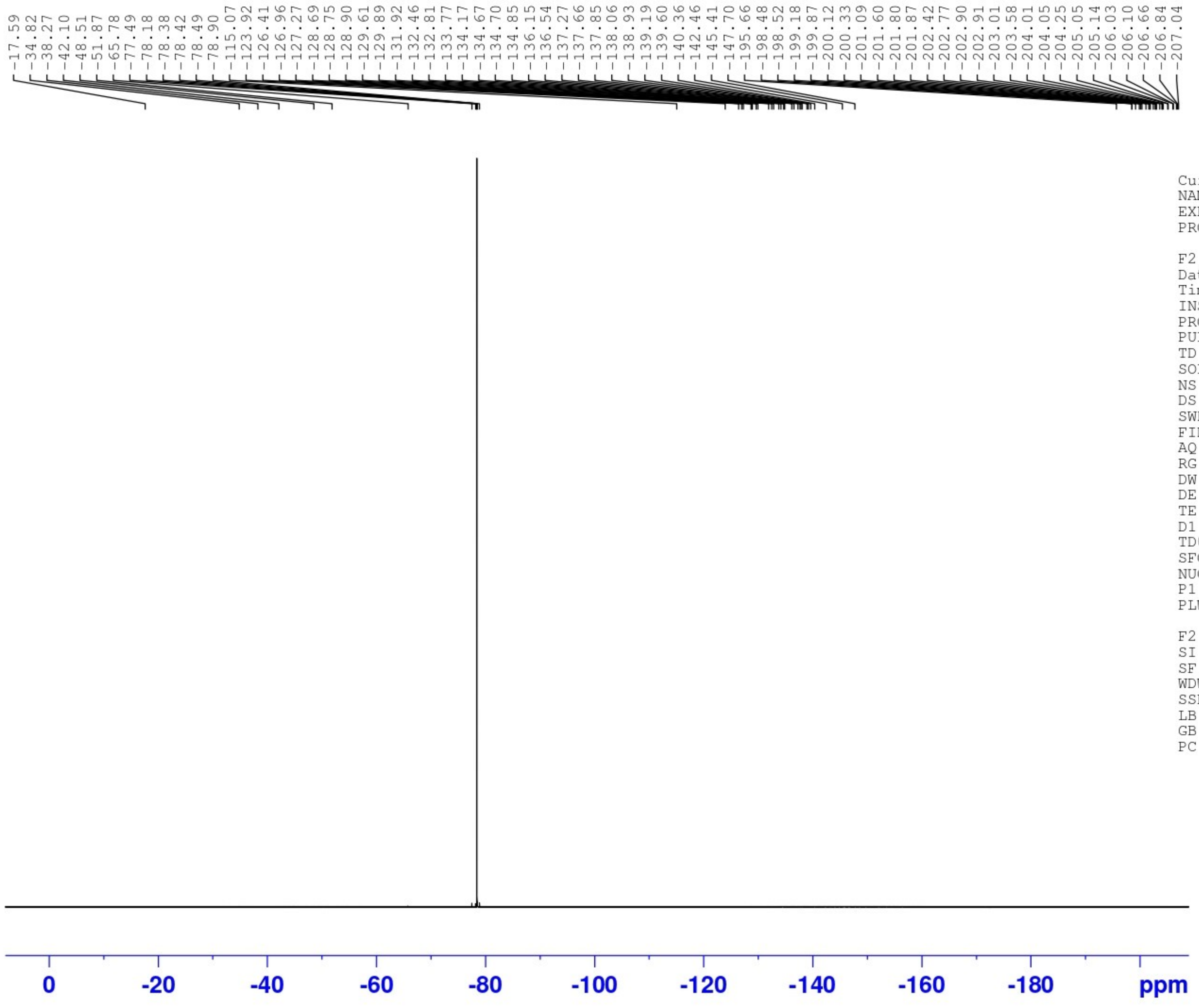

BRUKER

Current Data Parameters	
NAME	IX-Mn-82 Re_11
EXPNO	1
PROCNO	1

F2 - Acquisition Parameters	
Date_	20250915
Time	17.17
INSTRUM	Avance
PROBHD	Z166552_0018 (PI HR-
PULPROG	zg
TD	131072
SOLVENT	MeOD
NS	16
DS	4
SWH	90909.091
FIDRES	1.387163
AQ	0.7208960
RG	101
DW	5.500
DE	6.50
TE	298.0
D1	1.00000000
TD0	1
SFO1	375.9056172
NUC1	19F
P1	12.00
PLW1	32.47200012

F2 - Processing parameters	
SI	65536
SF	375.9432115
WDW	EM
SSB	0
LB	0.30
GB	0
PC	1.00

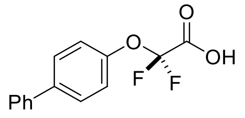

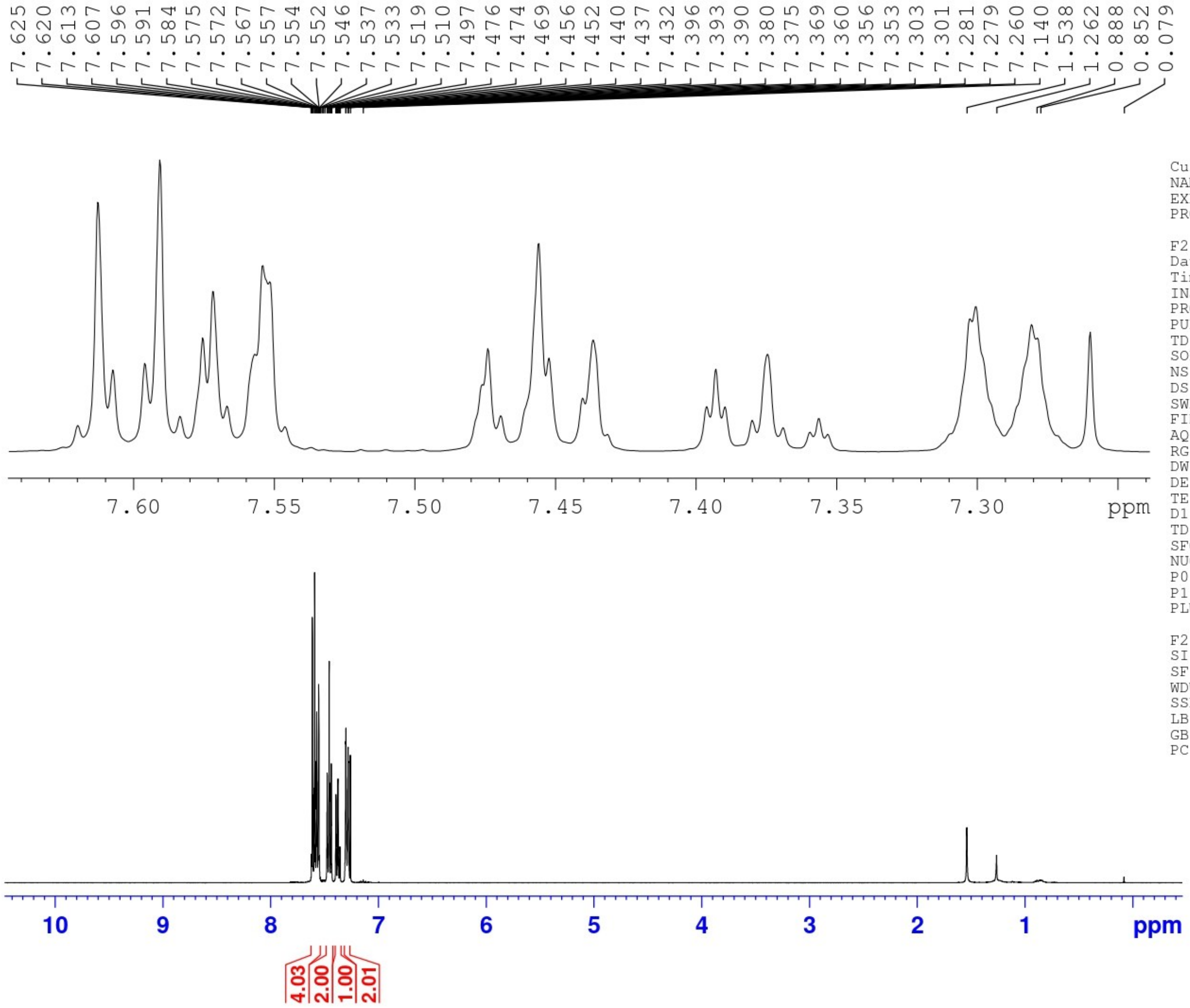

BRUKER

Current Data Parameters

NAME	IX-Mn-83 i_10
EXPNO	3
PROCNO	1

F2 - Acquisition Parameters

Date_	20250712
Time	18.10
INSTRUM	Avance
PROBHD	Z166552_0018 (PI HR-
PULPROG	zg30
TD	65536
SOLVENT	CDCl3
NS	16
DS	2
SWH	7812.500
FIDRES	0.238419
AQ	4.1943040
RG	101
DW	64.000
DE	6.67
TE	298.0
D1	1.00000000
TD0	1
SFO1	399.5424672
NUC1	1H
P0	2.60
P1	7.80
PLW1	21.19799995

F2 - Processing parameters

SI	65536
SF	399.5400096
WDW	EM
SSB	0
LB	0.30
GB	0
PC	1.00

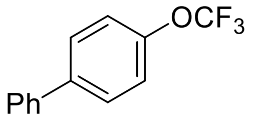

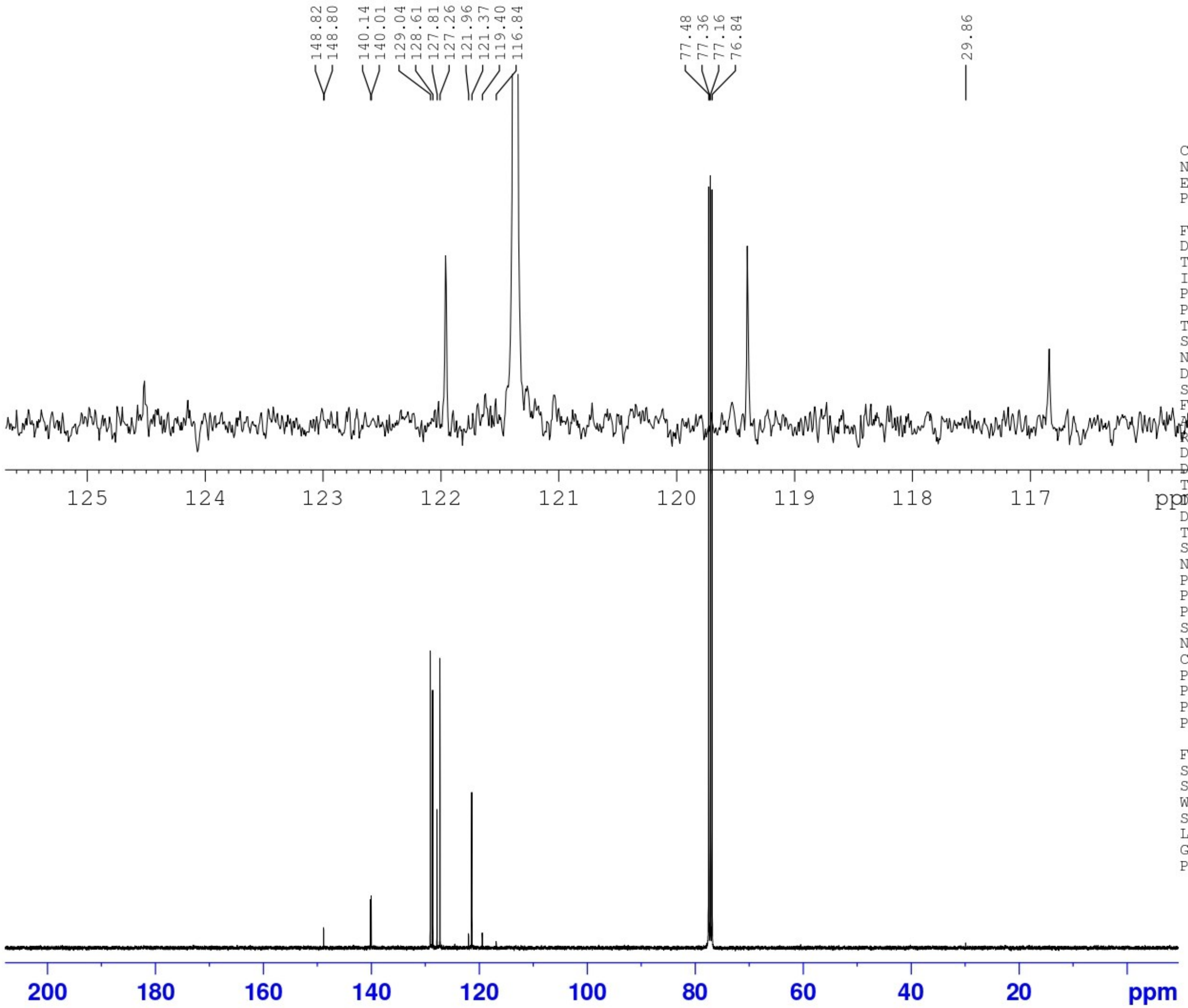

Current Data Parameters
NAME IX-Mn-83 i_12
EXPNO 2
PROCNO 1

F2 - Acquisition Parameters
Date_ 20250712
Time 20.11
INSTRUM Avance
PROBHD Z166552_0018 (PI HR-
PULPROG zgpg30
TD 65536
SOLVENT CDCl3
NS 2048
DS 4
SWH 23809.524
FIDRES 0.726609
AQ 1.3762560
RG 101
DW 21.000
DE 6.50
TE 298.0
[illegible] 2.00000000
D11 0.03000000
TD0 1
SFO1 100.4744593
NUC1 13C
P0 2.67
P1 8.00
PLW1 88.22599792
SFO2 399.5415982
NUC2 1H
CPDPRG[2] waltz65
PCPD2 90.00
PLW2 21.19799995
PLW12 0.15922000
PLW13 0.08008700

F2 - Processing parameters
SI 32768
SF 100.4643991
WDW EM
SSB 0
LB 1.00
GB 0
PC 1.40

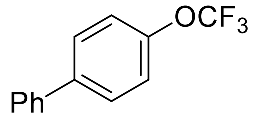

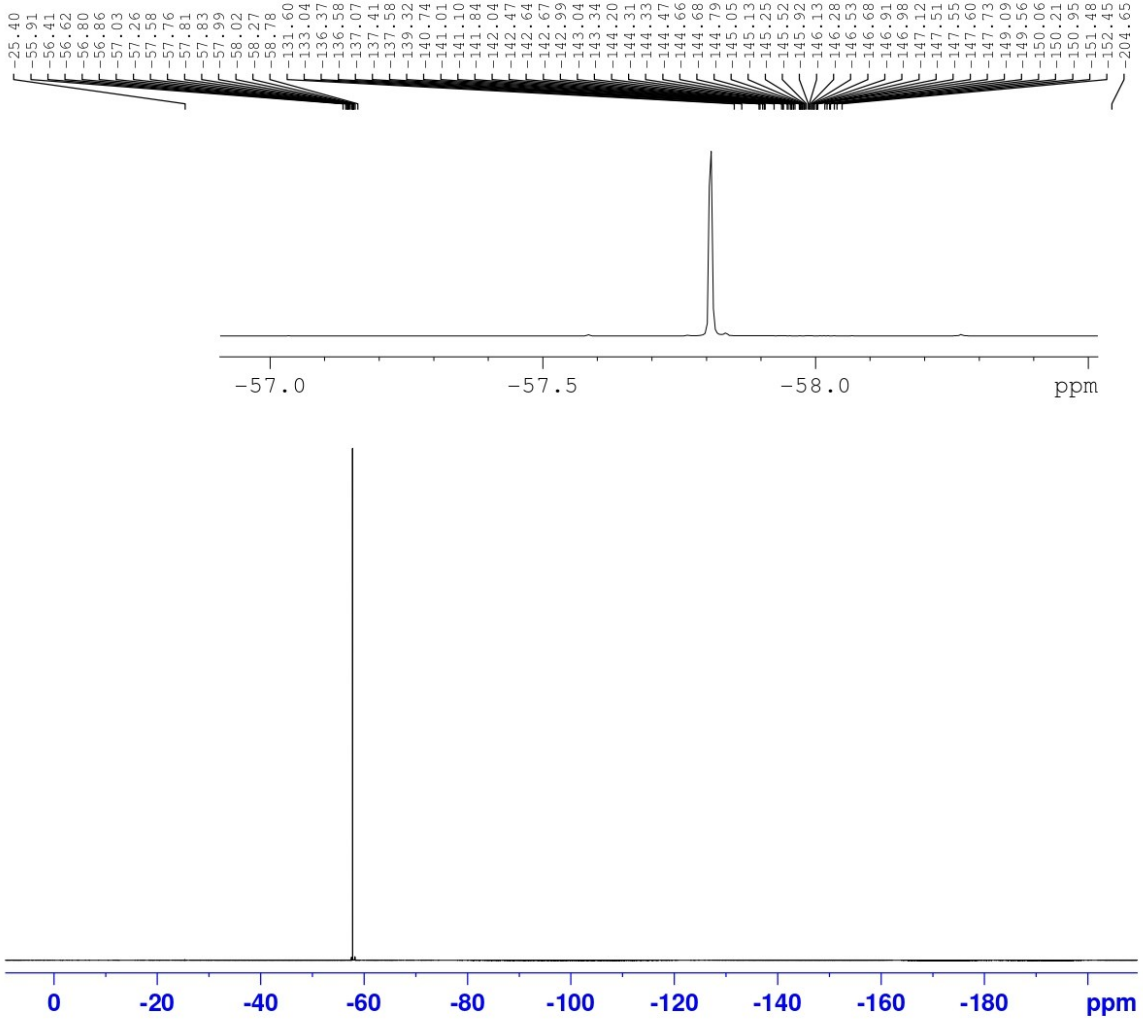

Current Data Parameters
NAME IX-Mn-83 i_11
EXPNO 2
PROCNO 1

F2 - Acquisition Parameters
Date_ 20250712
Time 18.11
INSTRUM Avance
PROBHD Z166552_0018 (PI HR-
PULPROG zg
TD 131072
SOLVENT CDCl3
NS 16
DS 4
SWH 90909.091
FIDRES 1.387163
AQ 0.7208960
RG 101
DW 5.500
DE 6.50
TE 298.0
D1 1.00000000
TD0 1
SFO1 375.9056172
NUC1 19F
P1 12.00
PLW1 32.47200012

F2 - Processing parameters
SI 65536
SF 375.9432115
WDW EM
SSB 0
LB 0.30
GB 0
PC 1.00

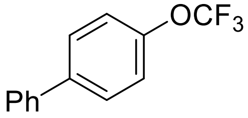

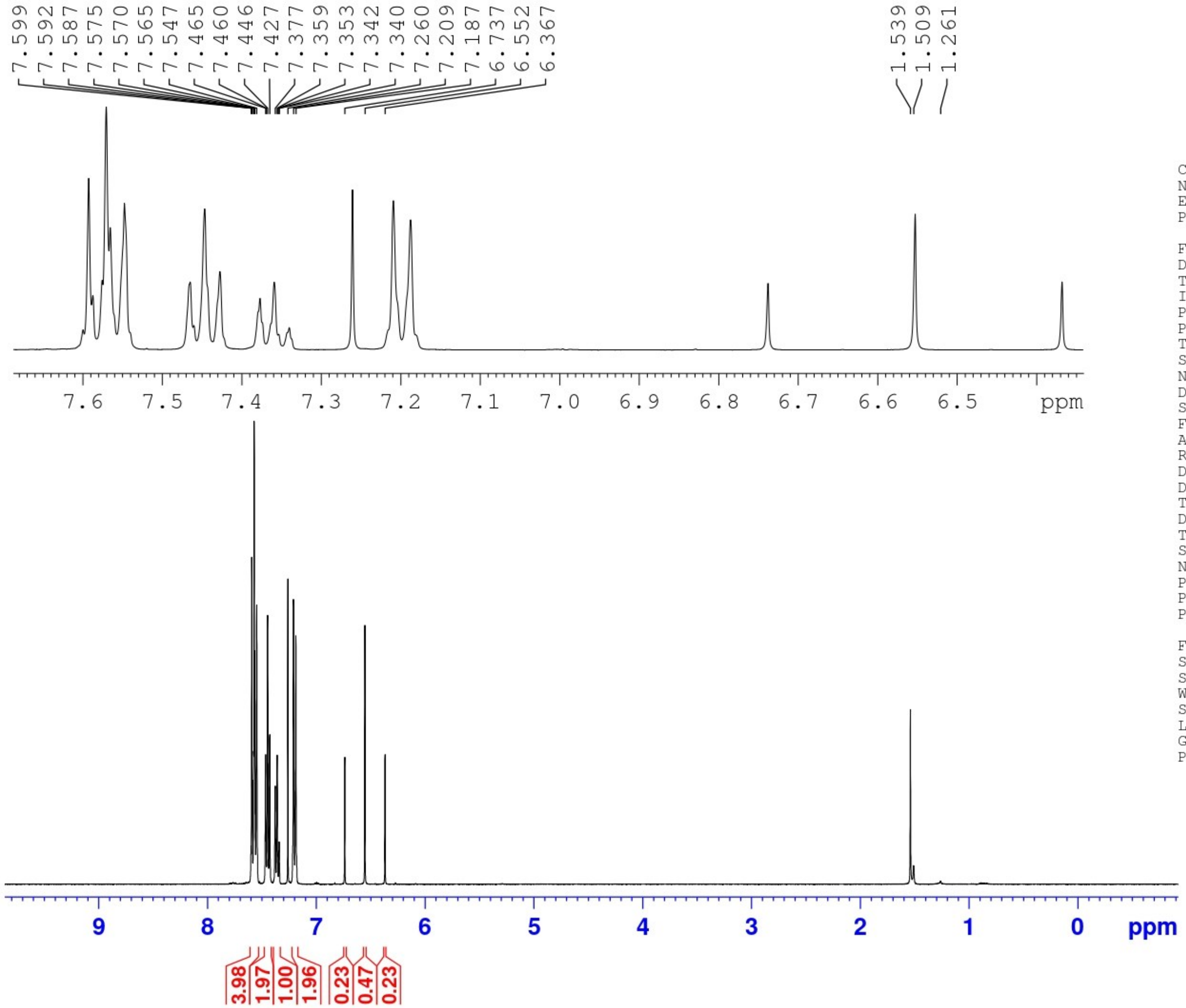

Current Data Parameters
NAME IX-MN-85 i_10
EXPNO 2
PROCNO 1

F2 - Acquisition Parameters
Date_ 20250707
Time 17.10
INSTRUM Avance
PROBHD Z166552_0018 (PI HR-
PULPROG zg30
TD 65536
SOLVENT CDCl3
NS 16
DS 2
SWH 7812.500
FIDRES 0.238419
AQ 4.1943040
RG 101
DW 64.000
DE 6.67
TE 298.0
D1 1.00000000
TD0 1
SFO1 399.5424672
NUC1 1H
P0 2.60
P1 7.80
PLW1 21.19799995

F2 - Processing parameters
SI 65536
SF 399.5400095
WDW EM
SSB 0
LB 0.30
GB 0
PC 1.00

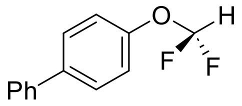

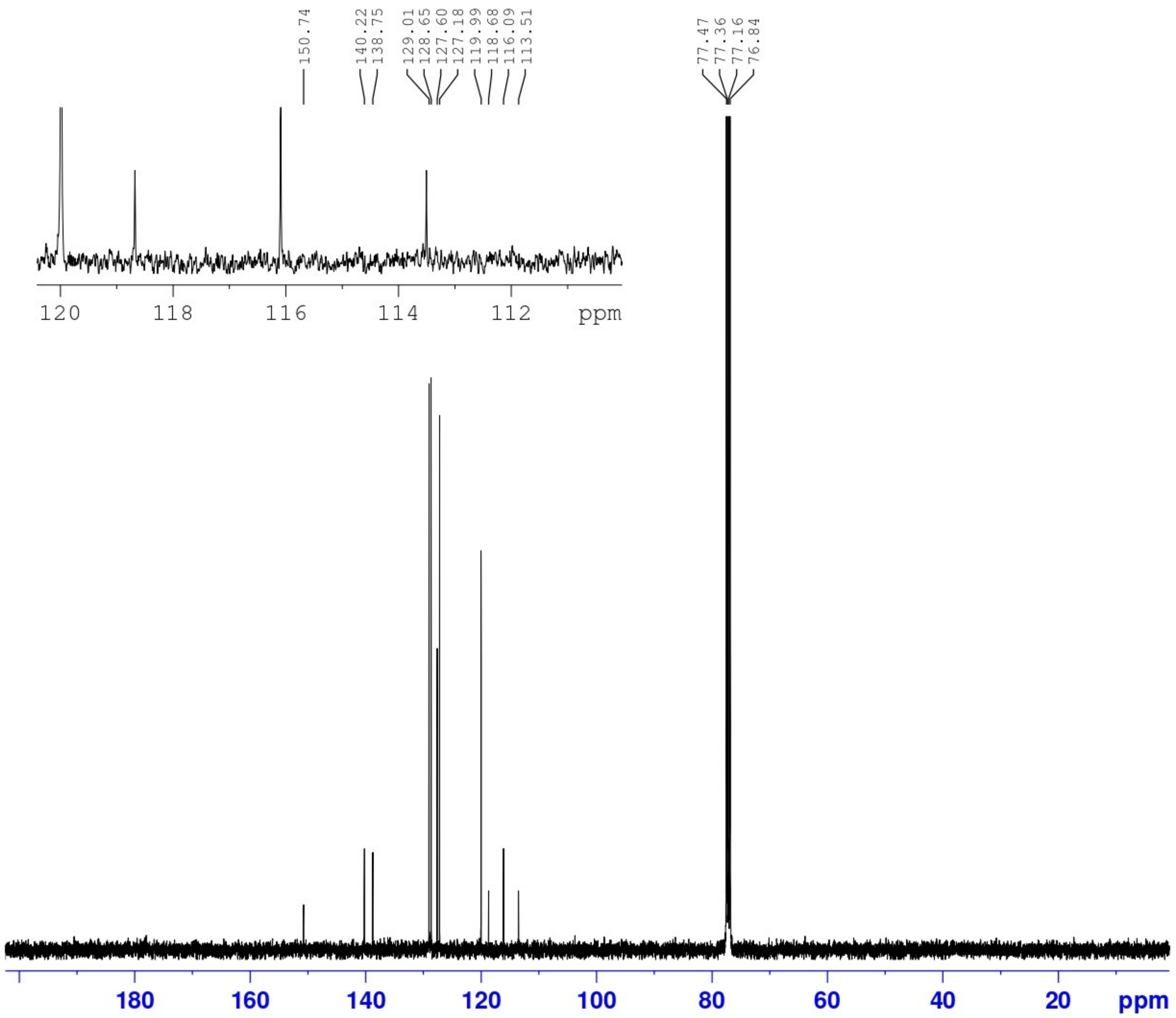

Current Data Parameters
NAME IX-MN-85 i_12
EXPNO 2
PROCNO 1

F2 - Acquisition Parameters
Date_ 20250707
Time 19.03
INSTRUM Avance
PROBHD Z166552_0018 (PI HR-
PULPROG zgpg30
TD 65536
SOLVENT CDCl3
NS 1024
DS 4
SWH 23809.524
FIDRES 0.726609
AQ 1.3762560
RG 101
DW 21.000
DE 6.50
TE 298.0
D1 2.00000000
D11 0.03000000
TD0 1
SFO1 100.4744593
NUC1 13C
P0 2.67
P1 8.00
PLW1 88.22599792
SFO2 399.5415982
NUC2 1H
CPDPRG[2] waltz65
PCPD2 90.00
PLW2 21.19799995
PLW12 0.15922000
PLW13 0.08008700

F2 - Processing parameters
SI 32768
SF 100.4643991
WDW EM
SSB 0
LB 1.00
GB 0
PC 1.40

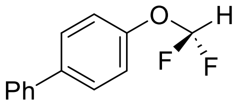

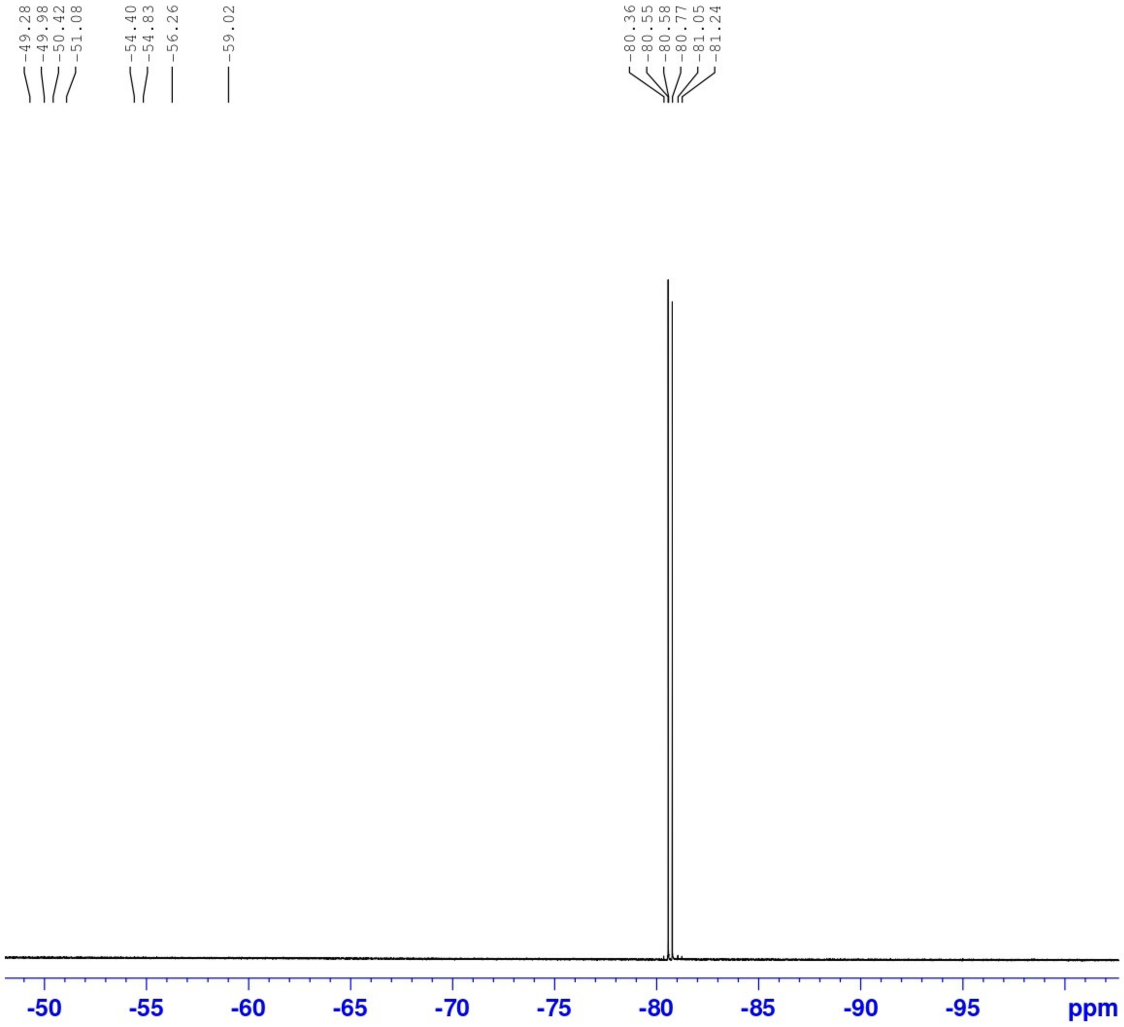

BRUKER

Current Data Parameters
NAME IX-MN-85 i_11
EXPNO 2
PROCNO 1

F2 - Acquisition Parameters
Date_ 20250707
Time 17.12
INSTRUM Avance
PROBHD Z166552_0018 (PI HR-
PULPROG zg
TD 131072
SOLVENT CDCl3
NS 16
DS 4
SWH 90909.091
FIDRES 1.387163
AQ 0.7208960
RG 101
DW 5.500
DE 6.50
TE 298.0
D1 1.00000000
TD0 1
SFO1 375.9056172
NUC1 19F
P1 12.00
PLW1 32.47200012

F2 - Processing parameters
SI 65536
SF 375.9432115
WDW EM
SSB 0
LB 0.30
GB 0
PC 1.00

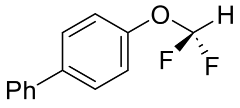

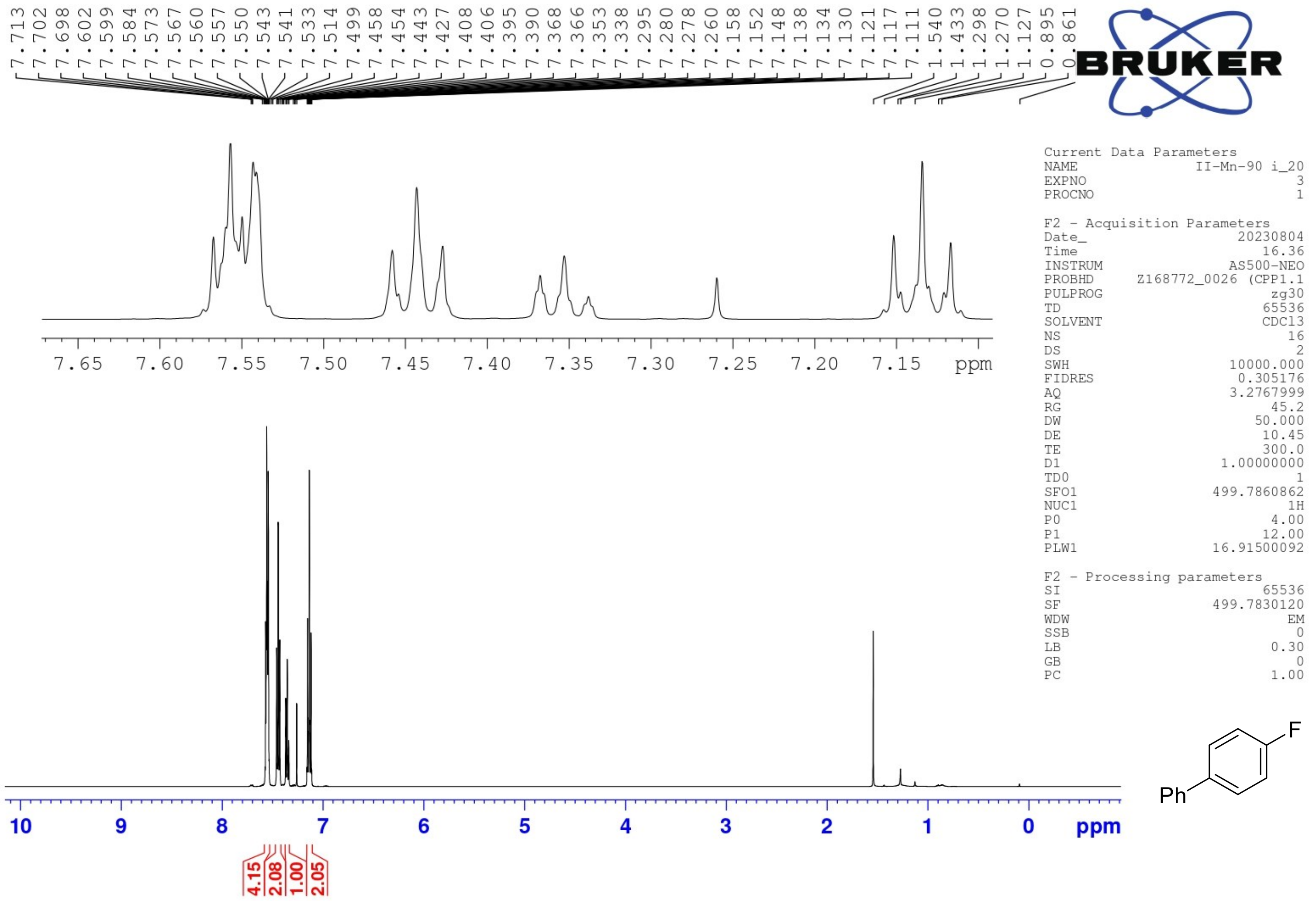
7.713
7.702
7.698
7.602
7.599
7.584
7.573
7.567
7.560
7.557
7.550
7.543
7.541
7.533
7.514
7.499
7.458
7.454
7.443
7.427
7.408
7.406
7.395
7.390
7.368
7.366
7.353
7.338
7.295
7.280
7.278
7.260
7.158
7.152
7.148
7.138
7.134
7.130
7.121
7.117
7.111
1.540
1.433
1.298
1.270
1.127
0.895
0.861
BRUKER
Current Data Parameters
NAME II-Mn-90 i_20
EXPNO 3
PROCNO 1
F2 - Acquisition Parameters
Date_ 20230804
Time 16.36
INSTRUM AS500-NEO
PROBHD Z168772_0026 (CPP1.1
PULPROG zg30
TD 65536
SOLVENT CDCl3
NS 16
DS 2
SWH 10000.000
FIDRES 0.305176
AQ 3.2767999
RG 45.2
DW 50.000
DE 10.45
TE 300.0
D1 1.00000000
TD0 1
SFO1 499.7860862
NUC1 1H
P0 4.00
P1 12.00
PLW1 16.91500092
F2 - Processing parameters
SI 65536
SF 499.7830120
WDW EM
SSB 0
LB 0.30
GB 0
PC 1.00
7.65 7.60 7.55 7.50 7.45 7.40 7.35 7.30 7.25 7.20 7.15 ppm
10 9 8 7 6 5 4 3 2 1 0 ppm
4.15
2.08
1.00
2.05
F
Ph

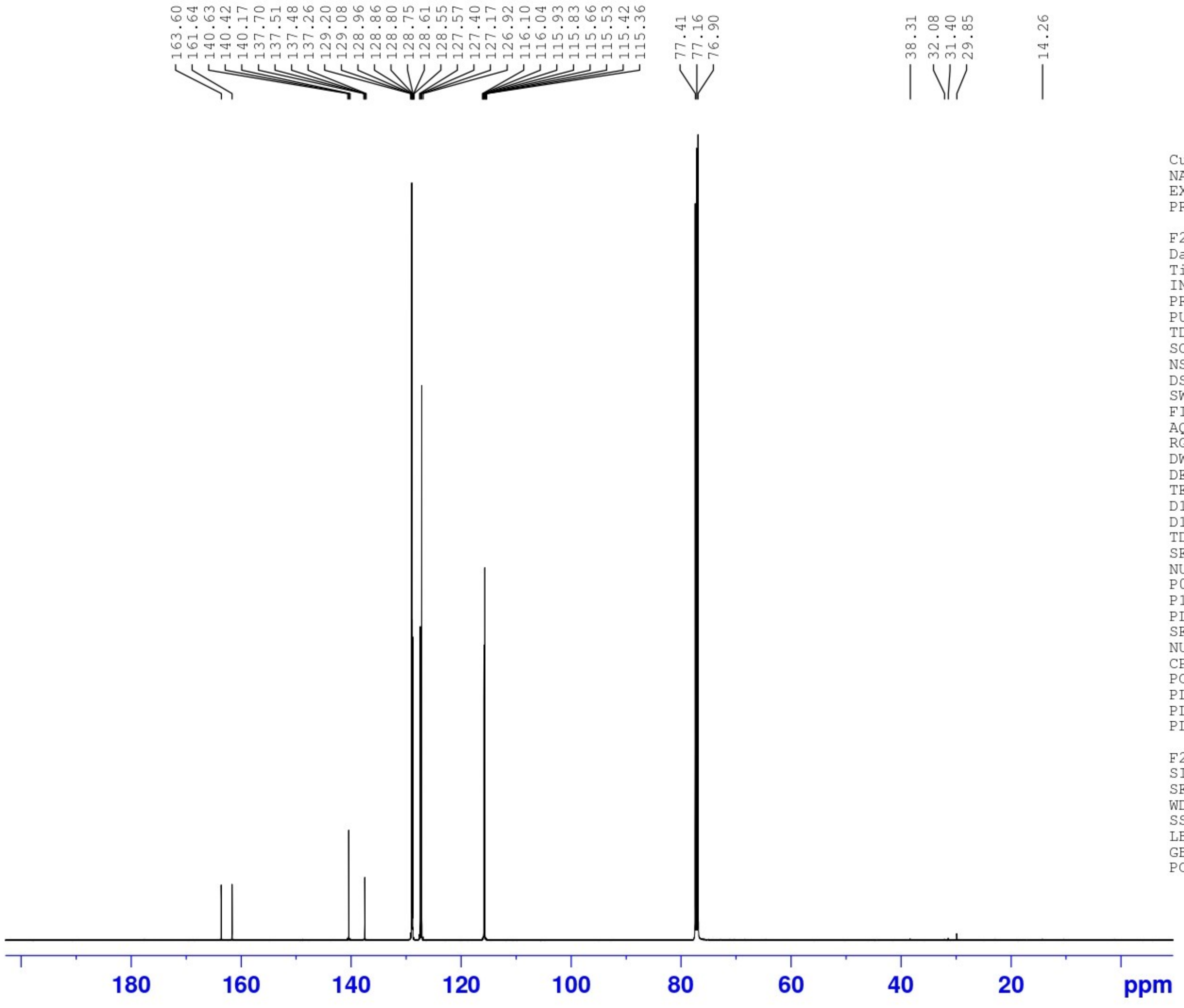


```
Current Data Parameters
NAME              II-Mn-90 i_30
EXPNO                         3
PROCNO                        1

F2 - Acquisition Parameters
Date_                  20230806
Time                       5.44
INSTRUM               AS500-NEO
PROBHD      Z168772_0026 (CPP1.1
PULPROG                  zgpg30
TD                        65536
SOLVENT                   CDCl3
NS                         8192
DS                            4
SWH                   30120.482
FIDRES                 0.919204
AQ                    1.0878977
RG                          101
DW                       16.600
DE                        18.00
TE                        300.0
D1                   2.00000000
D11                  0.03000000
TD0                           1
SFO1                125.6831024
NUC1                        13C
P0                         3.33
P1                        10.00
PLW1                59.16400146
SFO2                499.7849991
NUC2                         1H
CPDPRG[2]               waltz65
PCPD2                     80.00
PLW2                16.91500092
PLW12                0.38058999
PLW13                0.19113000

F2 - Processing parameters
SI                        32768
SF                  125.6705188
WDW                          EM
SSB                           0
LB                         1.00
GB                            0
PC                         1.40
```

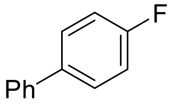

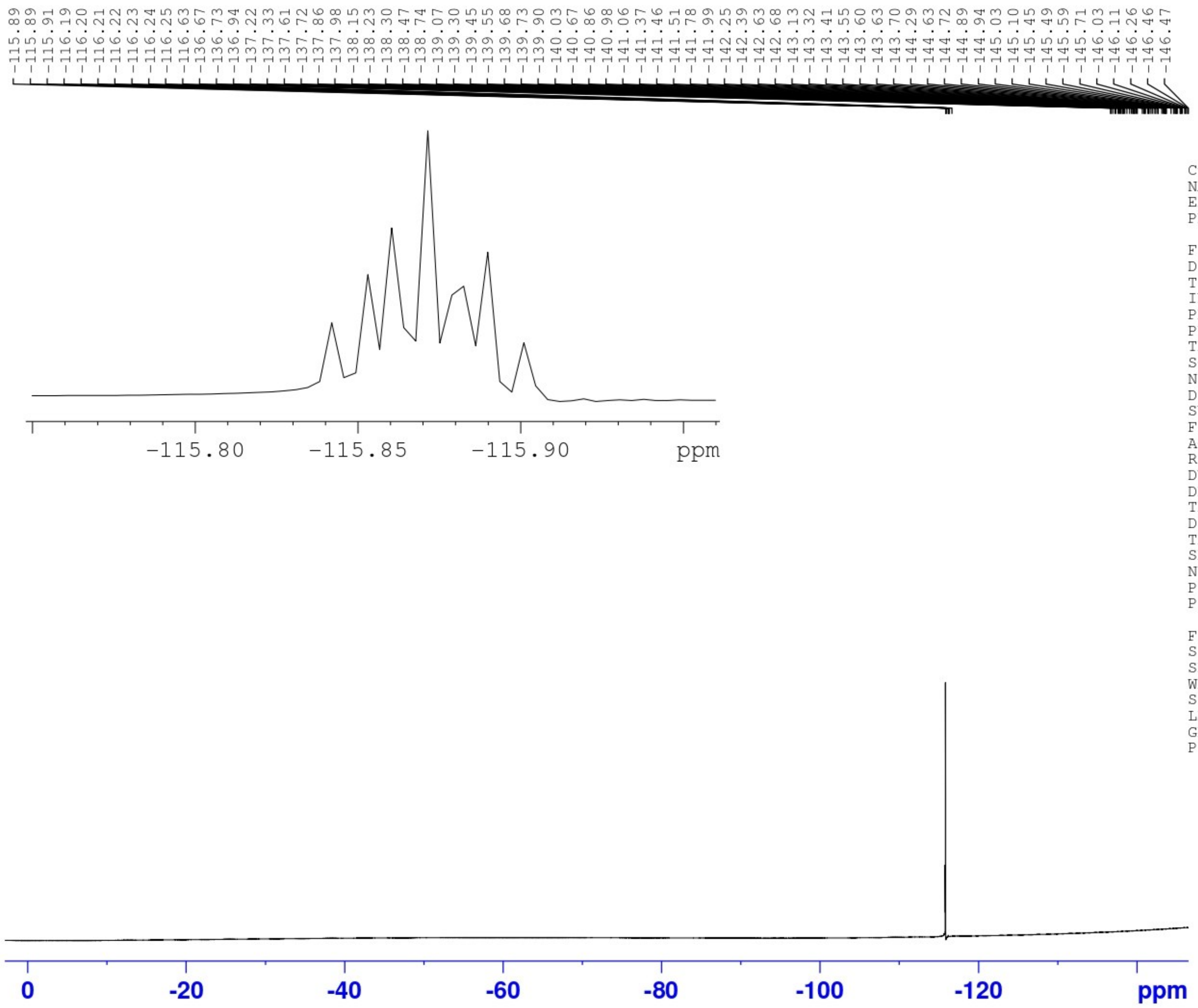

BRUKER

Current Data Parameters

NAME	II-Mn-90 i_21
EXPNO	3
PROCNO	1

F2 - Acquisition Parameters

Date_	20230804
Time	16.38
INSTRUM	AS500-NEO
PROBHD	Z168772_0026 (CPP1.1
PULPROG	zg
TD	131072
SOLVENT	CDCl3
NS	16
DS	4
SWH	113636.364
FIDRES	1.733953
AQ	0.5767168
RG	10
DW	4.400
DE	18.00
TE	300.0
D1	1.00000000
TD0	1
SFO1	470.2188444
NUC1	19F
P1	15.00
PLW1	10.89000034

F2 - Processing parameters

SI	65536
SF	470.2658710
WDW	EM
SSB	0
LB	0.30
GB	0
PC	1.00

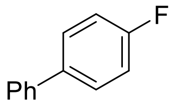

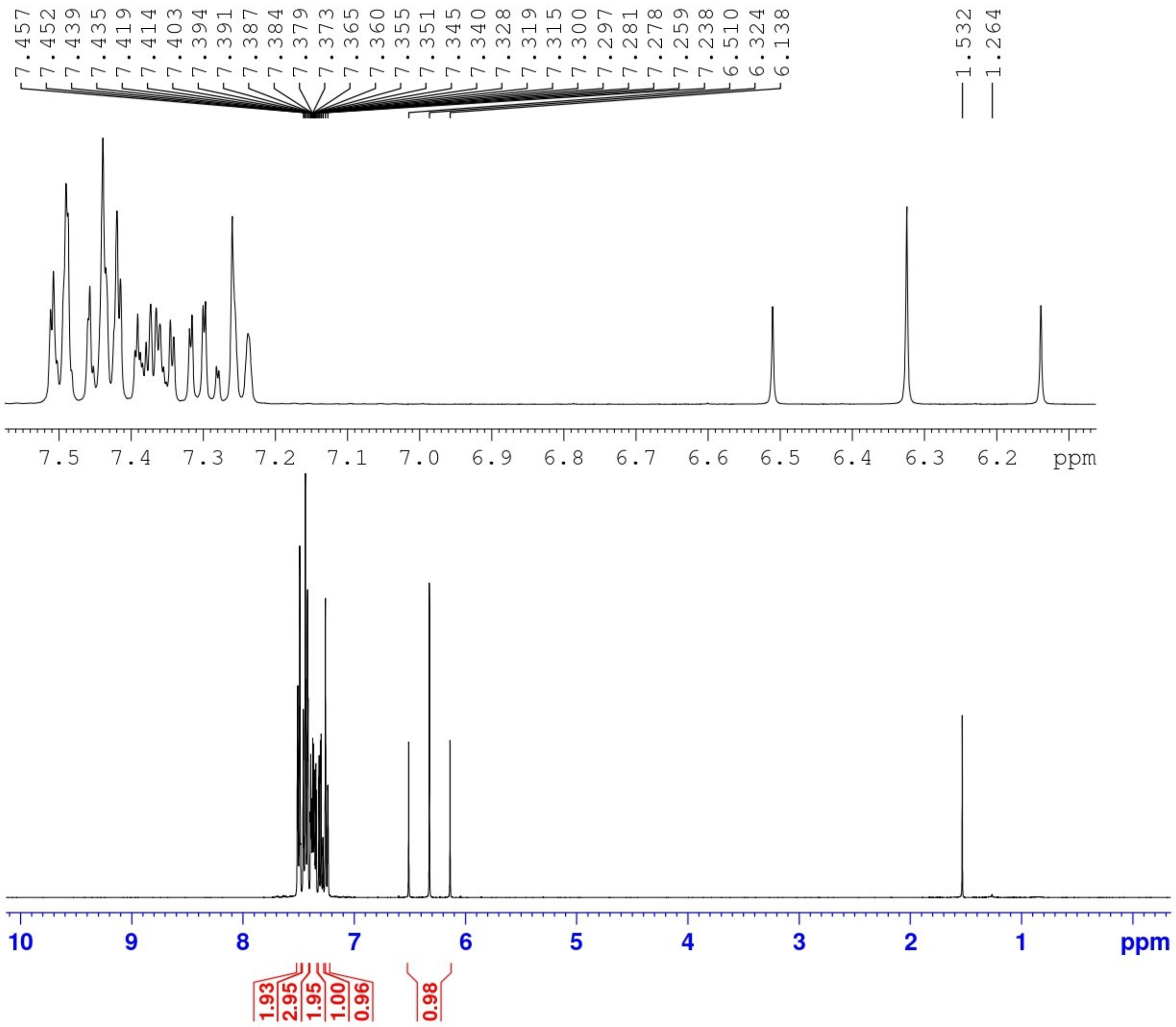

Current Data Parameters
NAME IV-Mn-50 i_10
EXPNO 4
PROCNO 1

F2 - Acquisition Parameters
Date_ 20240109
Time 12.57
INSTRUM Avance
PROBHD Z166552_0018 (PI HR-
PULPROG zg30
TD 65536
SOLVENT CDCl3
NS 16
DS 2
SWH 7812.500
FIDRES 0.238419
AQ 4.1943040
RG 101
DW 64.000
DE 6.67
TE 298.0
D1 1.00000000
TD0 1
SFO1 399.6024675
NUC1 1H
P0 2.60
P1 7.80
PLW1 21.19799995

F2 - Processing parameters
SI 65536
SF 399.6000097
WDW EM
SSB 0
LB 0.30
GB 0
PC 1.00

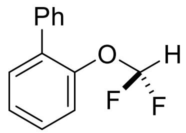

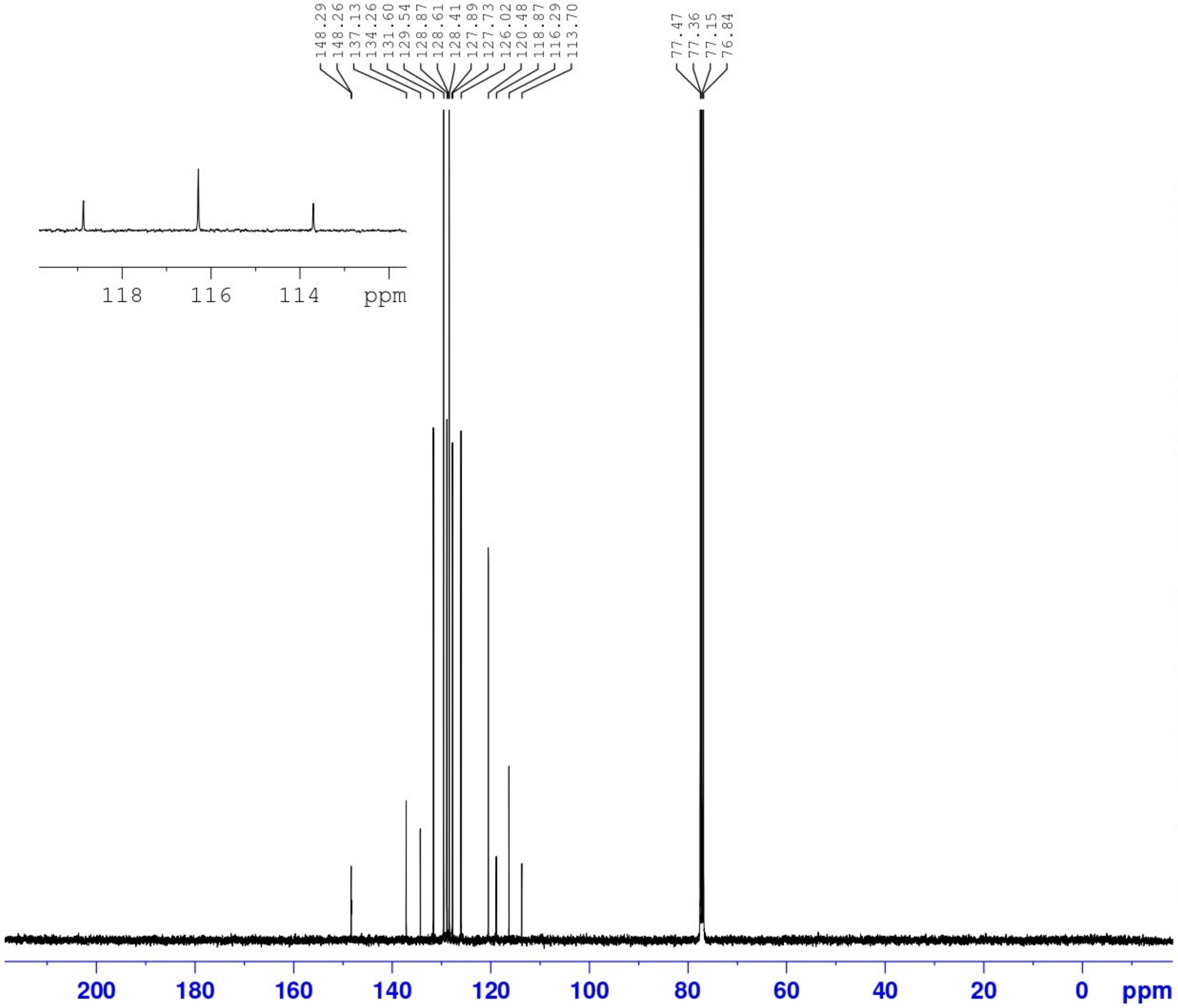

Current Data Parameters
NAME IV-Mn-50 i_12
EXPNO 2
PROCNO 1

F2 - Acquisition Parameters
Date_ 20240109
Time 20.02
INSTRUM Avance
PROBHD Z166552_0018 (PI HR-
PULPROG zgpg30
TD 65536
SOLVENT CDCl3
NS 2048
DS 4
SWH 23809.524
FIDRES 0.726609
AQ 1.3762560
RG 101
DW 21.000
DE 6.50
TE 298.0
D1 2.00000000
D11 0.03000000
TD0 1
SFO1 100.4895479
NUC1 13C
P0 2.67
P1 8.00
PLW1 88.22599792
SFO2 399.6015984
NUC2 1H
CPDPRG[2] waltz65
PCPD2 90.00
PLW2 21.19799995
PLW12 0.15922000
PLW13 0.08008700

F2 - Processing parameters
SI 32768
SF 100.4794872
WDW EM
SSB 0
LB 1.00
GB 0
PC 1.40

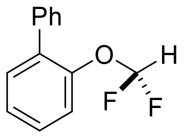

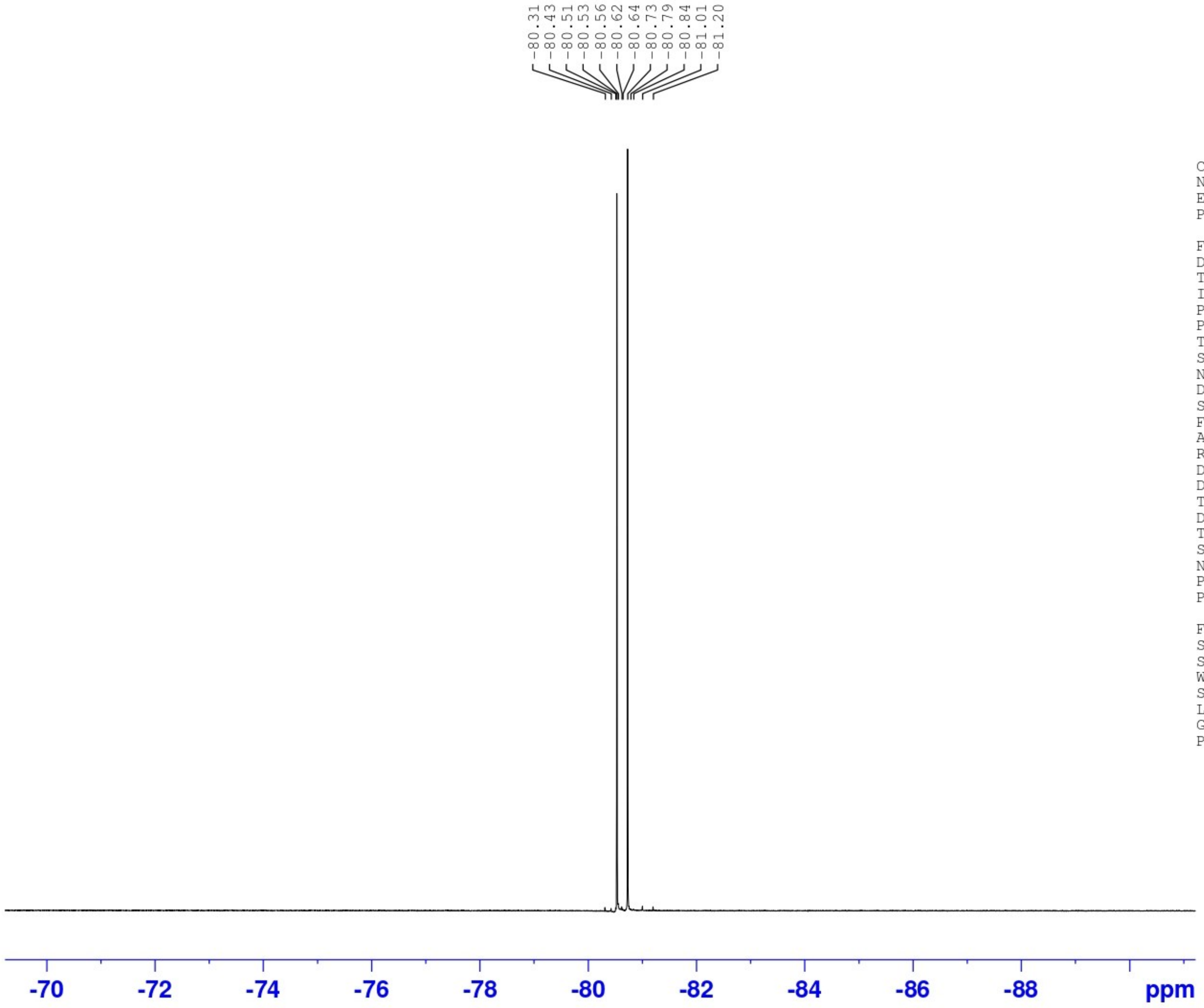

Current Data Parameters
NAME IV-Mn-50 i_11
EXPNO 4
PROCNO 1

F2 - Acquisition Parameters
Date_ 20240109
Time 13.00
INSTRUM Avance
PROBHD Z166552_0018 (PI HR-
PULPROG zg
TD 131072
SOLVENT CDCl3
NS 16
DS 4
SWH 90909.091
FIDRES 1.387163
AQ 0.7208960
RG 101
DW 5.500
DE 6.50
TE 298.0
D1 1.00000000
TD0 1
SFO1 375.9620680
NUC1 19F
P1 12.00
PLW1 32.47200012

F2 - Processing parameters
SI 65536
SF 375.9996680
WDW EM
SSB 0
LB 0.30
GB 0
PC 1.00

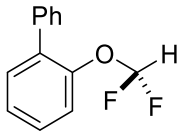

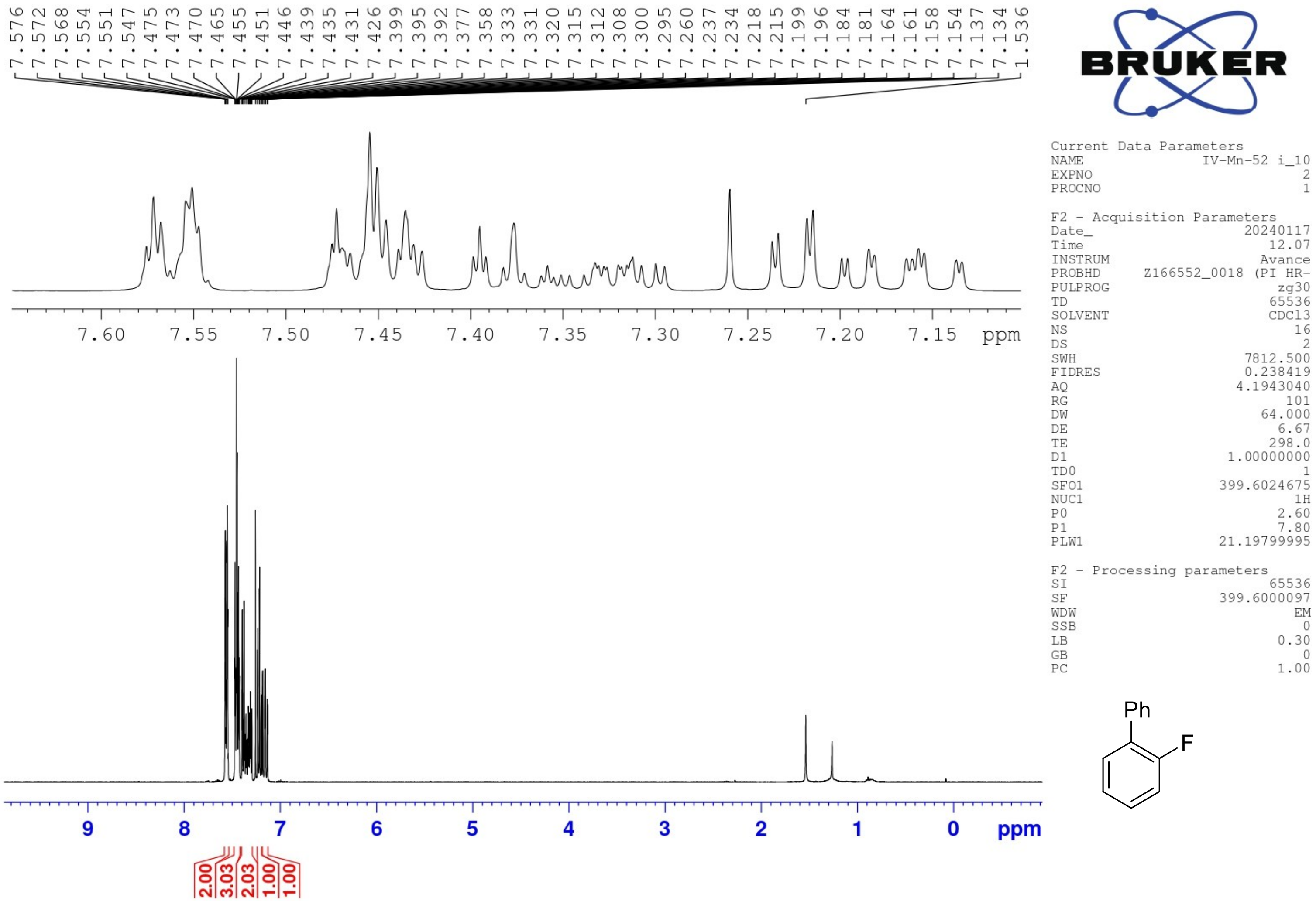
7.576
7.572
7.568
7.554
7.551
7.547
7.475
7.473
7.470
7.465
7.455
7.451
7.446
7.439
7.435
7.431
7.426
7.399
7.395
7.392
7.377
7.358
7.333
7.331
7.320
7.315
7.312
7.308
7.300
7.295
7.260
7.237
7.234
7.218
7.215
7.199
7.196
7.184
7.181
7.164
7.161
7.158
7.154
7.137
7.134
1.536
7.60 7.55 7.50 7.45 7.40 7.35 7.30 7.25 7.20 7.15 ppm
9 8 7 6 5 4 3 2 1 0 ppm
2.00
3.03
2.03
1.00
1.00
BRUKER
Current Data Parameters
NAME IV-Mn-52 i_10
EXPNO 2
PROCNO 1
F2 - Acquisition Parameters
Date_ 20240117
Time 12.07
INSTRUM Avance
PROBHD Z166552_0018 (PI HR-
PULPROG zg30
TD 65536
SOLVENT CDCl3
NS 16
DS 2
SWH 7812.500
FIDRES 0.238419
AQ 4.1943040
RG 101
DW 64.000
DE 6.67
TE 298.0
D1 1.00000000
TD0 1
SFO1 399.6024675
NUC1 1H
P0 2.60
P1 7.80
PLW1 21.19799995
F2 - Processing parameters
SI 65536
SF 399.6000097
WDW EM
SSB 0
LB 0.30
GB 0
PC 1.00
Ph
F

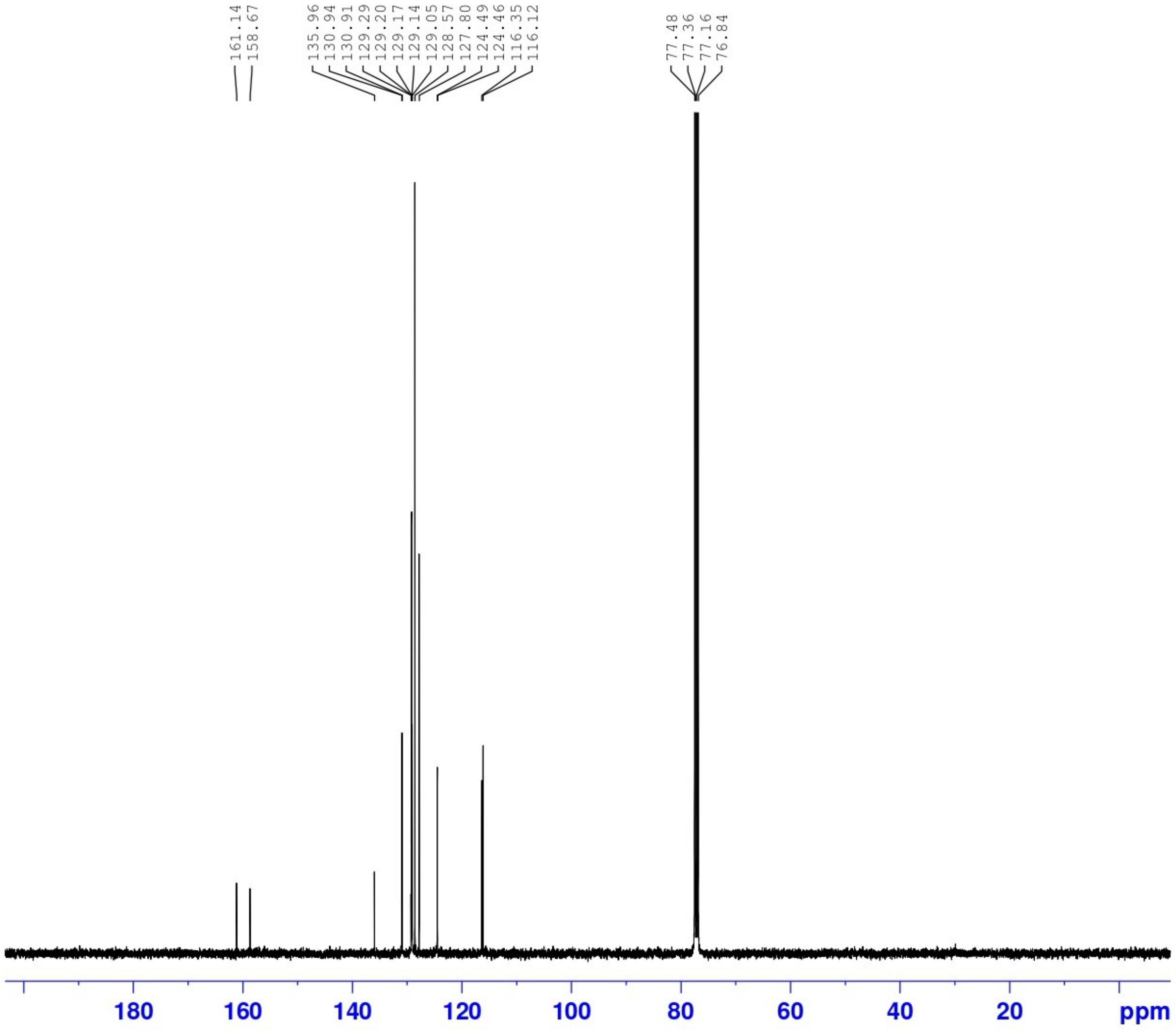

Current Data Parameters
NAME IV-Mn-52 i_12
EXPNO 1
PROCNO 1

F2 - Acquisition Parameters
Date_ 20240117
Time 20.04
INSTRUM Avance
PROBHD Z166552_0018 (PI HR-
PULPROG zgpg30
TD 65536
SOLVENT CDCl3
NS 2048
DS 4
SWH 23809.524
FIDRES 0.726609
AQ 1.3762560
RG 101
DW 21.000
DE 6.50
TE 298.0
D1 2.00000000
D11 0.03000000
TD0 1
SFO1 100.4895479
NUC1 13C
P0 2.67
P1 8.00
PLW1 88.22599792
SFO2 399.6015984
NUC2 1H
CPDPRG[2] waltz65
PCPD2 90.00
PLW2 21.19799995
PLW12 0.15922000
PLW13 0.08008700

F2 - Processing parameters
SI 32768
SF 100.4794866
WDW EM
SSB 0
LB 1.00
GB 0
PC 1.40

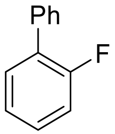

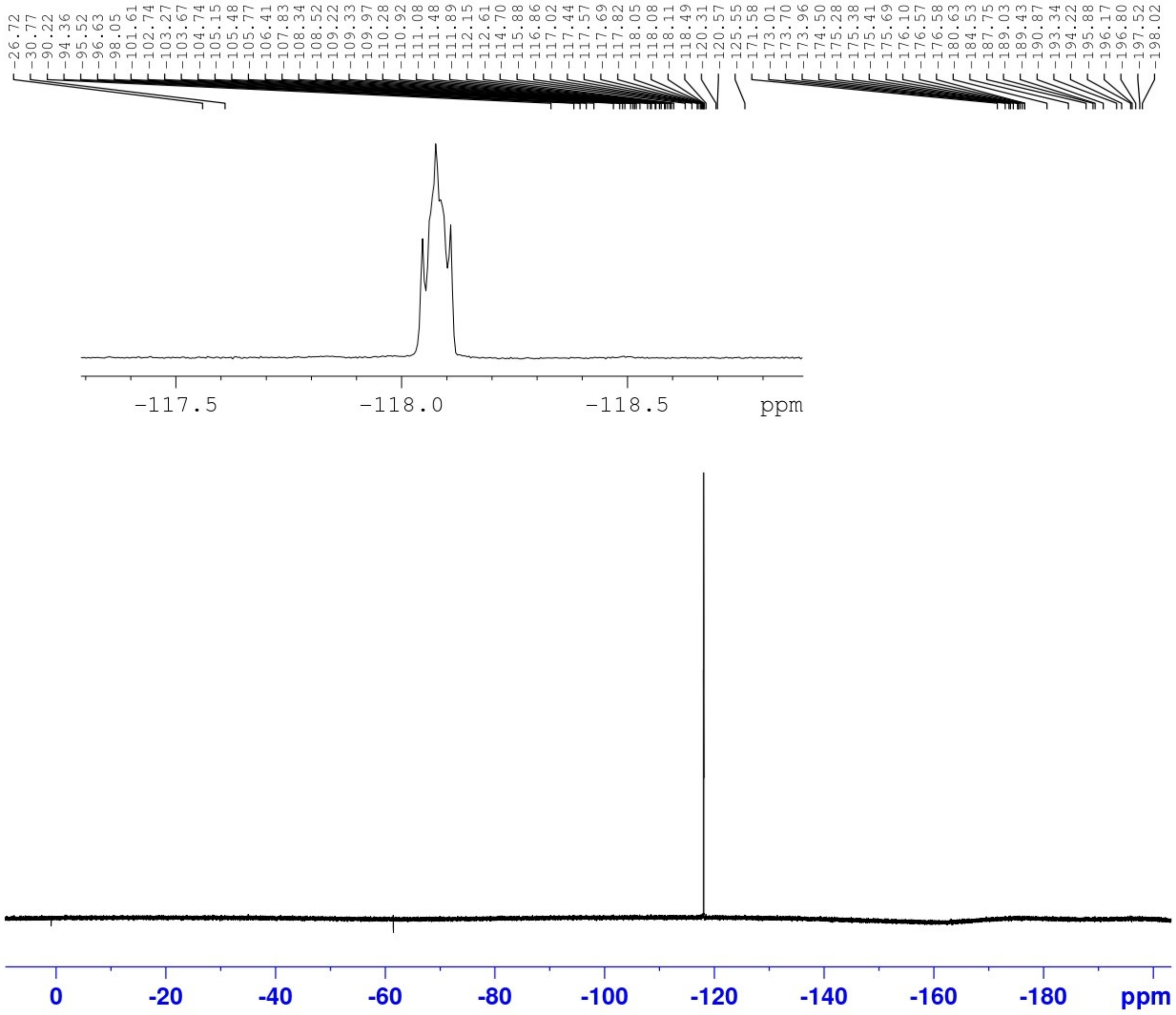

Current Data Parameters

NAME	IV-Mn-52 i_11
EXPNO	2
PROCNO	1

F2 - Acquisition Parameters

Date_	20240117
Time	12.11
INSTRUM	Avance
PROBHD	Z166552_0018 (PI HR-
PULPROG	zg
TD	131072
SOLVENT	CDCl3
NS	16
DS	4
SWH	90909.091
FIDRES	1.387163
AQ	0.7208960
RG	101
DW	5.500
DE	6.50
TE	298.0
D1	1.00000000
TD0	1
SFO1	375.9620680
NUC1	19F
P1	12.00
PLW1	32.47200012

F2 - Processing parameters

SI	65536
SF	375.9996680
WDW	EM
SSB	0
LB	0.30
GB	0
PC	1.00

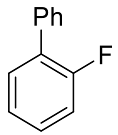

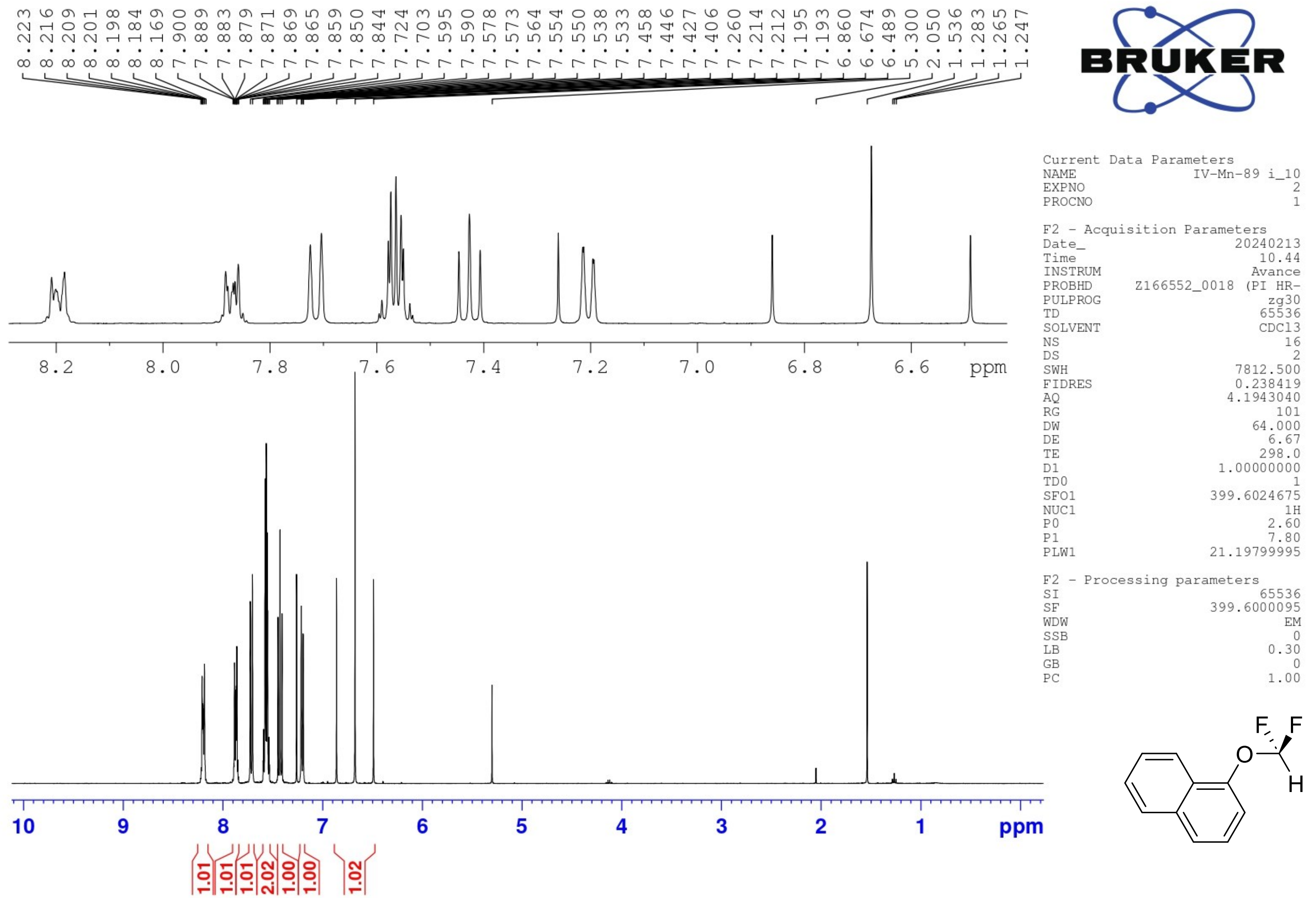
8.223
8.216
8.209
8.201
8.198
8.184
8.169
7.900
7.889
7.883
7.879
7.871
7.869
7.865
7.859
7.850
7.844
7.724
7.703
7.595
7.590
7.578
7.573
7.564
7.554
7.550
7.538
7.533
7.458
7.446
7.427
7.406
7.260
7.214
7.212
7.195
7.193
6.860
6.674
6.489
5.300
2.050
1.536
1.283
1.265
1.247
8.2
8.0
7.8
7.6
7.4
7.2
7.0
6.8
6.6
ppm
10
9
8
7
6
5
4
3
2
1
ppm
1.01
1.01
1.01
2.02
1.00
1.00
1.02
Current Data Parameters
NAME IV-Mn-89 i_10
EXPNO 2
PROCNO 1
F2 - Acquisition Parameters
Date_ 20240213
Time 10.44
INSTRUM Avance
PROBHD Z166552_0018 (PI HR-
PULPROG zg30
TD 65536
SOLVENT CDCl3
NS 16
DS 2
SWH 7812.500
FIDRES 0.238419
AQ 4.1943040
RG 101
DW 64.000
DE 6.67
TE 298.0
D1 1.00000000
TD0 1
SFO1 399.6024675
NUC1 1H
P0 2.60
P1 7.80
PLW1 21.19799995
F2 - Processing parameters
SI 65536
SF 399.6000095
WDW EM
SSB 0
LB 0.30
GB 0
PC 1.00
F
F
O
H

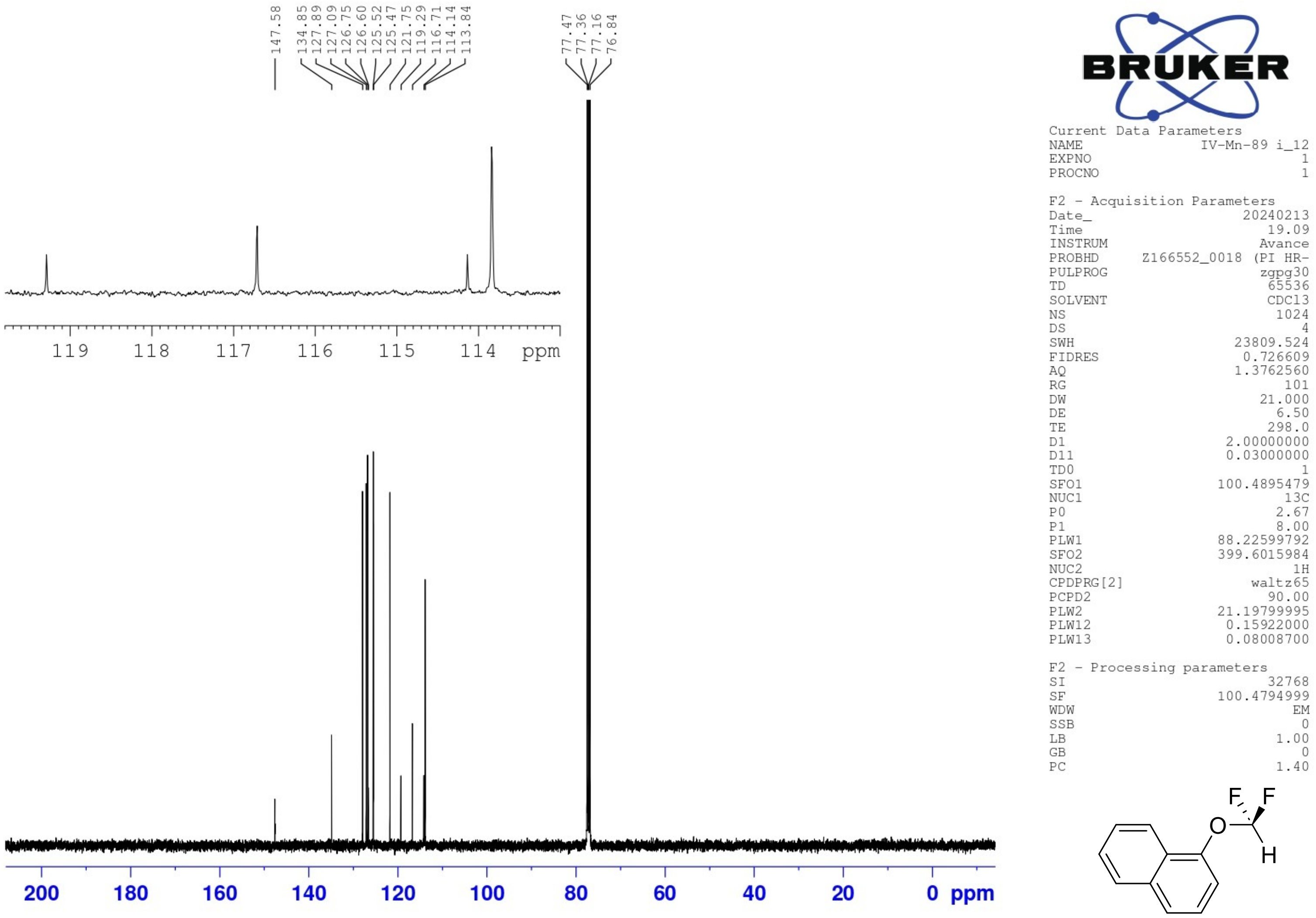
147.58
134.85
127.89
127.09
126.75
126.60
125.52
125.47
121.75
119.29
116.71
114.14
113.84
77.47
77.36
77.16
76.84
119 118 117 116 115 114 ppm
200 180 160 140 120 100 80 60 40 20 0 ppm
Current Data Parameters
NAME IV-Mn-89 i_12
EXPNO 1
PROCNO 1
F2 - Acquisition Parameters
Date_ 20240213
Time 19.09
INSTRUM Avance
PROBHD Z166552_0018 (PI HR-
PULPROG zgpg30
TD 65536
SOLVENT CDCl3
NS 1024
DS 4
SWH 23809.524
FIDRES 0.726609
AQ 1.3762560
RG 101
DW 21.000
DE 6.50
TE 298.0
D1 2.00000000
D11 0.03000000
TD0 1
SFO1 100.4895479
NUC1 13C
P0 2.67
P1 8.00
PLW1 88.22599792
SFO2 399.6015984
NUC2 1H
CPDPRG[2] waltz65
PCPD2 90.00
PLW2 21.19799995
PLW12 0.15922000
PLW13 0.08008700
F2 - Processing parameters
SI 32768
SF 100.4794999
WDW EM
SSB 0
LB 1.00
GB 0
PC 1.40
F
F
O
H

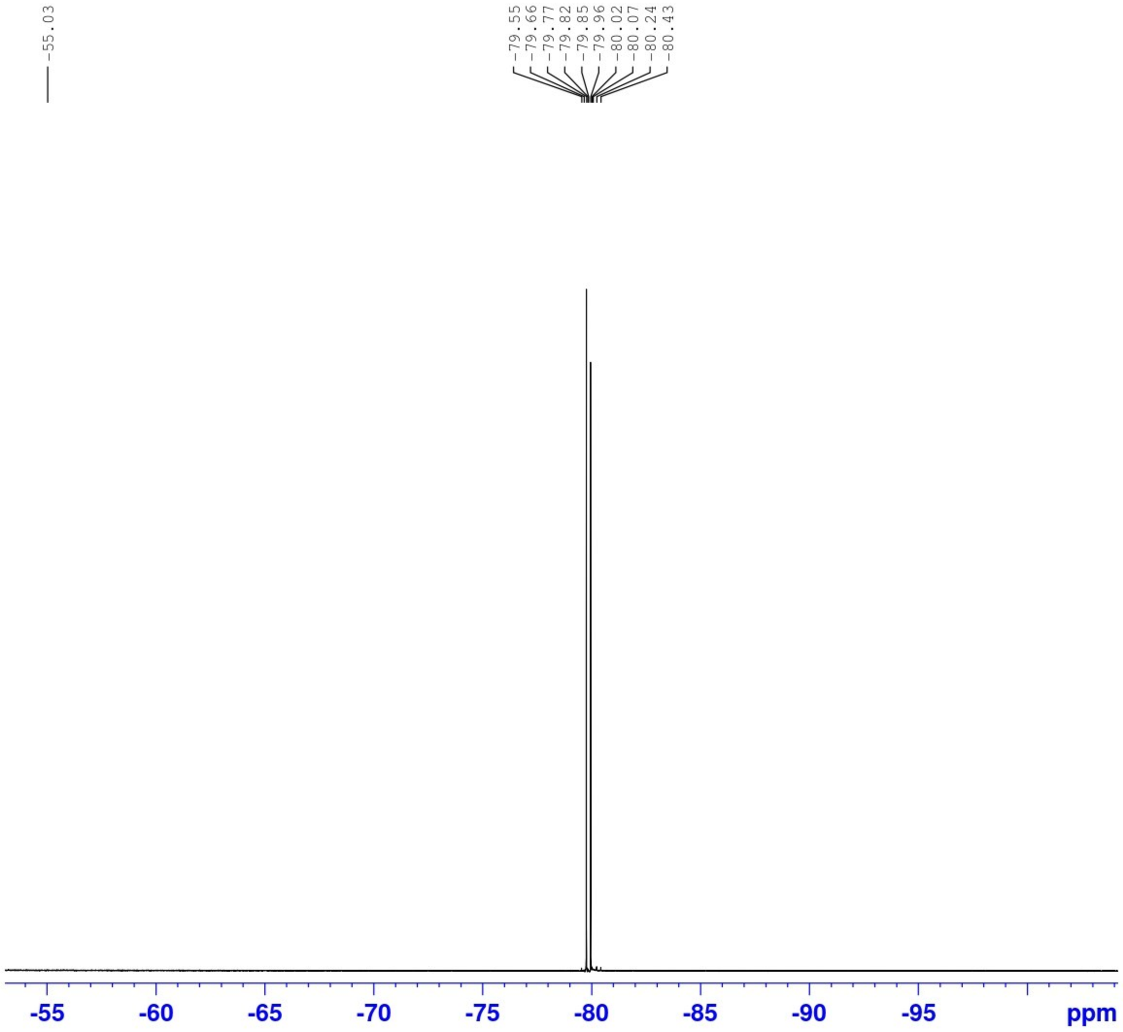

Current Data Parameters	
NAME	IV-Mn-89 i_11
EXPNO	2
PROCNO	1
F2 - Acquisition Parameters	
Date_	20240213
Time	10.46
INSTRUM	Avance
PROBHD	Z166552_0018 (PI HR-
PULPROG	zg
TD	131072
SOLVENT	CDCl3
NS	16
DS	4
SWH	90909.091
FIDRES	1.387163
AQ	0.7208960
RG	101
DW	5.500
DE	6.50
TE	298.0
D1	1.00000000
TD0	1
SFO1	375.9620680
NUC1	19F
P1	12.00
PLW1	32.47200012
F2 - Processing parameters	
SI	65536
SF	375.9996680
WDW	EM
SSB	0
LB	0.30
GB	0
PC	1.00

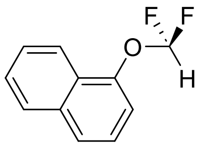

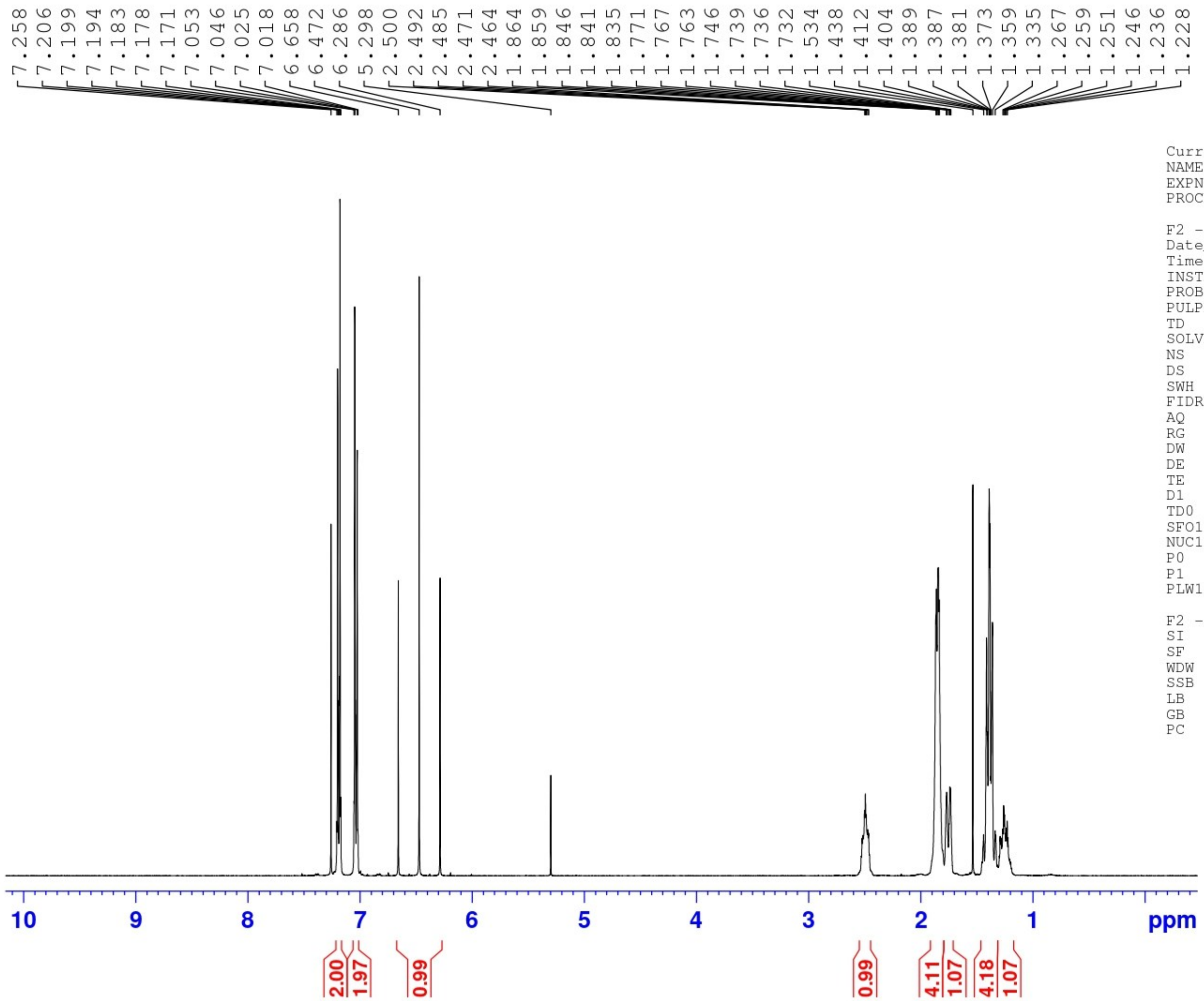

Current Data Parameters
NAME IV-Mn-99 i_10
EXPNO 2
PROCNO 1

F2 - Acquisition Parameters
Date_ 20240221
Time 14.53
INSTRUM Avance
PROBHD Z166552_0018 (PI HR-
PULPROG zg30
TD 65536
SOLVENT CDCl3
NS 16
DS 2
SWH 7812.500
FIDRES 0.238419
AQ 4.1943040
RG 101
DW 64.000
DE 6.67
TE 298.0
D1 1.00000000
TD0 1
SFO1 399.6024675
NUC1 1H
P0 2.60
P1 7.80
PLW1 21.19799995

F2 - Processing parameters
SI 65536
SF 399.6000103
WDW EM
SSB 0
LB 0.30
GB 0
PC 1.00

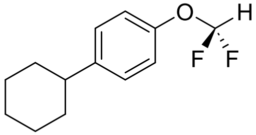

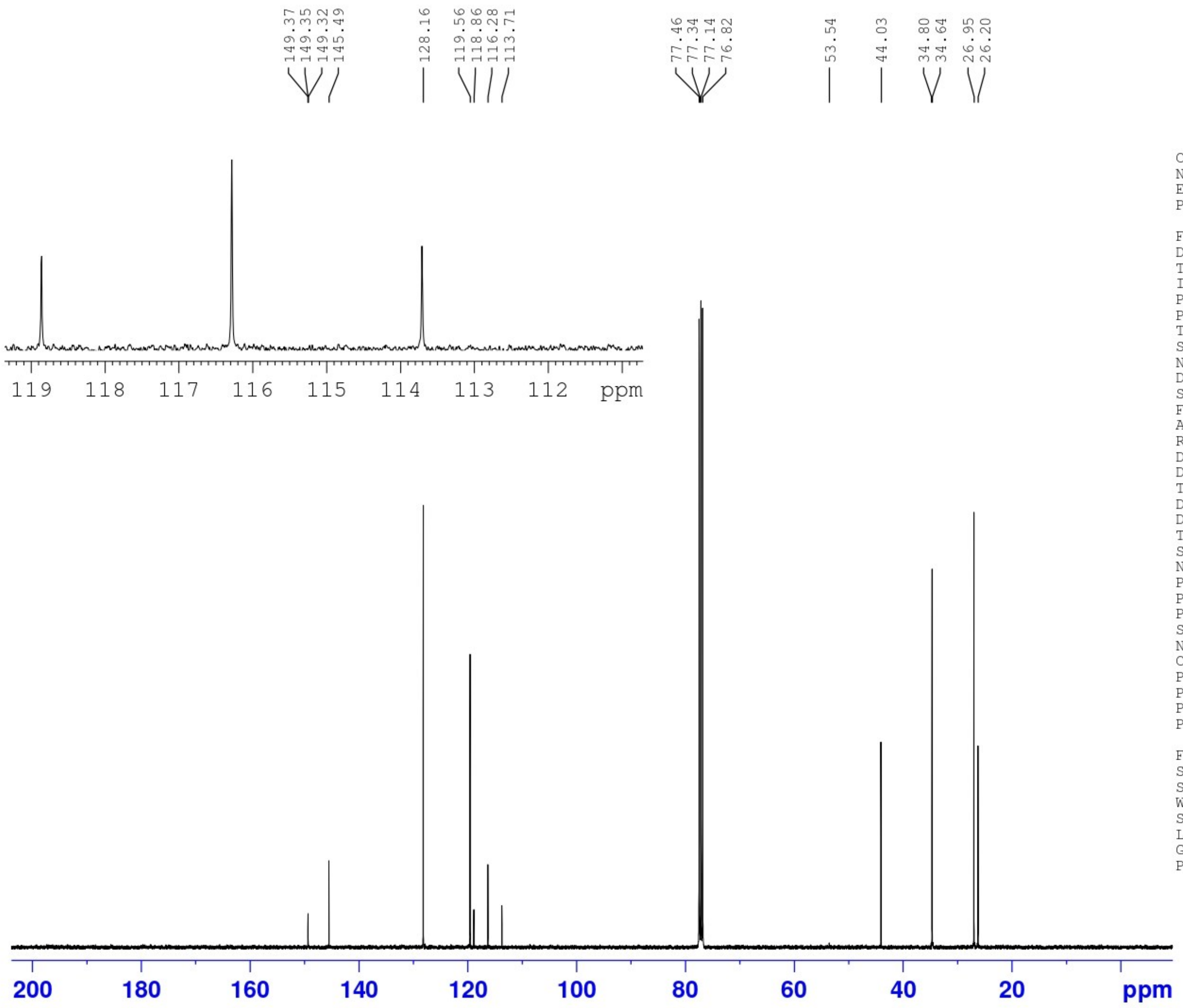

Current Data Parameters
NAME IV-Mn-99 i_12
EXPNO 2
PROCNO 1

F2 - Acquisition Parameters

Date_	20240222
Time	1.23
INSTRUM	Avance
PROBHD	Z166552_0018 (PI HR-
PULPROG	zgpg30
TD	65536
SOLVENT	CDCl3
NS	2048
DS	4
SWH	23809.524
FIDRES	0.726609
AQ	1.3762560
RG	101
DW	21.000
DE	6.50
TE	298.0
D1	2.00000000
D11	0.03000000
TD0	1
SFO1	100.4895479
NUC1	13C
P0	2.67
P1	8.00
PLW1	88.22599792
SFO2	399.6015984
NUC2	1H
CPDPRG[2]	waltz65
PCPD2	90.00
PLW2	21.19799995
PLW12	0.15922000
PLW13	0.08008700

F2 - Processing parameters

SI	32768
SF	100.4794881
WDW	EM
SSB	0
LB	1.00
GB	0
PC	1.40

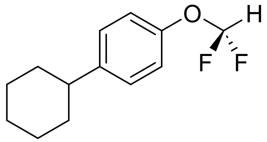

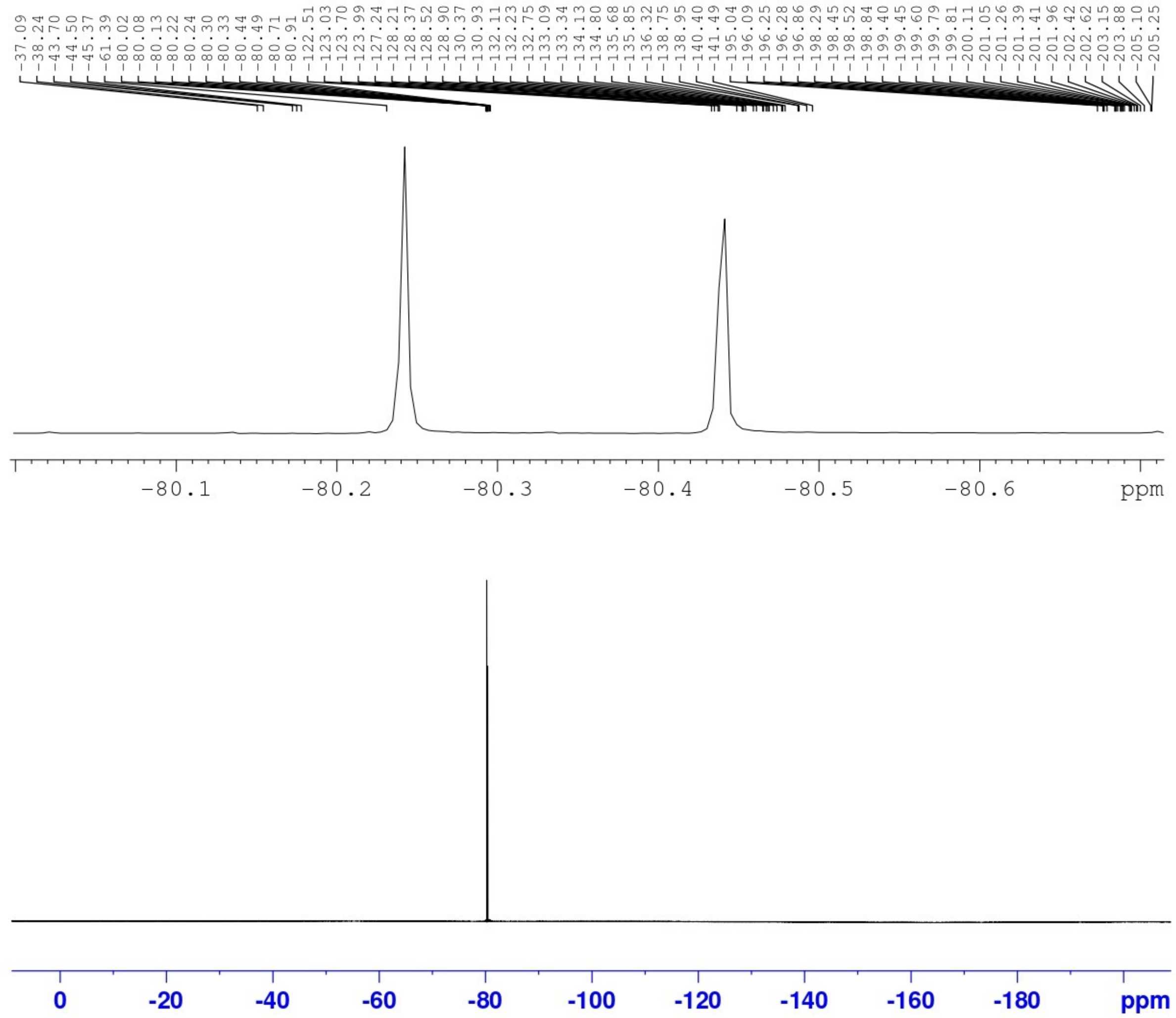

Current Data Parameters
NAME IV-Mn-99 i_11
EXPNO 2
PROCNO 1

F2 - Acquisition Parameters
Date_ 20240221
Time 14.55
INSTRUM Avance
PROBHD Z166552_0018 (PI HR-
PULPROG zg
TD 131072
SOLVENT CDCl3
NS 16
DS 4
SWH 90909.091
FIDRES 1.387163
AQ 0.7208960
RG 101
DW 5.500
DE 6.50
TE 298.0
D1 1.00000000
TD0 1
SFO1 375.9620680
NUC1 19F
P1 12.00
PLW1 32.47200012

F2 - Processing parameters
SI 65536
SF 375.9996680
WDW EM
SSB 0
LB 0.30
GB 0
PC 1.00

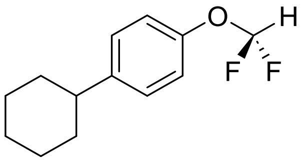

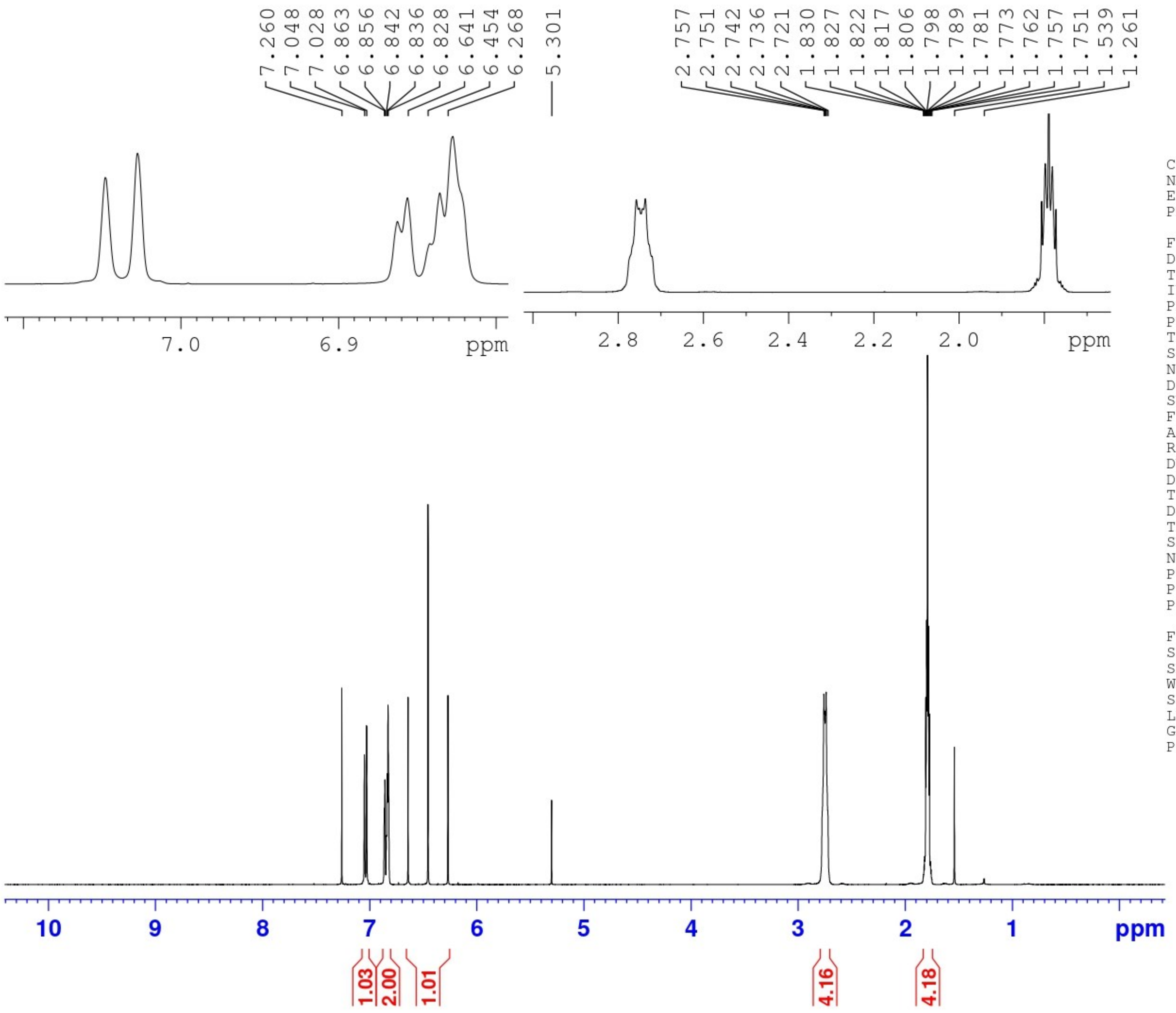
7.260
7.048
7.028
6.863
6.856
6.842
6.836
6.828
6.641
6.454
6.268
5.301
2.757
2.751
2.742
2.736
2.721
1.830
1.827
1.822
1.817
1.806
1.798
1.789
1.781
1.773
1.762
1.757
1.751
1.539
1.261
BRUKER
Current Data Parameters
NAME IV-Mn-98 i_10
EXPNO 3
PROCNO 1
F2 - Acquisition Parameters
Date_ 20240221
Time 14.45
INSTRUM Avance
PROBHD Z166552_0018 (PI HR-
PULPROG zg30
TD 65536
SOLVENT CDCl3
NS 16
DS 2
SWH 7812.500
FIDRES 0.238419
AQ 4.1943040
RG 101
DW 64.000
DE 6.67
TE 298.0
D1 1.00000000
TD0 1
SFO1 399.6024675
NUC1 1H
P0 2.60
P1 7.80
PLW1 21.19799995
F2 - Processing parameters
SI 65536
SF 399.6000097
WDW EM
SSB 0
LB 0.30
GB 0
PC 1.00
7.0
6.9
ppm
2.8
2.6
2.4
2.2
2.0
ppm
10
9
8
7
6
5
4
3
2
1
ppm
1.03
2.00
1.01
4.16
4.18

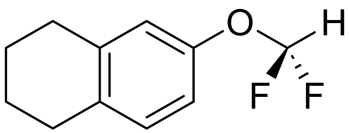
O
H
F
F

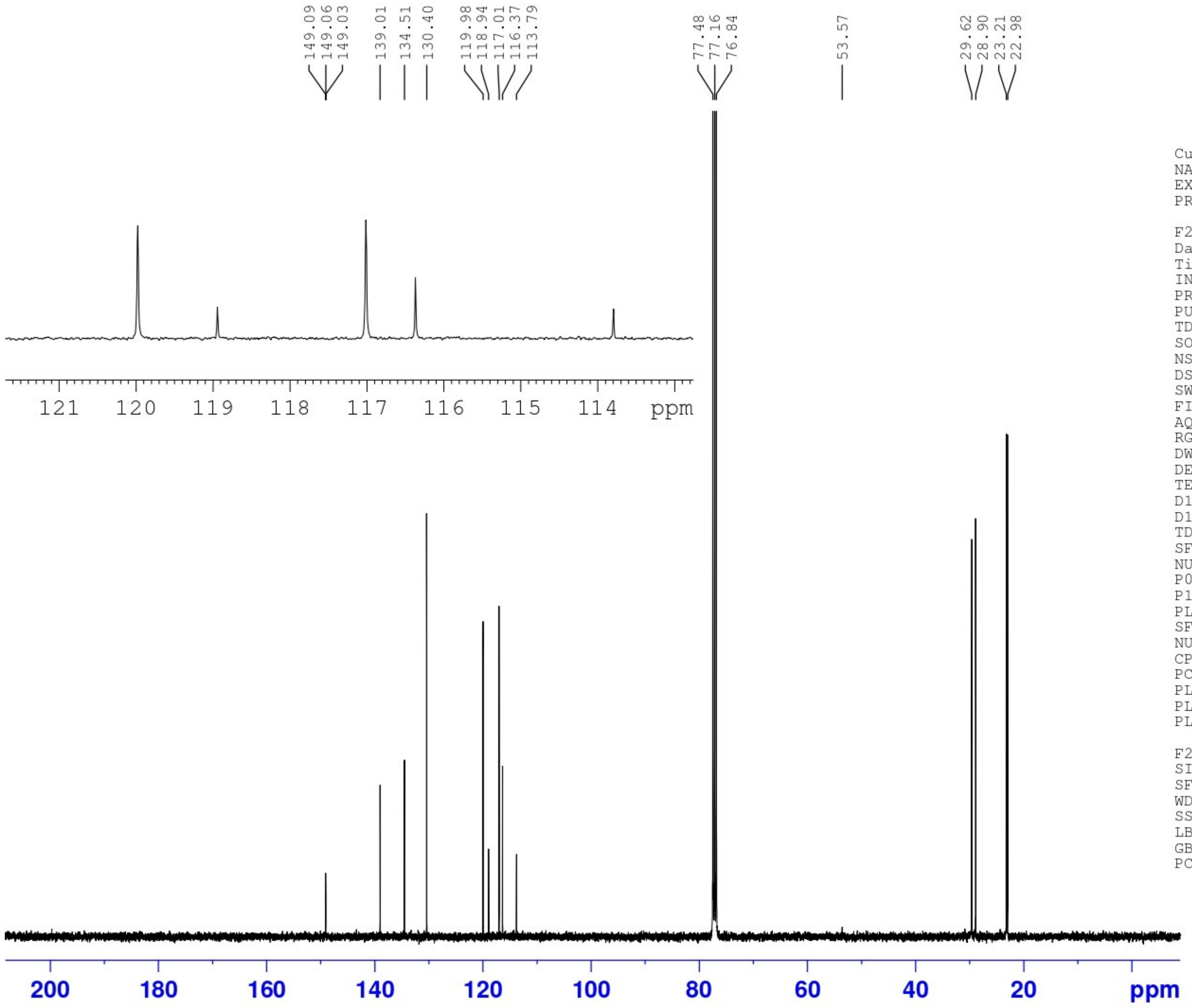

Current Data Parameters

NAME	IV-Mn-98 i_12
EXPNO	3
PROCNO	1

F2 - Acquisition Parameters

Date_	20240221
Time	23.21
INSTRUM	Avance
PROBHD	Z166552_0018 (PI HR-
PULPROG	zgpg30
TD	65536
SOLVENT	CDCl3
NS	2048
DS	4
SWH	23809.524
FIDRES	0.726609
AQ	1.3762560
RG	101
DW	21.000
DE	6.50
TE	298.0
D1	2.00000000
D11	0.03000000
TD0	1
SFO1	100.4895479
NUC1	13C
P0	2.67
P1	8.00
PLW1	88.22599792
SFO2	399.6015984
NUC2	1H
CPDPRG[2]	waltz65
PCPD2	90.00
PLW2	21.19799995
PLW12	0.15922000
PLW13	0.08008700

F2 - Processing parameters

SI	32768
SF	100.4794860
WDW	EM
SSB	0
LB	1.00
GB	0
PC	1.40

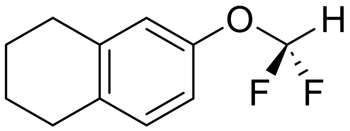

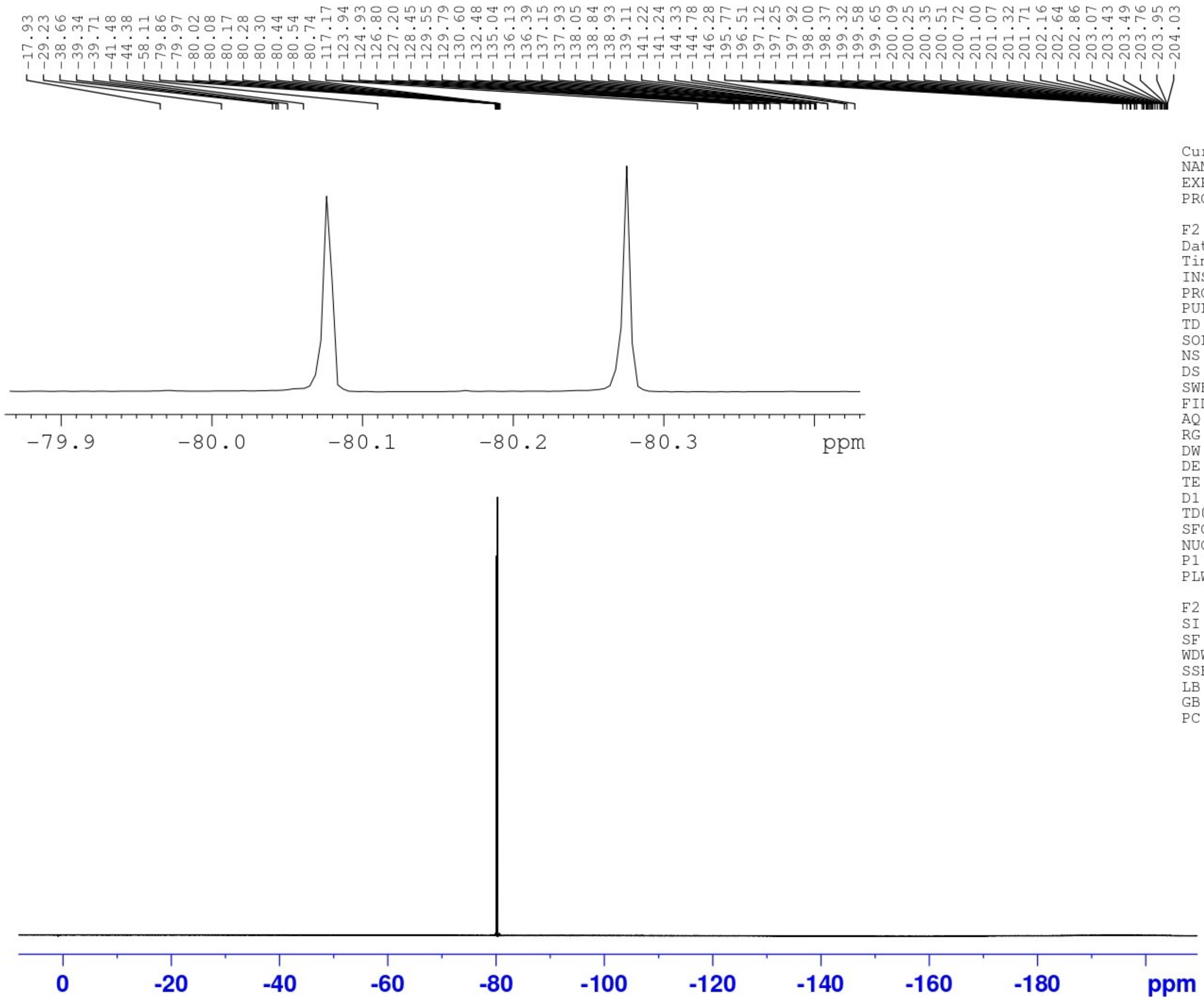

Current Data Parameters
NAME IV-Mn-98 i_11
EXPNO 3
PROCNO 1

F2 - Acquisition Parameters
Date_ 20240221
Time 14.47
INSTRUM Avance
PROBHD Z166552_0018 (PI HR-
PULPROG zg
TD 131072
SOLVENT CDCl3
NS 16
DS 4
SWH 90909.091
FIDRES 1.387163
AQ 0.7208960
RG 101
DW 5.500
DE 6.50
TE 298.0
D1 1.00000000
TD0 1
SFO1 375.9620680
NUC1 19F
P1 12.00
PLW1 32.47200012

F2 - Processing parameters
SI 65536
SF 375.9996680
WDW EM
SSB 0
LB 0.30
GB 0
PC 1.00

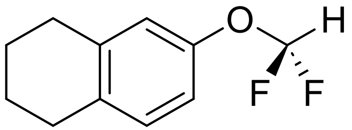

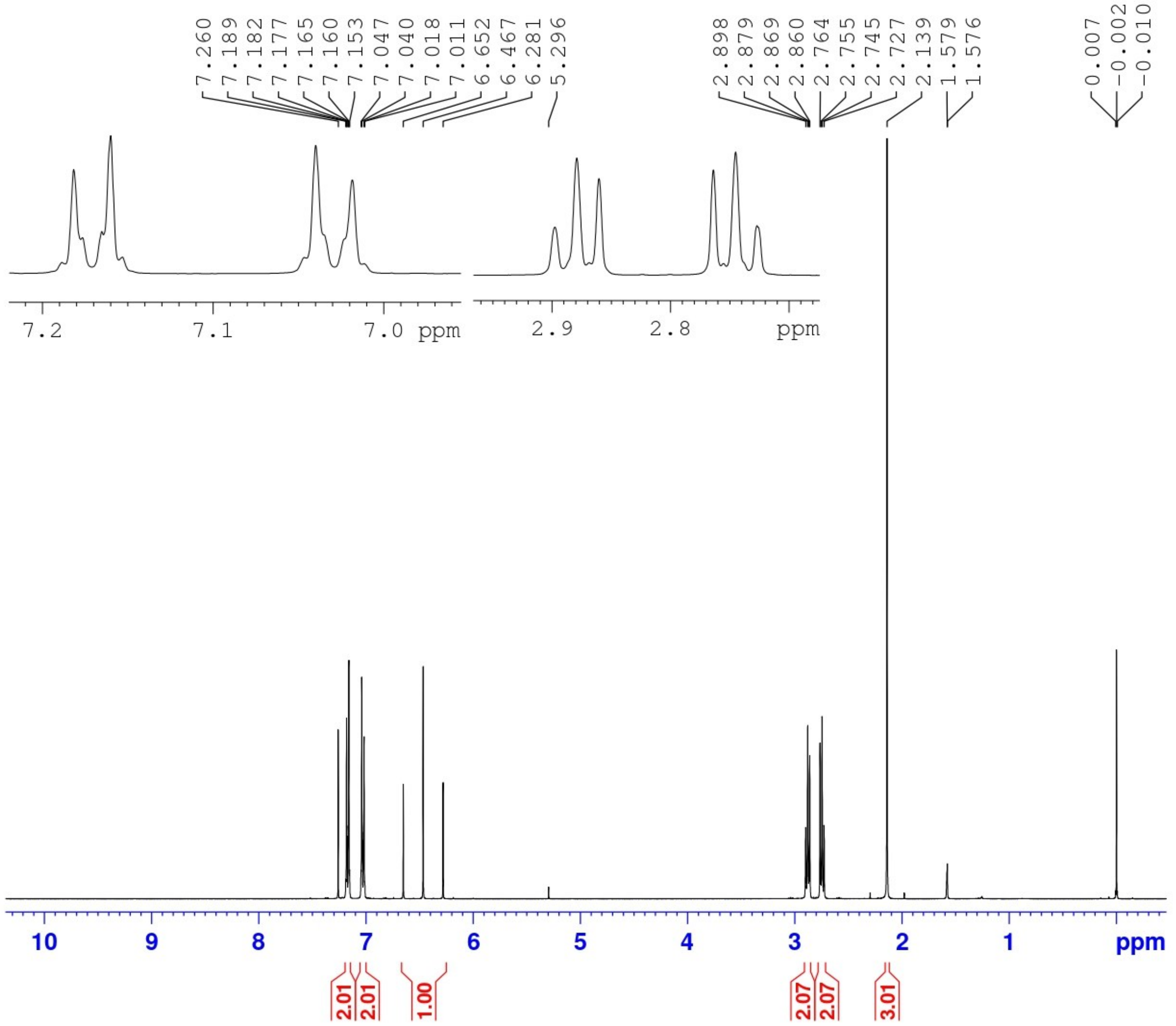

Current Data Parameters
NAME IV-Mn-127 i_10
EXPNO 3
PROCNO 1

F2 - Acquisition Parameters
Date_ 20240411
Time 13.46
INSTRUM Avance
PROBHD Z166552_0018 (PI HR-
PULPROG zg30
TD 65536
SOLVENT CDCl3
NS 16
DS 2
SWH 7812.500
FIDRES 0.238419
AQ 4.1943040
RG 101
DW 64.000
DE 6.67
TE 298.0
D1 1.00000000
TD0 1
SFO1 399.6024675
NUC1 1H
P0 2.60
P1 7.80
PLW1 21.19799995

F2 - Processing parameters
SI 65536
SF 399.6000097
WDW EM
SSB 0
LB 0.30
GB 0
PC 1.00

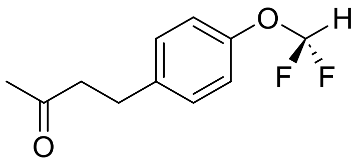

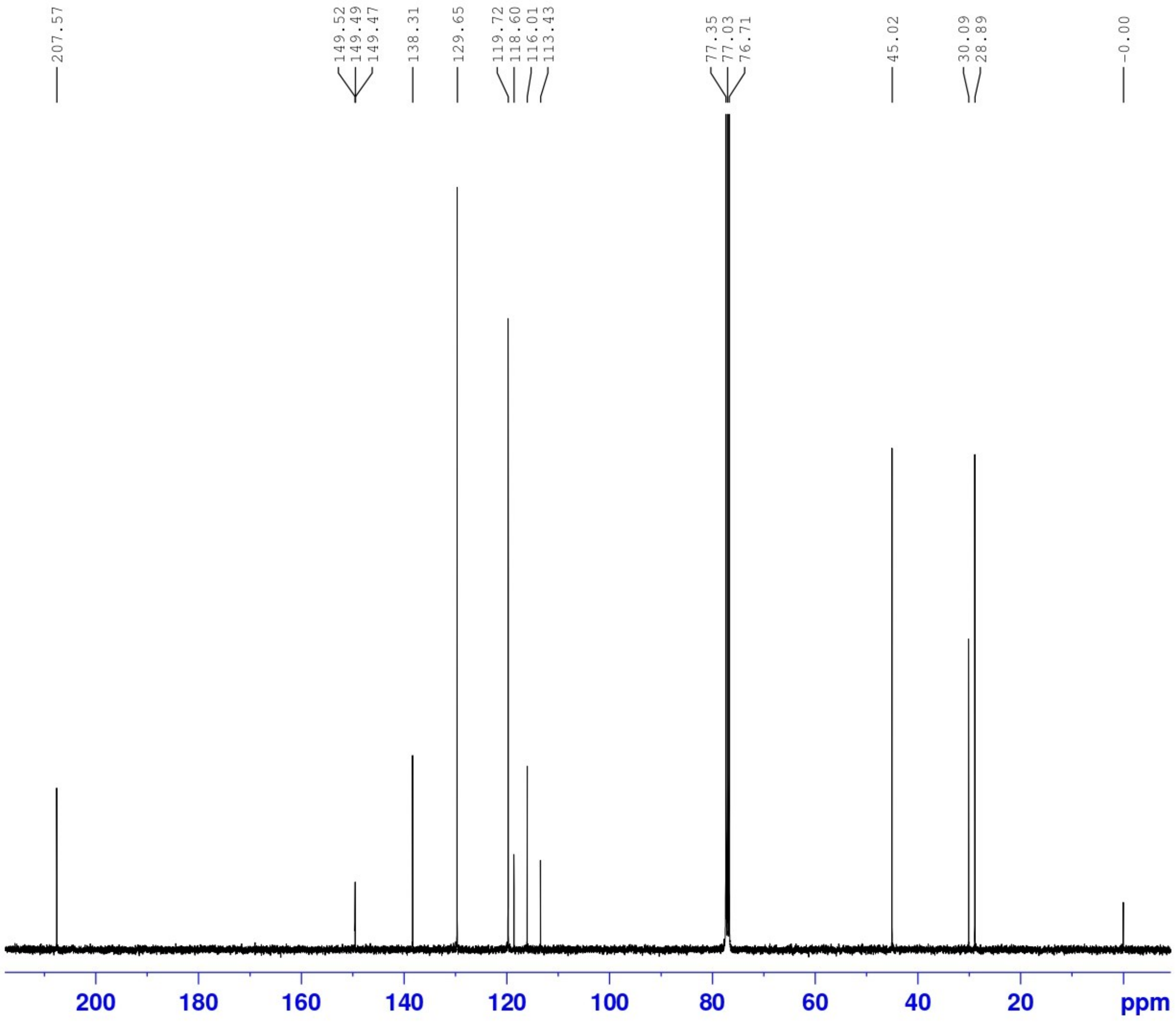
207.57
149.52
149.49
149.47
138.31
129.65
119.72
118.60
116.01
113.43
77.35
77.03
76.71
45.02
30.09
28.89
-0.00
200
180
160
140
120
100
80
60
40
20
ppm
BRUKER
Current Data Parameters
NAME IV-Mn-127 i_12
EXPNO 2
PROCNO 1
F2 - Acquisition Parameters
Date_ 20240411
Time 23.10
INSTRUM Avance
PROBHD Z166552_0018 (PI HR-
PULPROG zgpg30
TD 65536
SOLVENT CDCl3
NS 2048
DS 4
SWH 23809.524
FIDRES 0.726609
AQ 1.3762560
RG 101
DW 21.000
DE 6.50
TE 298.0
D1 2.00000000
D11 0.03000000
TD0 1
SFO1 100.4895479
NUC1 13C
P0 2.67
P1 8.00
PLW1 88.22599792
SFO2 399.6015984
NUC2 1H
CPDPRG[2] waltz65
PCPD2 90.00
PLW2 21.19799995
PLW12 0.15922000
PLW13 0.08008700
F2 - Processing parameters
SI 32768
SF 100.4794998
WDW EM
SSB 0
LB 1.00
GB 0
PC 1.40

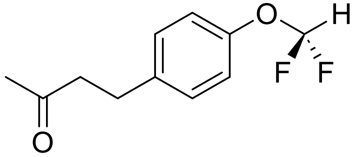
O
H
F
F
O

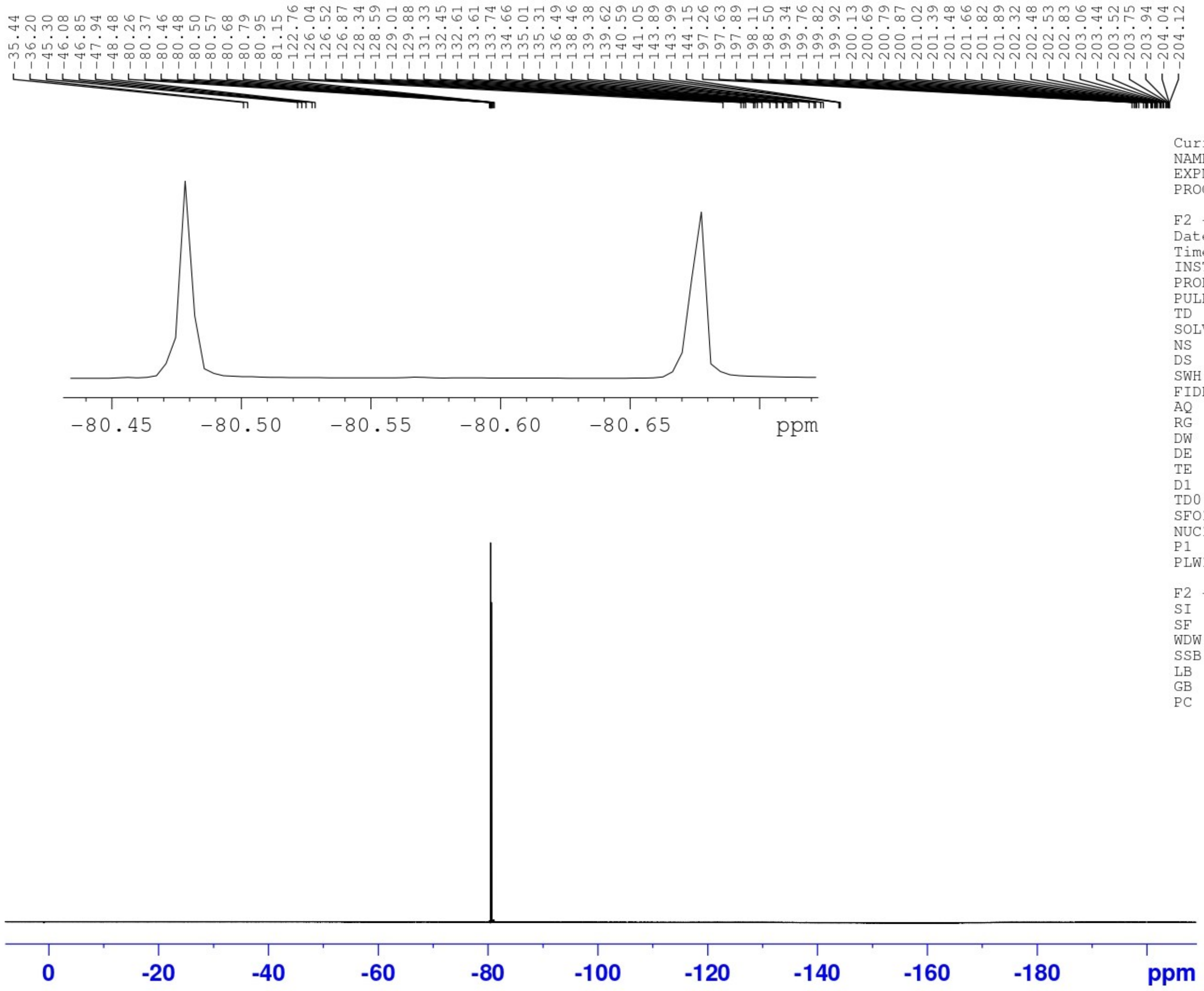

BRUKER

Current Data Parameters
NAME IV-Mn-127 i_11
EXPNO 2
PROCNO 1

F2 - Acquisition Parameters
Date_ 20240411
Time 13.48
INSTRUM Avance
PROBHD Z166552_0018 (PI HR-
PULPROG zg
TD 131072
SOLVENT CDCl3
NS 16
DS 4
SWH 90909.091
FIDRES 1.387163
AQ 0.7208960
RG 101
DW 5.500
DE 6.50
TE 298.0
D1 1.00000000
TD0 1
SFO1 375.9620680
NUC1 19F
P1 12.00
PLW1 32.47200012

F2 - Processing parameters
SI 65536
SF 375.9996680
WDW EM
SSB 0
LB 0.30
GB 0
PC 1.00

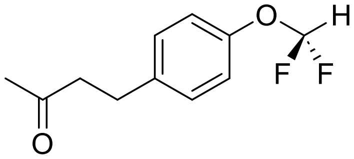

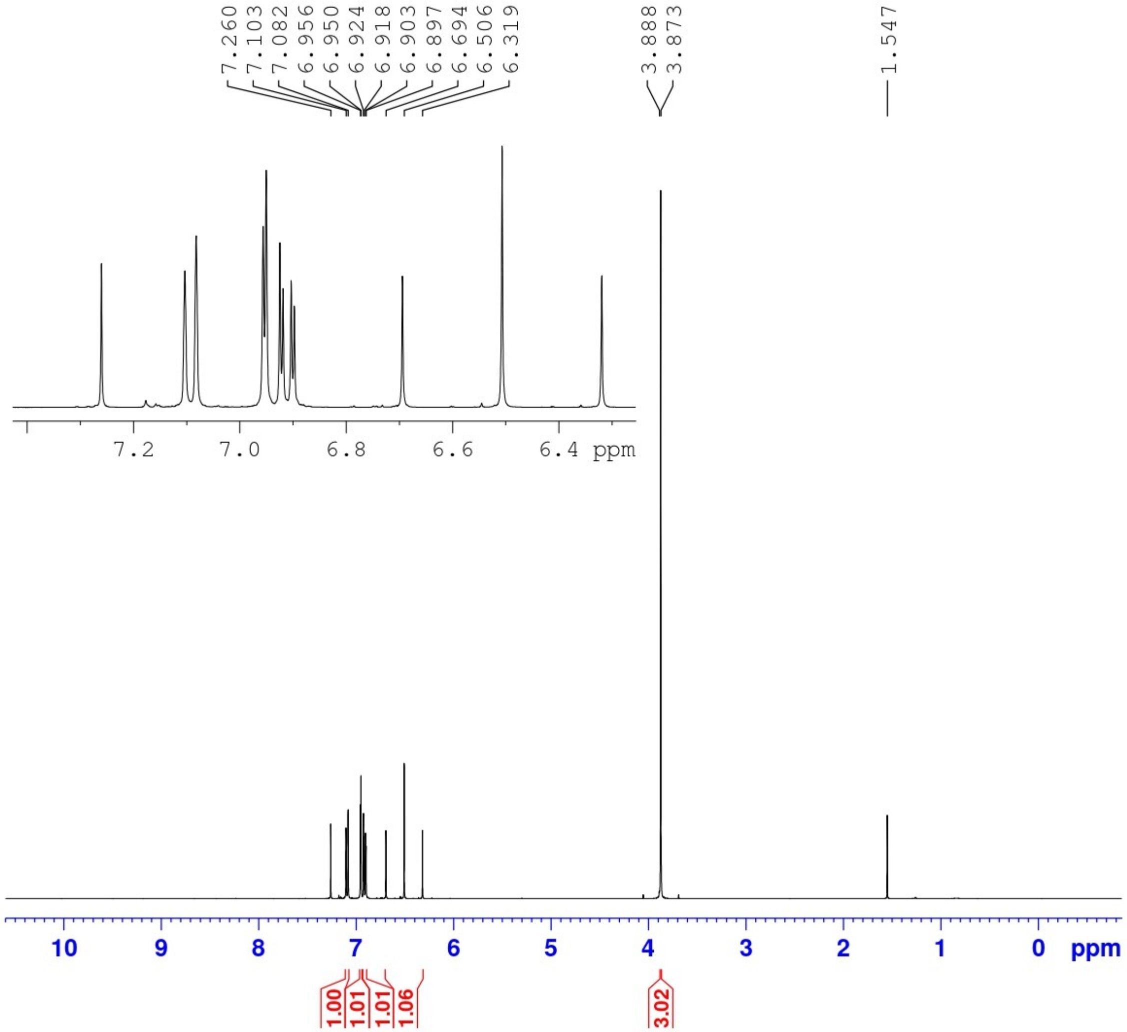

Current Data Parameters
NAME VII-Mn-26 i_10
EXPNO 1
PROCNO 1

F2 - Acquisition Parameters
Date_ 20241113
Time 17.39
INSTRUM Avance
PROBHD Z166552_0018 (PI HR-
PULPROG zg30
TD 65536
SOLVENT CDCl3
NS 16
DS 2
SWH 7812.500
FIDRES 0.238419
AQ 4.1943040
RG 101
DW 64.000
DE 6.67
TE 298.0
D1 1.00000000
TD0 1
SFO1 399.5701703
NUC1 1H
P0 2.60
P1 7.80
PLW1 21.19799995

F2 - Processing parameters
SI 65536
SF 399.5677126
WDW EM
SSB 0
LB 0.30
GB 0
PC 1.00

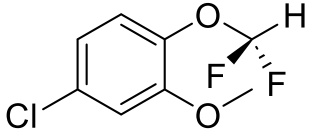

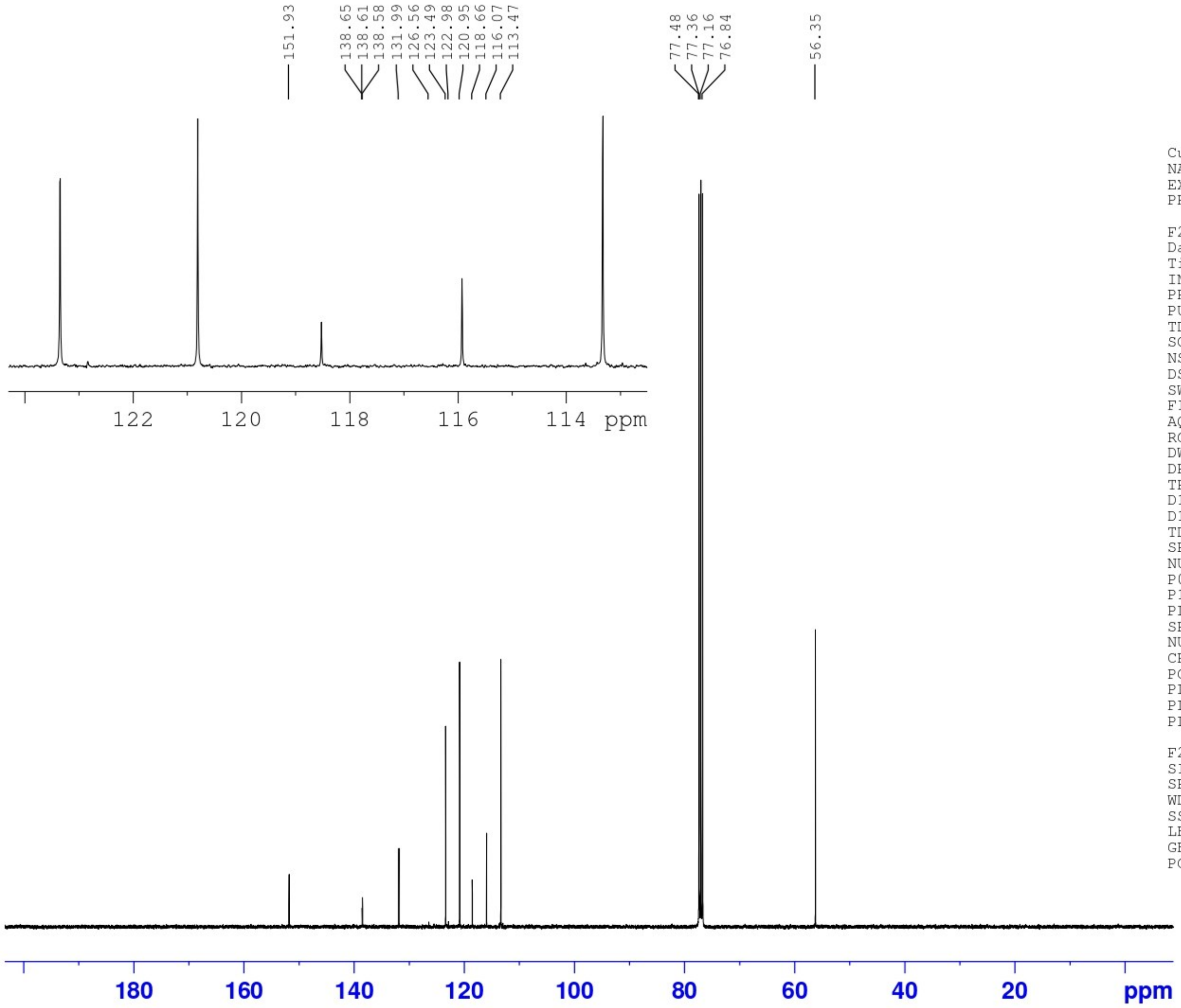

BRUKER

Current Data Parameters
NAME VII-Mn-26 i_12
EXPNO 1
PROCNO 1

F2 - Acquisition Parameters
Date_ 20241113
Time 21.12
INSTRUM Avance
PROBHD Z166552_0018 (PI HR-
PULPROG zgpg30
TD 65536
SOLVENT CDCl3
NS 2048
DS 4
SWH 23809.524
FIDRES 0.726609
AQ 1.3762560
RG 101
DW 21.000
DE 6.50
TE 298.0
D1 2.00000000
D11 0.03000000
TD0 1
SFO1 100.4814260
NUC1 13C
P0 2.67
P1 8.00
PLW1 88.22599792
SFO2 399.5693013
NUC2 1H
CPDPRG[2] waltz65
PCPD2 90.00
PLW2 21.19799995
PLW12 0.15922000
PLW13 0.08008700

F2 - Processing parameters
SI 32768
SF 100.4713788
WDW EM
SSB 0
LB 1.00
GB 0
PC 1.40

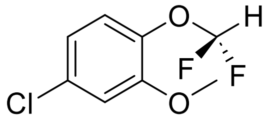

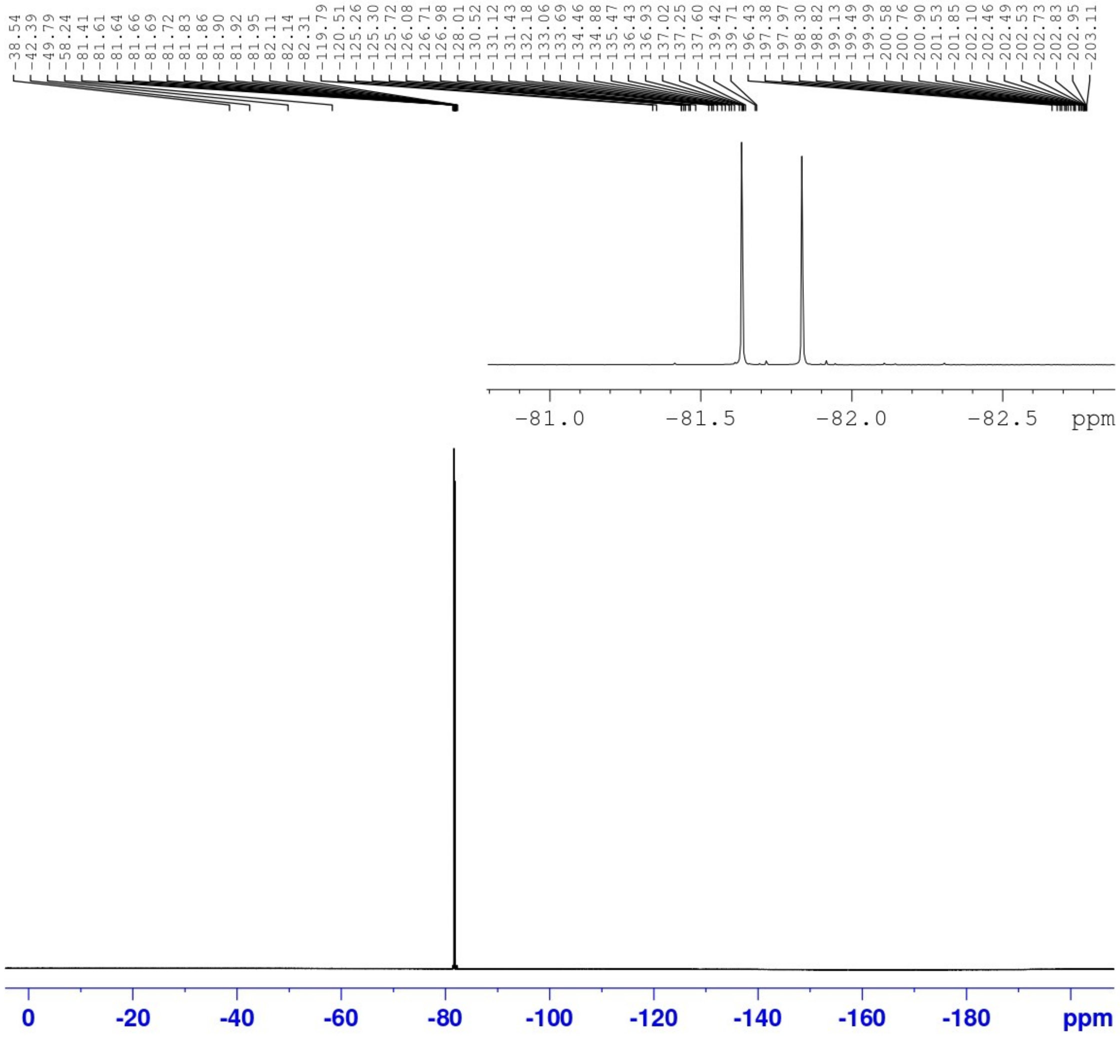

Current Data Parameters
NAME VII-Mn-26 i_11
EXPNO 1
PROCNO 1

F2 - Acquisition Parameters
Date_ 20241113
Time 17.41
INSTRUM Avance
PROBHD Z166552_0018 (PI HR-
PULPROG zg
TD 131072
SOLVENT CDCl3
NS 16
DS 4
SWH 90909.091
FIDRES 1.387163
AQ 0.7208960
RG 101
DW 5.500
DE 6.50
TE 298.0
D1 1.00000000
TD0 1
SFO1 375.9316815
NUC1 19F
P1 12.00
PLW1 32.47200012

F2 - Processing parameters
SI 65536
SF 375.9692784
WDW EM
SSB 0
LB 0.30
GB 0
PC 1.00

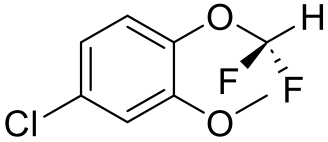

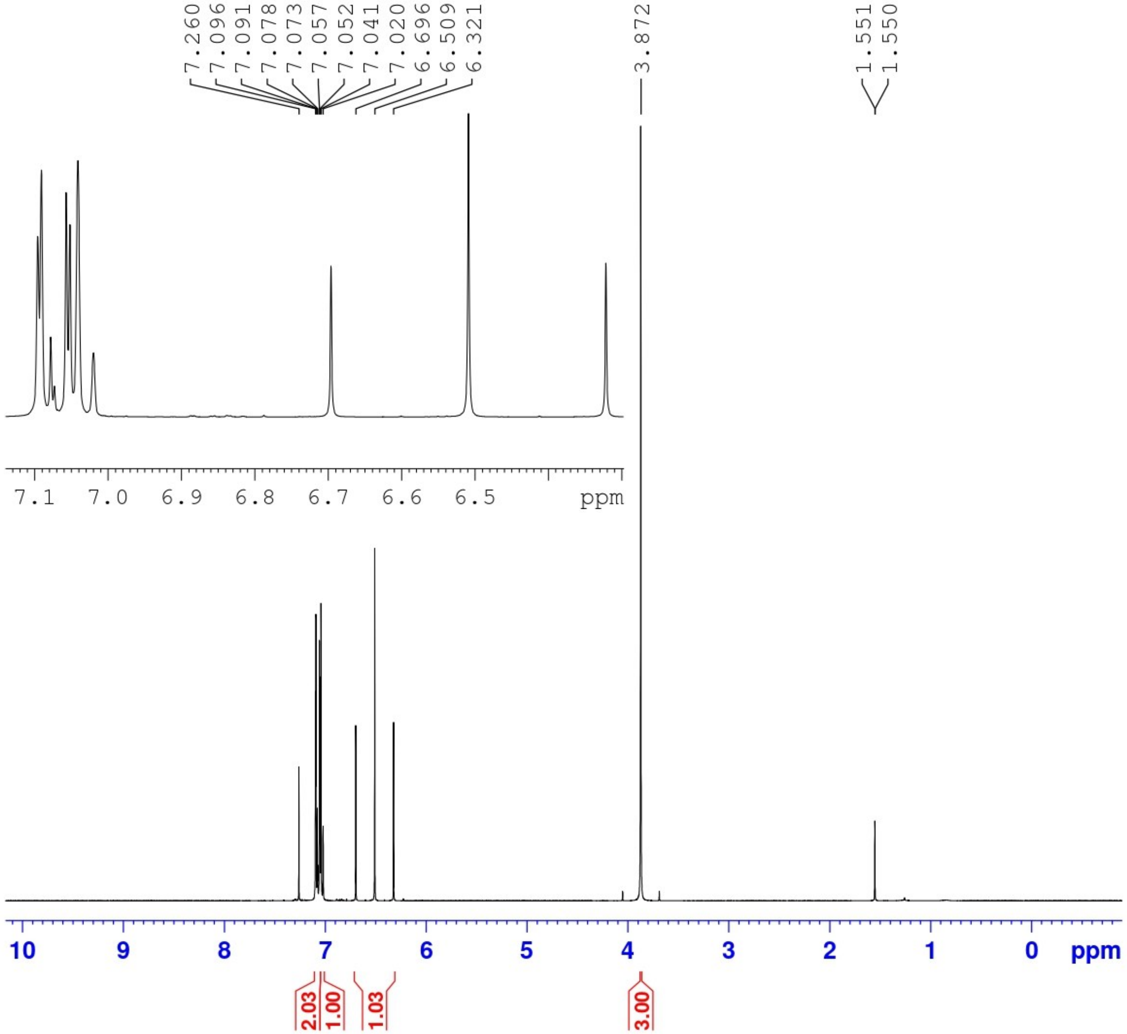

Current Data Parameters
NAME VII-Mn-12 i_10
EXPNO 4
PROCNO 1

F2 - Acquisition Parameters
Date_ 20241106
Time 22.00
INSTRUM Avance
PROBHD Z166552_0018 (PI HR-
PULPROG zg30
TD 65536
SOLVENT CDCl3
NS 16
DS 2
SWH 7812.500
FIDRES 0.238419
AQ 4.1943040
RG 101
DW 64.000
DE 6.67
TE 298.0
D1 1.00000000
TD0 1
SFO1 399.5701703
NUC1 1H
P0 2.60
P1 7.80
PLW1 21.19799995

F2 - Processing parameters
SI 65536
SF 399.5677126
WDW EM
SSB 0
LB 0.30
GB 0
PC 1.00

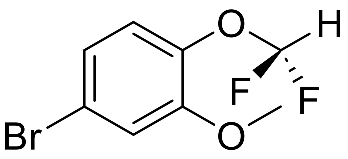

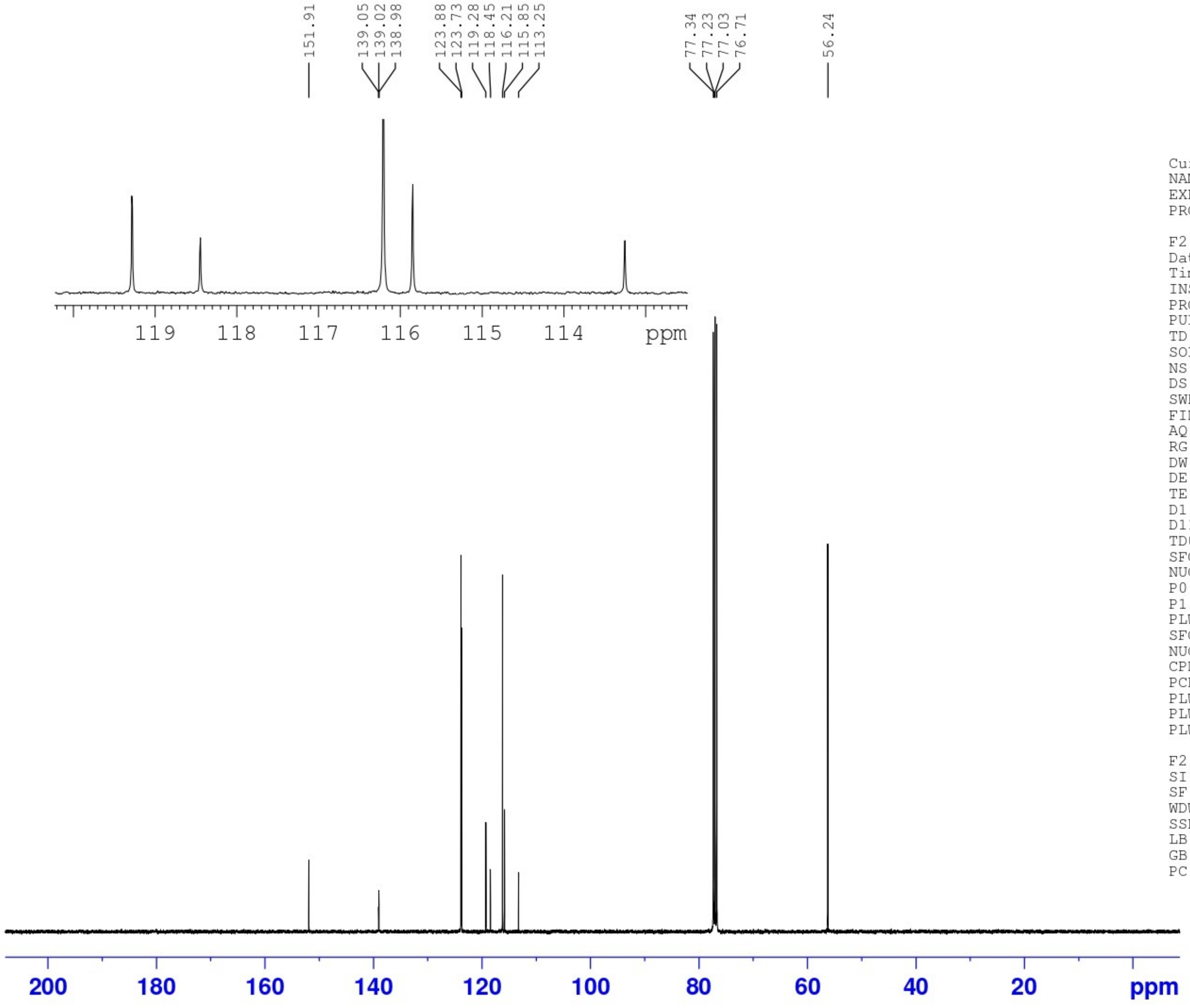

BRUKER

Current Data Parameters

NAME	VII-Mn-12 i_12
EXPNO	2
PROCNO	1

F2 - Acquisition Parameters

Date_	20241107
Time	0.01
INSTRUM	Avance
PROBHD	Z166552_0018 (PI HR-
PULPROG	zgpg30
TD	65536
SOLVENT	CDCl3
NS	2048
DS	4
SWH	23809.524
FIDRES	0.726609
AQ	1.3762560
RG	101
DW	21.000
DE	6.50
TE	298.0
D1	2.00000000
D11	0.03000000
TD0	1
SFO1	100.4814260
NUC1	13C
P0	2.67
P1	8.00
PLW1	88.22599792
SFO2	399.5693013
NUC2	1H
CPDPRG[2]	waltz65
PCPD2	90.00
PLW2	21.19799995
PLW12	0.15922000
PLW13	0.08008700

F2 - Processing parameters

SI	32768
SF	100.4713788
WDW	EM
SSB	0
LB	1.00
GB	0
PC	1.40

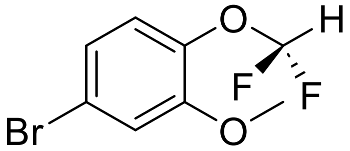

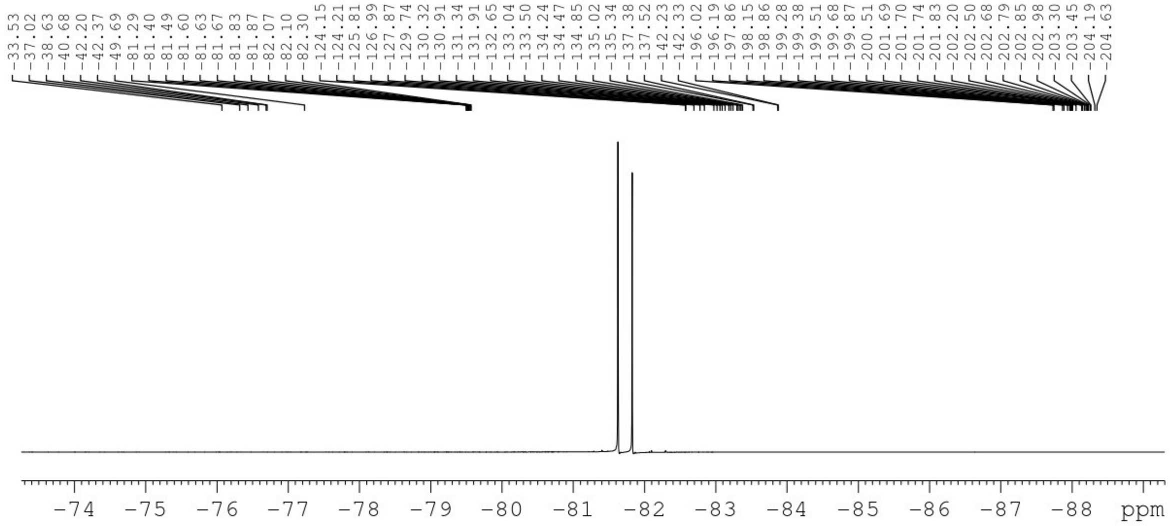

Current Data Parameters
NAME VII-Mn-12 i_11
EXPNO 1
PROCNO 1

F2 - Acquisition Parameters
Date_ 20241106
Time 22.02
INSTRUM Avance
PROBHD Z166552_0018 (PI HR-
PULPROG zg
TD 131072
SOLVENT CDCl3
NS 16
DS 4
SWH 90909.091
FIDRES 1.387163
AQ 0.7208960
RG 101
DW 5.500
DE 6.50
TE 298.0
D1 1.00000000
TD0 1
SFO1 375.9316815
NUC1 19F
P1 12.00
PLW1 32.47200012

F2 - Processing parameters
SI 65536
SF 375.9692784
WDW EM
SSB 0
LB 0.30
GB 0
PC 1.00

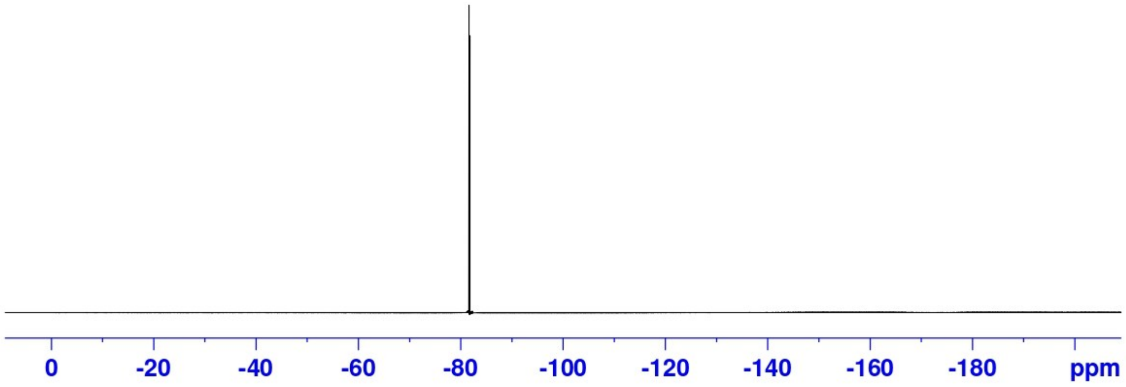

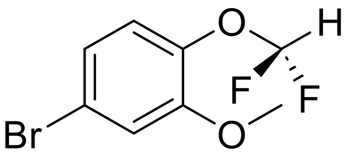

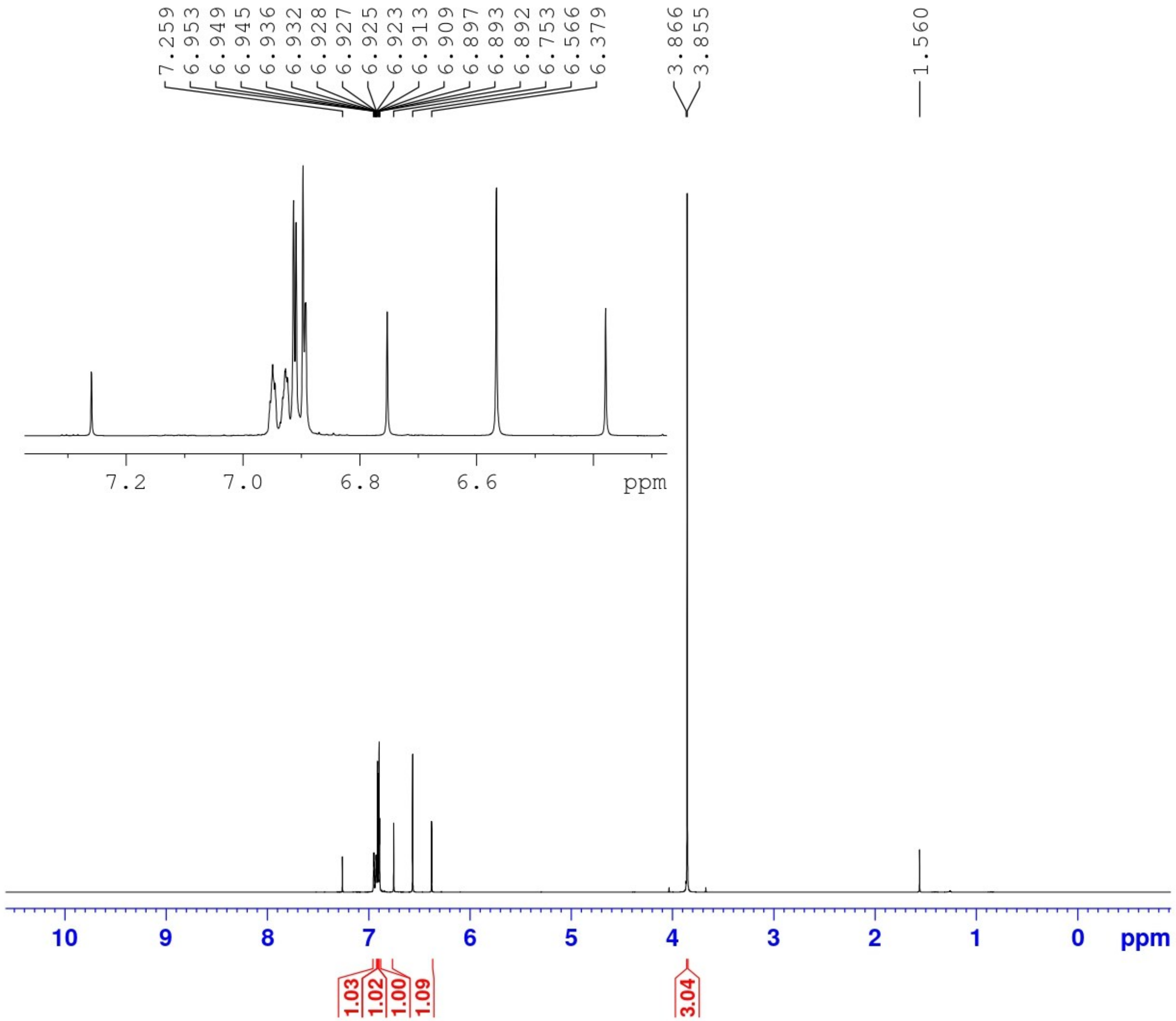

BRUKER

Current Data Parameters
NAME VII-Mn-37 i_10
EXPNO 2
PROCNO 1

F2 - Acquisition Parameters
Date_ 20241120
Time 16.29
INSTRUM Avance
PROBHD Z166552_0018 (PI HR-
PULPROG zg30
TD 65536
SOLVENT CDCl3
NS 16
DS 2
SWH 7812.500
FIDRES 0.238419
AQ 4.1943040
RG 101
DW 64.000
DE 6.67
TE 298.0
D1 1.00000000
TD0 1
SFO1 399.5701703
NUC1 1H
P0 2.60
P1 7.80
PLW1 21.19799995

F2 - Processing parameters
SI 65536
SF 399.5677128
WDW EM
SSB 0
LB 0.30
GB 0
PC 1.00

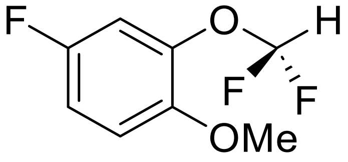

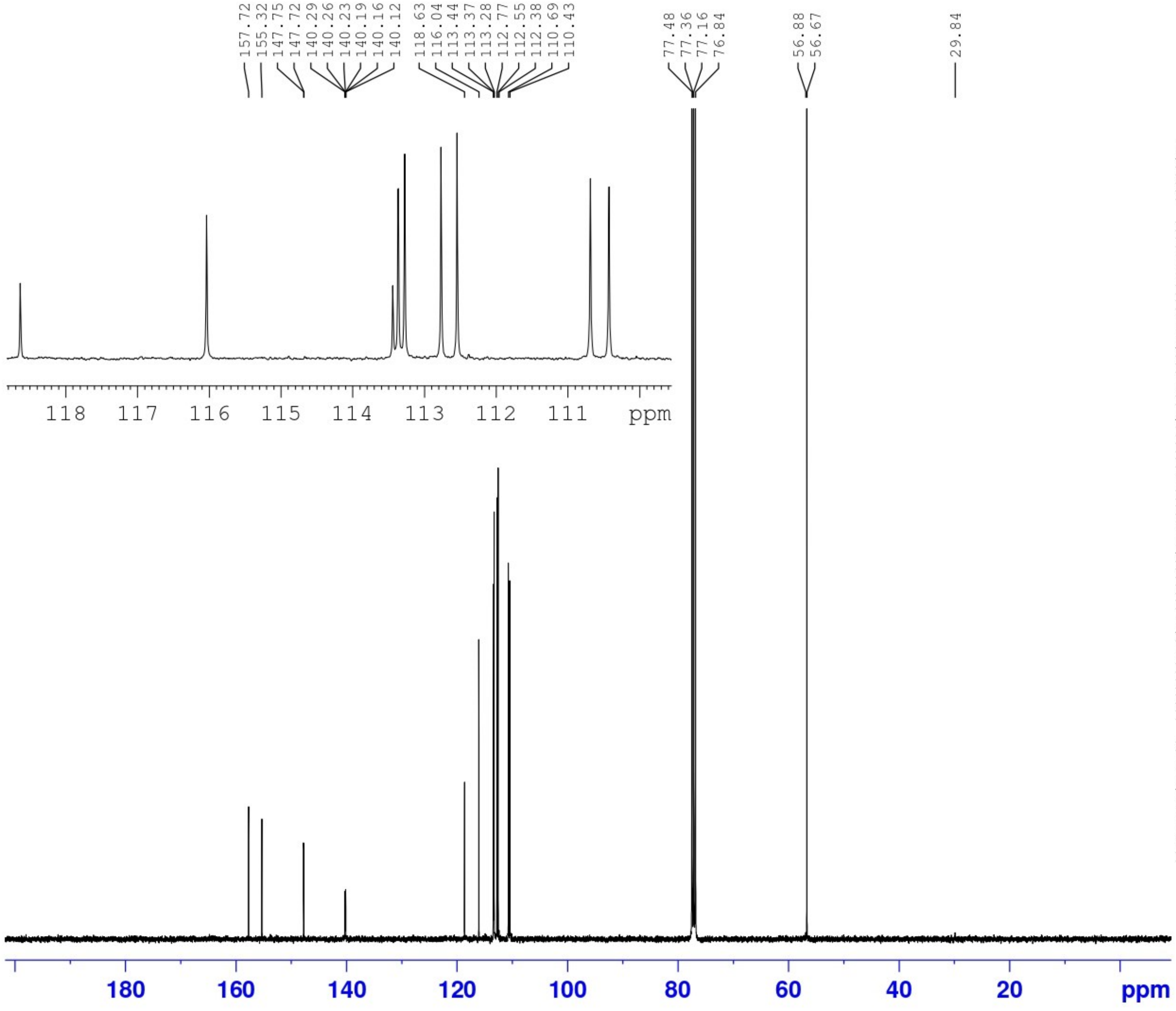
157.72
155.32
147.75
147.72
140.29
140.26
140.23
140.19
140.16
140.12
118.63
116.04
113.44
113.37
113.28
112.77
112.55
112.38
110.69
110.43
77.48
77.36
77.16
76.84
56.88
56.67
29.84
118 117 116 115 114 113 112 111 ppm
180 160 140 120 100 80 60 40 20 ppm
BRUKER
Current Data Parameters
NAME VII-Mn-37 i_12
EXPNO 2
PROCNO 1
F2 - Acquisition Parameters
Date_ 20241120
Time 21.10
INSTRUM Avance
PROBHD Z166552_0018 (PI HR-
PULPROG zgpg30
TD 65536
SOLVENT CDCl3
NS 2048
DS 4
SWH 23809.524
FIDRES 0.726609
AQ 1.3762560
RG 101
DW 21.000
DE 6.50
TE 298.0
D1 2.00000000
D11 0.03000000
TD0 1
SFO1 100.4814260
NUC1 13C
P0 2.67
P1 8.00
PLW1 88.22599792
SFO2 399.5693013
NUC2 1H
CPDPRG[2] waltz65
PCPD2 90.00
PLW2 21.19799995
PLW12 0.15922000
PLW13 0.08008700
F2 - Processing parameters
SI 32768
SF 100.4713648
WDW EM
SSB 0
LB 1.00
GB 0
PC 1.40

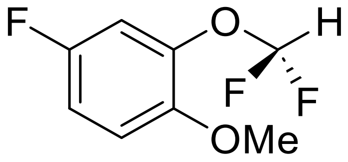
F
O
H
F
F
OMe

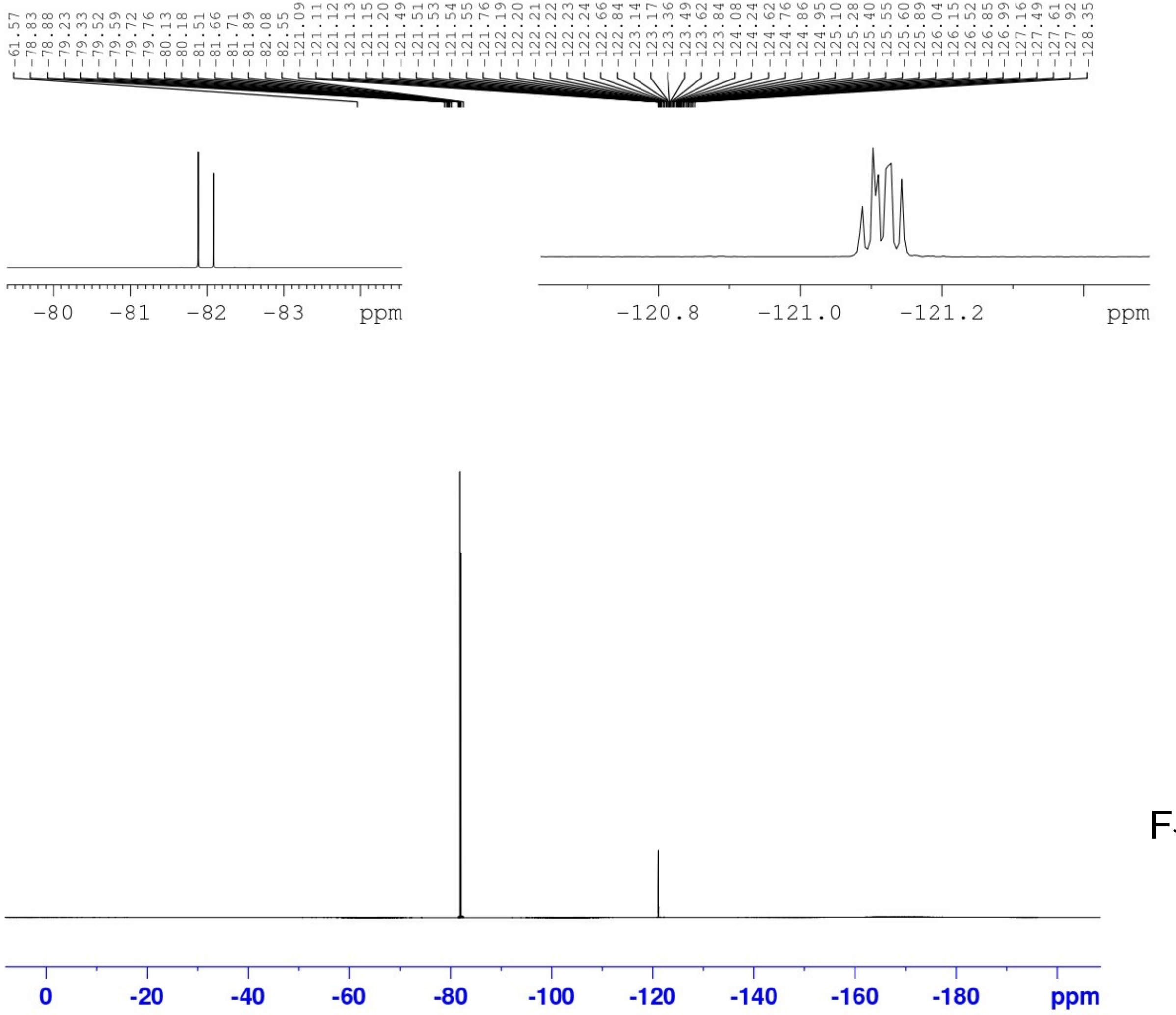

Current Data Parameters

NAME	VII-Mn-37 i_11
EXPNO	2
PROCNO	1

F2 - Acquisition Parameters

Date_	20241120
Time	16.30
INSTRUM	Avance
PROBHD	Z166552_0018 (PI HR-
PULPROG	zg
TD	131072
SOLVENT	CDCl3
NS	16
DS	4
SWH	90909.091
FIDRES	1.387163
AQ	0.7208960
RG	101
DW	5.500
DE	6.50
TE	298.0
D1	1.00000000
TD0	1
SFO1	375.9316815
NUC1	19F
P1	12.00
PLW1	32.47200012

F2 - Processing parameters

SI	65536
SF	375.9692784
WDW	EM
SSB	0
LB	0.30
GB	0
PC	1.00

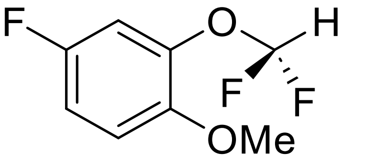

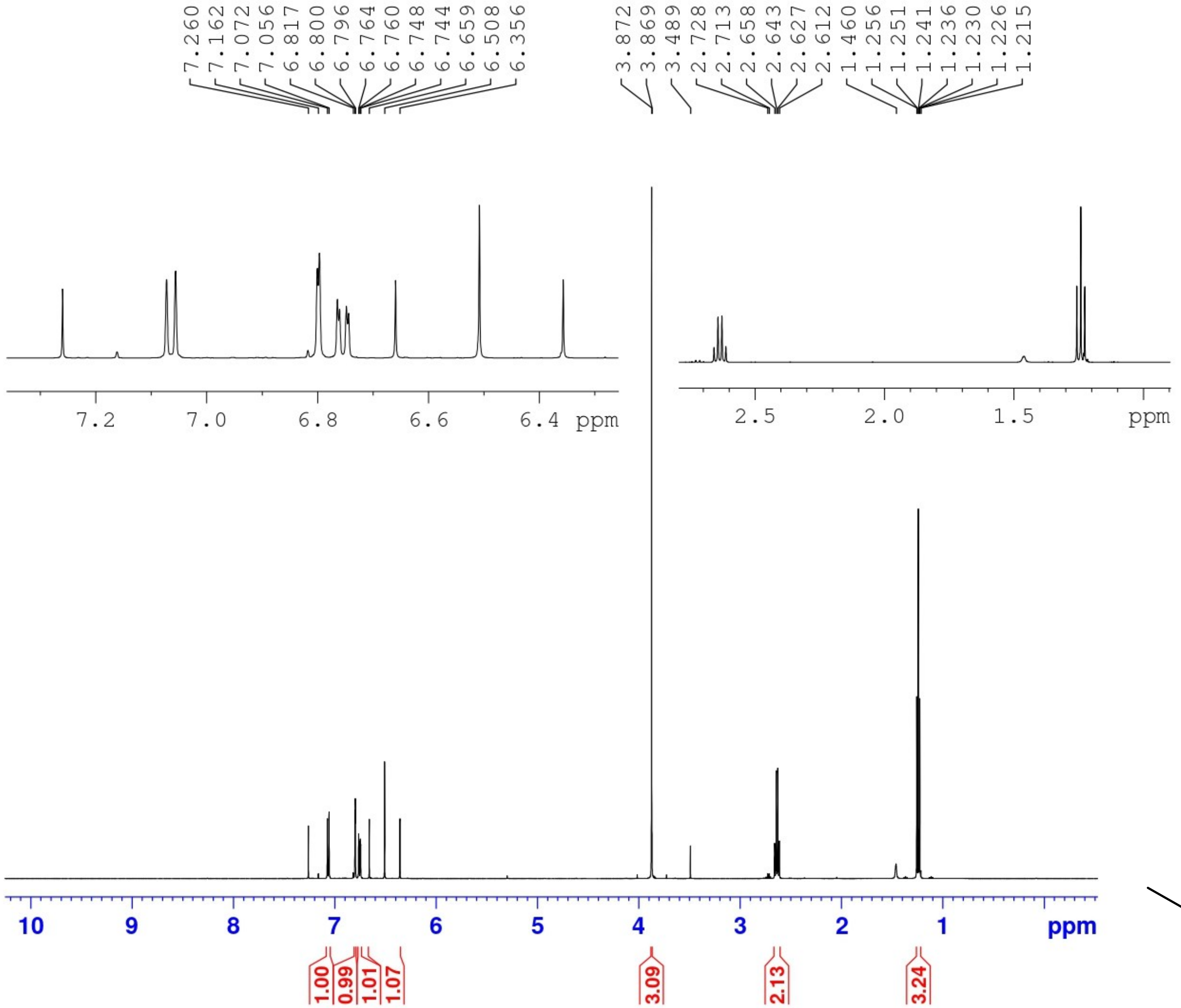

BRUKER

Current Data Parameters
NAME III-Mn-33 i_10
EXPNO 3
PROCNO 1

F2 - Acquisition Parameters
Date_ 20230909
Time 17.23
INSTRUM AS500-NEO
PROBHD Z168772_0026 (CPP1.1
PULPROG zg30
TD 65536
SOLVENT CDCl3
NS 16
DS 2
SWH 10000.000
FIDRES 0.305176
AQ 3.2767999
RG 45.2
DW 50.000
DE 10.45
TE 300.0
D1 1.00000000
TD0 1
SFO1 499.7860862
NUC1 1H
P0 4.00
P1 12.00
PLW1 16.91500092

F2 - Processing parameters
SI 65536
SF 499.7830120
WDW EM
SSB 0
LB 0.30
GB 0
PC 1.00

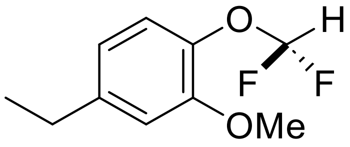

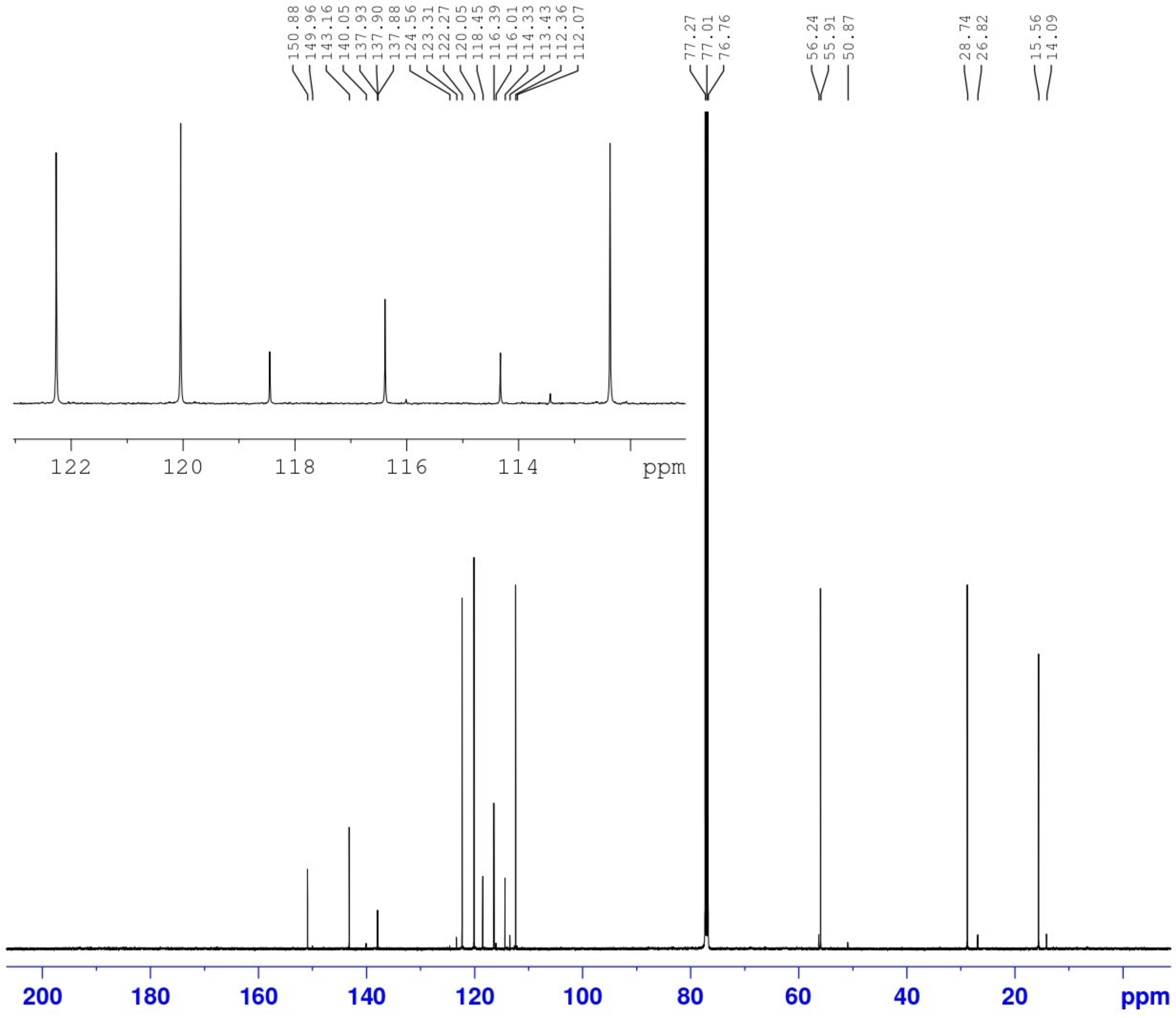

BRUKER

Current Data Parameters
NAME III-Mn-33 i_12
EXPNO 2
PROCNO 1

F2 - Acquisition Parameters
Date_ 20230909
Time 19.15
INSTRUM AS500-NEO
PROBHD Z168772_0026 (CPP1.1
PULPROG zgpg30
TD 65536
SOLVENT CDCl3
NS 2048
DS 4
SWH 30120.482
FIDRES 0.919204
AQ 1.0878977
RG 101
DW 16.600
DE 18.00
TE 300.0
D1 2.00000000
D11 0.03000000
TD0 1
SFO1 125.6831024
NUC1 13C
P0 3.33
P1 10.00
PLW1 59.16400146
SFO2 499.7849991
NUC2 1H
CPDPRG[2] waltz65
PCPD2 80.00
PLW2 16.91500092
PLW12 0.38058999
PLW13 0.19113000

F2 - Processing parameters
SI 32768
SF 125.6705353
WDW EM
SSB 0
LB 1.00
GB 0
PC 1.40

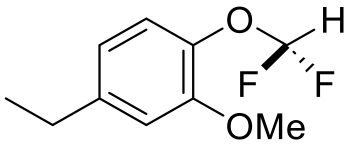

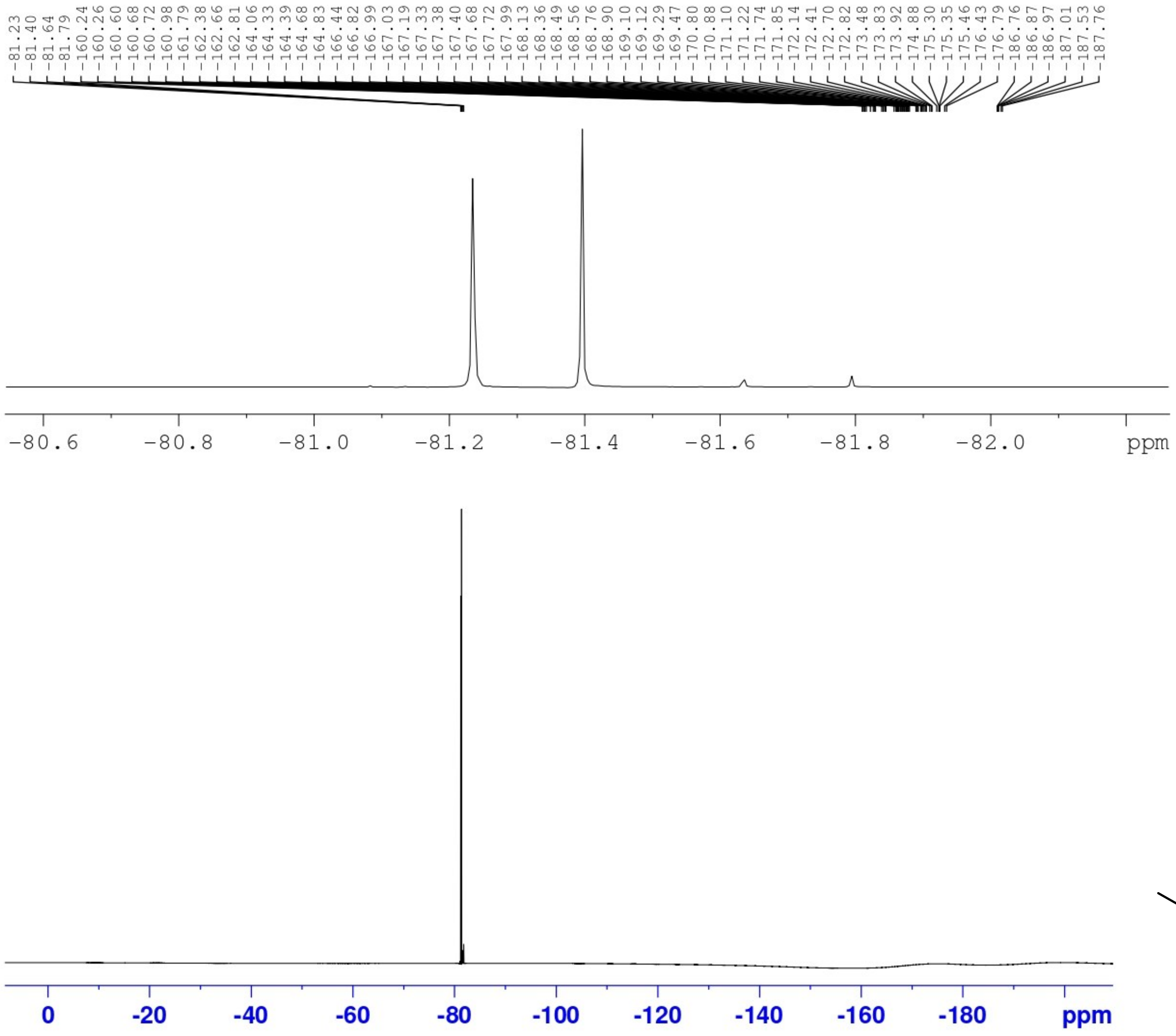

BRUKER

Current Data Parameters

NAME	III-Mn-33 i_11
EXPNO	3
PROCNO	1

F2 - Acquisition Parameters

Date_	20230909
Time	17.25
INSTRUM	AS500-NEO
PROBHD	Z168772_0026 (CPP1.1
PULPROG	zg
TD	131072
SOLVENT	CDCl3
NS	16
DS	4
SWH	113636.364
FIDRES	1.733953
AQ	0.5767168
RG	11.3
DW	4.400
DE	18.00
TE	300.0
D1	1.00000000
TD0	1
SFO1	470.2188444
NUC1	19F
P1	15.00
PLW1	10.89000034

F2 - Processing parameters

SI	65536
SF	470.2658710
WDW	EM
SSB	0
LB	0.30
GB	0
PC	1.00

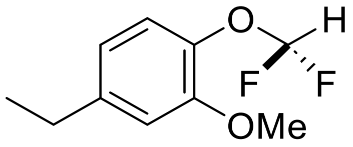

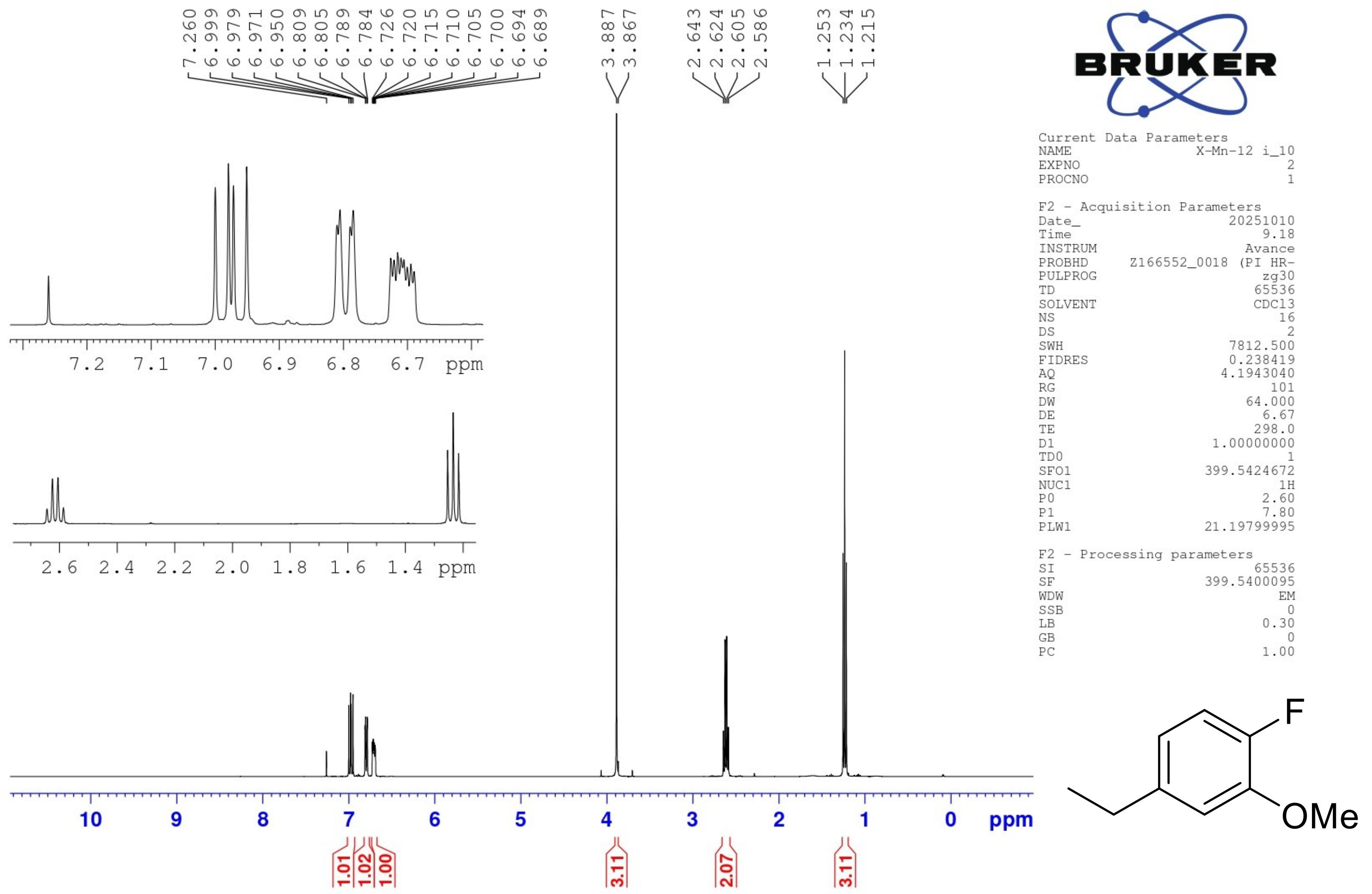
7.260
6.999
6.979
6.971
6.950
6.809
6.805
6.789
6.784
6.726
6.720
6.715
6.710
6.705
6.700
6.694
6.689
3.887
3.867
2.643
2.624
2.605
2.586
1.253
1.234
1.215
7.2 7.1 7.0 6.9 6.8 6.7 ppm
2.6 2.4 2.2 2.0 1.8 1.6 1.4 ppm
10 9 8 7 6 5 4 3 2 1 0 ppm
1.01
1.02
1.00
3.11
2.07
3.11
BRUKER
Current Data Parameters
NAME X-Mn-12 i_10
EXPNO 2
PROCNO 1
F2 - Acquisition Parameters
Date_ 20251010
Time 9.18
INSTRUM Avance
PROBHD Z166552_0018 (PI HR-
PULPROG zg30
TD 65536
SOLVENT CDCl3
NS 16
DS 2
SWH 7812.500
FIDRES 0.238419
AQ 4.1943040
RG 101
DW 64.000
DE 6.67
TE 298.0
D1 1.00000000
TD0 1
SFO1 399.5424672
NUC1 1H
P0 2.60
P1 7.80
PLW1 21.19799995
F2 - Processing parameters
SI 65536
SF 399.5400095
WDW EM
SSB 0
LB 0.30
GB 0
PC 1.00
F
OMe

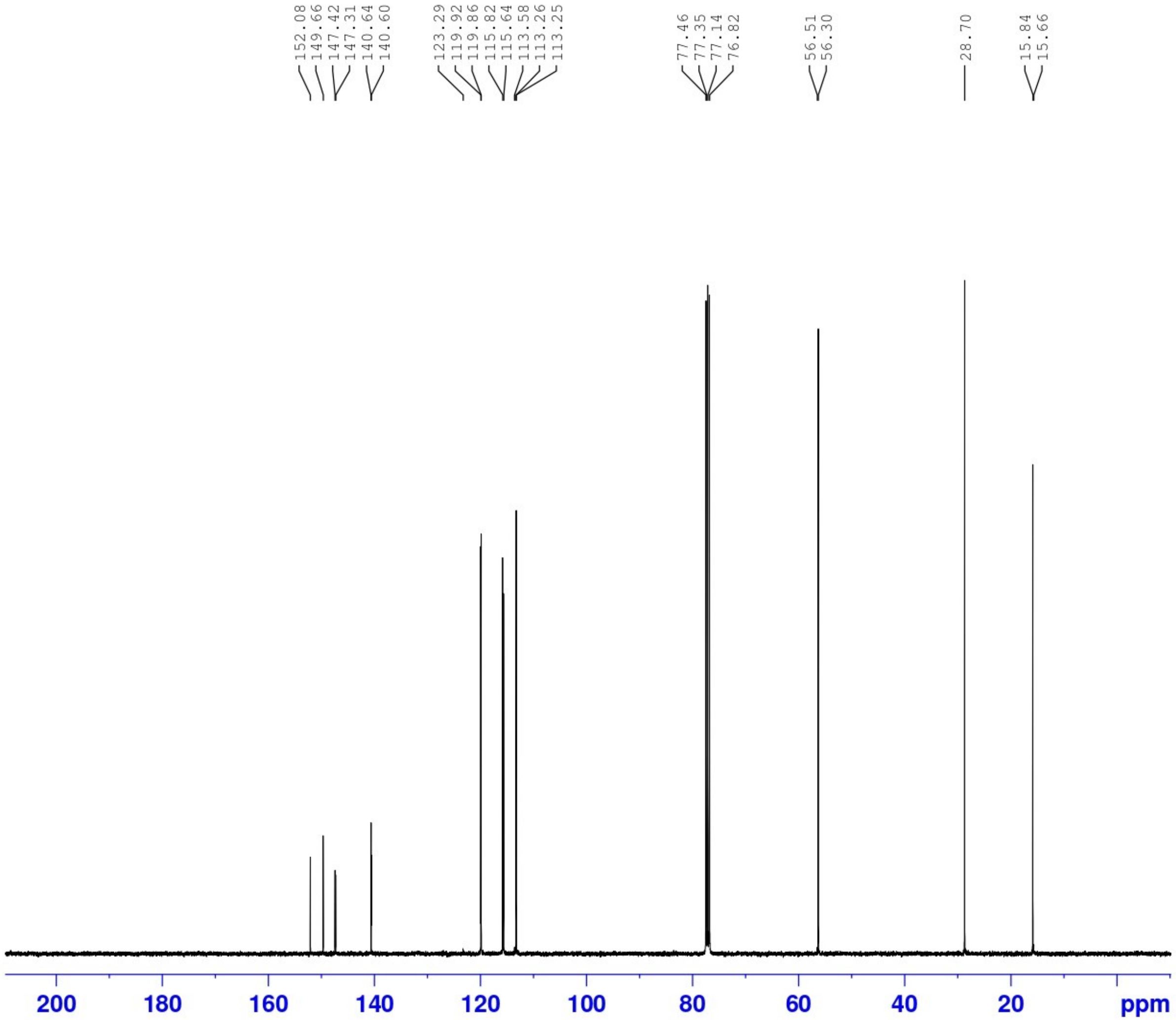

Current Data Parameters

NAME	X-Mn-12 i_12
EXPNO	3
PROCNO	1

F2 - Acquisition Parameters

Date_	20251010
Time	22.03
INSTRUM	Avance
PROBHD	Z166552_0018 (PI HR-
PULPROG	zgpg30
TD	65536
SOLVENT	CDCl3
NS	2048
DS	4
SWH	23809.524
FIDRES	0.726609
AQ	1.3762560
RG	101
DW	21.000
DE	6.50
TE	298.0
D1	2.00000000
D11	0.03000000
TD0	1
SFO1	100.4744593
NUC1	13C
P0	2.67
P1	8.00
PLW1	88.22599792
SFO2	399.5415982
NUC2	1H
CPDPRG[2]	waltz65
PCPD2	90.00
PLW2	21.19799995
PLW12	0.15922000
PLW13	0.08008700

F2 - Processing parameters

SI	32768
SF	100.4644025
WDW	EM
SSB	0
LB	1.00
GB	0
PC	1.40

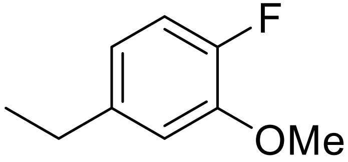

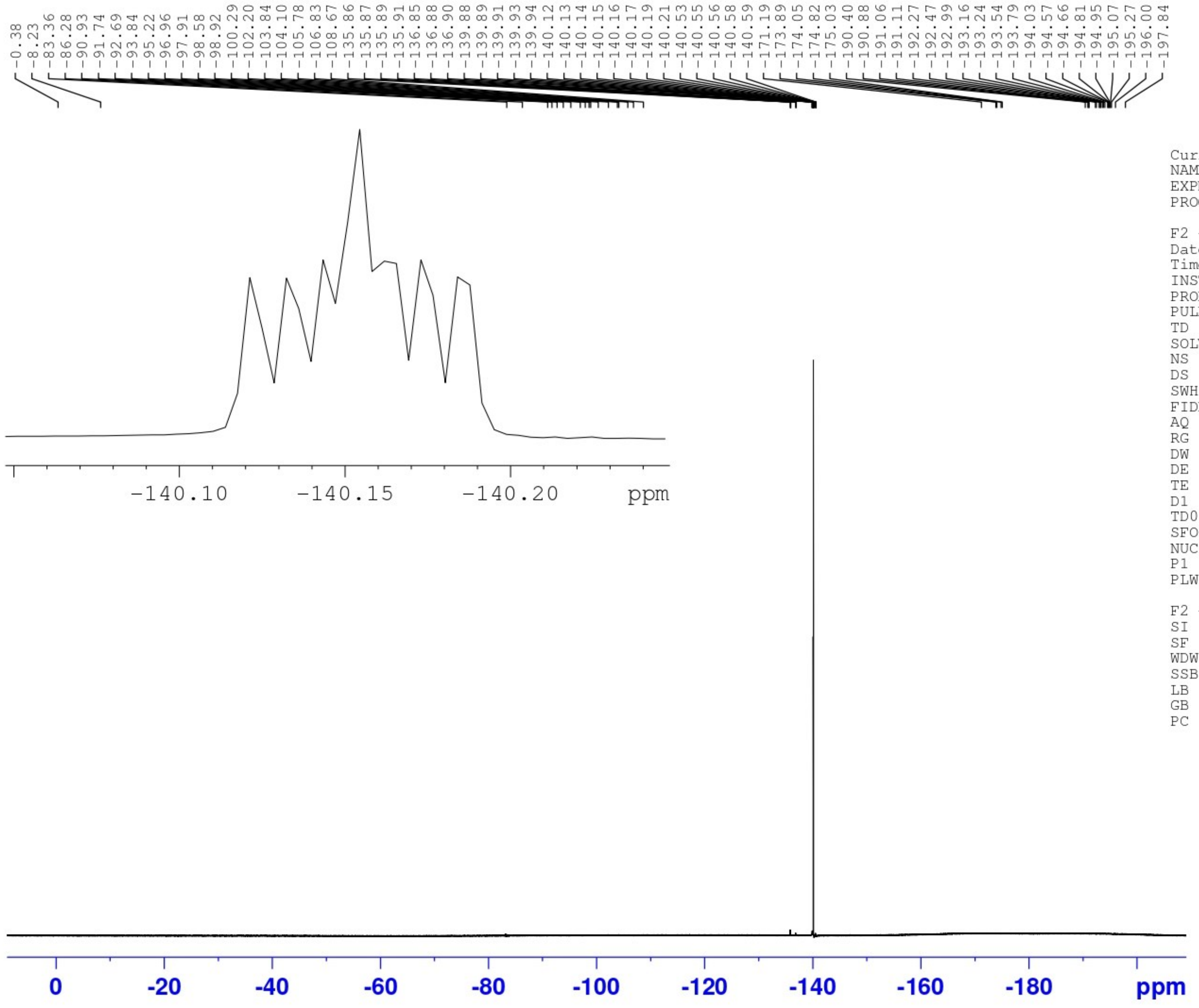

Current Data Parameters
NAME X-Mn-12 i_11
EXPNO 2
PROCNO 1

F2 - Acquisition Parameters
Date_ 20251010
Time 9.19
INSTRUM Avance
PROBHD Z166552_0018 (PI HR-
PULPROG zg
TD 131072
SOLVENT CDCl3
NS 16
DS 4
SWH 90909.091
FIDRES 1.387163
AQ 0.7208960
RG 101
DW 5.500
DE 6.50
TE 298.0
D1 1.00000000
TD0 1
SFO1 375.9056172
NUC1 19F
P1 12.00
PLW1 32.47200012

F2 - Processing parameters
SI 65536
SF 375.9432115
WDW EM
SSB 0
LB 0.30
GB 0
PC 1.00

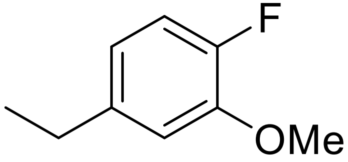

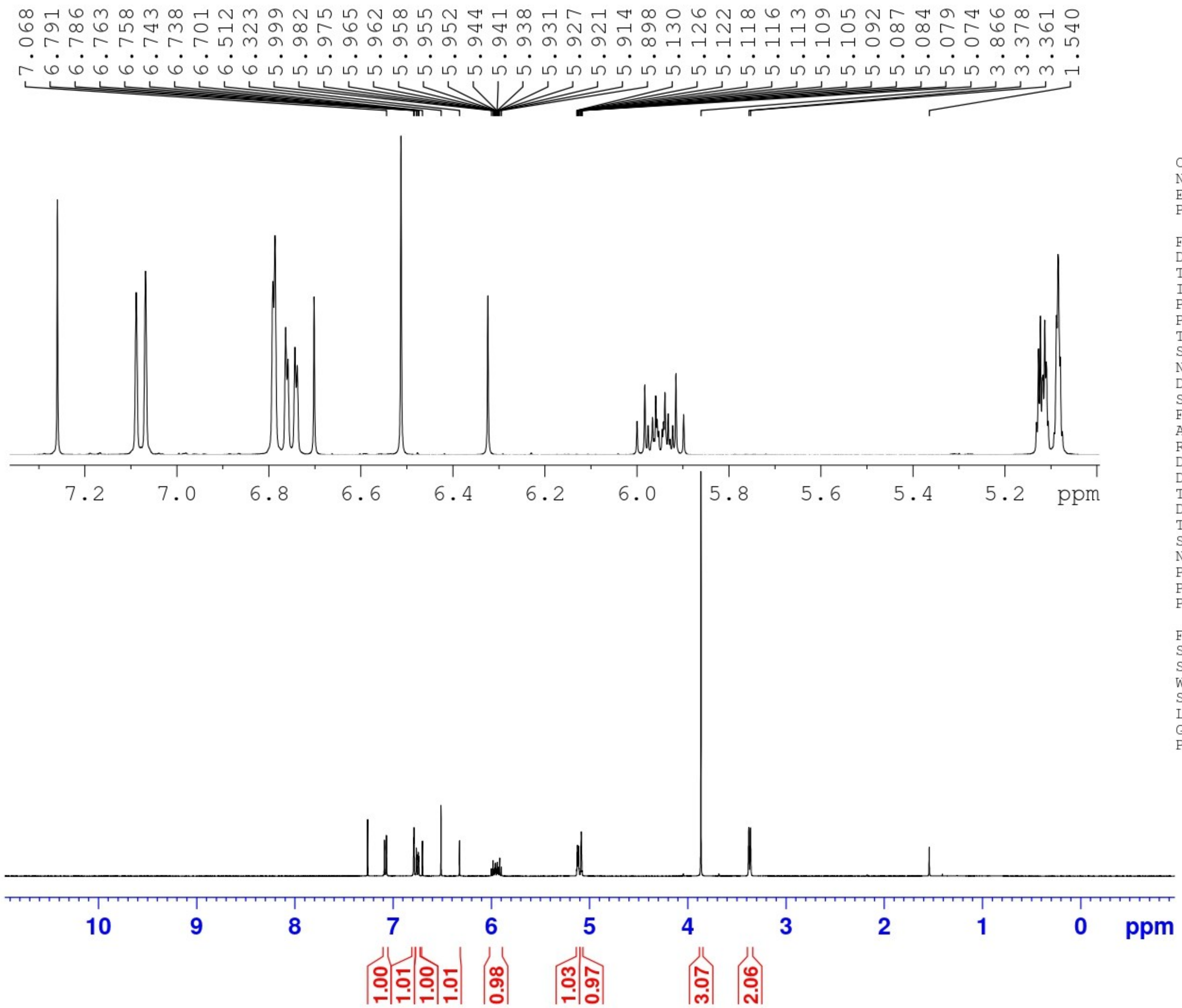

BRUKER

Current Data Parameters
NAME IV-Mn-102i_10
EXPNO 3
PROCNO 1

F2 - Acquisition Parameters
Date_ 20240221
Time 18.07
INSTRUM Avance
PROBHD Z166552_0018 (PI HR-
PULPROG zg30
TD 65536
SOLVENT CDCl3
NS 16
DS 2
SWH 7812.500
FIDRES 0.238419
AQ 4.1943040
RG 101
DW 64.000
DE 6.67
TE 298.0
D1 1.00000000
TD0 1
SFO1 399.6024675
NUC1 1H
P0 2.60
P1 7.80
PLW1 21.19799995

F2 - Processing parameters
SI 65536
SF 399.6000099
WDW EM
SSB 0
LB 0.30
GB 0
PC 1.00

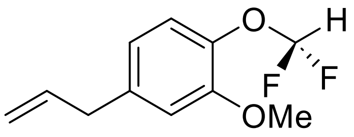

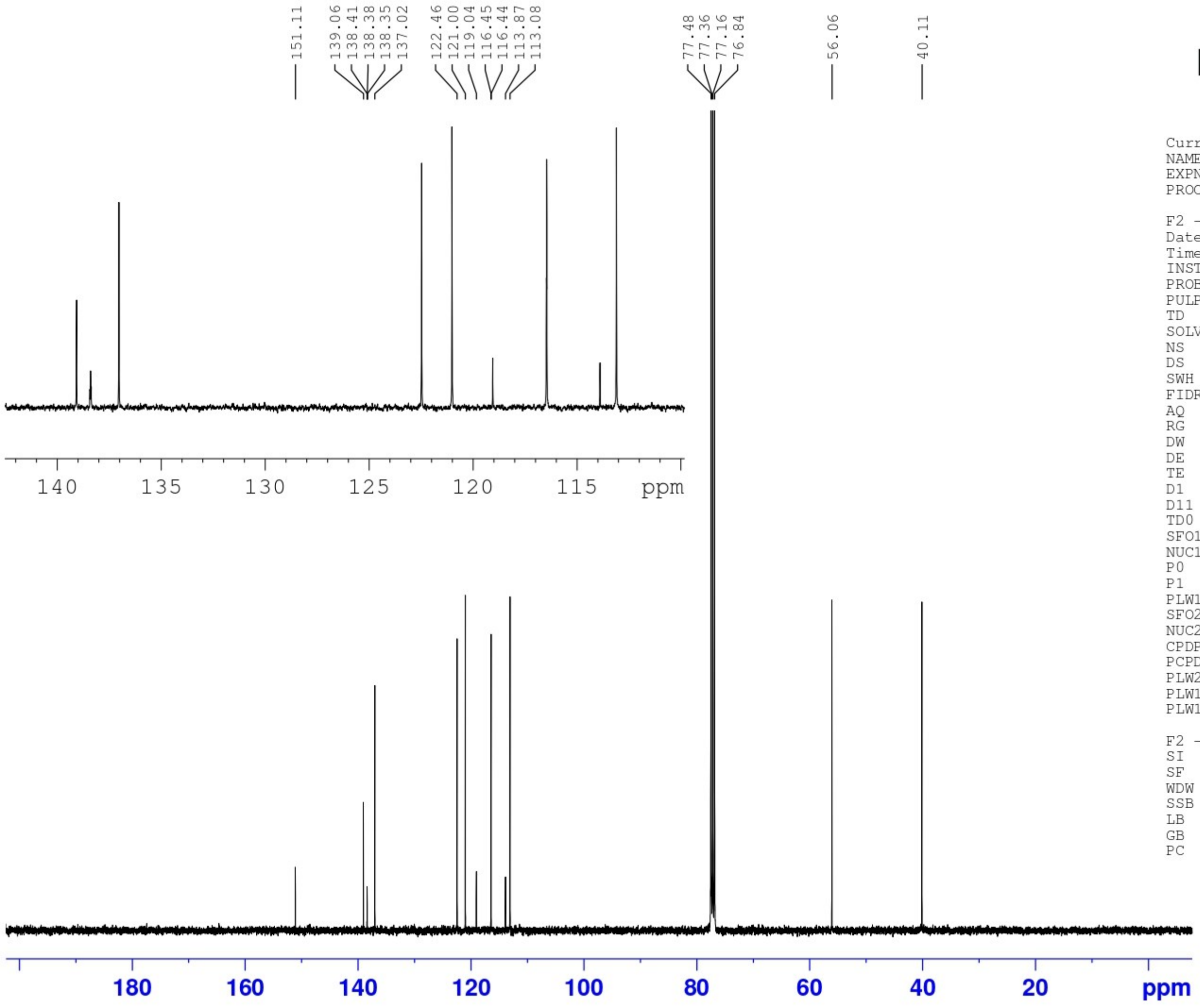

BRUKER

Current Data Parameters
NAME IV-Mn-102i_12
EXPNO 2
PROCNO 1

F2 - Acquisition Parameters
Date_ 20240221
Time 20.08
INSTRUM Avance
PROBHD Z166552_0018 (PI HR-
PULPROG zgpg30
TD 65536
SOLVENT CDCl3
NS 2048
DS 4
SWH 23809.524
FIDRES 0.726609
AQ 1.3762560
RG 101
DW 21.000
DE 6.50
TE 298.0
D1 2.00000000
D11 0.03000000
TD0 1
SFO1 100.4895479
NUC1 13C
P0 2.67
P1 8.00
PLW1 88.22599792
SFO2 399.6015984
NUC2 1H
CPDPRG[2] waltz65
PCPD2 90.00
PLW2 21.19799995
PLW12 0.15922000
PLW13 0.08008700

F2 - Processing parameters
SI 32768
SF 100.4794862
WDW EM
SSB 0
LB 1.00
GB 0
PC 1.40

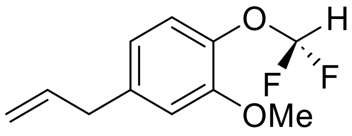

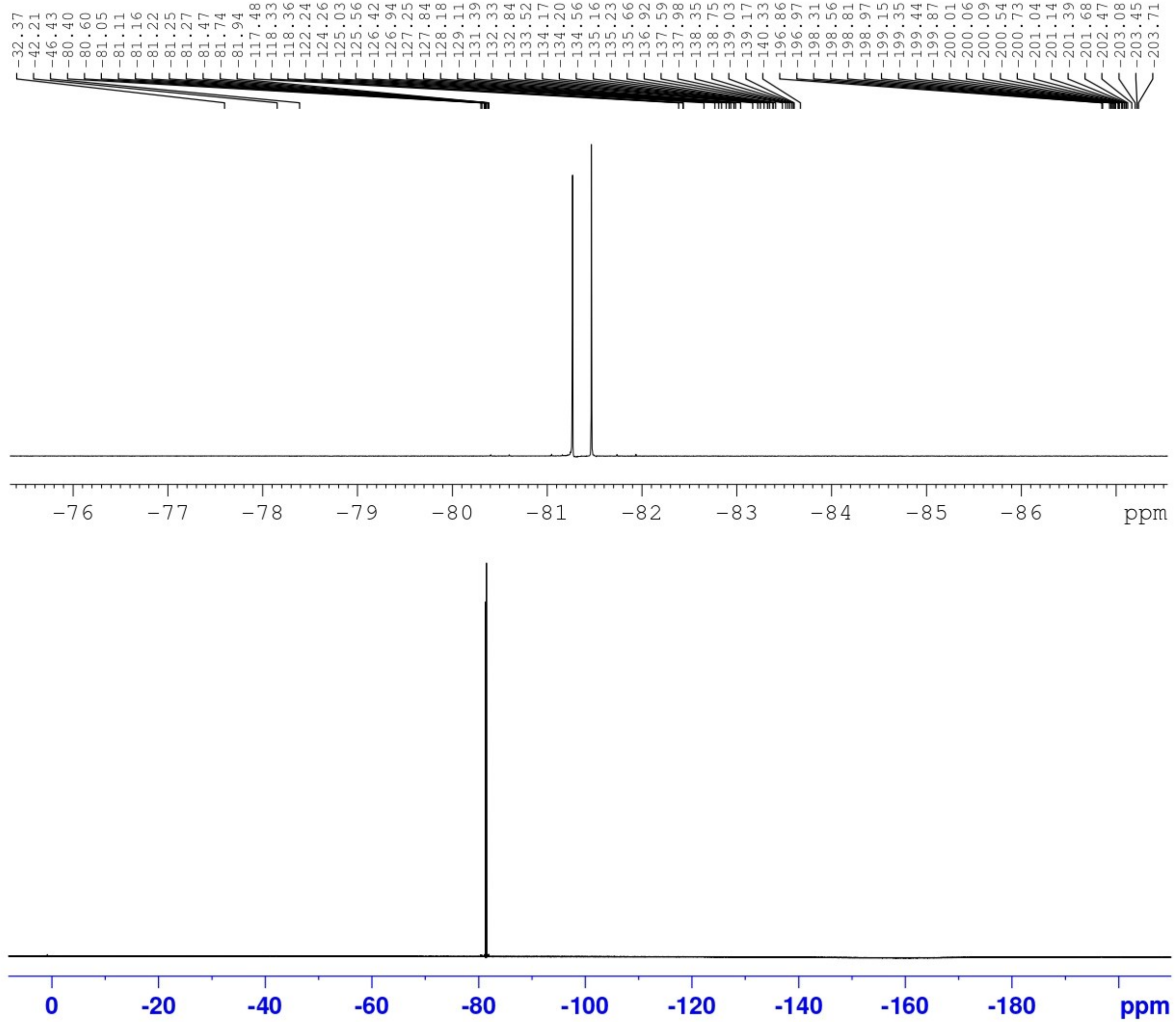

BRUKER

Current Data Parameters

NAME	IV-Mn-102i_11
EXPNO	3
PROCNO	1

F2 - Acquisition Parameters

Date_	20240221
Time	18.09
INSTRUM	Avance
PROBHD	Z166552_0018 (PI HR-
PULPROG	zg
TD	131072
SOLVENT	CDCl3
NS	16
DS	4
SWH	90909.091
FIDRES	1.387163
AQ	0.7208960
RG	101
DW	5.500
DE	6.50
TE	298.0
D1	1.00000000
TD0	1
SFO1	375.9620680
NUC1	19F
P1	12.00
PLW1	32.47200012

F2 - Processing parameters

SI	65536
SF	375.9996680
WDW	EM
SSB	0
LB	0.30
GB	0
PC	1.00

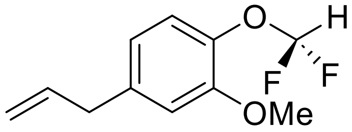

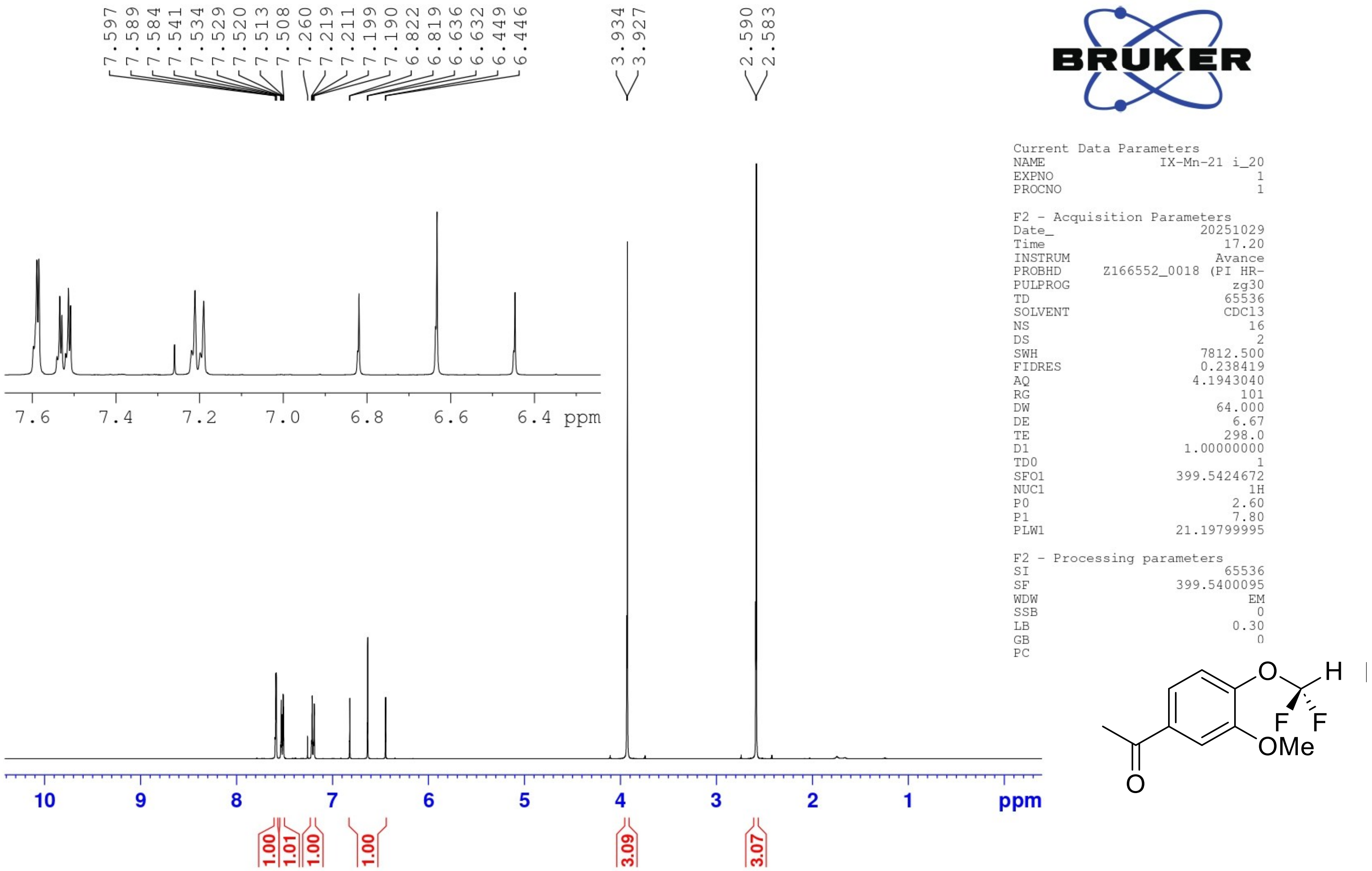
7.597
7.589
7.584
7.541
7.534
7.529
7.520
7.513
7.508
7.260
7.219
7.211
7.199
7.190
6.822
6.819
6.636
6.632
6.449
6.446
3.934
3.927
2.590
2.583
7.6
7.4
7.2
7.0
6.8
6.6
6.4 ppm
10
9
8
7
6
5
4
3
2
1
ppm
1.00
1.01
1.00
1.00
3.09
3.07
BRUKER
Current Data Parameters
NAME IX-Mn-21 i_20
EXPNO 1
PROCNO 1
F2 - Acquisition Parameters
Date_ 20251029
Time 17.20
INSTRUM Avance
PROBHD Z166552_0018 (PI HR-
PULPROG zg30
TD 65536
SOLVENT CDCl3
NS 16
DS 2
SWH 7812.500
FIDRES 0.238419
AQ 4.1943040
RG 101
DW 64.000
DE 6.67
TE 298.0
D1 1.00000000
TD0 1
SFO1 399.5424672
NUC1 1H
P0 2.60
P1 7.80
PLW1 21.19799995
F2 - Processing parameters
SI 65536
SF 399.5400095
WDW EM
SSB 0
LB 0.30
GB 0
PC
O
H
F
F
OMe
O

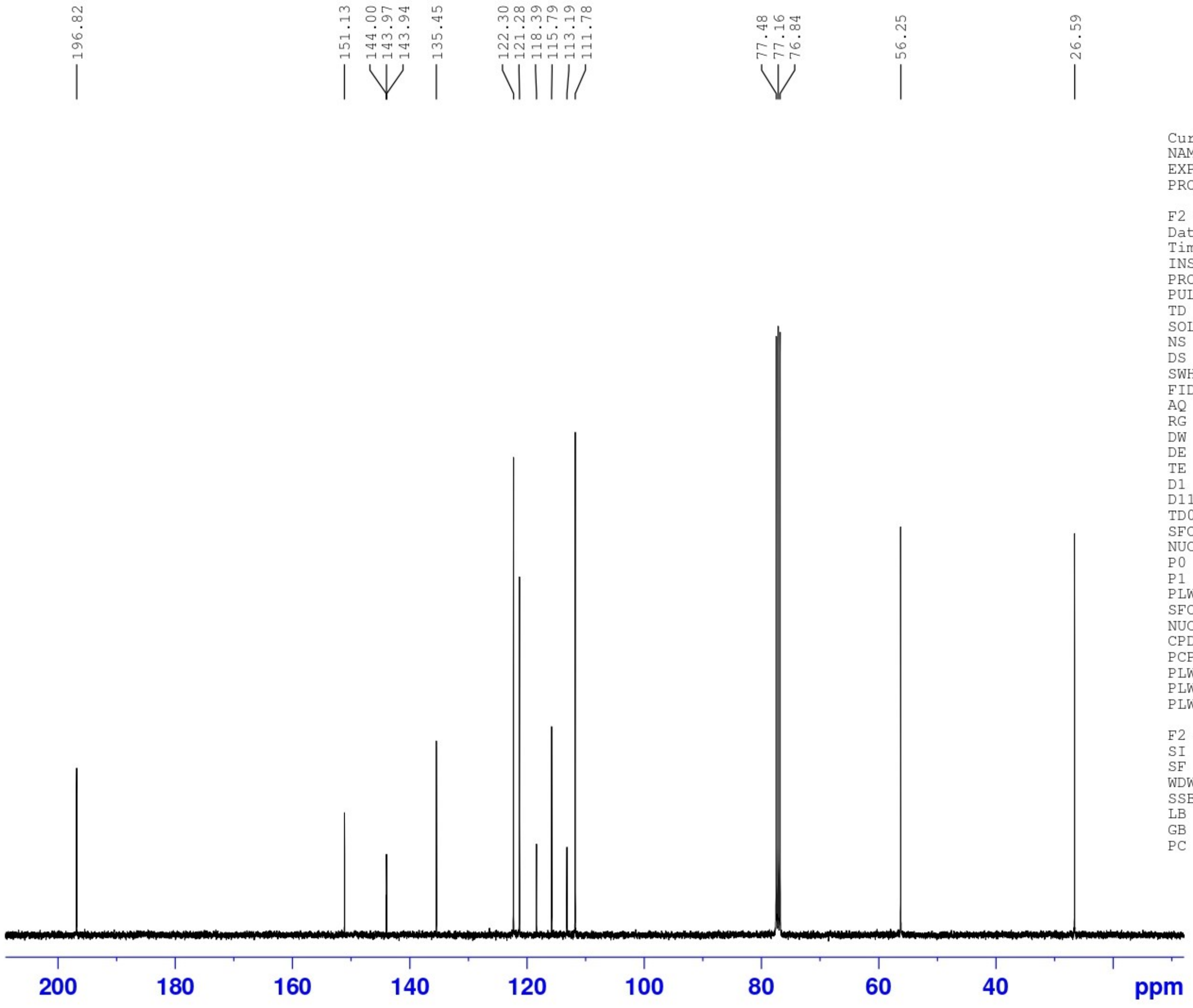

Current Data Parameters
NAME IX-Mn-21 i_22
EXPNO 1
PROCNO 1

F2 - Acquisition Parameters
Date_ 20251029
Time 17.53
INSTRUM Avance
PROBHD Z166552_0018 (PI HR-
PULPROG zgpg30
TD 65536
SOLVENT CDCl3
NS 512
DS 4
SWH 23809.524
FIDRES 0.726609
AQ 1.3762560
RG 101
DW 21.000
DE 6.50
TE 298.0
D1 2.00000000
D11 0.03000000
TD0 1
SFO1 100.4769709
NUC1 13C
P0 2.67
P1 8.00
PLW1 88.22599792
SFO2 399.5415982
NUC2 1H
CPDPRG[2] waltz65
PCPD2 90.00
PLW2 21.19799995
PLW12 0.15922000
PLW13 0.08008700

F2 - Processing parameters
SI 32768
SF 100.4644007
WDW EM
SSB 0
LB 1.00
GB
PC

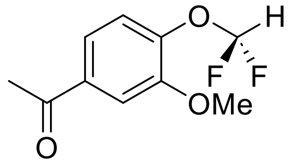

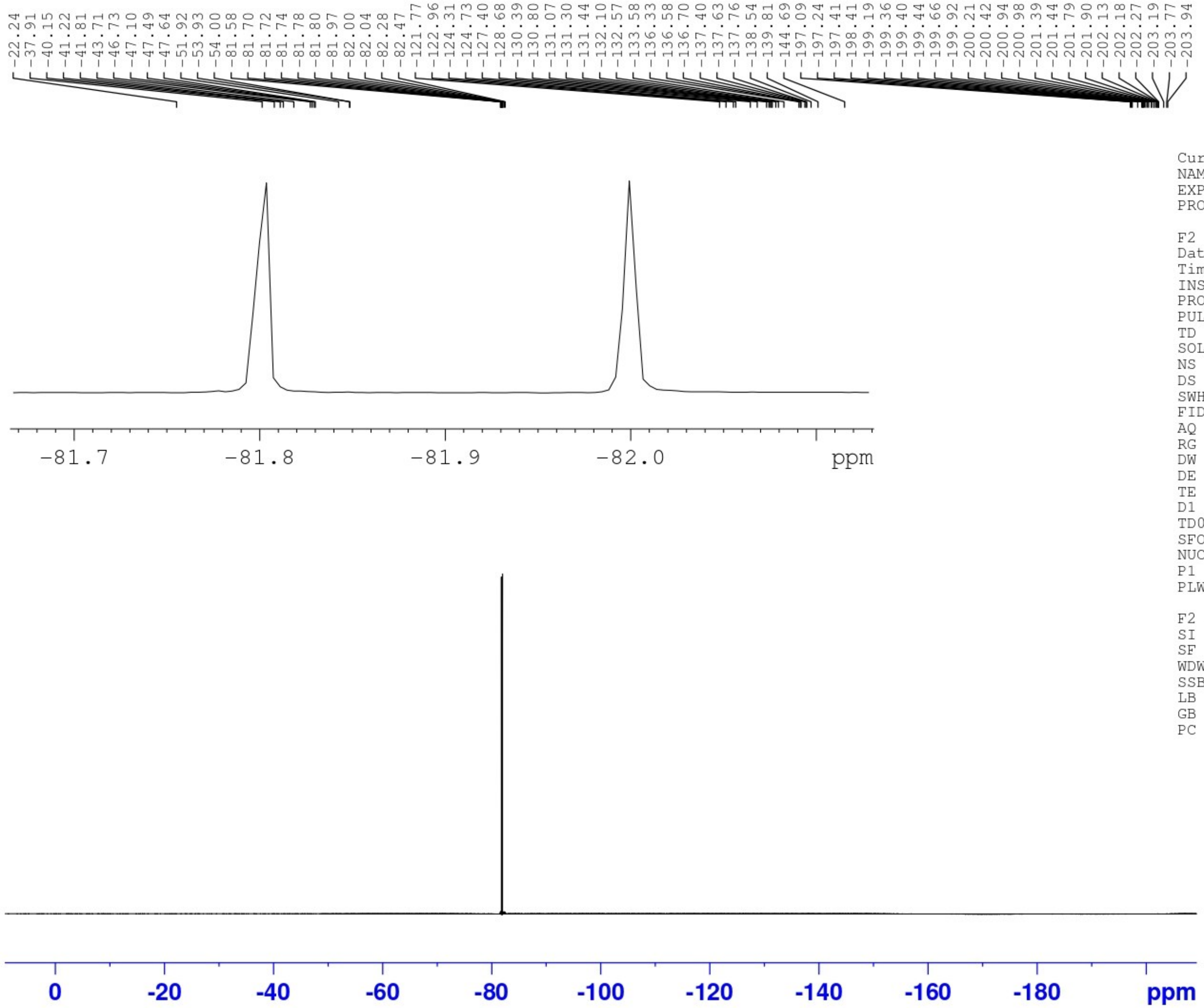

BRUKER

Current Data Parameters

NAME	IX-Mn-21 i_21
EXPNO	1
PROCNO	1

F2 - Acquisition Parameters

Date_	20251029
Time	17.22
INSTRUM	Avance
PROBHD	Z166552_0018 (PI HR-
PULPROG	zg
TD	131072
SOLVENT	CDCl3
NS	16
DS	4
SWH	90909.091
FIDRES	1.387163
AQ	0.7208960
RG	101
DW	5.500
DE	6.50
TE	298.0
D1	1.00000000
TD0	1
SFO1	375.9056172
NUC1	19F
P1	12.00
PLW1	32.47200012

F2 - Processing parameters

SI	65536
SF	375.9432115
WDW	EM
SSB	0
LB	0.30
GB	0
PC	1.00

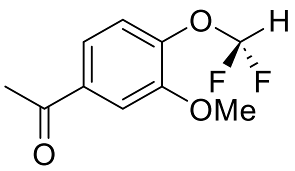

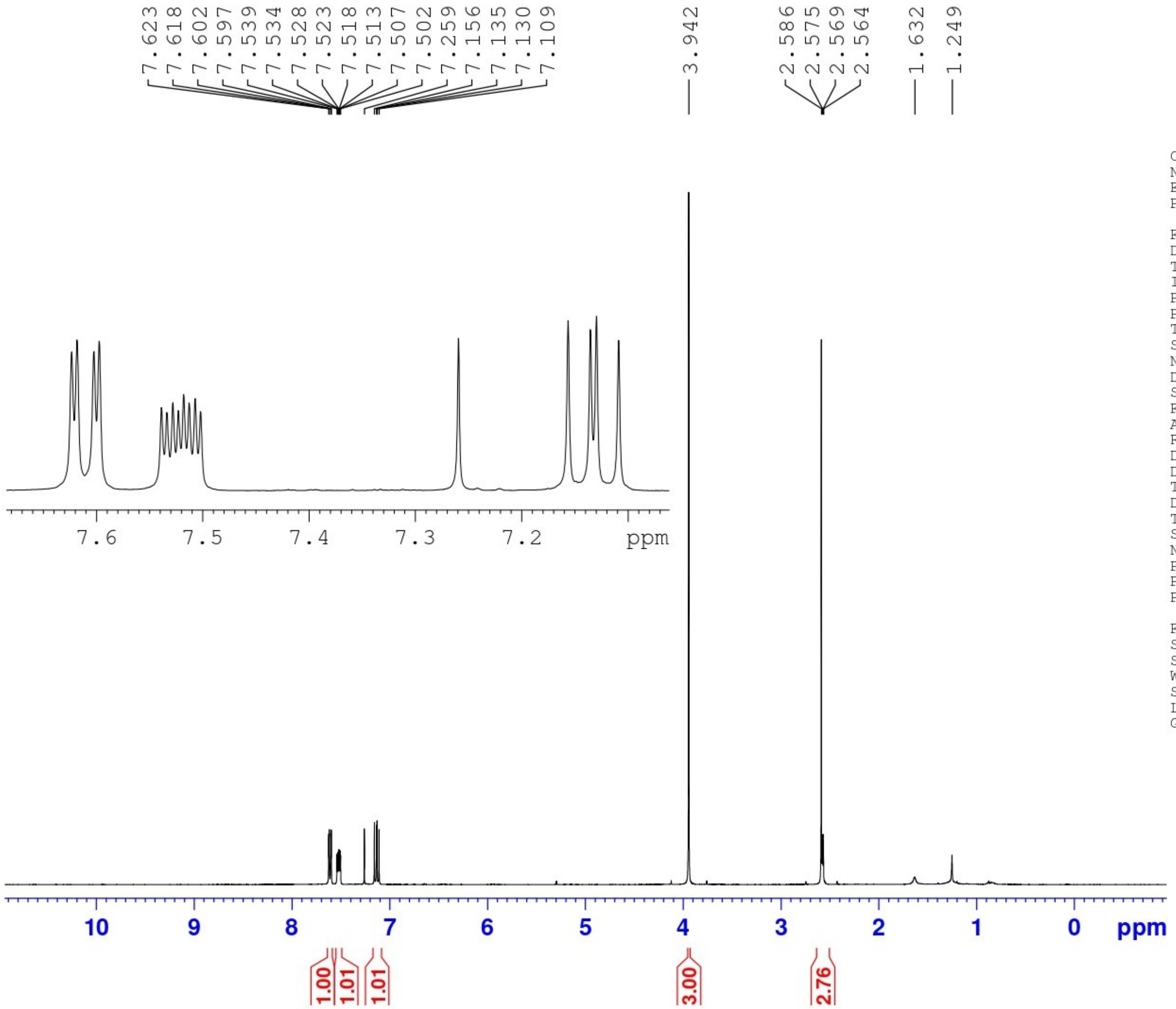


```
Current Data Parameters
NAME             IX-Mn-32 i_10
EXPNO                        2
PROCNO                       1

F2 - Acquisition Parameters
Date_                 20250528
Time                     17.51
INSTRUM                 Avance
PROBHD      Z166552_0018 (PI HR-
PULPROG                   zg30
TD                       65536
SOLVENT                  CDCl3
NS                          16
DS                           2
SWH                   7812.500
FIDRES                0.238419
AQ                   4.1943040
RG                         101
DW                      64.000
DE                        6.67
TE                       298.0
D1                  1.00000000
TD0                          1
SFO1               399.5424672
NUC1                        1H
P0                        2.60
P1                        7.80
PLW1               21.19799995

F2 - Processing parameters
SI                       65536
SF                 399.5400097
WDW                         EM
SSB                          0
LB                        0.30
GB                           0
```

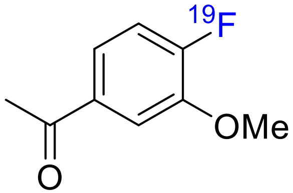

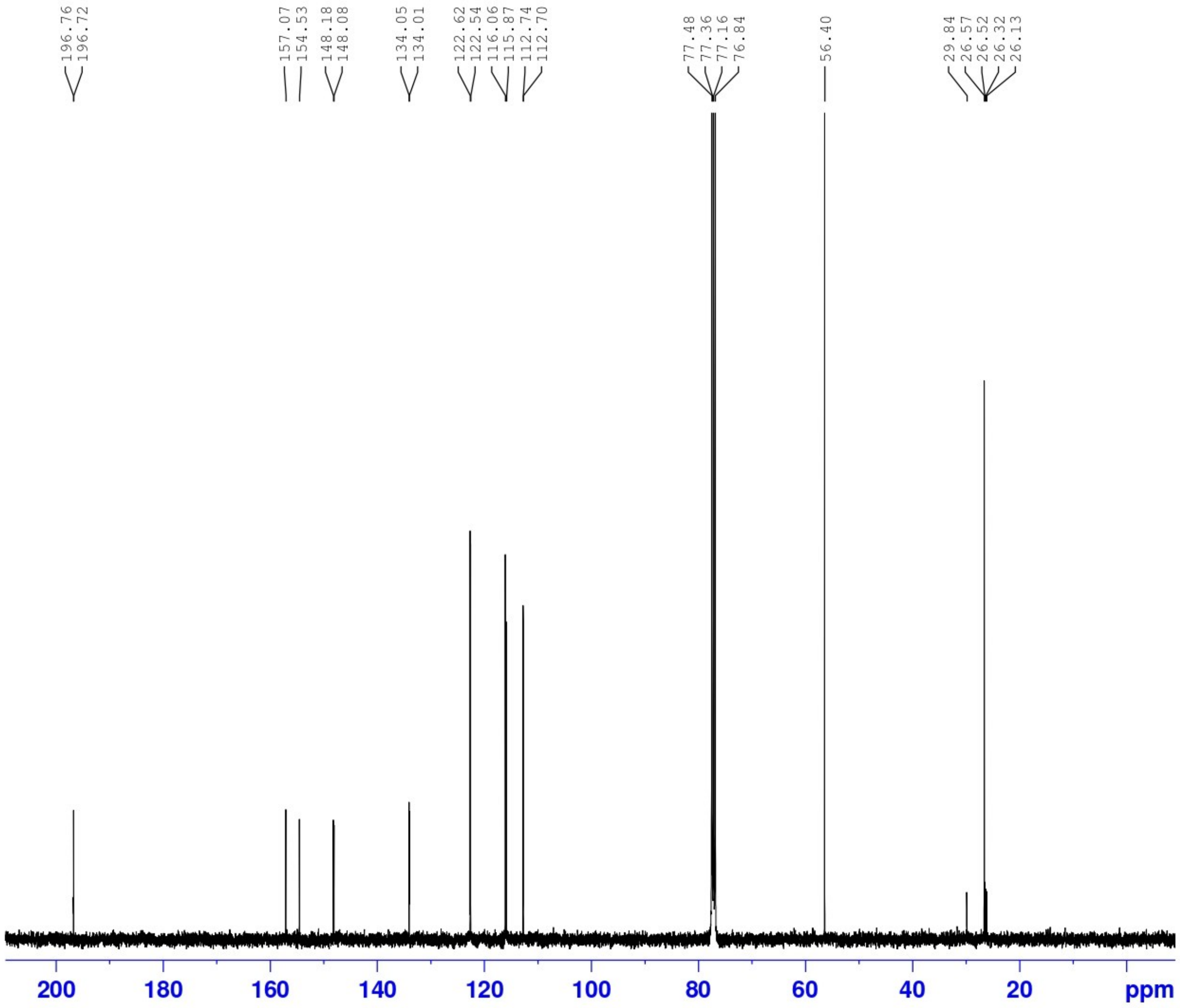

BRUKER

Current Data Parameters
NAME IX-Mn-32 i_12
EXPNO 2
PROCNO 1

F2 - Acquisition Parameters
Date_ 20250528
Time 22.16
INSTRUM Avance
PROBHD Z166552_0018 (PI HR-
PULPROG zgpg30
TD 65536
SOLVENT CDCl3
NS 2048
DS 4
SWH 23809.524
FIDRES 0.726609
AQ 1.3762560
RG 101
DW 21.000
DE 6.50
TE 298.0
D1 2.00000000
D11 0.03000000
TD0 1
SFO1 100.4744593
NUC1 13C
P0 2.67
P1 8.00
PLW1 88.22599792
SFO2 399.5415982
NUC2 1H
CPDPRG[2] waltz65
PCPD2 90.00
PLW2 21.19799995
PLW12 0.15922000
PLW13 0.08008700

F2 - Processing parameters
SI 32768
SF 100.4643993
WDW EM
SSB 0
LB 1.00
GB
PC

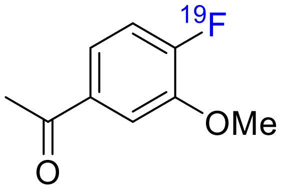

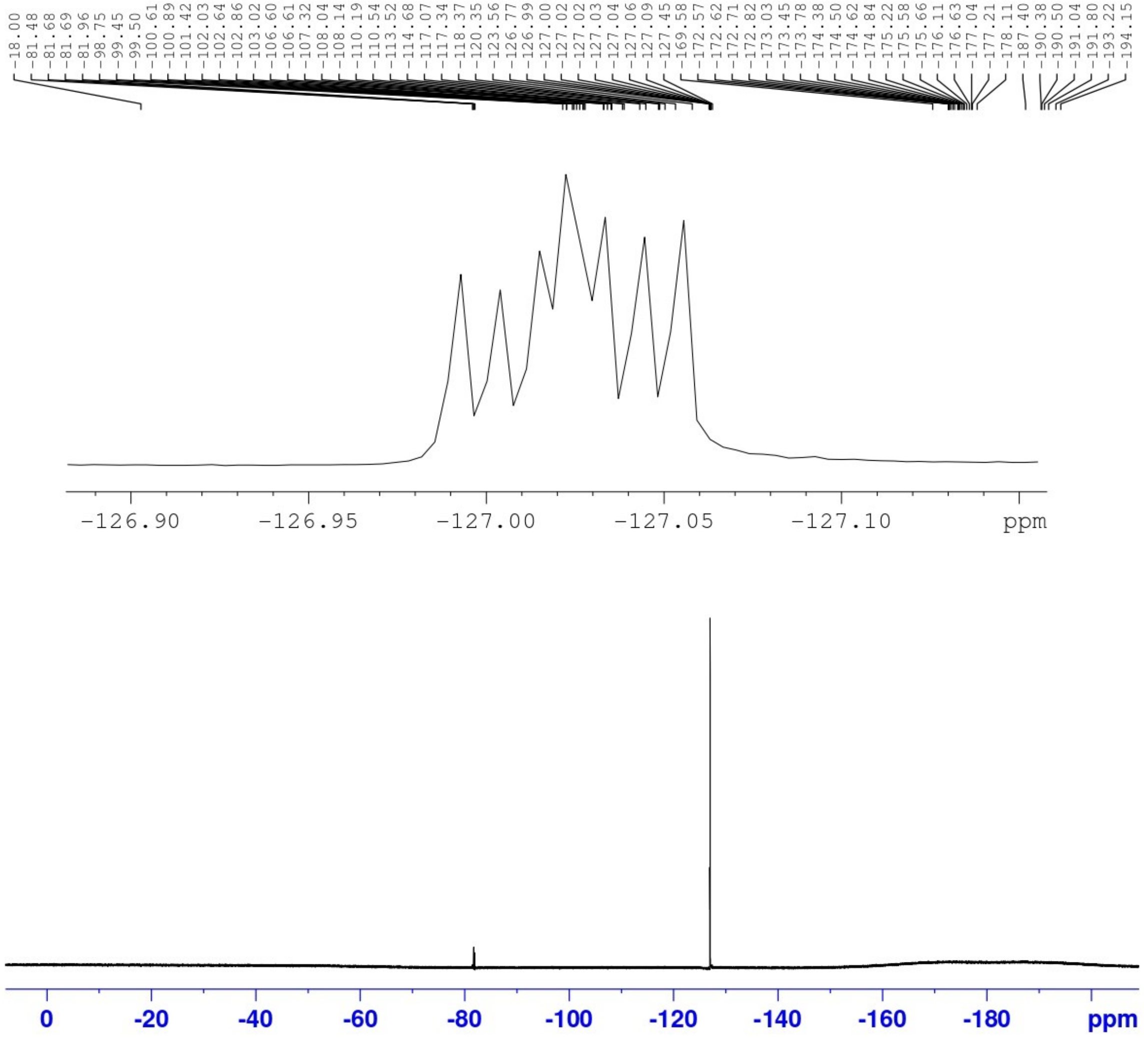


```
Current Data Parameters
NAME                IX-Mn-32 i_11
EXPNO                           2
PROCNO                          1

F2 - Acquisition Parameters
Date_                    20250528
Time                        17.53
INSTRUM                    Avance
PROBHD       Z166552_0018 (PI HR-
PULPROG                        zg
TD                         131072
SOLVENT                     CDCl3
NS                             16
DS                              4
SWH                     90909.091
FIDRES                   1.387163
AQ                      0.7208960
RG                            101
DW                          5.500
DE                           6.50
TE                          298.0
D1                     1.00000000
TD0                             1
SFO1                  375.9056172
NUC1                          19F
P1                          12.00
PLW1                  32.47200012

F2 - Processing parameters
SI                          65536
SF                    375.9432115
WDW                            EM
SSB                             0
LB                           0.30
GB                              0
PC                           1.00
```

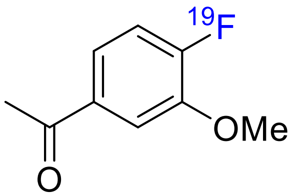

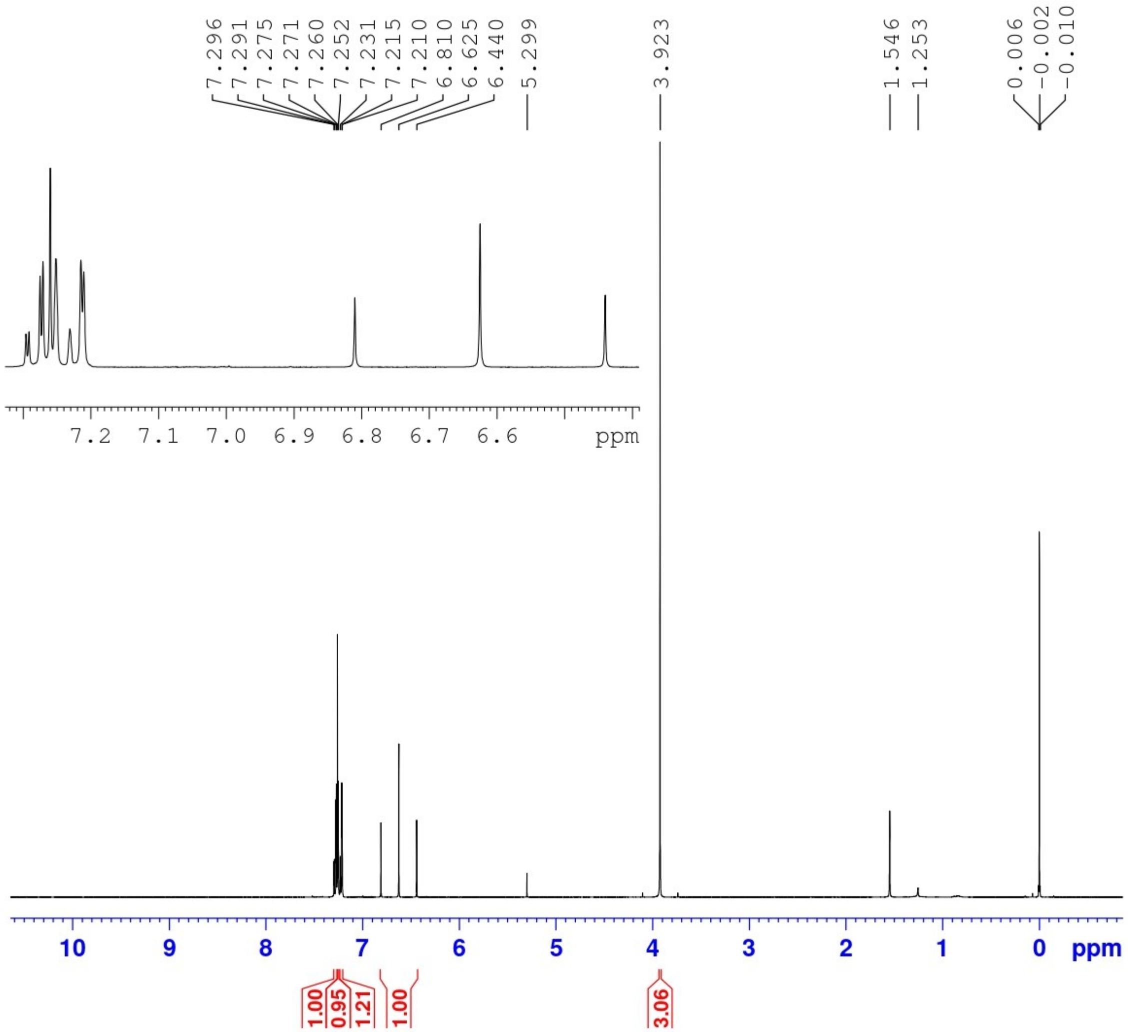

Current Data Parameters
NAME IV-Mn-126 i_10
EXPNO 2
PROCNO 1

F2 - Acquisition Parameters
Date_ 20240411
Time 13.38
INSTRUM Avance
PROBHD Z166552_0018 (PI HR-
PULPROG zg30
TD 65536
SOLVENT CDCl3
NS 16
DS 2
SWH 7812.500
FIDRES 0.238419
AQ 4.1943040
RG 101
DW 64.000
DE 6.67
TE 298.0
D1 1.00000000
TD0 1
SFO1 399.6024675
NUC1 1H
P0 2.60
P1 7.80
PLW1 21.19799995

F2 - Processing parameters
SI 65536
SF 399.6000096
WDW EM
SSB 0
LB 0.30
GB 0
PC 1.00

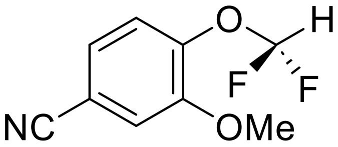

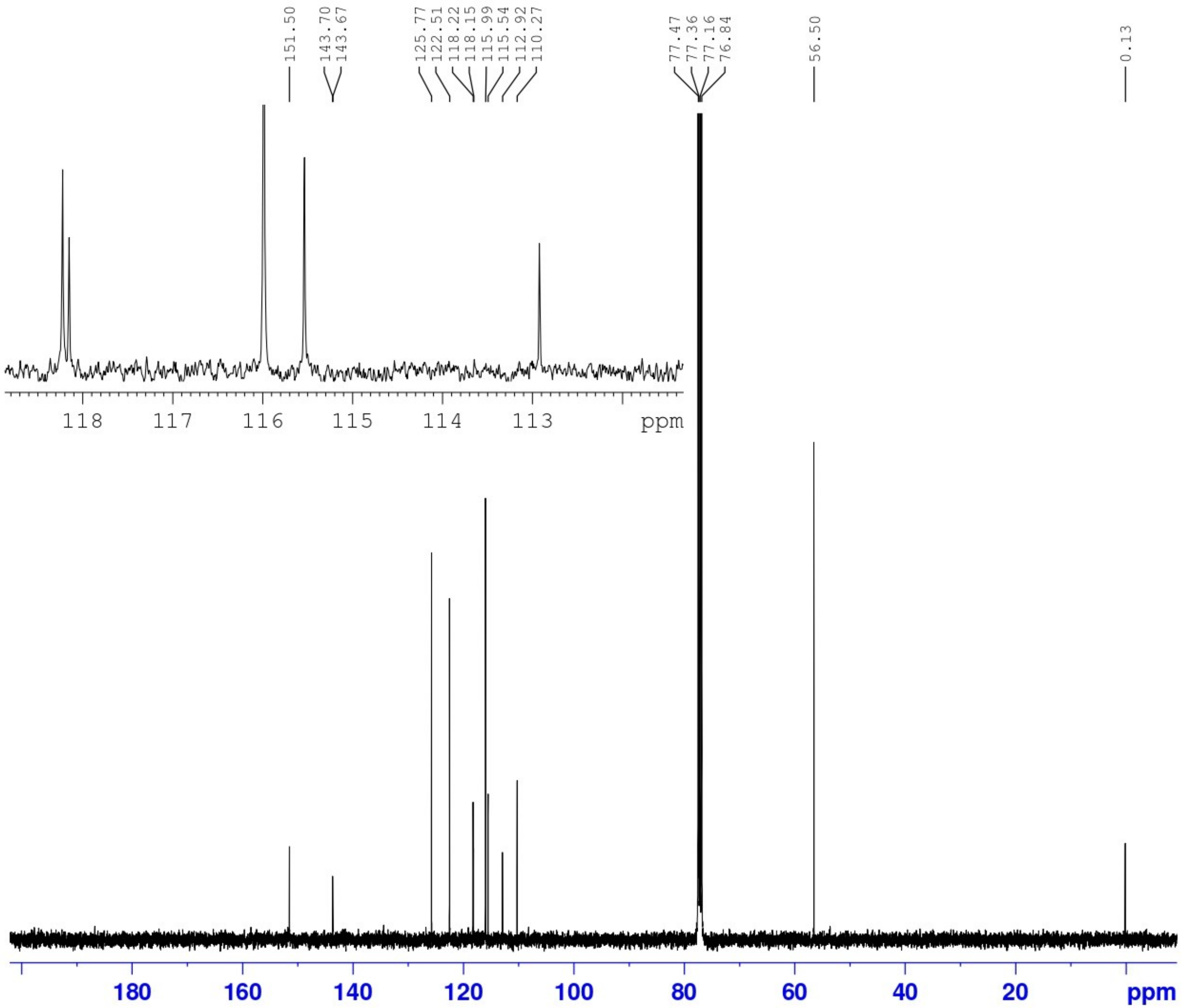

BRUKER

Current Data Parameters
NAME IV-Mn-126 i_12
EXPNO 2
PROCNO 1

F2 - Acquisition Parameters
Date_ 20240411
Time 21.07
INSTRUM Avance
PROBHD Z166552_0018 (PI HR-
PULPROG zgpg30
TD 65536
SOLVENT CDCl3
NS 2048
DS 4
SWH 23809.524
FIDRES 0.726609
AQ 1.3762560
RG 101
DW 21.000
DE 6.50
TE 298.0
D1 2.00000000
D11 0.03000000
TD0 1
SFO1 100.4895479
NUC1 13C
P0 2.67
P1 8.00
PLW1 88.22599792
SFO2 399.6015984
NUC2 1H
CPDPRG[2] waltz65
PCPD2 90.00
PLW2 21.19799995
PLW12 0.15922000
PLW13 0.08008700

F2 - Processing parameters
SI 32768
SF 100.4794864
WDW EM
SSB 0
LB 1.00
GB 0
PC 1.40

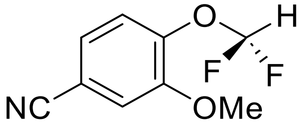

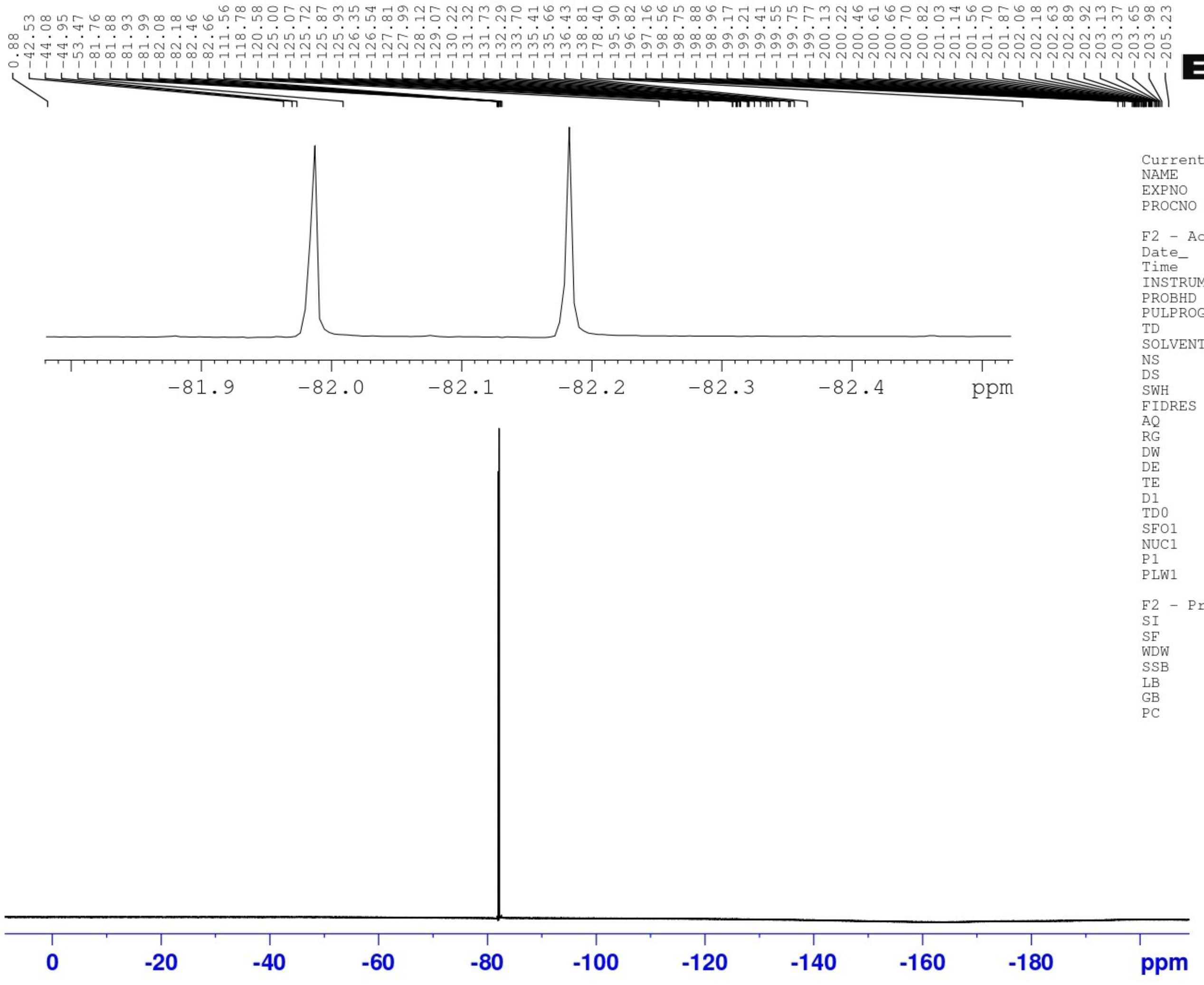

Current Data Parameters

NAME	IV-Mn-126 i_11
EXPNO	2
PROCNO	1

F2 - Acquisition Parameters

Date_	20240411
Time	13.40
INSTRUM	Avance
PROBHD	Z166552_0018 (PI HR-
PULPROG	zg
TD	131072
SOLVENT	CDCl3
NS	16
DS	4
SWH	90909.091
FIDRES	1.387163
AQ	0.7208960
RG	101
DW	5.500
DE	6.50
TE	298.0
D1	1.00000000
TD0	1
SFO1	375.9620680
NUC1	19F
P1	12.00
PLW1	32.47200012

F2 - Processing parameters

SI	65536
SF	375.9996680
WDW	EM
SSB	0
LB	0.30
GB	0
PC	1.00

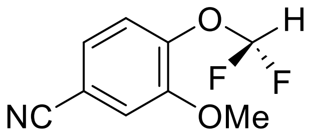

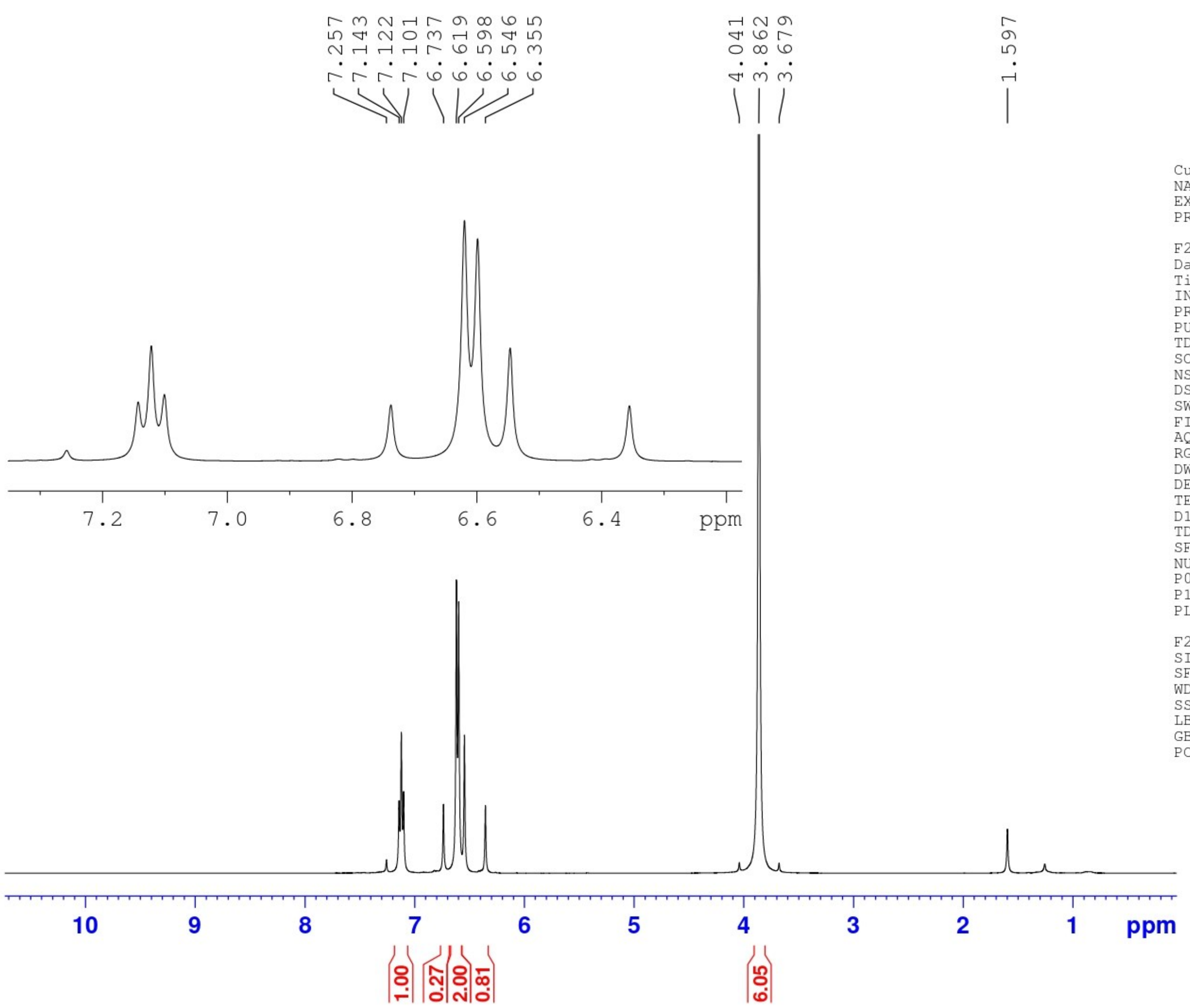
7.257
7.143
7.122
7.101
6.737
6.619
6.598
6.546
6.355
4.041
3.862
3.679
1.597
BRUKER
7.2 7.0 6.8 6.6 6.4 ppm
10 9 8 7 6 5 4 3 2 1 ppm
1.00 0.27 2.00 0.81 6.05
Current Data Parameters
NAME VI-Mn-84 i_10
EXPNO 2
PROCNO 1
F2 - Acquisition Parameters
Date_ 20241004
Time 9.16
INSTRUM Avance
PROBHD Z166552_0018 (PI HR-
PULPROG zg30
TD 65536
SOLVENT CDCl3
NS 16
DS 2
SWH 7812.500
FIDRES 0.238419
AQ 4.1943040
RG 90.5
DW 64.000
DE 6.67
TE 298.0
D1 1.00000000
TD0 1
SFO1 399.5701703
NUC1 1H
P0 2.60
P1 7.80
PLW1 21.19799995
F2 - Processing parameters
SI 65536
SF 399.5677134
WDW EM
SSB 0
LB 0.30
GB 0
PC 1.00
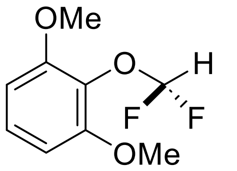
OMe
O
H
F
F
OMe

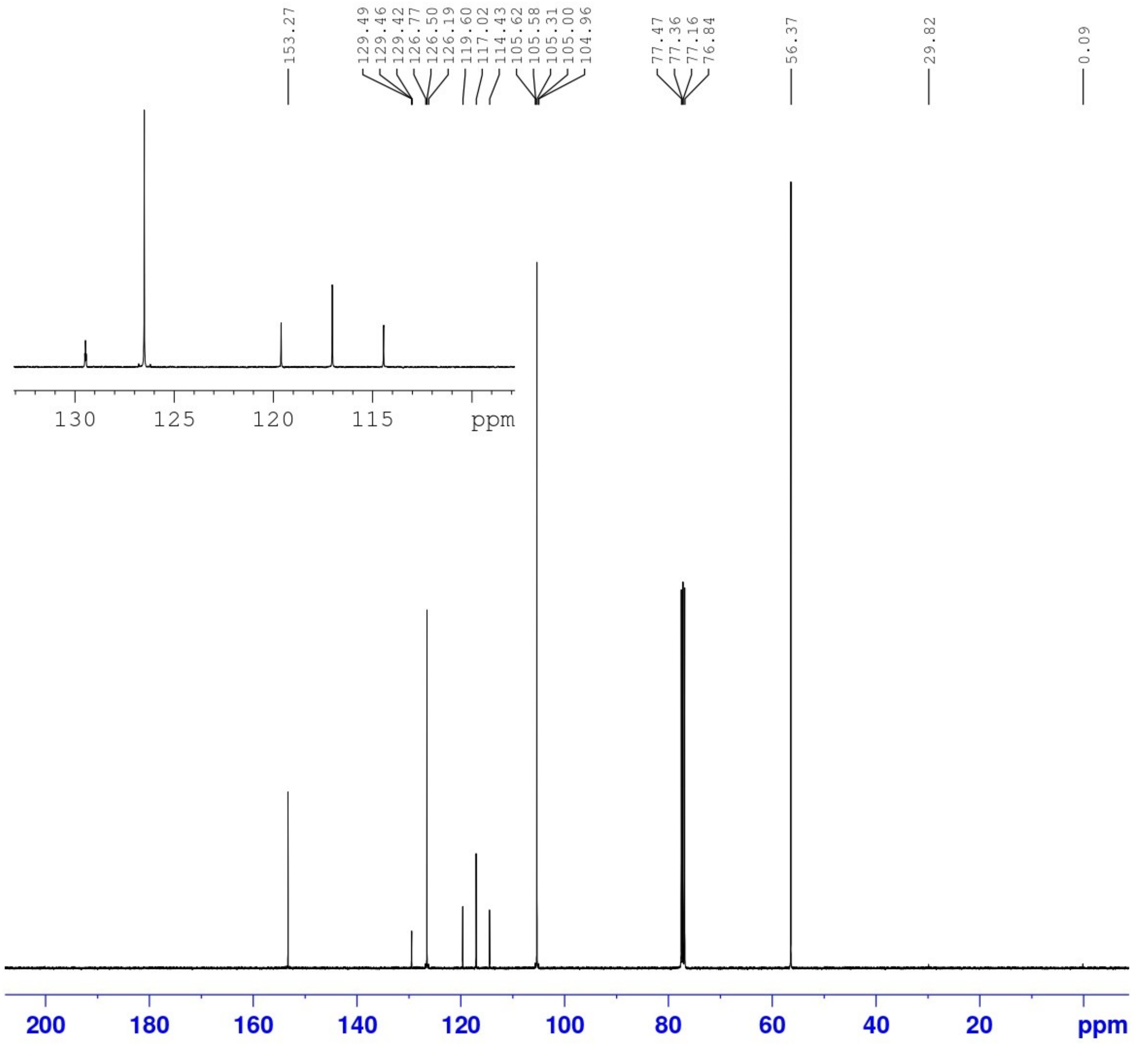

BRUKER

Current Data Parameters
NAME VI-Mn-84 i_12
EXPNO 1
PROCNO 1

F2 - Acquisition Parameters
Date_ 20241004
Time 20.05
INSTRUM Avance
PROBHD Z166552_0018 (PI HR-
PULPROG zgpg30
TD 65536
SOLVENT CDCl3
NS 2048
DS 4
SWH 23809.524
FIDRES 0.726609
AQ 1.3762560
RG 101
DW 21.000
DE 6.50
TE 298.0
D1 2.00000000
D11 0.03000000
TD0 1
SFO1 100.4814260
NUC1 13C
P0 2.67
P1 8.00
PLW1 88.22599792
SFO2 399.5693013
NUC2 1H
CPDPRG[2] waltz65
PCPD2 90.00
PLW2 21.19799995
PLW12 0.15922000
PLW13 0.08008700

F2 - Processing parameters
SI 32768
SF 100.4713681
WDW EM
SSB 0
LB 1.00
GB 0
PC 1.40

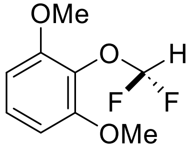

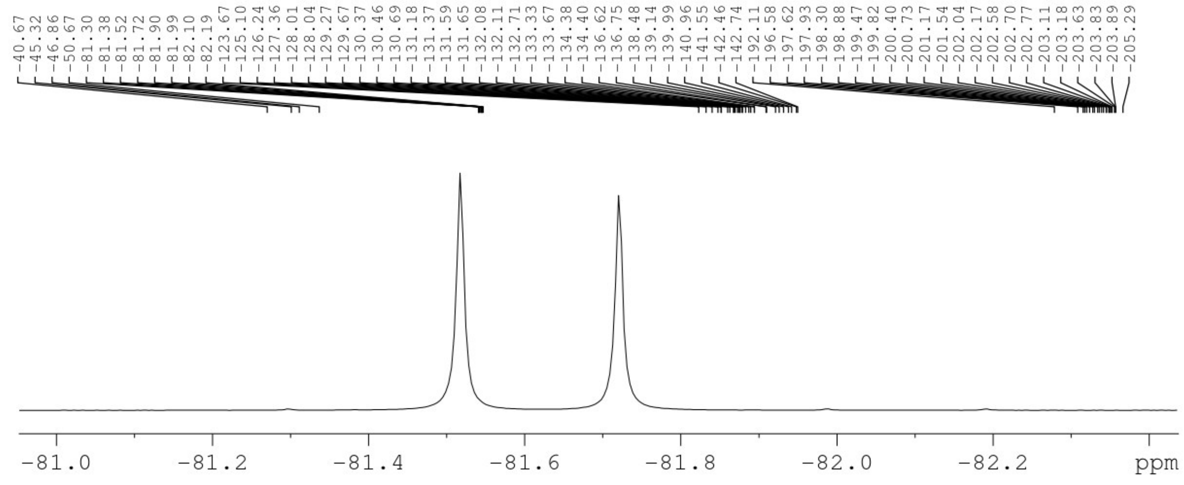

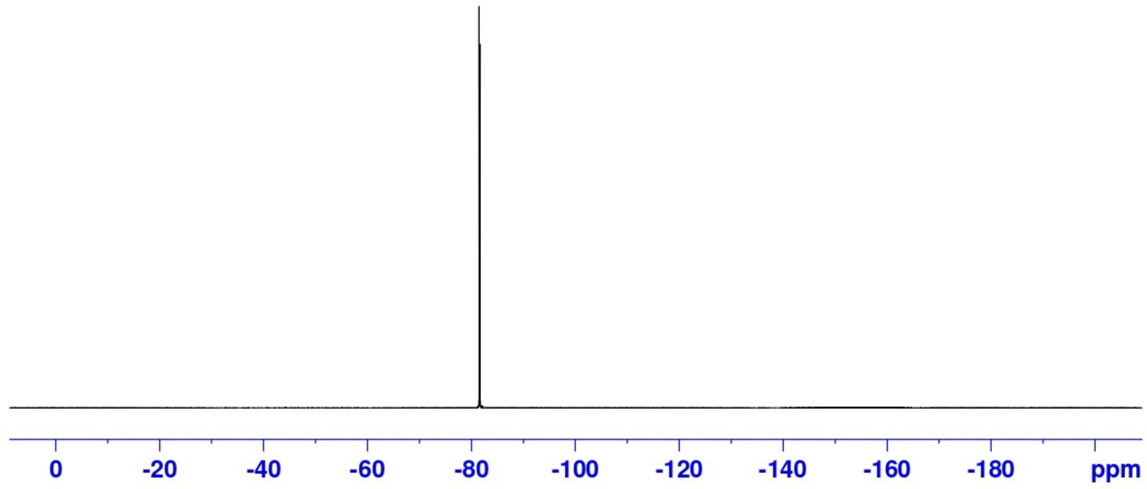

BRUKER

Current Data Parameters
NAME VI-Mn-84 i_11
EXPNO 2
PROCNO 1

F2 - Acquisition Parameters
Date_ 20241004
Time 9.18
INSTRUM Avance
PROBHD Z166552_0018 (PI HR-
PULPROG zg
TD 131072
SOLVENT CDCl3
NS 16
DS 4
SWH 90909.091
FIDRES 1.387163
AQ 0.7208960
RG 101
DW 5.500
DE 6.50
TE 298.0
D1 1.00000000
TD0 1
SFO1 375.9316815
NUC1 19F
P1 12.00
PLW1 32.47200012

F2 - Processing parameters
SI 65536
SF 375.9692784
WDW EM
SSB 0
LB 0.30
GB 0
PC 1.00

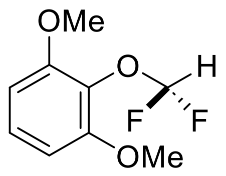

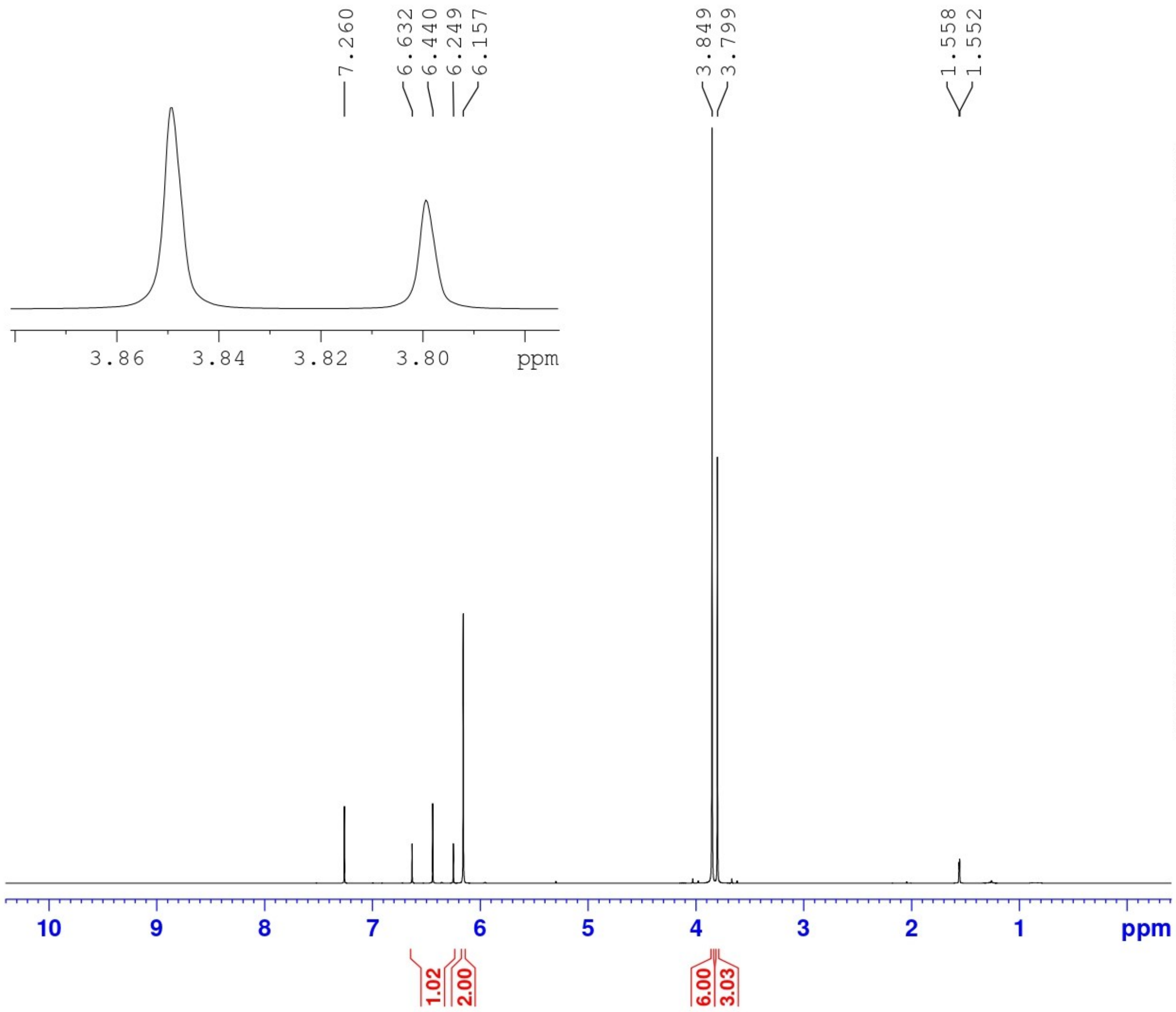

Current Data Parameters

NAME	VII-Mn-97 i (product
EXPNO	2
PROCNO	1

F2 - Acquisition Parameters

Date_	20250109
Time	18.04
INSTRUM	Avance
PROBHD	Z166552_0018 (PI HR-
PULPROG	zg30
TD	65536
SOLVENT	CDCl3
NS	16
DS	2
SWH	7812.500
FIDRES	0.238419
AQ	4.1943040
RG	101
DW	64.000
DE	6.67
TE	298.0
D1	1.00000000
TD0	1
SFO1	399.5701703
NUC1	1H
P0	2.60
P1	7.80
PLW1	21.19799995

F2 - Processing parameters

SI	65536
SF	399.5677127
WDW	EM
SSB	0
LB	0.30
GB	0
PC	1.00

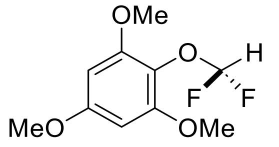

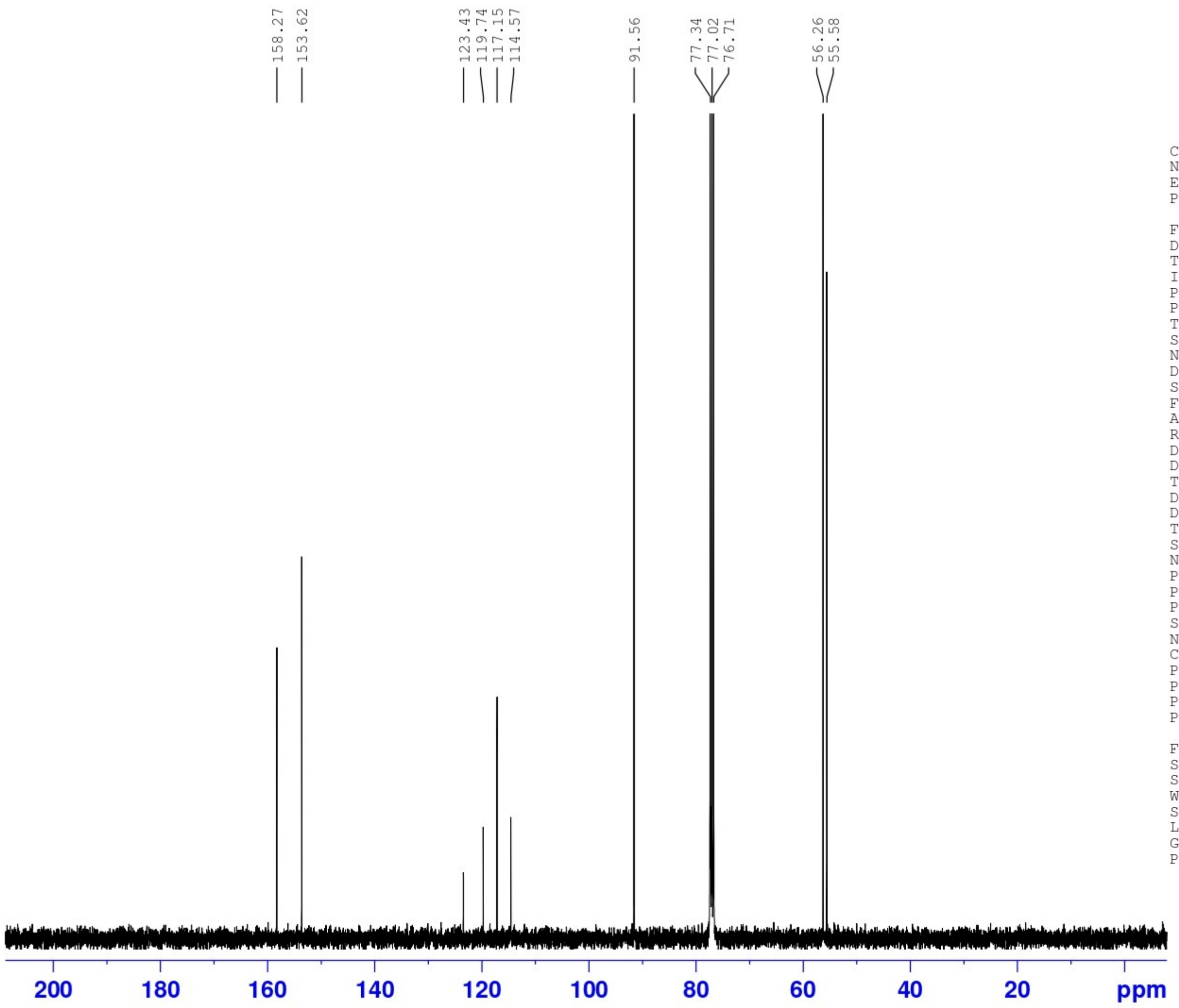

BRUKER

Current Data Parameters
NAME VII-Mn-97 i (product
EXPNO 2
PROCNO 1

F2 - Acquisition Parameters
Date_ 20250109
Time 19.06
INSTRUM Avance
PROBHD Z166552_0018 (PI HR-
PULPROG zgpg30
TD 65536
SOLVENT CDCl3
NS 1024
DS 4
SWH 23809.524
FIDRES 0.726609
AQ 1.3762560
RG 101
DW 21.000
DE 6.50
TE 298.0
D1 2.00000000
D11 0.03000000
TD0 1
SFO1 100.4814260
NUC1 13C
P0 2.67
P1 8.00
PLW1 88.22599792
SFO2 399.5693013
NUC2 1H
CPDPRG[2] waltz65
PCPD2 90.00
PLW2 21.19799995
PLW12 0.15922000
PLW13 0.08008700

F2 - Processing parameters
SI 32768
SF 100.4713788
WDW EM
SSB 0
LB 1.00
GB 0
PC 1.40

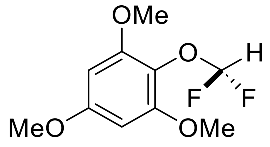

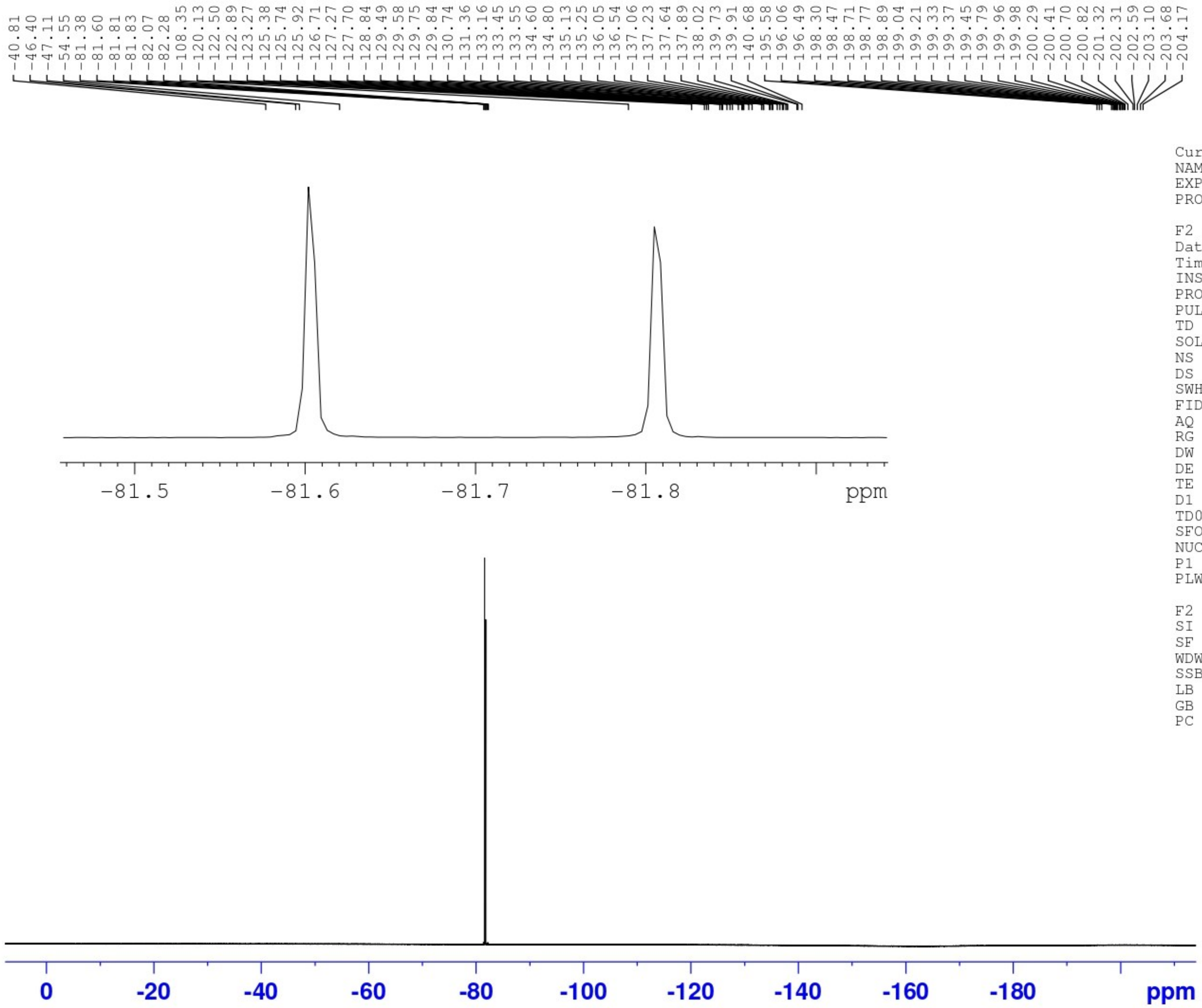

Current Data Parameters
NAME VII-Mn-97 i (product
EXPNO 2
PROCNO 1

F2 - Acquisition Parameters
Date_ 20250109
Time 18.06
INSTRUM Avance
PROBHD Z166552_0018 (PI HR-
PULPROG zg
TD 131072
SOLVENT CDCl3
NS 16
DS 4
SWH 90909.091
FIDRES 1.387163
AQ 0.7208960
RG 101
DW 5.500
DE 6.50
TE 298.0
D1 1.00000000
TD0 1
SFO1 375.9316815
NUC1 19F
P1 12.00
PLW1 32.47200012

F2 - Processing parameters
SI 65536
SF 375.9692784
WDW EM
SSB 0
LB 0.30
GB 0
PC 1.00

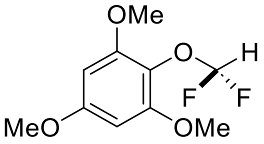

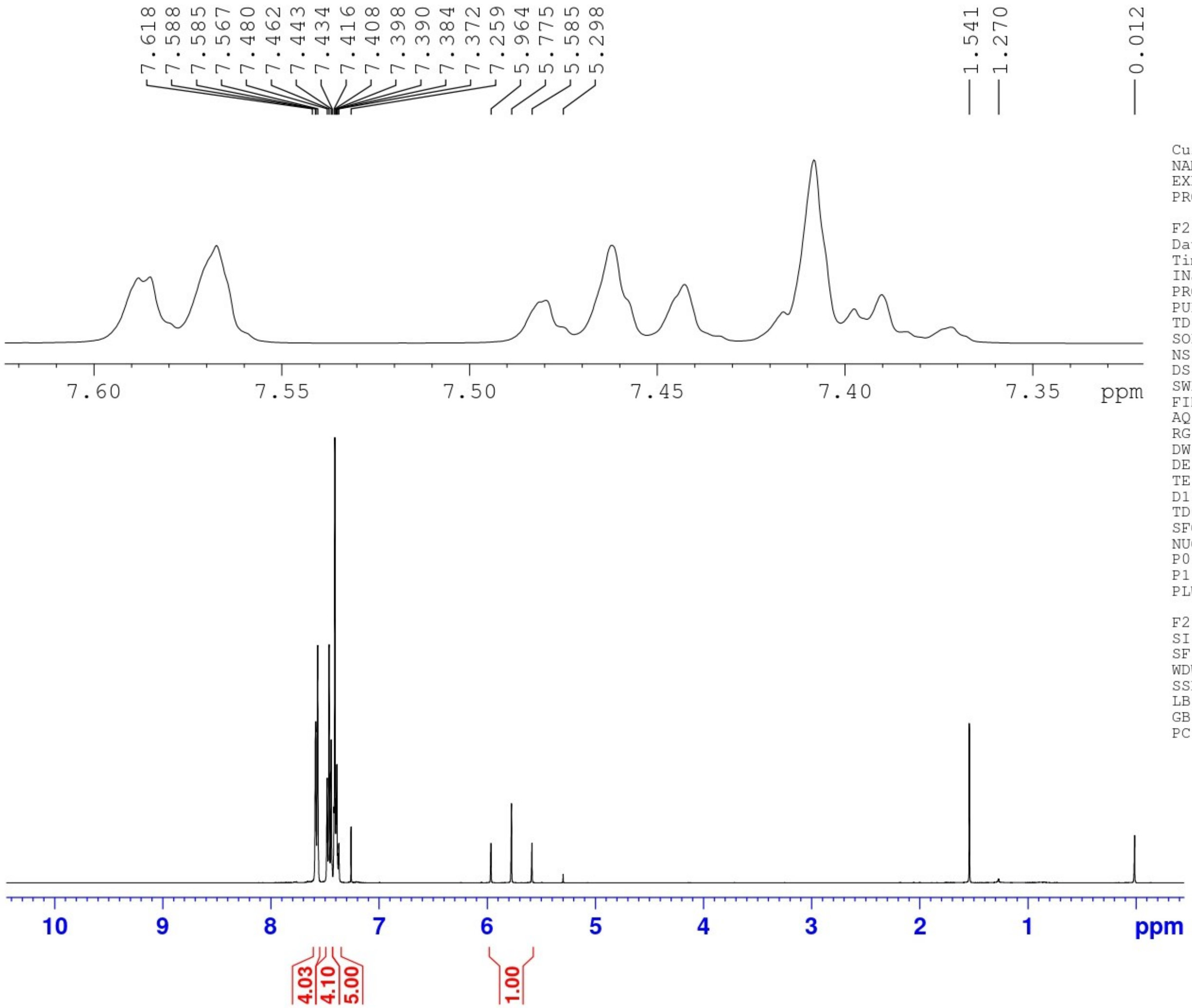

Current Data Parameters
NAME VI-Mn-92 i Ph_10
EXPNO 2
PROCNO 1

F2 - Acquisition Parameters
Date_ 20241009
Time 15.20
INSTRUM Avance
PROBHD Z166552_0018 (PI HR-
PULPROG zg30
TD 65536
SOLVENT CDCl3
NS 16
DS 2
SWH 7812.500
FIDRES 0.238419
AQ 4.1943040
RG 101
DW 64.000
DE 6.67
TE 298.0
D1 1.00000000
TD0 1
SFO1 399.5701703
NUC1 1H
P0 2.60
P1 7.80
PLW1 21.19799995

F2 - Processing parameters
SI 65536
SF 399.5677131
WDW EM
SSB 0
LB 0.30
GB 0
PC 1.00

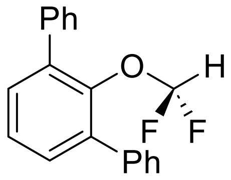

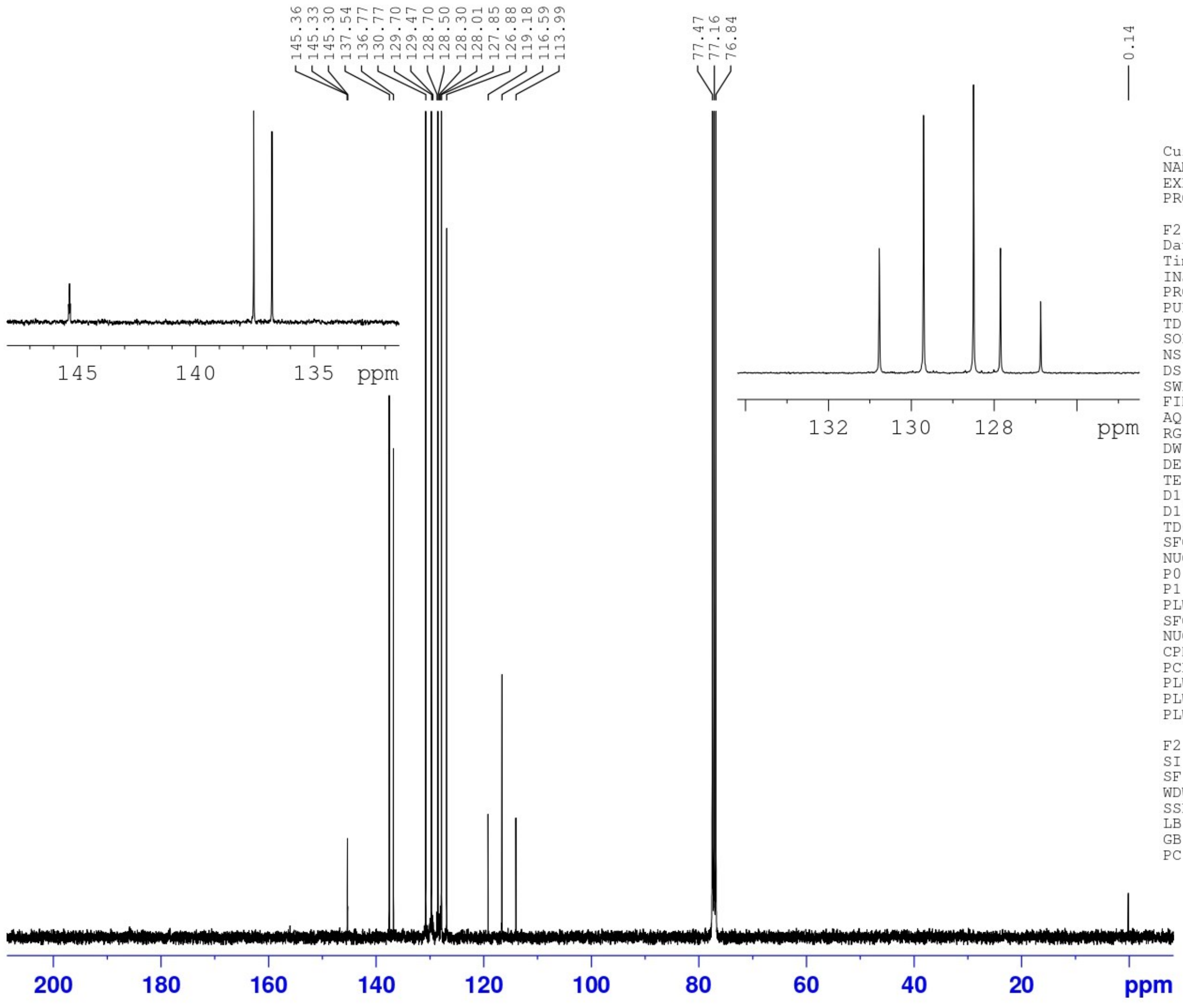

BRUKER

Current Data Parameters
NAME VI-Mn-92 i Ph_12
EXPNO 2
PROCNO 1

F2 - Acquisition Parameters
Date_ 20241009
Time 21.06
INSTRUM Avance
PROBHD Z166552_0018 (PI HR-
PULPROG zgpg30
TD 65536
SOLVENT CDCl3
NS 2048
DS 4
SWH 23809.524
FIDRES 0.726609
AQ 1.3762560
RG 101
DW 21.000
DE 6.50
TE 298.0
D1 2.00000000
D11 0.03000000
TD0 1
SFO1 100.4814260
NUC1 13C
P0 2.67
P1 8.00
PLW1 88.22599792
SFO2 399.5693013
NUC2 1H
CPDPRG[2] waltz65
PCPD2 90.00
PLW2 21.19799995
PLW12 0.15922000
PLW13 0.08008700

F2 - Processing parameters
SI 32768
SF 100.4713665
WDW EM
SSB 0
LB 1.00
GB 0
PC [illegible]

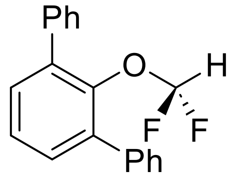

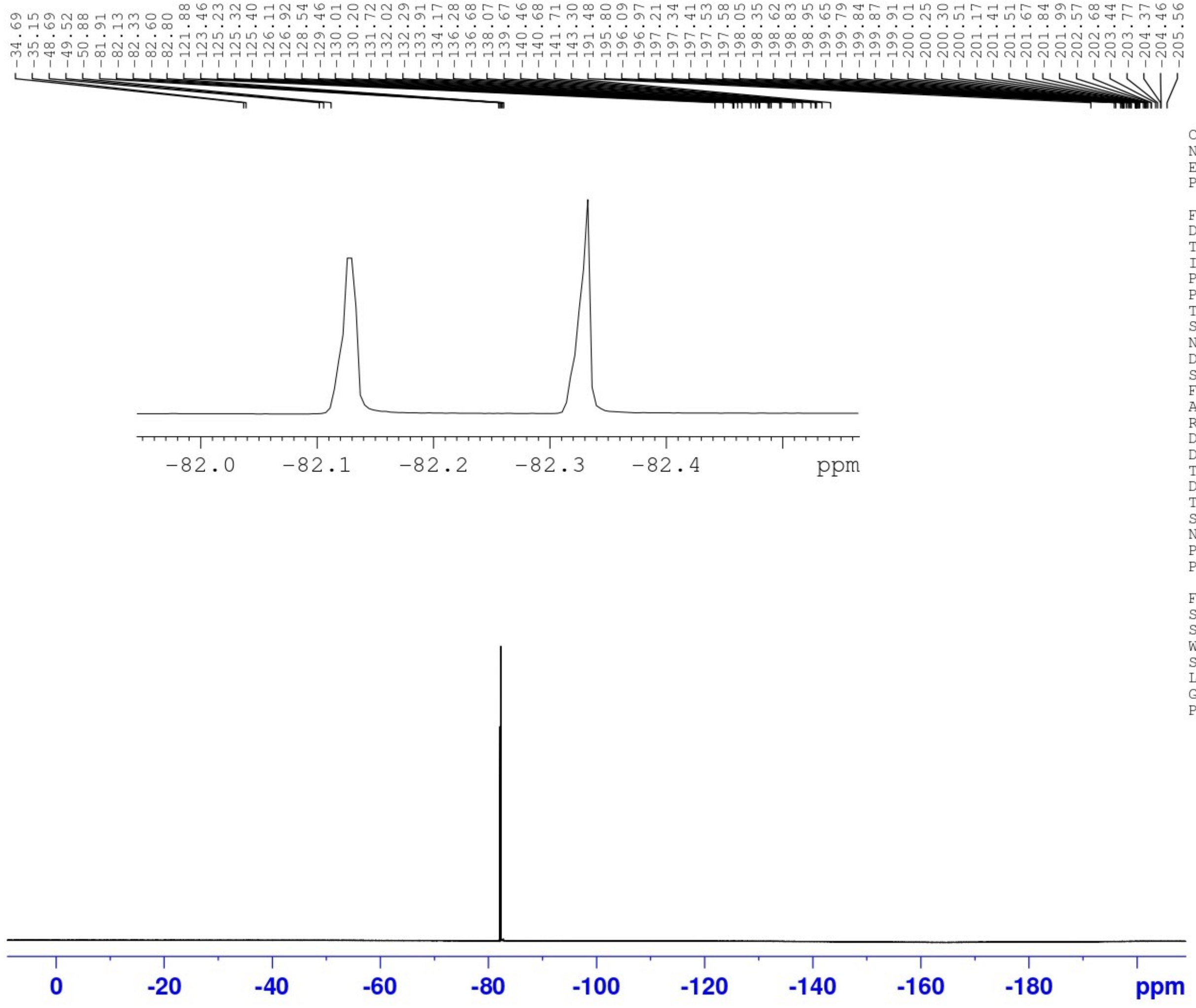

BRUKER

Current Data Parameters

NAME	VI-Mn-92 i Ph_11
EXPNO	2
PROCNO	1

F2 - Acquisition Parameters

Date_	20241009
Time	15.22
INSTRUM	Avance
PROBHD	Z166552_0018 (PI HR-
PULPROG	zg
TD	131072
SOLVENT	CDCl3
NS	16
DS	4
SWH	90909.091
FIDRES	1.387163
AQ	0.7208960
RG	101
DW	5.500
DE	6.50
TE	298.0
D1	1.00000000
TD0	1
SFO1	375.9316815
NUC1	19F
P1	12.00
PLW1	32.47200012

F2 - Processing parameters

SI	65536
SF	375.9692784
WDW	EM
SSB	0
LB	0.30
GB	0
PC	1.00

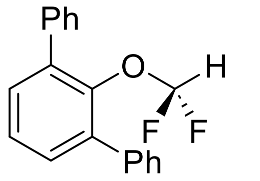

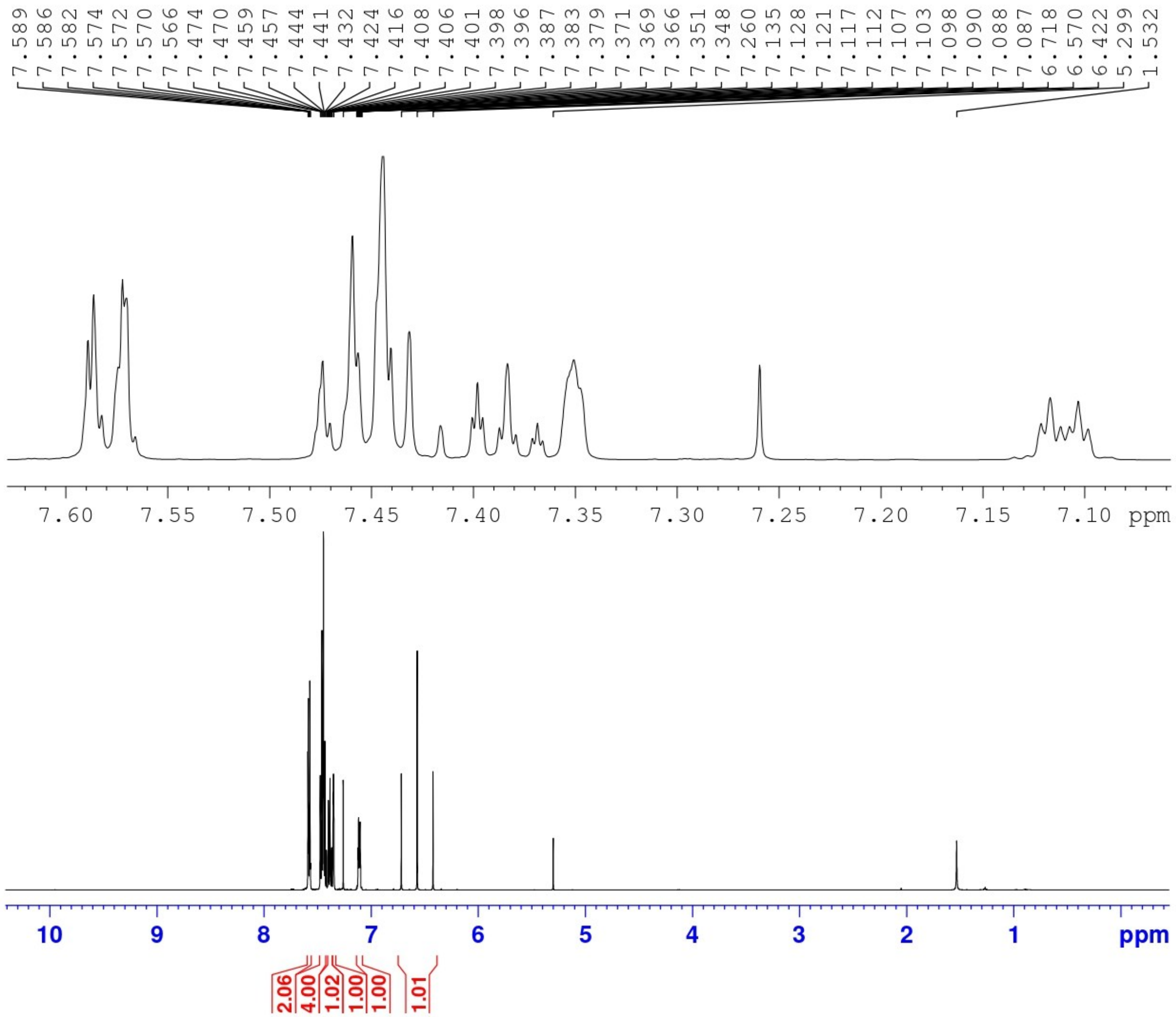

Current Data Parameters

NAME	III-MN-24 i_10
EXPNO	4
PROCNO	1

F2 - Acquisition Parameters

Date_	20230906
Time	16.18
INSTRUM	AS500-NEO
PROBHD	Z168772_0026 (CPP1.1
PULPROG	zg30
TD	65536
SOLVENT	CDCl3
NS	16
DS	2
SWH	10000.000
FIDRES	0.305176
AQ	3.2767999
RG	45.2
DW	50.000
DE	10.45
TE	300.0
D1	1.00000000
TD0	1
SFO1	499.7860862
NUC1	1H
P0	4.00
P1	12.00
PLW1	16.91500092

F2 - Processing parameters

SI	65536
SF	499.7830121
WDW	EM
SSB	0
LB	0.30
GB	0
PC	1.00

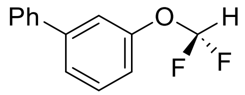

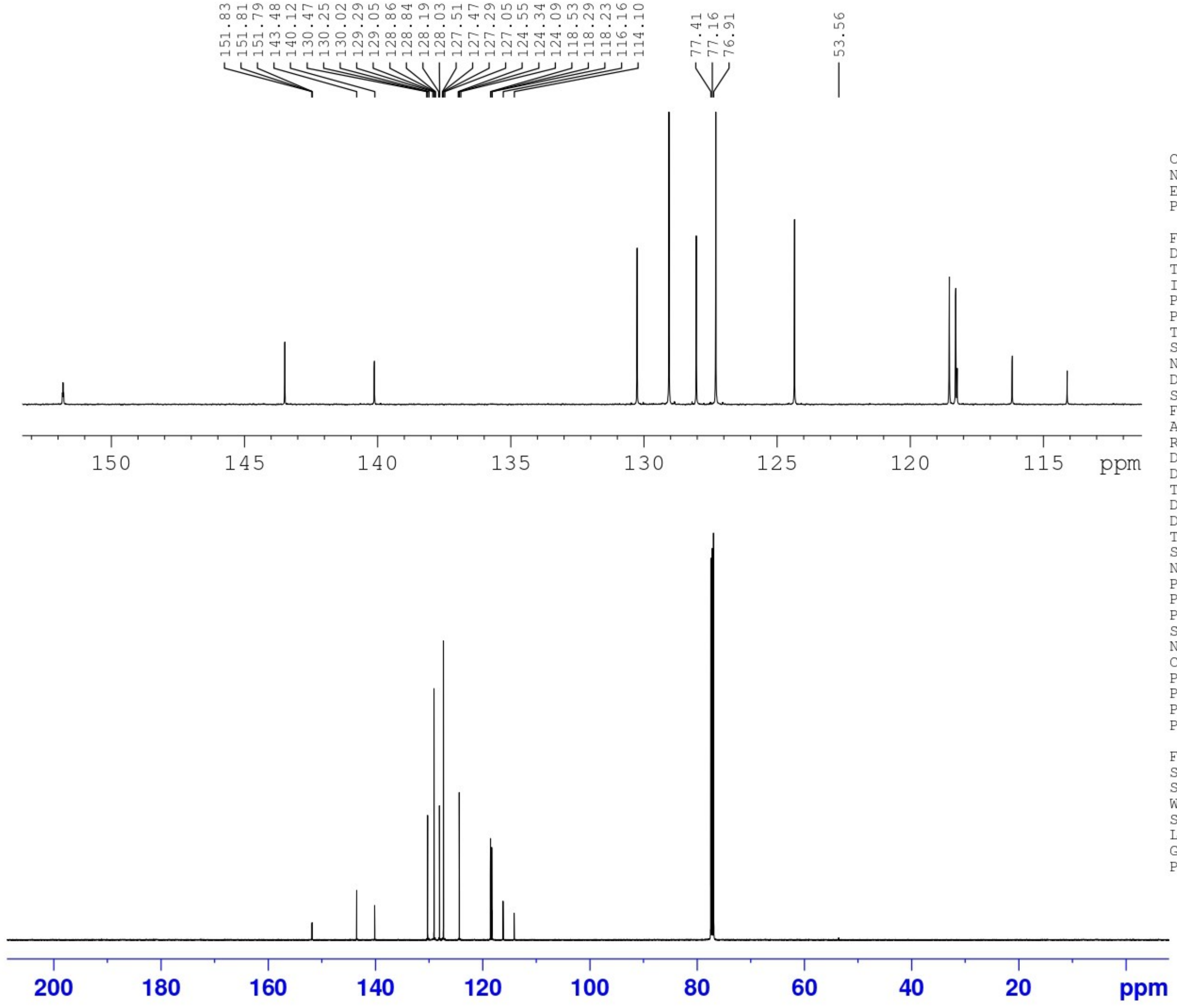

Current Data Parameters
NAME III-MN-24 i_12
EXPNO 3
PROCNO 1

F2 - Acquisition Parameters
Date_ 20230907
Time 4.07
INSTRUM AS500-NEO
PROBHD Z168772_0026 (CPP1.1
PULPROG zgpg30
TD 65536
SOLVENT CDCl3
NS 2048
DS 4
SWH 30120.482
FIDRES 0.919204
AQ 1.0878977
RG 101
DW 16.600
DE 18.00
TE 300.0
D1 2.00000000
D11 0.03000000
TD0 1
SFO1 125.6831024
NUC1 13C
P0 3.33
P1 10.00
PLW1 59.16400146
SFO2 499.7849991
NUC2 1H
CPDPRG[2] waltz65
PCPD2 80.00
PLW2 16.91500092
PLW12 0.38058999
PLW13 0.19113000

F2 - Processing parameters
SI 32768
SF 125.6705177
WDW EM
SSB 0
LB 1.00
GB 0
PC 1.40

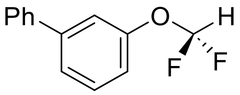

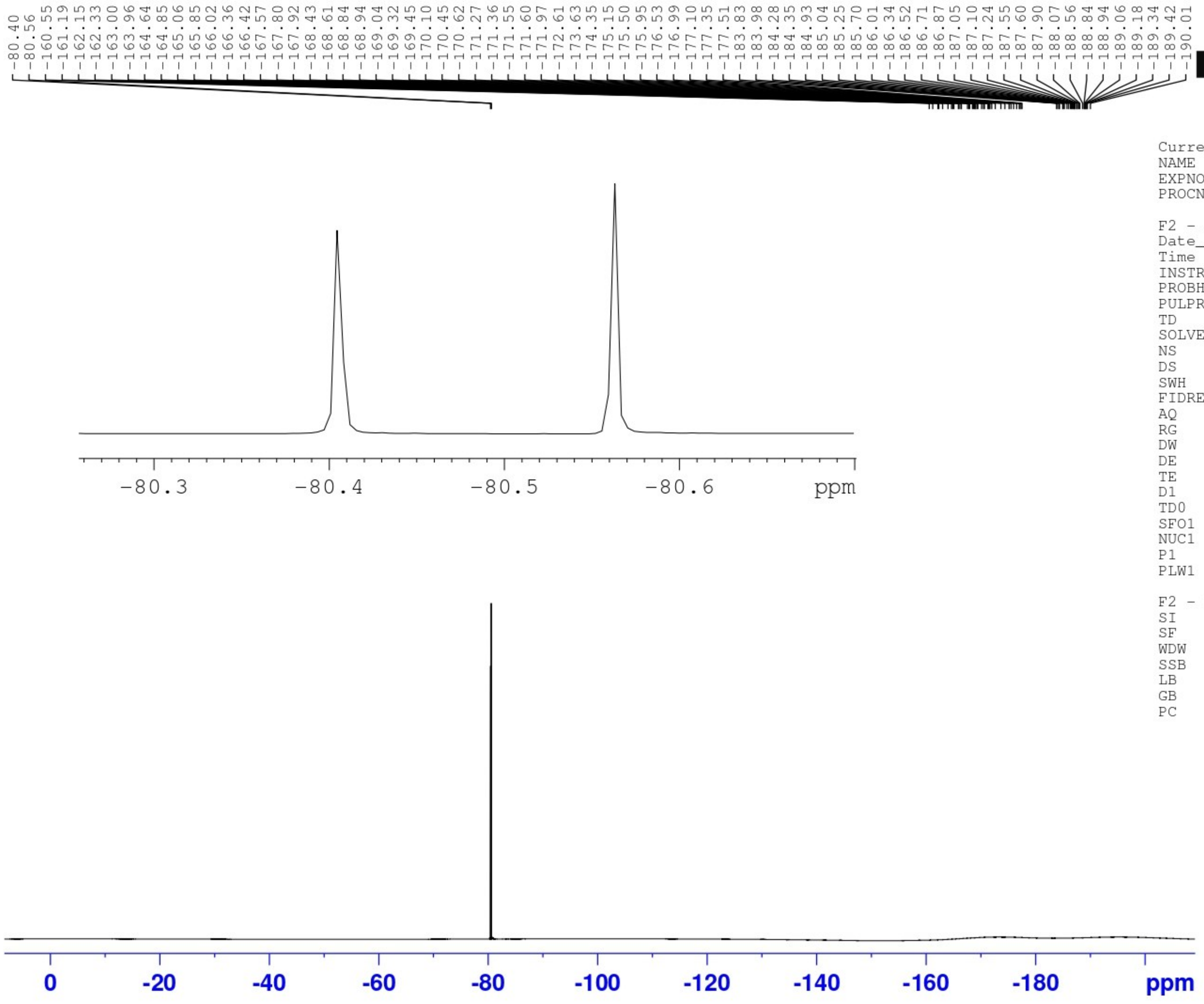

Current Data Parameters
NAME III-MN-24 i_11
EXPNO 5
PROCNO 1

F2 - Acquisition Parameters
Date_ 20230906
Time 16.20
INSTRUM AS500-NEO
PROBHD Z168772_0026 (CPP1.1
PULPROG zg
TD 131072
SOLVENT CDCl3
NS 16
DS 4
SWH 113636.364
FIDRES 1.733953
AQ 0.5767168
RG 10
DW 4.400
DE 18.00
TE 300.0
D1 1.00000000
TD0 1
SFO1 470.2188444
NUC1 19F
P1 15.00
PLW1 10.89000034

F2 - Processing parameters
SI 65536
SF 470.2658710
WDW EM
SSB 0
LB 0.30
GB 0
PC 1.00

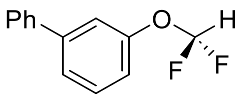

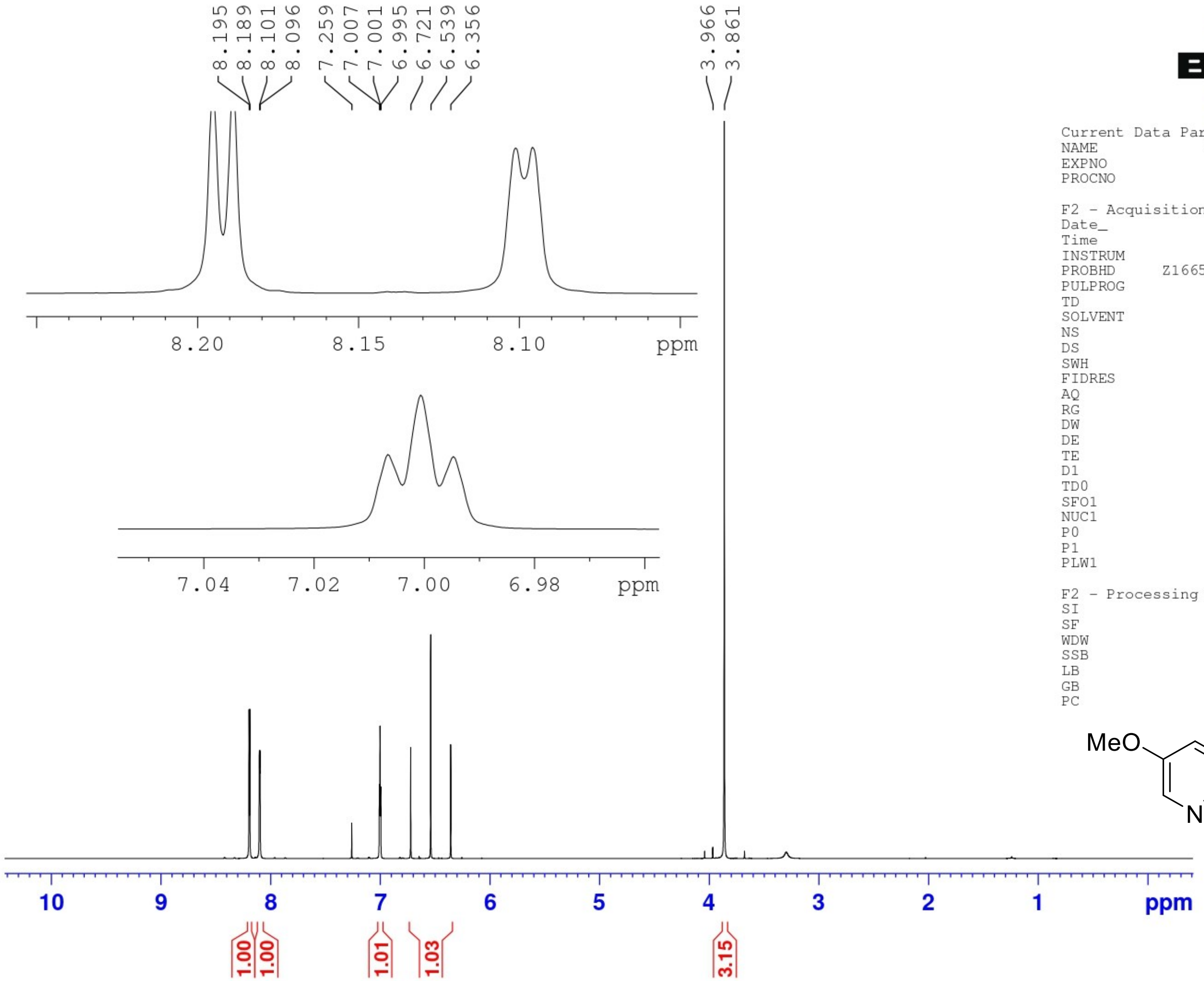

Current Data Parameters
NAME IX-Mn-36i re_10
EXPNO 1
PROCNO 1

F2 - Acquisition Parameters
Date_ 20251103
Time 16.43
INSTRUM Avance
PROBHD Z166552_0018 (PI HR-
PULPROG zg30
TD 65536
SOLVENT CDCl3
NS 16
DS 2
SWH 7812.500
FIDRES 0.238419
AQ 4.1943040
RG 101
DW 64.000
DE 6.67
TE 298.0
D1 1.00000000
TD0 1
SFO1 399.5424672
NUC1 1H
P0 2.60
P1 7.80
PLW1 21.19799995

F2 - Processing parameters
SI 65536
SF 399.5400099
WDW EM
SSB 0
LB 0.30
GB 0
PC 1.00

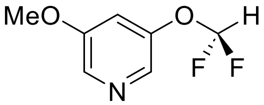

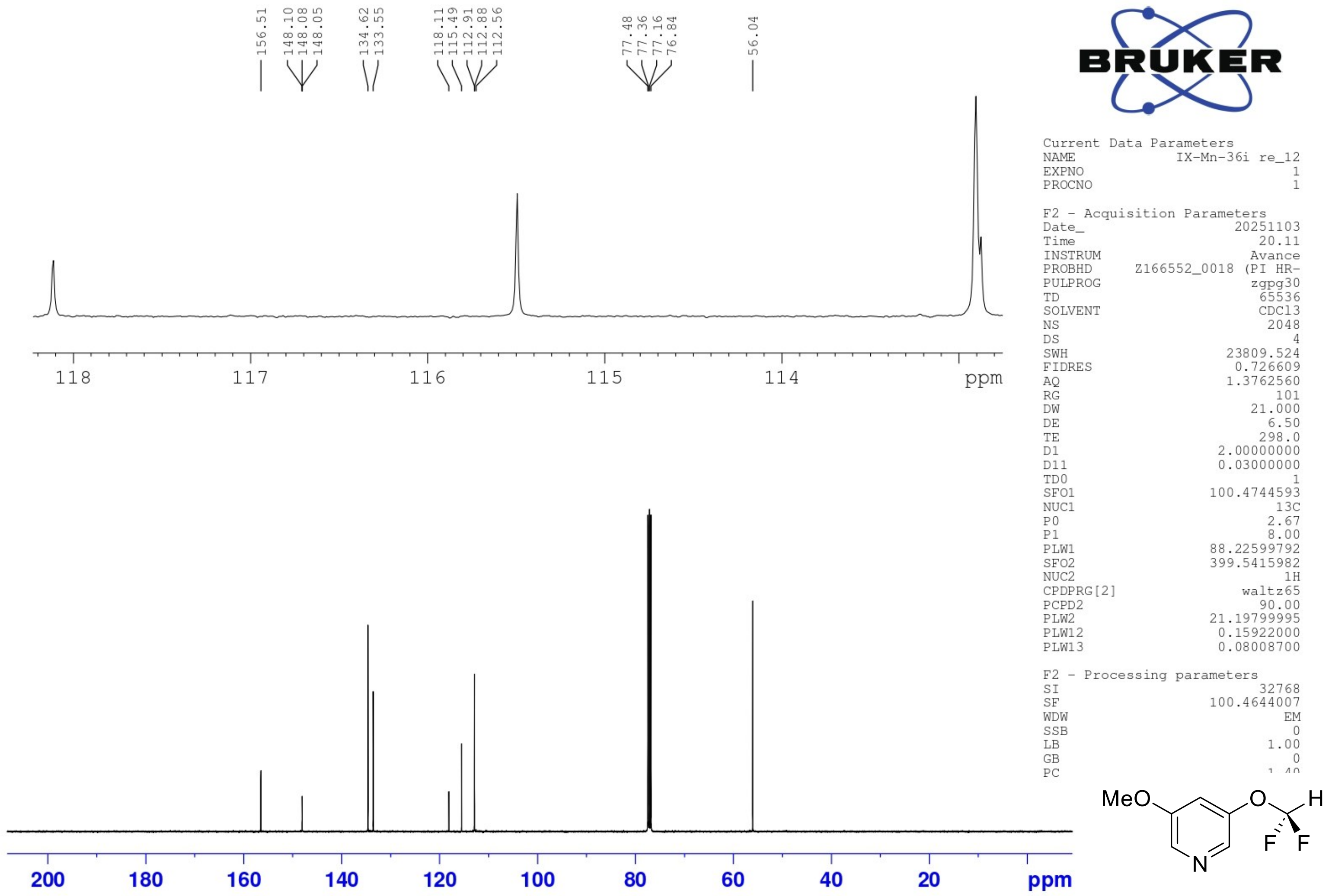

156.51
148.10
148.08
148.05
134.62
133.55
118.11
115.49
112.91
112.88
112.56
77.48
77.36
77.16
76.84
56.04
118
117
116
115
114
ppm
BRUKER
Current Data Parameters
NAME IX-Mn-36i re_12
EXPNO 1
PROCNO 1
F2 - Acquisition Parameters
Date_ 20251103
Time 20.11
INSTRUM Avance
PROBHD Z166552_0018 (PI HR-
PULPROG zgpg30
TD 65536
SOLVENT CDCl3
NS 2048
DS 4
SWH 23809.524
FIDRES 0.726609
AQ 1.3762560
RG 101
DW 21.000
DE 6.50
TE 298.0
D1 2.00000000
D11 0.03000000
TD0 1
SFO1 100.4744593
NUC1 13C
P0 2.67
P1 8.00
PLW1 88.22599792
SFO2 399.5415982
NUC2 1H
CPDPRG[2] waltz65
PCPD2 90.00
PLW2 21.19799995
PLW12 0.15922000
PLW13 0.08008700
F2 - Processing parameters
SI 32768
SF 100.4644007
WDW EM
SSB 0
LB 1.00
GB 0
PC
200
180
160
140
120
100
80
60
40
20
ppm
MeO
O
H
F
F
N

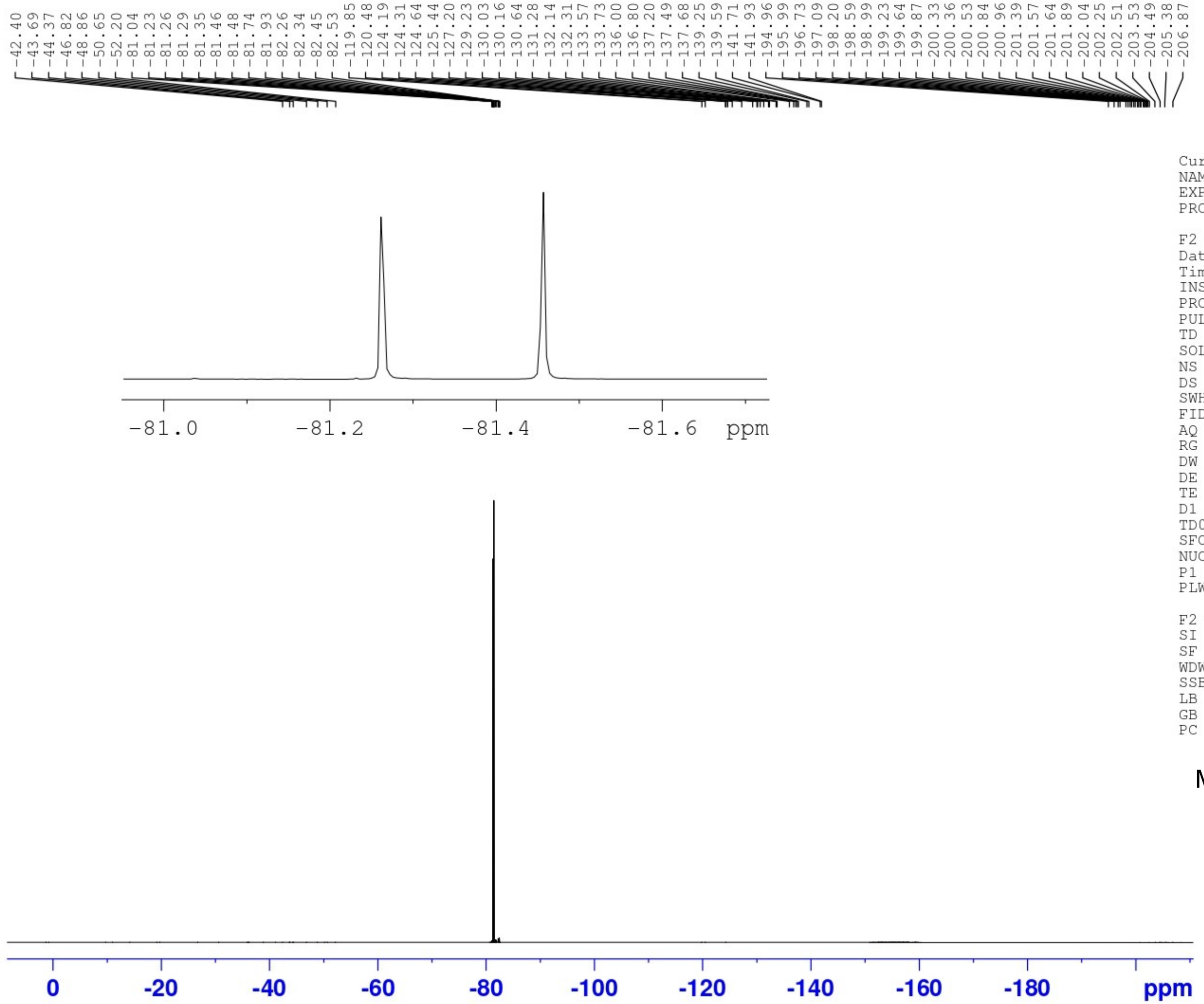

Current Data Parameters
NAME IX-Mn-36i re_11
EXPNO 1
PROCNO 1

F2 - Acquisition Parameters
Date_ 20251103
Time 16.45
INSTRUM Avance
PROBHD Z166552_0018 (PI HR-
PULPROG zg
TD 131072
SOLVENT CDCl3
NS 16
DS 4
SWH 90909.091
FIDRES 1.387163
AQ 0.7208960
RG 101
DW 5.500
DE 6.50
TE 298.0
D1 1.00000000
TD0 1
SFO1 375.9056172
NUC1 19F
P1 12.00
PLW1 32.47200012

F2 - Processing parameters
SI 65536
SF 375.9432115
WDW EM
SSB 0
LB 0.30
GB 0
PC 1.00

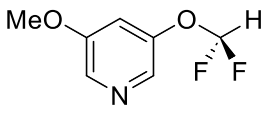

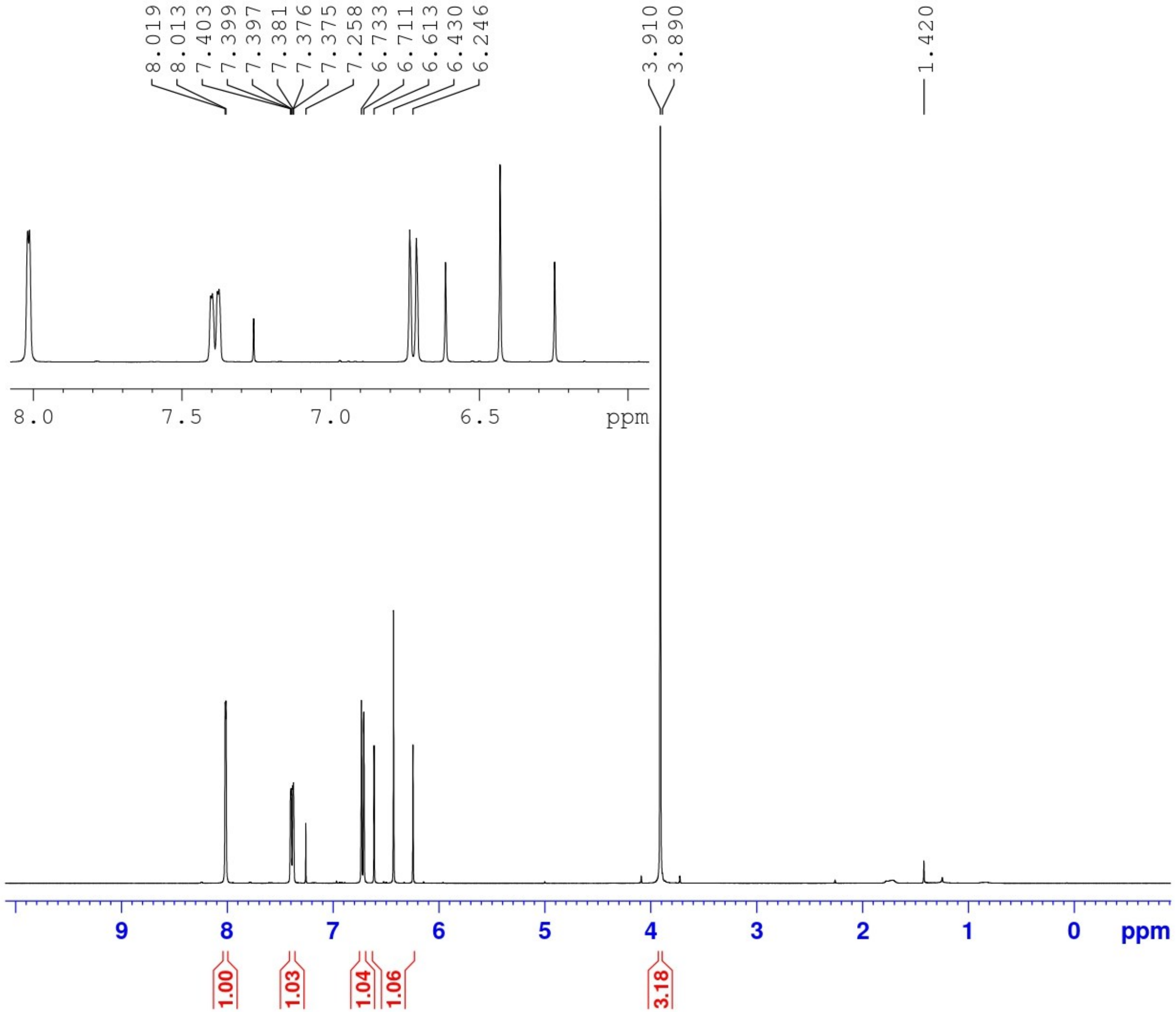

Current Data Parameters
NAME VIII-Mn-40i_10
EXPNO 2
PROCNO 1

F2 - Acquisition Parameters
Date_ 20250226
Time 11.04
INSTRUM Avance
PROBHD Z166552_0018 (PI HR-
PULPROG zg30
TD 65536
SOLVENT CDCl3
NS 16
DS 2
SWH 7812.500
FIDRES 0.238419
AQ 4.1943040
RG 90.5
DW 64.000
DE 6.67
TE 298.0
D1 1.00000000
TD0 1
SFO1 399.5701703
NUC1 1H
P0 2.60
P1 7.80
PLW1 21.19799995

F2 - Processing parameters
SI 65536
SF 399.5677132
WDW EM
SSB 0
LB 0.30
GB 0
PC 1.00

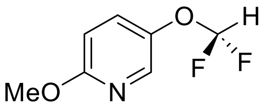

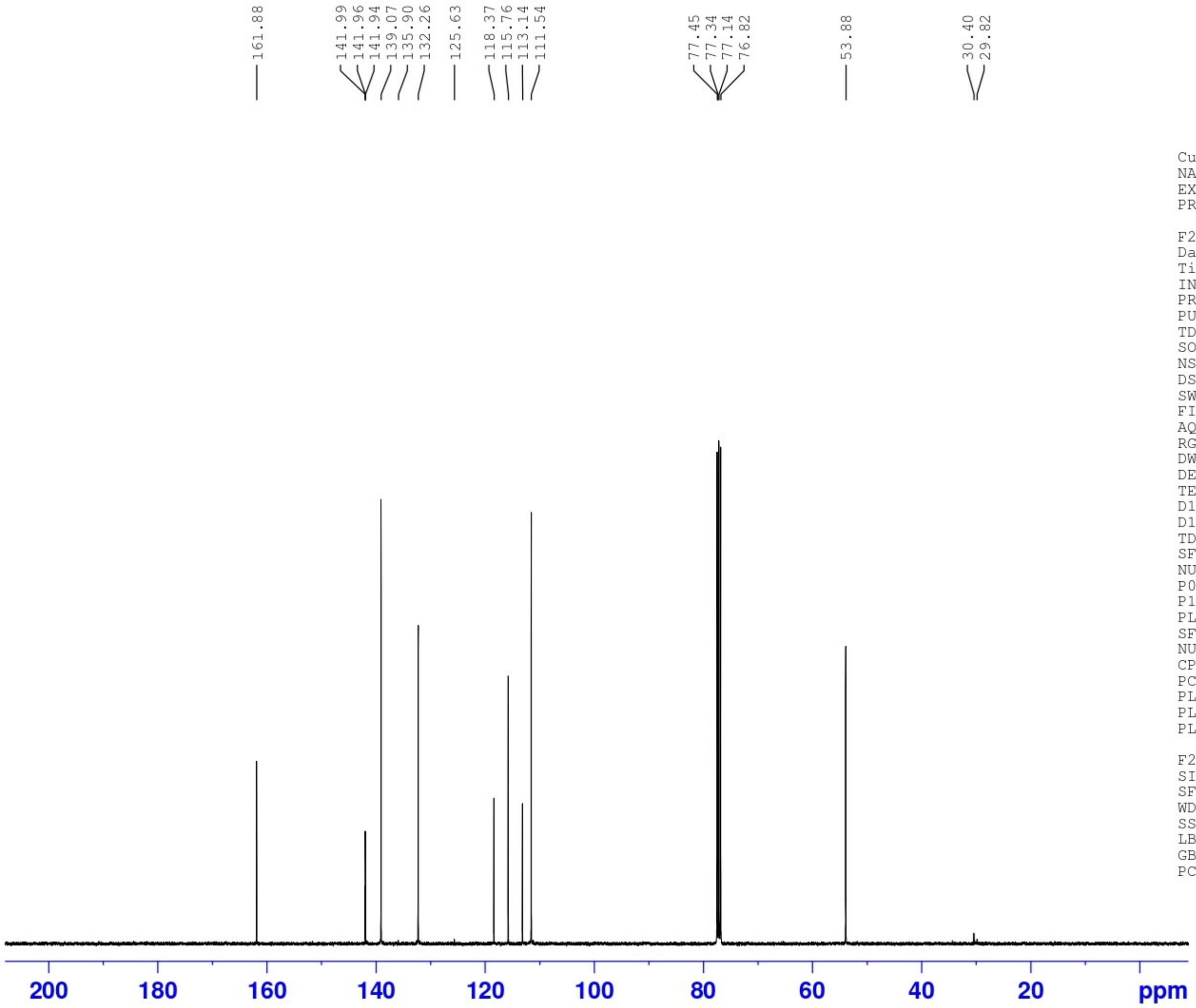

Current Data Parameters
NAME VIII-Mn-40 i re_12
EXPNO 1
PROCNO 1

F2 - Acquisition Parameters
Date_ 20250924
Time 23.26
INSTRUM Avance
PROBHD Z166552_0018 (PI HR-
PULPROG zgpg30
TD 65536
SOLVENT CDCl3
NS 2048
DS 4
SWH 23809.524
FIDRES 0.726609
AQ 1.3762560
RG 101
DW 21.000
DE 6.50
TE 298.0
D1 2.00000000
D11 0.03000000
TD0 1
SFO1 100.4744593
NUC1 13C
P0 2.67
P1 8.00
PLW1 88.22599792
SFO2 399.5415982
NUC2 1H
CPDPRG[2] waltz65
PCPD2 90.00
PLW2 21.19799995
PLW12 0.15922000
PLW13 0.08008700

F2 - Processing parameters
SI 32768
SF 100.4644008
WDW EM
SSB 0
LB 1.00
GB 0
PC 1.40

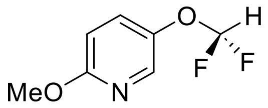

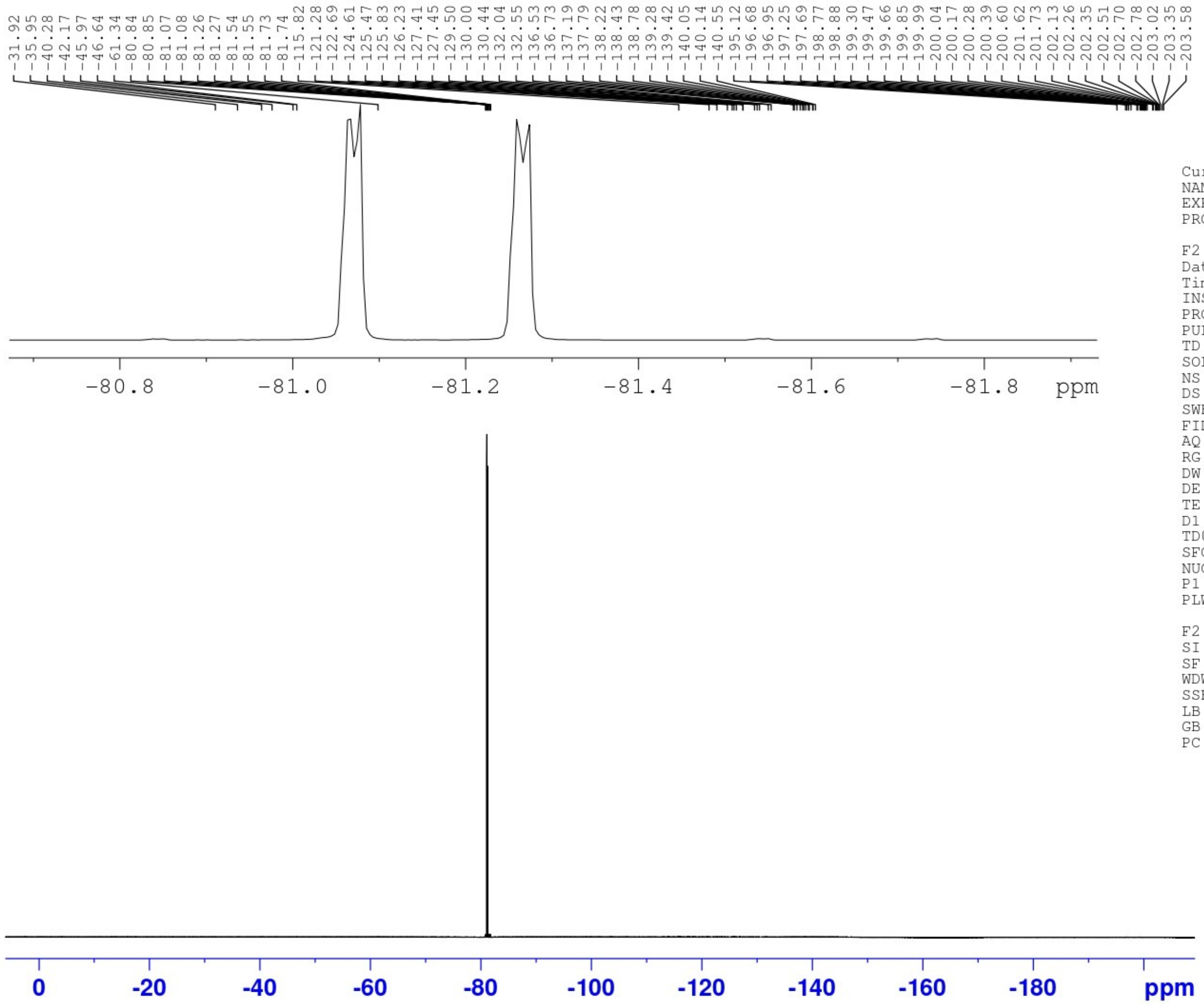

BRUKER

Current Data Parameters
NAME VIII-Mn-40i_11
EXPNO 2
PROCNO 1

F2 - Acquisition Parameters
Date_ 20250226
Time 11.06
INSTRUM Avance
PROBHD Z166552_0018 (PI HR-
PULPROG zg
TD 131072
SOLVENT CDCl3
NS 16
DS 4
SWH 90909.091
FIDRES 1.387163
AQ 0.7208960
RG 101
DW 5.500
DE 6.50
TE 298.0
D1 1.00000000
TD0 1
SFO1 375.9316815
NUC1 19F
P1 12.00
PLW1 32.47200012

F2 - Processing parameters
SI 65536
SF 375.9692784
WDW EM
SSB 0
LB 0.30
GB 0
PC 1.00

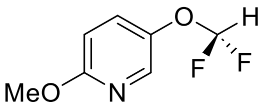

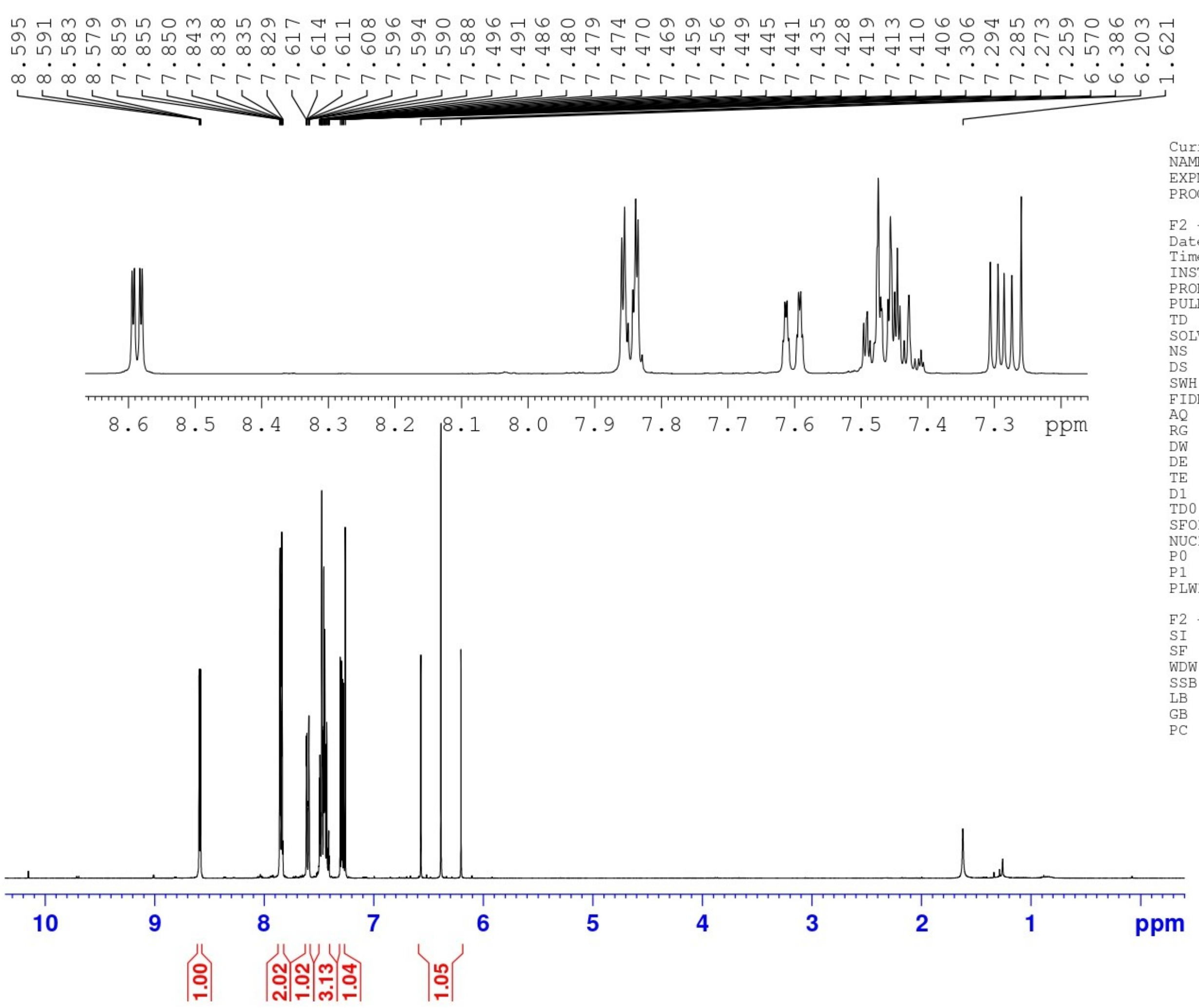
8.595 8.591 8.583 8.579 7.859 7.855 7.850 7.843 7.838 7.835 7.829 7.617 7.614 7.611 7.608 7.596 7.594 7.590 7.588 7.496 7.491 7.486 7.480 7.479 7.474 7.470 7.469 7.459 7.456 7.449 7.445 7.441 7.435 7.428 7.419 7.413 7.410 7.406 7.306 7.294 7.285 7.273 7.259 6.570 6.386 6.203 1.621
BRUKER
Current Data Parameters
NAME VII-Mn-113 i_10
EXPNO 3
PROCNO 1
F2 - Acquisition Parameters
Date_ 20250116
Time 14.01
INSTRUM Avance
PROBHD Z166552_0018 (PI HR-
PULPROG zg30
TD 65536
SOLVENT CDCl3
NS 16
DS 2
SWH 7812.500
FIDRES 0.238419
AQ 4.1943040
RG 101
DW 64.000
DE 6.67
TE 298.0
D1 1.00000000
TD0 1
SFO1 399.5701703
NUC1 1H
P0 2.60
P1 7.80
PLW1 21.19799995
F2 - Processing parameters
SI 65536
SF 399.5677129
WDW EM
SSB 0
LB 0.30
GB 0
PC 1.00
8.6 8.5 8.4 8.3 8.2 8.1 8.0 7.9 7.8 7.7 7.6 7.5 7.4 7.3 ppm
10 9 8 7 6 5 4 3 2 1 ppm
1.00 2.02 1.02 3.13 1.04 1.05

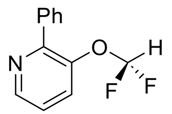
Ph
N
O
H
F
F

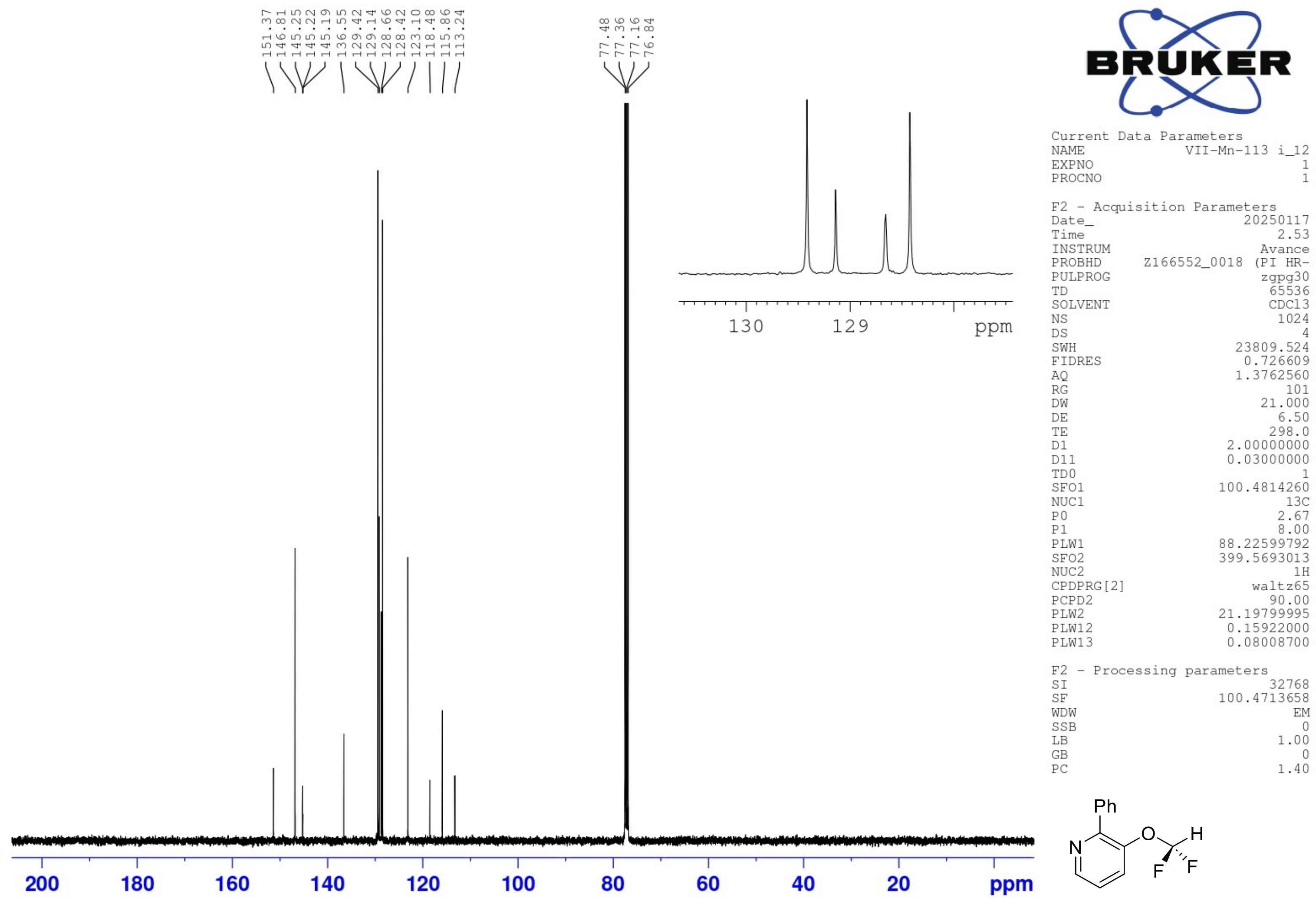
151.37
146.81
145.25
145.22
145.19
136.55
129.42
129.14
128.66
128.42
123.10
118.48
115.86
113.24
77.48
77.36
77.16
76.84
130
129
ppm
200
180
160
140
120
100
80
60
40
20
ppm
BRUKER
Current Data Parameters
NAME VII-Mn-113 i_12
EXPNO 1
PROCNO 1
F2 - Acquisition Parameters
Date_ 20250117
Time 2.53
INSTRUM Avance
PROBHD Z166552_0018 (PI HR-
PULPROG zgpg30
TD 65536
SOLVENT CDCl3
NS 1024
DS 4
SWH 23809.524
FIDRES 0.726609
AQ 1.3762560
RG 101
DW 21.000
DE 6.50
TE 298.0
D1 2.00000000
D11 0.03000000
TD0 1
SFO1 100.4814260
NUC1 13C
P0 2.67
P1 8.00
PLW1 88.22599792
SFO2 399.5693013
NUC2 1H
CPDPRG[2] waltz65
PCPD2 90.00
PLW2 21.19799995
PLW12 0.15922000
PLW13 0.08008700
F2 - Processing parameters
SI 32768
SF 100.4713658
WDW EM
SSB 0
LB 1.00
GB 0
PC 1.40
Ph
N
O
H
F
F

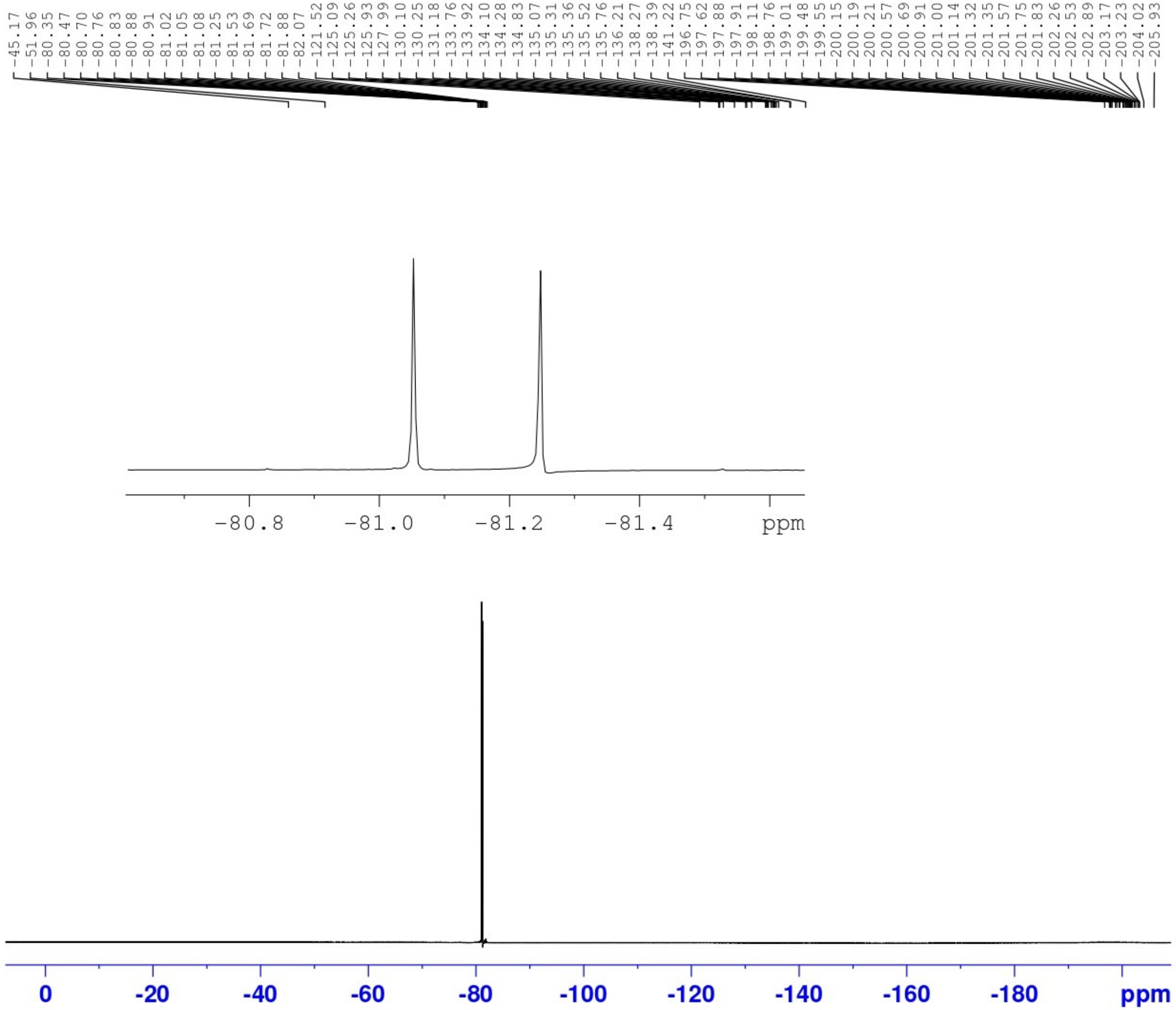

Current Data Parameters
NAME VII-Mn-113 i_11
EXPNO 2
PROCNO 1

F2 - Acquisition Parameters
Date_ 20250116
Time 14.03
INSTRUM Avance
PROBHD Z166552_0018 (PI HR-
PULPROG zg
TD 131072
SOLVENT CDCl3
NS 16
DS 4
SWH 90909.091
FIDRES 1.387163
AQ 0.7208960
RG 101
DW 5.500
DE 6.50
TE 298.0
D1 1.00000000
TD0 1
SFO1 375.9316815
NUC1 19F
P1 12.00
PLW1 32.47200012

F2 - Processing parameters
SI 65536
SF 375.9692784
WDW EM
SSB 0
LB 0.30
GB 0
PC 1.00

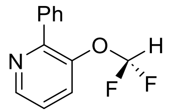

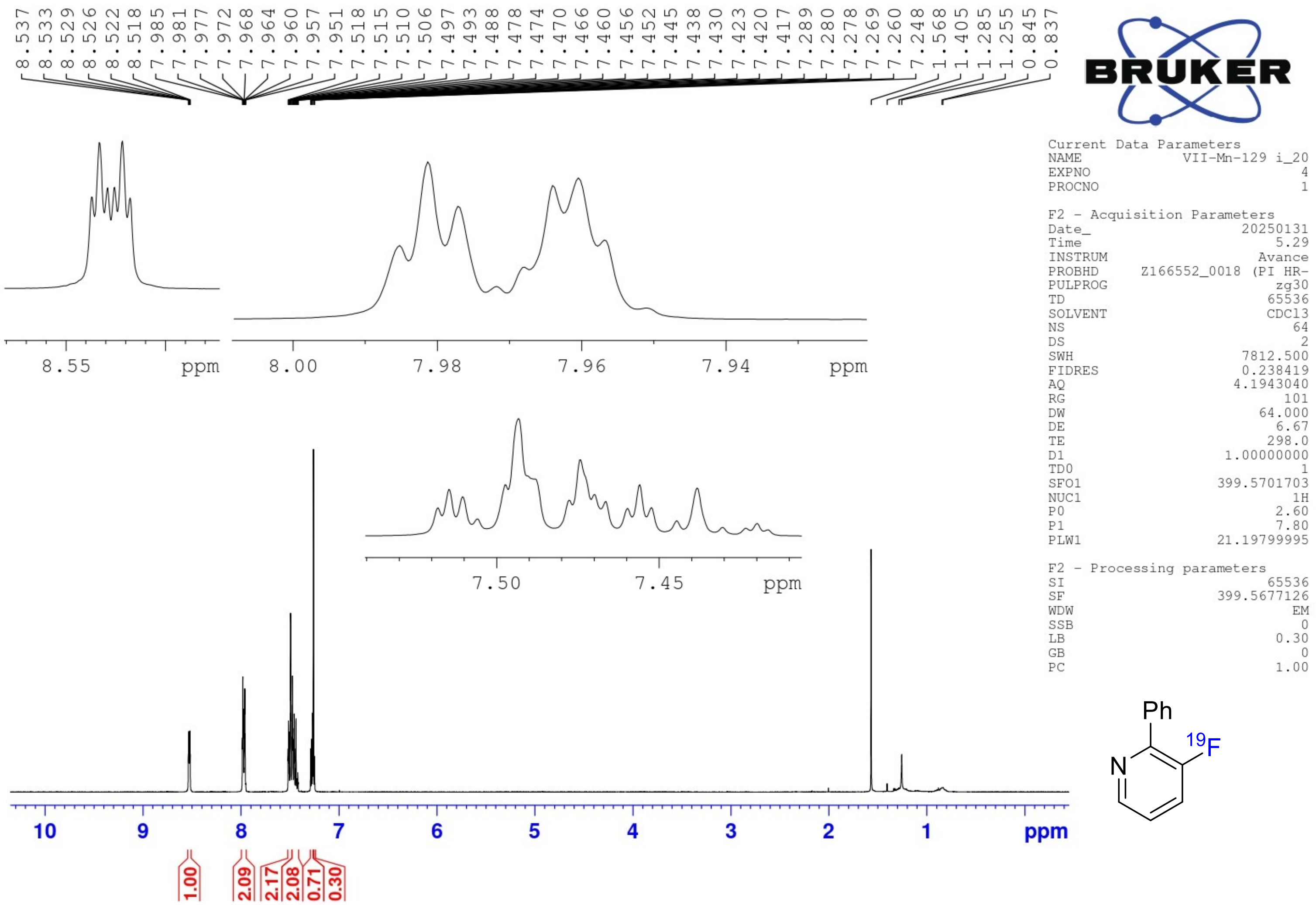
8.537 8.533 8.529 8.526 8.522 8.518 7.985 7.981 7.977 7.972 7.968 7.964 7.960 7.957 7.951 7.518 7.515 7.510 7.506 7.497 7.493 7.488 7.478 7.474 7.470 7.466 7.460 7.456 7.452 7.445 7.438 7.430 7.423 7.420 7.417 7.289 7.280 7.278 7.269 7.260 7.248 1.568 1.405 1.285 1.255 0.845 0.837
BRUKER
Current Data Parameters
NAME VII-Mn-129 i_20
EXPNO 4
PROCNO 1
F2 - Acquisition Parameters
Date_ 20250131
Time 5.29
INSTRUM Avance
PROBHD Z166552_0018 (PI HR-
PULPROG zg30
TD 65536
SOLVENT CDCl3
NS 64
DS 2
SWH 7812.500
FIDRES 0.238419
AQ 4.1943040
RG 101
DW 64.000
DE 6.67
TE 298.0
D1 1.00000000
TD0 1
SFO1 399.5701703
NUC1 1H
P0 2.60
P1 7.80
PLW1 21.19799995
F2 - Processing parameters
SI 65536
SF 399.5677126
WDW EM
SSB 0
LB 0.30
GB 0
PC 1.00
8.55 ppm
8.00 7.98 7.96 7.94 ppm
7.50 7.45 ppm
10 9 8 7 6 5 4 3 2 1 ppm
1.00 2.09 2.17 2.08 0.71 0.30
Ph
19F
N

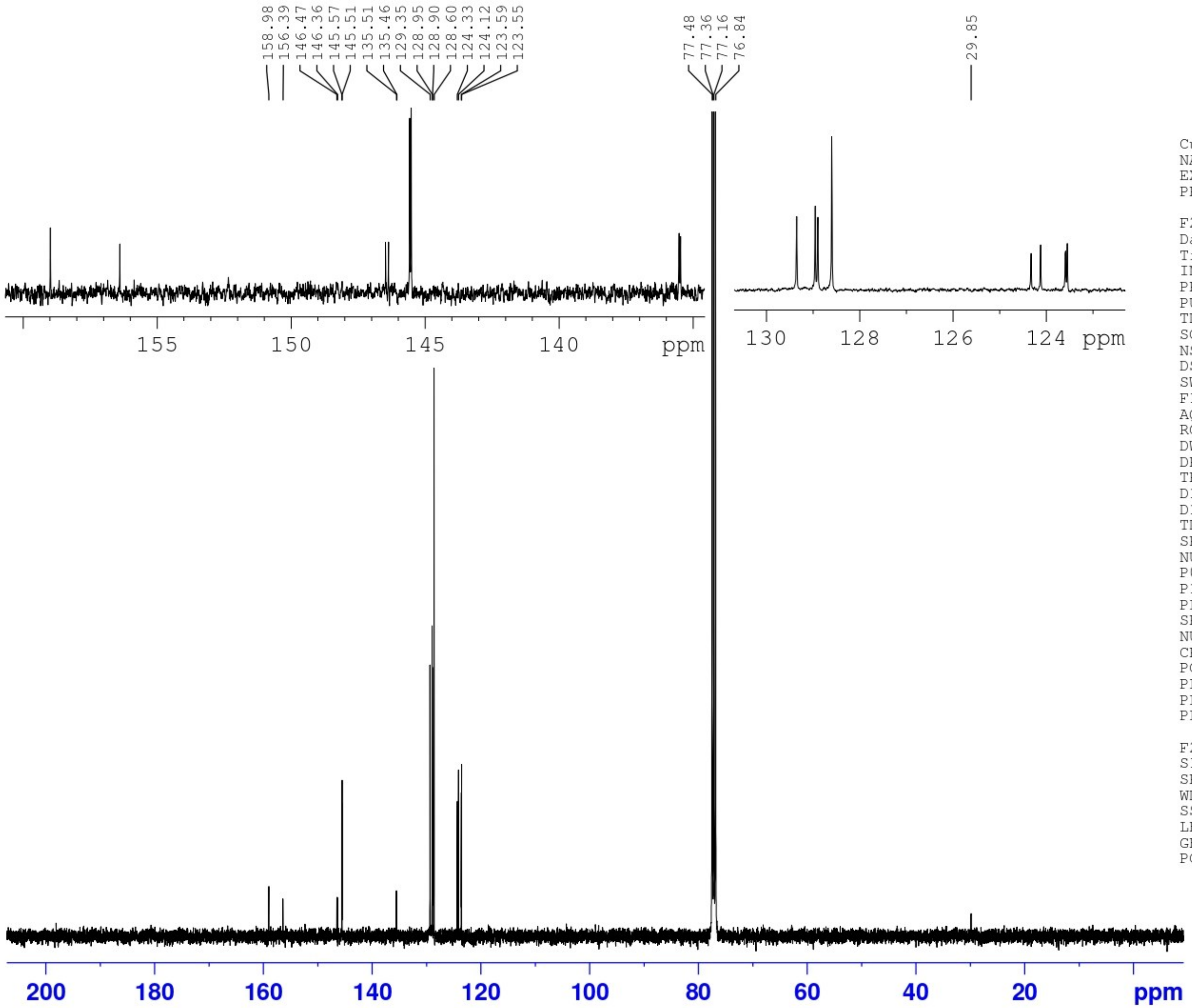

Current Data Parameters
NAME VII-Mn-129 i_22
EXPNO 4
PROCNO 1

F2 - Acquisition Parameters
Date_ 20250131
Time 7.32
INSTRUM Avance
PROBHD Z166552_0018 (PI HR-
PULPROG zgpg30
TD 65536
SOLVENT CDCl3
NS 2048
DS 4
SWH 23809.524
FIDRES 0.726609
AQ 1.3762560
RG 101
DW 21.000
DE 6.50
TE 298.0
D1 2.00000000
D11 0.03000000
TD0 1
SFO1 100.4814260
NUC1 13C
P0 2.67
P1 8.00
PLW1 88.22599792
SFO2 399.5693013
NUC2 1H
CPDPRG[2] waltz65
PCPD2 90.00
PLW2 21.19799995
PLW12 0.15922000
PLW13 0.08008700

F2 - Processing parameters
SI 32768
SF 100.4713651
WDW EM
SSB 0
LB 1.00
GB 0
PC 1.40

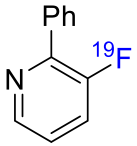

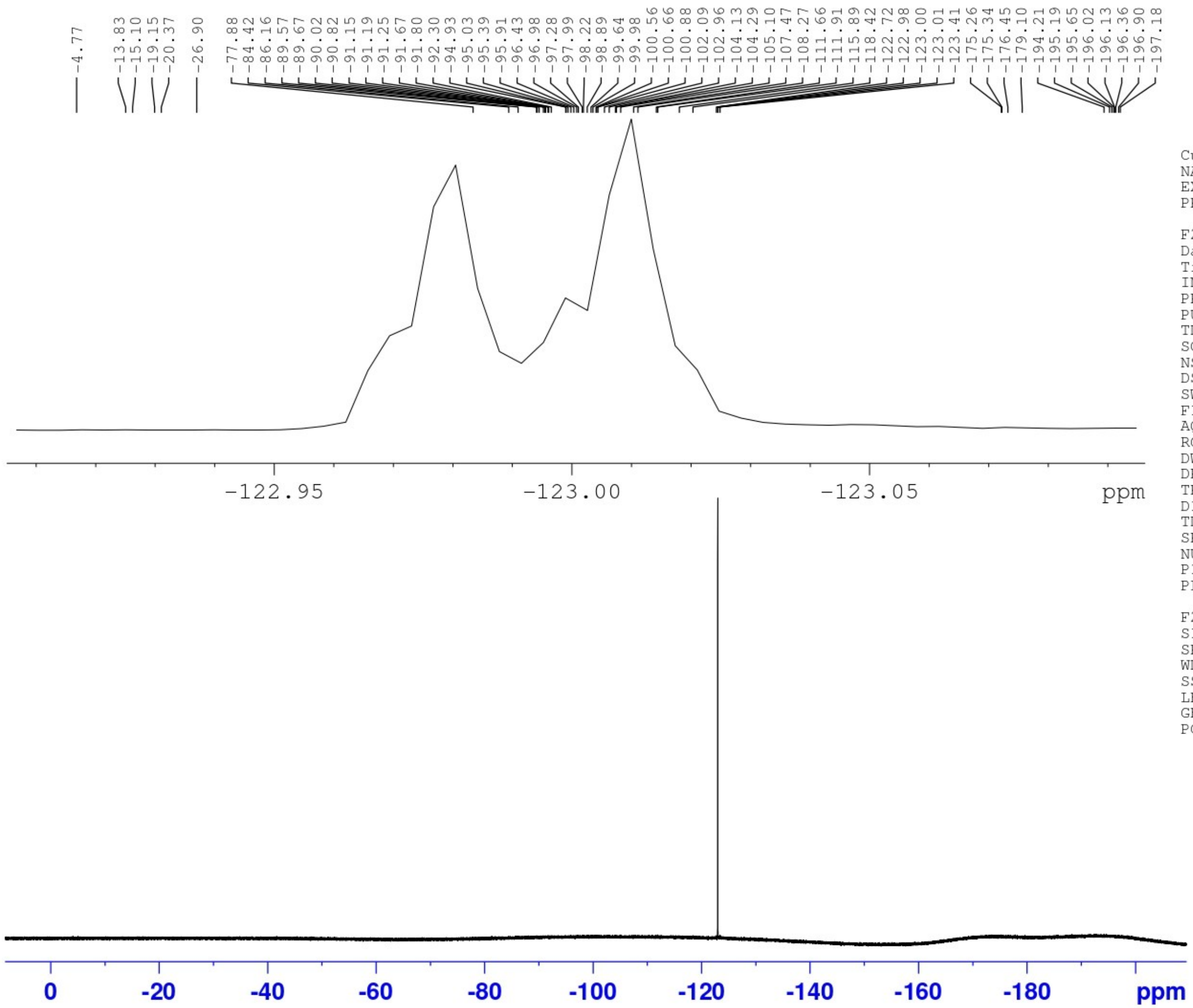

BRUKER

Current Data Parameters
NAME VII-Mn-129 i_21
EXPNO 4
PROCNO 1

F2 - Acquisition Parameters
Date_ 20250131
Time 5.33
INSTRUM Avance
PROBHD Z166552_0018 (PI HR-
PULPROG zg
TD 131072
SOLVENT CDCl3
NS 64
DS 4
SWH 90909.091
FIDRES 1.387163
AQ 0.7208960
RG 101
DW 5.500
DE 6.50
TE 298.0
D1 1.00000000
TD0 1
SFO1 375.9316815
NUC1 19F
P1 12.00
PLW1 32.47200012

F2 - Processing parameters
SI 65536
SF 375.9692784
WDW EM
SSB 0
LB 0.30
GB 0
PC 1.00

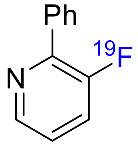

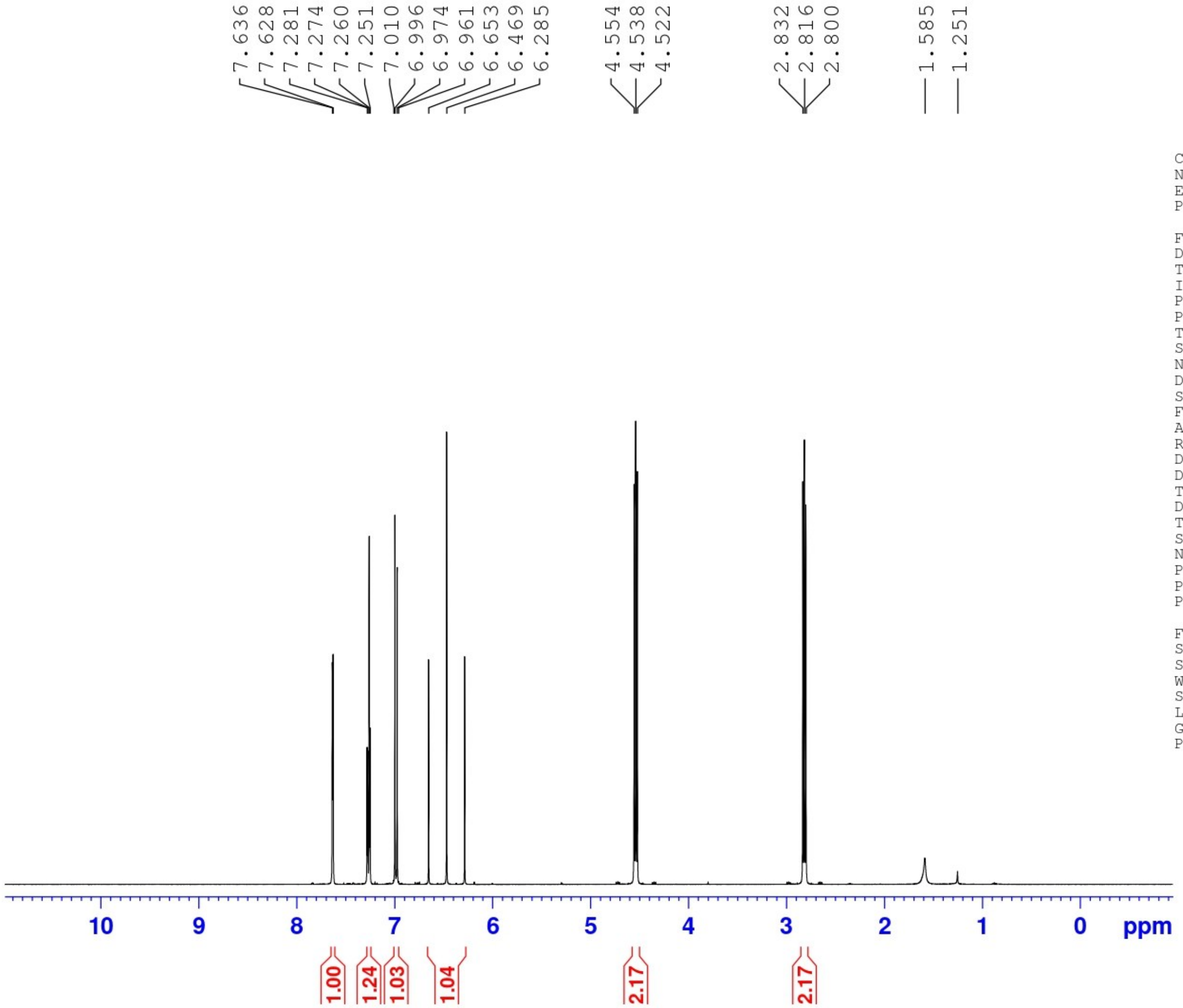


```
Current Data Parameters
NAME          VIII-Mn-101 i re_10
EXPNO                           2
PROCNO                          1

F2 - Acquisition Parameters
Date_                    20251013
Time                        16.26
INSTRUM                    Avance
PROBHD      Z166552_0018 (PI HR-
PULPROG                      zg30
TD                          65536
SOLVENT                     CDCl3
NS                             16
DS                              2
SWH                      7812.500
FIDRES                   0.238419
AQ                      4.1943040
RG                            101
DW                         64.000
DE                           6.67
TE                          298.0
D1                     1.00000000
TD0                             1
SFO1                  399.5424672
NUC1                           1H
P0                           2.60
P1                           7.80
PLW1                  21.19799995

F2 - Processing parameters
SI                          65536
SF                    399.5400096
WDW                            EM
SSB                             0
LB                           0.30
GB                              0
PC                           1.00
```

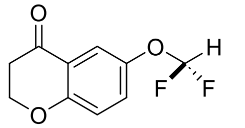

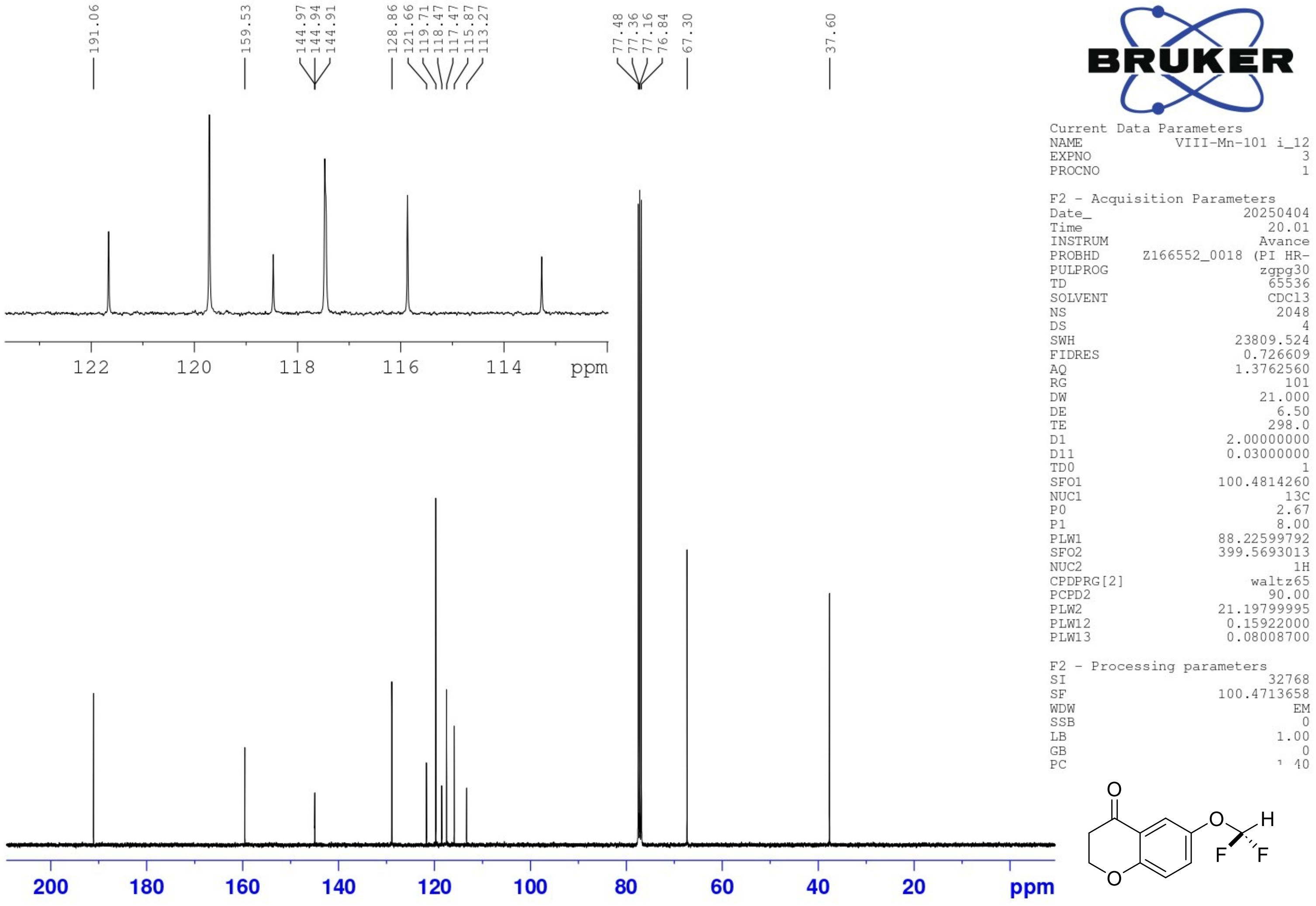

191.06
159.53
144.97
144.94
144.91
128.86
121.66
119.71
118.47
117.47
115.87
113.27
77.48
77.36
77.16
76.84
67.30
37.60
122
120
118
116
114
ppm
200
180
160
140
120
100
80
60
40
20
ppm
BRUKER
Current Data Parameters
NAME VIII-Mn-101 i_12
EXPNO 3
PROCNO 1
F2 - Acquisition Parameters
Date_ 20250404
Time 20.01
INSTRUM Avance
PROBHD Z166552_0018 (PI HR-
PULPROG zgpg30
TD 65536
SOLVENT CDCl3
NS 2048
DS 4
SWH 23809.524
FIDRES 0.726609
AQ 1.3762560
RG 101
DW 21.000
DE 6.50
TE 298.0
D1 2.00000000
D11 0.03000000
TD0 1
SFO1 100.4814260
NUC1 13C
P0 2.67
P1 8.00
PLW1 88.22599792
SFO2 399.5693013
NUC2 1H
CPDPRG[2] waltz65
PCPD2 90.00
PLW2 21.19799995
PLW12 0.15922000
PLW13 0.08008700
F2 - Processing parameters
SI 32768
SF 100.4713658
WDW EM
SSB 0
LB 1.00
GB 0
PC
O
O
O
H
F
F

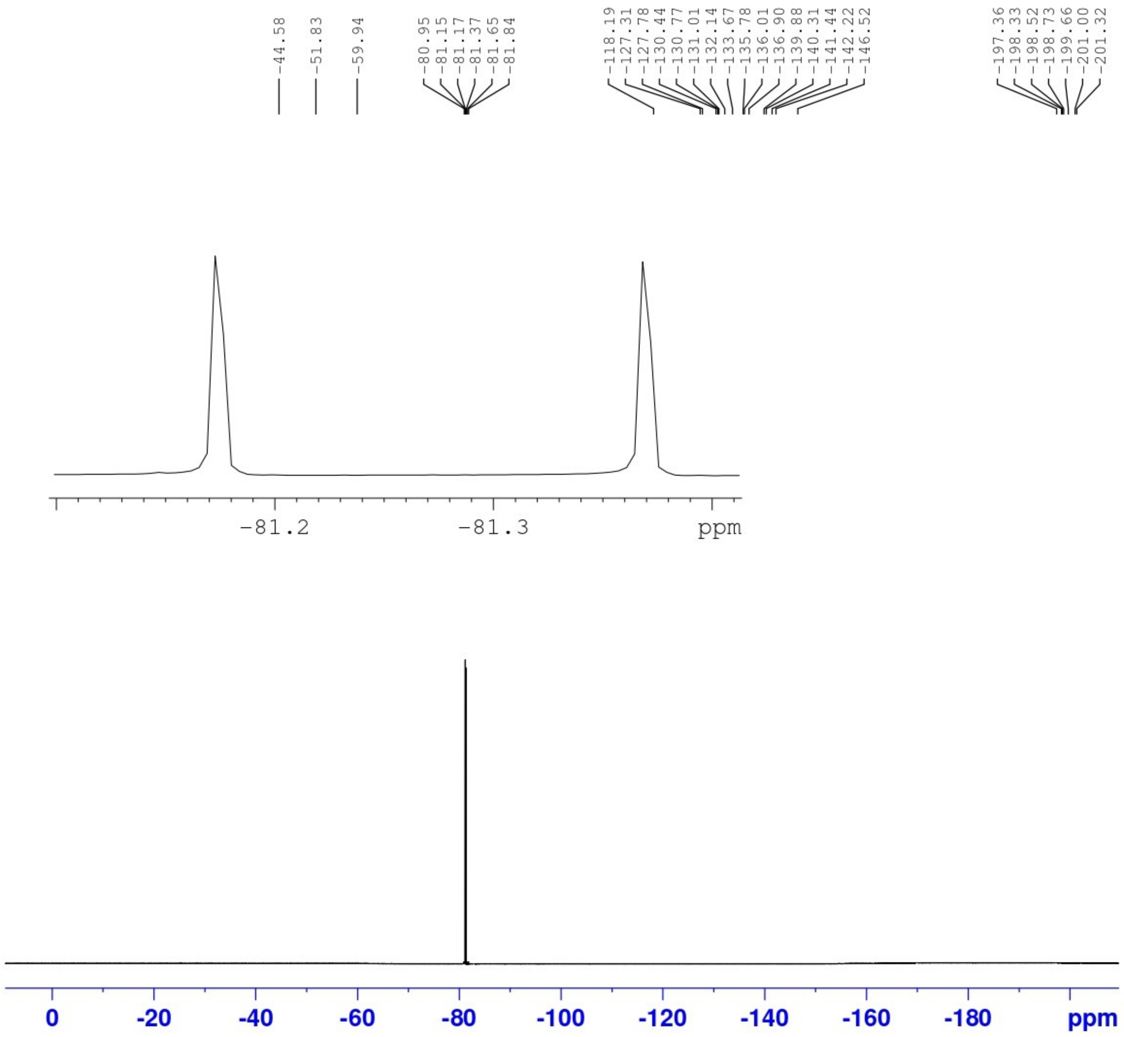

Current Data Parameters
NAME VIII-Mn-101 i re_11
EXPNO 2
PROCNO 1

F2 - Acquisition Parameters
Date_ 20251013
Time 16.28
INSTRUM Avance
PROBHD Z166552_0018 (PI HR-
PULPROG zg
TD 131072
SOLVENT CDCl3
NS 16
DS 4
SWH 90909.091
FIDRES 1.387163
AQ 0.7208960
RG 101
DW 5.500
DE 6.50
TE 298.0
D1 1.00000000
TD0 1
SFO1 375.9056172
NUC1 19F
P1 12.00
PLW1 32.47200012

F2 - Processing parameters
SI 65536
SF 375.9432115
WDW EM
SSB 0
LB 0.30
GB 0
PC 1.00

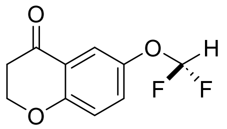

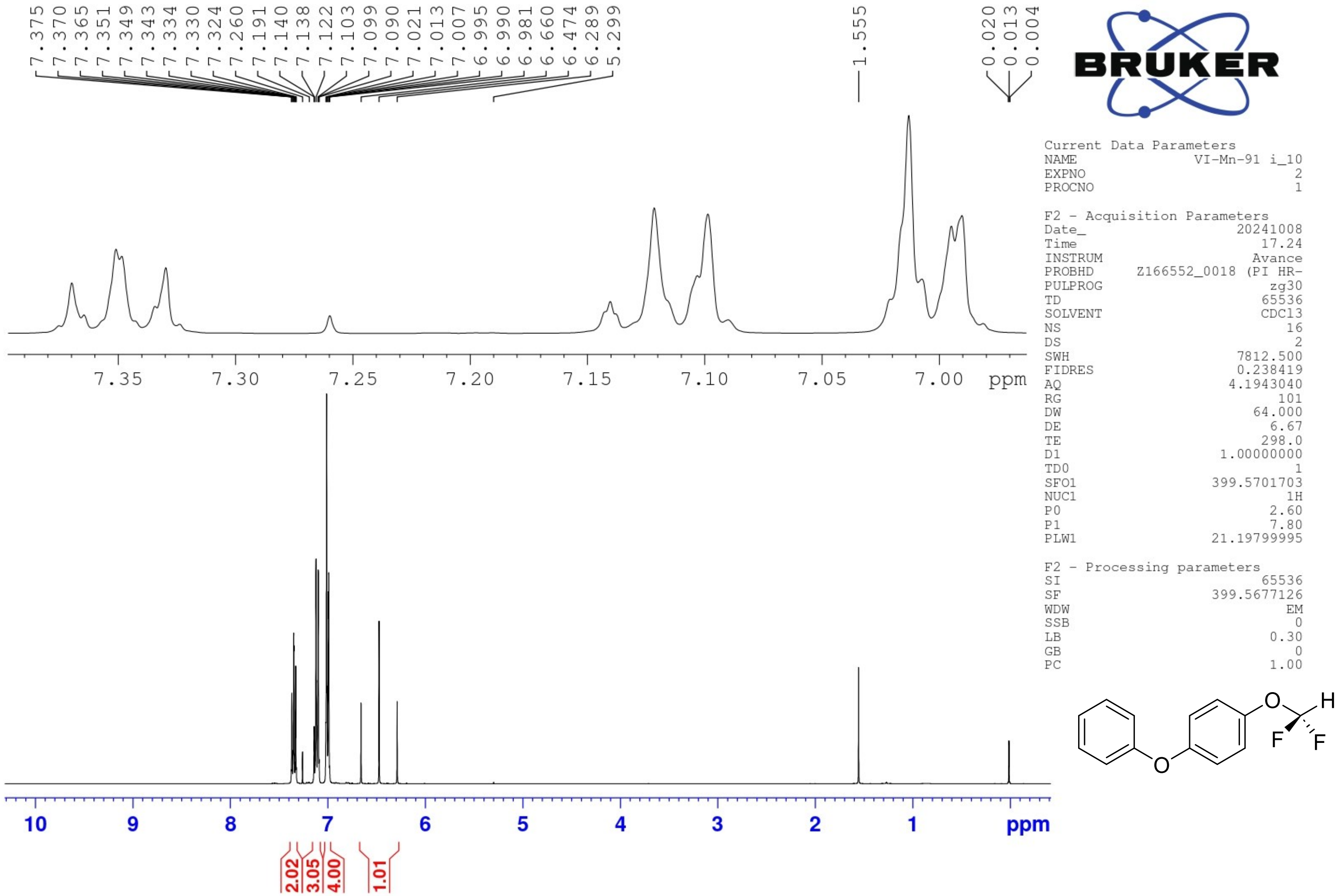
7.375
7.370
7.365
7.351
7.349
7.343
7.334
7.330
7.324
7.260
7.191
7.140
7.138
7.122
7.103
7.099
7.090
7.021
7.013
7.007
6.995
6.990
6.981
6.660
6.474
6.289
5.299
1.555
0.020
0.013
0.004
BRUKER
Current Data Parameters
NAME VI-Mn-91 i_10
EXPNO 2
PROCNO 1
F2 - Acquisition Parameters
Date_ 20241008
Time 17.24
INSTRUM Avance
PROBHD Z166552_0018 (PI HR-
PULPROG zg30
TD 65536
SOLVENT CDCl3
NS 16
DS 2
SWH 7812.500
FIDRES 0.238419
AQ 4.1943040
RG 101
DW 64.000
DE 6.67
TE 298.0
D1 1.00000000
TD0 1
SFO1 399.5701703
NUC1 1H
P0 2.60
P1 7.80
PLW1 21.19799995
F2 - Processing parameters
SI 65536
SF 399.5677126
WDW EM
SSB 0
LB 0.30
GB 0
PC 1.00
7.35
7.30
7.25
7.20
7.15
7.10
7.05
7.00
ppm
10
9
8
7
6
5
4
3
2
1
0
ppm
2.02
3.05
4.00
1.01
O
H
F
F
O

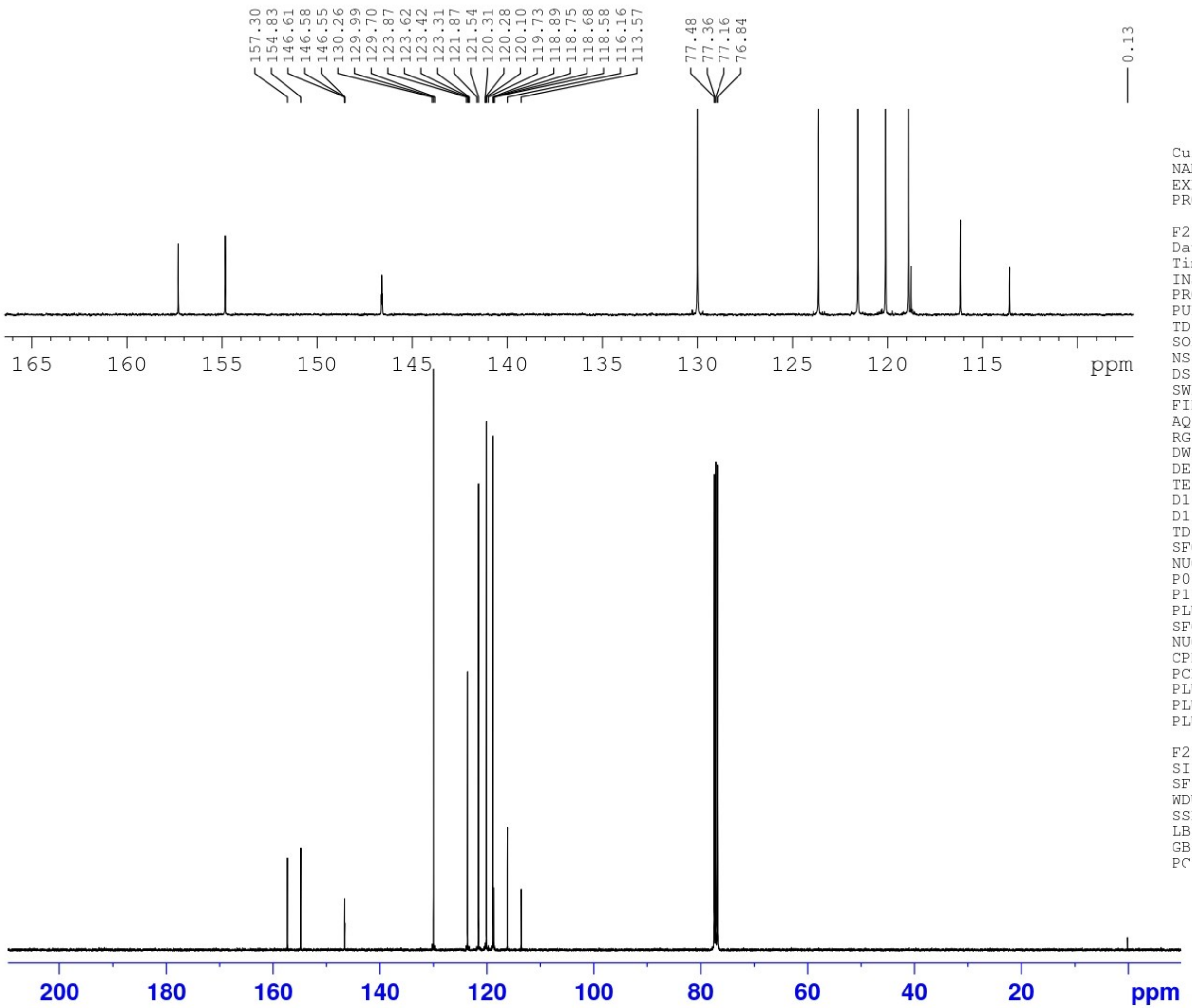

Current Data Parameters
NAME VI-Mn-91 i_12
EXPNO 2
PROCNO 1

F2 - Acquisition Parameters
Date_ 20241008
Time 20.05
INSTRUM Avance
PROBHD Z166552_0018 (PI HR-
PULPROG zgpg30
TD 65536
SOLVENT CDCl3
NS 2048
DS 4
SWH 23809.524
FIDRES 0.726609
AQ 1.3762560
RG 101
DW 21.000
DE 6.50
TE 298.0
D1 2.00000000
D11 0.03000000
TD0 1
SFO1 100.4814260
NUC1 13C
P0 2.67
P1 8.00
PLW1 88.22599792
SFO2 399.5693013
NUC2 1H
CPDPRG[2] waltz65
PCPD2 90.00
PLW2 21.19799995
PLW12 0.15922000
PLW13 0.08008700

F2 - Processing parameters
SI 32768
SF 100.4713661
WDW EM
SSB 0
LB 1.00
GB 0
PC [illegible]

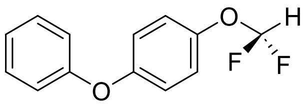

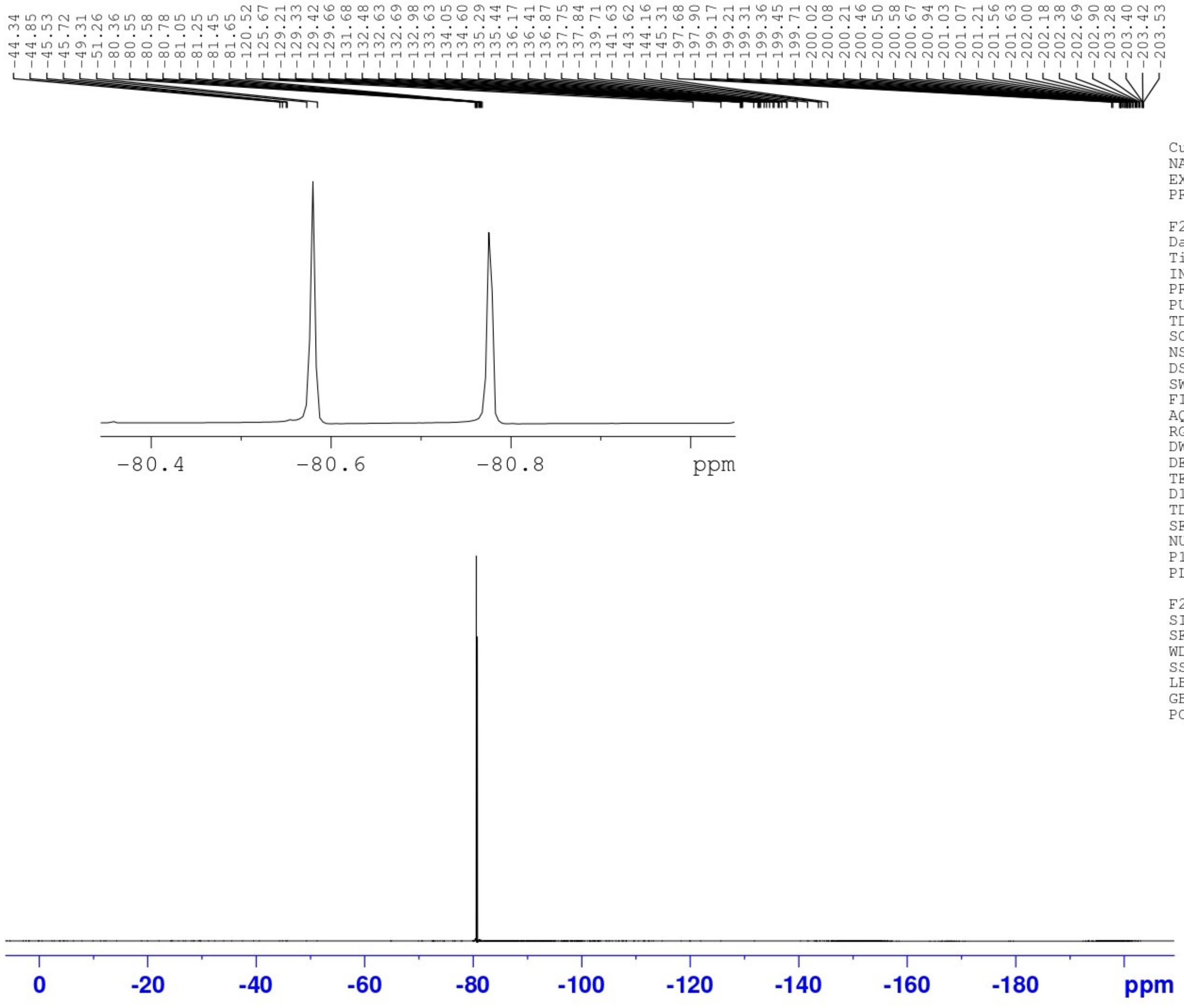

BRUKER

Current Data Parameters
NAME VI-Mn-91 i_11
EXPNO 2
PROCNO 1

F2 - Acquisition Parameters
Date_ 20241008
Time 17.25
INSTRUM Avance
PROBHD Z166552_0018 (PI HR-
PULPROG zg
TD 131072
SOLVENT CDCl3
NS 16
DS 4
SWH 90909.091
FIDRES 1.387163
AQ 0.7208960
RG 101
DW 5.500
DE 6.50
TE 298.0
D1 1.00000000
TD0 1
SFO1 375.9316815
NUC1 19F
P1 12.00
PLW1 32.47200012

F2 - Processing parameters
SI 65536
SF 375.9692784
WDW EM
SSB 0
LB 0.30
GB 0
PC 1.00

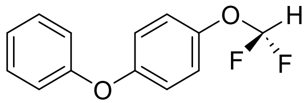

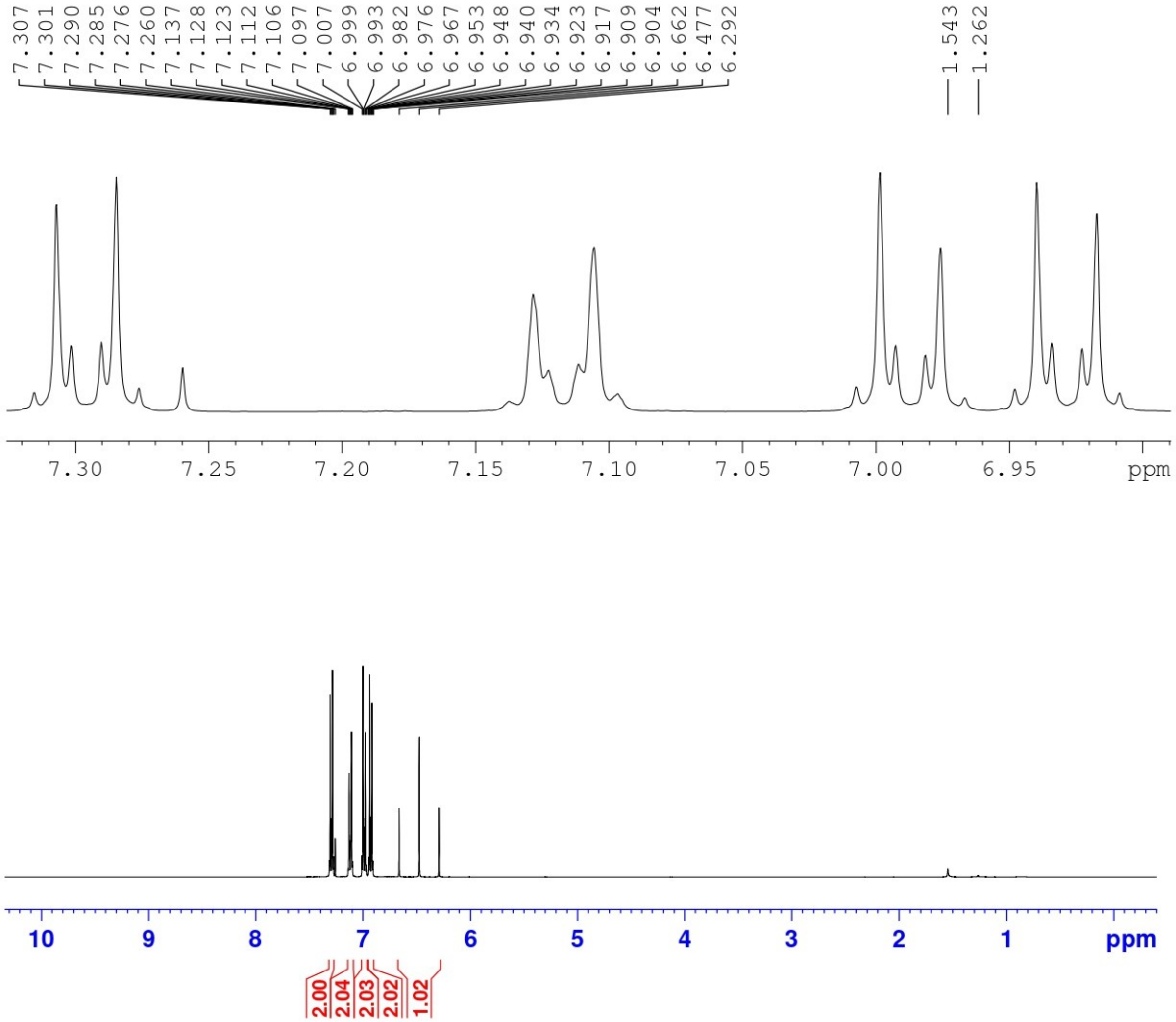

Current Data Parameters

NAME	IX-Mn-70 i_10
EXPNO	2
PROCNO	1

F2 - Acquisition Parameters

Date_	20250613
Time	22.20
INSTRUM	Avance
PROBHD	Z166552_0018 (PI HR-
PULPROG	zg30
TD	65536
SOLVENT	CDCl3
NS	16
DS	2
SWH	7812.500
FIDRES	0.238419
AQ	4.1943040
RG	101
DW	64.000
DE	6.67
TE	298.0
D1	1.00000000
TD0	1
SFO1	399.5424672
NUC1	1H
P0	2.60
P1	7.80
PLW1	21.19799995

F2 - Processing parameters

SI	65536
SF	399.5400096
WDW	EM
SSB	0
LB	0.30
GB	0
PC	1.00

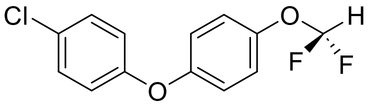

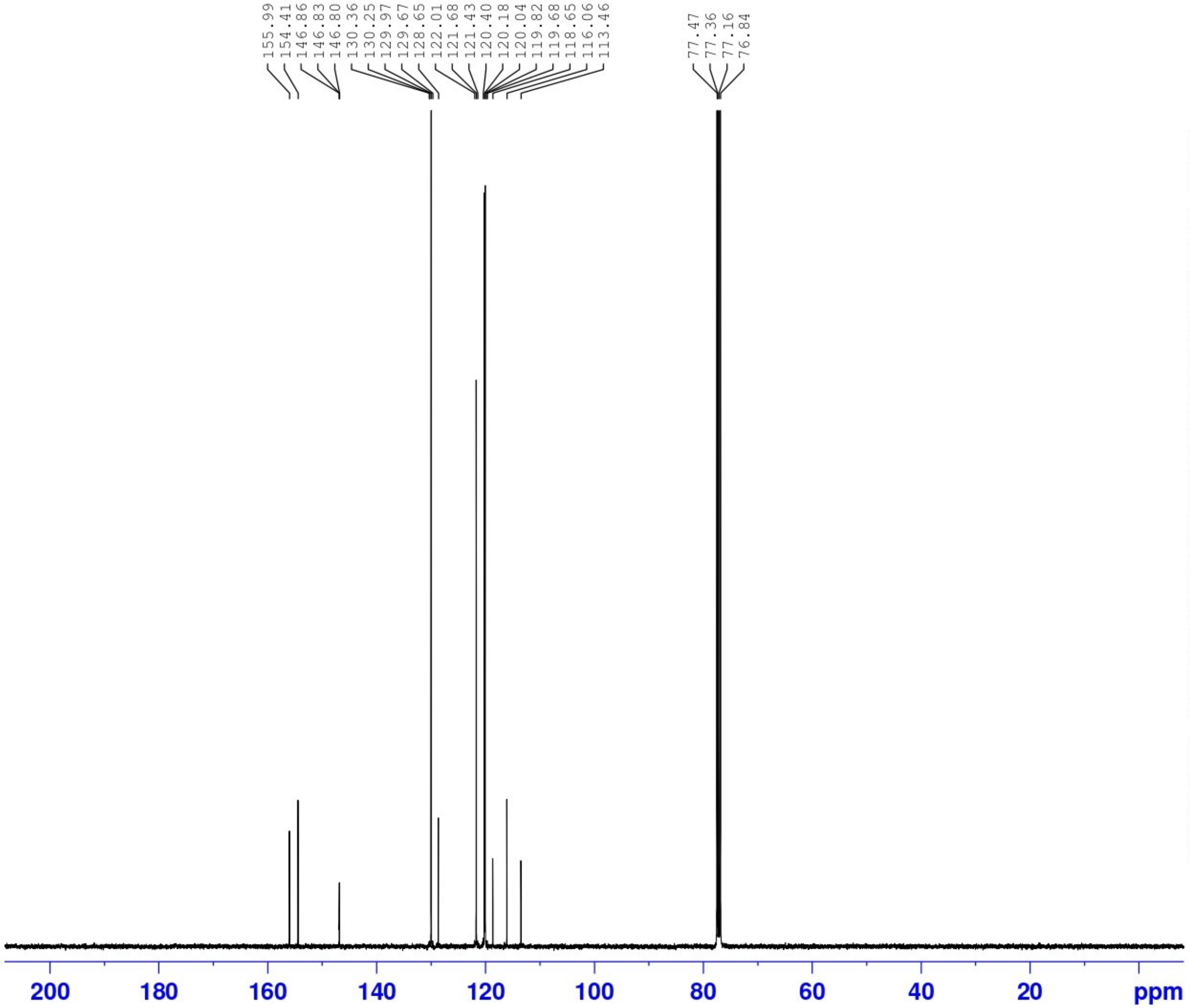

Current Data Parameters
NAME IX-Mn-70 i_12
EXPNO 2
PROCNO 1

F2 - Acquisition Parameters
Date_ 20250614
Time 0.21
INSTRUM Avance
PROBHD Z166552_0018 (PI HR-
PULPROG zgpg30
TD 65536
SOLVENT CDCl3
NS 2048
DS 4
SWH 23809.524
FIDRES 0.726609
AQ 1.3762560
RG 101
DW 21.000
DE 6.50
TE 298.0
D1 2.00000000
D11 0.03000000
TD0 1
SFO1 100.4744593
NUC1 13C
P0 2.67
P1 8.00
PLW1 88.22599792
SFO2 399.5415982
NUC2 1H
CPDPRG[2] waltz65
PCPD2 90.00
PLW2 21.19799995
PLW12 0.15922000
PLW13 0.08008700

F2 - Processing parameters
SI 32768
SF 100.4643998
WDW EM
SSB 0
LB 1.00
GB 0
PC 1.40

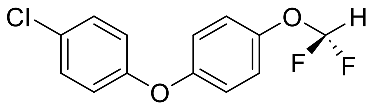

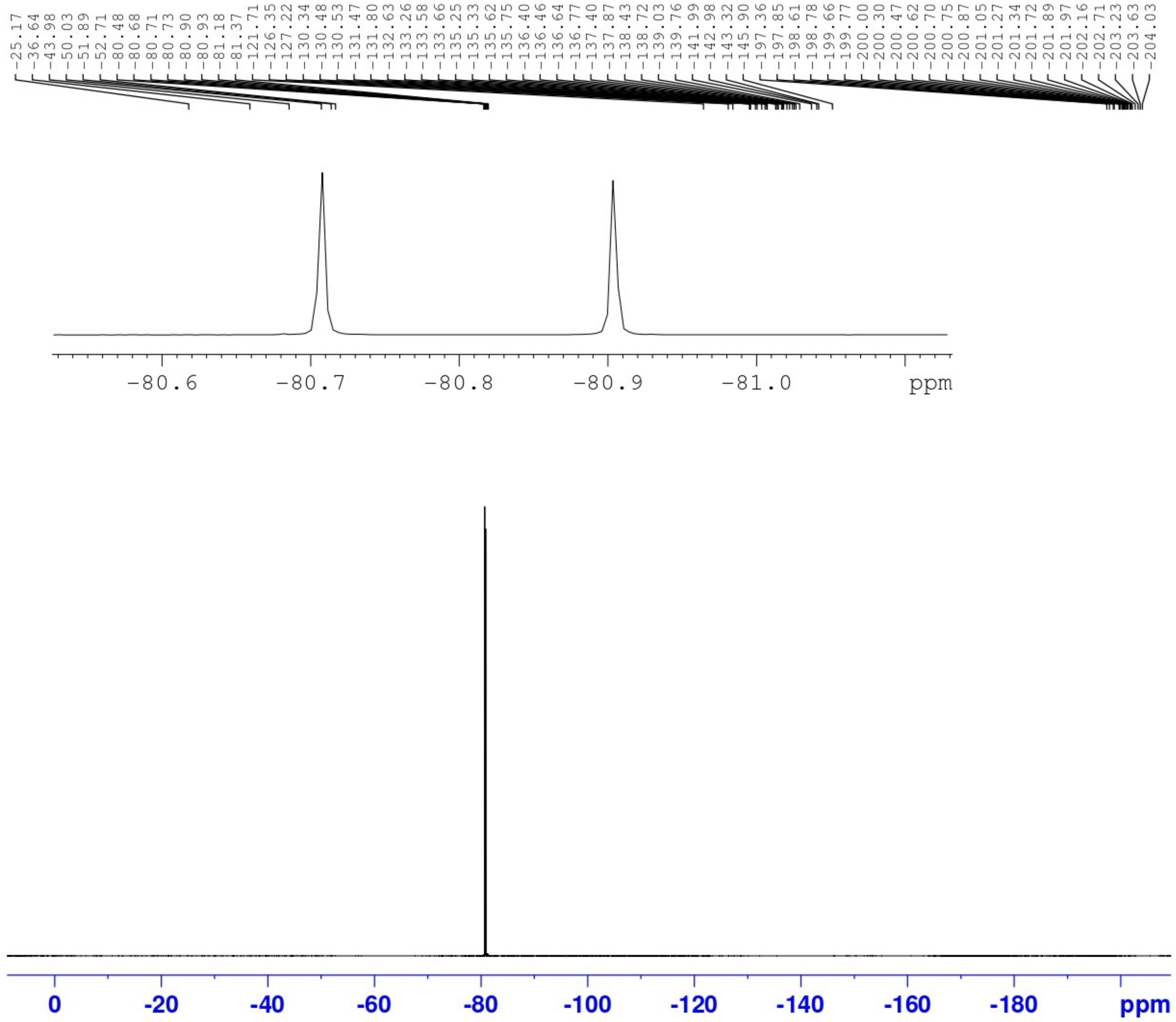

Current Data Parameters
NAME IX-Mn-70 i_11
EXPNO 2
PROCNO 1

F2 - Acquisition Parameters
Date_ 20250613
Time 22.22
INSTRUM Avance
PROBHD Z166552_0018 (PI HR-
PULPROG zg
TD 131072
SOLVENT CDCl3
NS 16
DS 4
SWH 90909.091
FIDRES 1.387163
AQ 0.7208960
RG 101
DW 5.500
DE 6.50
TE 298.0
D1 1.00000000
TD0 1
SFO1 375.9056172
NUC1 19F
P1 12.00
PLW1 32.47200012

F2 - Processing parameters
SI 65536
SF 375.9432115
WDW EM
SSB 0
LB 0.30
GB 0
PC 1.00

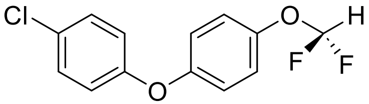

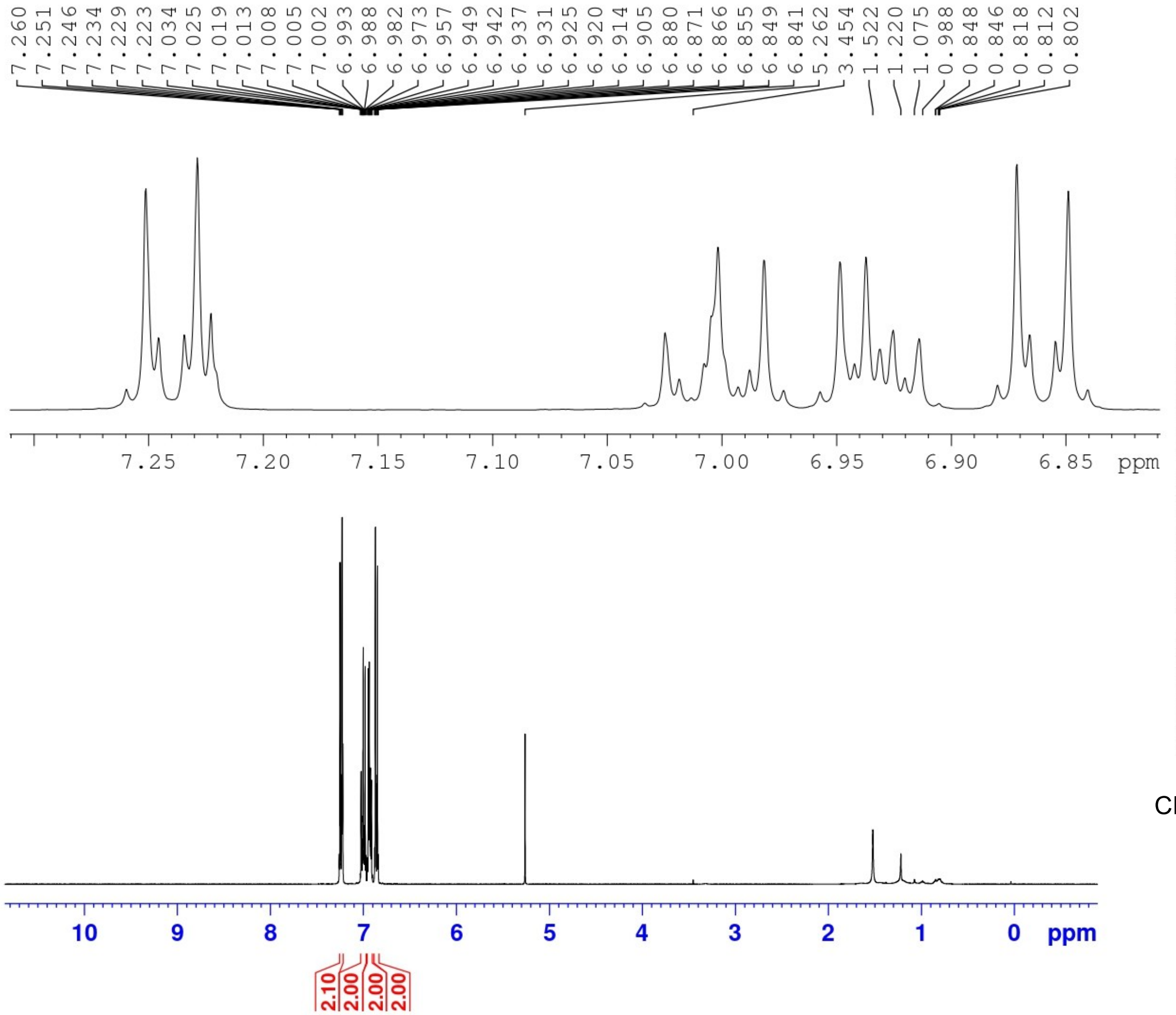

Current Data Parameters
NAME IX-Mn-71 ii dried_10
EXPNO 3
PROCNO 1

F2 - Acquisition Parameters
Date_ 20250707
Time 12.18
INSTRUM Avance
PROBHD Z166552_0018 (PI HR-
PULPROG zg30
TD 65536
SOLVENT CDCl3
NS 16
DS 2
SWH 7812.500
FIDRES 0.238419
AQ 4.1943040
RG 101
DW 64.000
DE 6.67
TE 298.0
D1 1.00000000
TD0 1
SFO1 399.5424672
NUC1 1H
P0 2.60
P1 7.80
PLW1 21.19799995

F2 - Processing parameters
SI 65536
SF 399.5400245
WDW EM
SSB 0
LB 0.30
GB 0
PC 1.00

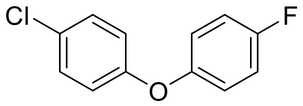

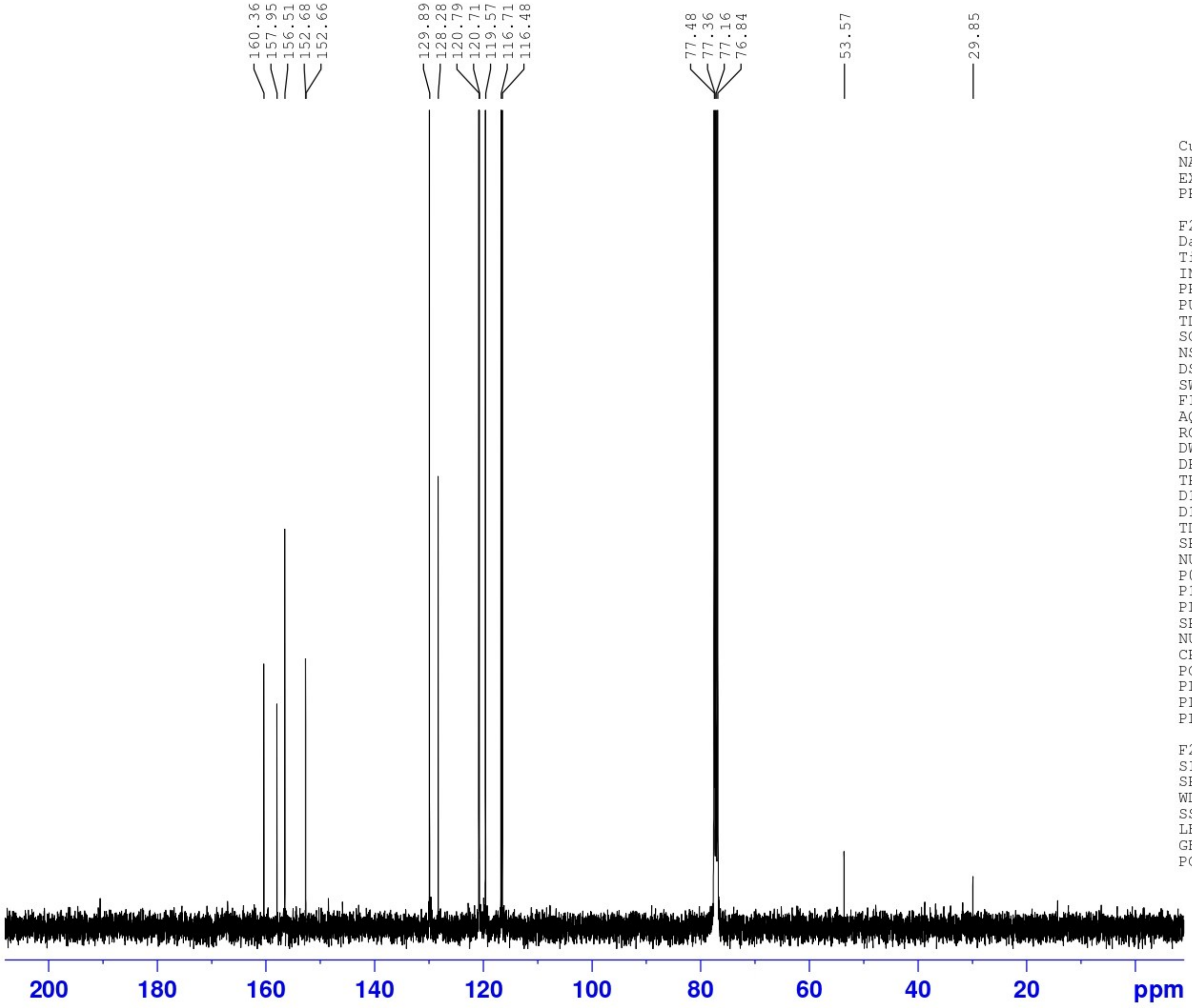

Current Data Parameters
NAME IX-Mn-71 ii_12
EXPNO 2
PROCNO 1

F2 - Acquisition Parameters
Date_ 20250707
Time 23.10
INSTRUM Avance
PROBHD Z166552_0018 (PI HR-
PULPROG zgpg30
TD 65536
SOLVENT CDCl3
NS 2048
DS 4
SWH 23809.524
FIDRES 0.726609
AQ 1.3762560
RG 101
DW 21.000
DE 6.50
TE 298.0
D1 2.00000000
D11 0.03000000
TD0 1
SFO1 100.4744593
NUC1 13C
P0 2.67
P1 8.00
PLW1 88.22599792
SFO2 399.5415982
NUC2 1H
CPDPRG[2] waltz65
PCPD2 90.00
PLW2 21.19799995
PLW12 0.15922000
PLW13 0.08008700

F2 - Processing parameters
SI 32768
SF 100.4643994
WDW EM
SSB 0
LB 1.00
GB 0
PC 1.40

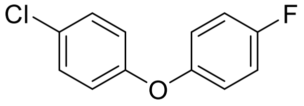

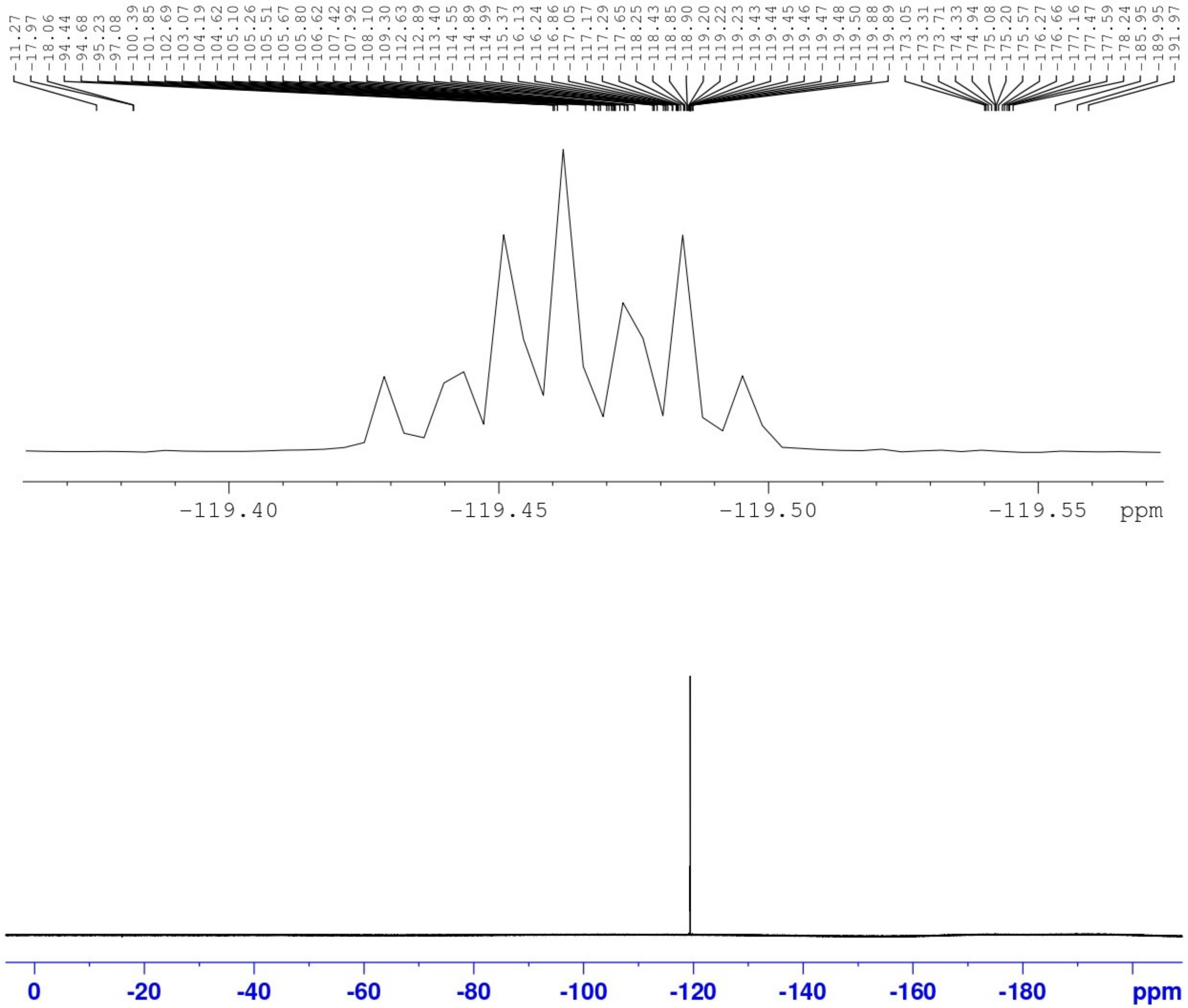

Current Data Parameters
NAME IX-Mn-71 ii dried_11
EXPNO 2
PROCNO 1

F2 - Acquisition Parameters
Date_ 20250707
Time 12.21
INSTRUM Avance
PROBHD Z166552_0018 (PI HR-
PULPROG zg
TD 131072
SOLVENT CDCl3
NS 16
DS 4
SWH 90909.091
FIDRES 1.387163
AQ 0.7208960
RG 101
DW 5.500
DE 6.50
TE 298.0
D1 1.00000000
TD0 1
SFO1 375.9056172
NUC1 19F
P1 12.00
PLW1 32.47200012

F2 - Processing parameters
SI 65536
SF 375.9432115
WDW EM
SSB 0
LB 0.30
GB 0
PC 1.00

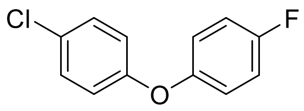

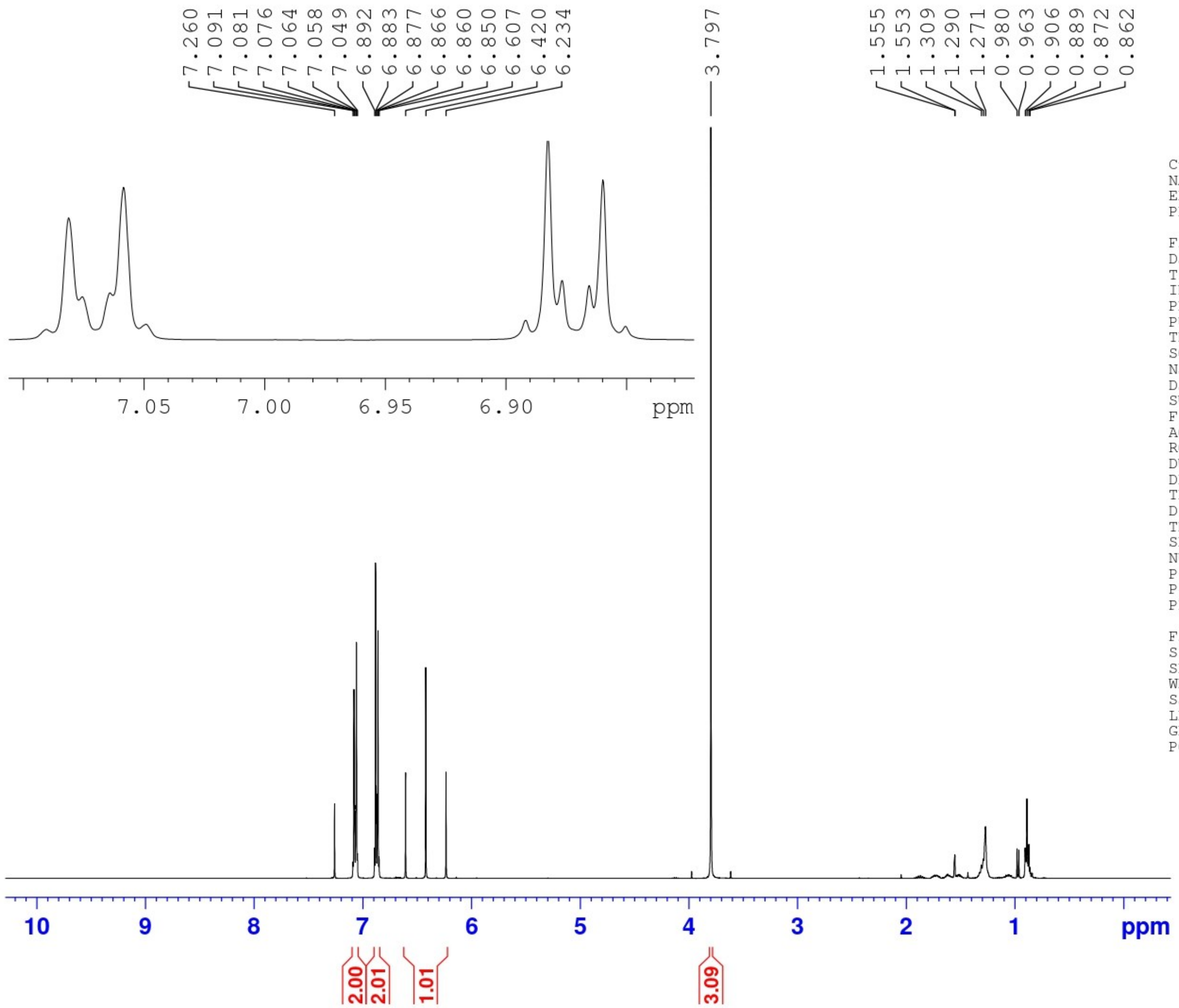

BRUKER

Current Data Parameters
NAME VII-Mn-96 i_10
EXPNO 2
PROCNO 1

F2 - Acquisition Parameters
Date_ 20250109
Time 13.24
INSTRUM Avance
PROBHD Z166552_0018 (PI HR-
PULPROG zg30
TD 65536
SOLVENT CDCl3
NS 16
DS 2
SWH 7812.500
FIDRES 0.238419
AQ 4.1943040
RG 101
DW 64.000
DE 6.67
TE 298.0
D1 1.00000000
TD0 1
SFO1 399.5701703
NUC1 1H
P0 2.60
P1 7.80
PLW1 21.19799995

F2 - Processing parameters
SI 65536
SF 399.5677126
WDW EM
SSB 0
LB 0.30
GB 0
PC 1.00

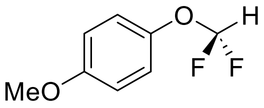

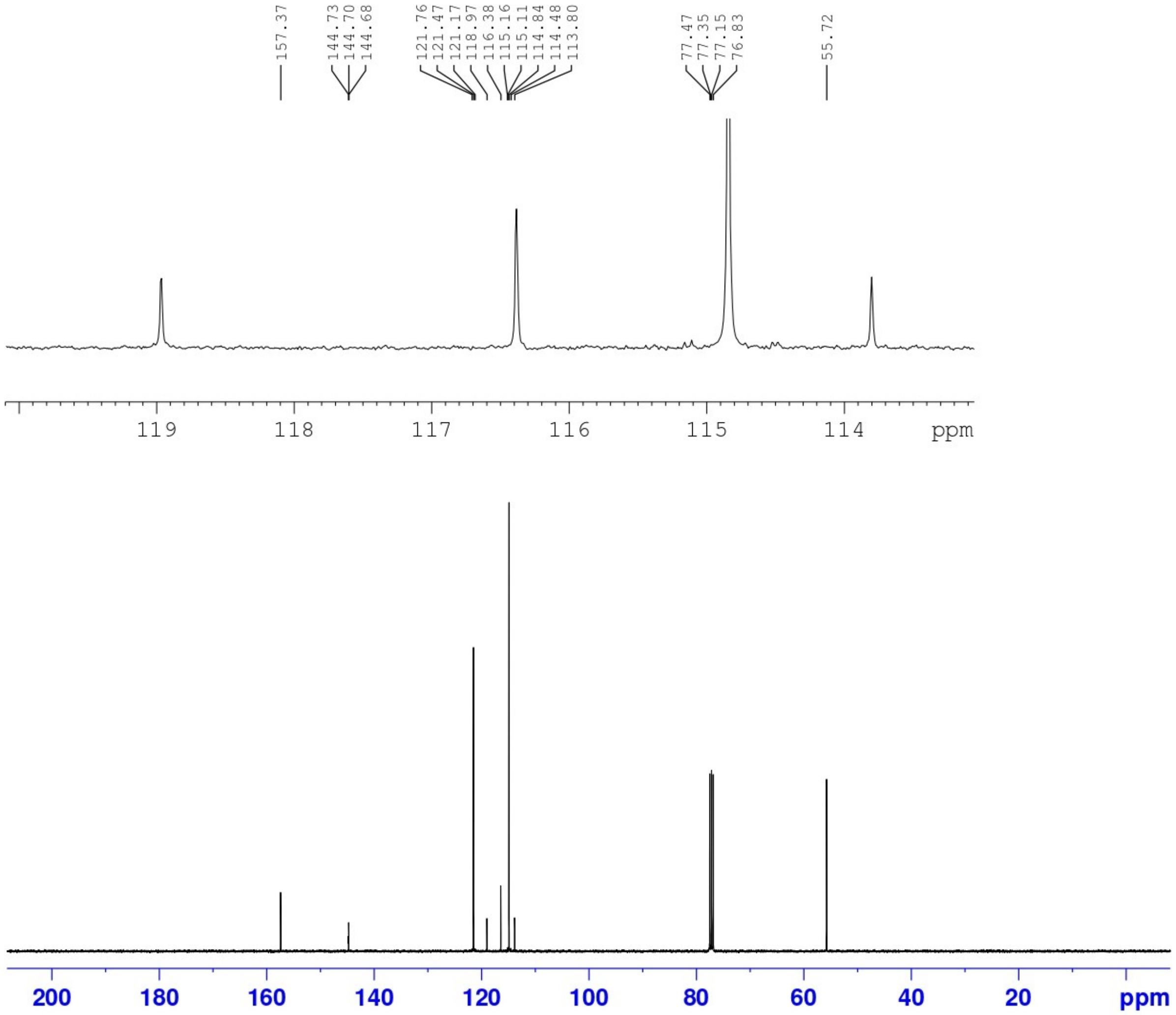

Current Data Parameters
NAME VII-Mn-96 i re_12
EXPNO 1
PROCNO 1

F2 - Acquisition Parameters
Date_ 20251103
Time 8.13
INSTRUM Avance
PROBHD Z166552_0018 (PI HR-
PULPROG zgpg30
TD 65536
SOLVENT CDCl3
NS 512
DS 4
SWH 23809.524
FIDRES 0.726609
AQ 1.3762560
RG 101
DW 21.000
DE 6.50
TE 298.0
D1 2.00000000
D11 0.03000000
TD0 1
SFO1 100.4744593
NUC1 13C
P0 2.67
P1 8.00
PLW1 88.22599792
SFO2 399.5415982
NUC2 1H
CPDPRG[2] waltz65
PCPD2 90.00
PLW2 21.19799995
PLW12 0.15922000
PLW13 0.08008700

F2 - Processing parameters
SI 32768
SF 100.4644008
WDW EM
SSB 0
LB 1.00
GB 0
PC 1.40

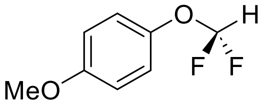

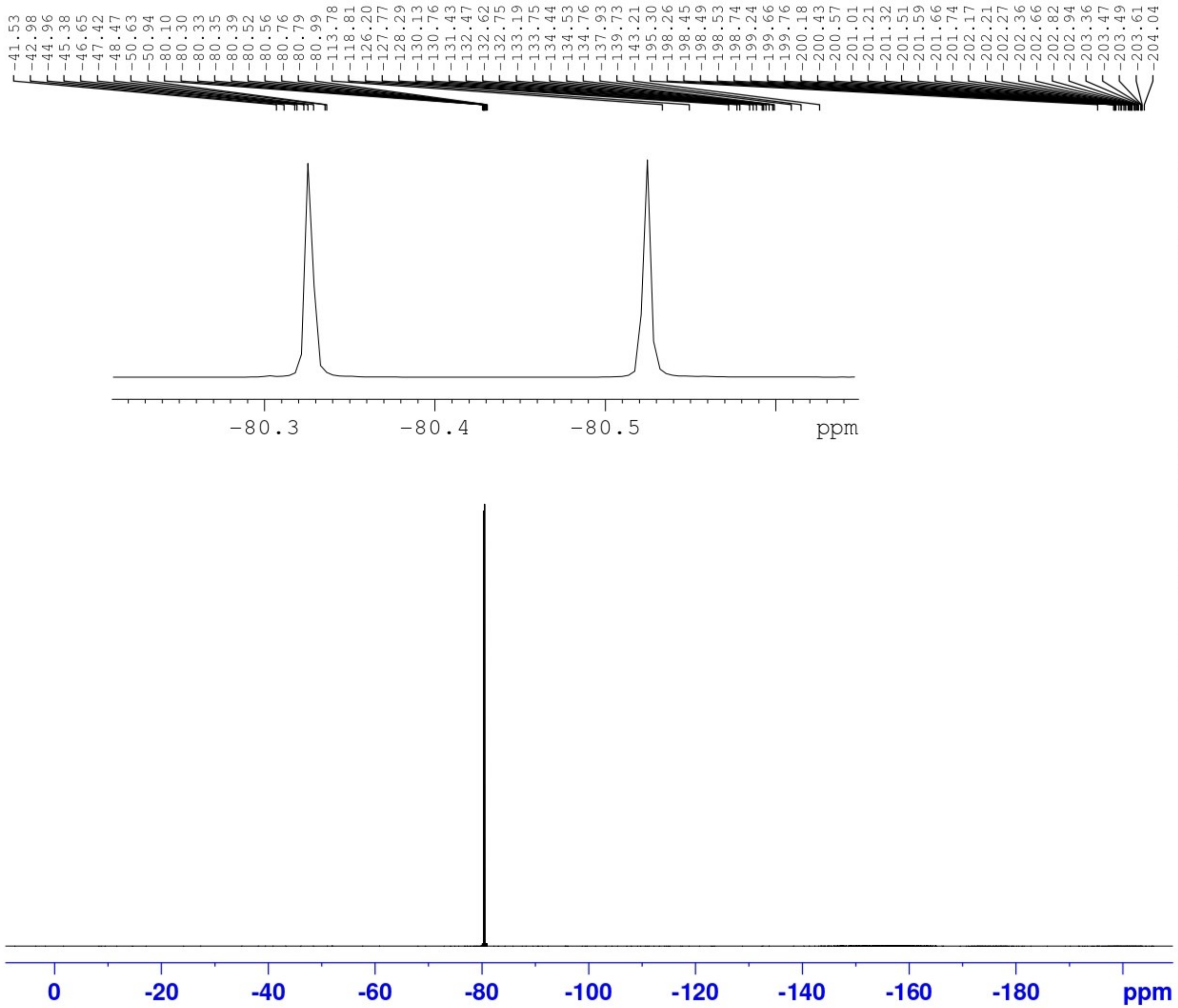

Current Data Parameters
NAME VII-Mn-96 i_11
EXPNO 2
PROCNO 1

F2 - Acquisition Parameters
Date_ 20250109
Time 13.25
INSTRUM Avance
PROBHD Z166552_0018 (PI HR-
PULPROG zg
TD 131072
SOLVENT CDCl3
NS 16
DS 4
SWH 90909.091
FIDRES 1.387163
AQ 0.7208960
RG 101
DW 5.500
DE 6.50
TE 298.0
D1 1.00000000
TD0 1
SFO1 375.9316815
NUC1 19F
P1 12.00
PLW1 32.47200012

F2 - Processing parameters
SI 65536
SF 375.9692784
WDW EM
SSB 0
LB 0.30
GB 0
PC 1.00

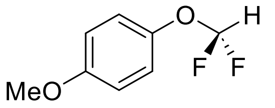

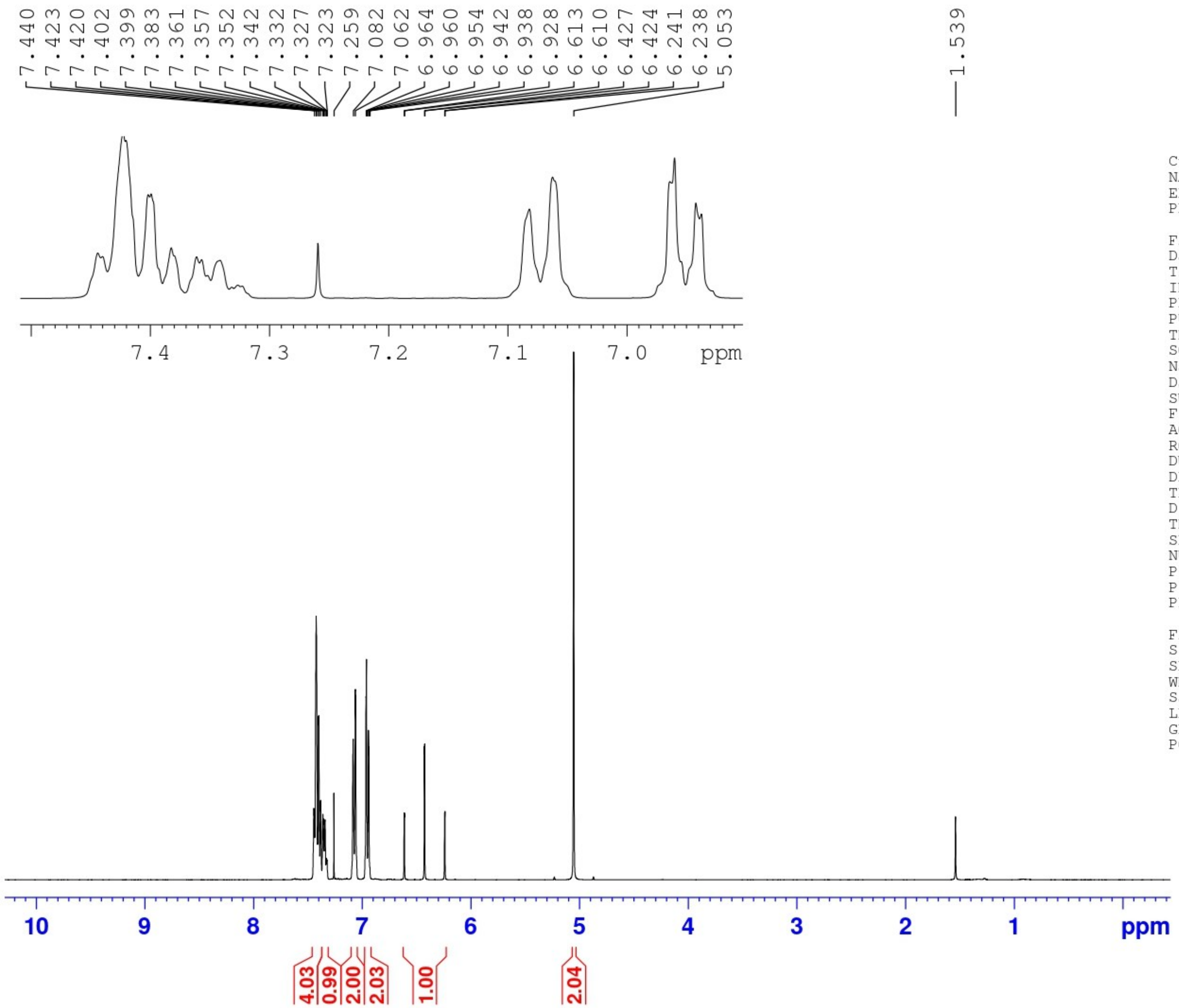

Current Data Parameters
NAME IX-Mn-104 i_10
EXPNO 2
PROCNO 1

F2 - Acquisition Parameters
Date_ 20251013
Time 16.36
INSTRUM Avance
PROBHD Z166552_0018 (PI HR-
PULPROG zg30
TD 65536
SOLVENT CDCl3
NS 16
DS 2
SWH 7812.500
FIDRES 0.238419
AQ 4.1943040
RG 101
DW 64.000
DE 6.67
TE 298.0
D1 1.00000000
TD0 1
SFO1 399.5424672
NUC1 1H
P0 2.60
P1 7.80
PLW1 21.19799995

F2 - Processing parameters
SI 65536
SF 399.5400097
WDW EM
SSB 0
LB 0.30
GB 0
PC 1.00

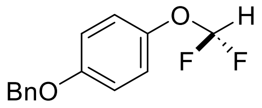

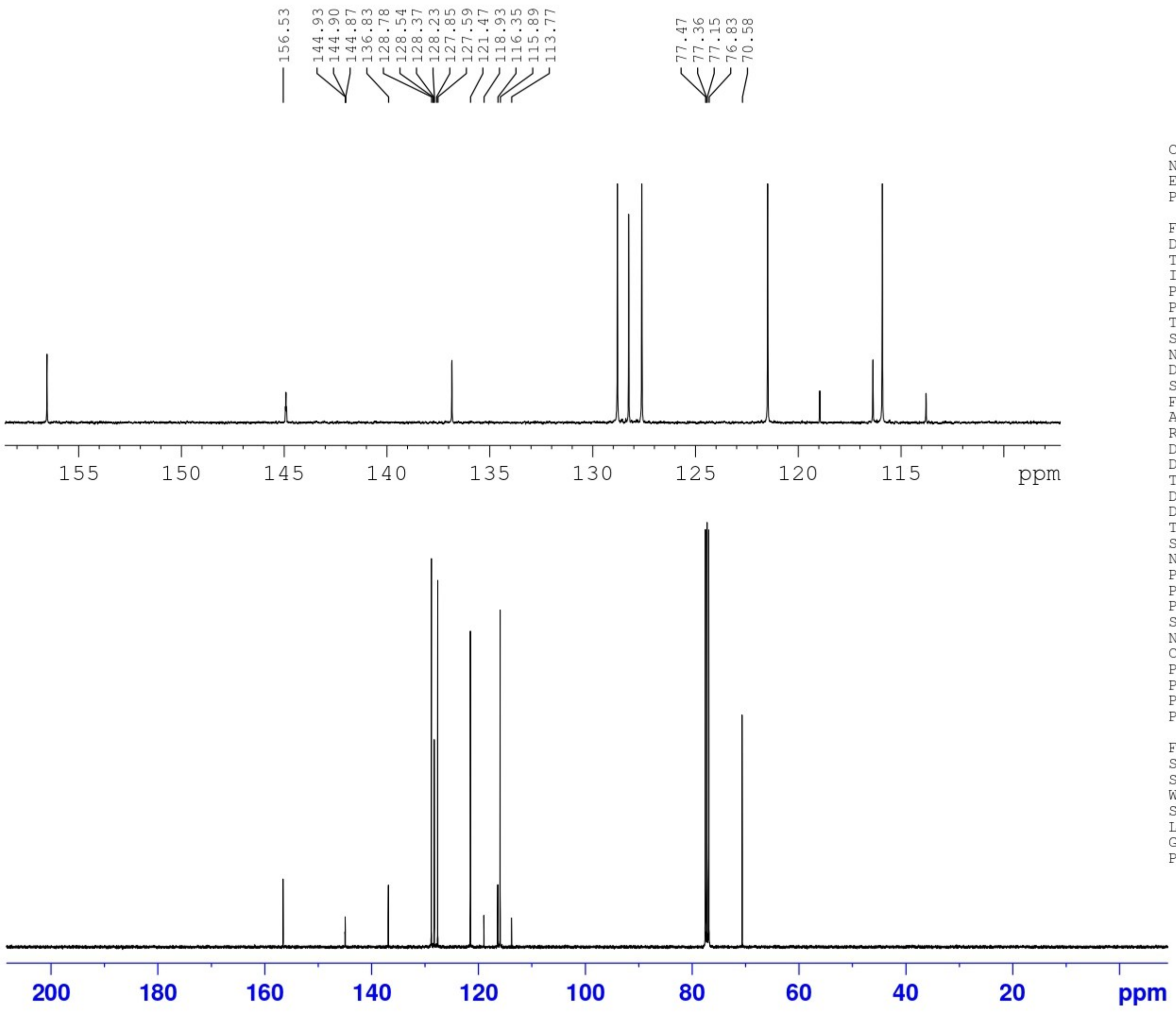

Current Data Parameters
NAME IX-Mn-104 i_12
EXPNO 2
PROCNO 1

F2 - Acquisition Parameters
Date_ 20251013
Time 20.02
INSTRUM Avance
PROBHD Z166552_0018 (PI HR-
PULPROG zgpg30
TD 65536
SOLVENT CDCl3
NS 2048
DS 4
SWH 23809.524
FIDRES 0.726609
AQ 1.3762560
RG 101
DW 21.000
DE 6.50
TE 298.0
D1 2.00000000
D11 0.03000000
TD0 1
SFO1 100.4744593
NUC1 13C
P0 2.67
P1 8.00
PLW1 88.22599792
SFO2 399.5415982
NUC2 1H
CPDPRG[2] waltz65
PCPD2 90.00
PLW2 21.19799995
PLW12 0.15922000
PLW13 0.08008700

F2 - Processing parameters
SI 32768
SF 100.4644011
WDW EM
SSB 0
LB 1.00
GB 0
PC 1.40

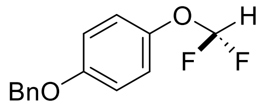

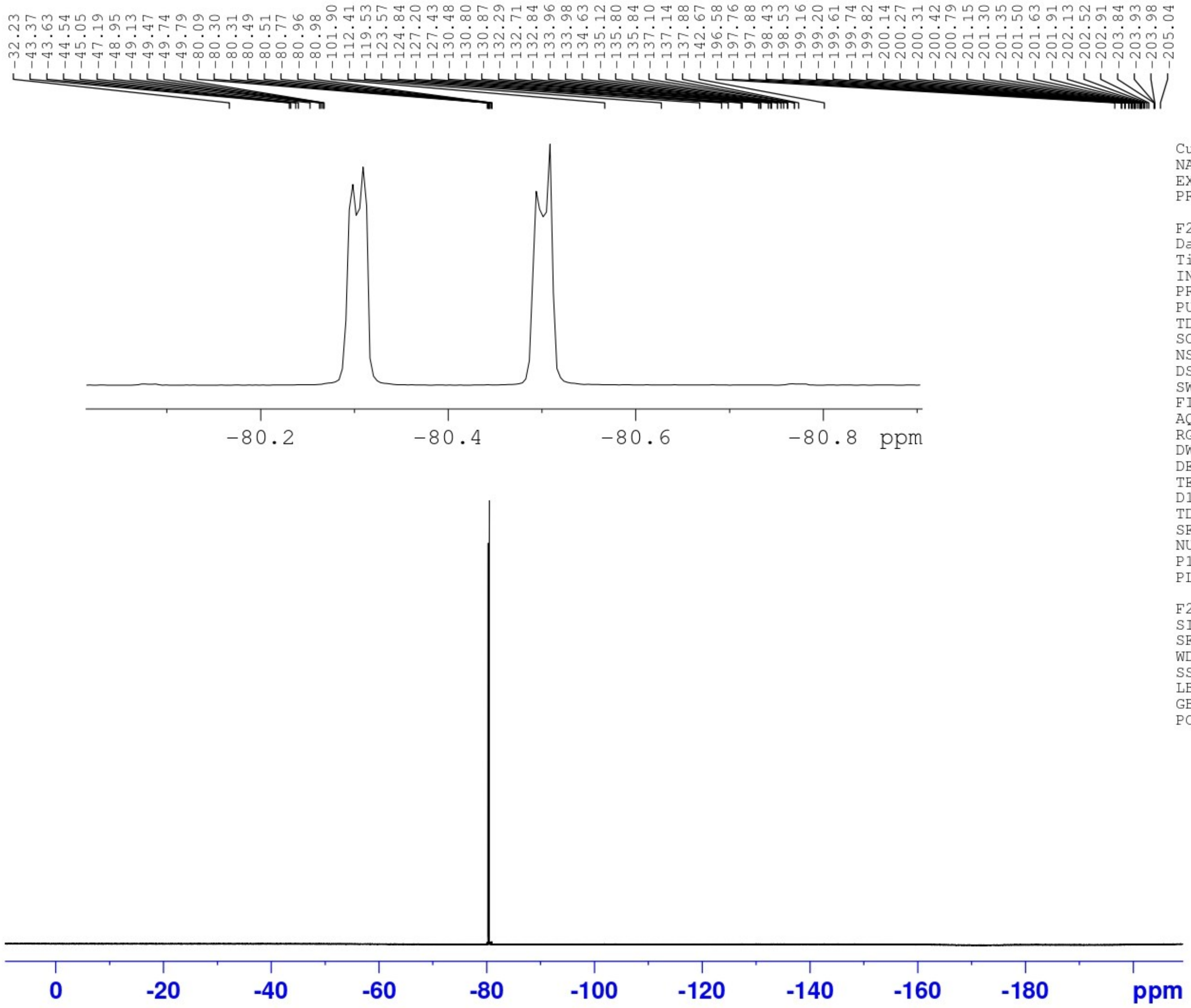

BRUKER

Current Data Parameters
NAME IX-Mn-104 i_11
EXPNO 2
PROCNO 1

F2 - Acquisition Parameters
Date_ 20251013
Time 16.37
INSTRUM Avance
PROBHD Z166552_0018 (PI HR-
PULPROG zg
TD 131072
SOLVENT CDCl3
NS 16
DS 4
SWH 90909.091
FIDRES 1.387163
AQ 0.7208960
RG 101
DW 5.500
DE 6.50
TE 298.0
D1 1.00000000
TD0 1
SFO1 375.9056172
NUC1 19F
P1 12.00
PLW1 32.47200012

F2 - Processing parameters
SI 65536
SF 375.9432115
WDW EM
SSB 0
LB 0.30
GB 0
PC 1.00

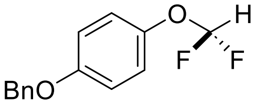

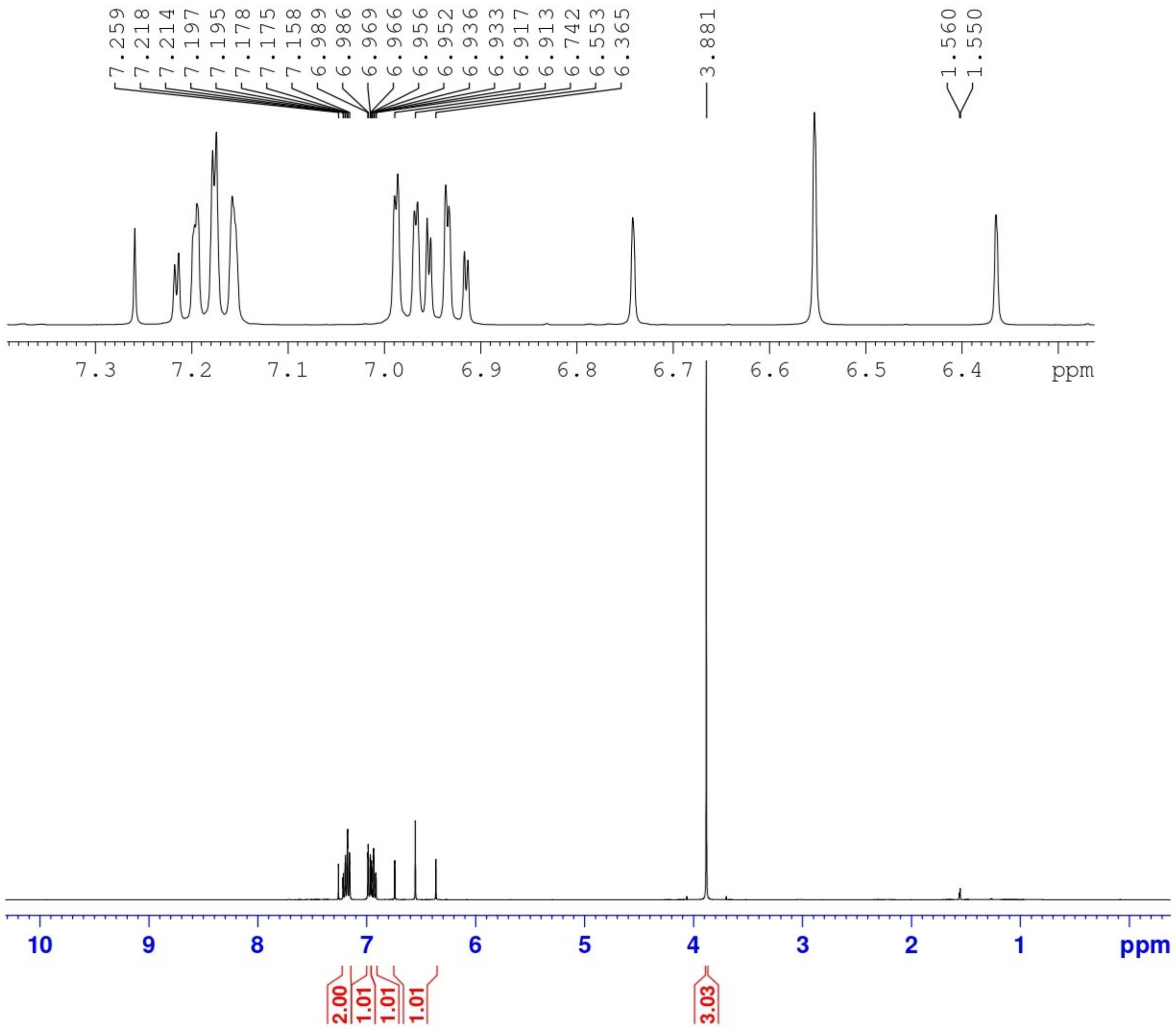

Current Data Parameters
NAME VI-Mn-122 i_10
EXPNO 3
PROCNO 1

F2 - Acquisition Parameters
Date_ 20241022
Time 15.10
INSTRUM Avance
PROBHD Z166552_0018 (PI HR-
PULPROG zg30
TD 65536
SOLVENT CDCl3
NS 16
DS 2
SWH 7812.500
FIDRES 0.238419
AQ 4.1943040
RG 101
DW 64.000
DE 6.67
TE 298.0
D1 1.00000000
TD0 1
SFO1 399.5701703
NUC1 1H
P0 2.60
P1 7.80
PLW1 21.19799995

F2 - Processing parameters
SI 65536
SF 399.5677128
WDW EM
SSB 0
LB 0.30
GB 0
PC 1.00

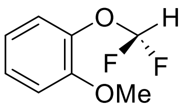

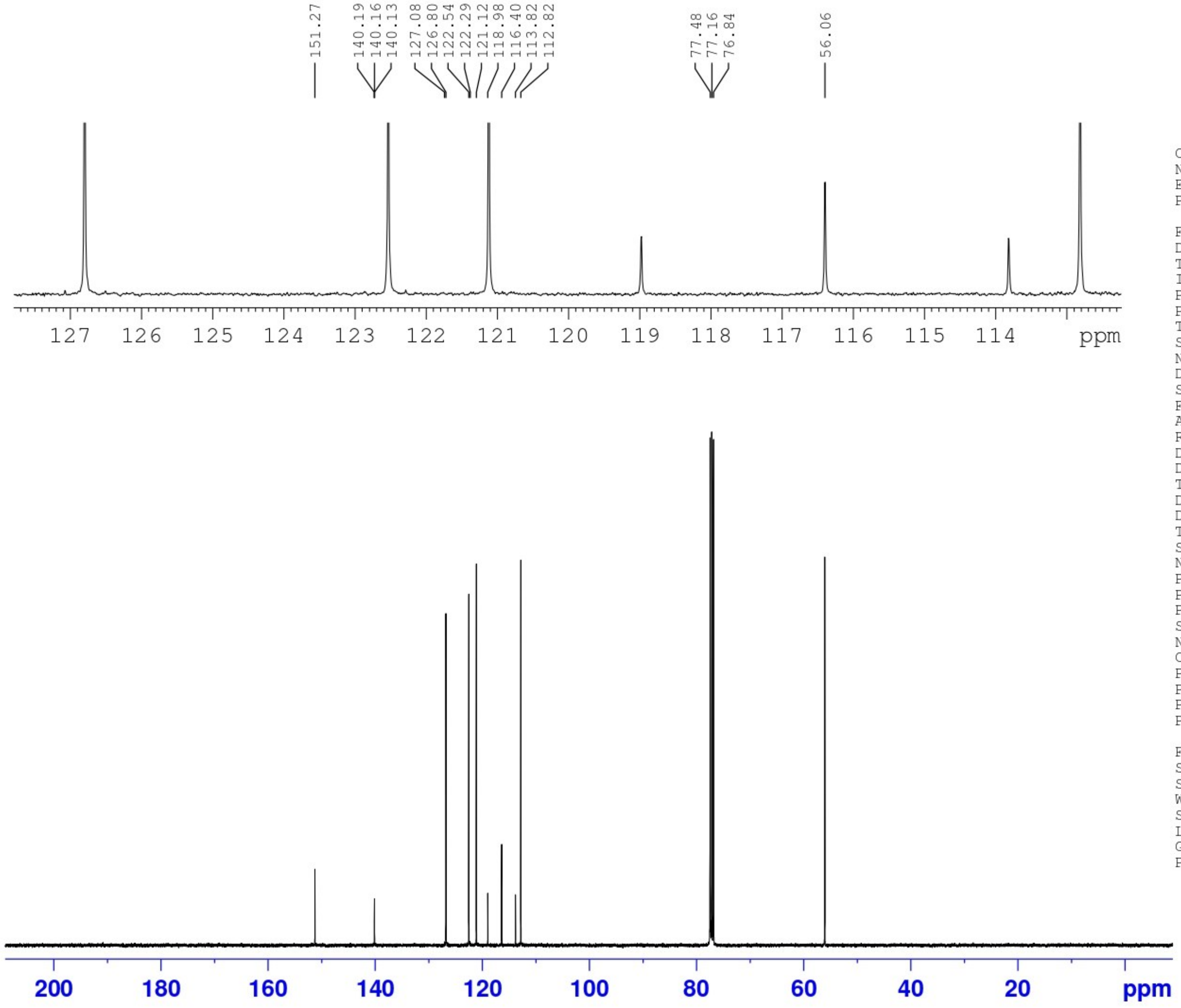

Current Data Parameters
NAME VI-Mn-122 i_12
EXPNO 3
PROCNO 1

F2 - Acquisition Parameters
Date_ 20241022
Time 20.03
INSTRUM Avance
PROBHD Z166552_0018 (PI HR-
PULPROG zgpg30
TD 65536
SOLVENT CDCl3
NS 2048
DS 4
SWH 23809.524
FIDRES 0.726609
AQ 1.3762560
RG 101
DW 21.000
DE 6.50
TE 298.0
D1 2.00000000
D11 0.03000000
TD0 1
SFO1 100.4814260
NUC1 13C
P0 2.67
P1 8.00
PLW1 88.22599792
SFO2 399.5693013
NUC2 1H
CPDPRG[2] waltz65
PCPD2 90.00
PLW2 21.19799995
PLW12 0.15922000
PLW13 0.08008700

F2 - Processing parameters
SI 32768
SF 100.4713660
WDW EM
SSB 0
LB 1.00
GB 0
PC 1.40

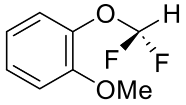

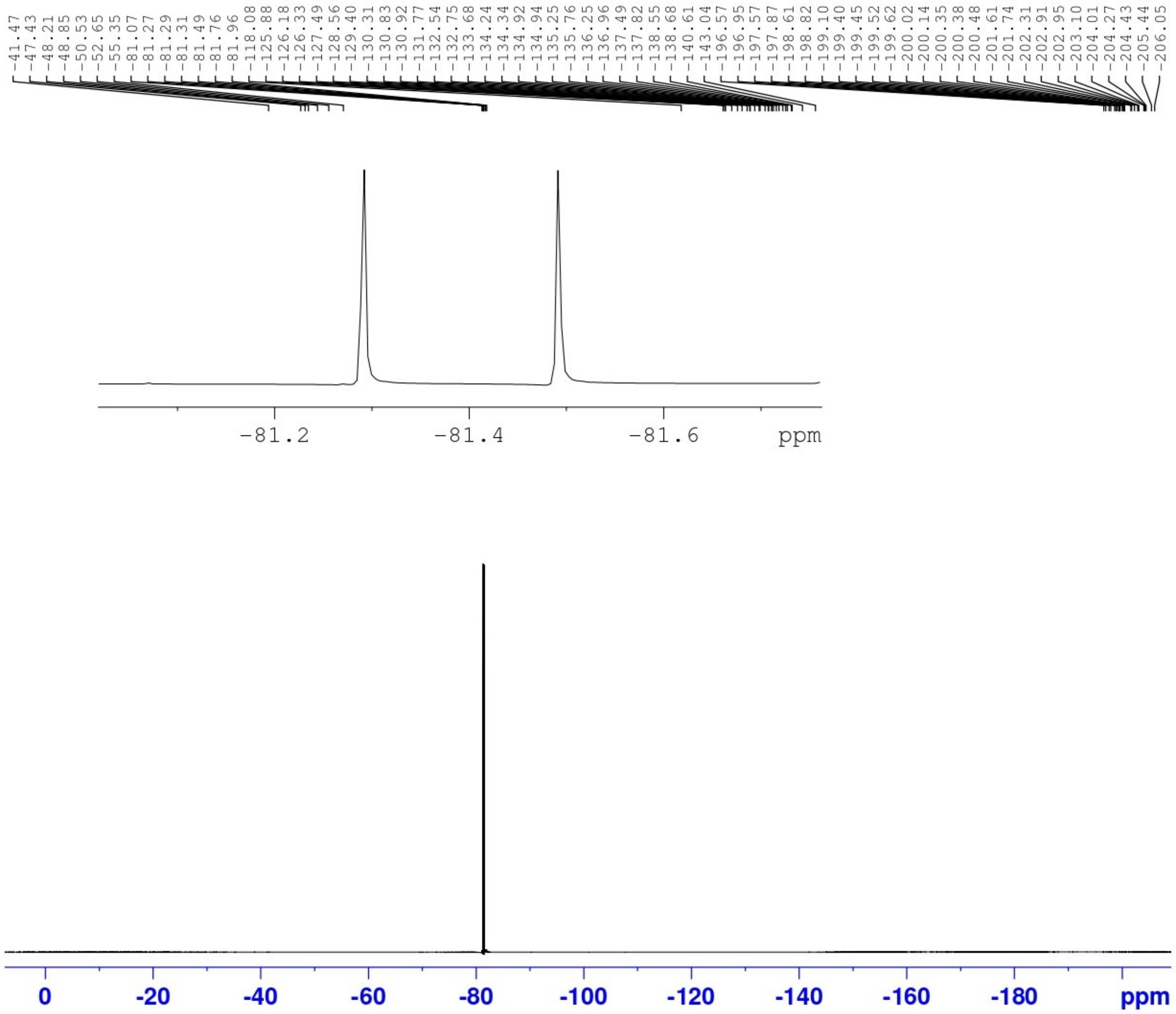

Current Data Parameters

NAME	VI-Mn-122 i_11
EXPNO	2
PROCNO	1

F2 - Acquisition Parameters

Date_	20241022
Time	15.12
INSTRUM	Avance
PROBHD	Z166552_0018 (PI HR-
PULPROG	zg
TD	131072
SOLVENT	CDCl3
NS	16
DS	4
SWH	90909.091
FIDRES	1.387163
AQ	0.7208960
RG	101
DW	5.500
DE	6.50
TE	298.0
D1	1.00000000
TD0	1
SFO1	375.9316815
NUC1	19F
P1	12.00
PLW1	32.47200012

F2 - Processing parameters

SI	65536
SF	375.9692784
WDW	EM
SSB	0
LB	0.30
GB	0
PC	1.00

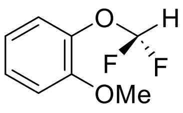

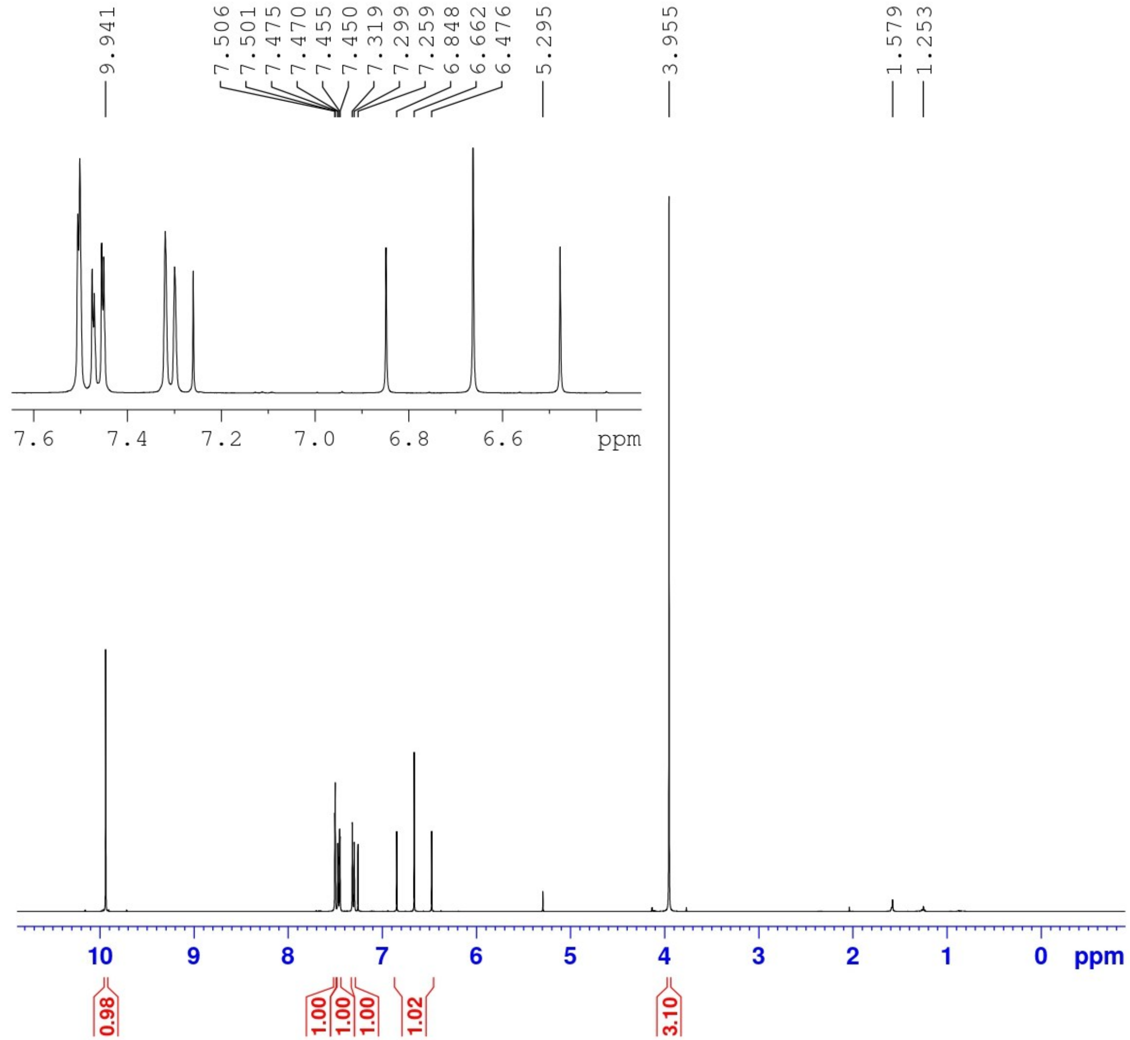

BRUKER

Current Data Parameters

NAME	IV-Mn-81 i_10
EXPNO	3
PROCNO	1

F2 - Acquisition Parameters

Date_	20240206
Time	17.37
INSTRUM	Avance
PROBHD	Z166552_0018 (PI HR-
PULPROG	zg30
TD	65536
SOLVENT	CDCl3
NS	16
DS	2
SWH	7812.500
FIDRES	0.238419
AQ	4.1943040
RG	101
DW	64.000
DE	6.67
TE	298.0
D1	1.00000000
TD0	1
SFO1	399.6024675
NUC1	1H
P0	2.60
P1	7.80
PLW1	21.19799995

F2 - Processing parameters

SI	65536
SF	399.6000099
WDW	EM
SSB	0
LB	0.30
GB	0
PC	1.00

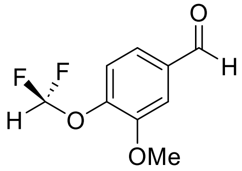

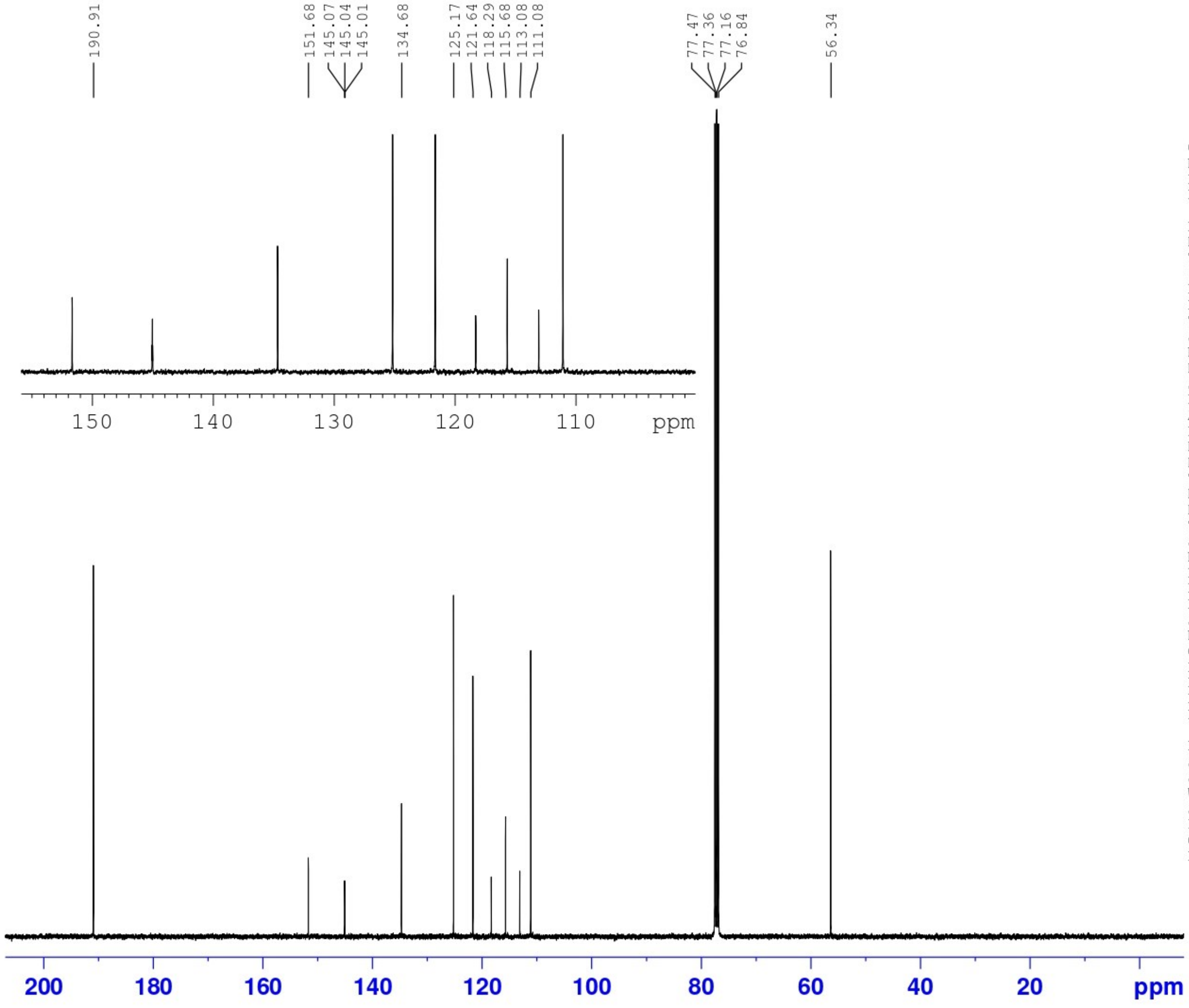


```
Current Data Parameters
NAME              IV-Mn-81 i_12
EXPNO                         2
PROCNO                        1

F2 - Acquisition Parameters
Date_                  20240206
Time                      20.09
INSTRUM                  Avance
PROBHD     Z166552_0018 (PI HR-
PULPROG                 zgpg30
TD                        65536
SOLVENT                   CDCl3
NS                         2048
DS                            4
SWH                   23809.524
FIDRES                 0.726609
AQ                    1.3762560
RG                          101
DW                       21.000
DE                         6.50
TE                        298.0
D1                   2.00000000
D11                  0.03000000
TD0                           1
SFO1                100.4895479
NUC1                        13C
P0                         2.67
P1                         8.00
PLW1                88.22599792
SFO2                399.6015984
NUC2                         1H
CPDPRG[2]               waltz65
PCPD2                     90.00
PLW2                21.19799995
PLW12                0.15922000
PLW13                0.08008700

F2 - Processing parameters
SI                        32768
SF                  100.4794869
WDW                          EM
SSB                           0
LB                         1.00
GB                            0
PC                         1.40
```

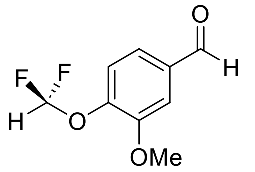

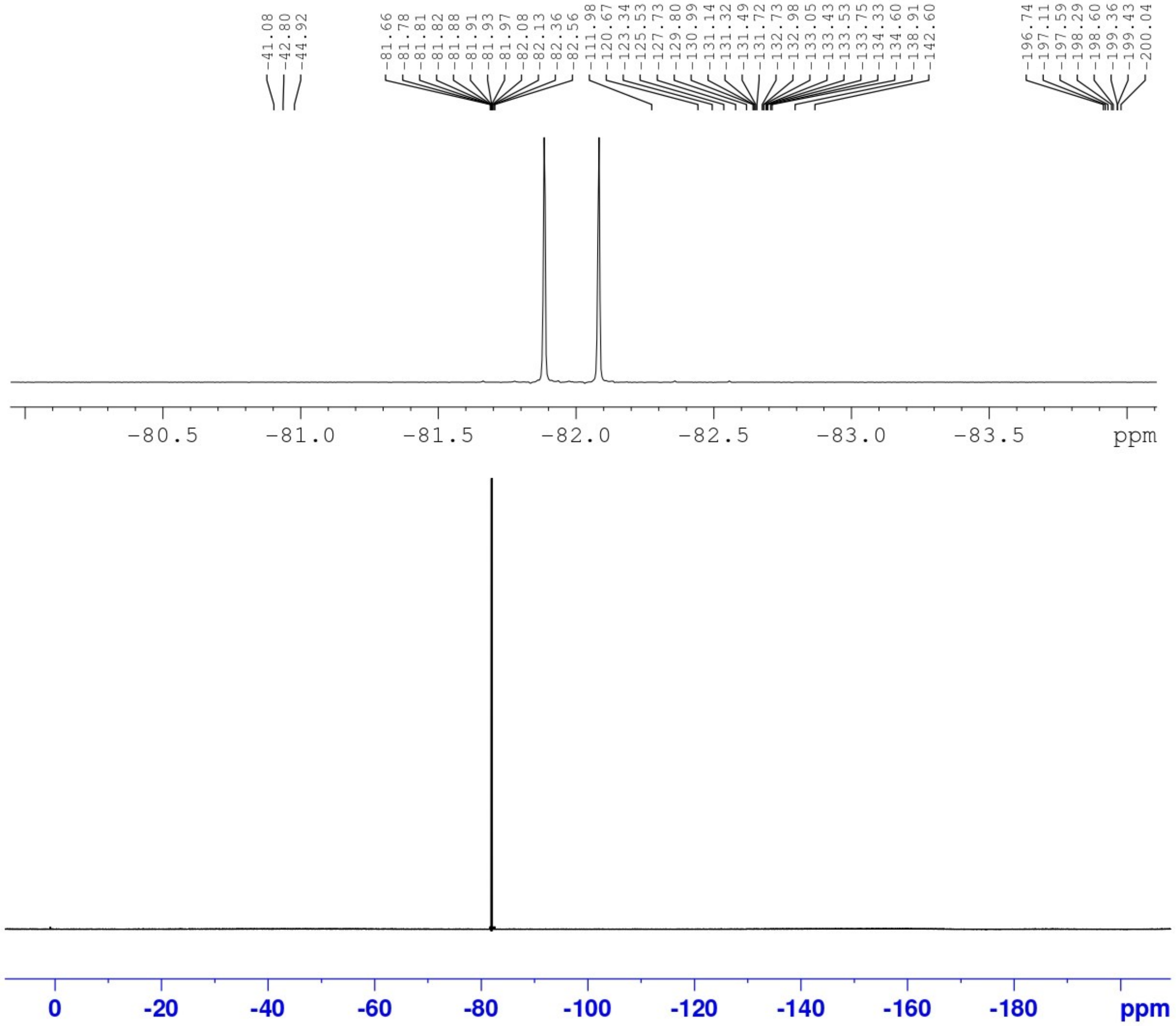

Current Data Parameters

NAME	IV-Mn-81 i_11
EXPNO	2
PROCNO	1

F2 - Acquisition Parameters

Date_	20240206
Time	17.39
INSTRUM	Avance
PROBHD	Z166552_0018 (PI HR-
PULPROG	zg
TD	131072
SOLVENT	CDCl3
NS	16
DS	4
SWH	90909.091
FIDRES	1.387163
AQ	0.7208960
RG	101
DW	5.500
DE	6.50
TE	298.0
D1	1.00000000
TD0	1
SFO1	375.9620680
NUC1	19F
P1	12.00
PLW1	32.47200012

F2 - Processing parameters

SI	65536
SF	375.9996680
WDW	EM
SSB	0
LB	0.30
GB	0
PC	1.00

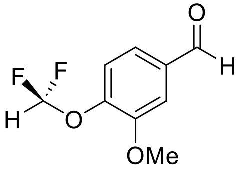

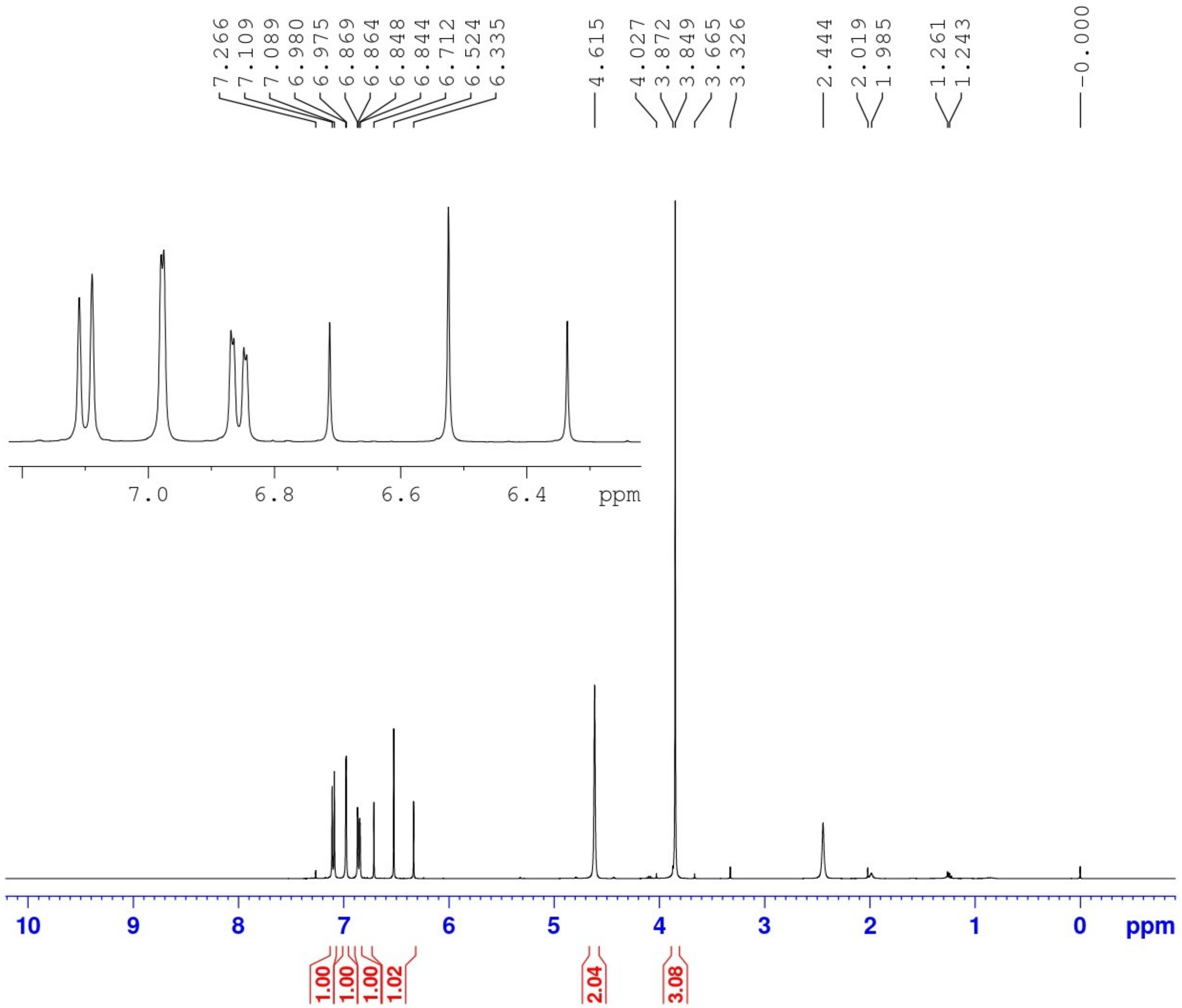


```
Current Data Parameters
NAME              VI-Mn-02 i_10
EXPNO                         4
PROCNO                        1

F2 - Acquisition Parameters
Date_                  20240730
Time                      17.56
INSTRUM                  Avance
PROBHD      Z166552_0018 (PI HR-
PULPROG                    zg30
TD                        65536
SOLVENT                   CDCl3
NS                           16
DS                            2
SWH                    7812.500
FIDRES                 0.238419
AQ                    4.1943040
RG                         45.2
DW                       64.000
DE                         6.67
TE                        298.0
D1                   1.00000000
TD0                           1
SFO1                399.5701703
NUC1                         1H
P0                         2.60
P1                         7.80
PLW1                21.19799995

F2 - Processing parameters
SI                        65536
SF                  399.5677101
WDW                          EM
SSB                           0
LB                         0.30
GB                            0
PC                         1.00
```

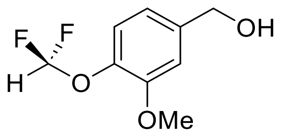

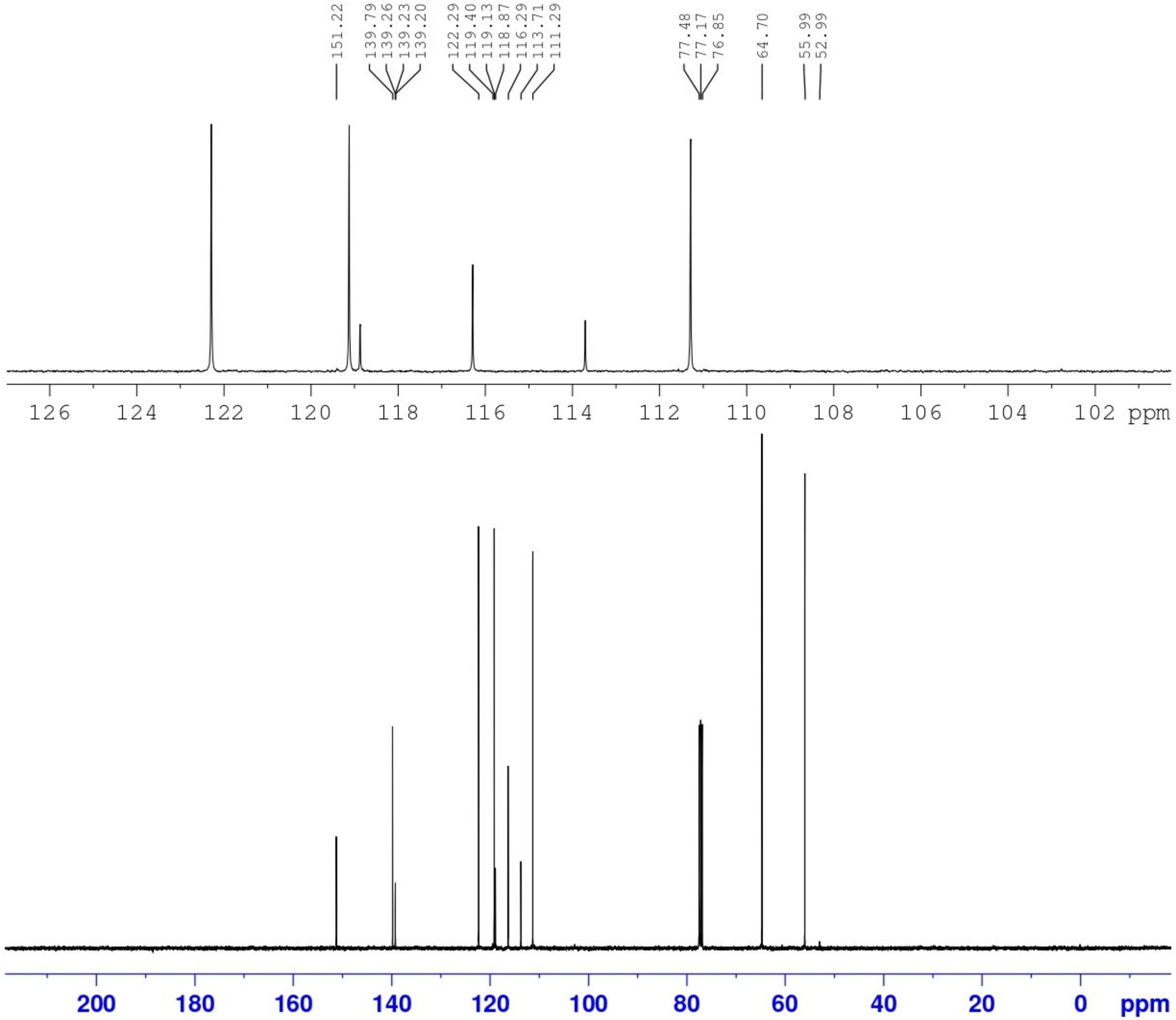

Current Data Parameters	
NAME	VI-Mn-02 i_12
EXPNO	2
PROCNO	1

F2 - Acquisition Parameters	
Date_	20240730
Time	18.16
INSTRUM	Avance
PROBHD	Z166552_0018 (PI HR-
PULPROG	zgpg30
TD	65536
SOLVENT	CDCl3
NS	256
DS	4
SWH	23809.524
FIDRES	0.726609
AQ	1.3762560
RG	101
DW	21.000
DE	6.50
TE	298.0
D1	2.00000000
D11	0.03000000
TD0	1
SFO1	100.4814260
NUC1	13C
P0	2.67
P1	8.00
PLW1	88.22599792
SFO2	399.5693013
NUC2	1H
CPDPRG[2]	waltz65
PCPD2	90.00
PLW2	21.19799995
PLW12	0.15922000
PLW13	0.08008700

F2 - Processing parameters	
SI	32768
SF	100.4713709
WDW	EM
SSB	0
LB	1.00
GB	0
PC	1.40

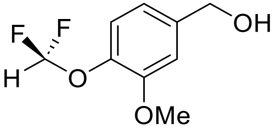

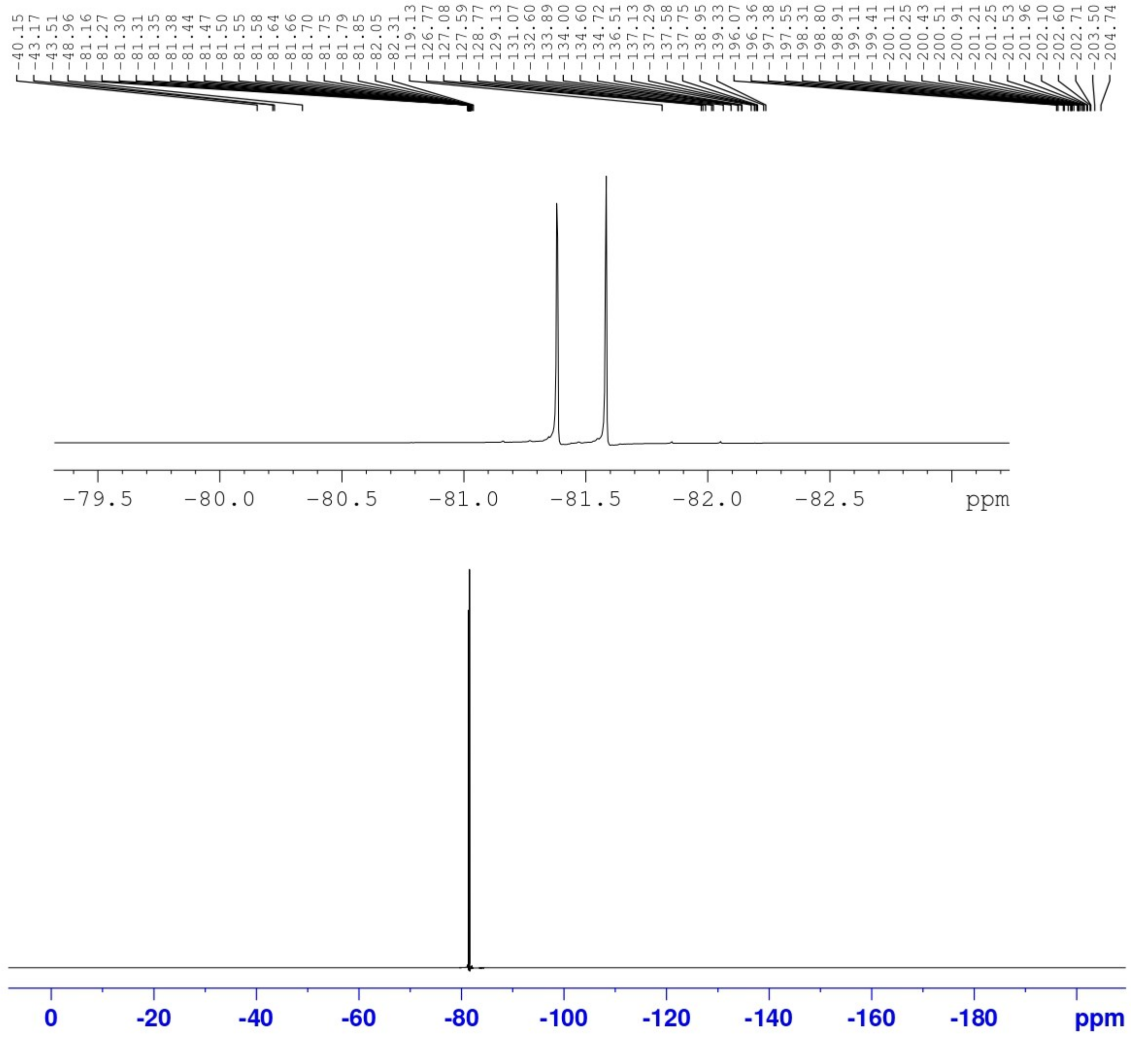

Current Data Parameters
NAME VI-Mn-02 i_11
EXPNO 3
PROCNO 1

F2 - Acquisition Parameters
Date_ 20240730
Time 17.59
INSTRUM Avance
PROBHD Z166552_0018 (PI HR-
PULPROG zg
TD 131072
SOLVENT CDCl3
NS 16
DS 4
SWH 90909.091
FIDRES 1.387163
AQ 0.7208960
RG 101
DW 5.500
DE 6.50
TE 298.0
D1 1.00000000
TD0 1
SFO1 375.9316815
NUC1 19F
P1 12.00
PLW1 32.47200012

F2 - Processing parameters
SI 65536
SF 375.9692784
WDW EM
SSB 0
LB 0.30
GB 0
PC 1.00

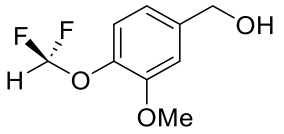

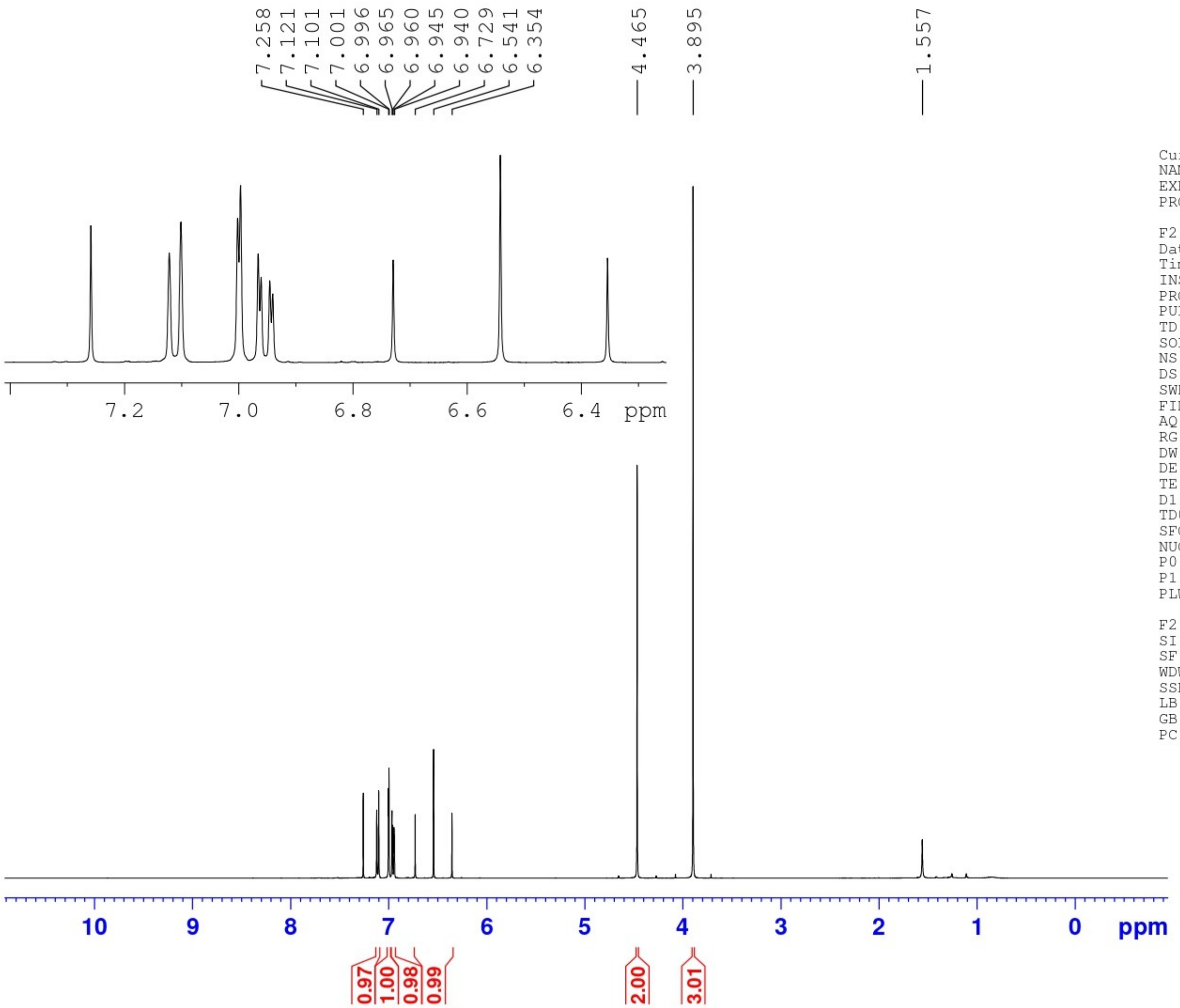

Current Data Parameters
NAME VIII-Mn-129 i_10
EXPNO 1
PROCNO 1

F2 - Acquisition Parameters
Date_ 20250423
Time 17.10
INSTRUM Avance
PROBHD Z166552_0018 (PI HR-
PULPROG zg30
TD 65536
SOLVENT CDCl3
NS 16
DS 2
SWH 7812.500
FIDRES 0.238419
AQ 4.1943040
RG 101
DW 64.000
DE 6.67
TE 298.0
D1 1.00000000
TD0 1
SFO1 399.5701703
NUC1 1H
P0 2.60
P1 7.80
PLW1 21.19799995

F2 - Processing parameters
SI 65536
SF 399.5677132
WDW EM
SSB 0
LB 0.30
GB 0
PC 1.00

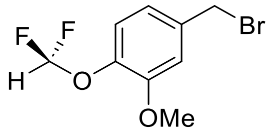

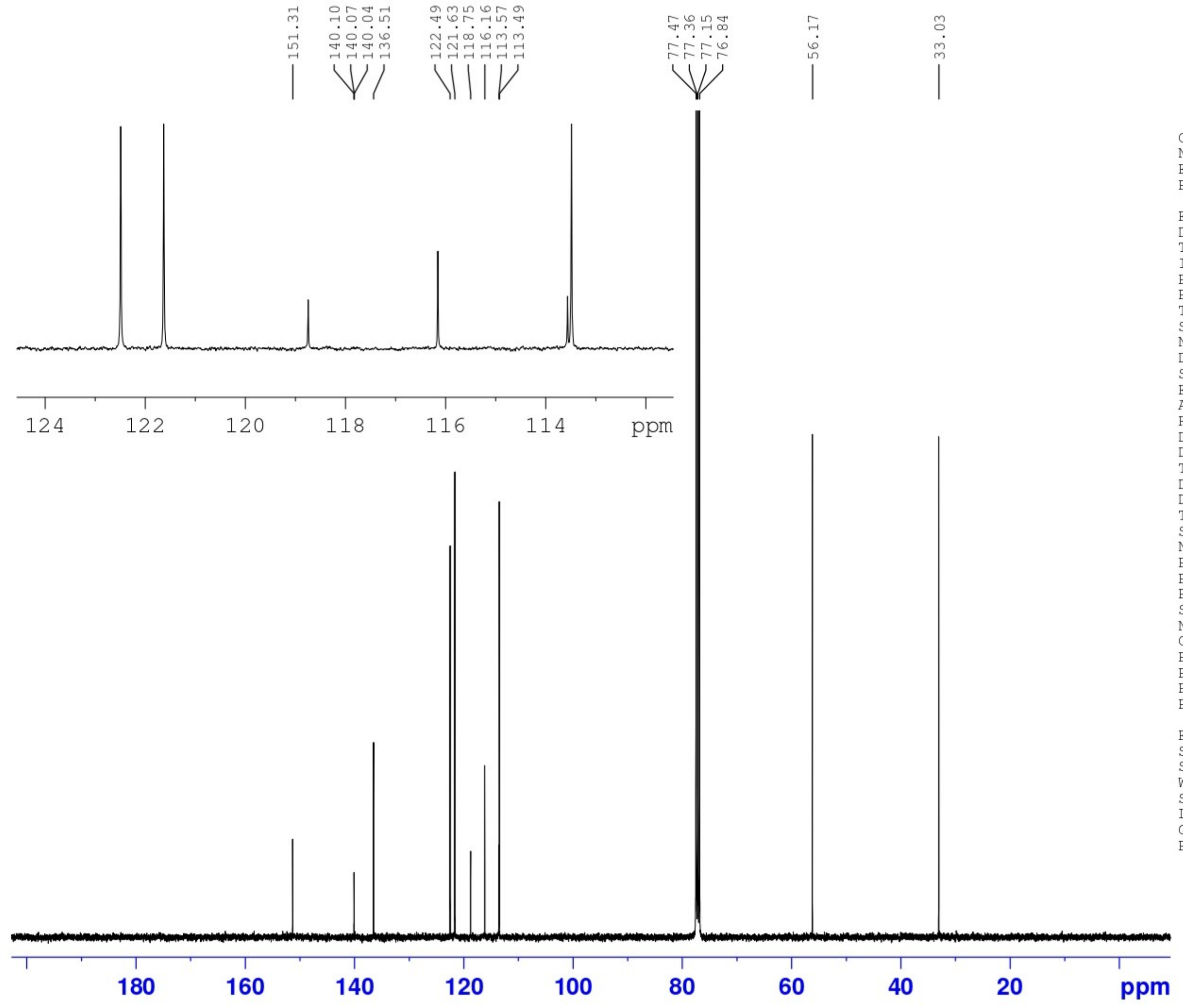


```
Current Data Parameters
NAME            VIII-Mn-129 i_12
EXPNO                          1
PROCNO                         1

F2 - Acquisition Parameters
Date_                   20250424
Time                        0.19
INSTRUM                   Avance
PROBHD     Z166552_0018 (PI HR-
PULPROG                  zgpg30
TD                         65536
SOLVENT                    CDCl3
NS                          2048
DS                             4
SWH                    23809.524
FIDRES                  0.726609
AQ                     1.3762560
RG                           101
DW                        21.000
DE                          6.50
TE                         298.0
D1                    2.00000000
D11                   0.03000000
TD0                            1
SFO1                 100.4814260
NUC1                         13C
P0                          2.67
P1                          8.00
PLW1                 88.22599792
SFO2                 399.5693013
NUC2                          1H
CPDPRG[2]                waltz65
PCPD2                      90.00
PLW2                 21.19799995
PLW12                 0.15922000
PLW13                 0.08008700

F2 - Processing parameters
SI                         32768
SF                   100.4713662
WDW                           EM
SSB                            0
LB                          1.00
GB                             0
PC                          1.40
```

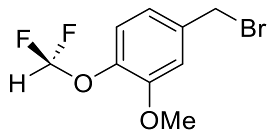

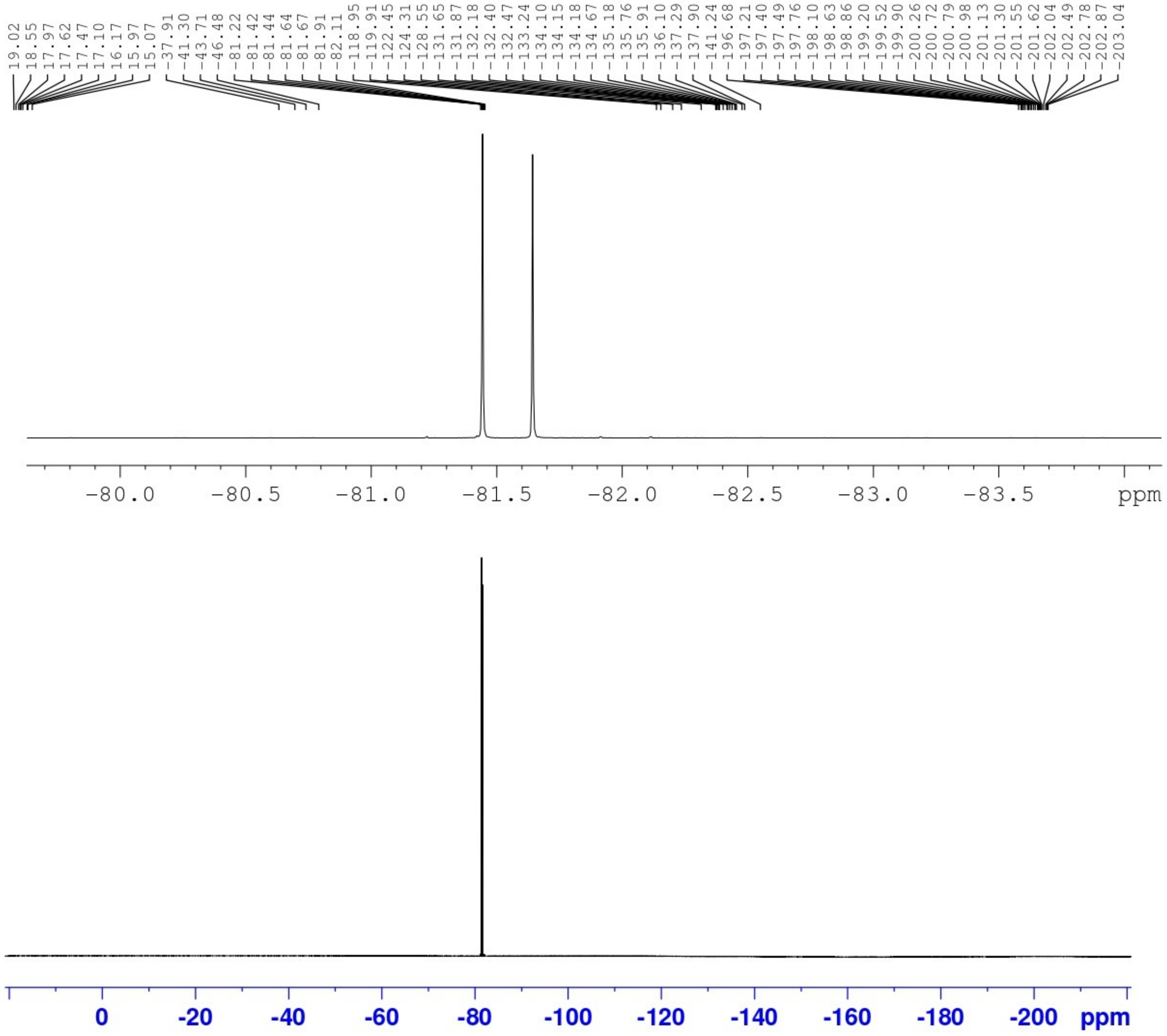

BRUKER

Current Data Parameters
NAME VIII-Mn-129 i_11
EXPNO 1
PROCNO 1

F2 - Acquisition Parameters
Date_ 20250423
Time 17.15
INSTRUM Avance
PROBHD Z166552_0018 (PI HR-
PULPROG zg
TD 131072
SOLVENT CDCl3
NS 16
DS 4
SWH 90909.091
FIDRES 1.387163
AQ 0.7208960
RG 101
DW 5.500
DE 6.50
TE 298.0
D1 1.00000000
TD0 1
SFO1 375.9316815
NUC1 19F
P1 12.00
PLW1 32.47200012

F2 - Processing parameters
SI 65536
SF 375.9692784
WDW EM
SSB 0
LB 0.30
GB 0
PC 1.00

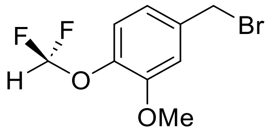

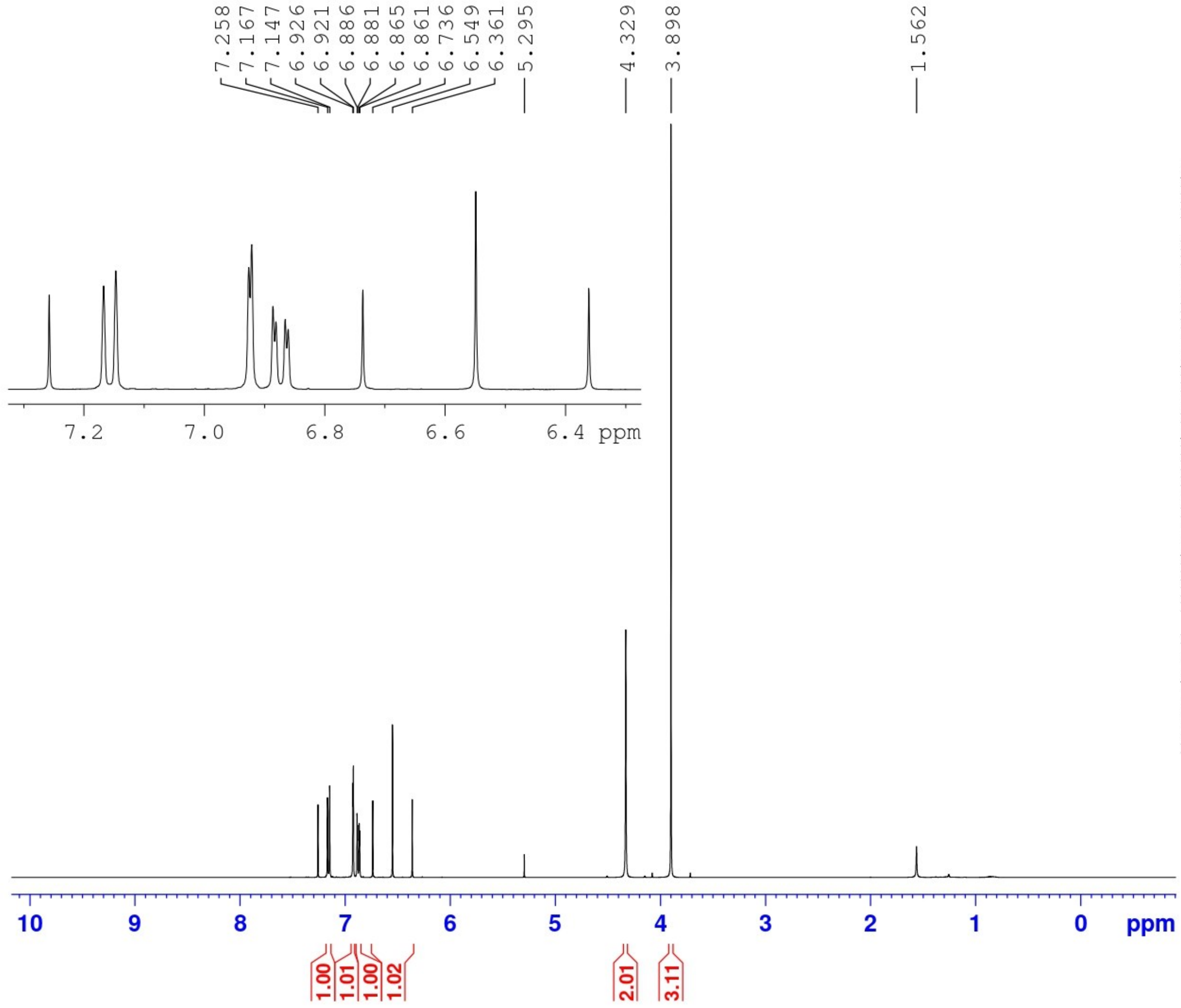

BRUKER

Current Data Parameters
NAME VIII-Mn-135 i_10
EXPNO 2
PROCNO 1

F2 - Acquisition Parameters
Date_ 20250426
Time 20.03
INSTRUM Avance
PROBHD Z166552_0018 (PI HR-
PULPROG zg30
TD 65536
SOLVENT CDCl3
NS 16
DS 2
SWH 7812.500
FIDRES 0.238419
AQ 4.1943040
RG 101
DW 64.000
DE 6.67
TE 298.0
D1 1.00000000
TD0 1
SFO1 399.5701703
NUC1 1H
P0 2.60
P1 7.80
PLW1 21.19799995

F2 - Processing parameters
SI 65536
SF 399.5677135
WDW EM
SSB 0
LB 0.30
GB 0
PC 1.00

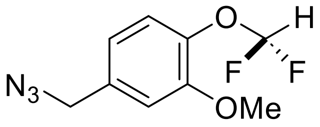

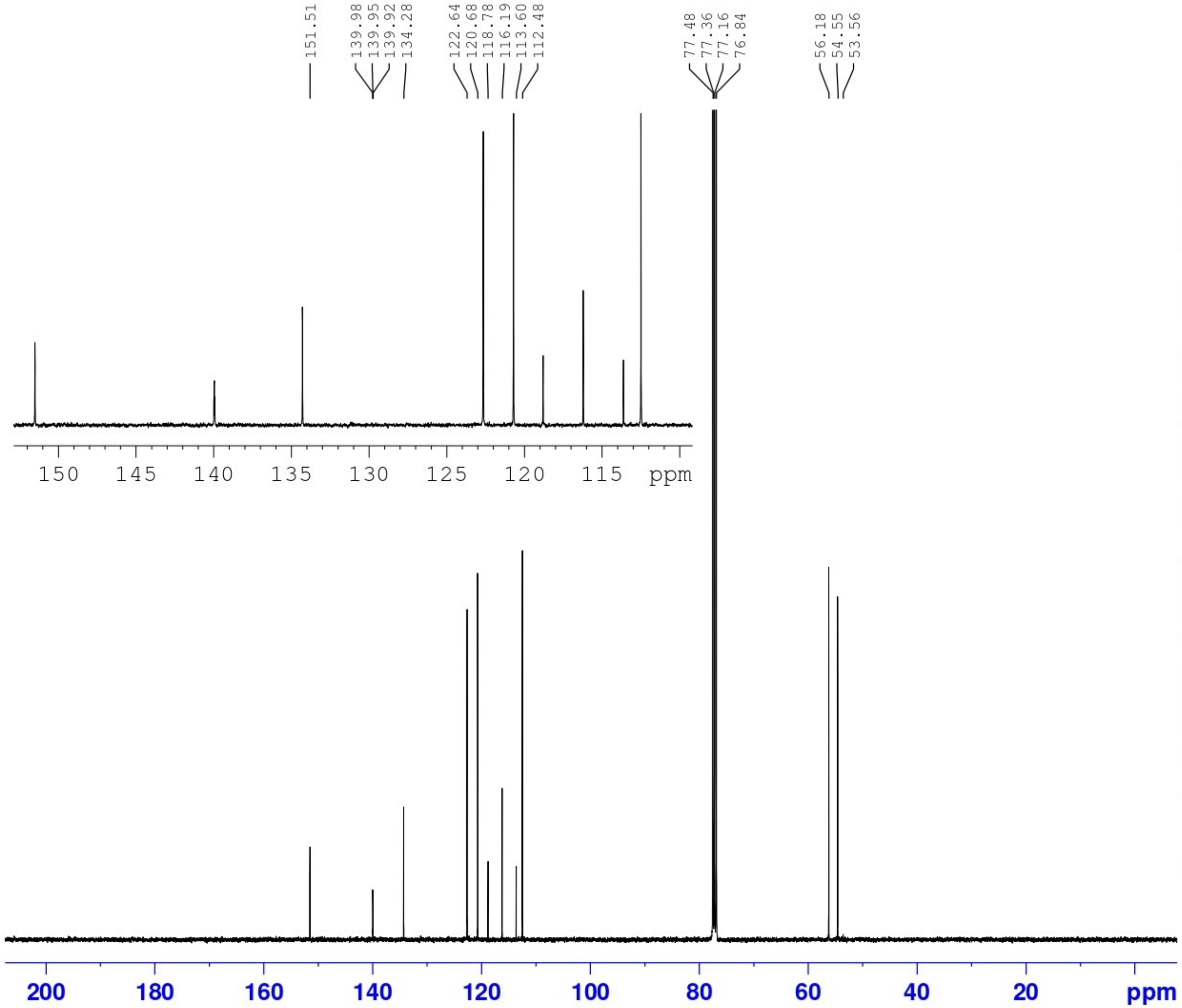

BRUKER

Current Data Parameters	
NAME	VIII-Mn-135 i_12
EXPNO	2
PROCNO	1
F2 - Acquisition Parameters	
Date_	20250426
Time	22.04
INSTRUM	Avance
PROBHD	Z166552_0018 (PI HR-
PULPROG	zgpg30
TD	65536
SOLVENT	CDCl3
NS	2048
DS	4
SWH	23809.524
FIDRES	0.726609
AQ	1.3762560
RG	101
DW	21.000
DE	6.50
TE	298.0
D1	2.00000000
D11	0.03000000
TD0	1
SFO1	100.4814260
NUC1	13C
P0	2.67
P1	8.00
PLW1	88.22599792
SFO2	399.5693013
NUC2	1H
CPDPRG[2]	waltz65
PCPD2	90.00
PLW2	21.19799995
PLW12	0.15922000
PLW13	0.08008700
F2 - Processing parameters	
SI	32768
SF	100.4713661
WDW	EM
SSB	0
LB	1.00
GB	0
PC	1.40

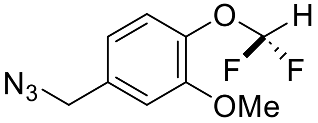

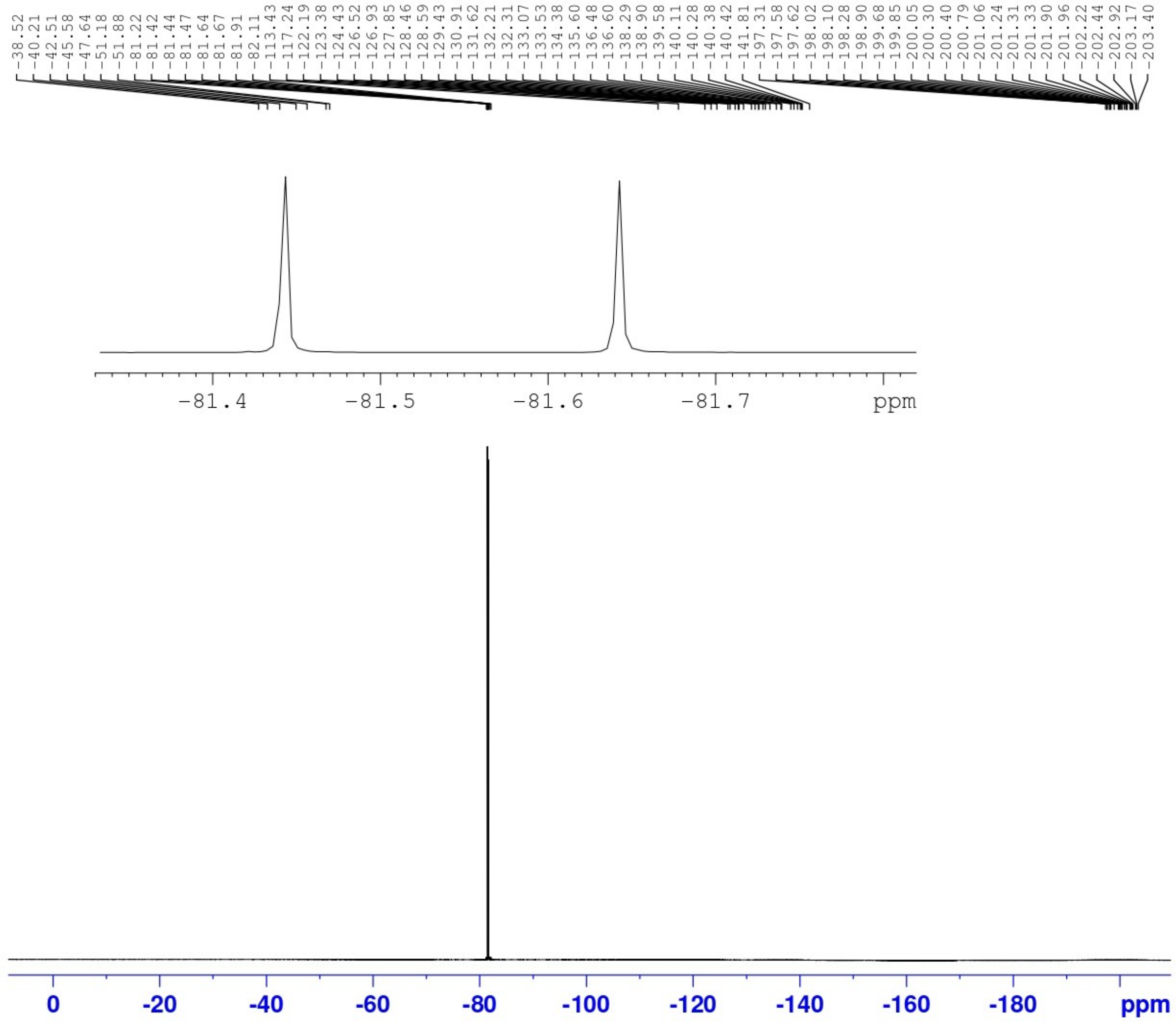

Current Data Parameters
NAME VIII-Mn-135 i_11
EXPNO 2
PROCNO 1

F2 - Acquisition Parameters
Date_ 20250426
Time 20.05
INSTRUM Avance
PROBHD Z166552_0018 (PI HR-
PULPROG zg
TD 131072
SOLVENT CDCl3
NS 16
DS 4
SWH 90909.091
FIDRES 1.387163
AQ 0.7208960
RG 101
DW 5.500
DE 6.50
TE 298.0
D1 1.00000000
TD0 1
SFO1 375.9316815
NUC1 19F
P1 12.00
PLW1 32.47200012

F2 - Processing parameters
SI 65536
SF 375.9692784
WDW EM
SSB 0
LB 0.30
GB 0
PC 1.00

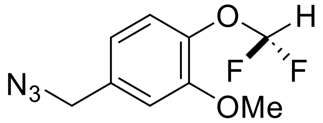

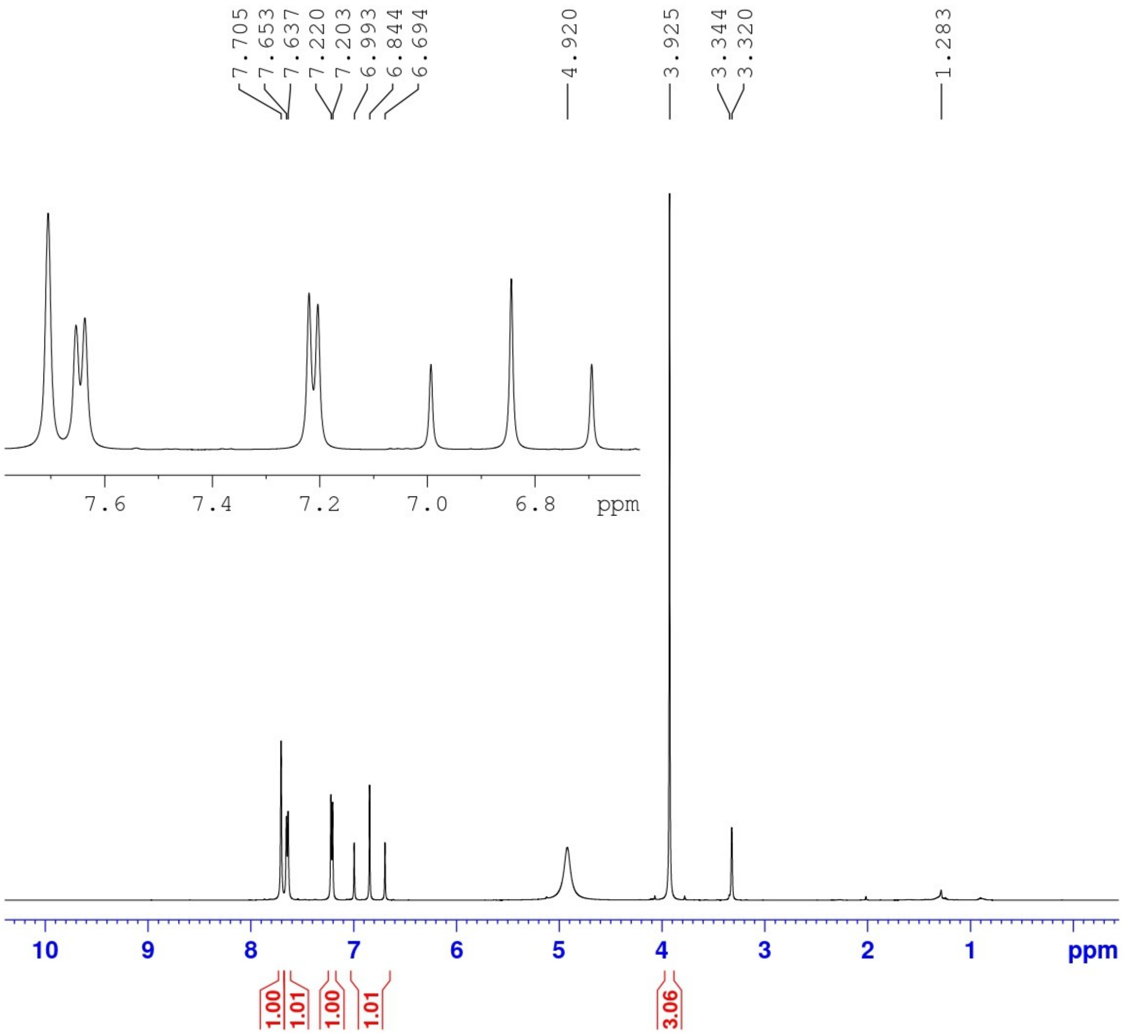

Current Data Parameters
NAME VIII-Mn-128_10
EXPNO 4
PROCNO 1

F2 - Acquisition Parameters
Date_ 20250422
Time 15.15
INSTRUM AS500-NEO
PROBHD Z168772_0026 (CPP1.1
PULPROG zg30
TD 65536
SOLVENT MeOD
NS 16
DS 2
SWH 10000.000
FIDRES 0.305176
AQ 3.2767999
RG 45.2
DW 50.000
DE 10.45
TE 298.0
D1 1.00000000
TD0 1
SFO1 499.7860862
NUC1 1H
P0 4.00
P1 12.00
PLW1 16.91500092

F2 - Processing parameters
SI 65536
SF 499.7830034
WDW EM
SSB 0
LB 0.30
GB 0
PC 1.00

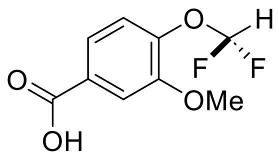

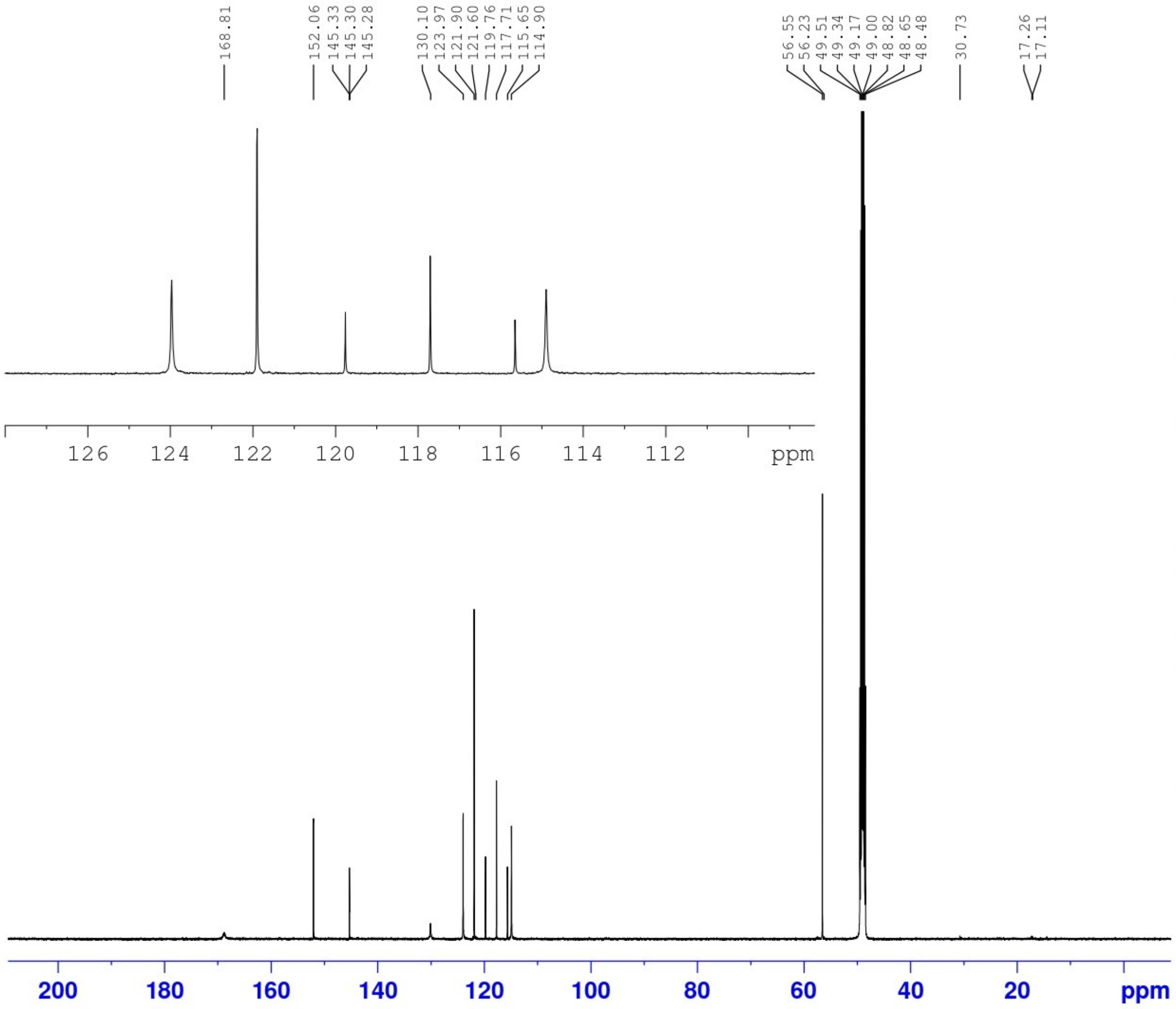

BRUKER

Current Data Parameters
NAME VIII-Mn-128_12
EXPNO 4
PROCNO 1

F2 - Acquisition Parameters
Date_ 20250422
Time 16.12
INSTRUM AS500-NEO
PROBHD Z168772_0026 (CPP1.1
PULPROG zgpg30
TD 65536
SOLVENT MeOD
NS 1024
DS 4
SWH 30120.482
FIDRES 0.919204
AQ 1.0878977
RG 101
DW 16.600
DE 18.00
TE 298.0
D1 2.00000000
D11 0.03000000
TD0 1
SFO1 125.6831024
NUC1 13C
P0 3.33
P1 10.00
PLW1 59.16400146
SFO2 499.7849991
NUC2 1H
CPDPRG[2] waltz65
PCPD2 80.00
PLW2 16.91500092
PLW12 0.38058999
PLW13 0.19113000

F2 - Processing parameters
SI 32768
SF 125.6703613
WDW EM
SSB 0
LB 1.00
GB 0
PC 1.40

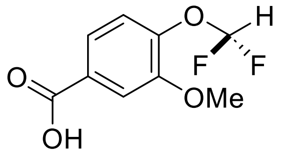

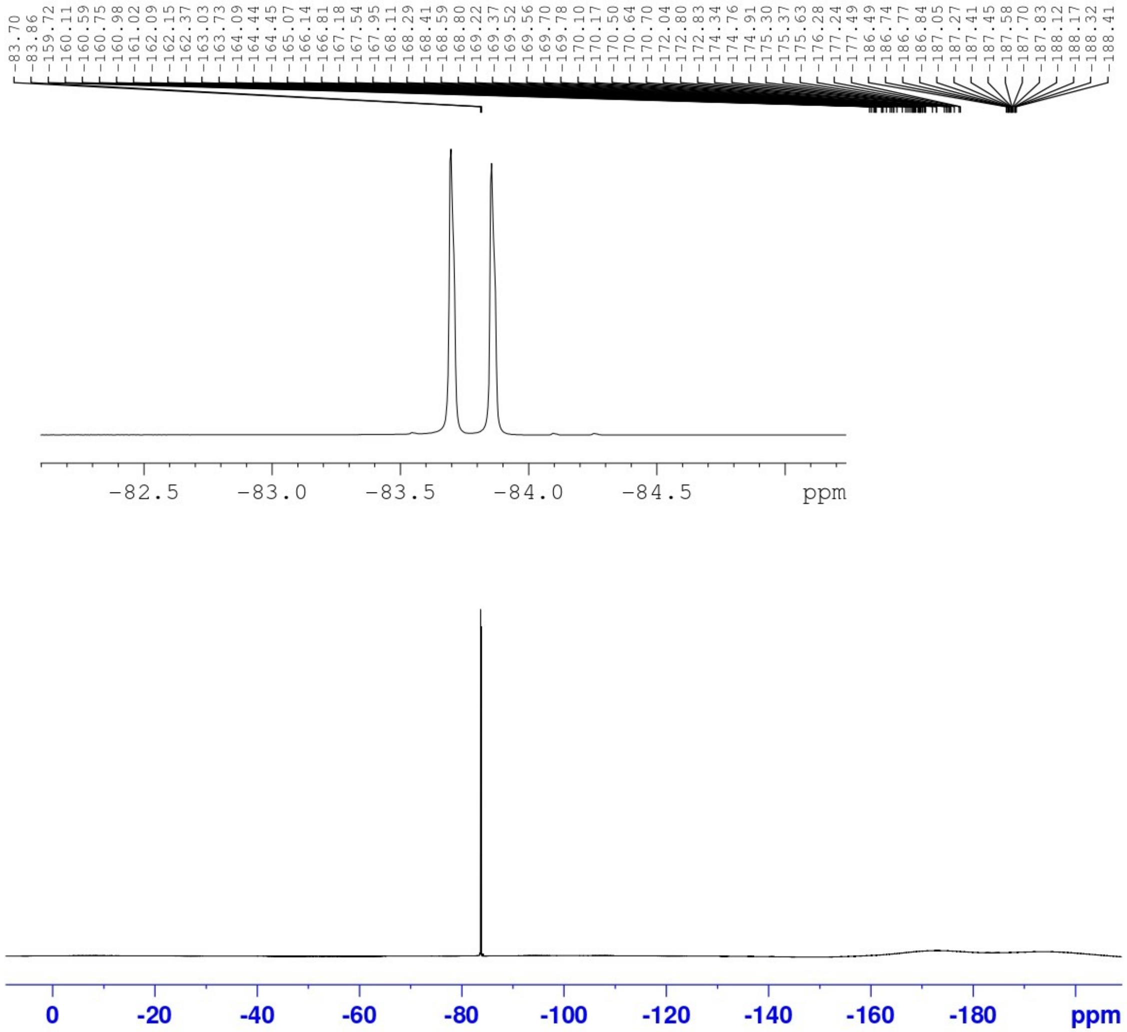

Current Data Parameters	
NAME	VIII-Mn-128_11
EXPNO	4
PROCNO	1

F2 - Acquisition Parameters	
Date_	20250422
Time	15.17
INSTRUM	AS500-NEO
PROBHD	Z168772_0026 (CPP1.1
PULPROG	zg
TD	131072
SOLVENT	MeOD
NS	16
DS	4
SWH	113636.364
FIDRES	1.733953
AQ	0.5767168
RG	10
DW	4.400
DE	18.00
TE	298.0
D1	1.00000000
TD0	1
SFO1	470.2188444
NUC1	19F
P1	15.00
PLW1	10.89000034

F2 - Processing parameters	
SI	65536
SF	470.2658710
WDW	EM
SSB	0
LB	0.30
GB	0
PC	1.00

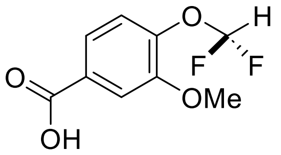

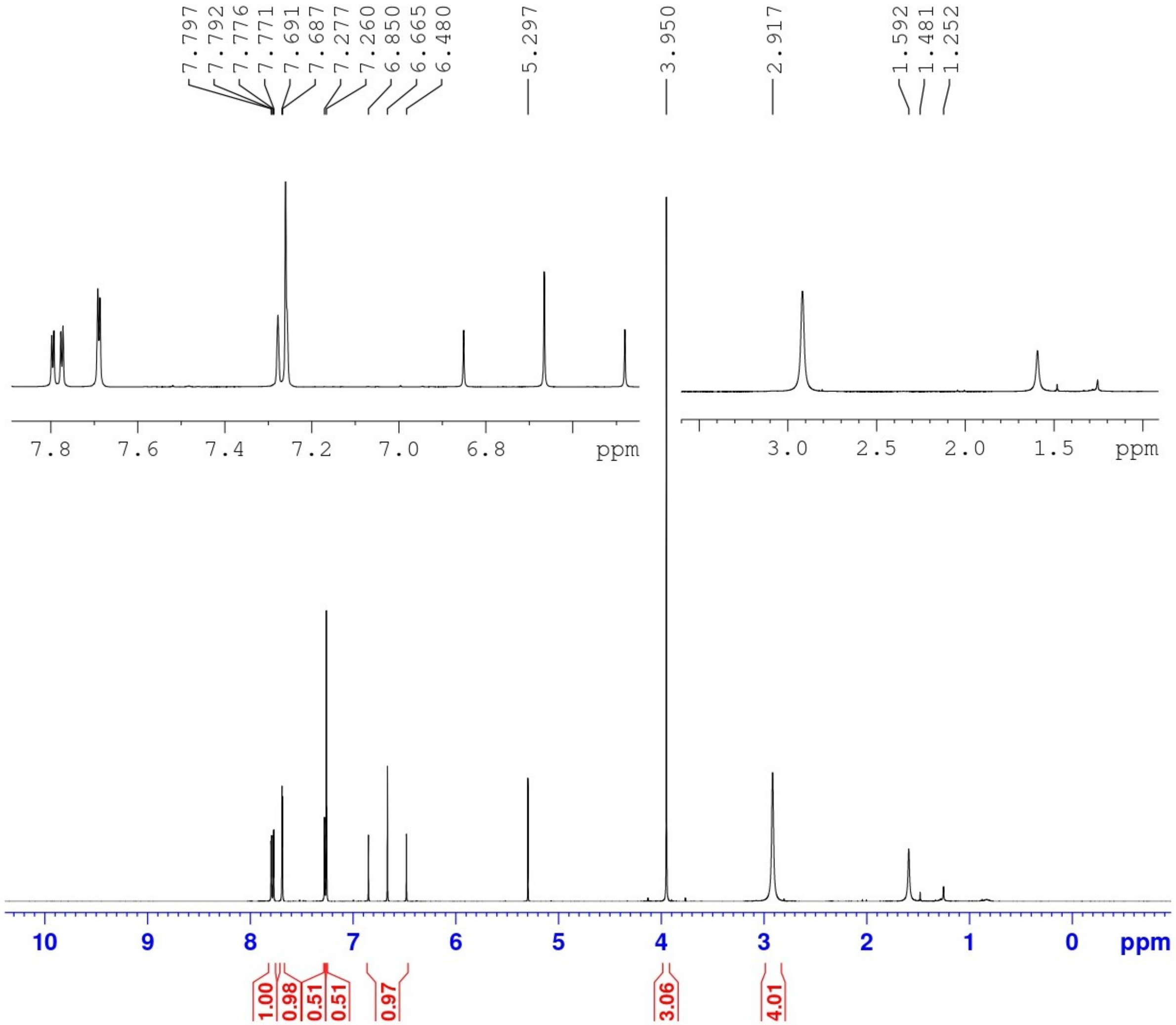

BRUKER

Current Data Parameters
NAME VIII-Mn-132 i re_10
EXPNO 2
PROCNO 1

F2 - Acquisition Parameters
Date_ 20250426
Time 17.54
INSTRUM Avance
PROBHD Z166552_0018 (PI HR-
PULPROG zg30
TD 65536
SOLVENT CDCl3
NS 16
DS 2
SWH 7812.500
FIDRES 0.238419
AQ 4.1943040
RG 101
DW 64.000
DE 6.67
TE 298.0
D1 1.00000000
TD0 1
SFO1 399.5701703
NUC1 1H
P0 2.60
P1 7.80
PLW1 21.19799995

F2 - Processing parameters
SI 65536
SF 399.5677126
WDW EM
SSB 0
LB 0.30
GB 0
PC 1.00

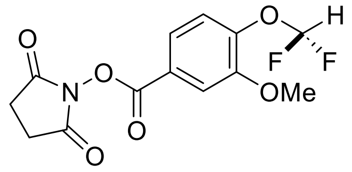

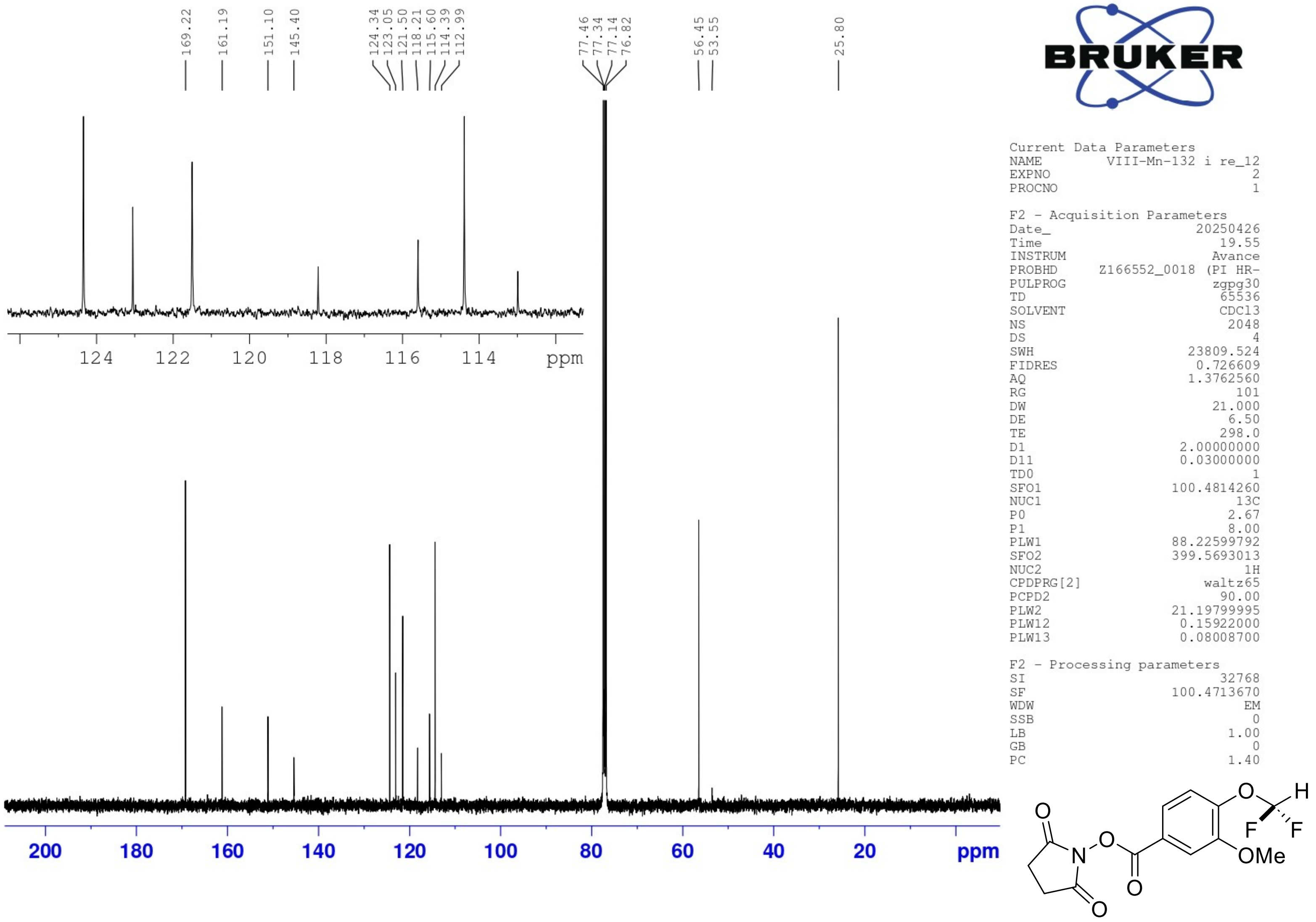
169.22
161.19
151.10
145.40
124.34
123.05
121.50
118.21
115.60
114.39
112.99
77.46
77.34
77.14
76.82
56.45
53.55
25.80
124 122 120 118 116 114 ppm
200 180 160 140 120 100 80 60 40 20 ppm
BRUKER
Current Data Parameters
NAME VIII-Mn-132 i re_12
EXPNO 2
PROCNO 1
F2 - Acquisition Parameters
Date_ 20250426
Time 19.55
INSTRUM Avance
PROBHD Z166552_0018 (PI HR-
PULPROG zgpg30
TD 65536
SOLVENT CDCl3
NS 2048
DS 4
SWH 23809.524
FIDRES 0.726609
AQ 1.3762560
RG 101
DW 21.000
DE 6.50
TE 298.0
D1 2.00000000
D11 0.03000000
TD0 1
SFO1 100.4814260
NUC1 13C
P0 2.67
P1 8.00
PLW1 88.22599792
SFO2 399.5693013
NUC2 1H
CPDPRG[2] waltz65
PCPD2 90.00
PLW2 21.19799995
PLW12 0.15922000
PLW13 0.08008700
F2 - Processing parameters
SI 32768
SF 100.4713670
WDW EM
SSB 0
LB 1.00
GB 0
PC 1.40
O
N
O
O
O
O
OMe
F
F
H

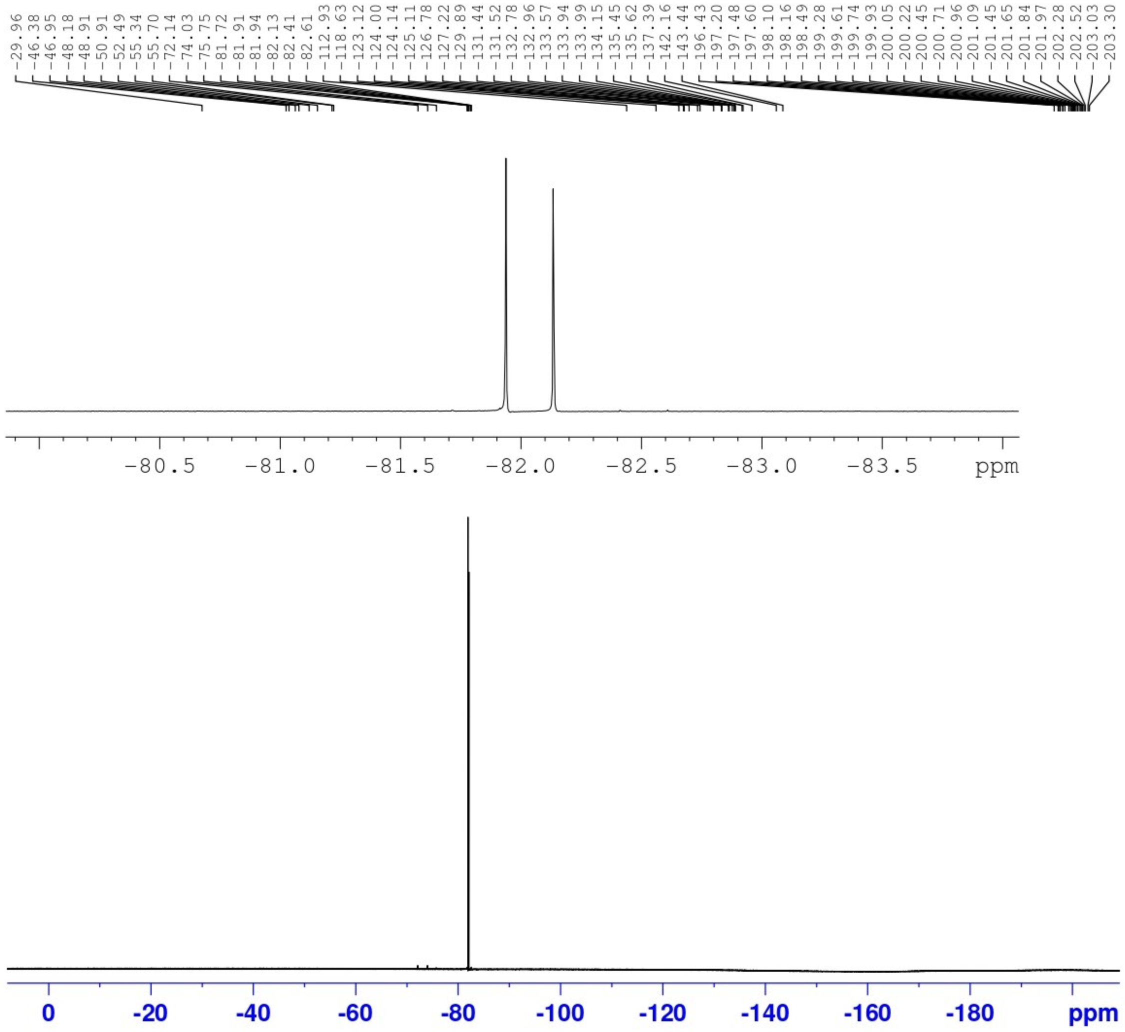


```
Current Data Parameters
NAME          VIII-Mn-132 i re_11
EXPNO                           2
PROCNO                          1

F2 - Acquisition Parameters
Date_                    20250426
Time                        17.56
INSTRUM                    Avance
PROBHD       Z166552_0018 (PI HR-
PULPROG                        zg
TD                         131072
SOLVENT                     CDCl3
NS                             16
DS                              4
SWH                     90909.091
FIDRES                   1.387163
AQ                      0.7208960
RG                            101
DW                          5.500
DE                           6.50
TE                          298.0
D1                     1.00000000
TD0                             1
SFO1                  375.9316815
NUC1                          19F
P1                          12.00
PLW1                  32.47200012

F2 - Processing parameters
SI                          65536
SF                    375.9692784
WDW                            EM
SSB                             0
LB                           0.30
GB                              0
PC                           1.00
```

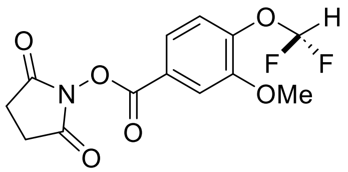

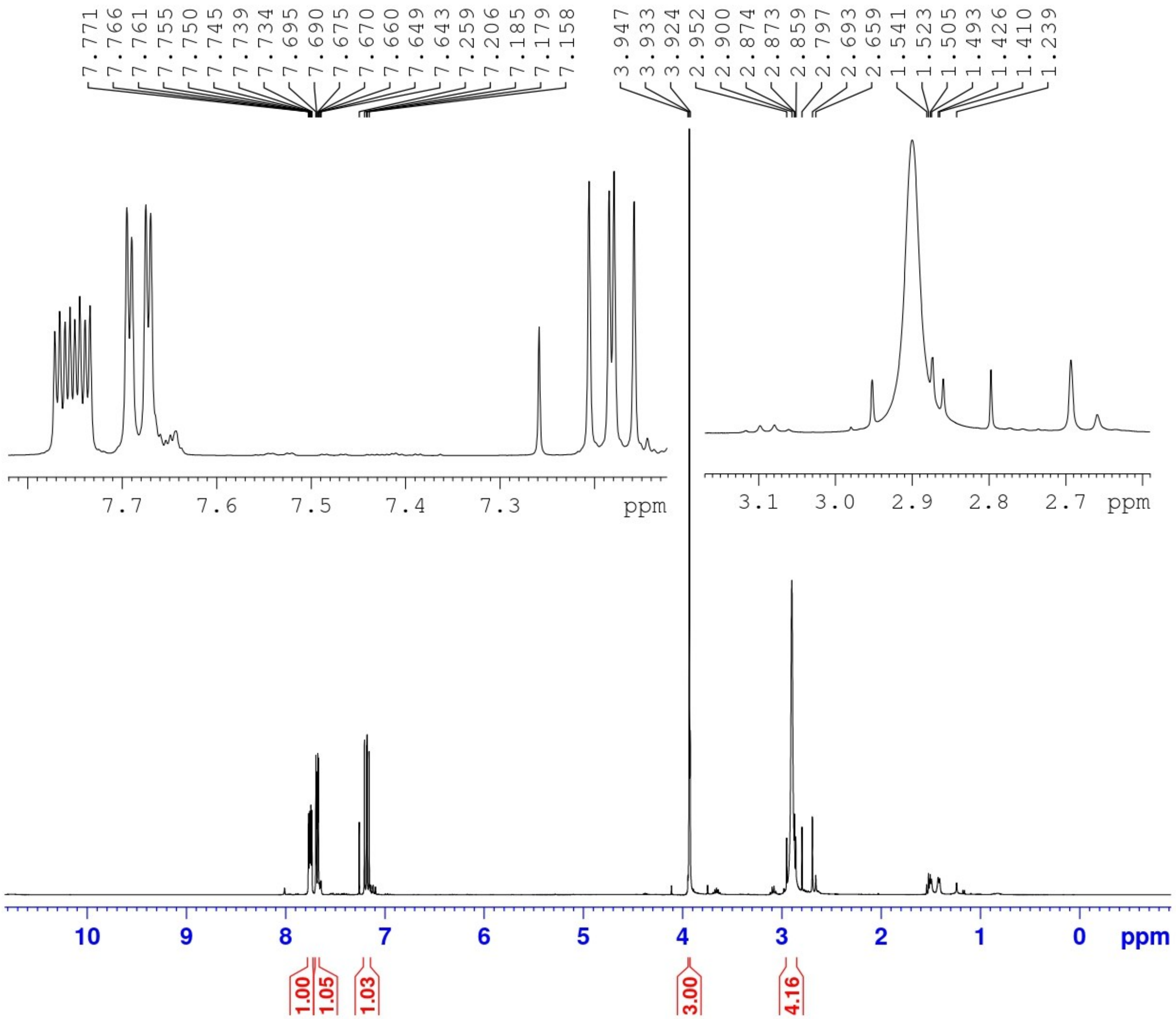

Current Data Parameters
NAME VIII-Mn-134 i_10
EXPNO 1
PROCNO 1

F2 - Acquisition Parameters
Date_ 20251007
Time 9.41
INSTRUM Avance
PROBHD Z166552_0018 (PI HR-
PULPROG zg30
TD 65536
SOLVENT CDCl3
NS 16
DS 2
SWH 7812.500
FIDRES 0.238419
AQ 4.1943040
RG 101
DW 64.000
DE 6.67
TE 298.0
D1 1.00000000
TD0 1
SFO1 399.5424672
NUC1 1H
P0 2.60
P1 7.80
PLW1 21.19799995

F2 - Processing parameters
SI 65536
SF 399.5400100
WDW EM
SSB 0
LB 0.30
GB 0
PC 1.00

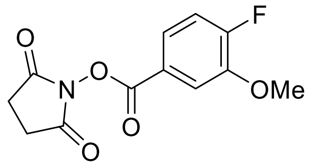

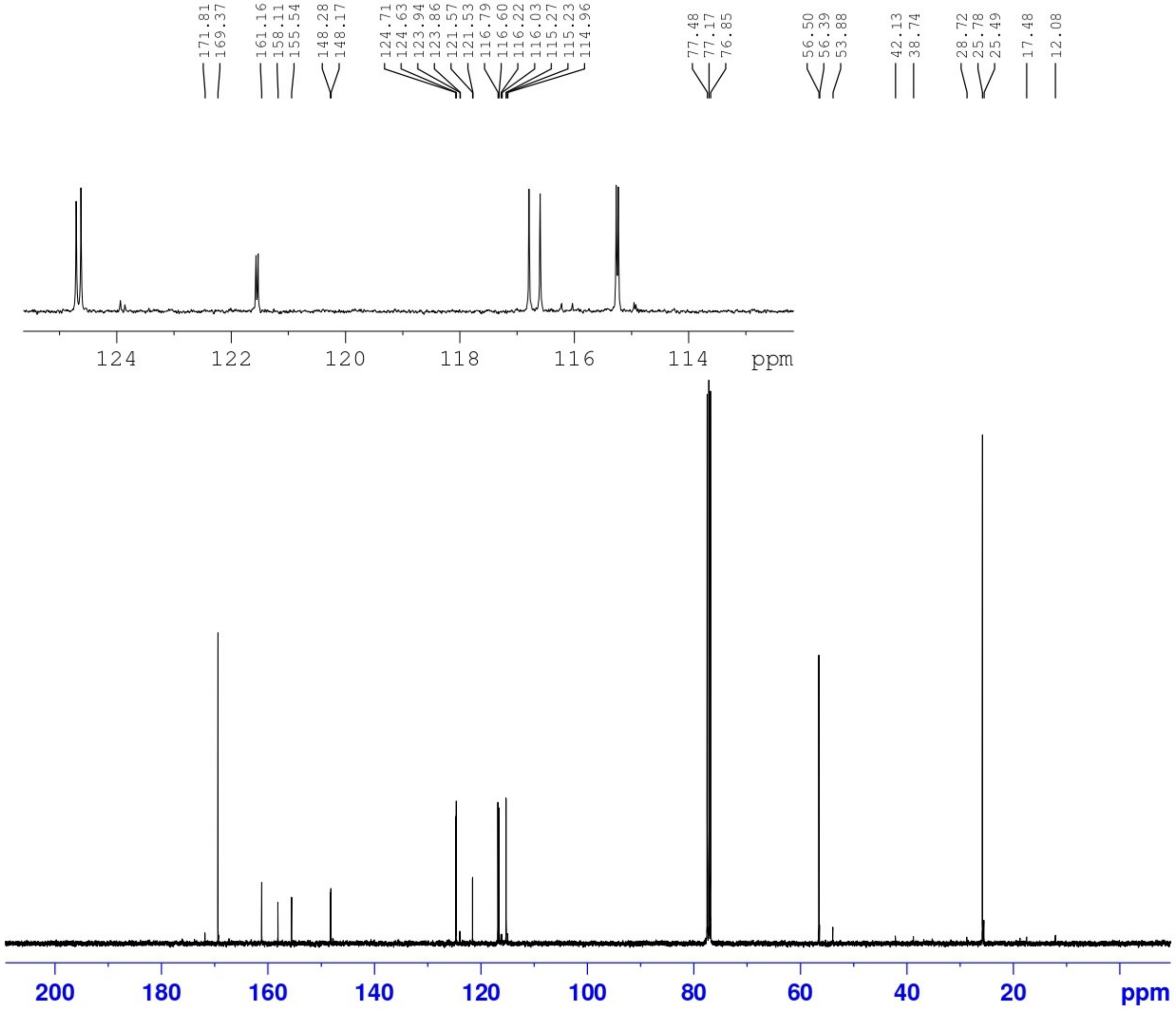

Current Data Parameters	
NAME	VIII-Mn-134 i_12
EXPNO	1
PROCNO	1
F2 - Acquisition Parameters	
Date_	20251007
Time	10.14
INSTRUM	Avance
PROBHD	Z166552_0018 (PI HR-
PULPROG	zgpg30
TD	65536
SOLVENT	CDCl3
NS	512
DS	4
SWH	23809.524
FIDRES	0.726609
AQ	1.3762560
RG	101
DW	21.000
DE	6.50
TE	298.0
D1	2.00000000
D11	0.03000000
TD0	1
SFO1	100.4744593
NUC1	13C
P0	2.67
P1	8.00
PLW1	88.22599792
SFO2	399.5415982
NUC2	1H
CPDPRG[2]	waltz65
PCPD2	90.00
PLW2	21.19799995
PLW12	0.15922000
PLW13	0.08008700
F2 - Processing parameters	
SI	32768
SF	100.4644025
WDW	EM
SSB	0
LB	1.00
GB	0
PC	1.40

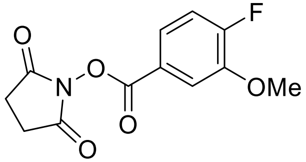

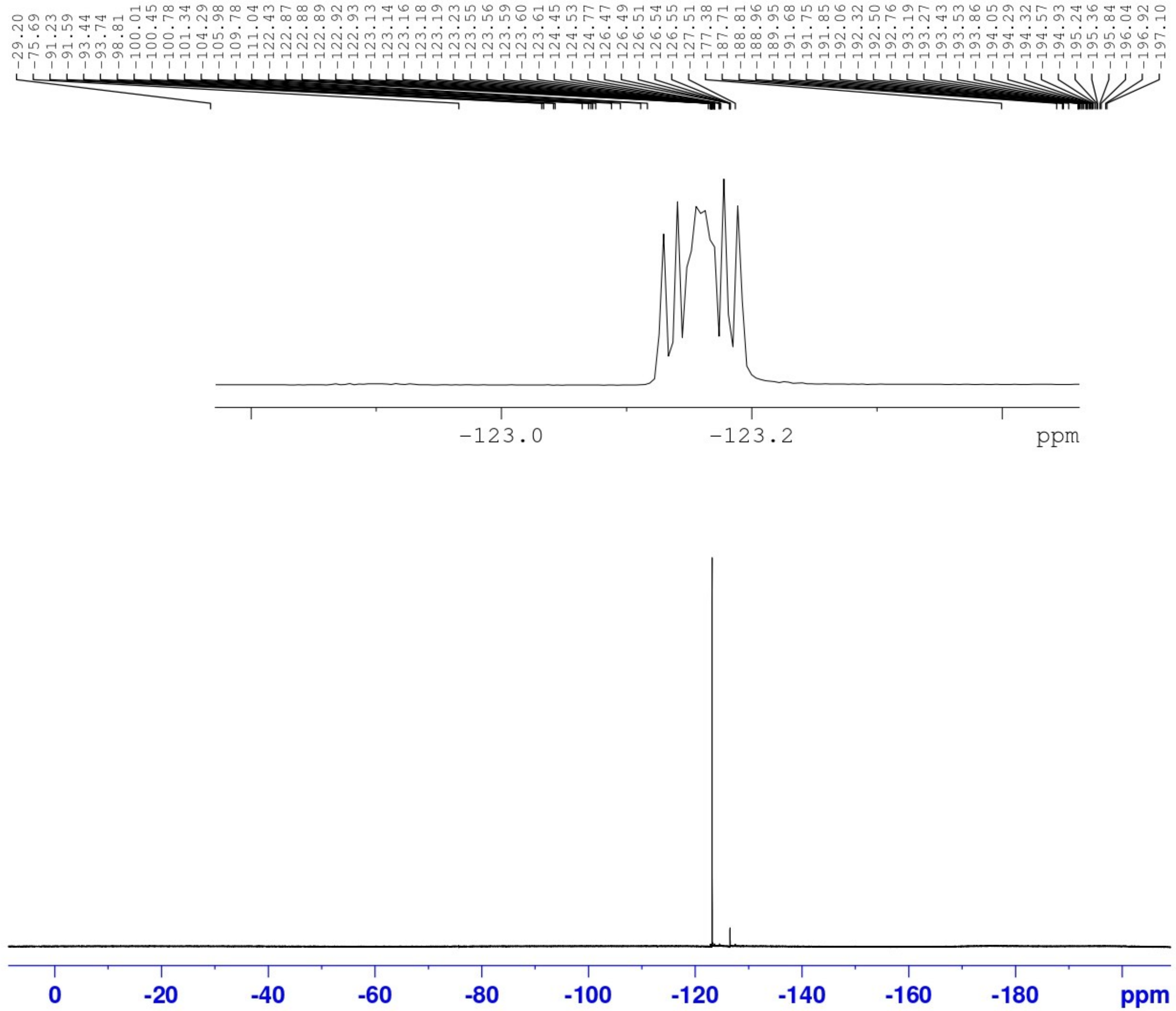

BRUKER

Current Data Parameters
NAME VIII-Mn-134 i_11
EXPNO 1
PROCNO 1

F2 - Acquisition Parameters
Date_ 20251007
Time 9.43
INSTRUM Avance
PROBHD Z166552_0018 (PI HR-
PULPROG zg
TD 131072
SOLVENT CDCl3
NS 16
DS 4
SWH 90909.091
FIDRES 1.387163
AQ 0.7208960
RG 101
DW 5.500
DE 6.50
TE 298.0
D1 1.00000000
TD0 1
SFO1 375.9056172
NUC1 19F
P1 12.00
PLW1 32.47200012

F2 - Processing parameters
SI 65536
SF 375.9432115
WDW EM
SSB 0
LB 0.30
GB 0
PC 1.00

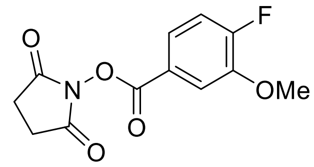

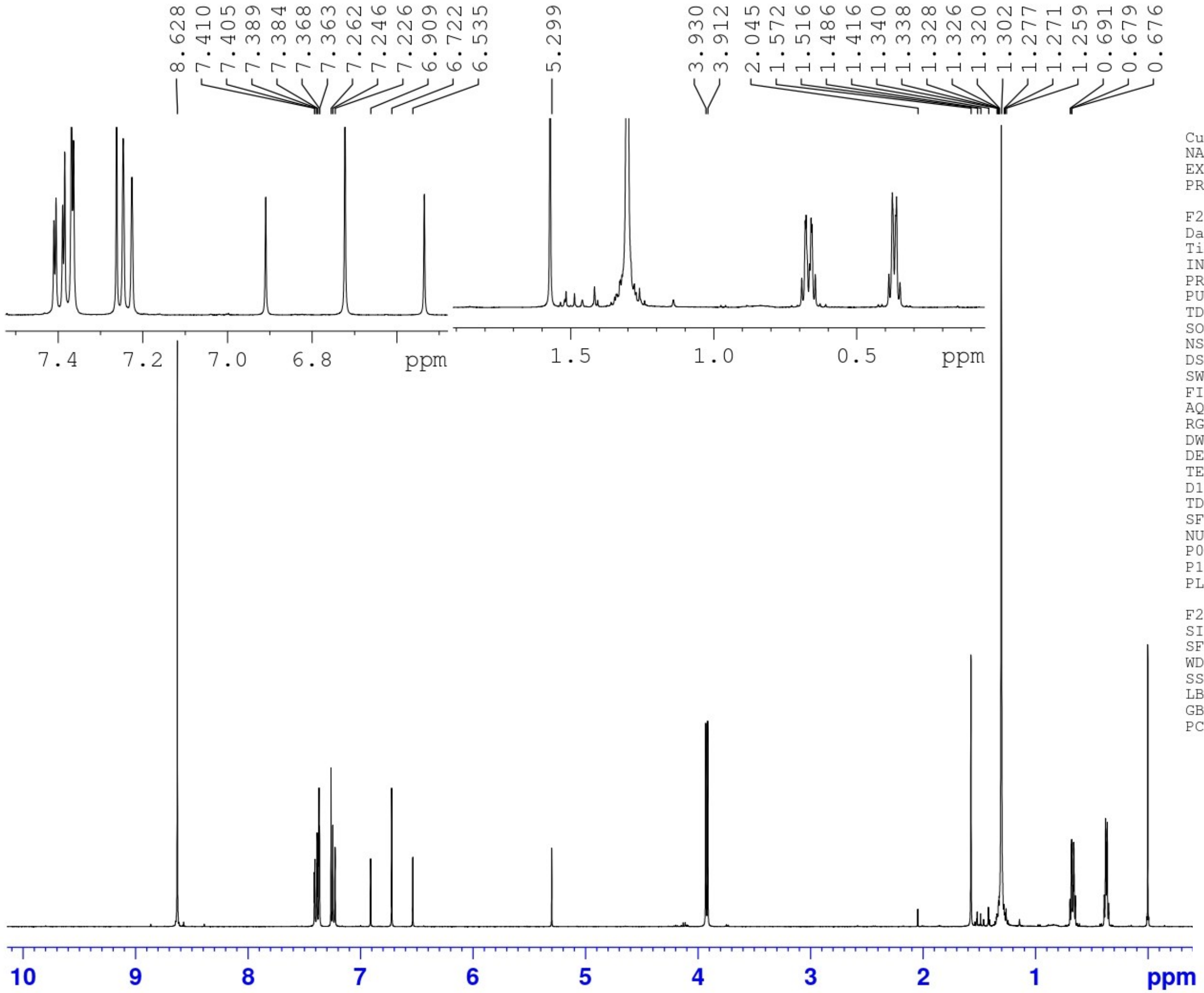

Current Data Parameters	
NAME	V-Mn-44 i_20
EXPNO	2
PROCNO	1
F2 - Acquisition Parameters	
Date_	20240530
Time	12.25
INSTRUM	Avance
PROBHD	Z166552_0018 (PI HR-
PULPROG	zg30
TD	65536
SOLVENT	CDCl3
NS	16
DS	2
SWH	7812.500
FIDRES	0.238419
AQ	4.1943040
RG	101
DW	64.000
DE	6.67
TE	298.0
D1	1.00000000
TD0	1
SFO1	399.6024675
NUC1	1H
P0	2.60
P1	7.80
PLW1	21.19799995
F2 - Processing parameters	
SI	65536
SF	399.6000088
WDW	EM
SSB	0
LB	0.30
GB	0
PC	1.00

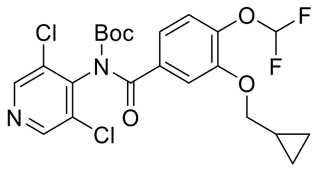

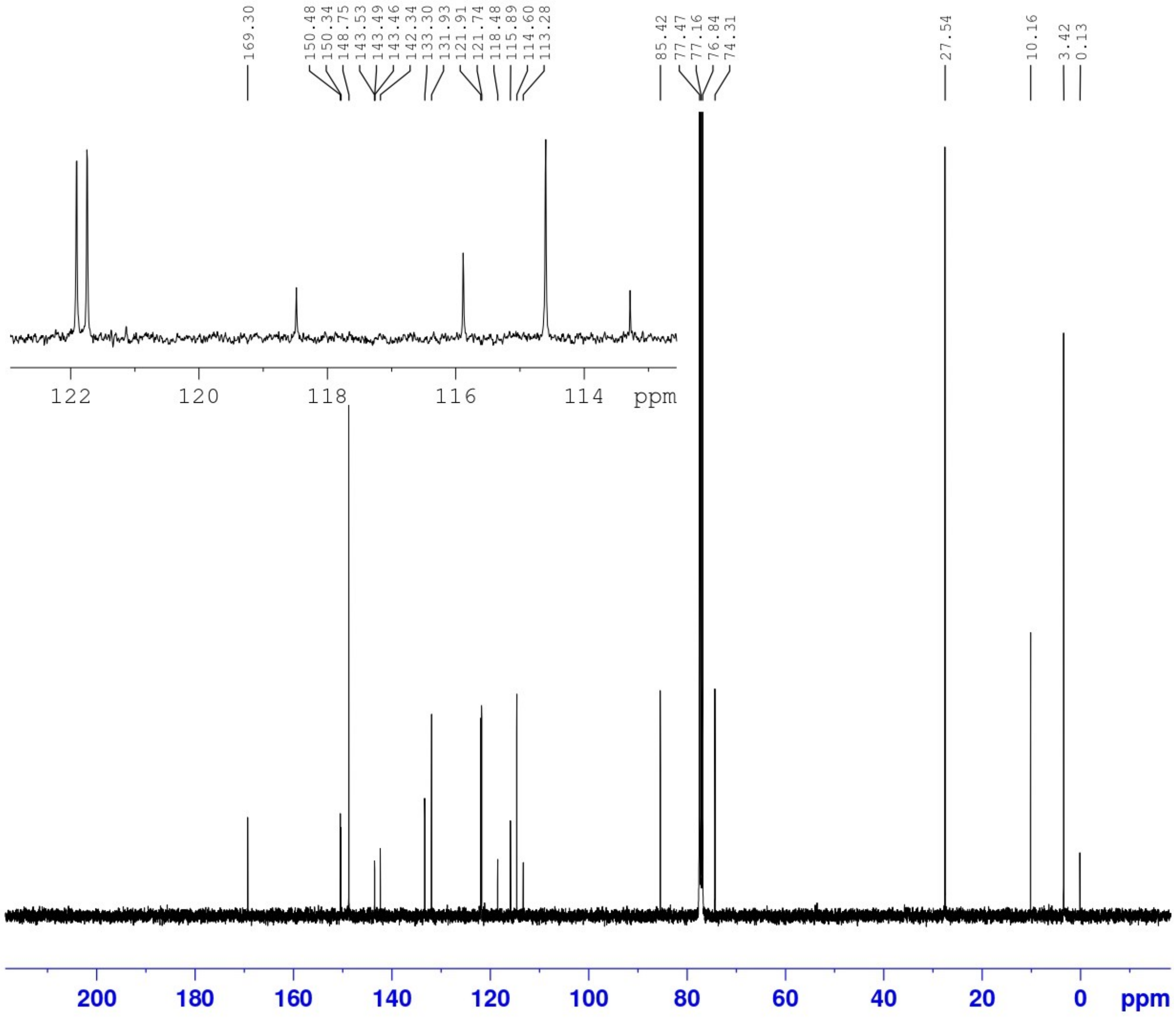

Current Data Parameters
NAME V-Mn-44 i_22
EXPNO 1
PROCNO 1

F2 - Acquisition Parameters
Date_ 20240530
Time 20.09
INSTRUM Avance
PROBHD Z166552_0018 (PI HR-
PULPROG zgpg30
TD 65536
SOLVENT CDCl3
NS 2048
DS 4
SWH 23809.524
FIDRES 0.726609
AQ 1.3762560
RG 101
DW 21.000
DE 6.50
TE 298.0
D1 2.00000000
D11 0.03000000
TD0 1
SFO1 100.4895479
NUC1 13C
P0 2.67
P1 8.00
PLW1 88.22599792
SFO2 399.6015984
NUC2 1H
CPDPRG[2] waltz65
PCPD2 90.00
PLW2 21.19799995
PLW12 0.15922000
PLW13 0.08008700

F2 - Processing parameters
SI 32768
SF 100.4794865
WDW EM
SSB 0
LB 1.00
GB 0
PC 1.40

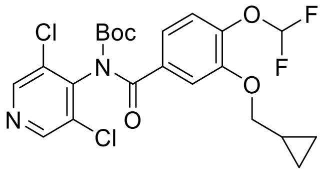

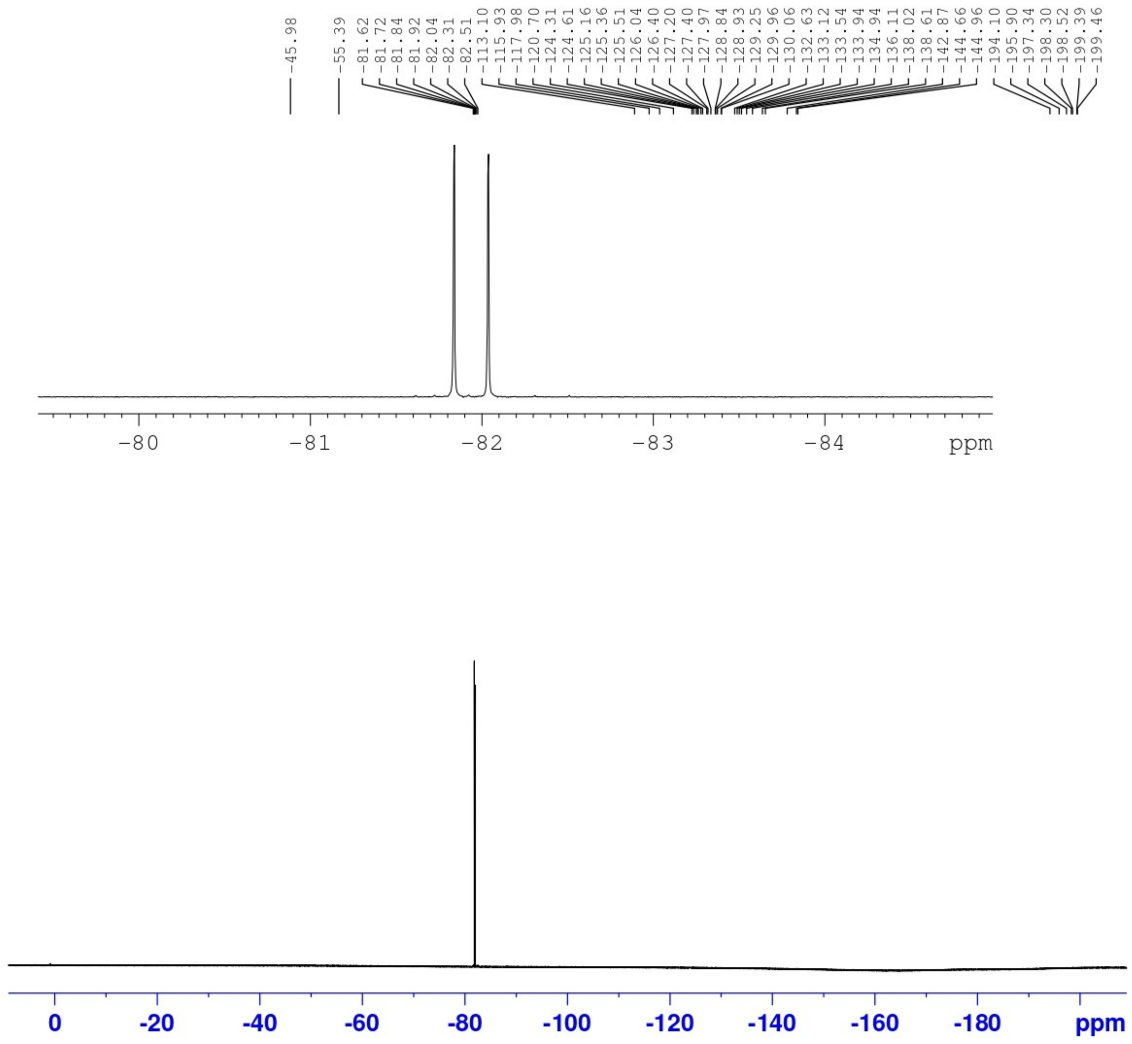

BRUKER

Current Data Parameters
NAME V-Mn-44 i_21
EXPNO 2
PROCNO 1

F2 - Acquisition Parameters
Date_ 20240530
Time 12.28
INSTRUM Avance
PROBHD Z166552_0018 (PI HR-
PULPROG zg
TD 131072
SOLVENT CDCl3
NS 16
DS 4
SWH 90909.091
FIDRES 1.387163
AQ 0.7208960
RG 101
DW 5.500
DE 6.50
TE 298.0
D1 1.00000000
TD0 1
SFO1 375.9620680
NUC1 19F
P1 12.00
PLW1 32.47200012

F2 - Processing parameters
SI 65536
SF 375.9996680
WDW EM
SSB 0
LB 0.30
GB 0
PC 1.00

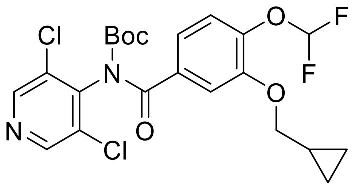

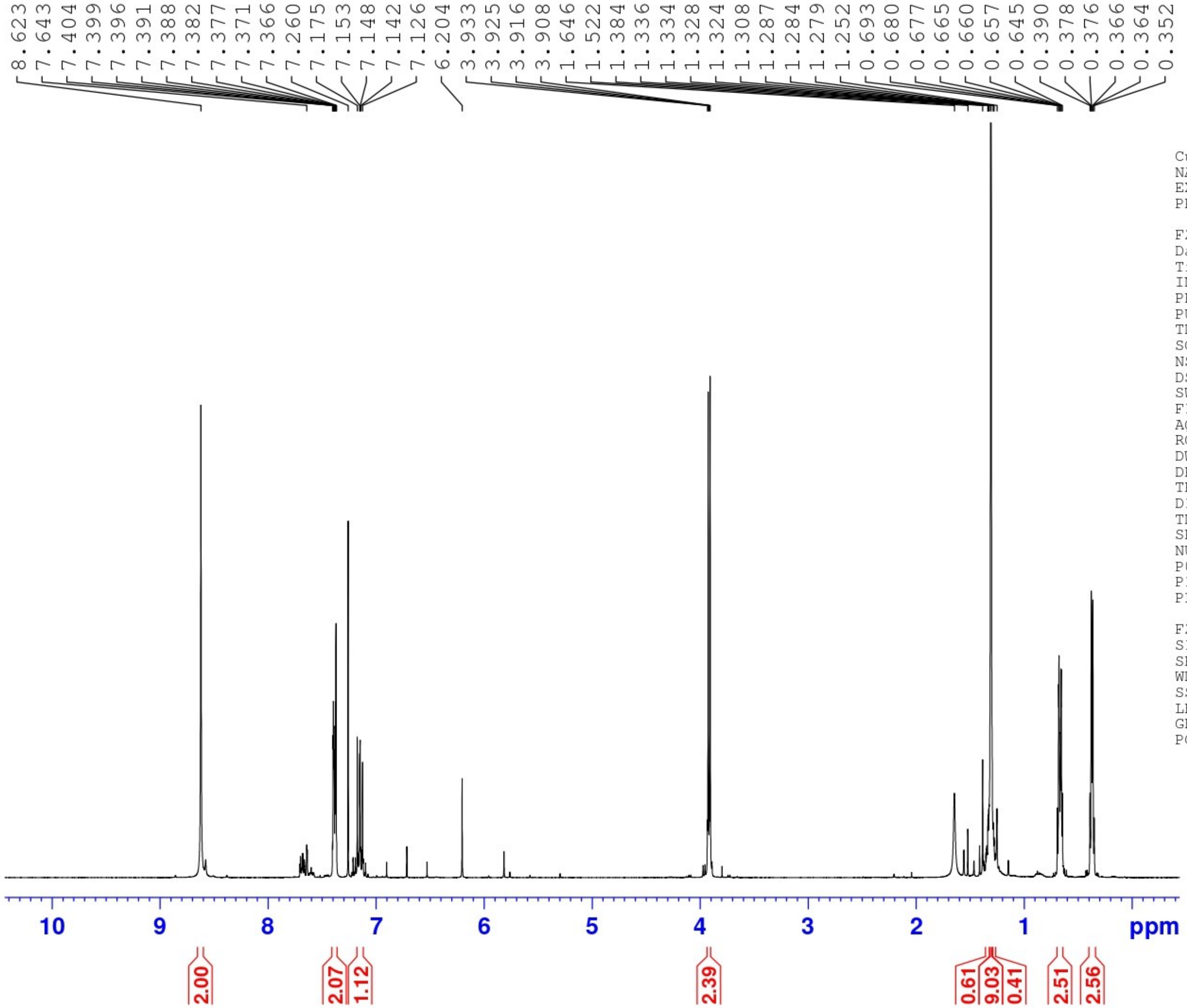

BRUKER

Current Data Parameters

NAME	IX-Mn-49 i Re_10
EXPNO	3
PROCNO	1

F2 - Acquisition Parameters

Date_	20250917
Time	17.30
INSTRUM	Avance
PROBHD	Z166552_0018 (PI HR-
PULPROG	zg30
TD	65536
SOLVENT	CDCl3
NS	64
DS	2
SWH	7812.500
FIDRES	0.238419
AQ	4.1943040
RG	101
DW	64.000
DE	6.67
TE	298.0
D1	1.00000000
TD0	1
SFO1	399.5424672
NUC1	1H
P0	2.60
P1	7.80
PLW1	21.19799995

F2 - Processing parameters

SI	65536
SF	399.5400096
WDW	EM
SSB	0
LB	0.30
GB	0
PC	1.00

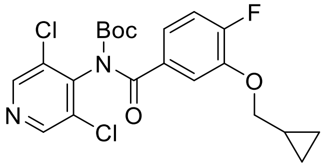

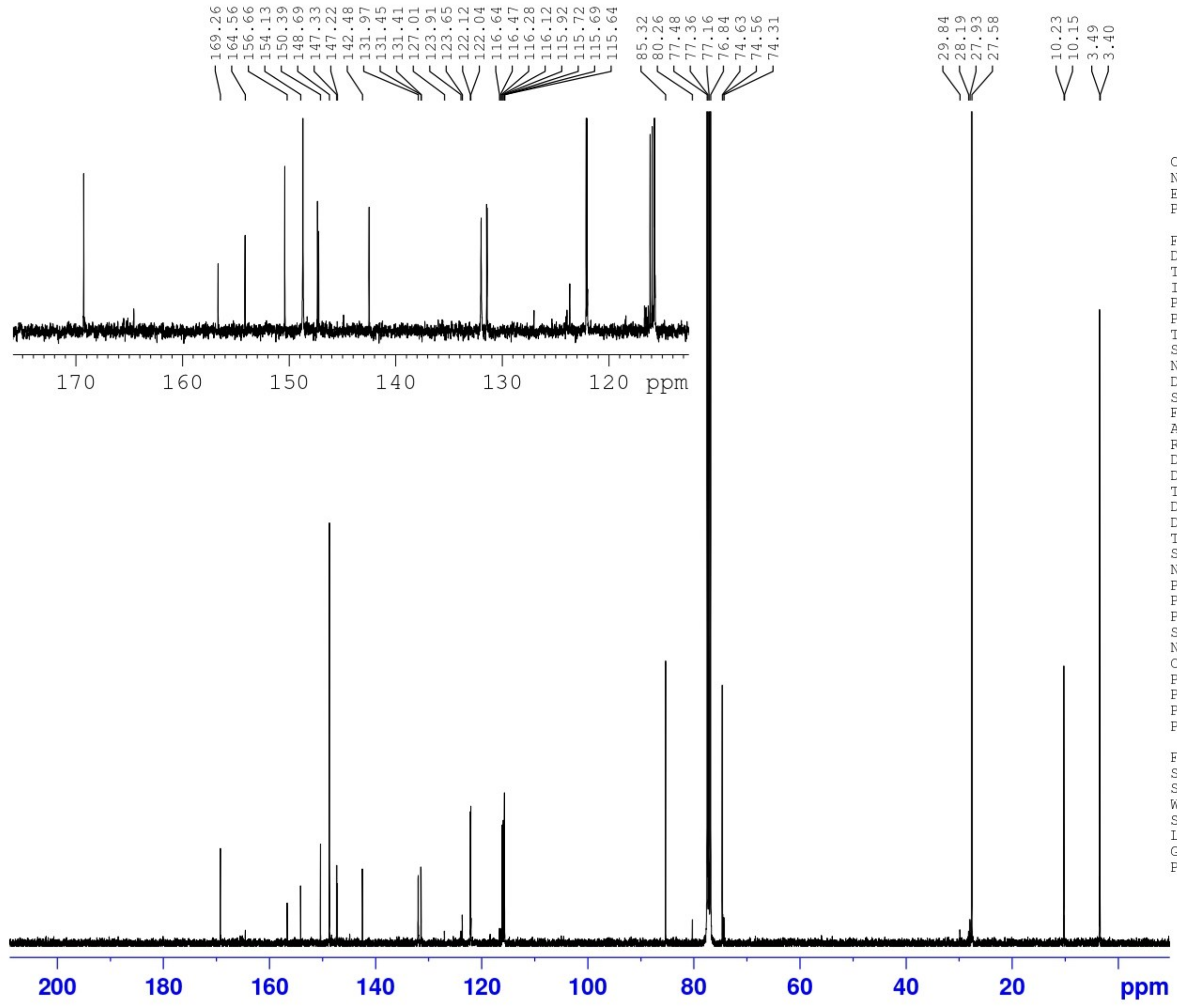

Current Data Parameters
NAME IX-Mn-49 i Re_12
EXPNO 2
PROCNO 1

F2 - Acquisition Parameters
Date_ 20250917
Time 21.59
INSTRUM Avance
PROBHD Z166552_0018 (PI HR-
PULPROG zgpg30
TD 65536
SOLVENT CDCl3
NS 4096
DS 4
SWH 23809.524
FIDRES 0.726609
AQ 1.3762560
RG 101
DW 21.000
DE 6.50
TE 298.0
D1 2.00000000
D11 0.03000000
TD0 1
SFO1 100.4744593
NUC1 13C
P0 2.67
P1 8.00
PLW1 88.22599792
SFO2 399.5415982
NUC2 1H
CPDPRG[2] waltz65
PCPD2 90.00
PLW2 21.19799995
PLW12 0.15922000
PLW13 0.08008700

F2 - Processing parameters
SI 32768
SF 100.4643995
WDW EM
SSB 0
LB 1.00
G [illegible]
P [illegible]

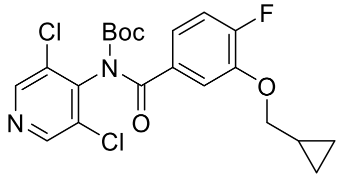

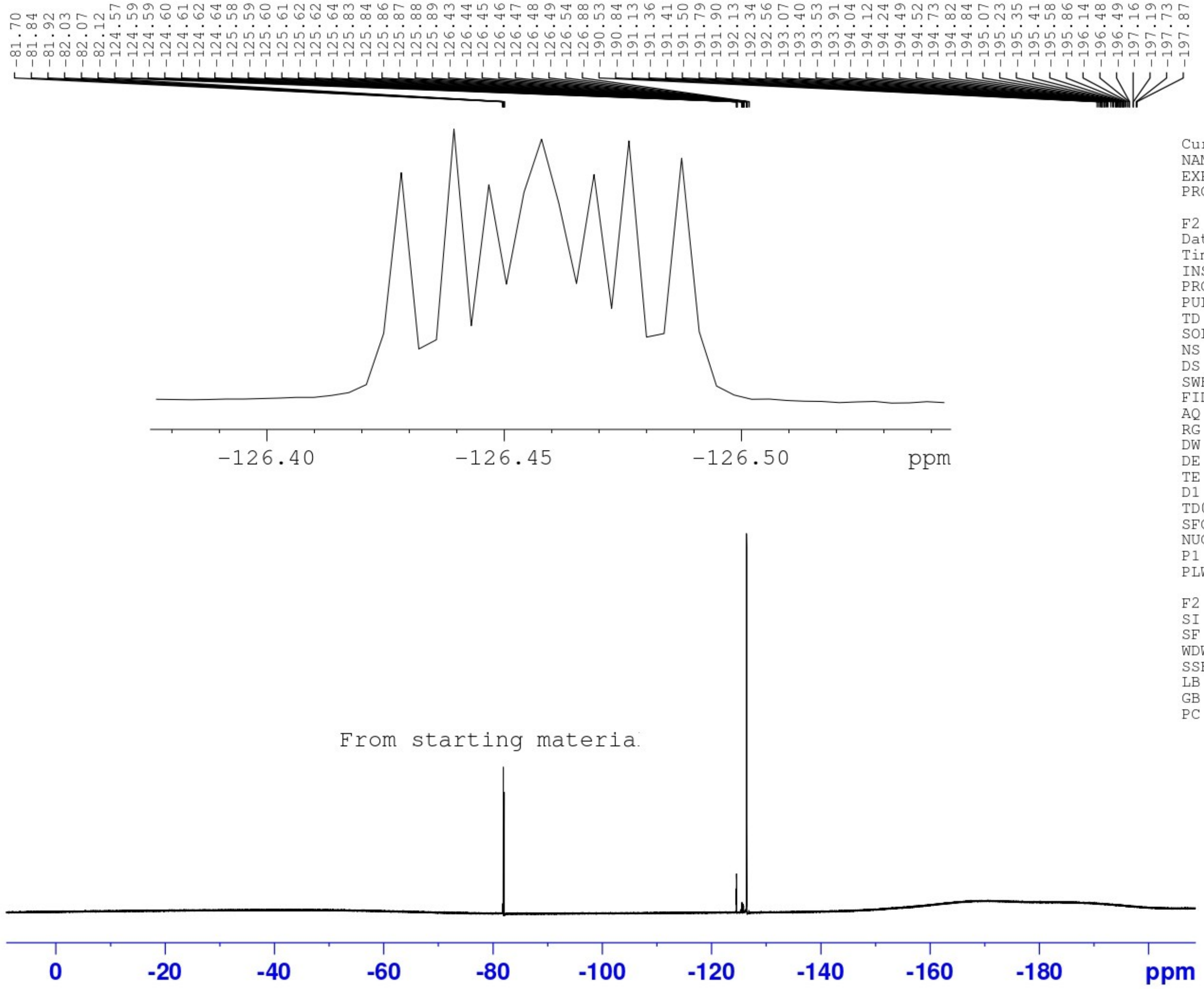

Current Data Parameters

NAME	IX-Mn-49 i Re_11
EXPNO	2
PROCNO	1

F2 - Acquisition Parameters

Date_	20250917
Time	17.33
INSTRUM	Avance
PROBHD	Z166552_0018 (PI HR-
PULPROG	zg
TD	131072
SOLVENT	CDCl3
NS	64
DS	4
SWH	90909.091
FIDRES	1.387163
AQ	0.7208960
RG	101
DW	5.500
DE	6.50
TE	298.0
D1	1.00000000
TD0	1
SFO1	375.9056172
NUC1	19F
P1	12.00
PLW1	32.47200012

F2 - Processing parameters

SI	65536
SF	375.9432115
WDW	EM
SSB	0
LB	0.30
GB	0
PC	1.00

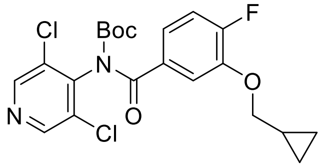

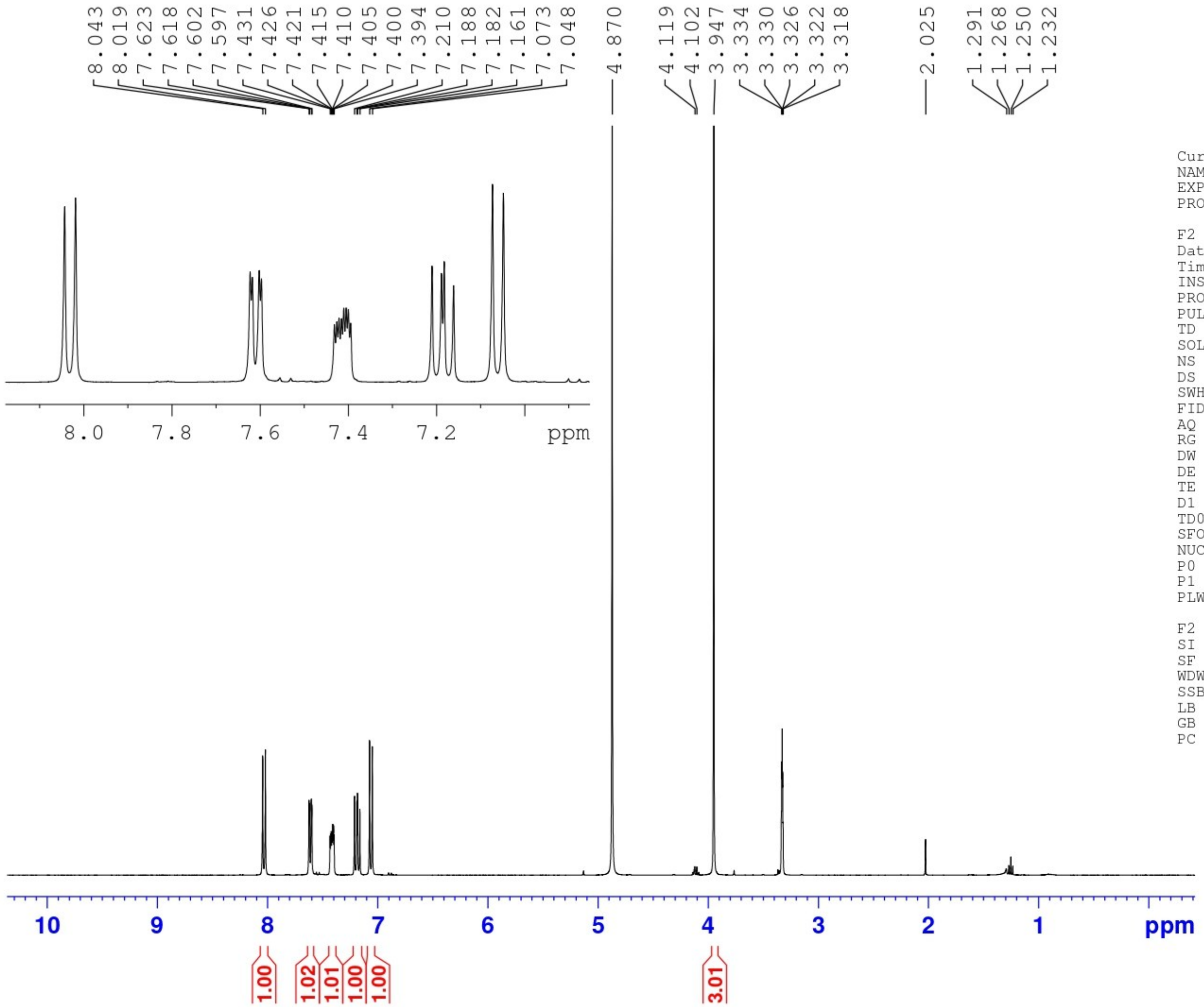

8.043
8.019
7.623
7.618
7.602
7.597
7.431
7.426
7.421
7.415
7.410
7.405
7.400
7.394
7.210
7.188
7.182
7.161
7.073
7.048
4.870
4.119
4.102
3.947
3.334
3.330
3.326
3.322
3.318
2.025
1.291
1.268
1.250
1.232
8.0
7.8
7.6
7.4
7.2
ppm
10
9
8
7
6
5
4
3
2
1
ppm
1.00
1.02
1.01
1.00
1.00
3.01

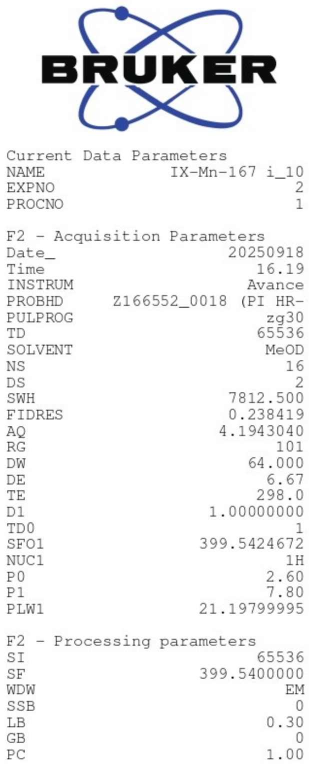

BRUKER
Current Data Parameters
NAME IX-Mn-167 i_10
EXPNO 2
PROCNO 1
F2 - Acquisition Parameters
Date_ 20250918
Time 16.19
INSTRUM Avance
PROBHD Z166552_0018 (PI HR-
PULPROG zg30
TD 65536
SOLVENT MeOD
NS 16
DS 2
SWH 7812.500
FIDRES 0.238419
AQ 4.1943040
RG 101
DW 64.000
DE 6.67
TE 298.0
D1 1.00000000
TD0 1
SFO1 399.5424672
NUC1 1H
P0 2.60
P1 7.80
PLW1 21.19799995
F2 - Processing parameters
SI 65536
SF 399.5400000
WDW EM
SSB 0
LB 0.30
GB 0
PC 1.00

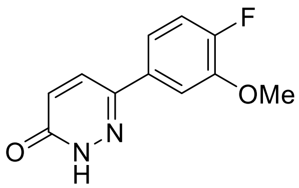

F
OMe
O
N
N
H

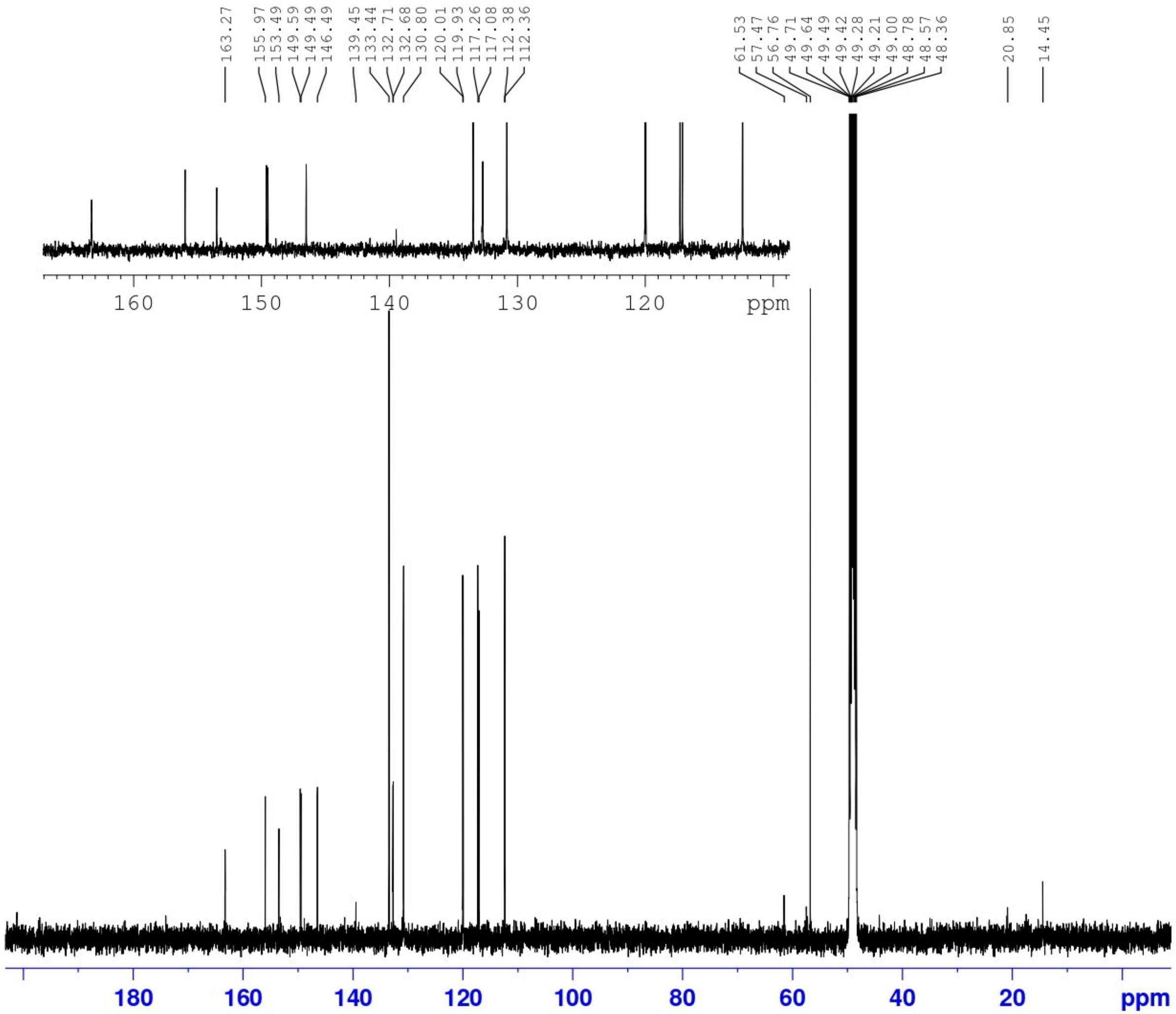

Current Data Parameters
NAME IX-Mn-167 i_12
EXPNO 2
PROCNO 1

F2 - Acquisition Parameters
Date_ 20250919
Time 0.24
INSTRUM Avance
PROBHD Z166552_0018 (PI HR-
PULPROG zgpg30
TD 65536
SOLVENT MeOD
NS 4096
DS 4
SWH 23809.524
FIDRES 0.726609
AQ 1.3762560
RG 101
DW 21.000
DE 6.50
TE 298.0
D1 2.00000000
D11 0.03000000
TD0 1
SFO1 100.4744593
NUC1 13C
P0 2.67
P1 8.00
PLW1 88.22599792
SFO2 399.5415982
NUC2 1H
CPDPRG[2] waltz65
PCPD2 90.00
PLW2 21.19799995
PLW12 0.15922000
PLW13 0.08008700

F2 - Processing parameters
SI 32768
SF 100.4642722
WDW EM
SSB 0
LB 1.00
GB 0
PC 1.40

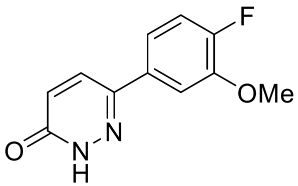

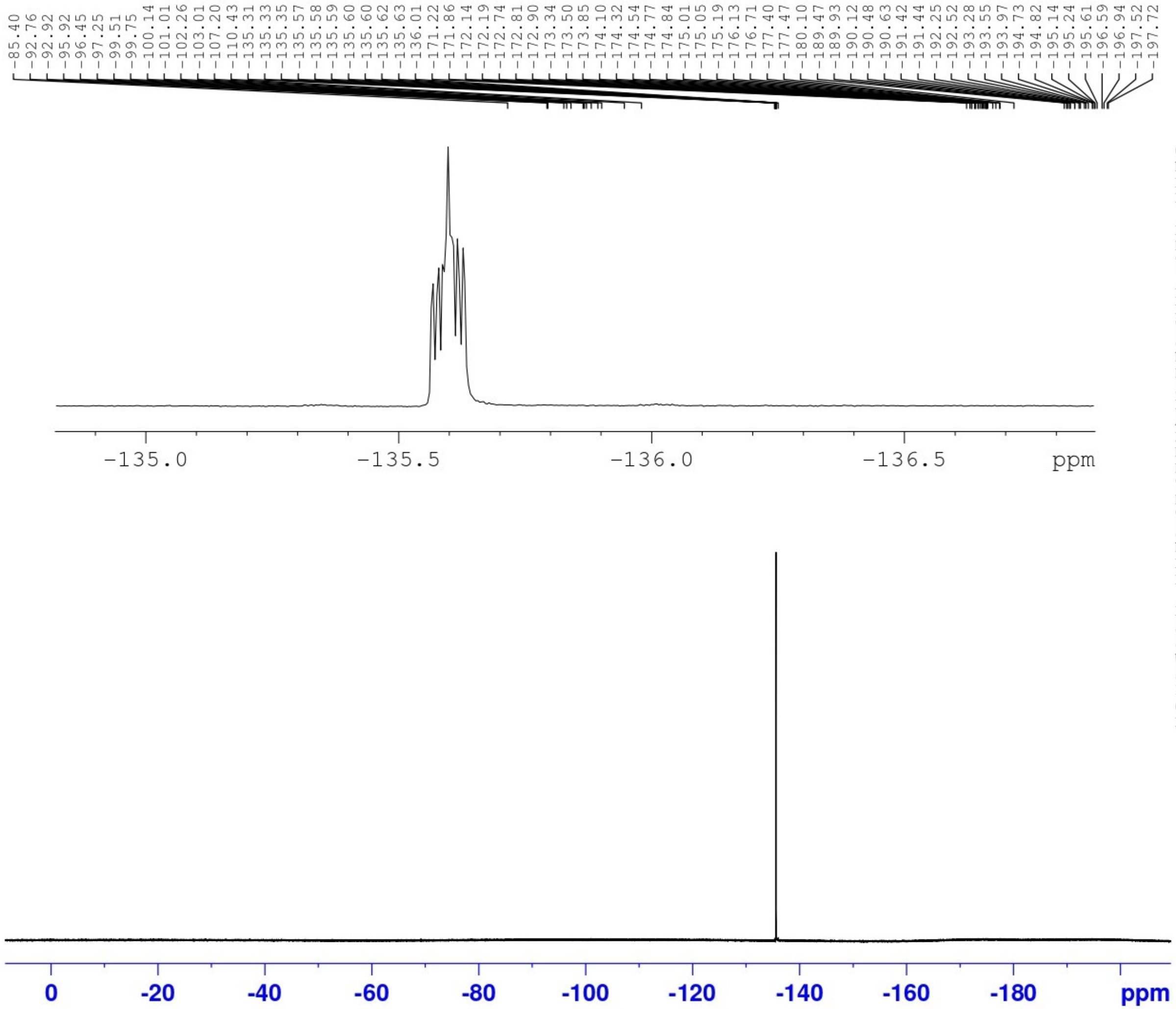

BRUKER

Current Data Parameters

NAME	IX-Mn-167 i_11
EXPNO	2
PROCNO	1

F2 - Acquisition Parameters

Date_	20250918
Time	16.20
INSTRUM	Avance
PROBHD	Z166552_0018 (PI HR-
PULPROG	zg
TD	131072
SOLVENT	MeOD
NS	16
DS	4
SWH	90909.091
FIDRES	1.387163
AQ	0.7208960
RG	101
DW	5.500
DE	6.50
TE	298.0
D1	1.00000000
TD0	1
SFO1	375.9056172
NUC1	19F
P1	12.00
PLW1	32.47200012

F2 - Processing parameters

SI	65536
SF	375.9432115
WDW	EM
SSB	0
LB	0.30
GB	0
PC	1.00

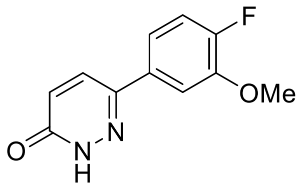

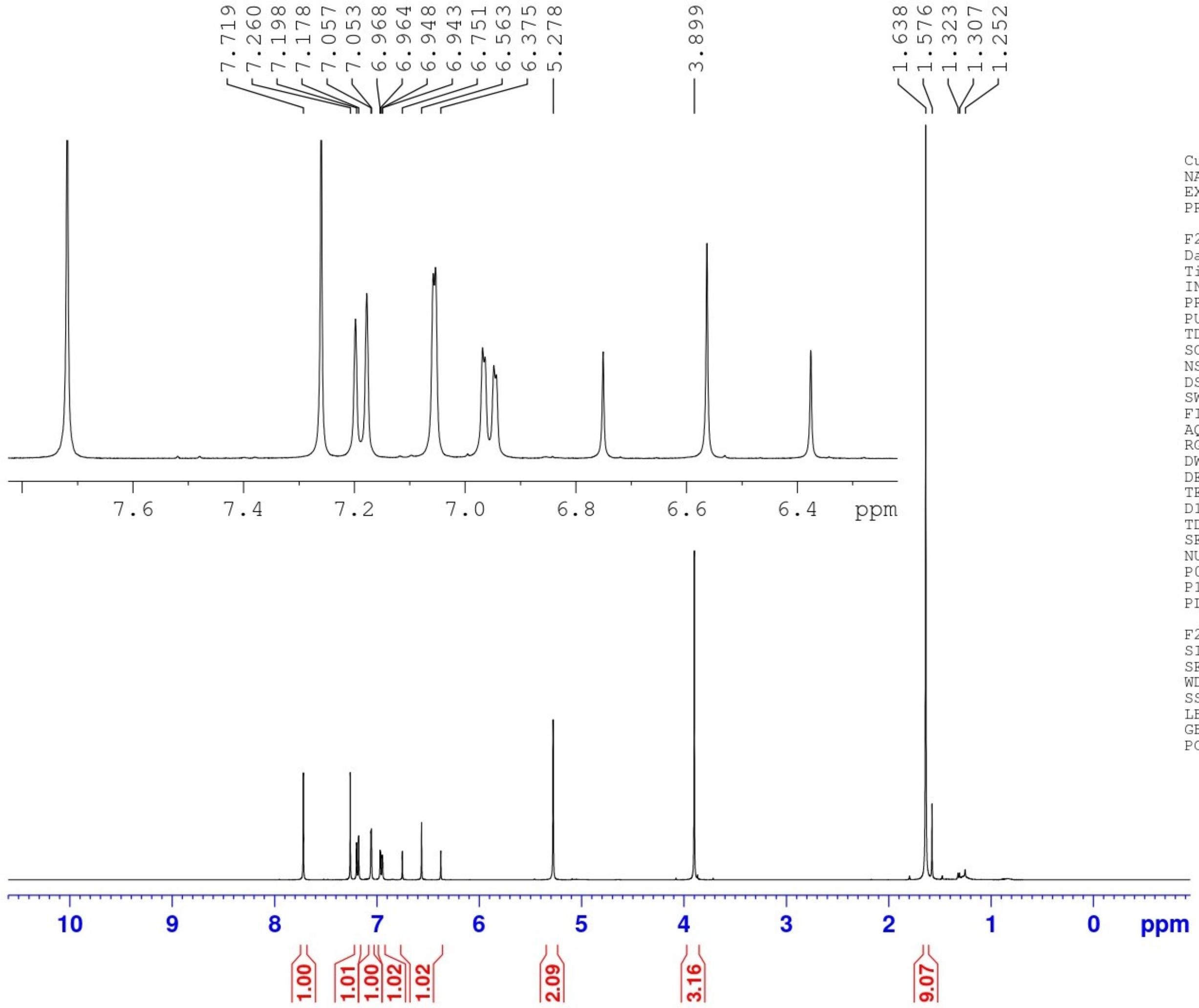

Current Data Parameters
NAME VI-Mn-98 iiRe_10
EXPNO 2
PROCNO 1

F2 - Acquisition Parameters
Date_ 20241011
Time 16.16
INSTRUM Avance
PROBHD Z166552_0018 (PI HR-
PULPROG zg30
TD 65536
SOLVENT CDCl3
NS 16
DS 2
SWH 7812.500
FIDRES 0.238419
AQ 4.1943040
RG 101
DW 64.000
DE 6.67
TE 298.0
D1 1.00000000
TD0 1
SFO1 399.5701703
NUC1 1H
P0 2.60
P1 7.80
PLW1 21.19799995

F2 - Processing parameters
SI 65536
SF 399.5677126
WDW EM
SSB 0
LB 0.30
GB 0
PC 1.00

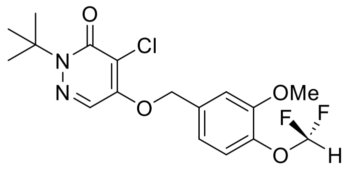

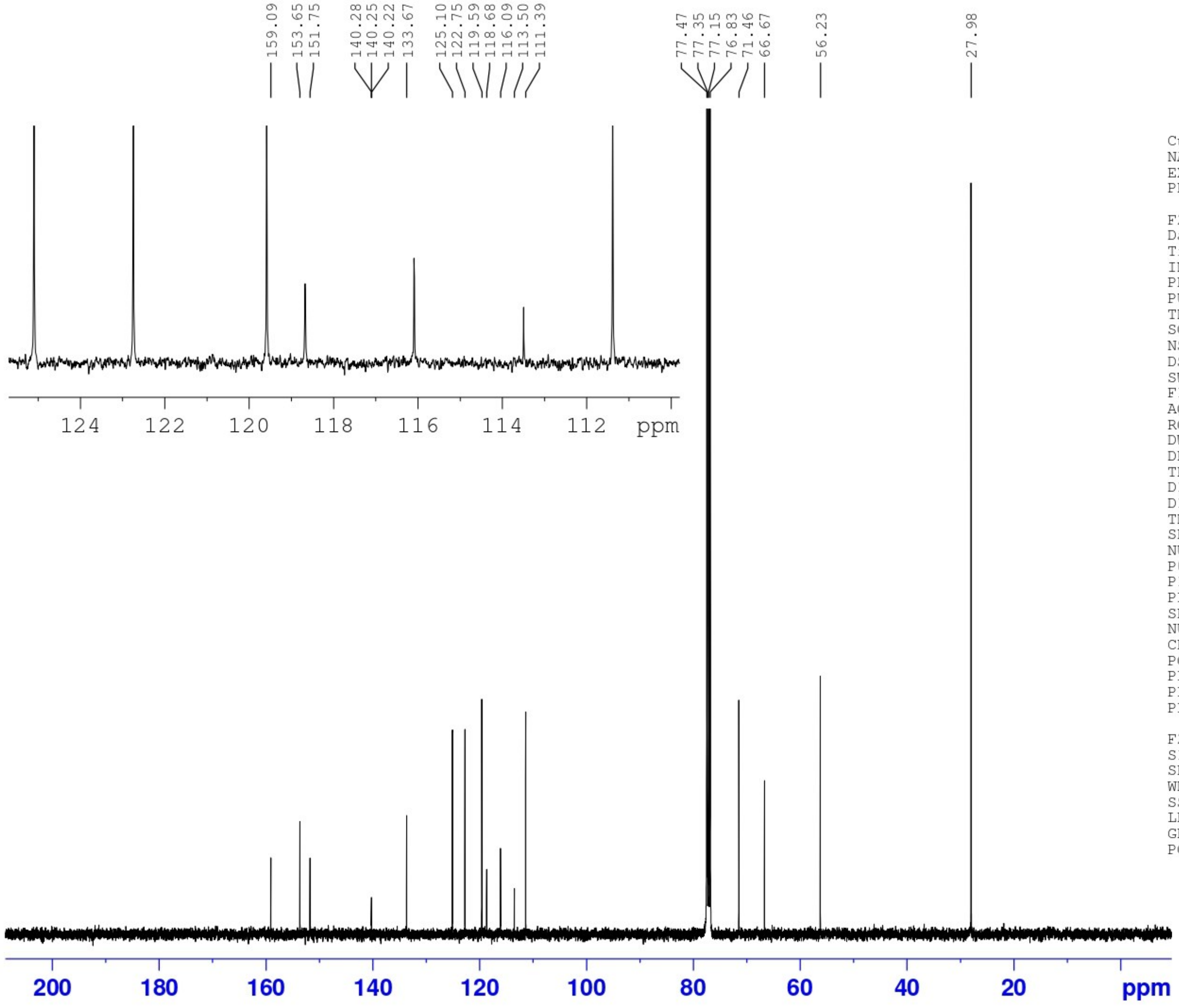

Current Data Parameters
NAME VI-Mn-98 iiRe_12
EXPNO 2
PROCNO 1

F2 - Acquisition Parameters
Date_ 20241011
Time 20.02
INSTRUM Avance
PROBHD Z166552_0018 (PI HR-
PULPROG zgpg30
TD 65536
SOLVENT CDCl3
NS 2048
DS 4
SWH 23809.524
FIDRES 0.726609
AQ 1.3762560
RG 101
DW 21.000
DE 6.50
TE 298.0
D1 2.00000000
D11 0.03000000
TD0 1
SFO1 100.4814260
NUC1 13C
P0 2.67
P1 8.00
PLW1 88.22599792
SFO2 399.5693013
NUC2 1H
CPDPRG[2] waltz65
PCPD2 90.00
PLW2 21.19799995
PLW12 0.15922000
PLW13 0.08008700

F2 - Processing parameters
SI 32768
SF 100.4713660
WDW EM
SSB 0
LB 1.00
GB 0
PC 1.40

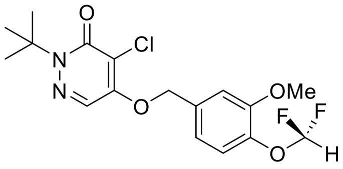

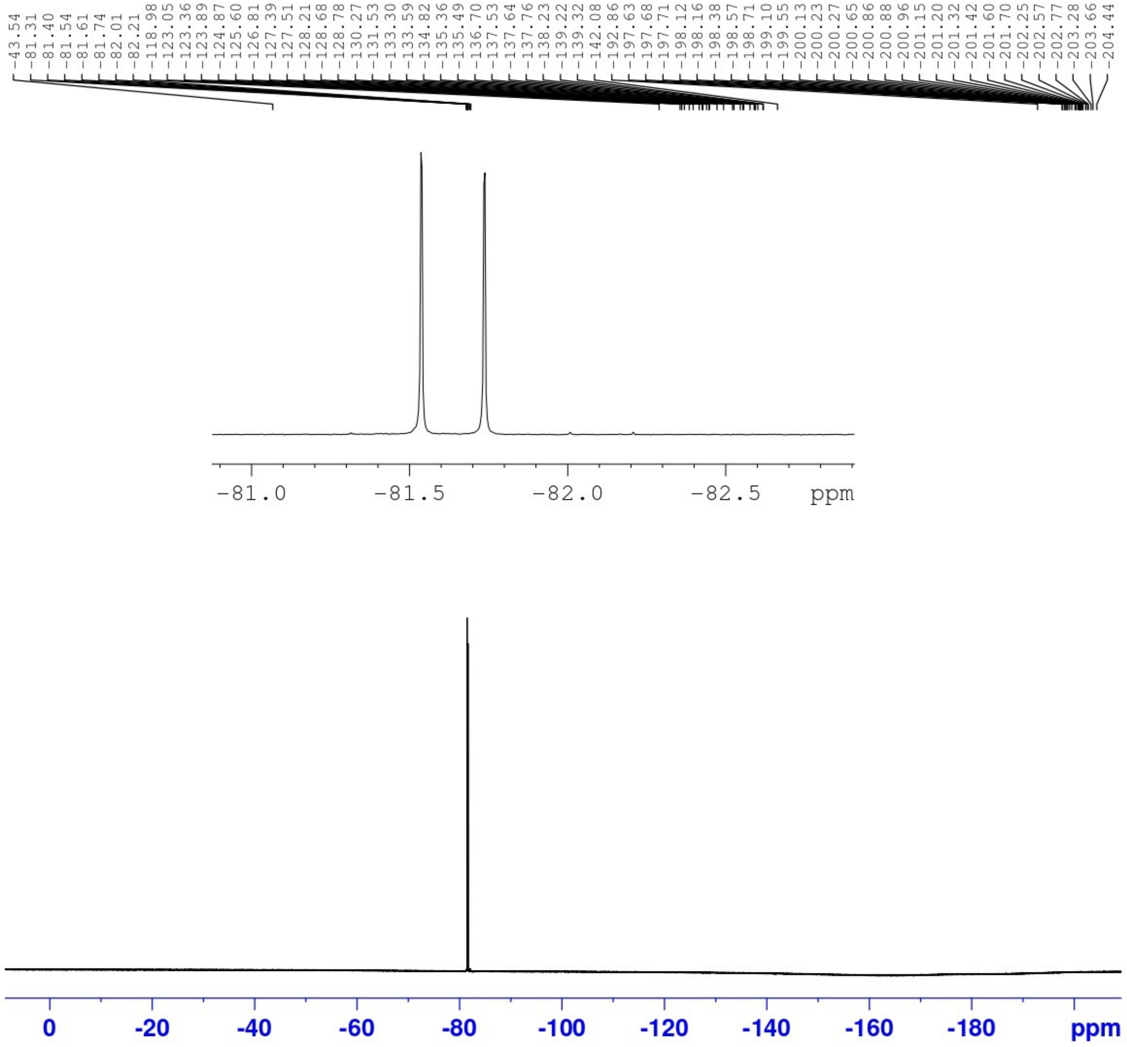

BRUKER

Current Data Parameters

NAME	VI-Mn-98 iiRe_11
EXPNO	2
PROCNO	1

F2 - Acquisition Parameters

Date_	20241011
Time	16.18
INSTRUM	Avance
PROBHD	Z166552_0018 (PI HR-
PULPROG	zg
TD	131072
SOLVENT	CDCl3
NS	16
DS	4
SWH	90909.091
FIDRES	1.387163
AQ	0.7208960
RG	101
DW	5.500
DE	6.50
TE	298.0
D1	1.00000000
TD0	1
SFO1	375.9316815
NUC1	19F
P1	12.00
PLW1	32.47200012

F2 - Processing parameters

SI	65536
SF	375.9692784
WDW	EM
SSB	0
LB	0.30
GB	0
PC	1.00

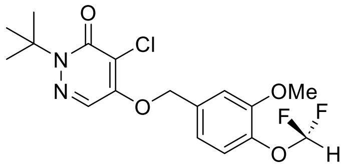

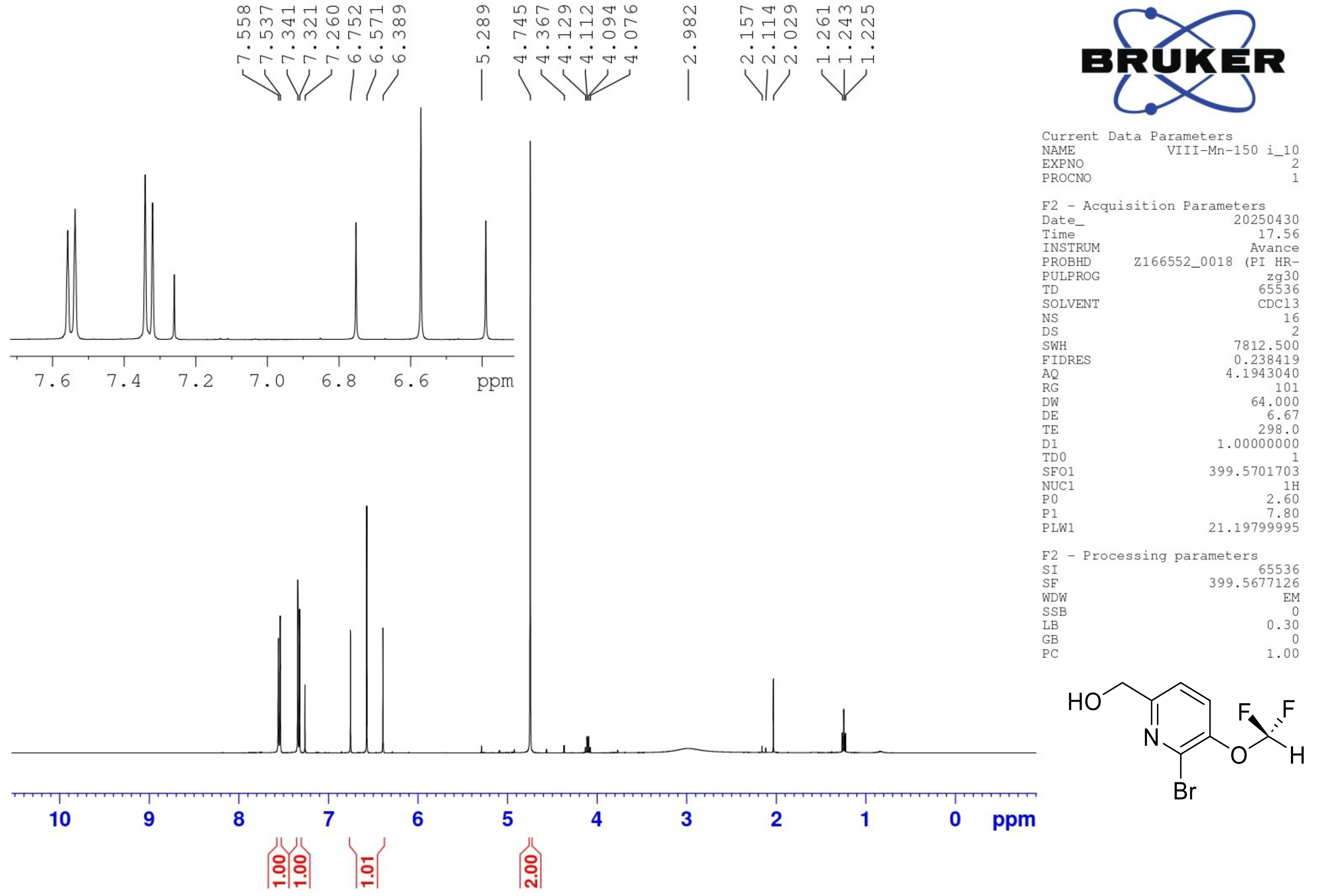

7.558
7.537
7.341
7.321
7.260
6.752
6.571
6.389
5.289
4.745
4.367
4.129
4.112
4.094
4.076
2.982
2.157
2.114
2.029
1.261
1.243
1.225
7.6 7.4 7.2 7.0 6.8 6.6 ppm
10 9 8 7 6 5 4 3 2 1 0 ppm
1.00
1.00
1.01
2.00
Current Data Parameters
NAME VIII-Mn-150 i_10
EXPNO 2
PROCNO 1
F2 - Acquisition Parameters
Date_ 20250430
Time 17.56
INSTRUM Avance
PROBHD Z166552_0018 (PI HR-
PULPROG zg30
TD 65536
SOLVENT CDCl3
NS 16
DS 2
SWH 7812.500
FIDRES 0.238419
AQ 4.1943040
RG 101
DW 64.000
DE 6.67
TE 298.0
D1 1.00000000
TD0 1
SFO1 399.5701703
NUC1 1H
P0 2.60
P1 7.80
PLW1 21.19799995
F2 - Processing parameters
SI 65536
SF 399.5677126
WDW EM
SSB 0
LB 0.30
GB 0
PC 1.00
HO
N
Br
O
F
F
H

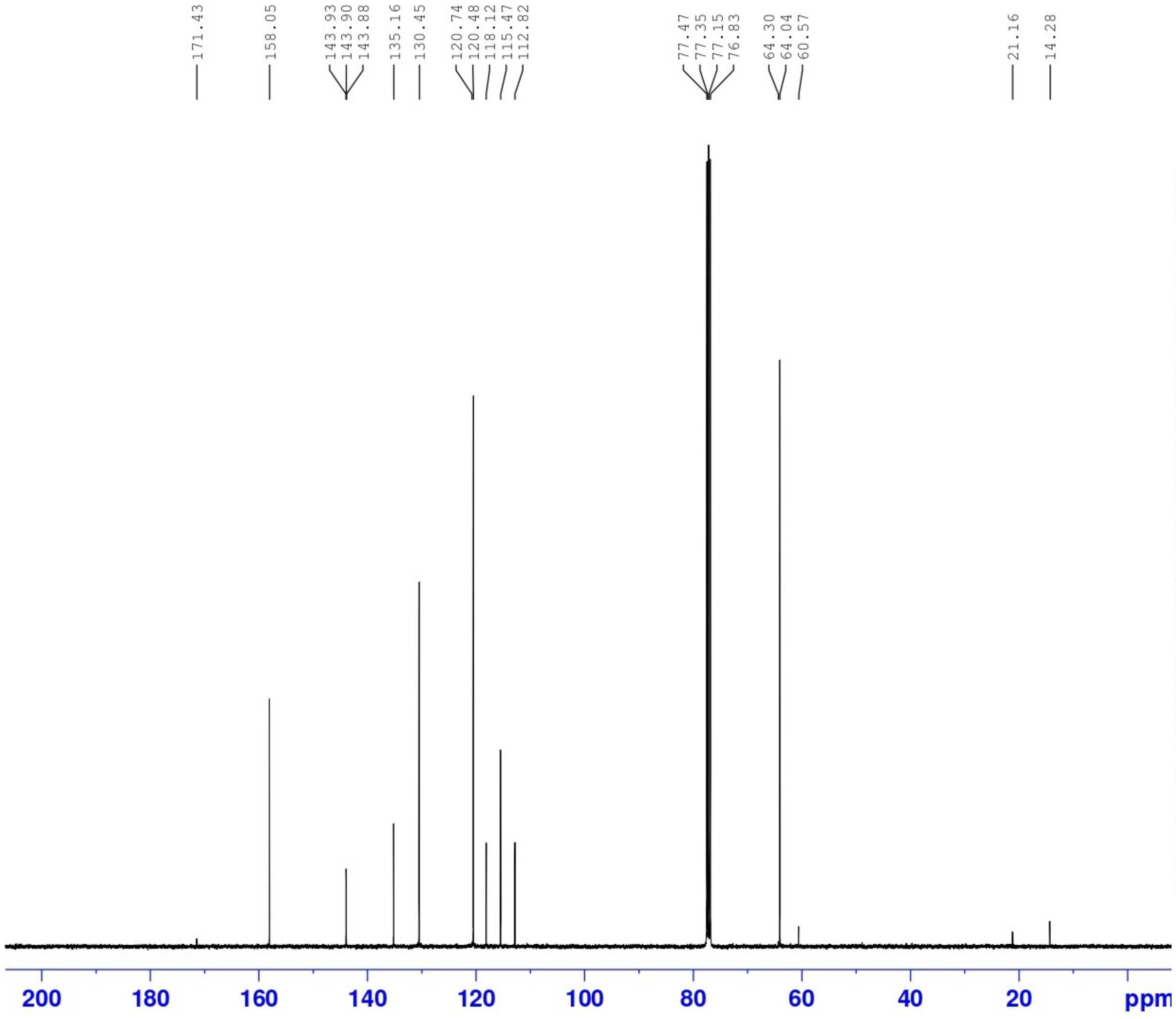

Current Data Parameters
NAME VIII-Mn-150 i_12
EXPNO 3
PROCNO 1

F2 - Acquisition Parameters
Date_ 20250430
Time 20.08
INSTRUM Avance
PROBHD Z166552_0018 (PI HR-
PULPROG zgpg30
TD 65536
SOLVENT CDCl3
NS 2048
DS 4
SWH 23809.524
FIDRES 0.726609
AQ 1.3762560
RG 101
DW 21.000
DE 6.50
TE 298.0
D1 2.00000000
D11 0.03000000
TD0 1
SFO1 100.4814260
NUC1 13C
P0 2.67
P1 8.00
PLW1 88.22599792
SFO2 399.5693013
NUC2 1H
CPDPRG[2] waltz65
PCPD2 90.00
PLW2 21.19799995
PLW12 0.15922000
PLW13 0.08008700

F2 - Processing parameters
SI 32768
SF 100.4713682
WDW EM
SSB 0
LB 1.00
GB 0
PC 1.40

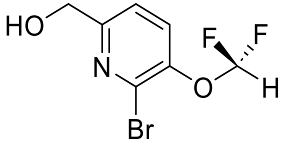

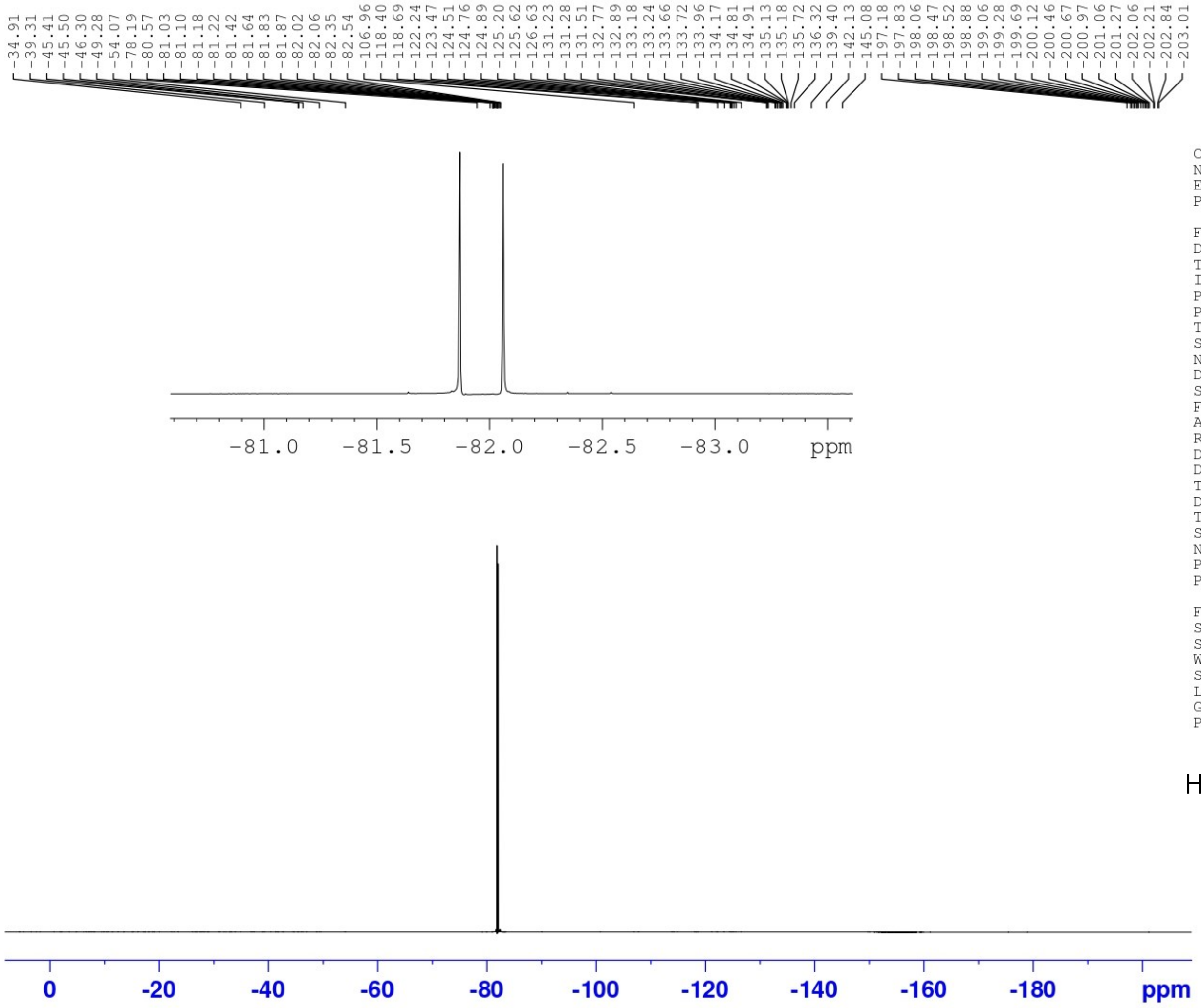

Current Data Parameters
NAME VIII-Mn-150 i_11
EXPNO 3
PROCNO 1

F2 - Acquisition Parameters
Date_ 20250430
Time 18.00
INSTRUM Avance
PROBHD Z166552_0018 (PI HR-
PULPROG zg
TD 131072
SOLVENT CDCl3
NS 16
DS 4
SWH 90909.091
FIDRES 1.387163
AQ 0.7208960
RG 101
DW 5.500
DE 6.50
TE 298.0
D1 1.00000000
TD0 1
SFO1 375.9316815
NUC1 19F
P1 12.00
PLW1 32.47200012

F2 - Processing parameters
SI 65536
SF 375.9692784
WDW EM
SSB 0
LB 0.30
GB 0
PC 1.00

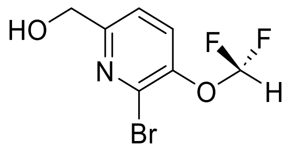

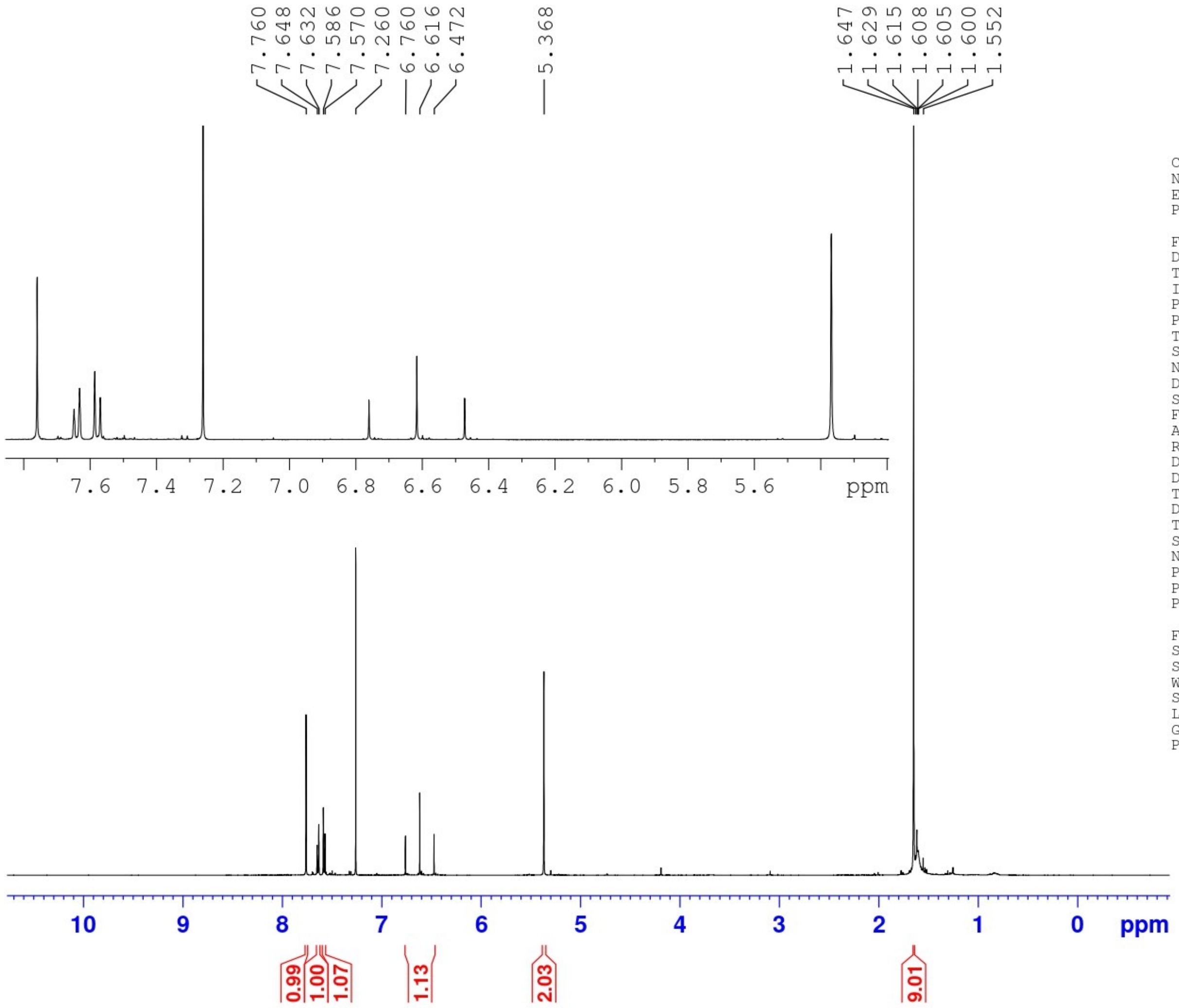

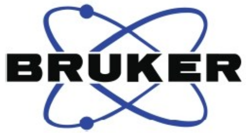

Current Data Parameters
NAME IX-Mn-12 i_10
EXPNO 3
PROCNO 1

F2 - Acquisition Parameters
Date_ 20250509
Time 16.36
INSTRUM AS500-NEO
PROBHD Z168772_0026 (CPP1.1
PULPROG zg30
TD 65536
SOLVENT CDCl3
NS 16
DS 2
SWH 10000.000
FIDRES 0.305176
AQ 3.2767999
RG 57
DW 50.000
DE 10.45
TE 298.0
D1 1.00000000
TD0 1
SFO1 499.7860862
NUC1 1H
P0 4.00
P1 12.00
PLW1 16.91500092

F2 - Processing parameters
SI 65536
SF 499.7830117
WDW EM
SSB 0
LB 0.30
GB 0
PC 1.00

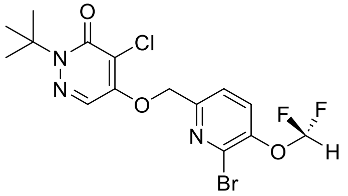

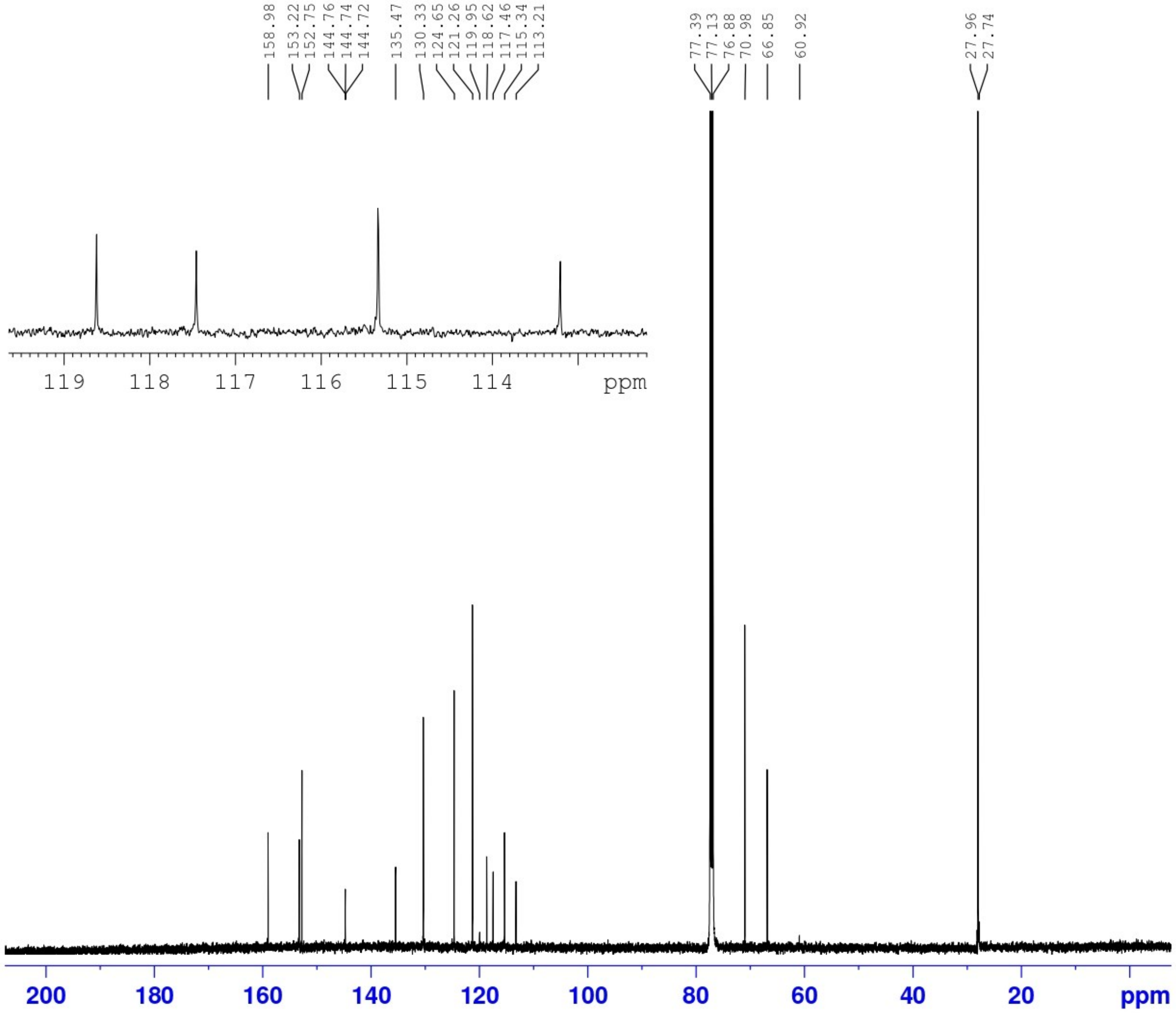

BRUKER

Current Data Parameters

NAME	IX-Mn-12 i_12
EXPNO	3
PROCNO	1

F2 - Acquisition Parameters

Date_	20250510
Time	10.44
INSTRUM	AS500-NEO
PROBHD	Z168772_0026 (CPP1.1
PULPROG	zgpg30
TD	65536
SOLVENT	CDCl3
NS	2048
DS	4
SWH	30120.482
FIDRES	0.919204
AQ	1.0878977
RG	101
DW	16.600
DE	18.00
TE	298.0
D1	2.00000000
D11	0.03000000
TD0	1
SFO1	125.6831024
NUC1	13C
P0	3.33
P1	10.00
PLW1	59.16400146
SFO2	499.7849991
NUC2	1H
CPDPRG[2]	waltz65
PCPD2	80.00
PLW2	16.91500092
PLW12	0.38058999
PLW13	0.19113000

F2 - Processing parameters

SI	32768
SF	125.6705212
WDW	EM
SSB	0

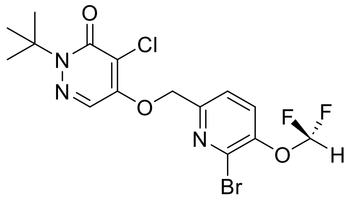

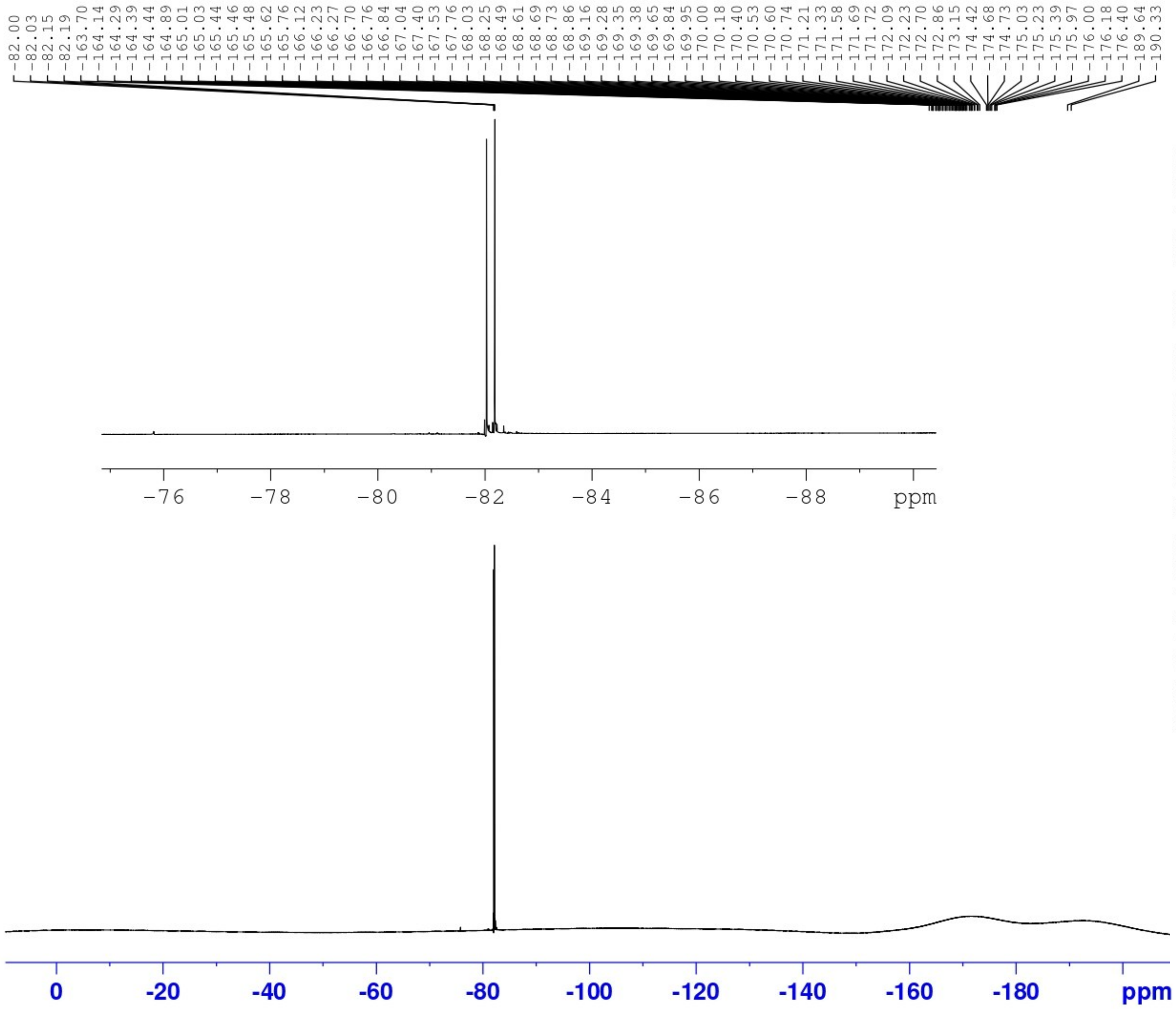

Current Data Parameters

NAME	IX-Mn-12 i_11
EXPNO	3
PROCNO	1

F2 - Acquisition Parameters

Date_	20250509
Time	16.38
INSTRUM	AS500-NEO
PROBHD	Z168772_0026 (CPP1.1
PULPROG	zg
TD	131072
SOLVENT	CDCl3
NS	16
DS	4
SWH	113636.364
FIDRES	1.733953
AQ	0.5767168
RG	11.3
DW	4.400
DE	18.00
TE	298.0
D1	1.00000000
TD0	1
SFO1	470.2188444
NUC1	19F
P1	15.00
PLW1	10.89000034

F2 - Processing parameters

SI	65536
SF	470.2658710
WDW	EM
SSB	0
LB	0.30
GB	0
PC	1.00

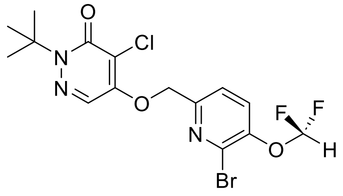

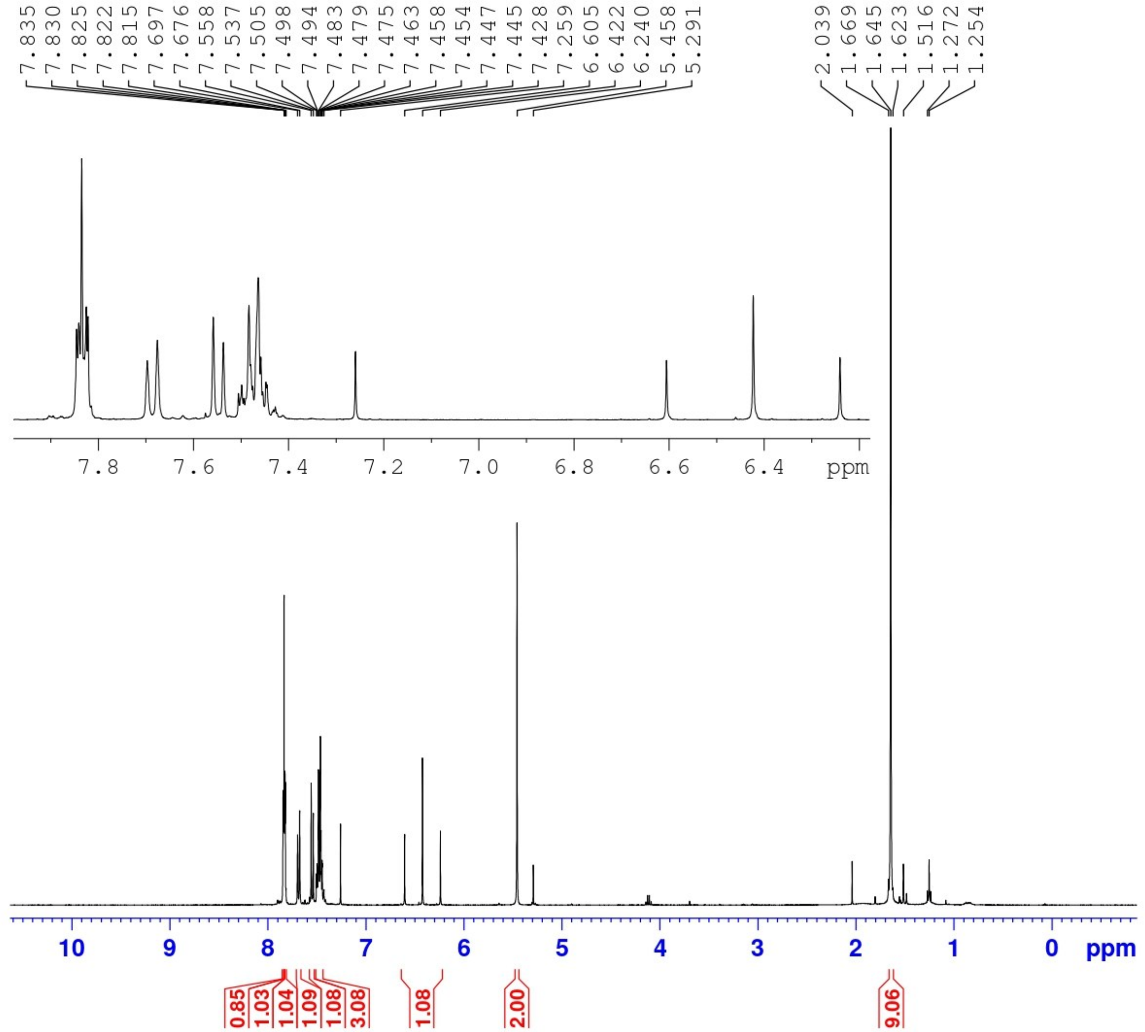

Current Data Parameters
NAME IX-Mn-40 ii_10
EXPNO 3
PROCNO 1

F2 - Acquisition Parameters

Date_	20250528
Time	17.37
INSTRUM	Avance
PROBHD	Z166552_0018 (PI HR-
PULPROG	zg30
TD	65536
SOLVENT	CDCl3
NS	16
DS	2
SWH	7812.500
FIDRES	0.238419
AQ	4.1943040
RG	101
DW	64.000
DE	6.67
TE	298.0
D1	1.00000000
TD0	1
SFO1	399.5424672
NUC1	1H
P0	2.60
P1	7.80
PLW1	21.19799995

F2 - Processing parameters

SI	65536
SF	399.5400100
WDW	EM
SSB	0
LB	0.30
GB	0
PC	1.00

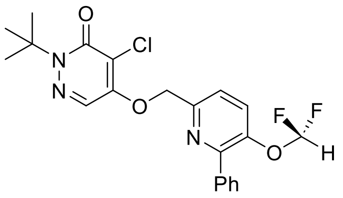

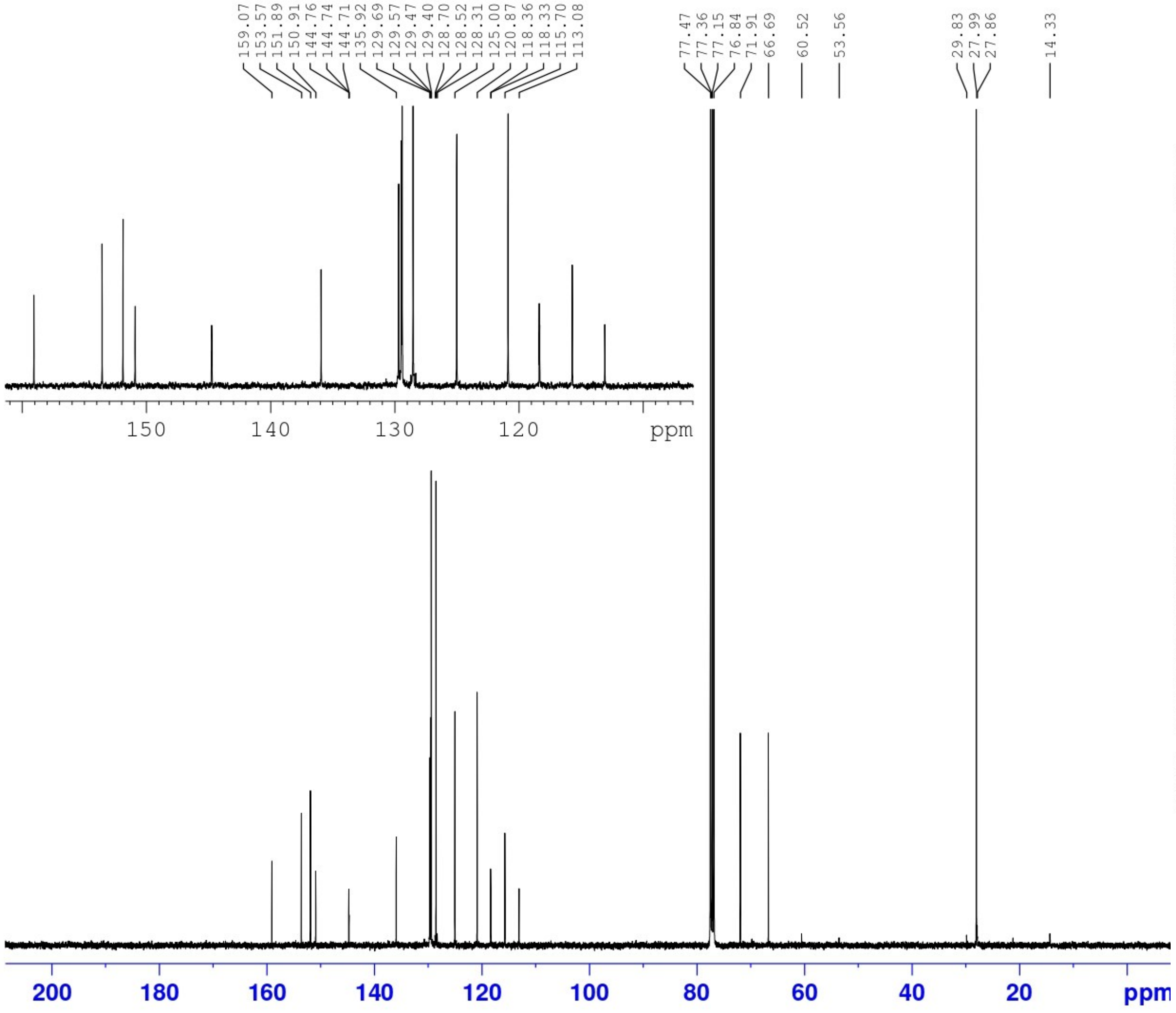

Current Data Parameters
NAME IX-Mn-40 ii_12
EXPNO 3
PROCNO 1

F2 - Acquisition Parameters
Date_ 20250528
Time 20.15
INSTRUM Avance
PROBHD Z166552_0018 (PI HR-
PULPROG zgpg30
TD 65536
SOLVENT CDCl3
NS 2048
DS 4
SWH 23809.524
FIDRES 0.726609
AQ 1.3762560
RG 101
DW 21.000
DE 6.50
TE 298.0
D1 2.00000000
D11 0.03000000
TD0 1
SFO1 100.4744593
NUC1 13C
P0 2.67
P1 8.00
PLW1 88.22599792
SFO2 399.5415982
NUC2 1H
CPDPRG[2] waltz65
PCPD2 90.00
PLW2 21.19799995
PLW12 0.15922000
PLW13 0.08008700

F2 - Processing parameters
SI 32768
SF 100.4644009
WDW EM

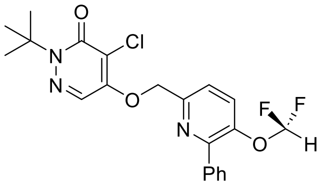

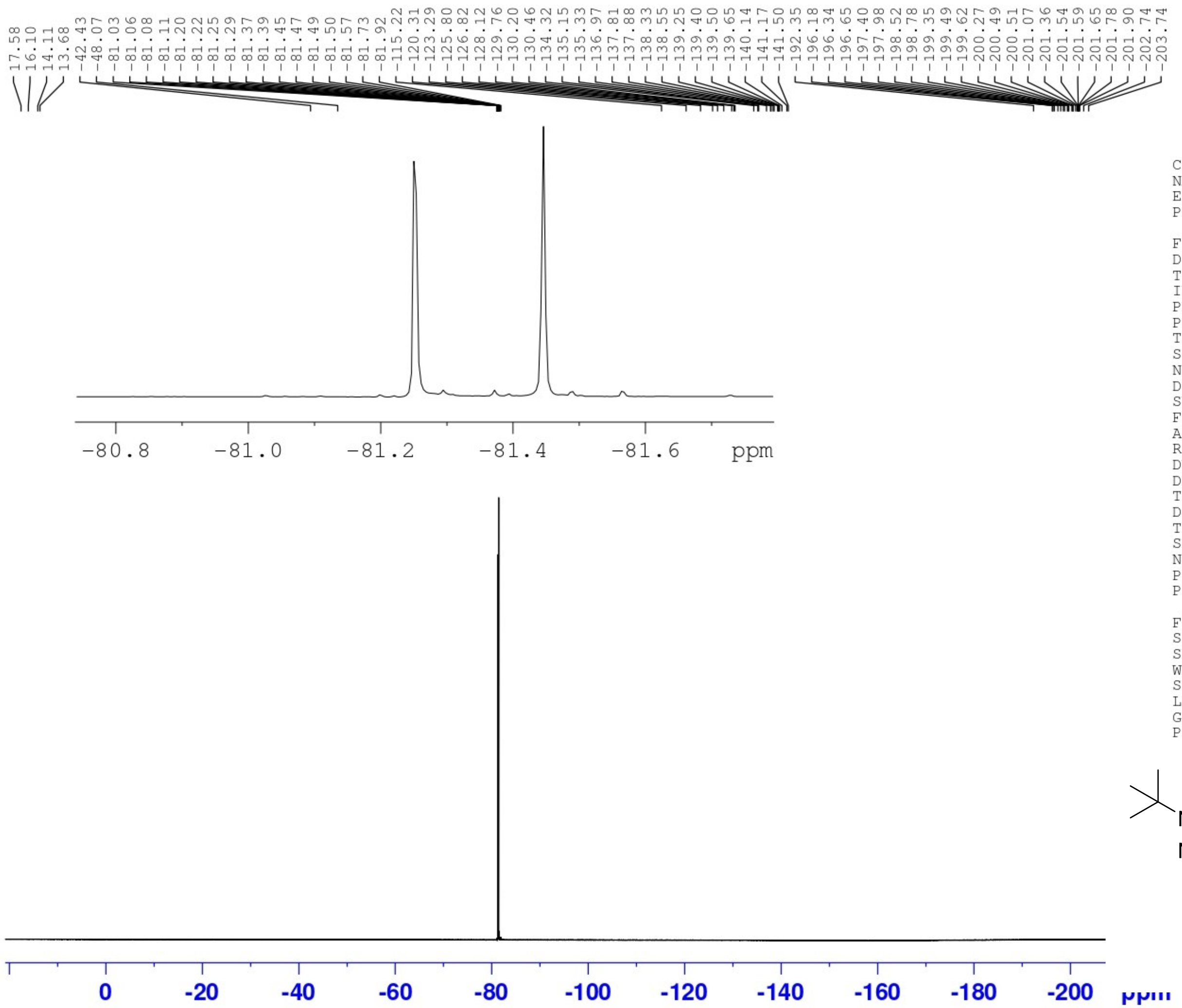

Current Data Parameters
NAME IX-Mn-40 ii_11
EXPNO 3
PROCNO 1

F2 - Acquisition Parameters
Date_ 20250528
Time 17.44
INSTRUM Avance
PROBHD Z166552_0018 (PI HR-
PULPROG zg
TD 131072
SOLVENT CDCl3
NS 16
DS 4
SWH 90909.091
FIDRES 1.387163
AQ 0.7208960
RG 101
DW 5.500
DE 6.50
TE 298.0
D1 1.00000000
TD0 1
SFO1 375.9056172
NUC1 19F
P1 12.00
PLW1 32.47200012

F2 - Processing parameters
SI 65536
SF 375.9432115
WDW EM
SSB 0
LB 0.30
GB 0
PC 1.00

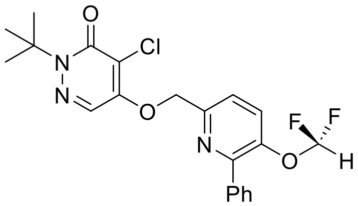

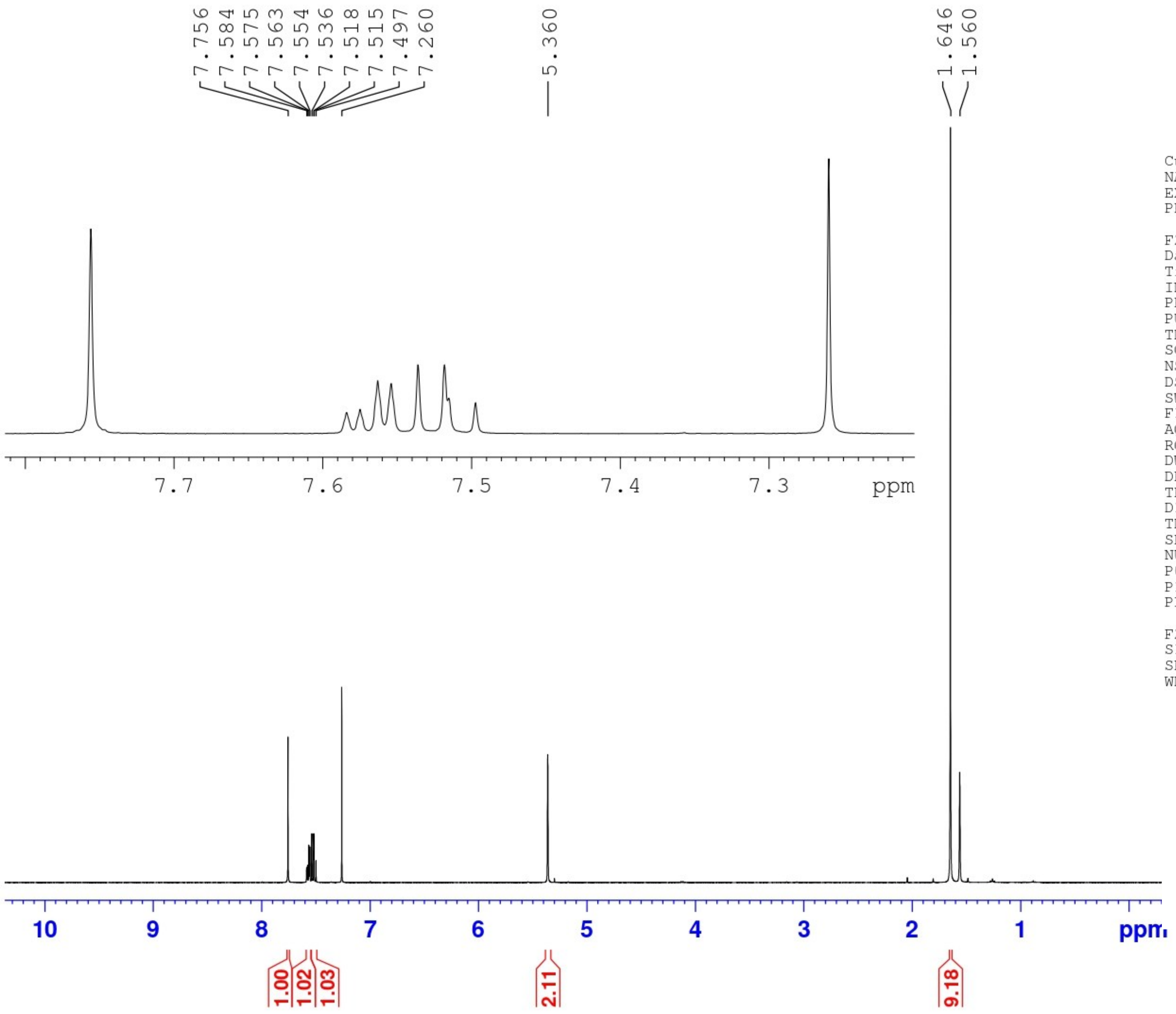

Current Data Parameters
NAME X-Mn-19 i_10
EXPNO 2
PROCNO 1

F2 - Acquisition Parameters
Date_ 20251014
Time 16.31
INSTRUM Avance
PROBHD Z166552_0018 (PI HR-
PULPROG zg30
TD 65536
SOLVENT CDCl3
NS 16
DS 2
SWH 7812.500
FIDRES 0.238419
AQ 4.1943040
RG 101
DW 64.000
DE 6.67
TE 298.0
D1 1.00000000
TD0 1
SFO1 399.5424672
NUC1 1H
P0 2.60
P1 7.80
PLW1 21.19799995

F2 - Processing parameters
SI 65536
SF 399.5400096
WDW EM

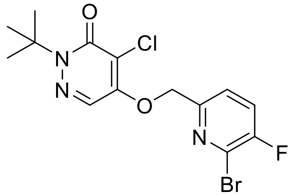

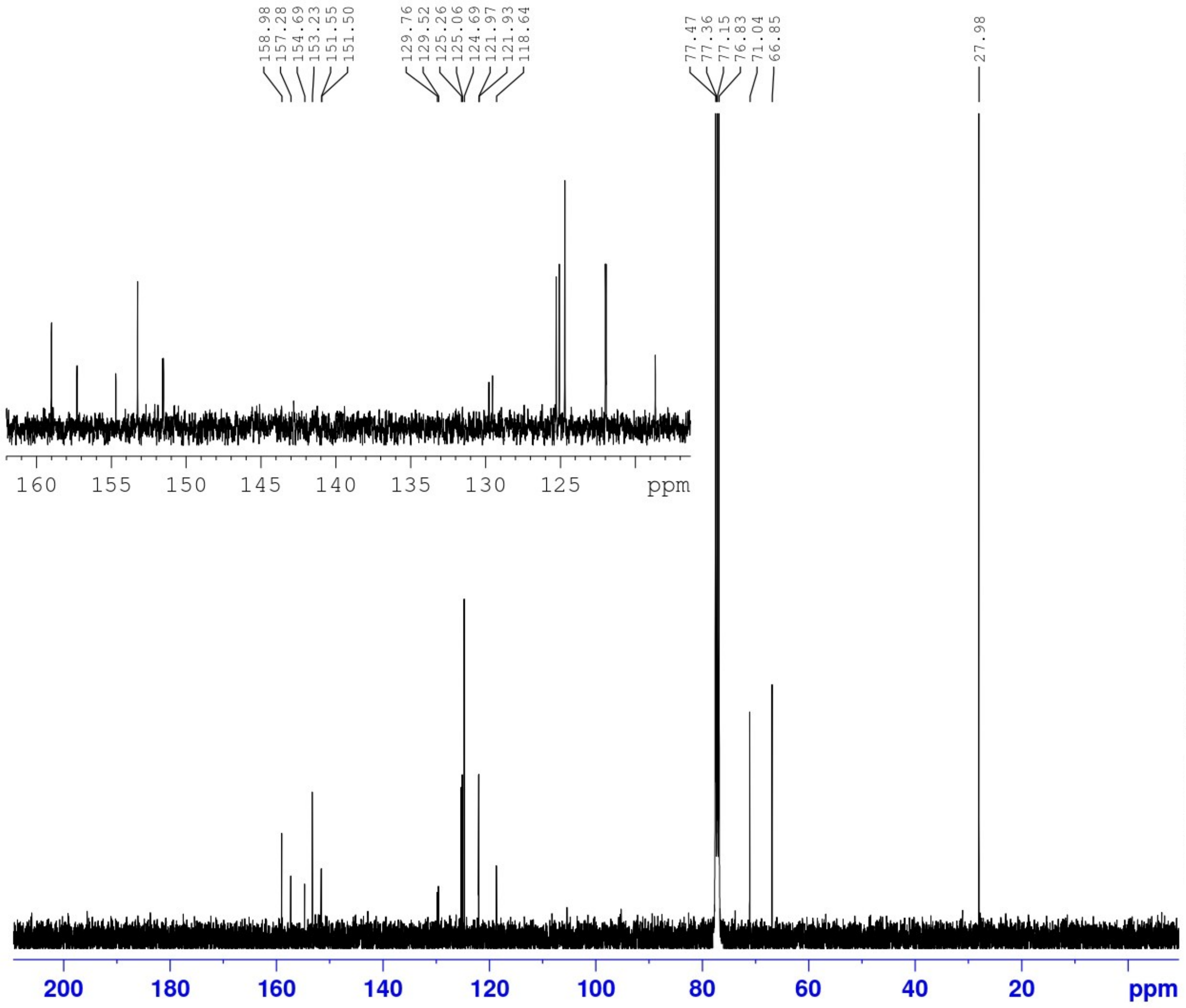

Current Data Parameters
NAME X-Mn-19 i_12
EXPNO 2
PROCNO 1

F2 - Acquisition Parameters
Date_ 20251014
Time 22.05
INSTRUM Avance
PROBHD Z166552_0018 (PI HR-
PULPROG zgpg30
TD 65536
SOLVENT CDCl3
NS 2048
DS 4
SWH 23809.524
FIDRES 0.726609
AQ 1.3762560
RG 101
DW 21.000
DE 6.50
TE 298.0
D1 2.00000000
D11 0.03000000
TD0 1
SFO1 100.4744593
NUC1 13C
P0 2.67
P1 8.00
PLW1 88.22599792
SFO2 399.5415982
NUC2 1H
CPDPRG[2] waltz65
PCPD2 90.00
PLW2 21.19799995
PLW12 0.15922000
PLW13 0.08008700

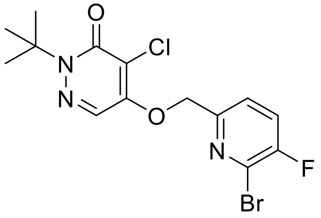

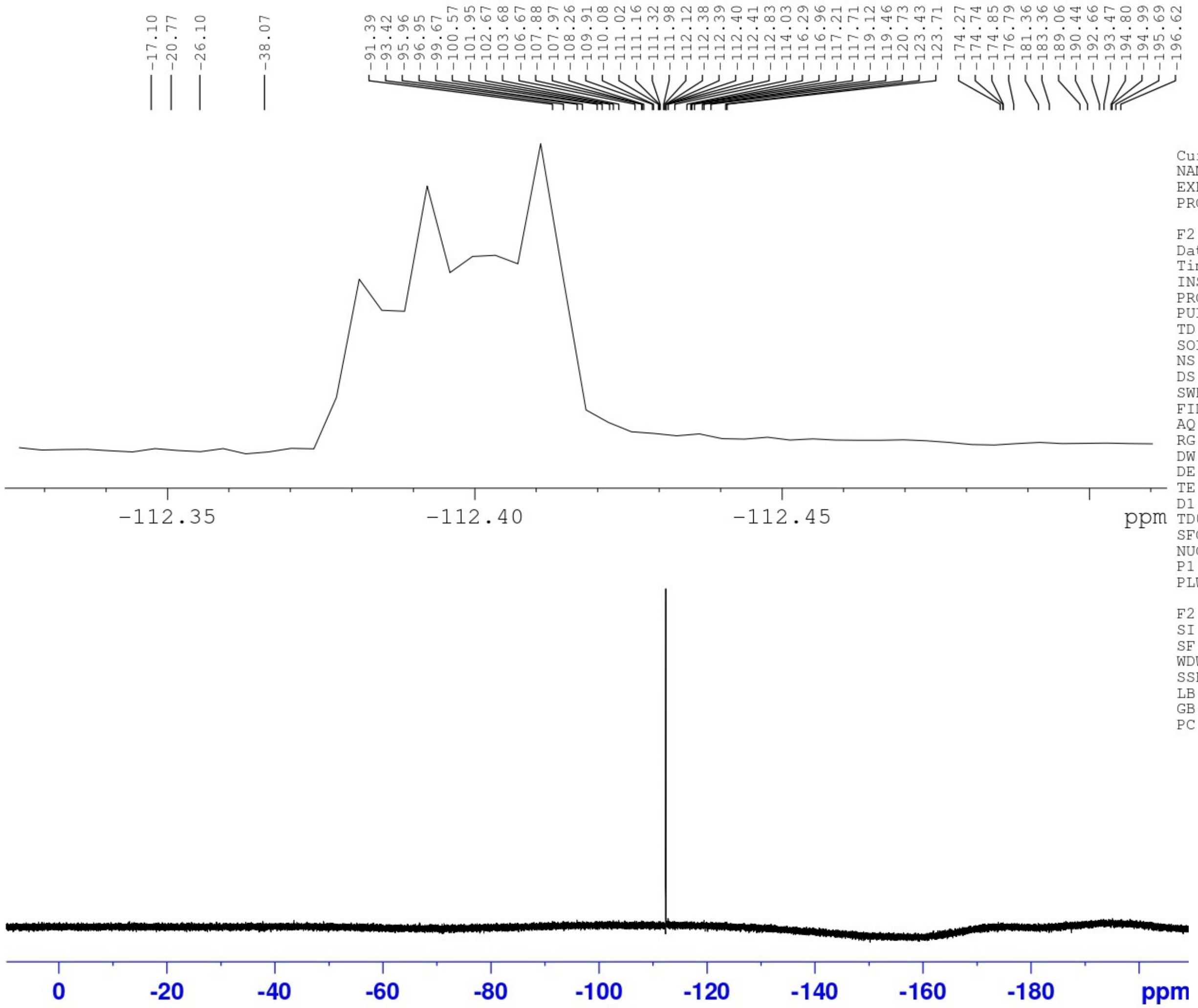

Current Data Parameters
NAME X-Mn-19 i_11
EXPNO 2
PROCNO 1

F2 - Acquisition Parameters
Date_ 20251014
Time 16.33
INSTRUM Avance
PROBHD Z166552_0018 (PI HR-
PULPROG zg
TD 131072
SOLVENT CDCl3
NS 16
DS 4
SWH 90909.091
FIDRES 1.387163
AQ 0.7208960
RG 101
DW 5.500
DE 6.50
TE 298.0
D1 1.00000000
TD0 1
SFO1 375.9056172
NUC1 19F
P1 12.00
PLW1 32.47200012

F2 - Processing parameters
SI 65536
SF 375.9432115
WDW EM
SSB 0
LB 0.30
GB 0
PC 1.00

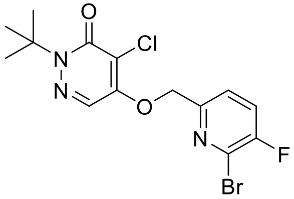

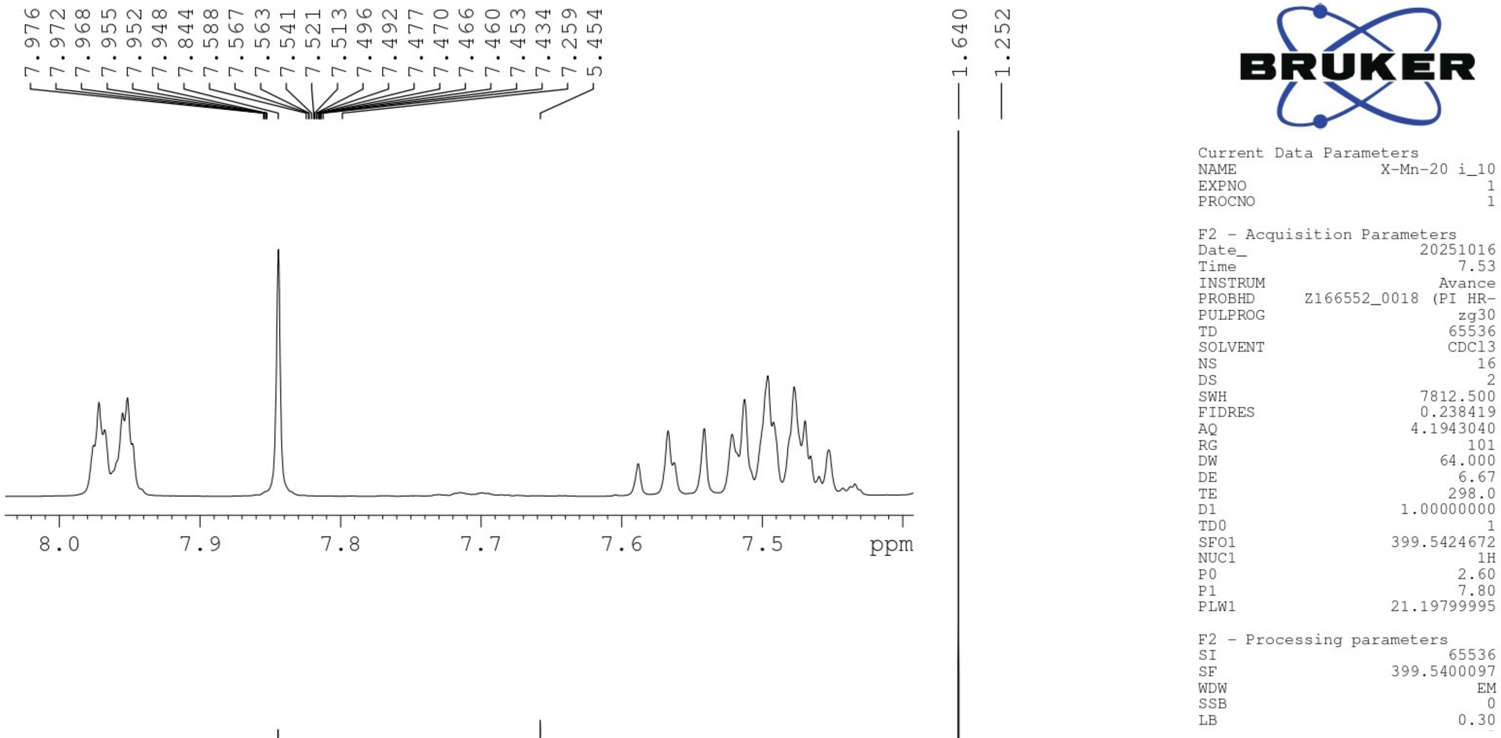
7.976 7.972 7.968 7.955 7.952 7.948 7.844 7.588 7.567 7.563 7.541 7.521 7.513 7.496 7.492 7.477 7.470 7.466 7.460 7.453 7.434 7.259 5.454
1.640 1.252
8.0 7.9 7.8 7.7 7.6 7.5 ppm
10 9 8 7 6 5 4 3 2 1 0
2.00 1.00 1.03 4.08 2.03 9.12
BRUKER
Current Data Parameters
NAME X-Mn-20 i_10
EXPNO 1
PROCNO 1
F2 - Acquisition Parameters
Date_ 20251016
Time 7.53
INSTRUM Avance
PROBHD Z166552_0018 (PI HR-
PULPROG zg30
TD 65536
SOLVENT CDCl3
NS 16
DS 2
SWH 7812.500
FIDRES 0.238419
AQ 4.1943040
RG 101
DW 64.000
DE 6.67
TE 298.0
D1 1.00000000
TD0 1
SFO1 399.5424672
NUC1 1H
P0 2.60
P1 7.80
PLW1 21.19799995
F2 - Processing parameters
SI 65536
SF 399.5400097
WDW EM
SSB 0
LB 0.30

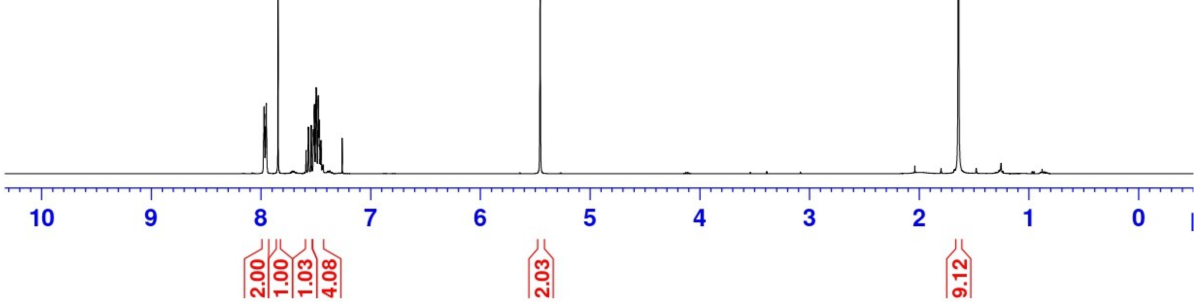

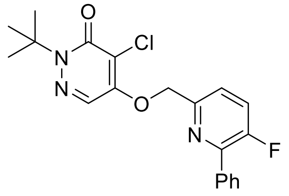
O
Cl
N
N
O
N
F
Ph

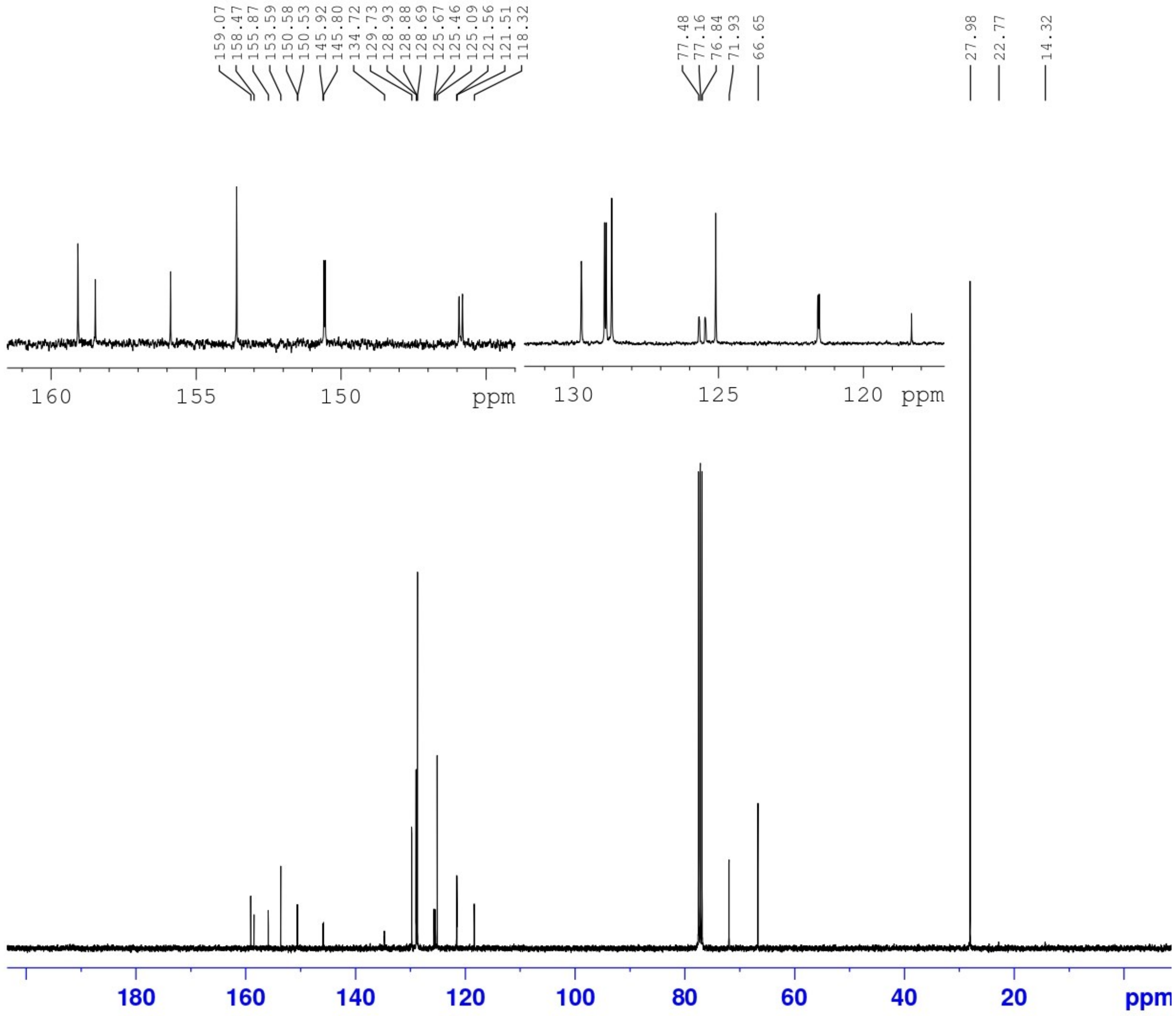

BRUKER

Current Data Parameters
NAME X-Mn-20 i_12
EXPNO 1
PROCNO 1

F2 - Acquisition Parameters
Date_ 20251016
Time 8.26
INSTRUM Avance
PROBHD Z166552_0018 (PI HR-
PULPROG zgpg30
TD 65536
SOLVENT CDCl3
NS 512
DS 4
SWH 23809.524
FIDRES 0.726609
AQ 1.3762560
RG 101
DW 21.000
DE 6.50
TE 298.0
D1 2.00000000
D11 0.03000000
TD0 1
SFO1 100.4744593
NUC1 13C
P0 2.67
P1 8.00
PLW1 88.22599792
SFO2 399.5415982
NUC2 1H
CPDPRG[2] waltz65
PCPD2 90.00
PLW2 21.19799995
PLW12 0.15922000
PLW13 0.08008700

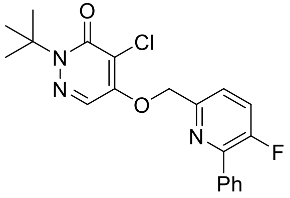

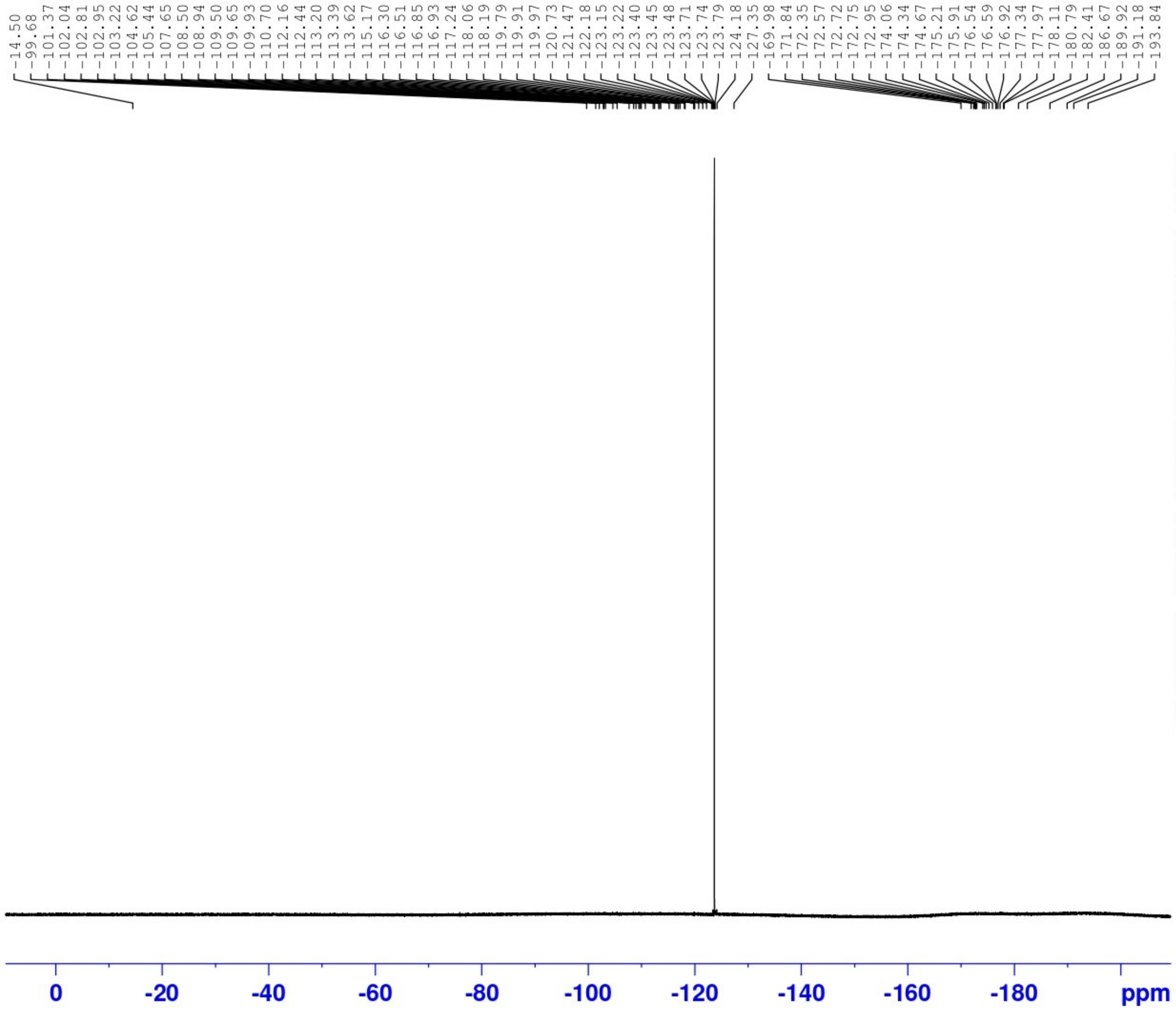

BRUKER

Current Data Parameters
NAME X-Mn-20 i_11
EXPNO 1
PROCNO 1

F2 - Acquisition Parameters
Date_ 20251016
Time 7.55
INSTRUM Avance
PROBHD Z166552_0018 (PI HR-
PULPROG zg
TD 131072
SOLVENT CDCl3
NS 16
DS 4
SWH 90909.091
FIDRES 1.387163
AQ 0.7208960
RG 101
DW 5.500
DE 6.50
TE 298.0
D1 1.00000000
TD0 1
SFO1 375.9056172
NUC1 19F
P1 12.00
PLW1 32.47200012

F2 - Processing parameters
SI 65536
SF 375.9432115
WDW EM
SSB 0
LB 0.30
GB 0

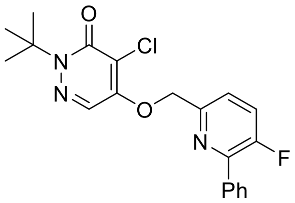

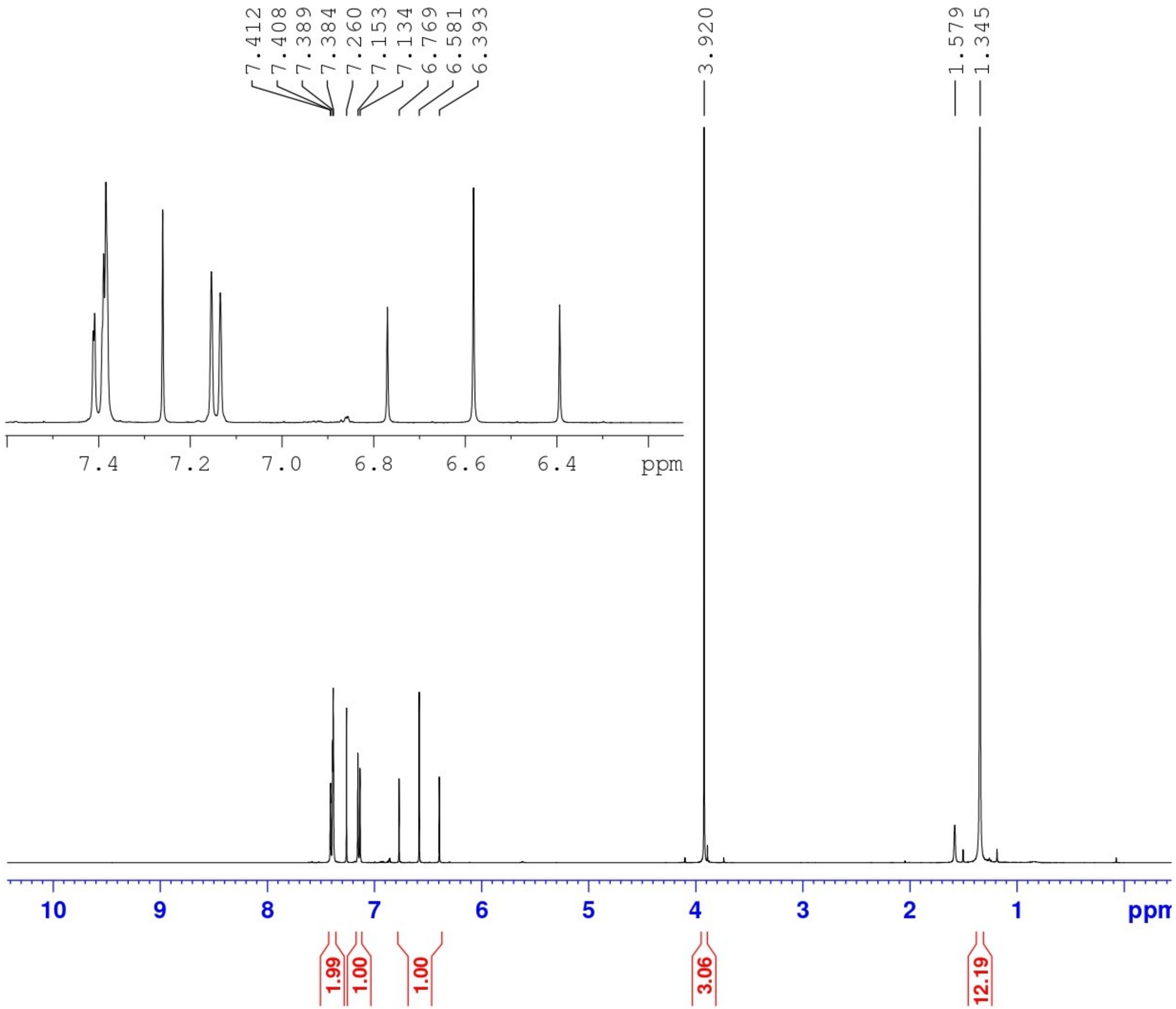

Current Data Parameters
NAME VIII-Mn-99 i_10
EXPNO 2
PROCNO 1

F2 - Acquisition Parameters
Date_ 20250403
Time 13.33
INSTRUM Avance
PROBHD Z166552_0018 (PI HR-
PULPROG zg30
TD 65536
SOLVENT CDCl3
NS 16
DS 2
SWH 7812.500
FIDRES 0.238419
AQ 4.1943040
RG 101
DW 64.000
DE 6.67
TE 298.0
D1 1.00000000
TD0 1
SFO1 399.5701703
NUC1 1H
P0 2.60
P1 7.80
PLW1 21.19799995

F2 - Processing parameters
SI 65536
SF 399.5677126
WDW EM
SSB 0
LB 0.30
GB 0
PC 1.00

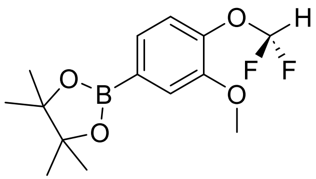

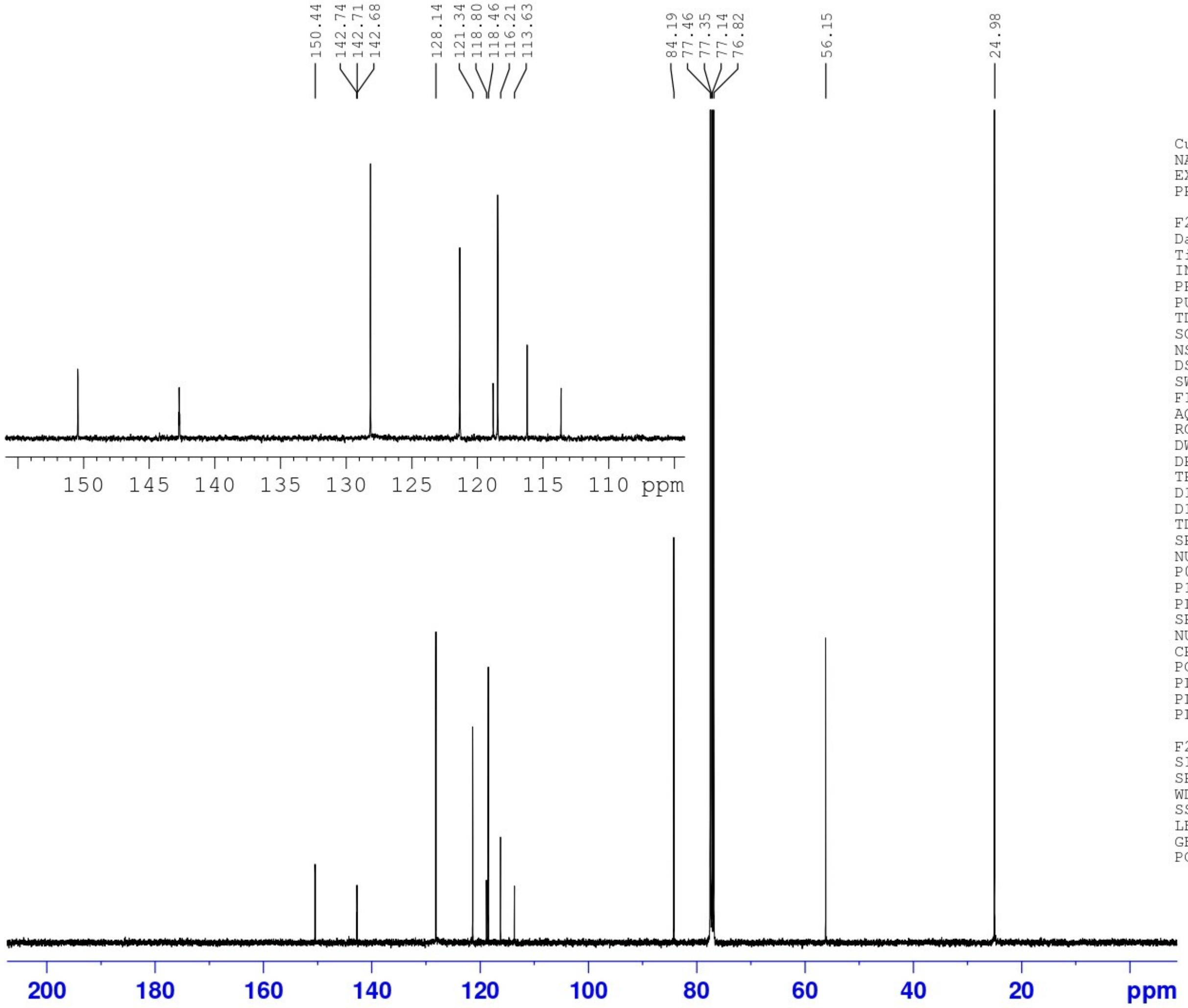

Current Data Parameters
NAME VIII-Mn-99 i_12
EXPNO 2
PROCNO 1

F2 - Acquisition Parameters
Date_ 20250404
Time 6.00
INSTRUM Avance
PROBHD Z166552_0018 (PI HR-
PULPROG zgpg30
TD 65536
SOLVENT CDCl3
NS 2048
DS 4
SWH 23809.524
FIDRES 0.726609
AQ 1.3762560
RG 101
DW 21.000
DE 6.50
TE 298.0
D1 2.00000000
D11 0.03000000
TD0 1
SFO1 100.4814260
NUC1 13C
P0 2.67
P1 8.00
PLW1 88.22599792
SFO2 399.5693013
NUC2 1H
CPDPRG[2] waltz65
PCPD2 90.00
PLW2 21.19799995
PLW12 0.15922000
PLW13 0.08008700

F2 - Processing parameters
SI 32768
SF 100.4713666
WDW EM
SSB 0
LB 1.00
GB 0
PC 1.40

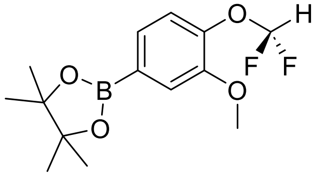

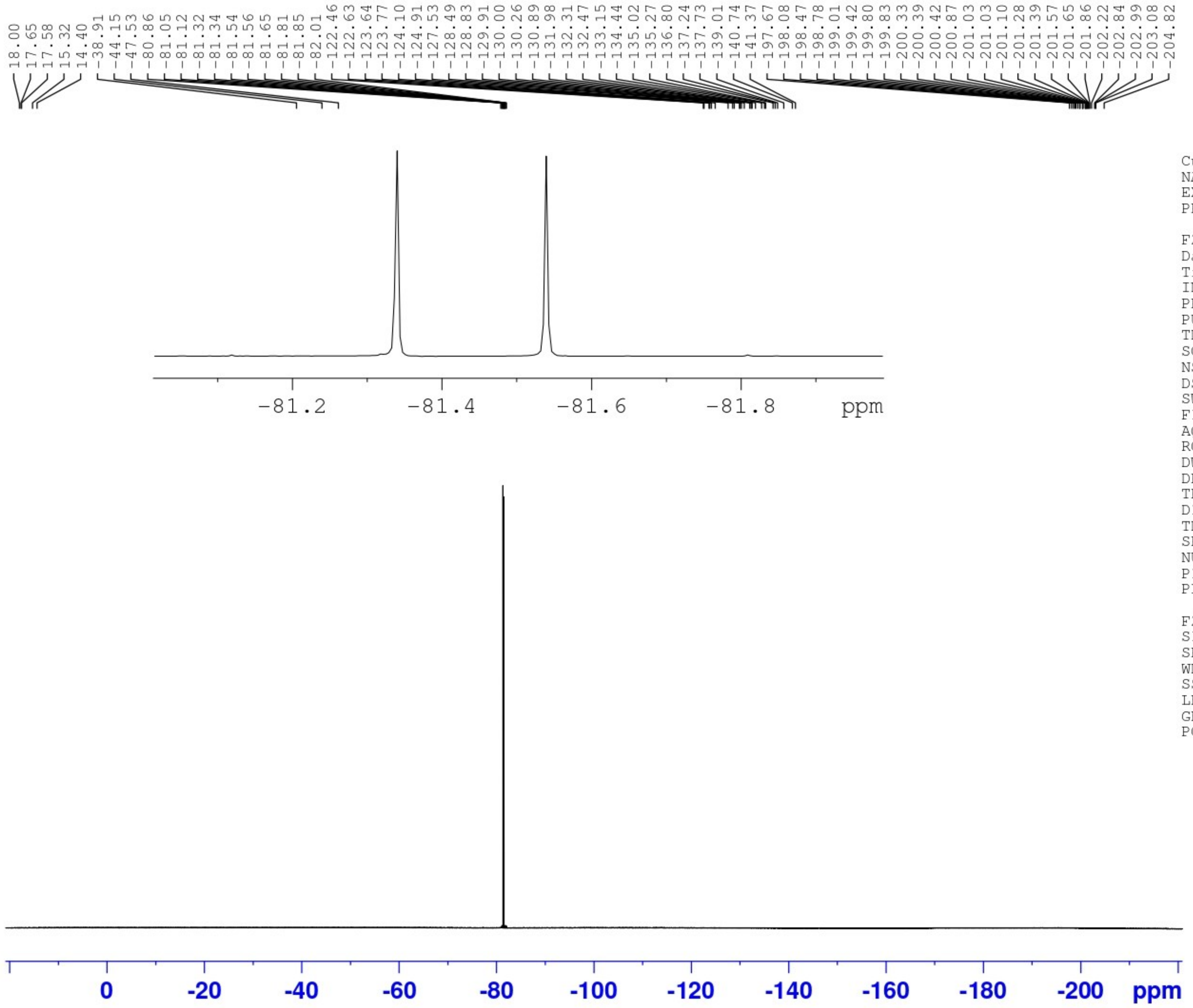

Current Data Parameters	
NAME	VIII-Mn-99 i_11
EXPNO	2
PROCNO	1
F2 - Acquisition Parameters	
Date_	20250403
Time	13.35
INSTRUM	Avance
PROBHD	Z166552_0018 (PI HR-
PULPROG	zg
TD	131072
SOLVENT	CDCl3
NS	16
DS	4
SWH	90909.091
FIDRES	1.387163
AQ	0.7208960
RG	101
DW	5.500
DE	6.50
TE	298.0
D1	1.00000000
TD0	1
SFO1	375.9316815
NUC1	19F
P1	12.00
PLW1	32.47200012
F2 - Processing parameters	
SI	65536
SF	375.9692784
WDW	EM
SSB	0
LB	0.30
GB	0
PC	1.00

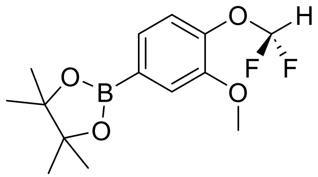

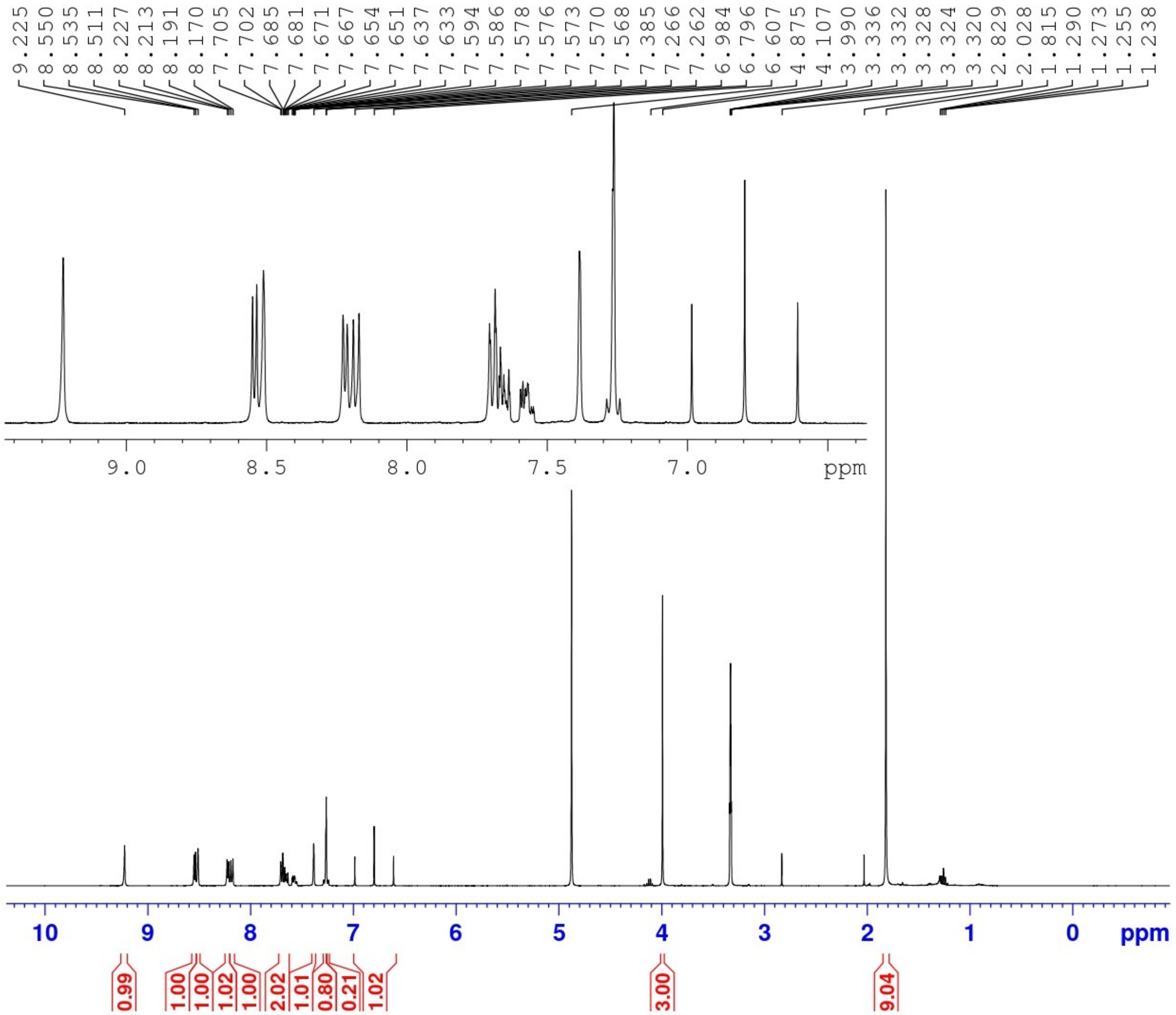

Current Data Parameters
NAME IX-Mn-120 i_10
EXPNO 2
PROCNO 1

F2 - Acquisition Parameters
Date_ 20250731
Time 17.17
INSTRUM Avance
PROBHD Z166552_0018 (PI HR-
PULPROG zg30
TD 65536
SOLVENT MeOD
NS 16
DS 2
SWH 7812.500
FIDRES 0.238419
AQ 4.1943040
RG 101
DW 64.000
DE 6.67
TE 298.0
D1 1.00000000
TD0 1
SFO1 399.5424672
NUC1 1H
P0 2.60
P1 7.80
PLW1 21.19799995

F2 - Processing parameters
SI 65536
SF 399.5400000
WDW EM
SSB 0
LB 0.30
GB 0
PC 1.00

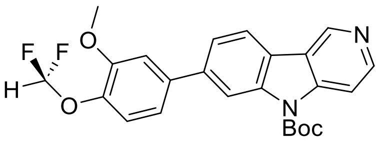

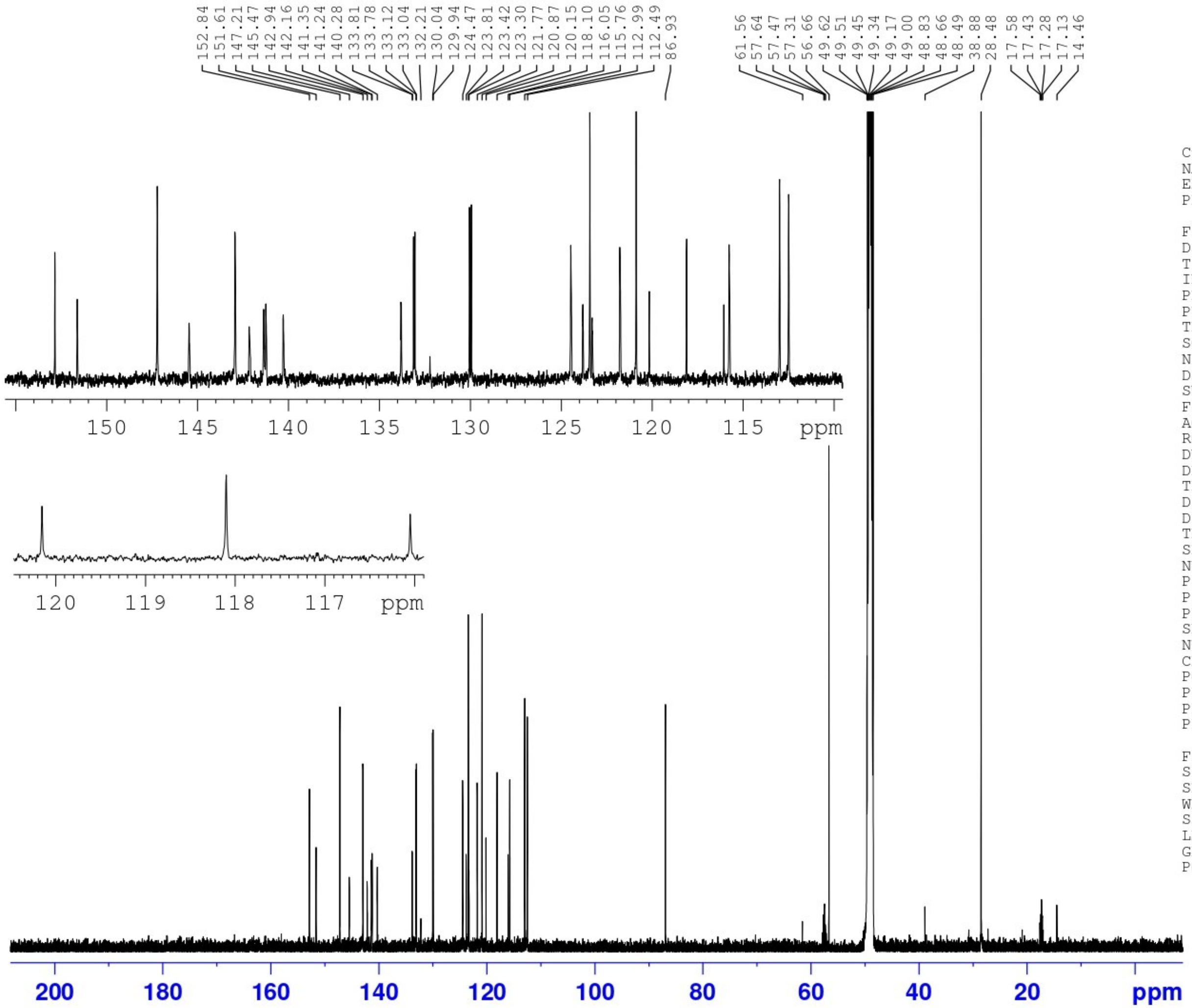

Current Data Parameters
NAME IX-Mn-120 i re_12
EXPNO 4
PROCNO 1

F2 - Acquisition Parameters
Date_ 20251007
Time 21.44
INSTRUM AS500-NEO
PROBHD Z168772_0026 (CPP1.1
PULPROG zgpg30
TD 65536
SOLVENT MeOD
NS 4096
DS 4
SWH 30120.482
FIDRES 0.919204
AQ 1.0878977
RG 101
DW 16.600
DE 18.00
TE 298.0
D1 2.00000000
D11 0.03000000
TD0 1
SFO1 125.6831024
NUC1 13C
P0 3.33
P1 10.00
PLW1 59.16400146
SFO2 499.7849991
NUC2 1H
CPDPRG[2] waltz65
PCPD2 80.00
PLW2 16.91500092
PLW12 0.38058999
PLW13 0.19113000

F2 - Processing parameters
SI 32768
SF 125.6703590
WDW EM
SSB 0
LB 1.00
GB 0
PC 1.40

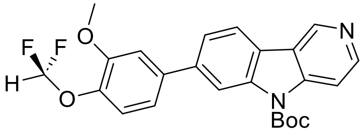

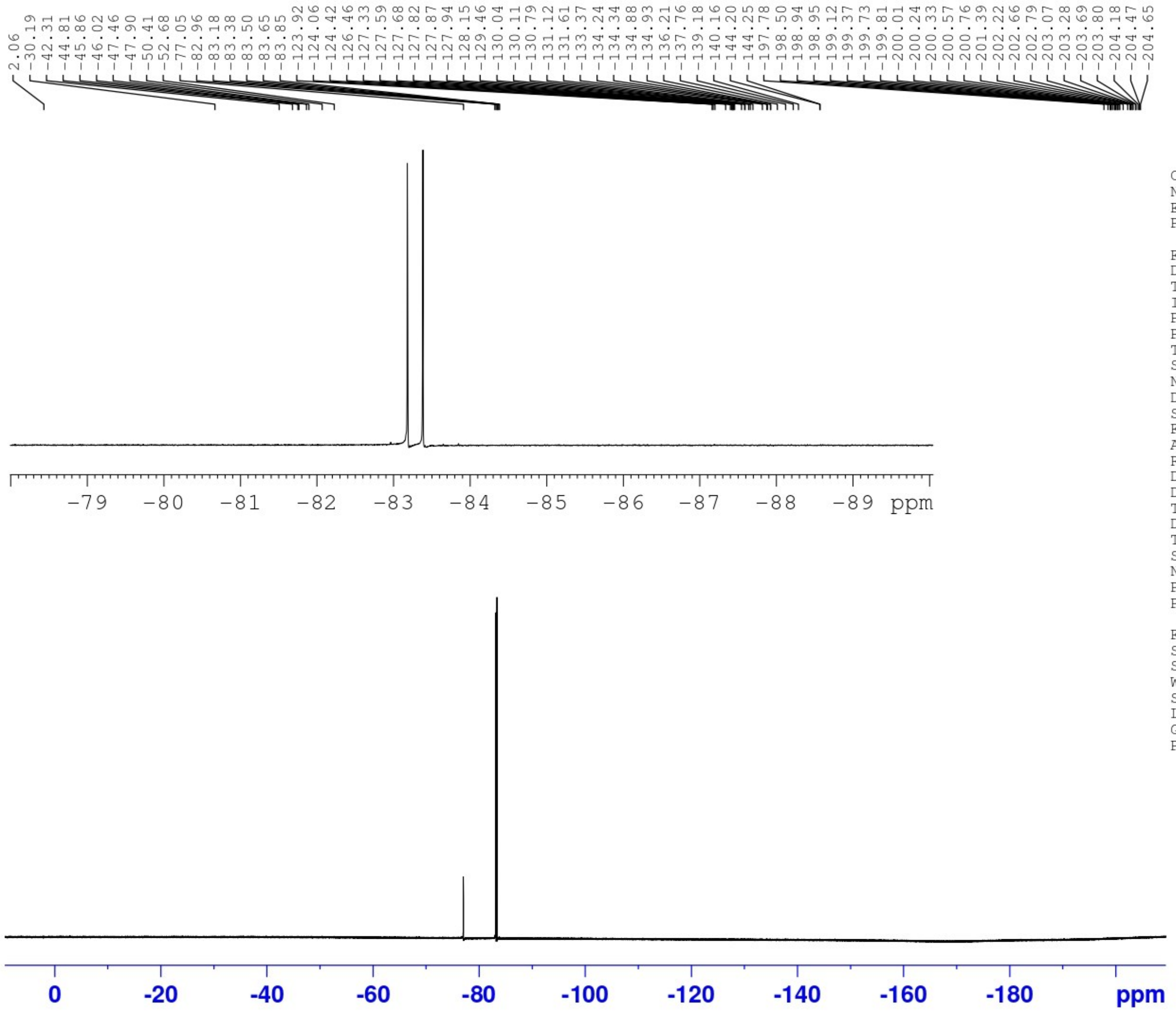

Current Data Parameters

NAME	IX-Mn-120 i_11
EXPNO	2
PROCNO	1

F2 - Acquisition Parameters

Date_	20250731
Time	17.18
INSTRUM	Avance
PROBHD	Z166552_0018 (PI HR-
PULPROG	zg
TD	131072
SOLVENT	MeOD
NS	16
DS	4
SWH	90909.091
FIDRES	1.387163
AQ	0.7208960
RG	101
DW	5.500
DE	6.50
TE	298.0
D1	1.00000000
TD0	1
SFO1	375.9056172
NUC1	19F
P1	12.00
PLW1	32.47200012

F2 - Processing parameters

SI	65536
SF	375.9432115
WDW	EM
SSB	0
LB	0.30
GB	0
PC	1.00

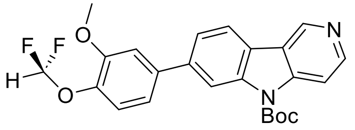

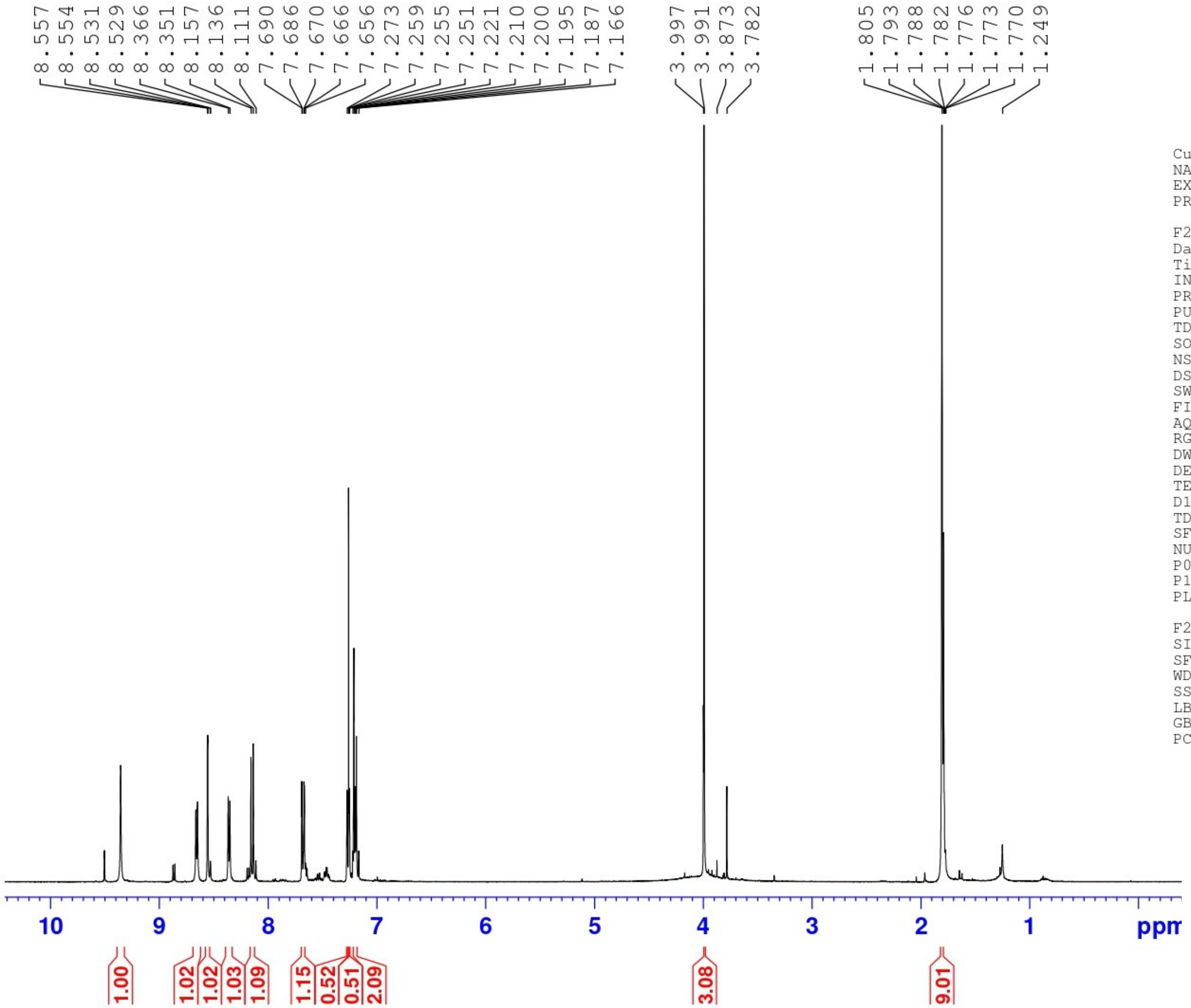

BRUKER

Current Data Parameters
NAME IX-Mn-144 i (P)_10
EXPNO 1
PROCNO 1

F2 - Acquisition Parameters
Date_ 20251007
Time 19.10
INSTRUM Avance
PROBHD Z166552_0018 (PI HR-
PULPROG zg30
TD 65536
SOLVENT CDCl3
NS 16
DS 2
SWH 7812.500
FIDRES 0.238419
AQ 4.1943040
RG 101
DW 64.000
DE 6.67
TE 298.0
D1 1.00000000
TD0 1
SFO1 399.5424672
NUC1 1H
P0 2.60
P1 7.80
PLW1 21.19799995

F2 - Processing parameters
SI 65536
SF 399.5400099
WDW EM
SSB 0
LB 0.30
GB 0
PC 1.00

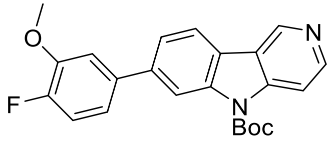

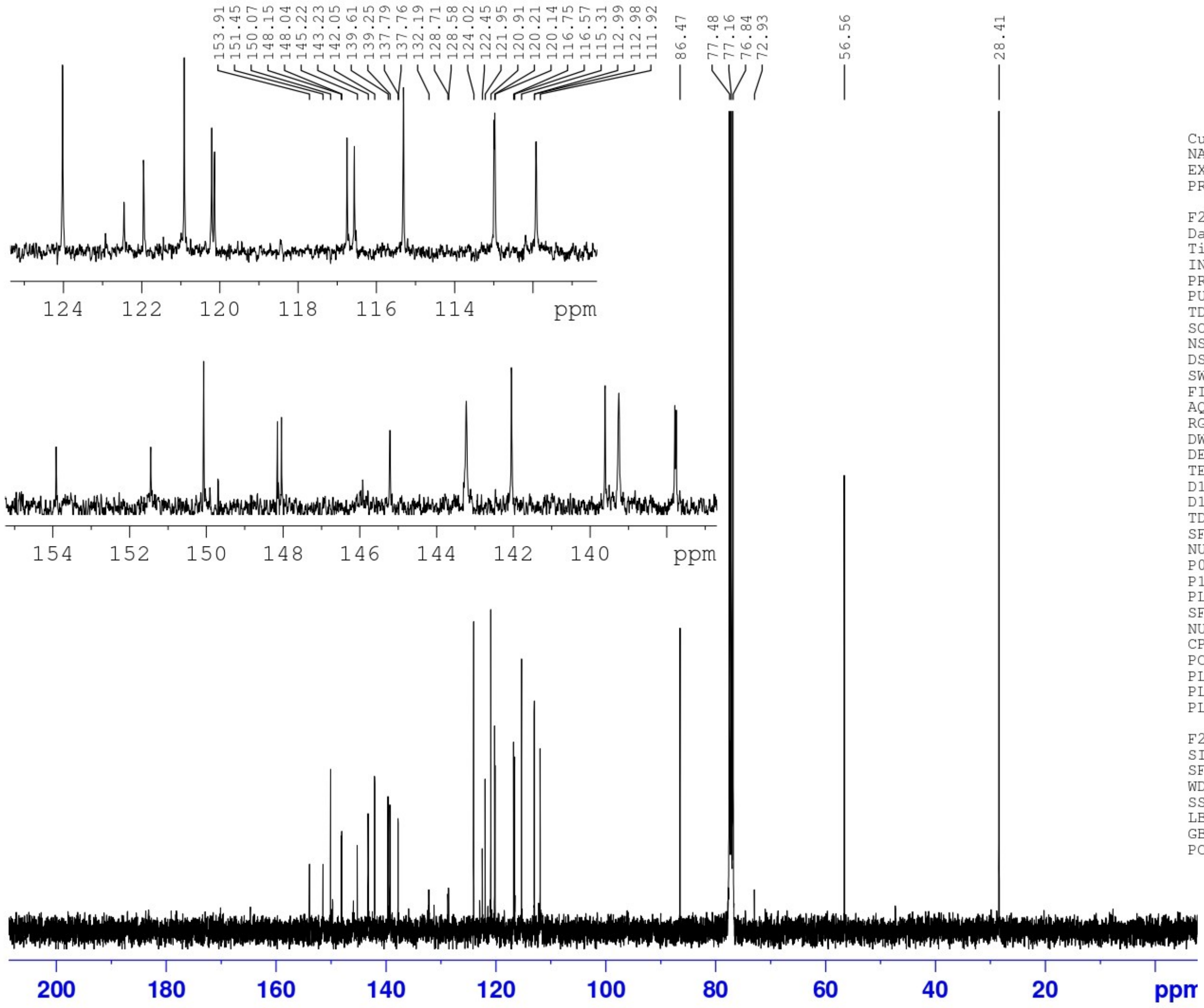

Current Data Parameters
NAME IX-Mn-144 i (P)_12
EXPNO 1
PROCNO 1

F2 - Acquisition Parameters

Date_	20251007
Time	21.11
INSTRUM	Avance
PROBHD	Z166552_0018 (PI HR-
PULPROG	zgpg30
TD	65536
SOLVENT	CDCl3
NS	2048
DS	4
SWH	23809.524
FIDRES	0.726609
AQ	1.3762560
RG	101
DW	21.000
DE	6.50
TE	298.0
D1	2.00000000
D11	0.03000000
TD0	1
SFO1	100.4744593
NUC1	13C
P0	2.67
P1	8.00
PLW1	88.22599792
SFO2	399.5415982
NUC2	1H
CPDPRG[2]	waltz65
PCPD2	90.00
PLW2	21.19799995
PLW12	0.15922000
PLW13	0.08008700

F2 - Processing parameters

SI	32768
SF	100.4644000
WDW	EM
SSB	0
LB	1.00
GB	0
PC	1.40

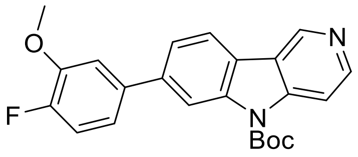

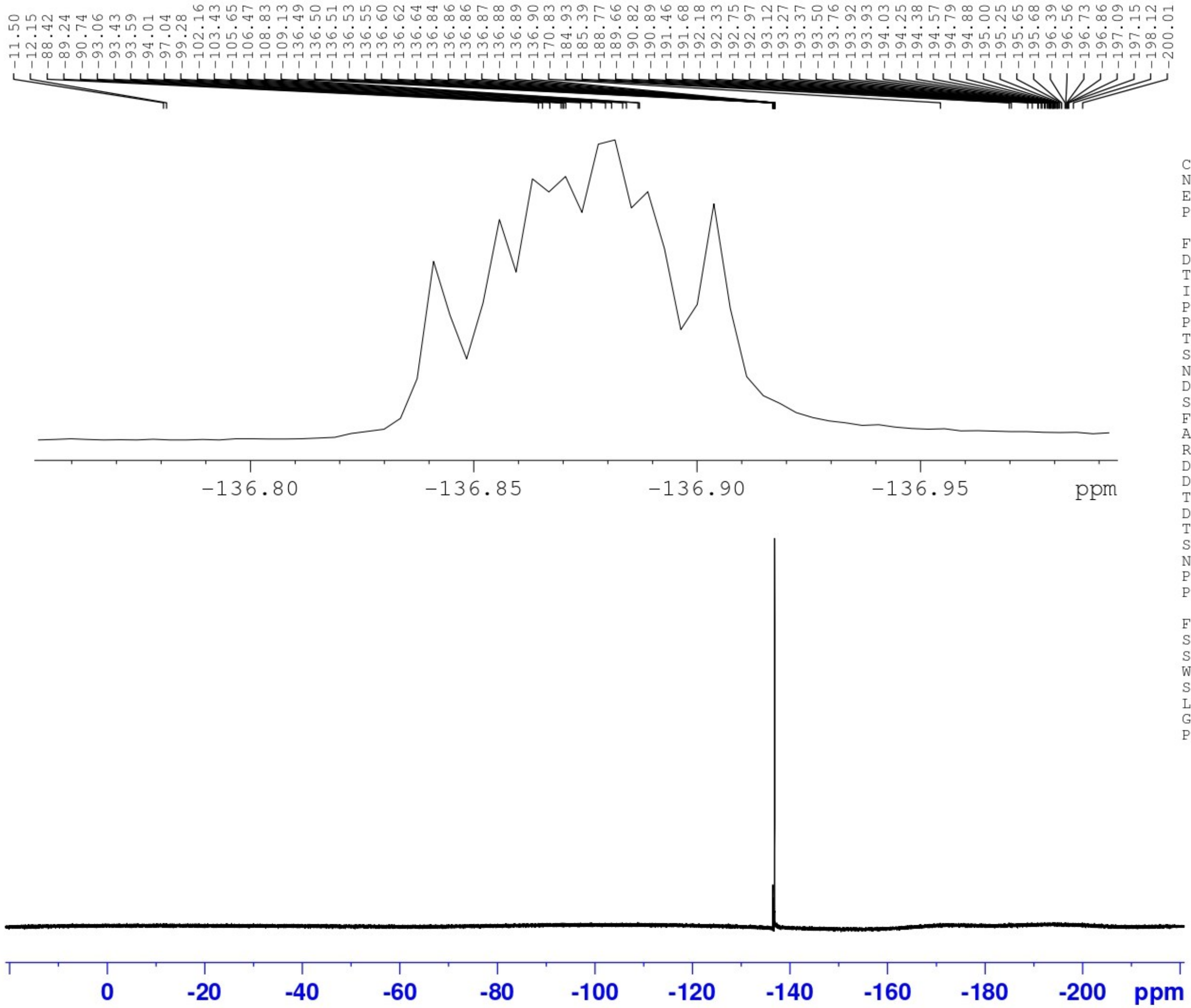

BRUKER

Current Data Parameters
NAME IX-Mn-144 i_11
EXPNO 2
PROCNO 1

F2 - Acquisition Parameters
Date_ 20250821
Time 15.40
INSTRUM Avance
PROBHD Z166552_0018 (PI HR-
PULPROG zg
TD 131072
SOLVENT CDCl3
NS 16
DS 4
SWH 90909.091
FIDRES 1.387163
AQ 0.7208960
RG 101
DW 5.500
DE 6.50
TE 298.0
D1 1.00000000
TD0 1
SFO1 375.9056172
NUC1 19F
P1 12.00
PLW1 32.47200012

F2 - Processing parameters
SI 65536
SF 375.9432115
WDW EM
SSB 0
LB 0.30
GB 0
PC 1.00

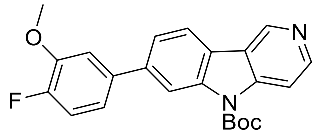

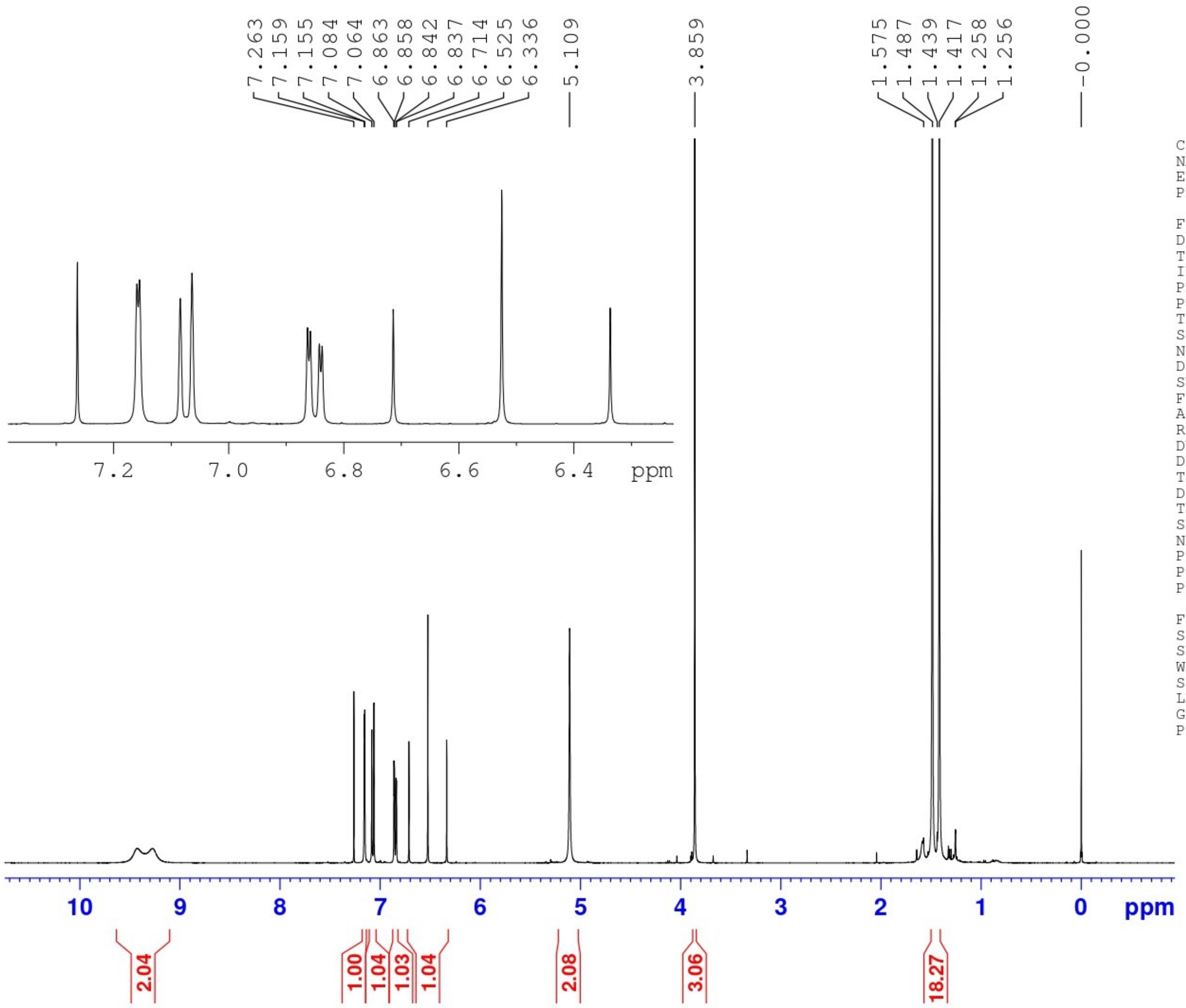


```
Current Data Parameters
NAME                  VI-Mn-04 i_10
EXPNO                             2
PROCNO                            1

F2 - Acquisition Parameters
Date_                      20240802
Time                          16.59
INSTRUM                      Avance
PROBHD      Z166552_0018 (PI HR-
PULPROG                        zg30
TD                            65536
SOLVENT                       CDCl3
NS                               16
DS                                2
SWH                        7812.500
FIDRES                     0.238419
AQ                        4.1943040
RG                              101
DW                           64.000
DE                             6.67
TE                            298.0
D1                       1.00000000
TD0                               1
SFO1                    399.5701703
NUC1                             1H
P0                             2.60
P1                             7.80
PLW1                    21.19799995

F2 - Processing parameters
SI                            65536
SF                      399.5677114
WDW                              EM
SSB                               0
LB                             0.30
GB                                0
PC                             1.00
```

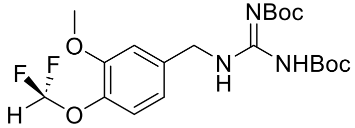

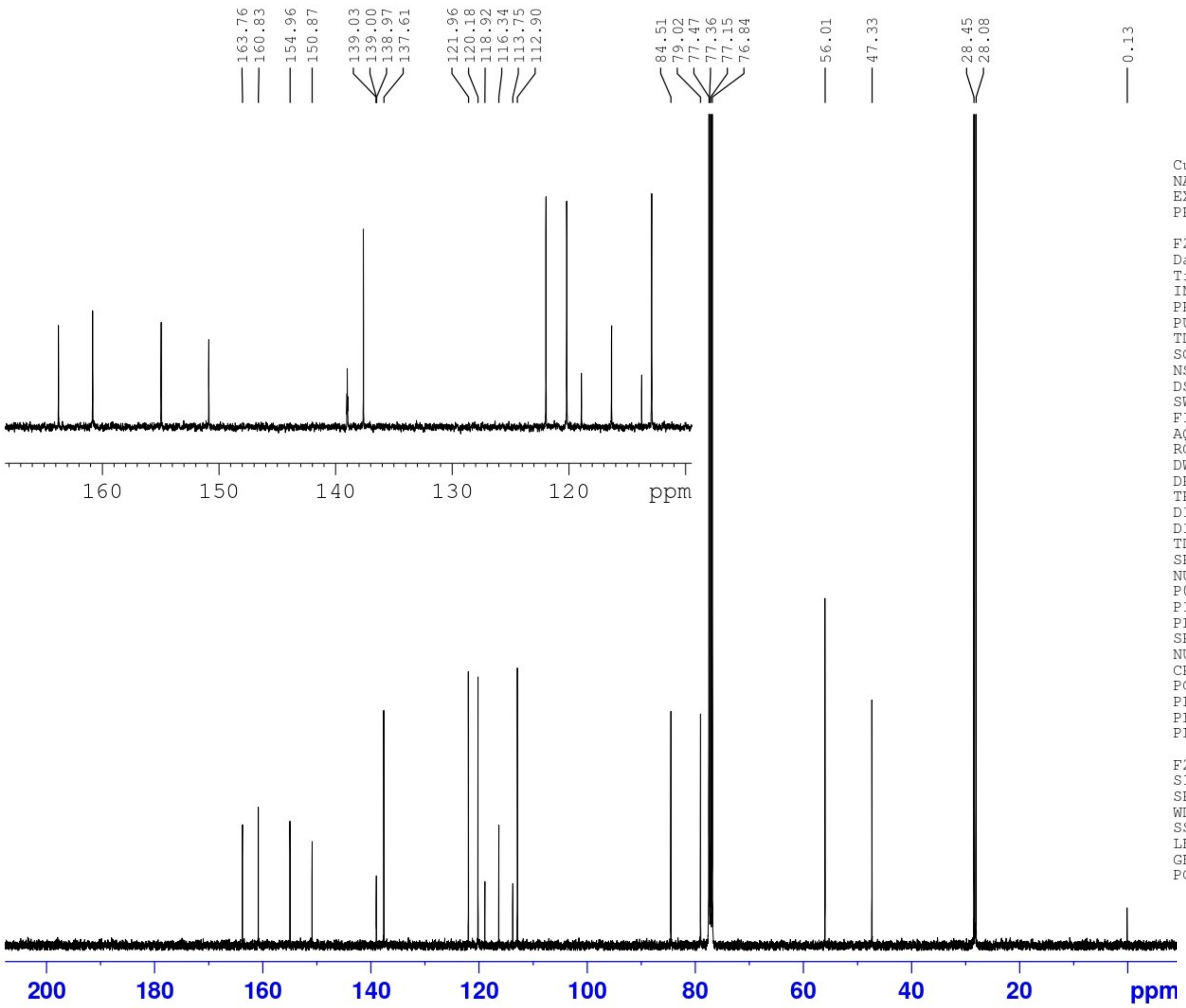

BRUKER

Current Data Parameters
NAME VI-Mn-04 i_12
EXPNO 2
PROCNO 1

F2 - Acquisition Parameters
Date_ 20240803
Time 1.00
INSTRUM Avance
PROBHD Z166552_0018 (PI HR-
PULPROG zgpg30
TD 65536
SOLVENT CDCl3
NS 2048
DS 4
SWH 23809.524
FIDRES 0.726609
AQ 1.3762560
RG 101
DW 21.000
DE 6.50
TE 298.0
D1 2.00000000
D11 0.03000000
TD0 1
SFO1 100.4814260
NUC1 13C
P0 2.67
P1 8.00
PLW1 88.22599792
SFO2 399.5693013
NUC2 1H
CPDPRG[2] waltz65
PCPD2 90.00
PLW2 21.19799995
PLW12 0.15922000
PLW13 0.08008700

F2 - Processing parameters
SI 32768
SF 100.4713657
WDW EM
SSB 0
LB 1.00
GB 0
PC 1.40

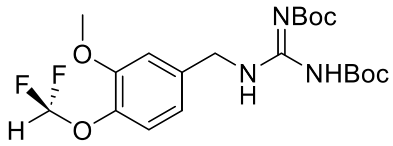

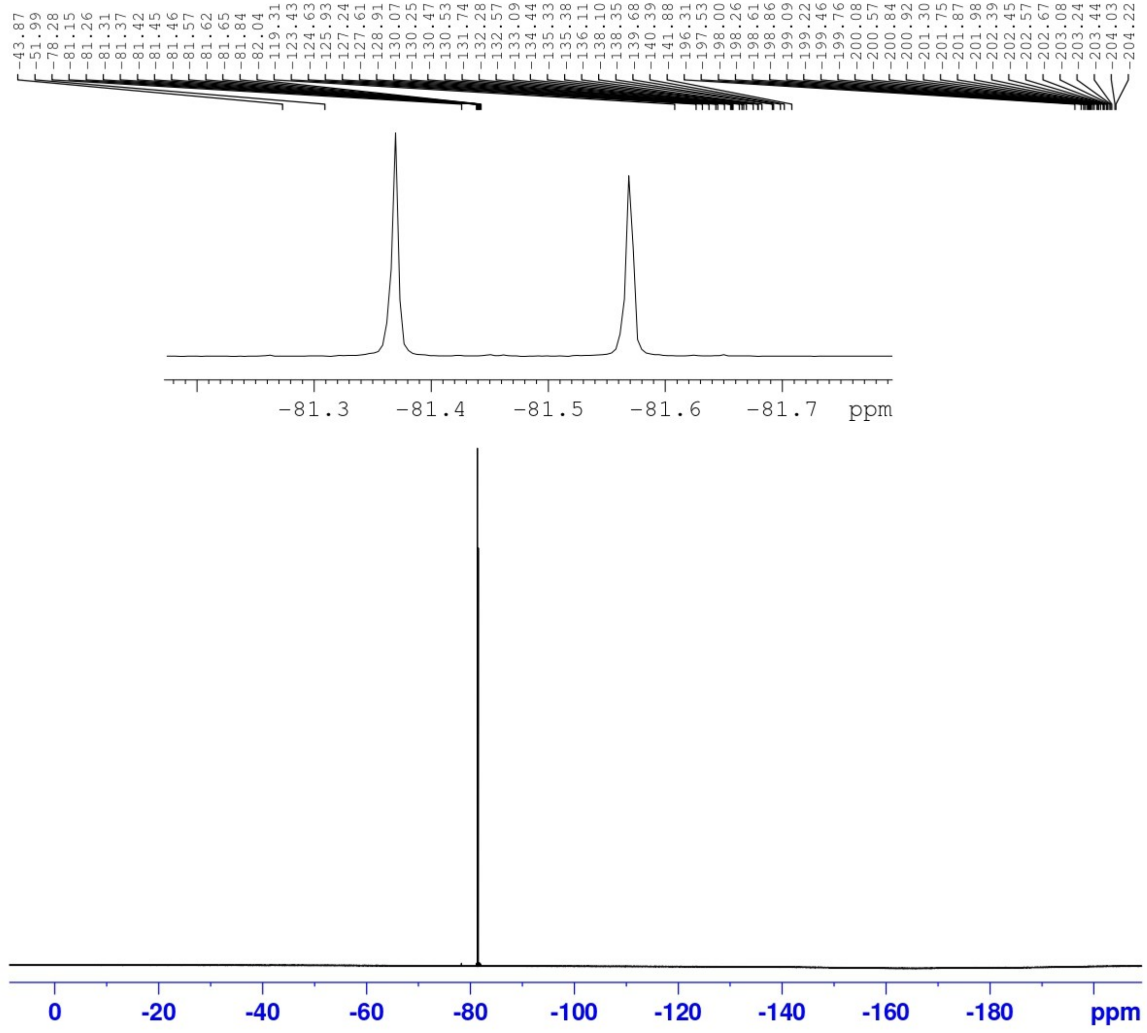

Current Data Parameters
NAME VI-Mn-04 i_11
EXPNO 2
PROCNO 1

F2 - Acquisition Parameters
Date_ 20240802
Time 17.02
INSTRUM Avance
PROBHD Z166552_0018 (PI HR-
PULPROG zg
TD 131072
SOLVENT CDCl3
NS 16
DS 4
SWH 90909.091
FIDRES 1.387163
AQ 0.7208960
RG 101
DW 5.500
DE 6.50
TE 298.0
D1 1.00000000
TD0 1
SFO1 375.9316815
NUC1 19F
P1 12.00
PLW1 32.47200012

F2 - Processing parameters
SI 65536
SF 375.9692784
WDW EM
SSB 0
LB 0.30
GB 0
PC 1.00

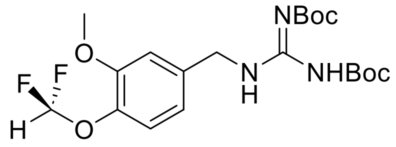

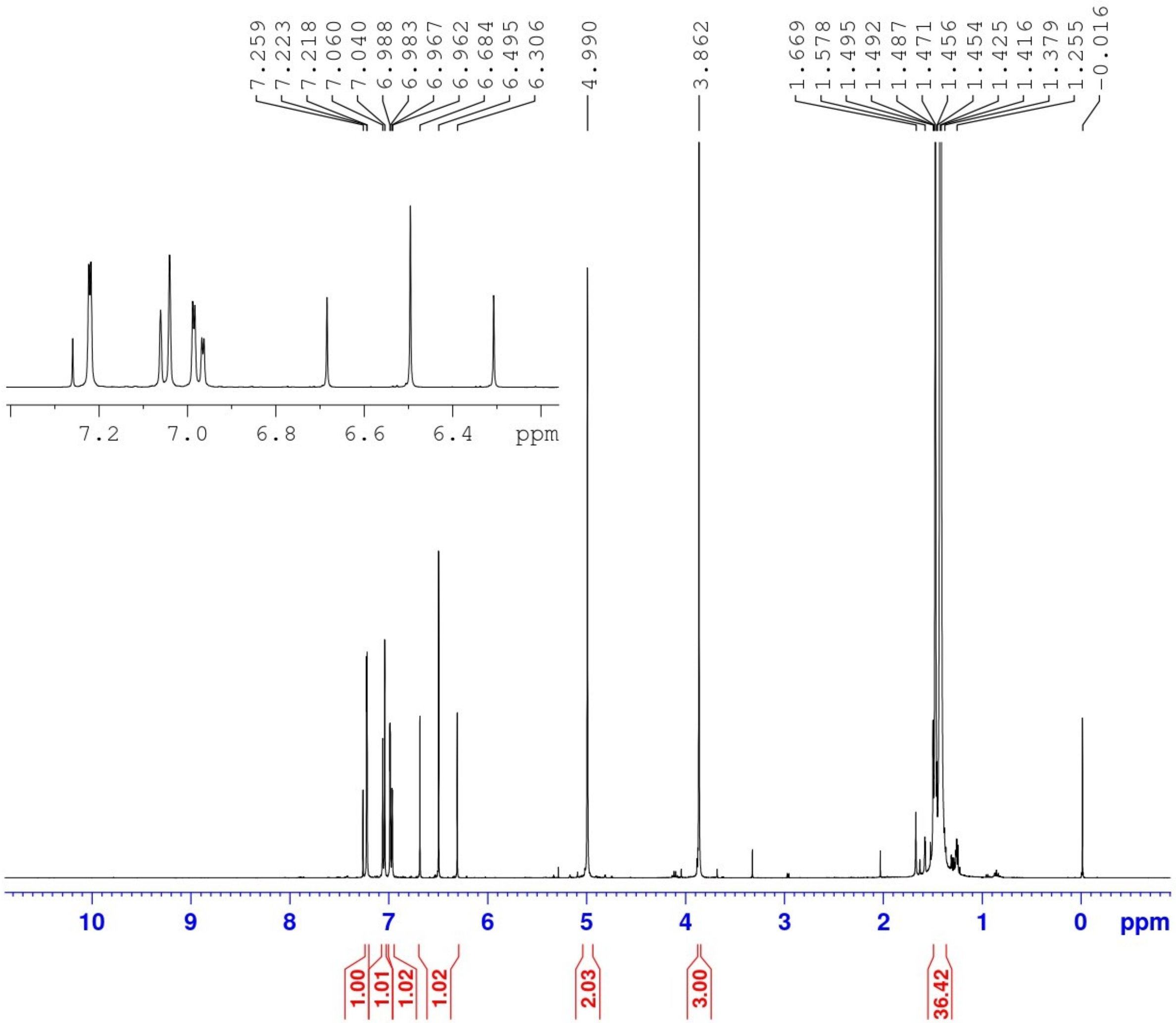

Current Data Parameters
NAME VI-Mn-06 i_10
EXPNO 3
PROCNO 1

F2 - Acquisition Parameters
Date_ 20240802
Time 17.09
INSTRUM Avance
PROBHD Z166552_0018 (PI HR-
PULPROG zg30
TD 65536
SOLVENT CDCl3
NS 16
DS 2
SWH 7812.500
FIDRES 0.238419
AQ 4.1943040
RG 45.2
DW 64.000
DE 6.67
TE 298.0
D1 1.00000000
TD0 1
SFO1 399.5701703
NUC1 1H
P0 2.60
P1 7.80
PLW1 21.19799995

F2 - Processing parameters
SI 65536
SF 399.5677127
WDW EM
SSB 0
LB 0.30
GB 0
PC 1.00

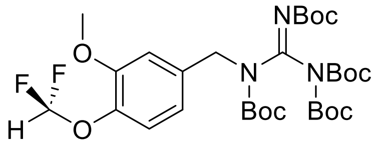

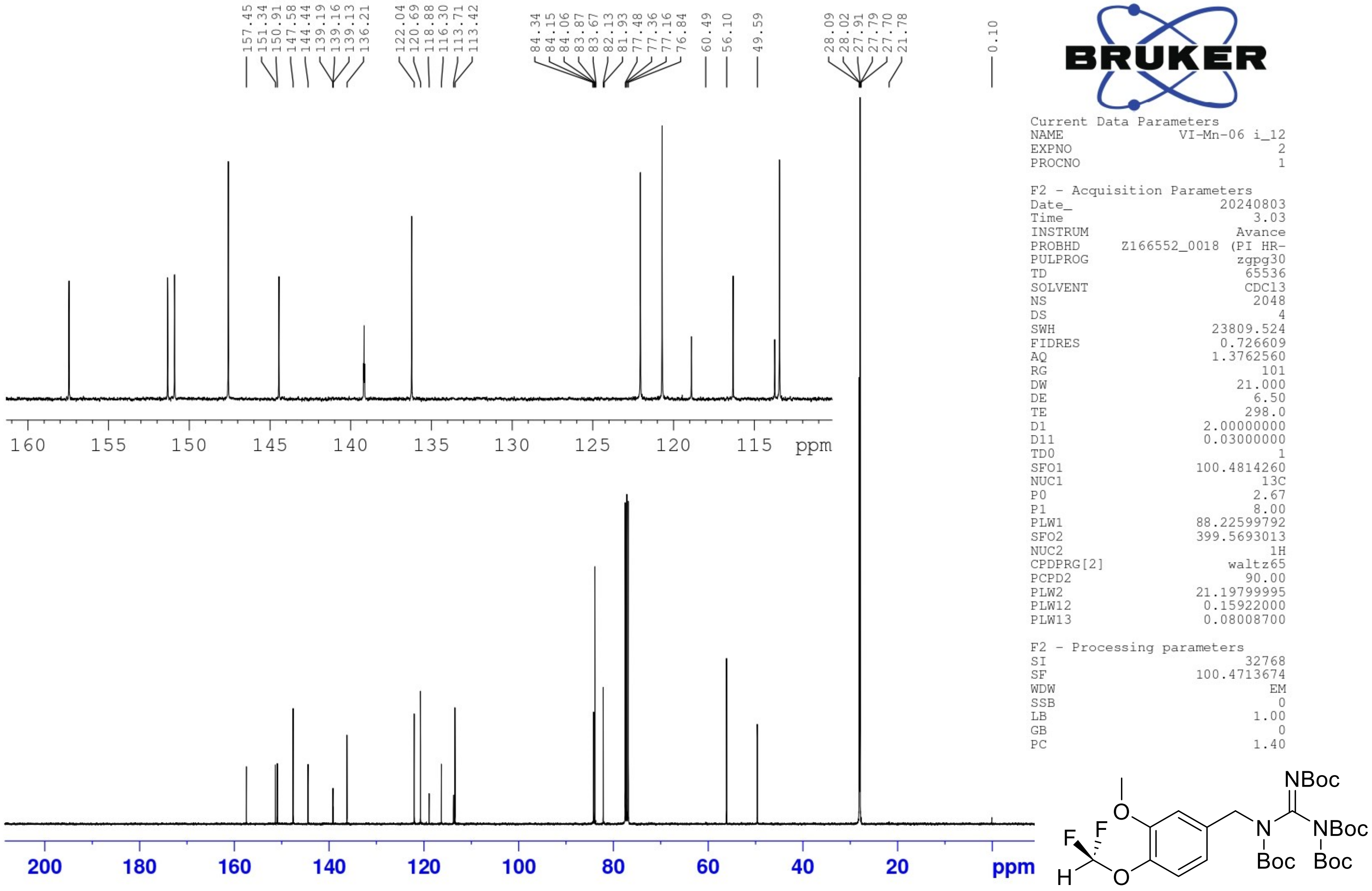
157.45
151.34
150.91
147.58
144.44
139.19
139.16
139.13
136.21
122.04
120.69
118.88
116.30
113.71
113.42
84.34
84.15
84.06
83.87
83.67
82.13
81.93
77.48
77.36
77.16
76.84
60.49
56.10
49.59
28.09
28.02
27.91
27.79
27.70
21.78
0.10
160 155 150 145 140 135 130 125 120 115 ppm
200 180 160 140 120 100 80 60 40 20 ppm
Current Data Parameters
NAME VI-Mn-06 i_12
EXPNO 2
PROCNO 1
F2 - Acquisition Parameters
Date_ 20240803
Time 3.03
INSTRUM Avance
PROBHD Z166552_0018 (PI HR-
PULPROG zgpg30
TD 65536
SOLVENT CDCl3
NS 2048
DS 4
SWH 23809.524
FIDRES 0.726609
AQ 1.3762560
RG 101
DW 21.000
DE 6.50
TE 298.0
D1 2.00000000
D11 0.03000000
TD0 1
SFO1 100.4814260
NUC1 13C
P0 2.67
P1 8.00
PLW1 88.22599792
SFO2 399.5693013
NUC2 1H
CPDPRG[2] waltz65
PCPD2 90.00
PLW2 21.19799995
PLW12 0.15922000
PLW13 0.08008700
F2 - Processing parameters
SI 32768
SF 100.4713674
WDW EM
SSB 0
LB 1.00
GB 0
PC 1.40
NBoc
O
F
F
N
NBoc
H
O
Boc
Boc

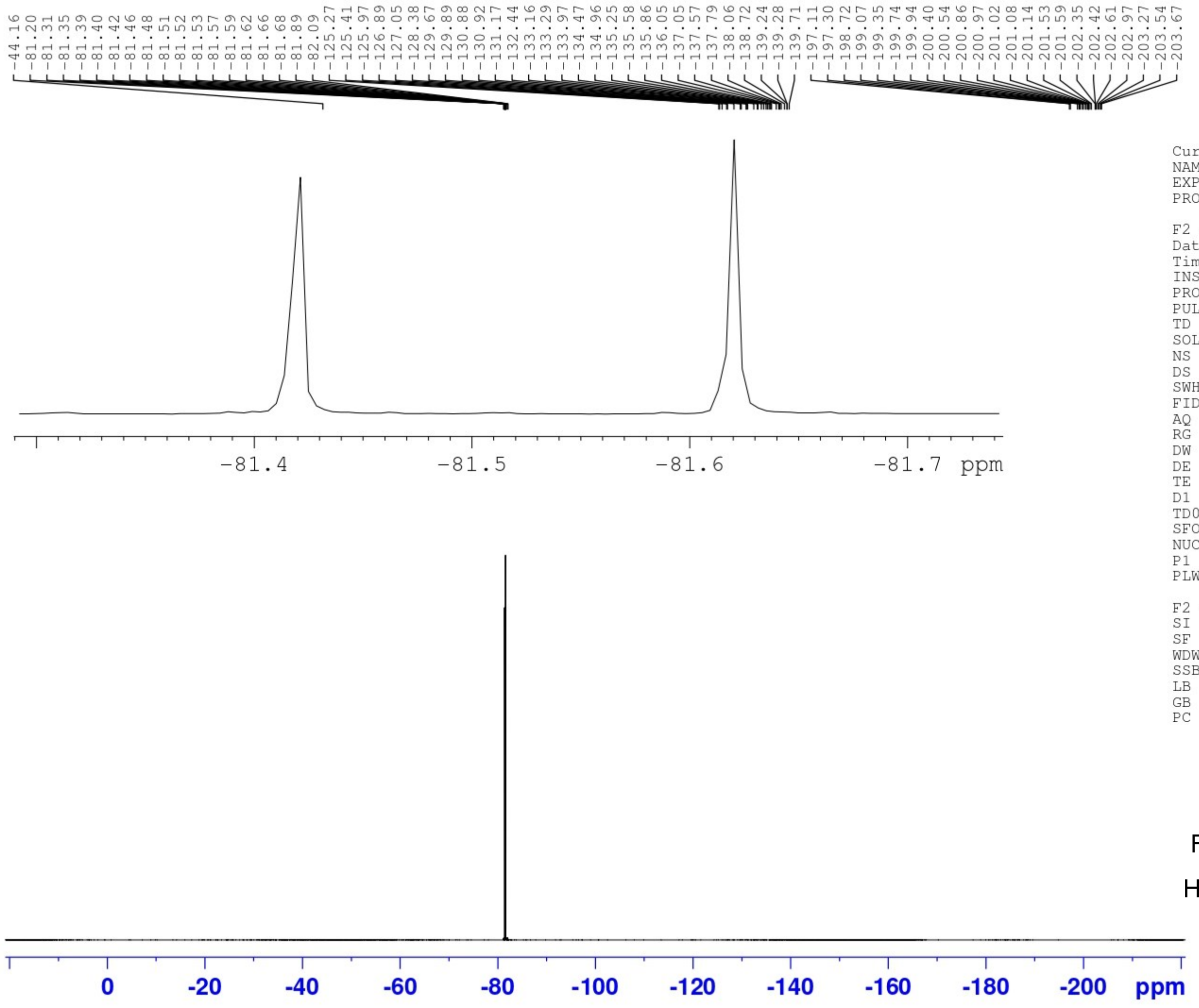

Current Data Parameters
NAME VI-Mn-06 i_11
EXPNO 2
PROCNO 1

F2 - Acquisition Parameters
Date_ 20240802
Time 17.11
INSTRUM Avance
PROBHD Z166552_0018 (PI HR-
PULPROG zg
TD 131072
SOLVENT CDCl3
NS 16
DS 4
SWH 90909.091
FIDRES 1.387163
AQ 0.7208960
RG 101
DW 5.500
DE 6.50
TE 298.0
D1 1.00000000
TD0 1
SFO1 375.9316815
NUC1 19F
P1 12.00
PLW1 32.47200012

F2 - Processing parameters
SI 65536
SF 375.9692784
WDW EM
SSB 0
LB 0.30
GB 0
PC 1.00

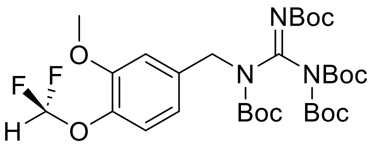

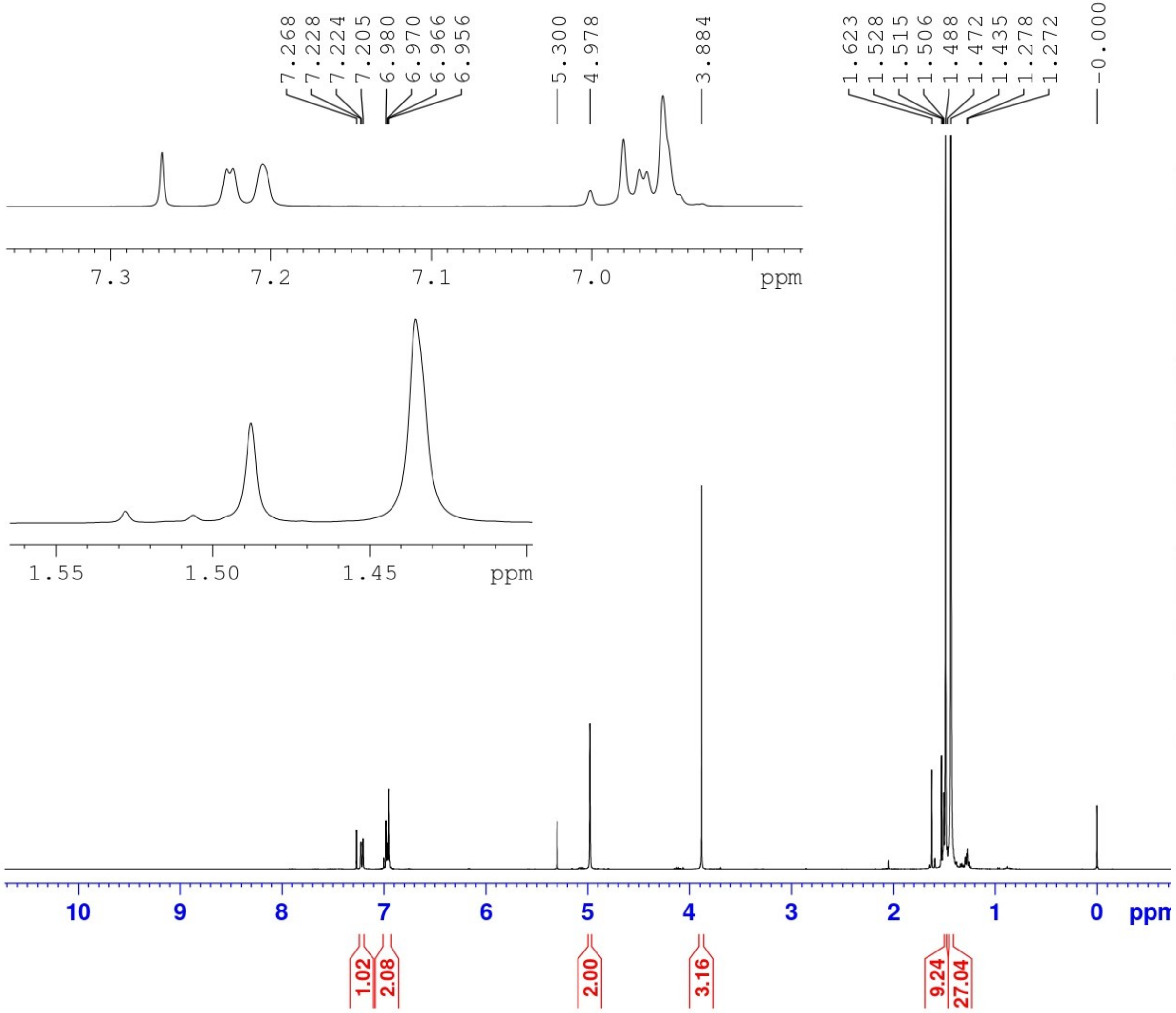

BRUKER

Current Data Parameters
NAME VI-Mn-48 i_10
EXPNO 2
PROCNO 1

F2 - Acquisition Parameters
Date_ 20240904
Time 17.11
INSTRUM Avance
PROBHD Z166552_0018 (PI HR-
PULPROG zg30
TD 65536
SOLVENT CDCl3
NS 16
DS 2
SWH 7812.500
FIDRES 0.238419
AQ 4.1943040
RG 101
DW 64.000
DE 6.67
TE 298.0
D1 1.00000000
TD0 1
SFO1 399.5701703
NUC1 1H
P0 2.60
P1 7.80
PLW1 21.19799995

F2 - Processing parameters
SI 65536
SF 399.5677094
WDW EM
SSB 0
LB 0.30
GB 0
PC 1.00

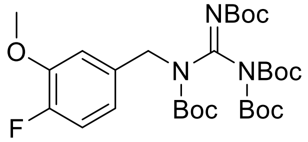

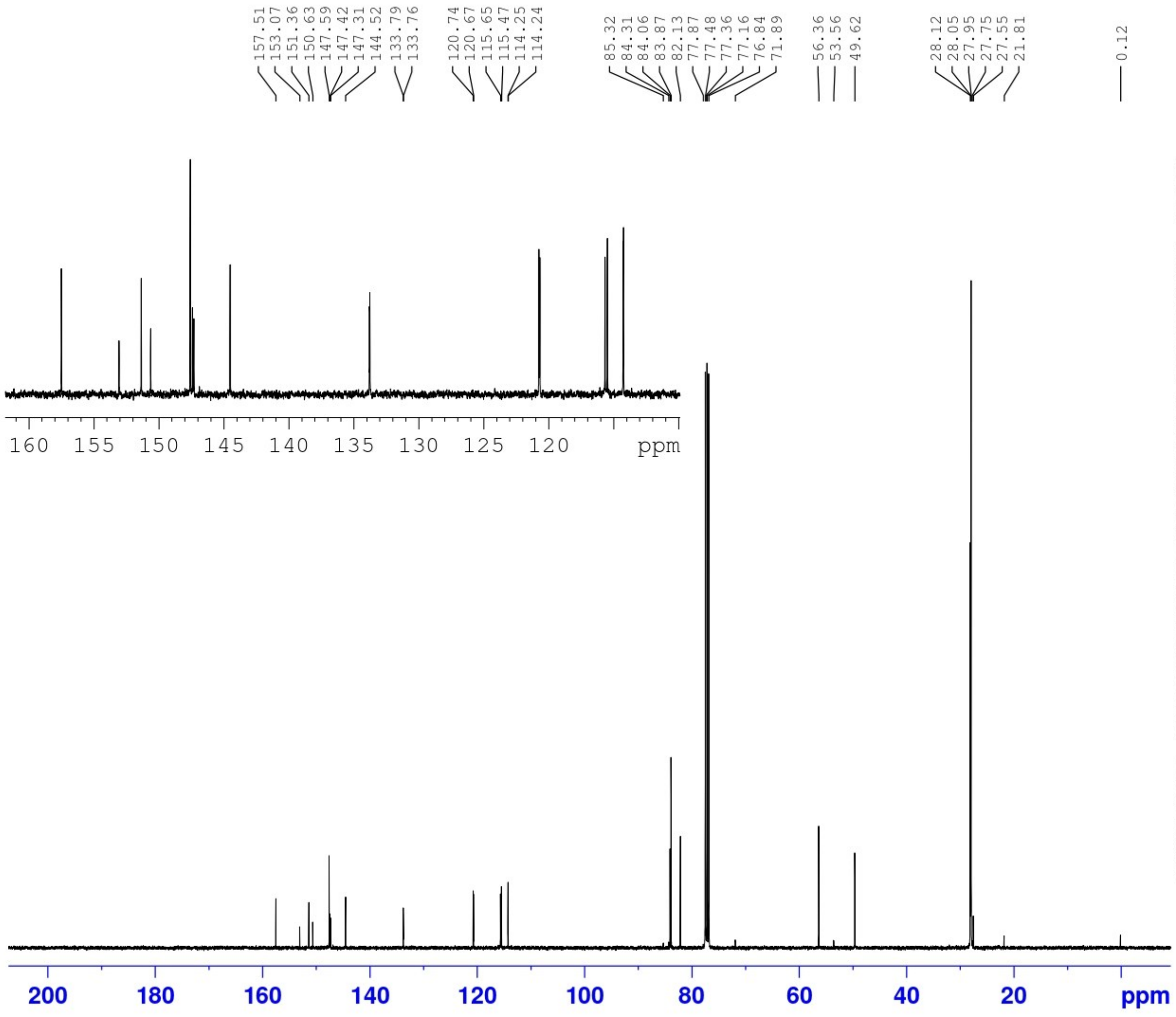

Current Data Parameters	
NAME	VI-Mn-48 i_12
EXPNO	2
PROCNO	1
F2 - Acquisition Parameters	
Date_	20240905
Time	1.55
INSTRUM	Avance
PROBHD	Z166552_0018 (PI HR-
PULPROG	zgpg30
TD	65536
SOLVENT	CDCl3
NS	2048
DS	4
SWH	23809.524
FIDRES	0.726609
AQ	1.3762560
RG	101
DW	21.000
DE	6.50
TE	298.0
D1	2.00000000
D11	0.03000000
TD0	1
SFO1	100.4814260
NUC1	13C
P0	2.67
P1	8.00
PLW1	88.22599792
SFO2	399.5693013
NUC2	1H
CPDPRG[2]	waltz65
PCPD2	90.00
PLW2	21.19799995
PLW12	0.15922000
PLW13	0.08008700
F2 - Processing parameters	
SI	32768
SF	100.4713659
WDW	EM
SSB	0
LB	1.00
GB	0
PC	1.40

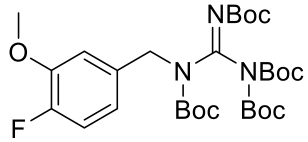

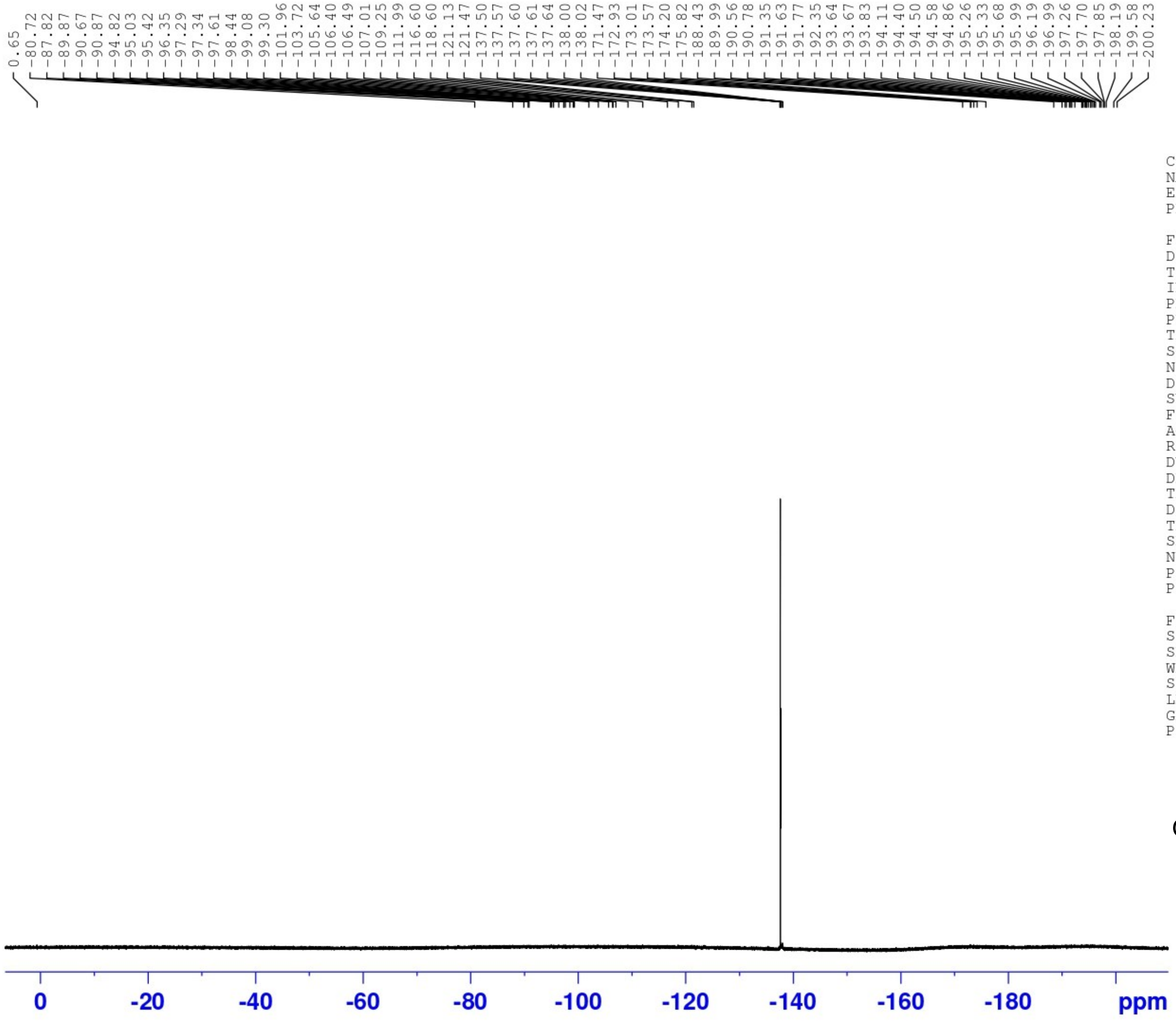

Current Data Parameters

NAME	VI-Mn-48 i_11
EXPNO	2
PROCNO	1

F2 - Acquisition Parameters

Date_	20240904
Time	17.13
INSTRUM	Avance
PROBHD	Z166552_0018 (PI HR-
PULPROG	zg
TD	131072
SOLVENT	CDCl3
NS	16
DS	4
SWH	90909.091
FIDRES	1.387163
AQ	0.7208960
RG	101
DW	5.500
DE	6.50
TE	298.0
D1	1.00000000
TD0	1
SFO1	375.9316815
NUC1	19F
P1	12.00
PLW1	32.47200012

F2 - Processing parameters

SI	65536
SF	375.9692784
WDW	EM
SSB	0
LB	0.30
GB	0
PC	1.00

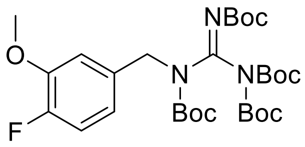

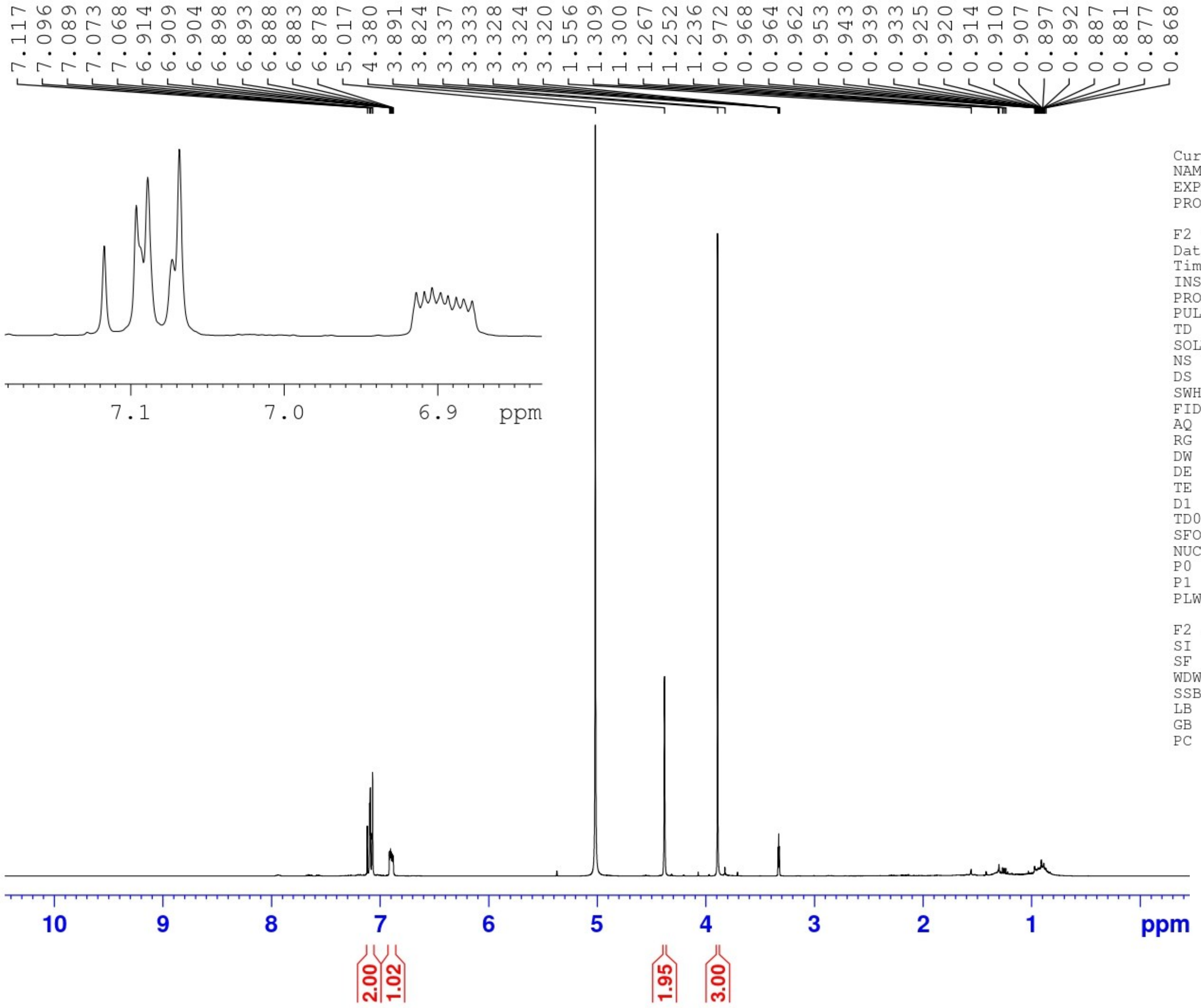

Current Data Parameters
NAME X-Mn-38_10
EXPNO 1
PROCNO 1

F2 - Acquisition Parameters
Date_ 20251031
Time 19.11
INSTRUM Avance
PROBHD Z166552_0018 (PI HR-
PULPROG zg30
TD 65536
SOLVENT MeOD
NS 16
DS 2
SWH 7812.500
FIDRES 0.238419
AQ 4.1943040
RG 90.5
DW 64.000
DE 6.67
TE 298.0
D1 1.00000000
TD0 1
SFO1 399.5424672
NUC1 1H
P0 2.60
P1 7.80
PLW1 21.19799995

F2 - Processing parameters
SI 65536
SF 399.5400000
WDW EM
SSB 0
LB 0.30
GB 0
PC 1.00

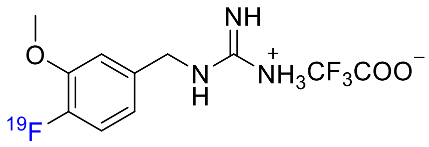

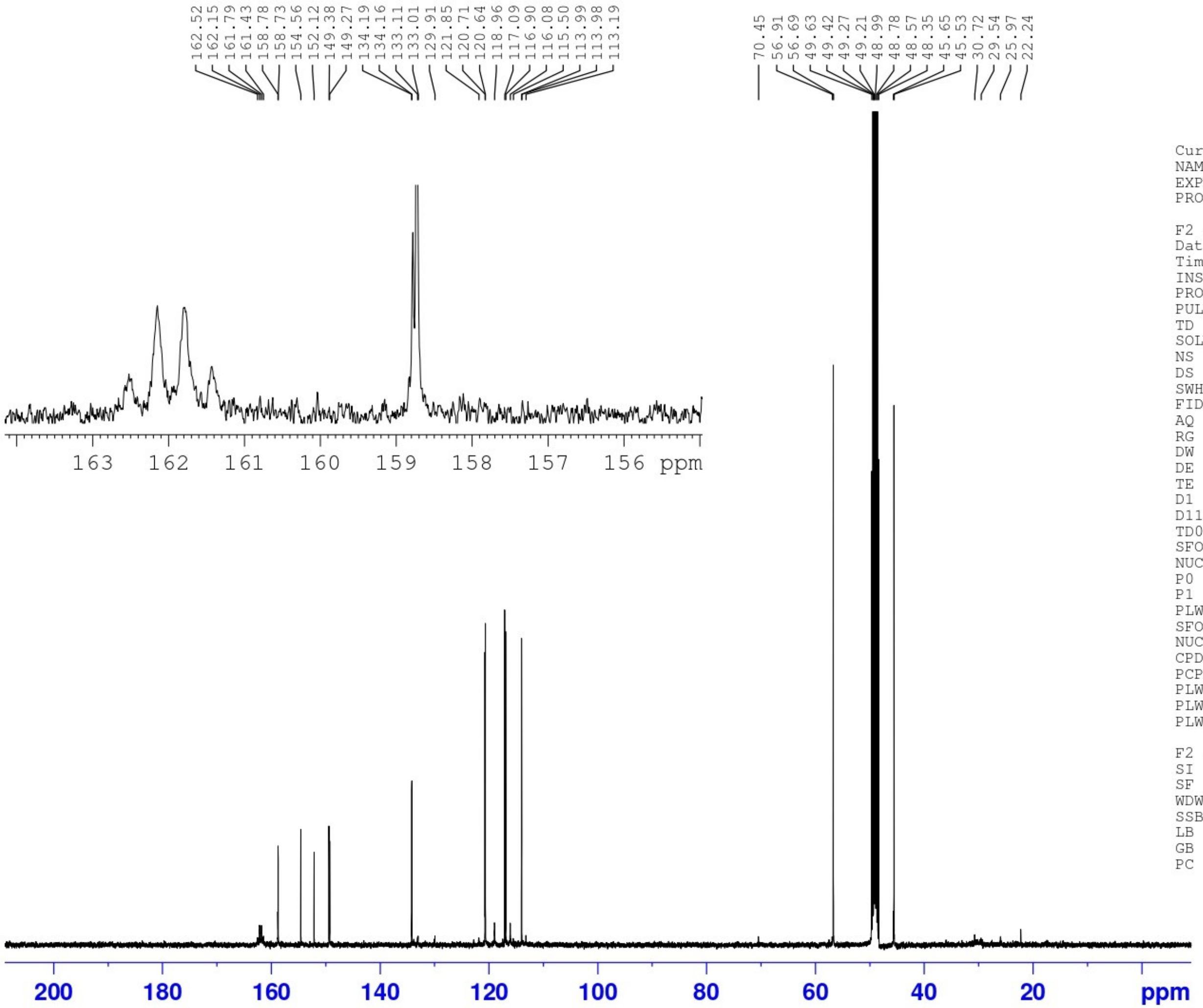

Current Data Parameters
NAME X-Mn-38_12
EXPNO 2
PROCNO 1

F2 - Acquisition Parameters
Date_ 20251031
Time 21.12
INSTRUM Avance
PROBHD Z166552_0018 (PI HR-
PULPROG zgpg30
TD 65536
SOLVENT MeOD
NS 2048
DS 4
SWH 23809.524
FIDRES 0.726609
AQ 1.3762560
RG 101
DW 21.000
DE 6.50
TE 298.0
D1 2.00000000
D11 0.03000000
TD0 1
SFO1 100.4744593
NUC1 13C
P0 2.67
P1 8.00
PLW1 88.22599792
SFO2 399.5415982
NUC2 1H
CPDPRG[2] waltz65
PCPD2 90.00
PLW2 21.19799995
PLW12 0.15922000
PLW13 0.08008700

F2 - Processing parameters
SI 32768
SF 100.4642741
WDW EM
SSB 0
LB 1.00
GB 0
PC 1.40

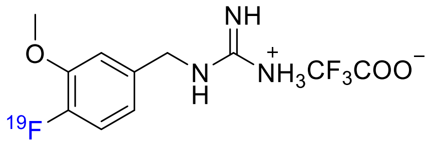

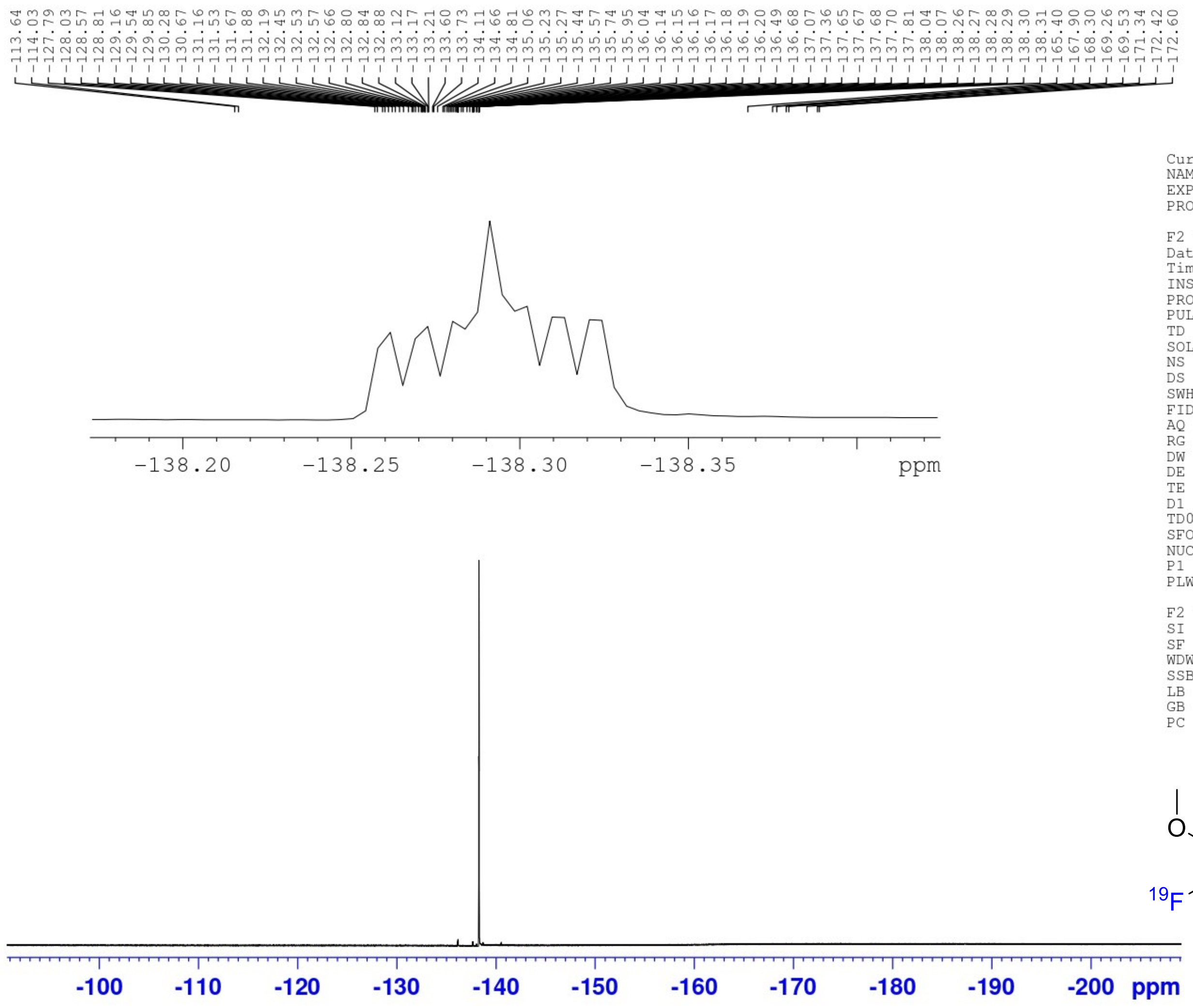

BRUKER

Current Data Parameters
NAME X-Mn-38_11
EXPNO 1
PROCNO 1

F2 - Acquisition Parameters
Date_ 20251031
Time 19.13
INSTRUM Avance
PROBHD Z166552_0018 (PI HR-
PULPROG zg
TD 131072
SOLVENT MeOD
NS 16
DS 4
SWH 90909.091
FIDRES 1.387163
AQ 0.7208960
RG 101
DW 5.500
DE 6.50
TE 298.0
D1 1.00000000
TD0 1
SFO1 375.9056172
NUC1 19F
P1 12.00
PLW1 32.47200012

F2 - Processing parameters
SI 65536
SF 375.9432115
WDW EM
SSB 0
LB 0.30
GB 0
PC 1.00

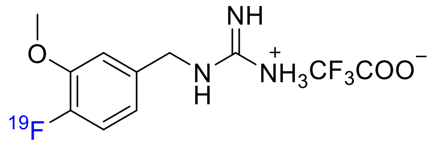

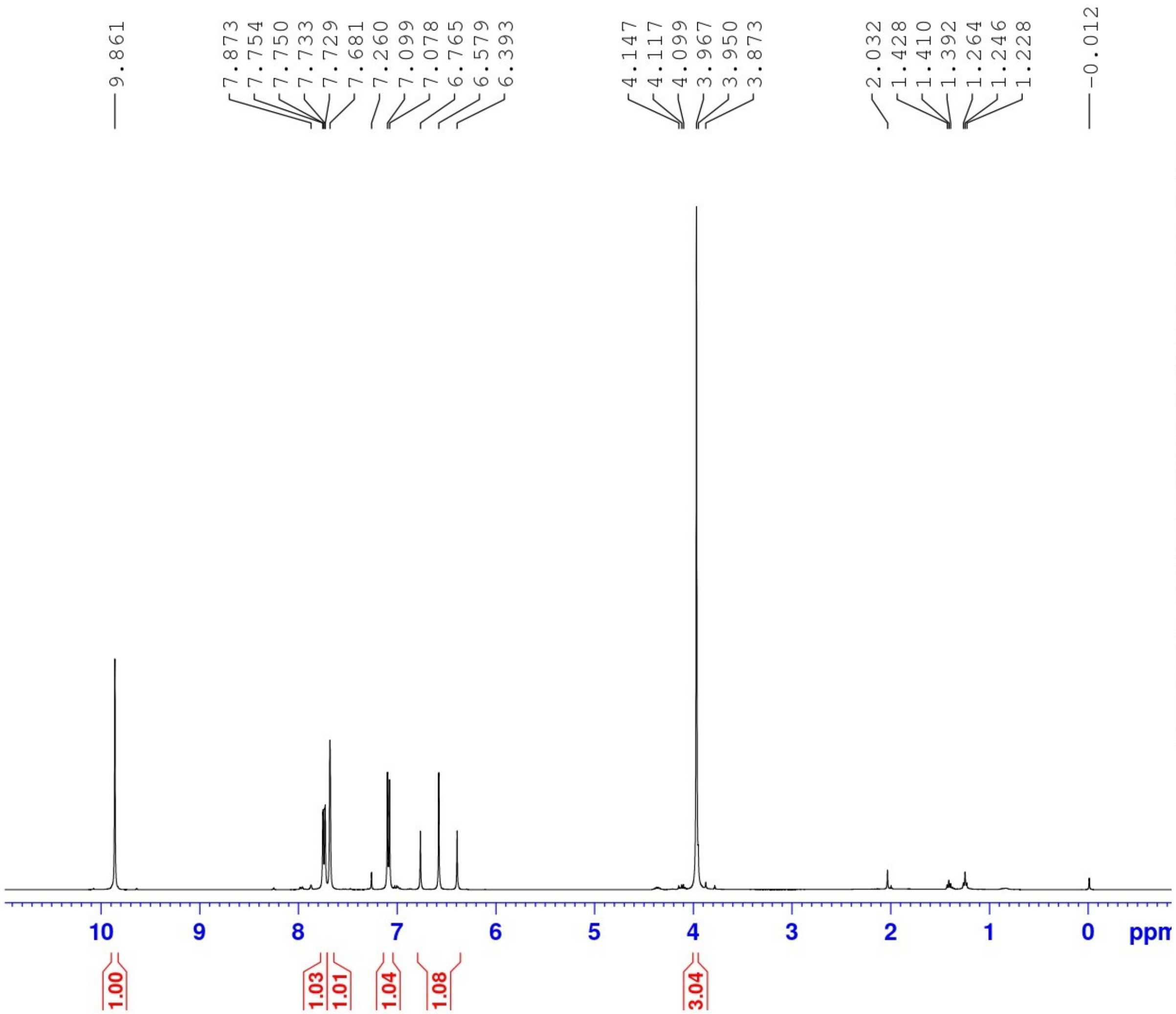

Current Data Parameters
NAME VI-Mn-42 i_10
EXPNO 2
PROCNO 1

F2 - Acquisition Parameters

Date_	20240829
Time	10.17
INSTRUM	Avance
PROBHD	Z166552_0018 (PI HR-
PULPROG	zg30
TD	65536
SOLVENT	CDCl3
NS	16
DS	2
SWH	7812.500
FIDRES	0.238419
AQ	4.1943040
RG	101
DW	64.000
DE	6.67
TE	298.0
D1	1.00000000
TD0	1
SFO1	399.5701703
NUC1	1H
P0	2.60
P1	7.80
PLW1	21.19799995

F2 - Processing parameters

SI	65536
SF	399.5677123
WDW	EM
SSB	0
LB	0.30
GB	0
PC	1.00

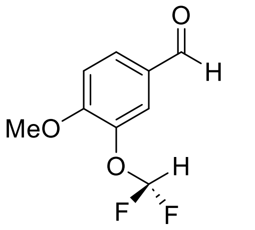

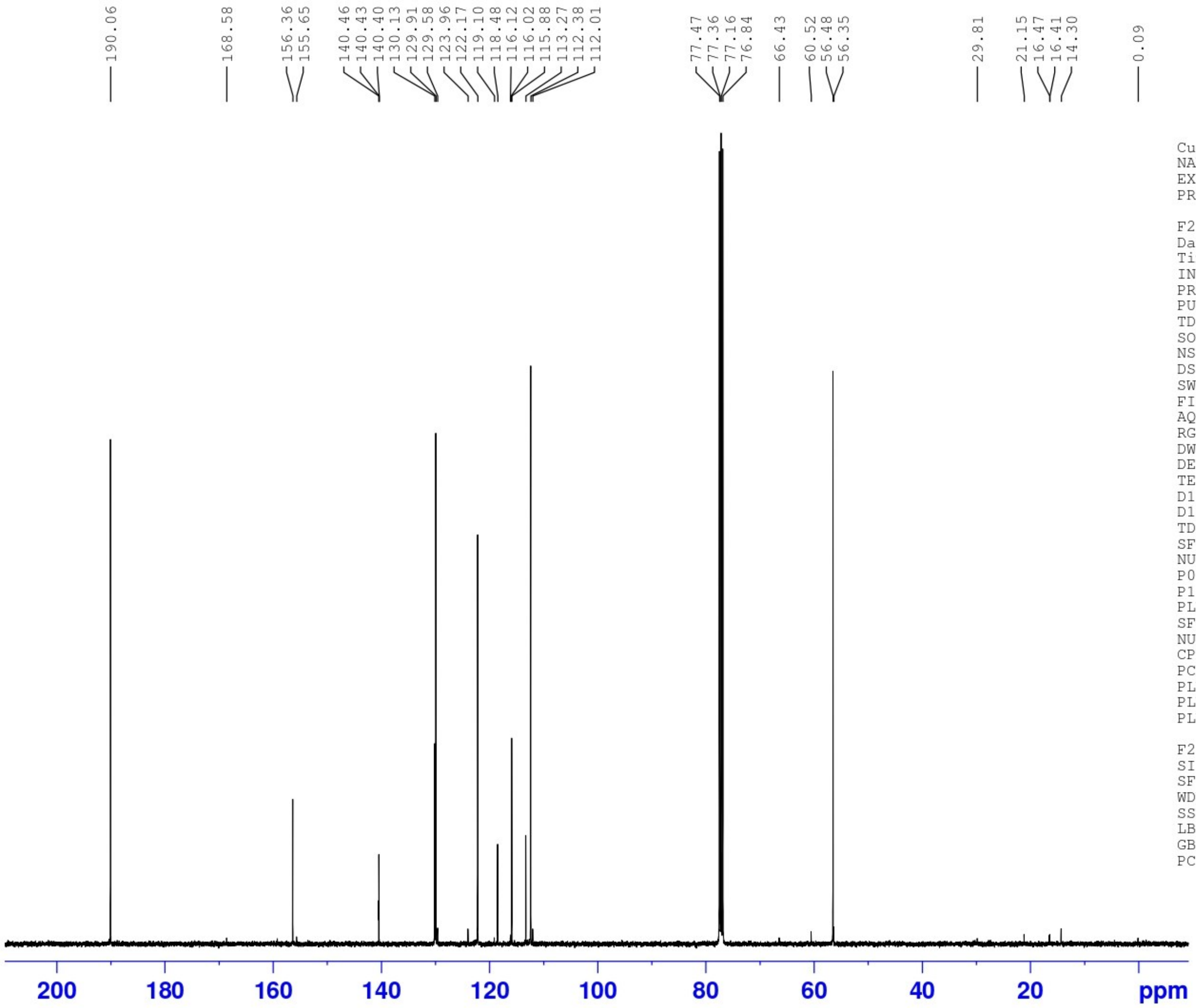

Current Data Parameters
NAME VI-Mn-42 i_12
EXPNO 2
PROCNO 1

F2 - Acquisition Parameters
Date_ 20240830
Time 4.08
INSTRUM Avance
PROBHD Z166552_0018 (PI HR-
PULPROG zgpg30
TD 65536
SOLVENT CDCl3
NS 2048
DS 4
SWH 23809.524
FIDRES 0.726609
AQ 1.3762560
RG 101
DW 21.000
DE 6.50
TE 298.0
D1 2.00000000
D11 0.03000000
TD0 1
SFO1 100.4814260
NUC1 13C
P0 2.67
P1 8.00
PLW1 88.22599792
SFO2 399.5693013
NUC2 1H
CPDPRG[2] waltz65
PCPD2 90.00
PLW2 21.19799995
PLW12 0.15922000
PLW13 0.08008700

F2 - Processing parameters
SI 32768
SF 100.4713672
WDW EM
SSB 0
LB 1.00
GB 0
PC 1.40

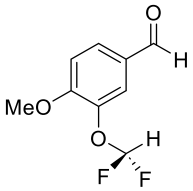

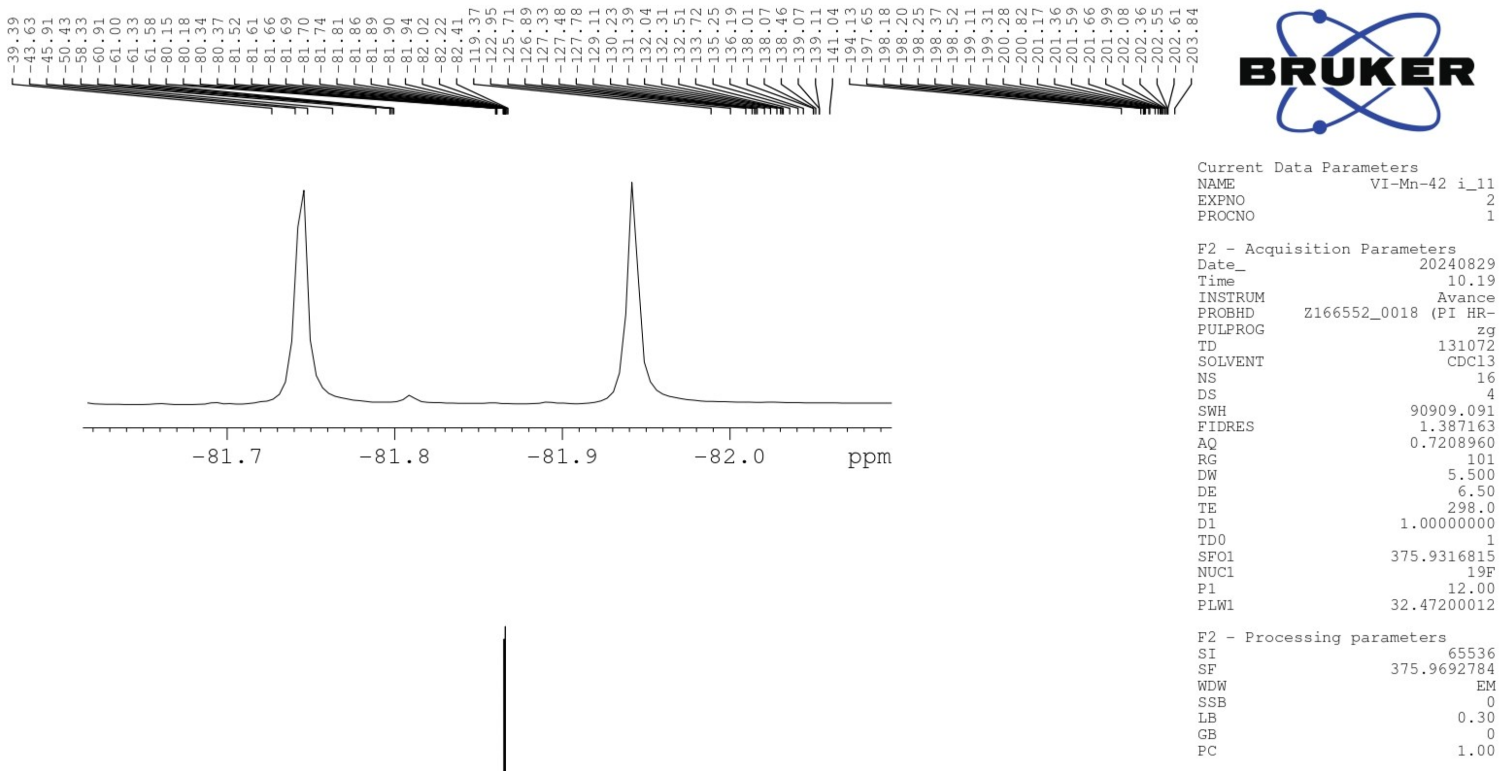
-39.39
-43.63
-45.91
-50.43
-58.33
-60.91
-61.00
-61.33
-61.58
-80.15
-80.18
-80.34
-80.37
-81.52
-81.61
-81.66
-81.69
-81.70
-81.74
-81.81
-81.86
-81.89
-81.90
-81.94
-82.02
-82.22
-82.41
-119.37
-122.95
-125.71
-126.89
-127.33
-127.48
-127.78
-129.11
-130.23
-131.39
-132.04
-132.31
-132.51
-133.72
-135.25
-136.19
-138.01
-138.07
-138.46
-139.07
-139.11
-141.04
-194.13
-197.65
-198.18
-198.20
-198.25
-198.37
-198.52
-199.11
-199.31
-200.28
-200.82
-201.17
-201.36
-201.59
-201.66
-201.99
-202.08
-202.36
-202.55
-202.61
-203.84
-81.7
-81.8
-81.9
-82.0
ppm
0
-20
-40
-60
-80
-100
-120
-140
-160
-180
ppm
Current Data Parameters
NAME VI-Mn-42 i_11
EXPNO 2
PROCNO 1
F2 - Acquisition Parameters
Date_ 20240829
Time 10.19
INSTRUM Avance
PROBHD Z166552_0018 (PI HR-
PULPROG zg
TD 131072
SOLVENT CDCl3
NS 16
DS 4
SWH 90909.091
FIDRES 1.387163
AQ 0.7208960
RG 101
DW 5.500
DE 6.50
TE 298.0
D1 1.00000000
TD0 1
SFO1 375.9316815
NUC1 19F
P1 12.00
PLW1 32.47200012
F2 - Processing parameters
SI 65536
SF 375.9692784
WDW EM
SSB 0
LB 0.30
GB 0
PC 1.00

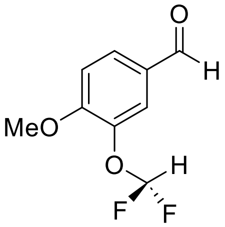
O
H
MeO
O
H
F
F

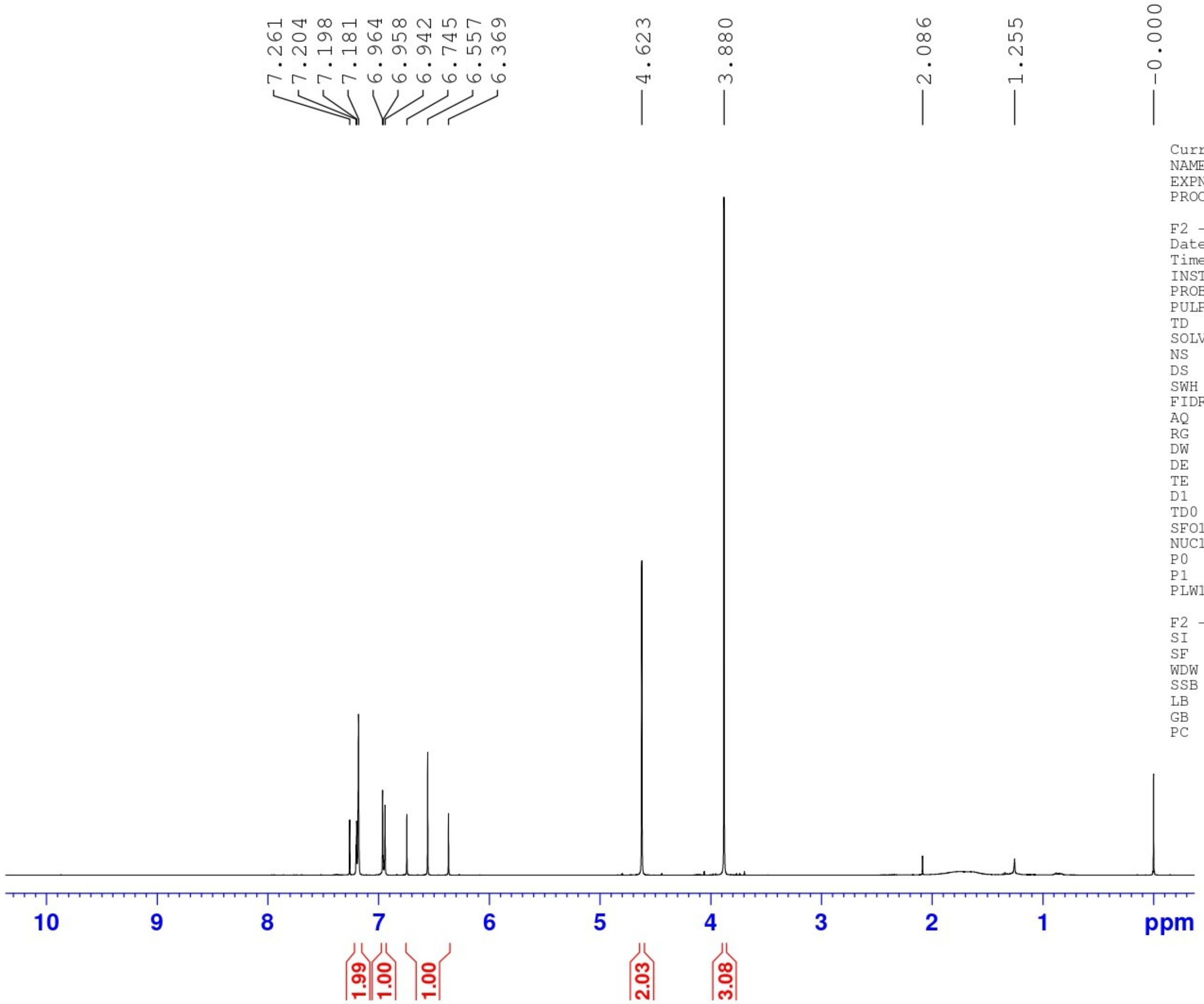

Current Data Parameters
NAME VI-Mn-43_10
EXPNO 3
PROCNO 1

F2 - Acquisition Parameters
Date_ 20240903
Time 11.38
INSTRUM Avance
PROBHD Z166552_0018 (PI HR-
PULPROG zg30
TD 65536
SOLVENT CDCl3
NS 16
DS 2
SWH 7812.500
FIDRES 0.238419
AQ 4.1943040
RG 101
DW 64.000
DE 6.67
TE 298.0
D1 1.00000000
TD0 1
SFO1 399.5701703
NUC1 1H
P0 2.60
P1 7.80
PLW1 21.19799995

F2 - Processing parameters
SI 65536
SF 399.5677121
WDW EM
SSB 0
LB 0.30
GB 0
PC 1.00

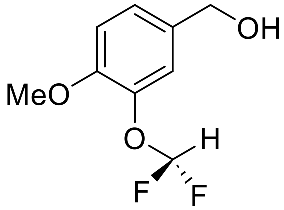

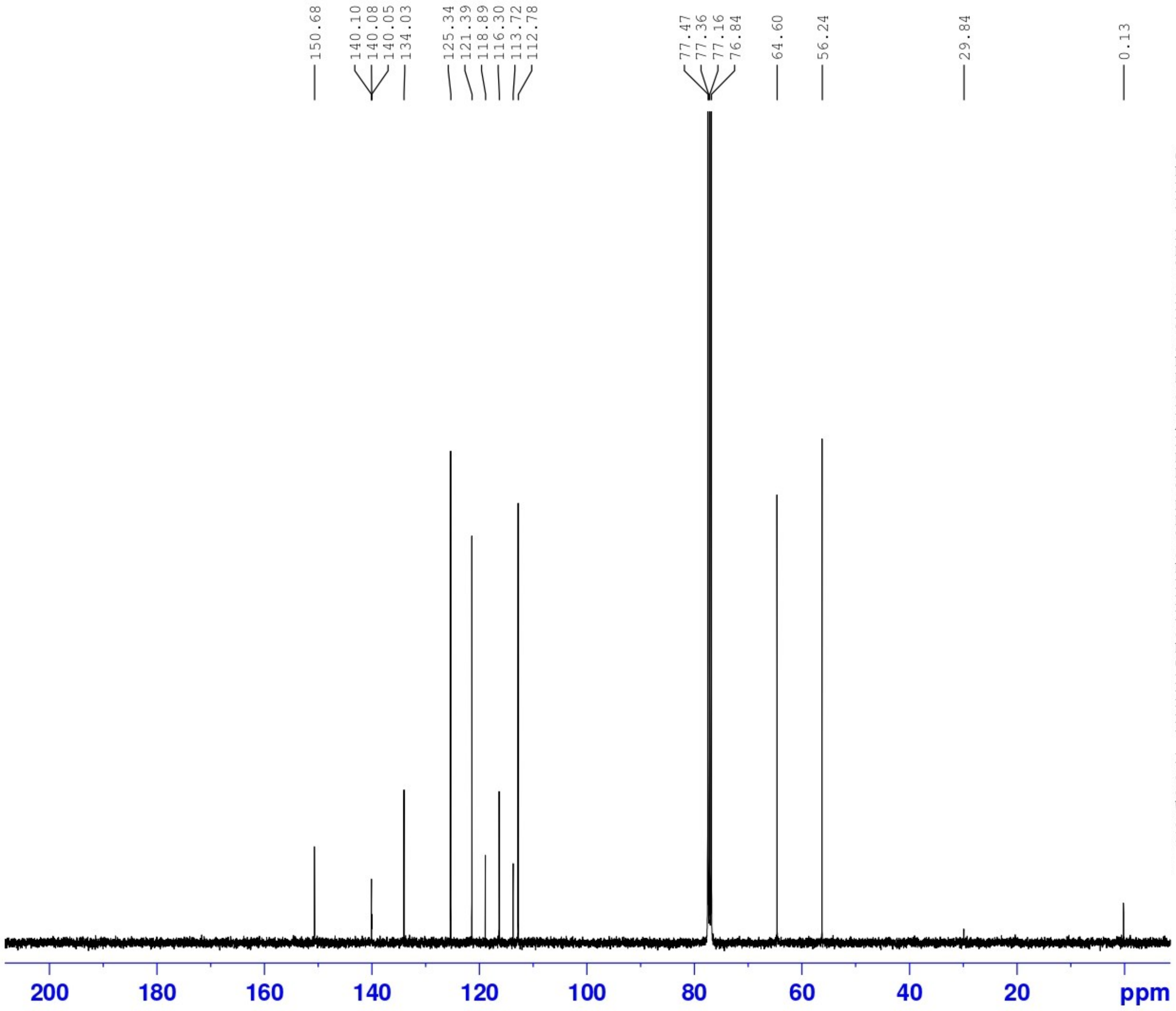

Current Data Parameters

NAME	VI-Mn-43_12
EXPNO	1
PROCNO	1

F2 - Acquisition Parameters

Date_	20240903
Time	22.47
INSTRUM	Avance
PROBHD	Z166552_0018 (PI HR-
PULPROG	zgpg30
TD	65536
SOLVENT	CDCl3
NS	2048
DS	4
SWH	23809.524
FIDRES	0.726609
AQ	1.3762560
RG	101
DW	21.000
DE	6.50
TE	298.0
D1	2.00000000
D11	0.03000000
TD0	1
SFO1	100.4814260
NUC1	13C
P0	2.67
P1	8.00
PLW1	88.22599792
SFO2	399.5693013
NUC2	1H
CPDPRG[2]	waltz65
PCPD2	90.00
PLW2	21.19799995
PLW12	0.15922000
PLW13	0.08008700

F2 - Processing parameters

SI	32768
SF	100.4713658
WDW	EM
SSB	0
LB	1.00

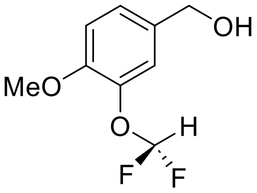

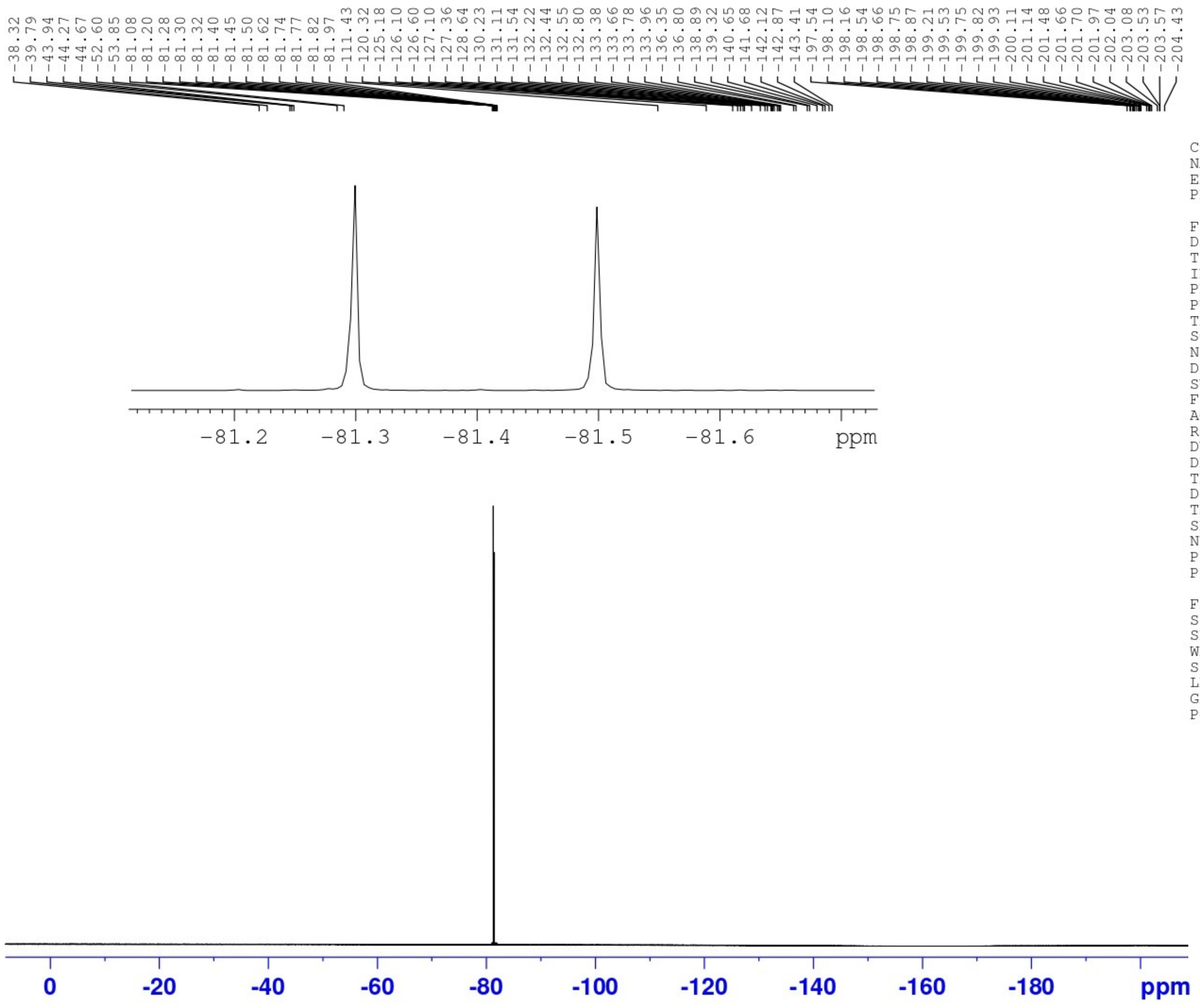

Current Data Parameters

NAME	VI-Mn-43_11
EXPNO	2
PROCNO	1

F2 - Acquisition Parameters

Date_	20240903
Time	11.41
INSTRUM	Avance
PROBHD	Z166552_0018 (PI HR-
PULPROG	zg
TD	131072
SOLVENT	CDCl3
NS	16
DS	4
SWH	90909.091
FIDRES	1.387163
AQ	0.7208960
RG	101
DW	5.500
DE	6.50
TE	298.0
D1	1.00000000
TD0	1
SFO1	375.9316815
NUC1	19F
P1	12.00
PLW1	32.47200012

F2 - Processing parameters

SI	65536
SF	375.9692784
WDW	EM
SSB	0
LB	0.30
GB	0
PC	1.00

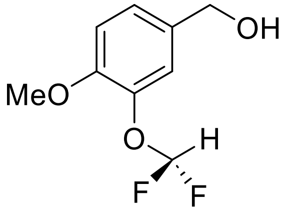

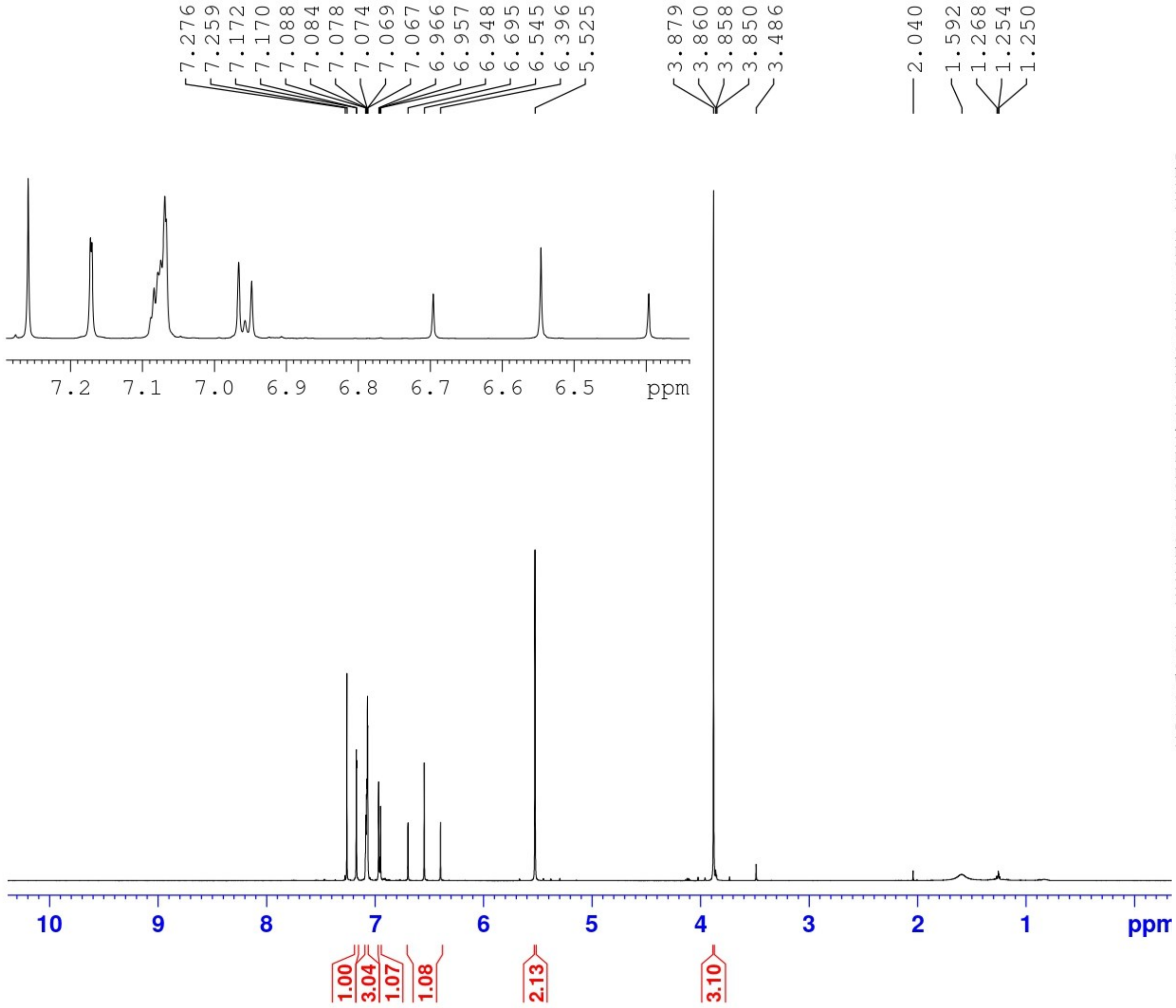

Current Data Parameters	
NAME	VII-Mn-86 Re i_10
EXPNO	2
PROCNO	1
F2 - Acquisition Parameters	
Date_	20241220
Time	11.11
INSTRUM	AS500-NEO
PROBHD	Z168772_0026 (CPP1.1
PULPROG	zg30
TD	65536
SOLVENT	CDCl3
NS	16
DS	2
SWH	10000.000
FIDRES	0.305176
AQ	3.2767999
RG	45.2
DW	50.000
DE	10.45
TE	298.0
D1	1.00000000
TD0	1
SFO1	499.7860862
NUC1	1H
P0	4.00
P1	12.00
PLW1	16.91500092
F2 - Processing parameters	
SI	65536
SF	499.7830123
WDW	EM
SSB	0
LB	0.30
GB	0
PC	1.00

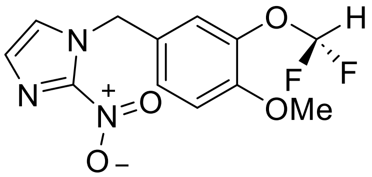

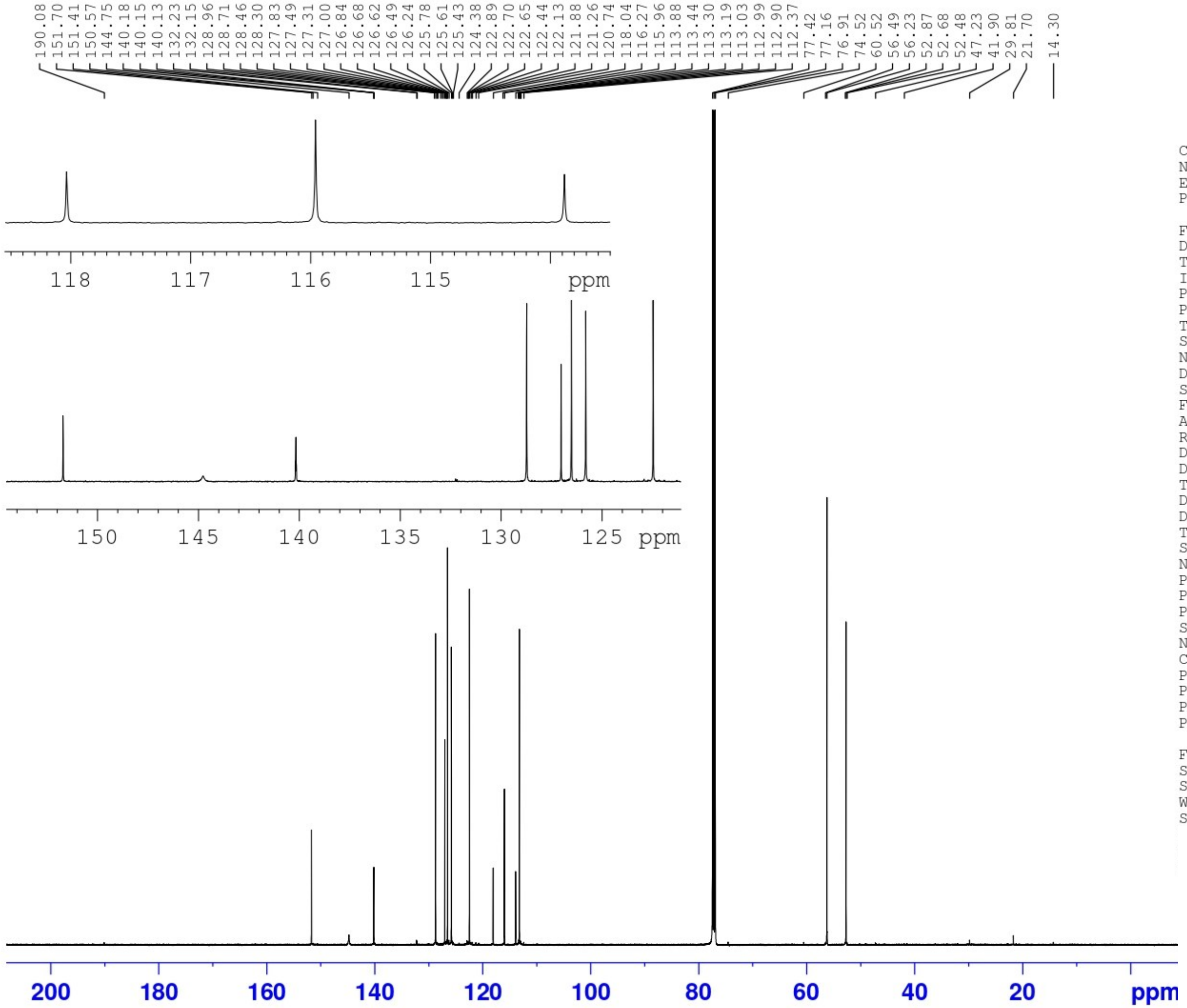

Current Data Parameters
NAME VII-Mn-86 Rei_12
EXPNO 1
PROCNO 1

F2 - Acquisition Parameters
Date_ 20251101
Time 2.26
INSTRUM AS500-NEO
PROBHD Z168772_0026
PULPROG zgpg30
TD 65536
SOLVENT CDCl3
NS 4096
DS 4
SWH 30120.482
FIDRES 0.919204
AQ 1.0878976
RG 101
DW 0.000
DE 18.00
TE 298.0
D1 2.00000000
D11 0.03000000
TD0 1
SFO1 125.6831024
NUC1 13C
P0 3.33
P1 10.00
PLW1 59.16400000
SFO2 499.7849991
NUC2 1H
CPDPRG2 waltz65
PCPD2 80.00
PLW2 16.91500000
PLW12 0.38059000
PLW13 0.19143000

F2 - Processing parameters
SI 32768
SF 125.6705221
WDW EM
SSB 0

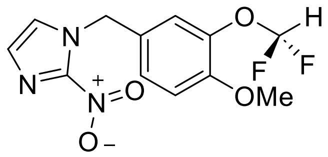

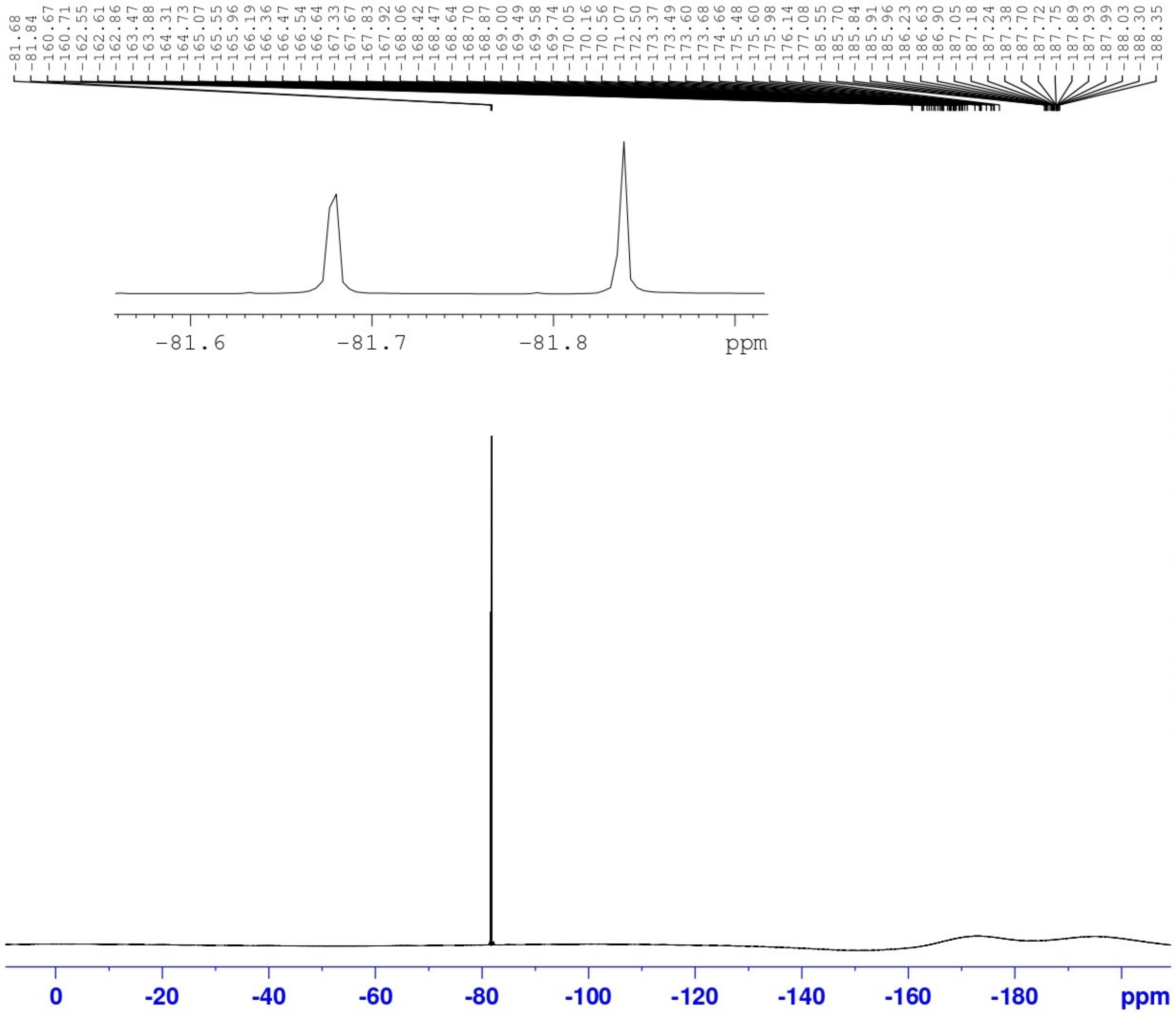

Current Data Parameters
NAME VII-Mn-86 Re i_11
EXPNO 2
PROCNO 1

F2 - Acquisition Parameters
Date_ 20241220
Time 11.13
INSTRUM AS500-NEO
PROBHD Z168772_0026 (CPP1.1
PULPROG zg
TD 131072
SOLVENT CDCl3
NS 16
DS 4
SWH 113636.364
FIDRES 1.733953
AQ 0.5767168
RG 11.3
DW 4.400
DE 18.00
TE 298.0
D1 1.00000000
TD0 1
SFO1 470.2188444
NUC1 19F
P1 15.00
PLW1 10.89000034

F2 - Processing parameters
SI 65536
SF 470.2658710
WDW EM
SSB 0
LB 0.30
GB 0
PC 1.00

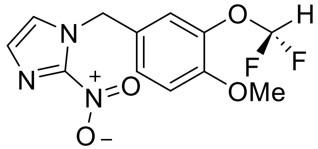

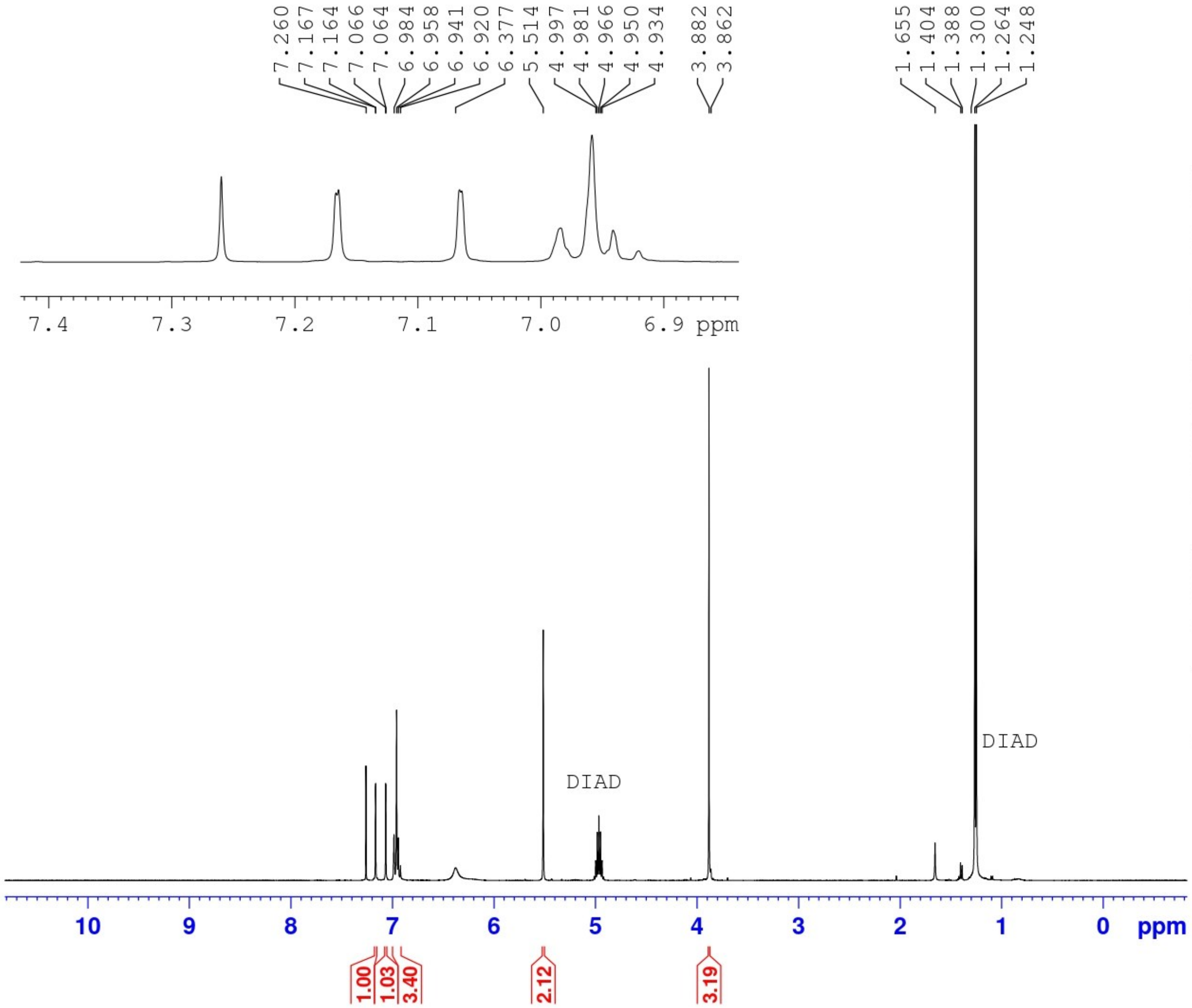

Current Data Parameters
NAME VII-Mn-99 i_10
EXPNO 3
PROCNO 1

F2 - Acquisition Parameters
Date_ 20250109
Time 13.40
INSTRUM Avance
PROBHD Z166552_0018 (PI HR-
PULPROG zg30
TD 65536
SOLVENT CDCl3
NS 16
DS 2
SWH 7812.500
FIDRES 0.238419
AQ 4.1943040
RG 101
DW 64.000
DE 6.67
TE 298.0
D1 1.00000000
TD0 1
SFO1 399.5701703
NUC1 1H
P0 2.60
P1 7.80
PLW1 21.19799995

F2 - Processing parameters
SI 65536
SF 399.5677126
WDW EM
SSB 0
LB 0.30
GB 0
PC 1.00

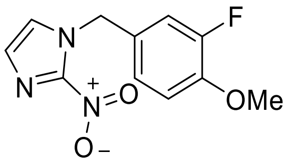

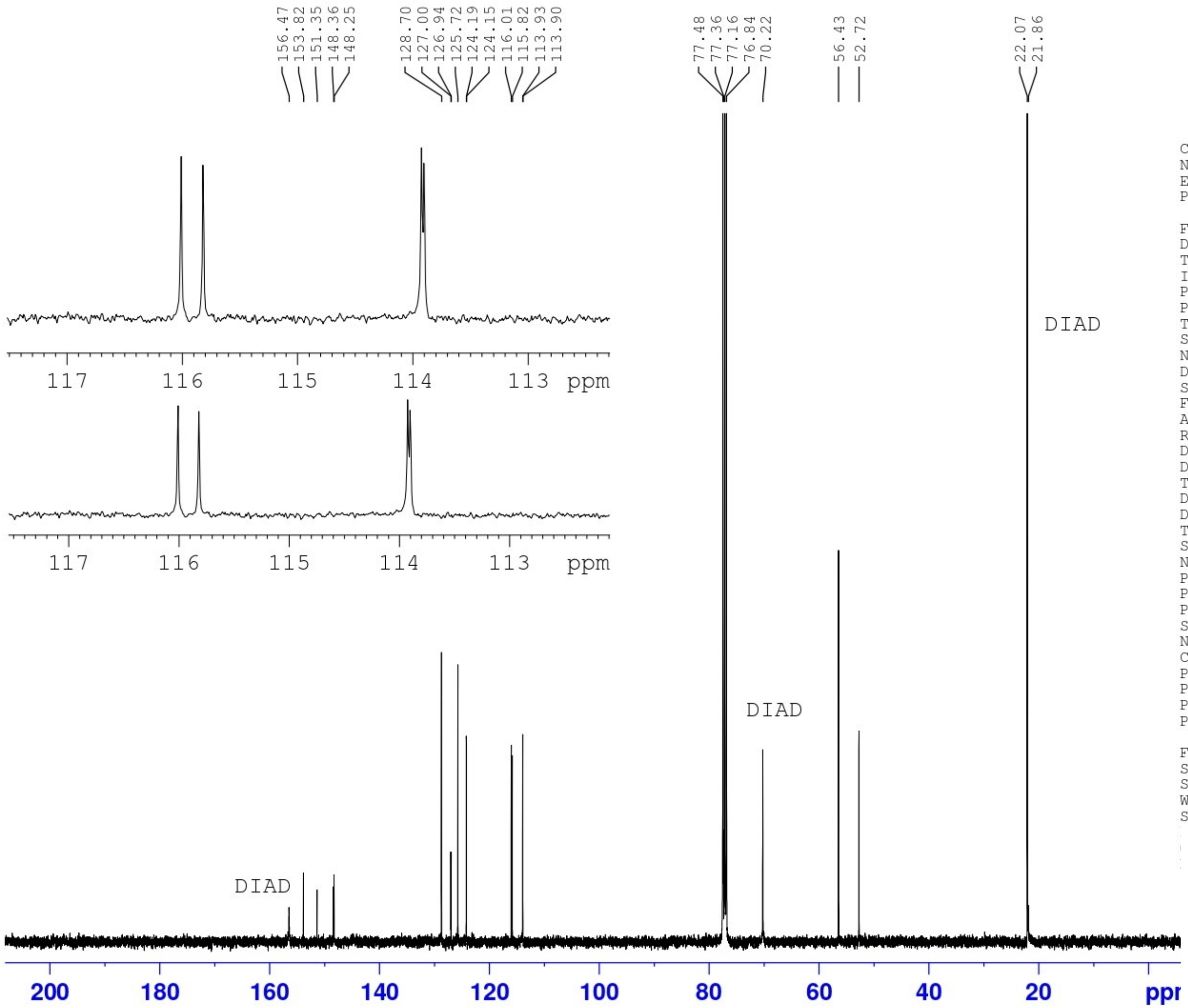

Current Data Parameters
NAME VII-Mn-99 i_12
EXPNO 3
PROCNO 1

F2 - Acquisition Parameters
Date_ 20250109
Time 21.17
INSTRUM Avance
PROBHD Z166552_0018 (PI HR-
PULPROG zgpg30
TD 65536
SOLVENT CDCl3
NS 2048
DS 4
SWH 23809.524
FIDRES 0.726609
AQ 1.3762560
RG 101
DW 21.000
DE 6.50
TE 298.0
D1 2.00000000
D11 0.03000000
TD0 1
SFO1 100.4814260
NUC1 13C
P0 2.67
P1 8.00
PLW1 88.22599792
SFO2 399.5693013
NUC2 1H
CPDPRG[2] waltz65
PCPD2 90.00
PLW2 21.19799995
PLW12 0.15922000
PLW13 0.08008700

F2 - Processing parameters
SI 32768
SF 100.4713664
WDW EM
SSB 0

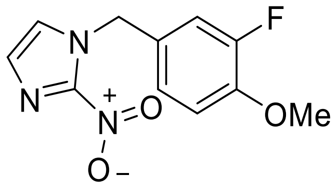

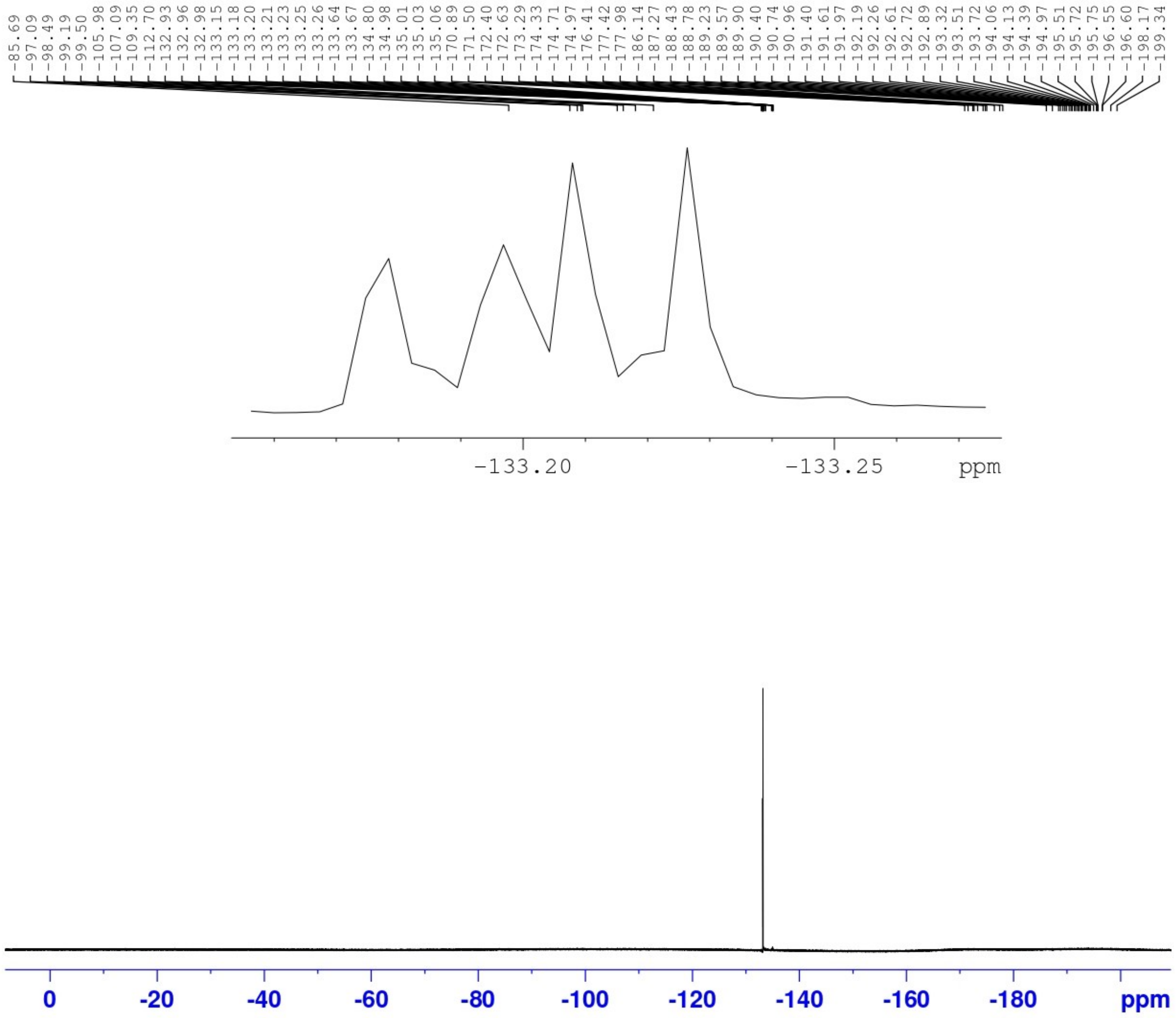

Current Data Parameters
NAME VII-Mn-99 i_11
EXPNO 3
PROCNO 1

F2 - Acquisition Parameters
Date_ 20250109
Time 13.42
INSTRUM Avance
PROBHD Z166552_0018 (PI HR-
PULPROG zg
TD 131072
SOLVENT CDCl3
NS 16
DS 4
SWH 90909.091
FIDRES 1.387163
AQ 0.7208960
RG 101
DW 5.500
DE 6.50
TE 298.0
D1 1.00000000
TD0 1
SFO1 375.9316815
NUC1 19F
P1 12.00
PLW1 32.47200012

F2 - Processing parameters
SI 65536
SF 375.9692784
WDW EM
SSB 0
LB 0.30
GB 0
PC 1.00

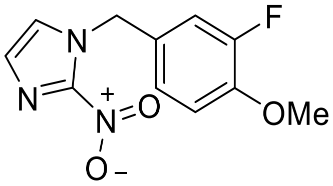

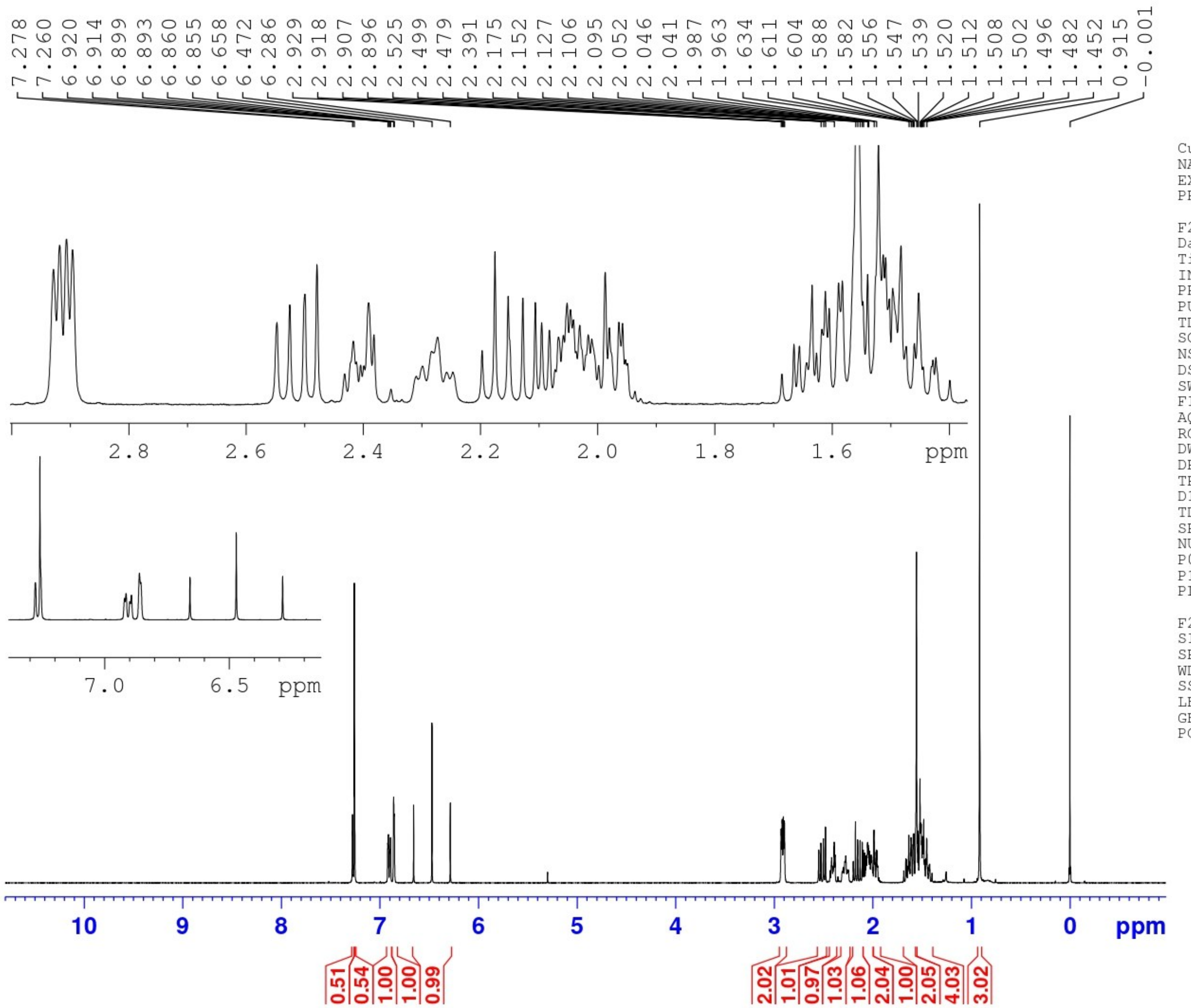
7.278
7.260
6.920
6.914
6.899
6.893
6.860
6.855
6.658
6.472
6.286
2.929
2.918
2.907
2.896
2.525
2.499
2.479
2.391
2.175
2.152
2.127
2.106
2.095
2.052
2.046
2.041
1.987
1.963
1.634
1.611
1.604
1.588
1.582
1.556
1.547
1.539
1.520
1.512
1.508
1.502
1.496
1.482
1.452
0.915
-0.001
BRUKER
Current Data Parameters
NAME V-Mn-55 i_10
EXPNO 2
PROCNO 1
F2 - Acquisition Parameters
Date_ 20240606
Time 11.28
INSTRUM Avance
PROBHD Z166552_0018 (PI HR-
PULPROG zg30
TD 65536
SOLVENT CDCl3
NS 16
DS 2
SWH 7812.500
FIDRES 0.238419
AQ 4.1943040
RG 101
DW 64.000
DE 6.67
TE 298.0
D1 1.00000000
TD0 1
SFO1 399.6024675
NUC1 1H
P0 2.60
P1 7.80
PLW1 21.19799995
F2 - Processing parameters
SI 65536
SF 399.6000096
WDW EM
SSB 0
LB 0.30
GB 0
PC 1.00
2.8
2.6
2.4
2.2
2.0
1.8
1.6
ppm
7.0
6.5
ppm
10
9
8
7
6
5
4
3
2
1
0
ppm
0.51
0.54
1.00
1.00
0.99
2.02
1.01
0.97
1.03
1.06
2.04
1.00
2.05
4.03
3.02

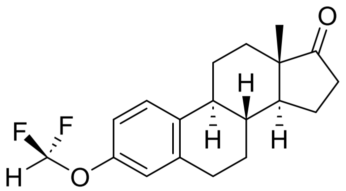
O
H
H
H
F
F
H
O

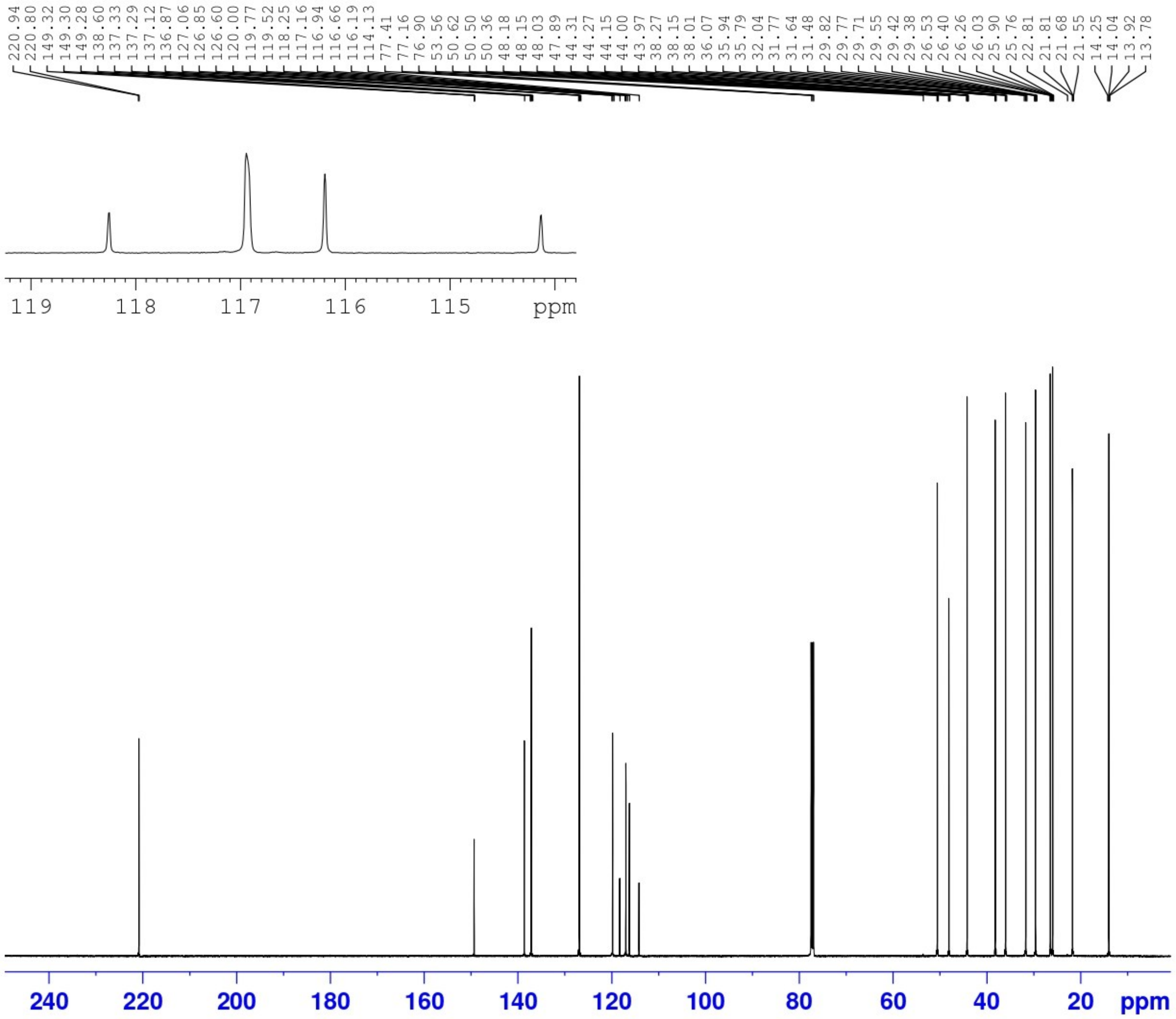

Current Data Parameters
NAME V-Mn-55 ire_10
EXPNO 2
PROCNO 1

F2 - Acquisition Parameters

Date_	20251014
Time	1.32
INSTRUM	AS500-NEO
PROBHD	Z168772_0026 (CPP1.1
PULPROG	zgpg30
TD	65536
SOLVENT	CDCl3
NS	2048
DS	4
SWH	31250.000
FIDRES	0.953674
AQ	1.0485760
RG	101
DW	16.000
DE	18.00
TE	298.0
D1	2.00000000
D11	0.03000000
TD0	1
SFO1	125.6862442
NUC1	13C
P0	3.33
P1	10.00
PLW1	59.16400146
SFO2	499.7849991
NUC2	1H
CPDPRG[2]	waltz65
PCPD2	80.00
PLW2	16.91500092
PLW12	0.38058999
PLW13	0.19113000

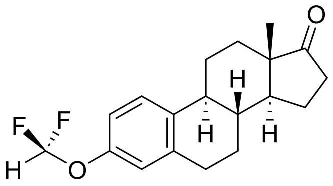

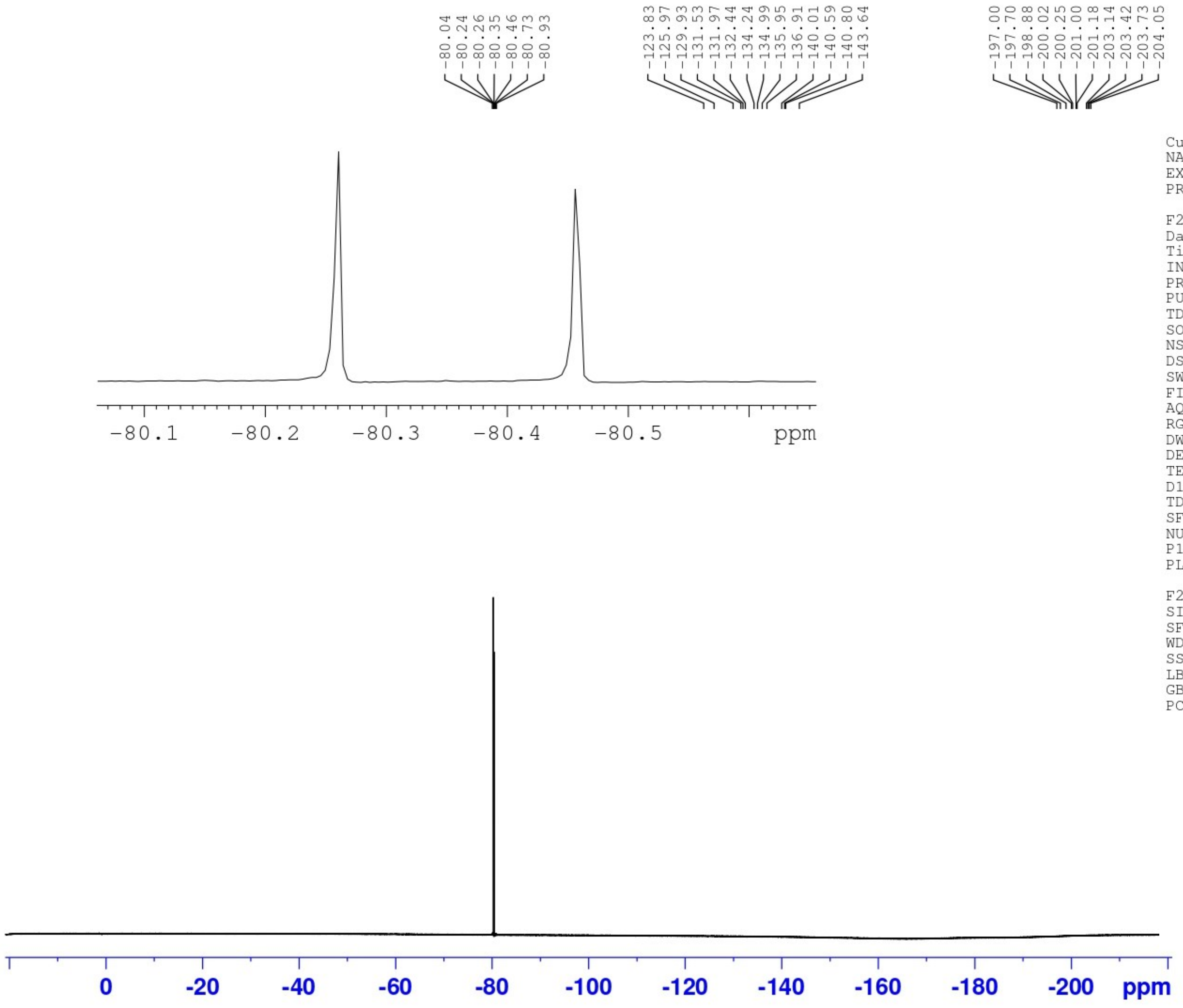

Current Data Parameters	
NAME	V-Mn-55 i_11
EXPNO	2
PROCNO	1
F2 - Acquisition Parameters	
Date_	20240606
Time	11.30
INSTRUM	Avance
PROBHD	Z166552_0018 (PI HR-
PULPROG	zg
TD	131072
SOLVENT	CDCl3
NS	16
DS	4
SWH	90909.091
FIDRES	1.387163
AQ	0.7208960
RG	101
DW	5.500
DE	6.50
TE	298.0
D1	1.00000000
TD0	1
SFO1	375.9620680
NUC1	19F
P1	12.00
PLW1	32.47200012
F2 - Processing parameters	
SI	65536
SF	375.9996680
WDW	EM
SSB	0
LB	0.30
GB	0
PC	1.00

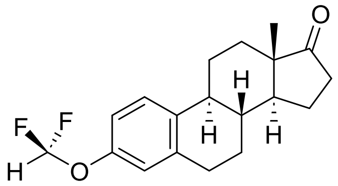

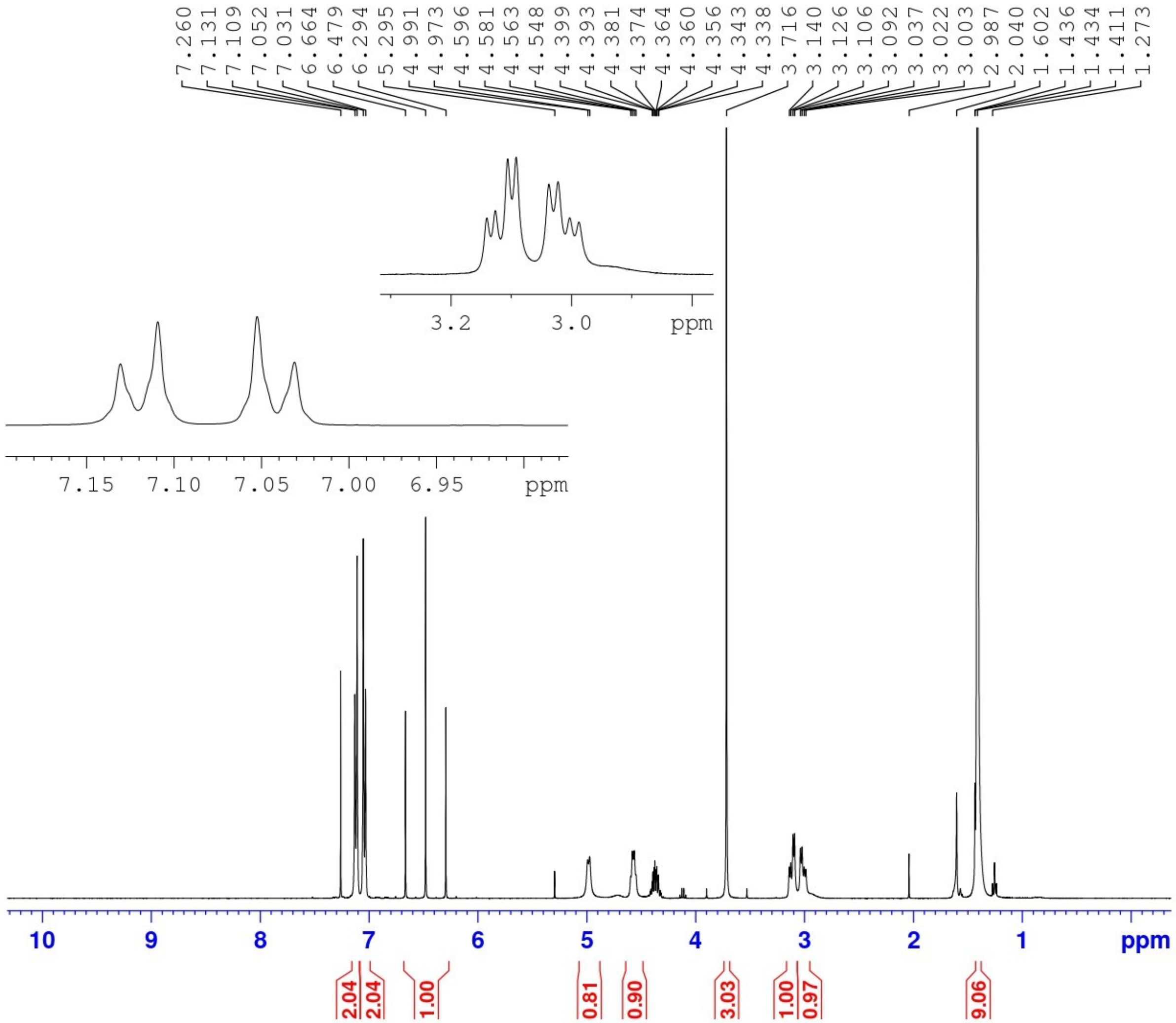

Current Data Parameters
NAME IX-Mn-181 i re_10
EXPNO 1
PROCNO 1

F2 - Acquisition Parameters
Date_ 20250930
Time 18.08
INSTRUM Avance
PROBHD Z166552_0018 (PI HR-
PULPROG zg30
TD 65536
SOLVENT CDCl3
NS 16
DS 2
SWH 7812.500
FIDRES 0.238419
AQ 4.1943040
RG 101
DW 64.000
DE 6.67
TE 298.0
D1 1.00000000
TD0 1
SFO1 399.5424672
NUC1 1H
P0 2.60
P1 7.80
PLW1 21.19799995

F2 - Processing parameters
SI 65536
SF 399.5400000
WDW EM
SSB 0
LB 0.30
GB 0
PC 1.00

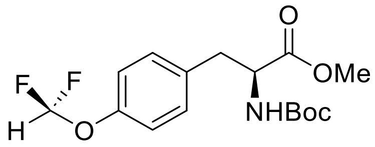

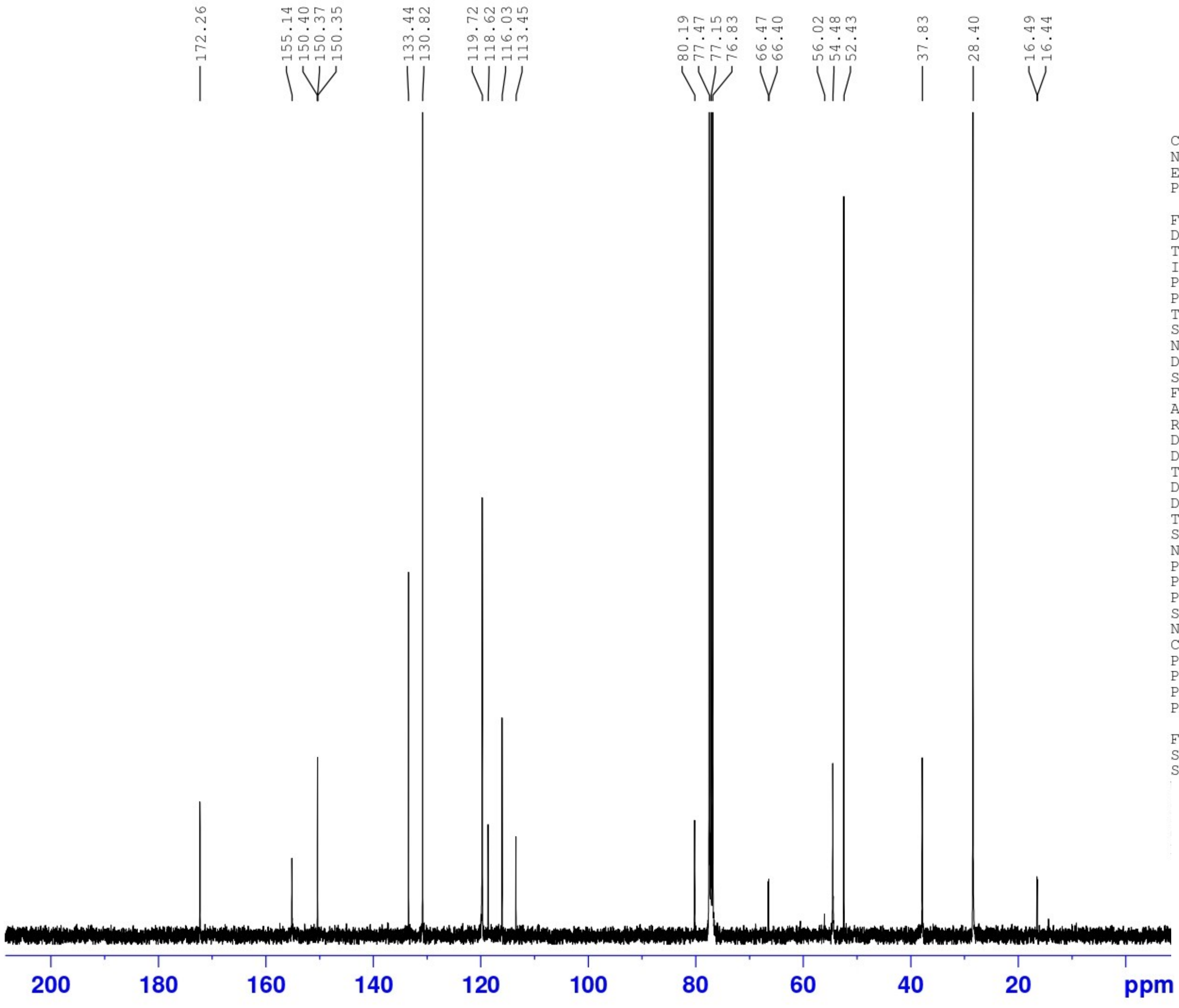

BRUKER

Current Data Parameters

NAME	IX-Mn-181 i re_12
EXPNO	1
PROCNO	1

F2 - Acquisition Parameters

Date_	20250930
Time	20.08
INSTRUM	Avance
PROBHD	Z166552_0018 (PI HR-
PULPROG	zgpg30
TD	65536
SOLVENT	CDCl3
NS	2048
DS	4
SWH	23809.524
FIDRES	0.726609
AQ	1.3762560
RG	101
DW	21.000
DE	6.50
TE	298.0
D1	2.00000000
D11	0.03000000
TD0	1
SFO1	100.4744593
NUC1	13C
P0	2.67
P1	8.00
PLW1	88.22599792
SFO2	399.5415982
NUC2	1H
CPDPRG[2]	waltz65
PCPD2	90.00
PLW2	21.19799995
PLW12	0.15922000
PLW13	0.08008700

F2 - Processing parameters

SI	32768
SF	100.4644003

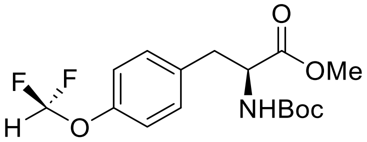

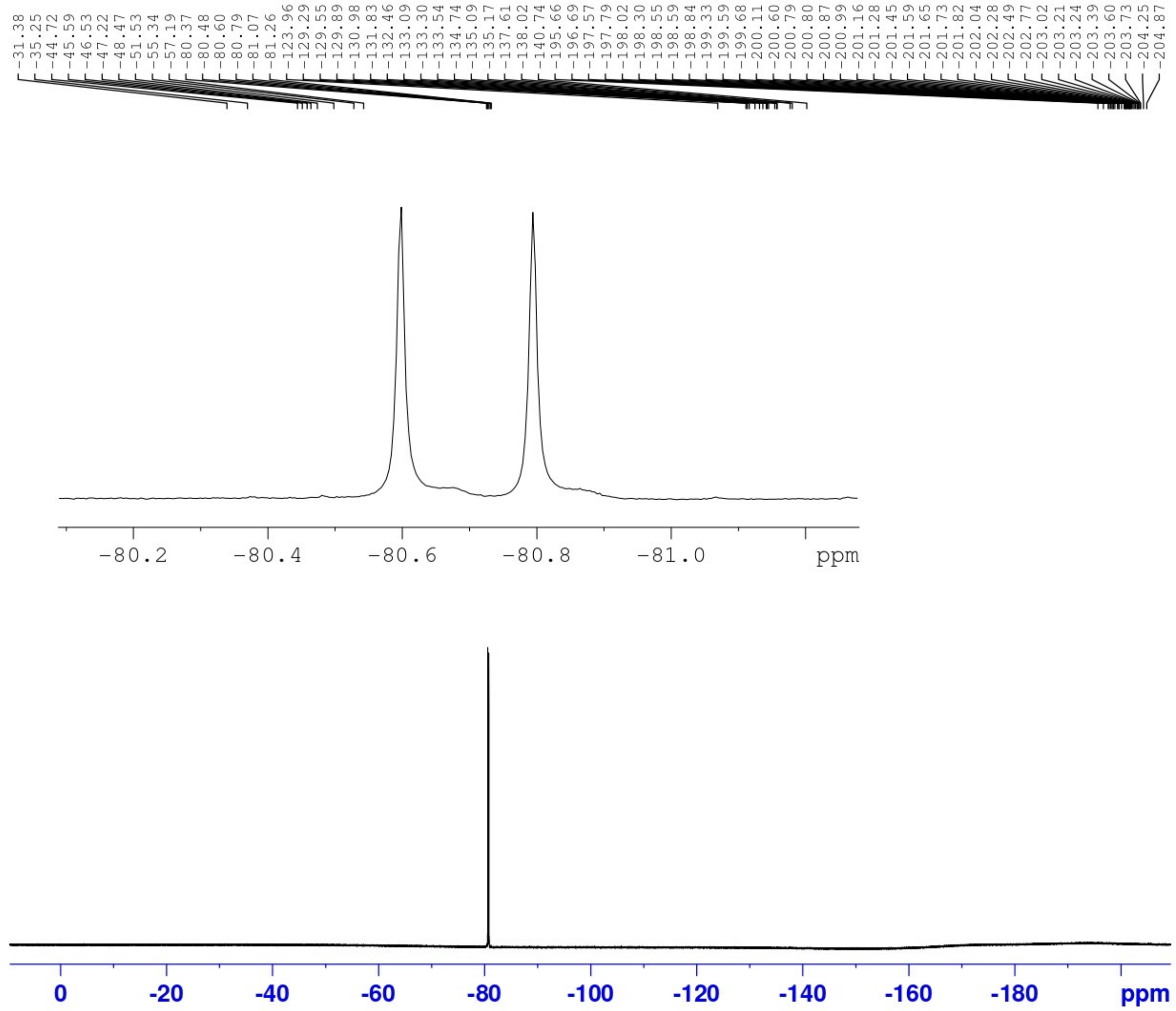

Current Data Parameters
NAME V-Mn-79 i_11
EXPNO 2
PROCNO 1

F2 - Acquisition Parameters
Date_ 20240624
Time 19.30
INSTRUM Avance
PROBHD Z166552_0018 (PI HR-
PULPROG zg
TD 131072
SOLVENT CDCl3
NS 16
DS 4
SWH 90909.091
FIDRES 1.387163
AQ 0.7208960
RG 101
DW 5.500
DE 6.50
TE 298.0
D1 1.00000000
TD0 1
SFO1 375.9316815
NUC1 19F
P1 12.00
PLW1 32.47200012

F2 - Processing parameters
SI 65536
SF 375.9692784
WDW EM
SSB 0
LB 0.30
GB 0

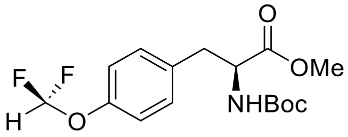

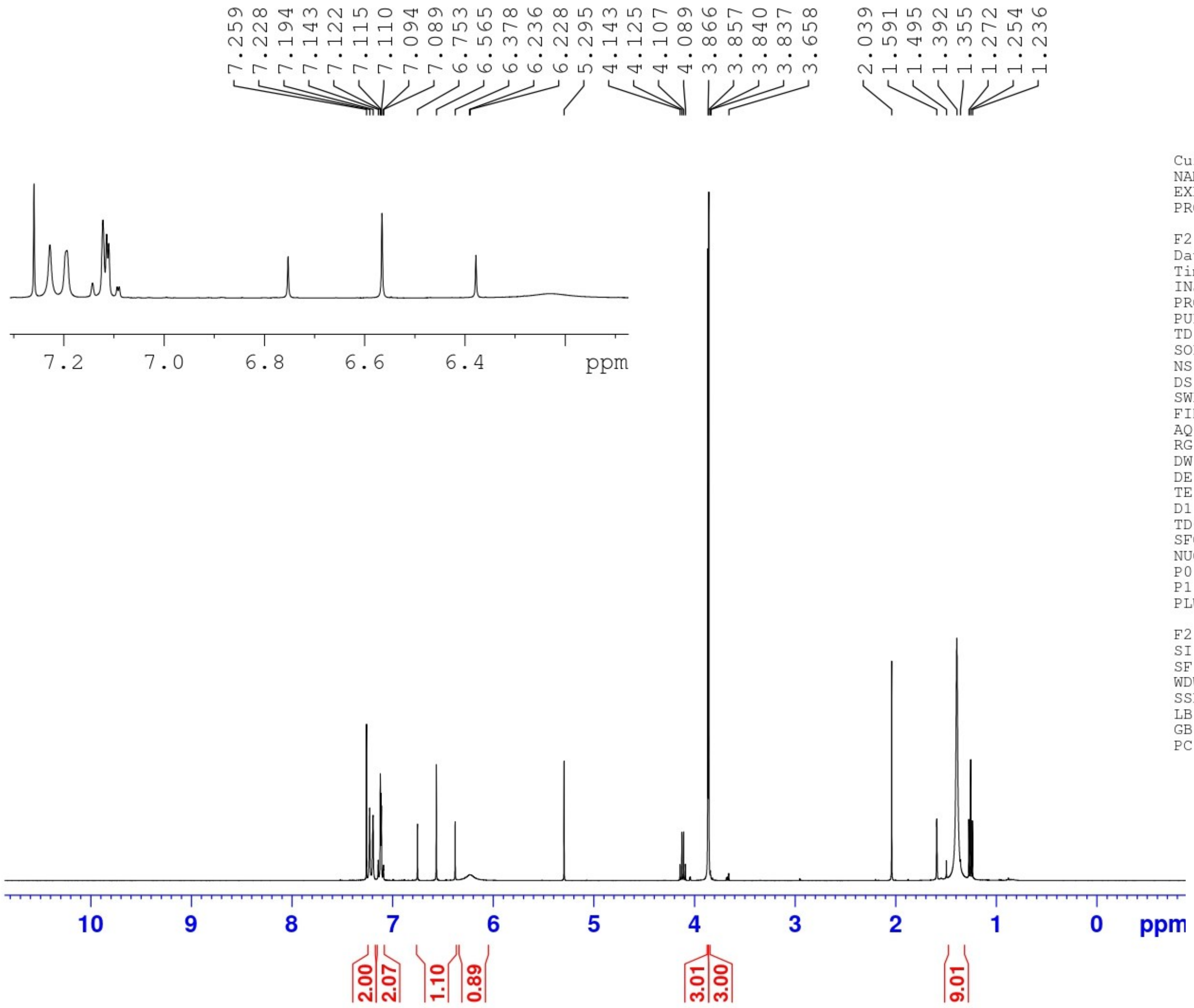

BRUKER

Current Data Parameters
NAME VIII-Mn-23 i_10
EXPNO 2
PROCNO 1

F2 - Acquisition Parameters
Date_ 20250212
Time 16.01
INSTRUM Avance
PROBHD Z166552_0018 (PI HR-
PULPROG zg30
TD 65536
SOLVENT CDCl3
NS 16
DS 2
SWH 7812.500
FIDRES 0.238419
AQ 4.1943040
RG 101
DW 64.000
DE 6.67
TE 298.0
D1 1.00000000
TD0 1
SFO1 399.5701703
NUC1 1H
P0 2.60
P1 7.80
PLW1 21.19799995

F2 - Processing parameters
SI 65536
SF 399.5677128
WDW EM
SSB 0
LB 0.30
GB 0
PC 1.00

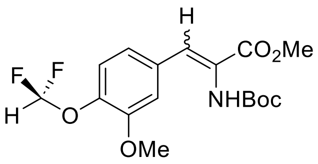

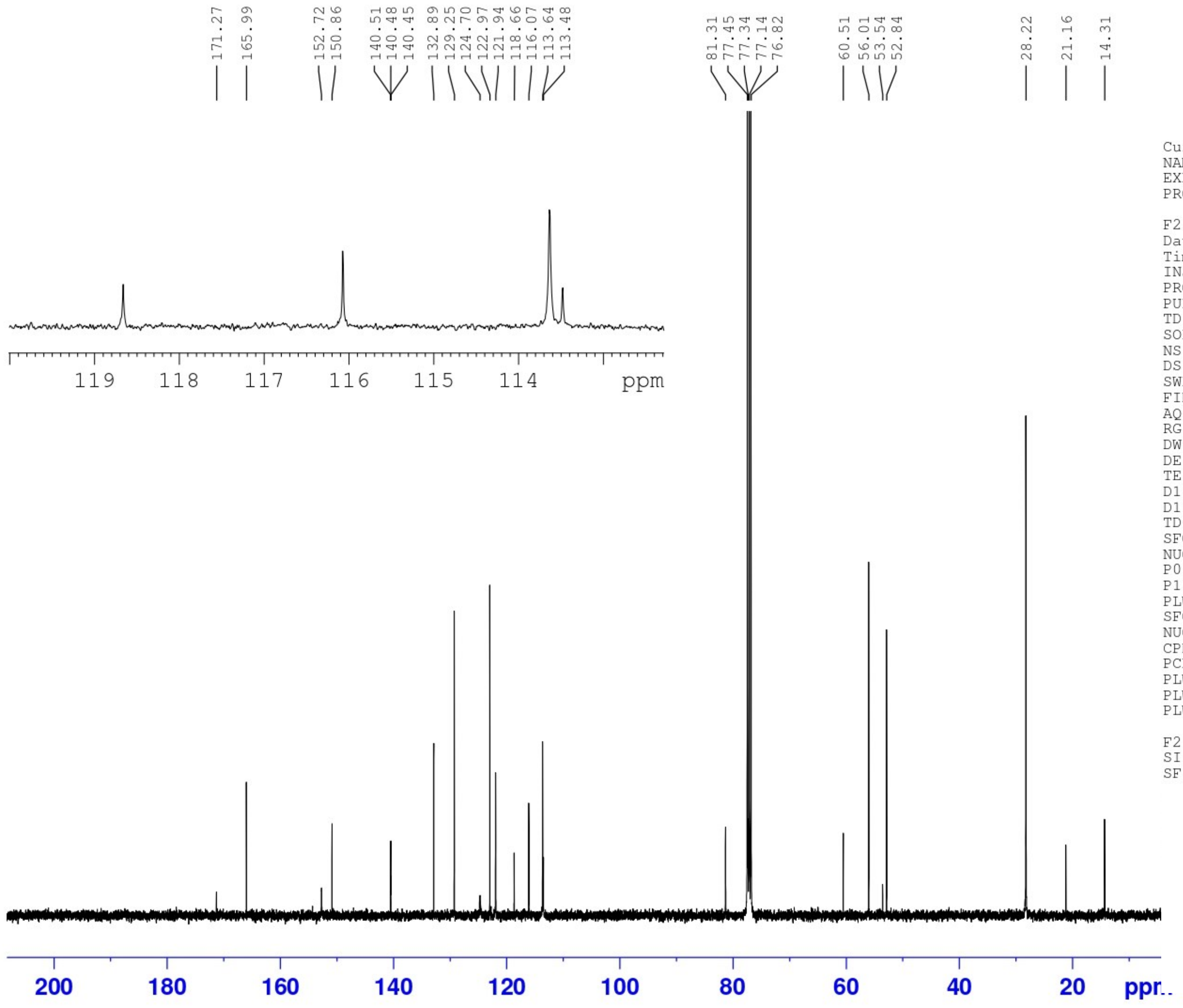

Current Data Parameters
NAME VIII-Mn-23 i_12
EXPNO 2
PROCNO 1

F2 - Acquisition Parameters

Date_	20250212
Time	21.08
INSTRUM	Avance
PROBHD	Z166552_0018 (PI HR-
PULPROG	zgpg30
TD	65536
SOLVENT	CDCl3
NS	2048
DS	4
SWH	23809.524
FIDRES	0.726609
AQ	1.3762560
RG	101
DW	21.000
DE	6.50
TE	298.0
D1	2.00000000
D11	0.03000000
TD0	1
SFO1	100.4814260
NUC1	13C
P0	2.67
P1	8.00
PLW1	88.22599792
SFO2	399.5693013
NUC2	1H
CPDPRG[2]	waltz65
PCPD2	90.00
PLW2	21.19799995
PLW12	0.15922000
PLW13	0.08008700

F2 - Processing parameters

SI	32768
SF	100.4713677

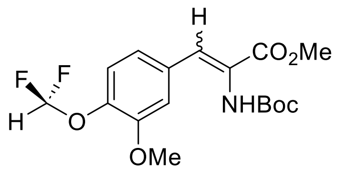

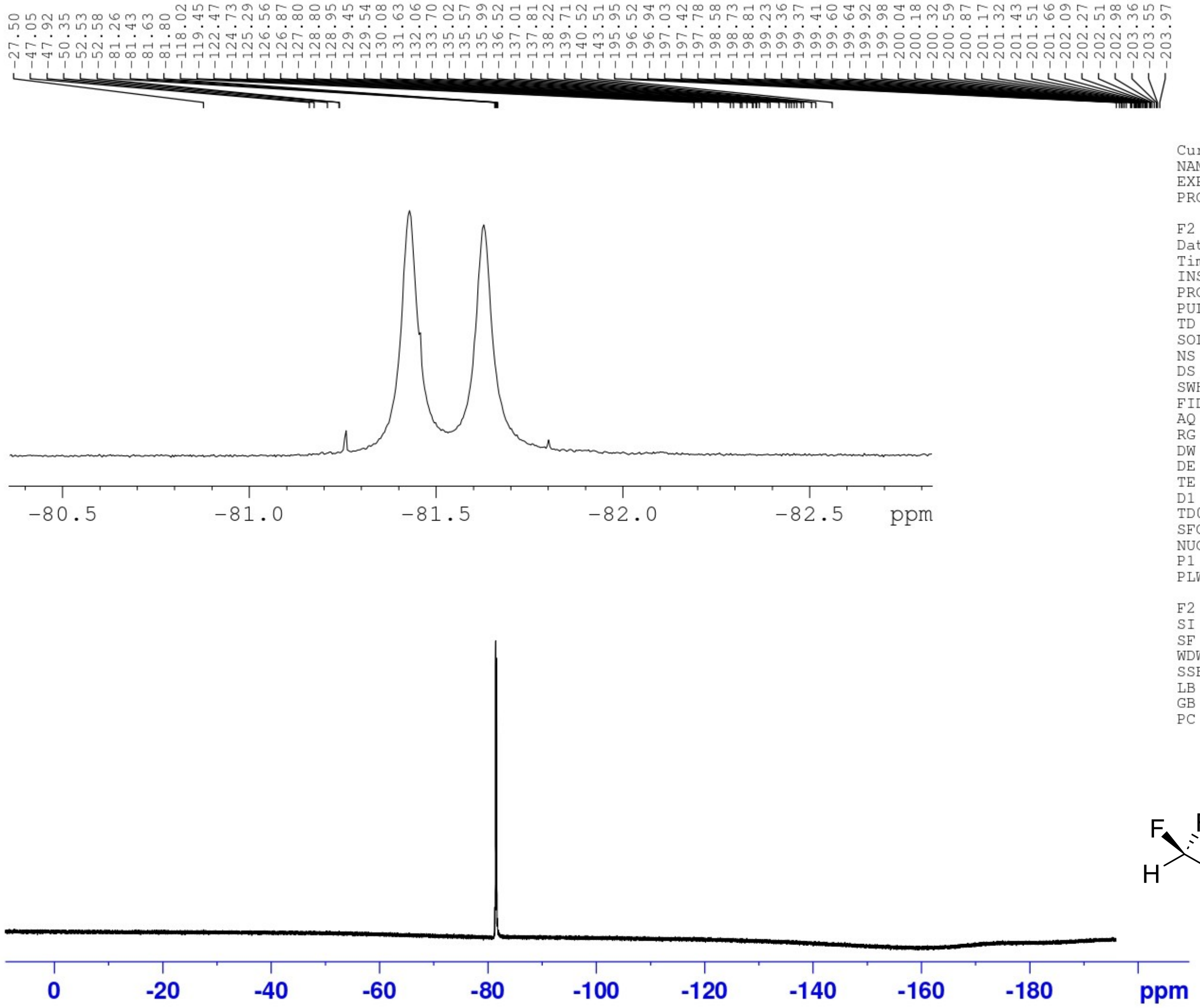

BRUKER

Current Data Parameters	
NAME	VIII-Mn-23 i_11
EXPNO	2
PROCNO	1
F2 - Acquisition Parameters	
Date_	20250212
Time	16.03
INSTRUM	Avance
PROBHD	Z166552_0018 (PI HR-
PULPROG	zg
TD	131072
SOLVENT	CDCl3
NS	16
DS	4
SWH	90909.091
FIDRES	1.387163
AQ	0.7208960
RG	101
DW	5.500
DE	6.50
TE	298.0
D1	1.00000000
TD0	1
SFO1	375.9316815
NUC1	19F
P1	12.00
PLW1	32.47200012
F2 - Processing parameters	
SI	65536
SF	375.9692784
WDW	EM
SSB	0
LB	0.30
GB	0
PC	1.00

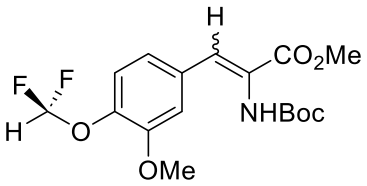

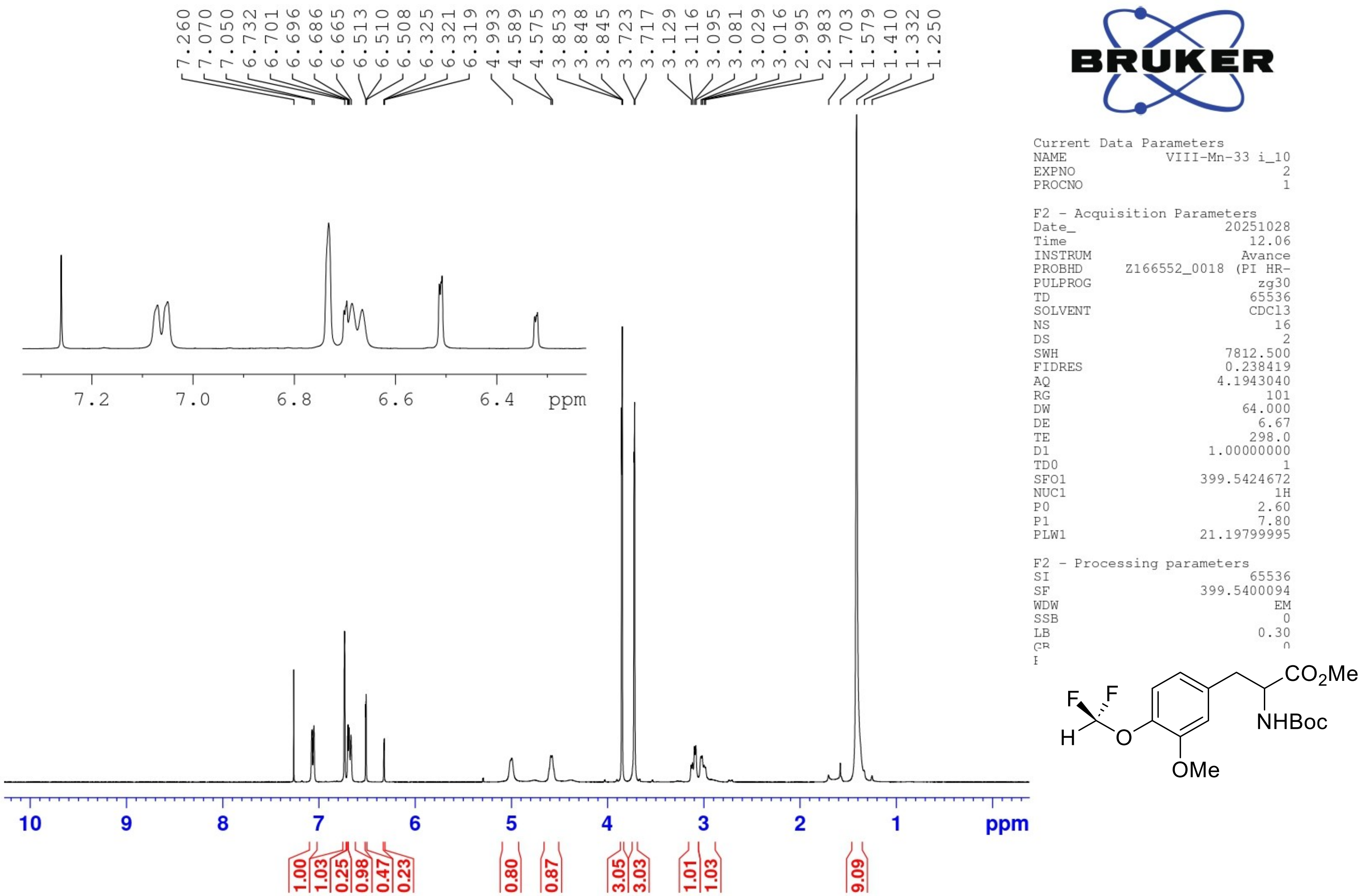
7.260
7.070
7.050
6.732
6.701
6.696
6.686
6.665
6.513
6.510
6.508
6.325
6.321
6.319
4.993
4.589
4.575
3.853
3.848
3.845
3.723
3.717
3.129
3.116
3.095
3.081
3.029
3.016
2.995
2.983
1.703
1.579
1.410
1.332
1.250
BRUKER
Current Data Parameters
NAME VIII-Mn-33 i_10
EXPNO 2
PROCNO 1
F2 - Acquisition Parameters
Date_ 20251028
Time 12.06
INSTRUM Avance
PROBHD Z166552_0018 (PI HR-
PULPROG zg30
TD 65536
SOLVENT CDCl3
NS 16
DS 2
SWH 7812.500
FIDRES 0.238419
AQ 4.1943040
RG 101
DW 64.000
DE 6.67
TE 298.0
D1 1.00000000
TD0 1
SFO1 399.5424672
NUC1 1H
P0 2.60
P1 7.80
PLW1 21.19799995
F2 - Processing parameters
SI 65536
SF 399.5400094
WDW EM
SSB 0
LB 0.30
7.2
7.0
6.8
6.6
6.4
ppm
CO2Me
F
F
H
O
NHBoc
OMe
10
9
8
7
6
5
4
3
2
1
ppm
1.00
1.03
0.25
0.98
0.47
0.23
0.80
0.87
3.05
3.03
1.01
1.03
9.09

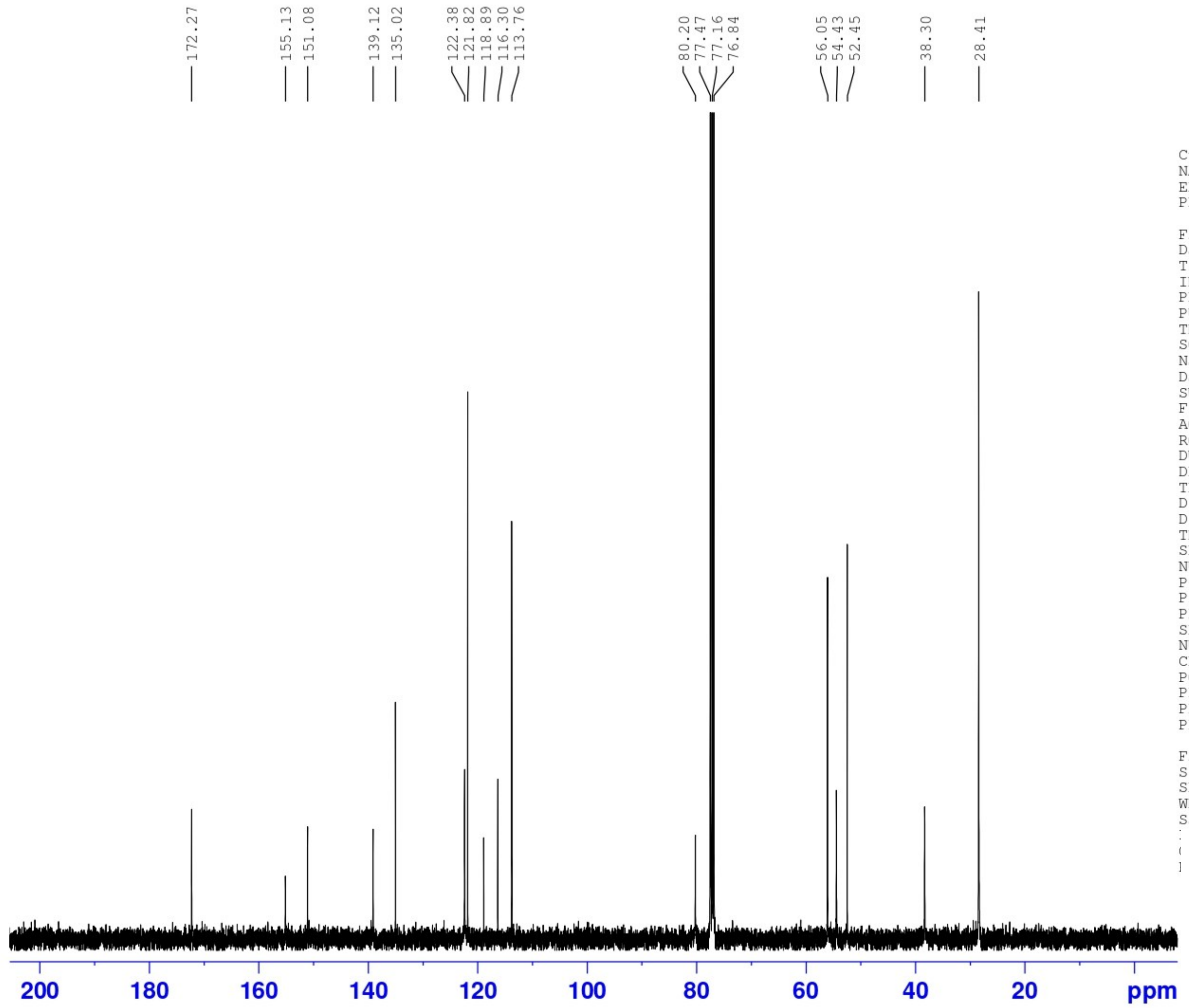

Current Data Parameters
NAME VIII-Mn-33 i_12
EXPNO 2
PROCNO 1

F2 - Acquisition Parameters
Date_ 20251028
Time 12.39
INSTRUM Avance
PROBHD Z166552_0018 (PI HR-
PULPROG zgpg30
TD 65536
SOLVENT CDCl3
NS 512
DS 4
SWH 23809.524
FIDRES 0.726609
AQ 1.3762560
RG 101
DW 21.000
DE 6.50
TE 298.0
D1 2.00000000
D11 0.03000000
TD0 1
SFO1 100.4744593
NUC1 13C
P0 2.67
P1 8.00
PLW1 88.22599792
SFO2 399.5415982
NUC2 1H
CPDPRG[2] waltz65
PCPD2 90.00
PLW2 21.19799995
PLW12 0.15922000
PLW13 0.08008700

F2 - Processing parameters
SI 32768
SF 100.4643998
WDW EM
SSB 0

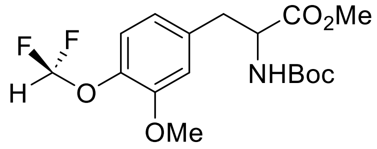

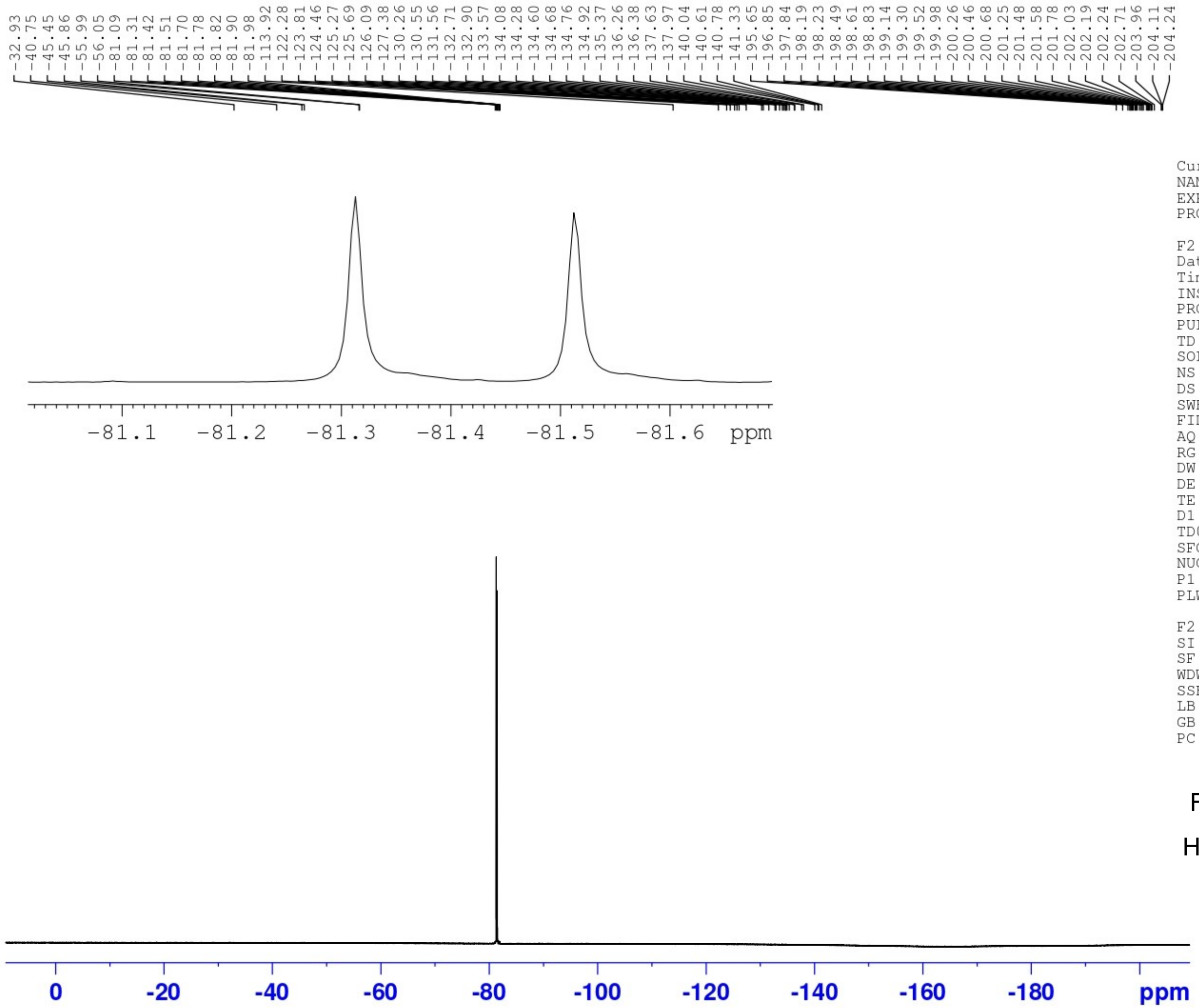

Current Data Parameters
NAME VIII-Mn-33 i_11
EXPNO 1
PROCNO 1

F2 - Acquisition Parameters
Date_ 20251028
Time 12.08
INSTRUM Avance
PROBHD Z166552_0018 (PI HR-
PULPROG zg
TD 131072
SOLVENT CDCl3
NS 16
DS 4
SWH 90909.091
FIDRES 1.387163
AQ 0.7208960
RG 101
DW 5.500
DE 6.50
TE 298.0
D1 1.00000000
TD0 1
SFO1 375.9056172
NUC1 19F
P1 12.00
PLW1 32.47200012

F2 - Processing parameters
SI 65536
SF 375.9432115
WDW EM
SSB 0
LB 0.30
GB 0
PC 1.00

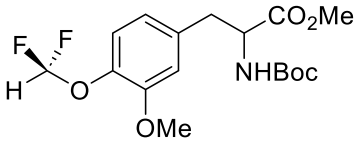

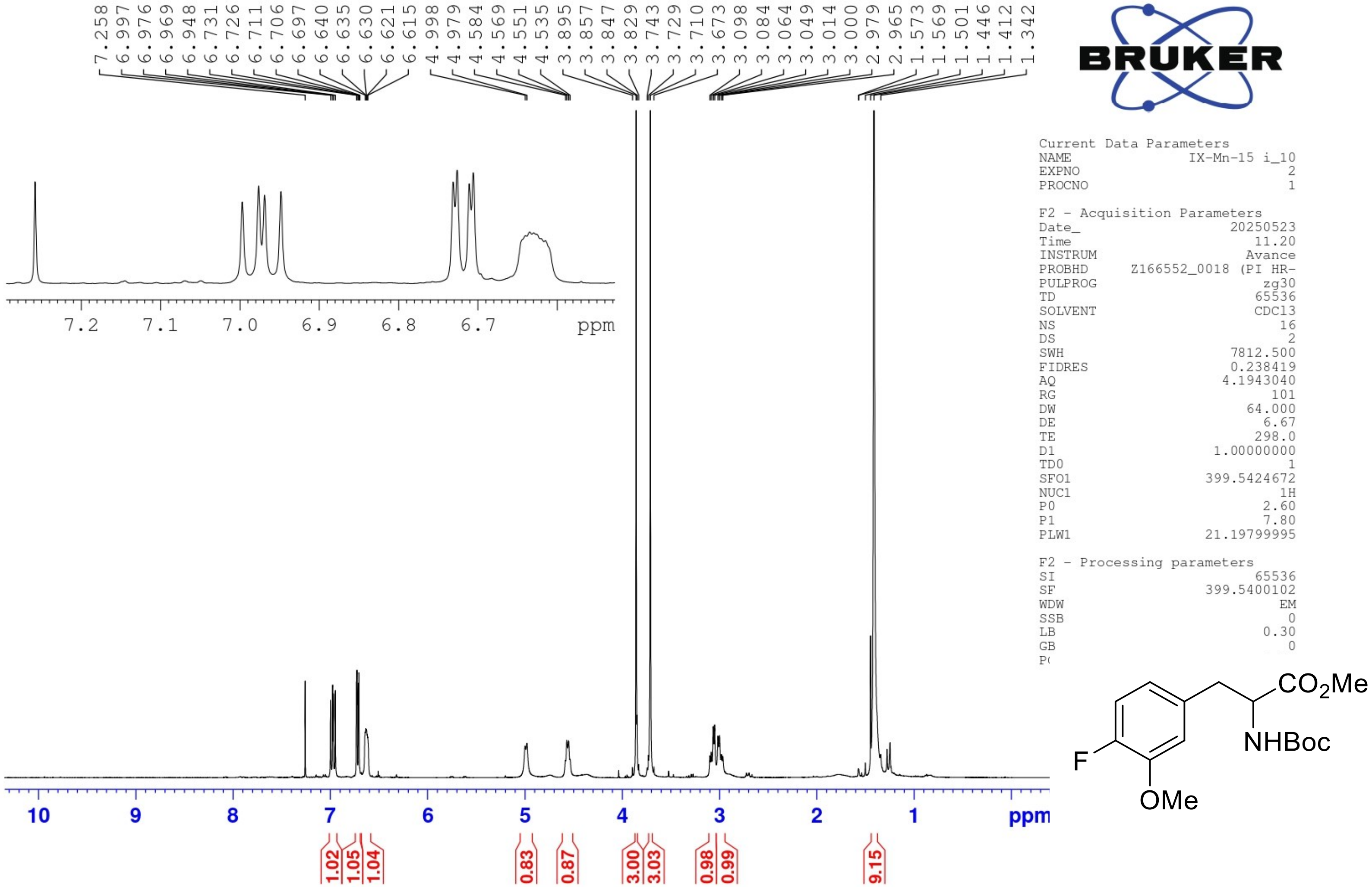
7.258 6.997 6.976 6.969 6.948 6.731 6.726 6.711 6.706 6.697 6.640 6.635 6.630 6.621 6.615 4.998 4.979 4.584 4.569 4.551 4.535 3.895 3.857 3.847 3.829 3.743 3.729 3.710 3.673 3.098 3.084 3.064 3.049 3.014 3.000 2.979 2.965 1.573 1.569 1.501 1.446 1.412 1.342
7.2 7.1 7.0 6.9 6.8 6.7 ppm
Current Data Parameters
NAME IX-Mn-15 i_10
EXPNO 2
PROCNO 1
F2 - Acquisition Parameters
Date_ 20250523
Time 11.20
INSTRUM Avance
PROBHD Z166552_0018 (PI HR-
PULPROG zg30
TD 65536
SOLVENT CDCl3
NS 16
DS 2
SWH 7812.500
FIDRES 0.238419
AQ 4.1943040
RG 101
DW 64.000
DE 6.67
TE 298.0
D1 1.00000000
TD0 1
SFO1 399.5424672
NUC1 1H
P0 2.60
P1 7.80
PLW1 21.19799995
F2 - Processing parameters
SI 65536
SF 399.5400102
WDW EM
SSB 0
LB 0.30
GB 0
10 9 8 7 6 5 4 3 2 1 ppm
1.02 1.05 1.04 0.83 0.87 3.00 3.03 0.98 0.99 9.15
CO2Me
NHBoc
F
OMe

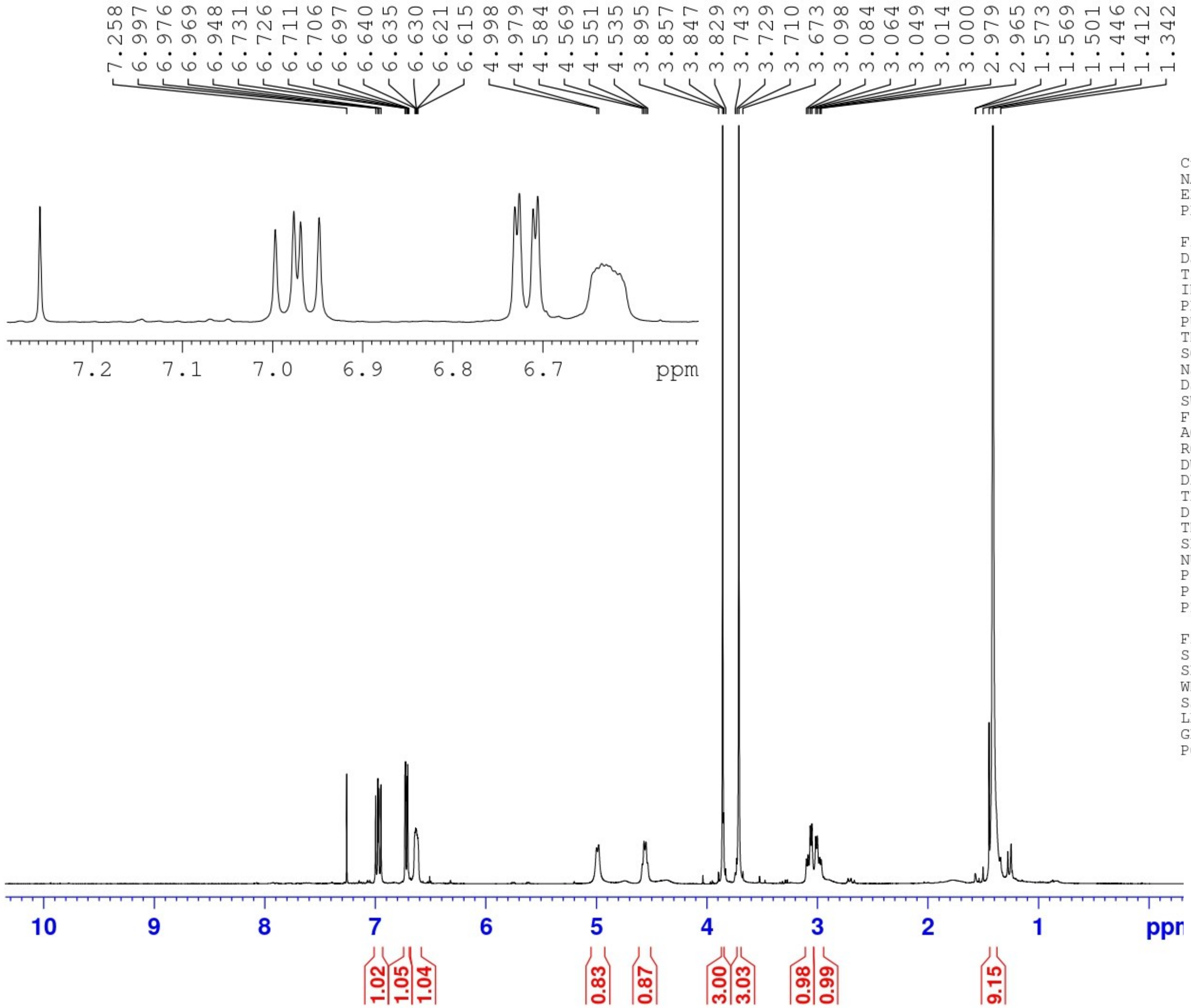


```
Current Data Parameters
NAME            IX-Mn-15 i_10
EXPNO                       2
PROCNO                      1

F2 - Acquisition Parameters
Date_                20250523
Time                    11.20
INSTRUM                Avance
PROBHD     Z166552_0018 (PI HR-
PULPROG                  zg30
TD                      65536
SOLVENT                 CDCl3
NS                         16
DS                          2
SWH                  7812.500
FIDRES               0.238419
AQ                  4.1943040
RG                        101
DW                     64.000
DE                       6.67
TE                      298.0
D1                 1.00000000
TD0                         1
SFO1              399.5424672
NUC1                       1H
P0                       2.60
P1                       7.80
PLW1              21.19799995

F2 - Processing parameters
SI                      65536
SF                399.5400102
WDW                        EM
SSB                         0
LB                       0.30
GB                          0
PC                       1.00
```

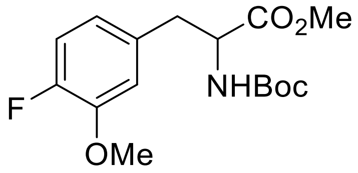

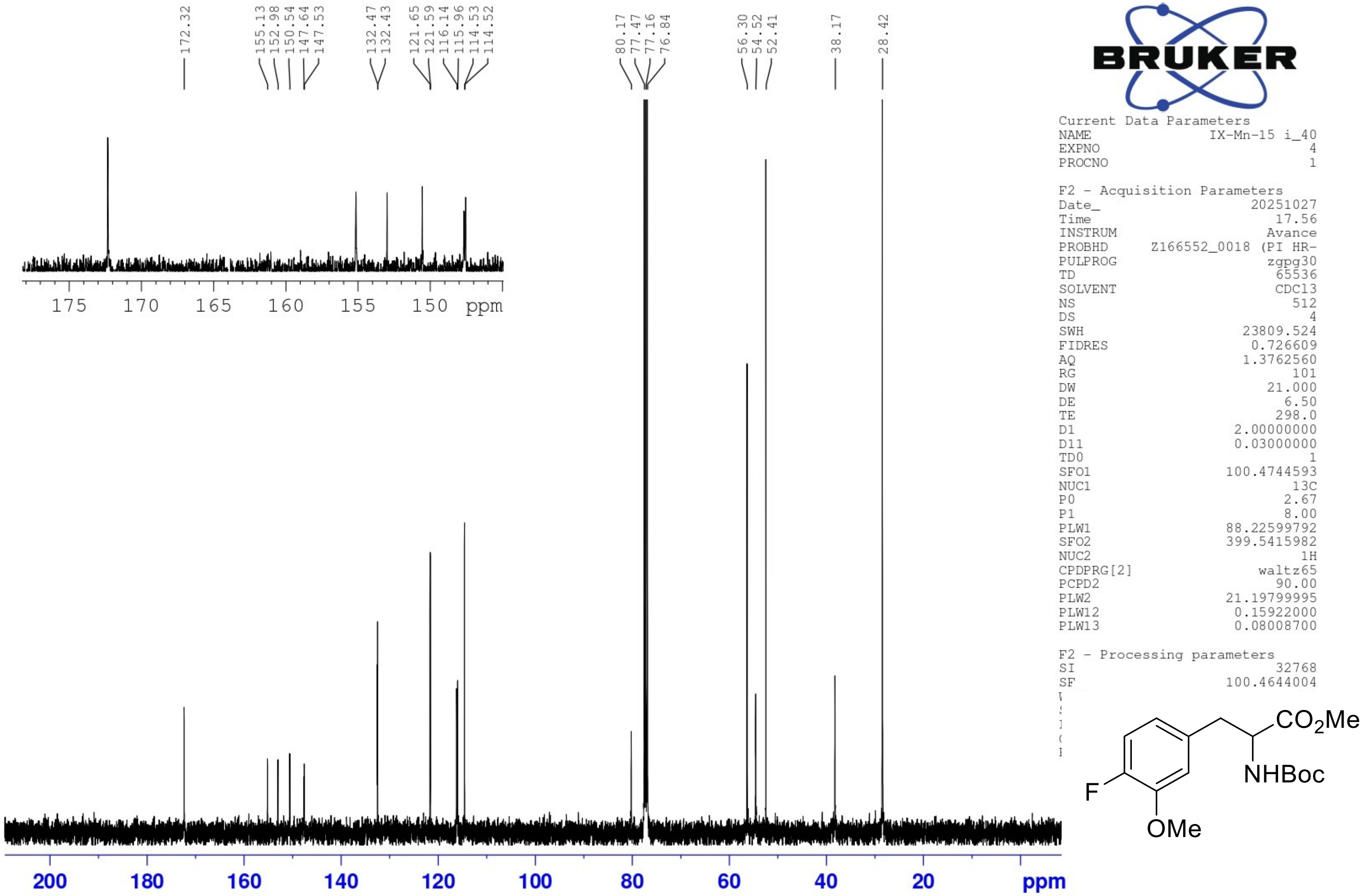
172.32
155.13
152.98
150.54
147.64
147.53
132.47
132.43
121.65
121.59
116.14
115.96
114.53
114.52
80.17
77.47
77.16
76.84
56.30
54.52
52.41
38.17
28.42
175 170 165 160 155 150 ppm
200 180 160 140 120 100 80 60 40 20 ppm
BRUKER
Current Data Parameters
NAME IX-Mn-15 i_40
EXPNO 4
PROCNO 1
F2 - Acquisition Parameters
Date_ 20251027
Time 17.56
INSTRUM Avance
PROBHD Z166552_0018 (PI HR-
PULPROG zgpg30
TD 65536
SOLVENT CDCl3
NS 512
DS 4
SWH 23809.524
FIDRES 0.726609
AQ 1.3762560
RG 101
DW 21.000
DE 6.50
TE 298.0
D1 2.00000000
D11 0.03000000
TD0 1
SFO1 100.4744593
NUC1 13C
P0 2.67
P1 8.00
PLW1 88.22599792
SFO2 399.5415982
NUC2 1H
CPDPRG[2] waltz65
PCPD2 90.00
PLW2 21.19799995
PLW12 0.15922000
PLW13 0.08008700
F2 - Processing parameters
SI 32768
SF 100.4644004
CO2Me
NHBoc
F
OMe

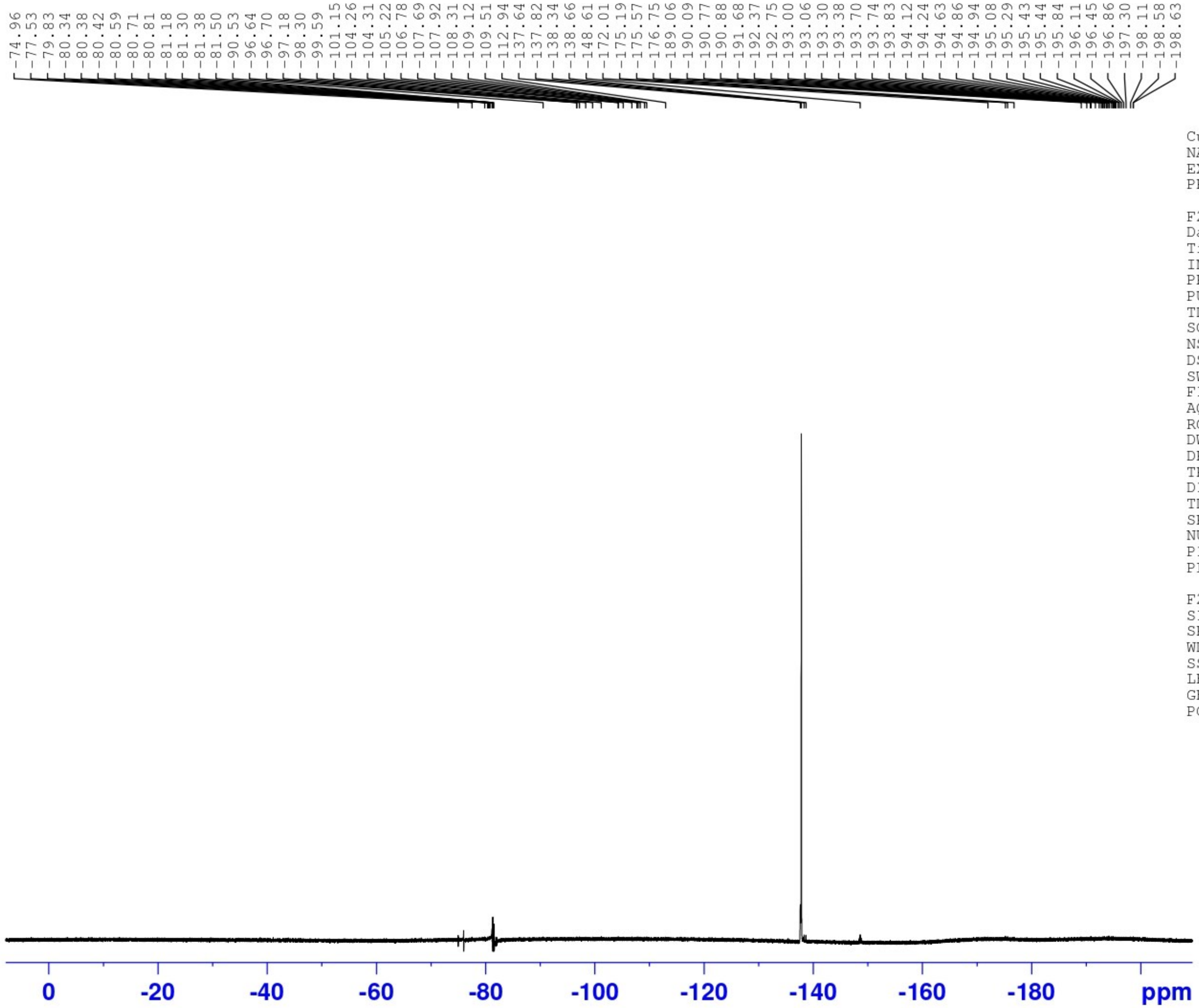

BRUKER

Current Data Parameters
NAME IX-Mn-15 i_11
EXPNO 2
PROCNO 1

F2 - Acquisition Parameters
Date_ 20250523
Time 11.22
INSTRUM Avance
PROBHD Z166552_0018 (PI HR-
PULPROG zg
TD 131072
SOLVENT CDCl3
NS 16
DS 4
SWH 90909.091
FIDRES 1.387163
AQ 0.7208960
RG 101
DW 5.500
DE 6.50
TE 298.0
D1 1.00000000
TD0 1
SFO1 375.9056172
NUC1 19F
P1 12.00
PLW1 32.47200012

F2 - Processing parameters
SI 65536
SF 375.9432115
WDW EM
SSB 0
LB 0.30
GB 0
PC 1.00

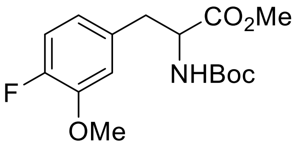

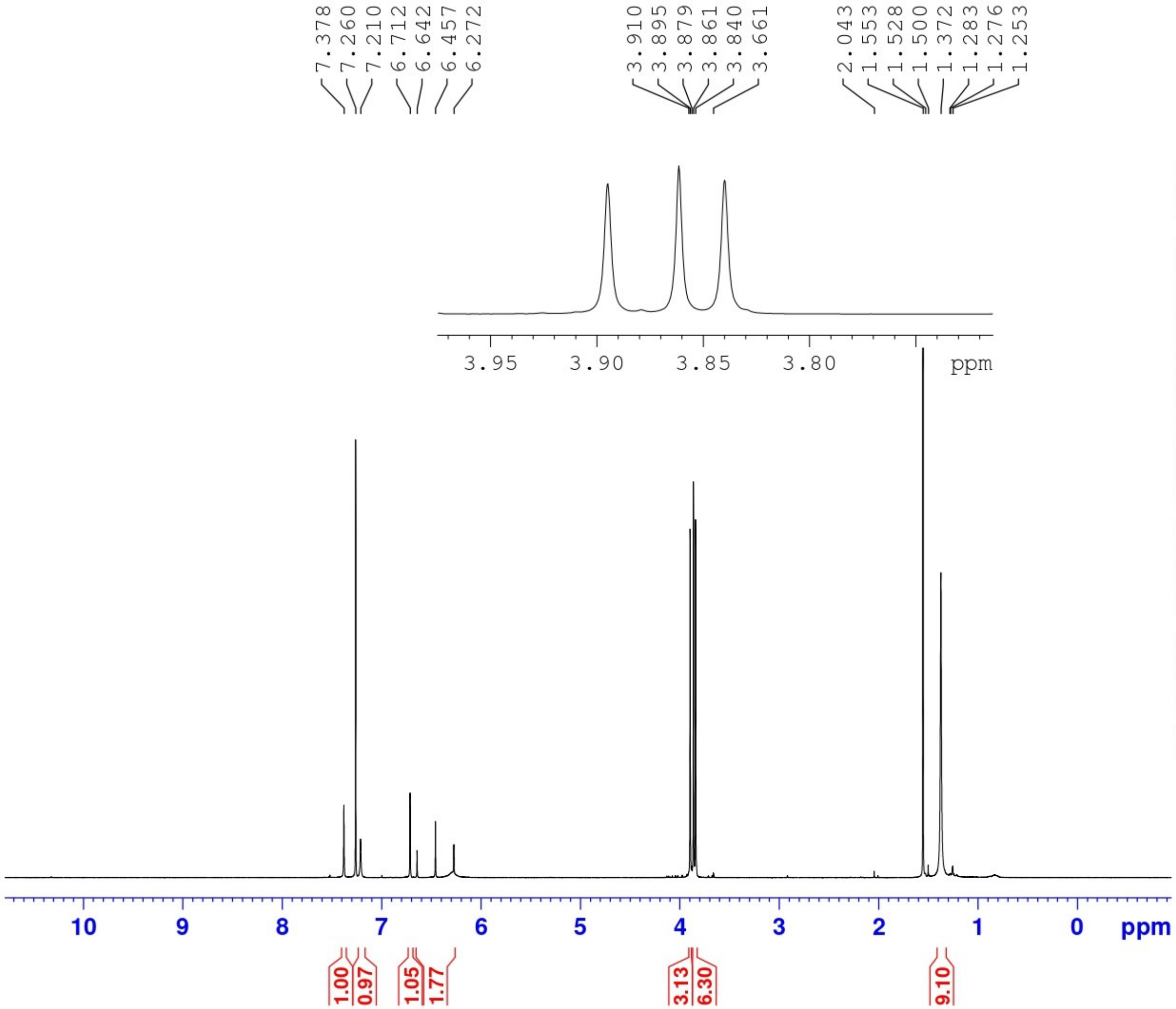

BRUKER

Current Data Parameters

NAME	VIII-Mn-67 i_10
EXPNO	2
PROCNO	1

F2 - Acquisition Parameters

Date_	20250312
Time	15.52
INSTRUM	Avance
PROBHD	Z166552_0018 (PI HR-
PULPROG	zg30
TD	65536
SOLVENT	CDCl3
NS	16
DS	2
SWH	7812.500
FIDRES	0.238419
AQ	4.1943040
RG	101
DW	64.000
DE	6.67
TE	298.0
D1	1.00000000
TD0	1
SFO1	399.5701703
NUC1	1H
P0	2.60
P1	7.80
PLW1	21.19799995

F2 - Processing parameters

SI	65536
SF	399.5677129
WDW	EM
SSB	0
LB	0.30
GB	0
PC	1.00

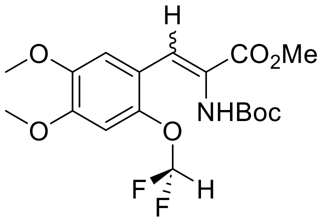

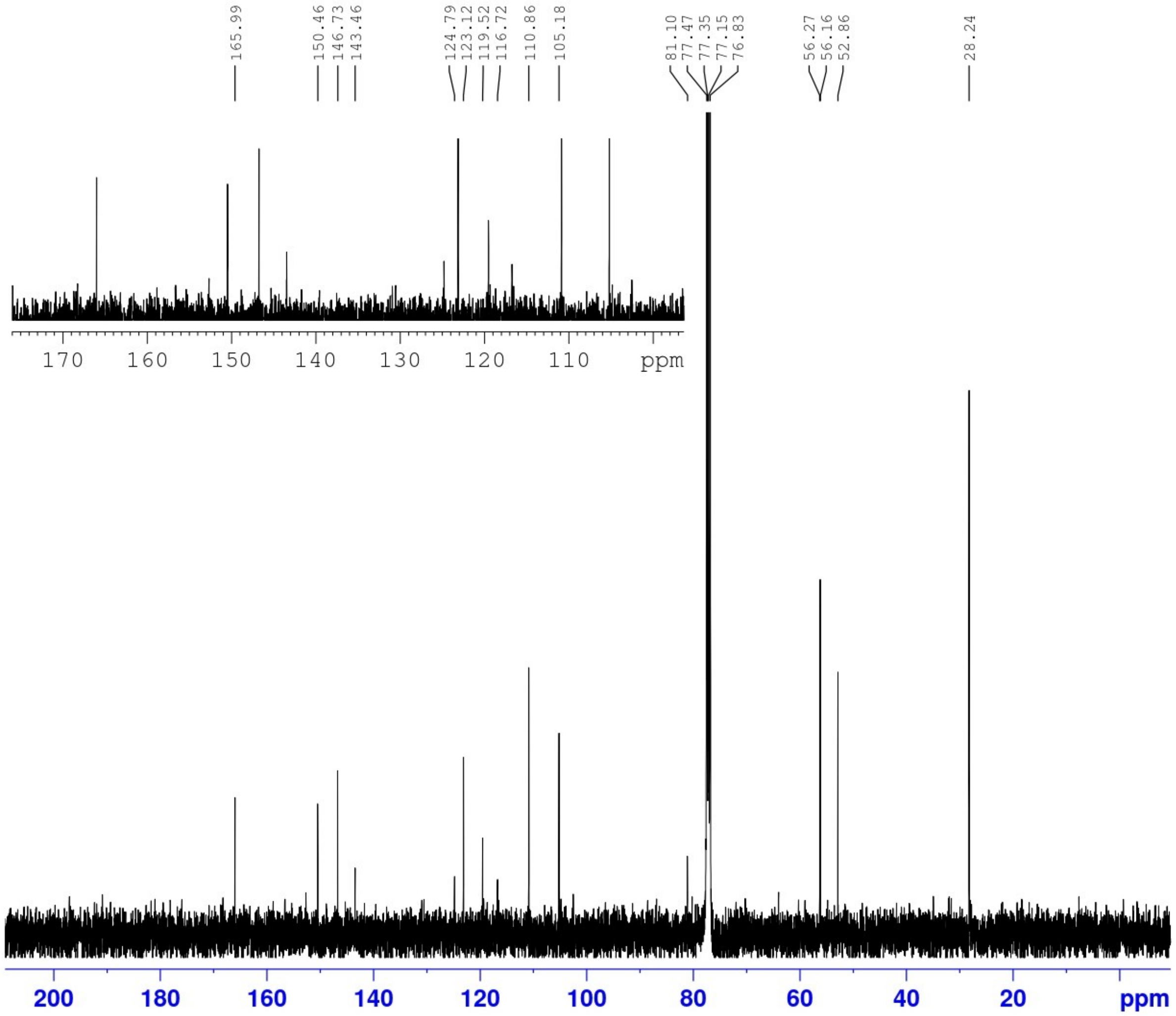


```
Current Data Parameters
NAME              VIII-Mn-67 i_12
EXPNO                           2
PROCNO                          1

F2 - Acquisition Parameters
Date_                    20250312
Time                        20.03
INSTRUM                    Avance
PROBHD       Z166552_0018 (PI HR-
PULPROG                   zgpg30
TD                         65536
SOLVENT                    CDCl3
NS                          2048
DS                             4
SWH                    23809.524
FIDRES                  0.726609
AQ                     1.3762560
RG                           101
DW                        21.000
DE                          6.50
TE                         298.0
D1                    2.00000000
D11                   0.03000000
TD0                            1
SFO1                 100.4814260
NUC1                         13C
P0                          2.67
P1                          8.00
PLW1                 88.22599792
SFO2                 399.5693013
NUC2                          1H
CPDPRG[2]                waltz65
PCPD2                      90.00
PLW2                 21.19799995
PLW12                 0.15922000
PLW13                 0.08008700

F2 - Processing parameters
SI                         32768
SF                   100.4713657
WDW                           EM
SSB                            0
```

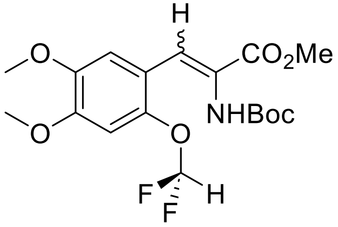

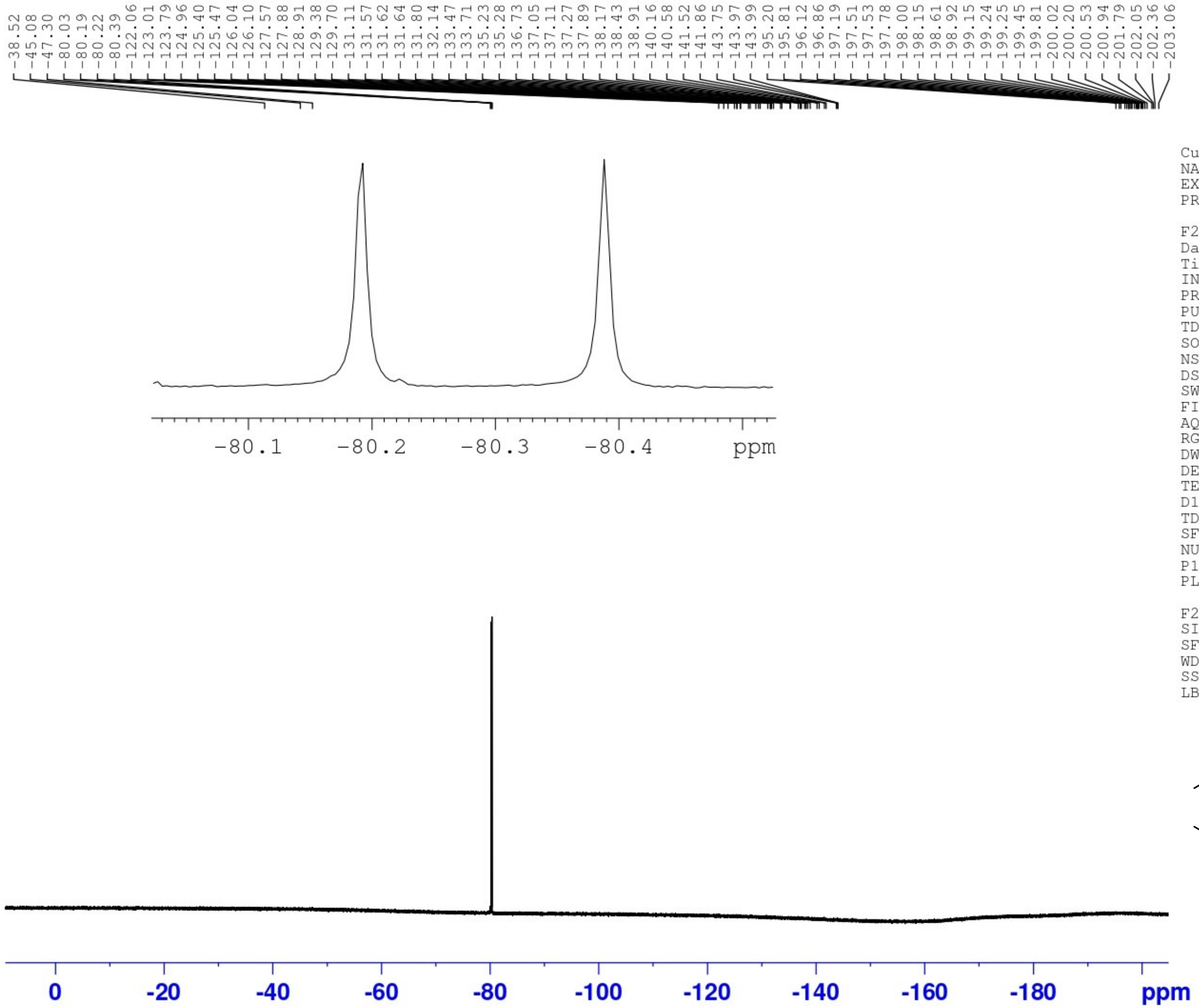

Current Data Parameters
NAME VIII-Mn-67 i_11
EXPNO 2
PROCNO 1

F2 - Acquisition Parameters
Date_ 20250312
Time 15.54
INSTRUM Avance
PROBHD Z166552_0018 (PI HR-
PULPROG zg
TD 131072
SOLVENT CDCl3
NS 16
DS 4
SWH 90909.091
FIDRES 1.387163
AQ 0.7208960
RG 101
DW 5.500
DE 6.50
TE 298.0
D1 1.00000000
TD0 1
SFO1 375.9316815
NUC1 19F
P1 12.00
PLW1 32.47200012

F2 - Processing parameters
SI 65536
SF 375.9692784
WDW EM
SSB 0
LB 0.30

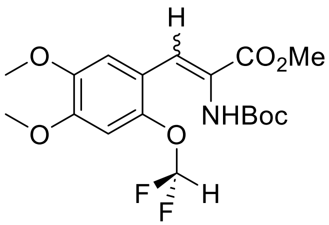

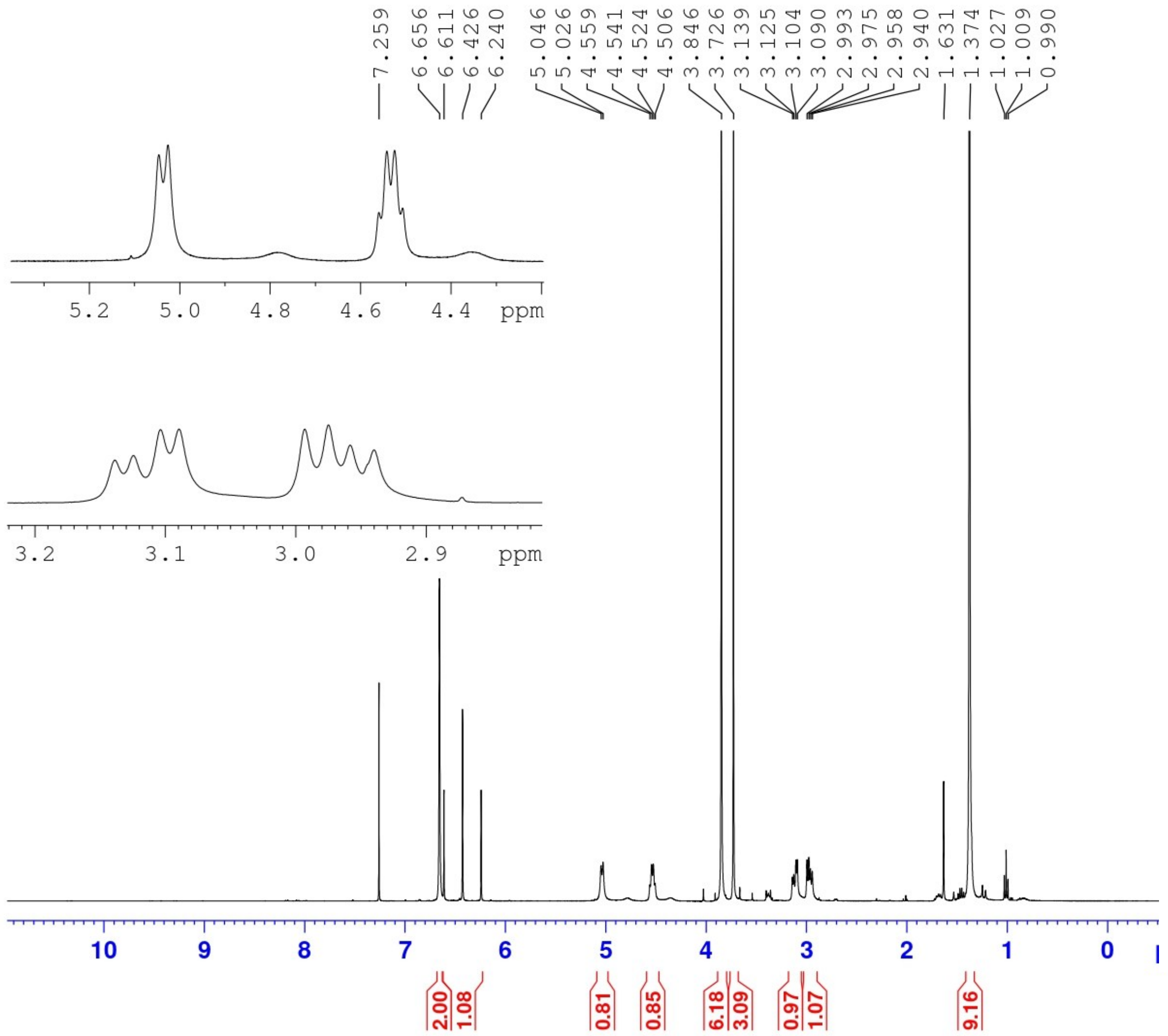

BRUKER

Current Data Parameters
NAME VIII-Mn-70 i_10
EXPNO 3
PROCNO 1

F2 - Acquisition Parameters
Date_ 20250318
Time 18.13
INSTRUM Avance
PROBHD Z166552_0018 (PI HR-
PULPROG zg30
TD 65536
SOLVENT CDCl3
NS 16
DS 2
SWH 7812.500
FIDRES 0.238419
AQ 4.1943040
RG 101
DW 64.000
DE 6.67
TE 298.0
D1 1.00000000
TD0 1
SFO1 399.5701703
NUC1 1H
P0 2.60
P1 7.80
PLW1 21.19799995

F2 - Processing parameters
SI 65536
SF 399.5677131
WDW EM
SSB 0
LB 0.30

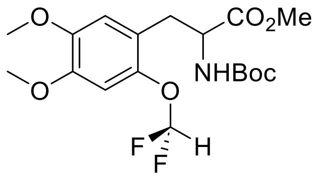

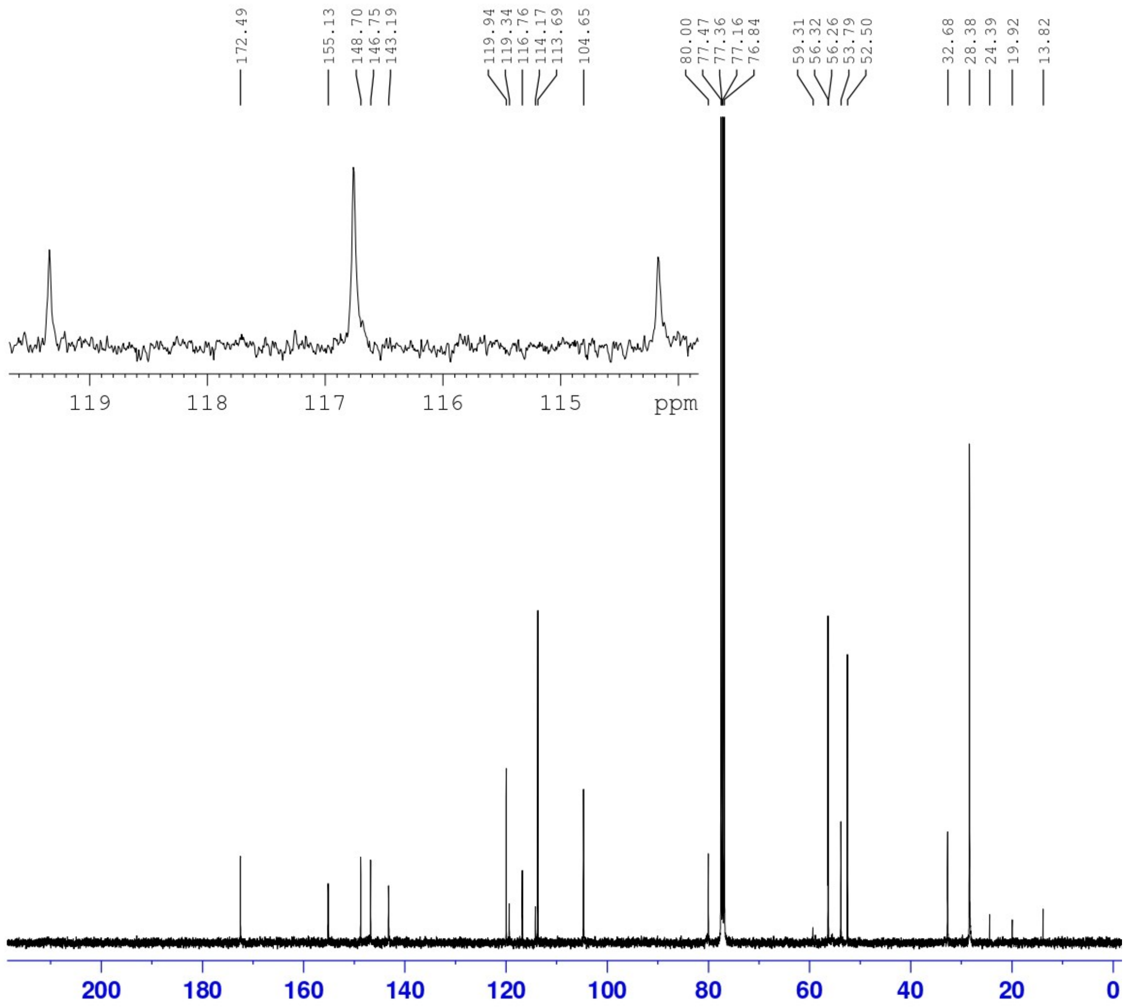

Current Data Parameters
NAME VIII-Mn-70 i_12
EXPNO 3
PROCNO 1

F2 - Acquisition Parameters
Date_ 20250318
Time 20.13
INSTRUM Avance
PROBHD Z166552_0018 (PI HR-
PULPROG zgpg30
TD 65536
SOLVENT CDCl3
NS 2048
DS 4
SWH 23809.524
FIDRES 0.726609
AQ 1.3762560
RG 101
DW 21.000
DE 6.50
TE 298.0
D1 2.00000000
D11 0.03000000
TD0 1
SFO1 100.4814260
NUC1 13C
P0 2.67
P1 8.00
PLW1 88.22599792
SFO2 399.5693013
NUC2 1H
CPDPRG[2] waltz65
PCPD2 90.00
PLW2 21.19799995
PLW12 0.15922000
PLW13 0.08008700

F2 - Processing parameters
SI 32768

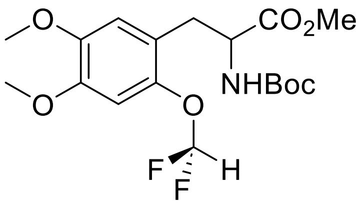

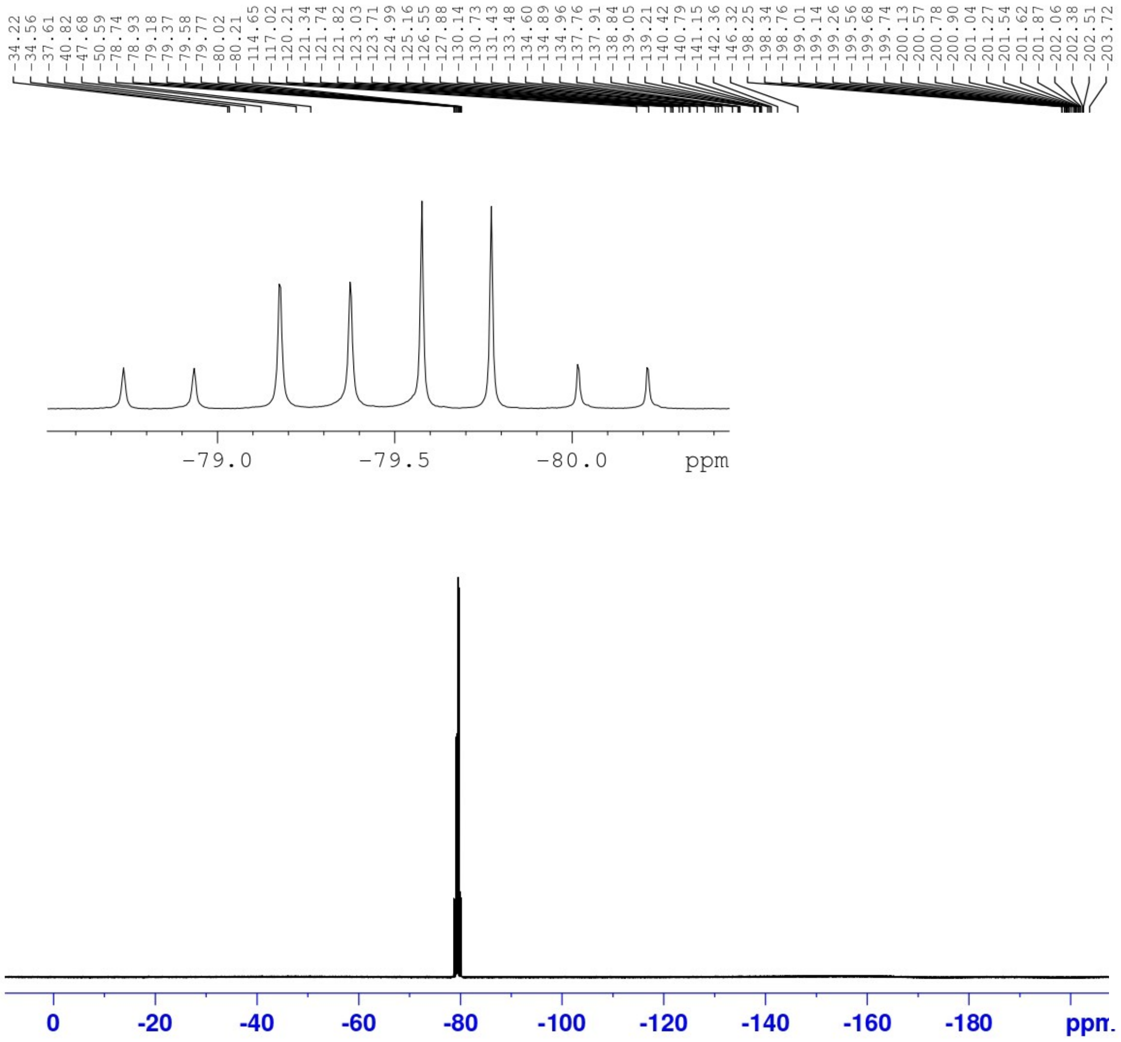

Current Data Parameters
NAME VIII-Mn-70 i_11
EXPNO 3
PROCNO 1

F2 - Acquisition Parameters
Date_ 20250318
Time 18.14
INSTRUM Avance
PROBHD Z166552_0018 (PI HR-
PULPROG zg
TD 131072
SOLVENT CDCl3
NS 16
DS 4
SWH 90909.091
FIDRES 1.387163
AQ 0.7208960
RG 101
DW 5.500
DE 6.50
TE 298.0
D1 1.00000000
TD0 1
SFO1 375.9316815
NUC1 19F
P1 12.00
PLW1 32.47200012

F2 - Processing parameters
SI 65536
SF 375.9692784
WDW EM
SSB 0
LB 0.30
GB 0
PC 1.00

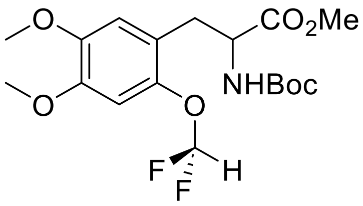

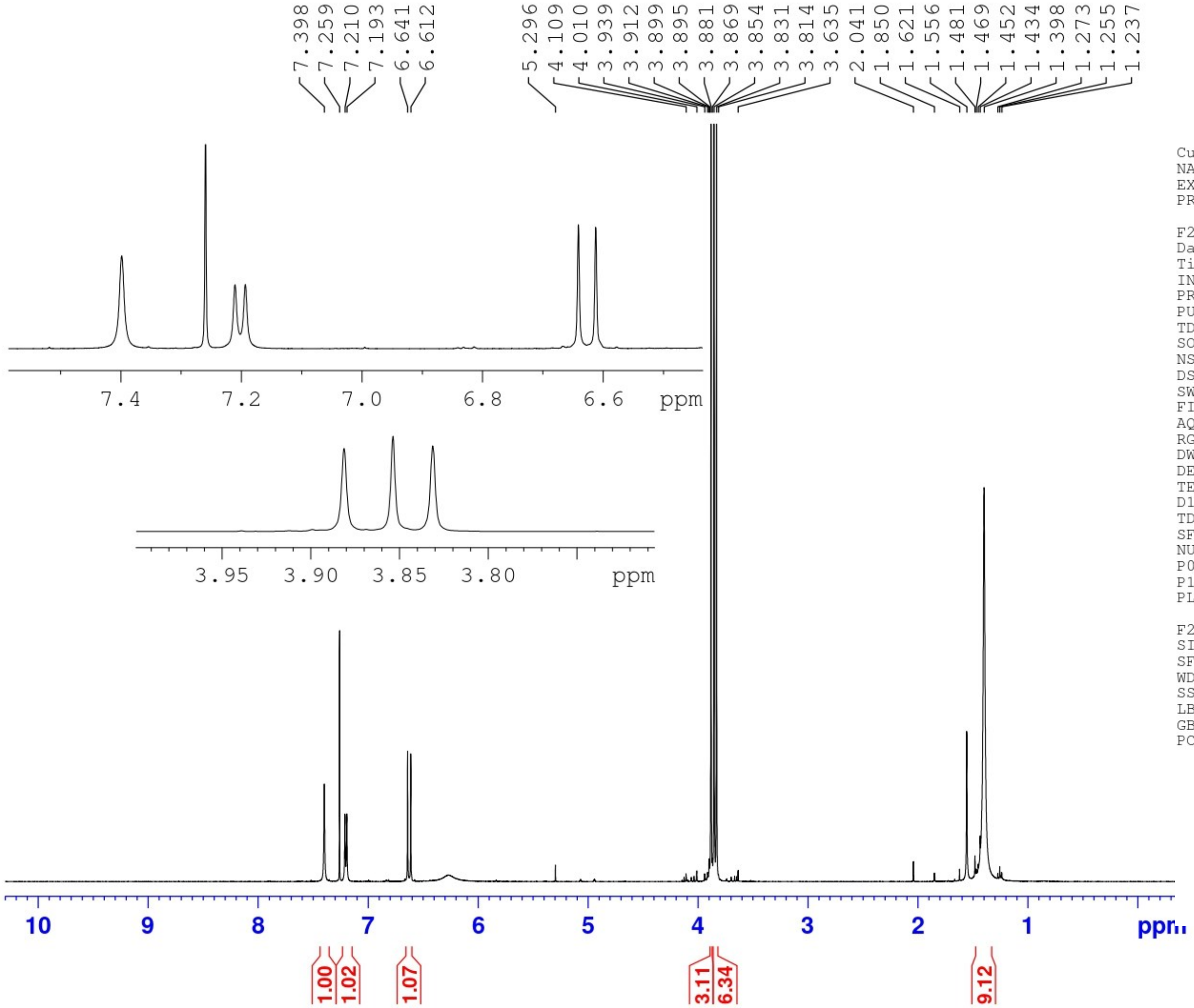
7.398
7.259
7.210
7.193
6.641
6.612
5.296
4.109
4.010
3.939
3.912
3.899
3.895
3.881
3.869
3.854
3.831
3.814
3.635
2.041
1.850
1.621
1.556
1.481
1.469
1.452
1.434
1.398
1.273
1.255
1.237
7.4
7.2
7.0
6.8
6.6
ppm
3.95
3.90
3.85
3.80
ppm
10
9
8
7
6
5
4
3
2
1
ppm
1.00
1.02
1.07
3.11
6.34
9.12

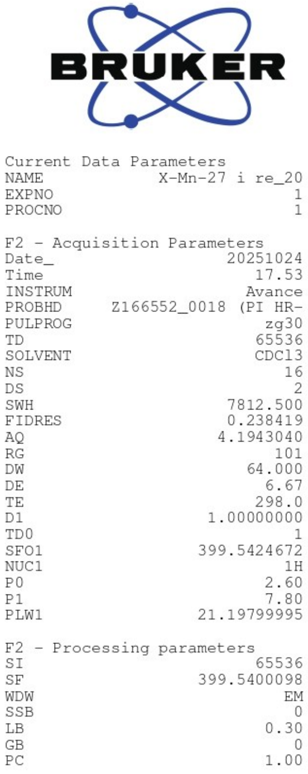
BRUKER
Current Data Parameters
NAME X-Mn-27 i re_20
EXPNO 1
PROCNO 1
F2 - Acquisition Parameters
Date_ 20251024
Time 17.53
INSTRUM Avance
PROBHD Z166552_0018 (PI HR-
PULPROG zg30
TD 65536
SOLVENT CDCl3
NS 16
DS 2
SWH 7812.500
FIDRES 0.238419
AQ 4.1943040
RG 101
DW 64.000
DE 6.67
TE 298.0
D1 1.00000000
TD0 1
SFO1 399.5424672
NUC1 1H
P0 2.60
P1 7.80
PLW1 21.19799995
F2 - Processing parameters
SI 65536
SF 399.5400098
WDW EM
SSB 0
LB 0.30
GB 0
PC 1.00

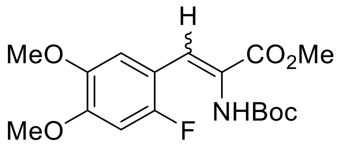
H
MeO
CO2Me
NHBoc
MeO
F

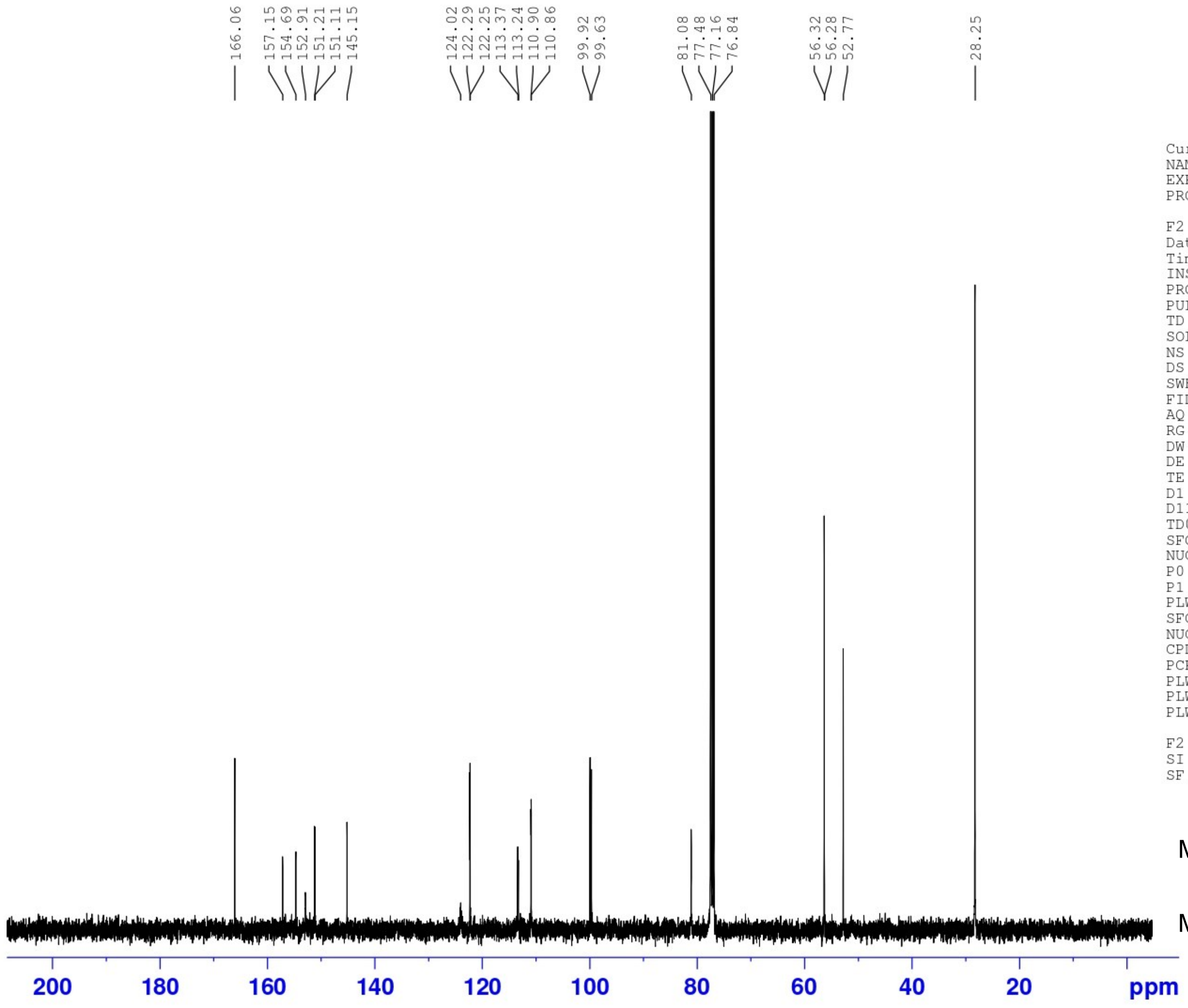


```
Current Data Parameters
NAME                X-Mn-27 i_12
EXPNO                          2
PROCNO                         1

F2 - Acquisition Parameters
Date_                   20251022
Time                       17.06
INSTRUM                   Avance
PROBHD      Z166552_0018 (PI HR-
PULPROG                  zgpg30
TD                         65536
SOLVENT                    CDCl3
NS                           516
DS                             4
SWH                    23809.524
FIDRES                  0.726609
AQ                     1.3762560
RG                           101
DW                        21.000
DE                          6.50
TE                         298.0
D1                    2.00000000
D11                   0.03000000
TD0                            1
SFO1                 100.4744593
NUC1                         13C
P0                          2.67
P1                          8.00
PLW1                 88.22599792
SFO2                 399.5415982
NUC2                          1H
CPDPRG[2]                waltz65
PCPD2                      90.00
PLW2                 21.19799995
PLW12                 0.15922000
PLW13                 0.08008700

F2 - Processing parameters
SI                         32768
SF                   100.4643998
```

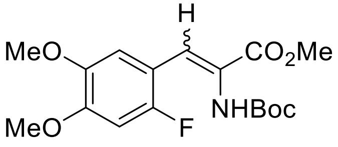

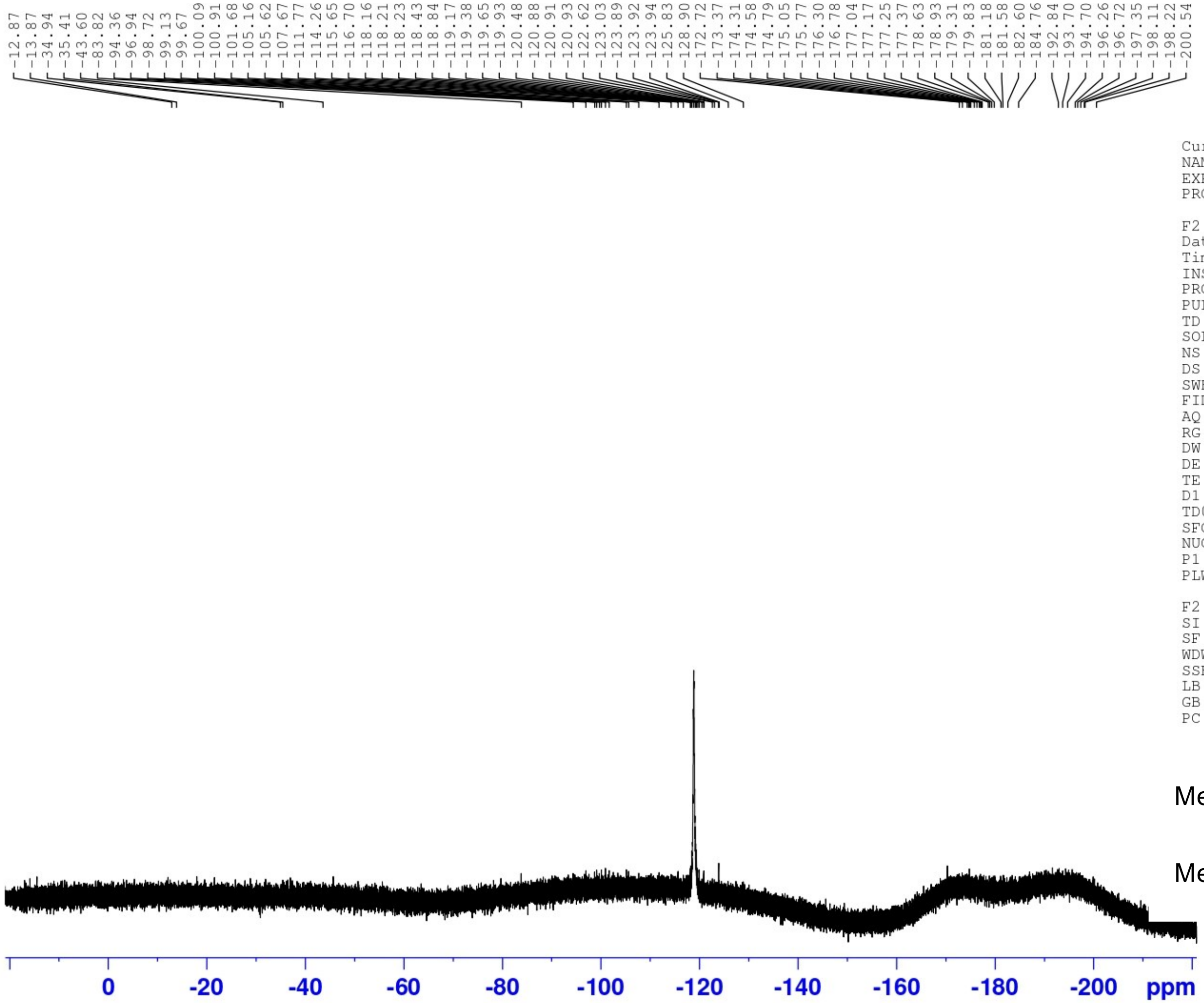

Current Data Parameters
NAME X-Mn-27 i re_21
EXPNO 1
PROCNO 1

F2 - Acquisition Parameters
Date_ 20251024
Time 17.55
INSTRUM Avance
PROBHD Z166552_0018 (PI HR-
PULPROG zg
TD 131072
SOLVENT CDCl3
NS 16
DS 4
SWH 90909.091
FIDRES 1.387163
AQ 0.7208960
RG 101
DW 5.500
DE 6.50
TE 298.0
D1 1.00000000
TD0 1
SFO1 375.9056172
NUC1 19F
P1 12.00
PLW1 32.47200012

F2 - Processing parameters
SI 65536
SF 375.9432115
WDW EM
SSB 0
LB 0.30
GB 0
PC 1.00

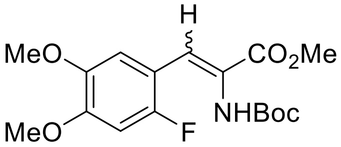

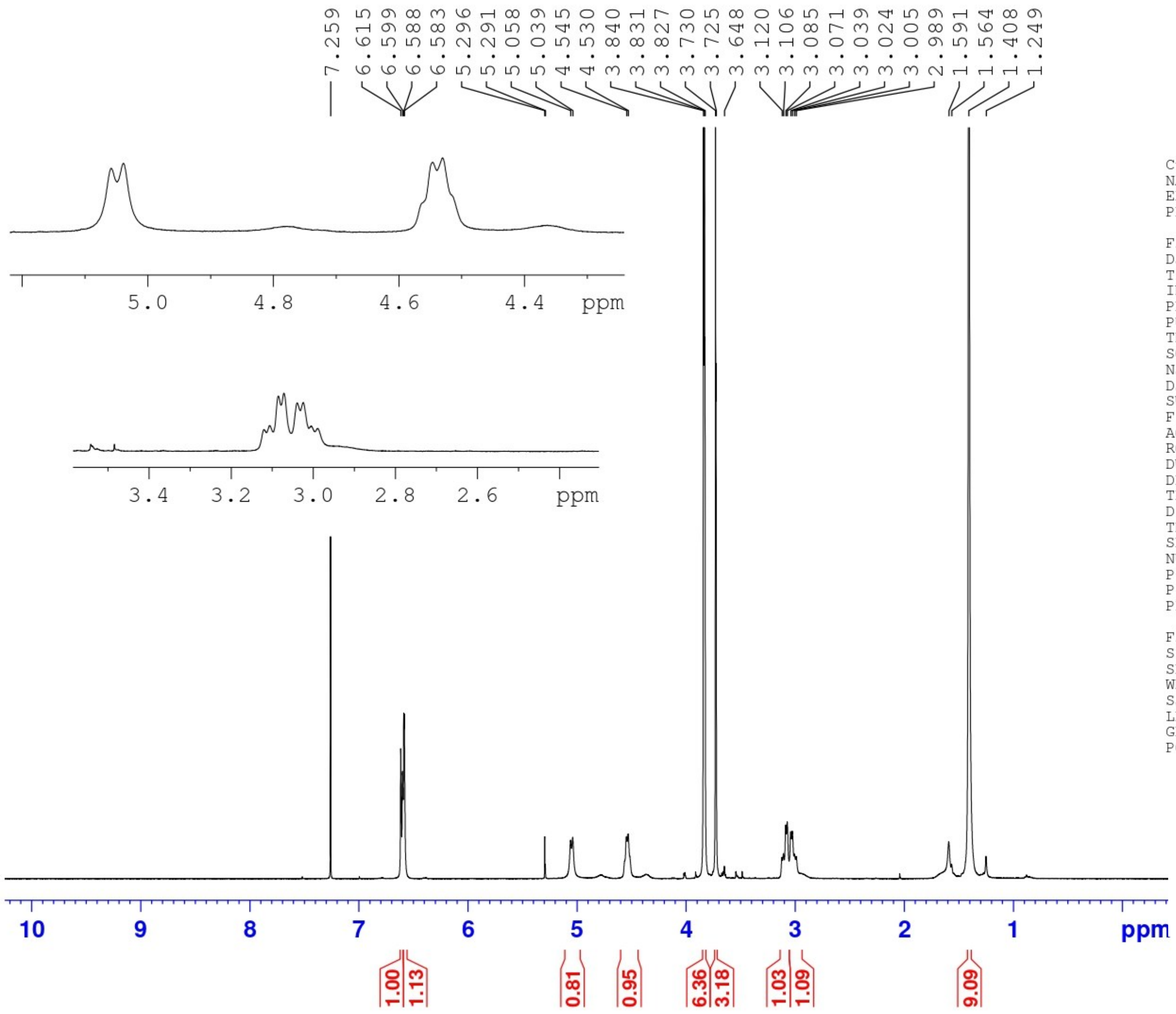

BRUKER

Current Data Parameters
NAME X-Mn-28 i_20
EXPNO 1
PROCNO 1

F2 - Acquisition Parameters
Date_ 20251024
Time 18.02
INSTRUM Avance
PROBHD Z166552_0018 (PI HR-
PULPROG zg30
TD 65536
SOLVENT CDCl3
NS 16
DS 2
SWH 7812.500
FIDRES 0.238419
AQ 4.1943040
RG 101
DW 64.000
DE 6.67
TE 298.0
D1 1.00000000
TD0 1
SFO1 399.5424672
NUC1 1H
P0 2.60
P1 7.80
PLW1 21.19799995

F2 - Processing parameters
SI 65536
SF 399.5400099
WDW EM
SSB 0
LB 0.30
GB 0
PC 1.00

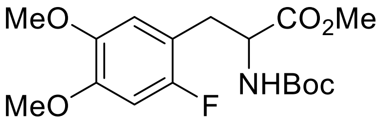

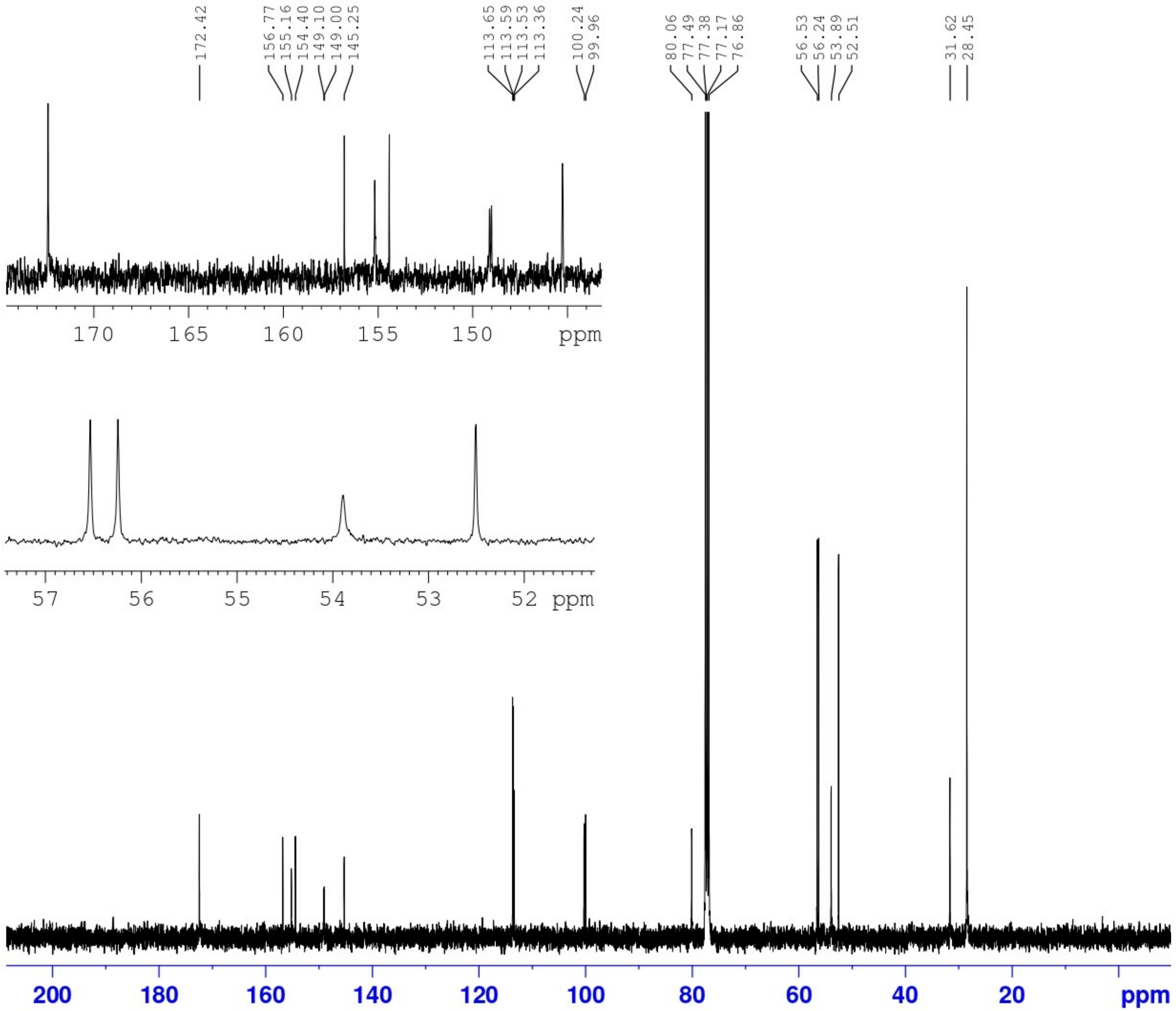


```
Current Data Parameters
NAME                X-Mn-28 i_22
EXPNO                          1
PROCNO                         1

F2 - Acquisition Parameters
Date_                   20251024
Time                       20.02
INSTRUM                   Avance
PROBHD     Z166552_0018 (PI HR-
PULPROG                  zgpg30
TD                         65536
SOLVENT                    CDCl3
NS                          2048
DS                             4
SWH                    23809.524
FIDRES                  0.726609
AQ                     1.3762560
RG                           101
DW                        21.000
DE                          6.50
TE                         298.0
D1                    2.00000000
D11                   0.03000000
TD0                            1
SFO1                 100.4744593
NUC1                         13C
P0                          2.67
P1                          8.00
PLW1                 88.22599792
SFO2                 399.5415982
NUC2                          1H
CPDPRG[2]                waltz65
PCPD2                      90.00
PLW2                 21.19799995
PLW12                 0.15922000
PLW13                 0.08008700

F2 - Processing parameters
SI                         32768
SF                   100.4643980
WDW                           EM
SSB                            0
```

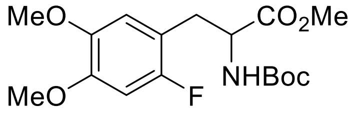

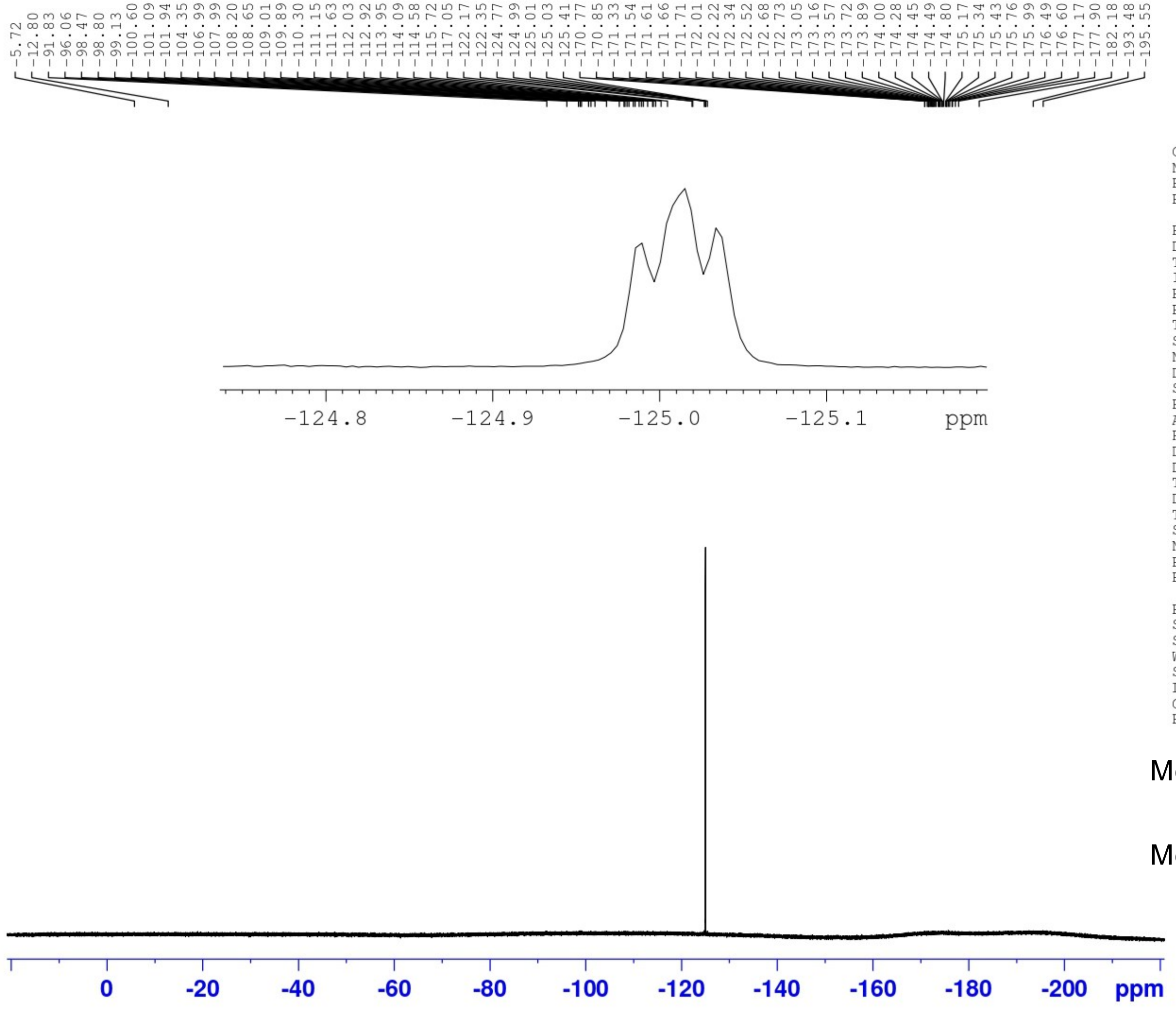

BRUKER

Current Data Parameters
NAME X-Mn-28 i_21
EXPNO 1
PROCNO 1

F2 - Acquisition Parameters
Date_ 20251024
Time 18.03
INSTRUM Avance
PROBHD Z166552_0018 (PI HR-
PULPROG zg
TD 131072
SOLVENT CDCl3
NS 16
DS 4
SWH 90909.091
FIDRES 1.387163
AQ 0.7208960
RG 101
DW 5.500
DE 6.50
TE 298.0
D1 1.00000000
TD0 1
SFO1 375.9056172
NUC1 19F
P1 12.00
PLW1 32.47200012

F2 - Processing parameters
SI 65536
SF 375.9432115
WDW EM
SSB 0
LB 0.30
GB 0
PC 1.00

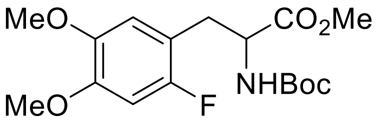

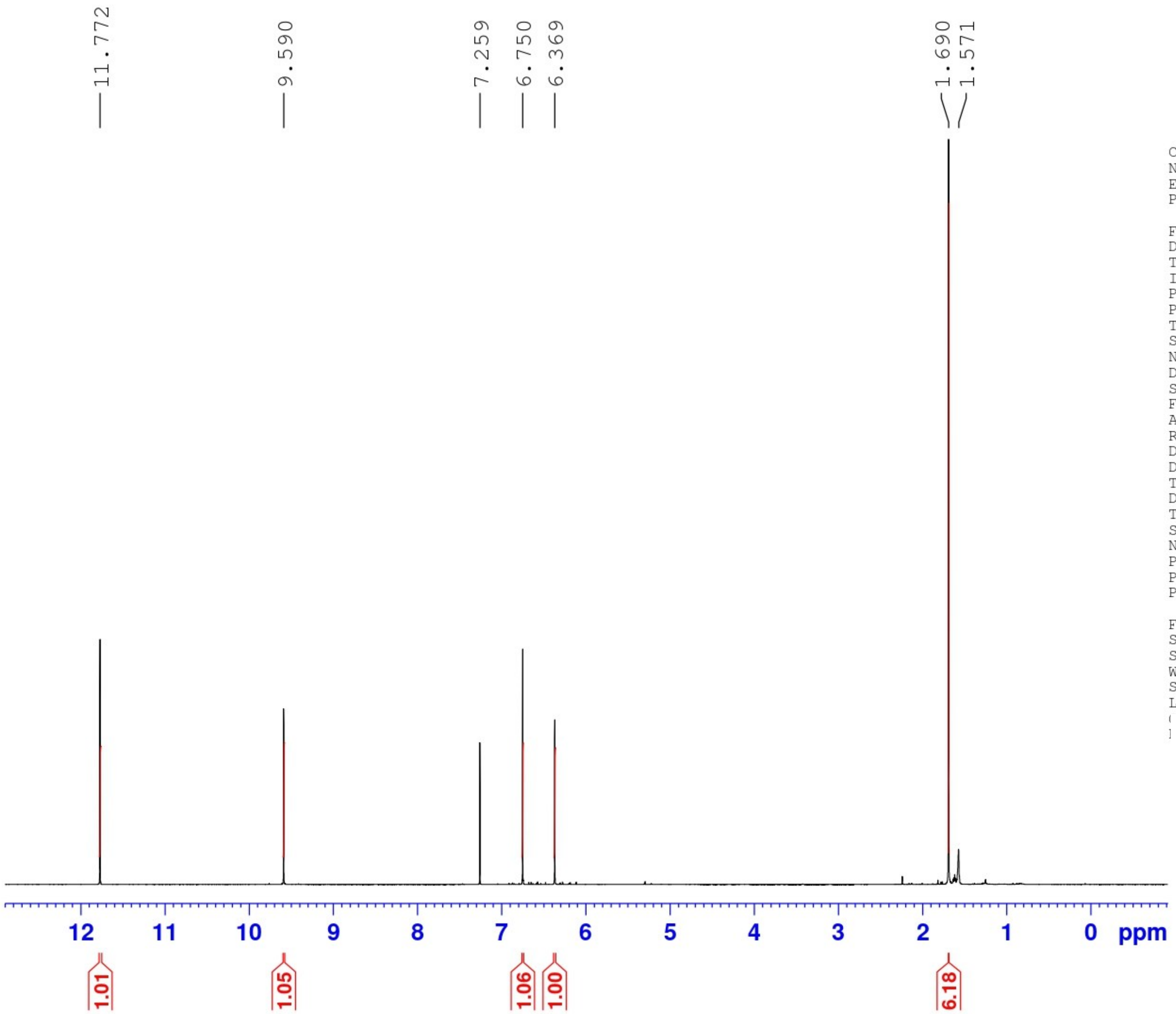

Current Data Parameters
NAME IX-Mn-01 i_10
EXPNO 3
PROCNO 1

F2 - Acquisition Parameters

Date_	20250507
Time	13.07
INSTRUM	AS500-NEO
PROBHD	Z168772_0026 (CPP1.1
PULPROG	zg30
TD	65536
SOLVENT	CDCl3
NS	16
DS	2
SWH	10000.000
FIDRES	0.305176
AQ	3.2767999
RG	57
DW	50.000
DE	10.45
TE	298.0
D1	1.00000000
TD0	1
SFO1	499.7860862
NUC1	1H
P0	4.00
P1	12.00
PLW1	16.91500092

F2 - Processing parameters

SI	65536
SF	499.7830124
WDW	EM
SSB	0
LB	0.30
[illegible]	0
[illegible]	[illegible].00

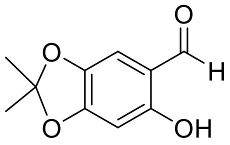

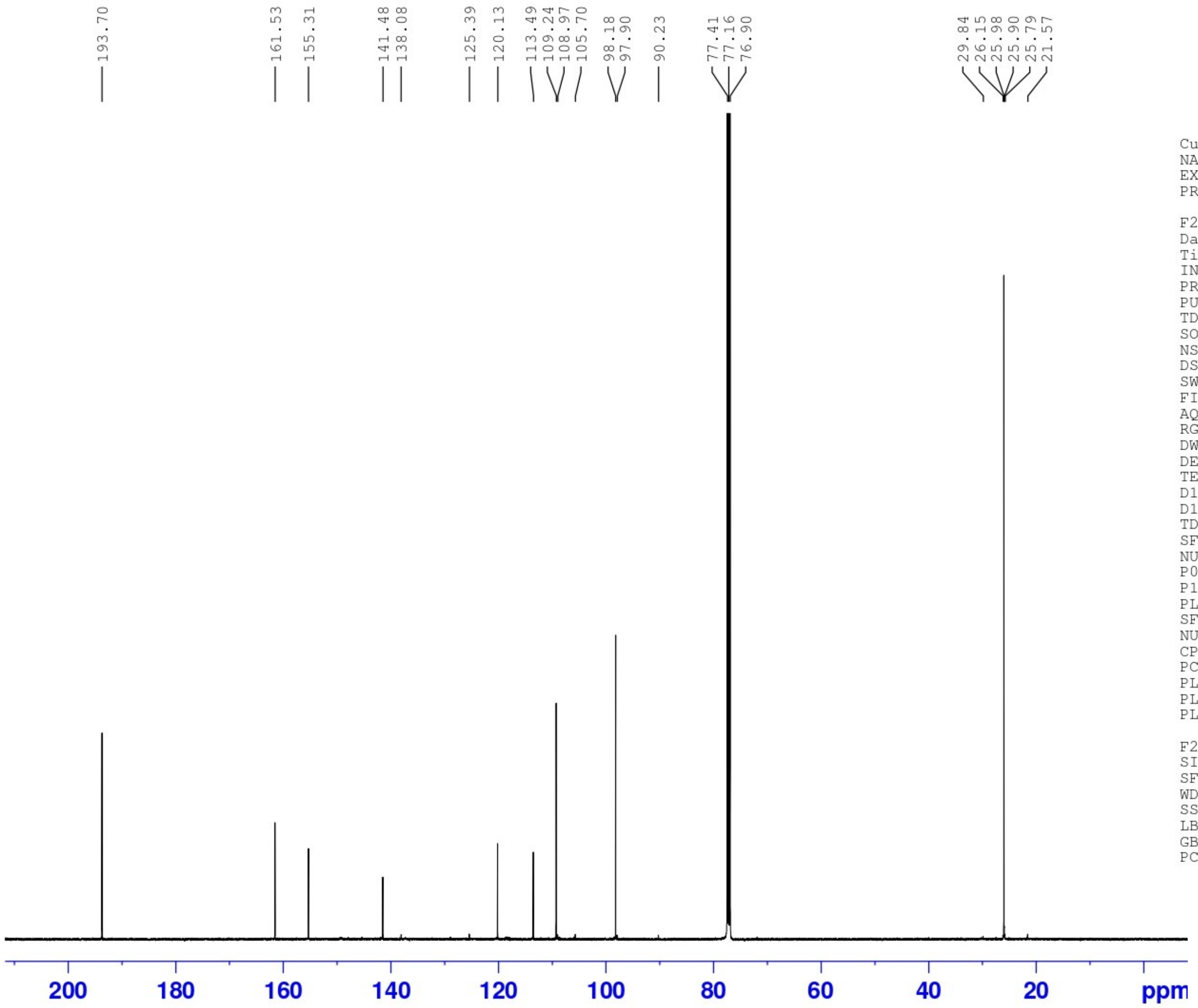

Current Data Parameters
NAME IX-Mn-01 i_12
EXPNO 2
PROCNO 1

F2 - Acquisition Parameters
Date_ 20250508
Time 23.41
INSTRUM AS500-NEO
PROBHD Z168772_0026 (CPP1.1
PULPROG zgpg30
TD 65536
SOLVENT CDCl3
NS 2048
DS 4
SWH 30120.482
FIDRES 0.919204
AQ 1.0878977
RG 101
DW 16.600
DE 18.00
TE 298.0
D1 2.00000000
D11 0.03000000
TD0 1
SFO1 125.6831024
NUC1 13C
P0 3.33
P1 10.00
PLW1 59.16400146
SFO2 499.7849991
NUC2 1H
CPDPRG[2] waltz65
PCPD2 80.00
PLW2 16.91500092
PLW12 0.38058999
PLW13 0.19113000

F2 - Processing parameters
SI 32768
SF 125.6705186
WDW EM
SSB 0
LB 1.00
GB 0
PC 1.40

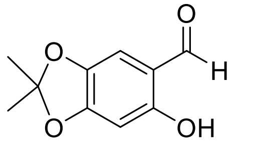

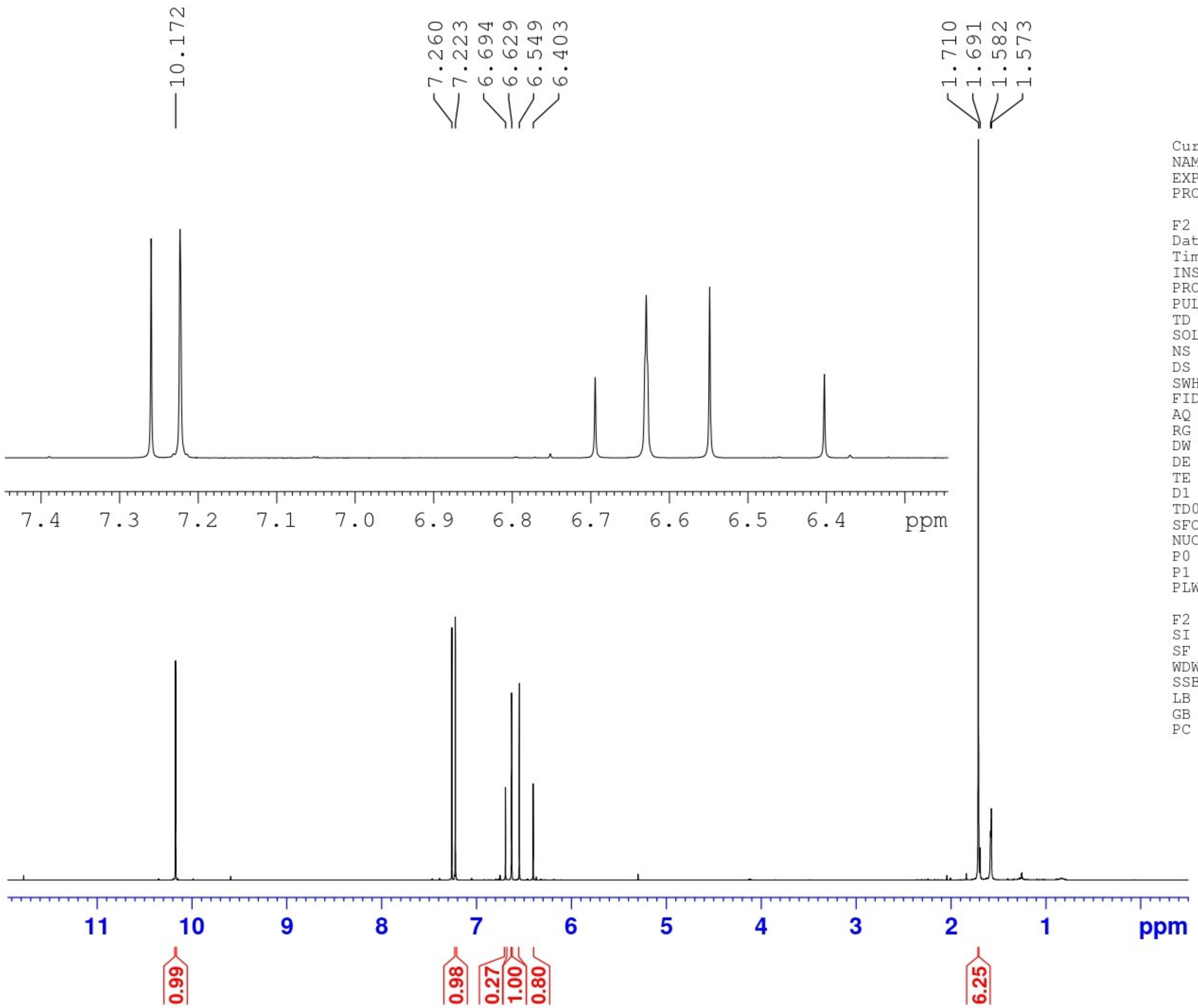

Current Data Parameters
NAME IX-Mn-02 i_10
EXPNO 3
PROCNO 1

F2 - Acquisition Parameters
Date_ 20250507
Time 13.13
INSTRUM AS500-NEO
PROBHD Z168772_0026 (CPP1.1
PULPROG zg30
TD 65536
SOLVENT CDCl3
NS 16
DS 2
SWH 10000.000
FIDRES 0.305176
AQ 3.2767999
RG 45.2
DW 50.000
DE 10.45
TE 298.0
D1 1.00000000
TD0 1
SFO1 499.7860862
NUC1 1H
P0 4.00
P1 12.00
PLW1 16.91500092

F2 - Processing parameters
SI 65536
SF 499.7830117
WDW EM
SSB 0
LB 0.30
GB 0
PC 1.00

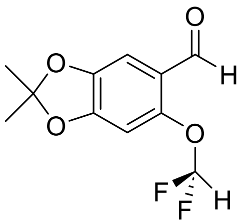

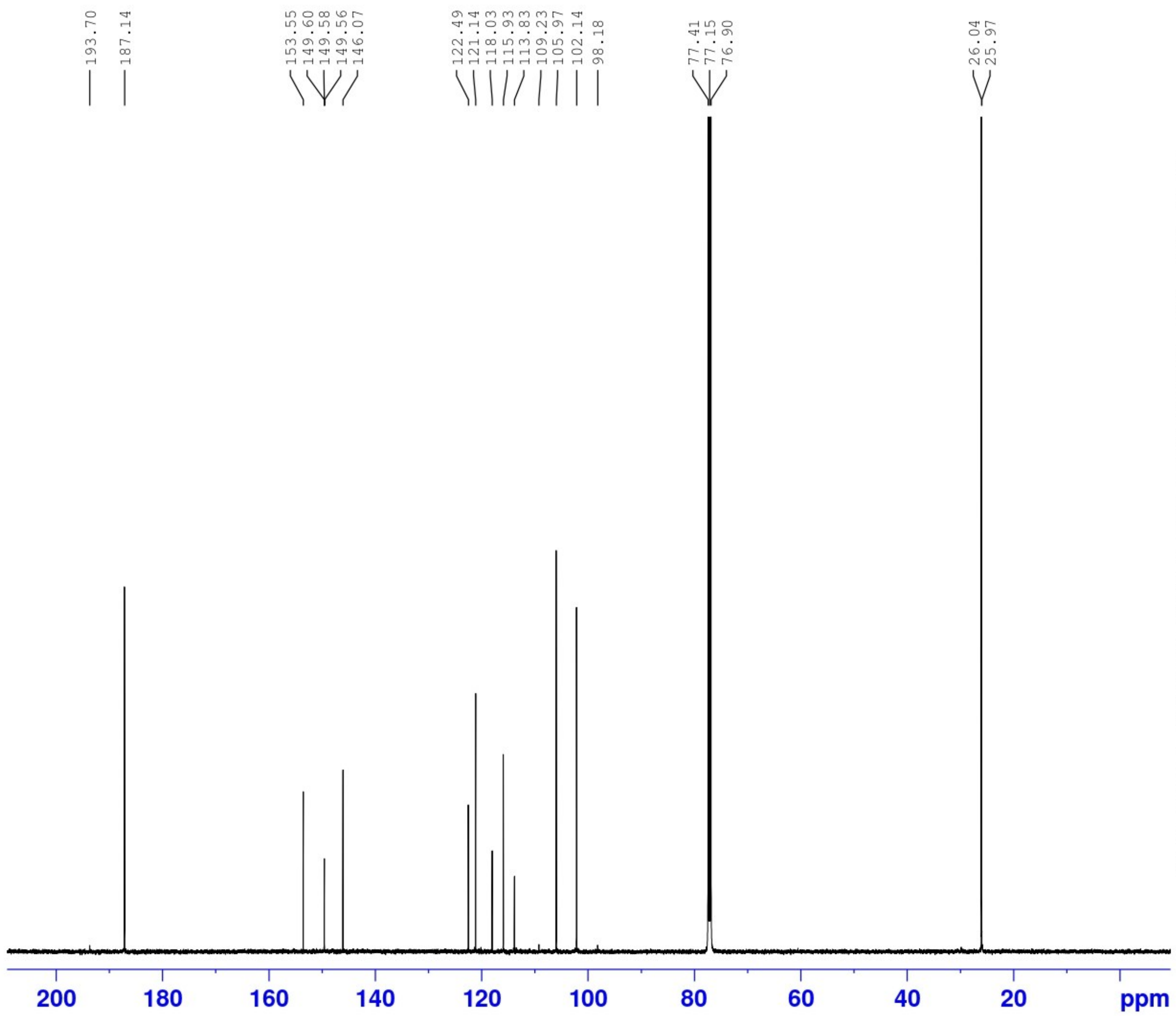
193.70
187.14
153.55
149.60
149.58
149.56
146.07
122.49
121.14
118.03
115.93
113.83
109.23
105.97
102.14
98.18
77.41
77.15
76.90
26.04
25.97
200
180
160
140
120
100
80
60
40
20
ppm
BRUKER
Current Data Parameters
NAME IX-Mn-02 i_12
EXPNO 1
PROCNO 1
F2 - Acquisition Parameters
Date_ 20250509
Time 1.34
INSTRUM AS500-NEO
PROBHD Z168772_0026 (CPP1.1
PULPROG zgpg30
TD 65536
SOLVENT CDCl3
NS 2048
DS 4
SWH 30120.482
FIDRES 0.919204
AQ 1.0878977
RG 101
DW 16.600
DE 18.00
TE 298.0
D1 2.00000000
D11 0.03000000
TD0 1
SFO1 125.6831024
NUC1 13C
P0 3.33
P1 10.00
PLW1 59.16400146
SFO2 499.7849991
NUC2 1H
CPDPRG[2] waltz65
PCPD2 80.00
PLW2 16.91500092
PLW12 0.38058999
PLW13 0.19113000
F2 - Processing parameters
SI 32768
SF 125.6705188
WDW EM

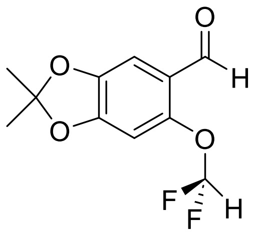
O
H
O
O
O
F
F
H

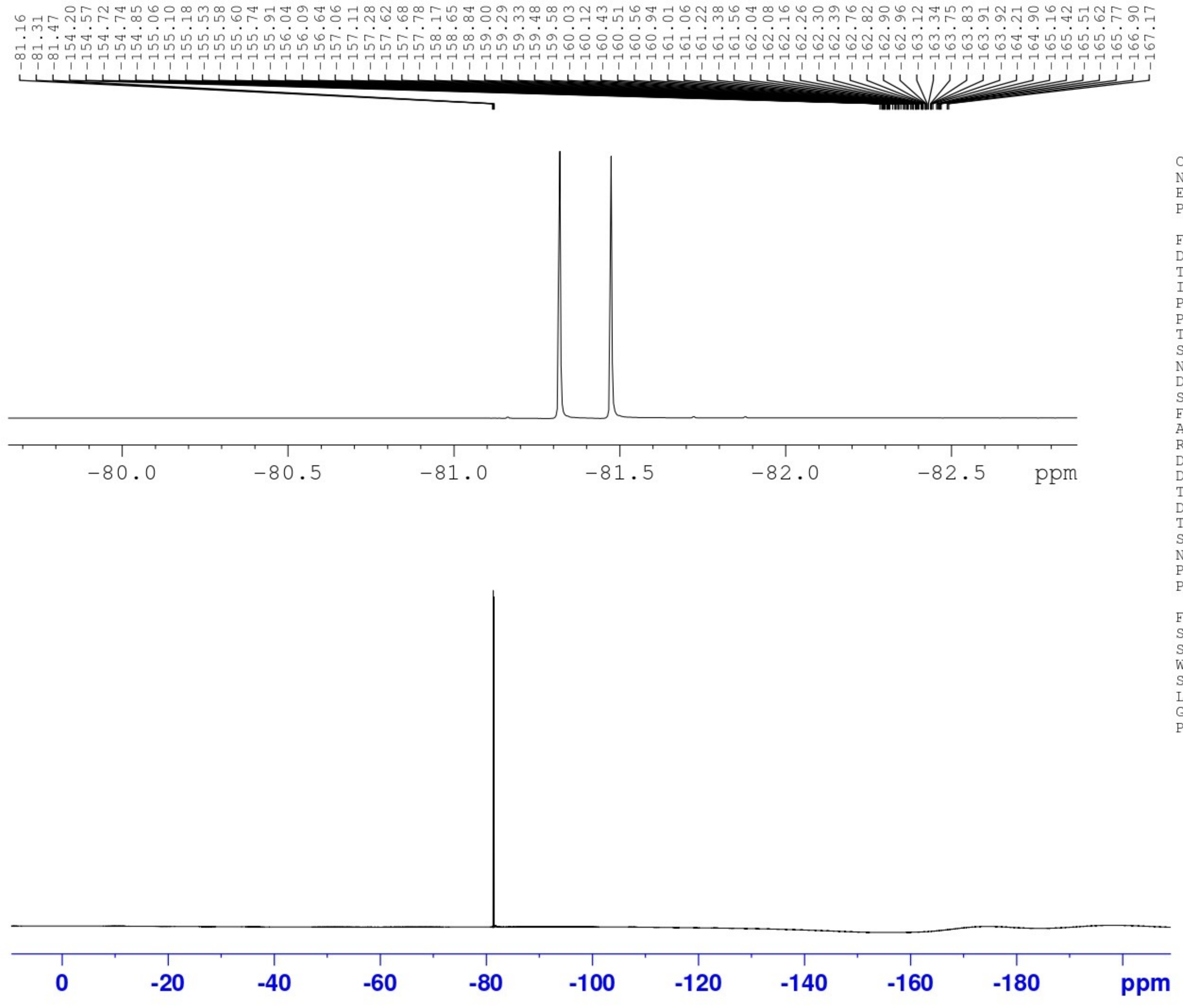

BRUKER

Current Data Parameters
NAME IX-Mn-02 i_11
EXPNO 3
PROCNO 1

F2 - Acquisition Parameters
Date_ 20250507
Time 13.15
INSTRUM AS500-NEO
PROBHD Z168772_0026 (CPP1.1
PULPROG zg
TD 131072
SOLVENT CDCl3
NS 16
DS 4
SWH 113636.364
FIDRES 1.733953
AQ 0.5767168
RG 11.3
DW 4.400
DE 18.00
TE 298.0
D1 1.00000000
TD0 1
SFO1 470.2188444
NUC1 19F
P1 15.00
PLW1 10.89000034

F2 - Processing parameters
SI 65536
SF 470.2658710
WDW EM
SSB 0
LB 0.30
GB 0
PC 1.00

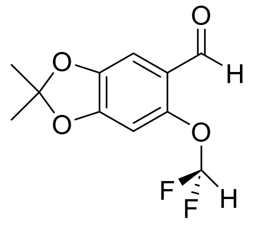

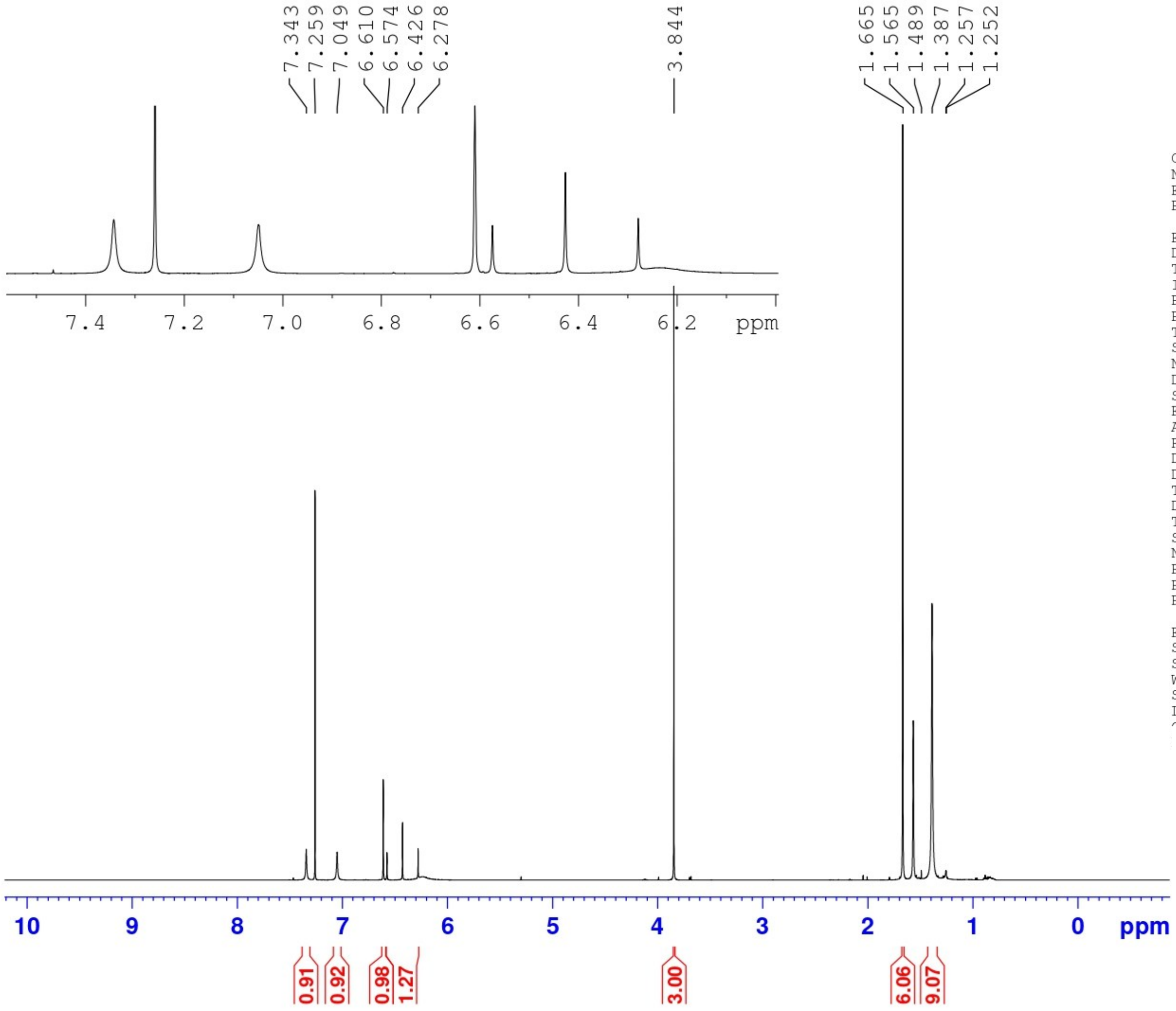


```
Current Data Parameters
NAME            IX-Mn-03 i_10
EXPNO                       2
PROCNO                      1

F2 - Acquisition Parameters
Date_                20250509
Time                    16.42
INSTRUM             AS500-NEO
PROBHD     Z168772_0026 (CPP1.1
PULPROG                  zg30
TD                      65536
SOLVENT                 CDCl3
NS                         16
DS                          2
SWH                 10000.000
FIDRES               0.305176
AQ                  3.2767999
RG                       45.2
DW                     50.000
DE                      10.45
TE                      298.0
D1                 1.00000000
TD0                         1
SFO1              499.7860862
NUC1                       1H
P0                       4.00
P1                      12.00
PLW1              16.91500092

F2 - Processing parameters
SI                      65536
SF                499.7830121
WDW                        EM
SSB                         0
LB                       0.30
```

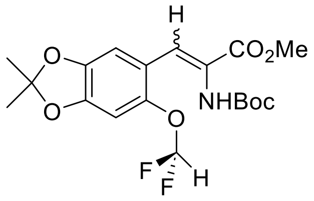

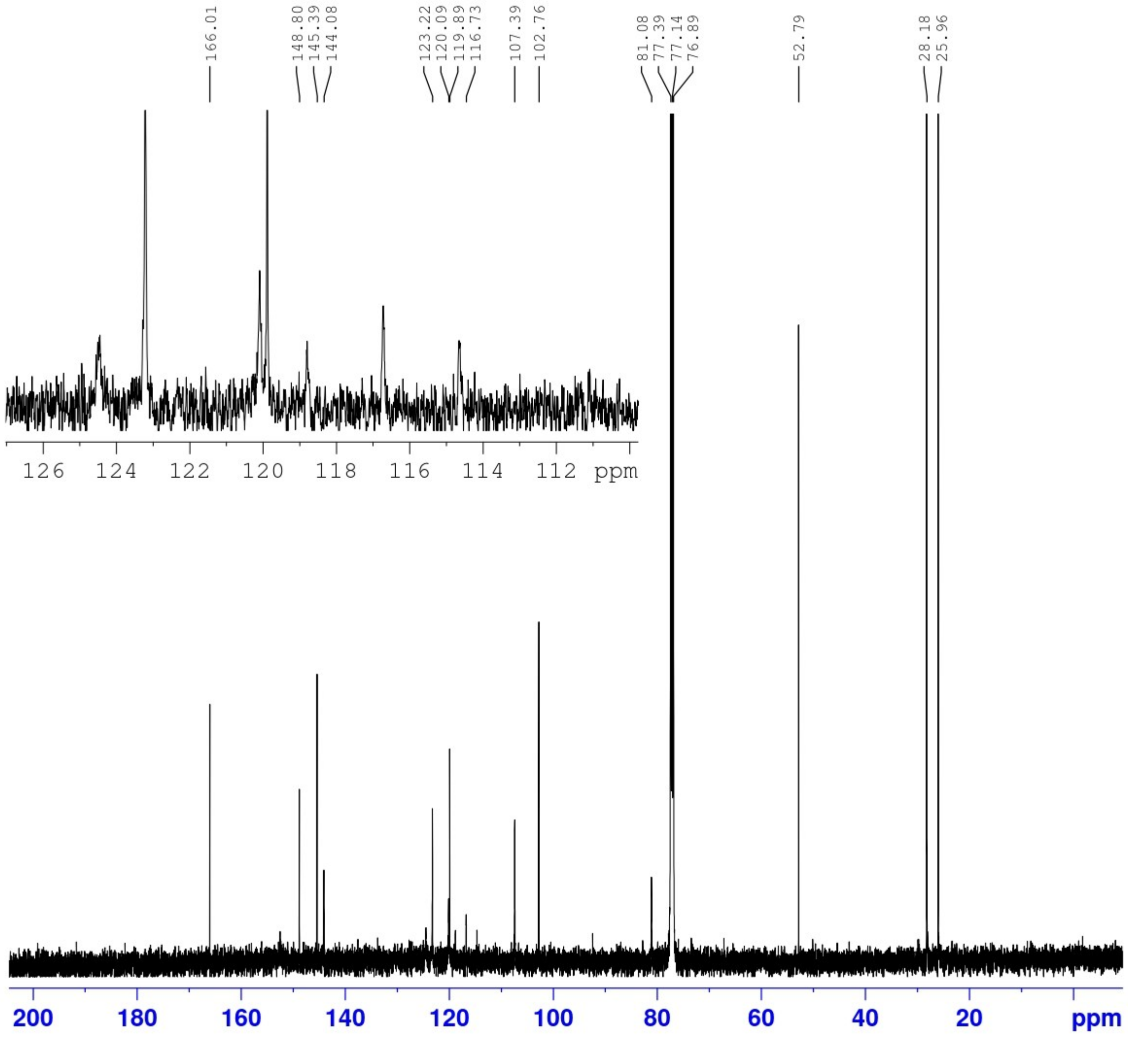

Current Data Parameters
NAME IX-Mn-03 i_12
EXPNO 2
PROCNO 1

F2 - Acquisition Parameters
Date_ 20250510
Time 11.42
INSTRUM AS500-NEO
PROBHD Z168772_0026 (CPP1.1
PULPROG zgpg30
TD 65536
SOLVENT CDCl3
NS 1024
DS 4
SWH 30120.482
FIDRES 0.919204
AQ 1.0878977
RG 101
DW 16.600
DE 18.00
TE 298.0
D1 2.00000000
D11 0.03000000
TD0 1
SFO1 125.6831024
NUC1 13C
P0 3.33
P1 10.00
PLW1 59.16400146
SFO2 499.7849991
NUC2 1H
CPDPRG[2] waltz65
PCPD2 80.00
PLW2 16.91500092
PLW12 0.38058999
PLW13 0.19113000

F2 - Processing parameters
SI 32768
SF 125.6705200
WDW EM
SSB 0
LB 1.00
GB 0
)

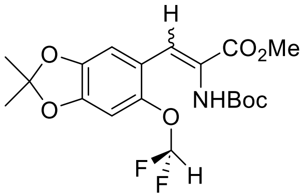

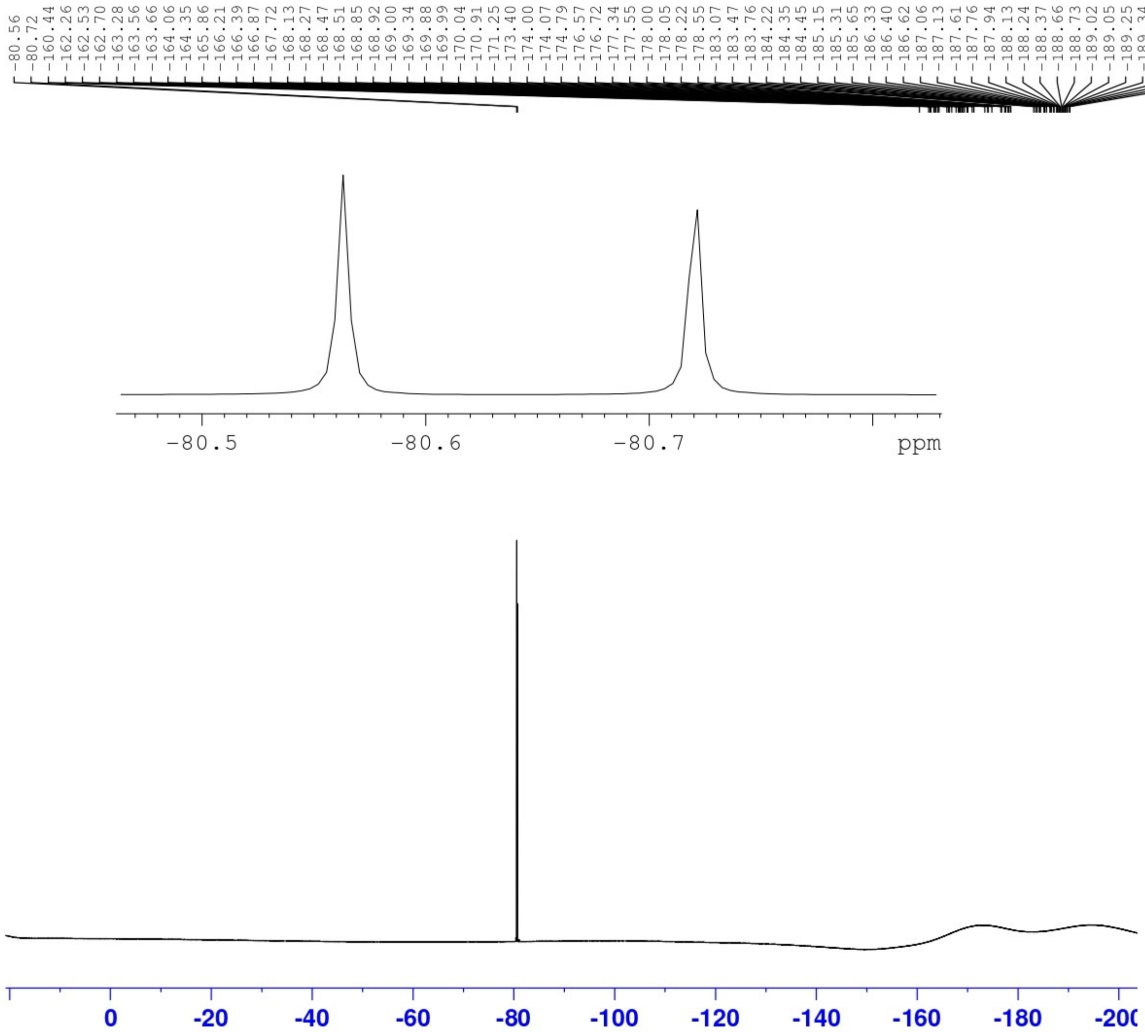

Current Data Parameters	
NAME	IX-Mn-03 i_11
EXPNO	2
PROCNO	1
**F2 - Acquisition Parameters**	
Date_	20250509
Time	16.43
INSTRUM	AS500-NEO
PROBHD	Z168772_0026 (CPP1.1
PULPROG	zg
TD	131072
SOLVENT	CDCl3
NS	16
DS	4
SWH	113636.364
FIDRES	1.733953
AQ	0.5767168
RG	11.3
DW	4.400
DE	18.00
TE	298.0
D1	1.00000000
TD0	1
SFO1	470.2188444
NUC1	19F
P1	15.00
PLW1	10.89000034
**F2 - Processing parameters**	
SI	65536
SF	470.2658710
WDW	EM
SSB	0
LB	0.30
GB	0
PC	1.00

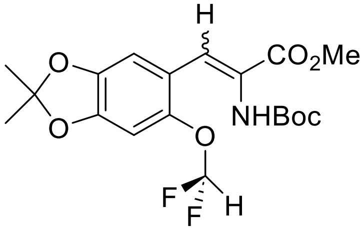

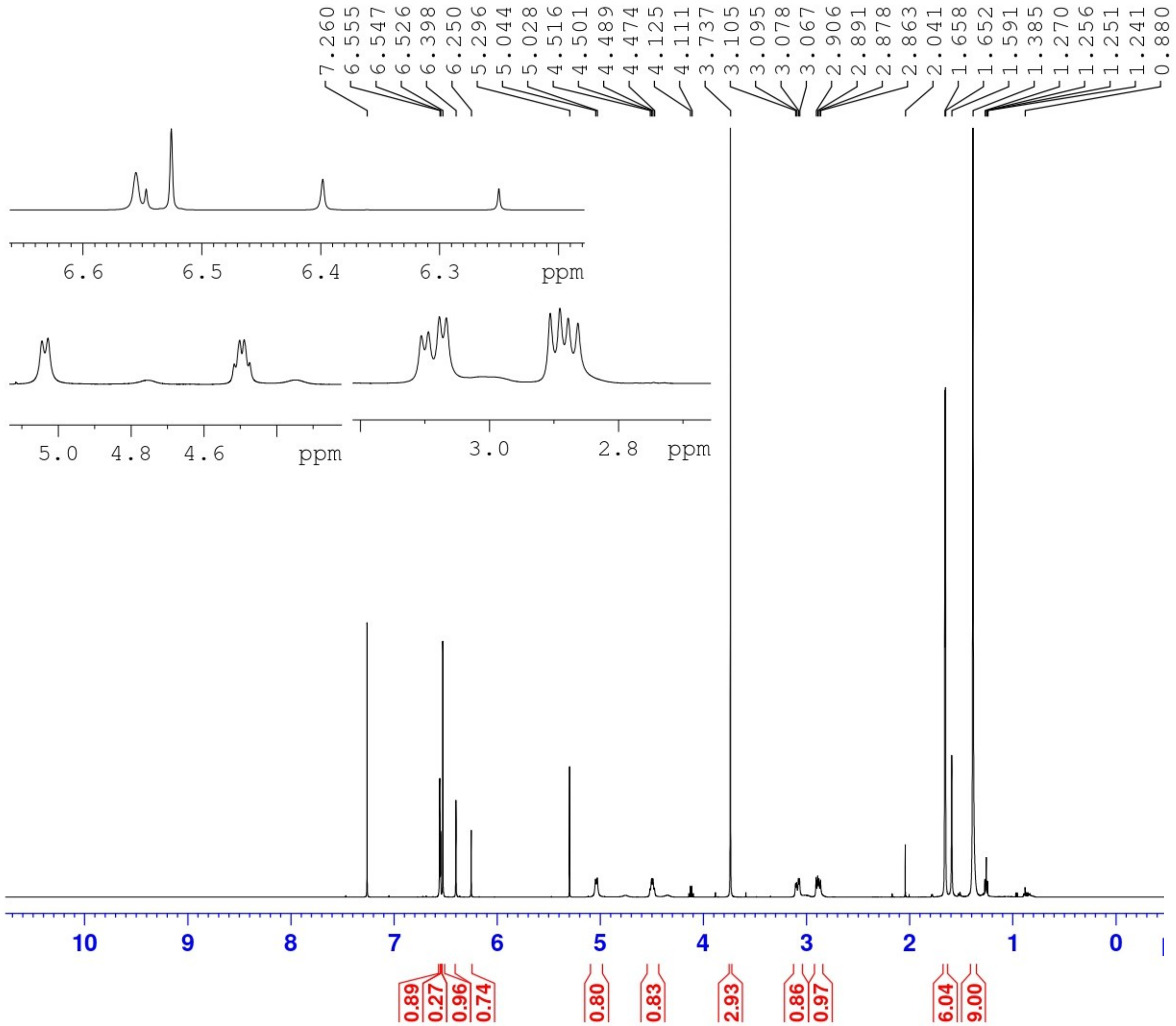

BRUKER

Current Data Parameters
NAME IX-Mn-04 i_10
EXPNO 2
PROCNO 1

F2 - Acquisition Parameters
Date_ 20250509
Time 16.48
INSTRUM AS500-NEO
PROBHD Z168772_0026 (CPP1.1
PULPROG zg30
TD 65536
SOLVENT CDCl3
NS 16
DS 2
SWH 10000.000
FIDRES 0.305176
AQ 3.2767999
RG 45.2
DW 50.000
DE 10.45
TE 298.0
D1 1.00000000
TD0 1
SFO1 499.7860862
NUC1 1H
P0 4.00
P1 12.00
PLW1 16.91500092

F2 - Processing parameters
SI 65536
SF 499.7830117
WDW EM
SSB 0
LB 0.30
GB 0
PC 1.00

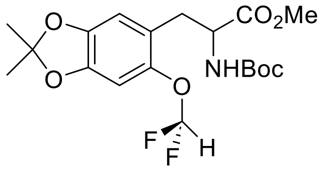

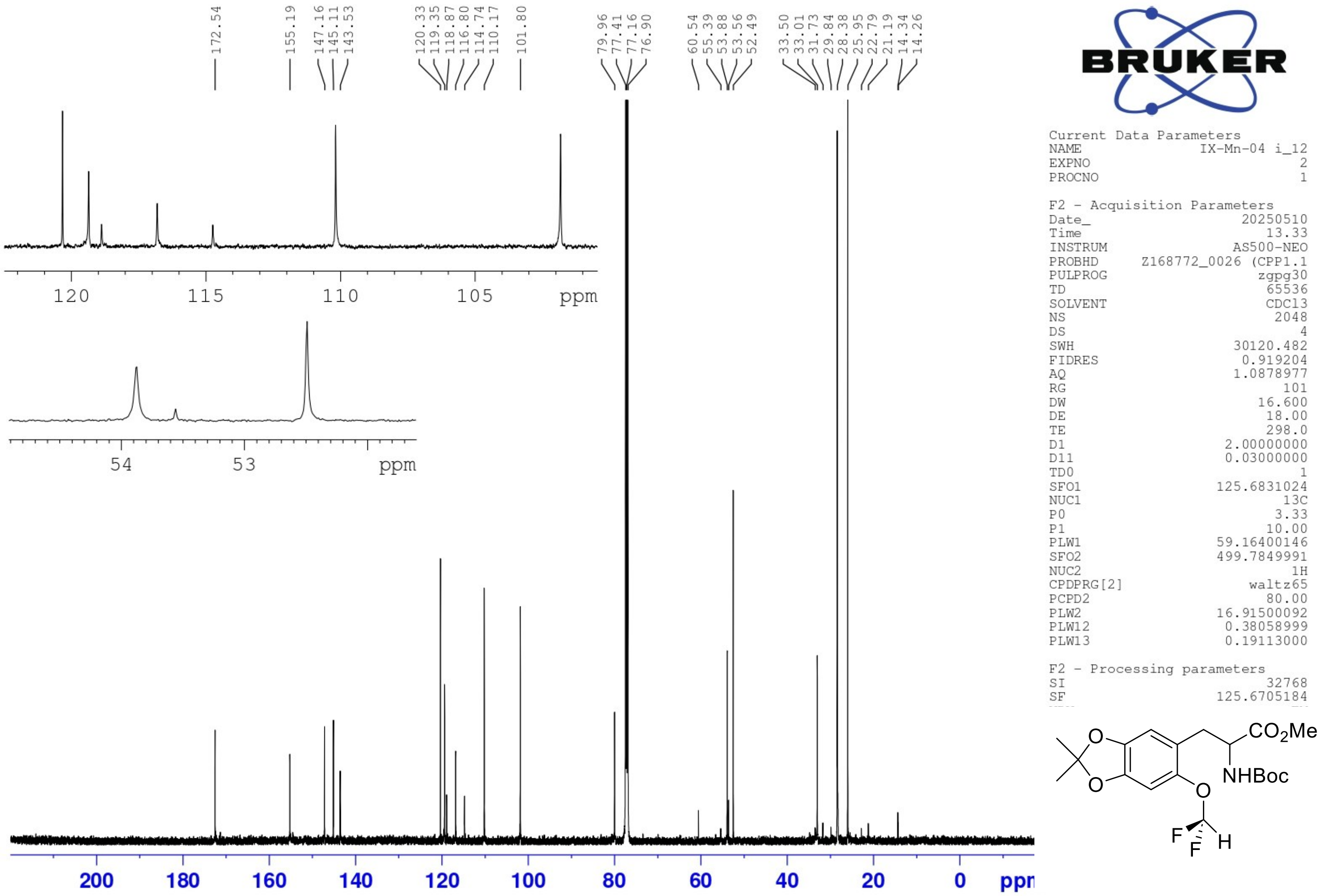

172.54 155.19 147.16 145.11 143.53 120.33 119.35 118.87 116.80 114.74 110.17 101.80 79.96 77.41 77.16 76.90 60.54 55.39 53.88 53.56 52.49 33.50 33.01 31.73 29.84 28.38 25.95 22.79 21.19 14.34 14.26
BRUKER
Current Data Parameters
NAME IX-Mn-04 i_12
EXPNO 2
PROCNO 1
F2 - Acquisition Parameters
Date_ 20250510
Time 13.33
INSTRUM AS500-NEO
PROBHD Z168772_0026 (CPP1.1
PULPROG zgpg30
TD 65536
SOLVENT CDCl3
NS 2048
DS 4
SWH 30120.482
FIDRES 0.919204
AQ 1.0878977
RG 101
DW 16.600
DE 18.00
TE 298.0
D1 2.00000000
D11 0.03000000
TD0 1
SFO1 125.6831024
NUC1 13C
P0 3.33
P1 10.00
PLW1 59.16400146
SFO2 499.7849991
NUC2 1H
CPDPRG[2] waltz65
PCPD2 80.00
PLW2 16.91500092
PLW12 0.38058999
PLW13 0.19113000
F2 - Processing parameters
SI 32768
SF 125.6705184
CO2Me
NHBoc
O
F
F
H
ppm

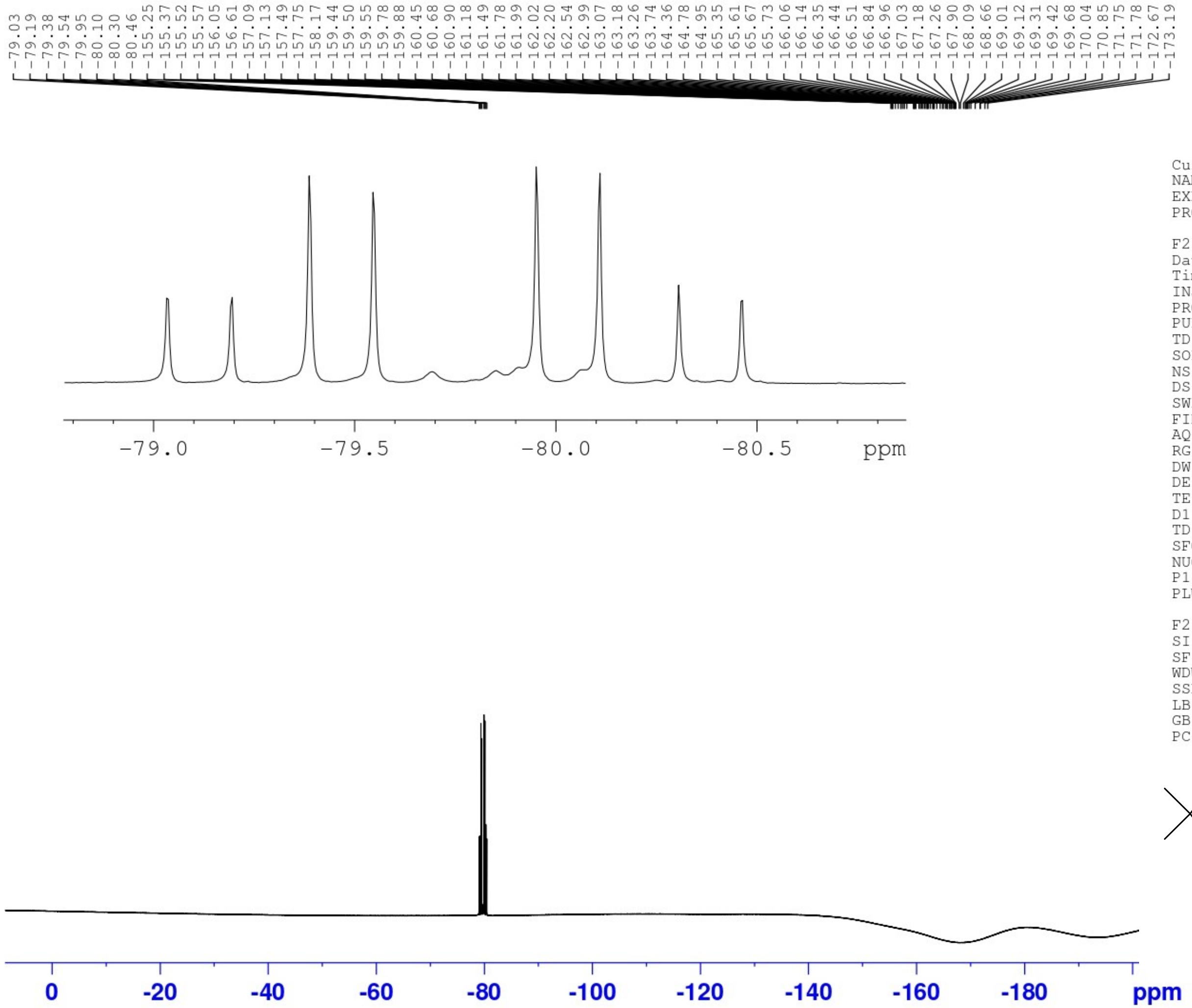

Current Data Parameters
NAME IX-Mn-04 i_11
EXPNO 3
PROCNO 1

F2 - Acquisition Parameters
Date_ 20250509
Time 16.50
INSTRUM AS500-NEO
PROBHD Z168772_0026 (CPP1.1
PULPROG zg
TD 131072
SOLVENT CDCl3
NS 16
DS 4
SWH 113636.364
FIDRES 1.733953
AQ 0.5767168
RG 11.3
DW 4.400
DE 18.00
TE 298.0
D1 1.00000000
TD0 1
SFO1 470.2188444
NUC1 19F
P1 15.00
PLW1 10.89000034

F2 - Processing parameters
SI 65536
SF 470.2658710
WDW EM
SSB 0
LB 0.30
GB 0
PC 1.00

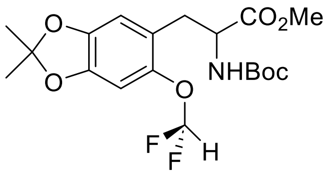

Supplement: Supplementary file 1 [file oc6c00127_si_001.pdf]
